# Supplementary material for: Integrative genomics reveals paths to sex dimorphism in Salix purpurea L
Source: Hortic Res. 2021 Aug 1;8:170. doi: 10.1038/s41438-021-00606-y (PMC8325687; doi:10.1038/s41438-021-00606-y)

Supplementary Figure S1

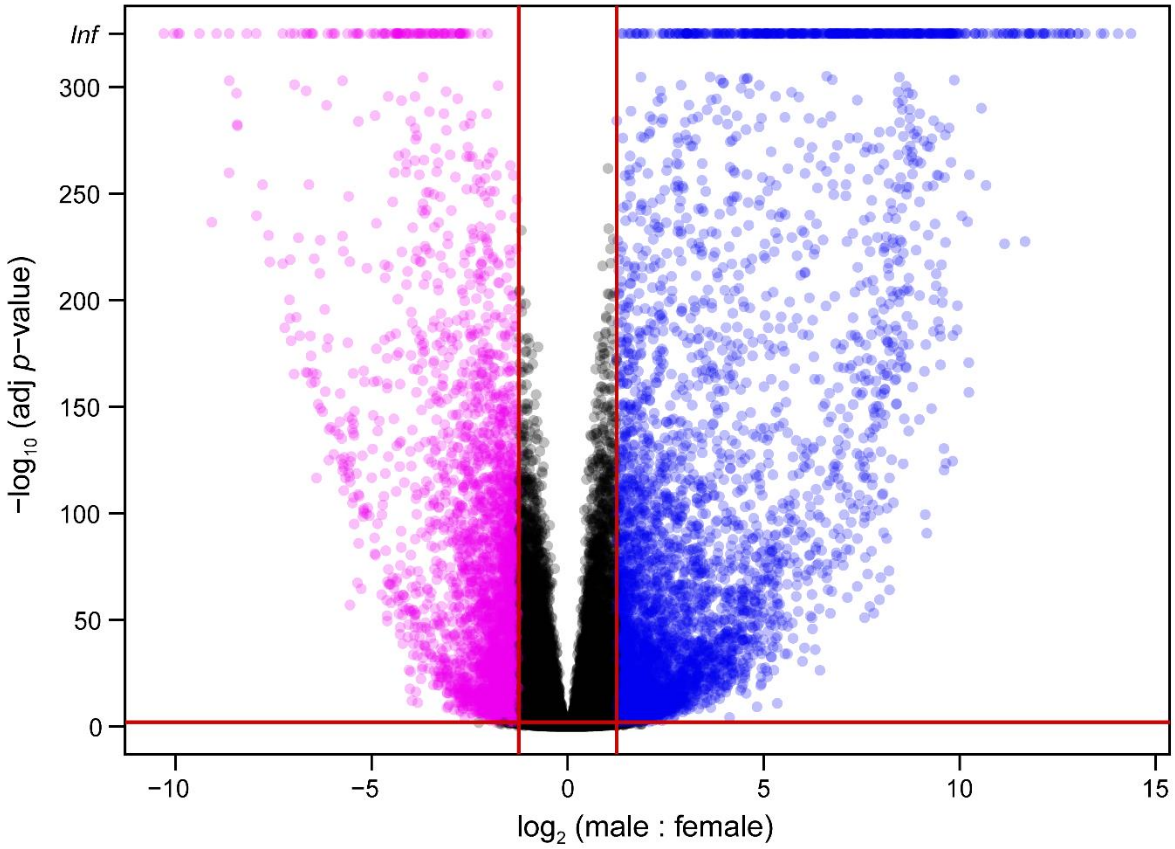

Supplementary Figure S2

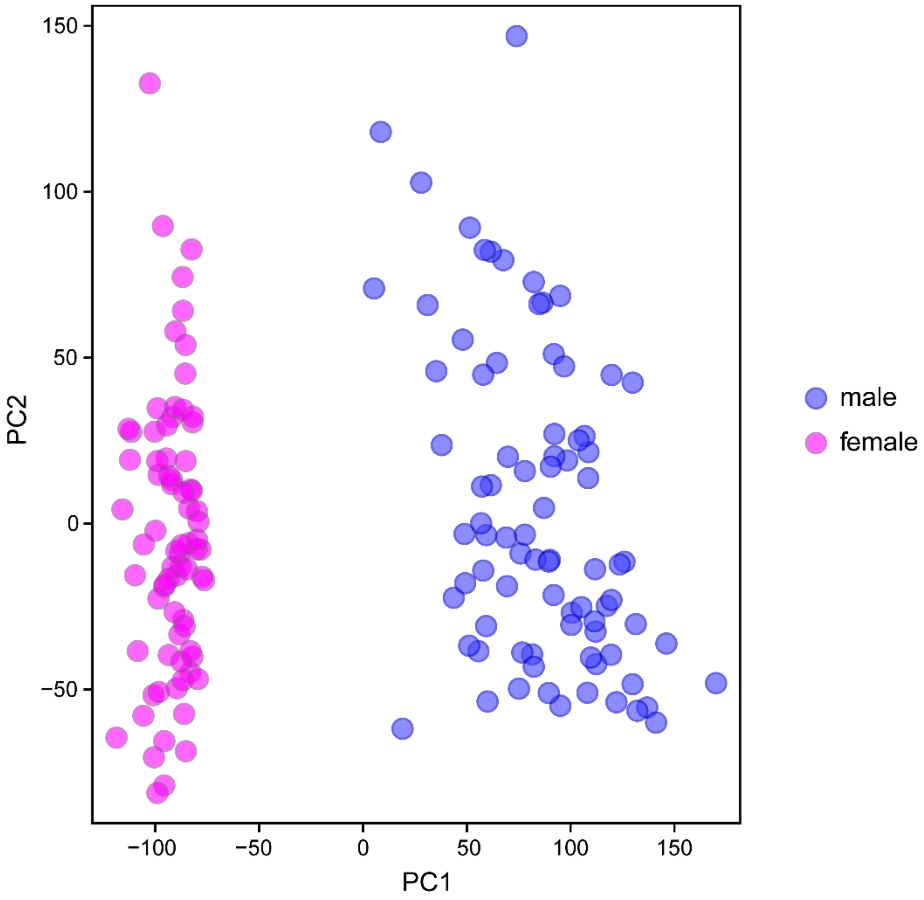

Supplementary Figure S3

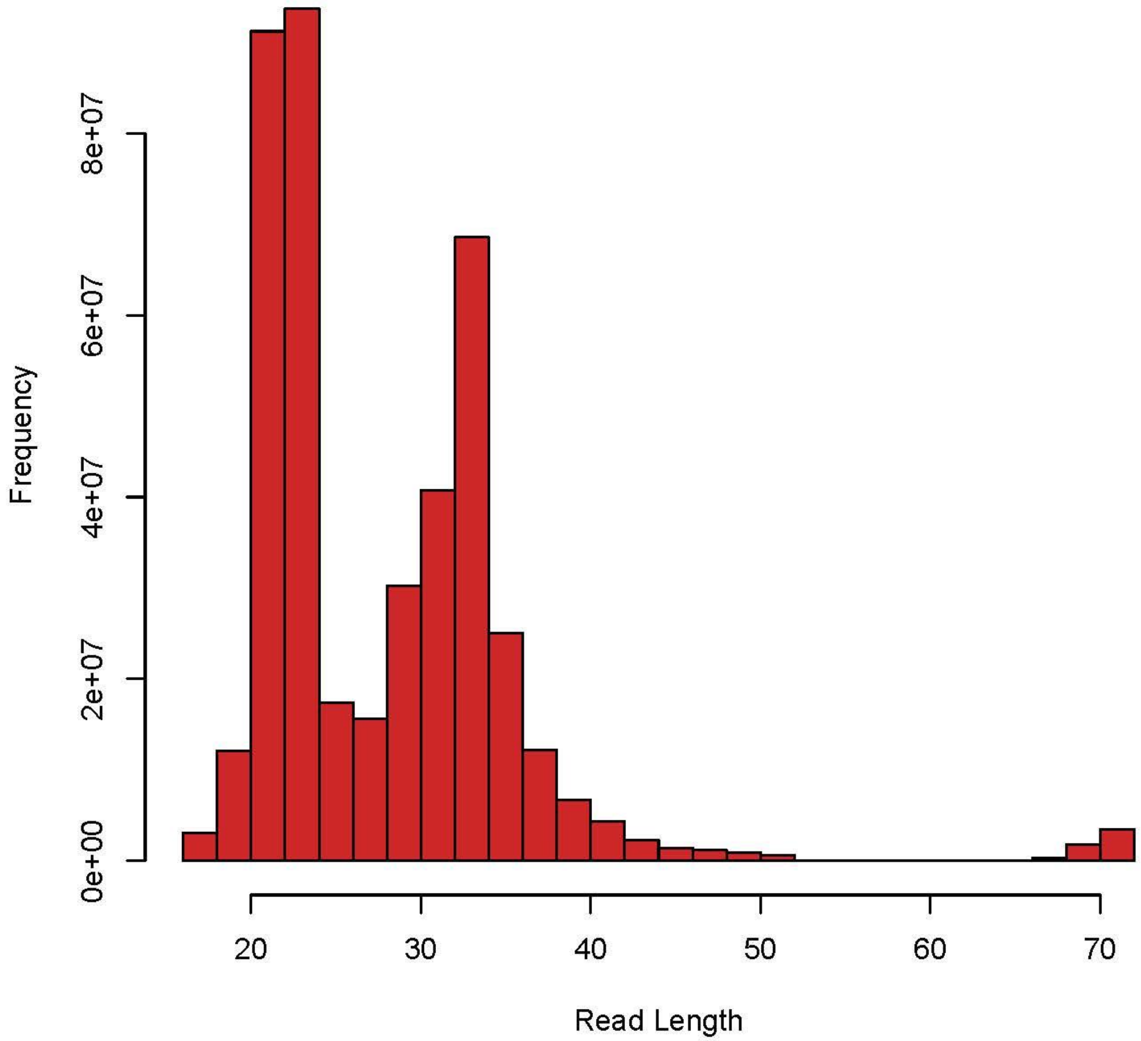

Supplementary Figure S4A

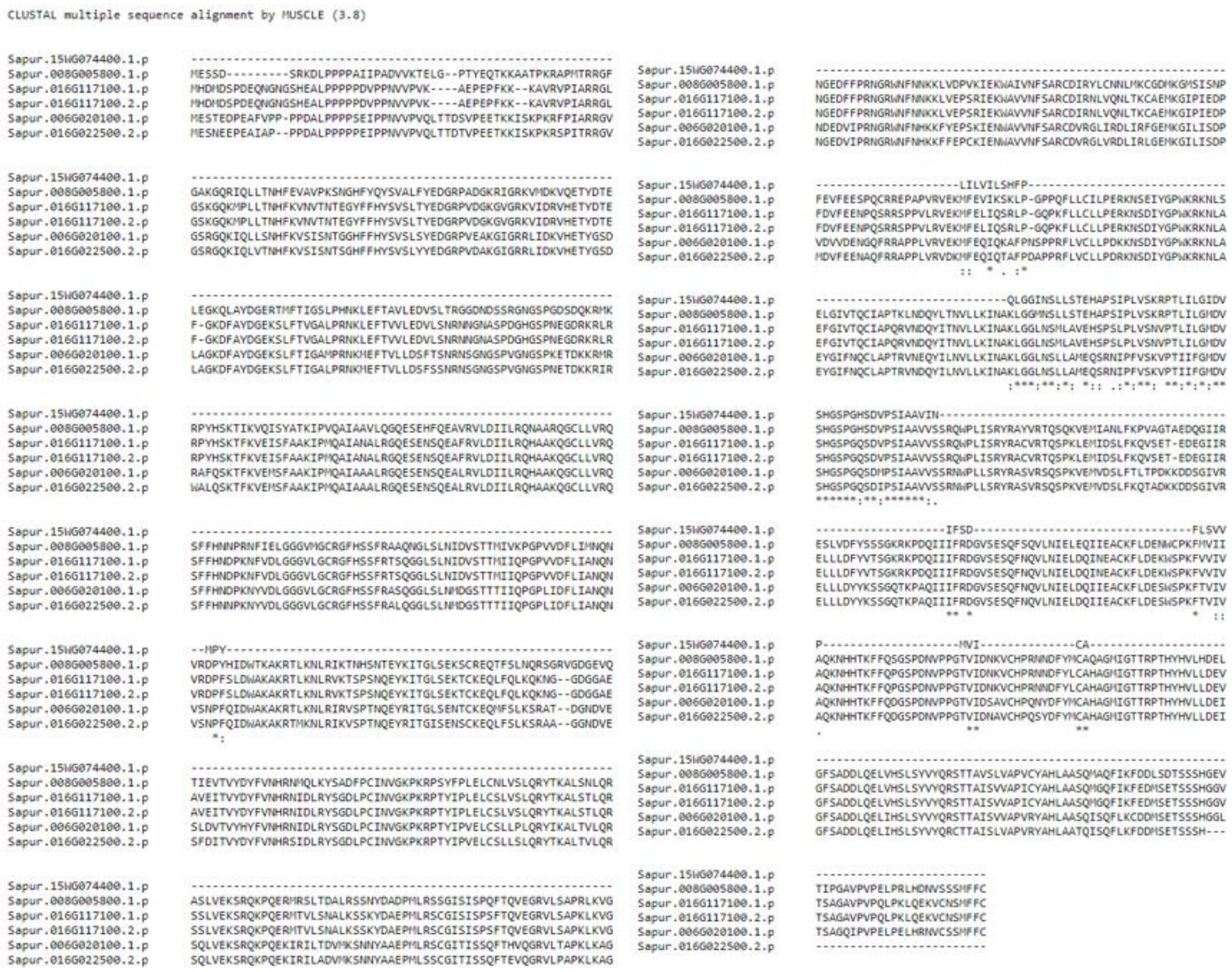

Supplementary Figure S4B

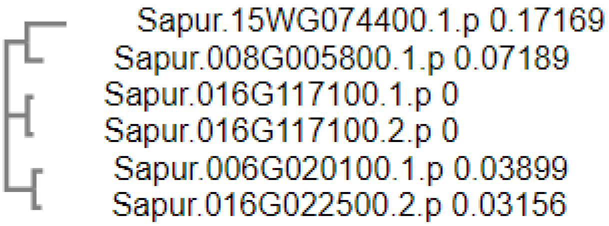

Supplementary Figure S5A

```
Cluster_187938 Original Location: Chr15Z:5200310-5200523 Displayed Location: Chr15Z:5200405-5200526 Strand: +

GGAGGCACUGAUGAUGUUGUUGACAGAAGAUAGAGAGCACAGAUGAUGGUAUGCAAUGGGCUCUGCAUCCCACUCCUUUGUGCUCUCUAAGCUUCUGUCAUCACUUUCAGCCCCCGACCCCC
((.(((.....(((...((.(((((((((.(((((((((((((.(((((.(((((........))))).))).)).)))))))))))))..))))))))).))...)))))).))..)))))
.................................................................................GCUCUCUAAGCUUCUGUCAUC.................... miRNA l=21 a=1487
....................UGACAGAAGAUAGAGAGCAC.................................................................................. miRNA-star l=20 a=4

....................................................................................CUCUAAGCUUCUGUCAUCc................... l=19 a=1
...................................................................................UCUCUAAGCUUCUGUCAUC.................... l=19 a=2
...................................................................................UCUCUAAGCUUCUGUCAUCACU................. l=22 a=5
..................................................................................CUCUCUAAGCUUCUGUCAUC.................... l=20 a=2
..................................................................................CUCUCUAAGCUUCUGUCAUCA................... l=21 a=1
..................................................................................CUCUCUAAGCUUCUGUCAUCACUUUCAGCCCCCGACu... l=37 a=2
..................................................................................CUCUCUAuGCUUCUGUCAUC.................... l=20 a=1
.................................................................................GCUCUCUAAGCUUCUGUC....................... l=18 a=1
.................................................................................GCUCUCUAAGCUUCUGUCA...................... l=19 a=1
.................................................................................GCUCUCUAAGCUUCUGUCAU..................... l=20 a=4
.................................................................................GCUCUCUAAGCUUCUGUCAUCA................... l=22 a=19
.................................................................................GCUCUCUAAGCUUCUGUCAUCACUUUCAGCCCCCGACu... l=38 a=1
.................................................................................GCUCUCUAAGCUUCUGUCAUCc................... l=22 a=3
.................................................................................GCUCUCUAAGCUUCUGUCAUCu................... l=22 a=98
.................................................................................GCUCUCUAAGCUUCUGUCAUCuC.................. l=23 a=1
.................................................................................GCUCUCUAAGCUUCUGUCAUa.................... l=21 a=4
.................................................................................GCUCUCUAAGCUUCUGUCAUu.................... l=21 a=36
.................................................................................GCUCUCUAAGCUUCUGUaAUC.................... l=21 a=4
.................................................................................GCUCUCUAAGCUUCUGaCAUC.................... l=21 a=1
.................................................................................GCUCUCUAAGCUUCUuUCAUC.................... l=21 a=1
.................................................................................GCUCUCUAAGCUUCaGUCAUC.................... l=21 a=3
.................................................................................GCUCUCUAAGCUUCcGUCAUC.................... l=21 a=1
.................................................................................GCUCUCUAAGCUUCgGUCAUC.................... l=21 a=1
.................................................................................GCUCUCUAAGCUUaUGUCAUC.................... l=21 a=1
.................................................................................GCUCUCUAAGCUUaUGUCAUCA................... l=22 a=1
.................................................................................GCUCUCUAAGCcUCUGUCAUC.................... l=21 a=1
.................................................................................GCUCUCUAAGaUUCUGUCAUC.................... l=21 a=4
.................................................................................GCUCUCUAuGCUUCUGUCAUC.................... l=21 a=77
.................................................................................GCUCUCUAuGCUUCUGUCAUCA................... l=22 a=3
.................................................................................GCUCUCaAAGCUUCUGUCAUC.................... l=21 a=1
.................................................................................GCUCUaUAAGCUUCUGUCAUC.................... l=21 a=1
.................................................................................GCUCUuUAAGCUUCUGUCAUC.................... l=21 a=1
.................................................................................GCUCaCUAAGCUUCUGUCAUC.................... l=21 a=2
.................................................................................GCUaUCUAAGCUUCUGUCAUC.................... l=21 a=1
.................................................................................GgUCUCUAAGCUUCUGUCAUC.................... l=21 a=2
.................................................................................cCUCUCUAAGCUUCUGUCAUC.................... l=21 a=1
.................................................................................nCUCUCUAAGCUUCUGUCAUC.................... l=21 a=1
.................................................................................uCUCUCUAAGCUUCUGUCAUC.................... l=21 a=5
................................................................................UGCUCUCUAAGCUUCUGUCAUC.................... l=22 a=1
........................................................................CUCCUUUGUGCUCUCUAAGCUU............................ l=22 a=5
.....................................................................CCACUCCUUUGUGCUCUCUAAGCU............................. l=24 a=1
.....................................................................CCACUCCUUUGUGCUCUCUAAGCUU............................ l=25 a=2
.................................................................CAUCCCACUCCUUUGUGCUCUCUAAGCUUCU.......................... l=31 a=1
........................................AGAUGAUGGUAUGCAAUGGG.............................................................. l=20 a=1
........................................AGAUGAUGGUAUGCAAUGGGC............................................................. l=21 a=2
........................................AGAUGAUGGUAUGCAAUGGGCU............................................................ l=22 a=1
........................................AGAUGAUGGUAUGCAAUGGGCUCUGCAUCC.................................................... l=30 a=1
........................................AGAUGAUGGUAUGCAAUGGGCUCUGCAUCCC................................................... l=31 a=3
........................................AGAUGAUGGUAUGCAAUGGGCUCUGCAUCCCA.................................................. l=32 a=1
........................................AGAUGAUGGUAUGCAAUGGGCUCUGCAUCCCACUCCUUUGU......................................... l=41 a=2
...................................AGCACAGAUGAUGGUAUGCAAUGGGC............................................................. l=26 a=1
............................GAUAGAGAGCACAGAUGAUGG......................................................................... l=21 a=2
.....................GACAGAAGAUAGAGAGCAC.................................................................................. l=19 a=1
....................UGACAGAAGAUAGAGAGCACAc................................................................................ l=22 a=1
...................UUGACAGAAGAUAGAGAGC.................................................................................... l=19 a=22
...................UUGACAGAAGAUAGAGAGCA................................................................................... l=20 a=8
...................UUGACAGAAGAUAGAGAGCAC.................................................................................. l=21 a=611
...................UUGACAGAAGAUAGAGAGCACA................................................................................. l=22 a=2
...................UUGACAGAAGAUAGAGAGCACAc................................................................................ l=23 a=1
...................UUGACAGAAGAUAGAGAGCACAu................................................................................ l=23 a=26
...................UUGACAGAAGAUAGAGAGCACc................................................................................. l=22 a=6
...................UUGACAGAAGAUAGAGAGCACu................................................................................. l=22 a=247
...................UUGACAGAAGAUAGAGAGCAaA................................................................................. l=22 a=1
...................UUGACAGAAGAUAGAGAGCAu.................................................................................. l=21 a=1
...................UUGACAGAAGAUAGAGAGCAuA................................................................................. l=22 a=7
...................UUGACAGAAGAUAGAGAGCu................................................................................... l=20 a=1
...................UUGACAGAAGAUAGAGgGCAC.................................................................................. l=21 a=1
...................UUGACAGAAcAUAGAGAGCACA................................................................................. l=22 a=1
...................UUGAgAGAAGAUAGAGAGCACA................................................................................. l=22 a=1
...................UUGgCAGAAGAUAGAGAGCAC.................................................................................. l=21 a=2
...................UUuACAGAAGAUAGAGAGCACA................................................................................. l=22 a=1
...................UaGACAGAAGAUAGAGAGCAC.................................................................................. l=21 a=1
...................UaGACAGAAGAUAGAGAGCACA................................................................................. l=22 a=3
...................UcGACAGAAGAUAGAGAGCAC.................................................................................. l=21 a=1
...................aUGACAGAAGAUAGAGAGCAC.................................................................................. l=21 a=1
................UUGUUGACAGAAGAUAGAGAGCA................................................................................... l=23 a=1
........UGAUGAUGUUGUUGACAGAAGAUAGAGAGCACA................................................................................. l=33 a=1
.....CACUGAUGAUGUUGUUGACAGAAGAUAGAGAGCAC.................................................................................. l=35 a=2
..AGGCACUGAUGAUGUUGUUGACAGAA.............................................................................................. l=26 a=2
..AGGCACUGAUGAUGUUGUUGACAGAAGAUAGAGAGCA................................................................................... l=37 a=1
<<<<<<<<<<<<<<<<<<<<<<<<<<<<<<<<<<<<<<<<<<<<<<<<<<<<<<<<<<<<<<<<<<<<<<<<<<<<<<<<cCGAGAGAUUCGAAGACAGUA<<<<<<<<<<<<<<<<<<<<< l=21 a=1
<<<<<<<<<<<<<<<<<<<<<<<<<<<<<<<<<<<<<<<<<<<<<<<<<<<<<<<<<<<<<<<<<<<<<<<<<<<<<<<CuCGAGAGAUUCGAAGACAGUA<<<<<<<<<<<<<<<<<<<<< l=22 a=1
<<<<<<<<<<<<<<<<<<<<<<<<<<<<<<<<<<<<<<<<<<<<<<<<<<<<<<<<<<<CGAGACGUAGGGUGAGGAAA<<<<<<<<<<<<<<<<<<<<<<<<<<<<<<<<<<<<<<<<<<< l=20 a=1
<<<<<<<<<<<<<<<<<<<<<<<<<<<<<<<<<<<<<<<<<<<<<<<<<<<<<<<<<<CCGAGACGUAGGGUGAGGAAA<<<<<<<<<<<<<<<<<<<<<<<<<<<<<<<<<<<<<<<<<<< l=21 a=1
<<<<<<<<<<<<<<<<<<<<<<<<<<<<<<<<<<<<<<<<<<<<<<<<<<<<<<<<<CCCGAGACGUAGGGUGAGGAAA<<<<<<<<<<<<<<<<<<<<<<<<<<<<<<<<<<<<<<<<<<< l=22 a=1
```

Supplementary Figure S5B

```
 Score = 212 bits (234),  Expect = 4e-54
 Identities = 121/122 (99%), Gaps = 1/122 (1%)
 Strand=Plus/Minus

Query  1        GGAGGCACTGATGATGTTGTTGACAGAAGATAGAGAGCACAGATGATGGTATGCAATGGG  60
                ||||||||||||||||||||||||||||||||||||||||||||||||||||||||||||
Sbjct  4026937  GGAGGCACTGATGATGTTGTTGACAGAAGATAGAGAGCACAGATGATGGTATGCAATGGG  4026878

Query  61       CTCTGCATCCCACTCCTTTGTGCTCTCTAAGCTTCTGTCATCACTTTCAGCCCCCGACCC  120
                |||||||||||||||||||||||||||||||||||||||||||||||||| |||||||||
Sbjct  4026877  CTCTGCATCCCACTCCTTTGTGCTCTCTAAGCTTCTGTCATCACTTTCAG-CCCCGACCC  4026819

Query  121      CC  122
                ||
Sbjct  4026818  CC  4026817
```

Supplementary Figure S6

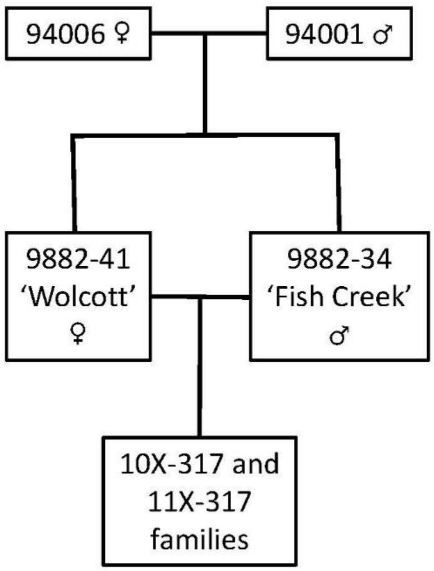

Supplementary Figure S7

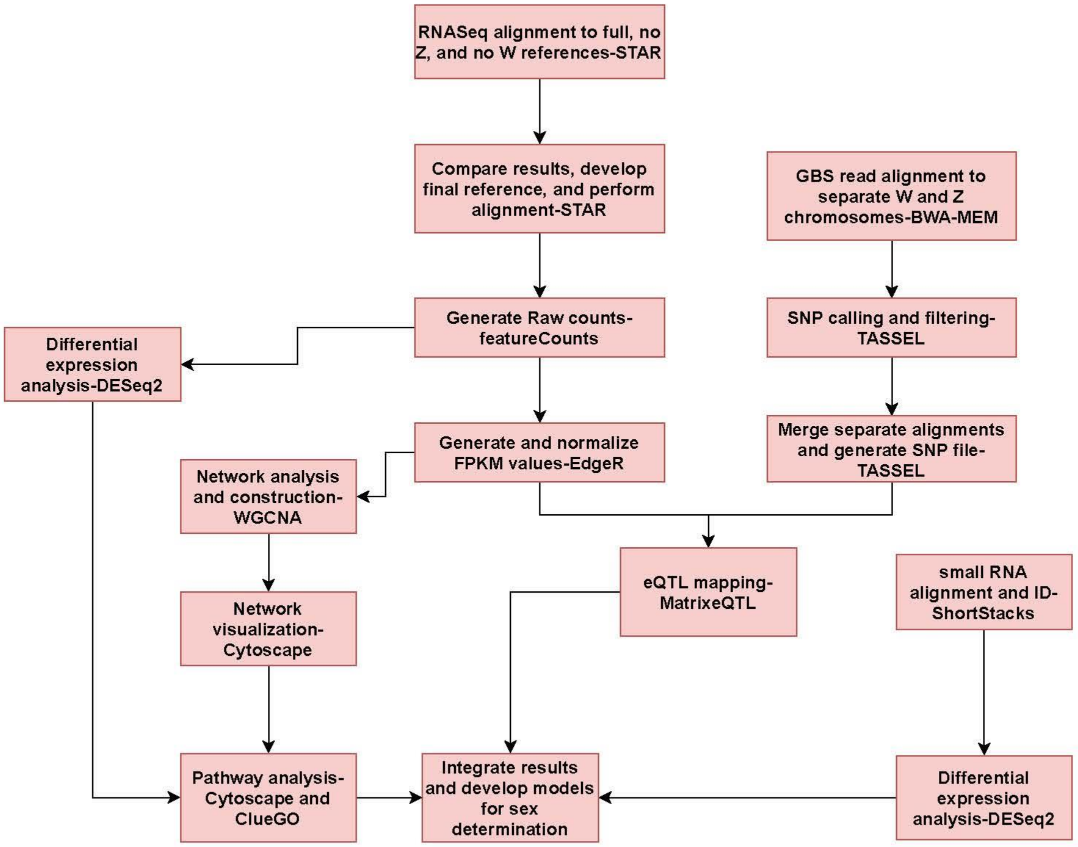

Supplementary Figure S8

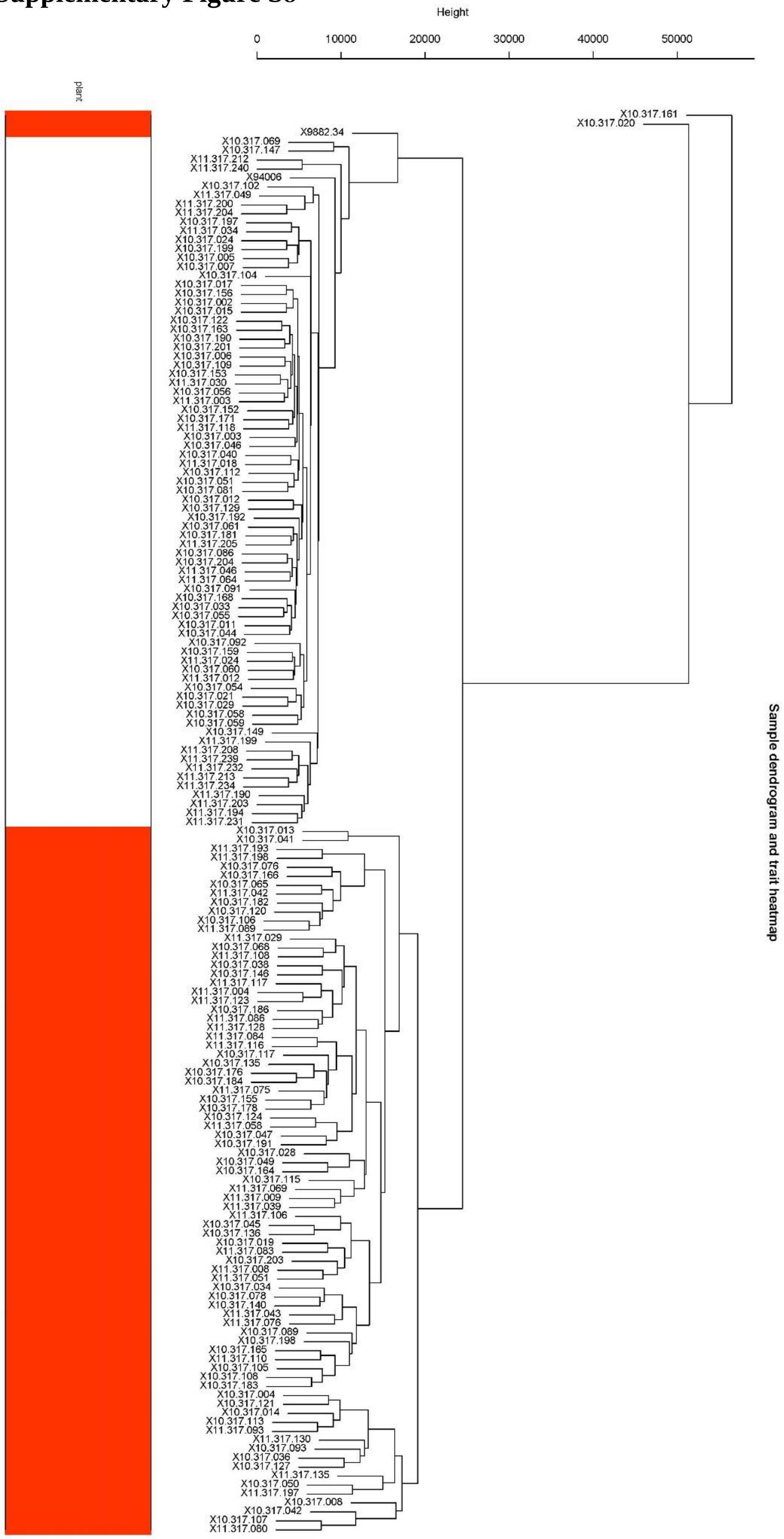

Supplement: Supplementary file 2 — Supplementary Figures [file 41438_2021_606_MOESM2_ESM.pdf]
