# Supplementary material for: Integrative genomics reveals paths to sex dimorphism in Salix purpurea L
Source: Hortic Res. 2021 Aug 1;8:170. doi: 10.1038/s41438-021-00606-y (PMC8325687; doi:10.1038/s41438-021-00606-y)
Supplement: Supplementary file 3 — Supplementary Tables S1-S4 [file 41438_2021_606_MOESM3_ESM.pdf]

Supplementary Table S1

GO ID	GO Description	Genes Present	FDR
272	polysaccharide catabolic process	28	7.25E-04
5975	carbohydrate metabolic process	131	4.29E-06
5976	polysaccharide metabolic process	52	1.09E-03
6468	protein amino acid phosphorylation	95	7.82E-04
6629	lipid metabolic process	92	9.42E-03
6793	phosphorus metabolic process	135	2.39E-03
6796	phosphate metabolic process	135	2.39E-03
6811	ion transport	54	7.25E-04
6812	cation transport	47	1.13E-04
6813	potassium ion transport	12	1.03E-02
6816	calcium ion transport	10	2.48E-02
6873	cellular ion homeostasis	23	1.03E-02
6885	regulation of pH	13	7.42E-04
6970	response to osmotic stress	27	1.81E-02
7276	gamete generation	6	3.73E-02
9269	response to desiccation	7	1.36E-02
9414	response to water deprivation	20	4.73E-02
9415	response to water	20	4.73E-02
9555	pollen development	20	6.94E-04
9651	response to salt stress	25	1.58E-02
9690	cytokinin metabolic process	7	3.81E-02
10208	pollen wall assembly	7	2.00E-02
10584	pollen exine formation	6	3.73E-02
10927	cellular component assembly involved in morphogenesis	7	2.00E-02
15672	monovalent inorganic cation transport	16	2.22E-02
15674	di-, tri-valent inorganic cation transport	19	1.30E-02
16042	lipid catabolic process	32	3.04E-03
16052	carbohydrate catabolic process	46	2.15E-04
16103	diterpenoid catabolic process	4	1.03E-02
16139	glycoside catabolic process	7	9.42E-03
16145	S-glycoside catabolic process	4	2.71E-02
16310	phosphorylation	114	2.00E-02
19748	secondary metabolic process	36	1.81E-02
19759	glycosinolate catabolic process	4	2.71E-02
19762	glucosinolate catabolic process	4	2.71E-02
19953	sexual reproduction	13	9.42E-03
30001	metal ion transport	46	7.12E-06
30198	extracellular matrix organization	7	2.71E-02

32504	multicellular organism reproduction	10	2.78E-02
33499	galactose catabolic process via UDP-galactose	5	4.73E-02
42221	response to chemical stimulus	159	1.58E-02
42542	response to hydrogen peroxide	16	3.73E-02
42545	cell wall modification	22	6.81E-04
42592	homeostatic process	51	9.87E-03
42743	hydrogen peroxide metabolic process	14	4.19E-02
42744	hydrogen peroxide catabolic process	14	4.19E-02
43062	extracellular structure organization	7	2.71E-02
44247	cellular polysaccharide catabolic process	18	1.17E-02
44262	cellular carbohydrate metabolic process	87	1.51E-02
44264	cellular polysaccharide metabolic process	41	1.78E-02
44273	sulfur compound catabolic process	5	4.73E-02
44275	cellular carbohydrate catabolic process	36	3.04E-03
45229	external encapsulating structure organization	9	2.20E-03
45487	gibberellin catabolic process	4	1.03E-02
45488	pectin metabolic process	22	1.49E-03
45490	pectin catabolic process	18	5.89E-04
45493	xylan catabolic process	5	2.78E-02
48229	gametophyte development	25	2.61E-03
48232	male gamete generation	6	2.50E-02
48235	pollen sperm cell differentiation	6	2.50E-02
48609	reproductive process in a multicellular organism	10	2.78E-02
48610	reproductive cellular process	18	2.39E-03
48878	chemical homeostasis	44	8.22E-06
50801	ion homeostasis	40	4.29E-06
51017	actin filament bundle assembly	5	4.73E-02
55046	microgametogenesis	6	2.50E-02
55066	di-, tri-valent inorganic cation homeostasis	22	2.50E-02
55067	monovalent inorganic cation homeostasis	14	1.06E-03
55080	cation homeostasis	36	3.69E-05
55082	cellular chemical homeostasis	23	1.30E-02
65008	regulation of biological quality	84	1.30E-02
70301	cellular response to hydrogen peroxide	14	4.73E-02
70838	divalent metal ion transport	12	3.09E-02
71554	cell wall organization or biogenesis	80	2.56E-10
71555	cell wall organization	66	6.44E-08
71669	plant-type cell wall organization or biogenesis	21	4.50E-02

Supplementary Table S2

GO ID	GO Description	Genes Present	FDR
375	RNA splicing, via transesterification reactions	1	3.64E-02
377	RNA splicing, via transesterification reactions with bulged adenosine as nucleophile	1	3.64E-02
6139	nucleobase, nucleoside, nucleotide and nucleic acid metabolic process	43	6.81E-25
6259	DNA metabolic process	11	9.50E-05
6260	DNA replication	2	9.57E-03
6261	DNA-dependent DNA replication	0	3.21E-03
6281	DNA repair	6	6.16E-03
6364	rRNA processing	0	1.66E-03
6396	RNA processing	9	9.19E-11
6397	mRNA processing	6	4.94E-05
6399	tRNA metabolic process	2	3.21E-02
6403	RNA localization	0	2.63E-02
6412	translation	11	1.56E-05
6511	ubiquitin-dependent protein catabolic process	11	4.35E-03
6605	protein targeting	3	2.15E-04
6807	nitrogen compound metabolic process	73	1.07E-24
6913	nucleocytoplasmic transport	0	1.51E-02
6974	response to DNA damage stimulus	7	4.35E-03
6996	organelle organization	55	2.87E-03
7005	mitochondrion organization	2	7.99E-03
8152	metabolic process	596	4.43E-02
8380	RNA splicing	1	4.49E-04
9058	biosynthetic process	149	9.42E-03
9059	macromolecule biosynthetic process	53	4.17E-05
9108	coenzyme biosynthetic process	1	4.33E-02
9451	RNA modification	4	5.90E-06
10467	gene expression	27	3.95E-19
16070	RNA metabolic process	19	4.04E-19
16071	mRNA metabolic process	7	5.90E-06
16072	rRNA metabolic process	0	5.94E-04
17038	protein import	0	4.76E-04
18130	heterocycle biosynthetic process	1	2.38E-03
19941	modification-dependent protein catabolic process	13	1.55E-02
22613	ribonucleoprotein complex biogenesis	2	5.07E-07
22618	ribonucleoprotein complex assembly	2	4.54E-02
30163	protein catabolic process	22	2.49E-02
33365	protein localization in organelle	0	1.08E-04

34470	ncRNA processing	3	1.73E-03
34613	cellular protein localization	22	4.43E-02
34621	cellular macromolecular complex subunit organization	11	9.09E-05
34622	cellular macromolecular complex assembly	10	1.36E-03
34641	cellular nitrogen compound metabolic process	62	2.53E-27
34645	cellular macromolecule biosynthetic process	52	2.49E-05
34660	ncRNA metabolic process	2	5.31E-06
42254	ribosome biogenesis	1	5.22E-06
42440	pigment metabolic process	1	4.33E-02
43170	macromolecule metabolic process	290	2.67E-08
43632	modification-dependent macromolecule catabolic process	13	1.00E-02
43933	macromolecular complex subunit organization	15	2.97E-04
44237	cellular metabolic process	451	3.55E-07
44238	primary metabolic process	481	4.43E-02
44249	cellular biosynthetic process	137	1.36E-03
44257	cellular protein catabolic process	18	1.76E-02
44260	cellular macromolecule metabolic process	262	3.36E-09
44265	cellular macromolecule catabolic process	23	6.53E-03
44271	cellular nitrogen compound biosynthetic process	19	1.20E-02
46483	heterocycle metabolic process	15	4.54E-02
46907	intracellular transport	25	2.81E-04
50657	nucleic acid transport	0	4.33E-02
50658	RNA transport	0	4.33E-02
51169	nuclear transport	0	1.51E-02
51186	cofactor metabolic process	13	5.81E-03
51188	cofactor biosynthetic process	1	1.00E-04
51236	establishment of RNA localization	0	4.33E-02
51276	chromosome organization	10	1.24E-02
51603	proteolysis involved in cellular protein catabolic process	18	2.14E-02
51641	cellular localization	37	6.53E-03
51649	establishment of localization in cell	33	6.54E-03
65003	macromolecular complex assembly	14	3.61E-03
70727	cellular macromolecule localization	22	3.50E-02
90304	nucleic acid metabolic process	29	6.81E-25

Supplementary Table S3

GO ID	GO Description	Genes Represented	FDR
3002	regionalization	12	2.56E-03
6355	regulation of transcription, DNA-dependent	109	2.74E-04
7275	multicellular organismal development	92	1.34E-02
7389	pattern specification process	13	6.35E-03
9719	response to endogenous stimulus	58	3.84E-02
9755	hormone-mediated signaling pathway	42	4.61E-02
9889	regulation of biosynthetic process	116	4.86E-04
9943	adaxial/abaxial axis specification	4	2.61E-02
9944	polarity specification of adaxial/abaxial axis	4	2.61E-02
9955	adaxial/abaxial pattern formation	4	2.61E-02
10103	stomatal complex morphogenesis	5	4.46E-02
10374	stomatal complex development	8	1.91E-03
10468	regulation of gene expression	111	9.35E-03
10556	regulation of macromolecule biosynthetic process	113	7.23E-04
19219	regulation of nucleobase, nucleoside, nucleotide and nucleic acid metabolic process	113	4.86E-04
19222	regulation of metabolic process	127	4.69E-03
22621	shoot system development	24	7.68E-06
30154	cell differentiation	35	5.34E-05
31323	regulation of cellular metabolic process	124	2.37E-05
31326	regulation of cellular biosynthetic process	115	3.35E-06
32501	multicellular organismal process	103	1.47E-05
32502	developmental process	110	1.04E-05
45449	regulation of transcription	109	4.26E-07
48367	shoot development	24	9.90E-04
48513	organ development	39	1.76E-03
48731	system development	40	1.04E-03
48869	cellular developmental process	43	4.62E-03
51171	regulation of nitrogen compound metabolic process	114	4.86E-04
51252	regulation of RNA metabolic process	109	4.86E-04
60255	regulation of macromolecule metabolic process	115	1.68E-02
65001	specification of axis polarity	4	2.61E-02
80090	regulation of primary metabolic process	115	4.62E-03

Supplementary Table S4

GO ID	GO Description	Genes Present	FDR
5996	monosaccharide metabolic process	2	3.45E-02
6139	nucleobase, nucleoside, nucleotide and nucleic acid metabolic process	29	1.22E-09
6396	RNA processing	5	2.98E-05
6412	translation	5	2.47E-03
6511	ubiquitin-dependent protein catabolic process	2	1.36E-03
6732	coenzyme metabolic process	2	1.86E-02
6807	nitrogen compound metabolic process	52	5.93E-08
6810	transport	51	1.85E-02
6996	organelle organization	26	1.05E-02
8104	protein localization	12	2.31E-03
8152	metabolic process	296	9.87E-05
9058	biosynthetic process	72	5.33E-03
9059	macromolecule biosynthetic process	28	5.44E-03
9451	RNA modification	1	8.80E-04
9987	cellular process	382	1.74E-04
10467	gene expression	17	2.21E-08
15031	protein transport	10	1.57E-03
16070	RNA metabolic process	14	1.69E-07
16071	mRNA metabolic process	5	2.13E-02
16192	vesicle-mediated transport	5	2.66E-02
19941	modification-dependent protein catabolic process	2	1.16E-03
22613	ribonucleoprotein complex biogenesis	1	1.64E-03
33036	macromolecule localization	20	2.32E-02
34641	cellular nitrogen compound metabolic process	46	7.27E-09
34645	cellular macromolecule biosynthetic process	28	5.68E-03
34660	ncRNA metabolic process	0	6.88E-04
42254	ribosome biogenesis	0	1.75E-03
43170	macromolecule metabolic process	153	1.36E-05
43412	macromolecule modification	72	2.48E-02
43632	modification-dependent macromolecule catabolic process	2	8.38E-04
44237	cellular metabolic process	212	1.84E-10
44238	primary metabolic process	232	6.39E-05
44249	cellular biosynthetic process	67	2.31E-03
44257	cellular protein catabolic process	5	4.37E-03
44260	cellular macromolecule metabolic process	124	2.98E-09
44262	cellular carbohydrate metabolic process	16	2.26E-02
44265	cellular macromolecule catabolic process	5	1.42E-04
44267	cellular protein metabolic process	91	5.96E-03

45184	establishment of protein localization	10	1.36E-03
46907	intracellular transport	12	8.40E-03
51179	localization	54	1.74E-02
51186	cofactor metabolic process	4	7.41E-03
51234	establishment of localization	52	1.87E-02
51603	proteolysis involved in cellular protein catabolic process	5	4.98E-03
51641	cellular localization	13	1.21E-03
51649	establishment of localization in cell	13	5.44E-03
90304	nucleic acid metabolic process	24	2.83E-08
