# Supplementary material for: Integrative genomics reveals paths to sex dimorphism in Salix purpurea L
Source: Hortic Res. 2021 Aug 1;8:170. doi: 10.1038/s41438-021-00606-y (PMC8325687; doi:10.1038/s41438-021-00606-y)
Supplement: Supplementary file 4 — Supplementary Table S5 [file 41438_2021_606_MOESM4_ESM.pdf]

Name	Chr	Start	end	Length	Reads	Unique Reads	Major RNA	Major RNA Reads	Dicer Call	<20
Spu-miR12420	Chr01	5390167	5390252	86	19	18	UUUUGAAAAAGAUUUGGGCUAAGC	12	24	0
Spu-miR167a	Chr02	2546206	2546454	249	21948	984	UGAAGCUGCCAGCAUGAUCUG	14491	21	108
Spu-miR12421	Chr02	6755707	6755829	123	925	683	UGAAACGCAGUGCCAAGCAAGCAUA	480	25	5
Spu-miR171a	Chr04	476403	476580	178	1111	15	AGAUAUUGGUACGGUUCAAUC	829	21	21
Spu-miR399a	Chr04	3155960	3156093	134	52	11	UGCCAAAGGAGAUUUGCCCUG	39	21	0
Spu-miR399b	Chr04	3158638	3158815	178	762	109	UGCCAAAGGAGAUUUGCCCUG	381	21	1
Spu-miR3627a	Chr04	9319512	9319746	235	21626	21626	UUGUCGCAGGAGCGGUGGCACC	13074	22	7
Spu-miR395a	Chr04	15610953	15611114	162	419	18	CUGAAGUGUUUGGGGGAACUC	287	21	19
Spu-miR395b	Chr04	15612449	15612536	88	88	1	CUGAAGUGUUUGGGGGAACUC	17	21	7
Spu-miR395c	Chr04	15628880	15629146	267	307	26	CUGAAGUGUUUGGGGGAACUC	62	21	35
Spu-miR167b	Chr05	2940103	2940284	182	75585	3130	UGAAGCUGCCAGCAUGAUCUG	45693	21	255
Spu-miR166a	Chr05	3924142	3924378	237	3091	17	UCGGACCAGGCUUCAUUCCCC	2575	21	23
Spu-miR167c	Chr05	15569344	15569484	141	3368	118	UGAAGCUGCCAGCAUGAUCUG	1694	21	11
Spu-miR171b	Chr06	3831004	3831090	87	86	0	CGAUGUUGGUGAGGUUCAAUC	65	21	0
Spu-miR171c	Chr06	3835891	3836056	166	90	0	CGAUGUUGGUGAGGUUCAAUC	69	21	0
Spu-miR12422	Chr06	4882875	4883149	275	6720	637	UCGGUUUUUCUAUUGUUACGGAAC	3336	24	55
Spu-miR2111	Chr06	5427587	5427760	174	465	454	CUAGGCCUUGGGUUGCAGAUUACC	325	24	3
Spu-miR390a	Chr06	6180906	6181056	151	26	3	AAGCUCAGGAGGGAUAGCGCC	10	21	0
Spu-miR164a	Chr06	6317292	6317530	239	1413	248	UGGAGAAGCAGGGCACGUGCA	595	21	21
Spu-miR156a	Chr06	13846849	13846964	116	461	79	UGACAGAAGAGAGUGAGCACU	108	21	3
Spu-miR156b	Chr06	19006933	19007076	144	230	27	UGACAGAAGAGAGUGAGCAC	46	21	0
Spu-miR403	Chr08	5423300	5423499	200	40893	879	UUAGAUUCACGCACAAACUCG	37892	21	124
Spu-miR167d	Chr08	8894197	8894487	291	9450	264	UGAAGCUGCCAGCAUGAUCUG	3784	21	47
Spu-miR166b	Chr08	9245708	9245886	179	607471	320	UCUCGGACCAGGCUUCAUUCC	393394	21	1953
Spu-miR12423	Chr08	12743624	12743755	132	70	66	UCAGAAGGAUAUAUUAGAAAC	23	21	1
Spu-miR172a	Chr09	2892935	2893114	180	619	350	GCGGCAGCAUCAAGAUUCACA	246	21	19
Spu-miR390b	Chr09	5612879	5613152	274	103394	14952	AAGCUCAGGAGGGAUAGCGCC	57097	21	1269
Spu-miR403a	Chr10	10076881	10077088	208	63298	1362	UUAGAUUCACGCACAAACUCG	59000	21	163
Spu-miR156c	Chr12	3197390	3197490	101	1667	23	GCUCUCUAAGCUUCUGUCAUC	898	21	18
Spu-miR171c	Chr12	8351984	8352111	128	319	35	UUGAGCCGUGCCAAUAUCACG	221	21	3
Spu-miR399	Chr12	10545966	10546254	289	2299	291	UGCCAAAGGAGAUUUGCCCUG	1070	21	10
Spu-miR393	Chr12	10816595	10816773	179	51462	24	UCCAAAGGGAUCGCAUUGAUUU	43394	22	12
Spu-miR164	Chr13	842171	842291	121	532	72	UGGAGAAGCAGGGCACGUGCA	170	21	5
Spu-miR319	Chr13	14611610	14611886	277	68220	427	UUGGACUGAAGGGAGCUCCC	26312	21	389
Spu-miR162	Chr15W	6070157	6070284	128	15830	394	UCGAUAAACCUCUGCAUCCAG	8010	21	1356
Spu-miR6445	Chr15W	9647264	9647437	174	3229	0	AUUUUAGGAAGGGAAUGAAUA	1079	21	31
Spu-miR1446-1	Chr15W	13324567	13324662	96	933	0	UUCUGAACUCUCUCCCUCAAC	378	21	3
Spu-miR156d	Chr15Z	5200310	5200523	214	2821	38	GCUCUCUAAGCUUCUGUCAUC	1487	21	30
Spu-miR1446-2	Chr15Z	10970968	10971061	94	972	0	UUCUGAACUCUCUCCCUCAAC	346	21	5
Spu-miR160a	Chr16	80655	80741	87	19	9	UGCCUGGCUCCCUGUAUGCCA	8	21	1
Spu-miR395d	Chr16	11568408	11568564	157	502	21	CUGAAGUGUUUGGGGGAACUC	347	21	19
Spu-miR12424	Chr16	13458436	13458691	256	53	50	UGUUUUAAUGGAUUGCAGAUG	30	21	1
Spu-miR172b	Chr16	15596694	15596955	262	35	16	GCGGCAUCAUCACGAUUCACA	9	21	3
Spu-miR160b	Chr16	30160705	30160920	216	149	56	UGCCUGGCUCCCUGUAUGCCA	59	21	1
Spu-miR396	Chr18	10287527	10287770	244	6542	44	GUUCAAUAAAGCUGUGGGAAG	2290	21	233

20	21	22	23	24	25	>25	PmiREN match
0	0	0	1	18	0	0	-
213	16062	5077	118	84	13	273	Ptr-miR167a
3	12	21	23	239	570	52	-
9	969	81	6	16	0	9	Ptr-miR171i
0	49	1	0	1	0	1	Ath-miR399a
4	680	4	22	50	0	1	Ath-miR399a
6	250	13344	266	7497	203	53	Mes-miR3627
69	318	8	2	2	0	1	Ptr-miR395a
22	54	0	0	3	0	2	Ptr-miR395a
66	171	2	5	4	0	24	Ptr-miR395a
2232	50174	21483	316	999	90	36	Ptr-miR167a
17	2860	125	56	8	0	2	Ptr-miR166a
52	2378	793	26	64	15	29	Ptr-miR167a
1	72	2	8	3	0	0	Gma-miR171l
1	75	7	4	1	1	1	Gma-miR171l
68	289	91	183	5465	313	256	-
1	45	5	9	392	7	3	Ptr-MIR2111a
0	19	3	0	0	3	1	Ptr-miR390a
48	1050	76	23	87	91	17	Ptr-miR164a
110	267	66	4	1	0	10	Mac-miR156f
48	97	55	7	10	2	11	Ptr-miR156a
115	39323	1162	23	135	1	10	Ptr-miR403a
141	5340	1362	130	2241	115	74	Ptr-miR167a
1883	590965	3739	2086	6767	36	42	Ptr-miR166o
4	31	8	4	9	11	2	-
89	485	20	2	2	0	2	Ptr-miR172a
6244	73429	5565	2390	13743	243	511	Ptr-miR390a
199	60792	1699	97	75	22	251	Ptr-miR403a
9	1397	215	23	5	0	0	Aco-miR156m
7	296	11	2	0	0	0	Ath-miR171b
24	2026	195	11	18	1	14	Ath-miR399a
36	1357	48716	1307	22	2	10	Aof-miR393a
9	433	30	5	40	4	6	Ptr-miR164a
28383	28476	9328	976	515	83	70	Ath-miR1319a
2486	11154	358	148	23	72	233	Ptr-miR162a
111	1154	578	518	174	74	589	Ptr-miR6445a
52	617	237	10	6	3	5	Ptr-miR1446a
22	2271	411	32	3	6	46	Aco-miR156m
55	579	303	12	11	1	6	Ptr-miR1446a
2	15	1	0	0	0	0	Ptr-miR160a
94	366	5	3	14	0	1	Ptr-miR395a
2	38	2	1	0	1	8	-
3	25	1	0	2	0	1	Ptr-miR172a
16	91	23	2	0	1	15	Ptr-miR160a
500	5672	91	7	7	9	23	Mac-miR396t
