# Supplementary material for: Integrative genomics reveals paths to sex dimorphism in Salix purpurea L
Source: Hortic Res. 2021 Aug 1;8:170. doi: 10.1038/s41438-021-00606-y (PMC8325687; doi:10.1038/s41438-021-00606-y)
Supplement: Supplementary file 5 — Supplementary Table S6 [file 41438_2021_606_MOESM5_ESM.pdf]

Chr	start	end	CpG sites	Male mean proportiionMethylated	Female mean proportion Methylated	Female:Male difference	FDR
Chr01	333391	333878	145	4.905E-01	3.519E-01	-1.386E-01	7.448E-03
Chr01	546756	547250	186	1.733E-01	6.072E-02	-1.126E-01	1.283E-02
Chr01	547253	547747	192	1.602E-01	4.433E-02	-1.158E-01	4.971E-02
Chr01	628596	629090	131	3.462E-01	1.826E-01	-1.636E-01	1.174E-02
Chr01	738998	739492	192	2.378E-01	1.199E-01	-1.179E-01	7.000E-07
Chr01	897952	898349	121	4.406E-01	3.367E-01	-1.039E-01	6.620E-05
Chr01	986492	986987	175	2.582E-01	1.461E-01	-1.122E-01	3.429E-02
Chr01	995163	995616	141	3.865E-01	2.715E-01	-1.150E-01	1.700E-06
Chr01	1508072	1508570	216	2.874E-01	1.168E-01	-1.706E-01	1.237E-02
Chr01	1682772	1683270	102	2.685E-01	1.664E-01	-1.020E-01	4.116E-04
Chr01	2273380	2273868	104	3.216E-01	2.019E-01	-1.196E-01	5.634E-03
Chr01	2406795	2407197	94	3.384E-01	4.860E-01	1.476E-01	2.859E-04
Chr01	2428135	2428597	57	3.871E-01	1.564E-01	-2.307E-01	3.920E-05
Chr01	2440908	2441398	177	4.122E-01	3.019E-01	-1.103E-01	1.777E-04
Chr01	2587558	2588042	145	4.207E-01	3.186E-01	-1.022E-01	0.000E+00
Chr01	2605581	2606049	138	2.078E-01	3.277E-01	1.199E-01	2.300E-03
Chr01	2882193	2882682	163	3.066E-01	1.966E-01	-1.100E-01	3.610E-03
Chr01	3247955	3248442	95	4.531E-01	3.448E-01	-1.083E-01	1.523E-03
Chr01	4170359	4170846	137	4.430E-01	2.782E-01	-1.648E-01	1.532E-03
Chr01	4294428	4294914	92	4.056E-01	9.008E-02	-3.156E-01	0.000E+00
Chr01	5635995	5636487	186	4.122E-01	2.657E-01	-1.465E-01	1.600E-02
Chr01	6226505	6226992	92	4.168E-01	2.891E-01	-1.277E-01	5.939E-04
Chr01	6623254	6623745	135	3.071E-01	1.872E-01	-1.199E-01	2.720E-03
Chr01	6826225	6826436	46	3.608E-01	2.559E-01	-1.049E-01	8.340E-05
Chr01	7658488	7658980	167	2.743E-01	1.674E-01	-1.069E-01	3.013E-03
Chr01	7667484	7667978	181	3.630E-01	2.587E-01	-1.043E-01	8.311E-03
Chr01	7808245	7808320	19	2.149E-01	3.841E-01	1.692E-01	2.865E-02
Chr01	7936275	7936697	82	5.330E-01	4.032E-01	-1.298E-01	7.392E-03
Chr01	8119255	8119518	55	3.642E-01	2.640E-01	-1.002E-01	3.542E-02
Chr01	8193877	8193984	27	2.592E-01	1.479E-01	-1.113E-01	2.364E-02
Chr01	8336829	8337112	46	2.865E-01	1.476E-01	-1.389E-01	6.000E-07
Chr01	9557519	9558002	197	2.809E-01	1.656E-01	-1.153E-01	2.816E-02
Chr01	10281475	10281565	16	3.519E-01	1.634E-01	-1.885E-01	6.037E-03
Chr01	10285544	10285647	22	3.810E-01	1.908E-01	-1.902E-01	4.286E-04
Chr01	10596878	10597351	180	4.436E-01	2.940E-01	-1.497E-01	2.000E-07
Chr01	11166065	11166170	31	2.599E-01	3.652E-01	1.053E-01	1.856E-02
Chr01	11828893	11829374	178	2.864E-01	1.688E-01	-1.176E-01	0.000E+00
Chr01	12065468	12065956	55	2.721E-01	1.247E-01	-1.473E-01	1.402E-03
Chr01	12179721	12180215	135	2.635E-01	1.491E-01	-1.144E-01	2.947E-03
Chr01	12329407	12329903	136	4.554E-01	2.952E-01	-1.601E-01	8.900E-05
Chr01	12341360	12341857	143	2.923E-01	1.756E-01	-1.166E-01	4.539E-04
Chr01	12408809	12409282	156	3.699E-01	2.513E-01	-1.186E-01	1.001E-04
Chr01	12943412	12943910	111	6.833E-02	2.588E-01	1.905E-01	3.540E-05
Chr01	12968075	12968178	18	3.978E-01	2.308E-01	-1.670E-01	3.252E-03

Chr01	12970953	12971437	77	3.036E-01	2.024E-01	-1.012E-01	4.475E-02
Chr01	13020827	13020999	36	3.699E-01	2.487E-01	-1.212E-01	4.598E-03
Chr01	13519246	13519729	140	3.153E-01	2.072E-01	-1.081E-01	6.419E-04
Chr01	13657779	13658272	162	4.560E-01	2.719E-01	-1.840E-01	9.450E-05
Chr01	13746071	13746540	134	2.704E-01	1.627E-01	-1.077E-01	1.000E-07
Chr01	14015455	14015946	183	2.546E-01	1.378E-01	-1.168E-01	1.450E-05
Chr01	14027423	14027902	130	3.735E-01	2.623E-01	-1.112E-01	4.389E-04
Chr01	14079323	14079448	23	1.878E-01	1.075E-03	-1.867E-01	3.730E-05
Chr01	14254957	14255454	144	2.298E-01	3.742E-01	1.444E-01	3.389E-02
Chr01	14523973	14524460	115	4.531E-01	3.467E-01	-1.065E-01	1.202E-02
Chr01	14539769	14540260	141	1.924E-01	5.285E-02	-1.395E-01	3.040E-03
Chr01	14569693	14570186	144	3.400E-01	2.215E-01	-1.185E-01	2.276E-04
Chr01	14756093	14756590	195	1.712E-01	5.779E-02	-1.134E-01	4.729E-02
Chr01	14852174	14852668	145	2.508E-01	1.436E-01	-1.072E-01	2.298E-03
Chr01	14852675	14853159	199	3.045E-01	1.128E-02	-2.932E-01	0.000E+00
Chr01	14853165	14853660	95	2.644E-01	9.064E-02	-1.737E-01	3.358E-03
Chr01	15321869	15322367	106	2.874E-01	1.403E-01	-1.471E-01	1.758E-03
Chr02	107586	108071	137	3.589E-01	2.460E-01	-1.129E-01	7.894E-03
Chr02	111564	112061	220	2.386E-01	3.463E-01	1.076E-01	1.682E-02
Chr02	1237103	1237600	169	3.369E-01	2.170E-01	-1.199E-01	1.270E-05
Chr02	1383784	1384238	56	2.213E-01	1.056E-01	-1.157E-01	4.100E-05
Chr02	1416100	1416578	119	1.918E-01	4.860E-02	-1.432E-01	4.370E-03
Chr02	1692612	1693105	149	2.450E-01	1.420E-01	-1.030E-01	5.000E-07
Chr02	2652868	2653238	107	3.265E-01	2.256E-01	-1.009E-01	1.027E-03
Chr02	3324004	3324491	156	2.605E-01	1.531E-01	-1.074E-01	2.000E-07
Chr02	3593627	3594122	162	3.667E-01	2.601E-01	-1.066E-01	3.040E-05
Chr02	4130394	4130889	177	5.702E-01	4.530E-01	-1.172E-01	7.715E-03
Chr02	4532860	4533109	57	4.088E-01	3.037E-01	-1.051E-01	4.395E-03
Chr02	4593978	4594469	140	4.952E-01	3.596E-01	-1.356E-01	0.000E+00
Chr02	5030553	5031043	95	1.661E-01	4.206E-01	2.545E-01	8.541E-03
Chr02	5075260	5075758	113	5.215E-01	3.980E-01	-1.235E-01	7.370E-05
Chr02	5078738	5079215	79	3.988E-01	2.574E-01	-1.415E-01	4.000E-07
Chr02	6054021	6054517	159	2.598E-01	9.430E-02	-1.655E-01	1.400E-06
Chr02	6124786	6125278	181	4.208E-01	3.096E-01	-1.112E-01	1.800E-06
Chr02	6627936	6628424	108	5.677E-01	4.638E-01	-1.040E-01	1.640E-05
Chr02	6809825	6810310	152	4.132E-01	3.015E-01	-1.117E-01	4.277E-03
Chr02	6911569	6912044	90	5.235E-01	3.398E-01	-1.837E-01	1.907E-03
Chr02	6912649	6913145	172	2.705E-01	1.192E-01	-1.513E-01	1.653E-02
Chr02	7450260	7450380	19	3.025E-01	1.110E-01	-1.915E-01	2.603E-03
Chr02	7683016	7683329	52	4.630E-01	3.500E-01	-1.129E-01	4.492E-04
Chr02	8108002	8108488	141	3.383E-01	2.317E-01	-1.065E-01	1.060E-05
Chr02	8417566	8418053	129	4.610E-01	2.836E-01	-1.774E-01	7.200E-05
Chr02	8783327	8783463	32	3.405E-01	1.609E-01	-1.796E-01	2.521E-02
Chr02	8808225	8808722	188	3.611E-01	2.596E-01	-1.016E-01	3.070E-05
Chr02	9130466	9130947	74	3.374E-01	2.223E-01	-1.151E-01	1.000E-07
Chr02	9322694	9323170	162	4.524E-01	3.468E-01	-1.056E-01	2.380E-03
Chr02	9523130	9523628	150	3.140E-01	1.650E-01	-1.490E-01	1.776E-02

Chr02	12593924	12594418	166	3.926E-01	2.881E-01	-1.045E-01	1.842E-04
Chr02	12685498	12685836	45	4.387E-01	3.157E-01	-1.230E-01	1.263E-02
Chr02	12802658	12802924	89	2.953E-01	1.479E-01	-1.474E-01	5.300E-06
Chr02	13166834	13167297	146	7.638E-02	1.784E-01	1.021E-01	1.845E-02
Chr02	13383471	13383967	207	3.979E-01	2.924E-01	-1.055E-01	8.947E-03
Chr02	14781116	14781394	32	3.868E-01	2.593E-01	-1.275E-01	8.681E-03
Chr02	15176288	15176785	123	2.529E-01	1.335E-01	-1.194E-01	2.093E-04
Chr02	16103877	16104368	131	3.178E-01	2.160E-01	-1.018E-01	3.993E-03
Chr02	16768410	16768590	32	3.221E-01	2.057E-01	-1.164E-01	3.333E-03
Chr03	337382	337817	119	1.980E-01	9.387E-02	-1.041E-01	3.564E-02
Chr03	430350	430803	79	3.785E-01	2.399E-01	-1.386E-01	3.013E-02
Chr03	436131	436555	42	3.711E-01	1.997E-01	-1.714E-01	3.100E-06
Chr03	439674	440088	90	4.256E-01	3.002E-01	-1.254E-01	8.914E-03
Chr03	454546	455014	102	9.558E-02	2.950E-01	1.994E-01	3.514E-04
Chr03	786090	786582	209	2.923E-01	1.800E-01	-1.123E-01	1.641E-02
Chr03	791774	792235	94	4.725E-01	3.629E-01	-1.096E-01	1.307E-02
Chr03	989910	990401	127	1.992E-01	7.293E-02	-1.263E-01	8.420E-04
Chr03	999294	999706	65	2.861E-01	5.409E-02	-2.320E-01	0.000E+00
Chr03	1014842	1015338	138	1.961E-01	5.345E-02	-1.426E-01	2.948E-02
Chr03	1119564	1120060	140	3.632E-01	2.544E-01	-1.088E-01	4.105E-02
Chr03	1430549	1431025	110	3.810E-01	2.701E-01	-1.109E-01	3.353E-04
Chr03	1438028	1438518	165	3.021E-01	1.999E-01	-1.021E-01	2.291E-03
Chr03	1947061	1947549	100	5.538E-01	4.311E-01	-1.227E-01	0.000E+00
Chr03	2681350	2681844	160	3.146E-01	1.900E-01	-1.246E-01	5.450E-05
Chr03	3485226	3485722	61	4.815E-01	2.159E-01	-2.655E-01	3.025E-03
Chr03	4917288	4917732	162	4.574E-01	3.489E-01	-1.086E-01	6.000E-07
Chr03	5178862	5179173	58	4.124E-01	2.797E-01	-1.327E-01	5.200E-05
Chr03	5207646	5207920	40	3.582E-01	1.545E-01	-2.036E-01	1.500E-06
Chr03	5663825	5664318	155	3.714E-01	2.618E-01	-1.096E-01	3.348E-04
Chr03	5771164	5771462	30	6.429E-01	4.082E-01	-2.347E-01	6.424E-04
Chr03	5793134	5793557	62	4.700E-02	1.827E-01	1.357E-01	0.000E+00
Chr03	5842006	5842479	123	4.056E-01	2.927E-01	-1.129E-01	7.582E-03
Chr03	6363018	6363507	196	2.557E-01	1.373E-01	-1.185E-01	1.072E-04
Chr03	7021543	7022034	126	3.308E-01	1.803E-01	-1.505E-01	3.000E-07
Chr03	7617502	7617994	95	3.051E-01	1.525E-01	-1.526E-01	2.504E-03
Chr03	7703595	7704080	203	3.047E-01	1.756E-01	-1.291E-01	2.842E-03
Chr03	8941410	8941902	171	3.959E-01	2.935E-01	-1.024E-01	0.000E+00
Chr03	9516251	9516738	152	5.723E-01	4.288E-01	-1.435E-01	3.173E-03
Chr03	10016713	10017002	53	1.235E-01	1.632E-02	-1.072E-01	8.165E-03
Chr03	10025173	10025351	27	2.507E-01	3.532E-01	1.025E-01	4.101E-02
Chr03	10330707	10331199	146	9.417E-02	2.030E-01	1.089E-01	3.867E-03
Chr03	10531563	10532050	187	2.306E-01	9.854E-02	-1.321E-01	1.305E-02
Chr03	10655115	10655600	143	4.715E-01	3.385E-01	-1.330E-01	6.375E-04
Chr03	12326408	12326903	189	2.960E-01	1.743E-01	-1.217E-01	3.107E-03
Chr03	12735238	12735727	178	3.146E-01	2.028E-01	-1.118E-01	9.115E-04
Chr03	12761697	12762193	203	3.152E-01	2.098E-01	-1.053E-01	6.553E-04
Chr03	12873542	12874039	163	3.982E-01	2.668E-01	-1.314E-01	4.877E-03

Chr03	13065585	13066082	123	5.019E-01	4.000E-01	-1.019E-01	8.792E-03
Chr03	13167073	13167570	164	2.281E-01	9.870E-02	-1.294E-01	1.353E-02
Chr03	13260564	13261059	123	2.635E-01	1.626E-01	-1.010E-01	6.790E-05
Chr03	13865365	13865827	195	3.822E-01	2.342E-01	-1.480E-01	4.000E-07
Chr04	165309	165771	100	4.795E-01	3.556E-01	-1.239E-01	1.600E-06
Chr04	205600	206090	193	1.200E-01	2.738E-01	1.538E-01	7.131E-03
Chr04	368210	368701	161	2.232E-01	8.149E-02	-1.417E-01	3.570E-02
Chr04	408944	409153	25	4.700E-01	3.325E-01	-1.375E-01	1.355E-02
Chr04	506929	507207	45	3.334E-01	2.225E-01	-1.109E-01	2.526E-02
Chr04	549401	549872	175	4.484E-01	2.973E-01	-1.511E-01	2.100E-06
Chr04	693558	693992	115	4.857E-01	3.535E-01	-1.322E-01	1.010E-03
Chr04	1617202	1617534	79	5.906E-01	4.003E-01	-1.903E-01	4.622E-03
Chr04	1732628	1733119	206	3.250E-01	2.004E-01	-1.246E-01	0.000E+00
Chr04	2634814	2635308	193	4.056E-01	3.051E-01	-1.004E-01	4.291E-04
Chr04	2888247	2888684	52	3.751E-01	2.615E-01	-1.136E-01	2.578E-04
Chr04	3609919	3610413	88	3.209E-01	2.083E-01	-1.127E-01	4.859E-03
Chr04	3713624	3714089	75	5.375E-01	3.957E-01	-1.419E-01	1.667E-04
Chr04	3767908	3768212	48	3.657E-01	2.534E-01	-1.122E-01	1.000E-07
Chr04	3770634	3771056	65	1.751E-01	3.110E-01	1.359E-01	2.797E-03
Chr04	3938502	3938998	153	3.991E-01	2.704E-01	-1.287E-01	7.587E-03
Chr04	4484369	4484834	159	2.215E-01	1.034E-01	-1.181E-01	1.566E-02
Chr04	5114298	5114781	108	4.344E-01	3.302E-01	-1.042E-01	1.991E-03
Chr04	5491866	5492156	101	2.811E-01	1.518E-01	-1.293E-01	1.713E-03
Chr04	5637187	5637656	166	2.164E-01	7.651E-02	-1.399E-01	1.627E-03
Chr04	5830362	5830655	66	4.144E-01	2.897E-01	-1.247E-01	5.758E-03
Chr04	5979622	5979863	44	3.425E-01	2.144E-01	-1.281E-01	1.303E-02
Chr04	6074335	6074630	69	5.383E-01	2.802E-01	-2.581E-01	3.252E-02
Chr04	6081961	6082237	74	3.545E-01	1.860E-01	-1.685E-01	2.047E-02
Chr04	6085537	6085638	18	5.336E-01	3.953E-01	-1.382E-01	4.782E-02
Chr04	6234898	6235264	52	3.939E-01	2.929E-01	-1.010E-01	1.247E-03
Chr04	6348031	6348233	38	3.696E-01	1.932E-01	-1.764E-01	2.357E-04
Chr04	6768704	6769179	83	3.949E-03	1.416E-01	1.377E-01	4.930E-05
Chr04	6816277	6816727	84	3.131E-01	1.470E-01	-1.661E-01	1.842E-04
Chr04	6878693	6879176	137	1.267E-01	2.753E-03	-1.240E-01	1.068E-02
Chr04	6955751	6956065	50	2.652E-01	1.205E-03	-2.640E-01	0.000E+00
Chr04	6987429	6987796	56	2.075E-01	2.239E-04	-2.073E-01	0.000E+00
Chr04	7092427	7092678	51	1.969E-01	7.458E-02	-1.223E-01	3.466E-02
Chr04	7999448	7999831	60	4.704E-02	1.771E-01	1.300E-01	1.140E-02
Chr04	8039476	8039886	113	4.938E-01	3.795E-01	-1.143E-01	2.461E-03
Chr04	8068583	8068729	37	1.011E-01	2.230E-04	-1.009E-01	2.147E-04
Chr04	8069457	8069672	58	2.077E-01	5.271E-02	-1.550E-01	6.006E-04
Chr04	8155354	8155725	79	1.111E-01	2.654E-01	1.543E-01	9.470E-04
Chr04	8279855	8280296	48	4.580E-01	7.004E-01	2.423E-01	2.917E-04
Chr04	8549060	8549492	154	5.164E-01	4.130E-01	-1.034E-01	2.111E-03
Chr04	9450831	9451194	72	3.913E-01	5.108E-01	1.194E-01	2.516E-03
Chr04	9650834	9651319	76	4.747E-01	3.247E-01	-1.500E-01	5.167E-04
Chr04	9652312	9652802	93	2.842E-01	1.530E-01	-1.312E-01	3.663E-04

Chr04	9653303	9653784	61	1.827E-01	4.403E-02	-1.387E-01	2.455E-03
Chr04	9934125	9934604	138	3.497E-01	4.961E-01	1.464E-01	8.576E-03
Chr04	9934612	9935089	95	5.869E-01	7.706E-01	1.837E-01	5.014E-04
Chr04	9940897	9941372	166	5.927E-01	4.753E-01	-1.175E-01	2.402E-02
Chr04	9942824	9943306	240	4.598E-01	5.915E-01	1.317E-01	4.379E-03
Chr04	9949791	9949964	71	3.886E-01	5.038E-01	1.152E-01	4.818E-02
Chr04	9953410	9953857	164	4.231E-01	5.781E-01	1.550E-01	1.385E-02
Chr04	9990354	9990799	145	4.421E-01	5.649E-01	1.228E-01	2.784E-02
Chr04	10045567	10045946	63	1.058E-01	2.167E-01	1.108E-01	5.196E-04
Chr04	10067103	10067442	55	1.553E-01	2.629E-04	-1.550E-01	1.320E-05
Chr04	11061778	11062257	152	2.628E-01	1.345E-01	-1.283E-01	1.777E-03
Chr04	11165538	11166033	234	1.150E-01	3.246E-03	-1.118E-01	4.089E-02
Chr04	11482149	11482643	151	2.373E-01	3.681E-01	1.308E-01	1.606E-02
Chr04	12931778	12932270	207	1.716E-01	5.556E-02	-1.161E-01	2.991E-03
Chr04	13708156	13708653	152	1.837E-01	7.422E-02	-1.095E-01	3.303E-02
Chr04	13857561	13858046	126	2.717E-01	1.510E-01	-1.207E-01	2.379E-03
Chr04	15065883	15066371	163	2.694E-01	1.444E-01	-1.251E-01	1.562E-03
Chr04	15066375	15066863	152	2.844E-01	1.345E-01	-1.499E-01	1.000E-07
Chr04	15088933	15089423	110	4.476E-01	3.352E-01	-1.124E-01	4.780E-05
Chr05	216978	217453	115	1.554E-01	4.951E-02	-1.059E-01	6.663E-04
Chr05	389841	390338	205	4.128E-01	2.690E-01	-1.438E-01	1.110E-02
Chr05	602841	603335	198	2.551E-01	1.480E-01	-1.071E-01	4.300E-06
Chr05	998875	999070	19	5.237E-01	1.890E-01	-3.347E-01	1.150E-05
Chr05	1089761	1090234	130	4.005E-01	2.784E-01	-1.221E-01	9.539E-03
Chr05	1421852	1422272	120	7.585E-02	2.010E-01	1.251E-01	3.948E-02
Chr05	1550215	1550707	127	5.133E-01	4.051E-01	-1.082E-01	1.645E-04
Chr05	1781563	1782052	155	2.567E-01	1.563E-01	-1.003E-01	0.000E+00
Chr05	1834801	1835059	92	3.972E-01	2.936E-01	-1.037E-01	8.638E-03
Chr05	2007014	2007506	71	2.386E-01	1.068E-01	-1.318E-01	1.795E-04
Chr05	2241513	2241970	113	4.555E-01	3.009E-01	-1.547E-01	2.072E-04
Chr05	2751804	2752276	72	5.840E-01	4.436E-01	-1.404E-01	2.440E-05
Chr05	2767181	2767657	77	2.449E-01	1.323E-01	-1.125E-01	8.729E-03
Chr05	3002225	3002692	91	2.801E-01	1.800E-01	-1.001E-01	9.400E-05
Chr05	3284144	3284281	33	2.324E-01	3.381E-01	1.058E-01	4.911E-02
Chr05	3799388	3799860	180	2.348E-01	1.334E-01	-1.014E-01	0.000E+00
Chr05	4717151	4717570	61	3.995E-01	2.782E-01	-1.212E-01	1.200E-06
Chr05	4719734	4720227	169	2.931E-01	1.888E-01	-1.042E-01	2.382E-02
Chr05	4724238	4724728	178	2.712E-01	1.060E-01	-1.652E-01	4.646E-03
Chr05	5000239	5000727	153	1.281E-01	2.946E-01	1.665E-01	1.351E-02
Chr05	5910848	5911324	151	5.309E-01	4.146E-01	-1.163E-01	3.509E-03
Chr05	5971866	5972359	163	2.345E-01	1.332E-01	-1.013E-01	1.430E-04
Chr05	6144769	6145264	147	3.158E-01	2.122E-01	-1.036E-01	8.600E-06
Chr05	6318276	6318756	144	3.127E-01	2.083E-01	-1.044E-01	2.433E-02
Chr05	7029285	7029773	142	1.628E-01	3.626E-02	-1.265E-01	1.196E-02
Chr05	7111408	7111886	152	3.965E-01	2.951E-01	-1.013E-01	4.243E-03
Chr05	7709054	7709534	70	4.667E-01	2.726E-01	-1.940E-01	2.790E-04
Chr05	8206017	8206505	78	5.932E-01	3.970E-01	-1.961E-01	2.780E-05

Chr05	8908821	8909303	125	4.939E-01	6.380E-01	1.441E-01	5.457E-04
Chr05	8968116	8968608	171	3.292E-01	2.221E-01	-1.071E-01	0.000E+00
Chr05	9168160	9168601	51	4.102E-01	3.079E-01	-1.024E-01	8.340E-03
Chr05	9191651	9191814	29	7.005E-01	5.841E-01	-1.165E-01	3.427E-03
Chr05	9497525	9497606	28	6.018E-01	4.707E-01	-1.311E-01	2.183E-02
Chr05	9766434	9766564	41	6.123E-01	4.345E-01	-1.778E-01	3.133E-03
Chr05	9782154	9782545	89	4.917E-01	7.063E-01	2.146E-01	1.302E-02
Chr05	9832099	9832531	52	7.144E-01	3.440E-01	-3.704E-01	0.000E+00
Chr05	9834023	9834141	19	9.300E-01	7.849E-01	-1.450E-01	4.978E-03
Chr05	9842057	9842385	50	7.526E-01	8.800E-01	1.273E-01	7.594E-03
Chr05	9847015	9847342	53	3.633E-01	2.377E-01	-1.256E-01	1.045E-03
Chr05	9851630	9852035	114	4.979E-01	7.173E-01	2.193E-01	9.880E-05
Chr05	9857812	9858303	111	6.011E-01	7.147E-01	1.136E-01	1.778E-02
Chr05	9861465	9861786	61	7.350E-01	9.182E-01	1.832E-01	5.057E-03
Chr05	9868735	9868773	20	6.604E-01	4.568E-01	-2.036E-01	5.457E-04
Chr05	9869410	9869657	30	8.562E-01	5.752E-01	-2.809E-01	2.151E-02
Chr05	9871929	9872235	37	5.064E-01	7.921E-01	2.856E-01	0.000E+00
Chr05	9873436	9873814	60	6.069E-01	4.106E-01	-1.963E-01	3.524E-04
Chr05	9881185	9881288	51	4.016E-01	5.154E-01	1.138E-01	9.793E-03
Chr05	9883396	9883840	60	5.544E-01	4.260E-01	-1.284E-01	7.621E-03
Chr05	9887662	9888029	47	6.818E-01	8.363E-01	1.545E-01	1.870E-03
Chr05	9888401	9888767	144	4.989E-01	6.140E-01	1.150E-01	2.249E-02
Chr05	9891400	9891447	22	4.812E-01	2.802E-01	-2.010E-01	2.975E-03
Chr05	9979107	9979483	132	5.382E-01	6.767E-01	1.384E-01	4.392E-02
Chr05	9979495	9979927	104	6.256E-01	8.138E-01	1.882E-01	1.441E-03
Chr05	10397402	10397881	264	5.040E-01	6.182E-01	1.142E-01	5.681E-03
Chr05	10398845	10399326	177	4.943E-01	7.040E-01	2.097E-01	2.710E-04
Chr05	10399491	10399801	30	7.117E-01	8.181E-01	1.064E-01	1.830E-03
Chr05	10405038	10405494	254	5.381E-01	6.674E-01	1.293E-01	2.338E-03
Chr05	10409325	10409761	147	4.342E-01	6.578E-01	2.236E-01	1.750E-04
Chr05	10410242	10410697	201	4.770E-01	6.004E-01	1.234E-01	2.320E-02
Chr05	10415165	10415662	236	4.431E-01	5.692E-01	1.261E-01	5.439E-03
Chr05	10441627	10442104	114	6.589E-01	8.080E-01	1.491E-01	1.940E-03
Chr05	10442600	10443080	249	5.227E-01	6.320E-01	1.093E-01	1.665E-02
Chr05	10512671	10512788	19	9.046E-01	7.301E-01	-1.745E-01	1.397E-03
Chr05	10514439	10514633	86	3.372E-01	5.063E-01	1.691E-01	3.080E-04
Chr05	10516988	10517207	23	9.245E-01	6.293E-01	-2.951E-01	3.000E-07
Chr05	10523107	10523477	36	8.436E-01	7.021E-01	-1.415E-01	1.043E-03
Chr05	10529013	10529427	86	7.840E-01	4.455E-01	-3.385E-01	5.000E-07
Chr05	10537132	10537424	99	6.824E-01	9.509E-01	2.685E-01	2.800E-06
Chr05	10538085	10538482	74	6.001E-01	7.543E-01	1.542E-01	3.456E-02
Chr05	10587566	10587783	37	7.097E-01	5.842E-01	-1.254E-01	3.761E-02
Chr05	11269686	11270180	204	3.864E-01	2.556E-01	-1.308E-01	1.000E-07
Chr05	11476595	11477083	178	3.089E-01	1.777E-01	-1.312E-01	5.727E-03
Chr05	11839095	11839533	152	2.855E-01	1.657E-01	-1.198E-01	2.160E-02
Chr05	11906879	11907373	107	6.725E-01	5.400E-01	-1.325E-01	3.293E-03
Chr05	12332743	12333189	125	1.779E-01	4.922E-02	-1.287E-01	1.715E-02

Chr05	12349042	12349432	115	2.092E-01	9.353E-02	-1.157E-01	9.210E-05
Chr05	12349438	12349830	94	2.205E-01	9.120E-02	-1.293E-01	1.787E-04
Chr05	12500441	12500916	129	2.121E-01	3.517E-01	1.396E-01	8.227E-03
Chr05	12618940	12619334	88	4.001E-01	2.529E-01	-1.471E-01	1.084E-03
Chr05	14024033	14024508	64	5.111E-01	3.829E-01	-1.281E-01	7.096E-04
Chr05	14216219	14216711	142	4.318E-01	2.515E-01	-1.803E-01	3.804E-04
Chr05	14325080	14325461	59	3.357E-01	2.277E-01	-1.080E-01	1.213E-03
Chr05	14328411	14328608	39	2.729E-01	9.341E-02	-1.794E-01	1.540E-05
Chr05	14372052	14372514	173	3.327E-01	2.063E-01	-1.265E-01	1.000E-07
Chr05	14750409	14750873	105	1.488E-01	1.056E-02	-1.383E-01	0.000E+00
Chr05	15634244	15634732	176	3.580E-01	2.194E-01	-1.386E-01	1.000E-07
Chr05	15833173	15833671	208	3.599E-01	2.403E-01	-1.196E-01	1.406E-02
Chr05	15906566	15907061	157	3.743E-01	2.454E-01	-1.289E-01	0.000E+00
Chr05	15977097	15977594	210	5.104E-01	4.011E-01	-1.094E-01	2.493E-02
Chr05	16062011	16062510	136	3.277E-01	2.123E-01	-1.154E-01	4.300E-03
Chr05	16204717	16205203	150	4.015E-01	2.701E-01	-1.315E-01	2.144E-04
Chr05	16218647	16219144	171	2.758E-01	1.538E-01	-1.220E-01	1.363E-03
Chr05	16601261	16601621	59	2.755E-01	1.541E-01	-1.214E-01	1.370E-05
Chr05	17165157	17165607	74	4.780E-01	3.760E-01	-1.020E-01	1.356E-04
Chr06	550088	550573	194	2.855E-01	1.734E-01	-1.122E-01	0.000E+00
Chr06	875139	875625	127	2.216E-01	6.009E-02	-1.615E-01	8.200E-06
Chr06	1020152	1020643	210	3.260E-01	2.205E-01	-1.055E-01	3.569E-02
Chr06	1530378	1530873	97	4.617E-01	3.441E-01	-1.176E-01	2.000E-07
Chr06	1853997	1854144	30	1.661E-01	3.212E-01	1.551E-01	2.510E-03
Chr06	2276086	2276576	209	4.144E-01	3.117E-01	-1.027E-01	3.109E-02
Chr06	2390989	2391487	166	3.367E-01	2.235E-01	-1.132E-01	5.418E-04
Chr06	6497700	6497789	7	2.882E-01	1.234E-01	-1.647E-01	1.185E-03
Chr06	6996766	6997263	120	4.032E-01	3.003E-01	-1.029E-01	0.000E+00
Chr06	7183099	7183262	41	6.084E-01	3.910E-01	-2.174E-01	7.226E-03
Chr06	7545956	7546449	112	3.748E-01	2.307E-01	-1.441E-01	3.600E-06
Chr06	7778387	7778884	191	2.159E-01	1.132E-01	-1.027E-01	7.955E-04
Chr06	8159081	8159576	160	3.507E-01	2.480E-01	-1.027E-01	4.517E-03
Chr06	8681808	8682304	158	2.147E-01	1.062E-01	-1.085E-01	6.470E-05
Chr06	8909766	8910248	117	2.639E-02	1.852E-01	1.588E-01	2.000E-07
Chr06	8912181	8912657	161	4.160E-01	2.454E-01	-1.706E-01	5.529E-03
Chr06	9075228	9075642	66	6.579E-01	3.688E-01	-2.891E-01	2.000E-07
Chr06	9204862	9205358	157	4.150E-01	2.820E-01	-1.329E-01	1.950E-05
Chr06	9486012	9486508	85	4.764E-01	3.683E-01	-1.082E-01	1.840E-05
Chr06	9486511	9487008	151	3.575E-01	2.312E-01	-1.263E-01	0.000E+00
Chr06	9487010	9487507	193	4.282E-01	3.256E-01	-1.026E-01	0.000E+00
Chr06	9487520	9488005	128	4.021E-01	2.769E-01	-1.253E-01	3.000E-07
Chr06	9488012	9488504	180	4.102E-01	2.811E-01	-1.291E-01	0.000E+00
Chr06	9488512	9489006	157	3.978E-01	2.867E-01	-1.111E-01	0.000E+00
Chr06	9490507	9491006	211	4.153E-01	3.150E-01	-1.003E-01	1.500E-06
Chr06	9491009	9491503	207	3.931E-01	2.896E-01	-1.035E-01	0.000E+00
Chr06	9491509	9492005	205	4.298E-01	3.283E-01	-1.015E-01	1.690E-05
Chr06	9494007	9494498	197	2.816E-01	1.700E-01	-1.116E-01	0.000E+00

Chr06	10506620	10507067	120	2.799E-01	1.158E-01	-1.642E-01	2.008E-04
Chr06	10543041	10543526	142	3.384E-01	1.696E-01	-1.688E-01	2.408E-03
Chr06	10598337	10598823	186	4.839E-01	3.510E-01	-1.329E-01	4.521E-04
Chr06	11077291	11077786	106	5.877E-01	4.244E-01	-1.633E-01	1.700E-06
Chr06	11283425	11283909	137	4.676E-01	5.752E-01	1.076E-01	2.298E-02
Chr06	11772454	11772601	37	1.342E-01	2.396E-01	1.054E-01	1.047E-02
Chr06	12470340	12470821	108	2.427E-01	1.416E-01	-1.011E-01	2.869E-04
Chr06	12474932	12475068	25	4.749E-01	1.853E-01	-2.896E-01	0.000E+00
Chr06	12628227	12628714	109	3.750E-01	2.749E-01	-1.001E-01	5.000E-06
Chr06	13015607	13016014	71	2.032E-01	9.476E-02	-1.084E-01	0.000E+00
Chr06	13543836	13544082	52	2.261E-01	8.462E-02	-1.415E-01	7.079E-04
Chr06	14269569	14270046	172	3.452E-01	2.075E-01	-1.377E-01	1.424E-02
Chr06	14339188	14339679	151	8.685E-02	3.123E-01	2.254E-01	3.194E-03
Chr06	14993627	14994071	127	7.668E-02	1.806E-01	1.039E-01	3.953E-02
Chr06	15646292	15646786	215	3.909E-01	1.730E-01	-2.179E-01	9.859E-04
Chr06	16014720	16015215	177	3.112E-01	2.047E-01	-1.065E-01	6.771E-03
Chr06	16167355	16167844	176	3.673E-01	2.668E-01	-1.005E-01	3.132E-04
Chr06	17055439	17055909	170	3.728E-01	2.688E-01	-1.040E-01	2.388E-04
Chr06	17349392	17349880	195	4.505E-01	3.323E-01	-1.182E-01	7.179E-03
Chr06	17540810	17541301	107	3.469E-01	2.405E-01	-1.064E-01	1.940E-05
Chr06	17929413	17929903	163	2.108E-01	1.103E-01	-1.005E-01	0.000E+00
Chr06	18547182	18547663	212	3.570E-01	2.264E-01	-1.306E-01	5.955E-04
Chr06	18949052	18949520	120	6.199E-01	5.119E-01	-1.080E-01	4.080E-03
Chr06	19198191	19198685	174	3.655E-01	2.306E-01	-1.348E-01	5.288E-04
Chr06	19534531	19535021	175	4.344E-01	2.578E-01	-1.767E-01	1.956E-02
Chr06	19563442	19563932	154	3.211E-01	1.319E-01	-1.891E-01	0.000E+00
Chr06	19570775	19571268	92	4.651E-01	3.387E-01	-1.264E-01	4.936E-02
Chr06	19607833	19608314	152	3.849E-01	2.815E-01	-1.034E-01	1.614E-04
Chr07	451459	451956	142	3.104E-01	1.551E-01	-1.554E-01	4.915E-02
Chr07	1002848	1003338	158	2.354E-01	1.295E-01	-1.059E-01	3.248E-03
Chr07	1010417	1010911	169	3.615E-01	2.394E-01	-1.221E-01	1.195E-04
Chr07	2036805	2037091	74	4.788E-01	3.471E-01	-1.317E-01	5.099E-03
Chr07	2517761	2518246	162	3.454E-01	2.141E-01	-1.313E-01	4.000E-07
Chr07	2599696	2600095	136	2.856E-01	1.846E-01	-1.009E-01	8.000E-07
Chr07	2643756	2644249	153	2.036E-01	1.028E-01	-1.008E-01	9.590E-05
Chr07	2678661	2679155	168	2.598E-01	1.569E-01	-1.029E-01	2.000E-07
Chr07	2842233	2842727	145	3.192E-01	2.177E-01	-1.015E-01	1.274E-04
Chr07	3175211	3175699	106	4.881E-01	3.331E-01	-1.550E-01	4.005E-04
Chr07	3320035	3320513	209	4.832E-01	3.485E-01	-1.348E-01	1.109E-03
Chr07	3776869	3777351	99	4.006E-01	2.773E-01	-1.233E-01	8.720E-05
Chr07	3906873	3907366	189	2.349E-01	1.149E-01	-1.201E-01	1.759E-02
Chr07	4071426	4071919	223	4.680E-01	3.383E-01	-1.297E-01	1.279E-04
Chr07	4271493	4271986	153	2.670E-01	1.496E-01	-1.174E-01	1.200E-06
Chr07	4504164	4504647	156	4.083E-01	2.970E-01	-1.113E-01	2.477E-03
Chr07	4580636	4581101	52	2.511E-01	1.491E-01	-1.020E-01	0.000E+00
Chr07	4799817	4800283	34	3.342E-01	1.944E-01	-1.399E-01	1.188E-02
Chr07	5091189	5091683	150	2.684E-01	3.727E-01	1.044E-01	2.113E-03

Chr07	5273085	5273507	116	4.058E-01	3.021E-01	-1.037E-01	5.969E-04
Chr07	5307580	5307952	53	1.513E-01	3.106E-02	-1.202E-01	1.790E-05
Chr07	5352110	5352435	80	3.818E-01	2.787E-01	-1.031E-01	1.862E-03
Chr07	5434388	5434844	92	1.709E-01	2.623E-02	-1.446E-01	6.243E-03
Chr07	5703884	5704286	67	3.939E-01	5.356E-01	1.417E-01	3.132E-03
Chr07	5704782	5705166	126	3.602E-01	4.671E-01	1.069E-01	6.600E-05
Chr07	5710895	5711310	67	7.780E-01	6.453E-01	-1.327E-01	1.164E-03
Chr07	5736368	5736651	142	6.095E-01	4.900E-01	-1.195E-01	7.109E-03
Chr07	5737848	5738265	192	4.496E-01	5.691E-01	1.195E-01	9.375E-03
Chr07	5750609	5751095	253	5.093E-01	6.365E-01	1.273E-01	5.802E-03
Chr07	5759071	5759541	241	5.157E-01	6.379E-01	1.221E-01	5.978E-04
Chr07	5759548	5760021	215	5.172E-01	6.811E-01	1.639E-01	4.875E-03
Chr07	5761782	5762254	109	3.907E-01	5.188E-01	1.280E-01	6.915E-03
Chr07	5767058	5767437	117	3.648E-01	5.225E-01	1.577E-01	1.157E-02
Chr07	5788977	5789115	23	1.304E-01	2.450E-01	1.146E-01	1.553E-02
Chr07	5806406	5806796	102	5.338E-01	6.788E-01	1.450E-01	1.965E-02
Chr07	5806910	5807362	228	4.348E-01	5.377E-01	1.029E-01	1.624E-02
Chr07	5820293	5820758	265	5.113E-01	6.258E-01	1.145E-01	1.682E-02
Chr07	5821229	5821690	226	5.090E-01	6.540E-01	1.451E-01	5.970E-04
Chr07	5833202	5833336	16	7.868E-01	9.453E-01	1.585E-01	2.745E-03
Chr07	5874990	5875424	141	5.087E-01	6.182E-01	1.095E-01	3.105E-02
Chr07	5887664	5888109	223	4.647E-01	5.937E-01	1.289E-01	4.279E-03
Chr07	5919756	5919834	21	6.629E-01	3.550E-01	-3.078E-01	3.980E-05
Chr07	5919954	5920309	136	3.642E-01	4.723E-01	1.081E-01	1.376E-02
Chr07	5930931	5931340	187	7.097E-01	5.248E-01	-1.849E-01	2.491E-04
Chr07	5936188	5936293	41	4.883E-01	3.882E-01	-1.001E-01	4.008E-02
Chr07	5948346	5948690	136	4.575E-01	5.800E-01	1.225E-01	1.858E-03
Chr07	6277176	6277652	178	6.537E-01	7.642E-01	1.105E-01	4.937E-03
Chr07	6284852	6285326	182	5.490E-01	6.954E-01	1.465E-01	2.611E-04
Chr07	6291929	6292264	120	5.350E-01	6.843E-01	1.493E-01	3.895E-03
Chr07	6462216	6462705	121	3.616E-01	2.535E-01	-1.081E-01	0.000E+00
Chr07	6501893	6502349	137	3.876E-01	2.855E-01	-1.021E-01	3.200E-06
Chr07	6516461	6516898	77	3.700E-01	2.368E-01	-1.331E-01	9.410E-05
Chr07	6624937	6625397	39	2.804E-02	1.387E-01	1.106E-01	1.770E-03
Chr07	6629952	6630146	20	4.717E-04	3.567E-01	3.562E-01	0.000E+00
Chr07	6660271	6660766	79	4.859E-01	3.751E-01	-1.108E-01	3.755E-03
Chr07	6964123	6964191	20	2.407E-01	8.435E-02	-1.564E-01	1.280E-02
Chr07	7378620	7379110	163	3.111E-01	1.865E-01	-1.246E-01	9.345E-03
Chr07	7441064	7441563	190	2.011E-01	1.010E-01	-1.001E-01	4.687E-02
Chr07	7442091	7442568	148	3.378E-01	2.278E-01	-1.100E-01	1.852E-03
Chr07	8200129	8200581	139	3.705E-01	2.612E-01	-1.093E-01	9.232E-04
Chr07	8709570	8709754	52	4.116E-01	5.294E-01	1.178E-01	1.434E-02
Chr07	10654624	10655118	174	3.849E-01	2.624E-01	-1.226E-01	2.270E-02
Chr07	10655119	10655605	185	4.610E-01	2.733E-01	-1.877E-01	6.370E-04
Chr07	10896398	10896888	112	3.953E-01	2.946E-01	-1.007E-01	1.012E-03
Chr07	10936405	10936830	119	4.089E-01	2.608E-01	-1.482E-01	4.218E-02
Chr07	10951505	10951994	150	3.126E-01	1.981E-01	-1.144E-01	1.046E-03

Chr07	11089188	11089677	170	4.386E-01	3.351E-01	-1.036E-01	1.710E-05
Chr07	11486108	11486603	146	4.071E-01	2.155E-01	-1.916E-01	4.262E-03
Chr07	11702528	11703023	136	2.444E-01	1.357E-01	-1.087E-01	3.266E-04
Chr07	11771605	11772098	178	2.796E-01	1.550E-01	-1.247E-01	1.584E-02
Chr07	11941297	11941776	124	4.437E-01	3.403E-01	-1.034E-01	4.426E-02
Chr07	12083446	12083942	162	5.283E-01	4.140E-01	-1.143E-01	5.610E-04
Chr07	12208242	12208732	133	3.320E-01	1.839E-01	-1.481E-01	5.246E-03
Chr08	313253	313751	201	7.299E-02	2.407E-01	1.678E-01	3.107E-04
Chr08	646750	647247	142	2.775E-01	1.560E-01	-1.215E-01	3.913E-04
Chr08	761800	762062	31	3.760E-01	2.680E-01	-1.080E-01	3.421E-03
Chr08	1099851	1100245	98	3.714E-01	2.686E-01	-1.028E-01	1.334E-02
Chr08	1256598	1257077	106	3.522E-01	2.391E-01	-1.130E-01	2.482E-04
Chr08	1441327	1441658	71	4.107E-01	2.970E-01	-1.137E-01	2.000E-07
Chr08	1664597	1665086	148	3.781E-02	1.409E-01	1.031E-01	1.327E-02
Chr08	1714088	1714566	173	3.201E-01	1.954E-01	-1.247E-01	6.427E-03
Chr08	1768051	1768546	136	3.374E-01	2.317E-01	-1.057E-01	2.300E-05
Chr08	2075817	2076270	127	3.087E-01	1.963E-01	-1.124E-01	7.932E-03
Chr08	2230473	2230662	27	2.604E-01	1.288E-01	-1.316E-01	5.770E-05
Chr08	2317484	2317976	198	2.021E-01	9.877E-02	-1.033E-01	4.394E-02
Chr08	2364143	2364633	156	2.615E-01	1.588E-01	-1.027E-01	3.016E-02
Chr08	2698281	2698772	162	2.518E-01	1.377E-01	-1.141E-01	5.414E-03
Chr08	3490602	3491093	178	1.962E-01	9.201E-02	-1.041E-01	1.898E-02
Chr08	3896356	3896840	117	1.500E-01	4.516E-02	-1.048E-01	4.031E-02
Chr08	3902404	3902775	69	5.038E-01	3.695E-01	-1.343E-01	6.731E-03
Chr08	4014969	4015466	179	2.247E-01	9.316E-02	-1.315E-01	4.740E-03
Chr08	4655997	4656489	144	4.666E-01	3.304E-01	-1.362E-01	3.735E-04
Chr08	4697495	4697976	113	4.201E-01	3.133E-01	-1.068E-01	2.605E-04
Chr08	5398038	5398471	80	5.092E-01	4.090E-01	-1.002E-01	3.709E-02
Chr08	5496840	5497332	140	1.332E-01	2.834E-02	-1.049E-01	3.040E-03
Chr08	6433863	6434350	165	3.922E-01	2.881E-01	-1.041E-01	9.707E-04
Chr08	6933718	6934196	77	4.451E-01	3.342E-01	-1.110E-01	8.830E-05
Chr08	6995979	6996393	89	3.365E-01	2.037E-01	-1.328E-01	0.000E+00
Chr08	8385081	8385510	114	3.768E-01	2.712E-01	-1.056E-01	2.066E-02
Chr08	8712073	8712556	167	3.462E-01	2.015E-01	-1.447E-01	2.209E-02
Chr08	8745017	8745507	164	2.676E-01	1.359E-01	-1.317E-01	3.900E-06
Chr08	9445984	9446480	143	3.833E-01	2.831E-01	-1.002E-01	1.353E-02
Chr08	9546102	9546579	156	9.161E-02	1.950E-01	1.034E-01	4.095E-02
Chr08	9655834	9656325	182	2.314E-01	1.167E-01	-1.147E-01	0.000E+00
Chr08	10151841	10152339	208	3.092E-01	1.422E-01	-1.670E-01	2.684E-04
Chr08	10223961	10224314	117	4.644E-01	3.579E-01	-1.066E-01	3.514E-02
Chr08	10759522	10760008	45	7.631E-01	6.477E-01	-1.154E-01	3.711E-03
Chr08	10811407	10811901	185	1.101E-01	9.009E-03	-1.011E-01	2.240E-02
Chr08	11019184	11019679	129	4.417E-01	3.056E-01	-1.361E-01	3.042E-02
Chr08	12360241	12360731	173	3.543E-01	2.303E-01	-1.240E-01	4.147E-03
Chr08	12774409	12774907	112	6.179E-01	5.178E-01	-1.002E-01	3.000E-07
Chr08	12968739	12969237	205	2.289E-01	9.050E-02	-1.384E-01	2.326E-03
Chr08	13272553	13273038	185	3.902E-01	2.506E-01	-1.395E-01	2.550E-05

Chr09	91400	91896	149	2.193E-03	1.819E-01	1.797E-01	2.032E-02
Chr09	206555	206678	28	1.528E-03	2.034E-01	2.019E-01	0.000E+00
Chr09	1618061	1618552	178	4.246E-01	3.201E-01	-1.045E-01	1.515E-03
Chr09	1744284	1744731	115	3.501E-01	2.419E-01	-1.083E-01	4.962E-03
Chr09	1745394	1745872	120	3.761E-01	2.484E-01	-1.276E-01	1.383E-03
Chr09	1752337	1752769	120	1.741E-01	2.760E-01	1.019E-01	6.772E-04
Chr09	2495053	2495443	56	3.980E-01	2.662E-01	-1.319E-01	5.931E-03
Chr09	3182963	3183451	152	3.286E-01	2.062E-01	-1.223E-01	1.559E-02
Chr09	3190881	3191371	146	3.513E-01	2.342E-01	-1.170E-01	4.260E-05
Chr09	3342214	3342475	33	4.233E-01	3.222E-01	-1.011E-01	1.575E-02
Chr09	3485999	3486497	142	6.276E-01	5.215E-01	-1.061E-01	2.806E-03
Chr09	4751170	4751663	185	2.877E-01	1.554E-01	-1.323E-01	3.391E-03
Chr09	4763107	4763600	173	2.673E-01	1.210E-01	-1.463E-01	2.251E-02
Chr09	5525256	5525359	23	5.814E-01	4.550E-01	-1.264E-01	1.764E-02
Chr09	5760706	5761200	128	2.942E-01	1.871E-01	-1.071E-01	3.360E-05
Chr09	6807304	6807794	95	3.149E-01	1.909E-01	-1.240E-01	2.278E-03
Chr09	7203022	7203520	113	3.698E-01	2.664E-01	-1.034E-01	9.400E-06
Chr09	7504455	7504945	153	6.108E-02	1.933E-01	1.322E-01	2.966E-04
Chr09	7639428	7639916	177	4.500E-01	3.341E-01	-1.159E-01	8.989E-03
Chr09	8231238	8231734	118	3.308E-01	2.228E-01	-1.080E-01	1.830E-05
Chr09	8234733	8235232	91	3.243E-01	1.907E-01	-1.336E-01	1.170E-05
Chr09	8572755	8573207	51	2.614E-01	1.224E-01	-1.390E-01	1.317E-04
Chr09	9450450	9450677	22	1.578E-01	5.396E-02	-1.039E-01	1.601E-02
Chr09	9450824	9450967	31	1.074E-01	5.950E-03	-1.014E-01	1.296E-03
Chr09	9471218	9471561	94	3.881E-01	2.590E-01	-1.291E-01	8.244E-03
Chr09	9899796	9900195	86	7.701E-02	4.665E-01	3.895E-01	3.100E-06
Chr10	174068	174565	114	4.435E-01	3.199E-01	-1.236E-01	1.450E-03
Chr10	351826	352319	123	6.511E-01	4.868E-01	-1.644E-01	9.500E-05
Chr10	404428	404916	144	2.532E-01	1.353E-01	-1.179E-01	1.500E-06
Chr10	1238856	1239031	59	1.469E-01	3.188E-01	1.719E-01	4.876E-02
Chr10	1719044	1719455	100	2.296E-01	9.012E-02	-1.395E-01	6.452E-03
Chr10	2090376	2090872	165	2.420E-01	1.063E-01	-1.357E-01	0.000E+00
Chr10	2491707	2492205	89	7.879E-02	2.493E-01	1.706E-01	4.852E-02
Chr10	2520475	2520757	36	4.298E-02	2.388E-01	1.958E-01	1.520E-05
Chr10	2985356	2985843	154	2.801E-01	1.675E-01	-1.126E-01	1.000E-07
Chr10	3001935	3002422	159	4.150E-01	3.102E-01	-1.048E-01	4.954E-04
Chr10	3011343	3011836	147	2.518E-01	1.400E-01	-1.118E-01	1.274E-02
Chr10	3039267	3039634	98	4.072E-01	3.003E-01	-1.069E-01	1.044E-03
Chr10	3957717	3958215	165	5.922E-01	4.347E-01	-1.575E-01	0.000E+00
Chr10	3977594	3978010	53	3.386E-01	1.968E-01	-1.419E-01	2.526E-03
Chr10	4029630	4030102	90	3.145E-01	1.945E-01	-1.200E-01	1.248E-03
Chr10	4247523	4247989	117	4.072E-01	3.055E-01	-1.017E-01	1.644E-03
Chr10	4362713	4363201	188	4.461E-01	3.442E-01	-1.018E-01	0.000E+00
Chr10	4407044	4407467	55	4.502E-01	6.189E-01	1.686E-01	4.138E-03
Chr10	5208012	5208504	142	5.558E-01	3.951E-01	-1.607E-01	4.880E-05
Chr10	5372906	5373345	27	2.612E-01	1.352E-01	-1.260E-01	6.347E-03
Chr10	7631655	7632139	106	3.802E-01	2.385E-01	-1.417E-01	0.000E+00

Chr10	7712852	7713347	157	3.060E-01	1.990E-01	-1.070E-01	3.090E-05
Chr10	7733277	7733767	189	3.265E-01	2.178E-01	-1.087E-01	6.410E-05
Chr10	8006923	8007411	91	2.440E-01	1.333E-01	-1.107E-01	1.138E-04
Chr10	8161283	8161754	70	4.224E-01	3.004E-01	-1.220E-01	1.160E-05
Chr10	8637633	8638129	113	3.559E-01	2.350E-01	-1.209E-01	1.216E-04
Chr10	8796343	8796834	173	4.287E-01	3.064E-01	-1.223E-01	2.547E-04
Chr10	9287859	9288348	107	4.171E-01	3.001E-01	-1.170E-01	2.640E-05
Chr10	10475916	10476412	133	2.622E-01	1.048E-01	-1.573E-01	1.196E-03
Chr10	10490845	10491332	113	3.964E-01	2.818E-01	-1.146E-01	2.095E-02
Chr10	10629695	10630191	158	2.589E-01	1.531E-01	-1.058E-01	4.009E-02
Chr10	11065978	11066169	46	2.721E-01	4.043E-01	1.322E-01	1.756E-02
Chr10	11096083	11096553	63	4.463E-01	3.324E-01	-1.139E-01	3.810E-02
Chr10	11566913	11567407	224	1.444E-01	5.345E-03	-1.391E-01	0.000E+00
Chr10	11567409	11567905	179	1.947E-01	9.975E-03	-1.847E-01	1.900E-04
Chr10	11567908	11568404	210	1.385E-01	4.434E-03	-1.341E-01	0.000E+00
Chr10	11576037	11576534	207	1.101E-01	6.097E-03	-1.040E-01	1.000E-07
Chr10	11922167	11922265	18	4.532E-01	2.746E-01	-1.785E-01	1.493E-02
Chr10	12260095	12260578	198	4.100E-01	2.922E-01	-1.178E-01	2.759E-02
Chr10	12746351	12746844	160	3.535E-01	2.122E-01	-1.413E-01	3.100E-04
Chr10	12958555	12959048	183	3.509E-01	2.310E-01	-1.200E-01	1.609E-04
Chr10	13122012	13122506	126	2.171E-01	3.330E-01	1.160E-01	3.107E-03
Chr10	13469932	13470282	52	2.592E-01	4.303E-01	1.711E-01	6.586E-03
Chr10	13753157	13753638	141	4.187E-01	3.079E-01	-1.109E-01	2.900E-06
Chr10	14712750	14713019	47	5.725E-01	4.477E-01	-1.248E-01	8.990E-05
Chr10	14895565	14895797	48	2.539E-01	5.352E-02	-2.003E-01	4.250E-05
Chr10	15053171	15053648	148	4.529E-01	3.431E-01	-1.097E-01	0.000E+00
Chr10	15365286	15365582	62	1.062E-01	5.510E-03	-1.007E-01	4.138E-04
Chr10	15381751	15382179	83	1.297E-01	2.397E-01	1.100E-01	1.333E-04
Chr11	440674	441170	88	6.049E-01	4.989E-01	-1.061E-01	1.060E-05
Chr11	689192	689689	184	2.265E-01	8.919E-02	-1.373E-01	1.382E-02
Chr11	770971	771453	87	2.402E-01	7.259E-02	-1.676E-01	3.570E-02
Chr11	781901	782396	154	2.660E-01	1.285E-01	-1.374E-01	9.687E-03
Chr11	782398	782895	79	2.628E-01	4.065E-03	-2.588E-01	3.132E-02
Chr11	783021	783502	197	2.639E-01	9.807E-02	-1.659E-01	1.215E-02
Chr11	832252	832701	77	5.164E-01	2.898E-01	-2.267E-01	0.000E+00
Chr11	845108	845274	24	1.887E-01	4.447E-01	2.560E-01	9.076E-04
Chr11	1166494	1166989	96	4.223E-01	5.764E-01	1.542E-01	2.038E-04
Chr11	1397106	1397593	122	2.283E-01	3.386E-01	1.104E-01	2.114E-04
Chr11	1401431	1401910	79	4.334E-01	3.195E-01	-1.139E-01	1.780E-05
Chr11	1604627	1605123	197	3.654E-01	2.253E-01	-1.400E-01	1.516E-04
Chr11	1612117	1612596	127	2.948E-01	1.648E-01	-1.301E-01	4.603E-04
Chr11	1694961	1695441	151	3.176E-01	1.506E-01	-1.671E-01	1.323E-02
Chr11	1912547	1913004	81	3.707E-01	2.489E-01	-1.217E-01	2.245E-04
Chr11	1915768	1916019	73	3.112E-01	1.423E-01	-1.689E-01	2.000E-07
Chr11	1916023	1916272	33	2.450E-01	9.333E-02	-1.517E-01	2.670E-05
Chr11	1948153	1948256	22	1.163E-01	1.097E-02	-1.053E-01	3.210E-03
Chr11	2084404	2084897	171	2.222E-01	7.734E-02	-1.449E-01	4.493E-02

Chr11	2212832	2213325	178	2.503E-01	1.337E-01	-1.166E-01	7.732E-03
Chr11	2234039	2234494	79	4.591E-01	3.567E-01	-1.025E-01	2.330E-03
Chr11	2369792	2370285	169	3.525E-01	2.444E-01	-1.081E-01	7.234E-03
Chr11	3171423	3171909	98	2.638E-01	1.347E-01	-1.291E-01	5.939E-04
Chr11	3394119	3394613	74	2.777E-01	1.656E-01	-1.120E-01	7.915E-04
Chr11	3477525	3478019	214	2.574E-01	1.475E-01	-1.099E-01	4.399E-02
Chr11	3539432	3539931	42	5.380E-01	4.314E-01	-1.067E-01	8.909E-03
Chr11	3863091	3863550	126	2.467E-01	1.411E-01	-1.056E-01	2.794E-03
Chr11	3970586	3971077	73	3.541E-01	2.259E-01	-1.282E-01	8.454E-04
Chr11	4387216	4387701	171	3.261E-01	1.862E-01	-1.400E-01	8.500E-06
Chr11	4428115	4428395	37	4.562E-01	3.416E-01	-1.146E-01	1.730E-04
Chr11	5271848	5272345	172	3.601E-01	2.591E-01	-1.009E-01	0.000E+00
Chr11	6337243	6337635	106	3.133E-01	2.122E-01	-1.011E-01	2.757E-02
Chr11	6353484	6353963	216	3.293E-01	2.071E-01	-1.222E-01	1.378E-02
Chr11	6866983	6867480	170	3.884E-01	2.410E-01	-1.474E-01	4.310E-02
Chr11	7130857	7131345	212	1.603E-01	5.567E-02	-1.046E-01	7.855E-03
Chr11	7211609	7212097	120	4.674E-01	3.286E-01	-1.388E-01	6.000E-07
Chr11	7350316	7350809	170	1.930E-01	8.873E-02	-1.042E-01	9.416E-04
Chr11	7539068	7539561	228	2.019E-01	9.372E-02	-1.081E-01	4.093E-02
Chr11	7820801	7821278	174	3.715E-01	2.599E-01	-1.115E-01	2.760E-05
Chr11	7826267	7826763	186	1.895E-01	8.478E-02	-1.047E-01	4.410E-02
Chr11	7930175	7930667	118	2.251E-01	1.064E-01	-1.186E-01	7.560E-03
Chr11	8076910	8077389	63	3.868E-01	2.505E-01	-1.363E-01	3.230E-05
Chr11	8152356	8152846	111	4.552E-01	3.448E-01	-1.103E-01	1.098E-03
Chr11	9235171	9235564	118	2.319E-01	3.820E-02	-1.937E-01	0.000E+00
Chr11	9253108	9253589	85	1.618E-01	4.924E-02	-1.125E-01	2.464E-02
Chr11	9963898	9964387	184	2.402E-01	1.306E-01	-1.096E-01	1.500E-06
Chr11	10187815	10187902	11	4.929E-01	2.804E-01	-2.125E-01	2.598E-02
Chr11	10212038	10212518	128	4.633E-01	3.536E-01	-1.097E-01	9.978E-04
Chr11	10308266	10308664	69	3.958E-01	2.878E-01	-1.080E-01	5.000E-07
Chr11	10440832	10441315	180	1.091E-01	6.191E-03	-1.029E-01	1.809E-02
Chr11	10538497	10538992	185	4.119E-01	2.485E-01	-1.635E-01	0.000E+00
Chr11	10812973	10813460	150	2.960E-01	1.876E-01	-1.084E-01	0.000E+00
Chr11	11222010	11222505	137	4.525E-01	3.515E-01	-1.010E-01	4.721E-03
Chr11	11263386	11263876	134	2.703E-01	1.685E-01	-1.018E-01	2.429E-02
Chr11	11468359	11468857	138	3.187E-01	2.132E-01	-1.055E-01	2.100E-06
Chr11	11471357	11471834	143	3.557E-01	2.408E-01	-1.149E-01	0.000E+00
Chr11	11686234	11686728	158	3.477E-01	2.470E-01	-1.008E-01	1.862E-03
Chr11	11795113	11795602	92	3.190E-01	2.083E-01	-1.106E-01	3.472E-03
Chr11	12125579	12126070	146	2.965E-01	1.644E-01	-1.321E-01	4.400E-06
Chr11	12205879	12206371	165	2.554E-01	1.412E-01	-1.142E-01	2.434E-03
Chr11	12214839	12215332	124	2.475E-01	1.057E-01	-1.418E-01	1.465E-02
Chr11	12696201	12696694	145	4.333E-01	3.113E-01	-1.220E-01	0.000E+00
Chr11	12986063	12986560	131	3.436E-01	2.118E-01	-1.318E-01	2.660E-05
Chr11	13054628	13055103	110	1.392E-01	3.078E-02	-1.084E-01	9.250E-04
Chr11	13149516	13150007	172	3.030E-01	1.439E-01	-1.590E-01	1.002E-03
Chr11	13153981	13154476	182	3.760E-01	1.360E-01	-2.400E-01	1.000E-06

Chr11	13218325	13218738	73	1.612E-01	4.267E-01	2.654E-01	1.303E-03
Chr11	13264631	13264694	14	2.308E-01	1.198E-01	-1.110E-01	2.935E-02
Chr11	13268973	13269434	126	3.618E-01	2.564E-01	-1.054E-01	2.000E-06
Chr11	13548931	13549413	206	3.211E-01	2.180E-01	-1.031E-01	7.087E-04
Chr12	17987	18466	80	3.603E-01	4.657E-01	1.054E-01	3.585E-02
Chr12	185033	185530	151	2.492E-01	1.254E-01	-1.238E-01	3.596E-02
Chr12	326259	326752	87	3.347E-01	2.247E-01	-1.099E-01	7.963E-03
Chr12	405804	406251	122	3.585E-01	2.409E-01	-1.176E-01	0.000E+00
Chr12	467695	468184	185	3.856E-03	1.417E-01	1.378E-01	3.884E-03
Chr12	487942	488434	171	7.516E-02	1.998E-01	1.246E-01	1.082E-02
Chr12	503356	503623	78	3.119E-01	2.075E-01	-1.044E-01	3.321E-03
Chr12	530453	530934	155	1.214E-01	1.743E-02	-1.040E-01	2.540E-05
Chr12	737566	738064	202	3.991E-01	2.636E-01	-1.355E-01	4.183E-03
Chr12	879908	880402	162	3.639E-01	2.582E-01	-1.056E-01	9.297E-03
Chr12	1308513	1309008	167	5.885E-01	4.629E-01	-1.256E-01	3.238E-03
Chr12	1636641	1637138	164	3.831E-01	1.914E-01	-1.917E-01	1.346E-02
Chr12	2003113	2003236	38	3.636E-01	2.354E-01	-1.282E-01	3.902E-02
Chr12	2817518	2818014	168	2.153E-01	1.015E-01	-1.138E-01	1.488E-02
Chr12	2976871	2977285	80	3.805E-01	2.605E-01	-1.199E-01	2.534E-03
Chr12	3421683	3421788	28	2.637E-01	1.324E-01	-1.314E-01	1.615E-03
Chr12	3478617	3479017	148	4.289E-01	2.147E-01	-2.142E-01	1.315E-03
Chr12	3616674	3617170	210	2.790E-01	1.715E-01	-1.076E-01	2.378E-03
Chr12	3925822	3926281	172	2.934E-01	1.756E-01	-1.178E-01	1.164E-04
Chr12	3976116	3976607	147	3.444E-01	2.208E-01	-1.236E-01	0.000E+00
Chr12	4238473	4238653	30	7.335E-02	2.203E-01	1.470E-01	6.651E-04
Chr12	4395398	4395896	150	4.056E-01	3.020E-01	-1.036E-01	1.378E-04
Chr12	4438803	4439288	152	3.313E-01	2.310E-01	-1.004E-01	2.000E-07
Chr12	4824194	4824623	37	1.542E-01	2.699E-01	1.157E-01	3.010E-05
Chr12	5387365	5387857	189	2.582E-01	1.227E-01	-1.356E-01	4.188E-02
Chr12	5747217	5747629	86	7.505E-01	5.698E-01	-1.807E-01	4.054E-04
Chr12	6080715	6081142	151	7.034E-01	5.570E-01	-1.464E-01	5.042E-03
Chr12	6085463	6085950	133	4.997E-01	6.797E-01	1.800E-01	4.905E-02
Chr12	6097211	6097690	188	5.835E-01	6.902E-01	1.067E-01	5.625E-03
Chr12	6108797	6109233	110	6.302E-01	7.521E-01	1.219E-01	6.888E-04
Chr12	6109696	6110057	95	5.535E-01	6.899E-01	1.364E-01	2.956E-03
Chr12	6112425	6112864	154	6.600E-01	5.304E-01	-1.296E-01	2.355E-02
Chr12	6122762	6123207	88	6.783E-01	8.091E-01	1.308E-01	2.220E-05
Chr12	6125074	6125385	93	5.546E-01	7.010E-01	1.464E-01	3.026E-02
Chr12	6134534	6134873	34	5.269E-01	7.602E-01	2.332E-01	1.221E-03
Chr12	6173877	6174212	101	6.680E-01	7.991E-01	1.312E-01	1.494E-02
Chr12	6960341	6960835	169	3.412E-01	2.338E-01	-1.074E-01	9.000E-07
Chr12	6963813	6964307	208	4.395E-01	2.921E-01	-1.473E-01	1.349E-02
Chr12	7367417	7367904	243	2.339E-01	1.132E-01	-1.206E-01	4.313E-02
Chr12	7500488	7500984	177	4.153E-01	2.752E-01	-1.401E-01	6.137E-03
Chr12	7539905	7540383	179	2.443E-01	1.425E-01	-1.018E-01	2.022E-04
Chr12	7672617	7673101	168	2.784E-01	4.091E-01	1.307E-01	7.818E-03
Chr12	7746011	7746505	200	1.739E-01	3.063E-01	1.323E-01	6.060E-05

Chr12	8060861	8061354	171	2.265E-01	1.131E-01	-1.134E-01	5.774E-04
Chr12	8287259	8287748	175	2.504E-01	5.648E-02	-1.939E-01	7.275E-03
Chr12	8817958	8818451	117	2.586E-01	9.370E-02	-1.649E-01	1.542E-02
Chr12	9071728	9072226	162	2.528E-01	7.281E-02	-1.800E-01	4.196E-03
Chr12	9100721	9101220	141	3.367E-01	1.919E-01	-1.448E-01	1.893E-03
Chr12	9103266	9103719	142	1.850E-01	7.444E-02	-1.105E-01	7.885E-04
Chr12	9192112	9192184	10	4.350E-01	2.382E-01	-1.969E-01	3.393E-02
Chr12	9217228	9217669	71	3.293E-01	2.153E-01	-1.140E-01	1.763E-03
Chr12	9218729	9219045	45	4.459E-01	2.999E-01	-1.461E-01	3.219E-03
Chr12	9270053	9270530	165	2.298E-01	1.198E-01	-1.100E-01	1.404E-03
Chr12	9384274	9384738	167	2.031E-01	1.015E-01	-1.017E-01	3.447E-02
Chr12	9620960	9621456	178	1.959E-01	8.880E-02	-1.072E-01	3.341E-02
Chr12	9682885	9683374	186	2.957E-01	1.459E-01	-1.498E-01	2.494E-02
Chr12	9705465	9705943	127	2.173E-01	1.082E-01	-1.091E-01	2.100E-03
Chr12	9800124	9800617	172	1.889E-01	3.493E-01	1.605E-01	3.984E-02
Chr12	9868284	9868755	88	3.186E-01	1.998E-01	-1.187E-01	1.126E-04
Chr12	10122473	10122961	93	3.297E-01	2.282E-01	-1.015E-01	1.800E-06
Chr12	10339017	10339113	23	3.672E-01	2.040E-01	-1.632E-01	1.586E-03
Chr12	10416247	10416549	49	4.796E-01	1.665E-01	-3.131E-01	0.000E+00
Chr12	10539817	10540298	95	6.045E-01	3.811E-01	-2.234E-01	0.000E+00
Chr13	49863	50359	241	2.638E-01	1.619E-01	-1.019E-01	2.066E-02
Chr13	103252	103722	142	2.703E-01	1.628E-01	-1.075E-01	1.000E-07
Chr13	571800	572294	164	4.654E-01	3.270E-01	-1.384E-01	4.332E-03
Chr13	876133	876612	136	3.181E-01	1.644E-01	-1.537E-01	2.114E-02
Chr13	950663	950813	21	2.024E-01	5.741E-02	-1.450E-01	2.539E-03
Chr13	967965	968324	49	4.255E-01	3.138E-01	-1.117E-01	1.366E-03
Chr13	981777	982188	124	1.723E-01	6.643E-02	-1.059E-01	7.180E-05
Chr13	1029688	1029974	46	2.009E-01	6.635E-02	-1.346E-01	1.977E-03
Chr13	1098135	1098467	70	2.121E-01	1.065E-01	-1.056E-01	1.473E-03
Chr13	1847093	1847589	152	4.797E-01	3.708E-01	-1.089E-01	7.270E-03
Chr13	2083719	2084215	164	4.382E-01	3.347E-01	-1.035E-01	1.693E-02
Chr13	2626507	2627001	148	2.331E-01	1.292E-01	-1.039E-01	7.627E-03
Chr13	2658529	2658908	71	2.131E-01	8.624E-02	-1.269E-01	1.837E-02
Chr13	2734628	2735122	122	3.545E-01	2.353E-01	-1.192E-01	6.000E-07
Chr13	3161973	3162058	26	1.973E-01	3.658E-01	1.685E-01	3.355E-03
Chr13	3348631	3348847	20	2.427E-01	1.336E-01	-1.091E-01	3.154E-03
Chr13	3534868	3535351	190	3.143E-01	2.130E-01	-1.013E-01	4.900E-06
Chr13	3996177	3996662	113	3.222E-01	2.065E-01	-1.157E-01	3.361E-03
Chr13	4932444	4932934	120	5.026E-01	3.923E-01	-1.103E-01	8.050E-05
Chr13	5278542	5279036	173	2.258E-01	1.232E-01	-1.026E-01	1.237E-03
Chr13	5807421	5807529	26	4.626E-01	2.873E-01	-1.753E-01	4.311E-04
Chr13	5924924	5925421	96	2.401E-01	3.954E-01	1.553E-01	1.504E-04
Chr13	6619343	6619827	199	2.672E-01	1.504E-01	-1.168E-01	1.127E-02
Chr13	6798274	6798377	19	1.683E-01	3.541E-02	-1.329E-01	3.583E-02
Chr13	6953141	6953624	58	2.586E-01	1.028E-01	-1.558E-01	7.370E-03
Chr13	7032374	7032824	117	4.287E-01	3.233E-01	-1.054E-01	1.417E-02
Chr13	7060168	7060319	26	1.466E-01	3.657E-02	-1.100E-01	1.010E-05

Chr13	7121640	7122021	85	1.159E-01	2.186E-01	1.027E-01	0.000E+00
Chr13	7538924	7539398	141	5.922E-01	4.757E-01	-1.165E-01	4.253E-02
Chr13	7550026	7550409	106	5.689E-01	4.644E-01	-1.045E-01	1.862E-02
Chr13	7559009	7559461	157	4.099E-01	5.123E-01	1.024E-01	2.400E-02
Chr13	7561426	7561916	173	4.613E-01	6.117E-01	1.504E-01	3.100E-05
Chr13	7567027	7567108	34	5.245E-01	3.656E-01	-1.590E-01	4.072E-02
Chr13	7579883	7580366	159	4.860E-01	6.879E-01	2.019E-01	2.080E-03
Chr13	7584536	7584997	213	5.433E-01	4.038E-01	-1.395E-01	5.417E-03
Chr13	7587117	7587576	163	3.756E-01	5.146E-01	1.389E-01	8.606E-03
Chr13	7599037	7599531	108	3.509E-01	2.322E-01	-1.187E-01	2.822E-04
Chr13	8281641	8282135	112	5.636E-01	4.612E-01	-1.024E-01	3.532E-02
Chr13	9243428	9243494	26	3.795E-01	2.428E-01	-1.367E-01	3.750E-02
Chr13	9244649	9245141	149	5.915E-01	4.776E-01	-1.139E-01	1.670E-05
Chr13	9452160	9452320	28	3.635E-01	2.004E-01	-1.632E-01	2.834E-04
Chr13	10158014	10158493	106	2.140E-01	1.009E-01	-1.131E-01	1.593E-03
Chr13	10578889	10579094	48	2.650E-01	3.764E-01	1.114E-01	3.968E-03
Chr13	11455599	11456065	165	3.960E-01	2.930E-01	-1.030E-01	1.079E-02
Chr13	11930945	11931396	118	2.315E-01	1.290E-01	-1.024E-01	2.000E-07
Chr13	12186505	12186994	188	3.913E-01	2.910E-01	-1.002E-01	1.920E-05
Chr13	12332032	12332516	131	4.669E-01	3.233E-01	-1.435E-01	1.795E-04
Chr13	12442322	12442814	189	2.624E-01	1.588E-01	-1.036E-01	3.755E-02
Chr13	12534082	12534562	187	1.445E-01	6.658E-03	-1.378E-01	1.592E-03
Chr13	12776276	12776768	197	2.602E-01	1.318E-01	-1.284E-01	1.009E-02
Chr13	13169032	13169517	86	4.736E-01	3.501E-01	-1.235E-01	1.268E-04
Chr13	13275778	13276185	82	2.395E-01	3.844E-01	1.449E-01	1.664E-02
Chr13	13368941	13369434	151	2.369E-01	1.176E-01	-1.193E-01	2.149E-02
Chr13	13523424	13523910	156	4.350E-01	2.861E-01	-1.489E-01	1.617E-02
Chr13	13855378	13855870	117	2.818E-01	1.515E-01	-1.303E-01	1.555E-03
Chr13	13878281	13878771	169	4.123E-01	3.061E-01	-1.062E-01	2.328E-02
Chr13	14559035	14559527	146	3.630E-01	2.298E-01	-1.332E-01	2.830E-05
Chr13	14603294	14603785	188	3.276E-01	2.027E-01	-1.249E-01	4.700E-06
Chr14	450834	451329	160	4.757E-01	3.742E-01	-1.016E-01	4.940E-05
Chr14	497390	497765	102	2.951E-01	1.756E-01	-1.196E-01	8.378E-03
Chr14	513060	513545	185	2.676E-01	1.429E-01	-1.247E-01	9.238E-03
Chr14	851673	851832	26	1.851E-01	6.333E-02	-1.217E-01	4.686E-04
Chr14	1000734	1001224	110	3.159E-01	4.476E-01	1.318E-01	2.702E-03
Chr14	1282755	1283241	185	4.050E-01	2.406E-01	-1.643E-01	7.581E-04
Chr14	1463352	1463719	120	3.881E-01	2.834E-01	-1.047E-01	3.625E-04
Chr14	1464279	1464624	57	1.205E-01	4.364E-04	-1.201E-01	4.000E-07
Chr14	1721057	1721539	88	3.244E-01	1.738E-01	-1.506E-01	9.187E-04
Chr14	1858292	1858789	116	5.340E-01	3.504E-01	-1.836E-01	2.060E-05
Chr14	1903563	1904058	90	3.082E-01	2.012E-01	-1.070E-01	5.463E-03
Chr14	1920961	1921441	74	1.434E-01	1.621E-04	-1.433E-01	5.108E-03
Chr14	1922419	1922890	105	2.497E-01	8.549E-02	-1.642E-01	5.300E-06
Chr14	2432434	2432911	141	3.038E-01	2.038E-01	-1.001E-01	1.904E-04
Chr14	2744526	2745013	161	4.026E-01	2.928E-01	-1.098E-01	1.120E-05
Chr14	3102628	3103114	149	2.535E-01	1.447E-01	-1.088E-01	4.708E-02

Chr14	3864110	3864593	186	3.506E-01	2.119E-01	-1.387E-01	2.700E-06
Chr14	3995780	3996270	173	2.139E-01	1.095E-01	-1.044E-01	3.190E-05
Chr14	4230527	4231024	172	4.685E-01	2.417E-01	-2.269E-01	2.800E-06
Chr14	5618935	5619056	21	3.077E-01	1.677E-01	-1.400E-01	1.034E-02
Chr14	5890579	5891078	150	4.244E-01	3.178E-01	-1.066E-01	6.502E-04
Chr14	5954816	5955312	162	2.882E-01	1.748E-01	-1.134E-01	2.900E-06
Chr14	7531593	7532088	138	3.187E-01	1.989E-01	-1.198E-01	1.678E-03
Chr14	7842065	7842527	136	2.855E-01	1.840E-01	-1.015E-01	3.041E-04
Chr14	8098519	8099016	187	4.808E-01	3.523E-01	-1.285E-01	8.969E-04
Chr14	8149974	8150472	147	3.435E-01	2.380E-01	-1.055E-01	0.000E+00
Chr14	8824377	8824866	99	3.555E-01	1.295E-01	-2.260E-01	6.238E-04
Chr14	9026075	9026567	238	2.653E-01	1.392E-01	-1.261E-01	3.000E-07
Chr14	10473676	10474107	100	3.364E-01	2.177E-01	-1.187E-01	0.000E+00
Chr14	10521281	10521737	59	4.386E-01	3.382E-01	-1.004E-01	3.110E-05
Chr14	10524333	10524776	76	2.084E-01	9.938E-02	-1.091E-01	7.344E-04
Chr15W	2396483	2396968	149	3.408E-01	2.107E-01	-1.301E-01	1.544E-04
Chr15W	2660329	2660819	83	1.463E-01	2.737E-01	1.274E-01	1.100E-06
Chr15W	2758897	2759390	173	2.639E-01	1.397E-01	-1.241E-01	3.317E-02
Chr15W	2769843	2770340	185	5.163E-02	1.576E-01	1.059E-01	1.800E-06
Chr15W	2792741	2793235	132	4.257E-01	3.128E-01	-1.129E-01	1.966E-04
Chr15W	2800209	2800701	132	6.173E-01	3.985E-01	-2.188E-01	2.472E-03
Chr15W	2848244	2848718	171	3.552E-01	2.056E-01	-1.496E-01	3.020E-05
Chr15W	2860662	2861122	110	2.020E-01	7.168E-02	-1.303E-01	3.400E-06
Chr15W	2908969	2909463	167	4.114E-02	1.696E-01	1.285E-01	1.000E-07
Chr15W	2916455	2916949	163	9.807E-02	2.108E-01	1.127E-01	2.700E-02
Chr15W	2918456	2918946	150	2.586E-01	3.973E-01	1.387E-01	4.089E-04
Chr15W	2921948	2922410	112	3.304E-02	1.556E-01	1.225E-01	1.200E-06
Chr15W	2958589	2959086	150	4.498E-02	1.779E-01	1.329E-01	1.226E-03
Chr15W	2959093	2959584	182	1.810E-02	1.249E-01	1.068E-01	1.538E-03
Chr15W	2984027	2984515	150	1.114E-01	2.538E-01	1.425E-01	1.406E-02
Chr15W	2988507	2989003	167	3.282E-02	1.495E-01	1.166E-01	0.000E+00
Chr15W	2992995	2993493	161	5.241E-02	3.203E-01	2.679E-01	0.000E+00
Chr15W	3063283	3063757	132	2.824E-01	4.109E-01	1.285E-01	3.958E-02
Chr15W	3074696	3075181	113	1.880E-01	2.901E-01	1.021E-01	2.270E-05
Chr15W	3105311	3105793	149	4.361E-01	1.553E-01	-2.808E-01	2.000E-07
Chr15W	3106313	3106807	168	1.808E-01	5.153E-03	-1.756E-01	3.156E-02
Chr15W	3109305	3109804	149	6.192E-02	1.864E-01	1.245E-01	1.260E-02
Chr15W	3118796	3119290	115	3.247E-01	2.046E-01	-1.201E-01	5.470E-04
Chr15W	3119291	3119789	150	2.927E-01	1.815E-01	-1.113E-01	2.461E-02
Chr15W	3154019	3154496	120	3.646E-01	2.514E-01	-1.132E-01	1.700E-06
Chr15W	3166724	3167208	128	2.936E-01	1.685E-01	-1.252E-01	8.790E-05
Chr15W	3180229	3180674	143	5.266E-01	4.255E-01	-1.011E-01	2.102E-02
Chr15W	3181132	3181581	64	3.982E-01	2.706E-01	-1.276E-01	1.871E-03
Chr15W	3229846	3230312	105	2.696E-01	1.598E-01	-1.098E-01	3.214E-04
Chr15W	3274169	3274639	157	5.799E-02	1.698E-01	1.118E-01	4.843E-02
Chr15W	3321196	3321677	156	4.362E-01	3.306E-01	-1.057E-01	7.003E-03
Chr15W	3377304	3377795	129	3.578E-01	2.359E-01	-1.219E-01	6.508E-04

Chr15W	3405140	3405626	86	3.733E-01	2.592E-01	-1.141E-01	5.000E-07
Chr15W	3417635	3418085	112	5.069E-01	1.899E-01	-3.170E-01	1.600E-06
Chr15W	3463507	3463942	157	3.492E-01	2.252E-01	-1.240E-01	8.234E-03
Chr15W	3474974	3475442	161	3.834E-01	2.524E-01	-1.310E-01	5.000E-07
Chr15W	3479239	3479706	190	5.223E-01	4.062E-01	-1.160E-01	0.000E+00
Chr15W	3522873	3523284	152	3.537E-01	2.333E-01	-1.204E-01	7.887E-03
Chr15W	3535011	3535447	70	3.110E-01	1.549E-01	-1.561E-01	4.618E-02
Chr15W	3549225	3549707	173	4.355E-01	1.555E-01	-2.801E-01	8.876E-03
Chr15W	3555697	3556194	165	3.781E-01	2.399E-01	-1.382E-01	7.997E-04
Chr15W	3573351	3573776	143	2.732E-01	1.532E-01	-1.200E-01	4.114E-02
Chr15W	3595631	3596111	99	4.126E-01	2.242E-01	-1.884E-01	0.000E+00
Chr15W	3603440	3603796	98	2.163E-01	4.006E-01	1.843E-01	1.798E-02
Chr15W	3658477	3658970	125	2.304E-02	1.503E-01	1.273E-01	9.000E-07
Chr15W	3660965	3661459	178	1.564E-01	3.965E-02	-1.168E-01	1.510E-02
Chr15W	3661463	3661954	198	2.181E-01	9.341E-02	-1.246E-01	9.670E-04
Chr15W	3667416	3667874	149	1.359E-01	1.288E-02	-1.230E-01	3.788E-02
Chr15W	3678478	3678952	114	1.189E-01	2.411E-01	1.222E-01	1.492E-02
Chr15W	3712268	3712761	167	6.443E-02	1.954E-01	1.310E-01	3.417E-03
Chr15W	3746099	3746581	106	2.835E-01	4.609E-01	1.774E-01	1.320E-05
Chr15W	3750496	3750982	141	2.607E-01	1.592E-01	-1.015E-01	2.615E-02
Chr15W	3771126	3771607	186	3.470E-01	2.289E-01	-1.180E-01	3.874E-02
Chr15W	3779386	3779862	200	4.394E-01	3.074E-01	-1.319E-01	0.000E+00
Chr15W	3827231	3827604	71	1.163E-01	2.261E-01	1.097E-01	2.528E-03
Chr15W	3833497	3833971	110	5.997E-01	4.087E-01	-1.911E-01	2.279E-03
Chr15W	3834452	3834921	227	5.392E-01	3.534E-01	-1.858E-01	2.027E-03
Chr15W	3917015	3917455	112	2.983E-02	2.625E-01	2.326E-01	0.000E+00
Chr15W	3917912	3918357	90	3.632E-01	1.587E-01	-2.045E-01	1.027E-03
Chr15W	3932040	3932382	42	2.537E-01	1.401E-01	-1.137E-01	2.042E-02
Chr15W	3997621	3998102	154	2.585E-01	1.013E-01	-1.571E-01	3.900E-06
Chr15W	4015439	4015932	163	2.281E-01	1.228E-01	-1.053E-01	6.278E-03
Chr15W	4021417	4021909	164	2.395E-01	1.077E-01	-1.318E-01	2.749E-03
Chr15W	4103673	4103997	55	4.465E-01	2.942E-01	-1.523E-01	2.530E-05
Chr15W	4108016	4108463	55	3.644E-01	2.583E-01	-1.061E-01	3.288E-04
Chr15W	4121505	4121988	191	4.265E-01	1.339E-01	-2.926E-01	0.000E+00
Chr15W	4131325	4131809	109	1.476E-01	3.070E-02	-1.169E-01	4.476E-02
Chr15W	4157369	4157866	129	1.025E-01	2.173E-01	1.148E-01	7.152E-04
Chr15W	4159373	4159861	139	2.362E-01	1.183E-01	-1.180E-01	1.054E-02
Chr15W	4162358	4162849	153	2.565E-01	1.298E-01	-1.267E-01	1.961E-02
Chr15W	4201081	4201578	148	1.868E-01	6.871E-02	-1.181E-01	1.516E-04
Chr15W	4235514	4236007	123	4.817E-01	3.078E-01	-1.739E-01	3.617E-04
Chr15W	4286437	4286921	140	3.153E-01	1.902E-01	-1.251E-01	1.329E-03
Chr15W	4296341	4296836	165	2.528E-01	1.381E-01	-1.147E-01	0.000E+00
Chr15W	4313371	4313858	161	2.738E-01	1.586E-01	-1.152E-01	8.974E-03
Chr15W	4363533	4364024	159	1.425E-01	2.849E-02	-1.140E-01	3.275E-02
Chr15W	4383717	4384195	153	1.716E-01	5.564E-02	-1.160E-01	2.025E-02
Chr15W	4384200	4384682	154	3.549E-01	7.932E-02	-2.755E-01	3.000E-07
Chr15W	4385651	4386132	84	6.013E-02	1.999E-01	1.398E-01	1.300E-06

Chr15W	4423432	4423919	151	3.273E-01	4.277E-01	1.003E-01	3.214E-04
Chr15W	4437961	4438440	82	2.005E-01	3.028E-01	1.024E-01	8.710E-05
Chr15W	4453948	4454436	87	4.293E-01	2.643E-01	-1.650E-01	3.449E-03
Chr15W	4464049	4464526	141	4.402E-01	2.914E-01	-1.488E-01	1.462E-02
Chr15W	4469531	4470003	132	2.752E-01	3.906E-01	1.153E-01	1.195E-02
Chr15W	4593598	4594086	143	2.958E-01	4.696E-01	1.738E-01	1.722E-02
Chr15W	4643536	4644012	142	8.724E-02	2.029E-01	1.157E-01	2.870E-03
Chr15W	4646013	4646497	101	1.227E-01	1.279E-02	-1.099E-01	3.891E-02
Chr15W	4947234	4947724	139	2.361E-01	1.028E-01	-1.333E-01	1.378E-04
Chr15W	4978217	4978542	52	3.847E-01	2.085E-01	-1.762E-01	1.106E-04
Chr15W	5087162	5087657	189	8.078E-02	2.320E-01	1.512E-01	4.273E-03
Chr15W	5105101	5105585	117	1.818E-01	6.411E-02	-1.177E-01	4.844E-02
Chr15W	5164611	5164991	131	1.692E-01	4.209E-02	-1.271E-01	1.252E-02
Chr15W	5174250	5174660	74	2.589E-01	9.109E-02	-1.678E-01	0.000E+00
Chr15W	5444203	5444697	202	2.201E-01	4.155E-02	-1.786E-01	8.579E-03
Chr15W	5444701	5445197	203	1.635E-01	5.932E-02	-1.042E-01	2.589E-02
Chr15W	5448694	5449193	145	1.289E-01	1.958E-02	-1.093E-01	3.167E-02
Chr15W	5496893	5497377	180	7.092E-02	1.729E-01	1.020E-01	9.731E-03
Chr15W	5515948	5516422	74	1.206E-01	2.275E-01	1.069E-01	3.964E-02
Chr15W	5552042	5552532	110	2.922E-01	1.300E-01	-1.622E-01	3.761E-02
Chr15W	5657629	5657892	44	2.628E-01	3.836E-01	1.208E-01	6.439E-04
Chr15W	5658642	5658877	44	2.821E-01	1.269E-01	-1.552E-01	3.000E-07
Chr15W	5859885	5860360	108	1.949E-01	2.987E-01	1.038E-01	7.562E-04
Chr15W	5870242	5870733	182	3.074E-01	1.733E-01	-1.341E-01	4.661E-03
Chr15W	5950023	5950511	107	2.217E-01	1.156E-01	-1.061E-01	3.418E-03
Chr15W	5972494	5972989	187	4.854E-01	2.198E-01	-2.656E-01	1.250E-03
Chr15W	6003778	6004243	173	2.572E-01	4.454E-01	1.882E-01	4.886E-02
Chr15W	6017614	6018099	118	4.011E-01	2.231E-01	-1.780E-01	8.557E-04
Chr15W	6033272	6033759	137	3.086E-01	4.481E-01	1.394E-01	1.931E-04
Chr15W	6076352	6076846	71	2.505E-01	6.064E-01	3.559E-01	0.000E+00
Chr15W	6089848	6090344	167	3.273E-01	2.168E-01	-1.106E-01	1.910E-03
Chr15W	6096851	6097345	90	4.878E-01	3.130E-01	-1.748E-01	0.000E+00
Chr15W	6100569	6101066	94	1.882E-01	3.945E-01	2.064E-01	1.063E-04
Chr15W	6101085	6101564	165	2.881E-01	1.769E-01	-1.112E-01	5.568E-03
Chr15W	6105052	6105548	94	3.136E-01	2.013E-01	-1.123E-01	1.275E-04
Chr15W	6131444	6131934	102	5.207E-01	3.785E-01	-1.422E-01	1.836E-02
Chr15W	6146878	6147373	156	3.007E-01	1.606E-01	-1.401E-01	3.586E-03
Chr15W	6147880	6148368	204	2.781E-01	1.012E-01	-1.769E-01	4.770E-02
Chr15W	6217537	6218023	130	3.700E-01	2.245E-01	-1.456E-01	0.000E+00
Chr15W	6259873	6260368	153	3.356E-01	1.170E-01	-2.187E-01	6.290E-05
Chr15W	6328005	6328493	117	5.528E-01	3.992E-01	-1.535E-01	0.000E+00
Chr15W	6330476	6330954	144	1.576E-01	3.692E-01	2.116E-01	2.000E-07
Chr15W	6359387	6359861	169	2.177E-01	4.004E-01	1.827E-01	1.680E-04
Chr15W	6366212	6366694	87	5.920E-02	4.110E-01	3.518E-01	0.000E+00
Chr15W	6373529	6374014	105	3.715E-01	1.442E-01	-2.272E-01	1.173E-03
Chr15W	6380579	6381066	163	3.824E-01	2.350E-01	-1.474E-01	0.000E+00
Chr15W	6398026	6398520	87	2.690E-02	1.564E-01	1.295E-01	0.000E+00

Chr15W	6409003	6409491	166	1.158E-01	2.762E-01	1.604E-01	3.140E-05
Chr15W	6445452	6445911	176	5.419E-01	4.203E-01	-1.216E-01	2.727E-02
Chr15W	6445914	6446378	133	6.439E-01	5.102E-01	-1.338E-01	8.475E-03
Chr15W	6464616	6465087	83	2.967E-01	1.651E-01	-1.316E-01	2.474E-04
Chr15W	6465090	6465558	143	5.643E-01	3.287E-01	-2.356E-01	3.000E-07
Chr15W	6507372	6507869	135	1.204E-01	2.808E-01	1.604E-01	0.000E+00
Chr15W	6507874	6508370	139	5.967E-02	2.248E-01	1.651E-01	3.430E-03
Chr15W	6545850	6546337	155	4.103E-01	2.980E-01	-1.124E-01	3.810E-05
Chr15W	6563862	6564352	168	1.986E-01	4.594E-02	-1.526E-01	2.840E-05
Chr15W	6570835	6571329	167	3.554E-01	1.757E-01	-1.797E-01	1.452E-04
Chr15W	6611672	6612086	95	2.961E-01	1.638E-01	-1.323E-01	2.137E-02
Chr15W	6758465	6758948	62	5.523E-01	3.514E-01	-2.009E-01	9.703E-04
Chr15W	6793539	6794030	127	3.606E-01	1.140E-01	-2.466E-01	1.658E-04
Chr15W	6803398	6803886	139	5.973E-02	1.934E-01	1.336E-01	7.590E-03
Chr15W	6821709	6821896	34	4.142E-01	3.012E-01	-1.131E-01	3.558E-02
Chr15W	6821985	6822212	29	2.414E-01	7.347E-02	-1.679E-01	1.088E-03
Chr15W	6826333	6826832	172	3.084E-01	1.344E-01	-1.740E-01	2.200E-06
Chr15W	6846310	6846804	146	3.771E-01	2.314E-01	-1.457E-01	3.409E-02
Chr15W	6847781	6848068	103	4.132E-01	5.722E-01	1.590E-01	5.399E-03
Chr15W	6849865	6850173	101	3.370E-01	5.243E-01	1.873E-01	3.671E-03
Chr15W	6885880	6886376	173	2.807E-01	8.805E-02	-1.927E-01	0.000E+00
Chr15W	6886380	6886877	119	2.345E-01	4.603E-02	-1.885E-01	0.000E+00
Chr15W	6886880	6887378	174	3.000E-01	1.192E-01	-1.808E-01	0.000E+00
Chr15W	6887381	6887875	107	3.198E-01	1.361E-01	-1.837E-01	0.000E+00
Chr15W	6892875	6893371	198	1.573E-01	2.640E-01	1.067E-01	1.989E-03
Chr15W	6919348	6919844	172	4.019E-01	2.527E-01	-1.492E-01	3.486E-03
Chr15W	6925944	6926441	170	2.532E-01	1.438E-01	-1.094E-01	3.711E-02
Chr15W	6926943	6927439	186	1.090E-01	2.925E-01	1.835E-01	2.800E-06
Chr15W	6935427	6935923	175	3.700E-02	1.893E-01	1.523E-01	4.759E-04
Chr15W	6940917	6941411	109	3.886E-01	2.767E-01	-1.119E-01	2.294E-02
Chr15W	6941918	6942414	142	3.018E-01	4.380E-01	1.363E-01	6.128E-03
Chr15W	6945917	6946404	180	2.594E-01	3.938E-01	1.344E-01	5.256E-03
Chr15W	6965873	6966371	150	4.405E-01	1.754E-01	-2.651E-01	1.259E-02
Chr15W	6966877	6967369	151	5.042E-01	3.967E-01	-1.076E-01	2.232E-03
Chr15W	6967876	6968347	140	6.448E-01	3.636E-01	-2.811E-01	1.300E-06
Chr15W	6970351	6970838	166	2.549E-01	8.972E-02	-1.652E-01	1.080E-05
Chr15W	6985062	6985544	213	2.828E-01	1.522E-01	-1.306E-01	0.000E+00
Chr15W	7012405	7012901	130	4.624E-01	3.428E-01	-1.195E-01	0.000E+00
Chr15W	7028328	7028821	171	3.840E-01	1.495E-01	-2.345E-01	1.800E-05
Chr15W	7041374	7041851	170	3.011E-01	1.622E-01	-1.389E-01	1.590E-05
Chr15W	7051791	7052284	137	2.011E-01	5.730E-02	-1.438E-01	1.287E-03
Chr15W	7055267	7055758	149	3.009E-02	2.273E-01	1.972E-01	0.000E+00
Chr15W	7061221	7061709	185	4.875E-01	3.817E-01	-1.058E-01	4.000E-06
Chr15W	7090499	7090991	150	1.352E-01	3.586E-01	2.234E-01	2.290E-02
Chr15W	7101176	7101659	121	3.080E-01	1.961E-01	-1.119E-01	1.400E-06
Chr15W	7107053	7107489	55	6.748E-01	5.205E-01	-1.543E-01	6.966E-04
Chr15W	7143522	7144015	150	4.832E-01	3.781E-01	-1.051E-01	1.800E-06

Chr15W	7147984	7148477	132	4.888E-01	2.550E-01	-2.338E-01	2.590E-02
Chr15W	7156913	7157405	186	3.932E-02	1.444E-01	1.051E-01	6.780E-05
Chr15W	7207495	7207988	143	6.571E-02	1.895E-01	1.238E-01	0.000E+00
Chr15W	7213940	7214429	185	2.696E-01	1.375E-01	-1.321E-01	6.397E-03
Chr15W	7246343	7246838	175	3.091E-01	1.863E-01	-1.228E-01	1.889E-02
Chr15W	7283719	7284209	203	5.918E-01	3.636E-01	-2.282E-01	0.000E+00
Chr15W	7346427	7346919	175	1.506E-01	9.521E-03	-1.411E-01	4.206E-04
Chr15W	7376941	7377428	137	3.892E-01	1.928E-01	-1.964E-01	1.800E-06
Chr15W	7377926	7378418	209	4.611E-01	3.428E-01	-1.183E-01	2.655E-02
Chr15W	7441218	7441707	116	1.156E-01	3.097E-01	1.941E-01	7.000E-07
Chr15W	7456212	7456680	146	1.729E-01	2.873E-01	1.144E-01	1.328E-04
Chr15W	7456714	7457210	106	6.532E-01	5.170E-01	-1.362E-01	0.000E+00
Chr15W	7457214	7457710	138	1.516E-01	3.032E-01	1.516E-01	0.000E+00
Chr15W	7457720	7458210	170	2.623E-01	3.988E-01	1.366E-01	3.410E-04
Chr15W	7461211	7461705	119	5.711E-01	4.692E-01	-1.019E-01	2.286E-04
Chr15W	7506267	7506756	139	3.180E-01	2.089E-01	-1.090E-01	3.190E-05
Chr15W	7510765	7511233	144	4.339E-01	3.222E-01	-1.117E-01	1.000E-07
Chr15W	7529721	7530214	144	2.497E-01	3.529E-01	1.032E-01	3.462E-03
Chr15W	7534211	7534705	218	2.673E-01	3.711E-01	1.038E-01	9.229E-03
Chr15W	7540701	7541189	112	3.119E-01	1.636E-01	-1.483E-01	9.655E-03
Chr15W	7614790	7615284	180	3.190E-01	2.039E-01	-1.151E-01	2.272E-02
Chr15W	7696425	7696910	132	6.327E-02	3.370E-01	2.737E-01	0.000E+00
Chr15W	7699898	7700391	142	1.158E-01	2.812E-01	1.654E-01	5.400E-06
Chr15W	7733883	7734360	132	4.003E-04	2.673E-01	2.669E-01	0.000E+00
Chr15W	7734883	7735377	158	3.577E-01	2.523E-01	-1.054E-01	1.552E-02
Chr15W	7736882	7737381	195	9.392E-02	1.972E-01	1.032E-01	4.862E-02
Chr15W	7746409	7746876	182	5.104E-01	3.662E-01	-1.443E-01	0.000E+00
Chr15W	7763918	7764396	181	1.056E-01	2.081E-01	1.026E-01	1.520E-05
Chr15W	7764400	7764883	199	1.544E-01	5.258E-02	-1.018E-01	8.017E-03
Chr15W	7785704	7786180	145	9.097E-02	2.646E-01	1.736E-01	4.073E-03
Chr15W	7807890	7808292	131	3.548E-02	2.188E-01	1.833E-01	0.000E+00
Chr15W	7808712	7809110	78	3.038E-02	1.713E-01	1.410E-01	1.300E-06
Chr15W	7817736	7818215	107	2.106E-01	3.322E-01	1.217E-01	7.400E-06
Chr15W	7819720	7820216	169	3.139E-02	1.352E-01	1.038E-01	1.138E-02
Chr15W	7824692	7825186	52	4.265E-01	5.431E-01	1.166E-01	4.460E-02
Chr15W	7827525	7828009	147	6.195E-02	2.027E-01	1.407E-01	6.920E-05
Chr15W	7831481	7831968	167	2.397E-01	1.290E-01	-1.108E-01	6.669E-03
Chr15W	7834945	7835439	78	3.179E-01	2.106E-01	-1.072E-01	4.000E-07
Chr15W	7851786	7852275	181	2.340E-01	1.319E-01	-1.022E-01	5.746E-04
Chr15W	7869669	7870155	64	1.203E-01	6.962E-03	-1.133E-01	3.477E-02
Chr15W	7877321	7877785	199	3.236E-01	1.558E-01	-1.678E-01	2.648E-02
Chr15W	7881898	7882368	125	3.835E-01	1.973E-01	-1.863E-01	2.146E-03
Chr15W	7885373	7885866	189	2.592E-02	1.512E-01	1.253E-01	6.072E-03
Chr15W	7885870	7886358	114	5.614E-02	1.634E-01	1.073E-01	4.306E-02
Chr15W	7886862	7887349	88	4.561E-01	3.522E-01	-1.039E-01	1.467E-03
Chr15W	7901424	7901900	119	4.423E-01	2.533E-01	-1.890E-01	4.086E-02
Chr15W	7916373	7916864	135	5.997E-02	1.631E-01	1.032E-01	0.000E+00

Chr15W	7920319	7920810	166	3.250E-01	1.541E-01	-1.709E-01	2.120E-02
Chr15W	7963142	7963616	139	1.472E-01	2.517E-01	1.045E-01	1.000E-07
Chr15W	7964131	7964630	171	2.125E-01	6.532E-02	-1.472E-01	1.112E-04
Chr15W	7968631	7969123	149	1.205E-01	2.349E-01	1.144E-01	8.950E-05
Chr15W	7982124	7982621	164	5.062E-02	1.944E-01	1.438E-01	2.162E-04
Chr15W	7986626	7987119	141	6.968E-02	2.598E-01	1.901E-01	1.627E-03
Chr15W	7987621	7988116	177	2.210E-02	1.494E-01	1.273E-01	0.000E+00
Chr15W	8040509	8041003	163	5.129E-01	3.006E-01	-2.123E-01	1.000E-06
Chr15W	8043455	8043917	122	5.212E-02	1.800E-01	1.279E-01	2.120E-03
Chr15W	8078427	8078831	92	4.655E-01	3.129E-01	-1.527E-01	7.764E-03
Chr15W	8168118	8168592	150	3.217E-01	1.594E-01	-1.623E-01	2.355E-03
Chr15W	8191577	8192072	165	2.593E-02	1.446E-01	1.187E-01	1.050E-05
Chr15W	8192074	8192566	187	7.135E-02	1.805E-01	1.091E-01	2.326E-02
Chr15W	8203002	8203495	107	1.339E-01	3.374E-01	2.035E-01	6.100E-06
Chr15W	8217405	8217894	150	5.140E-02	1.921E-01	1.407E-01	1.160E-05
Chr15W	8217897	8218380	173	8.492E-02	2.675E-01	1.825E-01	1.520E-04
Chr15W	8219863	8220348	157	6.591E-02	2.000E-01	1.341E-01	7.700E-06
Chr15W	8238362	8238828	176	3.809E-01	2.682E-01	-1.126E-01	2.319E-02
Chr15W	8245046	8245512	78	4.771E-02	1.808E-01	1.331E-01	0.000E+00
Chr15W	8288131	8288616	174	3.015E-01	1.623E-01	-1.392E-01	4.025E-04
Chr15W	8300615	8301083	154	2.095E-01	3.685E-01	1.590E-01	5.740E-05
Chr15W	8381013	8381508	127	3.265E-02	1.723E-01	1.397E-01	0.000E+00
Chr15W	8392989	8393468	126	1.236E-01	2.747E-01	1.511E-01	1.591E-02
Chr15W	8541758	8542208	205	4.430E-01	3.299E-01	-1.131E-01	6.400E-06
Chr15W	8607826	8608270	50	1.347E-01	2.936E-02	-1.053E-01	2.252E-02
Chr15W	8663943	8664439	159	2.657E-01	8.513E-02	-1.805E-01	4.523E-04
Chr15W	8667440	8667939	88	3.380E-01	4.856E-01	1.476E-01	2.000E-06
Chr15W	8689941	8690415	119	3.839E-01	5.190E-01	1.351E-01	1.729E-02
Chr15W	8691442	8691933	168	6.875E-02	1.913E-01	1.225E-01	2.680E-05
Chr15W	8716943	8717429	175	3.746E-03	1.262E-01	1.225E-01	1.000E-07
Chr15W	8743427	8743922	159	2.543E-01	1.351E-01	-1.192E-01	5.900E-06
Chr15W	8771503	8771997	150	3.880E-01	2.873E-01	-1.007E-01	1.300E-06
Chr15W	8772506	8772988	134	4.287E-01	3.202E-01	-1.085E-01	0.000E+00
Chr15W	8781426	8781918	193	5.282E-02	2.298E-01	1.770E-01	1.700E-06
Chr15W	8789867	8790354	184	1.877E-01	8.570E-02	-1.020E-01	3.559E-02
Chr15W	8830816	8831275	67	5.875E-01	4.332E-01	-1.543E-01	6.450E-03
Chr15W	8840900	8841330	133	2.416E-01	3.734E-01	1.318E-01	2.250E-05
Chr15W	8849100	8849593	126	1.243E-01	7.766E-03	-1.165E-01	3.439E-03
Chr15W	8874365	8874835	117	9.319E-02	2.315E-01	1.383E-01	1.755E-04
Chr15W	8899764	8900256	147	1.023E-01	2.116E-01	1.093E-01	2.925E-04
Chr15W	8905700	8906186	189	6.863E-02	2.315E-01	1.629E-01	2.826E-04
Chr15W	8906192	8906681	161	7.209E-02	2.763E-01	2.042E-01	0.000E+00
Chr15W	8906686	8907177	124	8.054E-02	2.522E-01	1.716E-01	4.793E-03
Chr15W	8907184	8907673	151	4.930E-02	2.568E-01	2.075E-01	0.000E+00
Chr15W	8913113	8913606	172	9.771E-02	2.658E-01	1.681E-01	3.813E-04
Chr15W	8913611	8914097	170	4.318E-02	2.011E-01	1.580E-01	0.000E+00
Chr15W	8914105	8914592	166	1.094E-01	2.205E-01	1.112E-01	1.234E-04

Chr15W	8919540	8920034	201	1.490E-01	3.057E-02	-1.184E-01	3.635E-04
Chr15W	8933653	8934076	91	7.526E-02	2.269E-01	1.516E-01	5.000E-07
Chr15W	8934547	8935005	138	2.093E-01	3.289E-01	1.195E-01	0.000E+00
Chr15W	8948397	8948856	52	3.030E-01	1.544E-01	-1.485E-01	2.900E-06
Chr15W	8951763	8952242	161	2.397E-01	5.233E-02	-1.873E-01	0.000E+00
Chr15W	8956950	8957419	86	3.546E-02	2.951E-01	2.596E-01	2.860E-05
Chr15W	8957421	8957904	189	1.123E-01	2.814E-01	1.691E-01	3.370E-02
Chr15W	8971925	8972400	100	1.245E-01	2.281E-01	1.036E-01	8.900E-06
Chr15W	8977900	8978365	100	3.672E-01	5.115E-01	1.443E-01	1.000E-07
Chr15W	9003466	9003900	150	1.068E-01	6.054E-03	-1.008E-01	4.466E-02
Chr15W	9015060	9015553	123	1.542E-01	4.505E-02	-1.092E-01	0.000E+00
Chr15W	9027943	9028436	176	3.056E-01	1.221E-01	-1.835E-01	1.511E-02
Chr15W	9049649	9050133	183	2.222E-01	9.436E-02	-1.278E-01	2.870E-02
Chr15W	9050136	9050621	191	2.950E-01	1.248E-01	-1.701E-01	2.362E-02
Chr15W	9053056	9053539	74	4.046E-01	2.460E-01	-1.587E-01	2.987E-03
Chr15W	9054032	9054514	93	4.996E-01	3.666E-01	-1.329E-01	8.000E-07
Chr15W	9966426	9966570	22	2.357E-01	1.123E-01	-1.234E-01	9.023E-03
Chr15W	10465791	10466134	37	3.671E-01	2.010E-01	-1.661E-01	1.833E-03
Chr15W	10542227	10542498	24	2.060E-01	3.478E-01	1.419E-01	3.105E-02
Chr15W	10927564	10927680	17	3.704E-01	1.702E-01	-2.002E-01	1.782E-04
Chr15W	10998660	10998917	39	3.422E-01	2.386E-01	-1.036E-01	3.000E-06
Chr15W	11021038	11021477	36	1.793E-01	2.961E-01	1.167E-01	2.825E-04
Chr15W	11209885	11210275	26	1.511E-01	3.078E-01	1.566E-01	7.436E-03
Chr15W	12052277	12052437	28	2.695E-01	4.218E-01	1.522E-01	3.306E-03
Chr15W	12304358	12304640	27	2.578E-01	1.207E-01	-1.372E-01	1.507E-03
Chr15W	12351429	12351683	31	2.764E-01	1.022E-01	-1.742E-01	2.300E-06
Chr15W	13713123	13713316	39	2.197E-01	3.918E-01	1.721E-01	6.695E-03
Chr15W	13738516	13738763	18	4.687E-01	2.075E-01	-2.612E-01	6.919E-04
Chr15W	15572937	15573232	53	2.853E-01	1.769E-01	-1.084E-01	2.501E-02
Chr15Z	645420	645742	30	1.620E-01	2.797E-01	1.177E-01	4.920E-05
Chr15Z	1186706	1186835	9	2.251E-01	5.332E-01	3.081E-01	8.728E-04
Chr15Z	2111676	2111826	31	5.282E-01	4.149E-01	-1.133E-01	3.017E-03
Chr15Z	2484365	2484854	73	1.515E-01	4.154E-02	-1.099E-01	3.076E-02
Chr15Z	2617668	2617771	18	1.174E-01	1.349E-02	-1.039E-01	1.744E-02
Chr15Z	2662545	2663029	129	3.038E-01	1.981E-01	-1.056E-01	2.173E-02
Chr15Z	2689436	2689871	85	2.306E-01	1.047E-01	-1.259E-01	2.300E-06
Chr15Z	2773876	2774336	155	3.844E-01	2.672E-01	-1.172E-01	0.000E+00
Chr15Z	3290088	3290585	160	2.599E-01	1.588E-01	-1.011E-01	5.017E-04
Chr15Z	3454249	3454729	39	1.192E-01	2.312E-01	1.120E-01	2.110E-02
Chr15Z	3504446	3504824	114	1.655E-01	2.879E-01	1.224E-01	1.800E-02
Chr15Z	3535378	3535868	102	2.684E-01	1.470E-01	-1.214E-01	4.293E-02
Chr15Z	3587883	3588364	110	3.848E-01	2.447E-01	-1.401E-01	3.816E-04
Chr15Z	3669799	3670224	99	4.022E-01	2.359E-01	-1.663E-01	1.284E-04
Chr15Z	3724407	3724895	209	3.914E-01	2.616E-01	-1.298E-01	2.443E-04
Chr15Z	3858668	3859057	58	3.428E-02	1.568E-01	1.225E-01	1.453E-02
Chr15Z	3919721	3920050	40	7.256E-02	2.072E-01	1.347E-01	2.420E-05
Chr15Z	4150878	4151363	202	2.856E-01	1.561E-01	-1.295E-01	3.110E-05

Chr15Z	4412260	4412708	95	2.967E-01	1.766E-01	-1.201E-01	2.438E-02
Chr15Z	4447609	4448096	75	5.142E-01	4.121E-01	-1.021E-01	2.199E-04
Chr15Z	4461365	4461851	125	2.818E-01	1.610E-01	-1.208E-01	1.669E-02
Chr15Z	4466293	4466772	177	2.404E-01	1.019E-01	-1.385E-01	4.718E-04
Chr15Z	4481469	4481955	186	3.070E-01	1.818E-01	-1.252E-01	6.029E-03
Chr15Z	4693531	4694019	225	1.845E-01	8.281E-02	-1.017E-01	1.323E-02
Chr15Z	4714375	4714870	180	5.196E-01	4.186E-01	-1.010E-01	1.090E-05
Chr15Z	4770925	4771414	183	4.428E-01	2.770E-01	-1.658E-01	8.410E-05
Chr15Z	4771420	4771914	181	4.023E-01	2.844E-01	-1.179E-01	0.000E+00
Chr15Z	4794363	4794842	106	4.435E-01	3.368E-01	-1.066E-01	9.016E-03
Chr15Z	5181151	5181645	149	4.563E-01	3.551E-01	-1.013E-01	1.653E-02
Chr15Z	5196071	5196559	192	3.051E-01	1.928E-01	-1.122E-01	3.020E-05
Chr15Z	5231275	5231693	60	5.467E-01	4.270E-01	-1.198E-01	1.167E-02
Chr15Z	5267226	5267612	63	2.493E-01	1.230E-01	-1.263E-01	7.100E-06
Chr15Z	6539663	6539994	48	1.732E-01	6.967E-02	-1.036E-01	3.449E-03
Chr15Z	7405076	7405233	17	2.152E-01	1.073E-01	-1.079E-01	8.419E-03
Chr15Z	8453795	8453879	23	8.700E-02	1.918E-01	1.048E-01	7.141E-03
Chr15Z	8856266	8856586	43	7.933E-02	1.878E-01	1.084E-01	2.127E-04
Chr15Z	9751721	9751990	39	4.742E-01	2.327E-01	-2.415E-01	4.370E-05
Chr15Z	10344282	10344684	33	4.444E-01	3.182E-01	-1.262E-01	7.608E-03
Chr15Z	10567192	10567537	54	5.370E-01	3.986E-01	-1.385E-01	2.027E-02
Chr15Z	11384610	11384951	41	4.601E-01	3.436E-01	-1.165E-01	1.864E-03
Chr15Z	11487251	11487356	15	3.131E-01	1.770E-01	-1.361E-01	1.493E-03
Chr15Z	11757541	11757886	45	2.376E-01	1.305E-01	-1.072E-01	1.994E-02
Chr15Z	12248712	12249135	44	8.209E-02	2.842E-01	2.021E-01	2.100E-06
Chr15Z	12458772	12459216	67	3.405E-01	2.180E-01	-1.224E-01	8.900E-06
Chr15Z	13208908	13209089	27	1.723E-01	2.763E-01	1.040E-01	1.989E-03
Chr15Z	13224456	13224849	64	1.553E-01	2.691E-01	1.138E-01	8.242E-04
Chr16	752615	753092	182	1.633E-01	3.669E-01	2.036E-01	2.589E-02
Chr16	863974	864457	129	6.372E-01	5.083E-01	-1.289E-01	5.390E-05
Chr16	872282	872703	76	3.203E-01	1.664E-01	-1.539E-01	2.345E-02
Chr16	876540	877034	101	5.986E-01	4.663E-01	-1.322E-01	1.213E-03
Chr16	969448	969945	172	3.536E-01	1.946E-01	-1.590E-01	2.867E-02
Chr16	1029538	1030033	197	2.157E-01	3.479E-01	1.321E-01	3.238E-02
Chr16	1439960	1440447	75	1.104E-01	2.523E-01	1.419E-01	5.397E-03
Chr16	1546108	1546595	95	5.434E-01	4.326E-01	-1.108E-01	4.050E-05
Chr16	1547092	1547576	160	2.948E-01	1.894E-01	-1.054E-01	5.991E-04
Chr16	1565972	1566464	106	4.497E-01	2.136E-01	-2.362E-01	1.163E-02
Chr16	1628193	1628671	112	2.835E-01	1.641E-01	-1.195E-01	3.594E-04
Chr16	1653230	1653649	72	2.698E-01	1.554E-01	-1.144E-01	9.407E-03
Chr16	1723839	1724325	128	1.773E-01	2.760E-02	-1.497E-01	6.000E-07
Chr16	1724329	1724781	95	4.876E-01	6.398E-01	1.522E-01	1.162E-03
Chr16	1816084	1816581	118	5.612E-01	4.574E-01	-1.038E-01	6.940E-05
Chr16	2020460	2020950	160	2.874E-01	1.832E-01	-1.042E-01	7.000E-07
Chr16	2343573	2344067	171	2.381E-01	1.000E-01	-1.382E-01	1.448E-02
Chr16	2460198	2460691	195	3.439E-01	2.430E-01	-1.008E-01	2.615E-04
Chr16	2640440	2640901	69	4.322E-01	2.980E-01	-1.342E-01	2.300E-06

Chr16	2833257	2833733	120	4.446E-01	3.158E-01	-1.288E-01	6.808E-03
Chr16	2925972	2926464	165	3.532E-01	2.319E-01	-1.213E-01	0.000E+00
Chr16	3252132	3252627	197	1.709E-01	6.698E-02	-1.040E-01	3.240E-02
Chr16	3420728	3420963	50	2.674E-01	1.303E-01	-1.372E-01	1.443E-04
Chr16	3602499	3602984	84	3.461E-01	2.398E-01	-1.063E-01	2.000E-07
Chr16	3624954	3625445	163	1.955E-01	8.297E-02	-1.125E-01	9.387E-04
Chr16	3820749	3821248	183	2.993E-01	1.860E-01	-1.132E-01	5.473E-03
Chr16	4023012	4023504	185	2.576E-01	1.347E-01	-1.229E-01	5.772E-04
Chr16	4161057	4161554	223	2.788E-01	1.435E-01	-1.353E-01	4.729E-04
Chr16	4161555	4162053	201	2.930E-01	1.801E-01	-1.129E-01	1.076E-03
Chr16	4162054	4162552	184	2.893E-01	1.862E-01	-1.031E-01	8.580E-05
Chr16	4316277	4316442	25	2.911E-01	1.647E-01	-1.264E-01	1.855E-04
Chr16	4513272	4513768	201	3.376E-01	2.314E-01	-1.062E-01	4.481E-02
Chr16	4904598	4905087	123	4.060E-01	2.526E-01	-1.534E-01	9.500E-06
Chr16	4925415	4925898	183	2.884E-01	1.883E-01	-1.001E-01	1.000E-07
Chr16	5195321	5195688	64	1.723E-01	2.922E-02	-1.431E-01	1.935E-03
Chr16	5476269	5476670	136	5.843E-01	4.593E-01	-1.249E-01	9.127E-03
Chr16	5640971	5641448	109	4.188E-01	9.216E-02	-3.267E-01	1.689E-03
Chr16	5641518	5641964	174	3.402E-01	2.070E-01	-1.332E-01	2.256E-02
Chr16	5778227	5778705	136	1.592E-01	2.897E-01	1.304E-01	1.232E-03
Chr16	6399370	6399864	131	1.730E-01	3.298E-01	1.568E-01	1.380E-05
Chr16	6433967	6434267	58	1.688E-01	3.253E-04	-1.685E-01	0.000E+00
Chr16	6696267	6696439	51	2.748E-01	4.293E-01	1.545E-01	1.568E-03
Chr16	7091291	7091757	57	2.001E-01	3.176E-01	1.175E-01	1.923E-02
Chr16	7405879	7406285	61	2.193E-01	1.156E-01	-1.037E-01	1.000E-07
Chr16	7576403	7576834	66	3.263E-01	2.256E-01	-1.007E-01	7.691E-04
Chr16	7923090	7923553	90	4.610E-01	3.385E-01	-1.225E-01	1.317E-04
Chr16	7923575	7924039	80	3.155E-01	2.008E-01	-1.147E-01	6.000E-07
Chr16	8165425	8165895	118	3.712E-01	2.636E-01	-1.076E-01	2.188E-02
Chr16	8440372	8440870	132	3.444E-01	1.982E-01	-1.463E-01	1.978E-02
Chr16	8552067	8552555	163	2.798E-01	1.618E-01	-1.180E-01	1.780E-05
Chr16	8592865	8593215	41	1.567E-01	2.691E-01	1.123E-01	2.678E-04
Chr16	8600480	8600914	64	3.230E-01	4.627E-01	1.397E-01	6.480E-04
Chr16	8660249	8660743	200	3.398E-01	2.117E-01	-1.281E-01	9.390E-05
Chr16	8894108	8894606	200	3.153E-01	1.787E-01	-1.366E-01	5.854E-03
Chr16	9165967	9166463	128	4.228E-01	3.139E-01	-1.089E-01	9.500E-06
Chr16	10894067	10894563	156	2.559E-01	1.433E-01	-1.126E-01	0.000E+00
Chr16	11137967	11138428	89	2.758E-01	1.189E-01	-1.569E-01	8.000E-07
Chr16	11505158	11505653	142	8.317E-02	2.044E-01	1.213E-01	1.482E-03
Chr16	11859822	11860311	180	3.670E-01	2.584E-01	-1.087E-01	7.105E-03
Chr16	12672259	12672754	146	4.455E-01	3.190E-01	-1.264E-01	4.906E-04
Chr16	12730421	12730901	74	4.870E-01	3.849E-01	-1.021E-01	1.360E-05
Chr16	12768505	12768675	33	3.591E-01	1.794E-01	-1.797E-01	2.836E-03
Chr16	12804587	12805067	123	3.466E-01	2.307E-01	-1.159E-01	6.169E-04
Chr16	12886984	12887158	44	3.164E-01	5.350E-01	2.186E-01	1.181E-02
Chr16	12926504	12926965	134	4.404E-01	2.742E-01	-1.663E-01	4.835E-03
Chr16	13435761	13436245	83	3.971E-01	2.915E-01	-1.056E-01	9.212E-03

Chr16	13496596	13497087	70	3.060E-01	1.891E-01	-1.169E-01	4.893E-04
Chr16	13545899	13546397	151	4.102E-01	2.728E-01	-1.375E-01	2.081E-02
Chr16	13840113	13840596	139	1.316E-01	3.123E-01	1.806E-01	1.787E-02
Chr16	14342435	14342929	111	3.328E-01	4.824E-01	1.495E-01	1.929E-02
Chr16	14481822	14482313	153	5.494E-01	3.717E-01	-1.777E-01	1.044E-02
Chr16	14545063	14545557	137	1.806E-01	3.062E-01	1.256E-01	2.525E-02
Chr16	15013131	15013623	75	3.439E-01	2.233E-01	-1.206E-01	6.224E-03
Chr16	15350475	15350951	48	4.798E-01	3.326E-01	-1.473E-01	1.323E-02
Chr16	15363742	15364228	166	4.620E-01	3.518E-01	-1.102E-01	0.000E+00
Chr16	16697673	16698165	184	3.273E-01	2.189E-01	-1.084E-01	2.240E-04
Chr16	18747501	18747997	53	6.386E-01	4.412E-01	-1.974E-01	1.200E-06
Chr16	18983919	18984413	118	1.370E-01	2.672E-02	-1.103E-01	1.243E-03
Chr16	19084846	19085329	97	2.772E-01	1.510E-01	-1.262E-01	3.008E-04
Chr16	20127836	20128319	91	6.792E-01	5.523E-01	-1.268E-01	8.295E-03
Chr16	20166018	20166433	34	3.659E-01	2.350E-01	-1.309E-01	2.000E-06
Chr16	20765342	20765830	197	2.469E-01	1.215E-01	-1.254E-01	5.753E-04
Chr16	20784225	20784716	149	3.059E-01	1.852E-01	-1.207E-01	5.200E-06
Chr16	20945425	20945922	122	3.710E-01	2.687E-01	-1.023E-01	1.055E-02
Chr16	21204411	21204901	164	3.442E-01	2.401E-01	-1.041E-01	8.730E-04
Chr16	21280598	21281096	173	2.415E-01	1.377E-01	-1.038E-01	6.147E-04
Chr16	21368355	21368841	136	7.210E-02	1.949E-01	1.228E-01	3.800E-03
Chr16	21368909	21369346	82	2.967E-01	1.638E-01	-1.328E-01	3.428E-02
Chr16	21480929	21481410	99	5.370E-01	4.173E-01	-1.197E-01	1.147E-02
Chr16	21512600	21513095	158	6.210E-01	4.855E-01	-1.355E-01	4.174E-04
Chr16	21606626	21607123	208	2.249E-01	5.057E-02	-1.743E-01	5.449E-03
Chr16	22087945	22088422	83	2.795E-01	1.166E-01	-1.629E-01	1.353E-03
Chr16	24248429	24248755	61	5.121E-01	3.158E-01	-1.963E-01	1.840E-05
Chr16	24441698	24442196	164	3.811E-01	2.640E-01	-1.171E-01	4.924E-03
Chr16	24520173	24520659	161	3.472E-01	2.378E-01	-1.095E-01	2.206E-03
Chr16	25189210	25189515	85	4.947E-01	3.711E-01	-1.237E-01	3.480E-05
Chr16	25736811	25737003	9	1.851E-01	1.328E-02	-1.719E-01	1.500E-05
Chr16	25945541	25946040	214	1.709E-01	2.794E-01	1.085E-01	0.000E+00
Chr16	26729914	26730412	164	4.864E-01	3.832E-01	-1.032E-01	2.696E-03
Chr16	27026337	27026819	120	3.716E-01	5.973E-01	2.257E-01	5.886E-03
Chr16	27068896	27069388	133	4.016E-01	5.114E-01	1.098E-01	3.390E-04
Chr16	27092744	27093219	134	3.090E-01	2.027E-01	-1.062E-01	1.700E-05
Chr16	27132716	27132827	25	3.138E-01	1.984E-01	-1.153E-01	4.127E-03
Chr16	27340386	27340884	149	1.446E-01	3.522E-02	-1.094E-01	3.463E-03
Chr16	27458795	27459258	149	6.154E-02	3.352E-01	2.736E-01	1.240E-02
Chr16	27462842	27463321	126	3.311E-01	1.970E-01	-1.342E-01	1.511E-04
Chr16	27536564	27537051	79	4.607E-01	3.170E-01	-1.436E-01	1.600E-06
Chr16	27549844	27550342	130	2.435E-01	7.144E-02	-1.721E-01	4.776E-03
Chr16	27820272	27820730	133	3.492E-01	2.465E-01	-1.027E-01	2.063E-03
Chr16	28171307	28171790	157	2.032E-01	6.157E-02	-1.416E-01	8.381E-03
Chr16	28249199	28249663	184	2.093E-01	9.919E-02	-1.101E-01	3.895E-02
Chr16	28332488	28332983	147	2.592E-01	4.385E-01	1.793E-01	2.622E-03
Chr16	28445582	28446077	134	3.764E-01	2.444E-01	-1.320E-01	4.270E-05

Chr16	28446712	28447209	84	2.749E-01	1.189E-01	-1.560E-01	7.690E-03
Chr16	28490524	28490891	57	3.212E-01	2.158E-01	-1.054E-01	3.026E-02
Chr16	28501699	28502123	56	3.247E-01	1.390E-01	-1.856E-01	1.700E-05
Chr16	28658249	28658744	164	4.552E-01	3.511E-01	-1.041E-01	1.900E-03
Chr16	29359359	29359832	127	2.229E-01	1.105E-01	-1.124E-01	2.468E-02
Chr16	29597003	29597499	191	3.636E-01	2.603E-01	-1.033E-01	2.487E-04
Chr16	29626361	29626854	150	2.786E-01	1.730E-01	-1.056E-01	1.185E-02
Chr16	29627383	29627847	114	1.443E-01	3.109E-01	1.667E-01	5.936E-04
Chr16	29631470	29631587	36	1.758E-01	3.338E-04	-1.754E-01	1.599E-04
Chr16	29644603	29645077	116	4.018E-01	2.808E-01	-1.210E-01	9.955E-04
Chr16	29648887	29649361	122	1.105E-01	9.230E-03	-1.013E-01	8.000E-07
Chr16	29672333	29672814	137	4.048E-01	2.012E-01	-2.036E-01	3.196E-02
Chr16	29690471	29690954	154	4.507E-01	2.631E-01	-1.876E-01	1.197E-03
Chr16	29690959	29691451	125	3.295E-01	2.256E-01	-1.039E-01	1.076E-02
Chr16	29708963	29709434	100	1.195E-01	2.255E-01	1.060E-01	2.400E-06
Chr16	29727878	29728240	66	1.390E-01	2.563E-01	1.174E-01	1.780E-02
Chr16	29777282	29777611	67	1.515E-01	4.512E-02	-1.064E-01	2.278E-02
Chr16	30257560	30258042	146	2.486E-01	1.471E-01	-1.015E-01	2.233E-02
Chr16	30462335	30462785	117	3.461E-01	2.398E-01	-1.064E-01	8.259E-04
Chr16	31062487	31062970	140	1.816E-01	4.904E-02	-1.325E-01	3.533E-04
Chr16	31181788	31182274	136	2.398E-01	1.283E-01	-1.115E-01	3.852E-03
Chr16	31217122	31217290	30	3.774E-01	3.388E-02	-3.435E-01	0.000E+00
Chr16	31301194	31301689	178	2.818E-01	1.803E-01	-1.015E-01	2.761E-04
Chr16	31346412	31346660	22	1.941E-01	2.270E-02	-1.714E-01	7.400E-06
Chr16	31384118	31384459	76	5.587E-01	3.157E-01	-2.429E-01	4.860E-04
Chr16	31415459	31415563	36	1.908E-01	4.701E-01	2.793E-01	9.000E-07
Chr16	31570508	31570822	68	3.295E-01	2.099E-01	-1.196E-01	1.657E-03
Chr16	31682243	31682576	104	2.999E-01	9.173E-04	-2.989E-01	0.000E+00
Chr16	31691363	31691683	60	2.707E-01	1.360E-01	-1.347E-01	7.295E-03
Chr16	31700379	31700656	59	2.984E-01	8.336E-02	-2.150E-01	2.950E-05
Chr16	31718926	31719337	49	4.277E-01	2.917E-01	-1.360E-01	1.145E-04
Chr16	31731768	31732182	96	3.508E-01	1.928E-01	-1.581E-01	1.900E-06
Chr16	31748336	31748824	91	3.967E-01	4.995E-01	1.029E-01	3.583E-02
Chr16	31758567	31758935	79	2.601E-01	4.252E-04	-2.596E-01	0.000E+00
Chr16	31759391	31759695	52	1.306E-02	1.717E-01	1.586E-01	1.000E-07
Chr16	31761557	31762054	126	1.063E-01	7.225E-04	-1.055E-01	3.934E-02
Chr16	31765081	31765303	49	2.009E-01	7.696E-04	-2.001E-01	1.900E-06
Chr16	31783364	31783844	80	1.478E-01	3.122E-01	1.644E-01	1.465E-02
Chr16	32159968	32160087	47	7.690E-01	4.818E-01	-2.872E-01	6.880E-05
Chr17	94553	95047	122	3.417E-01	2.105E-01	-1.313E-01	8.610E-05
Chr17	115604	116102	167	2.492E-01	1.471E-01	-1.021E-01	3.795E-02
Chr17	133093	133550	144	4.127E-01	2.646E-01	-1.480E-01	1.057E-03
Chr17	243404	243902	124	4.705E-01	2.427E-01	-2.277E-01	1.155E-02
Chr17	274385	274881	155	1.620E-01	2.662E-01	1.041E-01	9.743E-03
Chr17	328350	328841	95	1.717E-01	2.728E-01	1.011E-01	3.993E-04
Chr17	603914	604343	141	3.349E-01	1.588E-01	-1.761E-01	5.000E-07
Chr17	1046694	1046818	32	5.302E-01	2.597E-01	-2.706E-01	2.534E-03

Chr17	1048161	1048564	86	1.682E-03	1.804E-01	1.787E-01	3.613E-03
Chr17	1048669	1049165	96	4.315E-01	3.048E-01	-1.266E-01	1.824E-04
Chr17	1160684	1161160	99	3.851E-01	2.518E-01	-1.334E-01	1.686E-02
Chr17	1169554	1170041	141	3.605E-01	2.353E-01	-1.252E-01	1.632E-04
Chr17	1394033	1394517	172	1.528E-01	2.687E-01	1.158E-01	8.446E-03
Chr17	1394568	1394988	99	1.357E-01	3.025E-01	1.667E-01	6.224E-03
Chr17	1433594	1434090	143	2.677E-01	1.255E-01	-1.422E-01	3.000E-07
Chr17	1462497	1462993	229	4.887E-01	3.370E-01	-1.516E-01	1.910E-04
Chr17	1466483	1466965	146	3.049E-01	1.575E-01	-1.474E-01	1.247E-04
Chr17	1479938	1480432	153	2.918E-01	1.504E-01	-1.413E-01	7.350E-05
Chr17	1508450	1508946	163	8.596E-02	2.020E-01	1.160E-01	4.666E-03
Chr17	1564860	1565342	143	3.169E-01	2.027E-01	-1.143E-01	1.000E-07
Chr17	1603898	1604391	113	3.978E-02	1.581E-01	1.183E-01	2.891E-02
Chr17	1617252	1617742	139	3.546E-01	1.868E-01	-1.679E-01	7.061E-04
Chr17	1709206	1709655	157	1.477E-01	2.919E-01	1.443E-01	4.137E-02
Chr17	1714591	1715038	155	4.721E-01	3.647E-01	-1.073E-01	6.135E-03
Chr17	1796503	1796992	97	3.565E-01	2.561E-01	-1.003E-01	9.676E-03
Chr17	1800172	1800524	85	3.638E-01	5.239E-01	1.600E-01	1.729E-02
Chr17	1903734	1904231	139	3.006E-01	2.006E-01	-1.000E-01	6.992E-04
Chr17	1906735	1907222	111	1.989E-01	9.760E-02	-1.013E-01	1.665E-03
Chr17	1986589	1987084	151	2.268E-01	9.446E-02	-1.323E-01	1.192E-03
Chr17	1994554	1995043	98	4.160E-01	3.106E-01	-1.055E-01	3.810E-04
Chr17	2032585	2033023	124	2.109E-02	1.247E-01	1.036E-01	1.589E-02
Chr17	2070235	2070720	157	3.789E-01	2.690E-01	-1.099E-01	4.068E-03
Chr17	2192285	2192784	144	3.065E-01	1.473E-01	-1.592E-01	1.200E-06
Chr17	2318784	2319013	77	2.270E-01	8.331E-02	-1.437E-01	1.194E-03
Chr17	2343430	2343531	24	8.245E-01	6.152E-01	-2.093E-01	1.210E-05
Chr17	2415606	2416040	123	3.023E-01	1.605E-01	-1.417E-01	6.760E-04
Chr17	2449796	2450292	158	2.707E-01	3.943E-01	1.236E-01	5.385E-03
Chr17	2482029	2482286	42	3.396E-01	2.383E-01	-1.013E-01	5.976E-04
Chr17	2566099	2566595	141	3.059E-01	2.015E-01	-1.044E-01	1.215E-04
Chr17	2630014	2630498	135	3.863E-01	2.779E-01	-1.084E-01	1.187E-02
Chr17	2643389	2643848	166	2.803E-01	1.752E-01	-1.051E-01	1.461E-02
Chr17	2645704	2646166	220	2.170E-01	3.334E-02	-1.837E-01	2.691E-03
Chr17	2880086	2880573	159	3.435E-01	2.298E-01	-1.137E-01	9.110E-04
Chr17	2881089	2881540	147	4.863E-01	3.454E-01	-1.409E-01	8.707E-04
Chr17	2889853	2890347	118	4.361E-01	2.952E-01	-1.410E-01	7.176E-03
Chr17	3886715	3886788	35	2.500E-01	6.517E-02	-1.848E-01	6.530E-05
Chr17	4486555	4486952	42	3.661E-01	2.649E-01	-1.012E-01	2.382E-02
Chr17	4525245	4525404	50	8.308E-02	1.966E-01	1.135E-01	1.415E-02
Chr17	4680695	4681193	130	2.839E-01	1.649E-01	-1.190E-01	4.026E-02
Chr17	5051053	5051416	38	1.793E-02	3.852E-01	3.673E-01	7.660E-05
Chr17	5121419	5121917	69	4.296E-01	3.245E-01	-1.051E-01	1.193E-02
Chr17	5485802	5485967	36	6.843E-01	4.593E-01	-2.250E-01	2.400E-06
Chr17	5493441	5493821	153	5.912E-01	4.850E-01	-1.062E-01	4.717E-02
Chr17	5497632	5497756	20	7.433E-01	6.014E-01	-1.420E-01	7.850E-03
Chr17	5875585	5876078	168	8.040E-02	1.972E-01	1.168E-01	2.604E-02

Chr17	5933404	5933895	174	2.804E-01	1.235E-01	-1.569E-01	1.070E-05
Chr17	6371818	6372313	202	2.479E-01	3.493E-01	1.013E-01	3.170E-03
Chr17	6754268	6754765	184	3.483E-01	2.301E-01	-1.182E-01	6.110E-05
Chr17	7435113	7435605	72	4.298E-01	3.036E-01	-1.262E-01	5.548E-04
Chr17	7564697	7565192	163	2.663E-01	1.623E-01	-1.040E-01	2.753E-04
Chr17	8219836	8220329	173	3.263E-01	2.073E-01	-1.191E-01	9.340E-05
Chr17	8462586	8463080	132	4.891E-01	3.725E-01	-1.166E-01	8.000E-07
Chr17	8912408	8912812	62	3.314E-01	6.734E-01	3.420E-01	4.077E-02
Chr17	9495698	9496188	221	3.649E-01	2.628E-01	-1.020E-01	2.390E-02
Chr17	9797176	9797652	95	1.567E-02	1.350E-01	1.193E-01	1.670E-02
Chr17	9871189	9871679	76	7.170E-01	5.080E-01	-2.090E-01	4.000E-07
Chr17	9923229	9923728	179	1.553E-01	4.149E-02	-1.138E-01	3.116E-02
Chr17	9997658	9998113	115	3.599E-01	2.403E-01	-1.196E-01	4.610E-05
Chr17	10041602	10042039	72	1.632E-01	5.027E-02	-1.130E-01	1.772E-03
Chr17	10164236	10164726	147	3.514E-01	1.739E-01	-1.775E-01	2.714E-02
Chr17	10213446	10213935	43	1.575E-01	1.376E-02	-1.437E-01	9.842E-04
Chr17	10240849	10241293	165	1.592E-01	4.343E-02	-1.158E-01	7.265E-03
Chr17	10281054	10281544	89	4.386E-01	3.326E-01	-1.060E-01	1.400E-06
Chr17	10501026	10501523	221	2.291E-01	1.237E-01	-1.053E-01	5.857E-04
Chr17	10713813	10714146	69	9.143E-02	2.068E-01	1.154E-01	3.080E-05
Chr17	10717571	10717763	29	4.558E-01	2.439E-01	-2.119E-01	1.900E-06
Chr17	10728042	10728530	129	2.972E-01	1.258E-01	-1.713E-01	1.120E-03
Chr17	11255300	11255795	126	1.123E-01	1.045E-02	-1.019E-01	8.232E-03
Chr17	11635748	11636222	133	2.534E-01	1.125E-01	-1.409E-01	2.831E-03
Chr17	11735867	11736343	87	7.157E-02	2.828E-01	2.112E-01	1.000E-07
Chr17	11737297	11737700	113	1.482E-01	2.662E-01	1.179E-01	3.157E-03
Chr17	11740156	11740626	65	3.215E-02	3.971E-01	3.649E-01	0.000E+00
Chr17	11745073	11745558	126	1.210E-01	2.442E-01	1.231E-01	9.371E-03
Chr17	11915760	11916256	186	3.884E-01	2.830E-01	-1.055E-01	4.003E-04
Chr17	11916756	11917254	159	3.410E-01	2.398E-01	-1.012E-01	3.135E-04
Chr17	11917756	11918251	164	3.868E-01	2.712E-01	-1.155E-01	1.800E-05
Chr17	11918259	11918753	179	3.469E-01	2.466E-01	-1.003E-01	2.092E-03
Chr17	11958669	11959151	150	2.818E-01	1.784E-01	-1.035E-01	1.300E-05
Chr17	12060700	12061191	143	4.487E-02	2.150E-01	1.701E-01	8.250E-05
Chr17	12311144	12311308	36	3.502E-01	1.339E-01	-2.163E-01	5.810E-05
Chr17	12324599	12324708	17	9.278E-01	6.626E-01	-2.652E-01	2.600E-06
Chr18	82510	83005	151	2.139E-01	9.057E-02	-1.233E-01	1.277E-03
Chr18	117681	117959	56	5.098E-01	6.802E-01	1.704E-01	7.384E-03
Chr18	118567	119060	120	3.983E-01	2.656E-01	-1.327E-01	1.159E-04
Chr18	877533	878020	132	7.947E-04	1.821E-01	1.813E-01	0.000E+00
Chr18	903025	903519	181	2.917E-01	1.839E-01	-1.078E-01	1.710E-04
Chr18	948523	949018	140	2.603E-01	1.526E-01	-1.077E-01	6.117E-03
Chr18	977019	977517	164	3.936E-01	2.556E-01	-1.381E-01	8.944E-04
Chr18	1067612	1068108	128	3.613E-01	2.432E-01	-1.181E-01	2.200E-06
Chr18	1171510	1172005	98	2.147E-01	9.944E-02	-1.152E-01	2.499E-02
Chr18	1260256	1260750	142	4.223E-01	6.281E-01	2.058E-01	4.434E-02
Chr18	1354263	1354760	153	2.691E-01	1.586E-01	-1.105E-01	5.376E-03

Chr18	1661250	1661739	146	4.103E-01	3.001E-01	-1.102E-01	3.569E-04
Chr18	1758464	1758961	182	4.598E-01	3.110E-01	-1.488E-01	1.000E-06
Chr18	1761462	1761892	115	4.324E-01	2.985E-01	-1.339E-01	2.158E-02
Chr18	2112324	2112811	191	2.910E-01	1.858E-01	-1.052E-01	3.277E-03
Chr18	2277041	2277529	167	3.990E-01	2.959E-01	-1.031E-01	8.860E-05
Chr18	2717150	2717645	124	1.298E-01	2.345E-01	1.047E-01	1.399E-03
Chr18	3405166	3405642	154	2.565E-01	1.485E-01	-1.081E-01	2.030E-05
Chr18	4134009	4134500	191	1.538E-01	3.076E-01	1.538E-01	0.000E+00
Chr18	4183464	4183927	136	5.681E-01	4.628E-01	-1.053E-01	4.110E-05
Chr18	4239197	4239692	115	3.474E-01	2.006E-01	-1.468E-01	1.096E-02
Chr18	4725617	4726059	152	4.393E-01	3.223E-01	-1.170E-01	4.745E-02
Chr18	5109428	5109633	30	4.124E-01	2.863E-01	-1.261E-01	5.193E-04
Chr18	5412754	5413192	88	3.693E-01	2.269E-01	-1.424E-01	1.027E-04
Chr18	5782547	5783040	235	3.823E-01	2.634E-01	-1.189E-01	5.950E-04
Chr18	6768156	6768651	107	2.164E-01	9.602E-02	-1.204E-01	8.634E-03
Chr18	6879213	6879656	85	5.682E-01	4.382E-01	-1.300E-01	4.200E-06
Chr18	7320809	7321287	190	4.556E-01	2.988E-01	-1.568E-01	0.000E+00
Chr18	8367315	8367807	147	4.531E-01	3.495E-01	-1.035E-01	6.000E-07
Chr18	9188268	9188524	30	4.357E-01	3.270E-01	-1.086E-01	1.016E-02
Chr18	9217286	9217779	136	3.648E-01	1.925E-01	-1.722E-01	3.900E-06
Chr18	9280436	9280832	114	3.292E-01	2.228E-01	-1.064E-01	0.000E+00
Chr18	9606705	9606974	48	2.363E-01	9.195E-02	-1.444E-01	1.900E-06
Chr18	9689605	9689973	71	3.919E-01	1.902E-01	-2.017E-01	1.840E-05
Chr18	9815671	9816166	184	3.500E-01	2.288E-01	-1.212E-01	1.002E-02
Chr18	9830563	9831052	203	3.480E-01	2.473E-01	-1.007E-01	2.702E-02
Chr18	9861274	9861755	128	4.211E-01	2.835E-01	-1.375E-01	4.838E-04
Chr18	9901887	9902383	193	3.101E-01	1.724E-01	-1.377E-01	1.246E-02
Chr18	10110992	10111487	136	5.764E-01	4.669E-01	-1.095E-01	0.000E+00
Chr18	10418647	10419143	161	4.089E-01	2.992E-01	-1.097E-01	5.434E-03
Chr18	10426739	10427212	97	1.218E-01	2.720E-01	1.502E-01	5.587E-04
Chr18	10520443	10520929	148	3.039E-01	2.037E-01	-1.002E-01	0.000E+00
Chr18	10775656	10776153	147	2.779E-01	1.703E-01	-1.077E-01	1.264E-02
Chr18	11138470	11138727	39	4.700E-01	6.174E-01	1.474E-01	3.068E-03
Chr18	11169601	11170096	165	1.490E-01	3.208E-02	-1.169E-01	6.400E-03
Chr18	11219077	11219559	108	4.618E-01	3.456E-01	-1.162E-01	1.470E-05
Chr18	11230886	11231381	172	2.697E-01	1.101E-01	-1.596E-01	4.502E-04
Chr18	11231383	11231873	197	2.114E-01	6.002E-02	-1.514E-01	7.360E-04
Chr18	11231886	11232373	191	3.393E-01	1.773E-01	-1.620E-01	1.653E-02
Chr18	11290888	11291384	188	2.921E-01	1.538E-01	-1.383E-01	1.839E-03
Chr18	11372670	11373057	45	5.348E-01	4.083E-01	-1.266E-01	1.266E-03
Chr18	11637195	11637428	35	3.720E-04	2.950E-01	2.946E-01	0.000E+00
Chr18	11640376	11640815	169	3.025E-01	1.028E-01	-1.997E-01	7.643E-03
Chr18	11645056	11645519	97	5.884E-01	4.787E-01	-1.097E-01	7.397E-03
Chr18	11704929	11705398	142	2.240E-01	2.909E-02	-1.949E-01	2.110E-02
Chr18	11707776	11708250	111	2.076E-01	3.515E-01	1.439E-01	1.401E-02
Chr18	11713824	11714277	80	2.274E-01	1.072E-01	-1.202E-01	9.890E-05
Chr19	6131	6617	142	3.066E-01	1.569E-01	-1.497E-01	1.784E-02

Chr19	135778	136273	199	1.153E-01	2.185E-01	1.032E-01	3.030E-05
Chr19	341645	342143	187	4.406E-01	2.450E-01	-1.956E-01	3.141E-03
Chr19	459254	459475	56	2.860E-01	1.086E-01	-1.773E-01	2.000E-07
Chr19	580329	580734	34	1.397E-01	2.354E-02	-1.162E-01	1.575E-03
Chr19	657582	658064	132	1.463E-01	2.093E-02	-1.254E-01	7.761E-03
Chr19	768503	768999	162	2.818E-01	1.365E-01	-1.454E-01	4.542E-02
Chr19	890210	890680	141	2.598E-01	1.595E-01	-1.004E-01	4.158E-02
Chr19	992220	992705	77	3.425E-01	2.332E-01	-1.093E-01	5.636E-04
Chr19	1034484	1034600	27	2.246E-01	9.430E-02	-1.303E-01	5.711E-04
Chr19	2131273	2131748	140	3.022E-01	1.204E-01	-1.818E-01	2.723E-03
Chr19	2134244	2134632	53	4.698E-01	3.566E-01	-1.133E-01	4.786E-02
Chr19	2135618	2136045	152	9.757E-02	2.720E-01	1.745E-01	6.730E-05
Chr19	2191317	2191695	65	2.109E-01	8.538E-02	-1.255E-01	5.117E-03
Chr19	2205572	2205790	42	3.156E-01	7.633E-02	-2.392E-01	0.000E+00
Chr19	2206313	2206498	57	2.999E-01	1.873E-01	-1.126E-01	3.986E-02
Chr19	2226601	2227006	88	5.050E-01	3.119E-01	-1.931E-01	1.000E-06
Chr19	2274136	2274594	140	6.315E-01	5.188E-01	-1.127E-01	8.600E-06
Chr19	2318774	2319174	83	3.928E-01	2.529E-01	-1.398E-01	4.339E-02
Chr19	2399267	2399747	105	8.950E-02	1.899E-01	1.004E-01	1.877E-02
Chr19	2462751	2463237	157	4.605E-01	2.991E-01	-1.614E-01	4.804E-03
Chr19	2474907	2475392	104	4.018E-01	2.664E-01	-1.354E-01	4.100E-02
Chr19	2485911	2486363	100	3.812E-01	1.981E-01	-1.831E-01	2.755E-04
Chr19	2497195	2497314	20	1.934E-01	4.022E-01	2.088E-01	9.466E-04
Chr19	2503792	2504276	126	2.967E-01	1.251E-01	-1.716E-01	3.803E-02
Chr19	2523581	2524072	125	3.062E-01	1.758E-01	-1.304E-01	5.654E-04
Chr19	2662555	2663006	64	4.628E-01	3.417E-01	-1.211E-01	9.853E-04
Chr19	2743875	2744363	140	3.111E-01	2.023E-01	-1.088E-01	4.400E-06
Chr19	2757092	2757571	191	2.955E-01	1.652E-01	-1.303E-01	9.795E-03
Chr19	2804569	2805032	93	3.601E-01	2.336E-01	-1.265E-01	1.054E-03
Chr19	2896829	2897323	94	5.329E-01	3.994E-01	-1.334E-01	2.929E-02
Chr19	3263267	3263690	85	3.037E-01	2.018E-01	-1.019E-01	3.468E-02
Chr19	3286374	3286863	172	4.130E-01	3.113E-01	-1.017E-01	3.160E-03
Chr19	3337381	3337874	86	2.858E-01	1.790E-01	-1.068E-01	2.798E-02
Chr19	3372175	3372669	72	4.746E-01	3.559E-01	-1.187E-01	2.240E-02
Chr19	3476258	3476754	180	2.559E-01	1.381E-01	-1.178E-01	6.720E-05
Chr19	3532052	3532541	176	2.913E-01	1.750E-01	-1.163E-01	1.183E-02
Chr19	3908313	3908808	139	3.319E-02	1.343E-01	1.011E-01	2.049E-02
Chr19	4131432	4131921	153	3.511E-01	2.456E-01	-1.055E-01	9.640E-05
Chr19	4287234	4287714	146	3.477E-01	2.380E-01	-1.096E-01	3.500E-06
Chr19	5360988	5361487	187	6.135E-01	4.491E-01	-1.644E-01	5.121E-04
Chr19	5732866	5733356	180	3.767E-01	2.210E-01	-1.557E-01	2.031E-02
Chr19	6018987	6019480	106	3.523E-01	2.419E-01	-1.104E-01	6.400E-05
Chr19	6027946	6028439	98	3.888E-01	2.874E-01	-1.014E-01	1.318E-04
Chr19	6090340	6090632	33	2.535E-01	3.814E-01	1.279E-01	3.372E-02
Chr19	6118910	6119389	154	2.048E-01	5.597E-02	-1.488E-01	2.320E-02
Chr19	6119391	6119883	144	1.549E-01	4.865E-02	-1.062E-01	2.475E-02
Chr19	6799903	6800293	65	4.460E-01	2.326E-01	-2.135E-01	6.820E-05

Chr19	6916005	6916410	35	3.322E-01	2.207E-01	-1.116E-01	2.229E-02
Chr19	6939103	6939403	116	3.520E-01	4.579E-01	1.058E-01	8.593E-03
Chr19	7146091	7146584	206	3.414E-01	2.264E-01	-1.150E-01	1.010E-05
Chr19	7283442	7283925	153	3.835E-01	2.746E-01	-1.089E-01	6.820E-05
Chr19	7789202	7789518	127	3.256E-01	4.410E-01	1.154E-01	7.660E-03
Chr19	7798289	7798567	40	3.233E-01	4.318E-01	1.085E-01	3.950E-02
Chr19	7813365	7813496	37	3.656E-01	4.982E-01	1.325E-01	2.605E-03
Chr19	7851527	7851984	208	3.998E-01	2.577E-01	-1.421E-01	4.594E-02
Chr19	8217198	8217681	62	2.216E-01	3.533E-01	1.316E-01	2.933E-04
Chr19	8471481	8471978	202	1.679E-01	6.105E-02	-1.069E-01	1.269E-03
Chr19	8711401	8711892	79	1.972E-01	8.080E-02	-1.164E-01	1.160E-05
Chr19	8781731	8782210	103	3.265E-01	2.205E-01	-1.060E-01	8.594E-03
Chr19	8945505	8945993	97	4.788E-01	3.747E-01	-1.041E-01	2.057E-02
Chr19	8956477	8956958	131	2.518E-01	1.466E-01	-1.052E-01	1.714E-02
Chr19	8970929	8971404	143	4.115E-01	1.710E-01	-2.405E-01	7.995E-04
Chr19	8971429	8971924	147	3.293E-01	4.457E-01	1.164E-01	2.143E-03
Chr19	9038106	9038604	180	3.602E-01	1.981E-01	-1.620E-01	4.442E-04
Chr19	9234358	9234846	183	2.539E-01	1.213E-01	-1.326E-01	8.890E-05
Chr19	9913162	9913659	152	5.317E-01	4.200E-01	-1.117E-01	1.980E-05
Chr19	10080295	10080792	184	3.048E-01	1.933E-01	-1.115E-01	5.800E-06
Chr19	10637766	10638263	198	2.576E-01	1.203E-01	-1.374E-01	3.117E-02
Chr19	10759756	10760244	135	4.973E-01	3.863E-01	-1.110E-01	2.270E-05
Chr19	11275334	11275831	200	3.573E-01	2.441E-01	-1.132E-01	1.507E-03
Chr19	11334577	11335067	111	1.927E-01	7.660E-02	-1.161E-01	1.906E-03
Chr19	11495951	11496445	166	3.508E-01	1.949E-01	-1.560E-01	5.114E-03
Chr19	11722501	11722998	157	4.207E-01	3.151E-01	-1.057E-01	1.101E-04
Chr19	11854112	11854604	251	2.641E-01	1.741E-02	-2.467E-01	1.715E-03
Chr19	11873054	11873536	145	3.001E-01	1.711E-01	-1.290E-01	3.223E-03
Chr19	11998798	11999290	169	3.097E-01	2.036E-01	-1.061E-01	5.000E-07
Chr19	12006247	12006736	147	3.544E-01	2.499E-01	-1.045E-01	2.472E-03
Chr19	12018159	12018646	146	2.873E-01	1.845E-01	-1.028E-01	1.200E-06
Chr19	12124097	12124546	97	2.910E-01	4.513E-01	1.603E-01	3.159E-02
Chr19	12238380	12238870	151	3.177E-01	1.835E-01	-1.342E-01	3.000E-07
Chr19	12244858	12245352	88	3.474E-01	2.126E-01	-1.348E-01	1.196E-03
Chr19	12359515	12360012	176	2.623E-01	1.424E-01	-1.199E-01	1.764E-02
Chr19	12428699	12429192	88	1.420E-01	1.734E-02	-1.247E-01	3.233E-03
Chr19	12455930	12456417	164	1.572E-01	5.041E-02	-1.068E-01	2.560E-02
Chr19	12459115	12459267	26	4.292E-01	2.874E-01	-1.418E-01	3.164E-02
Chr19	12512657	12513149	177	1.316E-01	7.535E-03	-1.240E-01	3.319E-02
Chr19	12567524	12568017	136	2.002E-01	9.856E-02	-1.017E-01	9.631E-04
Chr19	12583461	12583953	206	4.447E-01	3.214E-01	-1.233E-01	2.100E-02
Chr19	13161596	13162092	174	2.848E-01	1.478E-01	-1.370E-01	3.418E-02
Chr19	13173063	13173561	203	3.056E-01	1.285E-01	-1.771E-01	7.791E-03
Chr19	13362564	13363053	111	2.569E-01	1.438E-01	-1.131E-01	1.453E-04
Chr19	13378580	13378762	30	2.528E-01	1.143E-01	-1.385E-01	4.177E-04
Chr19	13555986	13556332	71	3.497E-01	2.442E-01	-1.055E-01	5.650E-03
scaffold_104	160334	160815	125	4.693E-01	3.553E-01	-1.140E-01	1.135E-03

scaffold_104	256507	256799	40	4.008E-01	1.773E-01	-2.235E-01	1.000E-07
scaffold_104	304393	304674	33	2.047E-01	2.253E-02	-1.822E-01	1.700E-06
scaffold_109	4541	4939	142	6.260E-01	7.387E-01	1.127E-01	1.439E-02
scaffold_109	5821	6137	45	8.018E-01	5.932E-01	-2.086E-01	3.076E-04
scaffold_109	9580	9633	21	4.256E-01	5.904E-01	1.648E-01	7.782E-03
scaffold_109	12858	13345	132	6.692E-01	5.586E-01	-1.107E-01	4.307E-02
scaffold_109	16207	16365	46	5.250E-01	7.078E-01	1.829E-01	5.337E-04
scaffold_109	16782	17048	89	5.393E-01	6.504E-01	1.111E-01	2.279E-02
scaffold_109	19995	20345	65	6.526E-01	5.378E-01	-1.148E-01	7.979E-04
scaffold_109	22321	22772	178	5.080E-01	6.334E-01	1.254E-01	1.561E-02
scaffold_109	23238	23684	56	7.134E-01	8.896E-01	1.762E-01	2.417E-03
scaffold_109	84100	84365	119	5.693E-01	4.135E-01	-1.559E-01	6.100E-03
scaffold_109	90379	90444	35	3.100E-01	7.871E-04	-3.092E-01	0.000E+00
scaffold_109	174761	175175	126	5.244E-01	6.626E-01	1.382E-01	2.654E-02
scaffold_109	239711	240009	63	3.784E-01	6.256E-01	2.472E-01	1.000E-06
scaffold_109	241306	241663	134	4.427E-01	5.754E-01	1.327E-01	3.564E-02
scaffold_109	242115	242253	44	5.377E-01	2.925E-01	-2.451E-01	3.040E-05
scaffold_109	242839	243227	75	7.279E-01	8.757E-01	1.477E-01	8.201E-04
scaffold_109	245836	246032	65	6.420E-01	4.486E-01	-1.934E-01	3.300E-05
scaffold_109	246288	246724	92	7.182E-01	5.694E-01	-1.488E-01	4.017E-04
scaffold_109	247347	247790	60	8.126E-01	3.204E-01	-4.922E-01	0.000E+00
scaffold_109	253178	253516	105	5.456E-01	7.102E-01	1.646E-01	2.696E-02
scaffold_122	136298	136793	114	5.583E-01	4.577E-01	-1.005E-01	1.547E-02
scaffold_125	144138	144633	225	4.757E-01	3.710E-01	-1.047E-01	6.260E-05
scaffold_125	187517	187998	113	2.891E-01	1.559E-01	-1.332E-01	4.376E-02
scaffold_125	198869	199128	76	3.170E-01	2.152E-01	-1.018E-01	4.242E-04
scaffold_151	29128	29351	38	1.853E-01	8.133E-02	-1.039E-01	5.805E-04
scaffold_151	160760	161248	196	4.448E-01	2.073E-01	-2.375E-01	3.070E-05
scaffold_160	7353	7477	26	3.426E-01	1.208E-01	-2.219E-01	1.872E-03
scaffold_160	32322	32750	62	3.615E-04	1.387E-01	1.384E-01	0.000E+00
scaffold_160	47191	47546	72	2.800E-01	1.370E-01	-1.429E-01	5.850E-05
scaffold_160	99018	99338	52	5.504E-04	1.560E-01	1.554E-01	0.000E+00
scaffold_160	127066	127493	66	2.696E-01	1.080E-01	-1.617E-01	7.000E-07
scaffold_160	187309	187681	50	1.455E-02	2.100E-01	1.954E-01	1.177E-02
scaffold_165	57304	57789	196	1.283E-01	2.508E-01	1.225E-01	4.420E-02
scaffold_165	67571	68048	84	3.805E-01	1.143E-01	-2.661E-01	2.440E-05
scaffold_165	89305	89771	86	2.520E-01	3.700E-01	1.180E-01	1.780E-02
scaffold_165	110596	111089	73	5.563E-01	3.429E-01	-2.134E-01	7.170E-04
scaffold_165	146811	147286	90	6.716E-02	1.824E-01	1.152E-01	5.190E-05
scaffold_167	158117	158457	47	2.495E-01	1.197E-01	-1.298E-01	2.305E-03
scaffold_167	173237	173445	40	1.688E-01	2.829E-01	1.141E-01	1.763E-02
scaffold_167	181243	181595	68	3.492E-01	2.407E-01	-1.085E-01	1.066E-02
scaffold_168	22069	22477	72	1.486E-01	3.262E-02	-1.160E-01	3.731E-03
scaffold_168	34484	34771	78	5.336E-03	2.392E-01	2.339E-01	0.000E+00
scaffold_168	35288	35470	34	1.028E-01	2.319E-01	1.290E-01	3.282E-04
scaffold_168	42207	42657	72	3.101E-02	2.064E-01	1.754E-01	1.500E-06
scaffold_168	48146	48532	66	8.193E-02	2.363E-01	1.544E-01	4.450E-02

scaffold_168	59806	60208	58	3.152E-01	2.065E-01	-1.086E-01	6.436E-03
scaffold_168	64378	64550	48	3.500E-04	1.502E-01	1.498E-01	1.009E-04
scaffold_168	82184	82508	46	1.559E-01	3.993E-01	2.434E-01	1.735E-02
scaffold_168	84488	84887	91	5.717E-02	2.721E-01	2.149E-01	4.690E-05
scaffold_168	95427	95836	106	1.798E-04	1.095E-01	1.093E-01	2.852E-02
scaffold_168	134051	134539	86	5.869E-01	4.388E-01	-1.481E-01	6.199E-04
scaffold_168	151972	152461	114	1.427E-01	2.770E-01	1.343E-01	8.761E-03
scaffold_168	152472	152961	114	3.530E-02	1.496E-01	1.143E-01	2.910E-05
scaffold_168	155427	155844	59	1.903E-01	6.858E-02	-1.217E-01	7.062E-03
scaffold_168	156411	156654	57	9.085E-02	2.144E-01	1.235E-01	1.132E-02
scaffold_168	170179	170669	166	8.636E-02	1.865E-01	1.001E-01	9.335E-03
scaffold_168	171737	172045	61	1.357E-01	3.208E-01	1.851E-01	5.970E-03
scaffold_168	172047	172355	101	5.776E-02	2.354E-01	1.776E-01	2.682E-03
scaffold_168	172758	173074	100	1.151E-01	2.422E-01	1.271E-01	7.599E-03
scaffold_168	186587	186829	42	2.926E-02	1.917E-01	1.624E-01	3.700E-06
scaffold_172	74882	75369	144	1.640E-01	2.697E-01	1.056E-01	4.740E-04
scaffold_172	167938	168414	155	1.007E-01	2.174E-01	1.167E-01	8.817E-03
scaffold_174	58181	58555	82	4.428E-02	2.003E-01	1.560E-01	0.000E+00
scaffold_176	83023	83513	174	3.736E-01	2.566E-01	-1.170E-01	3.400E-06
scaffold_176	160175	160510	82	6.380E-01	4.719E-01	-1.662E-01	2.912E-02
scaffold_176	163082	163532	136	4.139E-01	5.507E-01	1.368E-01	1.832E-02
scaffold_176	179563	179763	27	8.750E-01	7.535E-01	-1.214E-01	8.903E-03
scaffold_176	182571	182676	20	8.932E-01	7.129E-01	-1.804E-01	2.537E-03
scaffold_178	5452	5923	167	4.007E-01	5.541E-01	1.534E-01	4.379E-03
scaffold_178	6706	6792	19	5.238E-01	4.003E-01	-1.236E-01	4.405E-02
scaffold_178	12941	13372	55	9.369E-01	8.241E-01	-1.127E-01	1.020E-03
scaffold_178	16509	16965	149	4.333E-01	6.026E-01	1.694E-01	1.791E-03
scaffold_178	24351	24779	99	5.986E-01	4.012E-01	-1.974E-01	4.210E-05
scaffold_178	26964	27078	16	8.660E-01	6.896E-01	-1.764E-01	1.239E-03
scaffold_178	51468	51721	107	4.086E-01	3.076E-01	-1.010E-01	4.700E-04
scaffold_178	55724	55869	75	4.997E-01	6.571E-01	1.574E-01	2.194E-02
scaffold_178	56024	56508	188	4.056E-01	6.032E-01	1.976E-01	3.136E-03
scaffold_178	57827	58109	42	7.817E-01	6.101E-01	-1.716E-01	2.710E-03
scaffold_178	71422	71556	24	7.458E-01	5.960E-01	-1.498E-01	3.220E-02
scaffold_178	90488	90927	82	5.457E-01	4.257E-01	-1.201E-01	1.527E-02
scaffold_178	98751	99004	80	5.284E-01	6.306E-01	1.022E-01	1.014E-02
scaffold_178	100224	100530	95	7.909E-01	6.412E-01	-1.497E-01	1.277E-02
scaffold_178	111265	111685	94	6.817E-01	5.475E-01	-1.341E-01	1.038E-02
scaffold_178	113235	113489	62	4.690E-01	6.227E-01	1.537E-01	1.193E-03
scaffold_178	113897	114252	96	5.746E-01	7.597E-01	1.850E-01	1.522E-02
scaffold_178	123370	123458	15	9.199E-01	7.711E-01	-1.488E-01	1.667E-03
scaffold_178	123897	124044	27	6.109E-01	7.264E-01	1.154E-01	1.563E-02
scaffold_178	125279	125593	62	5.762E-01	4.737E-01	-1.025E-01	1.504E-03
scaffold_178	128391	128858	154	5.989E-01	3.465E-01	-2.524E-01	1.604E-03
scaffold_178	137674	137812	32	7.126E-01	8.314E-01	1.188E-01	2.596E-02
scaffold_178	151824	152165	68	7.564E-01	6.421E-01	-1.142E-01	2.454E-04
scaffold_178	157414	157548	60	3.599E-01	5.120E-01	1.521E-01	2.575E-02

scaffold_178	158179	158567	33	8.490E-01	7.019E-01	-1.471E-01	3.120E-05
scaffold_178	166436	166623	21	7.270E-01	6.253E-01	-1.017E-01	2.149E-02
scaffold_178	173197	173371	19	8.729E-01	6.615E-01	-2.113E-01	1.438E-03
scaffold_178	175151	175443	32	9.461E-01	8.180E-01	-1.280E-01	3.472E-02
scaffold_178	176120	176548	91	6.104E-01	8.068E-01	1.964E-01	1.417E-02
scaffold_188	1140	1469	107	3.243E-01	4.454E-01	1.211E-01	5.329E-04
scaffold_189	222	319	18	2.353E-01	4.452E-01	2.098E-01	2.875E-04
scaffold_192	3025	3274	39	4.409E-01	2.905E-01	-1.504E-01	2.608E-02
scaffold_193	5299	5764	61	5.383E-01	4.293E-01	-1.090E-01	2.768E-02
scaffold_193	81751	82184	83	3.678E-01	2.453E-01	-1.225E-01	9.000E-07
scaffold_195	25658	25782	54	4.442E-01	3.133E-01	-1.309E-01	4.121E-02
scaffold_195	26671	26792	44	4.451E-01	3.139E-01	-1.312E-01	6.170E-04
scaffold_195	34649	35024	78	5.830E-01	6.868E-01	1.038E-01	4.034E-02
scaffold_195	59600	59783	34	6.771E-01	8.164E-01	1.393E-01	3.424E-02
scaffold_195	65884	66324	207	6.835E-01	5.153E-01	-1.682E-01	4.038E-03
scaffold_195	75005	75451	134	7.179E-01	5.872E-01	-1.306E-01	1.828E-02
scaffold_195	81589	81946	74	5.725E-01	4.440E-01	-1.284E-01	5.519E-03
scaffold_195	84724	84873	57	4.602E-01	3.117E-01	-1.486E-01	8.230E-05
scaffold_195	88435	88809	121	4.253E-01	5.681E-01	1.428E-01	3.926E-03
scaffold_195	101037	101496	215	4.923E-01	6.638E-01	1.715E-01	2.975E-03
scaffold_195	101501	101960	218	4.772E-01	5.929E-01	1.158E-01	1.375E-02
scaffold_195	107043	107486	84	7.473E-01	5.511E-01	-1.962E-01	1.421E-02
scaffold_195	107587	107944	88	6.829E-01	5.323E-01	-1.506E-01	3.031E-03
scaffold_195	118157	118613	112	3.666E-01	4.742E-01	1.076E-01	1.594E-02
scaffold_196	28288	28713	72	6.453E-01	7.533E-01	1.081E-01	1.901E-03
scaffold_196	28715	29134	162	6.001E-01	7.304E-01	1.303E-01	1.640E-05
scaffold_196	44074	44559	122	4.846E-01	3.528E-01	-1.318E-01	2.136E-03
scaffold_196	47110	47462	49	7.194E-01	8.298E-01	1.103E-01	1.590E-05
scaffold_196	52279	52765	107	5.890E-01	6.995E-01	1.105E-01	1.752E-02
scaffold_196	79522	79900	128	4.297E-01	6.281E-01	1.984E-01	3.090E-04
scaffold_196	89351	89504	22	5.887E-01	8.165E-01	2.278E-01	1.338E-04
scaffold_196	103077	103311	105	5.027E-01	3.810E-01	-1.218E-01	3.028E-02
scaffold_196	103777	104034	74	4.286E-01	7.151E-01	2.865E-01	1.853E-03
scaffold_196	133404	133881	156	6.274E-01	4.624E-01	-1.650E-01	1.070E-02
scaffold_196	134374	134858	146	5.589E-01	6.604E-01	1.015E-01	3.618E-02
scaffold_196	138724	138997	68	4.571E-01	3.348E-01	-1.223E-01	3.502E-02
scaffold_196	144836	145238	159	5.329E-01	6.459E-01	1.130E-01	1.346E-03
scaffold_196	155224	155677	131	4.889E-01	6.153E-01	1.264E-01	7.341E-03
scaffold_196	155685	156141	161	5.141E-01	6.446E-01	1.305E-01	1.195E-02
scaffold_196	159242	159645	129	5.356E-01	4.079E-01	-1.278E-01	4.530E-05
scaffold_196	163336	163825	123	5.579E-01	3.769E-01	-1.809E-01	6.100E-04
scaffold_197	146692	147183	114	2.391E-01	1.170E-01	-1.221E-01	7.580E-05
scaffold_197	152651	153100	100	2.963E-01	1.800E-01	-1.164E-01	9.764E-03
scaffold_206	30660	30763	28	1.023E-03	1.308E-01	1.298E-01	1.421E-02
scaffold_206	90768	90930	22	3.830E-02	1.775E-01	1.392E-01	5.910E-05
scaffold_207	120967	121389	102	3.583E-01	2.388E-01	-1.195E-01	2.140E-05
scaffold_209	60669	61144	186	4.617E-01	3.410E-01	-1.208E-01	2.086E-03

scaffold_209	103039	103291	83	6.549E-01	5.134E-01	-1.415E-01	3.674E-02
scaffold_209	108485	108812	76	4.355E-01	7.198E-01	2.843E-01	1.200E-06
scaffold_209	114276	114505	110	4.193E-01	7.007E-01	2.814E-01	1.340E-05
scaffold_209	129863	130014	70	3.527E-01	2.089E-01	-1.438E-01	3.941E-04
scaffold_209	130329	130777	78	8.993E-01	7.653E-01	-1.340E-01	2.568E-03
scaffold_209	136412	136891	85	5.090E-01	6.472E-01	1.382E-01	3.129E-02
scaffold_209	140270	140690	66	6.172E-01	7.246E-01	1.074E-01	2.607E-02
scaffold_209	144499	144564	31	4.708E-01	2.869E-01	-1.839E-01	2.167E-03
scaffold_209	151612	151720	55	3.915E-01	6.044E-01	2.129E-01	8.830E-05
scaffold_209	151858	152122	52	7.902E-01	9.337E-01	1.435E-01	8.265E-03
scaffold_211	16319	16806	164	3.523E-01	2.137E-01	-1.386E-01	3.202E-03
scaffold_211	107801	108211	45	1.045E-01	2.172E-01	1.127E-01	1.700E-06
scaffold_211	123778	124045	47	2.220E-01	4.464E-02	-1.774E-01	5.000E-07
scaffold_211	138441	138831	43	2.871E-01	1.214E-01	-1.657E-01	1.559E-04
scaffold_212	10771	11237	195	5.052E-01	6.070E-01	1.018E-01	1.287E-02
scaffold_212	11252	11732	171	5.093E-01	6.136E-01	1.043E-01	2.819E-02
scaffold_212	12219	12696	151	3.813E-01	4.992E-01	1.179E-01	1.332E-02
scaffold_212	20875	21300	54	8.607E-01	6.918E-01	-1.689E-01	6.000E-07
scaffold_212	39134	39390	55	7.613E-01	6.570E-01	-1.043E-01	5.718E-03
scaffold_212	40638	41044	96	7.780E-01	5.884E-01	-1.895E-01	9.306E-04
scaffold_212	47986	48351	175	5.868E-01	4.693E-01	-1.175E-01	2.286E-02
scaffold_212	54361	54774	162	4.047E-01	5.083E-01	1.036E-01	2.323E-02
scaffold_212	58814	59228	90	5.380E-01	6.854E-01	1.474E-01	1.018E-03
scaffold_212	63379	63753	100	5.502E-01	6.826E-01	1.324E-01	4.946E-02
scaffold_212	67098	67575	141	4.540E-01	5.990E-01	1.449E-01	7.376E-03
scaffold_212	71403	71879	111	6.128E-01	7.228E-01	1.100E-01	3.789E-03
scaffold_212	74138	74466	92	4.463E-01	5.809E-01	1.345E-01	2.755E-03
scaffold_212	75877	76303	106	7.152E-01	8.365E-01	1.213E-01	1.160E-02
scaffold_212	89436	89917	183	5.831E-01	6.987E-01	1.156E-01	1.620E-02
scaffold_212	95088	95437	32	8.271E-01	6.762E-01	-1.508E-01	6.090E-04
scaffold_212	97239	97625	101	5.820E-01	7.383E-01	1.563E-01	3.304E-03
scaffold_212	148910	149025	17	8.726E-01	9.894E-01	1.168E-01	9.890E-05
scaffold_214	8083	8524	108	2.218E-01	1.218E-01	-1.000E-01	1.284E-02
scaffold_214	8526	8968	260	2.816E-01	1.504E-01	-1.312E-01	3.049E-02
scaffold_214	18232	18647	159	3.577E-01	1.732E-01	-1.845E-01	4.698E-03
scaffold_214	92531	93022	145	2.797E-01	1.497E-01	-1.299E-01	7.640E-04
scaffold_214	95146	95547	63	3.071E-01	1.941E-01	-1.130E-01	6.752E-04
scaffold_223	48958	49449	130	4.658E-02	1.595E-01	1.130E-01	2.007E-03
scaffold_223	128764	129214	140	2.421E-01	4.348E-01	1.927E-01	7.400E-06
scaffold_229	42907	43287	42	3.355E-01	2.348E-01	-1.007E-01	2.241E-03
scaffold_230	133386	133730	50	2.964E-01	1.519E-01	-1.445E-01	2.517E-03
scaffold_231	30373	30676	35	3.944E-03	3.752E-01	3.713E-01	5.420E-05
scaffold_234	65567	65768	64	2.984E-01	5.497E-02	-2.434E-01	0.000E+00
scaffold_234	78070	78553	111	2.988E-01	1.368E-01	-1.620E-01	1.839E-02
scaffold_234	86333	86816	133	2.830E-01	6.978E-02	-2.132E-01	4.860E-05
scaffold_238	111046	111485	72	5.052E-01	4.024E-01	-1.027E-01	1.175E-03
scaffold_241	48532	48875	36	5.719E-01	4.553E-01	-1.166E-01	8.721E-03

scaffold_241	50313	50712	48	8.979E-02	2.121E-01	1.223E-01	3.600E-06
scaffold_241	68092	68360	51	3.087E-01	4.665E-01	1.578E-01	4.830E-05
scaffold_249	17394	17749	104	1.221E-01	9.751E-03	-1.123E-01	1.338E-02
scaffold_249	79784	80254	186	3.597E-01	2.312E-01	-1.285E-01	3.488E-02
scaffold_255	237	649	96	5.214E-01	6.418E-01	1.204E-01	7.106E-03
scaffold_255	2895	3368	82	6.919E-01	5.043E-01	-1.877E-01	1.675E-02
scaffold_255	6301	6745	166	4.760E-01	5.954E-01	1.194E-01	6.004E-03
scaffold_255	10604	11085	153	5.112E-01	6.275E-01	1.164E-01	7.000E-06
scaffold_255	11104	11583	198	5.136E-01	6.486E-01	1.350E-01	9.545E-03
scaffold_255	14643	15124	177	3.468E-01	4.564E-01	1.096E-01	1.151E-03
scaffold_255	26198	26672	203	6.414E-01	4.446E-01	-1.968E-01	1.770E-03
scaffold_255	41639	42008	60	6.182E-01	7.969E-01	1.787E-01	3.025E-03
scaffold_255	47418	47916	69	4.211E-01	6.160E-01	1.949E-01	3.400E-06
scaffold_255	50649	51141	172	5.807E-01	6.969E-01	1.161E-01	2.150E-02
scaffold_255	52136	52628	157	4.465E-01	5.668E-01	1.203E-01	3.060E-02
scaffold_255	54118	54608	189	5.496E-01	4.403E-01	-1.093E-01	1.297E-02
scaffold_255	71237	71703	201	4.259E-01	5.976E-01	1.717E-01	9.350E-04
scaffold_255	81225	81682	152	5.034E-01	6.608E-01	1.574E-01	9.113E-03
scaffold_255	81687	82098	104	4.093E-01	5.759E-01	1.666E-01	5.700E-06
scaffold_255	83186	83467	136	5.757E-01	4.500E-01	-1.258E-01	1.744E-02
scaffold_255	85321	85775	86	4.991E-01	6.593E-01	1.602E-01	1.304E-03
scaffold_255	89439	89916	115	6.249E-01	7.940E-01	1.691E-01	8.027E-03
scaffold_255	95907	96383	169	3.888E-01	4.941E-01	1.053E-01	1.423E-02
scaffold_255	98877	99365	113	4.971E-01	6.173E-01	1.202E-01	3.551E-02
scaffold_255	102465	102907	138	4.897E-01	6.579E-01	1.682E-01	1.196E-03
scaffold_255	103950	104305	159	3.940E-01	6.509E-01	2.568E-01	0.000E+00
scaffold_255	108118	108605	116	4.410E-01	5.607E-01	1.198E-01	1.427E-03
scaffold_255	111541	112017	149	4.328E-01	5.480E-01	1.152E-01	3.159E-02
scaffold_255	112031	112515	119	5.778E-01	7.872E-01	2.094E-01	2.385E-04
scaffold_259	19638	20025	60	5.422E-01	7.029E-01	1.607E-01	4.169E-04
scaffold_259	29499	29856	65	7.635E-01	5.102E-01	-2.534E-01	2.484E-04
scaffold_259	69298	69712	218	5.153E-01	6.204E-01	1.051E-01	4.210E-02
scaffold_259	100009	100423	43	5.023E-01	3.926E-01	-1.097E-01	7.452E-03
scaffold_259	109403	109807	51	3.215E-01	4.239E-01	1.024E-01	4.091E-02
scaffold_259	114387	114489	37	4.305E-01	3.188E-01	-1.118E-01	2.176E-02
scaffold_266	33457	33561	21	1.570E-01	3.610E-02	-1.209E-01	3.102E-04
scaffold_266	34340	34656	13	7.100E-02	1.961E-01	1.251E-01	6.541E-03
scaffold_268	96207	96641	126	2.103E-01	1.028E-01	-1.075E-01	9.572E-04
scaffold_272	20768	21259	180	3.216E-01	1.589E-01	-1.627E-01	1.334E-03
scaffold_272	75981	76479	90	2.752E-01	1.400E-01	-1.352E-01	1.813E-04
scaffold_272	76480	76959	118	4.127E-01	3.122E-01	-1.005E-01	1.780E-05
scaffold_273	5511	5981	85	1.995E-01	6.419E-02	-1.353E-01	1.923E-02
scaffold_286	45405	45750	71	5.406E-01	3.679E-01	-1.726E-01	6.810E-04
scaffold_288	53805	54244	134	4.069E-01	5.584E-01	1.515E-01	4.409E-03
scaffold_299	26274	26709	126	3.085E-01	1.325E-01	-1.760E-01	1.000E-05
scaffold_299	47821	48302	82	4.938E-01	3.589E-01	-1.349E-01	1.361E-04
scaffold_299	83469	83961	148	3.109E-01	2.056E-01	-1.053E-01	1.679E-02

scaffold_299	83964	84456	189	3.111E-01	1.595E-01	-1.516E-01	1.184E-04
scaffold_302	22699	23043	53	2.847E-01	1.324E-01	-1.523E-01	4.410E-05
scaffold_302	65538	65756	48	3.176E-01	1.861E-01	-1.315E-01	2.166E-04
scaffold_306	63472	63936	121	3.629E-01	2.625E-01	-1.004E-01	4.595E-03
scaffold_306	96480	96956	78	4.154E-01	2.561E-01	-1.593E-01	2.000E-07
scaffold_308	15267	15395	21	3.487E-01	2.124E-01	-1.363E-01	4.301E-02
scaffold_313	33813	34288	103	2.895E-01	1.437E-01	-1.458E-01	9.098E-03
scaffold_313	44804	45269	99	2.673E-01	1.415E-01	-1.258E-01	2.221E-02
scaffold_313	70935	71121	42	2.449E-01	1.382E-01	-1.067E-01	4.826E-03
scaffold_316	42014	42325	85	2.621E-01	3.872E-01	1.252E-01	2.182E-03
scaffold_317	53297	53578	52	3.932E-01	1.799E-01	-2.134E-01	1.782E-03
scaffold_317	70353	70769	75	1.619E-01	2.725E-01	1.106E-01	2.100E-06
scaffold_318	12565	12991	78	7.551E-01	6.282E-01	-1.269E-01	5.387E-03
scaffold_318	17075	17387	99	4.673E-01	6.739E-01	2.066E-01	1.300E-06
scaffold_318	25701	26128	108	8.319E-01	6.283E-01	-2.036E-01	3.110E-05
scaffold_318	52889	53231	65	6.523E-01	7.604E-01	1.081E-01	2.275E-02
scaffold_318	60357	60488	29	8.589E-01	9.913E-01	1.324E-01	2.400E-06
scaffold_318	63439	63753	111	5.914E-01	4.657E-01	-1.257E-01	1.004E-02
scaffold_318	63761	64069	48	8.674E-01	6.365E-01	-2.309E-01	3.479E-02
scaffold_318	64973	65347	129	6.325E-01	4.029E-01	-2.296E-01	6.550E-05
scaffold_318	67935	68247	72	6.819E-01	5.272E-01	-1.546E-01	1.562E-03
scaffold_318	72305	72378	13	9.251E-01	7.890E-01	-1.361E-01	1.794E-02
scaffold_318	83057	83506	81	6.688E-01	8.038E-01	1.350E-01	3.361E-03
scaffold_318	83635	83856	34	6.352E-01	7.559E-01	1.207E-01	8.180E-03
scaffold_318	89487	89848	91	5.705E-01	8.215E-01	2.510E-01	1.000E-07
scaffold_318	91977	92100	38	4.806E-01	5.938E-01	1.133E-01	4.151E-02
scaffold_32	12690	13089	114	2.737E-01	3.805E-01	1.068E-01	3.740E-02
scaffold_32	112487	112836	54	2.521E-01	1.267E-01	-1.254E-01	0.000E+00
scaffold_32	112962	113126	47	2.827E-01	8.687E-02	-1.958E-01	4.754E-03
scaffold_32	276601	277096	214	4.296E-01	3.206E-01	-1.090E-01	1.767E-03
scaffold_32	456432	456929	227	3.555E-01	2.501E-01	-1.054E-01	8.130E-05
scaffold_32	493516	493874	37	4.648E-01	3.523E-01	-1.124E-01	4.544E-03
scaffold_32	599688	600177	117	4.744E-01	3.676E-01	-1.069E-01	1.536E-02
scaffold_32	788304	788597	56	7.090E-01	5.581E-01	-1.510E-01	1.385E-03
scaffold_32	792213	792575	49	3.375E-01	4.611E-01	1.236E-01	4.705E-02
scaffold_32	807996	808329	34	7.408E-01	9.246E-01	1.838E-01	2.026E-03
scaffold_32	809155	809540	93	5.073E-01	6.556E-01	1.482E-01	1.138E-02
scaffold_32	817645	817716	26	8.782E-01	1.564E-01	-7.218E-01	0.000E+00
scaffold_32	824310	824603	26	5.836E-01	7.906E-01	2.070E-01	1.034E-04
scaffold_320	73597	74023	107	3.702E-01	2.631E-01	-1.070E-01	3.488E-02
scaffold_324	86752	86997	46	1.847E-01	1.095E-02	-1.738E-01	3.890E-05
scaffold_328	37187	37615	176	4.266E-01	2.820E-01	-1.447E-01	5.420E-05
scaffold_328	47434	47741	41	1.888E-01	8.797E-02	-1.008E-01	4.170E-04
scaffold_328	57060	57209	19	1.940E-01	3.750E-04	-1.936E-01	2.000E-07
scaffold_330	16976	17469	111	5.763E-01	7.717E-01	1.955E-01	1.564E-04
scaffold_330	25418	25826	71	1.739E-01	3.001E-01	1.262E-01	1.610E-05
scaffold_330	27110	27400	80	8.082E-02	1.856E-01	1.048E-01	1.075E-02

scaffold_333	56248	56581	52	3.674E-01	2.639E-01	-1.035E-01	8.288E-03
scaffold_333	73626	73757	26	2.916E-01	1.668E-01	-1.248E-01	3.113E-02
scaffold_336	14621	14870	69	2.589E-01	1.233E-01	-1.356E-01	4.864E-04
scaffold_336	35752	36054	35	7.880E-01	6.860E-01	-1.020E-01	2.497E-02
scaffold_336	36741	36831	34	4.723E-01	2.323E-01	-2.401E-01	7.900E-06
scaffold_336	40181	40626	101	5.189E-01	6.669E-01	1.480E-01	6.319E-03
scaffold_336	41075	41516	113	4.970E-01	7.037E-01	2.067E-01	9.017E-04
scaffold_336	42227	42560	32	6.838E-01	8.205E-01	1.367E-01	1.947E-03
scaffold_336	49523	49902	72	7.864E-01	5.667E-01	-2.197E-01	2.000E-05
scaffold_336	50383	50620	32	7.442E-01	8.781E-01	1.339E-01	7.890E-03
scaffold_336	51054	51245	27	4.584E-01	6.016E-01	1.432E-01	3.199E-02
scaffold_336	57653	58014	47	6.894E-01	8.150E-01	1.256E-01	7.342E-03
scaffold_336	58435	58740	105	3.875E-01	5.803E-01	1.927E-01	1.060E-02
scaffold_336	61255	61568	49	4.884E-01	6.060E-01	1.176E-01	1.036E-02
scaffold_345	28818	29027	39	4.663E-01	3.576E-01	-1.088E-01	1.455E-02
scaffold_347	9989	10066	22	1.852E-01	3.815E-01	1.964E-01	2.149E-04
scaffold_350	56755	57217	62	1.190E-01	4.161E-04	-1.186E-01	1.500E-06
scaffold_363	59407	59603	37	3.255E-02	1.580E-01	1.255E-01	2.939E-04
scaffold_363	60182	60299	23	7.321E-02	4.569E-01	3.837E-01	0.000E+00
scaffold_367	22132	22619	150	5.982E-01	7.388E-01	1.406E-01	6.610E-03
scaffold_367	23024	23169	26	8.584E-01	9.624E-01	1.040E-01	3.798E-03
scaffold_367	32047	32464	139	5.365E-01	6.513E-01	1.149E-01	4.659E-02
scaffold_368	64551	65014	168	6.339E-01	4.731E-01	-1.609E-01	1.879E-03
scaffold_368	71061	71371	85	5.545E-01	3.787E-01	-1.758E-01	1.423E-02
scaffold_371	37460	37865	70	2.837E-01	1.784E-01	-1.053E-01	3.011E-04
scaffold_371	63326	63782	98	5.870E-01	4.462E-01	-1.408E-01	4.880E-05
scaffold_374	25656	26113	147	5.896E-01	4.519E-01	-1.377E-01	0.000E+00
scaffold_382	35567	35851	50	3.454E-01	2.226E-01	-1.228E-01	1.695E-03
scaffold_386	55379	55498	19	1.749E-01	2.910E-02	-1.458E-01	4.272E-04
scaffold_39	23455	23946	198	3.809E-01	2.350E-01	-1.460E-01	3.200E-06
scaffold_39	33895	34388	193	1.288E-01	2.330E-01	1.042E-01	4.292E-02
scaffold_39	102505	102968	81	1.960E-01	4.554E-04	-1.956E-01	0.000E+00
scaffold_39	181717	182144	82	1.888E-01	5.029E-02	-1.385E-01	4.354E-03
scaffold_39	183346	183622	47	3.160E-01	1.627E-01	-1.533E-01	3.273E-03
scaffold_39	191617	192091	123	3.420E-01	9.770E-02	-2.443E-01	2.229E-03
scaffold_39	194123	194618	168	4.315E-01	3.203E-01	-1.112E-01	9.602E-03
scaffold_39	221709	221971	74	3.383E-01	1.935E-01	-1.448E-01	1.316E-02
scaffold_39	231469	231951	111	3.023E-01	1.762E-01	-1.261E-01	1.684E-02
scaffold_39	270108	270567	172	1.118E-01	9.840E-03	-1.019E-01	1.898E-02
scaffold_39	291637	292127	102	2.957E-01	1.783E-01	-1.174E-01	7.400E-04
scaffold_39	364612	365082	118	1.964E-01	3.560E-01	1.596E-01	7.830E-05
scaffold_39	366238	366716	112	2.432E-01	1.317E-01	-1.115E-01	6.010E-05
scaffold_39	390528	390948	77	2.897E-01	1.557E-01	-1.340E-01	1.383E-03
scaffold_39	391174	391502	42	1.876E-01	3.817E-02	-1.494E-01	1.100E-04
scaffold_39	411117	411598	71	6.523E-02	1.781E-01	1.129E-01	1.430E-02
scaffold_39	418134	418380	40	2.745E-01	1.635E-01	-1.110E-01	1.726E-02
scaffold_39	418840	419161	92	3.609E-01	2.423E-01	-1.185E-01	3.628E-02

scaffold_39	439349	439846	143	1.546E-01	5.487E-03	-1.492E-01	4.385E-02
scaffold_39	464253	464691	61	1.333E-01	2.335E-01	1.002E-01	1.819E-02
scaffold_39	468284	468675	68	6.091E-03	1.944E-01	1.883E-01	1.000E-07
scaffold_39	470430	470797	63	1.336E-01	2.922E-01	1.586E-01	2.687E-02
scaffold_39	500417	500891	129	2.485E-01	1.395E-01	-1.091E-01	4.950E-05
scaffold_39	530811	531301	122	2.228E-01	7.997E-02	-1.428E-01	3.104E-02
scaffold_39	531304	531790	65	3.266E-01	1.028E-01	-2.238E-01	1.109E-02
scaffold_39	559363	559800	97	1.733E-01	7.322E-02	-1.001E-01	4.484E-02
scaffold_39	617332	617823	92	3.817E-01	2.191E-01	-1.626E-01	1.037E-03
scaffold_39	626078	626540	91	1.930E-01	9.138E-02	-1.016E-01	7.033E-04
scaffold_39	629137	629592	97	1.627E-01	6.765E-03	-1.559E-01	0.000E+00
scaffold_39	635957	636444	164	2.618E-01	1.151E-01	-1.467E-01	1.614E-02
scaffold_39	636448	636934	119	3.221E-01	2.204E-01	-1.017E-01	1.060E-02
scaffold_39	691069	691386	85	2.152E-01	7.531E-04	-2.144E-01	0.000E+00
scaffold_390	31101	31398	50	4.281E-01	2.610E-01	-1.671E-01	2.236E-04
scaffold_390	67047	67487	65	2.940E-02	1.606E-01	1.312E-01	3.000E-07
scaffold_393	65777	66170	59	4.074E-02	1.719E-01	1.312E-01	1.187E-03
scaffold_394	21663	21811	21	4.113E-04	1.423E-01	1.419E-01	4.515E-02
scaffold_394	30741	30912	29	9.217E-02	3.018E-01	2.097E-01	3.000E-07
scaffold_397	2206	2444	52	7.904E-01	6.560E-01	-1.344E-01	4.208E-02
scaffold_397	5969	6287	88	4.538E-01	5.762E-01	1.225E-01	2.832E-02
scaffold_397	40324	40787	139	4.545E-01	3.514E-01	-1.031E-01	5.700E-05
scaffold_407	1257	1731	137	3.077E-01	1.699E-01	-1.378E-01	1.000E-07
scaffold_407	4077	4288	38	4.586E-01	3.122E-01	-1.464E-01	5.160E-03
scaffold_408	676	1111	138	2.777E-01	1.335E-01	-1.441E-01	3.698E-02
scaffold_410	19764	20143	65	1.651E-01	9.687E-03	-1.554E-01	2.364E-02
scaffold_410	39235	39303	23	1.605E-01	4.204E-04	-1.601E-01	3.501E-02
scaffold_410	66969	67172	25	2.788E-01	4.266E-01	1.477E-01	4.999E-02
scaffold_413	59184	59651	180	3.308E-01	2.258E-01	-1.049E-01	3.345E-03
scaffold_418	4966	5379	53	2.841E-01	1.775E-01	-1.066E-01	4.800E-05
scaffold_418	41462	41769	37	2.774E-01	1.340E-01	-1.433E-01	5.163E-03
scaffold_424	10796	10894	18	5.259E-01	7.610E-01	2.351E-01	1.014E-03
scaffold_424	18992	19473	116	6.416E-01	7.425E-01	1.010E-01	1.080E-02
scaffold_425	43012	43128	20	2.355E-01	9.321E-02	-1.423E-01	1.649E-04
scaffold_427	14233	14349	35	1.433E-01	2.489E-01	1.056E-01	1.007E-03
scaffold_427	16780	16860	40	7.424E-01	5.119E-01	-2.305E-01	3.700E-06
scaffold_427	39125	39424	125	3.190E-01	2.190E-01	-1.000E-01	1.408E-03
scaffold_429	9315	9566	65	5.765E-01	3.771E-01	-1.993E-01	9.672E-04
scaffold_429	15816	16151	61	6.772E-01	8.560E-01	1.788E-01	5.310E-05
scaffold_429	16641	16879	87	5.816E-01	3.475E-01	-2.341E-01	6.538E-03
scaffold_429	17625	18076	116	5.667E-01	7.163E-01	1.495E-01	7.962E-03
scaffold_429	30531	30817	119	4.041E-01	5.139E-01	1.098E-01	7.838E-04
scaffold_429	37076	37273	34	8.652E-01	7.553E-01	-1.100E-01	2.020E-03
scaffold_429	46483	46655	36	2.504E-02	3.111E-01	2.860E-01	0.000E+00
scaffold_429	57178	57282	21	5.845E-01	9.928E-01	4.083E-01	0.000E+00
scaffold_431	56037	56528	96	2.514E-01	6.703E-02	-1.844E-01	1.697E-03
scaffold_431	57033	57529	143	4.125E-01	2.939E-01	-1.187E-01	2.480E-02

scaffold_431	60626	61090	100	1.649E-01	8.420E-05	-1.648E-01	5.860E-03
scaffold_442	8387	8807	284	3.101E-01	1.885E-01	-1.216E-01	1.240E-04
scaffold_442	10258	10656	292	1.923E-01	4.771E-02	-1.446E-01	4.565E-03
scaffold_442	36398	36486	37	6.730E-01	5.562E-01	-1.168E-01	3.047E-02
scaffold_442	39009	39260	70	6.295E-01	4.304E-01	-1.991E-01	8.150E-05
scaffold_444	8334	8513	35	8.495E-01	7.119E-01	-1.375E-01	9.148E-03
scaffold_444	9165	9638	187	4.075E-01	5.243E-01	1.168E-01	4.490E-02
scaffold_444	9642	10116	113	5.401E-01	6.884E-01	1.484E-01	4.175E-03
scaffold_447	48938	49324	52	2.607E-01	1.319E-01	-1.288E-01	9.300E-06
scaffold_447	52267	52610	77	3.563E-01	2.281E-01	-1.282E-01	5.310E-05
scaffold_448	9772	10203	98	2.093E-01	3.710E-01	1.617E-01	8.000E-07
scaffold_457	17968	18124	21	3.534E-01	2.207E-01	-1.327E-01	4.848E-02
scaffold_459	953	1146	34	1.681E-01	3.268E-01	1.587E-01	1.361E-03
scaffold_460	12493	12777	36	2.488E-01	3.734E-02	-2.115E-01	3.000E-07
scaffold_465	17284	17767	98	4.324E-01	3.018E-01	-1.307E-01	0.000E+00
scaffold_469	27076	27310	34	1.479E-01	2.710E-01	1.231E-01	4.133E-04
scaffold_474	2114	2537	147	2.647E-01	1.287E-01	-1.359E-01	1.800E-06
scaffold_474	2572	3006	92	3.896E-01	2.493E-01	-1.403E-01	2.000E-07
scaffold_476	5787	5908	15	1.249E-01	2.644E-01	1.396E-01	5.137E-04
scaffold_476	25666	25986	32	5.819E-02	2.084E-01	1.502E-01	1.900E-06
scaffold_490	30453	30762	42	4.020E-01	6.695E-01	2.675E-01	2.000E-07
scaffold_490	34375	34640	37	7.247E-01	8.731E-01	1.484E-01	7.597E-04
scaffold_490	42055	42246	76	5.178E-01	7.309E-01	2.130E-01	6.776E-04
scaffold_490	48612	48978	122	5.637E-01	7.155E-01	1.518E-01	2.604E-04
scaffold_499	35372	35853	71	3.101E-01	1.933E-01	-1.168E-01	2.675E-04
scaffold_500	7747	8141	52	4.007E-01	6.661E-01	2.654E-01	2.402E-03
scaffold_500	9767	10115	64	8.418E-01	7.075E-01	-1.343E-01	3.000E-07
scaffold_500	10650	11137	180	4.188E-01	5.475E-01	1.287E-01	3.700E-02
scaffold_504	44128	44215	26	2.668E-01	1.298E-02	-2.538E-01	7.420E-05
scaffold_506	10252	10376	30	2.755E-01	1.701E-01	-1.054E-01	3.350E-02
scaffold_508	27588	27715	24	4.665E-04	1.207E-01	1.202E-01	9.190E-05
scaffold_510	46496	46937	135	3.189E-01	1.820E-01	-1.369E-01	2.051E-02
scaffold_513	26642	26930	67	2.744E-01	1.739E-01	-1.005E-01	7.295E-04
scaffold_513	26942	27231	49	2.712E-01	5.270E-02	-2.185E-01	0.000E+00
scaffold_513	34588	35067	159	3.364E-01	2.241E-01	-1.123E-01	1.280E-02
scaffold_523	12949	13441	173	1.353E-01	1.937E-02	-1.160E-01	2.000E-07
scaffold_523	38953	39411	118	2.238E-01	3.690E-01	1.452E-01	2.264E-03
scaffold_523	39879	40316	101	2.596E-01	3.728E-01	1.132E-01	1.252E-02
scaffold_525	13679	14170	66	8.486E-01	7.131E-01	-1.355E-01	9.252E-04
scaffold_535	10380	10694	80	2.893E-01	1.626E-01	-1.267E-01	5.738E-04
scaffold_537	866	1283	74	5.146E-01	4.087E-01	-1.059E-01	1.000E-07
scaffold_537	9357	9772	84	3.621E-01	2.601E-01	-1.020E-01	1.911E-02
scaffold_540	7518	7840	31	8.330E-01	6.706E-01	-1.624E-01	8.680E-03
scaffold_540	24369	24787	66	9.308E-01	7.546E-01	-1.763E-01	2.842E-02
scaffold_542	20418	20897	49	6.171E-01	4.850E-01	-1.322E-01	1.267E-03
scaffold_552	10500	10840	61	1.714E-01	5.957E-02	-1.118E-01	4.180E-02
scaffold_552	39581	39923	126	1.635E-01	8.115E-03	-1.554E-01	1.335E-02

scaffold_552	39927	40267	116	1.133E-01	4.414E-03	-1.089E-01	2.597E-02
scaffold_559	25497	25693	57	3.522E-01	2.418E-01	-1.104E-01	1.343E-03
scaffold_561	7915	8006	20	7.515E-02	1.870E-01	1.119E-01	3.723E-03
scaffold_561	16298	16498	29	3.991E-01	2.621E-01	-1.371E-01	5.029E-03
scaffold_564	10498	10670	25	1.927E-01	8.872E-02	-1.039E-01	1.037E-03
scaffold_571	5235	5388	29	3.484E-01	2.173E-01	-1.311E-01	5.855E-03
scaffold_573	5184	5574	63	5.916E-01	4.227E-01	-1.689E-01	1.908E-03
scaffold_573	33348	33739	58	4.368E-01	2.501E-01	-1.868E-01	0.000E+00
scaffold_576	22173	22617	77	4.684E-01	3.197E-01	-1.487E-01	6.625E-04
scaffold_588	5773	6220	128	1.500E-02	1.576E-01	1.426E-01	0.000E+00
scaffold_589	27571	27883	38	1.121E-01	2.362E-01	1.241E-01	1.900E-06
scaffold_591	10495	10925	118	4.488E-01	3.226E-01	-1.262E-01	7.000E-07
scaffold_596	34397	34517	17	2.715E-01	4.098E-01	1.383E-01	2.729E-02
scaffold_607	14993	15436	44	2.882E-01	1.694E-01	-1.187E-01	1.971E-04
scaffold_607	28662	28892	44	3.096E-01	1.449E-01	-1.647E-01	3.062E-03
scaffold_611	4133	4557	112	3.255E-01	2.242E-01	-1.013E-01	1.619E-04
scaffold_611	32164	32300	21	1.922E-01	3.311E-02	-1.591E-01	1.074E-03
scaffold_612	24298	24604	47	2.386E-01	1.124E-01	-1.261E-01	1.100E-05
scaffold_615	31305	31603	34	9.838E-02	2.020E-01	1.036E-01	7.831E-03
scaffold_622	9557	9976	176	1.387E-01	3.157E-02	-1.071E-01	3.650E-03
scaffold_622	9980	10401	234	2.803E-01	1.669E-01	-1.135E-01	1.078E-02
scaffold_622	20178	20490	73	4.346E-01	2.831E-01	-1.515E-01	1.420E-05
scaffold_622	29777	29897	20	2.492E-01	8.970E-02	-1.595E-01	1.196E-03
scaffold_626	8817	9008	35	1.888E-01	3.586E-01	1.697E-01	8.132E-03
scaffold_628	6723	7059	62	5.175E-01	4.019E-01	-1.156E-01	1.823E-02
scaffold_63	258882	259336	68	3.622E-01	2.198E-01	-1.423E-01	9.713E-04
scaffold_633	2521	2906	46	4.136E-01	2.889E-01	-1.247E-01	0.000E+00
scaffold_633	5163	5398	24	5.585E-01	2.988E-01	-2.597E-01	5.580E-05
scaffold_633	15598	16089	75	2.791E-01	1.677E-01	-1.114E-01	0.000E+00
scaffold_638	16815	17027	54	6.617E-01	5.571E-01	-1.046E-01	4.069E-02
scaffold_638	19661	19936	105	6.931E-02	1.909E-01	1.216E-01	3.937E-02
scaffold_639	21177	21630	90	8.671E-02	1.927E-01	1.060E-01	3.819E-04
scaffold_642	25148	25455	22	3.624E-01	2.519E-01	-1.105E-01	1.267E-03
scaffold_645	29466	29942	102	2.518E-01	1.006E-01	-1.512E-01	7.017E-04
scaffold_647	5417	5563	27	1.302E-01	3.844E-04	-1.298E-01	0.000E+00
scaffold_651	25727	26025	82	3.193E-01	4.555E-01	1.362E-01	5.778E-03
scaffold_657	11827	12176	102	2.361E-01	3.484E-01	1.123E-01	4.000E-07
scaffold_659	25767	25872	27	6.757E-04	1.015E-01	1.008E-01	0.000E+00
scaffold_660	19464	19606	26	3.464E-01	2.006E-01	-1.458E-01	9.323E-03
scaffold_664	1216	1555	44	3.217E-01	4.753E-01	1.535E-01	4.245E-02
scaffold_672	14222	14404	30	4.265E-01	5.441E-01	1.176E-01	4.641E-03
scaffold_675	9982	10107	14	2.639E-01	1.560E-01	-1.079E-01	3.545E-02
scaffold_684	11717	12028	38	6.174E-01	4.841E-01	-1.333E-01	3.770E-05
scaffold_686	6996	7112	35	4.298E-01	2.158E-01	-2.141E-01	1.800E-06
scaffold_695	21988	22105	22	2.672E-01	1.456E-01	-1.216E-01	5.247E-03
scaffold_706	21777	22229	78	1.315E-01	2.320E-01	1.005E-01	1.273E-03
scaffold_714	21201	21430	50	1.301E-01	3.002E-01	1.701E-01	4.683E-03

scaffold_721	10675	11094	65	2.024E-01	4.338E-02	-1.590E-01	1.500E-06
scaffold_725	16723	16982	40	3.957E-01	2.295E-01	-1.662E-01	7.443E-04
scaffold_739	13911	14393	126	5.375E-01	3.978E-01	-1.397E-01	2.266E-03
scaffold_739	15868	16354	224	5.191E-01	6.361E-01	1.171E-01	3.620E-02
scaffold_739	17832	18309	172	5.269E-01	6.524E-01	1.256E-01	1.592E-02
scaffold_739	19781	20266	137	5.586E-01	4.261E-01	-1.325E-01	3.264E-04
scaffold_750	8176	8363	59	1.480E-01	1.360E-02	-1.344E-01	2.780E-05
scaffold_751	5996	6297	43	8.544E-02	2.342E-01	1.488E-01	1.387E-02
scaffold_757	2203	2485	33	2.908E-01	1.823E-01	-1.084E-01	6.354E-03
scaffold_772	7596	7997	122	1.522E-01	4.196E-02	-1.102E-01	1.127E-02
scaffold_772	8320	8417	16	1.528E-03	3.729E-01	3.714E-01	0.000E+00
scaffold_777	12964	13384	62	1.442E-01	2.201E-02	-1.222E-01	1.630E-02
scaffold_844	160143	160638	156	2.280E-01	1.240E-01	-1.040E-01	4.840E-04
scaffold_844	233560	234053	178	4.449E-01	3.112E-01	-1.337E-01	2.987E-03
scaffold_844	259379	259870	172	1.532E-01	2.667E-01	1.135E-01	1.632E-02
scaffold_99	3696	4183	121	2.607E-01	1.410E-01	-1.197E-01	3.289E-03
scaffold_99	23720	24181	125	3.782E-01	1.864E-01	-1.919E-01	1.216E-04
scaffold_99	32543	33015	115	3.055E-01	6.687E-02	-2.386E-01	0.000E+00
scaffold_99	36372	36827	99	2.926E-01	7.232E-02	-2.203E-01	5.000E-07
scaffold_99	57955	58450	91	1.010E-01	2.426E-01	1.416E-01	1.143E-03
scaffold_99	58463	58948	146	1.783E-01	2.929E-01	1.146E-01	3.618E-02
scaffold_99	77864	77947	24	4.009E-01	2.644E-01	-1.365E-01	1.555E-02
scaffold_99	82604	83042	116	1.834E-01	2.682E-02	-1.566E-01	0.000E+00
scaffold_99	83746	84092	46	3.500E-01	8.822E-02	-2.618E-01	1.000E-07
scaffold_99	87009	87318	51	2.769E-01	1.079E-01	-1.690E-01	3.412E-03
scaffold_99	112571	113059	83	2.029E-01	6.531E-02	-1.376E-01	4.459E-04
scaffold_99	113557	114049	108	1.857E-01	5.478E-02	-1.310E-01	2.300E-06
scaffold_99	114966	115332	49	1.688E-01	5.901E-02	-1.098E-01	2.854E-03
scaffold_99	116095	116568	84	3.371E-03	1.082E-01	1.049E-01	1.380E-05
scaffold_99	140723	141127	85	1.148E-01	1.005E-03	-1.138E-01	3.769E-02
scaffold_99	147326	147737	65	1.081E-01	5.830E-04	-1.075E-01	2.690E-05
scaffold_99	147748	148150	80	2.671E-01	3.510E-04	-2.667E-01	0.000E+00
scaffold_99	151241	151731	137	1.702E-01	6.191E-02	-1.083E-01	1.043E-02
scaffold_99	159324	159793	93	2.849E-01	3.530E-04	-2.846E-01	0.000E+00
scaffold_99	183906	184394	71	1.168E-02	4.510E-01	4.394E-01	0.000E+00
scaffold_99	195068	195548	52	2.541E-01	1.333E-01	-1.207E-01	2.875E-03
scaffold_99	197826	198308	151	1.745E-01	5.702E-02	-1.175E-01	1.283E-04
scaffold_99	204104	204588	148	3.767E-01	3.115E-02	-3.456E-01	5.900E-05
scaffold_99	212990	213479	82	1.738E-01	3.986E-02	-1.340E-01	1.300E-05
scaffold_99	216448	216940	87	1.697E-01	5.789E-02	-1.119E-01	2.100E-06
scaffold_99	236071	236432	52	2.931E-01	1.622E-01	-1.308E-01	1.278E-02
scaffold_99	236448	236799	59	1.719E-01	2.316E-02	-1.488E-01	1.000E-07
scaffold_99	236810	237179	48	2.686E-01	1.039E-03	-2.676E-01	0.000E+00
scaffold_99	239861	240318	70	2.186E-01	3.656E-01	1.469E-01	1.071E-02
scaffold_99	270211	270701	85	6.223E-02	1.963E-01	1.341E-01	1.167E-03
scaffold_99	298841	299319	127	2.728E-01	3.931E-01	1.203E-01	1.150E-02

scaffold_99	307093	307535	104	1.703E-01	3.327E-01	1.624E-01	5.320E-05
