# Supplementary material for: Integrative genomics reveals paths to sex dimorphism in Salix purpurea L
Source: Hortic Res. 2021 Aug 1;8:170. doi: 10.1038/s41438-021-00606-y (PMC8325687; doi:10.1038/s41438-021-00606-y)
Supplement: Supplementary file 6 — Supplementary Table S7 [file 41438_2021_606_MOESM6_ESM.pdf]

SUPPLEMENTARY TABLE S7

Chr	DMR start	DMR end	CpG sites	Male mean proportion Methylated	Female mean proportion Methylated	Female:Male difference	FDR	Gene ID	Gene Start	Gene End	Sense	Gene log2FoldChange M:F	FDR	Gene Description	WGCNA module	SDR eQTL?
Chr01	547253	547747	192	0.160	0.044	-0.116	4.971E-02	Sapur.001G009300	548191	551999	+	-0.019	NA	Plastid-lipid associated protein PAP / fibrillin family protein	brown	NA
Chr01	628596	629090	131	0.346	0.183	-0.164	1.174E-02	Sapur.001G009800	627695	628278	-	0.550	2.326E-02	Ubiquitin-conjugating enzyme family protein	grey	NA
Chr01	2882193	2882682	163	0.307	0.197	-0.110	3.610E-03	Sapur.001G042800	2883343	2887756	+	-1.158	3.000E-39	bacterial outer membrane-like protein, putative	purple	SDR eQTL
Chr01	4170359	4170846	137	0.443	0.278	-0.165	1.532E-03	Sapur.001G061400	4171633	4173587	+	0.108	NA	GTP binding Elongation factor Tu family protein	pink	NA
Chr01	7936275	7936697	82	0.533	0.403	-0.130	7.392E-03	Sapur.001G109200	7937582	7946939	+	0.825	4.410E-102	ENHANCED DISEASE RESISTANCE 2	cyan	NA
Chr01	9557519	9558002	197	0.281	0.166	-0.115	2.816E-02	Sapur.001G124300	9557260	9557415	-	0.445	1.026E-01		grey	NA
Chr01	12341360	12341857	143	0.292	0.176	-0.117	4.539E-04	Sapur.001G147900	12339560	12340417	-	1.267	7.210E-11	myb domain protein 66	grey	NA
Chr01	12408809	12409282	156	0.370	0.251	-0.119	1.001E-04	Sapur.001G148700	12409493	12411326	+	1.488	1.060E-19	Protein kinase superfamily protein	grey	NA
Chr01	13657779	13658272	162	0.456	0.272	-0.184	9.450E-05	Sapur.001G166800	13659119	13660912	+	1.576	3.230E-99	hydroxyproline-rich glycoprotein family protein	cyan	NA
Chr01	14539769	14540260	141	0.192	0.053	-0.140	3.040E-03	Sapur.001G179100	14541090	14543644	+	-2.317	4.730E-18	related to ABI3/VP1 1	cyan	NA
Chr01	14569693	14570186	144	0.340	0.221	-0.118	2.276E-04	Sapur.001G179500	14564947	14569606	-	0.410	1.690E-27	zinc finger (C2H2 type) family protein	cyan	NA
Chr02	107586	108071	137	0.359	0.246	-0.113	7.894E-03	Sapur.002G001200	105957	106846	-	1.118	1.580E-14	mRNA, 952 bp sequence protein, putative	cyan	SDR eQTL
Chr02	5030553	5031043	95	0.166	0.421	0.254	8.541E-03	Sapur.002G066800	5031516	5033478	+	0.453	2.440E-23	hypothetical protein	purple	NA
Chr02	5075260	5075758	113	0.522	0.398	-0.124	7.370E-05	Sapur.002G067200	5057562	5075101	-	-0.421	2.900E-01	ARM repeat superfamily protein	grey	NA
Chr02	6809825	6810310	152	0.413	0.301	-0.112	4.277E-03	Sapur.002G087400	6810503	6813082	+	0.367	8.508E-01	Phosphoglycerate mutase family protein	brown	SDR eQTL
Chr02	8108002	8108488	141	0.338	0.232	-0.107	1.060E-05	Sapur.002G103400	8106678	8107868	-	0.491	2.160E-05	BRI1 kinase inhibitor 1	cyan	NA
Chr02	8783327	8783463	32	0.340	0.161	-0.180	2.521E-02	Sapur.002G112400	8780897	8783126	-	-0.350	3.810E-26	mago nashi family protein	black	NA
Chr02	13383471	13383967	207	0.398	0.292	-0.105	8.947E-03	Sapur.002G154400	13379348	13382739	-	-0.609	2.470E-25	WEE1 kinase homolog	purple	NA
Chr03	337382	337817	119	0.198	0.094	-0.104	3.564E-02	Sapur.003G004700	338702	341994	+	0.875	2.520E-103	NIFU-like protein 2	purple	NA
Chr03	430350	430803	79	0.379	0.240	-0.139	3.013E-02	Sapur.003G005900	429064	429417	-	0.234	9.389E-01	hypothetical protein	grey	NA
Chr03	999294	999706	65	0.286	0.054	-0.232	0.000E+00	Sapur.003G011600	1000697	1002147	+	-1.414	2.280E-64	light harvesting chlorophyll A-B-binding protein	cyan	NA
Chr03	1014842	1015338	138	0.196	0.053	-0.143	2.948E-02	Sapur.003G012000	1016295	1022521	+	-0.304	1.910E-05	patatin-like phospholipase	grey	NA
Chr03	6363018	6363507	196	0.256	0.137	-0.118	1.072E-04	Sapur.003G061300	6364284	6365938	+	0.322	2.680E-21	inositol-tetrakisphosphate 1-kinase	cyan	NA
Chr03	7617502	7617994	95	0.305	0.152	-0.153	2.504E-03	Sapur.003G078100	7618537	7623202	+	-0.057	NA	thylakoid lumenal protein	brown	NA
Chr04	6955751	6956065	50	0.265	0.001	-0.264	0.000E+00	Sapur.004G084900	6948037	6954931	-	0.509	2.280E-13	HEAT repeat-containing protein	cyan	NA
Chr04	7999448	7999831	60	0.047	0.177	0.130	1.140E-02	Sapur.004G092100	7996313	7999379	-	1.083	3.245E-03		grey	NA
Chr05	1089761	1090234	130	0.400	0.278	-0.122	9.539E-03	Sapur.005G012100	1090912	1092377	+	0.857	1.280E-09	S-locus lectin protein kinase family protein	grey	SDR eQTL
Chr05	1550215	1550707	127	0.513	0.405	-0.108	1.645E-04	Sapur.005G020400	1542320	1549905	-	0.840	3.190E-34	acyl-CoA binding protein 4	cyan	NA
Chr05	1781563	1782052	155	0.257	0.156	-0.100	0.000E+00	Sapur.005G023800	1778681	1780657	-	1.806	9.150E-13	P-loop containing nucleoside triphosphate hydrolases superfamily protein	cyan	NA
Chr05	2767181	2767657	77	0.245	0.132	-0.113	8.729E-03	Sapur.005G037700	2768649	2779439	+	0.163	1.480E-05	exoribonuclease 4	black	NA
Chr05	3002225	3002692	91	0.280	0.180	-0.100	9.400E-05	Sapur.005G041500	3002739	3006994	+	-1.043	3.160E-90	twin arginine-targeting protein translocase	cyan	NA
Chr05	5910848	5911324	151	0.531	0.415	-0.116	3.509E-03	Sapur.005G080100	5909525	5910088	-	-6.633	1.380E-166	Disease resistance-responsive (dirigent-like protein) family protein	purple	NA
Chr05	12500441	12500916	129	0.212	0.352	0.140	8.227E-03	Sapur.005G135600	12501185	12501460	+	0.129	NA		grey	NA
Chr05	15634244	15634732	176	0.358	0.219	-0.139	1.000E-07	Sapur.005G171500	15626932	15633291	-	1.505	1.540E-34	dwarf 27	cyan	NA
Chr06	8159081	8159576	160	0.351	0.248	-0.103	4.517E-03	Sapur.006G106300	8159842	8160561	+	2.867	2.600E-97	FASCICLIN-like arabinogalactan-protein 11	salmon	NA
Chr06	9488512	9489006	157	0.398	0.287	-0.111	0.000E+00	Sapur.006G119800	9487095	9487874	-	-0.568	1.128E-02		grey	NA
Chr06	9488012	9488504	180	0.410	0.281	-0.129	0.000E+00	Sapur.006G119900	9488739	9492127	+	0.051	8.322E-01		grey	NA
Chr06	11077291	11077786	106	0.588	0.424	-0.163	1.700E-06	Sapur.006G133000	11077905	11081517	+	NA	NA	nudix hydrolase homolog 24	grey	NA
Chr06	18547182	18547663	212	0.357	0.226	-0.131	5.955E-04	Sapur.006G210900	18543333	18546331	-	-0.100	8.771E-01	clathrin assembly protein, putative	brown	NA
Chr07	5352110	5352435	80	0.382	0.279	-0.103	1.862E-03	Sapur.007G056700	5350420	5351146	-	-1.455	7.790E-14	basic chitinase	grey	NA
Chr07	6660271	6660766	79	0.486	0.375	-0.111	3.755E-03	Sapur.007G064000	6660973	6663094	+	-0.293	6.114E-02	P-loop containing nucleoside triphosphate hydrolases superfamily protein	grey	NA
Chr07	10896398	10896888	112	0.395	0.295	-0.101	1.012E-03	Sapur.007G103600	10893931	10896233	-	0.846	8.020E-26	nicotinamidase 1	purple	SDR eQTL
Chr07	10936405	10936830	119	0.409	0.261	-0.148	4.218E-02	Sapur.007G104200	10933622	10935450	-	0.790	4.307E-01	Exostosin family protein	grey	NA
Chr07	11702528	11703023	136	0.244	0.136	-0.109	3.266E-04	Sapur.007G116100	11700648	11702152	-	0.224	8.767E-01	F-box and associated interaction domains-containing protein	grey	NA
Chr07	12208242	12208732	133	0.332	0.184	-0.148	5.246E-03	Sapur.007G123900	12203147	12207259	-	-0.885	3.495E-02	squamosa promoter binding protein-like 4	grey	NA
Chr08	1714088	1714566	173	0.320	0.195	-0.125	6.427E-03	Sapur.008G028600	1715159	1721475	+	4.965	3.380E-258	Pleckstrin homology (PH) and lipid-binding START domains-containing protein	cyan	NA
Chr08	1768051	1768546	136	0.337	0.232	-0.106	2.300E-05	Sapur.008G029100	1764803	1767262	-	0.130	8.985E-03	Acyl-CoA N-acyltransferases (NAT) superfamily protein	grey	NA
Chr08	2364143	2364633	156	0.262	0.159	-0.103	3.016E-02	Sapur.008G037900	2362508	2363604	-	0.162	8.841E-01	Disease resistance-responsive (dirigent-like protein) family protein	grey	NA
Chr08	3490602	3491093	178	0.196	0.092	-0.104	1.898E-02	Sapur.008G054100	3491471	3496002	+	2.556	4.270E-265	phospholipase C 2	cyan	NA
Chr08	4014969	4015466	179	0.225	0.093	-0.132	4.740E-03	Sapur.008G061300	4016461	4018703	+	-0.020	7.478E-01	Far-red impaired responsive (FAR1) family protein	grey	NA
Chr08	4655997	4656489	144	0.467	0.330	-0.136	3.735E-04	Sapur.008G070800	4652703	4655346	-	0.463	5.330E-12	fucosyltransferase 1	cyan	NA
Chr08	4697495	4697976	113	0.420	0.313	-0.107	2.605E-04	Sapur.008G071300	4693117	4696771	-	-0.685	3.824E-01	EXS (ERD1/XPR1/SYG1) family protein	darkred	NA
Chr08	8385081	8385510	114	0.377	0.271	-0.106	2.066E-02	Sapur.008G121700	8386390	8388278	+	8.209	5.570E-177	Major facilitator superfamily protein	cyan	NA
Chr08	8745017	8745507	164	0.268	0.136	-0.132	3.900E-06	Sapur.008G126600	8745894	8752312	+	-0.914	2.100E-84	Tetratricopeptide repeat (TPR)-like superfamily protein	purple	NA
Chr09	1752337	1752769	120	0.174	0.276	0.102	6.772E-04	Sapur.009G014400	1753409	1761862	+	-0.005	9.729E-01	villin 4	grey	NA
Chr09	3182963	3183451	152	0.329	0.206	-0.122	1.559E-02	Sapur.009G029000	3184189	3187242	+	0.012	9.348E-01	UDP-glucosyl transferase 76E2	grey	NA
Chr09	3190881	3191371	146	0.351	0.234	-0.117	4.260E-05	Sapur.009G029100	3190200	3190673	-	-0.281	7.498E-04	HSP20-like chaperones superfamily protein	grey	NA
Chr09	9450450	9450677	22	0.158	0.054	-0.104	1.601E-02	Sapur.009G121400	9447194	9449775	-	4.502	2.800E-88	wall-associated kinase 2	cyan	NA
Chr09	9471218	9471561	94	0.388	0.259	-0.129	8.244E-03	Sapur.009G121600	9456864	9470490	-	-0.521	7.020E-06	Structural maintenance of chromosomes (SMC) family protein	grey	NA
Chr10	404428	404916	144	0.253	0.135	-0.118	1.500E-06	Sapur.010G006900	404984	405212	+	1.587	5.340E-07		grey	NA
Chr10	3001935	3002422	159	0.415	0.310	-0.105	4.954E-04	Sapur.010G043200	3002897	3008735	+	0.129	NA	no pollen germination related 2	brown	NA
Chr10	4247523	4247989	117	0.407	0.306	-0.102	1.644E-03	Sapur.010G058400	4248615	4258867	+	-0.248	2.810E-05	Homeotic gene regulator	grey	NA
Chr10	7733277	7733767	189	0.327	0.218	-0.109	6.410E-05	Sapur.010G087000	7734620	7735154	+	13.015	0.000E+00	Late embryogenesis abundant protein (LEA) family protein	cyan	NA
Chr10	8796343	8796834	173	0.429	0.306	-0.122	2.547E-04	Sapur.010G097400	8797798	8805783	+	0.379	1.420E-22	Protein kinase superfamily protein	cyan	NA
Chr10	10490845	10491332	113	0.396	0.282	-0.115	2.095E-02	Sapur.010G118900	10480855	10489857	-	-2.063	2.820E-11	ARM repeat superfamily protein	brown	SDR eQTL
Chr10	11096083	11096553	63	0.446	0.332	-0.114	3.810E-02	Sapur.010G127400	11094300	11095372	-	0.421	3.327E-01	Glucose-1-phosphate adenylyltransferase family protein	grey	NA
Chr11	1397106	1397593	122	0.228	0.339	0.110	2.114E-04	Sapur.011G017700	1395520	1396892	-	1.503	9.000E-05	S-locus lectin protein kinase family protein	grey	NA
Chr11	1604627	1605123	197	0.365	0.225	-0.140	1.516E-04	Sapur.011G020100	1602491	1604311	-	0.500	8.120E-31		cyan	NA
Chr11	3394119	3394613	74	0.278	0.166	-0.112	7.915E-04	Sapur.011G040000	3382163	3393652	-	0.108	NA	homeobox-1	pink	NA
Chr11	3477525	3478019	214	0.257	0.148	-0.110	4.399E-02	Sapur.011G041000	3473059	3476677	-	1.564	1.180E-34	Seven transmembrane MLO family protein	purple	NA
Chr11	9253108	9253589	85	0.162	0.049	-0.113	2.464E-02	Sapur.011G077100	9253828	9256224	+	0.431	1.106E-03	nine-cis-epoxycarotenoid dioxygenase 3	pink	NA
Chr11	13268973	13269434	126	0.362	0.256	-0.105	2.000E-06	Sapur.011G117500	13267080	13268681	-	4.992	1.950E-41	FAD-binding Berberine family protein	greenyellow	NA
Chr12	17987	18466	80	0.360	0.466	0.105	3.585E-02	Sapur.012G000300	12833	17406	-	0.054	NA	Autophagy-related protein 13	brown	NA
Chr12	737566	738064	202	0.399	0.264	-0.136	4.183E-03	Sapur.012G009200	735398	737450	-	-0.019	NA	Rhodanese/Cell cycle control phosphatase superfamily protein	brown	NA
Chr12	1636641	1637138	164	0.383	0.191	-0.192	1.346E-02	Sapur.012G020000	1637265	1638226	+	-0.380	1.584E-03	sulfotransferase 16	grey	NA
Chr12	7539905	7540383	179	0.244	0.143	-0.102	2.022E-04	Sapur.012G065600	7541138	7544641	+	-0.792	1.880E-19	Leucine-rich repeat transmembrane protein kinase	purple	NA
Chr12	7672617	7673101	168	0.278	0.409	0.131	7.818E-03	Sapur.012G067300	7673213	7675437	+	-1.387	1.290E-20	Leucine-rich repeat protein kinase family protein	purple	NA
Chr12	9705465	9705943	127	0.217	0.108	-0.109	2.100E-03	Sapur.012G093300	9702943	9705025	-	-0.977	7.690E-23	patatin-like protein 6	purple	NA
Chr12	9868284	9868755	88	0.319	0.200	-0.119	1.126E-04	Sapur.012G095100	9869496	9870705	+	6.404	5.650E-107		cyan	SDR eQTL

Chr13	2658529	2658908	71	0.213	0.086	-0.127	1.837E-02	Sapur.013G037900	2656056	2658511	-	0.535	1.196E-02	GDP-D-mannose 3',5'-epimerase	grey	NA
Chr13	2658529	2658908	71	0.213	0.086	-0.127	1.837E-02	Sapur.013G038000	2659420	2662383	+	0.329	9.608E-02	GDP-D-mannose 3',5'-epimerase	grey	NA
Chr13	2734628	2735122	122	0.354	0.235	-0.119	6.000E-07	Sapur.013G038600	2722690	2733720	-	0.034	6.750E-01	RNA polymerase II transcription mediators	grey	NA
Chr13	10578889	10579094	48	0.265	0.376	0.111	3.968E-03	Sapur.013G095300	10576824	10577933	-	0.106	NA		grey	NA
Chr13	12534082	12534562	187	0.144	0.007	-0.138	1.592E-03	Sapur.013G114600	12535387	12543153	+	-0.355	1.870E-01	Sas10/U3 ribonucleoprotein (Utp) family protein	grey	NA
Chr13	12776276	12776768	197	0.260	0.132	-0.128	1.009E-02	Sapur.013G117400	12773881	12775449	-	2.443	1.050E-32	Protein of unknown function (DUF506)	salmon	NA
Chr13	13855378	13855870	117	0.282	0.151	-0.130	1.555E-03	Sapur.013G132300	13854231	13854808	-	1.297	7.740E-27	Wound-responsive family protein	purple	NA
Chr14	1463352	1463719	120	0.388	0.283	-0.105	3.625E-04	Sapur.014G019600	1460788	1463136	-	-0.583	3.910E-07	Protein of unknown function (DUF594)	grey	NA
Chr14	1920961	1921441	74	0.143	0.000	-0.143	5.108E-03	Sapur.014G025900	1917939	1920195	-	-0.459	4.780E-25	Serine protease inhibitor (SERPIN) family protein	cyan	NA
Chr14	3102628	3103114	149	0.254	0.145	-0.109	4.708E-02	Sapur.014G042700	3102028	3102348	-	3.567	8.460E-20		grey	NA
Chr14	5954816	5955312	162	0.288	0.175	-0.113	2.900E-06	Sapur.014G081500	5947478	5953840	-	0.031	7.188E-01	CDPK-related kinase 3	brown	NA
Chr14	7531593	7532088	138	0.319	0.199	-0.120	1.678E-03	Sapur.014G104400	7522046	7530852	-	2.676	8.930E-100	multidrug resistance-associated protein 4	cyan	NA
Chr14	7842065	7842527	136	0.285	0.184	-0.102	3.041E-04	Sapur.014G108800	7842628	7843776	+	-0.256	1.826E-03	hypothetical protein	grey	NA
Chr15W	2848244	2848718	171	0.355	0.206	-0.150	3.020E-05	Sapur.15WG030800	2848776	2851864	+	-0.618	9.242E-04		grey	NA
Chr15W	2988507	2989003	167	0.033	0.149	0.117	0.000E+00	Sapur.15WG032300	2989168	2993272	+	-3.075	6.130E-27	B-S glucosidase 44	cyan	NA
Chr15W	3109305	3109804	149	0.062	0.186	0.125	1.260E-02	Sapur.15WG033000	3107222	3109028	-	0.982	2.530E-09	glutathione S-transferase tau 4	grey	SDR eQTL
Chr15W	3109305	3109804	149	0.062	0.186	0.125	1.260E-02	Sapur.15WG033100	3110744	3113845	+	0.056	NA	NAD(P)-linked oxidoreductase superfamily protein	brown	NA
Chr15W	3463507	3463942	157	0.349	0.225	-0.124	8.234E-03	Sapur.15WG037100	3459178	3462993	-	-8.430	4.780E-283	porphyromonas-type peptidyl-arginine deiminase family protein	purple	SDR eQTL
Chr15W	3573351	3573776	143	0.273	0.153	-0.120	4.114E-02	Sapur.15WG038700	3571733	3573302	-	-4.355	0.000E+00	PLATZ transcription factor family protein	purple	NA
Chr15W	3678478	3678952	114	0.119	0.241	0.122	1.492E-02	Sapur.15WG040800	3679031	3684284	+	0.638	8.500E-18	zeta-carotene desaturase	purple	SDR eQTL
Chr15W	3917912	3918357	90	0.363	0.159	-0.205	1.027E-03	Sapur.15WG042200	3916487	3917001	-	-4.849	7.600E-163	Magnesium transporter CorA-like family protein	purple	SDR eQTL
Chr15W	4162358	4162849	153	0.257	0.130	-0.127	1.961E-02	Sapur.15WG044500	4163012	4166090	+	-6.777	0.000E+00	Protein kinase superfamily protein	purple	NA
Chr15W	4383717	4384195	153	0.172	0.056	-0.116	2.025E-02	Sapur.15WG046200	4384552	4385346	+	1.582	2.200E-06	P-loop containing nucleoside triphosphate hydrolases superfamily protein	cyan	SDR eQTL
Chr15W	4384200	4384682	154	0.355	0.079	-0.276	3.000E-07	Sapur.15WG046300	4385049	4388540	+	-0.371	1.076E-03	P-loop nucleoside triphosphate hydrolases superfamily protein with CH (Calponin Homology) domain	grey	SDR eQTL
Chr15W	4464049	4464526	141	0.440	0.291	-0.149	1.462E-02	Sapur.15WG046800	4459999	4463332	-	-5.814	1.110E-143	WUSCHEL related homeobox 1	purple	SDR eQTL
Chr15W	4593598	4594086	143	0.296	0.470	0.174	1.722E-02	Sapur.15WG048000	4586354	4592787	-	-0.696	4.668E-01	Glutathione S-transferase family protein	grey	NA
Chr15W	4593598	4594086	143	0.296	0.470	0.174	1.722E-02	Sapur.15WG048100	4594474	4599091	+	-4.385	5.300E-185	zinc finger (Ran-binding) family protein	purple	NA
Chr15W	5552042	5552532	110	0.292	0.130	-0.162	3.761E-02	Sapur.15WG053300	5552700	5555638	+	-0.084	8.759E-01	Leucine-rich repeat protein kinase family protein	grey	SDR eQTL
Chr15W	5657629	5657892	44	0.263	0.384	0.121	6.439E-04	Sapur.15WG053900	5656898	5657300	-	1.869	1.760E-08		cyan	SDR eQTL
Chr15W	5859885	5860360	108	0.195	0.299	0.104	7.562E-04	Sapur.15WG055000	5861125	5862011	+	4.051	1.150E-100	Ribosomal protein L19e family protein	cyan	NA
Chr15W	6330476	6330954	144	0.158	0.369	0.212	2.000E-07	Sapur.15WG058000	6331514	6336310	+	-3.403	0.000E+00	aluminum-activated malate transporter 9	purple	NA
Chr15W	6847781	6848068	103	0.413	0.572	0.159	5.399E-03	Sapur.15WG060700	6843872	6846941	-	-0.917	6.780E-08	expansin A4	grey	NA
Chr15W	7051791	7052284	137	0.201	0.057	-0.144	1.287E-03	Sapur.15WG062500	7046908	7051032	-	-1.367	2.770E-15	ubiquiting-conjugating enzyme 2	cyan	NA
Chr15W	7090499	7090991	150	0.135	0.359	0.223	2.290E-02	Sapur.15WG062900	7091838	7098258	+	-3.462	9.840E-135	targeting protein for XKLP2	purple	NA
Chr15W	7207495	7207988	143	0.066	0.190	0.124	0.000E+00	Sapur.15WG063700	7208039	7211446	+	-3.904	5.290E-53	Vacuolar protein sorting-associated protein 26	purple	SDR eQTL
Chr15W	7877321	7877785	199	0.324	0.156	-0.168	2.648E-02	Sapur.15WG067500	7878213	7885025	+	-0.350	1.160E-07	poly(A) polymerase 1	grey	NA
Chr15W	7963142	7963616	139	0.147	0.252	0.105	1.000E-07	Sapur.15WG067900	7964388	7966791	+	-5.443	3.360E-137	Pentatricopeptide repeat (PPR) superfamily protein	purple	SDR eQTL
Chr15W	8217897	8218380	173	0.085	0.267	0.183	1.520E-04	Sapur.15WG069400	8218553	8219163	+	-0.221	8.366E-01	hypothetical protein	grey	SDR eQTL
Chr15W	8300615	8301083	154	0.210	0.369	0.159	5.740E-05	Sapur.15WG069600	8301968	8305563	+	-1.386	2.750E-87	asterix protein	cyan	NA
Chr15W	8607826	8608270	50	0.135	0.029	-0.105	2.252E-02	Sapur.15WG072500	8605739	8607445	-	-0.781	8.880E-58	glycine-rich RNA-binding protein 3	black	SDR eQTL
Chr15W	8772506	8772988	134	0.429	0.320	-0.109	0.000E+00	Sapur.15WG073300	8770901	8771857	-	0.814	3.297E-02		grey	NA
Chr15W	8849100	8849593	126	0.124	0.008	-0.117	3.439E-03	Sapur.15WG074100	8847817	8848101	-	-0.507	6.701E-01	P-loop containing nucleoside triphosphate hydrolases superfamily protein	grey	SDR eQTL
Chr15W	8874365	8874835	117	0.093	0.231	0.138	1.755E-04	Sapur.15WG074400	8875049	8877043	+	-7.937	2.430E-240	Argonaute family protein	purple	SDR eQTL
Chr15W	8914105	8914592	166	0.109	0.221	0.111	1.234E-04	Sapur.15WG074600	8893246	8913533	-	-0.411	5.043E-02	Protein of unknown function (DUF789)	grey	NA
Chr15W	8977900	8978365	100	0.367	0.512	0.144	1.000E-07	Sapur.15WG075000	8979041	8981012	+	-1.512	9.240E-08		grey	NA
Chr15W	10465791	10466134	37	0.367	0.201	-0.166	1.833E-03	Sapur.15WG088800	10462497	10465609	-	-0.938	7.350E-25	formin 8	cyan	SDR eQTL
Chr15W	12351429	12351683	31	0.276	0.102	-0.174	2.300E-06	Sapur.15WG108500	12352605	12358885	+	0.206	4.440E-17	26S proteasome, regulatory subunit Rpn7;Proteasome component (PCI) domain	cyan	NA
Chr15Z	3587883	3588364	110	0.385	0.245	-0.140	3.816E-04	Sapur.15ZG038600	3588672	3590149	+	3.847	2.370E-41	NAD(P)-linked oxidoreductase superfamily protein	purple	SDR eQTL
Chr15Z	3669799	3670224	99	0.402	0.236	-0.166	1.284E-04	Sapur.15ZG039500	3667103	3668874	-	1.152	1.110E-05	nodulin MtN21 /EamA-like transporter family protein	grey	SDR eQTL
Chr15Z	3724407	3724895	209	0.391	0.262	-0.130	2.443E-04	Sapur.15ZG039700	3725724	3729473	+	0.659	2.450E-50	asterix protein	purple	SDR eQTL
Chr15Z	3919721	3920050	40	0.073	0.207	0.135	2.420E-05	Sapur.15ZG041500	3920111	3937140	+	0.623	1.001E-04	porphyromonas-type peptidyl-arginine deiminase family protein	grey	SDR eQTL
Chr15Z	5267226	5267612	63	0.249	0.123	-0.126	7.100E-06	Sapur.15ZG051100	5268208	5269980	+	0.604	6.049E-02	nodulin MtN21 /EamA-like transporter family protein	grey	SDR eQTL
Chr16	1724329	1724781	95	0.488	0.640	0.152	1.162E-03	Sapur.016G017200	1722381	1723722	-	2.023	3.710E-107	UDP-glucosyl transferase 85A3	cyan	NA
Chr16	2833257	2833733	120	0.445	0.316	-0.129	6.808E-03	Sapur.016G029900	2829393	2832411	-	0.104	NA	Cyclin family protein	grey	NA
Chr16	7576403	7576834	66	0.326	0.226	-0.101	7.691E-04	Sapur.016G074800	7577097	7580148	+	-0.584	5.590E-15	phosphoribosyl pyrophosphate (PRPP) synthase 3	grey	SDR eQTL
Chr16	11859822	11860311	180	0.367	0.258	-0.109	7.105E-03	Sapur.016G101300	11860382	11860684	+	-0.140	9.449E-01	Ribosomal protein S5 domain 2-like superfamily protein	grey	NA
Chr16	20784225	20784716	149	0.306	0.185	-0.121	5.200E-06	Sapur.016G198300	20785171	20788070	+	1.499	4.720E-36	B-box type zinc finger protein with CCT domain	salmon	NA
Chr16	20945425	20945922	122	0.371	0.269	-0.102	1.055E-02	Sapur.016G200200	20941847	20945364	-	-0.470	7.041E-02	S-locus lectin protein kinase family protein	grey	NA
Chr16	22087945	22088422	83	0.280	0.117	-0.163	1.353E-03	Sapur.016G213200	22086709	22087050	-	1.356	1.770E-19	high mobility group B3	cyan	NA
Chr16	24441698	24442196	164	0.381	0.264	-0.117	4.924E-03	Sapur.016G235600	24442877	24445990	+	8.416	3.770E-169	oligopeptide transporter 5	cyan	NA
Chr16	25736811	25737003	9	0.185	0.013	-0.172	1.500E-05	Sapur.016G247500	25735564	25735851	-	2.733	1.570E-12		grey	NA
Chr16	27549844	27550342	130	0.244	0.071	-0.172	4.776E-03	Sapur.016G264500	27547794	27549051	-	-1.709	1.350E-09	BED finger NBS-LRR resistance protein, putative	grey	NA
Chr16	29727878	29728240	66	0.139	0.256	0.117	1.780E-02	Sapur.016G283000	29725722	29727375	-	0.071	NA	receptor like protein 27	brown	NA
Chr16	29777282	29777611	67	0.152	0.045	-0.106	2.278E-02	Sapur.016G283800	29776681	29777069	-	-2.542	4.080E-10	Histone superfamily protein	grey	NA
Chr16	31759391	31759695	52	0.013	0.172	0.159	1.000E-07	Sapur.016G303300	31760242	31760722	+	-0.169	NA		brown	NA
Chr16	32159968	32160087	47	0.769	0.482	-0.287	6.880E-05	Sapur.016G308500	32156284	32159189	-	0.750	1.400E-11	Calcium-binding EF-hand family protein	grey	NA
Chr17	1046694	1046818	32	0.530	0.260	-0.271	2.534E-03	Sapur.017G013800	1047399	1048139	+	-1.386	4.775E-03		grey	SDR eQTL
Chr17	1466483	1466965	146	0.305	0.158	-0.147	1.247E-04	Sapur.017G016800	1464517	1466213	-	0.344	1.770E-06	RHOMBOID-like protein 13	grey	SDR eQTL
Chr17	1796503	1796992	97	0.356	0.256	-0.100	9.676E-03	Sapur.017G021300	1793143	1795769	-	1.527	5.210E-10	Protein kinase superfamily protein	grey	NA
Chr17	1800172	1800524	85	0.364	0.524	0.160	1.729E-02	Sapur.017G021400	1801435	1803503	+	0.328	2.674E-01	suppressor of npr1-1 constitutive 4	grey	NA
Chr17	2318784	2319013	77	0.227	0.083	-0.144	1.194E-03	Sapur.017G025500	2319456	2321333	+	-0.516	2.437E-03	LRR and NB-ARC domains-containing disease resistance protein	grey	NA
Chr17	11917756	11918251	164	0.387	0.271	-0.116	1.800E-05	Sapur.017G123200	11916127	11917230	-	0.194	2.308E-01	NB-ARC domain-containing disease resistance protein	pink	NA
Chr17	11916756	11917254	159	0.341	0.240	-0.101	3.135E-04	Sapur.017G123300	11917633	11919738	+	0.156	2.626E-01	NB-ARC domain-containing disease resistance protein	pink	NA
Chr17	11917756	11918251	164	0.387	0.271	-0.116	1.800E-05	Sapur.017G123400	11918283	11918717	+	0.417	1.433E-03	NB-ARC domain-containing disease resistance protein	grey	NA
Chr17	11958669	11959151	150	0.282	0.178	-0.103	1.300E-05	Sapur.017G124000	11958335	11958658	-	-0.142	6.466E-01	NB-ARC domain-containing disease resistance protein	grey	NA
Chr18	82510	83005	151	0.214	0.091	-0.123	1.277E-03	Sapur.018G001700	83885	86273	+	3.327	1.310E-136	ChaC-like family protein	cyan	NA
Chr18	903025	903519	181	0.292	0.184	-0.108	1.710E-04	Sapur.018G012700	904114	907983	+	0.562	8.150E-68	ubiquitin-specific protease 22	cyan	SDR eQTL
Chr18	948523	949018	140	0.260	0.153	-0.108	6.117E-03	Sapur.018G013300	949642	955615	+	0.168	1.945E-01	Protein phosphatase 2C family protein	grey	NA
Chr18	5782547	5783040	235	0.382	0.263	-0.119	5.950E-04	Sapur.018G052200	5777776	5781593	-	-0.423	8.080E-22	protein arginine methyltransferase 10	purple	NA
Chr18	7320809	7321287	190	0.456	0.299	-0.157	0.000E+00	Sapur.018G064700	7321952	7324086	+	-0.419	6.590E-05	translocase inner membrane subunit 17-2	grey	NA
Chr18	9606705	9606974	48	0.236	0.092	-0.144	1.900E-06	Sapur.018G091300	9607729	9608232	+	1.554	4.355E-03	S-adenosyl-L-methionine-dependent methyltransferases superfamily protein	cyan	NA
Chr18	11231383	11231873	197	0.211	0.060	-0.151	7.360E-04	Sapur.018G111400	11232100	11236296	+	2.254	7.550E-09	receptor like protein 9	cyan	NA

Chr19	135778	136273	199	0.115	0.218	0.103	3.030E-05	Sapur.019G001800	134197	135656	-	-0.464	4.961E-02	BURP domain-containing protein	grey	NA
Chr19	768503	768999	162	0.282	0.136	-0.145	4.542E-02	Sapur.019G006000	769809	785628	+	-0.447	2.731E-02	MAPK/ERK kinase kinase 1	grey	NA
Chr19	2503792	2504276	126	0.297	0.125	-0.172	3.803E-02	Sapur.019G018900	2502488	2503186	-	1.059	1.311E-03		grey	NA
Chr19	5360988	5361487	187	0.613	0.449	-0.164	5.121E-04	Sapur.019G043000	5361630	5363560	+	0.561	8.526E-04	Nucleotide-diphospho-sugar transferases superfamily protein	grey	NA
Chr19	5732866	5733356	180	0.377	0.221	-0.156	2.031E-02	Sapur.019G045800	5733759	5739930	+	-0.175	9.398E-01	RNApolymerase sigma subunit 2	brown	NA
Chr19	8471481	8471978	202	0.168	0.061	-0.107	1.269E-03	Sapur.019G055900	8463779	8471140	-	-0.310	1.450E-12	Tetratricopeptide repeat (TPR)-like superfamily protein	purple	NA
Chr19	11998798	11999290	169	0.310	0.204	-0.106	5.000E-07	Sapur.019G086600	11997728	11998258	-	0.680	4.334E-01	CLAVATA3/ESR-RELATED 4	grey	NA
Chr19	12238380	12238870	151	0.318	0.183	-0.134	3.000E-07	Sapur.019G089600	12236328	12237441	-	1.216	1.190E-07	homolog of carrot EP3-3 chitinase	grey	NA
Chr19	12512657	12513149	177	0.132	0.008	-0.124	3.319E-02	Sapur.019G094200	12513192	12519684	+	0.275	4.210E-01		grey	NA
