# Supplementary material for: Integrative genomics reveals paths to sex dimorphism in Salix purpurea L
Source: Hortic Res. 2021 Aug 1;8:170. doi: 10.1038/s41438-021-00606-y (PMC8325687; doi:10.1038/s41438-021-00606-y)
Supplement: Supplementary file 7 — Supplementary Tables S8-S9 [file 41438_2021_606_MOESM7_ESM.pdf]

Supplementary Table S8

GO ID	GO Description	Genes Present	FDR
6139	nucleobase, nucleoside, nucleotide and nucleic acid metabolic process	472	2.71E-05
6260	DNA replication	53	2.95E-02
6261	DNA-dependent DNA replication	41	4.73E-02
6334	nucleosome assembly	13	3.55E-02
6355	regulation of transcription, DNA-dependent	377	2.95E-02
6807	nitrogen compound metabolic process	584	1.99E-03
7018	microtubule-based movement	27	2.95E-02
9451	RNA modification	134	3.22E-16
9698	phenylpropanoid metabolic process	36	3.73E-02
16070	RNA metabolic process	297	4.27E-04
19219	regulation of nucleobase, nucleoside, nucleotide and nucleic acid metabolic process	407	2.95E-02
19685	photosynthesis, dark reaction	11	2.95E-02
34641	cellular nitrogen compound metabolic process	566	1.19E-03
45449	regulation of transcription	378	2.95E-02
51171	regulation of nitrogen compound metabolic process	409	2.95E-02
51252	regulation of RNA metabolic process	388	3.55E-02
90304	nucleic acid metabolic process	409	3.84E-06

Supplementary Table S9

GO ID	GO Description	Genes Present	FDR
3	reproduction	238	2.33E-04
902	cell morphogenesis	79	3.75E-05
904	cell morphogenesis involved in differentiation	42	1.11E-03
3006	reproductive developmental process	205	4.73E-03
5975	carbohydrate metabolic process	200	9.61E-04
5996	monosaccharide metabolic process	38	1.16E-02
6066	alcohol metabolic process	60	6.82E-03
6082	organic acid metabolic process	183	3.15E-07
6090	pyruvate metabolic process	9	1.23E-02
6091	generation of precursor metabolites and energy	75	3.15E-07
6457	protein folding	52	4.16E-02
6464	protein modification process	299	1.73E-03
6468	protein amino acid phosphorylation	202	2.03E-02
6470	protein amino acid dephosphorylation	19	4.22E-02
6487	protein amino acid N-linked glycosylation	10	4.24E-02
6629	lipid metabolic process	178	1.95E-08
6631	fatty acid metabolic process	62	2.17E-05
6633	fatty acid biosynthetic process	37	3.33E-03
6638	neutral lipid metabolic process	6	1.85E-02
6639	acylglycerol metabolic process	6	1.85E-02
6641	triglyceride metabolic process	6	1.85E-02
6694	steroid biosynthetic process	15	4.50E-02
6720	isoprenoid metabolic process	39	2.60E-02
6753	nucleoside phosphate metabolic process	44	2.26E-02
6775	fat-soluble vitamin metabolic process	8	1.85E-02
6778	porphyrin metabolic process	26	7.76E-04
6779	porphyrin biosynthetic process	21	7.76E-04
6793	phosphorus metabolic process	249	2.33E-04
6796	phosphate metabolic process	249	2.26E-04
6810	transport	439	1.63E-16
6811	ion transport	108	1.38E-05
6812	cation transport	81	1.10E-03
6820	anion transport	23	4.24E-02
6886	intracellular protein transport	77	3.70E-05
6888	ER to Golgi vesicle-mediated transport	7	4.22E-02
6897	endocytosis	7	2.57E-02
6944	cellular membrane fusion	13	4.41E-02
6950	response to stress	456	5.24E-06
6970	response to osmotic stress	114	1.30E-04

6979	response to oxidative stress	68	2.61E-02
7275	multicellular organismal development	423	1.82E-07
7398	ectoderm development	38	3.72E-02
7568	aging	30	5.96E-03
8104	protein localization	104	5.59E-05
8152	metabolic process	1455	2.34E-03
8202	steroid metabolic process	21	4.50E-02
8300	isoprenoid catabolic process	7	2.57E-02
8361	regulation of cell size	83	4.04E-05
8544	epidermis development	38	3.72E-02
8610	lipid biosynthetic process	102	1.38E-05
9056	catabolic process	172	1.10E-03
9117	nucleotide metabolic process	44	2.26E-02
9144	purine nucleoside triphosphate metabolic process	21	4.50E-02
9145	purine nucleoside triphosphate biosynthetic process	21	4.50E-02
9165	nucleotide biosynthetic process	31	4.55E-02
9199	ribonucleoside triphosphate metabolic process	21	4.50E-02
9201	ribonucleoside triphosphate biosynthetic process	21	4.50E-02
9205	purine ribonucleoside triphosphate metabolic process	21	4.50E-02
9206	purine ribonucleoside triphosphate biosynthetic process	21	4.50E-02
9266	response to temperature stimulus	99	4.39E-03
9314	response to radiation	118	3.53E-02
9409	response to cold	68	1.62E-02
9414	response to water deprivation	58	4.20E-03
9415	response to water	58	1.13E-02
9416	response to light stimulus	115	2.95E-02
9553	embryo sac development	29	2.10E-02
9555	pollen development	48	4.64E-03
9606	tropism	19	1.98E-02
9607	response to biotic stimulus	160	6.22E-06
9617	response to bacterium	73	1.54E-03
9628	response to abiotic stimulus	327	1.16E-09
9629	response to gravity	19	6.73E-03
9630	gravitropism	16	2.11E-02
9651	response to salt stress	106	2.31E-04
9653	anatomical structure morphogenesis	146	6.71E-05
9657	plastid organization	35	7.65E-03
9694	jasmonic acid metabolic process	11	2.93E-02
9743	response to carbohydrate stimulus	54	8.09E-03
9744	response to sucrose stimulus	16	8.53E-03
9756	carbohydrate mediated signaling	13	2.60E-02
9767	photosynthetic electron transport chain	15	3.66E-04
9773	photosynthetic electron transport in photosystem I	9	1.23E-02

9814	defense response, incompatible interaction	37	3.33E-03
9817	defense response to fungus, incompatible interaction	15	2.88E-02
9826	unidimensional cell growth	63	4.73E-05
9832	plant-type cell wall biogenesis	23	1.18E-02
9856	pollination	50	3.14E-04
9860	pollen tube growth	27	2.09E-03
9867	jasmonic acid mediated signaling pathway	16	2.60E-02
9887	organ morphogenesis	51	3.52E-02
9888	tissue development	69	4.98E-02
9913	epidermal cell differentiation	38	2.90E-02
9914	hormone transport	19	3.57E-02
9926	auxin polar transport	18	4.05E-02
9932	cell tip growth	33	1.03E-03
9966	regulation of signal transduction	19	4.22E-02
9987	cellular process	1637	5.10E-08
10033	response to organic substance	248	8.65E-03
10035	response to inorganic substance	128	3.75E-05
10038	response to metal ion	104	1.92E-04
10149	senescence	18	7.26E-03
10150	leaf senescence	13	2.04E-02
10182	sugar mediated signaling pathway	13	2.60E-02
10189	vitamin E biosynthetic process	5	4.63E-02
10193	response to ozone	11	2.93E-02
10236	plastoquinone biosynthetic process	4	2.24E-02
10260	organ senescence	14	9.48E-03
10324	membrane invagination	7	2.57E-02
10565	regulation of cellular ketone metabolic process	12	1.48E-02
10817	regulation of hormone levels	39	2.10E-02
15031	protein transport	103	1.38E-05
15698	inorganic anion transport	18	4.56E-02
15849	organic acid transport	25	3.96E-02
15979	photosynthesis	55	1.87E-09
15994	chlorophyll metabolic process	18	3.82E-03
15995	chlorophyll biosynthetic process	15	6.48E-04
16042	lipid catabolic process	25	2.00E-03
16043	cellular component organization	242	9.51E-05
16044	cellular membrane organization	32	3.14E-04
16049	cell growth	82	8.30E-06
16051	carbohydrate biosynthetic process	64	2.11E-02
16053	organic acid biosynthetic process	88	2.42E-03
16054	organic acid catabolic process	26	9.98E-03
16103	diterpenoid catabolic process	5	2.24E-02
16115	terpenoid catabolic process	7	2.57E-02

16192	vesicle-mediated transport	76	1.67E-08
16310	phosphorylation	229	8.58E-04
18130	heterocycle biosynthetic process	42	1.65E-03
19318	hexose metabolic process	29	4.53E-02
19684	photosynthesis, light reaction	28	2.55E-04
19685	photosynthesis, dark reaction	5	2.24E-02
19725	cellular homeostasis	45	2.77E-02
19752	carboxylic acid metabolic process	183	2.88E-07
21700	developmental maturation	20	2.16E-02
22414	reproductive process	233	2.73E-04
22900	electron transport chain	28	2.31E-05
23033	signaling pathway	150	4.60E-02
23051	regulation of signaling process	19	4.22E-02
30003	cellular cation homeostasis	23	3.71E-02
30004	cellular monovalent inorganic cation homeostasis	6	6.81E-03
30007	cellular potassium ion homeostasis	5	6.02E-03
30154	cell differentiation	92	9.74E-05
31407	oxylipin metabolic process	13	2.60E-02
31408	oxylipin biosynthetic process	11	4.16E-02
32501	multicellular organismal process	450	1.14E-08
32502	developmental process	469	1.25E-08
32535	regulation of cellular component size	83	4.64E-05
32787	monocarboxylic acid metabolic process	105	1.14E-08
32940	secretion by cell	17	4.41E-02
32989	cellular component morphogenesis	83	9.51E-05
33013	tetrapyrrole metabolic process	27	5.18E-04
33014	tetrapyrrole biosynthetic process	24	1.08E-04
33036	macromolecule localization	139	1.69E-03
33238	regulation of cellular amine metabolic process	5	2.24E-02
34285	response to disaccharide stimulus	16	1.17E-02
34613	cellular protein localization	77	1.57E-04
35295	tube development	33	4.76E-03
35466	regulation of signaling pathway	31	2.18E-02
40007	growth	92	8.95E-06
42180	cellular ketone metabolic process	187	1.37E-07
42221	response to chemical stimulus	443	1.69E-08
42360	vitamin E metabolic process	5	4.63E-02
42362	fat-soluble vitamin biosynthetic process	8	1.85E-02
42440	pigment metabolic process	37	1.94E-04
42592	homeostatic process	59	1.12E-02
42742	defense response to bacterium	61	1.55E-03
43412	macromolecule modification	312	4.24E-02
43436	oxoacid metabolic process	183	2.88E-07

43687	post-translational protein modification	260	8.87E-03
44038	cell wall macromolecule biosynthetic process	7	4.22E-02
44242	cellular lipid catabolic process	23	2.42E-03
44248	cellular catabolic process	129	2.29E-02
44255	cellular lipid metabolic process	134	1.95E-08
44262	cellular carbohydrate metabolic process	112	1.23E-02
44271	cellular nitrogen compound biosynthetic process	109	4.10E-04
44281	small molecule metabolic process	344	1.87E-09
44282	small molecule catabolic process	53	1.92E-03
44283	small molecule biosynthetic process	170	1.03E-04
45184	establishment of protein localization	103	1.38E-05
45487	gibberellin catabolic process	5	2.24E-02
46148	pigment biosynthetic process	31	6.22E-04
46394	carboxylic acid biosynthetic process	88	2.42E-03
46395	carboxylic acid catabolic process	26	9.98E-03
46483	heterocycle metabolic process	104	5.59E-05
46486	glycerolipid metabolic process	19	4.22E-02
46686	response to cadmium ion	93	5.10E-06
46903	secretion	17	4.41E-02
46907	intracellular transport	109	3.44E-07
46942	carboxylic acid transport	25	3.96E-02
48193	Golgi vesicle transport	20	2.32E-04
48229	gametophyte development	71	7.73E-05
48468	cell development	60	6.33E-04
48588	developmental cell growth	36	2.18E-04
48589	developmental growth	73	4.57E-06
48610	reproductive cellular process	45	9.18E-04
48856	anatomical structure development	350	1.67E-05
48868	pollen tube development	33	4.76E-03
48869	cellular developmental process	130	1.59E-05
48878	chemical homeostasis	39	5.24E-03
50801	ion homeostasis	29	3.70E-02
50896	response to stimulus	794	2.97E-11
51179	localization	449	9.62E-16
51186	cofactor metabolic process	70	3.89E-04
51188	cofactor biosynthetic process	50	9.74E-05
51234	establishment of localization	440	2.32E-16
51641	cellular localization	134	1.09E-07
51649	establishment of localization in cell	126	6.83E-08
51704	multi-organism process	207	1.69E-08
51707	response to other organism	154	8.51E-06
51716	cellular response to stimulus	187	1.34E-03
55046	microgametogenesis	10	3.03E-02

55075	potassium ion homeostasis	5	6.02E-03
55080	cation homeostasis	26	2.91E-02
55086	nucleobase, nucleoside and nucleotide metabolic process	54	1.49E-02
55114	oxidation reduction	48	5.01E-04
60560	developmental growth involved in morphogenesis	63	4.73E-05
60918	auxin transport	19	2.88E-02
61024	membrane organization	32	3.14E-04
61025	membrane fusion	13	4.41E-02
65008	regulation of biological quality	174	5.10E-08
70589	cellular component macromolecule biosynthetic process	7	4.22E-02
70592	cell wall polysaccharide biosynthetic process	7	4.22E-02
70727	cellular macromolecule localization	82	1.21E-04
70882	cellular cell wall organization or biogenesis	28	4.51E-02
70887	cellular response to chemical stimulus	103	9.81E-04
71310	cellular response to organic substance	93	1.48E-03
71322	cellular response to carbohydrate stimulus	13	2.60E-02
71395	cellular response to jasmonic acid stimulus	16	2.60E-02
71495	cellular response to endogenous stimulus	73	7.05E-03
90066	regulation of anatomical structure size	83	4.64E-05
