# Supplementary material for: Integrative genomics reveals paths to sex dimorphism in Salix purpurea L
Source: Hortic Res. 2021 Aug 1;8:170. doi: 10.1038/s41438-021-00606-y (PMC8325687; doi:10.1038/s41438-021-00606-y)
Supplement: Supplementary file 8 — Supplementary Table S10 [file 41438_2021_606_MOESM8_ESM.pdf]

SUPPLEMENTARY TABLE S10

Gene ID	log2-Fold Change M:F	FDR	Arabidopsis Homolog Name	Description	Category	Type	Notes on function	Source	Heterozygote Dominance Deviation	Percent distance from additive mean	p-value
Sapur.001G000400.3	-1.068	1.347E-01	PUR ALPHA-1	transcription factor Pur-alpha 1	Transcription	trans	binds telomeric repeat sequence	UniProt	-0.004015503	-26.76%	0.43996014
Sapur.001G003600.1	1.424	1.390E-10	HRE2	AP2/ERF domain transcription factor	Transcription	trans	senescence	TAIR; Uniprot	-0.236415777	-48.39%	0.203024626
Sapur.001G054400.1	-0.010	9.913E-01	LEP	AP2/ERF domain transcription factor	Transcription	trans	Positive regulator of gibberellin induced germination	TAIR	0.129477972	651.68%	0.000344165
Sapur.001G069800.1	-0.071	8.485E-01		Dof-type zinc finger DNA-binding family protein	Transcription	trans	-	-	-0.008440334	-32.28%	0.415962781
Sapur.001G136800.1	0.434	2.579E-01	CRF4	cytokinin response factor 4	Transcription; Hormone Signaling	trans	cytokinin signaling, transcriptional activator, cotyledon, leaf and embryo development	UniProt	-0.59777885	-91.11%	0.000594206
Sapur.001G178800.1	1.095	2.915E-04	EDF2,RAP2.8,RAV2,TEM2	B3 DNA-binding domain protein	Transcription	trans	repressor of flowering in long-day plants, activator of stress signal transduction pathways	UniProt	-0.391598244	-100.00%	0
Sapur.002G007100.1	-1.278	1.980E-28		anthranilate N-benzoyltransferase, putative	Secondary Metabolism	trans	involved upstream of phytoalexin production	UniProt	-0.342897914	-30.44%	0.281239997
Sapur.002G025600.1	0.286	2.109E-03	DFR,M318,TT3	dihydroflavonol 4-reductase	Secondary Metabolism	trans	Flavenoid metabolism	UniProt	-7.901753163	-18.12%	0.337520992
Sapur.002G081900.1	-5.566	6.980E-118		transcription factor bHLH96	Transcription	trans	-	-	-0.003698966	-0.35%	0.496852821
Sapur.002G137700.1	-1.189	4.190E-08	ATOMT1,OMT1	caffeic acid O-methyltransferase	Secondary Metabolism	trans	Converts quercetin into isorhamnetin, methylates flavenoids and monolignols	UniProt	0.148111307	26.08%	0.403629538
Sapur.003G027300.1	1.633	7.580E-116		Homeodomain-like superfamily protein	Transcription	trans	-	-	-5.508204805	-19.64%	0.0400617
Sapur.003G047200.1	-0.214	1.302E-01	ATMYB66,MYB66,WER,WER 1	Myb-like transcription factor 2, putative	Transcription	trans	Involved in epidermal cell fate specification in roots and hypocotyl	UniProt	2.466524275	17.63%	0.410301084
Sapur.003G069200.1	0.013	9.132E-01	ANAC002,ATAF1	NAC (No Apical Meristem) domain transcriptional regulator superfamily protein	Transcription	trans	Response to wounding and abscisic acid	TAIR	-0.326061428	-21.65%	0.237971333
Sapur.003G085800.1	-0.492	3.490E-01		AP2/ERF domain transcription factor	Transcription	trans	-	-	-0.400666216	-51.09%	0.400473393
Sapur.003G115100.1	-0.786	2.550E-01	ATMYB67,ATY53,MYB67	transcription factor Myb protein	Transcription	trans	-	-	-0.005143045	-34.67%	0.416216558
Sapur.003G133600.1	-1.086	1.620E-16	ATCHS,CHS,TT4	chalcone synthase	Secondary Metabolism	trans	flavenoid biosynthesis, naringenin synthesis	UniProt	1.974074992	21.24%	0.362919979
Sapur.003G149400.1	0.114	2.799E-01	ATST2A,ST2A	flavonol 4'-sulfotransferase, putative	Secondary Metabolism	trans	jasmonic acid metabolism	UniProt	8.272934231	10.03%	0.363014037
Sapur.003G155500.1	-1.059	1.780E-53	SCL-3,SCL3	scarecrow-like 3	Transcription	trans	axillary meristem reulation during vegetative and reproductive growth	TAIR; Uniprot	0.445974996	7.86%	0.366459646
Sapur.004G007600.1	-0.886	3.280E-30	ADT1	arogenate dehydratase 1	Secondary Metabolism	trans	catalyzes arogenate to phenylalanine	TAIR; UniProt	-1.013351596	-13.47%	0.361532543
Sapur.004G013400.1	0.104	2.693E-01	CPC	Homeodomain-like superfamily protein	Transcription	trans	Determines epidermal cell fate, supresses WER	UniProt	15.54322919	18.84%	0.337273825
Sapur.004G017600.2	-0.124	8.633E-01		transcription factor bHLH123	Transcription	trans	-	-	-0.04558561	-56.93%	0.181542539
Sapur.004G078800.1	-0.109	3.935E-01	CYP82G1	cytochrome P450, family 82, subfamily G, polypeptide 1	Secondary Metabolism	trans	homoterpene biosynthesis, floral herbivory response	UniProt	0.067209669	1.37%	0.486305998
Sapur.004G082600.1	-1.242	1.480E-27		LuxR transcriptional regulator, putative	Transcription	trans	-	-	0.088921054	1.89%	0.485279809
Sapur.004G088900.1	-0.629	3.636E-01	ATWRKY7,WRKY7	WRKY DNA-binding protein 7	Transcription	trans	Calmodulin binding protein	TAIR	-0.061916709	-41.71%	0.358660197
Sapur.004G089000.1	-0.370	6.169E-01	RRTF1	AP2/ERF domain transcription factor	Transcription	trans	-	-	0.145538835	433.93%	0.243465321
Sapur.004G103400.1	-1.147	1.159E-04	AGL29	AGAMOUS-like 29	Transcription; Floral Development	trans	transcription factor, activated by MIKC genes, supresses 76 genes, supressed by AGL18, high expression levels in pollen	TAIR; Verelst et. al. 2007	-0.0091499	-1.20%	0.49441233
Sapur.004G104400.1	-1.565	1.640E-179	ATHB30,HB30,ZFHD3	homeobox protein 30	Transcription	trans	-	-	0.759563089	2.07%	0.4841976
Sapur.004G104500.1	-0.451	6.700E-05	MIF2	mini zinc finger 2	Transcription	trans	Ovary development and floral meristem determination	TAIR	-0.214518422	-1.44%	0.485658161
Sapur.004G110200.3	0.056	NA	REM39,VRN1	B3 domain transcription factor VRN1	Transcription	trans	vernalization response and regulation of flower development	TAIR	-0.012603913	-27.86%	0.410562213
Sapur.004G117100.1	-0.277	2.016E-01	ATRL6,RL6,RSM3	RAD-like 6	Transcription	trans	-	-	0.39457724	4.38%	0.48063796
Sapur.004G124000.1	0.491	3.210E-06		SAUR-like auxin-responsive protein family	Hormone singaling	trans	-	-	21.31586427	41.21%	0.270397116
Sapur.004G124300.1	1.147	1.870E-08		SAUR-like auxin-responsive protein family	Hormone singaling	trans	-	-	0.439203741	41.03%	0.385145291
Sapur.004G124600.1	1.465	1.040E-08		SAUR-like auxin-responsive protein family	Hormone singaling	trans	-	-	-1.455155409	-23.51%	0.37049335
Sapur.005G022400.1	-0.454	2.390E-05	ALY1,ATALY1	DIRP Myb-like DNA-binding domain	Transcription	trans	-	-	-0.212068128	-4.34%	0.469163483
Sapur.005G057900.1	-1.987	1.376E-03	AtMYB93,MYB93	Myb-like DNA-binding domain protein	Transcription	trans	negative regulator of lateral root development, auxin response	UniProt	0.082635585	179.41%	0.317988668
Sapur.005G077400.1	3.986	4.150E-30	anac042,NAC042	NAC transcription factor 030	Transcription	trans	delays senescence	TAIR; Uniprot	-0.081157811	-38.19%	0.384642785
Sapur.005G087200.2	0.167	4.482E-01		winged-helix DNA-binding transcription factor family protein	Transcription	trans	-	-	-0.256348999	-6.48%	0.436227979
Sapur.005G093200.1	-2.591	1.490E-16	ANAC037,VND1	NAC transcription factor	Transcription	trans	xylem formation and secondary cell wall biosynthesis	UniProt	0.007492507	13.81%	0.445713995
Sapur.005G191800.1	0.401	3.375E-04	GASA1	gibberellin-regulated protein	Hormone singaling	trans	Involved in seed germination and maturation and flowering	UniProt	-4.51255671	-60.65%	1.37E-09
Sapur.006G089400.1	-0.919	7.050E-05	ATWRKY70,WRKY70	WRKY transcription factor	Transcription	trans	stress response, salicylic acid and jasmonic acid signalling	UniProt	0.885807794	32.67%	0.395298838
Sapur.006G118600.1	-0.145	1.640E-05		mediator of RNA polymerase II transcription subunit 20, putative	Transcription	trans	-	-	0.778167715	3.47%	0.417506187
Sapur.006G140600.1	0.860	5.430E-77	ATCOL5,COL5	CONSTANS-like 5	Transcription; Floral Development	trans	regulation of floral development	UniProt	-2.191113938	-10.90%	0.220112544
Sapur.007G068100.1	-0.836	5.804E-03	ABS,AGL32,TT16	MADS-box DNA-binding domain transcription factor	Transcription; Floral Development	trans	regulates proanthocyanidin biosynthesis, involved in seed coat and ovule development, maternal role in fertilization and seed development, transcription factor	TAIR	-0.034349935	-67.03%	0.221130563
Sapur.007G074000.1	-1.407	4.500E-07	ATWRKY13,WRKY13	WRKY transcription factor, putative	Transcription	trans	Stem development	TAIR	0.054450975	96.54%	0.185400858
Sapur.008G107000.1	-0.608	8.200E-52	RGA,RGA1	DELLA domain GRAS family transcription factor GAI	Transcription; Hormone Signaling	trans	positive regulation of transcription, repressor of GA response, DELLA protein, restrains cell proliferation and expansion, represses vegetative and floral growth	TAIR; UniProt	4.825409314	18.72%	0.168531993
Sapur.009G026400.1	0.034	7.898E-01		gibberellin-regulated protein	Hormone singaling	trans	-	-	-0.351131744	-3.85%	0.459347878
Sapur.009G039400.1	3.305	2.540E-17	ANAC002,ATAF1	NAC domain protein	Transcription	trans	Response to wounding and abscisic acid	TAIR	-0.071824254	-48.63%	0.30521124
Sapur.009G044100.1	0.701	2.830E-24		cinnamyl-alcohol dehydrogenase-like protein	Secondary Metabolism	trans	cinamaldehyde, coniferaldehyde, 5OH coniferaldehyde, sinapaldehyde, coumaraldehyde and caffyl aldehyde to alcohols, lignin monomers, involved in floral stem lignin biosynthesis	UniProt	-0.032843057	-86.43%	0.077113497
Sapur.009G078200.1	0.008	9.008E-01	CCoAOMT1	caffeoyl-CoA 3-O-methyltransferase	Secondary Metabolism	trans	phenylpropanoid biosynthesis	UniProt	-45.38773416	-13.72%	0.153140855
Sapur.010G016400.1	2.353	1.050E-193		flavonol synthase/flavanone 3-hydroxylase, putative	Secondary Metabolism	trans	flavenols from dihydroflavenols, oxidation of naringenin to dihyrokaempherol, dihydrokaempferol to kaempferol	UniProt	-5.036553	-75.19%	1.43E-08
Sapur.010G023900.1	-1.277	9.776E-02	DRN-LIKE,DRNL,ESR2,SOB2	DORNROSCHEN-like	Transcription	trans	embryo patterning, gibberellin singaling	UniProt	0.060502114	271.92%	0.198818425
Sapur.010G039800.1	0.444	3.700E-26	bHLH104	helix loop helix DNA-binding domain protein, putative	Transcription	trans	-	-	-2.046633148	-4.96%	0.410178102
Sapur.010G040000.1	-0.065	9.536E-01		ethylene-responsive transcription factor ERF098, putative	Transcription	trans	stress response	UniProt	-0.030673055	-36.19%	0.376793639
Sapur.010G076600.1	-1.287	3.400E-39		bHLH-MYC/R2R3-MYB transcription factor amino-terminal protein	Transcription	trans	-	-	2.242483938	3.99%	0.433517091
Sapur.010G128900.1	5.719	0.000E+00	AGL30	AGAMOUS-like 30	Transcription; Floral Development	trans	pollen maturation and pollen tube growth	UniProt	-12.65705924	-91.60%	0
Sapur.010G137700.4	0.045	NA	AS2	asymmetric leaves protein, putative	Transcription	trans	leaf symmetry and venation	UniProt	-0.002724643	-18.98%	0.474896427
Sapur.010G139800.1	-1.163	1.350E-32		shikimate/quinate hydroxycinnamoyltransferase	Secondary Metabolism	trans	synthsizing and catabolizing hydroxycinnamoylesters. Influences flavenoid production and auxin homeostasis	UniProt	1.250878455	11.89%	0.424965182
Sapur.010G149000.6	-1.362	3.850E-03	CBB3,CPD,CYP90,CYP90A,CYP90A1,DWF3	cytochrome P450 family 90 monooxygenase, putative	Secondary Metabolism; Floral Development	trans	involved in transition to flowering, affects FLC expression levels, necessary for male development	TAIR	0.077455955	189.65%	0.149530569
Sapur.010G149700.1	-1.770	1.090E-09	AUX1,MAP1,PIR1,WAV5	auxin influx carrier-like protein	Hormone singaling	trans	Mediates auxin gradient in dveloping leaves	UniProt	-0.01557154	-29.05%	0.377083799
Sapur.012G009500.1	-5.385	2.340E-119	LAS,SCL18	GRAS family transcription factor lateral protein	Transcription	trans	axillary meristem reulation during vegetative and reproductive growth	TAIR; Uniprot	-0.013251576	-1.89%	0.476386538
Sapur.012G020100.1	0.706	1.010E-20	CRF4	cytokinin response factor 4	Transcription	trans	cytokinin signaling, transcriptional activator, cotyledon, leaf and embryo development	UniProt	-1.06095952	-10.30%	0.354314831
Sapur.012G062200.1	-0.895	1.291E-04	LOB	lateral organ boundaries-like protein	Transcription	trans	lateral organ development	TAIR	0.166310562	128.99%	0.155738116
Sapur.012G096400.1	-3.575	1.630E-65	ABS,AGL32,TT16	MADS-box DNA-binding domain transcription factor	Transcription; Floral Development	trans	regulates proanthocyanidin biosynthesis, involved in seed coat and ovule development, maternal role in fertilization and seed development, transcription factor	TAIR	1.663209688	105.37%	0.106391536
Sapur.012G102000.1	0.379	1.581E-01	AGL6	AGAMOUS-like 6	Transcription; Floral Development	trans	TF, positive regulation of transcription, negative regulation of FLC/MAF and positive regulation of FT, vegetative to reproductive phase transition, promotes flowering	TAIR; Dreni and Zhang 2016	-0.036735375	-27.92%	0.356229975
Sapur.013G014400.2	0.437	1.247E-02		transcription factor VIP2, putative	Transcription	trans	Regulates gamma-tocopherol synthesis	UniProt	-0.024350455	-57.98%	0.305949531
Sapur.013G099600.3	-4.206	3.980E-57	AGL11,STK	agamous MADS box factor transcription factor	Transcription; Floral Development	trans	Maternal control of endothelium formation and female gametophyte development	UniProt	0.180494016	34.52%	0.378988347
Sapur.014G006800.1	-1.022	1.910E-142	WLIM1	LIM transcription factor	Transcription	trans	Cytoskeleton organization	TAIR	-20.11691212	-13.32%	0.353855873
Sapur.014G027100.1	-1.573	8.900E-07	CYP82C4	cytochrome P450 family protein	Secondary Metabolism	trans	Fe deficiency response, hydroxylates fraxetin to generate sideretin (catecholic coumarins)	TAIR	-0.091297243	-52.08%	0.215651744
Sapur.014G058300.1	-0.593	3.892E-01		AP2/B3-like transcriptional factor family protein	Transcription	trans	-	-	-0.003621898	-30.07%	0.441531892
Sapur.014G078300.1	-1.765	1.430E-41		AP2/ERF domain transcription factor	Transcription	trans	ethylene response, stress signal transduction	TAIR; Uniprot	-0.449452786	-22.42%	0.362254483
Sapur.016G011400.1	0.637	5.580E-07	UGT71C4	UDP-glucose flavonoid 3-O-glucosyltransferase, putative	Secondary Metabolism	trans	flavenol glucoside biosynthesis	UniProt	1.963748981	12.85%	0.353394587
Sapur.016G011500.1	-0.397	8.380E-05	HYR1	UDP-glucose flavonoid 3-O-glucosyltransferase, putative	Secondary Metabolism	trans	flavenol glucoside biosynthesis	UniProt	-1.426100907	-13.14%	0.336091687
Sapur.016G089900.1	0.069	6.019E-01		myb/SANT-like DNA-binding domain protein	Transcription	trans	-	-	1.614254318	29.21%	0.397746768
Sapur.016G114900.1	-1.171	9.760E-88	LHW	transcription factor-related	Transcription	trans	Regulates root development and promotes stele production	UniProt	8.469974273	29.23%	0.245840539
Sapur.016G174500.3	0.054	NA	WLIM1	transcription factor LIM1	Transcription	trans	Cytoskeleton organization	TAIR	0.069664186	880.30%	0.031696545
Sapur.016G184400.1	-2.628	5.680E-05	ATTPS21,TPS21	terpene synthase 21	Secondary Metabolism	trans	Sesquiterpene synthesis, produces beta-cayophyllene and alpha-humulene	TAIR	-0.05427384	-61.77%	0.29304533
Sapur.016G273800.1	-0.516	5.710E-07	SCL5	scarecrow-like 5	Transcription	trans	Involved in plant development	UniProt	-2.685639609	-26.48%	0.138134447
Sapur.016G273900.1	1.092	3.040E-06	ATTPS21,TPS21	terpene synthase 21	Secondary Metabolism	trans	Sesquiterpene synthesis, produces beta-cayophyllene and alpha-humulene	TAIR	9.482131021	50.28%	0.305830363

Sapur.016G274000.1	-0.404	7.670E-07	PAT1	GRAS family transcription factor	Transcription	trans	mRNA decapping	TAIR	0.048750239	0.46%	0.494543761
Sapur.017G008600.1	-0.117	2.097E-01	ATGLK1,GLK1,GPRI1	golden-like transcription factor, putative	Transcription	trans	regulates chloroplast development	TAIR	-0.408862145	-6.90%	0.398002416
Sapur.017G014200.1	-1.142	3.480E-06	SGR7,SHR	GRAS family transcription factor	Transcription	trans	quiescent center cell specification	UniProt	0.158284491	79.53%	0.18851619
Sapur.017G015700.1	-0.769	7.520E-08	SGR7,SHR	GRAS family transcription factor	Transcription	trans	quiescent center cell specification	UniProt	-0.085590236	-9.65%	0.421828771
Sapur.017G067900.2	0.107	8.718E-01	ATHB13	Homeobox-leucine zipper protein family	Transcription	trans	involved in seed to seedling transition	TAIR	0.060825254	261.60%	0.076929306
Sapur.017G107600.1	-1.178	4.740E-34	AtMYB43,MYB43	myb domain protein 43	Transcription	trans	phenylpropanoid biosynthesis	TAIR	1.145767164	37.76%	0.182486866
Sapur.018G062500.1	-1.591	9.370E-64	TINY2	AP2/ERF domain transcription factor	Transcription	trans	stress response	UniProt	-1.008529935	-19.21%	0.314412226
Sapur.019G065400.1	1.678	5.130E-124	CYP714A1	cytochrome P450 family protein	Hormone singaling	trans	Inactivation of early gibberellin intermediates	UniProt	-15.67688143	-46.21%	0.000609148
Sapur.019G074900.8	-5.779	5.390E-126	AGL11,STK	agamous MADS box factor transcription factor	Transcription; Floral Development	trans	Maternal control of endothelium formation and female gametophyte development	UniProt	0.913434876	203.65%	0.051954672
Sapur.019G112000.1	0.993	6.270E-85		BZIP transcription factor	Transcription	trans	-	-	-10.02029276	-25.77%	0.011738972
Sapur.15WG012900.1	-0.355	3.420E-44	ATO	splicing factor 3a subunit 3, putative	RNA splicing and regulation	trans	pre-splicosome formation, necessary for gametic cell fate determination	UniProt	-0.362459118	-0.85%	0.479573968
Sapur.15WG022500.3	0.018	NA	AGO1	argonaute protein 1B	RNA splicing and regulation	cis	component of RISC, involved in RNA post-transcriptional silencing via 21-22 nt RNAs	UniProt	0.015845775	146.21%	0.26040154
Sapur.15WG023500.1	-0.594	8.810E-27	ATBTF3,BTF3	basic transcription factor 3	Transcription	cis	seed development	TAIR; Uniprot	-1.011411406	-0.81%	0.48800724
Sapur.15WG023800.1	-1.228	1.580E-20	anac057,NAC057	NAC transcription factor	Transcription	cis	-	-	-0.00539782	-0.68%	0.495612738
Sapur.15WG024100.1	-0.456	3.180E-19	EPR1,RVE7	Homeodomain-like superfamily protein	Transcription	cis	Modification of cell-wall structure	UniProt	2.190480682	13.38%	0.27192435
Sapur.15WG026400.1	1.645	1.470E-59	ATMYB52,BW52,MYB52	myb domain protein 52	Transcription	cis	Drought tolerance, secondary cell wall biosynthesis	UniProt	-0.227529133	-7.30%	0.463064695
Sapur.15WG031800.1	-5.340	1.360E-109	AtMYB6,MYB6	MYB-like transcription factor	Transcription	cis	-	-	-0.032636883	-4.84%	0.487818481
Sapur.15WG032500.1	-0.646	2.420E-48	AtMYB4R1,MYB4R1	Myb transcription factor, putative	Transcription	cis	regulates accumulation of UV protectant sinapoylmalate	TAIR; Uniprot	-0.947710667	-17.94%	0.155123552
Sapur.15WG038800.1	-6.149	4.490E-292		PLATZ transcription factor family protein	Transcription	cis	-	-	NA	NA	NA
Sapur.15WG039100.1	-6.183	8.940E-158		PLATZ transcription factor family protein	Transcription	cis	-	-	NA	NA	NA
Sapur.15WG039400.1	-5.732	8.450E-124		PLATZ transcription factor family protein	Transcription	cis	-	-	-0.19635684	-20.72%	0.57106592
Sapur.15WG039600.1	-1.732	8.980E-29		PLATZ transcription factor family protein	Transcription	cis	-	-	0.408051975	17.42%	0.353073949
Sapur.15WG039700.1	-5.549	1.810E-115		PLATZ transcription factor family protein	Transcription	cis	-	-	0.971295514	68.66%	0.190619934
Sapur.15WG039800.1	-4.194	2.320E-62		PLATZ transcription factor family protein	Transcription	cis	-	-	0.294703766	20.73%	0.378742442
Sapur.15WG042400.1	-2.176	1.860E-138	ADT2	arogenate dehydratase 2	Secondary Metabolism	cis	catalyzes arogenate to phenylalanine	TAIR	-0.818118927	-56.55%	0.994724545
Sapur.15WG045900.1	-1.427	3.510E-09	anac058,NAC058	NAC transcription factor 005	Transcription	cis	nuclear and plasma membrane targeting	TAIR	0.025452877	13.96%	0.449794577
Sapur.15WG046800.1	-5.814	1.110E-143	WOX1	WUSCHEL related homeobox 1	Transcription; Floral Development	cis	Plant development	UniProt	0.995119791	55.44%	0.106423415
Sapur.15WG056200.1	-2.058	8.710E-12	ARR22,RR22	response regulator 22	Hormone singaling	cis	Involved in two-component cytokinin response	TAIR	-0.139820947	-61.12%	0.763291721
Sapur.15WG056300.1	-4.640	3.620E-83	WOX1	WUSCHEL related homeobox 1	Transcription; Floral Development	cis	Plant development	UniProt	0.320026593	69.74%	0.126632321
Sapur.15WG057600.1	-6.250	2.250E-148	ATTCP18,BRC1,TCP18	transcription factor TCP24	Transcription	cis	regulates bud growth	TAIR; Uniprot	0.542541753	82.82%	0.117104887
Sapur.15WG058800.1	-1.113	2.180E-20		constans-like zinc finger protein	Transcription; Floral Development	cis	transcription factor, regulation of flowering, acts on SOC1, FT, TFL, photoreceptor regulated, could be sexually antagonistic gene, upstream of flowering pathway, could be regulating many other genes	TAIR; UniProt	-1.077559494	-35.75%	0.87260121
Sapur.15WG060600.1	-1.119	8.240E-09	IDN2,RDM12	transcription factor X1-like protein	Transcription	cis	RDDM pathway, AGO4 dependant binding of lnc-RNA	UniProt	-0.015823678	-15.07%	0.602052562
Sapur.15WG062800.1	-7.630	4.500E-231	GATA15	GATA transcription factor 15	Transcription; Floral Development	cis	transcription factor, floral and vegetative development	TAIR	1.358814322	19.85%	0.291359137
Sapur.15WG065500.1	-0.066	7.959E-01	AtbZIP70,bZIP70	bZIP transcription factor	Transcription	cis	-	-	-0.500457344	-42.25%	0.77813865
Sapur.15WG073900.1	-2.847	3.750E-23	ARR17,RR17	response regulator 17	Hormone singaling	cis	Type-A cytokinin response regulator	TAIR	0.044790833	22.78%	0.410801924
Sapur.15WG074000.1	-4.056	3.410E-56	ARR17,RR17	response regulator 17	Hormone singaling	cis	Type-A cytokinin response regulator	TAIR	0.025216631	4.13%	0.475121304
Sapur.15WG074200.1	-2.806	5.030E-19	HCT	hydroxycinnamoyl-CoA shikimate/quinate hydroxycinnamoyl transferase	Secondary Metabolism	cis	lignin biosynthesis	UniProt	-0.038179274	-42.04%	0.75570537
Sapur.15WG074400.1	-7.937	2.430E-240	AGO4,OCP11	Argonaute family protein	RNA splicing and regulation	cis	RDDM pathway	TAIR	0.472315512	12.44%	0.403356956
Sapur.15WG075200.1	-4.031	3.510E-55	ARR17,RR17	response regulator 17	Hormone singaling	cis	Type-A cytokinin response regulator	TAIR	0.033180883	5.46%	0.467423277
Sapur.15WG079800.1	-0.672	5.640E-64	APRR1,AtTOC1,PRR1,TOC1	timing of cab expression 1/PRR response regulator 1	Hormone singaling; Floral Development	cis	Two componenet cytokinin response	UniProt	-5.345270439	-18.70%	0.933899357
Sapur.15WG084600.1	0.932	1.070E-47	ATHB-1,ATHB1,HAT5,HB-1,HD-ZIP-1	homeobox 1	Transcription	trans	Leaf development, transcriptional activator	UniProt	8.681407324	17.29%	0.333599211
Sapur.15WG088100.2	0.522	1.380E-05		MYB transcription factor, putative	Transcription	trans	-	-	0.24983345	36.02%	0.329170612
Sapur.15WG100700.1	-0.308	3.618E-04	ATMYB86,MYB86	R2R3-MYB transcription factor, putative	Transcription	trans	sinapiate ester production	UniProt	-0.124090837	-6.11%	0.572955811
Sapur.15WG121000.1	-0.657	1.280E-35	ARF1-BP,ARF2,HSS,ORE14	auxin response factor 2	Hormone Signaling; Floral Development	trans	promotes flowering and stamen development	UniProt	12.25471372	8.57%	0.35917787
Sapur.15ZG033300.5	-2.114	3.790E-15	ATPIN1,PIN1	auxin efflux carrier component 1	Hormone Signaling	cis	Auxin gradient formation during organogenesis	UniProt	0.017395467	27.70%	0.379957203
Sapur.15ZG035700.1	-1.305	2.310E-24	AtMYB6,MYB6	MYB-like transcription factor	Transcription	cis	-	-	0.500036709	33.96%	0.24995866
Sapur.15ZG039200.1	-0.314	2.745E-03	ADT2	arogenate dehydratase 2	Secondary Metabolism	cis	catalyzes arogenate to phenylalanine	TAIR	0.040965123	2.49%	0.470061794
Sapur.15ZG048100.1	1.519	2.800E-07	anac058,NAC058	NAC transcription factor 005	Transcription	cis	nuclear and plasma membrane targeting	TAIR	0.811795248	140.34%	0.209895275
Sapur.15ZG048600.1	-7.267	0.000E+00	BEE3	transcription factor BEE	Transcription	cis	brassinosteroid signalling	TAIR; Uniprot	3.927666448	24.30%	0.259643207
Sapur.15ZG053700.1	2.310	1.240E-34	AtbZIP70,bZIP70	bZIP transcription factor	Transcription	cis	-	-	1.064744139	35.48%	0.392857871
Sapur.15ZG055400.1	0.578	9.180E-08	ATTCP18,BRC1,TCP18	transcription factor TCP24	Transcription	cis	Delays axillary bud outgrowth and development	UniProt	-0.090461807	-8.88%	0.569140319
Sapur.15ZG055800.1	0.658	4.020E-72		AP2/B3-like transcriptional factor family protein	Transcription	cis	-	-	-0.294764643	-2.76%	0.538203755
Sapur.T004700.1	-0.758	1.240E-137	AtWRKY20,WRKY20	WRKY transcription factor	Transcription	trans	-	-	2.558173434	14.98%	0.022182773
Sapur.T004800.2	-1.076	9.248E-04	SCL5	scarecrow-like 5	Transcription	trans	Involved in plant development	UniProt	0.037533222	128.96%	0.132970903
Sapur.T077300.1	1.342	1.290E-12	CYP97B3	cytochrome P450, family 97, subfamily B, polypeptide 3	Secondary Metabolism	trans	xanthophyll biosynthesis, hydroxylates beta rings of beta carotene	UniProt	0.149471863	4.47%	0.476844747
Sapur.T078000.1	1.440	2.950E-28	ATO	splicing factor-related	RNA splicing and regulation	trans	pre-splicosome formation, necessary for gametic cell fate determination	UniProt	0.553787679	33.24%	0.325143777
Sapur.T078100.1	1.418	1.060E-07	CYP97B3	cytochrome P450, family 97, subfamily B, polypeptide 3	Secondary Metabolism	trans	xanthophyll biosynthesis, hydroxylates beta rings of beta carotene	UniProt	0.018230052	2.13%	0.490838807
Sapur.T168300.1	-0.211	9.064E-01		PLATZ transcription factor family protein	Transcription	trans	-	-	-0.000894472	-16.26%	0.522513077
