# Supplementary material for: Integrative genomics reveals paths to sex dimorphism in Salix purpurea L
Source: Hortic Res. 2021 Aug 1;8:170. doi: 10.1038/s41438-021-00606-y (PMC8325687; doi:10.1038/s41438-021-00606-y)
Supplement: Supplementary file 9 — Supplementary Table S11 [file 41438_2021_606_MOESM9_ESM.pdf]

SUPPLEMENTARY TABLE S11

Transcript ID	Base Mean	log2 Fold-Change M:F	log -fold change SE	Statistic	p-value	FDR	Module membership	Gene Correlation to Male	GSPvalue	Gene Ontology	Arabidopsis homolog	Arabidopsis Symbol	Arabidopsis Description	Salix gene description
Sapur.016G293900.2	23.56843842	-1.305412868	0.067535265	-19.3294	3.04E-83	2.78E-82	black	-0.59107255	3.66E-16	GO:0016020	AT1G33265.1		Transmembrane proteins 14C	transmembrane protein 14C
Sapur.016G145800.1	1123.812019	-0.704844446	0.036650616	-19.2314	2.02E-82	1.82E-81	black	-0.851003191	3.31E-45	GO:0003676	AT1G51510.1	Y14	RNA-binding (RRM/RBD/RNP motifs) family protein	RNA-binding protein RBM8A RNA recognition motif protein
Sapur.005G149500.1	172.150132	-0.840094252	0.044708722	-18.7904	9.05E-79	7.74E-78	black	-0.839273369	7.29E-43	GO:0003676,GO:0017069,GO:0000398	AT1G21320.2		nucleotide binding;nucleic acid binding	RNA-binding protein with multiple splicing protein, putative
Sapur.010G205500.1	167.0812601	-0.874182415	0.04705308	-18.5786	4.78E-77	3.99E-76	black	-0.831923851	1.73E-41		AT1G16790.1		ribosomal protein-related	hypothetical protein
Sapur.004G170400.1	426.3086412	-1.284436824	0.071388171	-17.9923	2.24E-72	1.75E-71	black	-0.842377615	1.83E-43	GO:0006414,GO:0003746,GO:0043043,GO:0005737	AT3G08740.1		elongation factor P (EF-P) family protein	elongation factor P
Sapur.002G006700.1	446.8872606	-1.261190715	0.070440681	-17.9043	1.09E-71	8.43E-71	black	-0.846003083	3.49E-44	GO:0006396	AT1G20580.1		Small nuclear ribonucleoprotein family protein	small nuclear ribonucleoprotein Sm protein D3
Sapur.010G147400.1	913.6818735	-0.559583072	0.031473616	-17.7794	1.02E-70	7.77E-70	black	-0.809434365	1.15E-37	GO:0005515,GO:0042393,GO:0006355	AT5G05610.2	AL1	alfin-like 1	PHD finger protein
Sapur.016G215800.1	211.4276984	-0.698691722	0.040737364	-17.1511	6.16E-66	4.36E-65	black	-0.8108713	6.77E-38		AT5G40570.1		Surfeit locus protein 2 (SURF2)	surfeit locus protein
Sapur.005G201200.1	706.8631866	-1.024277064	0.061097012	-16.7648	4.42E-63	2.99E-62	black	-0.825435311	2.49E-40	GO:0006396	AT1G20580.1		Small nuclear ribonucleoprotein family protein	small nuclear ribonucleoprotein Sm protein D3
Sapur.016G064800.1	491.7734777	-1.139627601	0.068568901	-16.6202	4.98E-62	3.32E-61	black	-0.817095352	6.56E-39		AT5G06360.1		Ribosomal protein S8e family protein	NA
Sapur.15WG072500.1	382.4935288	-0.781277832	0.048416101	-16.1367	1.41E-58	8.88E-58	black	-0.818620069	3.65E-39	GO:0003676	AT5G61030.1	GR-RBP3	glycine-rich RNA-binding protein 3	RNA recognition motif 2 in yeast gar2-like protein
Sapur.017G099000.1	189.571667	-1.009945526	0.062718968	-16.1027	2.44E-58	1.53E-57	black	-0.833640981	8.35E-42	GO:0019843,GO:0006412,GO:0005840,GO:0005622,GO:0003735	AT1G16740.1		Ribosomal protein L20	50S ribosomal protein L20
Sapur.008G150400.1	866.3909516	-1.052652732	0.065569081	-16.0541	5.35E-58	3.34E-57	black	-0.819715783	2.39E-39		AT1G70600.1		Ribosomal protein L18e/L15 superfamily protein	60S ribosomal protein L27a
Sapur.006G003900.1	317.6622394	-0.577775628	0.036055232	-16.0247	8.59E-58	5.35E-57	black	-0.760248639	7.70E-31		AT5G47680.1			tRNA (guanine(37)-N(1))-methyltransferase
Sapur.009G056500.1	285.6752159	-0.844487512	0.052857883	-15.9766	1.86E-57	1.15E-56	black	-0.828185877	8.13E-41		AT1G65700.3		Small nuclear ribonucleoprotein family protein	U6 snRNA-associated-like-Smprotein LSM8
Sapur.002G049200.1	3885.002981	-0.506533087	0.03201169	-15.8234	2.15E-56	1.31E-55	black	-0.791646979	5.49E-35		AT4G02450.1		HSP20-like chaperones superfamily protein	HSP90 co-chaperone, putative
Sapur.014G103200.1	1066.980522	-1.064833234	0.067351516	-15.8101	2.65E-56	1.61E-55	black	-0.818826737	3.37E-39	GO:0030532,GO:0008380	AT2G47640.3		Small nuclear ribonucleoprotein family protein	small nuclear ribonucleoprotein Sm protein D2
Sapur.014G043300.1	2310.49738	-0.598711346	0.037902873	-15.7959	3.32E-56	2.02E-55	black	-0.797132323	8.75E-36	GO:0006412,GO:0005840,GO:0003735	AT4G16720.1		Ribosomal protein L23/L15e family protein	60S ribosomal protein L15
Sapur.010G084600.1	378.8421678	-1.349311311	0.087777372	-15.372	2.52E-53	1.47E-52	black	-0.830390615	3.27E-41	GO:0006457,GO:0005737	AT1G23100.1		GroES-like family protein	10 kDa chaperonin
Sapur.013G116500.1	1165.467232	-1.039146331	0.068228392	-15.2304	2.22E-52	1.27E-51	black	-0.79658129	1.05E-35	GO:0006412,GO:0005840,GO:0005622,GO:0003735	AT4G34620.1	SSR16	small subunit ribosomal protein 16	30S ribosomal protein S16
Sapur.007G123000.1	480.7805675	-0.789852718	0.052008117	-15.1871	4.31E-52	2.45E-51	black	-0.808868533	1.41E-37		AT2G33845.1		Nucleic acid-binding, OB-fold-like protein	OB fold SoSSB-OBF: A subfamily protein, putative
Sapur.014G063500.1	203.2836743	-0.640713463	0.042188912	-15.1868	4.33E-52	2.46E-51	black	-0.767258103	1.04E-31		AT1G01730.1			hypothetical protein
Sapur.003G135500.1	199.8417967	-0.722624962	0.048599707	-14.8689	5.25E-50	2.87E-49	black	-0.76623098	1.40E-31	GO:0006412,GO:0005840,GO:0005622,GO:0003735	AT1G80620.1		S15/NS1, RNA-binding protein	NA
Sapur.007G087000.1	1248.91845	-0.719232247	0.048464615	-14.8404	8.03E-50	4.39E-49	black	-0.784295128	5.93E-34	GO:0006412,GO:0005840,GO:0005622,GO:0003735	AT5G10360.1	EMB3010,RPS6B	Ribosomal protein S6e	40S ribosomal protein S6
Sapur.005G008700.1	146.4801471	-1.101186447	0.074832474	-14.7154	5.14E-49	2.77E-48	black	-0.806096243	3.83E-37		AT5G26800.1			NA
Sapur.004G074600.1	2144.568813	-0.658170508	0.045123657	-14.5859	3.45E-48	1.84E-47	black	-0.772577214	2.18E-32		AT3G62870.1		Ribosomal protein L7Ae/L30e/S12e/Gadd45 family protein	60S ribosomal protein L7a
Sapur.018G028600.1	1473.078791	0.835215937	0.057313217	14.57283	4.18E-48	2.22E-47	black	0.752696179	6.17E-30		AT2G25660.1	emb2410	embryo defective 2410	embryo defective 2410 protein
Sapur.15WG008600.1	263.0177275	-0.77369944	0.053252798	-14.5288	7.96E-48	4.21E-47	black	-0.773746722	1.53E-32		AT5G24314.1	PDE225,PTAC7	plastid transcriptionally active7	plastid transcriptionally active protein, putative
Sapur.016G000800.1	633.1713977	-0.602847648	0.04152233	-14.5186	9.23E-48	4.87E-47	black	-0.779716638	2.49E-33	GO:0015934,GO:0006412,GO:0003735	AT5G64670.1		Ribosomal protein L18e/L15 superfamily protein	ribosomal protein L15
Sapur.019G056000.1	1152.96778	-0.671363235	0.046263272	-14.5118	1.02E-47	5.38E-47	black	-0.772109627	2.50E-32		AT1G08580.1			hypothetical protein
Sapur.007G085200.1	1279.241548	-0.819262429	0.057619305	-14.2185	7.03E-46	3.59E-45	black	-0.72578939	5.77E-27	GO:0006412,GO:0005840,GO:0005622,GO:0003735	AT5G65220.1		Ribosomal L29 family protein	50S ribosomal protein L29
Sapur.005G082000.1	43.60120739	-1.279981037	0.090440755	-14.1527	1.80E-45	9.12E-45	black	-0.761937236	4.79E-31	GO:0006412,GO:0005840,GO:0003735	AT4G35490.1	MRPL11	mitochondrial ribosomal protein L11	50S ribosomal protein L11
Sapur.014G127400.1	1016.871493	0.71351266	0.050608183	14.09876	3.86E-45	1.95E-44	black	0.708963059	2.79E-25	GO:0006511	AT4G15420.1		Ubiquitin fusion degradation UFD1 family protein	ubiquitin fusion degradation protein
Sapur.011G044700.1	960.3015422	-0.953345957	0.067791743	-14.0629	6.42E-45	3.23E-44	black	-0.790257944	8.67E-35	GO:0006412,GO:0005840,GO:0005622,GO:0003735	AT1G29070.1		Ribosomal protein L34	ribosomal protein L34
Sapur.003G063200.2	22.38798994	-1.221771213	0.087182098	-14.014	1.28E-44	6.39E-44	black	-0.535669712	4.87E-13		AT2G35500.1	SKL2	shikimate kinase like 2	shikimate kinase-like protein
Sapur.005G130800.1	1723.273284	0.919070117	0.065910272	13.94426	3.41E-44	1.69E-43	black	0.737843528	2.99E-28		AT4G30100.1		P-loop containing nucleoside triphosphate hydrolases superfamily protein	tRNA-splicing endonuclease positive effector-like protein
Sapur.014G076000.1	641.1384977	0.777009846	0.055913744	13.89658	6.64E-44	3.28E-43	black	0.770622797	3.89E-32		AT3G61690.1		nucleotidyltransferases	nucleotidyltransferase
Sapur.017G068200.1	1066.908286	-0.97407922	0.070310087	-13.854	1.20E-43	5.90E-43	black	-0.76860305	7.04E-32	GO:0006412,GO:0005840,GO:0005622,GO:0003735	AT1G26880.1		Ribosomal protein L34e superfamily protein	60S ribosomal protein L34
Sapur.T045100.1	393.7108675	-0.581580648	0.042026722	-13.8384	1.50E-43	7.34E-43	black	-0.763516331	3.06E-31		AT3G07510.1			transmembrane protein, putative
Sapur.010G204000.2	8.060494274	5.311054131	0.387060915	13.72149	7.55E-43	3.66E-42	black	0.468751218	5.96E-10		AT1G51560.1		Pyridoxamine 5\'-phosphate oxidase family protein	pyridoxamine 5'-phosphate oxidase family protein
Sapur.006G125200.1	73.65272106	-0.877816645	0.064461647	-13.6177	3.14E-42	1.51E-41	black	-0.742422576	9.29E-29		AT1G55160.1			transmembrane protein, putative
Sapur.003G053000.1	2368.996223	-0.635858352	0.046884461	-13.5622	6.71E-42	3.19E-41	black	-0.733113809	9.73E-28	GO:0006412,GO:0005840,GO:0005622,GO:0003735	AT3G10950.1		Zinc-binding ribosomal protein family protein	60S ribosomal protein L37a
Sapur.005G071400.1	432.8558205	-0.567267924	0.0419836	-13.5117	1.33E-41	6.34E-41	black	-0.757691572	1.57E-30		AT5G23290.1	PFD5	prefoldin 5	prefoldin subunit 5, putative
Sapur.005G021500.1	254.7477013	0.829625706	0.061490816	13.49186	1.75E-41	8.27E-41	black	0.742574121	8.93E-29	GO:0008270	AT3G62900.1		CW-type Zinc Finger	CW-type zinc finger protein
Sapur.007G032200.1	259.7612866	-1.06967741	0.079496803	-13.4556	2.85E-41	1.35E-40	black	-0.749705761	1.38E-29		AT4G37090.1			hypothetical protein
Sapur.009G076300.1	617.9634245	-0.512482137	0.038264254	-13.3932	6.62E-41	3.11E-40	black	-0.761365674	5.63E-31	GO:0046983,GO:0006351,GO:0003899	AT2G15430.1	NRPB3,NRPD3,NRPE3A,RBP	DNA-directed RNA polymerase family protein	DNA-directed RNA polymerase II subunit RPB3
Sapur.001G092400.1	410.1146261	-0.421189086	0.031943666	-13.1854	1.07E-39	4.89E-39	black	-0.721374456	1.64E-26		AT2G45640.1	ATSAP18,SAP18	SIN3 associated polypeptide P18	histone deacetylase complex subunit SAP18
Sapur.012G020800.1	93.22740056	-0.890142595	0.067741752	-13.1402	1.94E-39	8.84E-39	black	-0.702929442	1.05E-24		AT5G52960.1			DUF3143 family protein
Sapur.010G035700.1	115.4415633	-0.857642329	0.065294948	-13.1349	2.08E-39	9.48E-39	black	-0.749997905	1.27E-29	GO:0006412,GO:0005840,GO:0003735	AT5G09510.1		Ribosomal protein S19 family protein	40S ribosomal protein S15
Sapur.004G010300.1	192.5184691	-0.451665433	0.034471226	-13.1027	3.18E-39	1.45E-38	black	-0.697587364	3.30E-24		AT4G04780.1	MED21	mediator 21	mediator of RNA polymerase II transcription subunit 21
Sapur.016G195000.1	203.8511866	-1.138933904	0.087372819	-13.0353	7.70E-39	3.47E-38	black	-0.736628221	4.05E-28	GO:0003676	AT4G13850.1	ATGRP2,GR-RBP2,GRP2	glycine-rich RNA-binding protein 2	eukaryotic RNA-binding protein RBM24 RNA recognition motif protein
Sapur.002G067300.1	205.7131902	-0.574074002	0.044081223	-13.0231	9.04E-39	4.07E-38	black	-0.724319292	8.19E-27		AT1G48200.1			hypothetical protein
Sapur.T050600.1	99.13633386	-0.646882724	0.049751015	-13.0024	1.19E-38	5.32E-38	black	-0.704107477	8.12E-25	GO:0003676,GO:0006810,GO:0005622	AT5G43960.1		Nuclear transport factor 2 (NTF2) family protein with RNA binding (RRM-RBD-RNP motifs) domain	Ras GTPase-activating protein-binding protein
Sapur.017G008800.1	87.78023634	-0.947523493	0.072962955	-12.9864	1.46E-38	6.55E-38	black	-0.722746843	1.19E-26		AT1G06720.1		P-loop containing nucleoside triphosphate hydrolases superfamily protein	NA
Sapur.002G124300.1	1337.517236	0.653202322	0.050561151	12.91906	3.51E-38	1.56E-37	black	0.730258185	1.96E-27	GO:0003824,GO:0005515	AT2G42010.1	PLDBETA,PLDBETA1	phospholipase D beta 1	phospholipase D
Sapur.001G133800.1	982.7049908	-0.842087184	0.065300561	-12.8956	4.77E-38	2.12E-37	black	-0.763008376	3.53E-31	GO:0006412,GO:0005840,GO:0005622,GO:0003735,GO:0015935	AT1G72370.1	AP40,P40,RP40,RPSAA	40s ribosomal protein SA	40S ribosomal protein Sa protein
Sapur.001G144300.1	772.5371472	0.621127088	0.048188948	12.88941	5.16E-38	2.29E-37	black	0.749972563	1.28E-29		AT5G20030.1		Plant Tudor-like RNA-binding protein	agenet domain protein
Sapur.016G225000.2	17.75533306	-1.250866606	0.097124259	-12.879	5.91E-38	2.62E-37	black	-0.622879425	3.03E-18	GO:0008168	AT1G29790.1		S-adenosyl-L-methionine-dependent methyltransferases superfamily protein	S-adenosyl-L-methionine-dependent methyltransferase, putative
Sapur.010G064800.1	3540.035993	-0.746015817	0.057955703	-12.8722	6.46E-38	2.86E-37	black	-0.743835249	6.45E-29	GO:0006412,GO:0005840,GO:0005622,GO:0003735	AT4G34670.1		Ribosomal protein S3Ae	40S ribosomal protein S3a
Sapur.008G007800.1	2269.235892	-0.850764175	0.066139248	-12.8632	7.25E-38	3.21E-37	black	-0.738367416	2.62E-28	GO:0006412,GO:0005840,GO:0005622,GO:0003735	AT5G64140.1	RPS28	ribosomal protein S28	40S ribosomal protein S28
Sapur.008G088200.1	512.9442141	-1.142002025	0.088804653	-12.8597	7.59E-38	3.36E-37	black	-0.749398449	1.49E-29		AT5G08180.1		Ribosomal protein L7Ae/L30e/S12e/Gadd45 family protein	H/ACA ribonucleoprotein complex subunit 2
Sapur.001G023700.1	190.802384	-1.36147525	0.10592501	-12.8532	8.25E-38	3.65E-37	black	-0.693722125	7.43E-24	GO:0045454,GO:0015035,GO:0006662	AT3G06730.1	TRX P,TRX z	Thioredoxin z	thioredoxin-like protein CITRX protein
Sapur.009G037000.1	111.4833279	-0.933619072	0.073024276	-12.7851	1.99E-37	8.72E-37	black	-0.705001979	6.68E-25	GO:0005515,GO:0003723,GO:0016070,GO:0051028,GO:0045292,GO:0005846,GO:00003	AT2G13540.1	ABH1,ATCBP80,CBP80,ENS	ARM repeat superfamily protein	nuclear cap-binding protein
Sapur.15ZG050500.1	287.5255515	0.661138969	0.05185044	12.75088	3.08E-37	1.35E-36	black	0.733497958	8.84E-28	GO:0007165,GO:0005515,GO:0043531	AT1G27170.2		transmembrane receptors;ATP binding	TIR-NBS-LRR resistance protein
Sapur.15WG000400.1	1261.554663	-0.557342784	0.043772854	-12.7326	3.90E-37	1.70E-36	black	-0.735121585	5.91E-28	GO:0005515	AT5G24650.1		Mitochondrial import inner membrane translocase subunit Tim17/Tim22/Tim23 family protein	import inner membrane translocase subunit Tim17, putative
Sapur.008G123600.1	646.084405	-0.741861583	0.058674899	-12.6436	1.21E-36	5.25E-36	black	-0.750649624	1.07E-29	GO:0006412,GO:0005840,GO:0005622,GO:0003735	AT5G28060.1		Ribosomal protein S24e family protein	40S ribosomal protein S24
Sapur.012G072300.1	2206.82811	0.517396682	0.040962235	12.63107	1.42E-36	6.15E-36	black	0.755374959	2.97E-30	GO:0009378,GO:0006310,GO:0006281,GO:0005515	AT4G02480.1		AAA-type ATPase family protein	AAA family ATPase
Sapur.017G082500.1	922.0925096	-0.746717034	0.059151676	-12.6238	1.56E-36	6.74E-36	black	-0.735427239	5.48E-28		AT3G62870.1		Ribosomal protein L7Ae/L30e/S12e/Gadd45 family protein	NA
Sapur.15WG026500.1	2885.367571	-0.582701886	0.04616885	-12.6211	1.62E-36	6.97E-36	black	-0.730819117	1.71E-27	GO:0003723,GO:0006412,GO:0005840,GO:0005622,GO:0003735	AT5G07090.1		Ribosomal protein S4 (RPS4A) family protein	40S ribosomal protein S4
Sapur.011G051000.1	1264.175047	-0.562685943	0.04475736	-12.5719	3.01E-36	1.29E-35	black	-0.732064538	1.26E-27	GO:0006412,GO:0005840,GO:0003735	AT2G34480.1		Ribosomal protein L18ae/LX family protein	60S ribosomal protein L18a
Sapur.016G278000.1	1024.511212	-0.61185325	0.048754878	-12.5496	4.00E-36	1.71E-35	black	-0.758224522	1.36E-30		AT1G50900.1		Ankyrin repeat family protein	LHCP translocation defect protein
Sapur.012G056300.1	3223.480358	-0.415056432	0.033155848	-12.5183	5.93E-36	2.53E-35	black	-0.728258309	3.19E-27	GO:0003723	AT5G35530.1		Ribosomal protein S3 family protein	40S ribosomal protein S3
Sapur.016G144200.1	328.5126401	-0.584503765	0.046701336	-12.5158	6.12E-36	2.61E-35	black	-0.75583408	2.62E-30	GO:0003723	AT5G12410.1		THUMP domain-containing protein	THUMP domain protein
Sapur.001G168900.1	466.757446	-0.488988194	0.03909897	-12.5064	6.89E-36	2.93E-35	black	-0.700614802	1.73E-24		AT5G64160.1			plant/MHJ24-14 protein, putative
Sapur.005G111600.1	152.0988659	-0.685367973	0.054909862	-12.4817	9.40E-36	3.99E-35	black	-0.703066225	1.02E-24		AT5G64816.2			transmembrane protein, putative
Sapur.017G007500.1	829.9283671	-0.592069478	0.047505822	-12.4631	1.19E-35	5.02E-35	black	-0.769481916	5.44E-32		AT2G33845.1		Nucleic acid-binding, OB-fold-like protein	single-stranded DNA-binding protein, putative
Sapur.006G174700.1	351.2049512	-0.937616464	0.075292731	-12.4529	1.35E-35	5.70E-35	black	-0.709179336	2.66E-25		AT2G37400.1		Tetratricopeptide repeat (TPR)-like superfamily protein	TPR protein
Sapur.001G027800.1	2561.687788	-0.616229056	0.049773172	-12.3807	3.32E-35	1.39E-34	black	-0.724429999	7.97E-27		AT3G12390.1		Nascent polypeptide-associated complex (NAC), alpha subunit family protein	nascent polypeptide-associated complex subunit alpha-like protein
Sapur.002G044500.1	70.05636647	-0.964386783	0.07789939	-12.38	3.35E-35	1.41E-34	black	-0.710302172	2.07E-25	GO:0006412,GO:0005840,GO:0005622,GO:0003735	AT2G32060.1		Ribosomal protein L7Ae/L30e/S12e/Gadd45 family protein	NA
Sapur.010G197700.1	214.9499479	-0.732481817	0.059182499	-12.3767	3.50E-35	1.47E-34	black	-0.745220825	4.49E-29	GO:0005759	AT4G32605.1		Mitochondrial glycoprotein family protein	glycoprotein
Sapur.014G102900.1	1254.724585	-0.807455056	0.065362426	-12.3535	4.66E-35	1.95E-34	black	-0.740944811	1.36E-28	GO:0006412,GO:0005840,GO:0005622,GO:0003735	AT3G05560.2		Ribosomal L22e protein family	60S ribosomal protein L22, putative
Sapur.003G046800.1	358.668339	-0.716527834	0.058471089	-12.2544	1.59E-34	6.58E-34	black	-0.710823225	1.84E-25	GO:0006412,GO:0005840,GO:0003735	AT4G16720.1		Ribosomal protein L23/L15e family protein	60S ribosomal protein L15
Sapur.002G114900.1	1841.310472	-0.700146033	0.057773812	-12.1187	8.40E-34	3.43E-33	black	-0.696804592	3.89E-24	GO:0006412,GO:0005840,GO:0005622,GO:0003735	AT5G23740.1	RPS11-BETA	ribosomal protein S11-beta	40S ribosomal protein S11
Sapur.15WG032600.1	5318.722034	-0.523284271	0.043688577	-11.9776	4.66E-33	1.87E-32	black	-0.723680193	9.53E-27	GO:0005515	AT1G18080.1	ATARCA,RACK1A,RACK1A_A	Transducin/WD40 repeat-like superfamily protein	guanine nucleotide-binding protein subunit beta-like protein
Sapur.001G140900.1	1151.729371	-0.811132277	0.067828002	-11.9587	5.85E-33	2.35E-32	black	-0.714215564	8.56E-26	GO:0006412,GO:0005840,GO:0003735	AT4G16720.1		Ribosomal protein L23/L15e family protein	ribosomal protein L15
Sapur.009G127600.1	2604.729497	-0.561056315	0.04708968	-11.9146	9.93E-33	3.97E-32	black	-0.693477143	7.82E-24	GO:0015934,GO:0006412,GO:0003735				ribosomal protein L32
Sapur.004G142300.1	2293.742104	-0.612959747	0.051651046	-11.8673	1.75E-32	6.96E-32	black	-0.705076389	6.57E-25		AT3G44750.1	ATHD2A,HD2A,HDA3,HDT1	histone deacetylase 3	type 2 histone deacetylase
Sapur.016G137900.1	314.3796945	-0.710111685	0.059973978	-11.8403	2.42E-32	9.57E-32	black	-0.713782709	9.45E-26		AT5G52370.1			hypothetical protein
Sapur.007G083700.1	2754.18911	0.789151546	0.066715662	11.82858	2.78E-32	1.10E-31	black	0.682523917	7.30E-23	GO:0016887,GO:0005524,GO:0055085,GO:0042626,GO:0016021,GO:0006810	AT4G39850.1	ACN2,AtABCD1,CTS,PED3,P	peroxisomal ABC transporter 1	ABC transporter D family protein
Sapur.002G122300.1	95.72261816	-0.657038187	0.05554945	-11.828	2.80E-32	1.11E-31	black	-0.704740883	7.07E-25	GO:0006457,GO:0003755,GO:0000413	AT1G01940.1		Cyclophilin-like peptidyl-prolyl cis-trans isomerase family protein	peptidyl-prolyl cis-trans isomerase, cyclophilin-type protein
Sapur.006G084600.1	2735.203182	-0.516708287	0.043696932	-11.8248	2.91E-32	1.15E-31	black	-0.717272732	4.25E-26	GO:0003723,GO:0006412,GO:0005840,GO:0005622,GO:0003735	AT5G58420.1		Ribosomal protein S4 (RPS4A) family protein	40S ribosomal protein S4
Sapur.004G148400.1	2624.381848	-0.567436272	0.048028151	-11.8147	3.28E-32	1.30E-31	black	-0.714990683	7.18E-26	GO:0006412,GO:0005840,GO:0005622,GO:0003735	AT1G77940.1		Ribosomal protein L7Ae/L30e/S12e/Gadd45 family protein	60S ribosomal protein L30
Sapur.002G025400.1	1042.159322	-0.683392206	0.058018723	-11.7788	5.02E-32	1.97E-31	black	-0.662591565	3.34E-21	GO:0006412,GO:0005840,GO:0005622,GO:0003735	AT1G75350.1	emb2184	Ribosomal protein L31	50S ribosomal protein L31
Sapur.016G191300.1	2605.794518	-0.706535659	0.059985585	-11.7784	5.04E-32	1.98E-31	black	-0.714348795	8.31E-26	GO:0006412,GO:0005840,GO:0003735,GO:0015934	AT5G48760.1		Ribosomal protein L13 family protein	60S ribosomal protein L13a
Sapur.018G087200.1	1017.076043	-0.999160996	0.084945545	-11.7624	6.10E-32	2.39E-31	black	-0.693021137	8.60E-24	GO:0006412,GO:0005840,GO:0005622,GO:0003735	AT4G31985.1		Ribosomal protein L39 family protein	60S ribosomal protein L39
Sapur.008G071900.1	75.49825109	-0.982197408	0.083615033	-11.7467	7.35E-32	2.88E-31	black	-0.687381527	2.74E-23		AT1G26660.1		Prefoldin chaperone subunit family protein	prefoldin, alpha subunit
Sapur.005G161000.1	1565.654773	-0.742511774	0.063341298	-11.7224	9.79E-32	3.83E-31	black	-0.715602164	6.24E-26	GO:0006412,GO:0005840,GO:0003735	AT2G34480.1		Ribosomal protein L18ae/LX family protein	60S ribosomal protein L18a
Sapur.007G002500.1	1962.103581	0.571008463	0.048723482	11.71937	1.01E-31	3.97E-31	black	0.703256573	9.77E-25	GO:0008270,GO:0005515,GO:0006355,GO:0005634,GO:0004402,GO:0003712,GO:001657	AT1G79000.1	ATHAC1,ATHPCAT2,HAC1,P	histone acetyltransferase of the CBP family 1	histone acetyltransferase HAC12
Sapur.004G074500.1	3540.623926	-0.583924851	0.049957621	-11.6884	1.46E-31	5.69E-31	black	-0.691463168	1.19E-23		AT3G62870.1		Ribosomal protein L7Ae/L30e/S12e/Gadd45 family protein	60S ribosomal protein L7a
Sapur.014G091900.1	3235.28574	-0.660188385	0.056557274	-11.6729	1.75E-31	6.82E-31	black	-0.720013838	2.25E-26	GO:0006412,GO:0005840,GO:0003735	AT2G47110.1	UBQ6	ubiquitin 6	ubiquitin-40S ribosomal protein S27a
Sapur.012G044700.1	2485.31724	-0.593188198	0.050922513	-11.6488	2.33E-31	9.01E-31	black	-0.720757439	1.90E-26	GO:0003676	AT5G61030.1	GR-RBP3	glycine-rich RNA-binding protein 3	heterogeneous nuclear ribonucleoprotein G RNA recognition motif protein
Sapur.016G070900.1	2841.640113	-0.728431016	0.06260474	-11.6354	2.72E-31	1.05E-30	black	-0.734426059	7.03E-28	GO:0006412,GO:0005840,GO:0003735	AT3G52590.1	EMB2167,ERD16,HAP4,UBC	ubiquitin extension protein 1	ubiquitin-60S ribosomal L40 fusion protein
Sapur.003G160300.1	321.7544479	-0.608000895	0.052262061	-11.6337	2.78E-31	1.07E-30	black	-0.71592058	5.80E-26		AT3G57930.1			metal tolerance protein, putative
Sapur.008G052200.1	3772.814389	-0.634345798	0.054527868	-11.6334	2.79E-31	1.08E-30	black	-0.714988244	7.18E-26	GO:0042254,GO:0005622	AT2G40010.1		Ribosomal protein L10 family protein	60S acidic ribosomal protein P0
Sapur.15WG102300.1	702.8259322	-0.74605575	0.064211459	-11.6187	3.31E-31	1.28E-30	black	-0.679221509	1.40E-22	GO:0006412,GO:0005840,GO:0005622,GO:0003735	AT5G56670.1		Ribosomal protein S30 family protein	40S ribosomal protein S30
Sapur.007G037100.1	302.6627101	-0.797175439	0.068701775	-11.6034	3.96E-31	1.53E-30	black	-0.716722711	4.83E-26	GO:0008097,GO:0006412,GO:0005840,GO:0003735	AT5G66860.1		Ribosomal protein L25/Gln-tRNA synthetase, anti-codon-binding domain	50S ribosomal protein L25
Sapur.002G126400.1	967.3044576	-0.647468849	0.055919779	-11.5785	5.29E-31	2.04E-30	black	-0.673581931	4.21E-22		AT4G01310.1		Ribosomal L5P family protein	50S ribosomal protein L5
Sapur.002G167700.1	1168.643624	-0.832056912	0.071966106	-11.5618	6.44E-31	2.47E-30	black	-0.703559079	9.15E-25	GO:0006412,GO:0005840,GO:0003735	AT2G47110.2	UBQ6	ubiquitin 6	ubiquitin/ribosomal protein S27a
Sapur.014G058000.1	47.49358837	-0.942674994	0.081696616	-11.5387	8.42E-31	3.22E-30	black	-0.682498002	7.34E-23		AT1G01860.1	PFC1	Ribosomal RNA adenine dimethylase family protein	ribosomal RNA small subunit methyltransferase A
Sapur.013G057600.1	217.4590033	-0.890469366	0.077266081	-11.5247	9.90E-31	3.78E-30	black	-0.664183937	2.49E-21		AT3G03773.1		HSP20-like chaperones superfamily protein	p23 co-chaperone protein
Sapur.016G116900.1	84.8963723	-0.645607795	0.056035582	-11.5214	1.03E-30	3.93E-30	black	-0.68034743	1.13E-22		AT2G25570.3		binding	Sel1 domain protein
Sapur.016G080600.1	3966.518747	-0.526103282	0.045898415	-11.4623	2.04E-30	7.74E-30	black	-0.693332133	8.06E-24	GO:0006412,GO:0005840,GO:0003735	AT3G09630.1		Ribosomal protein L4/L1 family	60S ribosomal protein L4-1
Sapur.001G182500.1	200.7593176	-0.680327406	0.059356491	-11.4617	2.05E-30	7.79E-30	black	-0.68411644	5.31E-23	GO:0006412,GO:0003735	AT4G37660.1		Ribosomal protein L12/ ATP-dependent Clp protease adaptor protein ClpS family protein	ribosomal protein L7/L12 carboxy-terminal domain protein
Sapur.006G172700.1	296.1268155	-1.027930604	0.08992173	-11.4314	2.91E-30	1.10E-29	black	-0.730245803	1.97E-27		AT3G11500.1		Small nuclear ribonucleoprotein family protein	small nuclear ribonucleoprotein G, putative
Sapur.001G114500.1	16.74144341	-1.106249319	0.09686484	-11.4205	3.30E-30	1.25E-29	black	-0.665174068	2.07E-21		AT1G32310.1			hypothetical protein
Sapur.014G009800.1	209.911268	-0.580523654	0.050843994	-11.4177	3.41E-30	1.29E-29	black	-0.702241201	1.22E-24		AT5G65860.2		ankyrin repeat family protein	arginine N-methyltransferase
Sapur.017G082400.1	1399.331432	-0.494231315	0.04330319	-11.4133	3.59E-30	1.35E-29	black	-0.674356513	3.63E-22		AT3G62870.1		Ribosomal protein L7Ae/L30e/S12e/Gadd45 family protein	60S ribosomal protein L7a
Sapur.006G022500.1	592.2064455	-0.416108782	0.036535865	-11.389	4.74E-30	1.78E-29	black	-0.64009858	1.79E-19		AT3G12390.1		Nascent polypeptide-associated complex (NAC), alpha subunit family protein	nascent polypeptide associated complex protein alpha
Sapur.012G082000.1	1482.922448	-0.687359933	0.060365554	-11.3866	4.88E-30	1.83E-29	black	-0.701436423	1.45E-24	GO:0006412,GO:0005840,GO:0005622,GO:0003735	AT1G26880.1		Ribosomal protein L34e superfamily protein	60S ribosomal protein L34
Sapur.016G124300.1	8791.67637	0.694446238	0.061139617	11.35837	6.74E-30	2.53E-29	black	0.680542454	1.08E-22	GO:0016020,GO:0006075,GO:0003843,GO:0000148	AT2G31960.2	ATGSL03,ATGSL3,GSL03	glucan synthase-like 3	callose synthase
Sapur.013G043500.1	850.6862068	0.527740601	0.046463931	11.35807	6.76E-30	2.54E-29	black	0.690218305	1.54E-23	GO:0005634	AT1G08620.1	PKDM7D	Transcription factor jumonji (jmj) family protein / zinc finger (C5HC2 type) family protein	transcription factor jumonji family protein
Sapur.012G024100.1	1455.708894	-0.711891629	0.062766717	-11.3419	8.14E-30	3.05E-29	black	-0.693354099	8.03E-24	GO:0006412,GO:0005840,GO:0005622,GO:0003735	AT3G16780.1		Ribosomal protein L19e family protein	ribosomal protein L19
Sapur.014G038900.1	783.1336576	0.57248563	0.050536192	11.32823	9.51E-30	3.55E-29	black	0.699374548	2.25E-24	GO:0003676	AT2G44710.1		RNA-binding (RRM/RBD/RNP motifs) family protein	NA
Sapur.T049500.1	736.244808	-0.609172413	0.054025419	-11.2757	1.73E-29	6.44E-29	black	-0.682446665	7.42E-23	GO:0006412,GO:0005840,GO:0005622,GO:0003735	AT4G18100.1		Ribosomal protein L32e	60S ribosomal protein L32, putative
Sapur.006G172800.2	125.8433918	1.819822964	0.161612775	11.26039	2.06E-29	7.65E-29	black	0.36812716	2.10E-06	GO:0007165	AT5G22400.1		Rho GTPase activating protein with PAK-box/P21-Rho-binding domain	Rho GTPase-activating protein
Sapur.016G210300.1	67.39730169	-1.004724729	0.089256823	-11.2566	2.15E-29	7.99E-29	black	-0.691395206	1.21E-23	GO:0016020	AT5G40770.1	ATPHB3,PHB3	prohibitin 3	prohibitin
Sapur.005G168500.1	222.5118442	-0.676082899	0.06024618	-11.222	3.18E-29	1.18E-28	black	-0.668870694	1.04E-21		AT4G04614.1			hypothetical protein
Sapur.008G124900.1	264.9729594	-0.658288538	0.058957988	-11.1654	6.02E-29	2.22E-28	black	-0.708423807	3.14E-25	GO:0051082,GO:0016272,GO:0006457	AT3G22480.1	PDF2	prefoldin 2	prefoldin subunit 2
Sapur.009G043500.1	5683.946917	-0.564236027	0.050666212	-11.1363	8.35E-29	3.06E-28	black	-0.691544569	1.17E-23	GO:0006412,GO:0005840,GO:0005622,GO:0003735	AT5G59240.1		Ribosomal protein S8e family protein	40S ribosomal protein S8
Sapur.017G086300.1	129.4152304	-0.610436935	0.054837082	-11.1318	8.78E-29	3.22E-28	black	-0.649731177	3.39E-20		AT5G49550.1			biogenesis of lysosome-related organelles complex-1 subunit 2
Sapur.002G110200.1	41.2686862	-0.93281981	0.083856823	-11.124	9.59E-29	3.51E-28	black	-0.663029741	3.08E-21					transmembrane protein, putative
Sapur.005G156800.1	1812.129127	-0.366571014	0.032961925	-11.121	9.91E-29	3.63E-28	black	-0.66436291	2.41E-21		AT4G02450.1		HSP20-like chaperones superfamily protein	ripening regulated protein DDTFR8, putative
Sapur.017G009700.1	77.99573961	-0.824016642	0.074263892	-11.0958	1.31E-28	4.80E-28	black	-0.66236473	3.49E-21		AT5G44170.1		S-adenosyl-L-methionine-dependent methyltransferases superfamily protein	ribosomal protein L11 methyltransferase
Sapur.016G116400.1	3039.644136	-0.590295483	0.053506258	-11.0323	2.67E-28	9.70E-28	black	-0.680939095	1.00E-22	GO:0006412,GO:0005840,GO:0003735	AT3G52580.1		Ribosomal protein S11 family protein	40S ribosomal protein S14
Sapur.008G047400.1	1618.561675	-0.318788638	0.028896031	-11.0323	2.67E-28	9.70E-28	black	-0.659390763	6.02E-21	GO:0003676,GO:0005852,GO:0005737,GO:0003743	AT3G11400.1	ATEIF3G1,EIF3G1	eukaryotic translation initiation factor 3G1	eukaryotic translation initiation factor 3 subunit G
Sapur.016G059200.1	3846.945008	-0.523210601	0.047465682	-11.0229	2.96E-28	1.07E-27	black	-0.68762861	2.61E-23	GO:0006412	AT2G37270.2	ATRPS5B,RPS5B	ribosomal protein 5B	40S ribosomal protein S5, putative
Sapur.008G142500.1	2088.34503	-0.595928047	0.054088448	-11.0177	3.14E-28	1.14E-27	black	-0.699290794	2.29E-24	GO:0006412,GO:0005840,GO:0003735,GO:0015934	AT1G67430.1		Ribosomal protein L22p/L17e family protein	60S ribosomal protein L17
Sapur.010G152000.1	721.0148682	-0.868608354	0.078993298	-10.996	4.00E-28	1.45E-27	black	-0.684258115	5.16E-23	GO:0006412,GO:0005840,GO:0005622,GO:0003735	AT1G07070.1		Ribosomal protein L35Ae family protein	60S ribosomal protein L35a
Sapur.006G120600.1	1344.121695	0.577767958	0.052627699	10.9784	4.85E-28	1.75E-27	black	0.67402769	3.87E-22	GO:0016459,GO:0005524,GO:0003774,GO:0005515	AT5G20490.1	ATXIK,XI-17,XIK	Myosin family protein with Dil domain	myosin XI, putative
Sapur.005G092400.1	2067.693761	-0.614557996	0.056065888	-10.9614	5.86E-28	2.11E-27	black	-0.702106632	1.25E-24	GO:0006412,GO:0005840,GO:0005622,GO:0003735	AT4G36130.1		Ribosomal protein L2 family	60S ribosomal protein L2
Sapur.006G184700.1	16.60349008	-1.365513503	0.125492679	-10.8812	1.42E-27	5.06E-27	black	-0.64574394	6.80E-20		AT4G33100.1			protein phosphatase 2C, putative
Sapur.008G102000.1	1550.888168	0.553750658	0.050951718	10.86814	1.63E-27	5.84E-27	black	0.663433708	2.86E-21	GO:0005515	AT1G15130.1		Endosomal targeting BRO1-like domain-containing protein	mammalian alix-like protein interacting Bro1-like domain protein
Sapur.005G047500.1	98.40384736	1.034512897	0.095214494	10.86508	1.69E-27	6.03E-27	black	0.621410073	3.83E-18		AT2G13650.1	GONST1	golgi nucleotide sugar transporter 1	NA
Sapur.002G102200.1	274.9669415	-0.474363342	0.043730873	-10.8473	2.05E-27	7.31E-27	black	-0.639958129	1.83E-19		AT1G45230.1		Protein of unknown function (DUF3223)	DCL protein
Sapur.014G024200.1	31.20490079	-0.817140878	0.07537433	-10.8411	2.20E-27	7.82E-27	black	-0.654006884	1.59E-20	GO:0055114,GO:0006788,GO:0004392	AT2G26550.2	HO2	heme oxygenase 2	heme oxygenase
Sapur.15WG023500.1	1349.632915	-0.594223118	0.054867726	-10.8301	2.48E-27	8.81E-27	black	-0.644190543	8.89E-20		AT1G17880.1	ATBTF3,BTF3	basic transcription factor 3	transcription factor-BTF3-like protein
Sapur.003G101700.1	706.3469321	-0.37442999	0.034659839	-10.803	3.33E-27	1.18E-26	black	-0.674287649	3.68E-22		AT1G64350.1	SEH1H	Transducin/WD40 repeat-like superfamily protein	nucleoporin seh1
Sapur.001G188900.1	1174.363767	-0.279961288	0.025984966	-10.774	4.57E-27	1.61E-26	black	-0.636577201	3.24E-19	GO:0005515,GO:0042393,GO:0006355	AT2G02470.1	AL6	alfin-like 6	PHD finger ALFIN-like protein
Sapur.004G048600.1	550.1203534	0.663663841	0.061707568	10.75498	5.61E-27	1.98E-26	black	0.629867758	9.82E-19		AT1G73210.1		Protein of unknown function (DUF789)	DUF789 family protein
Sapur.005G036400.1	450.1518932	0.454519024	0.042409575	10.71737	8.44E-27	2.96E-26	black	0.666778874	1.53E-21	GO:0006468,GO:0004672	AT1G08720.1	ATEDR1,EDR1	Protein kinase superfamily protein	MAP kinase
Sapur.002G112400.1	618.194525	-0.350444355	0.03277107	-10.6937	1.09E-26	3.81E-26	black	-0.656363889	1.04E-20	GO:0005634	AT1G02140.1	HAP1,MAGO,MEE63	mago nashi family protein	mago nashi-like protein
Sapur.005G031800.1	436.0384471	0.487660164	0.045649999	10.68259	1.23E-26	4.29E-26	black	0.64602504	6.47E-20		AT1G54710.1	ATATG18H,ATG18H	homolog of yeast autophagy 18 (ATG18) H	breast carcinoma amplified sequence protein, putative
Sapur.15WG151100.1	2071.631763	-0.553472658	0.051816446	-10.6814	1.24E-26	4.34E-26	black	-0.641708815	1.36E-19	GO:0006412,GO:0005840,GO:0005622,GO:0003735	AT3G53740.2		Ribosomal protein L36e family protein	60S ribosomal protein L36
Sapur.008G030700.1	114.7355511	0.973823984	0.091188381	10.67926	1.27E-26	4.44E-26	black	0.657695367	8.19E-21					NA
Sapur.012G024200.1	35.3288466	-0.826573997	0.077409139	-10.678	1.29E-26	4.50E-26	black	-0.617589896	6.99E-18	GO:0006284	AT3G07930.3		DNA glycosylase superfamily protein	base excision DNA repair protein, HhH-GPD family protein
Sapur.006G146200.1	171.2849718	-0.662623441	0.06224057	-10.6462	1.82E-26	6.32E-26	black	-0.629364744	1.07E-18		AT4G30845.1			transmembrane protein, putative
Sapur.016G132700.1	2595.35662	-0.689723263	0.064858321	-10.6343	2.06E-26	7.17E-26	black	-0.715683795	6.13E-26		AT2G28900.1	ATOEP16-1,ATOEP16-L,OEP	outer plastid envelope protein 16-1	amino acid selective channel protein
Sapur.010G202200.1	174.7256377	-0.580614085	0.054648338	-10.6246	2.29E-26	7.94E-26	black	-0.619977979	4.80E-18		AT3G21200.1	PGR7	proton gradient regulation 7	glutamyl-tRNA reductase-binding protein
Sapur.006G092800.1	44.82292552	-0.82123138	0.077469182	-10.6007	2.96E-26	1.02E-25	black	-0.634311193	4.72E-19	GO:0006412,GO:0005840,GO:0005622,GO:0003735	AT3G54210.1		Ribosomal protein L17 family protein	50S ribosomal protein L17
Sapur.007G078500.1	2368.511153	-0.636816575	0.060242208	-10.5709	4.06E-26	1.40E-25	black	-0.67084862	7.11E-22	GO:0015934,GO:0006412,GO:0003735	AT3G49910.1		Translation protein SH3-like family protein	60S ribosomal protein L26
Sapur.006G176700.1	599.7010531	-0.468706765	0.044466111	-10.5408	5.60E-26	1.93E-25	black	-0.65743742	8.58E-21		AT5G02740.1		Ribosomal protein S24e family protein	hypothetical protein
Sapur.008G032200.1	229.1957544	-0.754394147	0.071606024	-10.5353	5.94E-26	2.04E-25	black	-0.699643313	2.13E-24		AT2G35790.1			transmembrane protein, putative
Sapur.016G070700.1	3739.636181	-0.710163424	0.067460601	-10.5271	6.48E-26	2.22E-25	black	-0.671760619	5.97E-22	GO:0019843,GO:0005622,GO:0003723	AT5G39850.1		Ribosomal protein S4	40S ribosomal protein S9
Sapur.016G107600.1	3779.191005	-0.517753572	0.049272198	-10.508	7.93E-26	2.72E-25	black	-0.671947107	5.77E-22	GO:0003723,GO:0006412,GO:0005840,GO:0005622,GO:0003735	AT5G58420.1		Ribosomal protein S4 (RPS4A) family protein	40S ribosomal protein S4
Sapur.018G090100.1	651.7485347	-0.691364739	0.065903929	-10.4905	9.55E-26	3.26E-25	black	-0.668435175	1.12E-21	GO:0006412,GO:0005840,GO:0005622,GO:0003735	AT5G23740.1	RPS11-BETA	ribosomal protein S11-beta	40S ribosomal protein S11
Sapur.018G009700.1	314.3137674	-0.495187106	0.047230159	-10.4846	1.02E-25	3.48E-25	black	-0.649658733	3.43E-20	GO:0007264,GO:0005525	AT2G24765.1	ARF3,ARL1,ATARL1	ADP-ribosylation factor 3	ADP-ribosylation factor(Arf)/Arf-like (Arl) small GTPase family protein
Sapur.010G206200.1	451.5121758	-0.524567973	0.050051889	-10.4805	1.06E-25	3.63E-25	black	-0.666129355	1.73E-21		AT5G63670.1	SPT42	SPT4 homolog 2	transcription elongation factor SPT4
Sapur.017G055900.1	16.35627471	-0.934797085	0.0896103	-10.4318	1.77E-25	6.03E-25	black	-0.659676511	5.71E-21					NA
Sapur.017G095400.1	2172.464883	-0.645084455	0.061963358	-10.4107	2.21E-25	7.51E-25	black	-0.668643032	1.08E-21	GO:0006412,GO:0005840,GO:0005622,GO:0003735	AT3G02560.2		Ribosomal protein S7e family protein	40S ribosomal protein S7, putative
Sapur.012G012800.1	254.4412947	-0.559261998	0.053771496	-10.4007	2.46E-25	8.33E-25	black	-0.644412061	8.56E-20		AT4G22000.1			hypothetical protein
Sapur.009G127700.1	358.4041025	0.3806862	0.036670464	10.38128	3.02E-25	1.02E-24	black	0.665193775	2.06E-21	GO:0030127,GO:0008270,GO:0006888,GO:0006886	AT2G27460.1		sec23/sec24 transport family protein	Sec23/Sec24 transport family protein, putative
Sapur.013G035600.1	3314.032826	-0.657804212	0.063384121	-10.3781	3.12E-25	1.05E-24	black	-0.676808679	2.25E-22	GO:0006412,GO:0005840,GO:0005622,GO:0003735	AT5G28060.1		Ribosomal protein S24e family protein	40S ribosomal protein S24
Sapur.005G119400.1	513.4436956	-0.539691112	0.052203292	-10.3383	4.73E-25	1.59E-24	black	-0.653201675	1.83E-20		AT4G38100.1			NA
Sapur.013G047300.2	61.18548508	1.030397923	0.099689112	10.33611	4.84E-25	1.63E-24	black	0.579540943	1.83E-15		AT3G04240.1	SEC	Tetratricopeptide repeat (TPR)-like superfamily protein	UDP-N-acetylglucosamine-peptide N-acetylglucosaminyltransferase, putative
Sapur.010G122400.1	97.96726138	-0.676775322	0.065511021	-10.3307	5.12E-25	1.72E-24	black	-0.604773293	4.95E-17		AT1G26460.1		Tetratricopeptide repeat (TPR)-like superfamily protein	PPR containing plant protein
Sapur.014G097100.1	348.174452	-0.615186197	0.05954975	-10.3306	5.12E-25	1.72E-24	black	-0.663406571	2.88E-21		AT1G47820.2			NA
Sapur.016G071400.1	1856.512017	-0.653752994	0.063322664	-10.3242	5.48E-25	1.84E-24	black	-0.690337775	1.50E-23		AT3G11500.1		Small nuclear ribonucleoprotein family protein	small nuclear ribonucleoprotein G, putative
Sapur.010G087600.1	120.688656	-0.60586338	0.058695692	-10.3221	5.60E-25	1.88E-24	black	-0.630113208	9.44E-19	GO:0016887,GO:0005524,GO:0016020	AT2G01320.2		ABC-2 type transporter family protein	ABC transporter family protein
Sapur.014G119900.1	276.2893421	-0.689694406	0.066970772	-10.2984	7.16E-25	2.40E-24	black	-0.630528151	8.82E-19		AT1G60710.1	ATB2	NAD(P)-linked oxidoreductase superfamily protein	aldo/keto reductase family protein
Sapur.017G039700.1	121.403989	-0.654035752	0.063563227	-10.2895	7.86E-25	2.63E-24	black	-0.636818033	3.11E-19	GO:0003676	AT5G65260.1		RNA-binding (RRM/RBD/RNP motifs) family protein	polyadenylate-binding family protein
Sapur.013G114000.2	30.04417937	-0.737994322	0.071744525	-10.2864	8.11E-25	2.72E-24	black	-0.352704109	5.88E-06	GO:0015031,GO:0006457	AT2G30695.1			hypothetical protein
Sapur.008G126500.1	5609.582277	-0.475322906	0.046235139	-10.2806	8.62E-25	2.89E-24	black	-0.643776896	9.55E-20	GO:0006412,GO:0005840,GO:0005622,GO:0003735	AT4G34670.1		Ribosomal protein S3Ae	NA
Sapur.018G031300.1	40.8184668	-0.663538469	0.064622432	-10.2679	9.83E-25	3.28E-24	black	-0.610738336	2.01E-17		AT3G10740.1	ARAF,ARAF1,ASD1,ATASD1	alpha-L-arabinofuranosidase 1	NA
Sapur.002G161900.1	459.4355673	-0.333649091	0.032505079	-10.2645	1.02E-24	3.40E-24	black	-0.608938781	2.65E-17		AT3G54170.1	ATFIP37,FIP37	FKBP12 interacting protein 37	FKBP12-interacting protein of 37 kDa protein
Sapur.006G112400.1	1339.924606	-0.374521512	0.036636954	-10.2225	1.57E-24	5.22E-24	black	-0.642208496	1.25E-19	GO:0005852,GO:0005737,GO:0003743	AT4G33250.1	ATTIF3K1,EIF3K,TIF3K1	eukaryotic translation initiation factor 3K	eukaryotic translation initiation factor 3 subunit K
Sapur.006G159800.1	866.9936438	-0.794714512	0.077791841	-10.2159	1.68E-24	5.59E-24	black	-0.622468482	3.24E-18	GO:0006412,GO:0005840,GO:0005622,GO:0003735	AT1G09590.1		Translation protein SH3-like family protein	60S ribosomal protein L21
Sapur.006G017200.1	1225.913012	-0.761260855	0.074588952	-10.2061	1.86E-24	6.18E-24	black	-0.661547834	4.05E-21	GO:0006412,GO:0005840,GO:0005622,GO:0003735	AT4G15000.1		Ribosomal L27e protein family	60S ribosomal protein L27
Sapur.006G072100.1	1093.263553	-0.659343488	0.06472504	-10.1868	2.27E-24	7.51E-24	black	-0.668782992	1.05E-21		AT5G02160.1			transmembrane protein, putative
Sapur.15WG131300.1	394.6799021	-0.611680952	0.060064708	-10.1837	2.34E-24	7.76E-24	black	-0.641674336	1.37E-19	GO:0016192,GO:0016021	AT1G04760.1	ATVAMP726,VAMP726	vesicle-associated membrane protein 726	vesicle-associated membrane protein, putative
Sapur.003G066400.1	236.4140065	-0.428172577	0.042047994	-10.1829	2.36E-24	7.82E-24	black	-0.646633549	5.83E-20	GO:0006412,GO:0005840,GO:0005622,GO:0003735	AT5G45590.1		Ribosomal protein L35	50S ribosomal protein L35
Sapur.013G134100.1	218.103368	-0.547183344	0.05398392	-10.136	3.82E-24	1.26E-23	black	-0.661289888	4.25E-21	GO:0016226,GO:0008199	AT4G03240.1	ATFH,FH	frataxin homolog	iron donor protein CyaY
Sapur.018G013900.1	405.5038561	-0.502374008	0.049600155	-10.1285	4.13E-24	1.36E-23	black	-0.669012333	1.01E-21	GO:0008152,GO:0003824	AT4G31810.1		ATP-dependent caseinolytic (Clp) protease/crotonase family protein	enoyl-CoA hydratase
Sapur.005G064400.1	1171.223186	-0.703624842	0.069602769	-10.1092	5.03E-24	1.65E-23	black	-0.657074022	9.16E-21	GO:0015934,GO:0006412,GO:0003735	AT3G49910.1		Translation protein SH3-like family protein	60S ribosomal protein L26
Sapur.018G030300.1	1648.218108	-0.722981428	0.071553361	-10.1041	5.30E-24	1.74E-23	black	-0.651159737	2.64E-20	GO:0006412,GO:0005840,GO:0005622,GO:0003735	AT1G15250.1		Zinc-binding ribosomal protein family protein	60S ribosomal protein L37, putative
Sapur.018G102400.1	628.9317772	-0.595395477	0.058956666	-10.0989	5.59E-24	1.83E-23	black	-0.685560956	3.97E-23	GO:0006412,GO:0005840,GO:0005622,GO:0003735	AT5G20180.2		Ribosomal protein L36	ribosomal protein L36
Sapur.001G021500.1	1023.315339	-0.720676079	0.071440655	-10.0878	6.26E-24	2.05E-23	black	-0.653864456	1.63E-20	GO:0006412,GO:0005840,GO:0003735	AT2G47110.2	UBQ6	ubiquitin 6	NA
Sapur.014G002000.1	1065.761375	-0.590047765	0.058524456	-10.0821	6.63E-24	2.17E-23	black	-0.680203334	1.16E-22	GO:0055085,GO:0005741	AT3G20000.1	TOM40	translocase of the outer mitochondrial membrane 40	import receptor subunit TOM40
Sapur.016G176400.1	2127.307037	-0.627403835	0.062276364	-10.0745	7.16E-24	2.34E-23	black	-0.638669358	2.28E-19	GO:0006412,GO:0005840,GO:0005622,GO:0003735	AT4G33865.1		Ribosomal protein S14p/S29e family protein	40S ribosomal protein S29
Sapur.008G131700.1	105.9705526	-0.801463333	0.079589405	-10.07	7.50E-24	2.45E-23	black	-0.652465861	2.09E-20	GO:0006412,GO:0005840,GO:0003735	AT5G09510.1		Ribosomal protein S19 family protein	40S ribosomal protein S15
Sapur.007G031900.1	921.4915339	-0.514486605	0.051132179	-10.0619	8.14E-24	2.65E-23	black	-0.619000597	5.60E-18		AT2G23670.1	YCF37	homolog of Synechocystis YCF37	hypothetical protein
Sapur.010G060800.1	1292.170587	-0.641566739	0.063880842	-10.0432	9.84E-24	3.20E-23	black	-0.646535613	5.93E-20	GO:0006412,GO:0005840,GO:0005622,GO:0003735	AT5G28060.1		Ribosomal protein S24e family protein	40S ribosomal protein S24
Sapur.002G157300.1	590.3362394	-0.713736539	0.071159349	-10.0301	1.12E-23	3.65E-23	black	-0.641811058	1.34E-19	GO:0006412,GO:0005840,GO:0005622,GO:0003735	AT3G05560.1		Ribosomal L22e protein family	60S ribosomal protein L22, putative
Sapur.018G113100.1	1056.940835	-0.604719583	0.060520375	-9.992	1.65E-23	5.34E-23	black	-0.633692328	5.24E-19	GO:0006412,GO:0005840,GO:0003735	AT2G37190.1		Ribosomal protein L11 family protein	60S ribosomal protein L12
Sapur.017G042900.1	4458.852336	-0.479002507	0.048087369	-9.96109	2.26E-23	7.27E-23	black	-0.653256954	1.82E-20	GO:0006412,GO:0005840,GO:0003735,GO:0015934	AT3G07110.1		Ribosomal protein L13 family protein	60S ribosomal protein L13a
Sapur.15WG111800.1	2498.595332	-0.608086067	0.061082911	-9.95509	2.40E-23	7.71E-23	black	-0.66334297	2.91E-21	GO:0006412,GO:0005840,GO:0003735,GO:0015934	AT1G67430.1		Ribosomal protein L22p/L17e family protein	60S ribosomal protein L17
Sapur.017G122400.1	2683.539236	-0.545176418	0.054822935	-9.94431	2.67E-23	8.59E-23	black	-0.662574245	3.36E-21		AT4G25740.1		RNA binding Plectin/S10 domain-containing protein	plectin/S10 domain protein
Sapur.006G118300.1	793.59074	0.429329518	0.043214404	9.934871	2.94E-23	9.43E-23	black	0.629866749	9.82E-19		AT5G35560.1		DENN (AEX-3) domain-containing protein	DENN (AEX-3) domain protein
Sapur.T050900.1	145.8319192	-0.803869389	0.081021911	-9.92163	3.35E-23	1.08E-22	black	-0.632348754	6.54E-19	GO:0006886,GO:0005741	AT5G43970.1	ATTOM22-V,TOM22-V,TOM	translocase of outer membrane 22-V	import receptor subunit Tom22
Sapur.014G063300.1	113.7671631	-1.025559138	0.10348164	-9.91054	3.75E-23	1.20E-22	black	-0.628709601	1.19E-18		AT2G45860.1			hypothetical protein
Sapur.011G045000.1	38.68698227	0.652871396	0.065960574	9.897904	4.25E-23	1.36E-22	black	0.618547049	6.01E-18					NA
Sapur.005G207400.1	1951.910461	0.417041449	0.042198778	9.882785	4.94E-23	1.58E-22	black	0.646927951	5.54E-20	GO:0005634,GO:0003677	AT1G20980.1	ATSPL14,FBR6,SPL14,SPL1R	squamosa promoter binding protein-like 14	squamosa promoter-binding-like protein
Sapur.007G094800.1	1920.223297	-0.616673267	0.062454371	-9.87398	5.40E-23	1.72E-22	black	-0.65321258	1.83E-20	GO:0042651,GO:0015979,GO:0009522,GO:0005516	AT5G64040.1	PSAN	photosystem I reaction center subunit PSI-N, chloroplast, putative / PSI-N, putative (PSAN)	photosystem I reaction center subunit N
Sapur.006G102200.1	862.9534438	-0.330560588	0.033485063	-9.87188	5.51E-23	1.76E-22	black	-0.640998037	1.54E-19		AT2G36930.1		zinc finger (C2H2 type) family protein	zinc finger-like protein
Sapur.016G194900.1	1811.406996	-0.732918658	0.074292373	-9.86533	5.88E-23	1.88E-22	black	-0.662668101	3.30E-21	GO:0003676	AT4G13850.1	ATGRP2,GR-RBP2,GRP2	glycine-rich RNA-binding protein 2	RNA-binding protein 42 (RBM42)-like RNA recognition motif protein
Sapur.15WG118200.1	405.9687826	-0.793322686	0.080426754	-9.86392	5.97E-23	1.90E-22	black	-0.682940053	6.72E-23		AT5G50810.1	TIM8	translocase inner membrane subunit 8	import inner membrane translocase subunit Tim8
Sapur.006G140900.1	486.2305572	-0.648954	0.065992057	-9.83382	8.05E-23	2.56E-22	black	-0.67438004	3.61E-22	GO:0005681,GO:0000398	AT2G18740.1		Small nuclear ribonucleoprotein family protein	small nuclear ribonucleoprotein E
Sapur.002G077600.1	176.7267475	-0.615790158	0.062637451	-9.83102	8.28E-23	2.63E-22	black	-0.619053425	5.56E-18		AT1G78190.1		Trm112p-like protein	TRM112-like protein
Sapur.15WG139000.1	3882.000356	-0.38555125	0.039228326	-9.82839	8.50E-23	2.70E-22	black	-0.63858073	2.31E-19	GO:0006412,GO:0005840,GO:0005622,GO:0003735	AT4G00100.1	ATRPS13A,PFL2,RPS13,RPS1	ribosomal protein S13A	40S ribosomal protein S13
Sapur.15WG122100.1	1078.346237	-0.773631233	0.079341118	-9.7507	1.83E-22	5.78E-22	black	-0.640783728	1.59E-19	GO:0006412,GO:0005840,GO:0005622,GO:0003735	AT1G26880.1		Ribosomal protein L34e superfamily protein	60S ribosomal protein L34
Sapur.005G202500.1	8015.184289	-0.586814787	0.060303632	-9.731	2.22E-22	7.00E-22	black	-0.62895044	1.14E-18		AT1G20693.2	HMG BETA 1,HMGB2,NFD0	high mobility group B2	HMG (high mobility group) box protein
Sapur.008G035000.1	657.3234098	-0.369740374	0.038005143	-9.72869	2.27E-22	7.16E-22	black	-0.6404606	1.68E-19		AT3G55005.1	TON1B	tonneau 1b (TON1b)	FGFR1 oncogene partner
Sapur.016G292700.1	457.3959117	-0.506037906	0.052047994	-9.72252	2.42E-22	7.60E-22	black	-0.652148499	2.21E-20		AT1G73940.1			hypothetical protein
Sapur.006G136300.1	49.69301651	-0.668614677	0.068773377	-9.722	2.43E-22	7.64E-22	black	-0.596808727	1.60E-16		AT2G18940.1		Tetratricopeptide repeat (TPR)-like superfamily protein	PPR containing plant protein
Sapur.001G065800.1	119.8577884	-0.429673866	0.044399867	-9.67737	3.76E-22	1.18E-21	black	-0.602699818	6.75E-17		AT5G16950.1			histone acetyltransferase type B subunit, putative
Sapur.006G040000.1	2923.686206	-0.422721301	0.043883097	-9.6329	5.81E-22	1.81E-21	black	-0.642497406	1.19E-19	GO:0006412,GO:0005840,GO:0003735,GO:0003723	AT3G57490.1		Ribosomal protein S5 family protein	40S ribosomal protein S2
Sapur.007G051400.1	9.848316207	-1.163592247	0.120852654	-9.62819	6.08E-22	1.89E-21	black	-0.639892404	1.85E-19	GO:0015934,GO:0006412,GO:0003735	AT3G49910.1		Translation protein SH3-like family protein	60S ribosomal protein L26
Sapur.012G111500.1	2.353797478	-1.879971167	0.195510498	-9.6157	6.86E-22	2.14E-21	black	-0.613759759	1.27E-17		AT3G58660.1		Ribosomal protein L1p/L10e family	ribosomal protein .1/L10e family protein
Sapur.003G012900.3	6.672194093	1.347297495	0.140744078	9.572676	1.04E-21	3.23E-21	black	0.511470789	7.63E-12		AT1G68140.1		Protein of unknown function (DUF1644)	DUF1644 family protein
Sapur.001G054200.1	2832.201226	-0.545022709	0.056938553	-9.57212	1.05E-21	3.25E-21	black	-0.639490156	1.98E-19	GO:0006412,GO:0005840,GO:0005622,GO:0003735	AT3G61110.1	ARS27A,RS27A	ribosomal protein S27	40S ribosomal protein S27
Sapur.009G024100.1	2408.249047	-0.600700732	0.06282154	-9.56202	1.15E-21	3.58E-21	black	-0.646507228	5.96E-20		AT5G57290.1		60S acidic ribosomal protein family	NA
Sapur.002G114400.1	1834.266734	-0.569006494	0.059526916	-9.55881	1.19E-21	3.69E-21	black	-0.607507934	3.29E-17	GO:0006412,GO:0005840,GO:0005622,GO:0003735	AT3G10950.1		Zinc-binding ribosomal protein family protein	60S ribosomal protein L37a
Sapur.006G188500.1	486.8034506	-0.579899493	0.060691425	-9.55488	1.24E-21	3.83E-21	black	-0.586980128	6.52E-16		AT5G60340.1		P-loop containing nucleoside triphosphate hydrolases superfamily protein	adenylate kinase
Sapur.013G101100.1	695.8369807	-0.53108668	0.055616478	-9.54909	1.31E-21	4.04E-21	black	-0.6270289	1.56E-18	GO:0006412,GO:0005840,GO:0003735	AT4G16720.1		Ribosomal protein L23/L15e family protein	60S ribosomal protein L15
Sapur.005G016400.1	108.7693429	-0.700624992	0.073403984	-9.54478	1.36E-21	4.21E-21	black	-0.615587206	9.55E-18	GO:0004857				NA

Sapur.014G033700.1	1213.895414	-0.410864309	0.043083308	-9.53651	1.48E-21	4.56E-21	black	-0.604809776	4.93E-17	GO:0006457,GO:0005737	AT3G60210.1		GroES-like family protein	chaperonin 10
Sapur.017G073500.1	2125.059786	0.329472765	0.034727572	9.487354	2.37E-21	7.28E-21	black	0.638705474	2.26E-19	GO:0016567,GO:0004842,GO:0034450,GO:0006511,GO:0000151	AT5G15400.1		U-box domain-containing protein	ubiquitin conjugation factor E4, putative
Sapur.15WG145700.1	78.69391213	-0.791282662	0.083485257	-9.47811	2.59E-21	7.94E-21	black	-0.583924057	1.00E-15	GO:0006351,GO:0003899,GO:0003677	AT5G51940.1	NRPB6A,NRPD6A,NRPE6A	RNA polymerase Rpb6	DNA-directed RNA polymerase
Sapur.005G058700.1	169.4624186	-0.53852232	0.056828086	-9.47634	2.63E-21	8.08E-21	black	-0.615880077	9.12E-18	GO:0006412,GO:0005840,GO:0003735	AT4G39880.1		Ribosomal protein L23/L15e family protein	ribosomal protein L23
Sapur.018G006000.1	246.8441657	-0.591274283	0.062473222	-9.46444	2.95E-21	9.04E-21	black	-0.670615119	7.43E-22	GO:0006412,GO:0005840,GO:0005622,GO:0003735	AT4G31460.1		Ribosomal L28 family	ribosomal protein L28
Sapur.017G115500.1	2941.689467	-0.50509464	0.053499237	-9.44116	3.69E-21	1.13E-20	black	-0.608647946	2.77E-17	GO:0006412,GO:0005840,GO:0005622,GO:0003735	AT3G16780.1		Ribosomal protein L19e family protein	ribosomal protein L19
Sapur.001G170100.1	174.4053914	-0.548532775	0.058171913	-9.42951	4.12E-21	1.26E-20	black	-0.600238142	9.70E-17					transmembrane protein, putative
Sapur.016G157200.1	3174.048651	-0.519214399	0.055220884	-9.4025	5.33E-21	1.62E-20	black	-0.644046184	9.11E-20	GO:0006412,GO:0005840,GO:0005622,GO:0003735	AT1G74050.1		Ribosomal protein L6 family protein	60S ribosomal protein L6
Sapur.018G051600.1	127.9086497	0.593787059	0.063209631	9.393933	5.78E-21	1.76E-20	black	0.662989814	3.11E-21					NA
Sapur.019G091000.1	94.63106115	-0.795821161	0.084736297	-9.39174	5.90E-21	1.79E-20	black	-0.652007571	2.27E-20		AT2G43780.2			transmembrane protein, putative
Sapur.008G037600.1	982.5274098	-0.687001126	0.073173815	-9.38862	6.08E-21	1.85E-20	black	-0.610373545	2.13E-17	GO:0006412,GO:0005840,GO:0005622,GO:0003735	AT2G39390.1		Ribosomal L29 family protein	60S ribosomal protein L35
Sapur.010G203400.1	1920.477083	-0.60869028	0.064888591	-9.38054	6.56E-21	1.99E-20	black	-0.644532059	8.38E-20	GO:0006412,GO:0005840,GO:0005622,GO:0003735	AT3G23390.1		Zinc-binding ribosomal protein family protein	60S ribosomal protein L44
Sapur.003G005500.1	810.5258419	-0.604099814	0.064399334	-9.38053	6.56E-21	1.99E-20	black	-0.632058941	6.86E-19		AT5G57290.1		60S acidic ribosomal protein family	60S acidic ribosomal protein P3, putative
Sapur.009G099000.1	117.7159507	-0.685564776	0.073188977	-9.36705	7.46E-21	2.26E-20	black	-0.599595894	1.07E-16		AT2G21290.1			30S ribosomal protein S31, putative
Sapur.006G051500.1	54.60848962	0.93511643	0.099883835	9.36204	7.82E-21	2.37E-20	black	0.571389924	5.50E-15	GO:0016157,GO:0005985	AT5G20280.1	ATSPS1F,SPS1F	sucrose phosphate synthase 1F	sucrose phosphate synthase
Sapur.004G164100.1	293.2721432	-0.384951676	0.041239137	-9.33462	1.01E-20	3.06E-20	black	-0.617112902	7.53E-18	GO:0006412,GO:0005840,GO:0005622,GO:0003735	AT5G56940.1		Ribosomal protein S16 family protein	30S ribosomal protein S16
Sapur.005G168300.1	794.4520761	-0.397981389	0.042710967	-9.31801	1.19E-20	3.57E-20	black	-0.612687512	1.49E-17	GO:0006468,GO:0005524,GO:0004672,GO:0004674	AT5G26110.1		Protein kinase superfamily protein	Kae1-associated kinase Bud32
Sapur.004G062500.1	152.0114719	-0.661997481	0.071087215	-9.31247	1.25E-20	3.76E-20	black	-0.586735744	6.75E-16		AT4G09460.1	AtMYB6,MYB6	myb domain protein 6	MYB-like transcription factor 6, putative
Sapur.008G025000.2	104.7097982	-0.739628954	0.079735267	-9.27606	1.76E-20	5.28E-20	black	-0.559293081	2.67E-14		AT3G10380.1	ATSEC8,SEC8	subunit of exocyst complex 8	SEC8 exocyst complex component specific domain protein
Sapur.002G070600.1	854.0477403	0.428248654	0.046200694	9.269312	1.87E-20	5.62E-20	black	0.582113156	1.28E-15	GO:0046488,GO:0016307	AT1G77740.1	PIP5K2	phosphatidylinositol-4-phosphate 5-kinase 2	phosphatidylinositol-4-phosphate 5-kinase
Sapur.011G097400.1	1542.039193	-0.373611222	0.040336939	-9.26226	2.00E-20	6.00E-20	black	-0.569335006	7.23E-15	GO:0003677	AT1G51060.1	HTA10	histone H2A 10	histone H2A
Sapur.011G084100.1	450.4393108	-0.310901381	0.033628879	-9.24507	2.35E-20	7.03E-20	black	-0.58495488	8.66E-16		AT5G53800.1			hypothetical protein
Sapur.003G104500.1	163.2353618	-0.782645714	0.084854655	-9.22337	2.88E-20	8.59E-20	black	-0.629307659	1.08E-18		AT5G42070.1			hypothetical protein
Sapur.004G065300.1	1166.798986	-0.258820827	0.028132053	-9.20021	3.57E-20	1.06E-19	black	-0.576298169	2.85E-15		AT1G66670.1	CLPP3,NCLPP3	CLP protease proteolytic subunit 3	ATP-dependent Clp protease proteolytic subunit
Sapur.013G012700.1	3369.097671	-0.474695117	0.05163918	-9.19254	3.84E-20	1.14E-19	black	-0.603514869	5.98E-17	GO:0006412,GO:0005840,GO:0005622,GO:0003735	AT3G05590.1	RPL18	ribosomal protein L18	60S ribosomal protein L18
Sapur.008G062300.1	707.0321515	-0.508441039	0.055509261	-9.15957	5.21E-20	1.54E-19	black	-0.644432033	8.53E-20		AT2G43810.1		Small nuclear ribonucleoprotein family protein	U6 snRNA-associated-like-Smprotein
Sapur.003G084000.1	617.1365769	-0.487700043	0.053255461	-9.15775	5.30E-20	1.57E-19	black	-0.611101279	1.90E-17		AT2G45640.1	ATSAP18,SAP18	SIN3 associated polypeptide P18	histone deacetylase complex subunit SAP18
Sapur.002G183100.1	722.6595936	-0.466605115	0.050953664	-9.15744	5.31E-20	1.57E-19	black	-0.577054561	2.57E-15		AT2G31410.1			hypothetical protein
Sapur.016G081700.1	3051.45034	-0.551418376	0.060310597	-9.1429	6.08E-20	1.80E-19	black	-0.628222953	1.28E-18	GO:0006412,GO:0005840,GO:0005622,GO:0003735	AT5G02960.1		Ribosomal protein S12/S23 family protein	ribosomal protein S23 component of cytosolic 80S ribosome and 40S small subunit
Sapur.014G129100.1	512.3833674	-0.498991482	0.054735252	-9.11646	7.76E-20	2.29E-19	black	-0.621325849	3.88E-18		AT5G55160.1	ATSUMO2,SUM2,SUMO 2,S	small ubiquitin-like modifier 2	small ubiquitin-related modifier
Sapur.002G098200.1	523.4182522	0.493875578	0.054209343	9.110525	8.20E-20	2.42E-19	black	0.590607483	3.91E-16	GO:0016020,GO:0006810,GO:0005215	AT1G47240.1	ATNRAMP2,NRAMP2	NRAMP metal ion transporter 2	metal transporter NRAMP2
Sapur.012G072600.1	1323.636614	-0.47112849	0.051751525	-9.10366	8.73E-20	2.57E-19	black	-0.645319274	7.32E-20	GO:0006412,GO:0005840,GO:0003735,GO:0015934	AT1G27400.1		Ribosomal protein L22p/L17e family protein	60S ribosomal protein L17
Sapur.001G107700.1	4729.530935	-0.48509268	0.053332364	-9.09565	9.40E-20	2.76E-19	black	-0.580475854	1.61E-15	GO:0006412,GO:0005840,GO:0005622,GO:0003735	AT3G49010.2	ATBBC1,BBC1,RSU2	breast basic conserved 1	60S ribosomal protein L13
Sapur.011G045900.1	1755.402221	-0.706264413	0.077804348	-9.07744	1.11E-19	3.26E-19	black	-0.596685014	1.63E-16	GO:0006412,GO:0005840,GO:0005622,GO:0003735	AT2G37600.1		Ribosomal protein L36e family protein	60S ribosomal protein L36
Sapur.001G079300.1	891.2178512	0.54175452	0.059786633	9.061466	1.29E-19	3.77E-19	black	0.555772325	4.17E-14		AT1G64140.1			transcription factor, putative
Sapur.017G075800.1	1912.865999	-0.44243664	0.048916902	-9.04466	1.50E-19	4.39E-19	black	-0.595828881	1.85E-16	GO:0006412,GO:0005840,GO:0003735	AT5G61170.1		Ribosomal protein S19e family protein	40S ribosomal protein S19
Sapur.016G218900.1	3519.160433	-0.457004229	0.050557382	-9.03932	1.58E-19	4.61E-19	black	-0.554064955	5.17E-14	GO:0006412,GO:0003735	AT3G27830.1	RPL12,RPL12-A	ribosomal protein L12-A	50S ribosomal protein L12
Sapur.016G212600.1	323.9650003	-0.349790199	0.038753355	-9.02606	1.78E-19	5.20E-19	black	-0.62121732	3.95E-18	GO:0043461	AT5G40660.1		ATP12 protein-related	ATP synthase F1 complex assembly factor 2
Sapur.006G062600.1	2264.348414	-0.584550575	0.064917161	-9.00456	2.17E-19	6.31E-19	black	-0.615587348	9.55E-18	GO:0006412,GO:0005840,GO:0003735	AT2G37190.1		Ribosomal protein L11 family protein	60S ribosomal protein L12
Sapur.009G029300.1	413.6473119	-0.564054684	0.062782404	-8.98428	2.60E-19	7.58E-19	black	-0.586919569	6.58E-16	GO:0045039	AT2G29530.2	TIM10	Tim10/DDP family zinc finger protein	import inner membrane translocase subunit Tim10
Sapur.010G024700.1	926.6227429	-0.530127287	0.059027126	-8.98108	2.68E-19	7.80E-19	black	-0.597669527	1.41E-16		AT1G70600.1		Ribosomal protein L18e/L15 superfamily protein	60S ribosomal protein L27a
Sapur.014G119500.1	1469.987897	-0.552692529	0.061558985	-8.97826	2.75E-19	8.00E-19	black	-0.615717903	9.35E-18	GO:0006412,GO:0005840,GO:0005622,GO:0003735	AT4G29390.1		Ribosomal protein S30 family protein	40S ribosomal protein S30
Sapur.15WG148100.1	1370.401676	-0.54664015	0.060986593	-8.96328	3.15E-19	9.15E-19	black	-0.589481261	4.58E-16		AT2G36620.1	RPL24A	ribosomal protein L24	60S ribosomal protein L24
Sapur.016G087000.1	693.5803622	-0.514116366	0.057369668	-8.96147	3.20E-19	9.30E-19	black	-0.601890721	7.60E-17		AT3G09860.1			hypothetical protein
Sapur.007G045400.2	14.54573035	-1.19530103	0.133728222	-8.93829	3.95E-19	1.14E-18	black	-0.556493437	3.81E-14		AT5G67240.1	SDN3	small RNA degrading nuclease 3	small RNA degrading nuclease
Sapur.018G051300.1	3270.632788	-0.578799687	0.064796844	-8.93253	4.16E-19	1.20E-18	black	-0.597651689	1.42E-16	GO:0006412,GO:0005840,GO:0005622,GO:0003735	AT2G19740.1		Ribosomal protein L31e family protein	60S ribosomal protein L31
Sapur.003G131800.1	549.0712833	-0.27913092	0.031292625	-8.92002	4.66E-19	1.35E-18	black	-0.569356724	7.21E-15	GO:0043565,GO:0008270,GO:0006355,GO:0003700	AT3G24050.1	GATA1	GATA transcription factor 1	GATA transcription factor
Sapur.017G093700.1	373.2019397	-0.670686759	0.075245795	-8.91328	4.95E-19	1.43E-18	black	-0.631403245	7.64E-19		AT5G16060.1		Cytochrome c oxidase biogenesis protein Cmc1-like	cytochrome C oxidase biogenesis Cmc1-like protein
Sapur.014G013500.1	37.76016619	-0.679881463	0.076322498	-8.90801	5.20E-19	1.50E-18	black	-0.581637916	1.37E-15		AT3G46630.1		Protein of unknown function (DUF3223)	DCL protein, putative
Sapur.014G046400.1	645.8181947	-0.215500362	0.024210563	-8.90109	5.53E-19	1.59E-18	black	-0.547571632	1.16E-13	GO:0005789	AT2G45140.1	PVA12	plant VAP homolog 12	vesicle-associated membrane protein, putative
Sapur.006G201500.1	2334.865817	-0.539039129	0.060582814	-8.89756	5.71E-19	1.64E-18	black	-0.585803995	7.69E-16	GO:0006412,GO:0005840,GO:0005622,GO:0003735	AT1G15250.1		Zinc-binding ribosomal protein family protein	60S ribosomal protein L37
Sapur.013G069900.1	13.88657478	-1.005567478	0.113163607	-8.88597	6.34E-19	1.82E-18	black	-0.566425704	1.06E-14	GO:0008270	AT5G17790.1	VAR3	zinc finger (Ran-binding) family protein	zinc finger protein VAR3, putative
Sapur.006G224700.1	488.9953449	1.206313256	0.136186822	8.857783	8.16E-19	2.34E-18	black	0.519592971	3.10E-12	GO:0046872,GO:0000166	AT2G24520.1	AHA5,HA5	H(+)-ATPase 5	plasma membrane H+-ATPase
Sapur.009G062500.1	308.898448	-0.474740405	0.053598214	-8.85739	8.19E-19	2.35E-18	black	-0.590935506	3.73E-16		AT5G61220.1		LYR family of Fe/S cluster biogenesis protein	catalytic/oxidoreductase, acting on NADH or NADPH protein
Sapur.008G136900.1	1190.718934	-0.629138902	0.071102703	-8.84831	8.89E-19	2.55E-18	black	-0.599131371	1.14E-16	GO:0006412,GO:0005840,GO:0005622,GO:0003735	AT4G27090.1		Ribosomal protein L14	60S ribosomal protein L14, putative
Sapur.018G082500.1	533.3485527	-0.473516387	0.05356373	-8.84024	9.55E-19	2.74E-18	black	-0.59221716	3.10E-16		AT5G57990.1	UBP23	ubiquitin-specific protease 23	NA
Sapur.016G199600.1	204.5896366	-0.509491644	0.057704102	-8.82938	1.05E-18	3.01E-18	black	-0.582724735	1.18E-15		AT1G27435.1			hypothetical protein
Sapur.013G016800.1	554.3893165	-0.556179502	0.06312567	-8.81067	1.24E-18	3.55E-18	black	-0.596585496	1.65E-16	GO:0005515	AT1G65030.1		Transducin/WD40 repeat-like superfamily protein	NA
Sapur.012G008600.1	996.5578499	-0.692162617	0.078875627	-8.77537	1.70E-18	4.85E-18	black	-0.588541706	5.23E-16		AT5G24510.1		60S acidic ribosomal protein family	60S acidic ribosomal protein P1, putative
Sapur.010G058600.1	2865.945526	-0.575563466	0.065781893	-8.74957	2.14E-18	6.08E-18	black	-0.601232257	8.38E-17	GO:0006412,GO:0005840,GO:0003735	AT5G18380.1		Ribosomal protein S5 domain 2-like superfamily protein	40S ribosomal protein S16
Sapur.004G088500.1	1295.607053	-0.429653808	0.049224075	-8.72853	2.58E-18	7.31E-18	black	-0.5809587	1.51E-15	GO:0006412	ATCG00900.1		Ribosomal protein S7p/S5e family protein	30S ribosomal protein S7
Sapur.018G068400.1	834.8067954	-0.582609369	0.06679047	-8.72294	2.71E-18	7.68E-18	black	-0.586404095	7.07E-16		AT4G30220.1	RUXF	small nuclear ribonucleoprotein F	small nuclear ribonucleoprotein F
Sapur.016G137600.1	317.2936142	-0.622678854	0.071550815	-8.70261	3.24E-18	9.17E-18	black	-0.60582691	4.23E-17	GO:0045039	AT2G29530.2	TIM10	Tim10/DDP family zinc finger protein	import inner membrane translocase subunit Tim10
Sapur.010G113900.1	860.5889963	-0.526136818	0.06060459	-8.68147	3.91E-18	1.10E-17	black	-0.594215212	2.33E-16		AT3G06610.1		DNA-binding enhancer protein-related	huntingtin-interacting protein K
Sapur.016G201300.1	83.12850742	-0.637954307	0.073540151	-8.67491	4.14E-18	1.17E-17	black	-0.556344492	3.88E-14		AT3G62870.1		Ribosomal protein L7Ae/L30e/S12e/Gadd45 family protein	NA
Sapur.001G056100.1	4145.080043	-0.430489397	0.049657067	-8.66925	4.35E-18	1.23E-17	black	-0.607427303	3.33E-17	GO:0006412,GO:0005840,GO:0005622,GO:0003735	AT1G09590.1		Translation protein SH3-like family protein	60S ribosomal protein L21
Sapur.008G038600.1	3084.729722	-0.613328595	0.070807461	-8.66192	4.64E-18	1.31E-17	black	-0.620598611	4.35E-18	GO:0006412,GO:0005840,GO:0003735	AT3G55280.1	RPL23AB	ribosomal protein L23AB	60S ribosomal protein L23a
Sapur.001G186100.1	239.5558166	-0.535470384	0.061847654	-8.65789	4.81E-18	1.35E-17	black	-0.636575426	3.24E-19		AT5G39600.1			39S ribosomal protein L53/MRP-L53
Sapur.002G193500.1	2718.063692	-0.603625288	0.069897686	-8.63584	5.83E-18	1.64E-17	black	-0.605310272	4.57E-17	GO:0006412,GO:0005840,GO:0005622,GO:0003735	AT4G18100.1		Ribosomal protein L32e	60S ribosomal protein L32
Sapur.005G095500.1	94.7595505	-0.457653455	0.053047612	-8.62722	6.29E-18	1.76E-17	black	-0.609694458	2.36E-17		AT5G49410.2			plant/F2P9-19 protein, putative
Sapur.002G189500.1	690.5627324	-0.337257846	0.03909324	-8.62701	6.30E-18	1.77E-17	black	-0.589368669	4.66E-16	GO:0005515	AT3G07170.1		Sterile alpha motif (SAM) domain-containing protein	SAM domain protein
Sapur.004G044100.1	3156.42882	-0.452510738	0.05246905	-8.62434	6.45E-18	1.81E-17	black	-0.635473368	3.89E-19	GO:0006412,GO:0005840,GO:0003735	AT2G34480.1		Ribosomal protein L18ae/LX family protein	NA
Sapur.006G034700.1	283.1314136	-0.462612898	0.053680662	-8.61787	6.82E-18	1.91E-17	black	-0.587775698	5.83E-16		AT3G57320.1			hypothetical protein
Sapur.016G125200.1	1349.256234	-0.395566656	0.04594694	-8.60921	7.36E-18	2.06E-17	black	-0.588564508	5.22E-16		AT2G44310.1		Calcium-binding EF-hand family protein	calcium-binding EF-hand protein
Sapur.003G026400.1	242.1441305	-0.496979645	0.057773244	-8.60225	7.82E-18	2.19E-17	black	-0.57385183	3.96E-15		AT3G27750.1			transmembrane protein, putative
Sapur.004G161200.1	147.0336872	-0.38759353	0.045081286	-8.59766	8.14E-18	2.28E-17	black	-0.572688296	4.63E-15	GO:0005515,GO:0043562,GO:0009744	AT2G28060.1		5\'-AMP-activated protein kinase beta-2 subunit protein	SNF1-related kinase regulatory subunit beta-2
Sapur.016G274600.1	1083.061314	-0.392655401	0.045708415	-8.59044	8.66E-18	2.42E-17	black	-0.551851264	6.83E-14	GO:0003677	AT4G27230.2	HTA2	histone H2A 2	histone H2A
Sapur.016G069600.1	785.0305172	0.456243373	0.053115599	8.589631	8.72E-18	2.44E-17	black	0.577050903	2.57E-15		AT3G11560.4		LETM1-like protein	LETM1-like protein
Sapur.010G193800.1	1012.355269	-0.531253524	0.061972138	-8.57246	1.01E-17	2.83E-17	black	-0.612515892	1.53E-17		AT4G39200.2		Ribosomal protein S25 family protein	40S ribosomal protein S25, putative
Sapur.008G014000.1	936.4964268	0.415334563	0.048579392	8.549604	1.24E-17	3.44E-17	black	0.587232805	6.29E-16		AT5G22450.1			hypothetical protein
Sapur.008G033400.1	1220.515371	-0.462437447	0.054242959	-8.5253	1.52E-17	4.23E-17	black	-0.637487919	2.78E-19	GO:0006412,GO:0005840,GO:0005622,GO:0003735	AT5G02960.1		Ribosomal protein S12/S23 family protein	ribosomal protein S23 component of cytosolic 80S ribosome and 40S small subunit
Sapur.15WG110300.1	1085.0066	-0.352689749	0.041423463	-8.51425	1.68E-17	4.64E-17	black	-0.58718074	6.34E-16	GO:0004017,GO:0019205,GO:0006139,GO:0005524	AT5G63400.1	ADK1	adenylate kinase 1	adenylate kinase
Sapur.005G174800.1	3745.850972	-0.462954889	0.054509958	-8.49303	2.01E-17	5.57E-17	black	-0.59394869	2.42E-16	GO:0006412,GO:0005840,GO:0003735	AT5G09500.1		Ribosomal protein S19 family protein	NA
Sapur.004G001400.1	11.78180659	-0.76913025	0.090571905	-8.49193	2.03E-17	5.62E-17	black	-0.574034903	3.86E-15		AT1G05000.1		Phosphotyrosine protein phosphatases superfamily protein	NA
Sapur.008G139400.1	1701.861093	-0.564861129	0.066638262	-8.47653	2.32E-17	6.41E-17	black	-0.60309392	6.36E-17	GO:0006412,GO:0005840,GO:0003735	AT2G33370.1		Ribosomal protein L14p/L23e family protein	60S ribosomal protein L23
Sapur.012G017400.1	123.8723256	-0.560438078	0.066146405	-8.47269	2.40E-17	6.62E-17	black	-0.594241105	2.32E-16		AT4G29660.1	EMB2752	embryo defective 2752	transmembrane protein, putative
Sapur.006G153900.1	1460.420925	-0.595034823	0.070300691	-8.46414	2.58E-17	7.11E-17	black	-0.581387945	1.42E-15	GO:0006412,GO:0005840,GO:0005622,GO:0003735	AT4G31985.1		Ribosomal protein L39 family protein	NA
Sapur.001G011700.1	396.4283799	-0.364170915	0.043056228	-8.45803	2.72E-17	7.49E-17	black	-0.62134219	3.87E-18		AT3G57930.1			metal tolerance protein, putative
Sapur.009G075400.1	114.9055817	-0.364399248	0.04317815	-8.43944	3.19E-17	8.76E-17	black	-0.546991974	1.25E-13		AT2G15270.1			DUF1168 family protein
Sapur.009G049500.1	4310.149028	-0.472933561	0.056190866	-8.41656	3.88E-17	1.06E-16	black	-0.618967416	5.63E-18	GO:0006412,GO:0005840,GO:0005622,GO:0003735	AT2G19740.1		Ribosomal protein L31e family protein	60S ribosomal protein L31
Sapur.016G063900.1	1336.73716	-0.458547487	0.054715181	-8.38063	5.26E-17	1.44E-16	black	-0.581236669	1.45E-15		AT1G70600.1		Ribosomal protein L18e/L15 superfamily protein	60S ribosomal protein L27a
Sapur.016G015600.1	2178.3837	-0.531111926	0.063396495	-8.37762	5.40E-17	1.47E-16	black	-0.59795932	1.35E-16	GO:0006412,GO:0005840,GO:0005622,GO:0003735	AT4G15000.1		Ribosomal L27e protein family	60S ribosomal protein L27
Sapur.006G174400.1	933.5865873	-0.53774979	0.064189976	-8.37747	5.41E-17	1.48E-16	black	-0.572745819	4.59E-15	GO:0006412,GO:0005840,GO:0003735	AT3G55280.1	RPL23AB	ribosomal protein L23AB	60S ribosomal protein L23a
Sapur.013G033900.1	142.0444712	-0.492067915	0.058762414	-8.37385	5.58E-17	1.52E-16	black	-0.579581209	1.82E-15		AT3G04950.1			SEC-C motif protein
Sapur.010G170000.1	591.4834236	0.38526253	0.046022026	8.371264	5.70E-17	1.55E-16	black	0.569425171	7.14E-15		AT5G13590.1			hypothetical protein
Sapur.002G191100.1	1269.056089	-0.515284666	0.061641711	-8.35935	6.31E-17	1.72E-16	black	-0.580430736	1.62E-15		AT3G07230.1		wound-responsive protein-related	NA
Sapur.002G188000.2	7.180068005	-0.933217403	0.111671928	-8.35678	6.45E-17	1.75E-16	black	-0.45454446	2.23E-09	GO:0006412,GO:0005840,GO:0003735,GO:0015934	AT5G48760.1		Ribosomal protein L13 family protein	60S ribosomal protein L13a
Sapur.004G044800.1	472.9765557	0.652107295	0.078111012	8.348468	6.92E-17	1.88E-16	black	0.552399212	6.38E-14	GO:0046983,GO:0003677,GO:0006355,GO:0005634,GO:0003700	AT4G18960.1	AG	K-box region and MADS-box transcription factor family protein	MADS-box transcription factor
Sapur.016G208500.1	2.965988264	-1.346937293	0.161541166	-8.33804	7.55E-17	2.05E-16	black	-0.105235404	0.189619982		AT4G17310.1			hypothetical protein
Sapur.016G113900.1	1149.773845	0.477875619	0.05731359	8.337911	7.56E-17	2.05E-16	black	0.568548202	8.02E-15	GO:0005515,GO:0016787	AT1G08420.1	BSL2	BRI1 suppressor 1 (BSU1)-like 2	serine/threonine-protein phosphatase
Sapur.009G061600.1	1653.864852	0.412603365	0.049553738	8.326382	8.34E-17	2.26E-16	black	0.570370337	6.30E-15	GO:0055114,GO:0016491,GO:0008152	AT2G14170.1	ALDH6B2	aldehyde dehydrogenase 6B2	methylmalonate-semialdehyde dehydrogenase (acylating)
Sapur.15WG120800.1	12497.01906	0.332459401	0.040003055	8.31085	9.50E-17	2.57E-16	black	0.564752142	1.32E-14	GO:0005515,GO:0016887,GO:0005524,GO:0019538	AT5G50920.1	ATHSP93-V,CLPC,CLPC1,DC	CLPC homologue 1	ATP-dependent Clp protease ATP-binding subunit clpA-like protein
Sapur.008G077000.1	277.4273465	-0.448141749	0.053960231	-8.30504	9.98E-17	2.70E-16	black	-0.584009216	9.88E-16	GO:0003676,GO:0006457,GO:0003755	AT1G13690.1	ATE1	ATPase E1	peptidyl-prolyl cis-trans isomerase E
Sapur.009G013300.1	1949.912912	-0.406045526	0.048935373	-8.29759	1.06E-16	2.87E-16	black	-0.571127006	5.70E-15	GO:0006412,GO:0005840,GO:0003735	AT3G52580.1		Ribosomal protein S11 family protein	40S ribosomal protein S14
Sapur.003G067700.1	1646.104593	0.649364923	0.078285698	8.294809	1.09E-16	2.94E-16	black	0.589566929	4.53E-16					NA
Sapur.011G056800.1	757.9992165	-0.498890974	0.060267658	-8.27792	1.25E-16	3.38E-16	black	-0.564324794	1.39E-14		AT1G30070.1		SGS domain-containing protein	SGS domain protein
Sapur.004G054600.1	2898.997198	-0.355015605	0.042951331	-8.26553	1.39E-16	3.75E-16	black	-0.550034767	8.56E-14	GO:0006412,GO:0005840,GO:0005622,GO:0003735	AT3G16780.1		Ribosomal protein L19e family protein	ribosomal protein L19
Sapur.003G001500.1	36.99693373	-0.579061527	0.070119519	-8.25821	1.48E-16	3.98E-16	black	-0.56595218	1.13E-14	GO:0008152,GO:0003824	AT5G23230.1	NIC2	nicotinamidase 2	isochorismatase hydrolase
Sapur.005G017200.2	36.76254651	-0.630573706	0.076394404	-8.25419	1.53E-16	4.12E-16	black	-0.385977322	5.96E-07	GO:0006412,GO:0005840,GO:0005622,GO:0003735	AT5G27850.1		Ribosomal protein L18e/L15 superfamily protein	60S ribosomal protein L18
Sapur.007G022300.1	178.0555516	-0.5818341	0.070541962	-8.24806	1.61E-16	4.33E-16	black	-0.552781918	6.08E-14	GO:0019843,GO:0006412,GO:0005840,GO:0003735	AT2G18400.1		ribosomal protein L6 family protein	ribosomal protein L6
Sapur.003G151900.3	2.355202564	-1.468298555	0.178629215	-8.21981	2.04E-16	5.46E-16	black	-0.589617545	4.50E-16		AT3G54430.1	SRS6	SHI-related sequence 6	zinc finger, LRP1 type protein, putative
Sapur.007G001100.1	291.7437824	-0.252242869	0.030711277	-8.21336	2.15E-16	5.76E-16	black	-0.548637346	1.02E-13	GO:0005515	AT2G19430.1	AtTHO6,DWA1,THO6	DWD (DDB1-binding WD40 protein) hypersensitive to ABA 1	NA
Sapur.004G114800.1	1162.429303	-0.238623637	0.029067224	-8.20937	2.22E-16	5.96E-16	black	-0.53798966	3.70E-13		AT1G11750.1	CLPP6,NCLPP1,NCLPP6	CLP protease proteolytic subunit 6	ATP-dependent Clp protease proteolytic subunit
Sapur.016G112500.1	1427.440755	0.49813545	0.060711785	8.204922	2.31E-16	6.18E-16	black	0.542258758	2.22E-13	GO:0030036,GO:0005856	AT2G38440.1	ATSCAR2,DIS3,ITB1,SCAR2,\	SCAR homolog 2	SCAR2
Sapur.011G066100.1	51.37699761	0.808072648	0.098650833	8.19124	2.59E-16	6.91E-16	black	0.517718133	3.83E-12					hypothetical protein
Sapur.008G072500.1	101.1814275	-0.488459779	0.059725105	-8.17847	2.87E-16	7.68E-16	black	-0.58330603	1.09E-15	GO:0051301,GO:0032955	AT1G69390.1	ARC12,ATMINE1,MINE1	homologue of bacterial MinE 1	cell division topological specificity factor MinE
Sapur.017G071400.1	3974.24597	0.445048775	0.054458258	8.172292	3.03E-16	8.07E-16	black	0.534279651	5.74E-13	GO:0030170	AT3G01120.1	ATCYS1,CGS,CGS1,MTO1	Pyridoxal phosphate (PLP)-dependent transferases superfamily protein	cystathionine gamma-synthase
Sapur.014G095600.1	1856.983844	-0.192415517	0.023594404	-8.15513	3.49E-16	9.29E-16	black	-0.535343181	5.06E-13		AT1G02560.1	CLPP5,NCLPP1,NCLPP5	nuclear encoded CLP protease 5	ATP-dependent Clp protease proteolytic subunit
Sapur.002G122100.1	871.8297429	-0.431818406	0.052979907	-8.15061	3.62E-16	9.63E-16	black	-0.576399227	2.81E-15	GO:0016829,GO:0008152	AT2G45440.1	DHDPS2	dihydrodipicolinate synthase	dihydrodipicolinate synthase
Sapur.004G108500.1	338.6409454	-0.45801792	0.056220384	-8.14683	3.74E-16	9.93E-16	black	-0.593755668	2.49E-16		AT1G49510.1	emb1273	embryo defective 1273	transmembrane protein, putative
Sapur.010G168700.1	2494.46039	-0.477106497	0.058608573	-8.14056	3.93E-16	1.05E-15	black	-0.569623978	6.95E-15	GO:0006412,GO:0005840,GO:0005622,GO:0003735	AT5G02610.1		Ribosomal L29 family protein	60S ribosomal protein L35
Sapur.009G050800.1	5395.488503	-0.381114606	0.047001466	-8.10857	5.12E-16	1.36E-15	black	-0.583003386	1.14E-15	GO:0006412,GO:0005840,GO:0005622,GO:0003735	AT1G74050.1		Ribosomal protein L6 family protein	60S ribosomal protein L6
Sapur.018G073800.1	870.5739949	0.353987994	0.043771265	8.097213	5.62E-16	1.49E-15	black	0.549196334	9.49E-14	GO:0019538	AT5G57710.1		Double Clp-N motif-containing P-loop nucleoside triphosphate hydrolases superfamily protein	101 kDa heat shock protein; HSP101-like protein
Sapur.016G057500.1	1831.326376	-0.527178415	0.065433336	-8.05673	7.84E-16	2.07E-15	black	-0.559517	2.59E-14	GO:0006412,GO:0005840,GO:0005622,GO:0003735	AT1G57660.1		Translation protein SH3-like family protein	60S ribosomal protein L21
Sapur.013G116800.1	10454.39803	-0.380178435	0.047328503	-8.03276	9.53E-16	2.51E-15	black	-0.558706684	2.87E-14	GO:0006412,GO:0005840,GO:0005622,GO:0003735,GO:0008097	AT3G25520.1	ATL5,OLI5,PGY3,RPL5A	ribosomal protein L5	60S ribosomal protein L5
Sapur.T091300.1	740.042577	-0.29831959	0.037143671	-8.03151	9.63E-16	2.53E-15	black	-0.534676943	5.48E-13	GO:0016020	AT5G40770.1	ATPHB3,PHB3	prohibitin 3	prohibitin
Sapur.002G195400.1	16.69861504	-0.968642761	0.120611023	-8.03113	9.66E-16	2.54E-15	black	-0.521169933	2.60E-12					NA
Sapur.010G041600.1	467.3531714	-0.426110067	0.053206602	-8.00859	1.16E-15	3.04E-15	black	-0.57186694	5.16E-15		AT3G23325.1		Splicing factor 3B subunit 5/RDS3 complex subunit 10	splicing factor 3B subunit
Sapur.014G069300.1	1686.209712	0.416234654	0.051987977	8.006364	1.18E-15	3.10E-15	black	0.55855368	2.93E-14		AT4G01290.2			hypothetical protein
Sapur.005G028800.1	628.840466	-0.334994234	0.041889774	-7.99704	1.27E-15	3.34E-15	black	-0.582690786	1.19E-15	GO:0016021,GO:0008233,GO:0006465,GO:0005787	AT5G27430.1		Signal peptidase subunit	signal peptidase subunit protein
Sapur.006G076800.3	18.90286346	-0.66579507	0.083459694	-7.97744	1.49E-15	3.91E-15	black	-0.538611893	3.43E-13	GO:0005515	AT3G09250.1		Nuclear transport factor 2 (NTF2) family protein	SnoaL-like domain protein
Sapur.010G010900.2	11.24484812	-1.041862583	0.131329021	-7.93322	2.14E-15	5.56E-15	black	-0.047848448	0.551783744	GO:0016021	AT2G42770.1		Peroxisomal membrane 22 kDa (Mpv17/PMP22) family protein	peroxisomal membrane protein, putative
Sapur.014G128300.1	23.98303451	-0.752948366	0.095040605	-7.92239	2.33E-15	6.06E-15	black	-0.597816053	1.38E-16	GO:0003676	AT3G08000.1		RNA-binding (RRM/RBD/RNP motifs) family protein	RNA recognition motif 2 in RNA-binding protein 28 (RBM28)-like protein
Sapur.007G015400.1	254.4731412	-0.511914137	0.064718622	-7.90984	2.58E-15	6.70E-15	black	-0.564608287	1.34E-14		AT3G51010.1			hypothetical protein
Sapur.006G228000.1	292.8593251	-0.429151729	0.054316337	-7.90097	2.77E-15	7.18E-15	black	-0.57508781	3.35E-15	GO:0008176,GO:0006400	AT5G24840.1		tRNA (guanine-N-7) methyltransferase	tRNA (guanine-N(7))-methyltransferase
Sapur.011G041900.1	2388.2405	-0.305176175	0.038743498	-7.87684	3.36E-15	8.70E-15	black	-0.529867529	9.61E-13		AT5G46020.1			heat- and acid-stable phosphoprotein
Sapur.005G202400.1	28.11910125	-0.741628399	0.094350086	-7.86039	3.83E-15	9.90E-15	black	-0.495894209	4.01E-11					NA
Sapur.018G107200.1	4686.783031	-0.385443947	0.049138823	-7.84397	4.37E-15	1.13E-14	black	-0.561086036	2.12E-14		AT3G13580.3		Ribosomal protein L30/L7 family protein	60S ribosomal protein L7
Sapur.001G121600.1	162.3162217	-0.422223538	0.053880249	-7.83633	4.64E-15	1.20E-14	black	-0.546727636	1.29E-13		AT3G59840.1			NA
Sapur.003G117400.1	502.1605147	-0.385635743	0.049338248	-7.81616	5.45E-15	1.40E-14	black	-0.547128631	1.22E-13		AT5G47890.1		NADH-ubiquinone oxidoreductase B8 subunit, putative	NADH dehydrogenase [ubiquinone] 1 alpha subcomplex subunit 2
Sapur.016G230100.1	46.00978955	0.607360861	0.077718416	7.81489	5.50E-15	1.42E-14	black	0.543796884	1.84E-13	GO:0016787	AT5G55260.1	PPX-2,PPX2	protein phosphatase X 2	serine/threonine-protein phosphatase
Sapur.004G060300.1	2130.818888	-0.410973984	0.052639416	-7.80734	5.84E-15	1.50E-14	black	-0.556017531	4.05E-14		AT2G36620.1	RPL24A	ribosomal protein L24	60S ribosomal protein L24
Sapur.002G194500.1	887.2167659	-0.300988776	0.038692094	-7.77908	7.31E-15	1.87E-14	black	-0.554843766	4.69E-14	GO:0016020	AT4G27990.1	ATYLMG1-2,YLMG1-2	YGGT family protein	YGGT family protein
Sapur.008G080600.1	656.0717908	-0.289169969	0.037228477	-7.76744	8.01E-15	2.05E-14	black	-0.522116227	2.34E-12	GO:0019843,GO:0006412,GO:0005840,GO:0003735	AT3G17170.1	RFC3	Translation elongation factor EF1B/ribosomal protein S6 family protein	30S ribosomal protein S6
Sapur.012G098400.1	978.1070809	-0.505676335	0.065115779	-7.7658	8.11E-15	2.08E-14	black	-0.545049163	1.58E-13	GO:0006412,GO:0005840,GO:0005622,GO:0003735	AT4G00100.1	ATRPS13A,PFL2,RPS13,RPS1	ribosomal protein S13A	40S ribosomal protein S13
Sapur.003G117800.1	955.3519464	-0.519561681	0.066925737	-7.76326	8.28E-15	2.12E-14	black	-0.540830164	2.63E-13	GO:0006412,GO:0005840,GO:0005622,GO:0003735	AT1G09590.1		Translation protein SH3-like family protein	60S ribosomal protein L21
Sapur.008G059100.1	1326.353398	-0.557463369	0.071832316	-7.76062	8.45E-15	2.16E-14	black	-0.511444471	7.65E-12		AT4G14270.1			polyadenylate-binding protein interacting protein, putative
Sapur.002G041000.2	6.24224265	-0.901332536	0.116236661	-7.75429	8.88E-15	2.27E-14	black	-0.483693804	1.39E-10	GO:0006412	AT3G63190.1	AtcpRRF,cpRRF,HFP108,RRF	ribosome recycling factor, chloroplast precursor	ribosome recycling factor
Sapur.006G000100.1	60.56363443	-0.564013481	0.072796616	-7.7478	9.35E-15	2.39E-14	black	-0.503021009	1.90E-11		AT3G52155.1		Phosphoglycerate mutase family protein	NA
Sapur.003G082900.1	206.4085138	-0.342520128	0.044244933	-7.74145	9.83E-15	2.51E-14	black	-0.523356096	2.03E-12	GO:0016787	AT4G11980.1	ATNUDT14,ATNUDX14,NUD	nudix hydrolase homolog 14	nudix hydrolase
Sapur.018G010100.1	105.4457847	-0.371855264	0.048047385	-7.73934	9.99E-15	2.55E-14	black	-0.577819881	2.31E-15		AT5G10745.1			transmembrane protein, putative
Sapur.009G108400.1	129.5032478	-0.52748812	0.068286851	-7.72459	1.12E-14	2.86E-14	black	-0.534941094	5.31E-13		AT2G20940.1		Protein of unknown function (DUF1279)	transmembrane protein, putative
Sapur.002G155900.1	257.110614	-0.390383927	0.05057068	-7.71957	1.17E-14	2.97E-14	black	-0.534759817	5.42E-13	GO:0006396	AT5G27720.1	emb1644	Small nuclear ribonucleoprotein family protein	U6 snRNA-associated-like-Smprotein LSm4, putative
Sapur.016G128700.1	1179.372835	-0.308473057	0.039973545	-7.71693	1.19E-14	3.04E-14	black	-0.589708415	4.44E-16	GO:0006412,GO:0005840,GO:0003735	AT3G55280.2	RPL23AB	ribosomal protein L23AB	60S ribosomal protein L23a
Sapur.007G048200.1	82.0157325	0.542134963	0.070304143	7.71128	1.25E-14	3.17E-14	black	0.54833654	1.06E-13		AT4G37440.1			hypothetical protein
Sapur.010G156400.1	3758.52619	-0.446356252	0.058015977	-7.69368	1.43E-14	3.63E-14	black	-0.577835014	2.31E-15	GO:0006412,GO:0005840,GO:0005622,GO:0003735	AT1G74270.1		Ribosomal protein L35Ae family protein	60S ribosomal protein L35a
Sapur.001G138400.1	1833.754021	0.514109174	0.066875024	7.687611	1.50E-14	3.81E-14	black	0.509975012	8.98E-12	GO:0046872,GO:0030001,GO:0000166	AT5G44790.1	HMA7,RAN1	copper-exporting ATPase / responsive-to-antagonist 1 / copper-transporting ATPase (RAN1)	copper-transporting ATPase P-type protein, putative
Sapur.010G172700.1	2006.843232	-0.402958669	0.052676615	-7.64967	2.01E-14	5.10E-14	black	-0.534147944	5.83E-13	GO:0006412,GO:0005840,GO:0005622,GO:0003735	AT5G02960.1		Ribosomal protein S12/S23 family protein	ribosomal protein S23 component of cytosolic 80S ribosome and 40S small subunit
Sapur.007G074400.1	213.9636807	-0.553377965	0.072778146	-7.60363	2.88E-14	7.24E-14	black	-0.531804417	7.67E-13		AT4G39470.1		Tetratricopeptide repeat (TPR)-like superfamily protein	lumen common family protein, putative
Sapur.001G134300.1	242.0769604	-0.520442085	0.06852104	-7.59536	3.07E-14	7.72E-14	black	-0.601379268	8.20E-17		AT1G53903.1		Protein of unknown function (DUF581)	DUF581 family protein
Sapur.003G008700.1	1906.497018	-0.571115564	0.075257603	-7.58881	3.23E-14	8.11E-14	black	-0.51189711	7.28E-12	GO:0051536,GO:0009055	AT1G60950.1	ATFD2,FED A	2Fe-2S ferredoxin-like superfamily protein	ferredoxin I
Sapur.018G044100.1	46.9833861	-0.582509678	0.076878082	-7.57706	3.53E-14	8.87E-14	black	-0.528191028	1.17E-12		AT4G21065.1		Tetratricopeptide repeat (TPR)-like superfamily protein	NA
Sapur.002G049600.1	198.0477651	-0.331433935	0.043801226	-7.56677	3.83E-14	9.59E-14	black	-0.51461439	5.40E-12	GO:0016020	AT5G36120.1	atylmg3,CCB3,YLMG3	cofactor assembly, complex C (B6F)	integral membrane protein, putative
Sapur.014G039600.1	1740.903006	-0.503921821	0.066740023	-7.55052	4.34E-14	1.08E-13	black	-0.544853814	1.62E-13	GO:0006412,GO:0005840,GO:0005622,GO:0003735	AT3G10950.1		Zinc-binding ribosomal protein family protein	60S ribosomal protein L37a
Sapur.011G045100.1	288.9279991	0.410106124	0.054525426	7.521374	5.42E-14	1.35E-13	black	0.541816811	2.34E-13	GO:0003676	AT3G15010.1		RNA-binding (RRM/RBD/RNP motifs) family protein	RNA recognition motif 1 in heterogeneous nuclear ribonucleoprotein A subfamily pro
Sapur.012G107100.1	1250.858133	-0.544855681	0.072798772	-7.48441	7.19E-14	1.79E-13	black	-0.568467868	8.10E-15		AT2G36620.1	RPL24A	ribosomal protein L24	60S ribosomal protein L24
Sapur.006G157200.1	1113.687826	-0.469758267	0.062847926	-7.47452	7.75E-14	1.92E-13	black	-0.556601196	3.76E-14	GO:0006412,GO:0005840,GO:0005622,GO:0003735	AT3G61110.1	ARS27A,RS27A	ribosomal protein S27	40S ribosomal protein S27
Sapur.016G160400.1	2371.766575	0.312729152	0.041870725	7.468921	8.09E-14	2.01E-13	black	0.547691114	1.14E-13		AT5G19400.1	SMG7	Telomerase activating protein Est1	telomerase-binding protein EST1A
Sapur.002G027500.1	2177.487394	-0.479546515	0.063914078	-7.44666	9.57E-14	2.37E-13	black	-0.516888287	4.20E-12	GO:0006412,GO:0005840,GO:0005622,GO:0003735	AT4G27090.1		Ribosomal protein L14	60S ribosomal protein L14, putative
Sapur.15WG122000.1	1222.021872	-0.461390603	0.062175926	-7.42073	1.16E-13	2.87E-13	black	-0.527837564	1.22E-12	GO:0006412,GO:0005840,GO:0005622,GO:0003735	AT1G26880.1		Ribosomal protein L34e superfamily protein	60S ribosomal protein L34
Sapur.019G070100.1	288.2749082	-0.366355948	0.049491726	-7.40237	1.34E-13	3.29E-13	black	-0.505702446	1.42E-11		AT2G21195.1			hypothetical protein
Sapur.018G107900.1	1515.258414	0.373443987	0.050569201	7.384811	1.53E-13	3.75E-13	black	0.497771736	3.30E-11	GO:0005515,GO:0006468,GO:0004672	AT3G23750.1		Leucine-rich repeat protein kinase family protein	receptor-like kinase Rhg4
Sapur.002G099400.1	168.7032983	-0.375035477	0.050872165	-7.37212	1.68E-13	4.12E-13	black	-0.513062062	6.41E-12		AT1G47200.1	WPP2	WPP domain protein 2	WPP domain protein
Sapur.008G121300.1	878.3877931	-0.563459952	0.076700934	-7.34619	2.04E-13	5.00E-13	black	-0.502672676	1.97E-11	GO:0006412,GO:0005840,GO:0003735	AT5G18380.3		Ribosomal protein S5 domain 2-like superfamily protein	40S ribosomal protein S16
Sapur.008G032100.1	2547.519986	-0.447782808	0.060954592	-7.34617	2.04E-13	5.00E-13	black	-0.553269096	5.72E-14	GO:0005525	AT1G07920.1		GTP binding Elongation factor Tu family protein	elongation factor 1-alpha
Sapur.012G025600.1	729.6704914	-0.521629402	0.071054234	-7.34129	2.12E-13	5.18E-13	black	-0.568146239	8.45E-15		AT3G46560.1	emb2474,TIM9	Tim10/DDP family zinc finger protein	import inner membrane translocase subunit Tim9
Sapur.002G110100.1	852.0642946	-0.383138191	0.052281542	-7.32836	2.33E-13	5.69E-13	black	-0.528130589	1.18E-12	GO:0006457,GO:0005737	AT3G60210.1		GroES-like family protein	chaperonin 10 kDa subunit
Sapur.008G171700.1	90.44429409	-0.498766104	0.068188317	-7.31454	2.58E-13	6.30E-13	black	-0.502211984	2.07E-11		AT5G18920.1		Cox19-like CHCH family protein	Cox19-like CHCH family protein, putative
Sapur.003G119300.1	2556.988727	-0.436381175	0.059675564	-7.31256	2.62E-13	6.39E-13	black	-0.54996409	8.63E-14	GO:0006412,GO:0005840,GO:0005622,GO:0003735	AT3G61110.1	ARS27A,RS27A	ribosomal protein S27	40S ribosomal protein S27
Sapur.003G107700.1	39.13860844	0.693639045	0.094937154	7.306297	2.75E-13	6.69E-13	black	0.523325459	2.04E-12					NA
Sapur.012G011400.1	504.2189248	-0.308074479	0.042333777	-7.27727	3.41E-13	8.27E-13	black	-0.552833955	6.04E-14	GO:0006412,GO:0005840,GO:0005622,GO:0003735	AT5G53070.1		Ribosomal protein L9/RNase H1	ribosomal protein L9
Sapur.014G057000.1	290.0228694	-0.347618641	0.047819847	-7.26934	3.61E-13	8.76E-13	black	-0.518091073	3.67E-12	GO:0034227,GO:0005737	AT3G61113.1		Ubiquitin related modifier 1	ubiquitin-related modifier-like protein
Sapur.009G080500.1	1144.93205	0.43561054	0.060046456	7.254559	4.03E-13	9.76E-13	black	0.500190695	2.56E-11	GO:0006914	AT3G19190.1	ATATG2,ATG2	autophagy 2	autophagy-related protein
Sapur.T017400.1	6.355623851	0.96168046	0.13260878	7.252012	4.11E-13	9.94E-13	black	0.525106288	1.66E-12	GO:0006468,GO:0005524,GO:0004672	AT1G29720.1		Leucine-rich repeat transmembrane protein kinase	NA
Sapur.010G111100.1	2657.79652	-0.456735033	0.062989873	-7.25093	4.14E-13	1.00E-12	black	-0.551687526	6.97E-14	GO:0006412,GO:0005840,GO:0005622,GO:0003735	AT3G06680.1		Ribosomal L29e protein family	NA
Sapur.014G099500.1	193.6557714	-0.563619146	0.078280163	-7.20003	6.02E-13	1.45E-12	black	-0.512983514	6.46E-12	GO:0043565,GO:0008270,GO:0006355,GO:0003700	AT5G49300.1	GATA16	GATA transcription factor 16	GATA transcription factor, putative
Sapur.002G198700.1	235.5785506	-0.328663373	0.045675411	-7.19563	6.22E-13	1.50E-12	black	-0.505368511	1.48E-11		AT1G04130.1		Tetratricopeptide repeat (TPR)-like superfamily protein	heat shock protein 70 (HSP70)-interacting protein, putative
Sapur.007G004200.1	23.4407953	-0.567364681	0.078984098	-7.18328	6.81E-13	1.64E-12	black	-0.481407659	1.74E-10					NA
Sapur.002G044700.1	2332.656957	-0.442842622	0.061807317	-7.16489	7.78E-13	1.87E-12	black	-0.498930259	2.92E-11	GO:0006412,GO:0005840,GO:0005622,GO:0003735	AT2G32060.3		Ribosomal protein L7Ae/L30e/S12e/Gadd45 family protein	40S ribosomal protein S12
Sapur.005G075000.1	94.73705584	-0.559205875	0.078118809	-7.1584	8.16E-13	1.96E-12	black	-0.482192135	1.61E-10	GO:0006511	AT3G21860.1	ASK10,SK10	SKP1-like 10	Skp1 family, dimerization domain protein
Sapur.016G132600.1	486.5568258	-0.481243589	0.067324166	-7.14816	8.80E-13	2.11E-12	black	-0.548738715	1.00E-13		AT2G28900.1	ATOEP16-1,ATOEP16-L,OEP	outer plastid envelope protein 16-1	amino acid selective channel protein
Sapur.006G175200.1	1553.519027	-0.415617557	0.058242587	-7.13597	9.61E-13	2.30E-12	black	-0.544813083	1.63E-13	GO:0006412,GO:0005840,GO:0005622,GO:0003735	AT5G02610.1		Ribosomal L29 family protein	60S ribosomal protein L35
Sapur.003G021700.1	523.6533822	-0.341488742	0.047856238	-7.13572	9.63E-13	2.30E-12	black	-0.518553912	3.49E-12	GO:0006412,GO:0005840,GO:0005622,GO:0003735	AT3G13882.1		Ribosomal protein L34	ribosomal protein L34
Sapur.008G124500.1	222.5268057	-0.331102007	0.046426453	-7.13175	9.91E-13	2.37E-12	black	-0.498305635	3.12E-11		AT4G14870.1		secE/sec61-gamma protein transport protein	SecE/Sec61-gamma subunit of protein translocation complex protein
Sapur.014G143200.1	22.92864779	-0.847891639	0.120038289	-7.06351	1.62E-12	3.86E-12	black	-0.487910053	9.08E-11	GO:0006412,GO:0005840,GO:0005622,GO:0003735,GO:0008097	AT5G39740.2	OLI7,RPL5B	ribosomal protein L5 B	NA
Sapur.009G123900.1	4346.970241	-0.373704746	0.052979586	-7.05375	1.74E-12	4.13E-12	black	-0.540460132	2.75E-13	GO:0006412,GO:0005840,GO:0005622,GO:0003735	AT1G77940.1		Ribosomal protein L7Ae/L30e/S12e/Gadd45 family protein	60S ribosomal protein L30
Sapur.003G008300.1	882.774684	-0.380598596	0.053977778	-7.05102	1.78E-12	4.21E-12	black	-0.549490719	9.15E-14	GO:0006397	AT1G03330.1		Small nuclear ribonucleoprotein family protein	snRNP core Sm Sm-X5-like protein
Sapur.019G064500.1	145.1361591	-0.343251376	0.048709287	-7.04694	1.83E-12	4.33E-12	black	-0.475716143	3.05E-10		AT4G10330.1		glycine-rich protein	transmembrane protein, putative
Sapur.009G029400.1	215.6390635	-0.449016213	0.06373499	-7.04505	1.85E-12	4.39E-12	black	-0.550177102	8.41E-14		AT5G52370.1			hypothetical protein
Sapur.012G071500.1	795.6587021	-0.278000917	0.039539431	-7.03098	2.05E-12	4.85E-12	black	-0.510124156	8.83E-12	GO:0004017,GO:0019205,GO:0006139,GO:0005524	AT5G63400.1	ADK1	adenylate kinase 1	adenylate kinase
Sapur.007G032400.1	5206.242078	-0.332836853	0.047390898	-7.02322	2.17E-12	5.13E-12	black	-0.523401712	2.02E-12		AT2G27530.2	PGY1	Ribosomal protein L1p/L10e family	60S ribosomal protein L10a
Sapur.005G196800.1	1871.194808	-0.351662458	0.050136393	-7.01412	2.31E-12	5.47E-12	black	-0.561784812	1.94E-14	GO:0008168,GO:0008152,GO:0006694	AT1G20330.1	CVP1,FRL1,SMT2	sterol methyltransferase 2	sterol 24-C-methyltransferase
Sapur.007G010600.1	4816.49906	-0.351203464	0.050159852	-7.00168	2.53E-12	5.96E-12	black	-0.534735284	5.44E-13	GO:0006412,GO:0005840,GO:0005622,GO:0003735	AT4G36130.1		Ribosomal protein L2 family	60S ribosomal protein L2
Sapur.001G173800.1	2336.54458	-0.483369165	0.069252662	-6.97979	2.96E-12	6.96E-12	black	-0.467971555	6.41E-10	GO:0016020,GO:0009765	AT1G15820.1	CP24,LHCB6	light harvesting complex photosystem II subunit 6	light harvesting chlorophyll A-B-binding protein
Sapur.010G086100.1	2929.063434	0.437098963	0.062664913	6.975179	3.05E-12	7.19E-12	black	0.487846853	9.14E-11	GO:0005524,GO:0046488,GO:0016307	AT1G71010.1	FAB1C	FORMS APLOID AND BINUCLEATE CELLS 1C	1-phosphatidylinositol-3-phosphate 5-kinase fab1
Sapur.002G116000.1	158.5988162	-0.388203211	0.055723971	-6.96654	3.25E-12	7.63E-12	black	-0.488910436	8.21E-11		AT3G15351.1			hypothetical protein
Sapur.004G032700.1	6.876495227	-1.016979016	0.146110183	-6.96036	3.39E-12	7.97E-12	black	-0.489632321	7.63E-11		AT5G46110.4	APE2,TPT	Glucose-6-phosphate/phosphate translocator-related	triose phosphate/phosphate translocator
Sapur.011G071500.1	575.920084	0.422961227	0.060798205	6.956805	3.48E-12	8.17E-12	black	0.496717879	3.68E-11	GO:0016020,GO:0006075,GO:0003843,GO:0000148	AT3G14570.1	ATGSL04,atgsl4,gsl04	glucan synthase-like 4	callose synthase
Sapur.014G037600.1	1723.249443	-0.309164949	0.044496092	-6.94814	3.70E-12	8.67E-12	black	-0.529590936	9.93E-13	GO:0006241,GO:0006228,GO:0006183,GO:0006165,GO:0004550	AT4G09320.1	NDPK1	Nucleoside diphosphate kinase family protein	nucleoside diphosphate kinase
Sapur.018G113200.1	1086.761294	-0.50027098	0.072063174	-6.94212	3.86E-12	9.04E-12	black	-0.488886265	8.23E-11	GO:0006412,GO:0005840,GO:0003735	AT2G37190.1		Ribosomal protein L11 family protein	60S ribosomal protein L12
Sapur.002G040000.1	3047.570346	-0.349163938	0.050338956	-6.93626	4.03E-12	9.42E-12	black	-0.476442147	2.84E-10	GO:0006412,GO:0005840,GO:0005622,GO:0003735,GO:0003723	AT1G34030.1		Ribosomal protein S13/S18 family	40S ribosomal protein S18
Sapur.003G068600.1	596.52509	-0.339592059	0.049076943	-6.91958	4.53E-12	1.06E-11	black	-0.503296798	1.84E-11	GO:0006351,GO:0003899,GO:0003677	AT1G61700.1		RNA polymerases N / 8 kDa subunit	DNA-directed RNA polymerase I, II
Sapur.006G102100.1	4734.967934	0.380214454	0.055230292	6.884165	5.81E-12	1.35E-11	black	0.486721166	1.02E-10	GO:0016887,GO:0005524,GO:0055085,GO:0042626,GO:0016021,GO:0006810	AT2G36910.1	ABCB1,ATPGP1,PGP1	ATP binding cassette subfamily B1	ABC transporter family B protein, group MDR/PGP protein PpABCB26 protein
Sapur.009G122800.1	552.9786872	-0.233954516	0.034091935	-6.86246	6.77E-12	1.57E-11	black	-0.508856446	1.01E-11	GO:0055114,GO:0016491	AT5G17770.1	ATCBR,CBR,CBR1	NADH:cytochrome B5 reductase 1	NADH-cytochrome b5 reductase
Sapur.016G289400.1	2856.823243	0.272695914	0.039739028	6.862169	6.78E-12	1.57E-11	black	0.504567631	1.61E-11	GO:0005975,GO:0004553	AT1G32860.1		Glycosyl hydrolase superfamily protein	1%2C3-beta-D-glucanase GH17-33
Sapur.014G102000.1	79.36428593	-0.643750354	0.094155772	-6.83708	8.08E-12	1.87E-11	black	-0.459568627	1.41E-09		AT4G02370.1		Protein of unknown function, DUF538	transmembrane protein, putative
Sapur.017G015800.1	2126.110855	-0.505385751	0.074090156	-6.82123	9.03E-12	2.09E-11	black	-0.539332265	3.15E-13	GO:0006412,GO:0005840,GO:0005622,GO:0003735	AT3G59540.1		Ribosomal L38e protein family	NA
Sapur.001G182700.1	2649.169786	-0.213997251	0.031379361	-6.81968	9.12E-12	2.11E-11	black	-0.473969065	3.61E-10		AT5G49910.1	cpHsc70-2,CPHSC70-2EAT S	chloroplast heat shock protein 70-2	NA
Sapur.003G079500.1	1598.475525	-0.435738157	0.06394489	-6.81428	9.47E-12	2.19E-11	black	-0.501727834	2.17E-11	GO:0006412,GO:0005840,GO:0003735	AT1G07770.2	RPS15A	ribosomal protein S15A	40S ribosomal protein S15a
Sapur.009G106500.1	1380.681229	-0.263007438	0.038606224	-6.81257	9.59E-12	2.21E-11	black	-0.54408021	1.78E-13	GO:0019773,GO:0006511,GO:0004175,GO:0051603,GO:0005839,GO:0004298	AT5G66140.1	PAD2	proteasome alpha subunit D2	proteasome subunit alpha type-7 protein
Sapur.001G151100.1	565.1194081	-0.505370751	0.074219519	-6.80914	9.82E-12	2.27E-11	black	-0.499562312	2.73E-11	GO:0006412,GO:0005840,GO:0005622,GO:0003735	AT3G16080.1		Zinc-binding ribosomal protein family protein	60S ribosomal protein L37, putative
Sapur.009G085000.1	262.5107908	-0.576326422	0.084725537	-6.80228	1.03E-11	2.37E-11	black	-0.471190775	4.72E-10		AT2G15890.1	MEE14	maternal effect embryo arrest 14	maternal effect embryo arrest 14-2 protein, putative
Sapur.016G049000.1	4724.801881	-0.303463781	0.044811188	-6.77205	1.27E-11	2.92E-11	black	-0.501075136	2.33E-11	GO:0006412,GO:0005840,GO:0003735,GO:0003723	AT2G41840.1		Ribosomal protein S5 family protein	40S ribosomal protein S2
Sapur.017G115800.1	2233.872082	-0.32627632	0.048216473	-6.76691	1.32E-11	3.02E-11	black	-0.506248866	1.34E-11		AT1G17880.1	ATBTF3,BTF3	basic transcription factor 3	BTF3-like transcription factor
Sapur.011G048600.1	216.2884066	-0.297944943	0.044200423	-6.74077	1.58E-11	3.61E-11	black	-0.467803167	6.52E-10					NA
Sapur.002G011200.1	1641.539502	-0.331987896	0.049302681	-6.73367	1.65E-11	3.78E-11	black	-0.545407645	1.51E-13	GO:0008168,GO:0008152,GO:0006694	AT1G20330.1	CVP1,FRL1,SMT2	sterol methyltransferase 2	sterol 24-C-methyltransferase
Sapur.016G077300.1	1027.003092	-0.36789076	0.054779471	-6.71585	1.87E-11	4.27E-11	black	-0.496180349	3.89E-11	GO:0006412,GO:0005840,GO:0005622,GO:0003735	AT3G49010.1	ATBBC1,BBC1,RSU2	breast basic conserved 1	60S ribosomal protein L13
Sapur.007G097800.1	838.642135	0.372659507	0.055601004	6.702388	2.05E-11	4.67E-11	black	0.517694229	3.84E-12	GO:0016773,GO:0048015,GO:0046854,GO:0006661,GO:0004430	AT5G64070.1	PI-4KBETA1,PI4KBETA1	phosphatidylinositol 4-OH kinase beta1	phosphatidylinositol 4-kinase, putative
Sapur.019G027900.1	717.3403387	0.268453779	0.040092933	6.695788	2.15E-11	4.89E-11	black	0.487713544	9.27E-11	GO:0005634	AT1G08620.1	PKDM7D	Transcription factor jumonji (jmj) family protein / zinc finger (C5HC2 type) family protein	transcription factor jumonji family protein
Sapur.008G056900.1	10.44586191	-0.886622576	0.13330368	-6.65115	2.91E-11	6.59E-11	black	-0.477364226	2.59E-10	GO:0019867	AT5G05520.1		Outer membrane OMP85 family protein	sorting and assembly machinery component-like protein
Sapur.009G093900.1	2079.276741	-0.422016858	0.063605013	-6.63496	3.25E-11	7.34E-11	black	-0.490380139	7.07E-11		AT4G39200.1		Ribosomal protein S25 family protein	40S ribosomal protein S25, putative
Sapur.001G183200.1	101.4959353	-0.434824862	0.065764393	-6.61186	3.80E-11	8.57E-11	black	-0.479662318	2.07E-10	GO:0009113,GO:0004637	AT1G09830.1		Glycinamide ribonucleotide (GAR) synthetase	phosphoribosylamine-glycine ligase
Sapur.001G012300.1	726.9998982	-0.661050277	0.100206061	-6.59691	4.20E-11	9.46E-11	black	-0.414718755	6.66E-08	GO:0005515,GO:0055114,GO:0046872,GO:0016702	AT3G45140.1	ATLOX2,LOX2	lipoxygenase 2	lipoxygenase
Sapur.010G186800.1	409.6473684	-0.341695386	0.052125382	-6.55526	5.55E-11	1.25E-10	black	-0.517970288	3.72E-12		AT3G52230.1			E24 ASN protein, putative
Sapur.007G078600.1	92.51686431	0.703658522	0.107761193	6.529795	6.59E-11	1.47E-10	black	0.44605207	4.78E-09	GO:0006468,GO:0004672,GO:0004674	AT5G58950.1		Protein kinase superfamily protein	tyrosine kinase family protein
Sapur.004G085300.1	28.3278125	0.482470886	0.074303069	6.493283	8.40E-11	1.87E-10	black	0.497864733	3.26E-11		AT1G67140.3	SWEETIE	HEAT repeat-containing protein	NA
Sapur.010G043400.1	1578.938116	-0.34962575	0.053940871	-6.48165	9.07E-11	2.02E-10	black	-0.514636763	5.39E-12	GO:0006412,GO:0005840,GO:0005622,GO:0003735	AT2G33450.1		Ribosomal L28 family	50S ribosomal protein L28

Sapur.012G098200.1	2665.219353	-0.339597179	0.052396168	-6.48134	9.09E-11	2.02E-10	black	-0.450711091	3.16E-09	GO:0006412,GO:0005840,GO:0003735	AT5G62300.2		Ribosomal protein S10p/S20e family protein	40S ribosomal protein S20
Sapur.018G044000.1	671.4274676	-0.186182707	0.02880293	-6.46402	1.02E-10	2.27E-10	black	-0.472825897	4.03E-10	GO:0006450	AT4G32915.1			aspartyl/glutamyl-tRNA(Asn/Gln) amidotransferase subunit C
Sapur.008G060800.1	1060.776238	-0.418980811	0.064851455	-6.46062	1.04E-10	2.32E-10	black	-0.514756435	5.32E-12	GO:0006412,GO:0005840,GO:0005622,GO:0003735	AT2G43460.1		Ribosomal L38e protein family	60S ribosomal protein L38
Sapur.003G044500.1	296.3469712	0.311223984	0.048215711	6.454825	1.08E-10	2.41E-10	black	0.485245818	1.19E-10	GO:0046872,GO:0030001,GO:0000166	AT5G44790.1	HMA7,RAN1	copper-exporting ATPase / responsive-to-antagonist 1 / copper-transporting ATPase (RAN1)	copper-transporting ATPase P-type protein, putative
Sapur.019G060600.1	2792.482809	-0.375397062	0.058175647	-6.45282	1.10E-10	2.44E-10	black	-0.489405144	7.81E-11	GO:0006412,GO:0005840,GO:0005622,GO:0003735	AT2G40510.1		Ribosomal protein S26e family protein	40S ribosomal protein S26
Sapur.004G137600.1	482.4016087	0.288964064	0.044797855	6.4504	1.12E-10	2.48E-10	black	0.455154645	2.11E-09	GO:0006468,GO:0005524,GO:0004672	AT4G35230.1	BSK1	BR-signaling kinase 1	kinase family protein
Sapur.009G040200.1	280.2980408	-0.234069835	0.036321448	-6.4444	1.16E-10	2.57E-10	black	-0.487514635	9.46E-11		AT3G20890.1		RNA-binding (RRM/RBD/RNP motifs) family protein	heterogeneous nuclear ribonucleoprotein, putative
Sapur.002G053700.1	637.7162081	-0.450350563	0.06999204	-6.43431	1.24E-10	2.75E-10	black	-0.484713103	1.25E-10		AT1G76860.1		Small nuclear ribonucleoprotein family protein	U6 snRNA-associated-like-Smprotein LsM3
Sapur.006G062500.1	62.9458512	-0.578470508	0.090185675	-6.41422	1.42E-10	3.13E-10	black	-0.469592546	5.50E-10		AT2G19080.1		metaxin-related	outer membrane import complex protein metaxin, putative
Sapur.002G171400.1	386.4575808	0.197909152	0.031041169	6.375699	1.82E-10	4.01E-10	black	0.444675852	5.40E-09		AT3G07790.1		DGCR14-related	nuclear protein Es2
Sapur.002G200400.1	461.1446193	-0.369014118	0.05793488	-6.36946	1.90E-10	4.17E-10	black	-0.523980109	1.89E-12	GO:0006886,GO:0005741	AT5G43970.1	ATTOM22-V.TOM22-V.TOM	translocase of outer membrane 22-V	import receptor subunit Tom22
Sapur.019G074700.1	1088.972565	-0.265849934	0.04174677	-6.36816	1.91E-10	4.21E-10	black	-0.477598043	2.54E-10	GO:0005515,GO:0006414,GO:0003746	AT1G09640.1		Translation elongation factor EF1B, gamma chain	elongation factor 1-gamma 1
Sapur.016G265500.1	256.6633125	-0.449623235	0.07075389	-6.35475	2.09E-10	4.58E-10	black	-0.472857418	4.02E-10		AT4G27380.1			plant/M4I22-190 protein, putative
Sapur.008G041200.1	312.7989603	-0.399024254	0.062820853	-6.35178	2.13E-10	4.67E-10	black	-0.488546991	8.52E-11		AT3G12587.1		Oligosaccaryltransferase	dolichyl-diphosphooligosaccharide-protein glycosyltransferase subunit 4B
Sapur.016G155600.1	790.8760906	-0.265238644	0.041930681	-6.32565	2.52E-10	5.51E-10	black	-0.474750761	3.35E-10	GO:0006412,GO:0005840,GO:0005622,GO:0003735	AT2G19740.1		Ribosomal protein L31e family protein	60S ribosomal protein L31
Sapur.004G102700.1	649.7485373	-0.288482649	0.045710289	-6.31111	2.77E-10	6.05E-10	black	-0.461625638	1.16E-09	GO:0051082,GO:0016272,GO:0006457	AT1G29990.1	PFD6	prefoldin 6	prefoldin subunit
Sapur.006G171700.1	2415.84733	-0.299418881	0.047548813	-6.29708	3.03E-10	6.61E-10	black	-0.451200246	3.02E-09	GO:0019843,GO:0005622,GO:0003723	AT5G39850.1		Ribosomal protein S4	NA
Sapur.013G007200.1	112.201717	-0.422736189	0.067204055	-6.29034	3.17E-10	6.90E-10	black	-0.496250813	3.86E-11		AT5G26800.1			IGR motif protein
Sapur.010G203300.1	94.91470387	-0.289852663	0.046228936	-6.26994	3.61E-10	7.85E-10	black	-0.427775932	2.30E-08	GO:0043565,GO:0008270,GO:0006355,GO:0003700,GO:0005515	AT1G51600.2	GATA28,TIFY2A,ZML2	ZIM-LIKE 2	GATA transcription factor
Sapur.005G016500.1	40.48442313	-0.534540345	0.085470564	-6.25409	4.00E-10	8.68E-10	black	-0.441045175	7.41E-09	GO:0004857				NA
Sapur.009G004000.1	35.29908205	-0.839773565	0.134495355	-6.24389	4.27E-10	9.25E-10	black	-0.427068295	2.43E-08					NA
Sapur.016G296200.1	423.5351247	-0.389531428	0.062443926	-6.2381	4.43E-10	9.60E-10	black	-0.500741648	2.41E-11	GO:0006351,GO:0003899,GO:0003677	AT5G41010.1	NRPB12,NRPD12,NRPE12	DNA directed RNA polymerase, 7 kDa subunit	DNA-directed RNA polymerase I, II
Sapur.001G105900.1	1799.622097	-0.14709757	0.02359845	-6.23336	4.57E-10	9.89E-10	black	-0.44966817	3.46E-09	GO:0005525	AT1G04170.1	EIF2 GAMMA	eukaryotic translation initiation factor 2 gamma subunit	eukaryotic translation initiation factor 2 gamma subunit
Sapur.007G076400.1	151.0172214	-0.379188788	0.061128323	-6.20316	5.53E-10	1.19E-09	black	-0.437968593	9.68E-09	GO:0005975,GO:0004134	AT5G64860.1	DPE1	disproportionating enzyme	4-alpha-glucanotransferase/amyloplastic protein
Sapur.008G121000.2	7.055871981	-0.901609445	0.145487769	-6.19715	5.75E-10	1.24E-09	black	-0.534331932	5.71E-13		AT5G19300.1			RNA methyltransferase, putative
Sapur.012G109500.1	1022.371667	-0.45618861	0.073762772	-6.18454	6.23E-10	1.34E-09	black	-0.466977453	7.05E-10	GO:0006412,GO:0005840,GO:0005622,GO:0003735	AT3G53740.4		Ribosomal protein L36e family protein	60S ribosomal protein L36
Sapur.001G139400.1	255.8875765	-0.224069751	0.036346543	-6.16482	7.06E-10	1.52E-09	black	-0.481333122	1.76E-10	GO:0005777	AT3G03490.1	AtPEX19-1,PEX19-1	peroxin 19-1	peroxisomal biogenesis factor
Sapur.008G050200.1	2778.015822	-0.299145463	0.048558972	-6.16046	7.25E-10	1.56E-09	black	-0.455738034	2.00E-09	GO:0006412,GO:0005840,GO:0005622,GO:0003735	AT1G74270.1		Ribosomal protein L35Ae family protein	60S ribosomal protein L35a
Sapur.010G042200.1	1726.522362	-0.339337234	0.055215477	-6.14569	7.96E-10	1.71E-09	black	-0.42857001	2.15E-08	GO:0006412,GO:0005840,GO:0005622,GO:0003735	AT4G27090.1		Ribosomal protein L14	60S ribosomal protein L14, putative
Sapur.014G116700.3	1.461053542	-1.542612759	0.252683033	-6.10493	1.03E-09	2.19E-09	black	-0.519350964	3.19E-12	GO:0008270,GO:0004553,GO:0015923,GO:0006013,GO:0005975,GO:0004559	AT5G13980.2		Glycosyl hydrolase family 38 protein	glycoside hydrolase family 38 protein
Sapur.016G258500.1	68.5675419	-0.70316829	0.115420298	-6.09224	1.11E-09	2.37E-09	black	-0.405426709	1.38E-07					hypothetical protein
Sapur.012G103200.1	68.20260234	-0.588971252	0.096704449	-6.09043	1.13E-09	2.40E-09	black	-0.447829257	4.08E-09		AT1G06010.1			transmembrane protein, putative
Sapur.009G039700.1	53.11097942	-0.549490438	0.090283483	-6.08628	1.16E-09	2.46E-09	black	-0.435987024	1.15E-08	GO:0055114,GO:0016491,GO:0051536,GO:0020037	AT5G04590.1	SIR	sulfite reductase	sulfite reductase [ferredoxin] protein
Sapur.15WG128800.1	1235.603107	-0.351228068	0.057848644	-6.0715	1.27E-09	2.69E-09	black	-0.460949725	1.24E-09	GO:0006412,GO:0005840,GO:0003735	AT2G47110.2	UBQ6	ubiquitin 6	NA
Sapur.001G046500.1	280.5749642	0.219650461	0.036240577	6.060899	1.35E-09	2.87E-09	black	0.462696856	1.05E-09		AT5G53150.1		DNAJ heat shock N-terminal domain-containing protein	chaperone protein DnaJ
Sapur.019G030800.1	557.0540148	-0.312885321	0.051641267	-6.05882	1.37E-09	2.91E-09	black	-0.470630092	4.98E-10	GO:0016491	AT3G06050.1	ATPRXIIF,PRXIIF	peroxiredoxin IIF	peroxiredoxin-2F protein
Sapur.006G129500.1	1071.449615	-0.359848195	0.05956859	-6.04091	1.53E-09	3.24E-09	black	-0.458123004	1.61E-09	GO:0006412,GO:0005840,GO:0005622,GO:0003735	AT1G43170.5	ARP1,emb2207,RP1,RPL3A	ribosomal protein 1	NA
Sapur.008G114400.1	1750.98908	-0.202317785	0.033569571	-6.02682	1.67E-09	3.54E-09	black	-0.458129588	1.61E-09	GO:0005515	AT2G46290.1		Transducin/WD40 repeat-like superfamily protein	eukaryotic translation initiation factor 3 subunit I
Sapur.016G120600.1	4045.900602	-0.198106775	0.032879659	-6.02521	1.69E-09	3.57E-09	black	-0.436106894	1.14E-08	GO:0006414,GO:0005853,GO:0003746	AT5G12110.1		Glutathione S-transferase, C-terminal-like;Translation elongation factor EF1B/ribosomal protein S6	elongation factor 1-beta
Sapur.006G181500.1	512.7042149	0.297944562	0.049546734	6.013405	1.82E-09	3.84E-09	black	0.433509894	1.42E-08		AT2G06040.1			Rad7, putative
Sapur.002G071700.1	1659.087886	0.298696591	0.049720299	6.007538	1.88E-09	3.97E-09	black	0.465893694	7.81E-10		AT1G77800.2		PHD finger family protein	PHD finger protein
Sapur.018G087900.1	1957.282155	0.315684333	0.052565847	6.005503	1.91E-09	4.02E-09	black	0.438433652	9.30E-09	GO:0008289	AT5G35180.2		Protein of unknown function (DUF1336)	plant/T25C13-60 protein
Sapur.002G046000.1	2633.39277	-0.348343817	0.058490354	-5.95558	2.59E-09	5.44E-09	black	-0.475791105	3.02E-10	GO:0006412,GO:0005840,GO:0003735	AT2G34480.1		Ribosomal protein L18ae/LX family protein	60S ribosomal protein L18a
Sapur.005G204100.2	12.25676176	-0.659883821	0.111130667	-5.93791	2.89E-09	6.05E-09	black	-0.138923563	0.082701575		AT1G76405.2			outer envelope pore protein 21B
Sapur.010G045300.1	6926.907436	-0.348847562	0.058948219	-5.91786	3.26E-09	6.82E-09	black	-0.497913137	3.25E-11	GO:0006412,GO:0005840,GO:0003735	AT3G04400.1	emb2171	Ribosomal protein L14p/L23e family protein	60S ribosomal protein L23
Sapur.006G074100.1	111.3272052	-0.296425826	0.050101404	-5.91652	3.29E-09	6.87E-09	black	-0.464383943	9.00E-10		AT3G53850.1		Uncharacterised protein family (UPF0497)	CASP-like protein Os09g0249400
Sapur.002G009200.1	198.1954108	-0.271903017	0.04609248	-5.89908	3.66E-09	7.63E-09	black	-0.46137701	1.19E-09		AT1G20430.1			transmembrane protein, putative
Sapur.018G037800.1	4279.529981	-0.227653557	0.038627232	-5.8936	3.78E-09	7.88E-09	black	-0.503346393	1.83E-11	GO:0003723	AT5G35530.1		Ribosomal protein S3 family protein	40S ribosomal protein S3
Sapur.005G115900.1	96.53741356	-0.568155008	0.096677139	-5.87683	4.18E-09	8.71E-09	black	-0.417086197	5.51E-08	GO:0006412,GO:0005840,GO:0005622,GO:0003735	AT5G10360.1	EMB3010,RPS6B	Ribosomal protein S6e	40S ribosomal protein S6
Sapur.012G012700.1	69.07660751	-0.32186856	0.055075615	-5.84412	5.09E-09	1.06E-08	black	-0.460945265	1.24E-09	GO:0008270,GO:0006351,GO:0003676	AT4G07950.1		DNA-directed RNA polymerase, subunit M, archaeal	DNA-directed RNA polymerase subunit
Sapur.006G198800.1	2562.470646	0.33733289	0.05777368	5.838868	5.26E-09	1.09E-08	black	0.428789709	2.11E-08		AT5G11560.1		catalytics	pqq-dh domain protein
Sapur.006G161300.1	229.4295711	-0.195075114	0.033500335	-5.82308	5.78E-09	1.20E-08	black	-0.414552494	6.75E-08	GO:0016592,GO:0006357,GO:0001104	AT5G03500.2		Mediator complex, subunit Med7	mediator of RNA polymerase II transcription subunit 7, putative
Sapur.016G188200.1	3087.21268	-0.300444073	0.051831565	-5.79655	6.77E-09	1.40E-08	black	-0.479865697	2.03E-10	GO:0006412,GO:0005840,GO:0005622,GO:0003735	AT5G10360.1	EMB3010,RPS6B	Ribosomal protein S6e	40S ribosomal protein S6
Sapur.010G050500.1	3414.895491	-0.334834063	0.057880827	-5.78489	7.26E-09	1.50E-08	black	-0.459806598	1.38E-09	GO:0006412,GO:0005840,GO:0003735,GO:0015934	AT1G67430.1		Ribosomal protein L22p/L17e family protein	60S ribosomal protein L17
Sapur.016G135200.1	58.29913952	-0.321028446	0.055732792	-5.76014	8.40E-09	1.73E-08	black	-0.457503311	1.70E-09		AT1G76440.2		HSP20-like chaperones superfamily protein	small heat shock protein
Sapur.007G066600.1	525.6175745	-0.202650556	0.035242289	-5.75021	8.91E-09	1.83E-08	black	-0.441377488	7.20E-09		AT5G23140.1	CLPP2,NCLPP7	nuclear-encoded CLP protease P7	ATP-dependent Clp protease proteolytic subunit
Sapur.001G113100.1	95.04592185	0.436756164	0.076004169	5.746476	9.11E-09	1.87E-08	black	0.459224075	1.45E-09					NA
Sapur.15WG076700.1	853.0278374	-0.402696651	0.070112553	-5.74357	9.27E-09	1.90E-08	black	-0.466163567	7.61E-10		AT5G08040.1	TOM5	mitochondrial import receptor subunit TOM5 homolog	import receptor subunit TOM5-like protein
Sapur.008G009300.1	789.8163773	0.213602936	0.037207597	5.740842	9.42E-09	1.93E-08	black	0.422185094	3.64E-08	GO:0006468,GO:0004672	AT5G04870.1	ATCPK1,CPK1	calcium dependent protein kinase 1	calcium/calmodulin-dependent kinase
Sapur.016G273300.1	82.14987859	-0.475768944	0.083390755	-5.7053	1.16E-08	2.37E-08	black	-0.444009439	5.72E-09	GO:0003676	AT5G54580.1		RNA-binding (RRM/RBD/RNP motifs) family protein	RNA recognition motif, a.k.a. RRM%2C RBD protein
Sapur.006G219900.1	1063.33668	-0.402703022	0.070716244	-5.69463	1.24E-08	2.52E-08	black	-0.426079889	2.64E-08		AT2G24940.1	AtMAPR2,MAPR2	membrane-associated progesterone binding protein 2	membrane steroid-binding protein
Sapur.006G035700.1	394.888149	0.28168395	0.049746853	5.662347	1.49E-08	3.04E-08	black	0.436968962	1.05E-08	GO:0005515	AT2G41520.1		Heat shock protein DnaJ with tetratricopeptide repeat	heat shock protein DnaJ with TPR protein, putative
Sapur.003G003400.1	35.89853179	0.455412444	0.080458471	5.660217	1.51E-08	3.07E-08	black	0.432683594	1.52E-08		AT4G24290.2		MAC/Perforin domain-containing protein	membrane-attack complex/perforin
Sapur.018G060200.2	5.348177525	0.826484431	0.146190512	5.653475	1.57E-08	3.19E-08	black	0.535690959	4.86E-13	GO:0055085,GO:0016021	AT1G67300.1		Major facilitator superfamily protein	plastidic glucose transporter, putative
Sapur.003G077800.1	325.1030305	0.764181616	0.135253684	5.649987	1.60E-08	3.26E-08	black	0.366579732	2.34E-06	GO:0004575,GO:0004564	AT1G12240.1	ATBETAFRUCT4,VAC-INV	Glycosyl hydrolases family 32 protein	acid vacuolar invertase
Sapur.009G117300.1	144.9349701	-0.464069576	0.08232789	-5.63685	1.73E-08	3.51E-08	black	-0.46561364	8.01E-10		AT2G27775.1			hypothetical protein
Sapur.013G018600.1	266.1402292	-0.244511829	0.043402659	-5.63357	1.77E-08	3.58E-08	black	-0.406720534	1.25E-07	GO:0005515,GO:0006508,GO:0004252	AT5G27660.1		Trypsin family protein with PDZ domain	trypsin-like serine protease
Sapur.012G024300.1	1120.225169	-0.323785804	0.057502674	-5.6308	1.79E-08	3.63E-08	black	-0.457750544	1.67E-09		AT1G17880.1	ATBTF3,BTF3	basic transcription factor 3	transcription factor-BTF3-like protein
Sapur.001G013000.1	652.8920731	0.218344583	0.039284072	5.558095	2.73E-08	5.49E-08	black	0.401293914	1.90E-07		AT1G73730.1	AtEIL3,ATSLIM,EIL3,SLIM1	ETHYLENE-INSENSITIVE3-like 3	ethylene insensitive (EIN3/EIL)-like transcription regulator
Sapur.019G054000.1	537.636053	-0.285823854	0.051634453	-5.53553	3.10E-08	6.23E-08	black	-0.460792039	1.26E-09		AT1G65290.1	mtACP2	mitochondrial acyl carrier protein 2	NA
Sapur.011G111000.1	336.2375355	-0.462575157	0.083906242	-5.513	3.53E-08	7.06E-08	black	-0.468514453	6.09E-10		AT1G61570.1	TIM13	translocase of the inner mitochondrial membrane 13	import inner membrane translocase subunit Tim13, putative
Sapur.006G039300.1	70.60046146	-0.424784605	0.077283769	-5.49643	3.88E-08	7.75E-08	black	-0.391999457	3.83E-07		AT2G41750.1		DTW domain-containing protein	DTW domain protein
Sapur.018G088000.1	646.0185168	-0.233076703	0.042447903	-5.49089	4.00E-08	7.99E-08	black	-0.388259447	5.05E-07	GO:0019843,GO:0006412,GO:0005840,GO:0003735	AT3G18760.1		Translation elongation factor EF1B/ribosomal protein S6 family protein	ribosomal protein S6
Sapur.001G091800.1	716.2546853	0.235283941	0.042966612	5.475971	4.35E-08	8.68E-08	black	0.419130592	4.67E-08		AT4G11860.1		Protein of unknown function (DUF544)	FAM63A protein
Sapur.009G006200.1	224.2944126	-0.367526083	0.067372918	-5.4551	4.89E-08	9.75E-08	black	-0.430549594	1.82E-08		AT4G27490.1		3\'-5\'-exoribonuclease family protein	exosome complex exonuclease
Sapur.014G119400.1	1512.771457	-0.154961454	0.028616688	-5.41507	6.13E-08	1.21E-07	black	-0.400266459	2.06E-07	GO:0005515,GO:0005852,GO:0005737,GO:0003743	AT1G10840.1	TIF3H1	translation initiation factor 3 subunit H1	eukaryotic translation initiation factor 3 subunit H
Sapur.006G199600.2	13.73287606	-0.571228176	0.105543757	-5.41224	6.22E-08	1.23E-07	black	-0.445179843	5.16E-09	GO:0005852,GO:0005737,GO:0003743	AT5G25757.1		RNA polymerase I-associated factor PAF67	eukaryotic translation initiation factor 3 subunit L
Sapur.008G094800.1	954.5021958	0.223157636	0.041257911	5.408845	6.34E-08	1.26E-07	black	0.414200866	6.94E-08		AT1G25540.1	PFT1	phytochrome and flowering time regulatory protein (PFT1)	mediator complex subunit 25 von willebrand factor type A protein
Sapur.016G287500.1	2212.517034	0.310015887	0.057404566	5.400544	6.64E-08	1.31E-07	black	0.425200117	2.84E-08		AT2G35630.1	GEM1,MOR1	ARM repeat superfamily protein	microtubule organization protein
Sapur.014G083300.1	865.5395623	0.287594586	0.053470192	5.378596	7.51E-08	1.48E-07	black	0.407665093	1.16E-07	GO:0016192,GO:0006886	AT4G00800.1		transducin family protein / WD-40 repeat family protein	transducin family protein/WD-40 repeat protein
Sapur.012G085600.1	3060.973881	-0.278131679	0.051792851	-5.37008	7.87E-08	1.55E-07	black	-0.462496098	1.07E-09	GO:0006412,GO:0005840,GO:0003735	AT2G47110.2	UBQ6	ubiquitin 6	ubiquitin-40S ribosomal protein S27a
Sapur.005G017200.1	694.6985863	-0.437087692	0.081510076	-5.36238	8.21E-08	1.62E-07	black	-0.552062228	6.65E-14	GO:0006412,GO:0005840,GO:0005622,GO:0003735	AT3G05590.1	RPL18	ribosomal protein L18	60S ribosomal protein L18
Sapur.010G144600.1	1247.646719	-0.174157469	0.032570944	-5.34702	8.94E-08	1.76E-07	black	-0.391953262	3.84E-07	GO:0003676,GO:0005850,GO:0003743,GO:0003723	AT2G40290.1		Eukaryotic translation initiation factor 2 subunit 1	eukaryotic translation initiation factor 2 alpha subunit
Sapur.003G124800.1	1038.078159	-0.217613867	0.040886716	-5.32236	1.02E-07	2.01E-07	black	-0.387072752	5.50E-07	GO:0006457,GO:0003755,GO:0000413	AT5G13120.1	ATCYP20-2,CYP20-2	cyclophilin 20-2	peptidyl-prolyl cis-trans isomerase, cyclophilin-type protein
Sapur.014G017300.1	806.1194029	-0.247650192	0.046736368	-5.29888	1.17E-07	2.28E-07	black	-0.385762457	6.05E-07		AT1G45230.1		Protein of unknown function (DUF3223)	DCL protein
Sapur.016G163600.1	1567.749924	-0.205263507	0.039231834	-5.23207	1.68E-07	3.26E-07	black	-0.411362945	8.70E-08	GO:0046907	AT2G30060.1		Pleckstrin homology (PH) domain superfamily protein	Ran-binding protein 2, Ran-binding domain protein
Sapur.013G081200.1	503.161399	-0.196950736	0.037711886	-5.22251	1.77E-07	3.43E-07	black	-0.383135453	7.32E-07		AT2G40600.1		appr-1-p processing enzyme family protein	appr-1-P processing enzyme family protein
Sapur.012G064100.1	1436.075008	-0.312604851	0.060009359	-5.20927	1.90E-07	3.68E-07	black	-0.422800764	3.46E-08	GO:0006412,GO:0005840,GO:0005622,GO:0003735	AT4G29390.1		Ribosomal protein S30 family protein	40S ribosomal protein S30
Sapur.001G173100.1	1054.797357	-0.416602839	0.080046888	-5.20449	1.95E-07	3.78E-07	black	-0.381029053	8.52E-07	GO:0005515	AT2G30860.1	ATGSTF7,ATGSTF9,GLUTTR	glutathione S-transferase PHI 9	glutathione S-transferase
Sapur.005G152800.1	1285.501265	-0.334720194	0.064468066	-5.19203	2.08E-07	4.03E-07	black	-0.422262347	3.62E-08	GO:0006412,GO:0005840,GO:0005622,GO:0003735	AT1G43170.2	ARP1,emb2207,RP1,RPL3A	ribosomal protein 1	60S ribosomal protein L3
Sapur.009G066200.1	1928.186245	0.296930737	0.05753185	5.161154	2.45E-07	4.74E-07	black	0.410109019	9.60E-08	GO:0005515,GO:0008144,GO:0016773	AT1G50030.1	TOR	target of rapamycin	Serine/Threonine-kinase TOR
Sapur.019G018300.1	144.3056415	0.330202434	0.064070734	5.153717	2.55E-07	4.93E-07	black	0.427061676	2.44E-08	GO:0005515,GO:0006468,GO:0004672	AT3G14840.2		Leucine-rich repeat transmembrane protein kinase	NA
Sapur.004G161900.1	87.33276837	-0.280438413	0.054499614	-5.1457	2.67E-07	5.14E-07	black	-0.388736495	4.87E-07	GO:0006412,GO:0005840,GO:0005622,GO:0003735	AT3G45020.1		Ribosomal L18p/L5e family protein	ribosomal protein L18
Sapur.016G130700.3	96.27871932	0.400971766	0.078105204	5.133739	2.84E-07	5.47E-07	black	0.410396047	9.39E-08		AT3G46450.2		SEC14 cytosolic factor family protein / phosphoglyceride transfer family protein	SEC14 cytosolic factor
Sapur.010G127900.1	523.8761169	-0.229765723	0.044758819	-5.13342	2.85E-07	5.48E-07	black	-0.394703508	3.13E-07		AT1G26750.1			hypothetical protein
Sapur.019G095500.1	740.1856047	-0.293109516	0.057255987	-5.11928	3.07E-07	5.90E-07	black	-0.390570214	4.26E-07	GO:0009231,GO:0004746	AT2G20690.1		lumazine-binding family protein	NA
Sapur.009G043900.1	1149.185085	-0.203864673	0.039863396	-5.11408	3.15E-07	6.05E-07	black	-0.434715798	1.28E-08	GO:0050662,GO:0003824	AT5G19440.1		NAD(P)-binding Rossmann-fold superfamily protein	cinnamyl-alcohol dehydrogenase-like protein
Sapur.014G040400.1	5559.872057	-0.244788276	0.047954464	-5.1046	3.31E-07	6.36E-07	black	-0.429381465	2.01E-08	GO:0006412,GO:0005840,GO:0005622,GO:0003735	AT5G23740.1	RPS11-BETA	ribosomal protein S11-beta	40S ribosomal protein S11
Sapur.005G166900.1	3564.011189	-0.261710152	0.051323628	-5.09921	3.41E-07	6.54E-07	black	-0.411119314	8.87E-08	GO:0006412,GO:0005840,GO:0005622,GO:0003735,GO:0003723	AT1G22780.1	PFL,PFL1,RPS18A	Ribosomal protein S13/S18 family	40S ribosomal protein S18
Sapur.005G034300.1	504.8451454	-0.579202519	0.113706511	-5.09384	3.51E-07	6.72E-07	black	-0.370545362	1.78E-06					hypothetical protein
Sapur.009G128200.1	647.5318287	-0.293956898	0.057752371	-5.08995	3.58E-07	6.86E-07	black	-0.400164973	2.07E-07	GO:0051536,GO:0009055	AT2G27510.1	ATFD3,FD3	ferredoxin 3	ferredoxin-3 protein
Sapur.005G164100.1	199.6018877	-0.334811425	0.066468132	-5.03717	4.72E-07	8.99E-07	black	-0.367929347	2.13E-06		AT3G52420.1	ATOEP7,OEP7	outer envelope membrane protein 7	transmembrane protein, putative
Sapur.016G210200.1	397.3928431	-0.261849147	0.05200709	-5.03487	4.78E-07	9.10E-07	black	-0.403171366	1.65E-07		AT5G14290.1		Mitochondrial ribosomal protein L37	ribosomal protein L37
Sapur.011G078500.1	648.425842	-0.208856011	0.041501736	-5.03246	4.84E-07	9.21E-07	black	-0.423431818	3.29E-08		AT2G33845.1		Nucleic acid-binding, OB-fold-like protein	OB fold SoSSB-OBF: A subfamily protein, putative
Sapur.016G215900.1	3312.28234	-0.303047504	0.060378177	-5.01916	5.19E-07	9.86E-07	black	-0.437454786	1.01E-08	GO:0006412,GO:0005840,GO:0005622,GO:0003735	AT4G15000.1		Ribosomal L27e protein family	60S ribosomal protein L27
Sapur.005G017300.1	640.0846187	-0.213297545	0.042585095	-5.00874	5.48E-07	1.04E-06	black	-0.439144074	8.74E-09	GO:0003723	AT1G09150.1		pseudouridine synthase and archaeosine transglycosylase (PUA) domain-containing protein	pseudouridine synthase and archaeosine transglycosylase domain protein
Sapur.011G109600.1	4353.46448	-0.280138104	0.056147185	-4.98935	6.06E-07	1.15E-06	black	-0.438946119	8.89E-09	GO:0019843,GO:0006412,GO:0005840,GO:0003735	AT1G33140.1	PGY2	Ribosomal protein L6 family	60S ribosomal protein L9
Sapur.006G222800.1	83.67841555	0.383471435	0.077464828	4.950265	7.41E-07	1.40E-06	black	0.410076115	9.63E-08		AT1G62930.1		Tetratricopeptide repeat (TPR)-like superfamily protein	PPR containing plant protein
Sapur.006G170400.1	197.0258649	-0.199202265	0.040405152	-4.93012	8.22E-07	1.55E-06	black	-0.362212923	3.14E-06	GO:0003676	AT5G06210.1		RNA binding (RRM/RBD/RNP motifs) family protein	RNA-binding protein 42 (RBM42)-like RNA recognition motif protein
Sapur.016G124100.2	5.076950714	-0.59490484	0.120769336	-4.92596	8.39E-07	1.58E-06	black	-0.365902064	2.45E-06					LIN37 protein
Sapur.009G115800.1	1977.42857	-0.39848056	0.081059984	-4.91587	8.84E-07	1.66E-06	black	-0.358880927	3.92E-06		AT3G44590.1		60S acidic ribosomal protein family	60S acidic ribosomal protein P2
Sapur.005G161800.1	110.2399083	-0.256611587	0.052223428	-4.91373	8.94E-07	1.68E-06	black	-0.383841545	6.96E-07		AT4G39235.1			NA
Sapur.008G013600.1	573.8847785	-0.394620169	0.080474958	-4.90364	9.41E-07	1.77E-06	black	-0.370083952	1.84E-06	GO:0006526,GO:0005524,GO:0004055	AT4G24830.1		arginosuccinate synthase family	argininosuccinate synthase
Sapur.006G057100.1	396.7462967	-0.220922928	0.045131906	-4.89505	9.83E-07	1.84E-06	black	-0.396044008	2.83E-07		AT5G40080.1		Mitochondrial ribosomal protein L27	ribosomal protein L27
Sapur.007G023700.2	1.863749474	-1.042971384	0.213172529	-4.89262	9.95E-07	1.87E-06	black	-0.567533569	9.16E-15		AT5G66770.1		GRAS family transcription factor	GRAS family transcription factor
Sapur.013G028300.1	2030.498227	-0.290674543	0.05999437	-4.84503	1.27E-06	2.36E-06	black	-0.400504053	2.02E-07		AT4G25740.1		RNA binding Plectin/S10 domain-containing protein	plectin/S10 domain protein
Sapur.013G014100.1	808.0851583	-0.321585204	0.066725704	-4.81951	1.44E-06	2.67E-06	black	-0.394316532	3.22E-07	GO:0006412,GO:0005840,GO:0005622,GO:0003735	AT3G05560.2		Ribosomal L22e protein family	60S ribosomal protein L22, putative
Sapur.019G070900.3	78.1979157	0.349905647	0.072973238	4.794986	1.63E-06	3.02E-06	black	0.40267291	1.71E-07		AT1G33780.1		Protein of unknown function (DUF179)	electron transporter, putative
Sapur.016G225900.1	3423.538544	-0.290653324	0.060924271	-4.77073	1.84E-06	3.40E-06	black	-0.408957887	1.05E-07	GO:0006412,GO:0005840,GO:0005622,GO:0003735	AT4G18100.1		Ribosomal protein L32e	60S ribosomal protein L32, putative
Sapur.T187400.1	55.20419024	0.23008632	0.04843759	4.75016	2.03E-06	3.75E-06	black	0.364709493	2.65E-06		AT1G59650.1	CW14	Protein of unknown function (DUF1336)	NA
Sapur.013G093700.1	110.8949189	-0.285435347	0.060213257	-4.74041	2.13E-06	3.93E-06	black	-0.369090649	1.97E-06		AT1G14060.1		GCK domain-containing protein	GCK domain protein
Sapur.004G170900.1	1206.988767	-0.157865101	0.033553189	-4.70492	2.54E-06	4.67E-06	black	-0.37198883	1.61E-06	GO:0016020	AT2G38550.1		Transmembrane proteins 14C	non-green plastid inner envelope membrane protein, putative
Sapur.009G086700.1	421.3007552	-0.300904692	0.063995644	-4.70196	2.58E-06	4.73E-06	black	-0.410689141	9.17E-08		AT1G49410.1	TOM6	translocase of the outer mitochondrial membrane 6	import receptor subunit TOM6-like protein
Sapur.005G187800.1	1032.110466	0.249461545	0.053255886	4.684206	2.81E-06	5.15E-06	black	0.338830101	1.42E-05		AT1G05150.1		Calcium-binding tetratricopeptide family protein	calcium-binding EF hand protein
Sapur.011G124900.1	661.8044255	0.218068188	0.047071194	4.632731	3.61E-06	6.58E-06	black	0.362344175	3.11E-06		AT5G56240.1			hypothetical protein
Sapur.006G065300.1	570.5505168	-0.215018736	0.04664011	-4.61017	4.02E-06	7.32E-06	black	-0.417066236	5.52E-08	GO:0006412,GO:0005840,GO:0005622,GO:0003735	AT3G18880.1		Nucleic acid-binding, OB-fold-like protein	30S ribosomal protein S17
Sapur.009G046900.1	123.1994402	-0.297480791	0.065006856	-4.57614	4.74E-06	8.59E-06	black	-0.332132753	2.14E-05					NA
Sapur.010G198600.1	2022.977001	-0.31620099	0.069169949	-4.57136	4.85E-06	8.78E-06	black	-0.385562596	6.14E-07	GO:0006412,GO:0005840,GO:0005622,GO:0003735	AT5G03850.1		Nucleic acid-binding, OB-fold-like protein	40S ribosomal protein S28
Sapur.15WG072700.1	244.986318	-0.286264684	0.062693627	-4.56609	4.97E-06	9.00E-06	black	-0.357459856	4.31E-06	GO:0003676	AT5G61030.1	GR-RBP3	glycine-rich RNA-binding protein 3	RNA recognition motif 2 in RNA-binding protein 28 (RBM28)-like protein
Sapur.003G077800.2	131.8291941	0.428545497	0.093968222	4.560536	5.10E-06	9.23E-06	black	0.356404865	4.62E-06		AT1G12240.1	ATBETAFRUCT4,VAC-INV	Glycosyl hydrolases family 32 protein	acid vacuolar invertase
Sapur.006G171600.1	2109.307271	-0.258441776	0.05718287	-4.51957	6.20E-06	1.12E-05	black	-0.390139935	4.40E-07	GO:0019843,GO:0005622,GO:0003723	AT5G39850.1		Ribosomal protein S4	NA
Sapur.005G195300.1	49.27851423	-0.438578539	0.097698169	-4.48912	7.15E-06	1.28E-05	black	-0.310624211	7.51E-05					NA
Sapur.011G104000.1	91.74854239	-0.319735278	0.071353723	-4.48099	7.43E-06	1.33E-05	black	-0.349635574	7.18E-06		AT3G18510.1			transmembrane protein, putative
Sapur.008G161900.1	70.80595973	-0.291540373	0.065277599	-4.46616	7.96E-06	1.43E-05	black	-0.313651417	6.33E-05		AT4G15820.1			hypothetical protein
Sapur.005G037700.1	1016.778246	0.16257746	0.036463942	4.458582	8.25E-06	1.48E-05	black	0.368832762	2.00E-06	GO:0008270,GO:0003676,GO:0004527	AT1G54490.1	AIN1,ATXRN4,EIN5,XRN4	exoribonuclease 4	5'-3' exoribonuclease
Sapur.014G114300.3	5.324368353	0.537159934	0.120692093	4.450664	8.56E-06	1.53E-05	black	0.377069679	1.13E-06	GO:0008152,GO:0003824	AT5G36880.2	ACS	acetyl-CoA synthetase	acetyl-CoA synthetase
Sapur.016G059100.1	765.3055341	-0.231044451	0.052030957	-4.44052	8.97E-06	1.60E-05	black	-0.327391099	2.85E-05	GO:0006412,GO:0005840,GO:0005622,GO:0003735	AT5G64140.1	RPS28	ribosomal protein S28	40S ribosomal protein S28
Sapur.005G021000.1	22.60449272	-0.509545492	0.115058179	-4.42859	9.49E-06	1.69E-05	black	-0.318054685	4.92E-05					hypothetical protein
Sapur.003G020300.2	83.39726742	-0.35088029	0.079692111	-4.40295	1.07E-05	1.90E-05	black	-0.320537448	4.26E-05	GO:0005515,GO:0042393,GO:0006355	AT2G02470.1	AL6	alfin-like 6	PHD finger ALFIN-like protein
Sapur.006G102000.1	820.0715931	-0.137132849	0.031498465	-4.35364	1.34E-05	2.37E-05	black	-0.364168183	2.75E-06		AT2G43630.1			transmembrane protein, putative
Sapur.006G207300.2	228.1655277	-0.367666389	0.084930434	-4.32903	1.50E-05	2.64E-05	black	-0.325079501	3.27E-05	GO:0005525	AT5G55190.1	ATRAN3,RAN3	RAN GTPase 3	GTP-binding nuclear protein Ran/TC4 protein
Sapur.010G133200.1	1180.096377	0.216292411	0.050873364	4.251585	2.12E-05	3.71E-05	black	0.355396497	4.93E-06	GO:0005524,GO:0003676,GO:0004386	AT5G13010.1	EMB3011	RNA helicase family protein	ATP-dependent RNA helicase, putative
Sapur.T063700.1	1352.387346	-0.411955754	0.097954226	-4.20559	2.60E-05	4.53E-05	black	-0.37952062	9.48E-07	GO:0006412,GO:0005840,GO:0005622,GO:0003735	AT3G59540.1		Ribosomal L38e protein family	60S ribosomal protein L38
Sapur.018G096900.1	53.41108505	-0.380036689	0.091208336	-4.16669	3.09E-05	5.35E-05	black	-0.314923229	5.89E-05					NA
Sapur.013G091200.1	3867.499798	-0.261202117	0.062699072	-4.16596	3.10E-05	5.37E-05	black	-0.376217113	1.20E-06	GO:0006412,GO:0005840,GO:0005622,GO:0003735	AT2G40590.1		Ribosomal protein S26e family protein	40S ribosomal protein S26
Sapur.016G293100.1	4884.374491	-0.266762171	0.064191887	-4.1557	3.24E-05	5.61E-05	black	-0.367311227	2.22E-06	GO:0019843,GO:0006412,GO:0005840,GO:0003735	AT4G10450.1		Ribosomal protein L6 family	60S ribosomal protein L9
Sapur.002G053100.2	3.563601973	-0.644560944	0.155130669	-4.15495	3.25E-05	5.63E-05	black	-0.483133612	1.47E-10					hypothetical protein
Sapur.019G060700.1	98.45564379	-0.293812614	0.070769243	-4.1517	3.30E-05	5.71E-05	black	-0.30205035	0.000120734	GO:0005737,GO:0003879,GO:0000287,GO:0000105	AT1G09795.1	ATATP-PRT2,ATP-PRT2,HISN	ATP phosphoribosyl transferase 2	ATP phosphoribosyltransferase
Sapur.15WG023200.1	3280.170523	-0.18274419	0.044397005	-4.11614	3.85E-05	6.63E-05	black	-0.316828791	5.28E-05	GO:0006412,GO:0005840,GO:0005622,GO:0003735	AT3G16780.1		Ribosomal protein L19e family protein	ribosomal protein L19
Sapur.016G292600.1	3055.671111	-0.234786775	0.057491714	-4.08384	4.43E-05	7.60E-05	black	-0.340122485	1.31E-05	GO:0019843,GO:0006412,GO:0005840,GO:0003735	AT1G33140.1	PGY2	Ribosomal protein L6 family	60S ribosomal protein L9
Sapur.016G222000.1	233.7630448	-0.214785237	0.052681132	-4.07708	4.56E-05	7.82E-05	black	-0.320759391	4.21E-05		AT5G14710.1			proteasome assembly chaperone 3-domain protein
Sapur.016G182700.1	1897.15829	-0.267474131	0.06576347	-4.06721	4.76E-05	8.15E-05	black	-0.353422774	5.62E-06	GO:0006412,GO:0005840,GO:0003735	AT5G18380.1		Ribosomal protein S5 domain 2-like superfamily protein	40S ribosomal protein S16
Sapur.005G001900.1	44.64956145	-0.253795783	0.062914037	-4.03401	5.48E-05	9.36E-05	black	-0.304064956	0.000108144					NA
Sapur.019G102900.1	239.0196708	-0.423895897	0.105293069	-4.02587	5.68E-05	9.68E-05	black	-0.279505028	0.000392426		AT4G28240.1		Wound-responsive family protein	DUF3774 domain protein
Sapur.005G194300.1	386.7249127	-0.215869293	0.05373937	-4.01697	5.90E-05	0.000100452	black	-0.350837261	6.64E-06	GO:0005515	AT5G42150.1		Glutathione S-transferase family protein	prostaglandin E synthase
Sapur.016G155500.1	1299.887673	-0.126957402	0.031780634	-3.9948	6.47E-05	0.00011005	black	-0.332979219	2.03E-05		AT2G03870.2		Small nuclear ribonucleoprotein family protein	U6 snRNA-associated-like-Smprotein LSm7
Sapur.016G161900.1	224.4053412	-0.22295844	0.056036785	-3.97879	6.93E-05	0.000117485	black	-0.333714873	1.95E-05		AT3G07910.1			reactive oxygen species modulator 1
Sapur.012G067500.1	144.4794843	-0.223536233	0.056715731	-3.94134	8.10E-05	0.000136846	black	-0.32179214	3.96E-05		AT5G63150.1			DUF1713 domain protein
Sapur.001G046400.2	149.5538752	0.299706402	0.076483817	3.91856	8.91E-05	0.000150027	black	0.330635947	2.34E-05	GO:0005515	AT1G27340.1		Galactose oxidase/kelch repeat superfamily protein	F-box only protein
Sapur.011G113600.1	1179.34922	0.274683256	0.071241458	3.855666	0.000115415	0.000193307	black	0.325026884	3.28E-05		AT2G35630.1	GEM1,MOR1	ARM repeat superfamily protein	microtubule organization protein
Sapur.013G087900.1	637.6322089	-0.115370741	0.030282272	-3.80984	0.000139054	0.000231785	black	-0.327146701	2.89E-05	GO:0005515	AT5G36230.1		ARM repeat superfamily protein	translation initiation factor
Sapur.006G108300.1	1492.846839	-0.252453431	0.066615953	-3.78968	0.000150839	0.000250847	black	-0.334097691	1.90E-05	GO:0006412,GO:0005840,GO:0005622,GO:0003735	AT5G02960.1		Ribosomal protein S12/S23 family protein	NA
Sapur.013G102800.1	421.1980226	-0.179551685	0.048082499	-3.73424	0.000188281	0.000311174	black	-0.299257944	0.00014046	GO:0006412,GO:0005840,GO:0005622,GO:0003735	AT3G20230.1		Ribosomal L18p/L5e family protein	50S ribosomal protein L18
Sapur.011G062000.3	0.87166412	-1.093824064	0.293970284	-3.72087	0.000198541	0.000327604	black	-0.435216849	1.23E-08	GO:0016021	AT1G78620.1		Protein of unknown function DUF92, transmembrane	integral membrane family protein
Sapur.15WG134700.1	360.2346426	-0.154262614	0.041524527	-3.71498	0.000203223	0.000335031	black	-0.294014849	0.000185852		AT1G31160.1	HINT 2	HISTIDINE TRIAD NUCLEOTIDE-BINDING 2	histidine triad nucleotide-binding protein
Sapur.004G140000.1	1435.706036	-0.31424038	0.084842139	-3.70382	0.000212373	0.000349541	black	-0.268261982	0.000681451		AT3G44590.1		60S acidic ribosomal protein family	60S acidic ribosomal protein P2
Sapur.001G097900.2	177.3329275	-0.311680381	0.084177915	-3.70264	0.000213369	0.000351145	black	-0.297072073	0.000157957		AT1G62780.1			subtilisin-like protease, putative
Sapur.003G028000.1	136.0574721	-0.170944038	0.046397714	-3.68432	0.000229314	0.000376524	black	-0.34196427	1.17E-05		AT1G15230.1			hypothetical protein
Sapur.005G073200.1	1632.613047	-0.157106571	0.043104809	-3.64476	0.000267644	0.000437225	black	-0.347871183	8.04E-06	GO:0006412,GO:0005840,GO:0005622,GO:0003735	AT4G14320.1		Zinc-binding ribosomal protein family protein	60S ribosomal protein L44
Sapur.010G016800.1	320.8673245	-0.196875555	0.054032364	-3.64362	0.000268827	0.000439071	black	-0.312725054	6.67E-05	GO:0006412,GO:0005840,GO:0003735	AT5G46160.1		Ribosomal protein L14p/L23e family protein	50S ribosomal protein L14
Sapur.007G121200.1	1582.391348	-0.222737989	0.061781409	-3.60526	0.000311842	0.000507347	black	-0.311926125	6.98E-05	GO:0016020	AT1G03860.3	ATPHB2,PHB2	prohibitin 2	prohibitin, putative
Sapur.003G153300.1	185.0154303	-0.187051642	0.052866259	-3.5382	0.000402858	0.000650814	black	-0.349042694	7.45E-06		AT3G07910.1			reactive oxygen species modulator 1
Sapur.002G180600.2	231.9419935	-0.293673944	0.083971569	-3.4973	0.000469989	0.000756287	black	-0.286058926	0.000281385	GO:0006412,GO:0005840,GO:0005622,GO:0003735	AT4G29390.1		Ribosomal protein S30 family protein	40S ribosomal protein S30
Sapur.013G010000.2	2.148357951	-0.693378056	0.199371857	-3.47781	0.000505522	0.000811712	black	-0.469991676	5.29E-10	GO:0003723,GO:0006412,GO:0005840,GO:0005622,GO:0003735	AT5G07090.1		Ribosomal protein S4 (RPS4A) family protein	40S ribosomal protein S4
Sapur.008G054600.1	495.4385829	-0.166658178	0.048030755	-3.46982	0.000520803	0.000835477	black	-0.27538272	0.000481748	GO:0005515,GO:0042393,GO:0006355	AT5G05610.1	AL1	alfin-like 1	PHD finger protein
Sapur.002G033100.1	3321.135306	-0.197199087	0.0571516	-3.45046	0.00055964	0.000895955	black	-0.33518524	1.78E-05	GO:0006412,GO:0005840,GO:0003735	AT5G09500.1		Ribosomal protein S19 family protein	40S ribosomal protein S15
Sapur.005G073300.3	6.681804363	-0.440084354	0.128112565	-3.43514	0.000592252	0.00094647	black	-0.289015628	0.000241531		AT5G23140.1	CLPP2,NCLPP7	nuclear-encoded CLP protease P7	ATP-dependent Clp protease proteolytic subunit
Sapur.006G062900.1	940.82912	-0.177648415	0.051715714	-3.4351	0.000592345	0.000946573	black	-0.314291406	6.11E-05	GO:0030150,GO:0005742	AT5G41685.1		Mitochondrial outer membrane translocase complex, subunit Tom7	import receptor subunit TOM7-1
Sapur.009G070500.1	958.98861	-0.241241501	0.070743222	-3.4101	0.000649389	0.001035329	black	-0.28301864	0.000328664	GO:0006412,GO:0005840,GO:0005622,GO:0003735	AT3G44010.1		Ribosomal protein S14p/S29e family protein	40S ribosomal protein S29
Sapur.016G193300.1	458.6533759	-0.126343114	0.038257612	-3.30243	0.000958507	0.001508885	black	-0.254238188	0.001313092		AT5G49210.1			plant/K21P3-8 protein, putative
Sapur.014G101900.1	780.2870881	-0.214203808	0.06496683	-3.29713	0.000976798	0.001536507	black	-0.320457433	4.28E-05	GO:0006412,GO:0005840,GO:0005622,GO:0003735	AT3G05590.1	RPL18	ribosomal protein L18	60S ribosomal protein L18
Sapur.010G015000.1	586.3661979	-0.220645993	0.067795592	-3.25458	0.001135613	0.001778711	black	-0.31425224	6.12E-05	GO:0006412,GO:0005840,GO:0005622,GO:0003735	AT3G06320.1		Ribosomal protein L33 family protein	50S ribosomal protein L33
Sapur.019G063100.1	297.0423243	-0.125190201	0.039303346	-3.18523	0.00144639	0.0022429	black	-0.239898462	0.002476344	GO:0006413,GO:0003743,GO:0003723	AT2G40780.1		Nucleic acid-binding, OB-fold-like protein	RNA-binding protein EIF1AD, putative
Sapur.008G005600.1	73.07556546	-0.207322957	0.065322633	-3.17383	0.001504416	0.00233645	black	-0.268414827	0.000676463		AT1G10865.1			cytochrome C oxidase assembly protein
Sapur.016G178800.1	196.0245535	-0.202108617	0.065098791	-3.10591	0.001896961	0.002924537	black	-0.257841509	0.001113203		AT2G15000.2			hypothetical protein
Sapur.002G096700.1	327.2920333	-0.206348858	0.066901711	-3.08436	0.002039916	0.003139662	black	-0.263778944	0.000843686	GO:0006412,GO:0005840,GO:0005622,GO:0003735	AT3G43980.1		Ribosomal protein S14p/S29e family protein	40S ribosomal protein S29
Sapur.005G056600.1	39.85211671	-0.281249026	0.091649904	-3.06873	0.002149691	0.003303544	black	-0.229250329	0.003875644		AT5G10320.1			hypothetical protein
Sapur.018G018500.1	797.0930406	-0.2309757	0.075610546	-3.05481	0.002252047	0.003456985	black	-0.245239187	0.001963505	GO:0006412,GO:0005840,GO:0005622,GO:0003735	AT4G31985.1		Ribosomal protein L39 family protein	NA
Sapur.002G200700.1	474.3284781	-0.235774762	0.077443973	-3.04446	0.002331016	0.003573562	black	-0.230952033	0.003612617	GO:0006412,GO:0005840,GO:0003735	AT3G04400.1	emb2171	Ribosomal protein L14p/L23e family protein	60S ribosomal protein L23
Sapur.010G140800.1	1305.846771	-0.208564938	0.068532636	-3.04329	0.002340039	0.003586895	black	-0.308780909	8.33E-05	GO:0006412,GO:0005840,GO:0005622,GO:0003735	AT2G43460.1		Ribosomal L38e protein family	60S ribosomal protein L38
Sapur.014G009000.1	1042.229461	-0.202799593	0.0677136	-2.99496	0.002744799	0.004185986	black	-0.323341598	3.62E-05	GO:0006412,GO:0005840,GO:0005622,GO:0003735	AT3G44010.1		Ribosomal protein S14p/S29e family protein	NA
Sapur.005G193000.1	227.8980688	-0.161183763	0.054736198	-2.94474	0.003232283	0.0049028	black	-0.2326378	0.003368016		AT4G24175.1			kinesin heavy chain isolog protein, putative
Sapur.003G040600.1	2901.037537	-0.13489511	0.04656587	-2.89687	0.003769101	0.005681825	black	-0.257376722	0.001137319	GO:0006412,GO:0005840,GO:0005622,GO:0003735,GO:0015935	AT3G04770.2	RPSAb	40s ribosomal protein SA B	40S ribosomal protein Sa protein
Sapur.003G025100.1	970.9311942	-0.201242379	0.07010738	-2.87049	0.00409839	0.006162491	black	-0.265625432	0.000772989	GO:0006412,GO:0005840,GO:0005622,GO:0003735	AT3G16080.1		Zinc-binding ribosomal protein family protein	60S ribosomal protein L37, putative
Sapur.010G091000.2	1.087972304	-0.757139304	0.267736998	-2.82792	0.004685124	0.007014431	black	-0.721354354	1.65E-26	GO:0005515	AT1G49540.1	ELP2	elongator protein 2	elongator complex protein
Sapur.013G019700.1	1.813359113	-0.685117195	0.245429711	-2.7915	0.005246425	0.007815525	black	-0.250440808	0.001558797					hypothetical protein
Sapur.003G088200.1	2863.64318	-0.15008633	0.053946586	-2.78213	0.005400367	0.008034716	black	-0.230026829	0.003753557		AT2G36620.1	RPL24A	ribosomal protein L24	60S ribosomal protein L24
Sapur.009G007300.1	233.4083166	-0.105473717	0.037957864	-2.77871	0.005457601	0.008115124	black	-0.256271275	0.00119661		AT2G28370.1		Uncharacterised protein family (UPF0497)	CASP-like plant protein
Sapur.016G279900.1	820.3664279	-0.117191331	0.043332359	-2.70448	0.006841219	0.010082727	black	-0.303650961	0.000110626	GO:0006412,GO:0005840,GO:0005622,GO:0003735	AT2G34520.1	RPS14	mitochondrial ribosomal protein S14	ribosomal protein S14
Sapur.006G072900.1	6035.090348	-0.127096839	0.047191175	-2.69323	0.007076276	0.010418945	black	-0.261690129	0.00093079		AT1G70600.1		Ribosomal protein L18e/L15 superfamily protein	60S ribosomal protein L27a
Sapur.003G019200.2	2307.402496	-0.211051883	0.081364039	-2.59392	0.009488831	0.013807021	black	-0.22974092	0.003798102	GO:0006412,GO:0005840,GO:0005622,GO:0003735	AT2G19730.3		Ribosomal L28e protein family	60S ribosomal protein L28
Sapur.005G168900.1	169.3716605	0.182433687	0.070651883	2.582149	0.009818719	0.014268831	black	0.237021457	0.002800316		AT1G03080.1		kinase interacting (KIP1-like) family protein	kinase interacting-like protein
Sapur.017G000900.1	2219.211415	-0.091858314	0.035974081	-2.55346	0.010665905	0.015452457	black	-0.247185344	0.00180206	GO:0009349,GO:0009231	AT2G44050.1	COS1	6,7-dimethyl-8-ribityllumazine synthase / DMRL synthase / lumazine synthase / riboflavin synthase	6%2C7-dimethyl-8-ribityllumazine synthase
Sapur.005G051400.2	6.709616303	-0.283258182	0.114574512	-2.47226	0.013426118	0.019247341	black	-0.444324996	5.56E-09	GO:0006355,GO:0003713,GO:0003677	AT5G09250.1	KIWI	ssDNA-binding transcriptional regulator	RNA polymerase II transcriptional coactivator KIWI protein, putative
Sapur.005G039500.1	649.8622297	-0.111583382	0.047057929	-2.37119	0.01773082	0.025101839	black	-0.167751699	0.035726095	GO:0006412,GO:0005840,GO:0005622,GO:0003735	AT4G34670.1		Ribosomal protein S3Ae	40S ribosomal protein S3a
Sapur.006G031100.1	302.0997937	-0.085365168	0.036108014	-2.36416	0.018070943	0.02556038	black	-0.223801817	0.004837638	GO:0055114,GO:0006561,GO:0004735	AT5G14800.1	AT-P5C1,AT-P5R,EMB2772,I	pyrroline-5- carboxylate (P5C) reductase	pyrroline-5-carboxylate reductase
Sapur.004G112200.1	535.0583448	-0.139166089	0.060375735	-2.305	0.021166556	0.029764569	black	-0.263632845	0.000849526		AT1G49410.1	TOM6	translocase of the outer mitochondrial membrane 6	import receptor subunit TOM6-like protein
Sapur.017G114400.1	164.2004753	-0.205102837	0.092625092	-2.21433	0.026805851	0.037326402	black	-0.156941096	0.049653894		AT1G74520.1	ATHVA22A,HVA22A	HVA22 homologue A	TB2/DP1, HVA22 family protein
Sapur.15WG005600.1	3916.609127	-0.131456127	0.060171929	-2.18468	0.028912661	0.040121564	black	-0.231927846	0.003469148	GO:0006412,GO:0005840,GO:0003735	AT3G52590.1	EMB2167,ERD16,HAP4,UBC	ubiquitin extension protein 1	ubiquitin-60S ribosomal L40 fusion protein
Sapur.013G142200.1	6633.300175	-0.09721456	0.045877762	-2.11899	0.034091237	0.046945357	black	-0.280269707	0.000377645	GO:0006412,GO:0005840,GO:0003735	AT1G26910.1	RPL10B	Ribosomal protein L16p/L10e family protein	60S ribosomal protein L10
Sapur.016G281700.1	1344.292089	-0.084012154	0.041097981	-2.04419	0.040934607	0.05589197	black	-0.188251511	0.018221528		AT3G20390.1		endoribonuclease L-PSP family protein	perchloric acid soluble translation inhibitor protein
Sapur.012G011700.1	87.41016122	-0.173031406	0.085290544	-2.02873	0.042485892	0.057895364	black	-0.168034005	0.035411923		AT5G53045.1			transcription factor Pcc1
Sapur.001G155500.1	320.3524492	-0.13307125	0.066642397	-1.9968	0.045847384	0.062235024	black	-0.213653918	0.007214449					hypothetical protein
Sapur.004G162400.1	389.954176	-0.136609915	0.068752698	-1.98698	0.04692513	0.06362749	black	-0.181107705	0.023210987		AT4G29735.1			transmembrane protein, putative
Sapur.002G040800.1	225.6060518	-0.083720646	0.04257168	-1.96658	0.049231564	0.066588342	black	-0.177175987	0.026428193		AT3G63170.1		Chalcone-flavanone isomerase family protein	chalcone-flavanone isomerase
Sapur.005G108200.1	1502.736061	-0.080299946	0.041382716	-1.94042	0.052328387	0.070589325	black	-0.17728606	0.026330598		AT3G57870.1	AHUS5,ATSCE1,EMB1637,SC	sumo conjugation enzyme 1	SUMO E2 conjugating enzyme SCE1
Sapur.008G003900.1	1557.769235	-0.091024997	0.050360141	-1.80748	0.070687322	0.093899609	black	-0.177344145	0.026283165	GO:0006412,GO:0005840,GO:0005622,GO:0003735	AT3G23390.1		Zinc-binding ribosomal protein family protein	60S ribosomal protein L44
Sapur.009G053500.1	616.1600229	0.125015528	0.073953696	1.690457	0.090940597	0.118997044	black	0.081590256	0.309701642	GO:0006457,GO:0005737	AT1G14980.1	CPN10	chaperonin 10	10 kDa chaperonin
Sapur.019G073500.2	0.909962942	-0.42417058	0.277031755	-1.53113	0.125738204	0.161627104	black	-0.238453765	0.002634522	GO:0008616,GO:0008479,GO:0006400				NA
Sapur.004G051300.1	2745.631764	-0.071289278	0.046581556	-1.53042	0.125913155	0.161833121	black	-0.19319711	0.015338196		AT4G25740.1		RNA binding Plectin/S10 domain-containing protein	plectin/S10 domain protein
Sapur.005G061000.1	499.2217902	0.083263605	0.054576308	1.525636	0.127100431	0.163276612	black	0.077486963	0.334744472	GO:0005515	AT2G35605.1		SWIB/MDM2 domain superfamily protein	SWIB/MDM2 domain protein
Sapur.014G138000.1	379.7167402	0.069592773	0.047249057	1.472892	0.140780075	0.179843963	black	0.074390488	0.354474399		AT2G04340.1			hypothetical protein
Sapur.016G227200.1	306.9524965	0.068860602	0.051119312	1.347057	0.177962034	0.223620543	black	0.037308714	0.642717413		AT5G55125.2		Ribosomal protein L31	ribosomal protein L31
Sapur.003G115400.1	158.4865491	-0.08701353	0.064921207	-1.34029	0.180149654	0.22619755	black	-0.151379698	0.058418232		AT4G20150.1			complex I subunit
Sapur.014G050100.1	1202.283742	-0.086905081	0.065023969	-1.33651	0.181383156	0.227642641	black	-0.144819196	0.070353063	GO:0006412,GO:0005840,GO:0005622,GO:0003735	AT3G60770.1		Ribosomal protein S13/S15	40S ribosomal protein S13
Sapur.010G094300.1	215.8915941	-0.08719328	0.065580115	-1.32957	0.183660357	0.230247239	black	-0.137315641	0.086355461	GO:0006950	AT1G68300.1		Adenine nucleotide alpha hydrolases-like superfamily protein	universal stress family protein
Sapur.006G205900.1	350.0226772	-0.057049359	0.043632089	-1.30751	0.191039819	0.238873804	black	-0.180970196	0.023317546	GO:0008080	AT5G11340.1		Acyl-CoA N-acyltransferases (NAT) superfamily protein	N-acetyltransferase NAT13
Sapur.008G139300.1	714.7480261	-0.055762873	0.044861485	-1.243	0.21386745	0.26544026	black	-0.144888635	0.070217083	GO:0006413,GO:0005737,GO:0003743,GO:0003723	AT5G35620.1	EIF(ISO)4E,EIF4E2,eIFiso4E,L	Eukaryotic translation initiation factor 4E protein	eukaryotic translation initiation factor iso4E
Sapur.002G138500.1	8105.753808	-0.060413476	0.049066845	-1.23125	0.218229968	0.270499492	black	-0.177614054	0.026051807		AT5G24510.1		60S acidic ribosomal protein family	60S acidic ribosomal protein P1
Sapur.014G083200.1	1134.879423	-0.079892779	0.065789048	-1.21438	0.224603374	0.277889121	black	-0.141516768	0.07707113		AT5G24510.1		60S acidic ribosomal protein family	60S acidic ribosomal protein P1
Sapur.002G120200.1	1485.441744	0.078207139	0.065685568	1.191505	0.23345552	0.287904391	black	0.067080324	0.403865149	GO:0006412,GO:0005840,GO:0005622,GO:0003735	AT3G60770.1		Ribosomal protein S13/S15	40S ribosomal protein S13
Sapur.013G073100.1	96.44094307	-0.067107032	0.061030139	-1.09957	0.271518651	0.331030959	black	-0.451600514	2.91E-09	GO:0004527,GO:0003676	AT1G75660.1	AtXRN3,XRN3	5\'-3\' exoribonuclease 3	5'-3' exoribonuclease
Sapur.004G130500.1	988.9819738	-0.04400194	0.04454412	-0.98783	0.323236771	0.388570238	black	-0.105375711	0.189027053	GO:0019773,GO:0006511,GO:0004175,GO:0051603,GO:0005839,GO:0004298	AT5G66140.1	PAD2	proteasome alpha subunit D2	proteasome subunit alpha type-7 protein
Sapur.002G070800.1	403.517282	-0.051608161	0.056876764	-0.90737	0.364212175	0.432898392	black	-0.14945521	0.061732721	GO:0006412,GO:0005840,GO:0005622,GO:0003735,GO:0003723	AT1G77750.1		Ribosomal protein S13/S18 family	NA
Sapur.003G023000.2	1.556543404	-0.203107747	0.228052138	-0.89062	0.373133178	0.442389622	black	-0.49643898	3.79E-11		AT1G55080.1	MED9		hypothetical protein
Sapur.016G190900.1	113.1673651	-0.046904345	0.05804195	-0.80811	0.419026617	0.490862771	black	-0.075377446	0.348108279		AT5G55125.1		Ribosomal protein L31	ribosomal protein L31
Sapur.018G057500.1	787.660229	-0.025121798	0.034993445	-0.7179	0.472818888	0.547166267	black	-0.066678387	0.406694105		AT3G24506.1			hypothetical protein

Sapur.016G092900.2	0.938006937	0.177020228	0.264394868	0.66953	0.503157614	0.578048384	black	0.450091242	3.34E-09		AT2G37840.1		Protein kinase superfamily protein	Serine/Threonine kinase domain protein
Sapur.010G150400.1	1535.056315	-0.020969524	0.034467194	-0.60839	0.542928302	0.618496561	black	-0.061595467	0.44346831	GO:0005515	AT2G39990.1	AteIF3f,EIF2,eIF3F	eukaryotic translation initiation factor 2	eukaryotic translation initiation factor 3 subunit F
Sapur.011G072000.1	2232.532247	-0.027798565	0.053176403	-0.52276	0.601140388	0.675343654	black	-0.123154491	0.124380073	GO:0019843,GO:0005622,GO:0003723	AT5G39850.1		Ribosomal protein S4	40S ribosomal protein S9
Sapur.016G224900.1	1176.123665	-0.018632797	0.037597365	-0.49559	0.62018523	0.693558961	black	-0.10637725	0.184833887	GO:0003677	AT1G29850.1		double-stranded DNA-binding family protein	double-stranded DNA-binding protein
Sapur.012G081400.2	5.362888845	-0.073269098	0.148846683	-0.49225	0.622545857	0.695681376	black	-0.457978938	1.63E-09		AT4G25080.3	CHLM	magnesium-protoporphyrin IX methyltransferase	Mg-protoporphyrin IX methyltransferase
Sapur.004G102800.2	252.3960176	0.03468463	0.070559942	0.491563	0.623028576	0.696056179	black	0.017994247	0.823003469	GO:0006412,GO:0005840,GO:0003735	AT3G52580.1		Ribosomal protein S11 family protein	40S ribosomal protein S14
Sapur.009G021600.1	390.1797058	-0.027756091	0.056552355	-0.4908	0.623565473	0.696538365	black	-0.077674271	0.333573913		AT1G47278.2			histone-lysine N-methyltransferase ATX2, putative
Sapur.009G127500.1	630.0346907	0.020746083	0.045558276	0.455375	0.648839738	0.720899137	black	-0.009721369	0.90381945		AT2G27730.1		copper ion binding	F1F0-ATPase inhibitor protein, putative
Sapur.011G082100.1	146.0014297	-0.025952013	0.077231166	-0.33603	0.736848006	0.801635175	black	-0.068111903	0.396658175	GO:0016531,GO:0006825,GO:0005758,GO:0005507	AT3G15352.1	ATCOX17,COX17	cytochrome c oxidase 17	cytochrome C oxidase copper chaperone
Sapur.011G082900.2	0.168013728	-0.156914504	0.601818058	-0.26073	0.794297549	0.851479001	black	-0.32267435	3.76E-05	GO:0003723	AT1G53120.1		RNA-binding S4 domain-containing protein	RNA-binding S4 domain protein
Sapur.017G028300.1	124.0940256	0.015171437	0.060135113	0.252289	0.800817567	0.857048933	black	-0.020742293	0.796521869	GO:0005680,GO:0004842	AT3G05870.1	APC11	anaphase-promoting complex/cyclosome 11	anaphase-promoting complex subunit 11 RING-H2 finger protein
Sapur.010G010900.1	31.79963009	-0.021502264	0.101023199	-0.21284	0.831448012	0.881134017	black	-0.537275931	4.03E-13	GO:0016021	AT2G42770.1		Peroxisomal membrane 22 kDa (Mpv17/PMP22) family protein	peroxisomal membrane protein, putative
Sapur.007G065100.2	0.449658237	0.066400046	0.359443168	0.18473	0.853440575	0.899340266	black	0.421375881	3.89E-08	GO:0006468,GO:0005524,GO:0004672	AT3G13530.1	MAP3KE1,MAPKKK7	mitogen-activated protein kinase kinase kinase 7	MAP3K epsilon kinase
Sapur.016G208600.1	392.2853166	-0.005405213	0.038977262	-0.13868	0.889706146	0.925961832	black	-0.02651544	0.741671592	GO:0006412,GO:0005840,GO:0003735	AT3G01790.1		Ribosomal protein L13 family protein	50S ribosomal protein L13
Sapur.005G038200.1	2763.760579	0.006850139	0.053127168	0.128939	0.897406293	0.931104424	black	-0.04627911	0.564919217	GO:0006412,GO:0005840,GO:0005622,GO:0003735	AT5G28060.1		Ribosomal protein S24e family protein	40S ribosomal protein S24
Sapur.011G077200.2	0.054607761	-0.179215633	1.53985739	-0.11638	0.907347767	0.938985316	black	-0.610849444	1.98E-17		AT1G31660.1			bystin, putative
Sapur.008G110400.2	2.928481777	0.009486712	0.171113592	0.055441	0.955787118	0.971024517	black	-0.338606277	1.44E-05	GO:0055114.GO:0051287.GO:0016620.GO:0046983.GO:0008652.GO:0005737.GO:000394	AT1G14810.1		semialdehyde dehydrogenase family protein	aspartate-semialdehyde dehydrogenase
Sapur.010G059100.5	0.043671962	0.085365484	1.690735716	0.05049	0.959731806	NA	black	0.7143987	8.21E-26	GO:0019901,GO:0006355,GO:0000079	AT4G19600.1	CYCT1;4	Cyclin family protein	cyclin T1
Sapur.009G038100.2	0.004112536	0.128661719	2.904823276	0.044292	0.964671295	NA	black	-0.218930761	0.005873258					NAD(P)H-quinone oxidoreductase chain, putative
Sapur.011G110100.2	0.011519773	0.091189602	2.904823276	0.031392	0.97495654	NA	black	-0.419889062	4.39E-08	GO:0016021,GO:0016020,GO:0022857	AT1G43650.1		nodulin MtN21 /EamA-like transporter family protein	60S ribosomal protein L9
Sapur.016G154200.2	20.11210769	5.089205813	0.204810254	24.84839	2.69E-136	4.65E-135	brown	0.104423736	0.193076692		AT5G13490.1	AAC2	ADP/ATP carrier 2	ADP%2CATP carrier protein
Sapur.013G028600.3	7.407332774	-3.872324023	0.174820794	-22.1502	1.04E-108	1.31E-107	brown	-0.3664656	2.35E-06	GO:0004871,GO:0000160	AT5G39340.1	AHP3,ATHP2	histidine-containing phosphotransmitter 3	histidine phosphotransfer protein
Sapur.011G036800.1	175.289554	0.894454241	0.040934567	21.85083	7.63E-106	9.38E-105	brown	-0.056250211	0.484092701		AT1G28240.1		Protein of unknown function (DUF616)	transmembrane protein, putative
Sapur.016G089100.4	26.13937444	-2.214361312	0.101785736	-21.7551	6.18E-105	7.50E-104	brown	-0.314136058	6.16E-05	GO:0006457	AT3G10060.1		FKBP-like peptidyl-prolyl cis-trans isomerase family protein	peptidyl-prolyl cis-trans isomerase FKBP16-4
Sapur.008G118300.3	7.470513405	-2.257119349	0.127970299	-17.6378	1.26E-69	9.43E-69	brown	-0.081608864	0.309590942	GO:0055085,GO:0016020,GO:0006810,GO:0005215	AT1G60030.1	ATNAT7,NAT7	nucleobase-ascorbate transporter 7	nucleobase ascorbate transporter
Sapur.007G031600.1	12.45279231	-1.84743549	0.108283196	-17.0611	2.89E-65	2.02E-64	brown	-0.690976758	1.31E-23		AT5G66560.1		Phototropic-responsive NPH3 family protein	root phototropism protein
Sapur.005G089100.3	34.8936508	1.270844213	0.075625063	16.80454	2.26E-63	1.54E-62	brown	0.039535467	0.62299391		AT2G17840.1	ERD7	Senescence/dehydration-associated protein-related	senescence-associated protein
Sapur.004G119100.3	46.36407232	1.135572914	0.068112667	16.67198	2.10E-62	1.40E-61	brown	0.011123546	0.890028513		AT4G39140.3		RING/U-box superfamily protein	mandelonitrile lyase
Sapur.006G154400.5	27.09572236	1.32995315	0.080164075	16.59039	8.18E-62	5.43E-61	brown	0.007822713	0.92253856		AT5G35180.2		Protein of unknown function (DUF1336)	plant/T25C13-60 protein
Sapur.003G158500.3	6.10499613	-2.774736339	0.173277255	-16.0133	1.03E-57	6.42E-57	brown	-0.104663231	0.192052003		AT4G31980.1			transmembrane protein, putative
Sapur.003G164100.1	12.10405694	-3.881390518	0.251070783	-15.4593	6.53E-54	3.82E-53	brown	-0.493060176	5.37E-11		AT1G55230.1		Family of unknown function (DUF716)	proteinase inhibitor I4, serpin%2C putative
Sapur.019G005700.1	16.05234372	-2.47724064	0.161652993	-15.3244	5.25E-53	3.03E-52	brown	-0.128085574	0.109892892		AT2G02130.1	LCR68,PDF2.3	low-molecular-weight cysteine-rich 68	gamma-thionin family protein
Sapur.002G193300.4	6.468388113	-2.206653229	0.146774289	-15.0343	4.37E-51	2.44E-50	brown	-0.081608864	0.309590942		AT5G48545.1	HINT3	histidine triad nucleotide-binding 3	HIT family: HIT (histidine triad) family protein
Sapur.016G128500.5	62.98787652	0.911935161	0.062925457	14.49231	1.36E-47	7.12E-47	brown	-0.042566126	0.59657657	GO:0055085	AT5G59740.1		UDP-N-acetylglucosamine (UAA) transporter family	adenosine 3'-phospho 5'-phosphosulfate transporter
Sapur.15ZG052800.3	6.567211646	1.663903484	0.117723285	14.13402	2.34E-45	1.19E-44	brown	0.029442002	0.714336253	GO:0005515	AT5G07910.1		Leucine-rich repeat (LRR) family protein	LRR and ubiquitin-like domain protein
Sapur.004G019300.3	6.657795226	1.706347233	0.121723048	14.01828	1.21E-44	6.03E-44	brown	-0.025018857	0.755778587	GO:0046983	AT5G54680.1	bHLH105,ILR3	basic helix-loop-helix (bHLH) DNA-binding superfamily protein	BHLH transcription factor
Sapur.018G042000.1	420.08832	-0.824039013	0.060127315	-13.7049	9.49E-43	4.58E-42	brown	-0.645834311	6.69E-20	GO:0046872	AT4G29190.1		Zinc finger C-x8-C-x5-C-x3-H type family protein	zinc finger CCCH domain protein
Sapur.014G115100.4	28.0652958	0.934147271	0.071439987	13.07597	4.52E-39	2.05E-38	brown	-0.081608864	0.309590942	GO:0046912	AT2G42790.1	CSY3	citrate synthase 3	citrate synthase 2, peroxisomal protein
Sapur.001G085800.2	54.73949164	-0.805395803	0.061978884	-12.9947	1.31E-38	5.88E-38	brown	-0.108114977	0.177720921	GO:0055114,GO:0016491,GO:0016209,GO:0006979,GO:0004602	AT1G63460.1	ATGPX8,GPX8	glutathione peroxidase 8	glutathione peroxidase
Sapur.018G006600.1	3.604903384	3.280229898	0.254103395	12.90904	4.00E-38	1.78E-37	brown	0.207459493	0.009130859	GO:0005515	AT3G25460.1		F-box and associated interaction domains-containing protein	NA
Sapur.005G185700.1	10.06184381	1.928085725	0.14959529	12.88868	5.21E-38	2.31E-37	brown	-0.081608864	0.309590942	GO:0030246,GO:0005975,GO:0004553	AT4G36360.1	BGAL3	beta-galactosidase 3	beta-galactosidase
Sapur.003G025900.2	43.02627582	0.724595709	0.057766022	12.54363	4.31E-36	1.84E-35	brown	-0.056621112	0.481210807		AT1G79830.2	GC5	golgin candidate 5	coiled-coil vesicle tethering protein, putative
Sapur.014G006500.2	18.64797595	-1.343676634	0.108190088	-12.4196	2.05E-35	8.62E-35	brown	-0.597010894	1.56E-16					NA
Sapur.012G081600.4	10.2326739	1.745450889	0.143507184	12.16281	4.90E-34	2.01E-33	brown	-0.081608864	0.309590942	GO:0003676	AT5G07290.1	AML4,ML4	MEI2-like 4	RNA recognition motif 1 in plant Mei2-like protein
Sapur.019G094600.3	12.67125867	-1.328703768	0.109296629	-12.1569	5.27E-34	2.16E-33	brown	-0.081608864	0.309590942		AT5G10460.1		Haloacid dehalogenase-like hydrolase (HAD) superfamily protein	HAD-family hydrolase IIA
Sapur.012G102900.4	11.18243534	-1.342779893	0.111320886	-12.0622	1.67E-33	6.78E-33	brown	-0.125984542	0.115895294		AT5G52030.2		TraB family protein	TraB family protein
Sapur.014G106200.1	56.43837115	1.673102669	0.13938853	12.00316	3.42E-33	1.38E-32	brown	0.078548531	0.32814497	GO:0071805,GO:0016020,GO:0015079	AT4G13420.1	ATHAK5,HAK5	high affinity K+ transporter 5	potassium transporter
Sapur.006G081800.3	7.210091227	-1.334196357	0.111538236	-11.9618	5.63E-33	2.26E-32	brown	-0.081608864	0.309590942		AT5G01470.2		S-adenosyl-L-methionine-dependent methyltransferases superfamily protein	S-adenosyl-L-methionine-dependent methyltransferase family protein
Sapur.017G103100.3	4.849096319	-1.705409429	0.144021338	-11.8414	2.39E-32	9.46E-32	brown	-0.323841054	3.51E-05	GO:0006418,GO:0000166,GO:0005524,GO:0004812	AT5G16715.1	EMB2247	ATP binding;valine-tRNA ligases;aminoacyl-tRNA ligases;nucleotide binding;ATP binding;aminoacyl-tRNA ligases	valyl-tRNA synthetase
Sapur.016G105100.1	19.43679081	-0.738102558	0.062350099	-11.838	2.48E-32	9.84E-32	brown	-0.347170043	8.40E-06	GO:0033699,GO:0006281,GO:0003677	AT5G01310.1	APTX	APRATAXIN-like	transcription factor bHLH140
Sapur.010G003700.1	9.981794354	-2.221035689	0.188579705	-11.7777	5.09E-32	2.00E-31	brown	-0.394296514	3.23E-07	GO:0006412,GO:0005840,GO:0005622,GO:0003735	AT1G48350.1		Ribosomal L18p/L5e family protein	50S ribosomal protein L18
Sapur.008G055600.3	18.93729764	-1.038151253	0.088948886	-11.6713	1.79E-31	6.94E-31	brown	-0.188364638	0.018150656	GO:0005680	AT5G05560.1	EMB2771	E3 ubiquitin ligase, putative	anaphase-promoting complex subunit 1
Sapur.016G008100.3	2.666738827	-2.231096825	0.192361851	-11.5984	4.20E-31	1.62E-30	brown	-0.111472864	0.164551558		AT5G47530.1		Auxin-responsive family protein	auxin-induced in root cultures protein, putative
Sapur.009G004800.3	22.52130068	0.878558699	0.076745309	11.44772	2.41E-30	9.14E-30	brown	0.096186009	0.230778064		AT5G60760.1		P-loop containing nucleoside triphosphate hydrolases superfamily protein	2-phosphoglycerate kinase
Sapur.002G052400.2	1.205021318	3.652548882	0.331877995	11.0057	3.59E-28	1.30E-27	brown	0.15692963	0.049670772		AT2G21770.1	CESA09,CESA9	cellulose synthase A9	cellulose synthase A catalytic subunit 6 [UDP-forming] protein, putative
Sapur.010G004300.4	15.39924264	-1.055367116	0.095964687	-10.9975	3.93E-28	1.42E-27	brown	-0.081608864	0.309590942		AT3G16810.1	APUM24,PUM24	pumilio 24	pumilio domain protein
Sapur.T192500.1	9.564834821	-1.450590818	0.133566706	-10.8604	1.78E-27	6.35E-27	brown	-0.147918636	0.06448844		AT2G02130.1	LCR68,PDF2.3	low-molecular-weight cysteine-rich 68	NA
Sapur.15WG014600.1	9.036988177	-0.979086914	0.095222573	-10.2821	8.49E-25	2.84E-24	brown	-0.618309895	6.24E-18		AT5G53130.1	ATCNGC1,CNGC1	cyclic nucleotide gated channel 1	NA
Sapur.009G063200.3	2.728562232	1.847192529	0.179868708	10.26967	9.65E-25	3.23E-24	brown	0.003350686	0.966778834		AT4G37880.1		LisH/CRA/RING-U-box domains-containing protein	sporulation protein RMD5, putative
Sapur.15WG005700.1	16.14218136	-1.4583163	0.142331273	-10.2459	1.23E-24	4.11E-24	brown	-0.439083789	8.79E-09	GO:0005975	AT5G24400.1	EMB2024,PGL3	NagB/RpiA/CoA transferase-like superfamily protein	6-phosphogluconolactonase, putative
Sapur.15WG116800.1	2.545456599	-2.314802267	0.227998831	-10.1527	3.22E-24	1.06E-23	brown	-0.081608864	0.309590942	GO:0016597,GO:0008152	AT1G69040.2	ACR4	ACT domain repeat 4	ACT domain protein, putative
Sapur.002G073100.3	26.81799	0.733343714	0.072343045	10.13703	3.78E-24	1.25E-23	brown	0.089594416	0.26446936	GO:0006914	AT4G08540.1		DNA-directed RNA polymerase II protein	DNA-directed RNA polymerase II
Sapur.008G109700.4	26.08242929	-0.740940245	0.073780485	-10.0425	9.91E-24	3.22E-23	brown	-0.081608864	0.309590942		AT1G68070.1		Zinc finger, C3HC4 type (RING finger) family protein	cleavage and polyadenylation specifity factor, putative
Sapur.T096700.3	70.79858429	-0.609606421	0.060784935	-10.0289	1.14E-23	3.69E-23	brown	-0.195606618	0.014083643	GO:0043531	AT3G14470.1		NB-ARC domain-containing disease resistance protein	NA
Sapur.007G102000.1	6.722745775	-3.743896304	0.374780179	-9.98958	1.69E-23	5.47E-23	brown	-0.373876042	1.41E-06					NA
Sapur.002G036900.16	10.38387366	-0.985510596	0.098883203	-9.96641	2.14E-23	6.89E-23	brown	-0.081608864	0.309590942		AT4G14720.1	PPD2,TIFY4B	TIFY domain/Divergent CCT motif family protein	jasmonate ZIM domain protein K.1, putative
Sapur.016G132400.2	13.27875354	-0.947437385	0.095879314	-9.88156	5.00E-23	1.60E-22	brown	-0.081608864	0.309590942	GO:0005525	AT1G07615.1		GTP-binding protein Obg/CgtA	NA
Sapur.008G002300.1	796.1795323	-0.639779564	0.066521135	-9.61769	6.73E-22	2.09E-21	brown	-0.526020898	1.50E-12	GO:0016829,GO:0006520	AT5G65720.1	ATNFS1.ATNIFS1.NFS1.NIFS	nitrogen fixation S (NIFS)-like 1	cysteine desulfurase
Sapur.017G113400.1	14.00826316	-1.083378471	0.113258891	-9.5655	1.12E-21	3.46E-21	brown	-0.495621484	4.12E-11	GO:0043531	AT3G14470.1		NB-ARC domain-containing disease resistance protein	CC-NBS-LRR resistance protein
Sapur.017G003000.3	3.906718915	-1.917169784	0.207211401	-9.25224	2.20E-20	6.58E-20	brown	-0.173550013	0.02972609	GO:0008168,GO:0008152	AT2G43940.1	ATHOL3,HOL3	S-adenosyl-L-methionine-dependent methyltransferases superfamily protein	thiopurine S-methyltransferase
Sapur.016G284100.1	5.916749301	-0.982032258	0.10677759	-9.19699	3.68E-20	1.09E-19	brown	-0.134663372	0.092663579	GO:0043531	AT4G27220.1		NB-ARC domain-containing disease resistance protein	NA
Sapur.002G192000.2	5.941338399	1.584194455	0.176359544	8.982754	2.64E-19	7.69E-19	brown	-0.115779006	0.148746091	GO:0006468,GO:0005524,GO:0004672	AT4G13020.1	MHK	Protein kinase superfamily protein	cyclin-dependent kinase-like Serine/Threonine kinase family protein
Sapur.018G063900.1	11.66811148	-0.998714768	0.112908818	-8.84532	9.13E-19	2.62E-18	brown	-0.536454292	4.44E-13	GO:0016887,GO:0005524,GO:0055085,GO:0042626,GO:0016021,GO:0006810	AT3G28345.1		ABC transporter family protein	NA
Sapur.010G041300.1	14.39693761	-1.524425557	0.173284165	-8.79726	1.40E-18	4.00E-18	brown	-0.282633559	0.000335151		AT1G07090.1	LSH6	Protein of unknown function (DUF640)	G1-like protein, putative
Sapur.005G032900.1	1.857028037	-2.278136472	0.261648786	-8.70685	3.12E-18	8.84E-18	brown	-0.361452552	3.30E-06		AT5G27230.1		Frigida-like protein	NA
Sapur.013G113000.3	1.894526406	2.087008225	0.239792591	8.703389	3.22E-18	9.11E-18	brown	-0.047680036	0.553186213	GO:0043565,GO:0006355,GO:0003700	AT3G58120.1	ATBZIP61,BZIP61	Basic-leucine zipper (bZIP) transcription factor family protein	BZIP transcription factor
Sapur.017G049700.1	11.75978221	-1.144463928	0.132456493	-8.6403	5.61E-18	1.58E-17	brown	-0.49623775	3.87E-11	GO:0003676	AT4G13850.1	ATGRP2,GR-RBP2,GRP2	glycine-rich RNA-binding protein 2	eukaryotic RNA-binding protein RBM24 RNA recognition motif protein
Sapur.011G039000.3	3.080776283	-1.508182491	0.175869618	-8.57557	9.86E-18	2.75E-17	brown	-0.116616999	0.14580838	GO:0005515	AT1G28340.1	AtRLP4,RLP4	receptor like protein 4	receptor-like kinase
Sapur.003G032800.1	18.82984805	-0.864816635	0.102015698	-8.47729	2.31E-17	6.37E-17	brown	-0.220356044	0.00555146		AT1G48630.1	RACK1B_AT	receptor for activated C kinase 1B	NA
Sapur.006G161700.1	155.0898418	-0.82779485	0.097697377	-8.47305	2.39E-17	6.60E-17	brown	-0.478246706	2.38E-10	GO:0006412	AT2G37270.1	ATRPS5B,RPS5B	ribosomal protein 5B	40S ribosomal protein S5, putative
Sapur.016G302700.1	18.00947722	-0.681175233	0.080416491	-8.47059	2.44E-17	6.74E-17	brown	-0.529334886	1.02E-12	GO:0007017,GO:0005874	AT2G29550.1	TUB7	tubulin beta-7 chain	NA
Sapur.010G096500.1	2.427656534	-2.27426488	0.269265353	-8.44618	3.01E-17	8.28E-17	brown	-0.419212871	4.64E-08	GO:0005975,GO:0004553	AT1G68560.1	ATXYL1,TRG1,XYL1	alpha-xylosidase 1	alpha-xylosidase
Sapur.004G173300.2	1.111564778	2.684472285	0.319173791	8.410691	4.08E-17	1.12E-16	brown	0.056318038	0.483564995	GO:0003993	AT2G38600.1		HAD superfamily, subfamily IIIB acid phosphatase	acid phosphatase
Sapur.010G047700.1	14.06860295	1.383991888	0.165983826	8.338113	7.55E-17	2.05E-16	brown	0.064097679	0.425135042		AT1G04560.1		AWPM-19-like family protein	plasma membrane associated protein
Sapur.016G283600.1	5.300866094	-0.943971763	0.11395928	-8.28341	1.20E-16	3.23E-16	brown	-0.182700185	0.022007129	GO:0043531	AT4G27220.1		NB-ARC domain-containing disease resistance protein	NA
Sapur.018G088700.1	3.859453283	-1.351597635	0.164236788	-8.22957	1.88E-16	5.04E-16	brown	-0.50529775	1.49E-11					NA
Sapur.001G067300.3	16.32546871	-0.835519278	0.101827016	-8.20528	2.30E-16	6.16E-16	brown	-0.051224376	0.524040785		AT4G24140.1		alpha/beta-Hydrolases superfamily protein	alpha/beta fold hydrolase
Sapur.019G012700.1	5.683481171	-1.37790409	0.168336687	-8.18541	2.71E-16	7.25E-16	brown	-0.423738939	3.21E-08	GO:0007165,GO:0005515,GO:0043531	AT5G36930.2		Disease resistance protein (TIR-NBS-LRR class) family	NA
Sapur.002G035700.3	3.996021359	-1.188748694	0.14590516	-8.14741	3.72E-16	9.89E-16	brown	-0.003909792	0.961239462	GO:0008168,GO:0008152	AT3G63410.1	APG1,E37,IEP37,VTE3	S-adenosyl-L-methionine-dependent methyltransferases superfamily protein	2-methyl-6-phytyl-1%2C4-benzoquinone methyltransferase
Sapur.016G299300.1	54.65435742	0.744408422	0.093266619	7.98151	1.45E-15	3.78E-15	brown	0.100063218	0.212433658		AT4G05320.2	UBQ10	polyubiquitin 10	NA
Sapur.004G063500.3	22.98099462	-1.126469366	0.141542408	-7.95853	1.74E-15	4.55E-15	brown	-0.102826508	0.200012351		AT1G66400.1	CML23	calmodulin like 23	NA
Sapur.001G058900.1	5.525906336	-1.536547202	0.193400716	-7.94489	1.94E-15	5.07E-15	brown	-0.462102778	1.11E-09	GO:0016021,GO:0005215	AT1G30840.2	ATPUP4,PUP4	purine permease 4	triose-phosphate transporter family protein
Sapur.006G157500.3	5.444713041	1.213610814	0.152921454	7.936171	2.09E-15	5.43E-15	brown	0.071253858	0.375185723		AT2G42040.1			WRC protein
Sapur.005G008300.1	33.9731799	-0.668666999	0.084368067	-7.92559	2.27E-15	5.91E-15	brown	0.078548531	0.32814497		AT1G09280.1			phospholipase/carboxylesterase family protein
Sapur.010G044000.1	1.495115859	-1.96847348	0.249380715	-7.89345	2.94E-15	7.63E-15	brown	-0.456311525	1.90E-09		AT2G33410.1		RNA-binding (RRM/RBD/RNP motifs) family protein	NA
Sapur.002G109800.1	85.76663539	-0.471118231	0.060318585	-7.8105	5.70E-15	1.46E-14	brown	-0.480038332	1.99E-10		AT2G44600.1			hypothetical protein
Sapur.013G057300.1	70.931763	-1.041461131	0.13338971	-7.80766	5.83E-15	1.50E-14	brown	-0.383270005	7.25E-07					NA
Sapur.010G185500.1	2.988852776	-1.526362037	0.195613476	-7.80295	6.05E-15	1.55E-14	brown	-0.45239654	2.71E-09		AT5G02420.1			cyclin-dependent kinase inhibitor siamese protein, putative
Sapur.15WG062400.1	6.797733897	-1.26690026	0.163922106	-7.72867	1.09E-14	2.77E-14	brown	-0.390518775	4.27E-07		AT5G17900.1		microfibrillar-associated protein-related	NA
Sapur.012G106300.3	61.81120201	0.474012283	0.061686184	7.684254	1.54E-14	3.91E-14	brown	-0.121578455	0.129308907	GO:0007264,GO:0005525	AT5G52210.1	ATARLB1,ATGB1,GB1	GTP-binding protein 1	ADP-ribosylation factor(Arf)/Arf-like (Arl) small GTPase family protein
Sapur.013G002600.1	3.201233568	-1.351954178	0.176307839	-7.66815	1.75E-14	4.42E-14	brown	-0.439564995	8.43E-09	GO:0010468,GO:0005777	AT5G61190.1		putative endonuclease or glycosyl hydrolase with C2H2-type zinc finger domain	NA
Sapur.016G200500.5	9.558478793	-0.859949853	0.112442445	-7.64791	2.04E-14	5.16E-14	brown	-0.081608864	0.309590942		AT3G26990.1		ENTH/VHS family protein	regulation of nuclear pre-mRNA domain protein 1B
Sapur.010G047600.1	2.291384196	1.81052146	0.237330333	7.628698	2.37E-14	5.98E-14	brown	0.078580115	0.327949911	GO:0003676	AT2G21660.1	ATGRP7.CCR2.GR-RBP7.GRI	cold, circadian rhythm, and rna binding 2	NA
Sapur.007G052800.2	60.01142327	0.543907577	0.071589061	7.597635	3.02E-14	7.58E-14	brown	-0.103621445	0.196538298	GO:0006468,GO:0004672	AT5G66210.2	CPK28	calcium-dependent protein kinase 28	calcium-dependent kinase
Sapur.006G036200.1	169.2325066	-1.754076886	0.231113254	-7.58969	3.21E-14	8.06E-14	brown	-0.355690062	4.84E-06	GO:0005975,GO:0004553	AT3G57240.1	BG3	beta-1,3-glucanase 3	beta-1%2C3-glucanase
Sapur.003G049900.1	19.82259362	0.788020224	0.104221169	7.561038	4.00E-14	1.00E-13	brown	0.042928683	0.59345038		AT4G16845.1	VRN2	VEFS-Box of polycomb protein	polycomb protein embryonic flower, putative
Sapur.002G176200.2	8.227442685	-1.018819673	0.135130549	-7.53952	4.72E-14	1.18E-13	brown	-0.119128608	0.137266905					NA
Sapur.007G030700.2	1.006606141	-2.151956027	0.289875204	-7.42373	1.14E-13	2.81E-13	brown	-0.291371156	0.000213605		AT5G66600.4		Protein of unknown function, DUF547	electron transporter, putative
Sapur.006G119300.1	2.621767529	-1.502859962	0.203128072	-7.39858	1.38E-13	3.39E-13	brown	-0.488427105	8.62E-11	GO:0008270,GO:0005515	AT5G17600.1		RING/U-box superfamily protein	RING-H2 zinc finger protein
Sapur.001G046200.3	36.09123886	-1.146976049	0.155278205	-7.38659	1.51E-13	3.70E-13	brown	-0.286599864	0.000273666	GO:0016020,GO:0009765	AT3G61470.1	LHCA2	photosystem I light harvesting complex gene 2	photosystem I light harvesting complex Lhca2/4, chlorophyll A/B-binding protein
Sapur.001G166200.3	2.172651428	-1.592850082	0.215841252	-7.37973	1.59E-13	3.90E-13	brown	-0.038830171	0.629213027	GO:0006412,GO:0005840,GO:0005622,GO:0003735	AT1G79850.1	CS17,PRPS17,RPS17	ribosomal protein S17	30S ribosomal protein S17
Sapur.007G003900.3	27.33011935	0.616041805	0.083647987	7.364694	1.78E-13	4.35E-13	brown	-0.034203333	0.670642572		AT5G67620.1			DUF4228 domain protein
Sapur.001G138900.1	1.01650146	-2.031164698	0.281359739	-7.2191	5.23E-13	1.26E-12	brown	-0.503938607	1.72E-11		AT4G12510.1		Bifunctional inhibitor/lipid-transfer protein/seed storage 2S albumin superfamily protein	protease inhibitor/seed storage/LTP family protein
Sapur.007G068000.1	1.53422956	-1.609752518	0.223569311	-7.20024	6.01E-13	1.45E-12	brown	-0.463792196	9.51E-10	GO:0006412,GO:0005840,GO:0003735	AT3G22300.1	RPS10	ribosomal protein S10	NA
Sapur.001G177300.5	8.711156788	-0.831805615	0.115851364	-7.17994	6.97E-13	1.68E-12	brown	-0.081608864	0.309590942					hypothetical protein
Sapur.005G130000.1	25.20574992	-0.653688604	0.091443285	-7.14857	8.77E-13	2.10E-12	brown	-0.426186945	2.62E-08	GO:0005515	AT1G78100.1		F-box family protein	F-box plant protein
Sapur.002G005600.4	5.321781699	-0.947499377	0.133309938	-7.10749	1.18E-12	2.82E-12	brown	-0.157683657	0.048570943	GO:0003677	AT4G35550.1	ATWOX13,HB-4,WOX13	WUSCHEL related homeobox 13	HB1 homeodomain protein
Sapur.005G163000.5	9.771310414	-0.789538168	0.112166962	-7.03895	1.94E-12	4.59E-12	brown	-0.081608864	0.309590942		AT3G10260.3		Reticulon family protein	reticulon-like protein
Sapur.010G047900.1	5.743403438	-1.566075477	0.223514798	-7.00659	2.44E-12	5.76E-12	brown	-0.397745247	2.49E-07		AT4G13480.1	AtMYB79,MYB79	myb domain protein 79	MYB transcription factor
Sapur.006G061500.1	8.046327738	-0.950705121	0.135725846	-7.0046	2.48E-12	5.84E-12	brown	-0.452278204	2.74E-09	GO:0016020,GO:0005618,GO:0004252,GO:0006508	AT2G19170.1	SLP3	subtilisin-like serine protease 3	subtilisin-like serine protease
Sapur.017G120200.2	3.139027872	-1.27296759	0.181998768	-6.99437	2.66E-12	6.28E-12	brown	-0.45995434	1.36E-09		AT5G52760.1		Copper transport protein family	NA
Sapur.004G084300.1	3.830650357	-1.109014687	0.161390146	-6.87164	6.35E-12	1.48E-11	brown	-0.425451447	2.78E-08					NA
Sapur.010G007400.1	105.5994321	-0.596478265	0.086938392	-6.86093	6.84E-12	1.59E-11	brown	-0.459083941	1.47E-09					NA
Sapur.15WG018600.1	231.9491739	0.960460235	0.140649971	6.828727	8.57E-12	1.98E-11	brown	-0.041432704	0.606397174	GO:0005975,GO:0004553	AT5G24090.1	ATCHIA,CHIA	chitinase A	acidic chitinase
Sapur.017G002300.1	0.98176215	2.547783436	0.37442984	6.804435	1.01E-11	2.34E-11	brown	0.259125942	0.001048973		AT3G59850.1		Pectin lyase-like superfamily protein	endo-polygalacturonase
Sapur.010G118900.1	1.135705275	-2.063015361	0.304419128	-6.77689	1.23E-11	2.82E-11	brown	-0.386717544	5.65E-07		AT3G17340.1		ARM repeat superfamily protein	protein transporter, putative
Sapur.011G055800.5	1.609296964	-1.552565341	0.230340414	-6.74031	1.58E-11	3.62E-11	brown	-0.081608864	0.309590942		AT4G18390.2	TCP2	TEOSINTE BRANCHED 1, cycloidea and PCF transcription factor 2	TCP family transcription factor
Sapur.004G123800.4	22.21816775	0.771180411	0.114416461	6.740118	1.58E-11	3.62E-11	brown	-0.081608864	0.309590942	GO:0006355	AT5G45600.1	GAS41,TAF14B	YEATS family protein	NA
Sapur.006G151800.3	4.117083366	-1.048768112	0.157040821	-6.67832	2.42E-11	5.50E-11	brown	-0.107354602	0.18080809		AT5G58000.1		Reticulon family protein	reticulon protein
Sapur.005G138200.18	36.66772569	-0.394509324	0.059684466	-6.60992	3.85E-11	8.68E-11	brown	-0.020043286	0.803237189	GO:0006355,GO:0006289,GO:0000439	AT1G18340.1		basal transcription factor complex subunit-related	transcription factor tfb4
Sapur.010G045800.2	1.195581404	-1.756747096	0.267352803	-6.57089	5.00E-11	1.12E-10	brown	-0.131478704	0.100716042		AT1G04520.1	PDLP2	plasmodesmata-located protein 2	cysteine-rich repeat secretory protein
Sapur.004G162500.2	95.97375982	0.740093512	0.112708108	6.566462	5.15E-11	1.16E-10	brown	0.375062825	1.30E-06	GO:0006508,GO:0004185	AT3G45010.1	scpl48	serine carboxypeptidase-like 48	serine carboxypeptidase
Sapur.013G070700.3	2.280113945	-1.187817764	0.181984326	-6.52703	6.71E-11	1.50E-10	brown	-0.362708956	3.04E-06	GO:0006432,GO:0005524,GO:0003723,GO:0000287,GO:0004826	AT1G72550.1		tRNA synthetase beta subunit family protein	phenylalanyl-tRNA synthetase beta chain, putative
Sapur.002G158100.2	8.228856114	0.7182498	0.110059711	6.526001	6.75E-11	1.51E-10	brown	-0.003355642	0.966729723	GO:0006904,GO:0000145	AT4G02350.1	SEC15B	exocyst complex component sec15B	exocyst complex subunit Sec15-like protein
Sapur.003G073100.1	34.36990421	-0.424519863	0.065293454	-6.50172	7.94E-11	1.77E-10	brown	-0.487078636	9.88E-11		AT4G25170.1		Uncharacterised conserved protein (UCP012943)	hypothetical protein
Sapur.005G098300.2	2.254176081	-1.216357657	0.188642125	-6.44796	1.13E-10	2.51E-10	brown	-0.199960194	0.012042927	GO:0016491,GO:0008152	AT2G29150.1		NAD(P)-binding Rossmann-fold superfamily protein	tropinone reductase II
Sapur.012G033900.1	33.62707533	-0.646033863	0.100456544	-6.43098	1.27E-10	2.81E-10	brown	-0.392214848	3.77E-07	GO:0005515	AT1G18080.1	ATARCA.RACK1A.RACK1A_A	Transducin/WD40 repeat-like superfamily protein	guanine nucleotide-binding protein subunit beta-like protein
Sapur.005G007800.3	3.528660017	1.05649297	0.165708697	6.375604	1.82E-10	4.01E-10	brown	0.002391049	0.976290055	GO:0033926	AT1G56560.1		Plant neutral invertase family protein	neutral/alkaline invertase
Sapur.010G111900.8	7.452879818	0.780735177	0.123329012	6.330507	2.44E-10	5.35E-10	brown	-0.01862744	0.816882801	GO:0005515	AT5G49430.1		WD40/YVTN repeat-like-containing domain;Bromodomain	cytochrome cd1-nitrite reductase-like, carboxy-terminal heme protein d1, putative
Sapur.15ZG046200.1	2.304488435	-1.085180865	0.171660391	-6.32167	2.59E-10	5.66E-10	brown	-0.433293271	1.44E-08	GO:0006468,GO:0004672	AT3G02130.1	RPK2,TOAD2	receptor-like protein kinase 2	NA
Sapur.003G095800.1	4.570636832	-1.654922163	0.262302083	-6.30922	2.80E-10	6.12E-10	brown	-0.350164733	6.94E-06		AT1G63910.1	AtMYB103,MYB103	myb domain protein 103	NA
Sapur.019G018500.1	62.77347595	0.309681734	0.049660054	6.236033	4.49E-10	9.72E-10	brown	-0.081608864	0.309590942	GO:0005515,GO:0006468,GO:0004672	AT3G14840.2		Leucine-rich repeat transmembrane protein kinase	NA
Sapur.T079500.1	10.24261456	0.995763612	0.160763856	6.193952	5.87E-10	1.26E-09	brown	0.356795153	4.50E-06	GO:0005509				NA
Sapur.T158800.3	0.360699327	-2.020025444	0.326927775	-6.17881	6.46E-10	1.39E-09	brown	-0.114902006	0.151868242		AT4G17790.1		SNARE associated Golgi protein family	NA
Sapur.014G020100.1	9.954113948	0.995946484	0.162911726	6.113412	9.75E-10	2.08E-09	brown	0.356512001	4.59E-06	GO:0005509				EF hand protein
Sapur.014G144300.2	2.649016015	-1.062466518	0.173880679	-6.11032	9.94E-10	2.12E-09	brown	-0.110935599	0.166608292	GO:0003723,GO:0032259,GO:0008168	AT3G25470.1		bacterial hemolysin-related	TlyA family rRNA methyltransferase/hemolysin protein, putative
Sapur.006G032500.2	1.312293765	-1.580588434	0.259656002	-6.08724	1.15E-09	2.45E-09	brown	0.078548531	0.32814497	GO:0006396,GO:0003723	AT3G57150.1	AtCBF5.AtNAP57.CBF5.NAP	homologue of NAP57	tRNA pseudouridine synthase
Sapur.016G092800.4	6.992798707	0.658879189	0.108391287	6.07871	1.21E-09	2.58E-09	brown	-0.163321145	0.040973418	GO:0030131,GO:0016192,GO:0006886,GO:0030117	AT1G60070.1		Adaptor protein complex AP-1, gamma subunit	AP-1 complex subunit gamma-2
Sapur.014G127700.4	4.758798858	-0.86607028	0.143038183	-6.05482	1.41E-09	2.98E-09	brown	-0.053754685	0.503721591		AT1G05060.1			hypothetical protein
Sapur.006G211500.2	68.07903587	-0.458726561	0.076219583	-6.01849	1.76E-09	3.72E-09	brown	-0.380481452	8.86E-07	GO:0045263,GO:0015986,GO:0015078	AT4G32260.1		ATPase, F0 complex, subunit B/B\', bacterial/chloroplast	ATP synthase subunit B' protein
Sapur.012G081400.1	84.96495508	-0.470287708	0.078155194	-6.01736	1.77E-09	3.75E-09	brown	-0.035956871	0.654815399		AT4G25080.2	CHLM	magnesium-protoporphyrin IX methyltransferase	Mg-protoporphyrin IX methyltransferase
Sapur.002G038100.2	5.057327942	-0.921214914	0.153213191	-6.01263	1.83E-09	3.85E-09	brown	0.046803126	0.560516638		AT3G04810.2	ATNEK2,NEK2	NIMA-related kinase 2	Serine/Threonine kinase domain protein
Sapur.019G003800.1	1.467926923	-1.274340868	0.217450384	-5.86038	4.62E-09	9.60E-09	brown	-0.440333878	7.89E-09	GO:0046872,GO:0000166,GO:0016887,GO:0016021,GO:0006812	AT5G23630.1	MIA,PDR2	phosphate deficiency response 2	cation-transporting ATPase, putative
Sapur.010G118000.3	3.172733672	0.87908561	0.150660169	5.834891	5.38E-09	1.12E-08	brown	-0.049116522	0.541279578	GO:0003824,GO:0006470,GO:0004722	AT1G48040.1		Protein phosphatase 2C family protein	protein phosphatase 2C
Sapur.017G051800.1	39.65507551	-0.406144766	0.070041235	-5.79865	6.68E-09	1.38E-08	brown	-0.37931131	9.63E-07	GO:0005515	AT3G26810.1	AFB2	auxin signaling F-box 2	transport inhibitor response protein
Sapur.004G010800.3	5.041605679	-0.680448955	0.117451551	-5.79344	6.90E-09	1.42E-08	brown	-0.0065031	0.93557451		AT4G21790.1	ATTOM1,TOM1	tobamovirus multiplication 1	tobamovirus multiplication protein
Sapur.T168600.1	3.559086623	-0.820858214	0.14184112	-5.78717	7.16E-09	1.48E-08	brown	-0.393113096	3.53E-07		AT3G07930.3		DNA glycosylase superfamily protein	NA
Sapur.009G013000.1	106.1582257	-0.34598153	0.060053478	-5.76122	8.35E-09	1.72E-08	brown	-0.409431306	1.01E-07		AT2G27260.1		Late embryogenesis abundant (LEA) hydroxyproline-rich glycoprotein family	late embryogenesis abundant protein
Sapur.006G211100.8	30.19463445	-0.422779938	0.07344522	-5.7564	8.59E-09	1.77E-08	brown	-0.095731004	0.23300179		AT4G35530.1		phosphatidylinositolglycan-related	GPI-GlcNAc transferase complex, PIG-H component
Sapur.011G119800.1	1.102160265	-1.744208647	0.303358518	-5.74966	8.94E-09	1.84E-08	brown	-0.381529697	8.22E-07	GO:0055114,GO:0050660,GO:0016491	AT5G44400.1		FAD-binding Berberine family protein	reticuline oxidase
Sapur.004G050500.4	7.946468929	-0.604276756	0.10512554	-5.74814	9.02E-09	1.85E-08	brown	-0.081608864	0.309590942		AT5G62650.1		Tic22-like family protein	plant/MRG21-7 protein, putative
Sapur.003G159500.2	2.149169985	-1.304065312	0.227718032	-5.72667	1.02E-08	2.10E-08	brown	-0.387432336	5.36E-07		AT5G12900.1			NA
Sapur.006G207100.2	15.50619715	1.296303172	0.227203507	5.705472	1.16E-08	2.37E-08	brown	0.289312936	0.000237828		AT5G11420.1		Protein of unknown function, DUF642	plant/F18G18-200 protein
Sapur.018G045000.3	24.49550829	0.427488375	0.075003251	5.699598	1.20E-08	2.45E-08	brown	-0.066031628	0.411270694					hypothetical protein
Sapur.002G044900.1	36.54457682	0.507957504	0.089157165	5.697327	1.22E-08	2.48E-08	brown	0.351500566	6.36E-06					zeta toxin protein
Sapur.016G168700.1	1364.573745	0.512841518	0.090194341	5.685961	1.30E-08	2.65E-08	brown	0.420258727	4.26E-08	GO:0016884	AT5G64440.1	AtFAAH,FAAH	fatty acid amide hydrolase	glutamyl-tRNA(Gln) amidotransferase subunit A
Sapur.014G118600.1	11.99875512	-0.56630101	0.099630736	-5.684	1.32E-08	2.68E-08	brown	-0.373026269	1.50E-06		AT4G14200.1		Pentatricopeptide repeat (PPR) superfamily protein	salt-inducible protein, putative
Sapur.018G092600.1	0.876952818	-1.511221395	0.266297774	-5.67493	1.39E-08	2.82E-08	brown	-0.397063059	2.62E-07	GO:0031515,GO:0030488,GO:0016429	AT5G14600.1		S-adenosyl-L-methionine-dependent methyltransferases superfamily protein	NA
Sapur.002G054700.1	167.7581802	-0.221517952	0.039247572	-5.64412	1.66E-08	3.37E-08	brown	-0.409560026	1.00E-07	GO:0005515	AT1G76920.1		F-box family protein	SKP1-interacting partner 15
Sapur.002G074600.1	1.546757151	-1.455552252	0.258850145	-5.62315	1.88E-08	3.80E-08	brown	-0.187943157	0.018415925	GO:0006633,GO:0005524,GO:0003989	AT1G36160.1	ACC1.AT-ACC1.EMB22.GK.P	acetyl-CoA carboxylase 1	acetyl-CoA carboxylase
Sapur.003G018000.3	1.392233209	1.336129364	0.238291652	5.607118	2.06E-08	4.16E-08	brown	-0.081608864	0.309590942		AT1G52080.1	AR791	actin binding protein family	CHUP1, putative
Sapur.006G153700.1	147.8720892	-0.825916649	0.148109744	-5.57638	2.46E-08	4.95E-08	brown	-0.409870399	9.78E-08	GO:0005975,GO:0004553	AT4G19810.1		Glycosyl hydrolase family protein with chitinase insertion domain	glycoside hydrolase family 18 protein
Sapur.T167600.4	2.855364922	-1.209827856	0.217135037	-5.57178	2.52E-08	5.08E-08	brown	-0.250407815	0.001561104		AT3G12340.1		FKBP-like peptidyl-prolyl cis-trans isomerase family protein	minichromosone maintenance protein
Sapur.T054700.1	2.886936284	-0.924579957	0.167034725	-5.53526	3.11E-08	6.24E-08	brown	-0.387833976	5.21E-07					NA
Sapur.009G012700.6	1.900014709	-1.119636526	0.204383283	-5.47812	4.30E-08	8.58E-08	brown	-0.081608864	0.309590942	GO:0006468,GO:0005524,GO:0004672	AT3G44200.1	ATNEK6,IBO1,NEK6	NIMA (never in mitosis, gene A)-related 6	Serine/Threonine-kinase, putative
Sapur.003G150200.1	4.179670795	-0.821614656	0.150648591	-5.45385	4.93E-08	9.81E-08	brown	-0.32122122	4.10E-05		AT5G09860.1	AtHPR1.AtTHO1.HPR1.THO:	nuclear matrix protein-related	THO complex subunit 1 transcription elongation factor
Sapur.001G043300.3	19.89186394	-0.411587668	0.075692871	-5.4376	5.40E-08	1.07E-07	brown	-0.102505665	0.201427015	GO:0016020	AT4G13590.1		Uncharacterized protein family (UPF0016)	transmembrane protein TPARL protein, putative
Sapur.014G085600.4	31.54671986	-0.391899071	0.072331173	-5.41812	6.02E-08	1.19E-07	brown	-0.081608864	0.309590942		AT1G02270.1		Calcium-binding endonuclease/exonuclease/phosphatase family	carbon catabolite repressor protein, putative
Sapur.013G028800.2	16.99801393	-0.417005765	0.077084836	-5.4097	6.31E-08	1.25E-07	brown	-0.394269406	3.24E-07		AT5G27320.1	ATGID1C,GID1C	alpha/beta-Hydrolases superfamily protein	gibberellin receptor GID1, putative
Sapur.012G097400.1	32.7554697	1.229844936	0.22767602	5.401732	6.60E-08	1.31E-07	brown	0.25365021	0.001348661	GO:0004857	AT4G25250.1		Plant invertase/pectin methylesterase inhibitor superfamily protein	pectinesterase/pectinesterase inhibitor, putative
Sapur.008G126300.2	0.72598102	-1.795175001	0.332528799	-5.39855	6.72E-08	1.33E-07	brown	-0.375111993	1.29E-06		AT4G14770.1	ATTCX2,TCX2	TESMIN/TSO1-like CXC 2	TSO1, putative
Sapur.007G030000.2	0.962583215	1.647281696	0.305561237	5.391003	7.01E-08	1.39E-07	brown	-0.036141691	0.653156004	GO:0008270	AT4G36860.1	DAR1	LIM domain-containing protein	zinc ion-binding protein, putative
Sapur.003G107200.3	0.469150756	1.850187057	0.343614951	5.384478	7.27E-08	1.44E-07	brown	-0.016661323	0.835922479	GO:0016021,GO:0005789	AT5G42000.1		ORMDL family protein	ORMDL family protein
Sapur.001G164800.2	1.045212623	-1.466538834	0.273643139	-5.35931	8.35E-08	1.65E-07	brown	-0.129501545	0.105986807		AT4G29100.1		basic helix-loop-helix (bHLH) DNA-binding superfamily protein	transcription factor, putative
Sapur.011G096100.2	14.28954323	-0.815084295	0.152812102	-5.3339	9.61E-08	1.89E-07	brown	-0.076723289	0.339544052	GO:0016491	AT4G27270.1		Quinone reductase family protein	quinone oxidoreductase, putative
Sapur.018G019500.1	6.714961526	-0.814531249	0.154664642	-5.26643	1.39E-07	2.72E-07	brown	-0.330212322	2.40E-05		AT5G25265.1			transmembrane protein, putative
Sapur.004G009000.1	2.742667398	-1.072995209	0.204212452	-5.25431	1.49E-07	2.90E-07	brown	-0.281217057	0.000360049	GO:0006468,GO:0004672	AT4G04720.1	CPK21	calcium-dependent protein kinase 21	calcium-dependent kinase
Sapur.006G123200.1	1.46927542	-1.285479158	0.244694752	-5.2534	1.49E-07	2.91E-07	brown	-0.350515913	6.78E-06	GO:0003723	AT4G29750.1		CRS1 / YhbY (CRM) domain-containing protein	CRS1/YhbY domain protein
Sapur.018G065700.1	2.603798171	-1.050023751	0.199977843	-5.2507	1.52E-07	2.96E-07	brown	-0.380249051	9.00E-07					NA
Sapur.016G162500.2	5.341181666	-0.740507385	0.141905149	-5.21833	1.81E-07	3.51E-07	brown	-0.348172561	7.88E-06		AT2G30000.1		PHF5-like protein	PHD finger-like domain protein 5A
Sapur.013G070700.1	1.754841505	-1.057508096	0.202980659	-5.2099	1.89E-07	3.67E-07	brown	-0.053853062	0.502939991	GO:0006432,GO:0005524,GO:0003723,GO:0000287,GO:0004826	AT1G72550.1		tRNA synthetase beta subunit family protein	phenylalanyl-tRNA synthetase beta chain, putative
Sapur.018G061300.2	5.690733545	-0.605334047	0.116864855	-5.17978	2.22E-07	4.30E-07	brown	-0.381664565	8.14E-07		AT5G19570.1			transmembrane protein, putative
Sapur.019G078200.1	1.385704334	1.91621854	0.370263222	5.175287	2.28E-07	4.41E-07	brown	0.010291096	0.898212268		AT4G03500.1		Ankyrin repeat family protein	ankyrin repeat protein
Sapur.016G246200.5	0.57717906	-1.73499567	0.336154188	-5.16131	2.45E-07	4.74E-07	brown	-0.089328523	0.265895437	GO:0005992,GO:0003824	AT1G78580.1	ATTPS1,TPS1	trehalose-6-phosphate synthase	trehalose-6-phosphate synthase
Sapur.016G014800.1	3.456076036	-0.831106669	0.161436559	-5.14819	2.63E-07	5.08E-07	brown	-0.347582868	8.19E-06		AT3G22160.1		VQ motif-containing protein	VQ motif protein
Sapur.006G075900.4	31.64330779	0.515982608	0.100311506	5.143803	2.69E-07	5.19E-07	brown	-0.073064326	0.363142303		AT5G02020.1			plant/T7H20-70 protein, putative
Sapur.T171600.1	2.015379227	-0.921958458	0.180067409	-5.12007	3.05E-07	5.87E-07	brown	-0.394932538	3.08E-07		AT1G06530.1		Tropomyosin-related	NA
Sapur.006G148100.1	14.93782319	0.547894395	0.107338246	5.104373	3.32E-07	6.37E-07	brown	0.320820746	4.19E-05					transmembrane protein, putative
Sapur.011G050900.5	2.823768359	-1.613708908	0.317629764	-5.08047	3.76E-07	7.20E-07	brown	-0.103853871	0.19553086		AT1G29750.2	RKF1	receptor-like kinase in flowers 1	receptor-like kinase, putative
Sapur.006G202000.1	0.896419069	-1.911427342	0.377089951	-5.06889	4.00E-07	7.65E-07	brown	-0.210892547	0.008019529		AT5G25590.1		Protein of unknown function (DUF630 and DUF632)	NA
Sapur.016G256300.1	6.611677712	-0.788425336	0.15570785	-5.06349	4.12E-07	7.86E-07	brown	-0.277067712	0.000443182	GO:0003824,GO:0006470,GO:0004722	AT3G15260.1		Protein phosphatase 2C family protein	protein phosphatase 2C
Sapur.003G136700.1	2.352559822	-0.921717271	0.182614628	-5.04734	4.48E-07	8.54E-07	brown	-0.364190986	2.75E-06	GO:0006810,GO:0005643	AT1G80680.1	MOS3,NUP96,PRE,SAR3	SUPPRESSOR OF AUXIN RESISTANCE 3	nucleoporin, putative
Sapur.002G060700.4	5.29258026	-0.802719653	0.160160412	-5.01197	5.39E-07	1.02E-06	brown	-0.070805882	0.378203	GO:0005515	AT1G77250.1		RING/FYVE/PHD-type zinc finger family protein	PHD-finger protein
Sapur.016G136700.1	825.6099064	-0.206671523	0.041371789	-4.99547	5.87E-07	1.11E-06	brown	-0.360182059	3.60E-06	GO:0003824,GO:0006470,GO:0004722	AT2G29380.1	HAI3	highly ABA-induced PP2C gene 3	protein phosphatase 2C
Sapur.003G045200.3	0.81030093	-1.449116737	0.290924446	-4.98108	6.32E-07	1.20E-06	brown	-0.037830542	0.638072215	GO:0008168,GO:0006479	AT4G16570.1	ATPRMT7,PRMT7	protein arginine methyltransferase 7	arginine N-methyltransferase
Sapur.010G085900.3	2.995609009	-0.960377099	0.192849303	-4.97994	6.36E-07	1.20E-06	brown	-0.081608864	0.309590942		AT1G71000.1		Chaperone DnaJ-domain superfamily protein	heat shock protein-binding protein, putative
Sapur.016G167400.1	2.813423552	0.854833297	0.171766925	4.976705	6.47E-07	1.22E-06	brown	0.371630586	1.65E-06		AT2G02560.2	ATCAND1.CAND1.ETA2.HVE	cullin-associated and neddylation dissociated	NA
Sapur.010G163200.3	3.302268406	-0.845663176	0.170949232	-4.94687	7.54E-07	1.42E-06	brown	-0.322818674	3.73E-05	GO:0006418,GO:0005524,GO:0004812,GO:0000166	AT3G55400.1	OVA1	methionyl-tRNA synthetase / methionine--tRNA ligase / MetRS (cpMetRS)	methionyl-tRNA synthetase
Sapur.008G015700.1	5.258676282	-0.933159875	0.188795343	-4.94271	7.70E-07	1.45E-06	brown	-0.285584952	0.000288315		AT5G04700.1		Ankyrin repeat family protein	ankyrin repeat protein
Sapur.019G042400.1	0.78288651	-1.464549694	0.297399446	-4.92452	8.46E-07	1.59E-06	brown	-0.362080665	3.17E-06	GO:0007165,GO:0005515,GO:0043531	AT5G36930.2		Disease resistance protein (TIR-NBS-LRR class) family	NA
Sapur.15WG013500.1	4.329812597	1.266127642	0.258087243	4.905813	9.30E-07	1.75E-06	brown	0.333723451	1.94E-05	GO:0006468,GO:0005524,GO:0004672	AT1G66920.2		Protein kinase superfamily protein	NA
Sapur.008G063100.1	0.97493116	1.921410971	0.392887645	4.890485	1.01E-06	1.89E-06	brown	0.31349938	6.39E-05	GO:0005975,GO:0004553	AT1G65610.1	ATGH9A2,KOR2	Six-hairpin glycosidases superfamily protein	endo-1%2C4-beta-glucanase
Sapur.010G090700.1	1.994569299	-1.172286513	0.239845399	-4.88768	1.02E-06	1.91E-06	brown	-0.225961823	0.004433241		AT1G15080.1	ATLPP2,ATPAP2,LPP2	lipid phosphate phosphatase 2	lipid phosphate phosphatase
Sapur.T048000.2	0.743905908	-1.373197013	0.282877851	-4.85438	1.21E-06	2.25E-06	brown	-0.388457068	4.97E-07	GO:0055114,GO:0016491,GO:0006633,GO:0005506	AT1G02205.3	CER1	Fatty acid hydroxylase superfamily	sterol desaturase, putative
Sapur.017G057100.3	0.900173876	-1.40695293	0.289876341	-4.85363	1.21E-06	2.26E-06	brown	-0.150950439	0.059144556	GO:0006355,GO:0005739,GO:0003690	AT4G14605.1		Mitochondrial transcription termination factor family protein	transcription termination factor-like protein
Sapur.006G017500.5	6.425182717	0.546319627	0.113014651	4.83406	1.34E-06	2.49E-06	brown	-0.184060018	0.021022105		AT3G22260.1		Cysteine proteinases superfamily protein	OTU-like cysteine protease
Sapur.15WG044700.1	0.51922244	-2.420214376	0.501390715	-4.827	1.39E-06	2.58E-06	brown	-0.334153146	1.89E-05	GO:0006950,GO:0005516	AT1G73805.1		Calmodulin binding protein-like	calmodulin-binding protein
Sapur.006G103900.5	3.987160936	0.628305695	0.130764689	4.804857	1.55E-06	2.87E-06	brown	-0.081608864	0.309590942		AT2G37025.1	TRFL8	TRF-like 8	Myb-like DNA-binding domain protein
Sapur.019G090500.3	13.62869845	-0.545483211	0.114064285	-4.78224	1.73E-06	3.21E-06	brown	-0.131333931	0.101094824		AT1G05710.3		basic helix-loop-helix (bHLH) DNA-binding superfamily protein	transcription factor BHLH protein
Sapur.002G183300.1	347.5056982	-0.204547274	0.042798326	-4.77933	1.76E-06	3.26E-06	brown	-0.369696732	1.89E-06		AT5G13890.3		Family of unknown function (DUF716)	transmembrane protein, putative
Sapur.013G013700.2	565.0849632	-0.427317539	0.08966277	-4.76583	1.88E-06	3.48E-06	brown	-0.283905028	0.000314171		AT1G56220.1		Dormancy/auxin associated family protein	dormancy/auxin associated protein
Sapur.006G091800.1	0.524309656	-1.773618777	0.37218757	-4.76539	1.88E-06	3.48E-06	brown	-0.377164465	1.12E-06	GO:0055085,GO:0016021	AT5G01990.1		Auxin efflux carrier family protein	auxin efflux carrier family transporter
Sapur.005G000500.2	4.209243206	-0.959052682	0.20213593	-4.74459	2.09E-06	3.85E-06	brown	-0.230903743	0.003619854	GO:0006418.GO:0005737.GO:0005524.GO:0004812.GO:0000166.GO:0043039.GO:001687	AT5G26710.1		Glutamyl/glutaminyl-tRNA synthetase, class Ic	glutamyl-tRNA synthetase
Sapur.012G035200.1	0.796983525	1.669820446	0.353703413	4.720962	2.35E-06	4.32E-06	brown	0.095606828	0.233611275	GO:0016021,GO:0016020,GO:0022857	AT5G07050.1		nodulin MtN21 /EamA-like transporter family protein	auxin-induced protein 5NG4
Sapur.001G108900.3	2.211160108	0.843224331	0.17873686	4.717686	2.39E-06	4.39E-06	brown	-0.150946015	0.05915208	GO:0032502,GO:0006351,GO:0005634	AT4G37740.1	AtGRF2,GRF2	growth-regulating factor 2	growth-regulating factor, putative
Sapur.003G151300.1	108.2106235	0.202913082	0.043133512	4.704302	2.55E-06	4.68E-06	brown	0.29451021	0.000181041	GO:0005515	AT1G08315.1		ARM repeat superfamily protein	ubiquitin-protein ligase, putative
Sapur.006G070000.4	6.04252626	-0.608608767	0.12981145	-4.68841	2.75E-06	5.05E-06	brown	-0.085896783	0.284771806	GO:0010338,GO:0006351,GO:0005634	AT2G37630.1	AS1.ATMYB91.ATPHAN.MYE	myb-like HTH transcriptional regulator family protein	MYB-related transcription factor, putative
Sapur.006G217000.1	12.0372242	-0.462004362	0.099010751	-4.6662	3.07E-06	5.62E-06	brown	-0.2922129	0.000204376	GO:0016020,GO:0008375	AT2G37585.1		Core-2/I-branching beta-1,6-N-acetylglucosaminyltransferase family protein	core-2/I-branching beta-1%2C6-N-acetylglucosaminyltransferase-like protein
Sapur.019G010800.1	0.676305462	-1.632942643	0.35070175	-4.65621	3.22E-06	5.89E-06	brown	-0.323352566	3.62E-05		AT5G14930.2	SAG101	senescence-associated gene 101	hypothetical protein
Sapur.001G135600.3	20.87906716	0.305032286	0.065548115	4.653563	3.26E-06	5.96E-06	brown	-0.057082019	0.477642493		AT1G17280.2	UBC34	ubiquitin-conjugating enzyme 34	ubiquitin-conjugating enzyme E2, putative
Sapur.001G136400.1	3.656728857	-0.927935487	0.199455088	-4.65235	3.28E-06	6.00E-06	brown	-0.244197493	0.002055221	GO:0046872,GO:0000166	AT1G17260.1	AHA10	autoinhibited H(+)-ATPase isoform 10	plasma membrane H+-ATPase
Sapur.010G096800.2	56.4113954	0.327735978	0.070639451	4.63956	3.49E-06	6.38E-06	brown	0.002227394	0.977912453	GO:0005515,GO:0043161,GO:0006289,GO:0003684	AT5G38470.1	RAD23D	Rad23 UV excision repair protein family	UV excision repair protein RAD23, putative
Sapur.004G084100.1	12.12926519	-0.488854733	0.105574541	-4.63042	3.65E-06	6.65E-06	brown	-0.264202205	0.000826975	GO:0005524,GO:0051082,GO:0006457	AT3G02530.1		TCP-1/cpn60 chaperonin family protein	NA
Sapur.003G056000.3	30.70118185	-0.32603715	0.070873825	-4.60025	4.22E-06	7.67E-06	brown	0.021762757	0.786745202	GO:0016021	AT1G32400.3	TOM2A	tobamovirus multiplication 2A	tetraspanin family protein
Sapur.008G161900.2	1.55107641	-1.038590071	0.225879925	-4.59797	4.27E-06	7.75E-06	brown	-0.320200015	4.35E-05		AT4G15820.1			hypothetical protein
Sapur.011G042600.2	10.17651377	0.635527624	0.138726847	4.581144	4.62E-06	8.39E-06	brown	0.078548531	0.32814497		AT4G18470.1	SNI1	negative regulator of systemic acquired resistance (SNI1)	SNI1
Sapur.006G114500.1	2.978029054	-0.88368729	0.193360844	-4.57015	4.87E-06	8.83E-06	brown	-0.308969721	8.24E-05	GO:0006355,GO:0003700	AT5G21960.1		Integrase-type DNA-binding superfamily protein	NA

Sapur.T033100.1	0.319757142	-1.789409224	0.397521968	-4.50141	6.75E-06	1.21E-05	brown	-0.20265116	0.010915634	GO:0006284	AT3G07930.3		DNA glycosylase superfamily protein	NA
Sapur.018G010500.1	0.886512302	-1.605869041	0.357439258	-4.4927	7.03E-06	1.26E-05	brown	-0.318382955	4.83E-05	GO:0006508,GO:0004190	AT5G10770.1		Eukaryotic aspartyl protease family protein	aspartic proteinase nepenthesin protein, putative
Sapur.002G042400.1	1.390989945	-1.071202794	0.23843342	-4.49267	7.03E-06	1.26E-05	brown	-0.322243654	3.86E-05	GO:0016020,GO:0015031	AT2G31530.1	EMB2289,SCY2	SecY protein transport family protein	preprotein translocase subunit SecY
Sapur.15WG080100.1	1.370747514	-1.249684559	0.279640582	-4.4689	7.86E-06	1.41E-05	brown	-0.270244333	0.000619317	GO:0016857,GO:0005975	AT5G61410.1	EMB2728,RPE	D-ribulose-5-phosphate-3-epimerase	ribulose-5-phosphate 3-epimerase
Sapur.T187700.2	8.422462812	0.546192221	0.122924536	4.443313	8.86E-06	1.58E-05	brown	-0.116624511	0.145782248					hypothetical protein
Sapur.012G002400.4	2.058842303	-1.213925641	0.273541051	-4.43782	9.09E-06	1.62E-05	brown	-0.248142713	0.001727159	GO:0000160,GO:0005515	AT5G24470.1	APRR5,PRR5	pseudo-response regulator 5	PRR response regulator
Sapur.009G093500.2	2.638594997	0.939984683	0.211861469	4.436789	9.13E-06	1.63E-05	brown	-0.170726222	0.032531367	GO:0016021	AT2G21600.1	ATRER1B,RER1B	endoplasmatic reticulum retrieval protein 1B	RER1A protein
Sapur.009G087600.4	3.080077098	-0.922788198	0.208728592	-4.421	9.82E-06	1.75E-05	brown	-0.225389586	0.00453731		AT3G18890.1		NAD(P)-binding Rossmann-fold superfamily protein	NAD-dependent epimerase/dehydratase family protein
Sapur.011G012200.4	2.544744014	-0.816227767	0.184644299	-4.42054	9.85E-06	1.75E-05	brown	-0.184124168	0.020976592		AT4G22290.1		Ubiquitin-specific protease family C19-related protein	ubiquitin thiolesterase, putative
Sapur.003G043800.1	7.942038444	-1.054347199	0.238600636	-4.41888	9.92E-06	1.77E-05	brown	-0.378499979	1.02E-06	GO:0006412,GO:0003735	AT1G70190.1		Ribosomal protein L7/L12, oligomerisation;Ribosomal protein L7/L12, C-terminal/adaptor protein ClpS-like	50S ribosomal protein L7/L12, putative
Sapur.005G031200.3	3.520317682	0.82708223	0.18786499	4.402535	1.07E-05	1.90E-05	brown	0.129219897	0.106754943	GO:0055085,GO:0016021	AT1G08930.1	ERD6	Major facilitator superfamily protein	NA
Sapur.003G116600.3	0.478811497	-1.656883616	0.378031441	-4.38293	1.17E-05	2.08E-05	brown	-0.189630914	0.017373531	GO:0006221,GO:0003883	AT1G30820.1		CTP synthase family protein	CTP synthase
Sapur.003G058000.1	13.28778437	-0.417422196	0.095427641	-4.37423	1.22E-05	2.16E-05	brown	-0.283851548	0.000315028		AT1G32540.3	LOL1	lsd one like 1	zinc finger, LSD1 subclass protein
Sapur.002G022900.1	27.62691115	-0.656068756	0.150119842	-4.3703	1.24E-05	2.20E-05	brown	-0.309738958	7.90E-05	GO:0055114,GO:0050660,GO:0016491,GO:0019139,GO:0009690	AT1G75450.1	ATCKX5,ATCKX6,CKX5	cytokinin oxidase 5	cytokinin oxidase/dehydrogenase
Sapur.006G145900.1	35.93461331	-0.617078297	0.141236118	-4.36913	1.25E-05	2.21E-05	brown	-0.394807804	3.11E-07	GO:0016758,GO:0008152	AT5G54010.1		UDP-Glycosyltransferase superfamily protein	UDP rhamnose:anthocyanidin-3-glucoside rhamnosyltransferase, putative
Sapur.010G111900.7	3.223424185	0.755547297	0.173724732	4.349106	1.37E-05	2.42E-05	brown	-0.081608864	0.309590942	GO:0005515	AT5G49430.1		WD40/YVTN repeat-like-containing domain;Bromodomain	cytochrome cd1-nitrite reductase-like, carboxy-terminal heme protein d1, putative
Sapur.017G081500.1	1.3302372	-1.09216436	0.252188538	-4.33075	1.49E-05	2.62E-05	brown	-0.331275425	2.26E-05	GO:0006355,GO:0005634,GO:0003700,GO:0046983,GO:0003677	AT3G02310.1	AGL4,SEP2	K-box region and MADS-box transcription factor family protein	MIKC MADS-box transcription factor SEPALLATA protein
Sapur.005G057600.1	10.43185954	-0.528956609	0.122159983	-4.33003	1.49E-05	2.63E-05	brown	-0.334507318	1.85E-05		AT5G22890.1		C2H2 and C2HC zinc fingers superfamily protein	NA
Sapur.005G052200.1	28.09507646	0.747625803	0.173308671	4.31384	1.60E-05	2.82E-05	brown	0.123807144	0.122381999	GO:0006629	AT3G52430.1	ATPAD4,PAD4	alpha/beta-Hydrolases superfamily protein	NA
Sapur.002G203900.3	2.47360193	-0.802709394	0.186164348	-4.31183	1.62E-05	2.85E-05	brown	-0.129433365	0.106172353	GO:0005975,GO:0004553	AT5G42720.1		Glycosyl hydrolase family 17 protein	glucan endo-1%2C3-beta-glucosidase, putative
Sapur.008G011700.3	7.717793757	-0.48405025	0.112441695	-4.3049	1.67E-05	2.94E-05	brown	-0.176176723	0.027304203	GO:0007034	AT5G04850.2	VPS60.2	SNF7 family protein	charged multivesicular body protein
Sapur.010G135600.4	19.14282922	-0.480816834	0.111995587	-4.29318	1.76E-05	3.09E-05	brown	-0.183074815	0.021731867		AT1G79380.1		Ca(2)-dependent phospholipid-binding protein (Copine) family	E3 ubiquitin-protein ligase RGLG1
Sapur.003G072100.5	1.037042421	-1.263250408	0.294293436	-4.29249	1.77E-05	3.10E-05	brown	-0.042508134	0.597077305	GO:0005089	AT1G31650.1	ATROPGEF14,ROPGEF14	RHO guanyl-nucleotide exchange factor 14	Rop guanine nucleotide exchange factor, putative
Sapur.017G100600.2	22.06474372	0.477585506	0.112054882	4.262068	2.03E-05	3.54E-05	brown	0.314639407	5.99E-05		AT3G14460.1		LRR and NB-ARC domains-containing disease resistance protein	NA
Sapur.002G123000.1	1.858222461	-1.197715965	0.282684249	-4.23694	2.27E-05	3.95E-05	brown	-0.180126826	0.023980333	GO:0005515,GO:0006950,GO:0006355	AT3G61050.1	NTMC2T4,NTMC2TYPE4	Calcium-dependent lipid-binding (CaLB domain) family protein	integral membrane single C2 domain protein
Sapur.004G114400.3	2.42164504	0.741406978	0.176340618	4.204403	2.62E-05	4.55E-05	brown	-0.063922497	0.426404114	GO:0048015,GO:0046854	AT1G49340.2	ATPI4K ALPHA	Phosphatidylinositol 3- and 4-kinase family protein	phosphatidylinositol 4-kinase
Sapur.018G003700.1	19.1484868	-0.470351992	0.111914113	-4.20279	2.64E-05	4.58E-05	brown	-0.315732719	5.62E-05		AT1G10330.1		Tetratricopeptide repeat (TPR)-like superfamily protein	NA
Sapur.014G036800.1	1.461631965	-1.33750387	0.31843738	-4.20021	2.67E-05	4.63E-05	brown	-0.283700925	0.000317454	GO:0043565,GO:0006355,GO:0003700				NA
Sapur.009G130000.3	3.972367469	-0.798009365	0.19040035	-4.19122	2.77E-05	4.82E-05	brown	-0.071910395	0.370790412	GO:0016567,GO:0004842,GO:0005515	AT5G42340.1	PUB15	Plant U-Box 15	E3 ubiquitin ligase PUB14, putative
Sapur.017G014800.1	3.587894307	-0.709063572	0.171273579	-4.13995	3.47E-05	6.00E-05	brown	-0.301534745	0.00012417		AT3G60470.1		Plant protein of unknown function (DUF247)	transmembrane protein, putative
Sapur.004G048300.1	18.75616123	-0.394372353	0.09527154	-4.13946	3.48E-05	6.01E-05	brown	-0.297102321	0.000157701		AT2G25720.1			hypothetical protein
Sapur.010G107100.1	18.23783993	-0.629730549	0.153421027	-4.10459	4.05E-05	6.96E-05	brown	-0.319593646	4.50E-05	GO:0016758,GO:0008152	AT5G03490.1		UDP-Glycosyltransferase superfamily protein	UDP-glucosyltransferase, putative
Sapur.012G003100.1	67.77393962	-0.376552675	0.091740157	-4.10456	4.05E-05	6.96E-05	brown	-0.294882834	0.000177499		AT3G49470.1	NACA2	nascent polypeptide-associated complex subunit alpha-like protein 2	nascent polypeptide-associated complex subunit alpha-like protein, putative
Sapur.004G146000.2	0.772701327	-1.101932422	0.268679387	-4.10129	4.11E-05	7.06E-05	brown	-0.371000519	1.72E-06	GO:0008270,GO:0003676	AT3G43590.1		zinc knuckle (CCHC-type) family protein	NA
Sapur.003G050500.1	135.3247104	-0.969769352	0.237363974	-4.08558	4.40E-05	7.54E-05	brown	-0.316356685	5.43E-05	GO:0016747	AT5G02890.1		HXXXD-type acyl-transferase family protein	transferase family protein
Sapur.014G018900.2	1.261653896	-0.996659836	0.244762323	-4.07195	4.66E-05	7.99E-05	brown	-0.13774921	0.085357723		AT4G33350.1		Tic22-like family protein	Tic22 family import component
Sapur.011G036300.2	1.927274057	-0.770390191	0.190058779	-4.05343	5.05E-05	8.63E-05	brown	-0.189457446	0.017478252		AT2G33770.1	ATUBC24,PHO2,UBC24	phosphate 2	ubiquitin-conjugating enzyme
Sapur.001G108700.5	3.104246431	-0.568358876	0.140761621	-4.03774	5.40E-05	9.22E-05	brown	-0.037887817	0.637563208		AT4G19070.1		Putative membrane lipoprotein	cadmium-induced protein AS8, putative
Sapur.005G175300.5	15.96500688	0.353798859	0.0876688	4.03563	5.45E-05	9.30E-05	brown	-0.081608864	0.309590942		AT4G19540.1	INDL	IND1(iron-sulfur protein required for NADH dehydrogenase)-like	MRP-like protein (ATP/GTP-binding protein)
Sapur.001G118600.1	0.46858472	-1.550200619	0.38477996	-4.0288	5.61E-05	9.56E-05	brown	-0.363843331	2.81E-06		AT4G17800.1		Predicted AT-hook DNA-binding family protein	AT-hook motif nuclear localized protein, putative
Sapur.010G204900.1	1.101899809	-1.18861348	0.29663541	-4.00698	6.15E-05	0.000104679	brown	-0.237374836	0.002758557		AT1G16810.2			7-dehydrocholesterol reductase, putative
Sapur.008G029900.1	130.3711588	-0.313751953	0.078556181	-3.99398	6.50E-05	0.000110422	brown	-0.265834517	0.000765337		AT5G19290.1		alpha/beta-Hydrolases superfamily protein	esterase/lipase/thioesterase family protein
Sapur.009G059600.2	1.115386731	-1.18087966	0.297791034	-3.96546	7.33E-05	0.000124048	brown	-0.280523523	0.000372855	GO:0050662,GO:0003824	AT5G58490.1		NAD(P)-binding Rossmann-fold superfamily protein	cinnamoyl-CoA reductase
Sapur.002G079400.3	0.86955034	-1.273145542	0.321922623	-3.95482	7.66E-05	0.00012955	brown	-0.207135622	0.009242419		AT4G08980.2	FBW2	F-BOX WITH WD-40 2	F-box protein, putative
Sapur.018G007500.1	3.00355458	-0.689658599	0.174457154	-3.95317	7.71E-05	0.000130427	brown	-0.290754379	0.000220611	GO:0008270,GO:0003676	AT4G31580.2	RSZP22,SRZ-22,SRZ22	serine/arginine-rich 22	RNA recognition motif 1 in vertebrate serine/arginine-rich splicing factor
Sapur.003G145200.1	2.284950159	-0.704660664	0.179162911	-3.93307	8.39E-05	0.000141471	brown	-0.184219445	0.020909151		AT3G11450.1		DnaJ domain ;Myb-like DNA-binding domain	DnaJ family C protein
Sapur.002G102500.4	9.24055636	0.518576404	0.132193771	3.922851	8.75E-05	0.000147476	brown	-0.074532331	0.353555021		AT5G42900.1	COR27	cold regulated gene 27	transmembrane protein, putative
Sapur.016G104100.6	0.85270654	-1.053652957	0.268652964	-3.92199	8.78E-05	0.000147992	brown	-0.078265667	0.329895244	GO:0016021,GO:0004659	AT3G51820.1	ATG4,CHLG,G4	UbiA prenyltransferase family protein	bacteriochlorophyll synthase, putative
Sapur.016G079600.1	72.31057625	0.318870106	0.081497981	3.912614	9.13E-05	0.000153669	brown	0.258176053	0.001096136	GO:0005515	AT5G02790.1	GSTL3	Glutathione S-transferase family protein	glutathione S-transferase
Sapur.019G035100.1	0.874943372	-1.351663059	0.350358884	-3.85794	0.000114347	0.000191577	brown	-0.231295527	0.003561516	GO:0045454	AT3G03860.1	APRL5,ATAPRL5	APR-like 5	5'-adenylylsulfate reductase-like protein, putative
Sapur.006G143900.2	0.542394309	1.313965778	0.343071405	3.830007	0.00012814	0.000214067	brown	-0.006746778	0.933165918	GO:0003968	AT2G19910.1		RNA-dependent RNA polymerase family protein	NA
Sapur.T091600.5	32.94052023	0.25547032	0.067099879	3.807314	0.000140484	0.000234121	brown	-0.169951101	0.033339589		AT3G59480.1		pfkB-like carbohydrate kinase family protein	pfkB-type carbohydrate kinase family protein
Sapur.15WG013600.1	1.971832146	1.107588368	0.291403201	3.800879	0.000144184	0.000240141	brown	0.265436918	0.000779948		AT5G53110.1		RING/U-box superfamily protein	NA
Sapur.002G052200.1	3.616635121	-0.676020215	0.178154736	-3.79457	0.000147901	0.000246085	brown	-0.257379947	0.00113715	GO:0006412,GO:0005840,GO:0005622,GO:0003735	AT1G43170.8	ARP1,emb2207,RP1,RPL3A	ribosomal protein 1	60S ribosomal protein L3
Sapur.017G100900.1	25.98269838	0.3939058	0.103834564	3.79359	0.000148485	0.000247025	brown	0.214193814	0.007065715		AT3G14470.1		NB-ARC domain-containing disease resistance protein	NA
Sapur.006G022900.1	0.513161102	-1.447354088	0.381823134	-3.79064	0.00015026	0.000249921	brown	-0.320084027	4.38E-05	GO:0016021	AT5G19750.1		Peroxisomal membrane 22 kDa (Mpv17/PMP22) family protein	Mpv17/PMP22 family protein
Sapur.013G029600.1	1.45251863	-0.997525823	0.263287489	-3.78873	0.000151418	0.000251785	brown	-0.233158326	0.003295565		AT5G27240.1		DNAJ heat shock N-terminal domain-containing protein	DnaJ heat shock amino-terminal domain protein, putative
Sapur.016G290900.3	3.358900244	-0.551018882	0.145788939	-3.77957	0.000157102	0.000260974	brown	0.070566546	0.379821058	GO:0055085,GO:0016021,GO:0008324,GO:0006812	AT3G58810.1	ATMTP3,ATMTPA2,MTP3,M	metal tolerance protein A2	metal tolerance protein
Sapur.009G003800.1	19.06978473	-0.375304956	0.099335225	-3.77817	0.000157988	0.000262406	brown	-0.2616617	0.00093203		AT5G57620.1	AtMYB36,MYB36	myb domain protein 36	transcription factor MYBJ7
Sapur.007G087100.3	2.630770261	0.797844041	0.212688698	3.751229	0.00017597	0.000291409	brown	-0.110658643	0.167675968	GO:0006468,GO:0004672	AT4G33950.1	ATOST1,OST1,P44,SNRK2-6	Protein kinase superfamily protein	Serine/Threonine-kinase SAPK2
Sapur.002G159600.4	4.039808911	0.762798426	0.203761435	3.743586	0.000181413	0.000300152	brown	-0.127292237	0.112130118	GO:0016829,GO:0006099	AT2G47510.2	FUM1	fumarase 1	NA
Sapur.010G193700.2	0.855008656	1.304231387	0.348770602	3.739511	0.000184379	0.000304907	brown	-0.081608864	0.309590942	GO:0006310,GO:0006281,GO:0006260,GO:0005634,GO:0003677	AT4G18590.1		Nucleic acid-binding, OB-fold-like protein	replication protein A 14 kDa subunit A
Sapur.013G101400.1	18.79353124	-0.321892447	0.086378485	-3.72654	0.00019413	0.0003206	brown	-0.281907202	0.000347712		AT1G35430.1			NA
Sapur.006G161000.1	152.5821932	-0.600393276	0.161385521	-3.72024	0.000199032	0.000328367	brown	-0.248468021	0.001702361	GO:0005515	AT2G44130.1		Galactose oxidase/kelch repeat superfamily protein	F-box protein
Sapur.013G052800.1	1.378723747	-0.775115667	0.208398224	-3.7194	0.000199699	0.000329403	brown	0.002894256	0.971302128		AT5G30495.1		Fcf2 pre-rRNA processing protein	NA
Sapur.009G121800.5	2.621507843	-0.584748138	0.157506022	-3.71254	0.000205186	0.000338132	brown	0.006697046	0.933657435		AT5G20930.1	TSL	Protein kinase superfamily protein	tousled-like kinase
Sapur.001G117500.1	1811.354939	-0.406438532	0.110062913	-3.69278	0.000221813	0.00036466	brown	-0.237103481	0.002790573					NA
Sapur.013G142600.6	3.22096519	-0.664113639	0.179932636	-3.6909	0.000223461	0.000367205	brown	-0.276015799	0.000466909		AT5G01960.1		RING/U-box superfamily protein	zinc finger, C3HC4 type (RING finger) protein
Sapur.009G036200.4	1.461114282	0.8050036	0.218445499	3.685146	0.000228571	0.000375398	brown	0.078548531	0.32814497	GO:0016021,GO:0015693,GO:0015095	AT1G34470.1		Protein of unknown function (DUF803)	Non-imprinted in prader-willi/angelman syndrome region protein, putative
Sapur.15WG090000.1	190.5550015	0.153294729	0.041721366	3.67425	0.000238549	0.0003913	brown	0.260415172	0.000987934	GO:0016567,GO:0004842,GO:0005515	AT5G65200.1	ATPUB38,PUB38	plant U-box 38	U-box protein
Sapur.017G078000.1	2.255303148	-1.142530897	0.311822724	-3.66404	0.000248268	0.000406556	brown	-0.270754674	0.000604189	GO:0003676	AT2G21660.1	ATGRP7.CCR2.GR-RBP7.GRI	cold, circadian rhythm, and rna binding 2	NA
Sapur.008G034100.1	0.780153513	-1.311936795	0.35909372	-3.65347	0.000258724	0.000423113	brown	-0.194722734	0.014533032		AT5G13610.1		Protein of unknown function (DUF155)	plant/MSH12-7 protein
Sapur.001G104200.3	14.84785698	-0.288670208	0.079160783	-3.64663	0.0002657	0.000434157	brown	-0.14527568	0.069463057	GO:0003723,GO:0003676,GO:0045131,GO:0000398	AT5G51300.3		splicing factor-related	zinc finger protein, putative
Sapur.15WG108400.3	3.184038937	-0.624020671	0.171205441	-3.64486	0.000267532	0.000437064	brown	-0.115551012	0.149553048	GO:0008234,GO:0006508	AT3G48480.1		Cysteine proteinases superfamily protein	sentrin/sumo-specific protease, putative
Sapur.T053800.1	9.272459368	-0.456394813	0.125346489	-3.64107	0.000271512	0.000443303	brown	-0.182766287	0.021958343	GO:0005515,GO:0006468,GO:0004672	AT3G14840.2		Leucine-rich repeat transmembrane protein kinase	NA
Sapur.013G100200.2	1.536315608	-0.874595898	0.241161451	-3.6266	0.000287179	0.000468189	brown	-0.285135641	0.00029503	GO:0006508,GO:0004176,GO:0030163,GO:0005524,GO:0004252	AT1G35340.1		ATP-dependent protease La (LON) domain protein	NA
Sapur.016G308600.3	6.147555429	0.465150132	0.128280268	3.626046	0.000287794	0.000469123	brown	-0.081608864	0.309590942		AT1G55690.3		Sec14p-like phosphatidylinositol transfer family protein	SEC14 cytosolic factor family protein
Sapur.001G148100.2	0.530222929	-1.344373299	0.371802145	-3.61583	0.000299387	0.000487563	brown	-0.318785034	4.72E-05	GO:0006355,GO:0005634,GO:0005524,GO:0032502,GO:0006351	AT3G13960.1	AtGRF5,GRF5	growth-regulating factor 5	QLQ protein
Sapur.016G139500.1	0.461348913	-1.707046087	0.47322998	-3.60722	0.000309492	0.000503672	brown	-0.2052388	0.009920296	GO:0051536,GO:0009228	AT2G29630.2	PY,THIC	thiaminC	thiamine biosynthesis protein ThiC
Sapur.002G064100.2	0.562538888	-1.213370349	0.336858662	-3.60202	0.000315759	0.000513517	brown	-0.26214677	0.000911072	GO:0005515	AT1G21700.1	ATSWI3C,CHB4,SWI3C	SWITCH/sucrose nonfermenting 3C	chromatin remodeling complex subunit
Sapur.006G182900.3	7.095974842	-0.502122615	0.139433002	-3.60117	0.000316782	0.000515157	brown	-0.1947537	0.014517079	GO:0016558,GO:0008270,GO:0007031,GO:0005779,GO:0005515	AT2G26350.1	ATPEX10,PEX10	peroxin 10	peroxisome biogenesis factor 10
Sapur.004G151200.1	1.277821933	-0.940567032	0.26131146	-3.59941	0.00031894	0.000518615	brown	-0.273371934	0.00053182	GO:0006355,GO:0005667,GO:0003700	AT5G22220.2	ATE2FB,E2F1,E2FB	E2F transcription factor 1	transcription factor E2F
Sapur.004G098000.2	1.074637376	-0.931245887	0.259112321	-3.59399	0.000325658	0.000529383	brown	-0.259673501	0.001022638	GO:0004045	AT5G38290.2		Peptidyl-tRNA hydrolase family protein	group IIB intron splicing facilitator CRS2
Sapur.003G005300.3	5.77541057	-0.625500332	0.174066204	-3.59346	0.000326313	0.000530395	brown	-0.069154573	0.389452387	GO:0003723	AT4G25880.1	APUM6,PUM6	pumilio 6	pumilio domain protein
Sapur.004G121900.2	17.7305993	-0.326987172	0.091129312	-3.58817	0.000333011	0.000540857	brown	-0.267175117	0.000717904	GO:0008270,GO:0003676	AT1G75560.1		zinc knuckle (CCHC-type) family protein	zinc knuckle protein
Sapur.005G101200.3	16.96953321	-0.494746069	0.137986986	-3.58545	0.000336492	0.000546322	brown	-0.099173288	0.216549061	GO:0003677	AT4G36740.1	ATHB40,HB-5,HB40	homeobox protein 40	homeobox leucine zipper protein ATHB-40
Sapur.007G006000.1	1.691472545	-1.191352114	0.332673964	-3.58114	0.000342098	0.000555234	brown	-0.245597401	0.001932836		AT3G49690.1	ATMYB84,MYB84,RAX3	myb domain protein 84	R2R3-Myb transcription factor MYB1.1
Sapur.008G085000.1	516.688175	-0.10883374	0.03049219	-3.56923	0.000358027	0.000580461	brown	-0.287736545	0.000258081		AT5G49400.1		zinc knuckle (CCHC-type) family protein	zinc knuckle protein
Sapur.001G062800.1	1.117228872	-1.01233648	0.284412937	-3.55939	0.000371718	0.000602125	brown	-0.21190621	0.007715182					NA
Sapur.016G283400.1	1.142133024	-0.792989423	0.223391144	-3.54978	0.000385553	0.000623742	brown	-0.284866382	0.000299123	GO:0005515	AT1G17170.1	ATGSTU24,GST,GSTU24	glutathione S-transferase TAU 24	tau class glutathione transferase GSTU8
Sapur.004G065600.1	287.998343	0.183544498	0.05214487	3.519896	0.000431717	0.000695974	brown	0.228874574	0.003935996		AT5G23530.1	AtCXE18,CXE18	carboxyesterase 18	neutral cholesterol ester hydrolase
Sapur.009G058900.1	2.34564911	-0.650526015	0.185328438	-3.51013	0.000447896	0.000721353	brown	-0.273940395	0.000517198	GO:0006468,GO:0004672	AT5G45840.2		Leucine-rich repeat protein kinase family protein	Serine/Threonine kinase domain protein
Sapur.005G166300.1	3.615039124	-0.564199328	0.160736788	-3.51008	0.000447968	0.000721435	brown	-0.217528093	0.006206102	GO:0008380,GO:0005681	AT1G03140.1		splicing factor Prp18 family protein	splicing factor Prp18 family protein
Sapur.009G020700.2	4.844941869	-0.412630524	0.11973165	-3.44629	0.000568331	0.000909428	brown	-0.267771193	0.000697696	GO:0005622,GO:0003779,GO:0030042,GO:0015629	AT5G59890.1	ADF4,ATADF4	actin depolymerizing factor 4	actin-depolymerizing factor
Sapur.008G046800.2	21.6324154	-0.400636213	0.116334397	-3.44383	0.00057353	0.000917526	brown	-0.261504847	0.000938901	GO:0006412,GO:0005840,GO:0005622,GO:0003735	AT1G74270.1		Ribosomal protein L35Ae family protein	60S ribosomal protein L35a
Sapur.010G135700.3	7.223205322	-0.360508822	0.106003397	-3.40092	0.000671601	0.001069246	brown	-0.178463968	0.02533464	GO:0005515,GO:0006355	AT1G79350.1	EMB1135	RING/FYVE/PHD zinc finger superfamily protein	strawberry notch-like protein
Sapur.005G190600.3	3.715589996	0.456059787	0.134901991	3.380675	0.00072308	0.001148773	brown	-0.081608864	0.309590942		AT2G28305.1	ATLOG1,LOG1	Putative lysine decarboxylase family protein	cytokinin riboside 5'-monophosphate phosphoribohydrolase
Sapur.009G032600.1	168.5810555	-0.227438208	0.06744936	-3.37198	0.000746286	0.001184446	brown	-0.272741073	0.000548492		AT2G29660.1		zinc finger (C2H2 type) family protein	zinc finger protein, putative
Sapur.016G080300.2	53.31223302	0.26486631	0.079129439	3.347254	0.000816165	0.001291694	brown	-0.01949924	0.808473797		AT3G09600.1		Homeodomain-like superfamily protein	MYB transcription factor
Sapur.002G080400.4	0.31536684	-1.304572692	0.390572811	-3.34015	0.000837324	0.001324296	brown	-0.081608864	0.309590942	GO:0006418,GO:0005524,GO:0004812,GO:0000166,GO:0006434,GO:0005737,GO:000482	AT1G11870.2	ATSRS,OVA7,SRS	Seryl-tRNA synthetase	seryl-tRNA synthetase/serine-tRNA ligase, putative
Sapur.005G057900.1	0.566381489	-1.987053121	0.596894281	-3.32899	0.000871626	0.001376374	brown	-0.251371661	0.001494967		AT1G34670.1	AtMYB93,MYB93	myb domain protein 93	Myb-like DNA-binding domain protein
Sapur.008G128700.1	0.83606707	-1.174763855	0.354358352	-3.31519	0.000915822	0.001443819	brown	-0.19916924	0.012393142		AT3G22910.1		ATPase E1-E2 type family protein / haloacid dehalogenase-like hydrolase family protein	NA
Sapur.019G011900.1	4.656677847	-0.491370666	0.148252843	-3.31441	0.000918368	0.001447627	brown	-0.219092781	0.005835857	GO:0007165,GO:0005515,GO:0043531	AT5G36930.1		Disease resistance protein (TIR-NBS-LRR class) family	NA
Sapur.010G194100.1	33.4704998	-0.293855311	0.088670462	-3.31402	0.000919663	0.00144953	brown	-0.242532215	0.002209949		AT3G54290.1			hemerythrin HHE cation-binding domain protein
Sapur.010G019600.3	1.490474537	-0.692174535	0.209171866	-3.30912	0.000935901	0.001474281	brown	-0.115783338	0.148730788		AT5G37190.1	CIP4	COP1-interacting protein 4	hypothetical protein
Sapur.010G066000.1	0.936988362	-1.065435503	0.322184744	-3.30691	0.000943317	0.001485679	brown	-0.190132578	0.017073741		AT1G04200.1			dyggve-melchior-clausen syndrome protein
Sapur.009G018800.2	2.816250188	-0.628969186	0.190283892	-3.30543	0.000948324	0.001493209	brown	-0.241068983	0.002354551	GO:0031011	AT3G45830.1			nuclear factor kappa-B-binding-like protein, putative
Sapur.002G035800.6	1.262319719	-0.786082315	0.238205581	-3.30002	0.000966792	0.001521564	brown	-0.081608864	0.309590942	GO:0006457,GO:0003755,GO:0000413	AT3G63400.3		Cyclophilin-like peptidyl-prolyl cis-trans isomerase family protein	peptidyl-prolyl cis-trans isomerase, cyclophilin-type protein
Sapur.003G038200.1	5.275691909	0.50008606	0.151588151	3.298979	0.000970373	0.001526765	brown	0.256713652	0.001172552		AT1G72510.2		Protein of unknown function (DUF1677)	DUF1677 family protein
Sapur.016G108300.1	46.5518539	0.240176112	0.072863889	3.29623	0.000979919	0.001541197	brown	0.222833157	0.005029493		AT2G38310.1	PYL4,RCAR10	PYR1-like 4	abscisic acid receptor PYL4
Sapur.018G119000.5	14.44699778	-0.328645578	0.100308991	-3.27633	0.001051648	0.001651106	brown	-0.0181084	0.82189923		AT5G53530.1	VPS26A	vacuolar protein sorting 26A	vacuolar protein sorting-associated protein
Sapur.018G060300.1	303.9815549	-0.130450147	0.039858412	-3.27284	0.001064733	0.001670936	brown	-0.27646753	0.00045658	GO:0006468,GO:0004672,GO:0005524	AT4G28980.2	CAK1AT,CDKF;1	CDK-activating kinase 1AT	cyclin-dependent kinase
Sapur.019G088400.1	3.136733059	0.566786706	0.173435729	3.267993	0.001083131	0.001698842	brown	0.213483064	0.007262093	GO:0046983,GO:0008171	AT4G35160.1		O-methyltransferase family protein	orcinol O-methyltransferase, putative
Sapur.018G045200.1	1.442245337	-0.84216292	0.25784413	-3.26617	0.001090125	0.001709407	brown	-0.223863517	0.004825643		AT2G34357.1		ARM repeat superfamily protein	RRP12-like protein
Sapur.008G160800.1	7.116001799	-0.368290938	0.112898024	-3.26216	0.001105684	0.001732818	brown	-0.244226912	0.002052578		AT3G16720.1	ATL2,TL2	TOXICOS EN LEVADURA 2	RING-H2 finger protein ATL2
Sapur.019G011800.1	31.48028425	-0.207123641	0.063514532	-3.26104	0.001110031	0.001739383	brown	-0.212307514	0.007597554	GO:0055114,GO:0016491,GO:0008270	AT5G63620.1		GroES-like zinc-binding alcohol dehydrogenase family protein	NA
Sapur.006G057600.1	88.39828027	0.552347498	0.170118736	3.246835	0.001166959	0.001826339	brown	0.227865281	0.004102309	GO:0048046,GO:0016762,GO:0006073,GO:0005618,GO:0005975,GO:0004553	AT4G25810.1	XTH23,XTR6	xyloglucan endotransglycosylase 6	xyloglucan endotransglycosylase/hydrolase
Sapur.013G074000.2	23.80040176	0.510635207	0.157278698	3.24669	0.001167554	0.001827183	brown	0.410183041	9.55E-08					NA
Sapur.016G095300.3	0.345409678	-1.271960957	0.392189117	-3.24323	0.001181812	0.001848972	brown	-0.17944497	0.024527956		AT2G38010.1		Neutral/alkaline non-lysosomal ceramidase	neutral/alkaline nonlysosomal ceramidase
Sapur.014G025700.5	40.15239405	0.222858686	0.06881971	3.238297	0.001202454	0.001879754	brown	-0.094099345	0.24109972	GO:0055085,GO:0016020,GO:0006810,GO:0005215	AT2G26510.2	PDE135	Xanthine/uracil permease family protein	nucleobase ascorbate transporter
Sapur.008G124700.1	0.939523506	-0.998129194	0.311083395	-3.20856	0.001334023	0.00207973	brown	-0.148366489	0.063675066		AT2G32090.1		Lactoylglutathione lyase / glyoxalase I family protein	lactoylglutathione lyase-like lyase
Sapur.009G076000.6	1.689610325	-0.641771468	0.200215877	-3.2054	0.001348761	0.002101716	brown	-0.260021413	0.001006222		AT2G01640.1			ribosome biogenesis protein SLX9
Sapur.008G163100.2	0.886536906	-1.139063761	0.355474543	-3.20435	0.001353693	0.002109102	brown	-0.188101716	0.018315741		AT4G15820.1			NA
Sapur.017G102200.4	0.44662427	-1.109387406	0.350324174	-3.16675	0.001541553	0.002392442	brown	-0.081608864	0.309590942	GO:0016742,GO:0009058	AT1G66520.1	pde194	formyltransferase, putative	methionyl-tRNA formyltransferase
Sapur.T079400.1	1.544119046	-0.608923827	0.192343798	-3.16581	0.00154652	0.002399813	brown	-0.181478963	0.022925381	GO:0005509	AT5G42380.1	CML37,CML39	calmodulin like 37	NA
Sapur.009G009800.4	27.80125354	0.242301873	0.076640562	3.161536	0.001569396	0.002434853	brown	-0.042149077	0.600181773	GO:0055114,GO:0016627,GO:0003995,GO:0050660,GO:0008152	AT3G45300.1	ATIVD,IVD,IVDH	isovaleryl-CoA-dehydrogenase	isovaleryl-CoA dehydrogenase
Sapur.004G173900.4	0.607278614	-0.99753426	0.315845138	-3.1583	0.001586912	0.002461105	brown	-0.081608864	0.309590942	GO:0006468,GO:0004672,GO:0005515	AT5G01950.1		Leucine-rich repeat protein kinase family protein	NA
Sapur.016G113700.1	820.6585246	-0.099106096	0.031624558	-3.13383	0.001725388	0.00266923	brown	-0.261002294	0.000961231	GO:0016020,GO:0008375	AT5G22070.1		Core-2/I-branching beta-1,6-N-acetylglucosaminyltransferase family protein	core-2/I-branching enzyme
Sapur.013G006600.1	0.432944109	-1.315797498	0.422030542	-3.11778	0.001822201	0.002813607	brown	-0.306005711	9.72E-05		AT1G09320.1		agenet domain-containing protein	agenet domain protein
Sapur.001G168800.2	0.472084415	-1.180869159	0.380310331	-3.10501	0.001902696	0.002933241	brown	-0.081608864	0.309590942		AT1G36050.2		Endoplasmic reticulum vesicle transporter protein	endoplasmic reticulum vesicle transporter protein
Sapur.008G029800.3	0.74339287	-1.19223566	0.387082229	-3.08006	0.002069604	0.003184169	brown	-0.249267172	0.001642813		AT5G49015.1		Expressed protein	hypothetical protein
Sapur.001G090800.5	6.293392917	-0.363374153	0.118372041	-3.06976	0.002142285	0.003292621	brown	-0.076434331	0.341371454		AT4G11790.1		Pleckstrin homology (PH) domain superfamily protein	RanBP1 domain protein
Sapur.006G175900.1	1.777790456	-1.155450711	0.376482811	-3.06907	0.002147289	0.003300007	brown	-0.218369897	0.006004386		AT2G39370.1			NA
Sapur.018G091700.1	1.90486318	-0.545343921	0.178021623	-3.06336	0.002188682	0.00336159	brown	-0.266507522	0.000741176	GO:0031515,GO:0030488,GO:0016429	AT5G14600.1		S-adenosyl-L-methionine-dependent methyltransferases superfamily protein	NA
Sapur.018G030600.2	1.078960362	-0.797021326	0.261106913	-3.05247	0.002269655	0.003482883	brown	-0.222854205	0.005025253		AT5G25630.1		Tetratricopeptide repeat (TPR)-like superfamily protein	PPR containing plant protein
Sapur.001G090900.4	1.197167138	-0.857412369	0.281527895	-3.04557	0.002322412	0.003560702	brown	-0.081608864	0.309590942	GO:0006351,GO:0003899,GO:0003677	AT1G63020.1	NRPD1.NRPD1A.POL IVA.SD	nuclear RNA polymerase D1A	DNA-directed RNA polymerase
Sapur.016G308400.2	0.360963192	-1.334800512	0.440493684	-3.03024	0.002443614	0.00373994	brown	-0.32173988	3.97E-05		AT5G56140.1		RNA-binding KH domain-containing protein	nucleic acid-binding protein, putative
Sapur.008G086000.7	0.271562106	-1.36416814	0.451138416	-3.02384	0.002495925	0.003816116	brown	-0.267355151	0.000711744		AT4G16146.1		cAMP-regulated phosphoprotein 19-related protein	negatively light-regulated protein, putative
Sapur.003G093600.1	10.40679261	-0.512380662	0.170422824	-3.00653	0.002642514	0.00403446	brown	-0.266721223	0.000733652		AT1G63800.1	UBC5	ubiquitin-conjugating enzyme 5	ubiquitin-conjugating enzyme E2
Sapur.007G068100.2	0.288031467	-1.659603234	0.552007747	-3.00649	0.002642867	0.004034627	brown	-0.238148579	0.002669084	GO:0046983,GO:0003677,GO:0006355,GO:0005634,GO:0003700	AT5G23260.2	ABS,AGL32,TT16	K-box region and MADS-box transcription factor family protein	MADS-box DNA-binding domain transcription factor
Sapur.001G107900.2	16.1228671	0.34968901	0.116448449	3.002951	0.002673753	0.004079894	brown	-0.10345754	0.197250991	GO:0008168	AT4G19120.1	ERD3	S-adenosyl-L-methionine-dependent methyltransferases superfamily protein	S-adenosyl-L-methionine-dependent methyltransferase, putative
Sapur.001G146600.3	37.16662207	-0.224113234	0.074646401	-3.00233	0.002679203	0.004088022	brown	-0.042157239	0.600111122	GO:0016020,GO:0008233,GO:0006465	AT1G52600.1		Peptidase S24/S26A/S26B/S26C family protein	microsomal signal peptidase subunit
Sapur.008G005500.4	1.176232341	-0.748194657	0.249528387	-2.99844	0.0027137	0.004140468	brown	-0.192326932	0.015814639	GO:0006418,GO:0005524,GO:0004812,GO:0000166	AT2G33840.1		Tyrosyl-tRNA synthetase, class Ib, bacterial/mitochondrial	tyrosyl-tRNA synthetase
Sapur.003G068200.5	2.428740233	0.819558532	0.27405546	2.990484	0.002785355	0.004245096	brown	0.246990994	0.001817621					hypothetical protein
Sapur.019G031600.1	56.98618307	-0.511384456	0.171473215	-2.9823	0.002860921	0.004356651	brown	-0.221637064	0.005275777	GO:0016788	AT5G33370.1		GDSL-like Lipase/Acylhydrolase superfamily protein	GDSL esterase/lipase
Sapur.013G099700.2	0.662104112	-0.91428095	0.306702459	-2.981	0.00287306	0.004374129	brown	-0.163311325	0.04098573		AT1G51965.1	ABO5	ABA Overly-Sensitive 5	PPR containing plant protein
Sapur.T079600.1	0.743285034	-0.806223775	0.271232598	-2.97244	0.002954389	0.004494019	brown	-0.297553445	0.000153938	GO:0005509	AT5G42380.1	CML37,CML39	calmodulin like 37	NA
Sapur.018G081100.1	0.299765077	-1.475159145	0.500658849	-2.94644	0.003214592	0.004874577	brown	-0.372464992	1.56E-06		AT3G43630.1		Vacuolar iron transporter (VIT) family protein	NA
Sapur.010G069200.1	0.487384656	-0.958512337	0.326407317	-2.93655	0.003318823	0.005027372	brown	-0.121078947	0.130902001	GO:0008017,GO:0007010	AT1G68060.1	ATMAP70-1,MAP70-1	microtubule-associated proteins 70-1	microtubule-associated protein 70-2
Sapur.005G207100.1	301.8792105	0.30078412	0.102658309	2.929954	0.003390122	0.005131809	brown	0.196109783	0.013833284		AT1G76560.1	CP12-3	CP12 domain-containing protein 3	CP12 domain protein
Sapur.010G186600.2	0.617480659	-1.048777466	0.35913166	-2.92031	0.003496781	0.005285764	brown	-0.25030536	0.001568289		AT3G52210.3		S-adenosyl-L-methionine-dependent methyltransferases superfamily protein	methyltransferase domain protein
Sapur.001G057100.1	1.403329703	-0.696292703	0.239385445	-2.90867	0.003629725	0.005478961	brown	-0.194663658	0.014563509		AT5G47860.1		Protein of unknown function (DUF1350)	plant/MCA23-20 protein
Sapur.005G206900.1	462.0478339	-0.108674847	0.037394186	-2.9062	0.003658518	0.005520912	brown	-0.21876003	0.005912901	GO:0046872,GO:0005634,GO:0003723	AT5G42820.1	ATU2AF35B,U2AF35B	Zinc finger C-x8-C-x5-C-x3-H type family protein	splicing factor U2af small protein
Sapur.002G068900.4	10.73798358	0.303905088	0.105012315	2.893995	0.003803745	0.005732483	brown	0.20161687	0.01133743		AT4G08240.1			hypothetical protein
Sapur.006G203200.1	0.566092801	-1.390005437	0.484256063	-2.87039	0.004099613	0.00616405	brown	-0.171473904	0.031767566	GO:0008270,GO:0005515	AT5G25560.1		CHY-type/CTCHY-type/RING-type Zinc finger protein	RING finger and CHY zinc finger protein
Sapur.004G059200.1	0.369880238	-1.247494423	0.435748874	-2.86287	0.004198166	0.006308215	brown	-0.329075942	2.57E-05		AT1G66140.1	ZFP4	zinc finger protein 4	zinc finger protein, putative
Sapur.001G183900.3	0.734282994	-0.982401067	0.346235432	-2.83738	0.004548561	0.006817071	brown	-0.031334039	0.696852582		AT1G09660.1		RNA-binding KH domain-containing protein	KH domain plant protein
Sapur.001G178900.1	4.714586735	-0.792672921	0.280718943	-2.82372	0.004746919	0.007103733	brown	-0.219133065	0.005826591	GO:0003677	AT1G13260.1	EDF4,RAV1	related to ABI3/VP1 1	B3 DNA-binding domain protein
Sapur.T190200.1	1.902711134	-0.488668046	0.174307563	-2.80348	0.005055412	0.007545588	brown	-0.244133806	0.002060952	GO:0055114,GO:0016491,GO:0045454	AT3G54660.1	ATGR2,EMB2360,GR	glutathione reductase	glutathione reductase
Sapur.008G155500.6	11.57193578	-0.225585568	0.080500766	-2.80228	0.005074304	0.007572078	brown	-0.115729365	0.148921509		AT1G70180.2		Sterile alpha motif (SAM) domain-containing protein	SAM domain protein, putative
Sapur.012G028800.1	39.99313939	-0.291761272	0.104229086	-2.79923	0.00512245	0.007639787	brown	-0.207977624	0.008954861		AT1G73500.1	ATMKK9,MKK9	MAP kinase kinase 9	MAP kinase kinase
Sapur.002G022500.2	5.703407318	0.363177683	0.129799548	2.797989	0.005142189	0.00766819	brown	0.179436277	0.024535006	GO:0055114,GO:0016491,GO:0016209,GO:0045454,GO:0015035,GO:0006662	AT1G45145.1	ATH5,ATTRX5,LIV1,TRX5	thioredoxin H-type 5	thioredoxin H-type protein
Sapur.005G019300.1	0.470330412	-1.431882337	0.512156646	-2.79579	0.005177306	0.007717772	brown	-0.192983393	0.015454036	GO:0006412,GO:0005840,GO:0005622,GO:0003735	AT5G27700.1		Ribosomal protein S21e	40S ribosomal protein S21
Sapur.003G122600.1	3.096015357	0.656131451	0.236756819	2.771331	0.005582769	0.008289315	brown	0.180704104	0.02352494		AT5G13210.1		Uncharacterised conserved protein UCP015417, vWA	NA
Sapur.002G173300.5	2.087342952	0.494200195	0.17865632	2.766206	0.005671269	0.008416941	brown	-0.081608864	0.309590942	GO:0046872	AT1G04990.1		Zinc finger C-x8-C-x5-C-x3-H type family protein	zinc finger CCCH domain protein
Sapur.013G038100.2	0.866403558	-0.787867512	0.284895015	-2.76547	0.005684151	0.008434925	brown	-0.33222114	2.13E-05		AT5G28840.1	GME	GDP-D-mannose 3\',5\'-epimerase	GDP-D-mannose-3'%2C5'-epimerase
Sapur.008G136000.1	11.34941357	-0.348890547	0.126295494	-2.76249	0.005736161	0.008510959	brown	-0.207900215	0.008980963		AT2G31160.1	LSH3	Protein of unknown function (DUF640)	DUF640 family protein
Sapur.15WG029400.1	5.843650466	-0.394896545	0.143380191	-2.75419	0.005883728	0.008719739	brown	-0.232641103	0.003367552		AT3G17930.1			NA
Sapur.017G017800.2	0.671381583	-0.815445594	0.296550968	-2.74977	0.005963794	0.008834044	brown	0.077982483	0.331653479		AT1G30890.1		Integral membrane HRF1 family protein	integral membrane HRF1 family protein
Sapur.013G108400.1	0.496847263	-1.043285253	0.381013717	-2.73818	0.00617797	0.009138202	brown	-0.200831842	0.011667073	GO:0005515	AT1G71840.1		transducin family protein / WD-40 repeat family protein	angio-associated migratory cell protein, putative
Sapur.013G056700.1	0.903466996	-0.884433125	0.323443395	-2.73443	0.00624885	0.009239738	brown	-0.176326699	0.027171164	GO:0005515,GO:0003723	AT5G17930.1		MIF4G domain-containing protein / MA3 domain-containing protein	MIF4G domain protein
Sapur.003G071100.1	0.418398122	-1.83447461	0.672060697	-2.72963	0.006340614	0.009370396	brown	-0.25807085	0.001101477	GO:0006508,GO:0004190	AT2G35615.1		Eukaryotic aspartyl protease family protein	aspartic proteinase nepenthesin protein
Sapur.001G117400.1	1808.745402	-0.358241968	0.131883585	-2.71635	0.006600611	0.009740267	brown	-0.174984547	0.028381679					NA
Sapur.008G124400.5	0.601402078	0.954003628	0.351777411	2.711952	0.006688818	0.009864709	brown	-0.081608864	0.309590942		AT4G14880.1	ATCYS-3A.CYTACS1.OASA1.(	O-acetylserine (thiol) lyase (OAS-TL) isoform A1	cysteine synthase
Sapur.002G021300.3	0.85352513	0.715492969	0.263935835	2.71086	0.006710905	0.009895077	brown	-0.081608864	0.309590942	GO:0033179,GO:0015991,GO:0015078	AT4G39080.1	VHA-A3	vacuolar proton ATPase A3	vacuolar proton ATPase a3
Sapur.014G002600.1	0.592021336	-0.97905988	0.361970066	-2.70481	0.006834368	0.010073528	brown	-0.228658647	0.003971058	GO:0043531	AT3G14460.1		LRR and NB-ARC domains-containing disease resistance protein	CC-NBS-LRR resistance protein
Sapur.016G297500.1	1.216408308	-0.698997241	0.259588156	-2.69272	0.007087256	0.010433718	brown	-0.196502548	0.013640571		AT5G23530.1	AtCXE18,CXE18	carboxyesterase 18	acetyl esterase, putative
Sapur.009G016200.1	0.233257603	1.514491713	0.566709827	2.672429	0.007530433	0.011061536	brown	0.08708668	0.278127621	GO:0055114,GO:0016491	AT4G25300.1		2-oxoglutarate (2OG) and Fe(II)-dependent oxygenase superfamily protein	flavonol synthase/flavanone 3-hydroxylase, putative
Sapur.005G021200.3	2.722696485	-0.414112499	0.155074996	-2.6704	0.00757606	0.011127075	brown	-0.132440013	0.09822921	GO:0019901,GO:0006355,GO:0000079,GO:0070985,GO:0016538,GO:0006351	AT5G27620.1	CYCH;1	cyclin H;1	cyclin H-1
Sapur.001G165500.3	1.316511779	0.609970279	0.228637758	2.667846	0.007633929	0.011207092	brown	0.042956499	0.593210842	GO:0008270,GO:0003676,GO:0016787,GO:0005524,GO:0003677	AT5G51280.1		DEAD-box protein abstrakt, putative	DEAD-box ATP-dependent RNA helicase, putative
Sapur.001G171200.1	1.037734109	-0.770196035	0.291039451	-2.64636	0.008136246	0.011913852	brown	-0.188326033	0.018174815	GO:0005515,GO:0006468,GO:0005524,GO:0004672	AT4G33430.1	ATBAK1.ATSERK3.BAK1.ELG	BRI1-associated receptor kinase	somatic embryogenesis receptor kinase
Sapur.014G062000.2	1.992655572	0.600298969	0.227269716	2.64135	0.008257625	0.012082494	brown	-0.035492717	0.658990221	GO:0009298,GO:0005737,GO:0004615	AT2G45790.1	ATPMM,PMM	phosphomannomutase	NA
Sapur.017G112400.2	0.409009605	1.310384732	0.496698571	2.638189	0.00833501	0.012189252	brown	-0.087483663	0.275934373	GO:0043531	AT3G14470.1		NB-ARC domain-containing disease resistance protein	CC-NBS-LRR resistance protein
Sapur.003G002300.1	1.088778476	-0.713418705	0.270604119	-2.63639	0.008379271	0.012248564	brown	-0.146755074	0.066641418	GO:0005515,GO:0006414,GO:0003746	AT1G09640.1		Translation elongation factor EF1B, gamma chain	elongation factor 1-gamma 1
Sapur.007G100400.3	2.628179541	-0.43828381	0.16708095	-2.62318	0.008711262	0.012715874	brown	-0.064664627	0.421042876		AT4G05440.1	EDA35	temperature sensing protein-related	cell division cycle-like protein
Sapur.010G129800.2	18.76397642	-0.249474939	0.095589144	-2.60987	0.009057747	0.013202412	brown	0.027839374	0.729263094	GO:0016020,GO:0008378,GO:0006486,GO:0030246	AT1G26810.1	GALT1	galactosyltransferase1	beta-1%2C3-galactosyltransferase
Sapur.T171300.1	6.931467057	-0.479151819	0.183626232	-2.60939	0.009070472	0.013219794	brown	-0.13059337	0.103050032	GO:0048046,GO:0016762,GO:0006073,GO:0005618,GO:0005975,GO:0004553	AT4G14130.1	XTH15,XTR7	xyloglucan endotransglucosylase/hydrolase 15	xyloglucan endotransglycosylase/hydrolase
Sapur.016G086100.1	11.5423089	0.280847996	0.108271201	2.593931	0.00948856	0.013807021	brown	0.198196413	0.012836111		AT5G03330.1		Cysteine proteinases superfamily protein	cysteine-type peptidase
Sapur.016G127200.1	0.961802808	-0.781197022	0.302143594	-2.58552	0.009723343	0.014135196	brown	-0.18781003	0.018500407	GO:0031011	AT3G45830.1			nuclear factor kappa-B-binding-like protein, putative
Sapur.014G006200.1	0.321172289	-1.471083874	0.569740191	-2.58203	0.009822224	0.014273297	brown	-0.476035392	2.95E-10	GO:0005515	AT4G07410.1		Transducin family protein / WD-40 repeat family protein	NA
Sapur.016G004400.4	3.324124289	-0.396347155	0.15425773	-2.56938	0.010187985	0.014785968	brown	-0.054811046	0.495362204	GO:0006468,GO:0004672,GO:0005524	AT5G47750.1	D6PKL2,PK5	D6 protein kinase like 2	Serine/Threonine kinase domain protein
Sapur.003G125700.1	1.765874441	-0.543916433	0.211844057	-2.56753	0.010242528	0.014863822	brown	-0.222067914	0.005185846		AT5G41020.1		myb family transcription factor	Myb-like DNA-binding domain protein
Sapur.004G078700.3	7.415905786	-0.31913135	0.124314485	-2.56713	0.010254439	0.014879801	brown	-0.115749337	0.148850914		AT5G15930.1	PAM1	plant adhesion molecule 1	plant adhesion molecule protein
Sapur.004G074700.4	1.930315789	-0.671106156	0.261925871	-2.5622	0.010401176	0.015084784	brown	-0.056877273	0.479225854	GO:0008270,GO:0006281,GO:0006351,GO:0006289,GO:0000439	AT1G05055.1	ATGTF2H2,GTF2H2	general transcription factor II H2	general transcription factor IIH subunit
Sapur.016G182100.1	8.661440694	0.322956743	0.12627941	2.557477	0.010543439	0.015284406	brown	-0.042702753	0.595397613	GO:0016758,GO:0008152	AT4G34131.1	UGT73B3	UDP-glucosyl transferase 73B3	UDP-glucose:glucosyltransferase
Sapur.014G138700.1	1.536269275	-0.558754873	0.219503101	-2.54554	0.010910751	0.015791273	brown	-0.108585588	0.175829782	GO:0008270,GO:0005622,GO:0005515	AT3G07650.3	COL9	CONSTANS-like 9	zinc finger constans-like protein
Sapur.016G174600.3	1.744044944	0.46645482	0.184088677	2.533859	0.011281399	0.016304173	brown	-0.071712452	0.372112234	GO:0007264,GO:0005525	AT3G49870.1	ARLA1C,ATARLA1C	ADP-ribosylation factor-like A1C	ADP-ribosylation factor-like protein
Sapur.017G019900.2	8.404172146	0.536483396	0.212339945	2.526531	0.011519531	0.016633791	brown	-0.07017963	0.382445746	GO:0016567,GO:0004842	AT2G31510.1	ARI7,ATARI7	IBR domain-containing protein	E3 ubiquitin-protein ligase ARI7, putative
Sapur.016G263200.1	0.452296329	-2.266981813	0.898280859	-2.52369	0.011613051	0.016764438	brown	-0.269781993	0.000633323		AT5G53742.1		Protein of unknown function (DUF1278)	prolamin-like protein
Sapur.014G037900.1	4.738137869	-0.715250024	0.283959386	-2.51885	0.011774007	0.01698419	brown	-0.247379984	0.001786597		AT1G02150.1		Tetratricopeptide repeat (TPR)-like superfamily protein	PPR containing plant protein
Sapur.002G100600.1	0.691378495	0.779406308	0.312717884	2.492362	0.012689647	0.018234236	brown	-0.081608864	0.309590942		AT3G57870.1	AHUS5.ATSCE1.EMB1637.S(	sumo conjugation enzyme 1	SUMO E2 conjugating enzyme SCE1
Sapur.012G068200.1	0.252922122	-1.352015596	0.543428014	-2.48794	0.012848587	0.0184522	brown	-0.404305372	1.51E-07		AT4G24790.1		AAA-type ATPase family protein	DNA polymerase III subunit gamma/tau
Sapur.014G068900.1	1.11484625	0.61989233	0.249219198	2.487338	0.012870313	0.018480994	brown	0.201457031	0.011403876		AT2G46080.1			NA
Sapur.152G052300.1	1.124837751	-0.59915448	0.242617667	-2.46954	0.01352862	0.019387563	brown	-0.174969611	0.028395406		AT2G27030.3	ACAM-2,CAM5	calmodulin 5	NA
Sapur.014G120100.2	13.68680104	0.338939352	0.137659749	2.462153	0.013810578	0.019772782	brown	0.021337749	0.790813093		AT1G60710.1	ATB2	NAD(P)-linked oxidoreductase superfamily protein	aldo/keto reductase family protein
Sapur.005G203900.4	0.373368734	-1.166159111	0.474063298	-2.45992	0.013896691	0.019889184	brown	-0.275496967	0.000479039	GO:0006457	AT1G20810.1		FKBP-like peptidyl-prolyl cis-trans isomerase family protein	peptidyl-prolyl cis-trans isomerase FKBP18
Sapur.005G094000.1	0.275545482	-1.449414945	0.596084957	-2.43156	0.015034055	0.02145387	brown	-0.26322532	0.000866012	GO:0043565,GO:0008270,GO:0006355,GO:0003700	AT5G66320.1	GATA5	GATA transcription factor 5	GATA transcription factor, putative
Sapur.017G127800.1	0.567659774	1.102888227	0.45431288	2.427596	0.015199255	0.021671846	brown	0.140956031	0.078261642	GO:0004866	AT1G17860.1		Kunitz family trypsin and protease inhibitor protein	kunitz-type protease inhibitor
Sapur.018G082200.2	2.135274269	-0.481779606	0.199117373	-2.41958	0.015538616	0.022136638	brown	-0.390208824	4.37E-07	GO:0043565,GO:0006355,GO:0003700	AT4G30935.1	ATWRKY32,WRKY32	WRKY DNA-binding protein 32	WRKY transcription factor, putative
Sapur.019G097100.1	1.37349745	-0.555914541	0.230341784	-2.41343	0.015803018	0.022492965	brown	-0.19374167	0.015046444		AT1G03670.1		ankyrin repeat family protein	NA
Sapur.017G042500.1	0.321882394	-1.432294219	0.593854149	-2.41186	0.01587129	0.022586251	brown	-0.20245612	0.010994101	GO:0007050,GO:0005634,GO:0004861	AT5G48820.1	ICK6,KRP3	inhibitor/interactor with cyclin-dependent kinase	cyclin-dependent kinase inhibitor
Sapur.012G057000.1	33.31464348	-0.171653728	0.071208499	-2.41058	0.015927213	0.022662908	brown	-0.189513965	0.017444072	GO:0003682,GO:0006351,GO:0005634,GO:0003677	AT3G48050.1		BAH domain ;TFIIS helical bundle-like domain	BAH and TFIIS domain protein
Sapur.006G205200.2	0.278185715	-1.328463478	0.552895417	-2.40274	0.016272789	0.023119818	brown	-0.298615225	0.000145407	GO:0055114,GO:0016491	AT1G52820.1		2-oxoglutarate (2OG) and Fe(II)-dependent oxygenase superfamily protein	NA
Sapur.010G111300.3	0.829736096	0.698741829	0.29126291	2.399007	0.01643959	0.023342764	brown	0.047900702	0.55134894		AT3G06670.1		binding	NA
Sapur.006G140100.4	0.469818324	0.886718428	0.372111815	2.382935	0.017175207	0.024340251	brown	0.002063705	0.979535279	GO:0008152,GO:0003824,GO:0016114,GO:0008661	AT4G15560.1	CLA,CLA1,DEF,DXPS2,DXS	Deoxyxylulose-5-phosphate synthase	1-deoxy-D-xylulose-5-phosphate synthase
Sapur.005G032100.2	10.38501438	-0.236524523	0.099810842	-2.36973	0.017801187	0.025197143	brown	-0.186034796	0.01965947	GO:0016787,GO:0008152	AT5G27320.1	ATGID1C,GID1C	alpha/beta-Hydrolases superfamily protein	gibberellin receptor GID1, putative
Sapur.012G081300.1	0.492339885	-0.870227825	0.367234496	-2.36968	0.017803541	0.025199398	brown	-0.224344661	0.004733012	GO:0008270,GO:0004553,GO:0005975,GO:0004559,GO:0015923,GO:0006013	AT5G13980.1		Glycosyl hydrolase family 38 protein	glycoside hydrolase family 38 protein
Sapur.016G263000.1	0.559736298	-1.263353712	0.533585393	-2.36767	0.017900532	0.025332343	brown	-0.18172757	0.022735823		AT5G53742.1		Protein of unknown function (DUF1278)	prolamin-like protein
Sapur.010G071100.1	0.320938972	-1.257022561	0.537747985	-2.33757	0.019409662	0.027387166	brown	-0.254283264	0.001310401		AT1G66140.1	ZFP4	zinc finger protein 4	zinc finger protein, putative
Sapur.002G180700.1	848.9619477	-0.095769477	0.041062806	-2.33227	0.019686584	0.02776014	brown	-0.210131037	0.008255122	GO:0055114,GO:0020037,GO:0016705,GO:0005506	AT1G64940.1	CYP89A6	cytochrome P450, family 87, subfamily A, polypeptide 6	cytochrome P450 family protein
Sapur.15WG086000.1	0.599736302	0.816058195	0.350720012	2.326808	0.019975476	0.028150706	brown	0.193082609	0.015400164	GO:0055085,GO:0016021	AT1G74780.1		Nodulin-like / Major Facilitator Superfamily protein	nodulin-like protein
Sapur.018G022300.1	5.169807714	-0.351331276	0.151012066	-2.32651	0.019991291	0.028171792	brown	-0.126116841	0.115509955	GO:0009116	AT5G11160.1	APT5	adenine phosphoribosyltransferase 5	adenine phosphoribosyltransferase
Sapur.010G009300.1	1.072582857	-0.682080231	0.294139571	-2.3189	0.020400463	0.028721476	brown	-0.148286494	0.06381973		AT4G15830.1		ARM repeat superfamily protein	clasp amino-terminal protein
Sapur.016G286400.1	0.816288541	-0.775638075	0.334968106	-2.31556	0.020582415	0.028966545	brown	-0.145508401	0.069012857	GO:0006468,GO:0004672	AT4G21390.1	B120	S-locus lectin protein kinase family protein	NA
Sapur.019G008600.1	0.455657272	-0.943765753	0.408137282	-2.31237	0.02075712	0.02920496	brown	-0.121522762	0.129485788		AT2G02100.1	LCR69,PDF2.2	low-molecular-weight cysteine-rich 69	gamma-thionin, putative
Sapur.001G143700.2	0.634500042	0.802855968	0.348484593	2.303849	0.021231107	0.0298518	brown	0.178915	0.024960972		AT1G80280.1		alpha/beta-Hydrolases superfamily protein	alpha/beta fold hydrolase
Sapur.017G020100.9	24.77451702	0.166017901	0.072162068	2.300626	0.021412803	0.030099326	brown	-0.115107846	0.151131044	GO:0055085,GO:0016021	AT2G43330.1	ATINT1,INT1	inositol transporter 1	sugar porter (SP) family MFS transporter
Sapur.T079700.1	0.994876975	0.917127031	0.39955602	2.295365	0.021712197	0.03049555	brown	-0.045228308	0.573796931	GO:0005509				NA
Sapur.001G141600.9	14.10333483	-0.249854631	0.109245112	-2.2871	0.022189915	0.031122906	brown	-0.081608864	0.309590942		AT4G19160.2			transglutaminase family protein
Sapur.002G107300.3	1.392252749	-0.667703322	0.295161918	-2.26216	0.023687554	0.033136232	brown	-0.112353917	0.161219824	GO:0016567,GO:0004842	AT1G65430.1	ARI8,ATARI8	IBR domain-containing protein	ariadne-1 protein, putative
Sapur.010G040700.1	0.469472676	1.34347623	0.594187602	2.26103	0.023757375	0.033228376	brown	0.151010251	0.059042908	GO:0055085,GO:0016021	AT5G43360.1	ATPT4,PHT1;3,PHT3	phosphate transporter 1;3	inorganic phosphate transporter, putative
Sapur.005G184900.2	1.296551701	-0.569967777	0.258171866	-2.20771	0.02726472	0.037933424	brown	-0.100926505	0.208495218		AT1G04945.3		HIT-type Zinc finger family protein	NA
Sapur.010G050700.2	0.809656	0.663815496	0.303052768	2.190429	0.028493157	0.039570971	brown	-0.014962225	0.852453854	GO:0055114,GO:0047134	AT1G60420.1		DC1 domain-containing protein	protein disulfide isomerase (PDI)-like protein
Sapur.003G003800.3	8.842755494	-0.290772456	0.133330917	-2.18083	0.029195775	0.040495746	brown	-0.146812324	0.066534132	GO:0006412,GO:0005840,GO:0005622,GO:0003735	AT1G61580.1	ARP2,RPL3B	R-protein L3 B	60S ribosomal protein L3
Sapur.15WG087400.3	0.988906049	-0.618253595	0.283551857	-2.18039	0.029228575	0.04053954	brown	-0.049600786	0.537294403	GO:0046983	AT5G62610.1		basic helix-loop-helix (bHLH) DNA-binding superfamily protein	BHLH transcription factor, putative
Sapur.15WG132800.2	0.36409258	0.87407504	0.404303049	2.16193	0.030623544	0.042370267	brown	0.207388044	0.009155368	GO:0005515	AT4G19880.1		Glutathione S-transferase family protein	glutathione S-transferase-like protein
Sapur.004G070100.3	3.010930698	-0.339765357	0.157882439	-2.15201	0.031396183	0.043389493	brown	-0.184387668	0.020790534	GO:0043565,GO:0006355,GO:0003700	AT2G03340.1	WRKY3	WRKY DNA-binding protein 3	WRKY transcription factor

Sapur.012G033700.1	0.338521019	-1.732756909	0.807234708	-2.14653	0.031830383	0.043963839	brown	-0.274288987	0.000508416		AT3G25170.1	RALFL26	ralf-like 26	rapid aLkalinization factor
Sapur.012G062900.3	1.601429829	0.503008018	0.234406043	2.145883	0.031882289	0.044030014	brown	0.176761573	0.026788529	GO:0005524,GO:0003676	AT5G63120.1		P-loop containing nucleoside triphosphate hydrolases superfamily protein	DEAD-box ATP-dependent RNA helicase
Sapur.010G167500.1	12.39950554	-0.223092161	0.104632054	-2.13216	0.032993788	0.045508009	brown	-0.147003943	0.066176067	GO:0005975,GO:0004553	AT3G55260.1	ATHEX2,HEXO1	beta-hexosaminidase 1	beta-hexosaminidase
Sapur.010G196300.5	9.814301073	-0.225089983	0.105595446	-2.13163	0.033037616	0.04556276	brown	-0.081608864	0.309590942	GO:0007034	AT3G10640.1	VPS60.1	SNF7 family protein	charged multivesicular body protein
Sapur.008G005200.1	203.4461884	0.093119829	0.043735017	2.129182	0.033239169	0.045823529	brown	0.130976587	0.102034588		AT1G51660.1	ATMEK4,ATMKK4,MKK4	mitogen-activated protein kinase kinase 4	MAP kinase kinase
Sapur.006G083200.3	0.418359642	-1.030340078	0.484248932	-2.12771	0.033361344	0.045984292	brown	-0.119733809	0.135267041	GO:0006629,GO:0055114,GO:0016717	AT5G05580.1	FAD8	fatty acid desaturase 8	omega-3 fatty acid desaturase
Sapur.016G106200.1	11.0884154	-0.247010074	0.116287029	-2.12414	0.033658334	0.046374328	brown	-0.162322304	0.042241767	GO:0016787	AT1G28960.4	ATNUDT15.ATNUDX15.NUI	nudix hydrolase homolog 15	nudix hydrolase
Sapur.016G171300.1	0.216630512	1.476662141	0.695881931	2.122001	0.033837656	0.046611688	brown	0.084155697	0.294683913		AT2G14610.1	ATPR1,PR 1,PR1	pathogenesis-related gene 1	cysteine-rich secretory family protein
Sapur.016G003600.1	36.87408598	0.185005251	0.088329964	2.094479	0.036217336	0.04973643	brown	0.10865342	0.175558428					transmembrane protein, putative
Sapur.018G088800.2	0.608800149	-0.669647179	0.320754655	-2.08772	0.036822742	0.050517478	brown	-0.120997319	0.131163768					NA
Sapur.016G263400.1	0.383606346	-1.306687121	0.626444245	-2.08588	0.036989552	0.050735805	brown	-0.21295503	0.007411112		AT5G53742.1		Protein of unknown function (DUF1278)	NA
Sapur.013G027300.1	1.660931406	0.785431304	0.377236566	2.082066	0.037336472	0.051196785	brown	0.144878373	0.070237165		AT4G24310.1		Protein of unknown function (DUF679)	NA
Sapur.013G036700.7	5.218976498	-0.270178427	0.129980214	-2.07861	0.037653031	0.051609463	brown	-0.152967746	0.055794692		AT3G04830.2		Protein prenylyltransferase superfamily protein	O-linked GlcNAc transferase-like protein
Sapur.007G007500.1	0.383517136	-0.916320968	0.440988658	-2.07788	0.037720527	0.051691266	brown	-0.194050425	0.014883186	GO:0003676	AT5G09880.1		Splicing factor, CC1-like	splicing factor-like protein
Sapur.001G161100.1	13.08806346	0.382230805	0.185029639	2.065781	0.038849116	0.053156365	brown	0.145761591	0.068525759		AT5G56550.1	ATOXS3,OXS3	oxidative stress 3	hypothetical protein
Sapur.013G117500.1	0.636395387	-0.78068618	0.37807221	-2.06491	0.038931243	0.053262128	brown	-0.138123061	0.084504837	GO:0006468,GO:0005524,GO:0004672	AT1G53050.1		Protein kinase superfamily protein	cyclin-dependent kinase-like Serine/Threonine kinase family protein
Sapur.003G072200.1	2.043496622	-0.426191267	0.206923319	-2.05966	0.039431249	0.053912738	brown	-0.142937098	0.074120913	GO:0016161,GO:0000272	AT5G45300.1	BAM8,BMY2	beta-amylase 2	beta-amylase
Sapur.017G058200.3	0.739723108	-0.692104037	0.33866678	-2.04361	0.040991735	0.055963041	brown	-0.127196191	0.112403365	GO:0005515,GO:0003723	AT2G39260.1		binding;RNA binding	NA
Sapur.014G020200.1	0.608346536	-0.586915801	0.287871813	-2.03881	0.041469039	0.056586641	brown	-0.228889897	0.003933518	GO:0005509	AT5G42380.1	CML37,CML39	calmodulin like 37	EF hand protein
Sapur.016G165400.1	19.0051816	0.227882863	0.111793649	2.038424	0.041507542	0.056636843	brown	0.151043811	0.058985938	GO:0006468,GO:0005524,GO:0004672	AT5G58520.1		Protein kinase superfamily protein	tyrosine kinase family protein
Sapur.004G134100.1	0.657032776	-0.828445659	0.407092413	-2.03503	0.041847759	0.05707752	brown	-0.086357931	0.282184322	GO:0008536,GO:0006886	AT2G16950.2	ATTRN1,TRN1	transportin 1	transportin-1 protein
Sapur.009G032100.1	488.894001	-0.079991828	0.039332251	-2.03375	0.04197716	0.057239851	brown	-0.169544108	0.03377073	GO:0005515	AT1G07310.1		Calcium-dependent lipid-binding (CaLB domain) family protein	C2 domain-like protein
Sapur.017G118900.1	23.59981419	-0.216401458	0.106420956	-2.03345	0.042007304	0.057276232	brown	-0.187265033	0.018849771	GO:0043565,GO:0006355,GO:0003700	AT5G52830.1	ATWRKY27,WRKY27	WRKY DNA-binding protein 27	WRKY DNA-binding domain protein
Sapur.012G009300.2	2.005010101	-0.409476912	0.201627529	-2.03086	0.042269386	0.057616949	brown	-0.133460814	0.095641911	GO:0016021,GO:0015689,GO:0015098	AT4G27720.1		Major facilitator superfamily protein	MFS transporter
Sapur.014G000600.2	2.442731791	-0.351585946	0.173355277	-2.02812	0.042547624	0.057977098	brown	-0.054143267	0.500638042		AT3G13060.2	ECT5	evolutionarily conserved C-terminal region 5	YTH domain protein
Sapur.005G078600.2	0.678970818	-0.612571306	0.304868776	-2.00929	0.044505858	0.060525771	brown	-0.159224416	0.046386488	GO:0016021,GO:0006952	AT2G17480.1	ATMLO8,MLO8	Seven transmembrane MLO family protein	MLO-like protein
Sapur.016G042700.2	0.346621205	-1.023411754	0.510813455	-2.00349	0.045124278	0.061306292	brown	-0.271689839	0.000577349					NA
Sapur.007G101200.1	1.157997572	-0.583016902	0.293952682	-1.98337	0.047326127	0.064144919	brown	-0.115127642	0.151060287	GO:0006457	AT5G64350.1	ATFKBP12,FKBP12	FK506-binding protein 12	peptidyl-prolyl cis-trans isomerase FKBP12
Sapur.009G033500.1	6.645016052	0.25039016	0.126690723	1.976389	0.048110728	0.065160288	brown	0.128263268	0.109396614	GO:0016758,GO:0008152	AT2G29730.1	UGT71D1	UDP-glucosyl transferase 71D1	UDP-glucosyltransferase, putative
Sapur.002G108700.2	1.081160294	-0.544929068	0.276159002	-1.97324	0.048467781	0.065614317	brown	-0.217034898	0.00632707	GO:0008725,GO:0006284	AT1G75090.1		DNA glycosylase superfamily protein	DNA-3-methyladenine glycosylase I
Sapur.019G044300.1	1.463686758	-0.51942557	0.263572263	-1.97071	0.048756589	0.065983691	brown	-0.125756216	0.116562675		AT5G36930.2		Disease resistance protein (TIR-NBS-LRR class) family	NA
Sapur.012G033600.1	0.300764217	-1.682722696	0.854391709	-1.9695	0.048895941	0.066153332	brown	-0.276287641	0.000460667		AT3G25170.1	RALFL26	ralf-like 26	rapid aLkalinization factor
Sapur.018G100800.1	0.45117121	0.8198242	0.420016447	1.951886	0.050951747	0.068844598	brown	0.154692245	0.053056595	GO:0009055	AT5G20230.1	ATBCB,BCB,SAG14	blue-copper-binding protein	NA
Sapur.010G126600.2	0.430453979	0.675251579	0.345993597	1.95163	0.050982116	0.068882818	brown	-0.085210144	0.288653877	GO:0005515,GO:0003676	AT1G69440.1	AGO7,ZIP	Argonaute family protein	argonaute protein
Sapur.013G142700.1	0.484528149	-0.897717793	0.461067777	-1.94704	0.051529838	0.069574538	brown	-0.128455674	0.108861226		AT2G30530.1			hypothetical protein
Sapur.T166800.7	0.322511505	0.858408743	0.441163751	1.945783	0.051680862	0.069764206	brown	-0.119859799	0.134853532		AT1G21080.3		DNAJ heat shock N-terminal domain-containing protein	DnaJ heat shock amino-terminal domain protein
Sapur.016G272600.1	1.249155738	-0.728999297	0.374762508	-1.94523	0.051747295	0.069851034	brown	-0.172742709	0.03050625		AT5G53742.1		Protein of unknown function (DUF1278)	prolamin-like protein
Sapur.008G085300.1	0.347953191	-0.833639498	0.429505977	-1.94093	0.052267203	0.070515418	brown	-0.114924621	0.151787115	GO:0006412,GO:0005840,GO:0005622,GO:0003735	AT3G06680.3		Ribosomal L29e protein family	60S ribosomal protein L29
Sapur.003G052500.3	0.873877791	-0.579565907	0.302879918	-1.91352	0.055681885	0.074902292	brown	-0.161898331	0.042789996	GO:0006468,GO:0005524,GO:0004672	AT4G33950.1	ATOST1.OST1.P44.SNRK2-6.	Protein kinase superfamily protein	Serine/Threonine-kinase SAPK3, putative
Sapur.010G161200.1	4.432062451	0.407475834	0.213303216	1.910313	0.056092968	0.075418465	brown	-0.062901819	0.433841643		AT5G02500.1	AT-HSC70-1.HSC70.HSC70-:	heat shock cognate protein 70-1	heat shock cognate 70 kDa protein
Sapur.016G270500.4	1.877304925	-0.358678029	0.189024875	-1.89752	0.057759657	0.077536443	brown	-0.081608864	0.309590942	GO:0043486,GO:0006355,GO:0006338,GO:0005634	AT2G36740.1	ATSWC2,SWC2	sequence-specific DNA binding transcription factors;DNA binding;DNA binding	YL1 nuclear protein
Sapur.002G005800.3	10.46986492	0.203098418	0.107307512	1.892677	0.05840089	0.078352711	brown	-0.130322444	0.103772716		AT1G20670.1		DNA-binding bromodomain-containing protein	bromodomain domain protein
Sapur.014G075900.6	1.192693095	-0.420011896	0.222236212	-1.88993	0.058766713	0.078817935	brown	0.032310139	0.687894046	GO:0046872	AT1G01350.1		Zinc finger (CCCH-type/C3HC4-type RING finger) family protein	RING zinc finger protein, putative
Sapur.T017000.1	7.816712025	-0.258027654	0.13666865	-1.88798	0.059028659	0.079149999	brown	-0.128494586	0.108753197	GO:0005524,GO:0051082,GO:0006457	AT3G02530.1		TCP-1/cpn60 chaperonin family protein	NA
Sapur.016G196900.1	33.35724839	-0.14327185	0.076174387	-1.88084	0.05999366	0.080375514	brown	-0.145030598	0.069939747		AT3G18790.1			NA
Sapur.010G040500.1	0.792019924	0.70151324	0.373231066	1.879568	0.060166952	0.080604414	brown	0.130942746	0.102123941					transmembrane protein, putative
Sapur.002G130600.3	8.469251442	-0.182072198	0.09697944	-1.87743	0.06045906	0.080972786	brown	-0.105799849	0.187242906	GO:0016021,GO:0004190	AT1G01650.1	ATSPPL4,SPPL4	SIGNAL PEPTIDE PEPTIDASE-LIKE 4	signal peptide peptidase-like protein
Sapur.018G115300.1	0.468014397	0.770426553	0.411961403	1.870143	0.061464023	0.082242136	brown	0.124013572	0.121755226	GO:0005515	AT1G58190.2	AtRLP9,RLP9	receptor like protein 9	NA
Sapur.003G039800.1	3.96728388	0.306194194	0.164378052	1.862744	0.062498324	0.083561853	brown	0.142165838	0.075711373	GO:0016192,GO:0016021	AT1G11890.1	ATSEC22,SEC22	Synaptobrevin family protein	25.3 kDa vesicle transporter
Sapur.003G062000.3	12.91968336	0.154168659	0.082946474	1.858652	0.063076431	0.084300719	brown	-0.001137159	0.988722516	GO:0043565,GO:0006355,GO:0005634,GO:0003700	AT1G32330.1	ATHSFA1D,HSFA1D	heat shock transcription factor A1D	heat shock transcription factor 1
Sapur.001G090800.4	3.540755631	-0.289454473	0.156092377	-1.85438	0.063684913	0.085038347	brown	-0.035805759	0.656173406		AT4G11790.1		Pleckstrin homology (PH) domain superfamily protein	RanBP1 domain protein
Sapur.004G112400.1	1.934120709	-0.451272242	0.243942143	-1.84992	0.064325794	0.085869846	brown	-0.173459824	0.029812395	GO:0055114,GO:0047134	AT2G16050.1		Cysteine/Histidine-rich C1 domain family protein	cysteine/histidine-rich C1 domain protein
Sapur.017G123700.1	0.687902975	-0.645541852	0.349224888	-1.8485	0.06453019	0.086121843	brown	-0.156245345	0.050686702		AT4G31980.1			DUF247 domain protein
Sapur.013G038500.3	1.151749141	-0.456098706	0.247304336	-1.84428	0.065142188	0.086903547	brown	-0.157492713	0.048847523	GO:0046983	AT4G02590.1	UNE12	basic helix-loop-helix (bHLH) DNA-binding superfamily protein	helix loop helix protein
Sapur.001G160600.1	0.90224634	-0.602809131	0.326944471	-1.84377	0.065217235	0.086996646	brown	-0.176281139	0.02721152		AT1G52080.1	AR791	actin binding protein family	CHUP1, putative
Sapur.016G134500.3	1.317931557	0.427392802	0.234738202	1.820721	0.068649259	0.091316989	brown	0.008657893	0.914298553	GO:0006511	AT2G29070.1		Ubiquitin fusion degradation UFD1 family protein	ubiquitin fusion degradation 1 protein
Sapur.006G156300.4	0.604210867	0.561177703	0.309578296	1.812717	0.069875549	0.092877222	brown	-0.081608864	0.309590942	GO:0055114,GO:0016717,GO:0006629	AT3G12120.2	FAD2	fatty acid desaturase 2	delta-12 fatty acid desaturase
Sapur.010G194000.1	0.280478016	-1.02859557	0.571176897	-1.80084	0.071728829	0.095180819	brown	-0.218709669	0.00592464					NA
Sapur.008G022000.1	0.339991227	-0.936415136	0.521077062	-1.79708	0.072323539	0.095922875	brown	-0.195373361	0.014201041					NA
Sapur.007G104800.1	0.32761551	-0.744180519	0.416095251	-1.78849	0.073697637	0.09764745	brown	-0.110236604	0.169312723	GO:0005525,GO:0003924	AT4G33650.1	ADL2,DRP3A	dynamin-related protein 3A	dynamin-related protein 3A
Sapur.004G148600.3	1.333281247	-0.427607793	0.240017984	-1.78157	0.074820092	0.099035487	brown	-0.094511092	0.239037946	GO:0016579,GO:0004843	AT2G27350.6		OTU-like cysteine protease family protein	rice genome annotation project, cysteine-type peptidase%2C putative
Sapur.018G079400.2	0.369974142	-0.794555987	0.446604666	-1.7791	0.075222772	0.099550083	brown	-0.195500416	0.014136989	GO:0055114,GO:0016491	AT4G30720.1		FAD/NAD(P)-binding oxidoreductase family protein	FAD/NAD(P)-binding oxidoreductase-like protein
Sapur.002G148200.6	0.382412429	0.699327324	0.393260716	1.778279	0.075358022	0.099723571	brown	-0.023056553	0.774397724	GO:0005515	AT3G61590.2	HS,HWS	Galactose oxidase/kelch repeat superfamily protein	F-box/kelch-repeat plant protein
Sapur.004G057400.3	0.712431581	0.515382022	0.290245765	1.775675	0.075786572	0.100242555	brown	0.078548531	0.32814497	GO:0003676	AT3G54230.1	SUA	suppressor of abi3-5	suppressor of abi3-5 protein
Sapur.012G081100.2	0.217987346	0.929685106	0.524549243	1.77235	0.076336396	0.100925406	brown	-0.116080592	0.147683723		AT5G61970.1		signal recognition particle-related / SRP-related	signal recognition particle subunit SRP68
Sapur.003G052800.4	1.109317195	0.428214301	0.243275056	1.760206	0.078372824	0.103477157	brown	0.149706073	0.061292121	GO:0019887,GO:0005956	AT5G47080.1	CKB1	casein kinase II beta chain 1	casein kinase II beta chain, putative
Sapur.014G121200.3	1.41563787	-0.49452521	0.281421847	-1.75724	0.078877223	0.104076649	brown	-0.099935773	0.21301956		AT5G42390.1		Insulinase (Peptidase family M16) family protein	metalloendopeptidase
Sapur.014G007300.2	1.70466116	0.376185502	0.215247905	1.747685	0.080518614	0.106106966	brown	-0.168138069	0.035296703	GO:0005515	AT1G27840.1	ATCSA-1	Transducin/WD40 repeat-like superfamily protein	ATCSA-1
Sapur.005G014600.9	2.336134341	0.300906579	0.172598849	1.743387	0.081266047	0.107062069	brown	-0.081608864	0.309590942	GO:0005515	AT3G06720.2	AIMP ALPHA.AT-IMP.ATKAP	importin alpha isoform 1	importin subunit alpha
Sapur.005G151300.1	1.282985314	-0.400132081	0.22957394	-1.74293	0.081345254	0.107145079	brown	-0.13560721	0.090378053		AT4G24370.1			NA
Sapur.004G117600.1	0.352951017	-0.787550277	0.453808289	-1.73543	0.082665544	0.108788804	brown	-0.230640657	0.003659509	GO:0042545,GO:0030599,GO:0005618	AT2G21610.1	ATPE11,PE11	pectinesterase 11	pectinesterase
Sapur.019G003600.1	1.956320611	-0.371508727	0.214535698	-1.73169	0.083329267	0.109605575	brown	-0.135037849	0.091751335	GO:0005515,GO:0043531	AT1G12220.1	RPS5	Disease resistance protein (CC-NBS-LRR class) family	NA
Sapur.008G154300.1	0.275278313	-1.204462457	0.695566024	-1.73163	0.083339599	0.109614805	brown	-0.263328902	0.000861794		AT1G70000.2		myb-like transcription factor family protein	MYB transcription factor, putative
Sapur.010G003600.2	4.735255928	0.23760565	0.137731822	1.725133	0.084503586	0.111066269	brown	-0.367746997	2.16E-06	GO:0005524,GO:0003676	AT3G06980.1		DEA(D/H)-box RNA helicase family protein	DEAD-box ATP-dependent RNA helicase, putative
Sapur.016G081200.2	0.288950115	-0.950371516	0.555226183	-1.71168	0.086955005	0.1140933	brown	-0.166474937	0.037176536	GO:0005515,GO:0006468,GO:0004672	AT2G37050.1		Leucine-rich repeat protein kinase family protein	NA
Sapur.002G063900.1	0.218178284	-1.066680861	0.623833568	-1.70988	0.087287971	0.114512013	brown	-0.317102646	5.20E-05	GO:0016020,GO:0006810,GO:0005215	AT1G15960.1	ATNRAMP6,NRAMP6	NRAMP metal ion transporter 6	nramp metal transporter
Sapur.016G246800.1	1.27853668	0.471531865	0.28100902	1.677995	0.093347981	0.121978236	brown	0.135805705	0.089903158	GO:0005515	AT3G49180.1	RID3	Transducin/WD40 repeat-like superfamily protein	NA
Sapur.008G164600.4	9.878407885	0.165109021	0.098823535	1.670746	0.094771868	0.123736174	brown	0.078548531	0.32814497	GO:0017053,GO:0007049,GO:0006351	AT3G21430.2	ALY3,ATALY3	DNA binding	always early protein
Sapur.012G095600.2	0.3876967	0.620588481	0.373433631	1.661844	0.096544035	0.125895888	brown	0.013137477	0.870279301		AT2G35110.1	GRL,NAP1,NAPP	transcription activators	NA
Sapur.005G138900.1	0.640788025	-0.66032771	0.40202537	-1.6425	0.100485891	0.130778355	brown	-0.069331179	0.388239737	GO:0008152,GO:0003824	AT1G77590.1	LACS9	long chain acyl-CoA synthetase 9	long-chain-fatty-acid-CoA ligase
Sapur.001G000400.3	0.223941315	-1.067787423	0.656193777	-1.62724	0.103685274	0.134698408	brown	-0.293746602	0.000188507		AT2G32080.1	PUR ALPHA-1	purin-rich alpha 1	transcription factor Pur-alpha 1
Sapur.002G156100.2	1.193104299	-0.45998882	0.282709856	-1.62707	0.10372216	0.134735742	brown	-0.104064136	0.194622713	GO:0016887,GO:0005524,GO:0055085,GO:0042626,GO:0016021,GO:0006810	AT2G47800.1	ATMRP4,EST3,MRP4	multidrug resistance-associated protein 4	multidrug resistance protein ABC transporter family protein
Sapur.002G014500.1	22.09228015	-0.171677335	0.105563788	-1.62629	0.103887975	0.134935237	brown	-0.139593582	0.081216108		AT1G75810.1			transmembrane protein, putative
Sapur.018G078800.1	0.695478202	0.530513275	0.327950471	1.617663	0.105735288	0.137147164	brown	-0.024187789	0.763647655	GO:0006508,GO:0004185	AT4G30810.1	scpl29	serine carboxypeptidase-like 29	serine carboxypeptidase II-2
Sapur.010G030200.1	0.396100375	0.694269988	0.431179844	1.610163	0.107362197	0.139141744	brown	0.117861827	0.141525935	GO:0016787	AT1G10430.1	PP2A-2	protein phosphatase 2A-2	serine/threonine-protein phosphatase PP2A catalytic subunit
Sapur.014G099900.1	1.28341606	-0.446846416	0.278555184	-1.60416	0.108679317	0.140793566	brown	-0.097966592	0.222219623		AT3G62650.2			hypothetical protein
Sapur.016G184900.1	1.372130232	0.427479533	0.267368413	1.598841	0.109855976	0.142234353	brown	0.123843734	0.122270722	GO:0006351,GO:0005634,GO:0003677	AT5G09850.1		Transcription elongation factor (TFIIS) family protein	transcription elongation factor, putative
Sapur.004G139200.1	65.72254476	0.115229646	0.072464825	1.590146	0.111801916	0.144612302	brown	0.128654732	0.108309489		AT3G07350.1		Protein of unknown function (DUF506)	NA
Sapur.012G053600.2	1.861855376	-0.339811172	0.214240319	-1.58612	0.112711755	0.145743553	brown	-0.126761453	0.113646691		AT5G47390.1		myb-like transcription factor family protein	MYB transcription factor, putative
Sapur.008G013200.1	26.16034369	0.156373618	0.09868626	1.584553	0.113067912	0.146169799	brown	0.130433065	0.103477159					transmembrane protein, putative
Sapur.003G032700.1	9.764282039	0.387416187	0.244797038	1.582602	0.113512296	0.146686534	brown	-0.0385402	0.63177752		AT1G71950.1		Proteinase inhibitor, propeptide	NA
Sapur.002G094900.2	0.690151694	0.492728319	0.313277738	1.572816	0.11576137	0.149488191	brown	0.095732284	0.232995514	GO:0005515	AT3G12350.2		F-box family protein	F-box plant protein
Sapur.004G086700.1	5.199848502	0.168848623	0.107414676	1.571933	0.115966203	0.149711784	brown	0.164275151	0.039791991		AT1G67140.3	SWEETIE	HEAT repeat-containing protein	NA
Sapur.15WG008800.1	3.66453881	0.256118836	0.16391988	1.562464	0.118178828	0.152395765	brown	0.121708338	0.128897122		AT5G26760.2			RNA polymerase II subunit B1 CTD phosphatase RPAP2-like protein, putative
Sapur.017G028900.3	9.183121095	0.154366796	0.098981325	1.559555	0.118865134	0.15324493	brown	-0.173066752	0.030191035		AT3G21865.1	PEX22	peroxin 22	peroxin, putative
Sapur.018G118500.2	0.403071729	-0.683127413	0.438082719	-1.55936	0.118911876	0.153293241	brown	-0.081608864	0.309590942	GO:0046872,GO:0030001	AT1G06330.1		Heavy metal transport/detoxification superfamily protein	heavy metal-associated domain protein
Sapur.002G084300.1	338.7196423	-0.097346089	0.063042142	-1.54414	0.12255367	0.157754342	brown	-0.147069904	0.066053174		AT5G49700.1		Predicted AT-hook DNA-binding family protein	AT-hook DNA-binding protein, putative
Sapur.001G023500.1	178.7679305	0.103438091	0.067117338	1.541153	0.123279538	0.158626962	brown	0.09964856	0.214344192		AT1G08315.1		ARM repeat superfamily protein	ubiquitin-protein ligase, putative
Sapur.010G137100.1	0.85562482	-0.48474553	0.316221989	-1.53293	0.125293605	0.161080645	brown	-0.082087533	0.306752215	GO:0046872	AT3G12680.1	HUA1	floral homeotic protein (HUA1)	zinc finger CCCH domain protein
Sapur.008G100200.3	2.260050076	0.29325325	0.192010988	1.527273	0.126693074	0.162791248	brown	0.011429236	0.887026164		AT3G02600.2	ATLPP3,LPP3	lipid phosphate phosphatase 3	lipid phosphate phosphatase
Sapur.017G067000.1	1.431019212	0.404235079	0.265833666	1.520632	0.128352306	0.164820786	brown	0.123853877	0.122239889	GO:0055114,GO:0016491,GO:0009073,GO:0003856	AT3G28760.2			3-dehydroquinate synthase
Sapur.004G074000.2	0.407650785	0.6256697	0.411702663	1.519713	0.128583238	0.165078877	brown	-0.121588358	0.129277477	GO:0046983,GO:0003677,GO:0006355,GO:0005634,GO:0003700	AT3G02310.1	AGL4,SEP2	K-box region and MADS-box transcription factor family protein	MADS-box transcription factor
Sapur.010G022700.2	1.587879794	0.361983579	0.239592821	1.510828	0.13083224	0.167835913	brown	0.11047566	0.168384162		AT3G26400.1	EIF4B1	eukaryotic translation initiation factor 4B1	eukaryotic translation initiation factor 4B
Sapur.006G062300.1	1.335756048	0.343154214	0.227436964	1.508788	0.13135291	0.168445045	brown	-0.037653892	0.639643153	GO:0046872,GO:0000166	AT4G30110.1	ATHMA2,HMA2	heavy metal atpase 2	heavy metal transporting P-type ATPase, putative
Sapur.15WG100600.3	1.326239815	-0.319485291	0.2118878	-1.5078	0.131604675	0.168761361	brown	-0.053704722	0.504118781		AT5G63110.1	ATHDA6.AXE1.HDA6.RPD3B	histone deacetylase 6	histone deacetylase family protein
Sapur.005G128400.1	0.518959138	-0.617911508	0.414066492	-1.4923	0.135620465	0.173614772	brown	-0.123544573	0.123182845	GO:0003824,GO:0006470,GO:0004722	AT1G34750.1		Protein phosphatase 2C family protein	protein phosphatase 2C
Sapur.016G139600.1	176.1207682	-0.085341765	0.057198847	-1.49202	0.135694171	0.173702404	brown	-0.128009143	0.110106896	GO:0005515	AT1G07310.1		Calcium-dependent lipid-binding (CaLB domain) family protein	C2 domain protein
Sapur.001G107900.5	8.623180915	0.171890917	0.115301269	1.490798	0.136014503	0.174078773	brown	0.20665864	0.009408911	GO:0008168	AT4G19120.1	ERD3	S-adenosyl-L-methionine-dependent methyltransferases superfamily protein	S-adenosyl-L-methionine-dependent methyltransferase, putative
Sapur.15WG123800.1	1.874302632	-0.312787806	0.210981592	-1.48254	0.138197732	0.176712323	brown	-0.137425271	0.086102302		AT5G45470.1		Protein of unknown function (DUF594)	NA
Sapur.014G059400.1	0.294454538	-0.791083265	0.534337885	-1.48049	0.138741844	0.177377185	brown	-0.194592682	0.014600199		AT3G61060.1	AtPP2-A13,PP2-A13	phloem protein 2-A13	F-box protein PP2-A13
Sapur.004G069400.1	0.251707353	-0.814254517	0.551886012	-1.4754	0.140104113	0.179046887	brown	-0.189654674	0.01735923	GO:0016758,GO:0008152	AT3G16520.3	UGT88A1	UDP-glucosyl transferase 88A1	UDP-glucosyltransferase, putative
Sapur.012G030200.1	8.623923287	-0.173596178	0.11823598	-1.46822	0.142045055	0.181284899	brown	-0.12765487	0.11110311	GO:0016020	AT3G17910.1	SURF1	Surfeit locus 1 cytochrome c oxidase biogenesis protein	surfeit locus protein
Sapur.009G105500.1	8.385429637	-0.187408099	0.128501744	-1.45841	0.144727846	0.184502342	brown	-0.116923769	0.144740023	GO:0006950	AT2G21620.1	RD2	Adenine nucleotide alpha hydrolases-like superfamily protein	NA
Sapur.004G084200.1	8.786933999	-0.173093643	0.119123209	-1.45306	0.146205993	0.18629337	brown	-0.091146039	0.256251665	GO:0005524,GO:0051082,GO:0006457	AT3G02530.1		TCP-1/cpn60 chaperonin family protein	NA
Sapur.006G021500.3	0.906410394	-0.389264034	0.269743242	-1.44309	0.148994849	0.189563623	brown	-0.110730555	0.167398257	GO:0046556,GO:0046373	AT3G10740.1	ARAF,ARAF1,ASD1,ATASD1	alpha-L-arabinofuranosidase 1	alpha-L-arabinofuranosidase
Sapur.003G055400.1	8.733305103	0.289466446	0.200779184	1.441715	0.149382672	0.190018941	brown	0.102641475	0.200827322		AT4G21700.1		Protein of unknown function (DUF2921)	transmembrane protein, putative
Sapur.019G005300.1	0.297357735	0.725042615	0.503284804	1.440621	0.149691808	0.190375562	brown	0.147843465	0.064625793		AT4G34990.1	AtMYB32,MYB32	myb domain protein 32	NA
Sapur.011G115600.1	2.289327321	-0.391154744	0.271848417	-1.43887	0.150187137	0.190946774	brown	-0.087864767	0.273839866	GO:0055114,GO:0050660,GO:0016491	AT4G20800.1		FAD-binding Berberine family protein	reticuline oxidase, putative
Sapur.010G201300.2	1.120012865	-0.381616635	0.269362567	-1.41674	0.156559053	0.198589941	brown	-0.059888566	0.456226893	GO:0055085,GO:0016021	AT1G55730.2	ATCAX5,CAX5	cation exchanger 5	vacuolar cation/proton exchanger 2
Sapur.005G129300.1	0.349630415	-0.607154074	0.429115148	-1.4149	0.157098467	0.199228324	brown	-0.187292145	0.018832257	GO:0003676	AT5G64200.1	ATSC35,SC35	ortholog of human splicing factor SC35	arginine/serine-rich splicing factor SC30 transcript protein
Sapur.007G070400.1	0.814133754	-0.483227818	0.341675068	-1.41429	0.157276612	0.199408367	brown	-0.128225813	0.109501074		AT5G23340.1		RNI-like superfamily protein	glucose regulated repressor protein, putative
Sapur.002G196600.2	0.495791493	-0.586036441	0.416342501	-1.40758	0.159254716	0.201707597	brown	-0.12775521	0.110820243		AT4G28820.1		HIT-type Zinc finger family protein	HIT zinc finger protein, putative
Sapur.009G043300.1	0.860490571	-0.411668462	0.293385064	-1.40317	0.16056682	0.203229383	brown	-0.145940234	0.068183764		AT5G13760.1		Plasma-membrane choline transporter family protein	NA
Sapur.15WG007000.3	3.564812408	0.186568767	0.133474385	1.397787	0.162177023	0.205102587	brown	0.069770074	0.385235986	GO:0006890	AT5G24350.1			neuroblastoma-amplified sequence protein
Sapur.003G123200.1	7.135159977	-0.223736785	0.160150931	-1.39704	0.162402428	0.205364095	brown	-0.10156036	0.205637037	GO:0005515	AT1G28050.1		B-box type zinc finger protein with CCT domain	B-box type zinc finger protein, putative
Sapur.017G052500.3	13.99295979	0.129668384	0.093053133	1.393488	0.163472401	0.206582844	brown	-0.008024016	0.920551695	GO:0016021,GO:0005783	AT5G14030.3		translocon-associated protein beta (TRAPB) family protein	translocon-associated protein subunit beta
Sapur.014G028600.6	2.826844577	-0.226848114	0.163559392	-1.38695	0.1654581	0.208972437	brown	-0.081608864	0.309590942	GO:0006468,GO:0005524,GO:0004672	AT3G13690.1		Protein kinase protein with adenine nucleotide alpha hydrolases-like domain	receptor tyrosine kinase
Sapur.001G038000.1	0.343391111	-0.558555517	0.403373486	-1.38471	0.166140995	0.209762837	brown	-0.101215484	0.207188635		AT1G80750.1		Ribosomal protein L30/L7 family protein	60S ribosomal protein L7
Sapur.016G244500.3	0.765468716	0.415467954	0.300894316	1.380777	0.167347535	0.211221659	brown	0.100313704	0.211285464	GO:0007165,GO:0006355,GO:0005634,GO:0004871,GO:0003700	AT1G78540.2	ATSHB,SHB,STATLB	SH2 domain protein B	SH2 domain protein A
Sapur.014G111200.1	6.278957663	0.182281872	0.132282231	1.377977	0.16821039	0.212245934	brown	0.116805103	0.145155042	GO:0016021,GO:0008381	AT2G48060.1			FAM38B protein
Sapur.002G173900.1	184.6590735	-0.10602289	0.076991539	-1.37707	0.168489998	0.212574412	brown	-0.103261556	0.198105635		AT5G55160.1	ATSUMO2.SUM2.SUMO 2.S	small ubiquitin-like modifier 2	small ubiquitin-related modifier
Sapur.016G184200.1	0.287404921	-1.022190499	0.749805051	-1.36328	0.17279585	0.217550436	brown	-0.177592999	0.026069792		AT4G39340.1		Protein of unknown function (DUF1278)	prolamin-like protein
Sapur.019G054300.1	0.475273827	-0.696236175	0.511589679	-1.36093	0.173536779	0.218450005	brown	-0.113016928	0.158746087	GO:0016020,GO:0006810,GO:0005215	AT2G40460.1		Major facilitator superfamily protein	peptide transporter
Sapur.016G003900.1	1.84721356	-0.473307126	0.348465142	-1.35826	0.174380413	0.219436814	brown	-0.095877645	0.232283481					NA
Sapur.001G024500.6	0.350244707	0.530741282	0.391484922	1.355713	0.175190433	0.220389045	brown	-0.081608864	0.309590942	GO:0043565,GO:0006355,GO:0003700,GO:0006351	AT3G12250.2	BZIP45,TGA6	TGACG motif-binding factor 6	transcription factor TGA2
Sapur.005G079600.1	5.016989165	0.189276731	0.140034159	1.351647	0.176488313	0.221903611	brown	0.119706836	0.135355697	GO:0016779,GO:0006396,GO:0003723	AT2G17580.1		Polynucleotide adenylyltransferase family protein	poly(A) polymerase, putative
Sapur.011G031400.2	0.339440717	0.56193196	0.416269964	1.349922	0.177041021	0.222539325	brown	0.179485951	0.024494742	GO:0006508,GO:0004185	AT1G11080.2	scpl31	serine carboxypeptidase-like 31	serine carboxypeptidase-like protein
Sapur.012G042300.1	0.400360423	-0.596043309	0.442748842	-1.34623	0.178227234	0.223936768	brown	-0.144804747	0.070381385	GO:0030983,GO:0006298,GO:0005524	AT3G18524.1	ATMSH2,MSH2	MUTS homolog 2	DNA mismatch repair protein Msh2
Sapur.010G101200.2	7.806234422	0.167334993	0.124822372	1.340585	0.180055248	0.226096179	brown	-0.113291485	0.157730071		AT3G25690.1	CHUP1	Hydroxyproline-rich glycoprotein family protein	CHUP1
Sapur.019G009100.1	1.466850929	-0.344998664	0.258681272	-1.33368	0.18230796	0.228751222	brown	-0.083907594	0.296114795	GO:0006886,GO:0005622	AT5G63640.1		ENTH/VHS/GAT family protein	hepatocyte growth factor-regulated tyrosine kinase substrate protein, putative
Sapur.008G048900.4	0.577001655	-0.488580306	0.366553507	-1.3329	0.182563605	0.22904443	brown	-0.094351629	0.239834971		AT2G39855.2			hypothetical protein
Sapur.005G208700.1	0.621960462	-0.524612702	0.393591685	-1.33289	0.182569338	0.22904443	brown	-0.068501075	0.393959372		AT1G21080.1		DNAJ heat shock N-terminal domain-containing protein	DnaJ heat shock amino-terminal domain protein
Sapur.017G045300.1	0.252980684	-0.881408548	0.661694068	-1.33205	0.182844273	0.22932846	brown	-0.167941367	0.035514759		AT3G07550.1		RNI-like superfamily protein	F-box/LRR protein
Sapur.017G003700.2	9.020159575	-0.20466533	0.153666653	-1.33188	0.182900079	0.229389755	brown	-0.098173143	0.221241589	GO:0006412,GO:0005840,GO:0003735,GO:0005622	AT2G44065.1		Ribosomal protein L2 family	ribosomal protein L2
Sapur.002G051300.2	0.5558128	0.493198938	0.371103648	1.329006	0.183846022	0.230462528	brown	0.112402395	0.161037979		AT1G34000.1	OHP2	one-helix protein 2	one helix protein
Sapur.002G154900.2	4.328141328	-0.198831515	0.15026598	-1.3232	0.185769826	0.232724186	brown	-0.366590921	2.33E-06	GO:0005515	AT2G47730.1	ATGSTF5.ATGSTF8.GST6.GS'	glutathione S-transferase phi 8	Phi class glutathione S-transferase
Sapur.014G117100.4	94.51974255	0.062960137	0.047772573	1.317914	0.187532481	0.234798958	brown	0.265193821	0.000789008		AT3G19340.1		Protein of unknown function (DUF3754)	transmembrane protein, putative
Sapur.004G114100.2	0.680602179	-0.418745854	0.318699394	-1.31392	0.188872761	0.236351786	brown	-0.024091298	0.764562879	GO:0016773,GO:0048015,GO:0046854	AT1G49340.1	ATPI4K ALPHA	Phosphatidylinositol 3- and 4-kinase family protein	NA
Sapur.016G170800.1	368.0795434	0.530733864	0.405936649	1.30743	0.191066621	0.238898285	brown	0.212652261	0.007497777	GO:0055114,GO:0020037,GO:0016705,GO:0005506	AT4G31500.1	ATR4.CYP83B1.RED1.RNT1.	cytochrome P450, family 83, subfamily B, polypeptide 1	NA
Sapur.008G011300.2	0.530414985	-0.50166719	0.384914606	-1.30332	0.192465282	0.240501615	brown	-0.123297067	0.123941447	GO:0006887,GO:0000145	AT5G03540.1	ATEXO70A1,EXO70A1	exocyst subunit exo70 family protein A1	exocyst subunit EXO70
Sapur.005G148500.1	0.223750296	-0.747690476	0.575131437	-1.30003	0.193589284	0.241833056	brown	-0.254565745	0.001293651		AT5G41470.1		Nuclear transport factor 2 (NTF2) family protein	NA
Sapur.003G131200.3	0.377107419	-0.573707678	0.442524203	-1.29644	0.194822693	0.24330951	brown	-0.067295305	0.402356848	GO:0016020	AT2G33205.1		Serinc-domain containing serine and sphingolipid biosynthesis protein	TMS membrane protein/tumor differentially protein
Sapur.002G192800.1	0.90601434	-0.34076267	0.263732761	-1.29208	0.196330959	0.245045101	brown	-0.093725699	0.242981416	GO:0006457	AT3G25230.1	ATFKBP62,FKBP62,ROF1	rotamase FKBP 1	peptidyl-prolyl cis-trans isomerase FKBP65
Sapur.008G022300.1	0.285601405	-0.786078903	0.615400353	-1.27735	0.201480279	0.25107414	brown	-0.159902164	0.045451862					transmembrane protein, putative
Sapur.017G127700.1	0.313323495	0.618820701	0.484611219	1.276943	0.20162252	0.251232461	brown	0.071053047	0.376536432	GO:0004866	AT1G17860.1		Kunitz family trypsin and protease inhibitor protein	kunitz-type protease inhibitor
Sapur.005G111500.4	14.0185314	-0.097272993	0.076268137	-1.27541	0.202164863	0.25181338	brown	-0.08231554	0.305406053		AT2G01710.1		Chaperone DnaJ-domain superfamily protein	AT hook motif protein
Sapur.016G041500.1	1.7938974	0.270758573	0.213640242	1.267358	0.205027472	0.255186792	brown	0.092918084	0.247083461		AT2G42130.4		Plastid-lipid associated protein PAP / fibrillin family protein	plastid-lipid-associated protein, putative
Sapur.008G150900.1	1.64392263	-0.551987031	0.44453347	-1.24172	0.214339115	0.265995719	brown	-0.08646527	0.28158432		AT3G07040.1	RPM1,RPS3	NB-ARC domain-containing disease resistance protein	disease resistance protein RPM1, putative
Sapur.003G112700.2	1.748531903	-0.298114509	0.243242683	-1.22558	0.220355001	0.272890616	brown	-0.099424178	0.215383134					hypothetical protein
Sapur.017G056300.8	7.925446699	0.117166842	0.095667531	1.224729	0.220677176	0.273266573	brown	-0.113172583	0.158169473	GO:0006355,GO:0005634	AT1G07705.2		NOT2 / NOT3 / NOT5 family	CCR4-NOT transcription complex subunit, putative
Sapur.012G088100.1	8.723548843	-0.56073901	0.465269821	-1.20519	0.228129568	0.281851226	brown	-0.154775186	0.052927757					NA
Sapur.019G015800.3	0.182695959	-0.861305524	0.716530071	-1.20205	0.229343858	0.283213933	brown	-0.262689281	0.000888144	GO:0016021,GO:0015693,GO:0015095	AT2G21120.1		Protein of unknown function (DUF803)	Non-imprinted in prader-willi/angelman syndrome region protein
Sapur.008G077400.4	1.007529146	-0.36323189	0.302339033	-1.20141	0.229593798	0.283501411	brown	-0.072789752	0.364953224	GO:0005634,GO:0003677	AT1G69170.1		Squamosa promoter-binding protein-like (SBP domain) transcription factor family protein	LIGULELESS 1 protein, putative
Sapur.005G109900.1	0.169735872	-0.842100856	0.703939034	-1.19627	0.231591374	0.285786624	brown	-0.316529842	5.37E-05	GO:0003676	AT4G36960.1		RNA-binding (RRM/RBD/RNP motifs) family protein	heterogeneous nuclear ribonucleoprotein G RNA recognition motif protein
Sapur.004G113700.1	0.229428246	0.64144028	0.537179625	1.194089	0.232443156	0.286752143	brown	0.143044769	0.073901044					hypothetical protein
Sapur.006G216500.2	5.662442182	-0.175754613	0.14749682	-1.19158	0.233425037	0.287877532	brown	-0.08554933	0.286731822	GO:0005634	AT2G32070.1		Polynucleotidyl transferase, ribonuclease H-like superfamily protein	CCR4-associated factor-like protein, putative
Sapur.019G073800.3	0.34434482	-0.510254751	0.432929646	-1.17861	0.238553931	0.29372097	brown	-0.137846113	0.085135994		AT1G33810.1			hypothetical protein
Sapur.003G108400.4	0.297368871	-0.58504501	0.496397309	-1.17858	0.238564601	0.293723174	brown	-0.12476833	0.119484706	GO:0006468,GO:0005524,GO:0004672	AT4G10730.1		Protein kinase superfamily protein	Serine/Threonine kinase domain protein
Sapur.001G022800.1	1.115685801	0.329555983	0.280271003	1.175848	0.239655786	0.294956851	brown	0.100991022	0.208203003	GO:0005975,GO:0004553,GO:0009341,GO:0004565	AT3G54440.1		glycoside hydrolase family 2 protein	beta-galactosidase
Sapur.001G149600.2	8.911847369	-0.144245643	0.123125261	-1.17154	0.241383504	0.29691751	brown	0.14139455	0.07732936		AT3G13845.1			transmembrane protein, putative
Sapur.15WG025600.2	1.422624296	0.26580797	0.232809157	1.141742	0.253561317	0.310775876	brown	-0.268710344	0.000666916		AT1G66410.2	ACAM-4,CAM4	calmodulin 4	calmodulin
Sapur.013G132400.1	87.82900231	-0.082067572	0.071970704	-1.14029	0.254164937	0.311469529	brown	-0.081761993	0.308680954	GO:0015979,GO:0010207	AT2G20890.1	PSB29,THF1	photosystem II reaction center PSB29 protein	thylakoid FORMATION protein
Sapur.002G011000.2	0.376881886	0.464936145	0.410057656	1.133831	0.25686538	0.314522438	brown	0.096860786	0.227507823	GO:0003824,GO:0005515	AT4G35790.2	ATPLDDELTA,PLDDELTA	phospholipase D delta	phospholipase D
Sapur.005G001200.1	0.330688332	-0.484338411	0.427508047	-1.13293	0.257241961	0.31493691	brown	-0.170558587	0.032704738	GO:0016021	AT3G05880.1	RCI2A	Low temperature and salt responsive protein family	stress-induced hydrophobic peptide protein
Sapur.013G055400.6	0.347655779	-0.482644852	0.426545984	-1.13152	0.257836743	0.315606678	brown	-0.16221659	0.04237791		AT2G23840.1		HNH endonuclease	HNH nuclease, putative
Sapur.006G143000.3	4.979157729	-0.143930911	0.12740951	-1.12967	0.258614612	0.316418307	brown	-0.081608864	0.309590942		AT2G23820.2		Metal-dependent phosphohydrolase	metal-dependent phosphohydrolase HD domain protein
Sapur.006G029200.3	0.292518131	-0.569785393	0.506179073	-1.12566	0.26030958	0.318362572	brown	-0.126095853	0.115571017		AT3G56720.3			hypothetical protein
Sapur.013G001300.1	0.15819311	-0.776240851	0.691003653	-1.12335	0.261287711	0.319464335	brown	-0.093638079	0.243424149	GO:0016021	AT3G05880.1	RCI2A	Low temperature and salt responsive protein family	stress-induced hydrophobic peptide protein
Sapur.018G092100.1	0.289530905	-0.547783805	0.489448366	-1.11919	0.263060757	0.321453908	brown	-0.160204806	0.04503962	GO:0031515,GO:0030488,GO:0016429	AT5G14600.1		S-adenosyl-L-methionine-dependent methyltransferases superfamily protein	NA
Sapur.005G201700.1	0.934957655	0.308856222	0.276601959	1.116609	0.264161564	0.322739447	brown	0.081144068	0.312363786		AT1G20640.2		Plant regulator RWP-RK family protein	NA
Sapur.017G076300.1	4.383606509	0.136391055	0.122277351	1.115424	0.264668897	0.323335393	brown	-0.023622843	0.76901081		AT3G02090.1	MPPBETA	Insulinase (Peptidase family M16) protein	processing peptidase beta subunit
Sapur.004G009900.1	1.022520327	-0.347168024	0.314124954	-1.10519	0.269076901	0.328320277	brown	-0.025331267	0.752826926		AT1G11660.1		heat shock protein 70 (Hsp 70) family protein	heat shock 70 kDa protein
Sapur.005G141100.8	1.083525782	0.317270376	0.287309258	1.104282	0.269470929	0.328728294	brown	-0.081608864	0.309590942		AT4G31115.1		Protein of unknown function (DUF1997)	plant/F6E21-30 protein, putative
Sapur.016G288000.1	1.349871637	-0.309983811	0.281302627	-1.10196	0.270479728	0.329885925	brown	-0.054810476	0.4953667		AT1G79160.1			hypothetical protein
Sapur.016G040100.1	0.264261279	-0.729469756	0.663918984	-1.09873	0.271884505	0.331428134	brown	-0.097961372	0.222244379	GO:0016787,GO:0005524,GO:0003677,GO:0016817	AT3G57300.1	ATINO80,INO80	INO80 ortholog	DNA helicase INO80 complex-like protein
Sapur.010G061900.6	0.660442143	-0.341952832	0.311540196	-1.09762	0.272370341	0.331995896	brown	-0.103491805	0.197101845		AT1G05410.1		Protein of unknown function (DUF1423)	CDPK adapter protein, putative
Sapur.009G106900.1	11.21703454	-0.125419117	0.114656369	-1.09387	0.27401217	0.333861798	brown	-0.093303967	0.245117526	GO:0004842	AT4G38600.1	KAK,UPL3	HEAT repeat ;HECT-domain (ubiquitin-transferase)	E3 ubiquitin-protein ligase UPL3
Sapur.15WG062000.1	3.397966903	-0.27035023	0.247833644	-1.09085	0.275337295	0.335303424	brown	-0.078573882	0.327988398		AT4G08910.1			NA
Sapur.008G048900.2	0.236091586	-0.761100421	0.69787244	-1.0906	0.275448464	0.335426455	brown	-0.143868802	0.072235772		AT2G39855.2			hypothetical protein
Sapur.010G205700.2	0.3224983	0.451505151	0.415058349	1.087811	0.276678431	0.336849828	brown	-0.113047939	0.158631083	GO:0016887,GO:0005524	AT3G54540.1	ATGCN4,GCN4	general control non-repressible 4	ABC transporter F family protein
Sapur.007G124700.1	0.212576676	0.605294731	0.557534739	1.085663	0.277628201	0.337906643	brown	0.138820494	0.082932007	GO:0006468,GO:0005524,GO:0004672	AT2G43790.1	ATMAPK6.ATMPK6.MAPK6.I	MAP kinase 6	MAP kinase
Sapur.T062600.1	0.259902264	1.000110708	0.926282829	1.079703	0.280274285	0.340826226	brown	0.177251804	0.02636272		AT1G67020.1			NA
Sapur.009G103200.1	1.926413974	-0.200096888	0.186011205	-1.07572	0.282050299	0.342834678	brown	-0.105254788	0.189537987	GO:0005634	AT2G21150.1	XCT	XAP5 family protein	XAP5 circadian timekeeper
Sapur.018G096100.1	2.813261282	-0.231201647	0.215583546	-1.07245	0.283519889	0.344481717	brown	-0.053584248	0.505077197	GO:0007165	AT1G30270.1	ATCIPK23.CIPK23.LKS1.SnRI	CBL-interacting protein kinase 23	CBL-interacting Serine/Threonine-kinase
Sapur.004G142400.1	0.234588657	-0.711894348	0.664228196	-1.07176	0.283827074	0.344816951	brown	-0.147340028	0.065551843	GO:0003676,GO:0046872,GO:0005634,GO:0003723	AT5G42820.1	ATU2AF35B,U2AF35B	Zinc finger C-x8-C-x5-C-x3-H type family protein	splicing factor U2af small protein, putative
Sapur.018G100900.1	0.306452693	-0.631425902	0.595480332	-1.06036	0.288979037	0.350599489	brown	-0.150527119	0.059868084	GO:0009055	AT2G25060.1	AtENODL14,ENODL14	early nodulin-like protein 14	NA
Sapur.003G053900.3	3.88954279	-0.137466192	0.130820229	-1.0508	0.293349419	0.355406377	brown	-0.081234473	0.31182319	GO:0005524,GO:0030163,GO:0004252,GO:0004176	AT5G47040.1	LON2	lon protease 2	Lon protease-like, peroxisomal protein
Sapur.017G018400.1	2.364028811	-0.234144536	0.224542679	-1.04276	0.297058611	0.359663085	brown	-0.067985047	0.397540269	GO:0016747	AT3G26040.1		HXXXD-type acyl-transferase family protein	benzylalcohol acetyltransferase
Sapur.008G130700.3	1.15247323	-0.244737296	0.23640915	-1.03523	0.300562594	0.363477189	brown	-0.089823893	0.263242802	GO:0016020,GO:0005509,GO:0004571	AT5G43710.1		Glycosyl hydrolase family 47 protein	alpha-mannosidase, putative
Sapur.018G030400.1	0.229230268	-0.877655708	0.848598875	-1.03424	0.301023534	0.363957316	brown	-0.132510764	0.098048119	GO:0005525	AT5G11480.1		P-loop containing nucleoside triphosphate hydrolases superfamily protein	ribosome biogenesis GTP-binding protein YsxC
Sapur.15WG092200.3	2.05102884	0.214466928	0.207428988	1.033929	0.30116917	0.36409347	brown	-0.141768803	0.076540812	GO:0005992,GO:0003824	AT1G23870.1	ATTPS9,TPS9	trehalose-phosphatase/synthase 9	trehalose-6-phosphate synthase
Sapur.005G149900.3	1.013305958	0.35010351	0.33957728	1.030998	0.302541739	0.365605812	brown	0.058337835	0.467993199	GO:0030170,GO:0019752,GO:0016831	AT1G43710.1	emb1075	Pyridoxal phosphate (PLP)-dependent transferases superfamily protein	serine decarboxylase
Sapur.010G037100.1	0.39182878	-0.402280227	0.391855729	-1.0266	0.304607489	0.367860229	brown	-0.127166485	0.112487983	GO:0016021	AT1G09330.1			NA
Sapur.008G032700.2	0.226776082	-0.643301521	0.630040934	-1.02105	0.307232085	0.370840261	brown	-0.158033676	0.048067327		AT2G35795.1		Chaperone DnaJ-domain superfamily protein	import inner membrane translocase subunit TIM14
Sapur.013G086000.1	1.059445321	0.237335018	0.234369628	1.012653	0.311226117	0.375305086	brown	0.15309793	0.055584001		AT3G03140.1		Tudor/PWWP/MBT superfamily protein	PWWP domain protein
Sapur.016G223200.1	0.385307925	0.382618705	0.378836966	1.009982	0.312503676	0.376708335	brown	-0.060401171	0.452373997	GO:0008484,GO:0008152,GO:0016740,GO:0006506,GO:0005789	AT3G01380.1		transferases;sulfuric ester hydrolases;catalytics;transferases	phosphatidylinositolglycan class N protein
Sapur.005G003800.1	0.219030012	-0.599074727	0.594928768	-1.00697	0.313949752	0.378341195	brown	-0.225183661	0.004575293		AT1G56700.2		Peptidase C15, pyroglutamyl peptidase I-like	pyrrolidone-carboxylate peptidase
Sapur.009G005400.2	1.673127292	-0.252030157	0.250380682	-1.00659	0.314132868	0.378534284	brown	-0.050013134	0.533912604	GO:0006355,GO:0003677	AT2G27990.1	BLH8,PNF	BEL1-like homeodomain 8	BEL1 homeotic protein, putative
Sapur.004G089300.1	0.593307065	-0.405247672	0.40283842	-1.00598	0.314424859	0.378872333	brown	-0.095732944	0.232992274		AT4G34419.1			hypothetical protein
Sapur.005G080400.3	29.34749844	-0.070371929	0.071030148	-0.99073	0.321815852	0.386946504	brown	-0.081608864	0.309590942	GO:0006468,GO:0004672	AT4G35600.2	CONNEXIN 32	Protein kinase superfamily protein	Serine/Threonine-kinase Cx32, putative
Sapur.014G058300.1	0.208203718	-0.59341457	0.601426717	-0.98668	0.32380047	0.389205435	brown	-0.203834072	0.010450213	GO:0003677	AT5G42700.1		AP2/B3-like transcriptional factor family protein	B3 DNA-binding domain protein
Sapur.016G132000.3	0.168059527	-0.656774025	0.666997969	-0.98467	0.324785357	0.390289972	brown	-0.098435559	0.220003449		AT3G46540.1		ENTH/VHS family protein	hypothetical protein
Sapur.013G000800.4	0.233347252	-0.641437329	0.651742661	-0.98419	0.325023067	0.390533057	brown	-0.20764759	0.009066616	GO:0016787	AT3G05910.2		Pectinacetylesterase family protein	pectin acetylesterase, putative
Sapur.010G155200.6	2.219578535	-0.186305869	0.190225041	-0.9794	0.327383769	0.393041155	brown	-0.08863982	0.269613532		AT2G39805.2		Integral membrane Yip1 family protein	integral membrane Yip1 family protein
Sapur.005G184200.10	13.33390504	-0.087997908	0.089884121	-0.97902	0.327572537	0.393253506	brown	-0.081608864	0.309590942	GO:0004518	AT1G75380.1	ATBBD1,BBD1	bifunctional nuclease in basal defense response 1	wound responsive protein, putative
Sapur.011G036900.1	1.655037114	-0.225188198	0.230433843	-0.97724	0.328452435	0.394252589	brown	-0.09385095	0.242349511	GO:0006468,GO:0005524,GO:0004672,GO:0030246	AT4G04960.1		Concanavalin A-like lectin protein kinase family protein	lectin-receptor-like kinase
Sapur.007G102600.1	63.81928498	0.054360191	0.055692562	0.976076	0.329026642	0.394913164	brown	0.060685555	0.450244359		AT5G64360.3		Chaperone DnaJ-domain superfamily protein	DnaJ heat shock amino-terminal domain protein, putative
Sapur.004G013900.1	0.530136091	0.325716358	0.336530741	0.967865	0.333111747	0.399526325	brown	-0.053682595	0.504294737	GO:0005515,GO:0008081,GO:0006281,GO:0005634	AT5G15170.1	TDP1	tyrosyl-DNA phosphodiesterase-related	tyrosyl-DNA phosphodiesterase
Sapur.15WG091000.2	0.168219581	-0.850835928	0.886536626	-0.95973	0.337191046	0.403935552	brown	-0.249471072	0.001627927		AT5G50090.1			DUF4228 domain protein
Sapur.004G031200.4	58.33509292	-0.074204932	0.077454983	-0.95804	0.338042848	0.404895741	brown	-0.105591742	0.188116767		AT1G28330.4	DRM1,DYL1	dormancy-associated protein-like 1	auxin-repressed/dormancy-associated protein, putative
Sapur.009G061700.1	0.195424858	-0.548947231	0.573527037	-0.95714	0.338495221	0.405351061	brown	-0.208791548	0.008684443	GO:0055114,GO:0016491,GO:0008152	AT2G14170.1	ALDH6B2	aldehyde dehydrogenase 6B2	methylmalonate-semialdehyde dehydrogenase (acylating)
Sapur.008G151700.2	0.206733461	-0.564126806	0.589729292	-0.95659	0.33877624	0.405638071	brown	-0.202584248	0.010942498	GO:0008168,GO:0008152	AT1G23360.2	MENG	S-adenosyl-L-methionine-dependent methyltransferases superfamily protein	ubiquinone/menaquinone biosynthesis methyltransferase
Sapur.004G047700.1	0.208953219	-0.579954642	0.606946294	-0.95553	0.339310366	0.406194841	brown	-0.243515811	0.002117328		AT3G54710.1	ATCDT1B,CDT1,CDT1B	homolog of yeast CDT1 B homolog of yeast CDT1 B	DNA replication factor CDT1-like protein
Sapur.018G083200.1	2.598040252	-0.157904319	0.165513904	-0.95402	0.340071242	0.406943676	brown	-0.049293967	0.539817625		AT5G58003.1	CPL4	C-terminal domain phosphatase-like 4	RNA polymerase II carboxy-terminal domain phosphatase-like protein
Sapur.010G169900.1	0.195073933	-0.906933392	0.958703977	-0.946	0.34414889	0.411272596	brown	-0.12322531	0.124162053		AT2G39340.1		SAC3/GANP/Nin1/mts3/eIF-3 p25 family	leukocyte receptor cluster (Lrc) protein, putative
Sapur.009G013800.1	2.777277984	0.149088934	0.160529405	0.928733	0.353027544	0.420985506	brown	0.067731706	0.399305373	GO:0016853,GO:0005975	AT5G57330.1		Galactose mutarotase-like superfamily protein	NA
Sapur.15WG045200.1	0.143826444	-0.808918596	0.872960957	-0.92664	0.354114637	0.422084024	brown	-0.295008998	0.000176314		AT1G67060.1			peptidase M50B-like protein
Sapur.001G160200.1	0.225634162	-1.29078371	1.394004931	-0.92595	0.354470159	0.422477335	brown	-0.200123599	0.011971668		AT5G35405.1		Protein of unknown function (DUF784)	NA
Sapur.010G206500.1	0.415306203	-0.388575994	0.419711595	-0.92582	0.354541259	0.422533163	brown	-0.03951502	0.623173838		AT5G08400.2		Protein of unknown function (DUF3531)	plant/F8L15-130 protein, putative
Sapur.016G224600.1	1.085248364	-0.278403878	0.300894529	-0.92525	0.35483378	0.422834532	brown	-0.057756014	0.472450375		AT2G34410.2		O-acetyltransferase family protein	O-acetyltransferase-like protein
Sapur.013G001100.4	0.305573863	-0.406253256	0.439257985	-0.92486	0.355037414	0.423016226	brown	-0.121212945	0.130473167	GO:0046983	AT1G09530.2	PAP3,PIF3,POC1	phytochrome interacting factor 3	phytochrome-interacting factor 3.1, putative
Sapur.007G024800.1	0.207644101	-0.704761736	0.763126425	-0.92352	0.355736815	0.42380374	brown	-0.110546665	0.168109092	GO:0017025,GO:0008270,GO:0006355,GO:0006352	AT4G36650.1	ATPBRP,PBRP	plant-specific TFIIB-related protein	transcription factor IIB
Sapur.009G068500.3	7.149042267	0.095721157	0.105440386	0.907823	0.363971999	0.432710636	brown	-0.049291835	0.539835179	GO:0005525	AT3G49870.1	ARLA1C,ATARLA1C	ADP-ribosylation factor-like A1C	ADP-ribosylation factor-like protein 8a
Sapur.003G166200.1	0.29095326	-0.418371125	0.461325928	-0.90689	0.364465825	0.43314203	brown	-0.145614118	0.068809133	GO:0055114,GO:0051537,GO:0016491,GO:0010277	AT4G25650.1	ACD1-LIKE,PTC52,TIC55-IV	ACD1-like	pheophorbide A oxygenase
Sapur.003G116200.2	0.233398014	-0.440535072	0.488613551	-0.9016	0.367268201	0.436143351	brown	0.017387466	0.828878863	GO:0006367,GO:0005634	AT4G20280.1	TAF11	TBP-associated factor 11	transcription initiation factor TFIID subunit 11
Sapur.017G101600.1	0.810350129	0.254860431	0.282910414	0.900852	0.367666981	0.436585564	brown	0.054664067	0.496520899		AT5G38060.1			hypothetical protein
Sapur.006G082600.1	0.685816457	-0.278120728	0.310074584	-0.89695	0.369746751	0.438818862	brown	-0.090085655	0.261848428	GO:0005515,GO:0042393,GO:0006355	AT5G05610.1	AL1	alfin-like 1	PHD finger protein

Sapur.001G189900.1	0.175899529	0.781291884	0.87450539	0.89341	0.371637635	0.440809896	brown	0.132408515	0.098309917		AT1G67020.1			NA
Sapur.012G073500.3	0.949363883	0.258082804	0.289076873	0.892783	0.371973604	0.441176755	brown	-0.073974136	0.357181665		AT3G48550.1			nucleic acid-binding protein, putative
Sapur.001G101800.2	0.274610212	-0.490982778	0.550611701	-0.8917	0.37255149	0.441735427	brown	-0.133343828	0.095935651	GO:0005515,GO:0006468,GO:0004672	AT1G16270.2		Protein kinase superfamily protein with octicosapeptide/Phox/Bem1p domain	Serine/Threonine kinase, putative
Sapur.005G105600.1	0.322496343	0.385510106	0.434465095	0.887321	0.374905973	0.444320069	brown	-0.046391479	0.56397375	GO:0006468,GO:0005524,GO:0004672	AT5G66880.1	SNRK2-3,SNRK2.3,SRK2I	sucrose nonfermenting 1(SNF1)-related protein kinase 2.3	Serine/Threonine kinase, putative
Sapur.010G116900.1	0.423957142	-0.372207166	0.421382083	-0.8833	0.377073727	0.446681104	brown	-0.047441459	0.555175955		AT3G17210.1	ATHS1,HS1	heat stable protein 1	stress responsive A/B barrel domain protein
Sapur.017G031300.1	0.198398369	-0.522831906	0.592731494	-0.88207	0.377737826	0.447406937	brown	-0.118402297	0.139696687	GO:0005525,GO:0003924	AT4G33650.1	ADL2,DRP3A	dynamin-related protein 3A	dynamin-related protein 3A
Sapur.010G060700.4	0.35971506	0.408309579	0.462900852	0.882067	0.377740561	0.447406937	brown	0.061471694	0.444386634	GO:0006468,GO:0005524,GO:0004672	AT3G04910.1	ATWNK1,WNK1,ZIK4	with no lysine (K) kinase 1	Serine/Threonine-kinase WNK1, putative
Sapur.T169700.1	0.149829408	-0.696084227	0.79088409	-0.88013	0.378786551	0.448485229	brown	-0.286873808	0.000269832		AT4G00850.1	GIF3	GRF1-interacting factor 3	SSXT protein (amino-terminal region) protein
Sapur.007G034200.1	10.30227827	-0.11227244	0.127707632	-0.87914	0.379327273	0.449061143	brown	-0.051713457	0.520081118		AT2G23610.1	ATMES3,MES3	methyl esterase 3	polyneuridine-aldehyde esterase, putative
Sapur.008G085200.3	35.95038507	0.085620223	0.097724212	0.876141	0.380953197	0.450873002	brown	-0.079467263	0.322501394		AT3G06670.1		binding	serine/threonine protein phosphatase 4 regulatory subunit
Sapur.009G063000.2	0.999082706	0.234686747	0.269509769	0.870791	0.383868147	0.453981803	brown	-0.081608864	0.309590942		AT5G64460.3		Phosphoglycerate mutase family protein	phosphoglycerate mutase-like protein
Sapur.016G140400.4	0.978715134	0.248642335	0.286122993	0.869005	0.384844352	0.454973624	brown	-0.004345567	0.956923261		AT2G29670.1		Tetratricopeptide repeat (TPR)-like superfamily protein	plant/F22G5-32 protein, putative
Sapur.003G133200.1	1.317792271	0.212970491	0.245707116	0.866766	0.38607042	0.456129637	brown	0.082618665	0.30362241	GO:0016747,GO:0008152	AT2G33150.1	KAT2,PED1,PKT3	peroxisomal 3-ketoacyl-CoA thiolase 3	3-ketoacyl-CoA thiolase
Sapur.002G063200.1	1035.743336	0.070782245	0.082155894	0.86156	0.388929604	0.459097674	brown	0.068731239	0.392368444		AT1G77380.1	AAP3,ATAAP3	amino acid permease 3	amino acid permease
Sapur.T115500.1	0.760363517	-0.228063374	0.264762029	-0.86139	0.38902326	0.459191838	brown	-0.107630649	0.179682797	GO:0005515,GO:0043531	AT4G27220.1		NB-ARC domain-containing disease resistance protein	NA
Sapur.002G184300.1	0.61639871	-0.283399685	0.329362721	-0.86045	0.389541817	0.459721893	brown	-0.081608864	0.309590942	GO:0016758,GO:0008152,GO:0030259,GO:0005975	AT3G07020.2		UDP-Glycosyltransferase superfamily protein	sterol 3-beta-glucosyltransferase
Sapur.003G019600.1	0.182580381	-0.631124645	0.737542523	-0.85571	0.392156607	0.46249421	brown	-0.219053118	0.005844993		AT1G76750.1		Protein of unknown function (DUF1278)	NA
Sapur.010G072400.3	0.293792859	0.388467545	0.471499064	0.823899	0.409997024	0.48152557	brown	0.099346888	0.215741836		AT1G68130.1	AtIDD14,IDD14	indeterminate(ID)-domain 14	NA
Sapur.007G016100.1	0.736551434	0.278244106	0.338653273	0.821619	0.411293533	0.482859659	brown	0.040088681	0.618134353		AT3G51000.1		alpha/beta-Hydrolases superfamily protein	NA
Sapur.T115300.1	0.366222202	0.391957995	0.479917871	0.816719	0.414089088	0.485658954	brown	0.07446322	0.354002788					NA
Sapur.011G114500.4	1.890441061	0.154855932	0.189886555	0.815518	0.414775781	0.486343609	brown	-0.060859963	0.448941071		AT1G32540.1	LOL1	lsd one like 1	LSD1 zinc finger protein
Sapur.004G155000.1	0.173019589	0.509823534	0.627149544	0.812922	0.416262897	0.487862476	brown	0.106527081	0.184212494		AT4G38260.1		Protein of unknown function (DUF833)	ser/thr-rich protein T10 in DGCR region protein
Sapur.006G140100.2	0.278110046	-0.522150722	0.647240756	-0.80673	0.419820108	0.491717907	brown	-0.059193263	0.461482081	GO:0008152,GO:0003824,GO:0016114,GO:0008661	AT4G15560.1	CLA,CLA1,DEF,DXPS2,DXS	Deoxyxylulose-5-phosphate synthase	1-deoxy-D-xylulose-5-phosphate synthase
Sapur.004G089400.1	1.569902292	-0.192586422	0.241457152	-0.7976	0.425102176	0.497376162	brown	-0.053969909	0.502012482	GO:0006914	AT3G19190.1	ATATG2,ATG2	autophagy 2	autophagy-related protein
Sapur.T047500.4	0.184397397	-0.565333114	0.710111113	-0.79612	0.425962737	0.498277271	brown	-0.150894078	0.05924047		AT3G17668.1	ENA	DnaJ/Hsp40 cysteine-rich domain superfamily protein	hypothetical protein
Sapur.011G070800.5	8.307513296	0.079739501	0.100531429	0.79318	0.427673062	0.500101077	brown	-0.081608864	0.309590942		AT1G78420.2		RING/U-box superfamily protein	RING E3 ubiquitin ligase
Sapur.001G096700.1	0.181063631	0.463882414	0.585085481	0.792846	0.42786782	0.500222704	brown	0.107163393	0.181590581	GO:0055114,GO:0048038,GO:0009308,GO:0008131,GO:0005507	AT1G62810.1		Copper amine oxidase family protein	amine oxidase [copper containing] protein, putative
Sapur.005G027800.2	1.172134647	-0.211353901	0.266966186	-0.79169	0.428542675	0.500923147	brown	-0.068758266	0.392181882	GO:0016020	AT5G27490.1		Integral membrane Yip1 family protein	integral membrane Yip1 family protein
Sapur.019G006300.1	4.247952403	0.138069354	0.175211256	0.788016	0.430687078	0.503091913	brown	0.053631034	0.504704881		AT5G23630.1	MIA,PDR2	phosphate deficiency response 2	NA
Sapur.006G158300.1	0.523834757	-0.376289439	0.480193198	-0.78362	0.433262573	0.50583241	brown	-0.036646555	0.648631772	GO:0007275,GO:0006511,GO:0005634	AT3G58040.1	SINAT2	seven in absentia of Arabidopsis 2	E3 ubiquitin-protein ligase SINAT2
Sapur.012G056800.3	0.467990797	-0.303943883	0.392189138	-0.77499	0.438343725	0.511259314	brown	-0.078563653	0.32805157	GO:0055085,GO:0016020,GO:0006810,GO:0005215	AT5G62890.1		Xanthine/uracil permease family protein	nucleobase ascorbate transporter
Sapur.013G127300.1	1.052566754	0.215545447	0.278580878	0.773727	0.439092462	0.512078423	brown	0.058600689	0.465987129	GO:0051536,GO:0003824,GO:0051539,GO:0016992,GO:0009107	AT5G08415.1		Radical SAM superfamily protein	lipoyl synthase
Sapur.017G122100.2	0.178160348	-0.493270063	0.65343034	-0.75489	0.450313119	0.523852699	brown	-0.167120941	0.036436568	GO:0046872,GO:0030001	AT1G01490.2		Heavy metal transport/detoxification superfamily protein	metal ion-binding protein, putative
Sapur.004G040900.1	0.775593665	-0.234456193	0.310964818	-0.75396	0.450871003	0.524464796	brown	-0.085754198	0.285575053	GO:0006468,GO:0004672	AT4G18710.1	ATSK21,BIN2,DWF12,SK21,U	Protein kinase superfamily protein	shaggy-related kinase
Sapur.013G060900.2	0.214786537	-0.409522523	0.54326534	-0.75382	0.450959254	0.524549003	brown	-0.16579442	0.037969699		AT5G16450.2		Ribonuclease E inhibitor RraA/Dimethylmenaquinone methyltransferase	NA
Sapur.019G112900.1	0.193288633	-0.611673506	0.813446828	-0.75195	0.452079528	0.525778124	brown	-0.105684778	0.187725733		AT5G41700.2	ATUBC8,UBC8	ubiquitin conjugating enzyme 8	ubiquitin-conjugating enzyme E2
Sapur.006G106500.1	0.187866326	-0.600928171	0.809316729	-0.74251	0.457776595	0.531600122	brown	-0.122557863	0.12622859	GO:0005515	AT3G09740.1	ATSYP71,SYP71	syntaxin of plants 71	syntaxin-71 protein
Sapur.001G158900.1	0.195661164	-1.100276559	1.482325065	-0.74226	0.457927388	0.531737894	brown	-0.186029152	0.019663253		AT5G35405.1		Protein of unknown function (DUF784)	NA
Sapur.016G272700.2	0.208608118	-0.682698328	0.922300552	-0.74021	0.459171109	0.533069791	brown	-0.078512236	0.328369222	GO:0005515	AT5G54520.1		Transducin/WD40 repeat-like superfamily protein	WD domain, G-beta repeat protein
Sapur.005G000700.3	0.174257124	-0.487996537	0.660793778	-0.7385	0.460210362	0.534088829	brown	-0.055884773	0.486941189	GO:0016787	AT3G05910.2		Pectinacetylesterase family protein	pectin acetylesterase, putative
Sapur.016G110100.1	0.477574412	-0.330839525	0.450556108	-0.73429	0.462771091	0.536740464	brown	-0.072902936	0.364206056					NA
Sapur.014G117900.2	0.989747954	-0.199607468	0.271999388	-0.73385	0.463038558	0.536983785	brown	-0.252764109	0.001403928	GO:0005515	AT5G46860.1	ATSYP22,ATVAM3,SGR3,SYP	Syntaxin/t-SNARE family protein	syntaxin
Sapur.016G061700.3	0.778373234	-0.212060951	0.289656236	-0.73211	0.464099907	0.538130708	brown	-0.081608864	0.309590942	GO:0008270,GO:0005515	AT5G22000.1	RHF2A	RING-H2 group F2A	RING-H2 zinc finger protein
Sapur.002G063000.1	0.258641134	0.385015255	0.526824879	0.730822	0.464887833	0.538893179	brown	0.073517924	0.360162884		AT1G21660.1		Chaperone DnaJ-domain superfamily protein	heat shock protein-binding protein
Sapur.004G110600.1	0.175117973	-0.814318247	1.119340007	-0.7275	0.466920595	0.540965137	brown	-0.10787221	0.178702317	GO:0009512	AT2G26500.1		cytochrome b6f complex subunit (petM), putative	plastoquinol-plastocyanin reductase, putative
Sapur.012G059500.2	0.438266452	0.294084958	0.410423255	0.716541	0.473657557	0.54797512	brown	-0.123960599	0.12191583	GO:0003723,GO:0000178	AT4G32175.1		PNAS-3 related	exosome complex exonuclease RRP40
Sapur.016G031200.1	0.116668519	-0.617959961	0.864751899	-0.71461	0.474850345	0.54909448	brown	-0.325309646	3.22E-05	GO:0055085,GO:0016021	AT1G73590.1	ATPIN1,PIN1	Auxin efflux carrier family protein	auxin efflux facilitator PIN1
Sapur.005G094900.2	0.183745247	-0.388602631	0.552942121	-0.70279	0.482186136	0.556740244	brown	-0.188086679	0.018325221		AT4G36280.1		Histidine kinase-, DNA gyrase B-, and HSP90-like ATPase family protein	zinc finger protein, putative
Sapur.016G160900.1	0.143523071	-0.615760138	0.887972204	-0.69345	0.488030151	0.562584407	brown	-0.169310772	0.034020034		AT5G12330.4	LRP1	Lateral root primordium (LRP) protein-related	transcription factor, putative
Sapur.001G045500.2	0.240759262	-0.407837688	0.591199687	-0.68985	0.490290029	0.564857446	brown	-0.092826113	0.247553627	GO:0005634,GO:0003677	AT5G43270.3	SPL2	squamosa promoter binding protein-like 2	squamosa promoter-binding-like protein
Sapur.003G055700.2	1.522696518	-0.18216766	0.265140079	-0.68706	0.49204369	0.566638336	brown	-0.039815513	0.620531869		AT1G32410.2		Vacuolar protein sorting 55 (VPS55) family protein	vacuolar protein sorting-associated-like protein
Sapur.018G006400.1	1.650871152	0.154445096	0.226103817	0.683072	0.4945616	0.569220851	brown	-0.040997504	0.610186911	GO:0030126,GO:0016192,GO:0006886,GO:0005198,GO:0030117,GO:0005737	AT4G31480.1		Coatomer, beta subunit	coatomer subunit beta
Sapur.005G117800.4	6.277810762	-0.076036718	0.11247686	-0.67602	0.499027324	0.573781669	brown	0.008255794	0.918264641		AT2G34320.1		Polynucleotidyl transferase, ribonuclease H-like superfamily protein	ribonuclease HI
Sapur.019G017700.1	0.112163566	0.556654822	0.826686576	0.673357	0.500720483	0.575548386	brown	0.210020834	0.00828972					NA
Sapur.003G014500.1	2.804433728	-0.13274874	0.197537993	-0.67202	0.501573355	0.576408518	brown	-0.021954114	0.784915531		AT1G15780.1			transcription cofactor, putative
Sapur.010G154500.4	1.762640713	0.141015209	0.212652239	0.663126	0.50724992	0.582223976	brown	0.078548531	0.32814497	GO:0008270	AT2G39830.1	DAR2	DA1-related protein 2	zinc ion-binding protein, putative
Sapur.006G095900.1	0.181568791	0.547810968	0.830451434	0.659654	0.509475616	0.584575768	brown	0.074303129	0.355041366	GO:0055114,GO:0020037,GO:0016705,GO:0005506	AT3G52970.1	CYP76G1	cytochrome P450, family 76, subfamily G, polypeptide 1	cytochrome P450 family protein
Sapur.002G103800.1	0.135449911	-0.683127926	1.03586928	-0.65947	0.509592014	0.58468904	brown	-0.184075508	0.021011108		AT4G33380.1			plant/F17M5-140 protein, putative
Sapur.006G046300.1	0.324520234	-0.270548592	0.410345585	-0.65932	0.509691021	0.584762066	brown	-0.103225995	0.198260993	GO:0005515	AT3G57880.1		Calcium-dependent lipid-binding (CaLB domain) plant phosphoribosyltransferase family protein	anthranilate phosphoribosyltransferase-like protein
Sapur.018G036300.4	4.125820892	0.089910644	0.13682691	0.657112	0.511108718	0.586080678	brown	-0.08727045	0.277110875	GO:0006508,GO:0004176	AT2G25740.1		ATP-dependent protease La (LON) domain protein	ATP-dependent protease LA (LON) domain protein
Sapur.017G123700.2	0.177164426	-0.451425673	0.688459024	-0.6557	0.51201428	0.58693887	brown	-0.08690621	0.279128556		AT4G31980.1			DUF247 domain protein
Sapur.005G075800.1	0.195516179	-0.49775193	0.762139469	-0.6531	0.513692961	0.588699665	brown	-0.123573093	0.123095662	GO:0030170,GO:0008483	AT1G80600.1	WIN1	HOPW1-1-interacting 1	acetylornithine aminotransferase
Sapur.018G012000.2	6.028017996	-0.235485886	0.361091794	-0.65215	0.514304638	0.589319277	brown	-0.080876687	0.313966224	GO:0005515	AT2G02230.1	AtPP2-B1,PP2-B1	phloem protein 2-B1	NA
Sapur.004G110200.4	0.476413039	-0.254573639	0.390736157	-0.65152	0.51470886	0.589700736	brown	-0.081608864	0.309590942	GO:0003677	AT1G49480.1	RTV1	related to vernalization1 1	B3 domain transcription factor VRN1
Sapur.005G032600.4	1.874356943	0.151118307	0.234463666	0.644528	0.519233358	0.594554912	brown	-0.113520383	0.156886751	GO:0055114,GO:0004764,GO:0003855	AT3G06350.1	EMB3004,MEE32	dehydroquinate dehydratase, putative / shikimate dehydrogenase, putative	dehydroquinate dehydratase/shikimate dehydrogenase
Sapur.15WG007600.3	0.190033901	-0.551824509	0.85707911	-0.64384	0.519677154	0.59498069	brown	-0.081348192	0.311144047		AT3G26100.2		Regulator of chromosome condensation (RCC1) family protein	RCC1 and BTB domain protein, putative
Sapur.016G177400.1	0.228455863	-0.500339899	0.782280928	-0.63959	0.522438487	0.597728329	brown	-0.073703899	0.358945723	GO:0008270,GO:0005515	AT1G49850.1		RING/U-box superfamily protein	RING-H2 finger protein, putative
Sapur.014G120600.1	0.156527714	-0.37447784	0.585890295	-0.63916	0.522718629	0.597966102	brown	-0.132095106	0.09911581		AT4G29400.1		Protein of unknown function (DUF3531)	oxidoreductase/transition metal ion-binding protein, putative
Sapur.019G029200.1	1.203324214	-0.155895126	0.244953451	-0.63643	0.524497787	0.599628061	brown	-0.056338823	0.483403348		AT3G04450.2		Homeodomain-like superfamily protein	transcription factor, putative
Sapur.007G067500.2	0.383075531	-0.246919345	0.389436931	-0.63404	0.526053456	0.601115672	brown	-0.094142785	0.240881618	GO:0005681,GO:0000398	AT5G08290.1	YLS8	mRNA splicing factor, thioredoxin-like U5 snRNP	thioredoxin-like protein 4A
Sapur.014G104800.6	2.247844393	-0.140198058	0.222013105	-0.63149	0.527723124	0.602794495	brown	-0.075807744	0.345355424	GO:0030975,GO:0009229,GO:0005524,GO:0004788	AT1G02880.1	TPK1	thiamin pyrophosphokinase1	thiamine pyrophosphokinase
Sapur.006G026100.1	0.774139851	-0.200552486	0.321340409	-0.62411	0.532553869	0.60770379	brown	-0.115782905	0.148732318	GO:0006508,GO:0004185	AT1G28110.1	SCPL45	serine carboxypeptidase-like 45	serine carboxypeptidase
Sapur.016G144600.1	0.522132777	0.243233688	0.393050814	0.618835	0.536024915	0.611390421	brown	0.011641541	0.884941943	GO:0006468,GO:0004672	AT5G12480.1	CPK7	calmodulin-domain protein kinase 7	calcium-dependent kinase
Sapur.008G143300.2	0.418284691	0.232703258	0.376687624	0.617762	0.536732309	0.612049524	brown	-0.151017222	0.059031071	GO:0051603,GO:0005839,GO:0004298	AT3G26340.1		N-terminal nucleophile aminohydrolases (Ntn hydrolases) superfamily protein	proteasome subunit beta type-5 protein
Sapur.005G161300.3	0.192909026	0.444327253	0.719977668	0.61714	0.537142171	0.612474667	brown	0.084502307	0.292692607		AT1G53580.1	ETHE1,GLX2-3,GLY3	glyoxalase II 3	hydroxyacylglutathione hydrolase
Sapur.001G166200.2	0.326964009	0.262004943	0.424617041	0.617038	0.537209516	0.612530339	brown	0.000309292	0.996932577	GO:0006412,GO:0005840,GO:0005622,GO:0003735	AT1G79850.1	CS17,PRPS17,RPS17	ribosomal protein S17	30S ribosomal protein S17
Sapur.006G086600.1	0.186554887	-0.589183059	0.95742511	-0.61538	0.538301869	0.613691221	brown	-0.090253814	0.26095534	GO:0030036,GO:0005856	AT5G01730.1	ATSCAR4,SCAR4,WAVE3	SCAR family protein 4	SCAR2
Sapur.010G170400.2	0.222102526	0.341953814	0.556130417	0.614881	0.538633564	0.61402704	brown	-0.132856757	0.097166331					HMG (high mobility group) box protein
Sapur.002G051000.3	1.217624802	0.148843313	0.242267505	0.614376	0.538966969	0.614343587	brown	0.078548531	0.32814497		AT4G09810.1		Nucleotide-sugar transporter family protein	triose-phosphate transporter family protein
Sapur.002G112700.1	1.936511003	-0.126362568	0.207850286	-0.60795	0.543220677	0.618765693	brown	-0.041204179	0.608385875		AT4G17520.1		Hyaluronan / mRNA binding family	nuclear RNA-binding protein
Sapur.009G024400.1	0.105651651	-0.547948614	0.908883289	-0.60288	0.546587748	0.622151054	brown	-0.316115004	5.50E-05		AT1G07530.1	ATGRAS2,GRAS2,SCL14	SCARECROW-like 14	GRAS family transcription factor
Sapur.017G100200.4	0.132016741	-0.411835058	0.686713052	-0.59972	0.548693335	0.624078887	brown	-0.081608864	0.309590942		AT2G24960.1			Myb/SANT-like DNA-binding domain protein
Sapur.002G128800.2	0.116715449	-0.609590182	1.021571641	-0.59672	0.550695676	0.625959602	brown	-0.238598614	0.00261826		AT2G45820.1		Remorin family protein	remorin, amino-terminal region protein
Sapur.013G003900.1	0.713399761	-0.175765175	0.296701792	-0.5924	0.553584948	0.628838734	brown	-0.049676763	0.536670493		AT1G09430.1	ACLA-3	ATP-citrate lyase A-3	ATP-citrate synthase
Sapur.001G173500.1	0.181504567	-0.452435022	0.773052029	-0.58526	0.558374132	0.633865318	brown	-0.133162772	0.096391675		AT3G26070.1		Plastid-lipid associated protein PAP / fibrillin family protein	PAP fibrillin
Sapur.016G062200.7	0.154439781	-0.445781097	0.763569602	-0.58381	0.559346804	0.634773408	brown	-0.181606257	0.022828152		AT5G06440.2			START-2 domain protein, putative
Sapur.011G031100.5	1.02535901	0.144066143	0.249920492	0.576448	0.564312474	0.639706292	brown	-0.063887384	0.426658752	GO:0005515	AT1G61150.1		LisH and RanBPM domains containing protein	Ran-binding protein in the microtubule-organising centre protein
Sapur.010G206600.4	0.174688751	-0.326020511	0.573210345	-0.56876	0.569517372	0.644899259	brown	-0.141143795	0.077861357	GO:0005515,GO:0051013,GO:0008352,GO:0008017	AT5G23430.1		Transducin/WD40 repeat-like superfamily protein	katanin p80 subunit-like protein
Sapur.003G074400.3	11.60037284	-0.055757086	0.098119154	-0.56826	0.569859162	0.645220017	brown	-0.035938308	0.654982164		AT2G45010.1		PLAC8 family protein	cell number regulator 6
Sapur.014G127100.2	0.542519483	0.2217621	0.390348642	0.568113	0.569958308	0.645310183	brown	0.009389431	0.907088552		AT3G21610.1		Acid phosphatase/vanadium-dependent haloperoxidase-related protein	NA
Sapur.014G114800.6	0.177189326	-0.402910484	0.709269626	-0.56806	0.569991566	0.645325747	brown	-0.067118091	0.403599932		AT1G06460.1	ACD31.2,ACD32.1	alpha-crystallin domain 32.1	small heat-shock protein, putative
Sapur.018G001800.1	0.090659331	0.544857175	0.959698423	0.567738	0.570212947	0.64555429	brown	-0.045749625	0.569384396		AT4G15060.1			NA
Sapur.016G119700.3	0.138256655	-0.742866326	1.31858192	-0.56338	0.573174271	0.648596067	brown	-0.121570996	0.129332589	GO:0005975,GO:0004553	AT2G44480.1	BGLU17	beta glucosidase 17	glycoside hydrolase family 1 protein
Sapur.016G119700.4	0.138683339	-0.445038181	0.790715643	-0.56283	0.573550912	0.648933459	brown	-0.010354245	0.89759107	GO:0005975,GO:0004553	AT2G44480.1	BGLU17	beta glucosidase 17	glycoside hydrolase family 1 protein
Sapur.019G078600.1	3.383922989	-0.081714692	0.145707103	-0.56081	0.574923831	0.650153206	brown	-0.063926472	0.426375295		AT1G07400.1		HSP20-like chaperones superfamily protein	17.5 kDa class I heat shock protein
Sapur.001G089100.1	0.134590413	-0.57434836	1.026662877	-0.55943	0.575866733	0.65106366	brown	-0.133926031	0.094480849		AT1G63100.1		GRAS family transcription factor	GRAS family transcription factor
Sapur.003G131900.1	0.728929377	-0.164730276	0.294484627	-0.55938	0.575899013	0.651077899	brown	-0.044861929	0.576907604	GO:0006468,GO:0005524,GO:0004672	AT2G33170.1		Leucine-rich repeat receptor-like protein kinase family protein	LRR receptor-like kinase plant, putative
Sapur.15WG019200.1	0.208336988	-0.309752278	0.561697474	-0.55146	0.581320102	0.656107734	brown	-0.100494757	0.210458313	GO:0005524,GO:0003676,GO:0004386	AT3G26560.1		ATP-dependent RNA helicase, putative	ATP-dependent RNA helicase DHX8
Sapur.004G009100.2	0.105865287	-0.583885357	1.068852222	-0.54627	0.584878134	0.659650789	brown	-0.29819967	0.000148692	GO:0003824	AT1G78960.1	ATLUP2,LUP2	lupeol synthase 2	beta-amyrin synthase
Sapur.006G140200.1	7.516881285	-0.137919221	0.25261608	-0.54596	0.585090858	0.659845707	brown	-0.045866463	0.568397661	GO:0055114,GO:0020037,GO:0016705,GO:0005506	AT4G31500.1	ATR4,CYP83B1,RED1,RNT1,	cytochrome P450, family 83, subfamily B, polypeptide 1	NA
Sapur.008G037100.1	0.261956069	-0.343889836	0.632057116	-0.54408	0.586386205	0.661103672	brown	-0.052073838	0.517173237		AT5G13560.1			hypothetical protein
Sapur.006G126700.4	24.49068785	-0.038317554	0.070813291	-0.54111	0.588433966	0.66307332	brown	-0.087772236	0.274347414		AT4G29330.1	DER1	DERLIN-1	derlin protein
Sapur.001G039600.1	0.108419075	-0.547676064	1.012800247	-0.54075	0.588676963	0.663324539	brown	-0.119706309	0.13535743					transmembrane protein, putative
Sapur.019G044400.1	0.205995893	0.387049832	0.716011621	0.540564	0.5888084	0.663427439	brown	0.103233841	0.198226709		AT5G14930.2	SAG101	senescence-associated gene 101	NA
Sapur.005G145000.1	0.176781333	-0.399965266	0.740353511	-0.54024	0.589034618	0.663591898	brown	-0.114502612	0.153306369	GO:0008168	AT2G34300.2		S-adenosyl-L-methionine-dependent methyltransferases superfamily protein	methyltransferase PMT24, putative
Sapur.008G143100.1	6.585255512	0.065572384	0.121768586	0.5385	0.590231902	0.664736947	brown	0.036045058	0.65402341		AT5G20165.1			NA
Sapur.008G148900.1	0.130162847	-0.514104793	0.959889028	-0.53559	0.592243505	0.666820824	brown	-0.205910025	0.009675562		AT1G13030.1		sphere organelles protein-related	hypothetical protein
Sapur.003G113200.1	0.401729981	-0.203514461	0.382251184	-0.53241	0.594441864	0.66906824	brown	-0.062108632	0.439672402	GO:0006281,GO:0003684	AT5G44740.2	POLH	Y-family DNA polymerase H	DNA polymerase eta
Sapur.009G011900.1	0.153157425	-0.74573927	1.401185234	-0.53222	0.594573403	0.669170746	brown	-0.134053703	0.094164176	GO:0019773,GO:0006511,GO:0004175,GO:0051603,GO:0005839,GO:0004298	AT2G27020.1	PAG1	20S proteasome alpha subunit G1	proteasome subunit alpha type-3 protein
Sapur.018G002100.1	0.153423645	-0.447503498	0.841953351	-0.53151	0.595067987	0.669681806	brown	-0.154961016	0.052640035		AT3G14470.1		NB-ARC domain-containing disease resistance protein	CC-NBS-LRR resistance protein
Sapur.T032000.1	0.224396774	-0.300154656	0.565807178	-0.53049	0.595772723	0.670292446	brown	-0.102815932	0.200058868	GO:0005509	AT4G13440.1		Calcium-binding EF-hand family protein	calcium ion-binding protein, putative
Sapur.008G051500.1	448.8377285	-0.020106851	0.037992616	-0.52923	0.596645556	0.671114648	brown	-0.079387108	0.322991255	GO:0005515	AT1G07310.1		Calcium-dependent lipid-binding (CaLB domain) family protein	C2 domain protein
Sapur.016G251600.3	0.969866377	0.171662002	0.324562315	0.528903	0.596872698	0.671278823	brown	0.073300178	0.361591226	GO:0004635,GO:0000105	AT1G31860.1	AT-IE,HISN2	histidine biosynthesis bifunctional protein (HISIE)	histidine biosynthesis hisIE protein
Sapur.009G055500.1	0.118266075	-0.609625458	1.155743641	-0.52747	0.597864024	0.672233716	brown	-0.157238243	0.049218152	GO:0006412,GO:0005840,GO:0003735	AT4G29430.1	rps15ae	ribosomal protein S15A E	40S ribosomal protein S15a
Sapur.010G165700.2	0.106316285	-0.603238917	1.14985054	-0.52462	0.599844764	0.674094177	brown	-0.208977757	0.008623602	GO:0019898,GO:0015979,GO:0009654,GO:0009523,GO:0005509	AT2G39470.1	PPL2	PsbP-like protein 2	photosystem II reaction center PsbP family protein
Sapur.016G054200.2	0.139999558	-0.39989589	0.763401314	-0.52383	0.600393706	0.674642299	brown	-0.205396882	0.009862172	GO:0017025,GO:0008270,GO:0006355,GO:0006352	AT3G10330.1		Cyclin-like family protein	transcription initiation factor IIB
Sapur.008G010600.1	12.4892212	-0.066116465	0.127041409	-0.52043	0.602762237	0.676911134	brown	-0.049463334	0.538424059	GO:0048544	AT5G03700.1		D-mannose binding lectin protein with Apple-like carbohydrate-binding domain	D-mannose-binding lectin protein
Sapur.006G194800.1	0.872150004	-0.162509105	0.316805298	-0.51296	0.607977846	0.681867049	brown	-0.089502868	0.264959771		AT2G19770.1	PRF5	profilin 5	profilin
Sapur.001G038600.3	0.20071973	-0.31345612	0.61538142	-0.50937	0.610493713	0.684294204	brown	-0.098197112	0.221128293	GO:0005515	AT1G80670.1		Transducin/WD40 repeat-like superfamily protein	RNA export protein
Sapur.008G071800.7	0.599571201	-0.180931285	0.356533435	-0.50747	0.611822595	0.685667543	brown	-0.04448641	0.580104026	GO:0016020	AT1G69450.1		Early-responsive to dehydration stress protein (ERD4)	early-responsive to dehydration stress protein (ERD4), putative
Sapur.002G186500.1	0.125030094	-0.665842248	1.31656227	-0.50574	0.613037026	0.686820535	brown	-0.133295837	0.096056359	GO:0005525	AT3G07050.1		GTP-binding family protein	GTP-binding family protein, putative
Sapur.018G066900.1	0.487324236	0.188928738	0.377482394	0.500497	0.616725295	0.690273178	brown	-0.05574026	0.488070096	GO:0016021,GO:0004659	AT2G18950.1	ATHPT,HPT1,TPT1,VTE2	homogentisate phytyltransferase 1	homogentisate phytyltransferase
Sapur.014G034900.1	0.205819127	-0.28228178	0.564968536	-0.49964	0.617327464	0.690853642	brown	-0.120803464	0.131787034	GO:0006457	AT3G60370.1		FKBP-like peptidyl-prolyl cis-trans isomerase family protein	peptidyl-prolyl cis-trans isomerase
Sapur.019G006100.1	0.200195403	0.290532715	0.58193766	0.499251	0.617602865	0.691091693	brown	0.056234349	0.484216154					hypothetical protein
Sapur.006G128300.1	4.99922614	-0.127960041	0.25670849	-0.49846	0.618156762	0.691571111	brown	-0.034244468	0.670269588	GO:0005515	AT1G76900.2	AtTLP1,TLP1	tubby like protein 1	tubby-like F-box protein
Sapur.007G013900.2	0.161106606	-0.49910671	1.005442161	-0.49641	0.619608562	0.693007798	brown	-0.090480608	0.259754149	GO:0043565,GO:0008270,GO:0006355,GO:0003700	AT5G66320.2	GATA5	GATA transcription factor 5	GATA transcription factor
Sapur.008G039700.1	0.200496776	-0.364111718	0.739137935	-0.49262	0.62228342	0.695458601	brown	-0.058682934	0.465360419	GO:0005525	AT5G59840.1		Ras-related small GTP-binding family protein	Ras small GTPase family Ras protein
Sapur.012G077200.1	3.01501218	-0.087395434	0.177557983	-0.49221	0.622572412	0.695687545	brown	-0.033203956	0.679728541		AT4G24960.1	ATHVA22D,HVA22D	HVA22 homologue D	TB2/DP1, HVA22 family protein
Sapur.006G164800.1	0.2016689	0.276506835	0.56793762	0.486861	0.626356642	0.699296948	brown	0.073139224	0.362649294	GO:0008270,GO:0005515	AT5G06490.1		RING/U-box superfamily protein	RING-H2 zinc finger protein
Sapur.002G030700.3	0.172111061	-0.325845753	0.670369709	-0.48607	0.626918436	0.699881873	brown	-0.17573575	0.027698605		AT2G31260.1	APG9,ATAPG9	autophagy 9 (APG9)	autophagy protein Apg9 containing protein
Sapur.013G085000.5	4.041396492	0.1014771	0.209184679	0.485108	0.627599945	0.700571757	brown	-0.290142606	0.000227772		AT3G26580.1		Tetratricopeptide repeat (TPR)-like superfamily protein	transmembrane protein, putative
Sapur.006G028500.2	0.12042594	0.419411556	0.865385542	0.484653	0.627922629	0.700884648	brown	-0.081608864	0.309590942		AT5G09225.1			transmembrane protein, putative
Sapur.001G046300.1	5.143850074	-0.084858383	0.178394295	-0.47568	0.63430323	0.707099836	brown	-0.046089649	0.566515038		AT1G67340.1		HCP-like superfamily protein with MYND-type zinc finger	zinc finger protein, putative
Sapur.012G110600.4	0.123465236	-0.515433246	1.083923452	-0.47553	0.634412459	0.707197765	brown	-0.177835004	0.025863715	GO:0055085,GO:0016021	AT1G02260.1		Divalent ion symporter	silicon transporter
Sapur.016G286300.1	0.182965488	0.355888269	0.750710036	0.474069	0.635450787	0.708164274	brown	-0.074944011	0.350895124	GO:0004674,GO:0006468,GO:0004672	AT3G16030.1	CES101	lectin protein kinase family protein	Serine/Threonine-kinase
Sapur.014G037100.1	0.165984781	-0.371985781	0.788506875	-0.47176	0.637099075	0.709690304	brown	-0.105047747	0.190415129		AT4G17520.1		Hyaluronan / mRNA binding family	nuclear RNA-binding protein, putative
Sapur.002G173800.1	0.149995408	-0.391764426	0.833605344	-0.46996	0.638380803	0.711022283	brown	-0.158798103	0.046982547		AT2G31380.1	emb1381	embryo defective 1381	embryo defective 1381 protein
Sapur.017G014700.1	0.503574553	0.145037785	0.313904851	0.462044	0.644049922	0.716491998	brown	0.00296865	0.970564806		AT2G28580.1		Plant protein of unknown function (DUF247)	NA
Sapur.15WG078400.1	1.95353649	0.084569878	0.183137007	0.461785	0.644235684	0.716650444	brown	0.055451343	0.490331243	GO:0031225,GO:0016049,GO:0010215	AT5G60920.1	COB	COBRA-like extracellular glycosyl-phosphatidyl inositol-anchored protein family	phytochelatin synthetase-like protein
Sapur.001G016600.5	3.912874615	-0.065006158	0.141599003	-0.45909	0.646172219	0.718562973	brown	0.048680683	0.544878692	GO:0008270,GO:0005515	AT3G63530.1	BB,BB2	RING/U-box superfamily protein	E3 ubiquitin-protein ligase
Sapur.014G081500.5	20.4369506	0.031149356	0.067911833	0.458673	0.646468664	0.71883676	brown	-0.108817865	0.174901877	GO:0016773,GO:0016020,GO:0005524	AT2G46700.1	ATCRK3,CRK3	CDPK-related kinase 3	CDPK-related kinase, putative
Sapur.016G115600.1	0.150954516	-0.299599837	0.660209728	-0.45379	0.649976422	0.721992282	brown	-0.102577387	0.201110152	GO:0006468,GO:0005524,GO:0004672	AT3G23750.1		Leucine-rich repeat protein kinase family protein	receptor-like kinase, putative
Sapur.004G165500.1	0.183793455	-0.453714322	0.999907231	-0.45376	0.650004156	0.72199884	brown	-0.076212776	0.342776806		AT1G08470.1	SSL3	strictosidine synthase-like 3	strictosidine synthase
Sapur.006G043700.7	0.240772708	-0.277873198	0.613928615	-0.45261	0.650826115	0.722741931	brown	-0.092719099	0.248101476	GO:0006468,GO:0005524,GO:0004672	AT3G20860.1	ATNEK5,NEK5	NIMA-related kinase 5	Serine/Threonine kinase domain protein
Sapur.001G117300.2	0.112415893	-0.548082703	1.212622484	-0.45198	0.651282445	0.72317584	brown	-0.188474214	0.018082237					transmembrane protein, putative
Sapur.006G134900.1	184.7587442	-0.022382131	0.04957602	-0.45147	0.651650187	0.723389887	brown	-0.073692335	0.359021337	GO:0015450,GO:0006886,GO:0005744	AT1G20350.1	ATTIM17-1,TIM17-1	translocase inner membrane subunit 17-1	import inner membrane translocase subunit Tim17
Sapur.016G032100.1	0.669392493	0.146689129	0.325213139	0.451055	0.651949651	0.723698028	brown	0.0244157	0.761487208	GO:0008017,GO:0007010	AT1G68060.1	ATMAP70-1,MAP70-1	microtubule-associated proteins 70-1	microtubule associated protein
Sapur.008G051900.2	0.167917322	-0.38169403	0.850672643	-0.4487	0.653650447	0.725391231	brown	-0.100514853	0.21036665	GO:0016020,GO:0006810,GO:0005215	AT2G45960.2	ATHH2,PIP1;2,PIP1B,TMP-A	plasma membrane intrinsic protein 1B	plasma membrane intrinsic protein PIP1-1
Sapur.009G041800.1	0.126418215	-0.352373737	0.786944823	-0.44777	0.654316029	0.726056778	brown	-0.242168091	0.00224516		AT3G51970.1	ASAT1,ATASAT1,ATSAT1	acyl-CoA sterol acyl transferase 1	membrane-bound O-acyltransferase family MBOAT protein
Sapur.T138000.1	1.418809606	-0.116162062	0.259453947	-0.44772	0.65435712	0.726078015	brown	-0.107039892	0.182097306	GO:0005524,GO:0051082,GO:0006457	AT3G02530.1		TCP-1/cpn60 chaperonin family protein	NA
Sapur.013G136600.2	0.14995224	-0.333106657	0.745083487	-0.44715	0.654770077	0.726390016	brown	-0.134560862	0.092914553		AT5G37055.1	ATSWC6,SEF	HIT-type Zinc finger family protein	zinc finger protein
Sapur.007G041600.1	0.149797806	-0.493218034	1.10380877	-0.44683	0.65499574	0.726591619	brown	-0.098128077	0.221454721	GO:0003676	AT4G36960.2		RNA-binding (RRM/RBD/RNP motifs) family protein	heterogeneous nuclear ribonucleoprotein G RNA recognition motif protein
Sapur.006G131200.3	0.106282433	-0.512556901	1.147439809	-0.4467	0.65509449	0.726652418	brown	-0.181153323	0.023175729		AT2G18660.1	PNP-A	plant natriuretic peptide A	rare lipoprotein A-like double-psi beta-barrel protein
Sapur.016G194300.1	0.139152045	-0.77837881	1.750686973	-0.44461	0.656599194	0.728101715	brown	-0.122846506	0.125331668		AT4G30990.1		ARM repeat superfamily protein	small subunit processome component-like protein, putative
Sapur.018G017900.1	0.090918958	-0.476392059	1.079748636	-0.44121	0.65906355	0.730540514	brown	-0.267367835	0.000711312		AT5G10970.1		C2H2 and C2HC zinc fingers superfamily protein	NA
Sapur.007G081900.1	0.100978596	-0.474368175	1.080456189	-0.43904	0.660629408	0.732104441	brown	-0.213563224	0.007239705	GO:0005975,GO:0004553,GO:0051015,GO:0007015				glucan 1%2C3-beta-glucosidase
Sapur.014G009300.3	0.201149782	0.208275111	0.478700348	0.435085	0.66350108	0.735089764	brown	-0.081608864	0.309590942	GO:0006352,GO:0005669	AT5G43130.2	TAF4,TAF4B	TBP-associated factor 4	transcription initiation factor, putative
Sapur.002G087700.1	0.278112248	-0.242773858	0.56000176	-0.43352	0.664634582	0.736222258	brown	-0.053483621	0.505878445		AT2G22660.2		Protein of unknown function (duplicated DUF1399)	NA
Sapur.002G152300.2	0.266535099	-0.182213802	0.420883274	-0.43293	0.665064233	0.736565368	brown	-0.107629849	0.179686048		AT2G47940.1	DEGP2	DEGP protease 2	NA
Sapur.016G016300.2	0.313190928	0.164083877	0.37958134	0.432276	0.665540878	0.737028695	brown	-0.088579536	0.269940664		AT3G48880.2		RNI-like superfamily protein	F-box protein
Sapur.008G125200.1	0.098562001	-0.435994109	1.015168847	-0.42948	0.667574381	0.738736096	brown	-0.239793555	0.002487533		AT3G22550.1		Protein of unknown function (DUF581)	DUF581 family protein
Sapur.002G135600.1	0.17480967	-0.399880032	0.934181152	-0.42805	0.668611765	0.739562741	brown	-0.105260864	0.189512287	GO:0005525	AT1G02280.1	ATTOC33,PPI1,TOC33	translocon at the outer envelope membrane of chloroplasts 33	translocase of protein
Sapur.011G082200.1	0.122261484	-0.550802712	1.291739999	-0.4264	0.669813708	0.740514081	brown	-0.213487011	0.007260989		AT1G53035.1			transmembrane protein, putative
Sapur.007G016700.1	0.120285989	-0.512479952	1.208046702	-0.42422	0.67140394	0.741906849	brown	-0.207122461	0.009246977	GO:0005515	AT4G18905.1		Transducin/WD40 repeat-like superfamily protein	transducin/WD40 domain protein
Sapur.15WG073600.1	0.103142122	-0.459298291	1.086857472	-0.42259	0.672592281	0.743001346	brown	-0.167283264	0.036252601	GO:0016747	AT5G48930.1	HCT	hydroxycinnamoyl-CoA shikimate/quinate hydroxycinnamoyl transferase	NA
Sapur.006G064500.1	0.157287839	-0.339304656	0.805413408	-0.42128	0.673550546	0.743831539	brown	-0.129095965	0.107094324		AT2G07170.1		ARM repeat superfamily protein	microtubule-associated protein TORTIFOLIA protein
Sapur.T115200.1	0.129050435	-0.318303252	0.757601444	-0.42015	0.674378766	0.744621864	brown	-0.09301752	0.246575828		AT4G38620.1	ATMYB4,MYB4	myb domain protein 4	NA
Sapur.005G101400.1	28.73416483	0.032425255	0.077624937	0.417717	0.676154044	0.746357803	brown	0.02376023	0.767705544		AT1G75080.1	BZR1	Brassinosteroid signalling positive regulator (BZR1) family protein	brassinazole-resistant 1 protein, putative
Sapur.008G027700.1	0.098924294	0.446793432	1.07608194	0.415204	0.677992635	0.748137604	brown	-0.055353251	0.491100204		AT5G03300.1	ADK2	adenosine kinase 2	adenosine kinase
Sapur.004G084500.1	0.099380561	0.420896242	1.021651282	0.411976	0.680356704	0.750395759	brown	0.122341784	0.126903266		AT5G16080.1	AtCXE17,CXE17	carboxyesterase 17	carboxylesterase
Sapur.008G050400.1	0.170114388	-0.350547327	0.85382732	-0.41056	0.681395215	0.751415888	brown	-0.119276144	0.136777309		AT2G39910.1		ARM repeat superfamily protein	DUF2454 family protein
Sapur.005G074800.1	0.109272124	-0.606499874	1.501256854	-0.40399	0.686216586	0.75595135	brown	-0.160817185	0.044215015	GO:0005515,GO:0006396,GO:0003723	AT1G14650.1		SWAP (Suppressor-of-White-APricot)/surp domain-containing protein / ubiquitin family protein	suppressor-of-white-APricot splicing regulator, putative
Sapur.007G090200.2	2.54130316	0.090178003	0.233368833	0.386418	0.699186852	0.768243777	brown	-0.057620803	0.473489504		AT5G09220.1	AAP2	amino acid permease 2	amino acid permease
Sapur.010G023000.1	0.11662888	-0.436356351	1.131186288	-0.38575	0.699681016	0.768684602	brown	-0.184619398	0.020628088	GO:0003824,GO:0006470,GO:0004722	AT1G67820.1		Protein phosphatase 2C family protein	protein phosphatase 2C
Sapur.014G136500.1	1.021412775	-0.117571187	0.308633092	-0.38094	0.703246575	0.771858274	brown	-0.029082652	0.717674064	GO:0005515	AT4G16360.3		5\'-AMP-activated protein kinase beta-2 subunit protein	SNF1-related kinase regulatory subunit beta-2
Sapur.007G006400.3	0.317514774	-0.161403365	0.425668923	-0.37918	0.704557303	0.772930918	brown	-0.081608864	0.309590942		AT5G65740.2		zinc ion binding	NA
Sapur.001G042600.1	0.081780337	-0.432300118	1.140492902	-0.37905	0.704653154	0.772969095	brown	-0.29817397	0.000148897					transmembrane protein, putative
Sapur.15WG107300.1	0.107689941	0.360817789	0.962081893	0.375039	0.707631804	0.775641754	brown	0.100891118	0.208655624	GO:0055085,GO:0016020,GO:0006810,GO:0005215,GO:0006863,GO:0005345	AT5G50300.1	ATAZG2,AZG2	Xanthine/uracil permease family protein	adenine/guanine permease
Sapur.010G023300.2	0.159577535	-0.28733407	0.770513943	-0.37291	0.709213772	0.777118218	brown	-0.114646807	0.152785977		AT1G67850.2		Protein of unknown function (DUF707)	lysine ketoglutarate reductase trans-splicing protein
Sapur.009G060800.1	2.936301944	0.062836997	0.168555954	0.372796	0.709300231	0.777187206	brown	0.014587437	0.856109274		AT1G07030.1		Mitochondrial substrate carrier family protein	substrate carrier family protein
Sapur.006G017900.1	0.145364825	-0.284405606	0.764412219	-0.37206	0.709849739	0.777686253	brown	-0.148508728	0.063418498	GO:0055114,GO:0020037,GO:0016705,GO:0005506	AT2G46950.1	CYP709B2	cytochrome P450, family 709, subfamily B, polypeptide 2	cytochrome P450 family protein
Sapur.010G028000.8	0.64326285	-0.116130523	0.315627476	-0.36794	0.712921399	0.78037936	brown	-0.114216666	0.154335013	GO:0005975,GO:0004650	AT1G60590.1		Pectin lyase-like superfamily protein	polygalacturonase
Sapur.006G144100.1	0.713064239	-0.106149414	0.291206087	-0.36452	0.71547238	0.782731328	brown	-0.060619457	0.450738837					NA
Sapur.003G148500.1	0.11246572	-0.438558428	1.212988699	-0.36155	0.717686878	0.784505265	brown	-0.190532819	0.016837795	GO:0008017,GO:0000910,GO:0000226	AT2G38720.1	MAP65-5	microtubule-associated protein 65-5	microtubule-associated protein
Sapur.15WG075100.1	0.139381931	-0.274352401	0.76495917	-0.35865	0.719857138	0.786487678	brown	-0.133810726	0.094767571	GO:0016747	AT5G48930.1	HCT	hydroxycinnamoyl-CoA shikimate/quinate hydroxycinnamoyl transferase	NA
Sapur.011G100300.1	0.379096508	-0.172343567	0.480601792	-0.3586	0.719894714	0.786502141	brown	-0.01340157	0.867695185	GO:0044599	AT3G15160.1			NA
Sapur.005G162700.1	0.231003892	-0.19379038	0.545346268	-0.35535	0.722325226	0.788897551	brown	-0.060163039	0.454161601	GO:0005524,GO:0003676,GO:0004386	AT2G30800.1	ATVT-1,HVT1	helicase in vascular tissue and tapetum	ATP-dependent RNA helicase, putative
Sapur.011G011300.1	0.096219333	-0.494501861	1.406987176	-0.35146	0.725242131	0.79161276	brown	-0.134494686	0.093076857	GO:0005515	AT1G62390.1		Octicosapeptide/Phox/Bem1p (PB1) domain-containing protein / tetratricopeptide repeat (TPR)-containing protei	heat shock protein 70 (HSP70)-interacting protein, putative
Sapur.15WG004600.1	0.090058996	-0.529742914	1.511307212	-0.35052	0.725948727	0.792227148	brown	-0.16203879	0.042607715	GO:0005515	AT4G00820.1	iqd17	IQ-domain 17	IQ calmodulin-binding motif protein, putative
Sapur.003G079600.1	3.252117272	-0.05615465	0.16046257	-0.34995	0.726372606	0.792663573	brown	-0.02584339	0.747996114		AT4G12230.1		alpha/beta-Hydrolases superfamily protein	esterase/lipase/thioesterase family protein, putative
Sapur.008G005500.1	0.842385599	0.102591159	0.300037562	0.341928	0.732405289	0.797746501	brown	0.019822332	0.805362898	GO:0030132,GO:0030130,GO:0016192,GO:0006886,GO:0005198	AT3G11130.1		Clathrin, heavy chain	clathrin heavy chain
Sapur.010G153800.3	1.312668411	0.101159206	0.296898695	0.34072	0.733314678	0.798579226	brown	0.013203344	0.869634671		AT2G39855.1			hypothetical protein
Sapur.003G002300.3	0.116434185	-0.386101776	1.143855711	-0.33754	0.735706754	0.800630599	brown	-0.109526427	0.172093669	GO:0005515,GO:0006414,GO:0003746	AT1G09640.1		Translation elongation factor EF1B, gamma chain	elongation factor 1-gamma 1
Sapur.011G057800.1	0.107939455	-0.364636834	1.081605857	-0.33713	0.736022352	0.800894992	brown	-0.150988928	0.05907913		AT4G18260.1		Cytochrome b561/ferric reductase transmembrane protein family	cytochrome b561/ferric reductase transmembrane family protein, putative
Sapur.009G033900.1	0.132838254	-0.266019193	0.78998621	-0.33674	0.736313635	0.801132876	brown	-0.1646516	0.039333745	GO:0007010,GO:0003779,GO:0051017	AT2G29890.2	ATVLN1,VLN1	villin-like 1	villin, putative
Sapur.003G127100.1	0.261227788	-0.284178031	0.852113624	-0.3335	0.738758539	0.803359174	brown	-0.067562914	0.400483991					NA
Sapur.014G089100.4	0.106837832	-0.400622932	1.216394689	-0.32935	0.74188908	0.806191333	brown	-0.16701653	0.036555317		AT4G01790.1		Ribosomal protein L7Ae/L30e/S12e/Gadd45 family protein	ribonuclease P, putative
Sapur.016G101100.1	0.120275293	-0.339672302	1.032917061	-0.32885	0.742270878	0.806526727	brown	-0.13176805	0.099962353		AT5G01450.1		RING/U-box superfamily protein	ubiquitin-protein ligase, putative
Sapur.008G033000.1	0.213590591	-0.217110153	0.664360367	-0.3268	0.743822365	0.807947088	brown	-0.065527139	0.414861476	GO:0015986,GO:0015078,GO:0000276	AT3G52300.1	ATPQ	ATP synthase D chain, mitochondrial	ATP synthase subunit D
Sapur.15WG012100.2	0.139175014	-0.248636516	0.763598172	-0.32561	0.744718142	0.808734168	brown	-0.107495371	0.180233604	GO:0005515	AT4G27910.1	ATX4,SDG16	SET domain protein 16	histone-lysine N-methyltransferase ATX5
Sapur.012G102900.3	0.132866686	-0.29630879	0.920765231	-0.32181	0.747598834	0.811249839	brown	-0.210786522	0.008051969		AT5G52030.2		TraB family protein	TraB family protein
Sapur.005G027600.6	0.391078131	-0.111898671	0.350709981	-0.31906	0.749678541	0.813053124	brown	-0.081608864	0.309590942	GO:0007264,GO:0005525	AT5G27540.2	emb2473,MIRO1	MIRO-related GTP-ase 1	Rho GTPase
Sapur.013G135300.2	0.166854222	-0.229933508	0.726325513	-0.31657	0.751569233	0.81464952	brown	-0.131600901	0.100397196	GO:0009378,GO:0006310,GO:0006281	AT3G01610.1	CDC48C,emb1354	cell division cycle 48C	cell division control-like protein
Sapur.006G141500.3	0.541780076	0.13051704	0.413031336	0.315998	0.752004093	0.815067454	brown	0.002905577	0.97118993	GO:0005992,GO:0003824	AT2G18700.1	ATTPS11,ATTPSB,TPS11	trehalose phosphatase/synthase 11	trehalose-6-phosphate synthase
Sapur.001G150100.1	0.295342579	-0.144862635	0.46003576	-0.31489	0.752841916	0.815868591	brown	-0.064337646	0.423400182		AT4G08520.1		SNARE-like superfamily protein	nonclathrin coat protein
Sapur.013G036500.2	3.558586837	0.059677788	0.189604865	0.314748	0.752952854	0.815914912	brown	-0.090440828	0.259964563		AT5G28150.1		Plant protein of unknown function (DUF868)	plant/T9J14-19 protein
Sapur.017G127300.1	0.065714757	0.408737658	1.316553185	0.31046	0.756210852	0.81876838	brown	-0.02991047	0.709993026	GO:0055085,GO:0016021,GO:0016020,GO:0006810,GO:0005215	AT5G14940.1		Major facilitator superfamily protein	peptide transporter
Sapur.018G068500.2	3.540234265	0.058083821	0.189233234	0.306943	0.758886741	0.821047435	brown	-0.081608864	0.309590942	GO:0016020,GO:0009055,GO:0008137	AT4G26965.1		NADH:ubiquinone oxidoreductase, 17.2kDa subunit	NA
Sapur.007G000500.1	0.106312885	-0.350831875	1.157593948	-0.30307	0.761836597	0.823646169	brown	-0.106024547	0.18630271	GO:0005524	AT1G50410.1		SNF2 domain-containing protein / helicase domain-containing protein / zinc finger protein-related	DNA helicase
Sapur.008G003300.3	0.182992179	0.208695001	0.695638512	0.300005	0.764173375	0.825929552	brown	0.0018179	0.981972361		AT1G51560.1		Pyridoxamine 5\'-phosphate oxidase family protein	pyridoxamine 5'-phosphate oxidase family protein
Sapur.002G197800.6	0.231950467	-0.152089009	0.515116576	-0.29525	0.767801663	0.829210735	brown	-0.081608864	0.309590942	GO:0005515,GO:0016459,GO:0005524,GO:0003774	AT1G04160.1	ATXIB,XI-8,XI-B,XIB	myosin XI B	myosin XI, putative
Sapur.003G074600.1	0.126333766	-0.416720366	1.414184931	-0.29467	0.768244636	0.829516604	brown	-0.069469733	0.387289961	GO:0006376,GO:0005685,GO:0003729	AT5G51410.2		LUC7 N_terminus domain-containing protein	LUC7 amino-terminal protein
Sapur.014G061500.1	0.192605742	-0.193411696	0.658577622	-0.29368	0.769001719	0.830252754	brown	-0.047063607	0.558334319		AT4G00585.1			hypothetical protein
Sapur.007G066100.2	0.07336734	0.352673473	1.216984039	0.289793	0.771974592	0.833008751	brown	-0.304028922	0.000108358		AT5G23120.1	HCF136	photosystem II stability/assembly factor, chloroplast (HCF136)	photosystem II stability/assembly factor HCF136
Sapur.019G081900.1	0.108133148	-0.51211446	1.767250705	-0.28978	0.771984311	0.833008751	brown	-0.097016228	0.22675915	GO:0052726,GO:0052725,GO:0047325,GO:0032957,GO:0005622,GO:0005524,GO:00028	AT5G16760.1		Inositol 1,3,4-trisphosphate 5/6-kinase family protein	inositol 1%2C3%2C4-trisphosphate 5/6-kinase
Sapur.008G151700.4	0.154107291	-0.210954971	0.72977787	-0.28907	0.772529821	0.833463312	brown	-0.101098129	0.207718533		AT1G23360.1	MENG	S-adenosyl-L-methionine-dependent methyltransferases superfamily protein	ubiquinone/menaquinone biosynthesis methyltransferase
Sapur.013G087500.1	0.257422348	0.161013449	0.558272504	0.288414	0.773030067	0.833975811	brown	0.004610556	0.954299248	GO:0006468,GO:0005524,GO:0004672	AT1G17910.1		Wall-associated kinase family protein	NA
Sapur.006G017800.2	0.078417473	0.375374985	1.304334244	0.28779	0.773507121	0.834381274	brown	0.032948889	0.682054971		AT4G16660.1		heat shock protein 70 (Hsp 70) family protein	heat shock 70 kDa protein
Sapur.004G084600.1	0.224625227	-0.129209505	0.450188199	-0.28701	0.77410298	0.834833767	brown	-0.133922082	0.094490656		AT1G67140.3	SWEETIE	HEAT repeat-containing protein	NA
Sapur.003G108900.2	0.283401594	0.168298407	0.593234362	0.283696	0.776643113	0.83691823	brown	-0.14191208	0.076240653		AT3G50790.1		esterase/lipase/thioesterase family protein	alpha/beta hydrolase family protein
Sapur.011G089900.1	0.196669462	-0.172130443	0.607937182	-0.28314	0.777070643	0.837269822	brown	-0.116411432	0.146524924	GO:0006468,GO:0004672	AT5G54380.1	THE1	protein kinase family protein	tyrosine kinase domain protein
Sapur.003G165500.1	140.8707724	0.022323759	0.079330875	0.281401	0.778403124	0.838204665	brown	0.010209065	0.8990193	GO:0005515	AT2G44130.1		Galactose oxidase/kelch repeat superfamily protein	F-box protein
Sapur.010G170900.1	2.245877338	0.063225771	0.225685891	0.280149	0.779362871	0.839070721	brown	0.012283692	0.87864272		AT3G55020.1		Ypt/Rab-GAP domain of gyp1p superfamily protein	RabGAP/TBC domain protein
Sapur.001G130300.2	0.188550179	-0.217536228	0.777667623	-0.27973	0.779685387	0.839308144	brown	-0.087797536	0.274208576	GO:0005515,GO:0055114,GO:0046872,GO:0016702	AT1G17420.1	LOX3	lipoxygenase 3	linoleate 13S-lipoxygenase
Sapur.016G119400.1	0.129836339	0.270449919	0.969185278	0.279044	0.780211389	0.839751097	brown	0.052355364	0.514907439		AT5G53530.1	VPS26A	vacuolar protein sorting 26A	vacuolar protein sorting-associated protein
Sapur.T004500.9	0.199135577	0.176334288	0.635986251	0.277261	0.781579577	0.840789195	brown	0.009998025	0.901096024	GO:0003676	AT3G07810.2		RNA-binding (RRM/RBD/RNP motifs) family protein	RNA recognition motif 1 in heterogeneous nuclear ribonucleoprotein A subfamily protein
Sapur.010G054600.4	0.166766262	-0.152041004	0.551959251	-0.27546	0.782965207	0.84203002	brown	-0.21215638	0.007641665	GO:0018342,GO:0008318	AT4G24490.2	ATRGTA1,RGTA1	RAB geranylgeranyl transferase alpha subunit 1	NA
Sapur.013G078500.1	0.088701252	0.276151911	1.013013478	0.272604	0.785157344	0.843975885	brown	-0.052198208	0.516171641	GO:0003723	AT4G13070.1		RNA-binding CRS1 / YhbY (CRM) domain protein	NA
Sapur.019G001900.4	0.138209262	-0.222773571	0.82861299	-0.26885	0.788044216	0.846301905	brown	-0.156562193	0.050214168		AT5G23610.1			hypothetical protein
Sapur.018G119000.4	0.167813119	-0.226082002	0.845618205	-0.26736	0.789194253	0.847241324	brown	-0.067519948	0.400784337		AT5G53530.1	VPS26A	vacuolar protein sorting 26A	vacuolar protein sorting-associated protein
Sapur.016G190700.1	0.155114968	-0.224245025	0.839404641	-0.26715	0.789355437	0.847359355	brown	-0.089830391	0.263208126		AT1G15800.1			hypothetical protein
Sapur.008G090000.3	0.078782249	-0.286260713	1.075749234	-0.2661	0.790159444	0.848139858	brown	-0.092003815	0.251784893	GO:0008725,GO:0006284	AT1G13635.2		DNA glycosylase superfamily protein	DNA-3-methyladenine glycosylase I
Sapur.016G234600.1	0.089282635	-0.325356004	1.224365742	-0.26573	0.790443826	0.848390041	brown	-0.146571214	0.066986923	GO:0008168	AT4G26600.1		S-adenosyl-L-methionine-dependent methyltransferases superfamily protein	NOL1/NOP2/sun family RNA methylase, putative
Sapur.005G063000.1	0.100013609	-0.254669234	0.965202966	-0.26385	0.79189516	0.849644473	brown	-0.164816294	0.039134664	GO:0006412,GO:0005840,GO:0005622,GO:0003735	AT1G77940.1		Ribosomal protein L7Ae/L30e/S12e/Gadd45 family protein	60S ribosomal protein L30
Sapur.002G087400.3	0.083254445	0.367297498	1.401541069	0.262067	0.793269879	0.850788248	brown	0.107360602	0.180783578		AT5G64460.2		Phosphoglycerate mutase family protein	phosphoglycerate mutase family protein
Sapur.004G136300.1	0.247054617	-0.168618697	0.645642703	-0.26116	0.79396596	0.851396338	brown	-0.059017514	0.462815694		AT1G47670.1		Transmembrane amino acid transporter family protein	NA
Sapur.002G096900.1	0.112610909	-0.423960182	1.624840943	-0.26092	0.794151029	0.851429581	brown	-0.083560069	0.298126799		AT2G26680.1			FkbM family methyltransferase

Sapur.016G080400.2	0.157827438	-0.194967707	0.750053066	-0.25994	0.794911174	0.852023598	brown	0.02646886	0.74210939		AT3G53470.2			transmembrane protein, putative
Sapur.006G200600.1	0.211150108	-0.139595657	0.537338052	-0.25979	0.795024897	0.852090264	brown	-0.081861714	0.308089289		AT1G12700.1		ATP binding;nucleic acid binding;helicases	NA
Sapur.T117800.1	0.156624236	-0.286952662	1.105801586	-0.2595	0.79525147	0.85230548	brown	-0.050039395	0.533697593					NA
Sapur.013G038700.2	0.077034588	-0.358916842	1.399518759	-0.25646	0.797597726	0.854321756	brown	-0.214950548	0.006861835	GO:0006355,GO:0005634	AT1G04240.1	IAA3,SHY2	AUX/IAA transcriptional regulator family protein	indole-3-acetic acid inducible protein
Sapur.005G020700.1	2.279453078	-0.046211045	0.183412061	-0.25195	0.801078127	0.857300048	brown	0.014061976	0.861239383		AT3G50700.1	AtIDD2,IDD2	indeterminate(ID)-domain 2	zinc finger protein
Sapur.012G057400.1	1.238337575	0.076266082	0.305505596	0.249639	0.80286661	0.858769453	brown	0.024186987	0.763655258	GO:0005515	AT5G40200.1	DegP9	DegP protease 9	serine endopeptidase degp2 protein, putative
Sapur.002G039600.1	0.095563179	-0.441921336	1.775344126	-0.24892	0.803421498	0.859307397	brown	-0.114230923	0.154290505		AT4G02715.1			hypothetical protein
Sapur.15WG151200.1	0.110903486	-0.438158645	1.763664736	-0.24844	0.803796717	0.859604915	brown	-0.069213483	0.389047632		AT5G23890.1			oxidoreductase/transition metal ion-binding protein, putative
Sapur.003G157600.3	0.19119753	0.138101617	0.559907966	0.246651	0.805178666	0.860677249	brown	-0.115732775	0.148909453	GO:0055085,GO:0046873,GO:0030001,GO:0016020	AT3G20870.1	ZTP29	ZIP metal ion transporter family	zinc transporter
Sapur.005G102500.1	0.102035463	0.262435668	1.069516002	0.245378	0.806163784	0.861376913	brown	-0.081608864	0.309590942	GO:0008270	AT4G36860.1	DAR1	LIM domain-containing protein	zinc ion-binding protein, putative
Sapur.005G041800.1	0.090366281	-0.433643315	1.76768145	-0.24532	0.80621055	0.861399054	brown	-0.154896971	0.052739049	GO:0016592,GO:0006357,GO:0001104	AT3G04740.1	ATMED14,MED14,SWP	RNA polymerase II transcription mediators	mediator of RNA polymerase II transcription subunit 14
Sapur.018G103300.1	0.102528616	0.250478594	1.021670736	0.245166	0.806328154	0.861496879	brown	0.068062657	0.397000464	GO:0055085,GO:0008324,GO:0006812	AT4G10310.1	ATHKT1,HKT1	high-affinity K+ transporter 1	sodium transporter HKT1
Sapur.018G116200.1	0.276449083	0.107985661	0.444487591	0.242944	0.808048662	0.862944832	brown	-0.027412593	0.733255588	GO:0030247,GO:0006468,GO:0005524,GO:0004672	AT1G16120.1	WAKL1	wall associated kinase-like 1	tyrosine kinase domain protein
Sapur.010G036100.1	0.135033398	-0.320540059	1.320436837	-0.24275	0.808196714	0.863075074	brown	-0.054193731	0.500238321	GO:0008289	AT4G14500.1		Polyketide cyclase/dehydrase and lipid transport superfamily protein	phosphatidylcholine transfer protein, putative
Sapur.006G150300.2	0.089992749	0.276318373	1.138816321	0.242636	0.808287026	0.863133765	brown	-0.084796066	0.291011963	GO:0016746,GO:0008152	AT4G30580.1	ATS2,EMB1995,LPAT1	Phospholipid/glycerol acyltransferase family protein	1-acyl-sn-glycerol-3-phosphate acyltransferase
Sapur.018G090500.1	212.2507985	-0.018487863	0.076659416	-0.24117	0.809424249	0.863828132	brown	-0.019399706	0.80943277		AT5G11090.1		serine-rich protein-related	hypothetical protein
Sapur.002G016600.1	0.107123104	-0.336993256	1.40961629	-0.23907	0.811053345	0.865203791	brown	-0.080507266	0.316188999					hypothetical protein
Sapur.006G084100.1	0.202224259	0.183481603	0.7709174	0.238004	0.811877813	0.865943654	brown	-0.029772504	0.711271163					transmembrane protein, putative
Sapur.004G170300.1	0.090988626	-0.3108003	1.310051247	-0.23724	0.81246839	0.866381808	brown	-0.134541126	0.092962933	GO:0008270,GO:0003677	AT4G32010.1	HSI2-L1,HSL1,VAL2	HSI2-like 1	transcription factor, putative
Sapur.002G127400.1	0.096877213	0.238675929	1.010855829	0.236113	0.813345196	0.867061371	brown	-0.257737126	0.001118578					transmembrane protein, putative
Sapur.012G085800.2	0.081358508	-0.308590397	1.307539441	-0.23601	0.81342609	0.867119657	brown	-0.164161779	0.039930873		AT5G61670.2			orange protein
Sapur.001G068500.2	0.445689238	0.081542533	0.346891961	0.235066	0.814157446	0.867619647	brown	-0.101476504	0.206013537	GO:0006468,GO:0005524,GO:0004672	AT4G10730.1		Protein kinase superfamily protein	Serine/Threonine kinase domain protein
Sapur.001G149900.3	14.23606758	0.020709819	0.088295699	0.234551	0.814557471	0.867926155	brown	-0.177701793	0.025976976	GO:0016787	AT1G72880.1		Survival protein SurE-like phosphatase/nucleotidase	acid phosphatase, putative
Sapur.008G031600.1	0.093076864	-0.352128384	1.506763296	-0.2337	0.81521902	0.86841516	brown	-0.142867068	0.0742642		AT3G52250.1		Duplicated homeodomain-like superfamily protein	nuclear receptor corepressor, putative
Sapur.005G186200.1	0.094027257	-0.325170041	1.394461667	-0.23319	0.815616363	0.868642588	brown	-0.142817054	0.07436667	GO:0005525,GO:0003924,GO:0000287	AT5G18570.1	ATOBGC,CPSAR1,EMB269	GTP1/OBG family protein	GTP-binding protein Obg2, putative
Sapur.017G067900.2	0.282868017	0.106547326	0.467295829	0.228008	0.81963979	0.871833629	brown	-0.009037343	0.91055781	GO:0003677,GO:0043565,GO:0006355,GO:0003700	AT1G69780.1	ATHB13	Homeobox-leucine zipper protein family	transcription factor, putative
Sapur.018G097600.2	1.802493851	0.058440523	0.257156217	0.227257	0.820223989	0.872229703	brown	0.038045359	0.636164025	GO:0016157,GO:0005985	AT5G20280.1	ATSPS1F,SPS1F	sucrose phosphate synthase 1F	sucrose phosphate synthase
Sapur.010G140700.1	0.076285023	0.260384365	1.14856013	0.226705	0.820653111	0.872629919	brown	0.070113071	0.38289837	GO:0006355,GO:0003700,GO:0007275	AT5G17430.1	BBM	Integrase-type DNA-binding superfamily protein	AP2-like ethylene-responsive transcription factor BBM2
Sapur.013G026400.3	0.184823921	-0.162489269	0.72110377	-0.22533	0.821719374	0.873426744	brown	-0.113721862	0.156147258	GO:0055085,GO:0016021,GO:0015299,GO:0006812	AT3G05030.1	ATNHX2,NHX2	sodium hydrogen exchanger 2	NA
Sapur.004G045600.1	0.269194514	-0.165025493	0.748360373	-0.22052	0.825469254	0.87656746	brown	-0.016350766	0.838938912	GO:0016491	AT4G18360.1		Aldolase-type TIM barrel family protein	peroxisomal (S)-2-hydroxy-acid oxidase, putative
Sapur.014G081800.1	0.072624018	-0.394304623	1.791571419	-0.22009	0.825802076	0.876765631	brown	-0.155160182	0.052333104		AT4G00850.1	GIF3	GRF1-interacting factor 3	NA
Sapur.016G184300.1	0.072092117	-0.431718642	1.966259468	-0.21956	0.826211187	0.87702202	brown	-0.17973899	0.024290516		AT1G76750.1		Protein of unknown function (DUF1278)	prolamin-like protein
Sapur.006G210900.1	0.272370851	-0.099922316	0.455555303	-0.21934	0.82638381	0.87708806	brown	-0.025644018	0.749875616	GO:0005543	AT2G25430.1		epsin N-terminal homology (ENTH) domain-containing protein / clathrin assembly protein-related	clathrin assembly protein, putative
Sapur.001G140200.1	0.111785148	-0.274600925	1.267677381	-0.21662	0.828506558	0.87899011	brown	-0.121897816	0.128298203	GO:0016020,GO:0006810,GO:0005215	AT5G47450.1	ATTIP2:3.DELTA-TIP3.TIP2:	tonoplast intrinsic protein 2;3	aquaporin TIP-type protein, putative
Sapur.016G150200.2	0.091022358	-0.325079415	1.509934545	-0.21529	0.829538331	0.879814993	brown	-0.102533933	0.201302085	GO:0043531	AT3G14470.1		NB-ARC domain-containing disease resistance protein	CC-NBS-LRR resistance protein
Sapur.011G050400.3	19.02088719	-0.017365279	0.081756786	-0.2124	0.831793668	0.881359126	brown	-0.152563263	0.056453515		AT1G78700.1	BEH4	BES1/BZR1 homolog 4	brassinosteroid signaling positive regulator-like protein, putative
Sapur.15WG116100.2	0.108652034	-0.202944796	0.96095037	-0.21119	0.832737644	0.881992021	brown	-0.149301217	0.062004468	GO:0046872,GO:0030001	AT5G50740.1		Heavy metal transport/detoxification superfamily protein	heavy metal-associated domain protein
Sapur.004G095900.1	0.081780494	-0.34601333	1.642408064	-0.21067	0.833141354	0.882391352	brown	-0.12591537	0.116097163	GO:0016758,GO:0005789	AT5G38460.2		ALG6, ALG8 glycosyltransferase family	ALG6, ALG8 glycosyltransferase family protein
Sapur.009G098900.1	64.4142282	-0.02406235	0.116316574	-0.20687	0.836111785	0.885055567	brown	-0.019299147	0.810401912		AT4G34700.1		LYR family of Fe/S cluster biogenesis protein	NADH ubiquinone oxidoreductase B22-like subunit
Sapur.003G018100.2	0.136837161	-0.171059883	0.828520515	-0.20646	0.836428272	0.885229555	brown	-0.10138602	0.206420347	GO:0016887,GO:0005524,GO:0016020	AT1G31770.1	ABCG14	ATP-binding cassette 14	white-brown-complex ABC transporter family protein, putative
Sapur.019G019800.2	0.08024978	0.265979606	1.2926234	0.205767	0.836972698	0.885570076	brown	0.077663816	0.333639181	GO:0006468,GO:0004672	AT1G07650.2		Leucine-rich repeat transmembrane protein kinase	NA
Sapur.016G039200.3	0.08690036	-0.39796066	1.955158602	-0.20354	0.838709915	0.886888055	brown	-0.089406142	0.265478599		AT2G41430.5	CID1,ERD15,LSR1	dehydration-induced protein (ERD15)	polyadenylate-binding protein interacting protein, putative
Sapur.016G173700.2	0.105704738	-0.220716617	1.092178871	-0.20209	0.839847662	0.887914407	brown	-0.081608864	0.309590942	GO:0006508,GO:0004185	AT1G33540.1	scpl18	serine carboxypeptidase-like 18	serine carboxypeptidase, putative
Sapur.005G087900.4	1.339040572	0.046492428	0.230064488	0.202084	0.839850796	0.887914407	brown	-0.081608864	0.309590942		AT4G35920.3	MCA1	PLAC8 family protein	MID1-complementing activity protein
Sapur.010G203100.2	0.100154174	-0.216919116	1.075802902	-0.20163	0.840202373	0.888191686	brown	-0.122460437	0.126532445	GO:0016020,GO:0005509,GO:0004571	AT1G51590.1	MANIB,MNS1	alpha-mannosidase 1	mannosyl-oligosaccharide 1%2C2-alpha-mannosidase, putative
Sapur.006G066600.1	0.100087765	-0.252184083	1.297050073	-0.19443	0.845840024	0.893294791	brown	-0.079850745	0.320164402		AT5G24710.1		Transducin/WD40 repeat-like superfamily protein	peptidase S8 and S53, subtilisin%2C kexin, sedolisin; WD40-like protein
Sapur.002G113000.2	0.087227246	-0.235879207	1.219009429	-0.1935	0.846566839	0.893805518	brown	-0.138692506	0.083218868		AT4G17520.1		Hyaluronan / mRNA binding family	NA
Sapur.008G148700.3	0.061310937	-0.319419958	1.653692284	-0.19316	0.846837094	0.894033773	brown	-0.21246202	0.007552692		AT1G24560.1			paramyosin, putative
Sapur.010G124700.8	0.758164451	-0.055714406	0.288798774	-0.19292	0.847023394	0.894201913	brown	-0.115430784	0.149979911	GO:0005515,GO:0003677	AT2G03390.2		uvrB/uvrC motif-containing protein	hemimethylated DNA-binding domain protein
Sapur.T117000.1	4.139782351	0.030735883	0.159767469	0.192379	0.847445454	0.894590372	brown	-0.07774424	0.333137324	GO:0005524,GO:0005515	AT4G27220.1		NB-ARC domain-containing disease resistance protein	BED finger NBS-LRR resistance protein
Sapur.T005100.2	0.085207849	-0.252166384	1.312949094	-0.19206	0.847694383	0.89479603	brown	-0.108798626	0.174978594		AT1G55930.1		CBS domain-containing protein / transporter associated domain-containing protein	magnesium and cobalt efflux protein CorC, putative
Sapur.006G062500.2	0.076379164	-0.233665928	1.220244684	-0.19149	0.848140897	0.895181644	brown	-0.217780183	0.006145071		AT2G19080.1		metaxin-related	outer membrane import complex protein metaxin, putative
Sapur.002G072200.1	0.090569407	-0.287611654	1.503180496	-0.19134	0.848262822	0.895281762	brown	-0.124432844	0.12048985	GO:0051087,GO:0042803,GO:0006457,GO:0000774	AT1G36390.1		Co-chaperone GrpE family protein	co-chaperone GrpE
Sapur.008G165100.3	0.269077318	-0.094898402	0.499756559	-0.18989	0.84939591	0.896163084	brown	-0.040088311	0.618137595	GO:0046872	AT2G47850.1		Zinc finger C-x8-C-x5-C-x3-H type family protein	zinc finger CCCH domain protein, putative
Sapur.008G106400.1	0.189239683	0.13702987	0.724242777	0.189204	0.849932674	0.896586395	brown	0.013645421	0.865310383		AT5G28010.1		Polyketide cyclase/dehydrase and lipid transport superfamily protein	pathogenesis-related protein Bet V I family protein
Sapur.004G119700.1	0.065745243	-0.341567658	1.806792862	-0.18905	0.850056463	0.896602591	brown	-0.138470719	0.083717848	GO:0006355,GO:0003677	AT4G34610.2	BLH6	BEL1-like homeodomain 6	BEL1-related homeotic protein
Sapur.T118100.1	0.071834714	-0.283887602	1.511674712	-0.1878	0.851035981	0.897263757	brown	-0.178085606	0.025651789					hypothetical protein
Sapur.012G073500.1	0.087396369	0.189411535	1.012413299	0.187089	0.851590742	0.897791412	brown	0.055811253	0.487515333		AT3G48550.1			nucleic acid-binding protein, putative
Sapur.005G192500.1	31.09885907	-0.013447535	0.072316365	-0.18595	0.852480642	0.898557739	brown	-0.063922497	0.426404114		AT5G42470.1			brain and reproductive organ-expressed protein
Sapur.006G066100.2	0.062541965	-0.282023589	1.518820011	-0.18569	0.85269102	0.898722205	brown	-0.085667566	0.286063832	GO:0005515	AT1G62440.1	LRX2	leucine-rich repeat/extensin 2	LRR amino-terminal domain protein
Sapur.010G002600.2	0.107587545	-0.215497324	1.166140293	-0.1848	0.853389541	0.899315141	brown	-0.083402183	0.299043864					transmembrane protein, putative
Sapur.003G095000.1	0.087814887	-0.397907409	2.15830669	-0.18436	0.853730325	0.899566821	brown	-0.085341881	0.28790635	GO:0016757,GO:0016020	AT5G41460.1		Protein of unknown function (DUF604)	transferring glycosyl group transferase, putative
Sapur.018G092200.1	0.273394396	-0.079437956	0.430903207	-0.18435	0.853737164	0.899566821	brown	-0.040124013	0.617824545	GO:0005515	AT1G74190.1	AtRLP15,RLP15	receptor like protein 15	NA
Sapur.013G086300.2	2.82367313	-0.044685267	0.245260698	-0.18219	0.855429715	0.900891017	brown	-0.033422457	0.677738012	GO:0003723	AT4G13070.1		RNA-binding CRS1 / YhbY (CRM) domain protein	NA
Sapur.007G025500.4	0.085994669	-0.235857003	1.302829852	-0.18103	0.856340585	0.90162062	brown	-0.125476029	0.117385725	GO:0003676	AT4G36690.4	ATU2AF65A	U2 snRNP auxilliary factor, large subunit, splicing factor	U2 snRNP auxilliary factor, large subunit%2C splicing factor
Sapur.004G040000.1	0.077160522	-0.321222856	1.775555813	-0.18091	0.856435101	0.901691429	brown	-0.180309038	0.023835782		AT2G38300.1		myb-like HTH transcriptional regulator family protein	myb transcription factor, putative
Sapur.004G133300.3	0.080372301	-0.221624652	1.227975481	-0.18048	0.856775986	0.901992104	brown	-0.1308845	0.102277877	GO:0005515	AT1G12970.1	PIRL3	plant intracellular ras group-related LRR 3	plant/F13K23-18 protein
Sapur.018G036100.1	0.117979902	-0.18285117	1.018208285	-0.17958	0.857481284	0.90259177	brown	-0.08581729	0.285219443		AT1G15215.2			hypothetical protein
Sapur.016G060900.1	0.115688817	0.189543124	1.056746692	0.179365	0.857651295	0.902684538	brown	-0.05054954	0.529529354		AT3G24255.1		RNA-directed DNA polymerase (reverse transcriptase)-related family protein	NA
Sapur.005G113500.1	0.111258059	-0.18283571	1.023933145	-0.17856	0.858281512	0.903204134	brown	-0.106263679	0.185305921		AT5G49980.1	AFB5	auxin F-box protein 5	transport inhibitor response 1 protein, putative
Sapur.001G131100.1	0.063430779	-0.319357045	1.798751106	-0.17754	0.859081291	0.90379842	brown	-0.198186335	0.012840771		AT5G35405.1		Protein of unknown function (DUF784)	NA
Sapur.001G131200.1	0.063430779	-0.319357045	1.798751106	-0.17754	0.859081291	0.90379842	brown	-0.198186335	0.012840771		AT5G35405.1		Protein of unknown function (DUF784)	NA
Sapur.004G080800.2	0.468586376	-0.071960335	0.407101455	-0.17676	0.859694823	0.904259845	brown	-0.007869196	0.922079733	GO:0016021	AT3G02420.1			NA
Sapur.005G156500.1	0.079605972	0.189600897	1.078301694	0.175833	0.860425234	0.904740389	brown	-0.073895347	0.357695429	GO:0009058,GO:0008676,GO:0008152,GO:0005737	AT1G79500.1	AtkdsA1	Aldolase-type TIM barrel family protein	NA
Sapur.003G105700.1	0.064348021	-0.266644761	1.518192884	-0.17563	0.86058228	0.904847989	brown	-0.219973019	0.005636353	GO:0005515	AT4G24020.1	NLP7	NIN like protein 7	transcription factor, putative
Sapur.005G072900.3	0.06952094	0.228526237	1.30702328	0.174845	0.861201578	0.905326457	brown	-0.115272829	0.150542122		AT5G23200.1			NA
Sapur.T001600.2	0.112043464	-0.165479049	0.952287793	-0.17377	0.862046223	0.905897651	brown	-0.139718401	0.08094176	GO:0016887,GO:0005524,GO:0055085,GO:0042626,GO:0016021,GO:0006810	AT2G34660.1	ATMRP2,EST4,MRP2	multidrug resistance-associated protein 2	multidrug resistance protein ABC transporter family protein
Sapur.014G050000.4	0.725861409	-0.048397329	0.279693969	-0.17304	0.86262256	0.906361231	brown	-0.081608864	0.309590942		AT2G45320.1			hypothetical protein
Sapur.010G022500.1	4.319791534	-0.032492376	0.188366496	-0.1725	0.863047986	0.906604703	brown	-0.03449405	0.668008336		AT3G26380.1		Melibiase family protein	alpha-galactosidase
Sapur.008G007500.1	71.58693924	-0.012571917	0.07483754	-0.16799	0.866591582	0.909373259	brown	-0.028654234	0.72166039	GO:0016627,GO:0016021,GO:0006629,GO:0005737	AT5G16010.1		3-oxo-5-alpha-steroid 4-dehydrogenase family protein	3-oxo-5-alpha-steroid 4-dehydrogenase
Sapur.018G115700.1	1.359675819	-0.043095432	0.257625406	-0.16728	0.867150184	0.909793938	brown	-0.030144927	0.70782283	GO:0006468,GO:0005524,GO:0004672	AT1G69730.1		Wall-associated kinase family protein	NA
Sapur.001G096700.4	0.209620468	0.135666492	0.811057944	0.167271	0.867156803	0.909793938	brown	0.073053059	0.363216505	GO:0055114,GO:0048038,GO:0009308,GO:0008131,GO:0005507	AT1G62810.1		Copper amine oxidase family protein	amine oxidase [copper containing] protein, putative
Sapur.005G066900.1	0.27107783	0.071039328	0.427992509	0.165983	0.868170609	0.910395521	brown	0.052073242	0.517178038	GO:0007264,GO:0005525	AT5G64813.1	LIP1	Ras-related small GTP-binding family protein	Ras-related small GTP-binding family protein
Sapur.014G025700.2	0.083214364	-0.323017571	1.94969095	-0.16568	0.868411703	0.910599087	brown	-0.081608864	0.309590942	GO:0055085,GO:0016020,GO:0006810,GO:0005215	AT2G26510.1	PDE135	Xanthine/uracil permease family protein	nucleobase ascorbate transporter
Sapur.008G081700.1	0.069818035	-0.321154602	1.974778335	-0.16263	0.8708112	0.912224565	brown	-0.114049607	0.154949924		AT4G21800.1	QQT2	P-loop containing nucleoside triphosphate hydrolases superfamily protein	GPN-loop GTPase-like protein
Sapur.012G079100.1	0.17308681	-0.115410588	0.711050908	-0.16231	0.871061834	0.91238031	brown	-0.11604045	0.147824797	GO:0006468,GO:0005524,GO:0004672	AT5G50860.1		Protein kinase superfamily protein	cyclin-dependent kinase-like Serine/Threonine kinase family protein
Sapur.016G236000.1	0.083025227	-0.469039512	2.904824395	-0.16147	0.871723911	0.912819245	brown	-0.116892682	0.144851611		AT1G55830.2			DUF812 family protein
Sapur.016G296900.1	0.059959474	-0.246405516	1.526353288	-0.16143	0.871751487	0.912819245	brown	-0.210118432	0.008259073	GO:0005525	AT1G56050.1		GTP-binding protein-related	NA
Sapur.016G159500.3	0.058447058	0.225134388	1.420089016	0.158535	0.874034924	0.914399667	brown	0.052895242	0.510576696		AT5G16950.1			plant/F2K13-100 protein, putative
Sapur.017G090500.1	0.093848273	-0.235819494	1.499324355	-0.15728	0.875021155	0.915195417	brown	-0.079989477	0.31932166					NA
Sapur.016G021800.2	0.064721758	-0.283739102	1.804377634	-0.15725	0.87504751	0.915195417	brown	-0.135687496	0.09018573	GO:0008168	AT3G57000.1		nucleolar essential protein-related	EMG1/NEP1 methyltransferase
Sapur.002G177500.1	0.136231931	-0.36443809	2.323682686	-0.15684	0.875373764	0.915323566	brown	-0.032987041	0.681706802	GO:0016020,GO:0004970	AT2G32400.1	ATGLR3.7,GLR3.7,GLR5	glutamate receptor 5	NA
Sapur.001G131000.1	0.058316295	-0.281881155	1.807970392	-0.15591	0.876103749	0.915810853	brown	-0.201710585	0.01129863		AT5G35405.1		Protein of unknown function (DUF784)	NA
Sapur.018G059900.2	11.53269823	0.024003879	0.156848702	0.153038	0.878367969	0.917452778	brown	0.005279551	0.947676852	GO:0055114,GO:0016491,GO:0006631,GO:0003857	AT4G29010.1	AIM1	Enoyl-CoA hydratase/isomerase family	peroxisomal fatty acid beta-oxidation protein
Sapur.002G170000.6	19.31066593	-0.014103483	0.092220502	-0.15293	0.878451755	0.917511316	brown	-0.112511804	0.160628137		AT3G27970.1		Exonuclease family protein	exonuclease domain protein
Sapur.016G164300.7	0.068345738	0.228741529	1.514201195	0.151064	0.879925105	0.918702031	brown	0.046828882	0.560300672		AT5G58575.1			Sgf11 (transcriptional regulation protein) protein
Sapur.005G157400.1	0.059726571	0.230087255	1.524172297	0.150959	0.880008198	0.918730781	brown	0.097905703	0.222508515	GO:0005507,GO:0055114,GO:0016491	AT5G09360.1	LAC14	laccase 14	laccase
Sapur.003G050100.2	0.070989277	-0.196172543	1.309202332	-0.14984	0.880889846	0.919390033	brown	-0.182721282	0.021991548		AT4G17520.1		Hyaluronan / mRNA binding family	nuclear RNA-binding protein, putative
Sapur.003G014300.1	0.072002554	-0.431567395	2.904824395	-0.14857	0.881893589	0.920091448	brown	-0.088211202	0.271945263	GO:0005525,GO:0003924	AT1G60500.1	DRP4C	Dynamin related protein 4C	interferon-induced GTP-binding protein mx protein, putative
Sapur.005G082300.5	0.058995813	-0.246330346	1.65881942	-0.1485	0.881950249	0.920091448	brown	-0.161550226	0.043244562	GO:0055085,GO:0016020,GO:0015297,GO:0015238,GO:0006855	AT4G38380.1		MATE efflux family protein	MATE efflux family protein
Sapur.006G114200.2	0.098572462	-0.400003387	2.70547616	-0.14785	0.882461517	0.920351975	brown	-0.071715099	0.37209454	GO:0006355,GO:0005634,GO:0003677,GO:0009725	AT1G19220.1	ARF11,ARF19,IAA22	auxin response factor 19	auxin response factor, putative
Sapur.016G118200.2	0.070320846	-0.321102113	2.186452491	-0.14686	0.883242633	0.920944731	brown	-0.104051146	0.194678731	GO:0005515	AT2G26060.1	emb1345	Transducin/WD40 repeat-like superfamily protein	cytosolic iron-sulfur protein assembly protein, putative
Sapur.004G010900.2	0.061892981	-0.319244492	2.201425143	-0.14502	0.884697298	0.922141705	brown	-0.158930851	0.046796262		AT4G04860.1	DER2.2	DERLIN-2.2	derlin-2 protein
Sapur.005G182800.2	0.063828194	-0.356670798	2.472166583	-0.14427	0.88528365	0.922636566	brown	-0.111295887	0.16522695		AT1G19530.1			hypothetical protein
Sapur.019G006200.1	0.222228781	-0.06541716	0.453673083	-0.14419	0.885346889	0.9226734	brown	-0.088219067	0.271902355		AT5G63660.1	LCR74,PDF2.5	Scorpion toxin-like knottin superfamily protein	gamma-thionin family protein
Sapur.016G308800.1	0.109986112	-0.145375588	1.019325551	-0.14262	0.886590784	0.923678686	brown	-0.082362364	0.305130084		AT5G56170.1	LLG1	LORELEI-LIKE-GPI-ANCHORED PROTEIN 1	GPI-anchored protein, putative
Sapur.014G038000.1	0.744866066	0.052991115	0.373797838	0.141764	0.88726634	0.924207827	brown	0.003810194	0.962226104		AT2G44740.1	CYCP4;1	cyclin p4;1	cyclin-U4 protein
Sapur.008G154100.1	0.198295953	0.102248217	0.724434455	0.141142	0.887757667	0.924591521	brown	-0.009654151	0.904481319	GO:0016787	AT1G10430.1	PP2A-2	protein phosphatase 2A-2	serine/threonine-protein phosphatase PP2A catalytic subunit
Sapur.008G146800.1	0.056884373	-0.281820069	1.998503096	-0.14102	0.887857636	0.924644976	brown	-0.161030085	0.043931306		AT1G24440.1		RING/U-box superfamily protein	ubiquitin-protein ligase, putative
Sapur.001G033100.1	0.129518274	-0.179121689	1.279337135	-0.14001	0.888651042	0.925183976	brown	-0.014799449	0.85404107		AT1G16000.1			GAG1At protein
Sapur.005G074900.3	0.089978979	-0.268835467	1.929527142	-0.13933	0.889191657	0.925581692	brown	-0.040946102	0.610635207		AT3G19800.1		Protein of unknown function (DUF177)	hypothetical protein
Sapur.013G011200.4	0.058030151	-0.208932359	1.527425715	-0.13679	0.891198964	0.926987095	brown	-0.197392659	0.013212499	GO:0003824,GO:0006470,GO:0004722	AT5G27930.2		Protein phosphatase 2C family protein	protein phosphatase 2C, putative
Sapur.016G096400.3	0.063332424	-0.24626807	1.800786496	-0.13676	0.89122379	0.926987095	brown	-0.147016381	0.066152879	GO:0055085,GO:0022857,GO:0016021	AT2G38060.1	PHT4;2	phosphate transporter 4;2	anion transporter, putative
Sapur.009G096200.1	0.059311553	-0.246263364	1.813116324	-0.13582	0.891961005	0.927287304	brown	-0.14068979	0.078832072	GO:0016787	AT2G16430.2	ATPAP10,PAP10	purple acid phosphatase 10	purple acid phosphatase
Sapur.018G115800.1	3.288883969	0.035018026	0.257962724	0.135748	0.892020187	0.92730876	brown	-0.000621809	0.993833224	GO:0030247,GO:0006468,GO:0005524,GO:0004672	AT1G16120.1	WAKL1	wall associated kinase-like 1	NA
Sapur.006G136800.4	0.080159939	-0.177228942	1.306083043	-0.1357	0.892062399	0.92730876	brown	-0.119384793	0.136417613	GO:0006468,GO:0005524,GO:0004672	AT2G18890.2		Protein kinase superfamily protein	receptor-like kinase
Sapur.010G185600.2	0.25026799	-0.056734846	0.420226476	-0.13501	0.892603838	0.927693157	brown	-0.319199557	4.61E-05		AT3G52170.2		DNA binding	plastid DNA-binding protein, putative
Sapur.019G030500.1	0.226667512	-0.078019733	0.582354221	-0.13397	0.893423938	0.928341292	brown	-0.095499633	0.234138313	GO:0016020,GO:0015696,GO:0008519,GO:0072488	AT1G64780.1	AMT1;2,ATAMT1;2	ammonium transporter 1;2	ammonium transporter 1 protein
Sapur.007G095200.2	0.056215729	-0.266613296	2.005478699	-0.13294	0.894238877	0.92877957	brown	-0.135367389	0.090954485	GO:0055114,GO:0016655,GO:0005886	AT1G74880.1	NDH-O	NAD(P)H:plastoquinone dehydrogenase complex subunit O	NAD(P)H-quinone oxidoreductase subunit, putative
Sapur.016G147600.2	0.088688258	0.151293508	1.141132348	0.132582	0.894524039	0.928959057	brown	0.037032416	0.645182592	GO:0055085,GO:0016021	AT5G12860.1	DiT1	dicarboxylate transporter 1	2-oxoglutarate/malate translocator
Sapur.006G014100.3	0.104194012	0.192568722	1.466639524	0.131299	0.895538557	0.929662342	brown	-0.049356474	0.539303103		AT4G16380.2		Heavy metal transport/detoxification superfamily protein	heavy metal-associated domain protein
Sapur.012G027000.1	0.073454607	-0.23358255	1.794728286	-0.13015	0.896448341	0.930314793	brown	-0.094306582	0.240060461		AT1G73480.1		alpha/beta-Hydrolases superfamily protein	alpha/beta fold hydrolase
Sapur.002G174600.1	478.8953973	-0.004573718	0.035197677	-0.12994	0.896610922	0.93045432	brown	-0.032031718	0.690445015	GO:0005515	AT4G15410.1	PUX5	serine/threonine protein phosphatase 2A 55 kDa regulatory subunit B prime gamma	plant UBX domain protein
Sapur.011G073300.2	0.078916266	-0.212584522	1.636881369	-0.12987	0.896667956	0.930484312	brown	-0.086094889	0.283658299		AT1G53300.1	TTL1	tetratricopetide-repeat thioredoxin-like 1	TPR repeat thioredoxin TTL4
Sapur.T112400.1	0.102944508	0.111107213	0.864836155	0.128472	0.897775472	0.931312171	brown	-0.025672803	0.749604164		AT4G17900.2		PLATZ transcription factor family protein	NA
Sapur.012G027700.1	0.054604862	0.192624444	1.537163103	0.125312	0.90027683	0.933350757	brown	0.04746833	0.55495168	GO:0016787	AT3G17790.1	ATACP5,ATPAP17,PAP17	purple acid phosphatase 17	purple acid phosphatase
Sapur.011G061500.1	0.094243277	-0.14185753	1.136437647	-0.12483	0.90066091	0.933661148	brown	-0.083374072	0.299207339		AT4G22320.1			hypothetical protein
Sapur.006G140400.1	0.084793383	-0.125757016	1.01345425	-0.12409	0.901245986	0.934033458	brown	-0.08545254	0.287279424	GO:0055114,GO:0020037,GO:0016705,GO:0005506	AT4G31500.1	ATR4.CYP83B1.RED1.RNT1.	cytochrome P450, family 83, subfamily B, polypeptide 1	NA
Sapur.003G041000.1	0.12789366	-0.120023351	0.971097349	-0.1236	0.901635483	0.934378567	brown	-0.076305583	0.342187672	GO:0006355,GO:0003700	AT1G53910.2	RAP2.12	related to AP2 12	ethylene-responsive-element-binding factor 2
Sapur.016G265000.1	0.16933583	-0.109847642	0.907716876	-0.12102	0.903678907	0.936176801	brown	-0.030047628	0.708723172	GO:0005096	AT5G54310.1	AGD5,NEV	ARF-GAP domain 5	ARF-GAP with coiled-coil, ANK repeat and PH domain protein, putative
Sapur.001G122500.1	0.07565125	-0.200522797	1.657058077	-0.12101	0.903682063	0.936176801	brown	-0.067535742	0.400673918	GO:0005525	AT2G44610.1	ATRAB6A.ATRABH1B.RAB6.	Ras-related small GTP-binding family protein	Ras-related protein Rab-6
Sapur.019G043400.1	0.097963558	-0.180685774	1.511914963	-0.11951	0.904872986	0.937146364	brown	-0.060471307	0.451848254		AT5G08630.1		DDT domain-containing protein	NA
Sapur.004G035500.1	0.189105205	0.081443823	0.682508008	0.11933	0.905013751	0.937233454	brown	-0.055539398	0.489641509		AT2G34040.1		Apoptosis inhibitory protein 5 (API5)	apoptosis inhibitory protein 5 (API5)
Sapur.012G056800.7	0.094276045	0.114279799	0.958736387	0.119198	0.905118211	0.937312283	brown	-0.094401937	0.23958332	GO:0055085,GO:0016020,GO:0006810,GO:0005215	AT5G62890.1		Xanthine/uracil permease family protein	nucleobase ascorbate transporter
Sapur.010G087600.2	0.105946531	-0.108259734	0.910459285	-0.11891	0.905349252	0.937463484	brown	-0.073826226	0.358146527	GO:0016887,GO:0005524,GO:0016020	AT2G01320.4		ABC-2 type transporter family protein	ABC transporter family protein
Sapur.012G075200.2	0.089932792	-0.127924864	1.077590573	-0.11871	0.905502109	0.937565754	brown	-0.10975961	0.171176851	GO:0016788	AT3G48610.1	NPC6	non-specific phospholipase C6	phospholipase C
Sapur.010G091800.1	0.069580172	-0.285435549	2.457738477	-0.11614	0.907543587	0.93950946	brown	-0.081608864	0.309590942		AT2G01260.1		Protein of unknown function (DUF789)	DUF789 family protein
Sapur.019G045800.4	0.065122011	-0.175180239	1.523575189	-0.11498	0.908461202	0.939803127	brown	-0.109918638	0.170553676	GO:0016987,GO:0006355,GO:0006352,GO:0003700,GO:0003677	AT1G08540.1	ABC1.ATSIG1.ATSIG2.SIG1.S	RNApolymerase sigma subunit 2	NA
Sapur.004G002100.2	0.06808608	-0.321013863	2.794001387	-0.11489	0.908529178	0.939814658	brown	-0.080598284	0.315640405	GO:0006096,GO:0003872	AT1G12000.1		Phosphofructokinase family protein	pyrophosphate-fructose 6-phosphate 1-phosphotransferase subunit beta
Sapur.016G174900.5	0.552479379	-0.037967299	0.331832071	-0.11442	0.908907073	0.94017616	brown	-0.115274554	0.150535973	GO:0006281,GO:0003684	AT1G49980.1		DNA/RNA polymerases superfamily protein	DNA polymerase kappa subunit
Sapur.009G059900.3	0.059223754	-0.283576872	2.493501062	-0.11373	0.909454693	0.940594044	brown	-0.092089419	0.25134209	GO:0008270	AT5G58470.2	TAF15b	TBP-associated factor 15B	RNA recognition motif protein
Sapur.001G105800.1	0.08020016	-0.149266886	1.315761112	-0.11345	0.909677544	0.940737762	brown	-0.090584715	0.25920403	GO:0006468,GO:0005524,GO:0004672	AT1G31420.2	FEI1	Leucine-rich repeat protein kinase family protein	LRR receptor-like Serine/Threonine-kinase FEI
Sapur.007G050000.1	0.065787419	-0.158681584	1.408190885	-0.11268	0.910280522	0.941196989	brown	-0.16808158	0.035359208					NA
Sapur.15WG073700.1	0.061691224	-0.158680282	1.416116987	-0.11205	0.910781313	0.941437671	brown	-0.148668743	0.063130878	GO:0016747	AT5G48930.1	HCT	hydroxycinnamoyl-CoA shikimate/quinate hydroxycinnamoyl transferase	NA
Sapur.011G070200.2	0.531781595	0.039866305	0.363014499	0.10982	0.912552013	0.942844126	brown	-0.206089596	0.009610999	GO:0055114,GO:0016616,GO:0006694,GO:0003854,GO:0019752	AT1G53240.1	mMDH1	Lactate/malate dehydrogenase family protein	malate dehydrogenase
Sapur.019G041900.1	0.546748337	-0.042979982	0.392248023	-0.10957	0.912747642	0.942969186	brown	-0.011963402	0.881783699	GO:0005515	AT5G60570.1		Galactose oxidase/kelch repeat superfamily protein	kelch repeat F-box protein
Sapur.004G049100.1	0.078992677	0.130707561	1.217362939	0.107369	0.914495908	0.944307913	brown	0.04118546	0.608548906		AT1G21280.1			NA
Sapur.003G114700.3	0.056653058	-0.193897281	1.816528228	-0.10674	0.914994787	0.944612091	brown	-0.149730228	0.061249833	GO:0005515	AT4G20130.1	PTAC14	plastid transcriptionally active 14	ribulose-1%2C5 bisphosphate carboxylase/oxygenase large subunit N-methyltransferase, putative
Sapur.006G063400.1	0.065744202	-0.210591072	1.980963762	-0.10631	0.915338474	0.944841672	brown	-0.102737506	0.200404057	GO:0005524,GO:0003676	AT2G06990.1	HEN2	RNA helicase, ATP-dependent, SK12/DOB1 protein	DEAD-box-like helicase superfamily protein
Sapur.008G158800.2	0.064728103	-0.210590878	1.981631819	-0.10627	0.915366986	0.944841672	brown	-0.094737939	0.237907309	GO:0043565,GO:0008270,GO:0006355,GO:0003700	AT3G06740.1	GATA15	GATA transcription factor 15	GATA transcription factor, putative
Sapur.010G189400.1	0.123916901	-0.095961073	0.911750231	-0.10525	0.916177993	0.945420453	brown	-0.100492072	0.210470563	GO:0055114,GO:0020037,GO:0016705,GO:0005506	AT2G27690.1	CYP94C1	cytochrome P450, family 94, subfamily C, polypeptide 1	cytochrome P450 family protein
Sapur.005G185100.2	0.115961648	-0.114356024	1.092908701	-0.10463	0.916665788	0.945717271	brown	-0.069591842	0.386454083	GO:0016021	AT1G35180.1		TRAM, LAG1 and CLN8 (TLC) lipid-sensing domain containing protein	TRAM, LAG1 and CLN8 (TLC) lipid-sensing domain protein, putative
Sapur.007G046100.4	16.6692064	0.008038067	0.076950106	0.104458	0.916805761	0.945832176	brown	-0.18952833	0.017435394	GO:0046872,GO:0000166	AT4G37270.1	ATHMA1,HMA1	heavy metal atpase 1	heavy metal ATPase, putative
Sapur.008G022200.1	0.05642712	-0.208730309	2.000032649	-0.10436	0.916880926	0.945880217	brown	-0.119567133	0.135815577					hypothetical protein
Sapur.010G010000.6	0.055566497	-0.208730054	2.000951993	-0.10432	0.916919078	0.945890072	brown	-0.081608864	0.309590942	GO:0008152,GO:0003824,GO:0016114,GO:0008661	AT4G15560.1	CLA,CLA1,DEF,DXPS2,DXS	Deoxyxylulose-5-phosphate synthase	1-deoxy-D-xylulose-5-phosphate synthase
Sapur.T117100.1	0.075471585	-0.283545807	2.752920955	-0.103	0.917964427	0.946496102	brown	-0.063446924	0.429860344		AT4G38620.1	ATMYB4,MYB4	myb domain protein 4	NA
Sapur.008G166200.3	0.068402342	-0.285394742	2.791570908	-0.10223	0.918570577	0.946884938	brown	-0.088012611	0.27303024	GO:0046872,GO:0008270,GO:0016567	AT5G18650.1		CHY-type/CTCHY-type/RING-type Zinc finger protein	CHY zinc finger protein
Sapur.006G047500.4	0.062774558	0.155141953	1.52368917	0.10182	0.918899591	0.946960931	brown	0.025261167	0.753488924	GO:0055114,GO:0050661,GO:0050660,GO:0004499	AT1G19250.1	FMO1	flavin-dependent monooxygenase 1	flavin containing monooxygenase, putative
Sapur.019G065000.3	0.10763448	-0.137838412	1.361047736	-0.10127	0.919333151	0.947228182	brown	-0.12681603	0.113490016	GO:0006415,GO:0003747	AT1G33330.1		Class I peptide chain release factor	peptide chain release factor, putative
Sapur.006G050500.1	0.063767758	-0.283539938	2.812397195	-0.10082	0.919695029	0.947512487	brown	-0.085646694	0.286181673	GO:0016758,GO:0009247	AT5G20410.1	ATMGD2,MGD2	monogalactosyldiacylglycerol synthase 2	monogalactosyldiacylglycerol synthase
Sapur.016G093300.1	0.12404863	0.087088321	0.867379743	0.100404	0.920023663	0.9476363	brown	-0.04734324	0.55599611					NA
Sapur.018G012800.1	0.060965189	-0.283538379	2.828900117	-0.10023	0.920162369	0.947698757	brown	-0.084736475	0.291352371	GO:0055085,GO:0016021	AT5G25050.1		Major facilitator superfamily protein	folate/biopterin transporter
Sapur.016G290600.1	0.060161758	0.223161397	2.232630431	0.099954	0.92038047	0.947834838	brown	0.050959039	0.526195375		AT1G11925.1		Stigma-specific Stig1 family protein	Stig1 stigma-specific protein
Sapur.017G120300.2	0.063008562	-0.246108262	2.46788168	-0.09972	0.920563051	0.947993347	brown	-0.113500689	0.156959174		AT5G52760.1		Copper transport protein family	hypothetical protein
Sapur.008G145700.1	0.075306399	-0.176894252	1.795873456	-0.0985	0.921534955	0.948513286	brown	-0.063527017	0.429277141	GO:0008601,GO:0007165,GO:0000159	AT3G26020.1		Protein phosphatase 2A regulatory B subunit family protein	serine/threonine protein phosphatase 2A 59 kDa regulatory subunit B' protein
Sapur.012G089100.1	0.052856444	-0.281681664	2.872858235	-0.09805	0.921893167	0.948801799	brown	-0.103928287	0.195209103	GO:0046872,GO:0016567,GO:0004842	AT4G19670.2		RING/U-box superfamily protein	zinc finger protein, putative
Sapur.003G013500.1	0.10064935	-0.094313395	0.965566077	-0.09768	0.922188946	0.948946874	brown	-0.13900758	0.082514119	GO:0005515,GO:0006468,GO:0005524,GO:0004672	AT4G33430.1	ATBAK1.ATSERK3.BAK1.ELG	BRI1-associated receptor kinase	somatic embryogenesis receptor kinase
Sapur.016G173100.1	0.178076532	0.068567238	0.705276696	0.09722	0.922551418	0.949213426	brown	0.017783199	0.825045933					NA
Sapur.15WG134800.1	0.067197198	-0.175035004	1.803424724	-0.09706	0.922681124	0.949314575	brown	-0.09026	0.260922526		AT2G35100.1	ARAD1	Exostosin family protein	exostosin family protein
Sapur.010G187200.1	0.055790201	-0.281678926	2.904824395	-0.09697	0.922750737	0.949329908	brown	-0.09705072	0.226593258	GO:0005524,GO:0003676	AT2G28600.1		P-loop containing nucleoside triphosphate hydrolases superfamily protein	DEAD-box ATP-dependent RNA helicase, putative
Sapur.016G176500.1	0.11917321	-0.141463887	1.47043075	-0.09621	0.923357168	0.949730029	brown	-0.001320086	0.986908531	GO:0055085,GO:0016020,GO:0006810,GO:0015171,GO:0003333	AT3G19553.1		Amino acid permease family protein	neutral amino acid transporter, putative
Sapur.002G055800.3	0.064215633	-0.173179708	1.800818411	-0.09617	0.923387761	0.949730029	brown	-0.098144582	0.221376645	GO:0055114,GO:0016491,GO:0016209	AT1G21350.3		Thioredoxin superfamily protein	antioxidant, AhpC/TSA family protein
Sapur.016G147100.1	0.195895184	-0.049701405	0.520418264	-0.0955	0.923915461	0.950097182	brown	-0.086578002	0.280955094		AT5G19855.1		Chaperonin-like RbcX protein	chaperonin-like RbcX protein
Sapur.009G081400.1	0.104858044	-0.109464377	1.149401634	-0.09524	0.924127402	0.950194784	brown	-0.098378883	0.220270439	GO:0006508	AT1G49630.2	ATPREP2,PREP2	presequence protease 2	presequence protease
Sapur.008G137800.1	0.121410209	0.087225705	0.917542588	0.095064	0.924263614	0.950253343	brown	-0.083647775	0.297618172					hypothetical protein
Sapur.019G087100.1	0.070758526	-0.123438268	1.308035628	-0.09437	0.924815886	0.950632708	brown	-0.09297926	0.246771063		AT3G58110.2			hypothetical protein
Sapur.002G090700.1	0.101715528	-0.106181578	1.135537997	-0.09351	0.925500211	0.951063973	brown	-0.069960063	0.38394009	GO:0003723	AT5G64390.1	HEN4	RNA-binding KH domain-containing protein	K-like RNA-binding domain protein
Sapur.005G106200.4	0.052223906	-0.208674541	2.238644617	-0.09321	0.925733013	0.951214541	brown	-0.119867938	0.134826852	GO:0006457,GO:0003755,GO:0000413	AT2G15790.1	CYP40,SQN	peptidyl-prolyl cis-trans isomerase / cyclophilin-40 (CYP40) / rotamase	peptidyl-prolyl cis-trans isomerase, cyclophilin-type protein
Sapur.019G078000.1	0.095272904	0.119294715	1.296363319	0.092023	0.926680089	0.951862395	brown	-0.000625523	0.993796392		AT4G03500.1		Ankyrin repeat family protein	ankyrin repeat protein
Sapur.006G033000.3	0.188008638	0.064243556	0.702086053	0.091504	0.927092271	0.952137926	brown	-0.083897255	0.296174522		AT2G41430.2	CID1,ERD15,LSR1	dehydration-induced protein (ERD15)	polyadenylate-binding protein interacting protein, putative
Sapur.016G303000.1	0.068411589	-0.179333114	1.969312779	-0.09106	0.927441893	0.95240827	brown	-0.076708751	0.339635848		AT4G03500.1		Ankyrin repeat family protein	NA
Sapur.019G066700.3	0.062646525	0.118911097	1.313248153	0.090547	0.927852295	0.952740975	brown	-0.11176627	0.163436377	GO:0008270,GO:0005515	AT1G21651.1		zinc ion binding	protein translocase subunit SECA2, putative
Sapur.002G188800.1	0.078293068	-0.176834341	1.963839737	-0.09005	0.928251297	0.952870107	brown	-0.05642601	0.482725594		AT3G07120.1		RING/U-box superfamily protein	zinc finger, C3HC4 type (RING finger) protein
Sapur.010G146900.3	0.641471474	-0.027893272	0.315299291	-0.08847	0.929506294	0.953758297	brown	-0.12853298	0.108646693	GO:0050826	AT3G55990.1	ESK1,TBL29	Plant protein of unknown function (DUF828)	Pmr5/Cas1p GDSL/SGNH-like acyl-esterase family protein
Sapur.012G025700.1	0.066662917	0.108450333	1.230823291	0.088112	0.929787639	0.953956952	brown	-0.082973788	0.30154154	GO:0005525	AT1G76810.1		eukaryotic translation initiation factor 2 (eIF-2) family protein	translation initiation factor IF-2
Sapur.004G155400.1	0.054991053	0.134522406	1.533656749	0.087714	0.930104387	0.954135185	brown	0.031761199	0.692926911	GO:0055085,GO:0016021	AT4G35300.1	TMT2	tonoplast monosaccharide transporter2	hexose transporter
Sapur.009G045900.2	0.659094998	-0.029900059	0.3453335	-0.08658	0.931002868	0.954731144	brown	-0.007685741	0.923890764		AT3G08020.1		PHD finger family protein	RING/FYVE/PHD zinc finger protein, putative
Sapur.004G110000.1	0.136588517	0.090219001	1.061416865	0.084999	0.932262464	0.955628568	brown	0.055515471	0.489828876	GO:0035091	AT2G15900.1		Phox-associated domain;Phox-like;Sorting nexin, C-terminal	sorting nexin-16 protein, putative
Sapur.010G060900.1	0.074363098	0.110533553	1.300801068	0.084973	0.932282499	0.955628568	brown	-0.073514053	0.360188246	GO:0005515	AT4G14920.1		Acyl-CoA N-acyltransferase with RING/FYVE/PHD-type zinc finger protein	acyl-CoA N-acyltransferase with RING/FYVE/PHD-type zinc finger protein, putative
Sapur.013G069200.1	0.052314286	-0.206777032	2.506881129	-0.08248	0.934262016	0.957014118	brown	-0.144920885	0.070154002		AT5G35405.1		Protein of unknown function (DUF784)	NA
Sapur.003G009500.1	0.058820885	-0.135772433	1.655289935	-0.08202	0.934628142	0.957291232	brown	0.078548531	0.32814497	GO:0005622,GO:0003676,GO:0005524,GO:0043140,GO:0006281,GO:0006260	AT1G10930.1	ATRECQ4A.ATSGS1.RECQ4A	DNA helicase (RECQl4A)	ATP-dependent DNA helicase
Sapur.002G024900.10	0.511330318	0.029076318	0.355287698	0.081839	0.934774903	0.95739559	brown	-0.079502226	0.322287866		AT2G21520.1		Sec14p-like phosphatidylinositol transfer family protein	SEC14 cytosolic factor
Sapur.010G119300.5	0.053445369	-0.135765945	1.668167887	-0.08139	0.935134776	0.957656498	brown	-0.144209125	0.071556957	GO:0008168,GO:0008152	AT3G17365.1		S-adenosyl-L-methionine-dependent methyltransferases superfamily protein	thiopurine S-methyltransferase
Sapur.019G102500.1	0.068764537	-0.123398825	1.521291032	-0.08111	0.935350861	0.957764532	brown	-0.09863043	0.219087188		AT1G03670.1		ankyrin repeat family protein	NA
Sapur.003G092400.1	0.086514972	-0.104152883	1.29959092	-0.08014	0.936123666	0.958359624	brown	-0.079387513	0.322988778	GO:0016787,GO:0006541,GO:0006177,GO:0006164,GO:0005524,GO:0003922	AT1G63660.1		GMP synthase (glutamine-hydrolyzing), putative / glutamine amidotransferase, putative	GMP synthase (glutamine-hydrolyzing)
Sapur.006G083600.2	0.05965417	-0.121191799	1.526937479	-0.07937	0.93673897	0.958800009	brown	-0.144276816	0.071422559		AT3G51800.3	ATEBP1,ATG2,EBP1	metallopeptidase M24 family protein	proliferation-associated protein 2g4
Sapur.018G029100.1	0.059512697	-0.173061652	2.213121225	-0.0782	0.937670553	0.959426924	brown	-0.109009872	0.174137583		AT5G11460.1		Protein of unknown function (DUF581)	DUF581 family protein
Sapur.014G055900.5	0.070735626	-0.102280154	1.309508889	-0.07811	0.937743946	0.959435558	brown	-0.118392221	0.139730625	GO:0006355,GO:0005634,GO:0003700,GO:0046983,GO:0003677	AT2G45650.1	AGL6	AGAMOUS-like 6	MADS-box transcription factor
Sapur.019G029700.1	0.059280535	-0.173061013	2.216226252	-0.07809	0.937757932	0.959435558	brown	-0.08872449	0.269154533	GO:0005515	AT4G33270.1	CDC20.1	Transducin family protein / WD-40 repeat family protein	cell division cycle 20, cofactor of APC complex protein
Sapur.012G009600.2	0.054733132	-0.141830114	1.823810317	-0.07777	0.938014332	0.959499745	brown	-0.117968622	0.141163042	GO:0006890	AT5G24350.1			neuroblastoma-amplified sequence protein
Sapur.T117500.1	0.077365613	0.108372474	1.397610283	0.077541	0.938192963	0.959570954	brown	0.014366244	0.858268071		AT1G14870.1	PCR2	PLANT CADMIUM RESISTANCE 2	NA
Sapur.016G102200.2	0.05288108	-0.171203315	2.233100221	-0.07667	0.938889091	0.959961391	brown	-0.108039091	0.178027265	GO:0055114,GO:0016491	AT5G05600.1		2-oxoglutarate (2OG) and Fe(II)-dependent oxygenase superfamily protein	leucoanthocyanidin dioxygenase
Sapur.008G138600.2	0.055038702	0.127780576	1.684670059	0.075849	0.939539214	0.960474512	brown	-0.012092507	0.880517384		AT3G23510.1		Cyclopropane-fatty-acyl-phospholipid synthase	cyclopropane-fatty-acyl-phospholipid synthase
Sapur.004G075100.1	0.056999091	-0.135702594	1.81293331	-0.07485	0.940332069	0.961139219	brown	-0.115585857	0.149429504	GO:0046983	AT1G69010.1	BIM2	BES1-interacting Myc-like protein 2	transcription factor BIM1, putative
Sapur.005G131600.6	0.051222401	-0.133842289	1.823397718	-0.0734	0.941485681	0.961842757	brown	-0.030529363	0.704269476	GO:0006633,GO:0005524,GO:0003989	AT1G36160.1	ACC1.AT-ACC1.EMB22.GK.P	acetyl-CoA carboxylase 1	acetyl-CoA carboxylase
Sapur.018G113300.3	0.067640385	-0.102183281	1.408152187	-0.07257	0.942151874	0.962255847	brown	-0.095336019	0.234944348	GO:0030170,GO:0009058,GO:0003824				orn/lys/arg decarboxylase
Sapur.15WG073400.1	0.052107661	-0.121175232	1.677475871	-0.07224	0.942413581	0.962387497	brown	-0.12256138	0.126217632	GO:0016747	AT5G48930.1	HCT	hydroxycinnamoyl-CoA shikimate/quinate hydroxycinnamoyl transferase	NA
Sapur.012G028600.1	0.077307441	-0.137511215	1.949285633	-0.07054	0.94376035	0.963342072	brown	-0.039838286	0.620331849	GO:0055114,GO:0020037,GO:0006979,GO:0004601	AT1G05260.1	RCI3,RCI3A	Peroxidase superfamily protein	cationic peroxidase kDa protein
Sapur.T091400.4	0.236701797	-0.038985922	0.562460464	-0.06931	0.944740337	0.963976735	brown	-0.046287713	0.564846801		AT3G27470.1		Protein of unknown function (DUF707)	lysine ketoglutarate reductase trans-splicing-related protein
Sapur.001G103000.1	0.099880895	-0.068993966	1.007108571	-0.06851	0.945382065	0.964517527	brown	-0.092052394	0.251533541	GO:0051276,GO:0005694,GO:0005524,GO:0005515,GO:0007076,GO:0000796	AT5G62410.1	ATCAP-E1.ATSMC4.SMC2.T'	structural maintenance of chromosomes 2	structural maintenance of chromosomes protein
Sapur.006G098200.3	0.064787136	-0.123367304	1.806823403	-0.06828	0.945563889	0.964568084	brown	-0.07620521	0.342824858		AT1G32700.1		PLATZ transcription factor family protein	PLATZ transcription factor
Sapur.016G150800.1	0.060362799	-0.135644483	1.987308936	-0.06826	0.94558236	0.964568084	brown	-0.075493075	0.347367181		AT3G63490.1		Ribosomal protein L1p/L10e family	50S ribosomal protein L1
Sapur.006G003800.6	0.058050528	-0.135642375	1.994659806	-0.068	0.945783436	0.964708646	brown	-0.08100376	0.313203997		AT5G47690.1		binding	androgen induced inhibitor of proliferation (As3)/PDS5 protein, putative
Sapur.005G154500.1	0.063614694	0.119255955	1.800083784	0.06625	0.947178611	0.965630522	brown	-0.000259327	0.997428114		AT4G09830.1		Uncharacterised conserved protein UCP009193	holocarboxylase synthetase
Sapur.013G035500.2	0.060753726	0.081192036	1.232443514	0.065879	0.947474231	0.965753289	brown	-0.115779006	0.148746091	GO:0008270	AT5G28050.2		Cytidine/deoxycytidylate deaminase family protein	deoxycytidylate deaminase (cytidine deaminase), putative
Sapur.008G109500.2	0.097701917	0.068987078	1.070184612	0.064463	0.948601741	0.966426009	brown	-0.02652309	0.741599697	GO:0070772,GO:0043550	AT2G01690.1		ARM repeat superfamily protein	VAC14-like protein
Sapur.012G064200.1	0.183876832	-0.040848119	0.647230846	-0.06311	0.949677214	0.967187681	brown	-0.053497475	0.505768098	GO:0006468,GO:0005524,GO:0004672	AT3G48260.1	WNK3	with no lysine (K) kinase 3	Serine/Threonine-kinase WNK2, putative
Sapur.009G126500.2	0.061199737	-0.135591192	2.204152043	-0.06152	0.950948065	0.967943213	brown	-0.064423513	0.422780406		AT2G27290.1		Protein of unknown function (DUF1279)	transmembrane protein C20orf108, putative
Sapur.004G045600.4	0.077243944	0.079983381	1.303152174	0.061377	0.951059085	0.967951726	brown	-0.019366105	0.809756573	GO:0016491	AT4G18360.1		Aldolase-type TIM barrel family protein	peroxisomal (S)-2-hydroxy-acid oxidase, putative
Sapur.010G027000.1	0.075942594	0.079986946	1.306054388	0.061243	0.951165528	0.967994359	brown	-0.08185274	0.308142506	GO:0003677,GO:0006355,GO:0005634	AT1G23380.1	KNAT6,KNAT6L,KNAT6S	KNOTTED1-like homeobox gene 6	homeobox knotted-like protein
Sapur.010G034200.2	0.093059637	0.068938923	1.140845331	0.060428	0.951814813	0.968476331	brown	-0.011407464	0.887239946	GO:0016020,GO:0006810,GO:0005215	AT1G69850.1	ATNRT1:2,NRT1:2,NTL1	nitrate transporter 1:2	nitrate transporter 1.2
Sapur.003G094200.2	0.051458074	-0.121151103	2.022410551	-0.0599	0.952231849	0.968721854	brown	-0.08649834	0.281399638	GO:0003676	AT4G35785.5		RNA-binding (RRM/RBD/RNP motifs) family protein	heterogeneous nuclear ribonucleoprotein G RNA recognition motif protein
Sapur.017G127600.1	0.141024044	-0.050134905	0.848927106	-0.05906	0.95290688	0.969050892	brown	-0.053005943	0.509691014	GO:0004866	AT1G17860.1		Kunitz family trypsin and protease inhibitor protein	kunitz-type protease inhibitor
Sapur.005G191400.3	0.076189258	0.080090819	1.400573038	0.057184	0.954398367	0.970000971	brown	-0.03370651	0.675153641	GO:0016773.GO:0005975.GO:0005524.GO:0046835.GO:0005536.GO:0004396.GO:000167	AT1G47840.1	HXK3	hexokinase 3	hexokinase
Sapur.008G006500.1	0.50638484	-0.019489842	0.352072211	-0.05536	0.955853643	0.97103191	brown	-0.079468341	0.322494805		AT3G20770.1	AtEIN3,EIN3	Ethylene insensitive 3 family protein	ethylene insensitive 3 class transcription factor
Sapur.001G051500.1	0.061095876	-0.100088188	1.808456771	-0.05534	0.955863987	0.97103191	brown	-0.082095329	0.306706122		AT1G28100.2			PPR containing protein, putative
Sapur.010G145500.1	0.058833656	-0.100085866	1.8143875	-0.05516	0.956009129	0.971130745	brown	-0.09189192	0.252364505	GO:0019867	AT5G05520.1		Outer membrane OMP85 family protein	sorting and assembly machinery component-like protein
Sapur.001G051500.4	0.056426207	-0.100083306	1.820997374	-0.05496	0.956169767	0.971211083	brown	-0.064928683	0.419144796		AT1G28100.2			PPR containing protein, putative
Sapur.002G034100.3	0.05792021	-0.121142534	2.219329029	-0.05459	0.956468925	0.97138897	brown	-0.071827323	0.371344799	GO:0006355,GO:0005634	AT5G43700.1	ATAUX2-11,IAA4	AUX/IAA transcriptional regulator family protein	indole-3-acetic acid inducible protein
Sapur.002G089600.1	0.097297536	0.052062376	0.95975001	0.054246	0.956739357	0.97153887	brown	-0.087945153	0.273399457	GO:0043565,GO:0008270,GO:0006355,GO:0003700,GO:0005515	AT4G24470.1	GATA25,TIFY1,ZIM	GATA-type zinc finger protein with TIFY domain	GATA transcription factor, putative
Sapur.008G129100.2	0.072012893	0.081465594	1.509528398	0.053968	0.956960994	0.971598035	brown	0.023870514	0.766658254					hypothetical protein
Sapur.004G101300.1	0.112112439	-0.056742242	1.075923048	-0.05274	0.957940505	0.972256552	brown	-0.003920814	0.961130275	GO:0006468,GO:0005524,GO:0004672	AT3G48750.1	CDC2.CDC2A.CDC2AAT.CDK	cell division control 2	cyclin-dependent kinase A
Sapur.008G027200.1	0.062407104	-0.100028404	1.983686986	-0.05043	0.959783318	0.973547719	brown	-0.088397078	0.270932405		AT5G04240.1	ELF6	Zinc finger (C2H2 type) family protein / transcription factor jumonji (jmj) family protein	jumonji domain protein, putative
Sapur.009G021300.1	0.07775624	0.057503264	1.144006896	0.050265	0.959911383	0.973599592	brown	-0.088558043	0.270057358	GO:0051205,GO:0016021	AT2G28800.1	ALB3	63 kDa inner membrane family protein	inner membrane protein
Sapur.002G203300.2	0.065795476	-0.064805339	1.311617521	-0.04941	0.960593582	0.974112283	brown	-0.114388133	0.153720465	GO:0046080,GO:0016787	AT3G46940.1	DUT1	DUTP-PYROPHOSPHATASE-LIKE 1	NA
Sapur.016G062400.1	0.054987059	-0.098168394	2.000334143	-0.04908	0.960858731	0.974317008	brown	-0.080300516	0.317437444	GO:0006760,GO:0004150	AT3G11750.1	FOLB1	Dihydroneopterin aldolase	dihydroneopterin aldolase
Sapur.012G072500.1	0.070665253	0.059499925	1.220637233	0.048745	0.961122538	0.974409674	brown	-0.081463422	0.310456874	GO:0006414,GO:0005853,GO:0003746	AT2G18110.1		Translation elongation factor EF1B/ribosomal protein S6 family protein	elongation factor 1-delta 1

Sapur.005G065700.2	0.103196372	-0.05491839	1.136215238	-0.04833	0.961449663	0.974651694	brown	-0.022092801	0.783590209		AT5G64930.1	CPR5,HYS1	CPR5 protein, putative	CPR-5, putative
Sapur.006G187400.1	0.129295545	0.03584012	0.75097567	0.047725	0.961935616	0.974835595	brown	-0.048078935	0.54986714		AT3G13790.1	ATBFRUCT1,ATCWINV1	Glycosyl hydrolases family 32 protein	fructan 1-exohydrolase
Sapur.013G105800.1	0.078028602	-0.066577879	1.400147553	-0.04755	0.96207439	0.974926416	brown	-0.066833118	0.405603679	GO:0016021,GO:0015693,GO:0015095	AT1G34470.1		Protein of unknown function (DUF803)	Non-imprinted in prader-willi/angelman syndrome region protein, putative
Sapur.006G172100.1	0.059655065	-0.085894049	1.817074652	-0.04727	0.96229763	0.974983584	brown	-0.059368944	0.460151106		AT5G06130.2		chaperone protein dnaJ-related	chaperone dnaJ-like protein
Sapur.019G106700.1	0.141447521	0.03750705	0.795923371	0.047124	0.962414443	0.974983584	brown	-0.037109348	0.6444958	GO:0030247,GO:0048544	AT4G03230.1		S-locus lectin protein kinase family protein	wall-associated receptor kinase galacturonan-binding protein
Sapur.004G021500.1	0.081899322	-0.052947494	1.144715624	-0.04625	0.96310793	0.975525424	brown	-0.05251653	0.513612638		AT1G61340.1		F-box family protein	F-box plant protein
Sapur.010G032300.3	0.161718301	0.031792566	0.698684913	0.045503	0.963706034	0.975892146	brown	-0.081608864	0.309590942	GO:0005515	AT1G70180.2		Sterile alpha motif (SAM) domain-containing protein	SAM domain (sterile alpha motif) protein
Sapur.016G093400.7	0.193790231	0.024900112	0.555930038	0.04479	0.964274688	0.976202476	brown	-0.036132092	0.653242148		AT3G54020.1	AtIPCS1	Arabidopsis Inositol phosphorylceramide synthase 1	phosphatidylinositol:ceramide inositolphosphotransferase
Sapur.003G070200.1	0.052728482	0.085483906	2.002608773	0.042686	0.965951622	0.977358172	brown	0.035606773	0.657963369	GO:0006351,GO:0005634,GO:0003700,GO:0043565,GO:0006355	AT2G38250.1		Homeodomain-like superfamily protein	transcription factor, putative
Sapur.005G000700.2	0.069192449	-0.064626768	1.515884899	-0.04263	0.965994064	0.977371216	brown	-0.186140388	0.019588811	GO:0016787	AT3G05910.1		Pectinacetylesterase family protein	pectin acetylesterase, putative
Sapur.016G057800.1	0.064002088	-0.062767849	1.51617842	-0.0414	0.966978032	0.978252068	brown	-0.093309407	0.245089891		AT3G58560.1		DNAse I-like superfamily protein	carbon catabolite repressor-like protein
Sapur.006G185300.7	0.082663209	0.041975017	1.014904331	0.041359	0.967010021	0.978252068	brown	-0.089789644	0.263425616	GO:0017069,GO:0000398	AT1G76940.1		RNA-binding (RRM/RBD/RNP motifs) family protein	RNA recognition motif, a.k.a. RRM%2C RBD protein
Sapur.002G093000.2	0.100850736	-0.034931735	0.865168969	-0.04038	0.967793666	0.978777989	brown	-0.090407214	0.260142459		AT1G44920.1			transmembrane protein, putative
Sapur.003G130700.1	0.078131753	-0.044650735	1.106592277	-0.04035	0.967814282	0.978777989	brown	-0.100724214	0.209413365	GO:0055114,GO:0020037,GO:0016705,GO:0005506	AT1G74110.1	CYP78A10	cytochrome P450, family 78, subfamily A, polypeptide 10	cytochrome P450 family protein
Sapur.016G309000.5	0.081480051	-0.06456194	1.619369384	-0.03987	0.96819791	0.978972108	brown	-0.016080811	0.841562887	GO:0005515	AT5G56180.1	ARP8,ATARP8	actin-related protein 8	actin-6
Sapur.004G135200.2	21.71802338	0.003807762	0.096334628	0.039526	0.968470699	0.979188062	brown	-0.008854428	0.912360802		AT2G17020.1		F-box/RNI-like superfamily protein	F-box/LRR protein
Sapur.004G120400.1	0.064455637	-0.064543313	1.653445935	-0.03904	0.968861977	0.979400322	brown	-0.054258742	0.499723619	GO:0033179,GO:0015991,GO:0015078	AT4G39080.1	VHA-A3	vacuolar proton ATPase A3	vacuolar proton ATPase a3
Sapur.006G223500.1	0.06297743	0.05957626	1.526567989	0.039026	0.968869441	0.979400322	brown	-0.013842798	0.863380966	GO:0006887,GO:0000145	AT4G31540.1	ATEXO70G1,EXO70G1	exocyst subunit exo70 family protein G1	plant/F3L17-110 protein
Sapur.008G169800.3	0.06071012	-0.062686565	1.652331653	-0.03794	0.969736919	0.9799804	brown	-0.072703851	0.365520922	GO:0006468,GO:0005524,GO:0004672	AT5G19010.1	MPK16	mitogen-activated protein kinase 16	MAP kinase
Sapur.014G031300.1	0.055285825	-0.062680755	1.663593958	-0.03768	0.969944483	0.980138828	brown	-0.087915801	0.273560208	GO:0008168,GO:0006139	AT1G19340.1		Methyltransferase MT-A70 family protein	methyltransferase-like protein
Sapur.003G062200.2	0.078643468	-0.066337888	1.784453977	-0.03718	0.970345109	0.980430903	brown	-0.02486995	0.757186717		AT5G46860.1	ATSYP22.ATVAM3.SGR3.SYF	Syntaxin/t-SNARE family protein	syntaxin
Sapur.017G062300.2	0.157478373	0.029478725	0.888696466	0.033171	0.973538426	0.982659685	brown	0.002291627	0.977275663	GO:0055085,GO:0042626,GO:0016021,GO:0006810,GO:0005524	AT1G27940.1	PGP13	P-glycoprotein 13	NA
Sapur.006G211000.2	0.062916624	-0.050650389	1.531394866	-0.03307	0.973615038	0.982671847	brown	-0.059395807	0.459947779	GO:0006355,GO:0005634	AT4G32280.1	IAA29	indole-3-acetic acid inducible 29	auxin-responsive protein IAA29
Sapur.008G132100.2	0.059165623	-0.064406804	1.998396275	-0.03223	0.974289234	0.98302752	brown	-0.055008361	0.493808957	GO:0008289	AT4G14500.1		Polyketide cyclase/dehydrase and lipid transport superfamily protein	phosphatidylcholine transfer protein, putative
Sapur.001G092500.3	0.098911371	-0.041473816	1.295899817	-0.032	0.974468961	0.983178866	brown	-0.049613061	0.537193578		AT4G22890.5	PGR5-LIKE A	PGR5-LIKE A	PGR5-like protein 1A
Sapur.007G009400.2	0.098637306	-0.039351631	1.292030459	-0.03046	0.975702426	0.983917502	brown	-0.00455954	0.95480439	GO:0006468,GO:0005524,GO:0004672	AT5G65530.1		Protein kinase superfamily protein	cysteine-rich receptor-like kinase
Sapur.011G049700.1	0.062901508	0.045936513	1.522172583	0.030178	0.97592489	0.984047432	brown	-0.007000855	0.930655186	GO:0005992,GO:0003824	AT1G06410.1	ATTPS7,ATTPSA,TPS7	trehalose-phosphatase/synthase 7	trehalose-6-phosphate synthase
Sapur.004G140500.2	0.059609475	0.045940826	1.528410681	0.030058	0.976020871	0.984081371	brown	-0.052442364	0.514208277	GO:0006470,GO:0004725	AT3G44620.2		protein tyrosine phosphatases;protein tyrosine phosphatases	Low molecular weight tyrosine-phosphatase, putative
Sapur.016G144600.2	0.131657095	-0.03582332	1.202544417	-0.02979	0.976234851	0.984239947	brown	0.016492045	0.837566378	GO:0006468,GO:0004672	AT5G12480.1	CPK7	calmodulin-domain protein kinase 7	calcium-dependent kinase
Sapur.001G137100.2	0.227804825	-0.015248491	0.523247168	-0.02914	0.976751305	0.984546635	brown	-0.097408502	0.224877532		AT1G30970.1	SUF4	zinc finger (C2H2 type) family protein	zinc finger protein
Sapur.006G132200.1	0.056028648	-0.064349189	2.231778465	-0.02883	0.976997667	0.984738856	brown	-0.054713784	0.496128798		AT3G24530.1		AAA-type ATPase family protein / ankyrin repeat family protein	NA
Sapur.014G044100.6	0.052241581	-0.060641356	2.229553641	-0.0272	0.978301111	0.985752222	brown	-0.089374388	0.265649075		AT4G00030.1		Plastid-lipid associated protein PAP / fibrillin family protein	plastid-lipid associated protein PAP
Sapur.008G079200.5	0.075516415	-0.031093568	1.223895675	-0.02541	0.979731598	0.986878379	brown	-0.077815323	0.332694153	GO:0016021,GO:0006820,GO:0016020,GO:0005452	AT2G47160.1	BOR1	HCO3- transporter family	boron transporter
Sapur.016G224000.1	0.055392346	-0.041594464	1.674386075	-0.02484	0.980181293	0.987165532	brown	-0.110731147	0.167395974	GO:0008270,GO:0005515	AT5G40250.1		RING/U-box superfamily protein	RING finger protein
Sapur.001G118100.1	0.122208178	-0.031030958	1.270192836	-0.02443	0.980509526	0.987405903	brown	0.019796396	0.805612504					hypothetical protein
Sapur.004G060600.2	0.082964455	-0.030891836	1.396904714	-0.02211	0.982356627	0.988754203	brown	-0.048803427	0.543863899	GO:0016787	AT1G14860.1	atnudt18,NUDT18	nudix hydrolase homolog 18	nudix hydrolase
Sapur.T012300.3	0.08924773	-0.030803027	1.502329429	-0.0205	0.983641712	0.989866914	brown	-0.004774084	0.952680171	GO:0008017,GO:0000910,GO:0000226	AT5G55230.1	ATMAP65-1,MAP65-1	microtubule-associated proteins 65-1	microtubule-associated protein MAP65-1a
Sapur.010G149300.1	0.537959673	-0.005720877	0.314158452	-0.01821	0.985471195	0.991074666	brown	-0.082097651	0.306692396	GO:0016598,GO:0004057	AT5G05700.1	ATATE1,ATE1,DLS1	arginine-tRNA protein transferase 1	arginyl-tRNA-protein transferase
Sapur.001G087400.3	0.073965737	-0.027163756	1.502450285	-0.01808	0.985575323	0.991078952	brown	-0.020455942	0.799271048	GO:0005524,GO:0003676	AT1G63250.1		DEA(D/H)-box RNA helicase family protein	DEAD-box ATP-dependent RNA helicase, putative
Sapur.003G116000.1	0.115999698	0.014853664	0.824387122	0.018018	0.985624632	0.991078952	brown	-0.093687432	0.243174705	GO:0051716,GO:0046658	AT4G20260.3	ATPCAP1,PCAP1	plasma-membrane associated cation-binding protein 1	DREPP plasma membrane protein
Sapur.018G048700.2	0.098982389	0.018148177	1.010079928	0.017967	0.985665123	0.991078952	brown	0.020796161	0.796004979		AT5G56750.1	NDL1	N-MYC downregulated-like 1	pollen specific protein SF21, putative
Sapur.016G144900.1	0.062529907	-0.027150404	1.521534145	-0.01784	0.985763226	0.991124862	brown	-0.070685361	0.379017267	GO:0006412,GO:0005840,GO:0003735	AT1G59359.1		Ribosomal protein S5 family protein	40S ribosomal protein S2
Sapur.016G024300.1	1.066804719	0.00499814	0.288767107	0.017309	0.986190466	0.99134592	brown	-0.063087233	0.432485067					NA
Sapur.002G131200.1	0.292673939	-0.008290945	0.491240843	-0.01688	0.986534298	0.991511174	brown	-0.030480955	0.704716571		AT1G63800.1	UBC5	ubiquitin-conjugating enzyme 5	ubiquitin-conjugating enzyme E2
Sapur.005G100400.4	0.082126426	-0.027090613	1.617383746	-0.01675	0.986636337	0.99156185	brown	0.004372474	0.9566568	GO:0003676	AT4G36690.2	ATU2AF65A	U2 snRNP auxilliary factor, large subunit, splicing factor	splicing factor U2af large protein
Sapur.017G051000.5	0.139239486	-0.013004795	0.787671545	-0.01651	0.986827183	0.99156185	brown	-0.05388536	0.502683528	GO:0008152,GO:0003824	AT3G23790.1	AAE16	AMP-dependent synthetase and ligase family protein	long-chain-fatty-acid-CoA ligase
Sapur.004G114100.1	0.050954827	0.026509714	1.671974451	0.015855	0.987349803	0.991867766	brown	-0.048388741	0.547296092	GO:0016773,GO:0048015,GO:0046854	AT1G49340.2	ATPI4K ALPHA	Phosphatidylinositol 3- and 4-kinase family protein	NA
Sapur.002G031800.1	0.08645519	-0.027021417	1.756117456	-0.01539	0.987723418	0.992042646	brown	0.005455561	0.945935109	GO:0046983,GO:0003677	AT2G03710.1	AGL3,SEP4	K-box region and MADS-box transcription factor family protein	NA
Sapur.007G067600.1	0.052292133	-0.02520585	1.66844606	-0.01511	0.987946513	0.9920597	brown	-0.059060632	0.462488309					NA
Sapur.15WG108900.3	0.054061236	-0.026930839	2.010795807	-0.01339	0.989314152	0.993117042	brown	-0.057097959	0.477519345	GO:0055114,GO:0016620	AT1G13440.1	GAPC-2,GAPC2	glyceraldehyde-3-phosphate dehydrogenase C2	glyceraldehyde-3-phosphate dehydrogenase
Sapur.009G031700.1	0.05475404	-0.025078584	2.001143208	-0.01253	0.99000107	0.993606099	brown	-0.041020518	0.609986243	GO:0005975,GO:0004553	AT3G47000.1		Glycosyl hydrolase family protein	glycoside hydrolase family 3 protein
Sapur.006G202600.1	0.149450054	0.007966592	0.643210456	0.012386	0.990117918	0.993663067	brown	-0.008393989	0.916901325	GO:0005515	AT5G41840.1		F-box/RNI-like superfamily protein	F-box protein
Sapur.013G124900.1	0.137840229	-0.009118771	0.744115294	-0.01225	0.990222557	0.993737927	brown	-0.084561249	0.292354874		AT4G28260.1			NA
Sapur.009G093800.3	0.172637104	-0.007383635	0.619681748	-0.01192	0.990493267	0.99394928	brown	-0.056989923	0.47835435	GO:0016779,GO:0009058	AT4G39210.1	APL3	Glucose-1-phosphate adenylyltransferase family protein	glucose-1-phosphate adenylyltransferase
Sapur.016G140100.2	0.056325467	0.016820193	1.537638497	0.010939	0.991272133	0.994640329	brown	-0.084009119	0.29552871	GO:0055085,GO:0016021	AT2G29650.1	ANTR1,PHT4;1	phosphate transporter 4;1	sodium-dependent phosphate transporter
Sapur.005G034400.1	0.23744786	0.004218301	0.475341175	0.008874	0.992919458	0.995870274	brown	-0.081608864	0.309590942	GO:0006468,GO:0005524,GO:0004672	AT1G54610.3		Protein kinase superfamily protein	cyclin-dependent kinase-like Serine/Threonine kinase family protein
Sapur.002G137200.2	0.055586289	-0.021233223	2.830652038	-0.0075	0.994014982	0.996688599	brown	0.018910688	0.814148373	GO:0016787.GO:0005524.GO:0003677.GO:0016891.GO:0005515.GO:0006396.GO:000452	AT1G01040.1	ASU1.ATDCL1.CAF.DCL1.EM	dicer-like 1	endoribonuclease dicer-like protein
Sapur.002G088600.1	0.050786883	0.012335371	1.824773635	0.00676	0.994606385	0.996843814	brown	-0.038875956	0.628808509		AT1G09920.1		TRAF-type zinc finger-related	TRAF-type zinc finger protein
Sapur.003G063800.2	0.052380287	0.010334056	1.53934273	0.006713	0.994643609	0.996843814	brown	-0.065104152	0.417886249	GO:0043565,GO:0006355,GO:0003700	AT2G35530.1	AtbZIP16,bZIP16	basic region/leucine zipper transcription factor 16	BZIP transcription factor
Sapur.004G133500.4	0.072725628	0.008268381	1.302883778	0.006346	0.994936487	0.996967005	brown	-0.060156058	0.454214063	GO:0003824,GO:0006470,GO:0004722	AT4G38520.2		Protein phosphatase 2C family protein	protein phosphatase 2C, putative
Sapur.T171000.1	0.208710591	0.004045453	0.651550724	0.006209	0.995045998	0.9969752	brown	-0.026892177	0.738133705		AT1G40390.1		DNAse I-like superfamily protein	NA
Sapur.006G083200.4	0.052056202	0.010481057	1.829223457	0.00573	0.995428318	0.997120491	brown	-0.052375213	0.514747884	GO:0006629,GO:0055114,GO:0016717	AT5G05580.1	FAD8	fatty acid desaturase 8	omega-3 fatty acid desaturase
Sapur.004G030800.2	0.055662885	0.010538587	2.000619377	0.005268	0.995797033	0.99718568	brown	-0.033483742	0.677180108	GO:0006355,GO:0003677	AT1G28310.2		Dof-type zinc finger DNA-binding family protein	Dof zinc finger protein
Sapur.007G060000.1	0.091224354	0.005402907	1.02883533	0.005251	0.995809945	0.99718568	brown	-0.049796256	0.535689962		AT4G35510.1			hypothetical protein
Sapur.003G082300.1	0.064787539	-0.006953551	1.418413066	-0.0049	0.99608851	0.997204086	brown	-0.098421767	0.2200684	GO:0030170,GO:0009058	AT1G62960.1	ACS10	ACC synthase 10	1-aminocyclopropane-1-carboxylate synthase, putative
Sapur.003G063700.1	0.055748223	0.008686305	2.009650233	0.004322	0.996551317	0.997546632	brown	-0.045789224	0.569049874		AT1G32160.1		Protein of unknown function (DUF760)	plant/F3C3-6 protein
Sapur.017G068000.1	0.152982435	-0.002732851	0.865010281	-0.00316	0.997479225	0.998263976	brown	0.036565607	0.64935631	GO:0016817	AT5G39840.1		ATP-dependent RNA helicase, mitochondrial, putative	ATP-dependent RNA helicase
Sapur.009G008400.5	0.115203593	0.001863388	0.959059112	0.001943	0.998449765	0.998850173	brown	-0.061672361	0.442898348	GO:0055114,GO:0046429,GO:0016114	AT5G60600.1	CLB4,CSB3,GCPE,HDS,ISPG	4-hydroxy-3-methylbut-2-enyl diphosphate synthase	4-hydroxy-3-methylbut-2-en-1-yl diphosphate synthase
Sapur.014G080200.3	0.039007172	0.282514811	1.868412361	0.151206	0.879813365	NA	brown	-0.093780512	0.242704738	GO:0046872,GO:0000166	AT4G00900.1	ATECA2,ECA2	ER-type Ca2+-ATPase 2	endoplasmic reticulum-type calcium-transporting ATPase
Sapur.004G054900.1	0.048983638	-0.244399481	1.833287449	-0.13331	0.893946524	NA	brown	-0.201663579	0.011318076		AT1G17880.1	ATBTF3,BTF3	basic transcription factor 3	transcription factor-BTF3-like protein
Sapur.004G177300.2	0.035747657	0.269076331	2.077521254	0.129518	0.896947809	NA	brown	-0.068533868	0.39373247		AT1G33400.1		Tetratricopeptide repeat (TPR)-like superfamily protein	heat shock protein 70 (HSP70)-interacting protein, putative
Sapur.012G003400.1	0.027619823	0.246986125	1.925807342	0.128251	0.897950584	NA	brown	0.127840483	0.110580293	GO:0055114,GO:0020037,GO:0006979,GO:0004601	AT1G05260.1	RCI3,RCI3A	Peroxidase superfamily protein	NA
Sapur.012G029600.1	0.045530008	-0.206987636	1.684996277	-0.12284	0.902232533	NA	brown	-0.081608864	0.309590942		AT4G37110.1		Zinc-finger domain of monoamine-oxidase A repressor R1	monoamine-oxidase A repressor R1 zf-4CXXC-R1: zinc-finger protein
Sapur.019G074900.1	0.043705349	-0.206985026	1.690637486	-0.12243	0.90255835	NA	brown	-0.23333694	0.00327103	GO:0046983,GO:0003677,GO:0006355,GO:0005634,GO:0003700	AT4G09960.3	AGL11,STK	K-box region and MADS-box transcription factor family protein	agamous MADS box factor transcription factor
Sapur.014G119300.2	0.046738353	0.194145867	1.683926787	0.115294	0.908212465	NA	brown	-0.037496486	0.641044299		AT5G16490.1	RIC4	ROP-interactive CRIB motif-containing protein 4	wiscott-aldrich syndrome, carboxy-terminal protein%2C putative
Sapur.016G275600.1	0.038138813	0.233535012	2.061563019	0.113281	0.909808129	NA	brown	-0.057124784	0.477312141		AT5G54730.1	ATATG18F,ATG18F,G18F	homolog of yeast autophagy 18 (ATG18) F	breast carcinoma amplified sequence protein, putative
Sapur.15WG101100.2	0.049776679	0.156501528	1.430974608	0.109367	0.912911336	NA	brown	-0.140954332	0.078265271	GO:0006950	AT5G47740.1		Adenine nucleotide alpha hydrolases-like superfamily protein	universal stress family protein
Sapur.002G088000.1	0.04363067	0.194222059	1.852367462	0.104851	0.916494265	NA	brown	0.049519676	0.537960868	GO:0055085	AT1G09930.1	ATOPT2,OPT2	oligopeptide transporter 2	oligopeptide transporter OPT family protein
Sapur.019G050100.1	0.031862381	0.196013467	1.900507308	0.103137	0.917853887	NA	brown	0.060211619	0.453796597		AT5G17020.1	ATCRM1.ATXPO1.HIT2.XPO	exportin 1A	NA
Sapur.014G124700.1	0.033226636	0.27111279	2.639616925	0.102709	0.918193818	NA	brown	0.139845908	0.080662276					NA
Sapur.003G028500.3	0.049610834	-0.206871031	2.014405672	-0.1027	0.918204396	NA	brown	-0.086109756	0.283574858					hypothetical protein
Sapur.007G036700.2	0.043761731	0.17201802	1.693161191	0.101596	0.919077535	NA	brown	0.030268559	0.706679408		AT4G37180.2		Homeodomain-like superfamily protein	NA
Sapur.004G130400.3	0.050530453	-0.281678926	2.904824395	-0.09697	0.922750737	NA	brown	-0.089724663	0.263772711		AT2G17840.1	ERD7	Senescence/dehydration-associated protein-related	senescence-associated protein
Sapur.018G062300.1	0.04271765	-0.244240546	2.574509591	-0.09487	0.924419066	NA	brown	-0.129232903	0.106719375		AT5G19670.1		Exostosin family protein	exostosin family protein
Sapur.009G004500.1	0.045104939	-0.191667268	2.038808557	-0.09401	0.925101651	NA	brown	-0.150682475	0.059601714		AT3G45050.1			hypothetical protein
Sapur.009G064300.1	0.050301358	-0.17131264	1.8289785	-0.09367	0.92537468	NA	brown	-0.179682836	0.02433571		AT4G17000.1			hypothetical protein
Sapur.006G198300.3	0.040829437	0.158458156	1.700125076	0.093204	0.925741638	NA	brown	0.043959622	0.584601847		AT4G14740.1		Plant protein of unknown function (DUF828) with plant pleckstrin homology-like region	auxin canalisation protein
Sapur.15WG093100.1	0.036849696	-0.169440124	1.875415559	-0.09035	0.928010644	NA	brown	-0.114599155	0.152957804		AT5G13700.1	APAO,ATPAO1,PAO1	polyamine oxidase 1	polyamine oxidase
Sapur.014G031200.2	0.045844743	-0.229126413	2.559577245	-0.08952	0.928670815	NA	brown	-0.098983376	0.2174346					NAC domain protein
Sapur.013G035300.1	0.038733453	0.158530075	1.869605041	0.084793	0.93242568	NA	brown	0.027066286	0.736500524	GO:0030127,GO:0008270,GO:0006888,GO:0006886	AT4G32640.2		Sec23/Sec24 protein transport family protein	protein transporter Sec24-like CEF protein
Sapur.012G003300.1	0.045337693	-0.191660086	2.269516111	-0.08445	0.932698847	NA	brown	-0.12864878	0.108325955	GO:0055114,GO:0020037,GO:0006979,GO:0004601	AT1G05260.1	RCI3,RCI3A	Peroxidase superfamily protein	NA
Sapur.005G162300.1	0.034332251	0.196121984	2.328991635	0.084209	0.932890287	NA	brown	0.068319088	0.395220029	GO:0005515	AT5G45110.1	ATNPR3,NPR3	NPR1-like protein 3	NPR1/NIM1-like regulatory protein, putative
Sapur.008G019500.1	0.046030362	-0.244206808	2.904824395	-0.08407	0.933001264	NA	brown	-0.081608864	0.309590942					hypothetical protein
Sapur.010G063100.1	0.046030362	-0.244206808	2.904824395	-0.08407	0.933001264	NA	brown	-0.081608864	0.309590942		AT3G22600.1		Bifunctional inhibitor/lipid-transfer protein/seed storage 2S albumin superfamily protein	alpha-amylase inhibitors, lipid transfer (LT) and seed storage (SS) family protein
Sapur.014G105300.2	0.047675509	-0.244206808	2.904824395	-0.08407	0.933001264	NA	brown	-0.103892473	0.195363909	GO:0016758,GO:0016021,GO:0005783	AT2G47760.3	ALG3,AtALG3	asparagine-linked glycosylation 3	dolichyl-P-Man:Man(5)GlcNAc(2)-PP-dolichyl mannosyltransferase, putative
Sapur.007G109000.1	0.026597321	0.19798647	2.391796689	0.082777	0.93402862	NA	brown	0.10704722	0.182067211	GO:0055114,GO:0020037,GO:0006979,GO:0004601	AT5G15180.1		Peroxidase superfamily protein	NA
Sapur.T159800.1	0.02610683	0.197987299	2.396844557	0.082603	0.934166968	NA	brown	0.107116019	0.181784833		AT5G24280.1	GMI1	gamma-irradiation and mitomycin c induced 1	NA
Sapur.T079800.1	0.025200979	0.197988865	2.40648488	0.082273	0.93442958	NA	brown	-0.075102413	0.349875031					NA
Sapur.T079900.1	0.025200979	0.197988865	2.40648488	0.082273	0.93442958	NA	brown	-0.074144297	0.356073667					NA
Sapur.018G059300.1	0.019157992	0.235520464	2.904820075	0.081079	0.935378974	NA	brown	-0.076757111	0.339330567					NA
Sapur.010G136200.3	0.045047036	-0.206770695	2.555533233	-0.08091	0.935512749	NA	brown	-0.132554318	0.097936772		AT1G79270.1	ECT8	evolutionarily conserved C-terminal region 8	YTH domain protein
Sapur.001G181400.1	0.04421098	-0.20676997	2.561294706	-0.08073	0.935657719	NA	brown	-0.131697887	0.100144702	GO:0005515	AT1G58025.3		DNA-binding bromodomain-containing protein	bromodomain domain protein
Sapur.003G057000.2	0.043352299	-0.206769213	2.567353563	-0.08054	0.93580947	NA	brown	-0.129355928	0.106383403		AT1G32470.1		Single hybrid motif superfamily protein	glycine cleavage system H protein
Sapur.016G250200.1	0.042108541	-0.206768093	2.576396958	-0.08025	0.936034646	NA	brown	-0.127365428	0.11192224	GO:0008270,GO:0003677	AT3G14890.2		phosphoesterase	polynucleotide phosphatase/kinase
Sapur.013G069300.1	0.040697462	-0.169339106	2.28595327	-0.07408	0.94094823	NA	brown	-0.16165678	0.043104993		AT5G35405.1		Protein of unknown function (DUF784)	prolamin-like protein
Sapur.008G049300.2	0.038851206	-0.169336808	2.2988764	-0.07366	0.941280383	NA	brown	-0.166368575	0.037299575		AT2G39870.1			hypothetical protein
Sapur.010G162000.1	0.045719135	-0.133835536	1.841919108	-0.07266	0.942075948	NA	brown	-0.136549123	0.088142192	GO:0008270,GO:0005515	AT5G17600.1		RING/U-box superfamily protein	RING-H2 finger protein
Sapur.005G161900.1	0.040121719	-0.133827865	1.863684727	-0.07181	0.942754539	NA	brown	-0.13580341	0.089908636		AT1G06660.1			hypothetical protein
Sapur.016G168400.2	0.02219351	0.198036645	2.779507733	0.071249	0.943199728	NA	brown	0.082922749	0.301840023	GO:0005515	AT1G73150.1	GTE3	global transcription factor group E3	global transcription factor group protein
Sapur.001G160300.1	0.037294875	-0.206734691	2.904824395	-0.07117	0.943262913	NA	brown	-0.0977476	0.223259905		AT5G35405.1		Protein of unknown function (DUF784)	NA
Sapur.002G168600.1	0.038747901	-0.206734691	2.904824395	-0.07117	0.943262913	NA	brown	-0.102360415	0.202069824	GO:0005634,GO:0003677	AT2G47070.1	SPL1	squamosa promoter binding protein-like 1	squamosa promoter-binding protein
Sapur.003G090900.1	0.044087877	-0.206734691	2.904824395	-0.07117	0.943262913	NA	brown	-0.125536871	0.117206616	GO:0003677,GO:0005515,GO:0055114,GO:0016706	AT1G63490.1		transcription factor jumonji (jmjC) domain-containing protein	lysine-specific demethylase 5D
Sapur.007G024500.1	0.041875009	-0.206734691	2.904824395	-0.07117	0.943262913	NA	brown	-0.094532293	0.238932118		AT5G49610.1		F-box family protein	F-box protein interaction domain protein
Sapur.010G022800.2	0.040915878	-0.206734691	2.904824395	-0.07117	0.943262913	NA	brown	-0.081608864	0.309590942	GO:0016788	AT1G67830.1	ATFXG1,FXG1	alpha-fucosidase 1	NA
Sapur.016G195300.1	0.04169213	-0.206734691	2.904824395	-0.07117	0.943262913	NA	brown	-0.094186586	0.240661838	GO:0030870,GO:0016887,GO:0006281,GO:0005634,GO:0000723	AT2G31970.1	ATRAD50,RAD50	DNA repair-recombination protein (RAD50)	DNA repair protein Rad50
Sapur.016G229600.2	0.041981016	-0.206734691	2.904824395	-0.07117	0.943262913	NA	brown	-0.094713568	0.238028598	GO:0016020	AT1G30360.1	ERD4	Early-responsive to dehydration stress protein (ERD4)	early-responsive to dehydration protein
Sapur.018G070800.2	0.041738451	-0.206734691	2.904824395	-0.07117	0.943262913	NA	brown	-0.094341346	0.239886431	GO:0048046,GO:0016762,GO:0006073,GO:0005618,GO:0005975,GO:0004553	AT4G25810.1	XTH23,XTR6	xyloglucan endotransglycosylase 6	xyloglucan endotransglycosylase/hydrolase
Sapur.001G091900.3	0.041419064	0.158641516	2.285525766	0.069411	0.944662164	NA	brown	0.04123523	0.608115496	GO:0046983,GO:0003677,GO:0006355,GO:0005634,GO:0003700	AT4G22950.1	AGL19,GL19	AGAMOUS-like 19	MIKC MADS-box transcription factor
Sapur.002G106100.1	0.017109345	0.199900829	2.904821116	0.068817	0.945135353	NA	brown	-0.069932009	0.384131275	GO:0003677	AT4G33280.1		AP2/B3-like transcriptional factor family protein	NA
Sapur.013G031800.1	0.023689878	0.199900829	2.904821116	0.068817	0.945135353	NA	brown	0.078548531	0.32814497					transmembrane protein, putative
Sapur.011G107600.2	0.030480922	0.160508419	2.357555049	0.068083	0.945719901	NA	brown	-0.07619091	0.342915702	GO:0055085,GO:0016021	AT1G20925.1		Auxin efflux carrier family protein	auxin efflux carrier family protein
Sapur.008G137000.1	0.029851483	0.160509369	2.363077031	0.067924	0.945846226	NA	brown	0.049053047	0.541803015	GO:0051861,GO:0046836,GO:0017089,GO:0005737	AT2G33470.2	ATGLTP1,GLTP1	glycolipid transfer protein 1	glycolipid transfer protein, putative
Sapur.016G167800.2	0.047152423	-0.171153045	2.546264209	-0.06722	0.9464087	NA	brown	-0.098235935	0.220944875	GO:0006355,GO:0005634,GO:0003700,GO:0046983,GO:0003677	AT2G14210.1	AGL44,ANR1	AGAMOUS-like 44	MADS-box transcription factor
Sapur.010G197800.1	0.044355202	0.122904024	1.844334891	0.066639	0.94686936	NA	brown	-0.077250676	0.336224854		AT5G22040.2			disease resistance protein, putative
Sapur.012G030400.2	0.04703916	0.12104654	1.839887428	0.06579	0.947544868	NA	brown	0.008163267	0.919177563	GO:0048500,GO:0008312,GO:0006614	AT1G48160.1		signal recognition particle 19 kDa protein, putative / SRP19, putative	signal recognition particle 19 kDa protein
Sapur.007G076100.3	0.037626779	0.122914115	1.871859445	0.065664	0.947645191	NA	brown	0.008846222	0.912441706		AT2G21940.4	ATSK1,SK1	shikimate kinase 1	shikimate kinase
Sapur.001G018000.1	0.037065004	0.122915028	1.874416558	0.065575	0.947716124	NA	brown	-0.018969462	0.813581262	GO:0005515	AT5G13460.1	IQD11	IQ-domain 11	IQ calmodulin-binding motif protein
Sapur.010G171300.2	0.049705714	-0.118951467	1.832260179	-0.06492	0.948237203	NA	brown	-0.105308303	0.189311746		AT3G55005.1	TON1B	tonneau 1b (TON1b)	FGFR1 oncogene partner, putative
Sapur.013G102500.4	0.036573688	-0.169291089	2.616334762	-0.06471	0.948408532	NA	brown	-0.133371676	0.095865662		AT1G35510.1		O-fucosyltransferase family protein	GDP-fucose protein O-fucosyltransferase
Sapur.002G111000.3	0.036525042	-0.116729487	1.876923815	-0.06219	0.950410001	NA	brown	-0.19558544	0.014094267	GO:0098599	AT3G60340.2		alpha/beta-Hydrolases superfamily protein	palmitoyl-protein thioesterase, putative
Sapur.016G059500.2	0.037697024	0.122973927	2.066586053	0.059506	0.952549219	NA	brown	-0.205452028	0.009841966	GO:0008168	AT3G58470.1		nucleic acid binding;methyltransferases	N6-adenine methyltransferase, putative
Sapur.006G150700.2	0.04749933	-0.171115219	2.904823275	-0.05891	0.953025965	NA	brown	-0.07300904	0.363506489	GO:0016021	AT2G24170.1		Endomembrane protein 70 protein family	endomembrane family protein
Sapur.002G200200.1	0.022828408	0.162415905	2.766664251	0.058705	0.953187399	NA	brown	-0.110576635	0.167993087	GO:0006810,GO:0005622	AT5G43960.1		Nuclear transport factor 2 (NTF2) family protein with RNA binding (RRM-RBD-RNP motifs) domain	Ras GTPase-activating protein-binding protein
Sapur.001G070500.2	0.037771266	-0.169262574	2.904824395	-0.05827	0.953533987	NA	brown	-0.114908768	0.151843983		AT5G49330.1	ATMYB111,MYB111,PFG3	myb domain protein 111	R2R3-MYB transcription factor, putative
Sapur.002G046600.3	0.035801393	-0.169262574	2.904824395	-0.05827	0.953533987	NA	brown	-0.081608864	0.309590942		AT3G59090.1			plant/F17J16-140 protein, putative
Sapur.006G027900.1	0.036852113	-0.169262574	2.904824395	-0.05827	0.953533987	NA	brown	-0.096520425	0.229153239		AT5G19400.3	SMG7	Telomerase activating protein Est1	Est1 DNA/RNA-binding domain protein
Sapur.007G052000.1	0.035801393	-0.169262574	2.904824395	-0.05827	0.953533987	NA	brown	-0.081608864	0.309590942	GO:0008168	AT5G66180.1		S-adenosyl-L-methionine-dependent methyltransferases superfamily protein	S-adenosyl-L-methionine-dependent methyltransferase domain protein
Sapur.007G112400.1	0.035062145	-0.169262574	2.904824395	-0.05827	0.953533987	NA	brown	-0.108849011	0.174777731		AT1G05870.3		Protein of unknown function (DUF1685)	DUF1685 family protein
Sapur.010G090100.1	0.039319894	-0.169262574	2.904824395	-0.05827	0.953533987	NA	brown	-0.1179924	0.141082343	GO:0030247,GO:0006468,GO:0004672	AT1G66920.2		Protein kinase superfamily protein	NA
Sapur.010G155000.3	0.037115279	-0.169262574	2.904824395	-0.05827	0.953533987	NA	brown	-0.096996422	0.226854448		AT5G05987.1	PRA1.A2	prenylated RAB acceptor 1.A2	prenylated rab acceptor family protein, putative
Sapur.016G303300.1	0.035801393	-0.169262574	2.904824395	-0.05827	0.953533987	NA	brown	-0.081608864	0.309590942					NA
Sapur.001G082200.2	0.019837975	0.162420949	2.818384948	0.057629	0.954044081	NA	brown	0.076726347	0.339524746	GO:0016757,GO:0016020	AT5G41460.1		Protein of unknown function (DUF604)	transferring glycosyl group transferase, putative
Sapur.001G159100.1	0.038022438	-0.131864251	2.300354783	-0.05732	0.954287548	NA	brown	-0.161746404	0.042987892		AT5G35405.1		Protein of unknown function (DUF784)	NA
Sapur.005G112400.3	0.037944043	-0.131864159	2.300875365	-0.05731	0.954297911	NA	brown	-0.081608864	0.309590942	GO:0019904	AT5G65430.1	14-3-3KAPPA.GF14 KAPPA.G	general regulatory factor 8	14-3-like protein GF14 lambda protein
Sapur.014G027500.1	0.019033682	0.162422428	2.83419082	0.057308	0.954299673	NA	brown	0.073319992	0.361461113	GO:0055114,GO:0020037,GO:0016705,GO:0005506	AT4G31940.1	CYP82C4	cytochrome P450, family 82, subfamily C, polypeptide 4	NA
Sapur.013G069400.1	0.035582977	-0.131861326	2.317194609	-0.05691	0.954620401	NA	brown	-0.156084567	0.050927887		AT5G35405.1		Protein of unknown function (DUF784)	prolamin-like protein
Sapur.013G069700.1	0.035582977	-0.131861326	2.317194609	-0.05691	0.954620401	NA	brown	-0.156084567	0.050927887		AT5G35405.1		Protein of unknown function (DUF784)	prolamin-like protein
Sapur.005G052300.2	0.040594729	-0.116722078	2.051517354	-0.0569	0.95462845	NA	brown	-0.174453707	0.028872997		AT5G09220.1	AAP2	amino acid permease 2	amino acid permease
Sapur.019G015100.1	0.017360128	0.164281247	2.904822183	0.056555	0.954899948	NA	brown	-0.079774479	0.320628304					NA
Sapur.005G126700.2	0.01230166	0.164281247	2.904822183	0.056555	0.954899948	NA	brown	-0.081608864	0.309590942		AT3G28370.1			NA
Sapur.012G083100.2	0.011954249	0.164281246	2.904822183	0.056555	0.954899949	NA	brown	0.009727869	0.903755456		AT5G51050.1		Mitochondrial substrate carrier family protein	substrate carrier family protein
Sapur.006G059300.3	0.017216722	0.162425915	2.872654208	0.056542	0.954909954	NA	brown	0.063809465	0.427224123		AT2G44120.2		Ribosomal protein L30/L7 family protein	60S ribosomal protein L7
Sapur.002G096400.1	0.034171329	0.115341178	2.08952979	0.0552	0.955979459	NA	brown	0.021339384	0.790797424		AT5G67420.1	ASL39,LBD37	LOB domain-containing protein 37	LOB domain protein, putative
Sapur.004G046700.3	0.033145237	0.11534026	2.09591511	0.055031	0.956113784	NA	brown	-0.24320634	0.002146083		AT1G73350.2			hypothetical protein
Sapur.004G156400.4	0.01117195	0.157814843	2.904822379	0.054329	0.956673388	NA	brown	-0.102104766	0.203204799	GO:0032012,GO:0005086	AT4G38200.1		SEC7-like guanine nucleotide exchange family protein	brefeldin A-inhibited guanine nucleotide-exchange protein
Sapur.001G002200.1	0.032223663	0.124886841	2.340203519	0.053366	0.957440451	NA	brown	-0.034476928	0.668163363		AT5G56520.1			hypothetical protein
Sapur.012G085100.1	0.031376299	0.124888073	2.347311269	0.053205	0.957568783	NA	brown	0.04806752	0.549961985	GO:0003676	AT5G65260.1		RNA-binding (RRM/RBD/RNP motifs) family protein	type II polyadenylate-binding family RNA recognition motif protein
Sapur.002G162500.3	0.036572925	-0.154177938	2.904823918	-0.05308	0.957670941	NA	brown	-0.091103876	0.25647262	GO:0006950	AT3G62550.1		Adenine nucleotide alpha hydrolases-like superfamily protein	universal stress family protein
Sapur.002G005700.2	0.027789953	0.124893517	2.379589188	0.052485	0.958141987	NA	brown	-0.077967807	0.331744766		AT1G20696.2	HMGB3,NFD03,NFD3	high mobility group B3	high mobility group box protein
Sapur.017G081100.2	0.027082836	0.124894719	2.386914187	0.052325	0.958269922	NA	brown	0.019915002	0.804471183	GO:0055114,GO:0020037,GO:0016705,GO:0005506	AT3G30180.1	BR6OX2,CYP85A2	brassinosteroid-6-oxidase 2	cytochrome P450 family protein
Sapur.15WG000900.1	0.026893305	0.124895028	2.388807598	0.052283	0.958302865	NA	brown	0.045046272	0.575341486		AT1G18000.1		Major facilitator superfamily protein	UNC93-like protein C922.05c
Sapur.T186200.1	0.026392859	0.124895853	2.393887972	0.052173	0.958391001	NA	brown	0.002289373	0.977298008		AT5G17020.1	ATCRM1.ATXPO1.HIT2.XPO	exportin 1A	NA
Sapur.002G162500.2	0.041601988	-0.133676754	2.578206885	-0.05185	0.958649226	NA	brown	-0.114721026	0.152518644	GO:0006950	AT3G62550.1		Adenine nucleotide alpha hydrolases-like superfamily protein	universal stress family protein
Sapur.010G093200.1	0.041167071	-0.133676345	2.581496412	-0.05178	0.958701998	NA	brown	-0.109776359	0.171111139	GO:0008270,GO:0005515	AT3G18290.1	BTS,EMB2454	zinc finger protein-related	zinc finger protein, putative
Sapur.003G156600.5	0.034037904	-0.131816809	2.634301827	-0.05004	0.960091618	NA	brown	-0.154011695	0.054123565	GO:0046872,GO:0000166	AT2G41560.1	ACA4	autoinhibited Ca(2+)-ATPase, isoform 4	calcium-transporting ATPase 4, plasma membrane-type protein
Sapur.002G034100.2	0.033712661	-0.131816451	2.637451151	-0.04998	0.960139341	NA	brown	-0.157527654	0.048796813	GO:0006355,GO:0005634	AT5G43700.1	ATAUX2-11,IAA4	AUX/IAA transcriptional regulator family protein	indole-3-acetic acid inducible protein
Sapur.017G028800.3	0.033476828	-0.13181604	2.641074175	-0.04991	0.9601941	NA	brown	-0.148484694	0.06346179	GO:0005515	AT3G21820.1	ATXR2,SDG36	histone-lysine N-methyltransferase ATXR2	histone-lysine N-methyltransferase ATXR2
Sapur.006G179100.2	0.031872994	-0.131814221	2.657320446	-0.0496	0.96043781	NA	brown	-0.145548298	0.068935915	GO:0005515	AT2G45700.1		sterile alpha motif (SAM) domain-containing protein	DNA cross-link repair 1A protein
Sapur.008G021700.1	0.031689064	-0.131814007	2.659248483	-0.04957	0.960466534	NA	brown	-0.145294833	0.069425915					NA
Sapur.019G055100.1	0.006480264	0.143384081	2.904822821	0.049361	0.960631849	NA	brown	0.091897757	0.252334244					NA
Sapur.018G105100.1	0.007094491	0.143384081	2.904822821	0.049361	0.960631849	NA	brown	0.090817653	0.257976035	GO:0055114,GO:0020037,GO:0016705,GO:0005506	AT5G36110.1	CYP716A1	cytochrome P450, family 716, subfamily A, polypeptide 1	cytochrome P450 family 716 protein
Sapur.T129000.1	0.008986688	0.143384081	2.904822821	0.049361	0.960631849	NA	brown	-0.074820339	0.351692855					NA
Sapur.T054400.1	0.049792282	-0.135495692	2.904822183	-0.04665	0.962796094	NA	brown	-0.061867584	0.441453141					NA
Sapur.010G070900.1	0.028791823	0.124935376	2.687671098	0.046485	0.96292399	NA	brown	-0.088817185	0.268652634	GO:0005515,GO:0006468,GO:0004672	AT2G01820.1		Leucine-rich repeat protein kinase family protein	receptor kinase, putative
Sapur.019G100300.2	0.038658926	0.085441024	1.869931888	0.045692	0.963555699	NA	brown	-0.020391872	0.799886506		AT4G03320.1	tic20-IV	translocon at the inner envelope membrane of chloroplasts 20-IV	Tic20-IV, putative
Sapur.002G131100.3	0.034843087	-0.131790456	2.904824395	-0.04537	0.963812784	NA	brown	-0.136641527	0.087925249	GO:0016787,GO:0005524,GO:0003677,GO:0006355,GO:0005634	AT2G46020.2	ATBRM,BRM,CHA2,CHR2	transcription regulatory protein SNF2, putative	ATP-dependent helicase BRM
Sapur.006G207800.7	0.032291271	-0.131790456	2.904824395	-0.04537	0.963812784	NA	brown	-0.084530771	0.292529477		AT3G63400.3		Cyclophilin-like peptidyl-prolyl cis-trans isomerase family protein	peptidyl-prolyl cis-trans isomerase, cyclophilin-type protein
Sapur.007G102300.3	0.030686908	-0.131790456	2.904824395	-0.04537	0.963812784	NA	brown	-0.081608864	0.309590942		AT5G09520.1		hydroxyproline-rich glycoprotein family protein	transmembrane protein, putative
Sapur.010G071500.1	0.031758162	-0.131790456	2.904824395	-0.04537	0.963812784	NA	brown	-0.098905052	0.217800562	GO:0048046,GO:0016762,GO:0006073,GO:0005618,GO:0005975,GO:0004553	AT1G14720.1	ATXTH28.EXGT-A2.XTH28.X	xyloglucan endotransglucosylase/hydrolase 28	xyloglucan endotransglucosylase/hydrolase, putative
Sapur.010G197400.1	0.030664735	-0.131790456	2.904824395	-0.04537	0.963812784	NA	brown	-0.096488186	0.229309525	GO:0008253,GO:0005737,GO:0000287	AT2G38680.1		5\'-nucleotidases;magnesium ion binding	cytosolic 5'-nucleotidase III-like protein
Sapur.011G048200.1	0.028876407	-0.131790456	2.904824395	-0.04537	0.963812784	NA	brown	-0.091928207	0.252176439	GO:0006468,GO:0005524,GO:0004672	AT4G21410.1	CRK29	cysteine-rich RLK (RECEPTOR-like protein kinase) 29	tyrosine kinase domain protein
Sapur.011G082600.4	0.03424425	-0.131790456	2.904824395	-0.04537	0.963812784	NA	brown	-0.136935803	0.087237197	GO:0016020,GO:0008375	AT3G15350.1		Core-2/I-branching beta-1,6-N-acetylglucosaminyltransferase family protein	core-2/I-branching beta-1%2C6-N-acetylglucosaminyltransferase family protein, putative
Sapur.013G112000.1	0.030686908	-0.131790456	2.904824395	-0.04537	0.963812784	NA	brown	-0.081608864	0.309590942		AT1G28520.1	ATVOZ1,VOZ1	vascular plant one zinc finger protein	transcription factor VOZ1
Sapur.014G013800.4	0.030686908	-0.131790456	2.904824395	-0.04537	0.963812784	NA	brown	-0.081608864	0.309590942		AT1G60060.1		Serine/threonine-protein kinase WNK (With No Lysine)-related	bHLH-MYC/R2R3-MYB transcription factor amino-terminal protein
Sapur.014G103100.1	0.030686908	-0.131790456	2.904824395	-0.04537	0.963812784	NA	brown	-0.081608864	0.309590942		AT3G62860.1		alpha/beta-Hydrolases superfamily protein	monoglyceride lipase
Sapur.016G058500.2	0.031772376	-0.131790456	2.904824395	-0.04537	0.963812784	NA	brown	-0.098763117	0.218464867	GO:0003676	AT1G55310.3	ATSCL33,SCL33,SR33	SC35-like splicing factor 33	serine/arginine-rich splicing factor 10 (SRSF10)-like RNA recognition motif protein
Sapur.T129100.1	0.030686908	-0.131790456	2.904824395	-0.04537	0.963812784	NA	brown	-0.081608864	0.309590942					NA
Sapur.009G130600.3	0.021200635	0.124946679	2.796283891	0.044683	0.964359891	NA	brown	0.005207813	0.948386828	GO:0016020,GO:0008158	AT4G38350.1		Patched family protein	niemann-pick C type family protein
Sapur.018G067300.2	0.014918953	0.128661719	2.904823276	0.044292	0.964671295	NA	brown	-0.206507948	0.009462058	GO:0006468,GO:0005524,GO:0004672	AT2G18890.1		Protein kinase superfamily protein	receptor-like kinase
Sapur.005G206200.2	0.010741554	0.128661719	2.904823276	0.044292	0.964671295	NA	brown	-0.081608864	0.309590942	GO:0003677	AT1G76510.1		ARID/BRIGHT DNA-binding domain-containing protein	ARID/bright DNA-binding domain protein
Sapur.012G036300.1	0.007980969	0.128661719	2.904823276	0.044292	0.964671295	NA	brown	0.078548531	0.32814497					hypothetical protein
Sapur.014G032800.3	0.00778933	0.128661719	2.904823276	0.044292	0.964671295	NA	brown	0.078548531	0.32814497		AT2G38740.1		Haloacid dehalogenase-like hydrolase (HAD) superfamily protein	haloacid dehalogenase-like hydrolase family protein
Sapur.005G051800.5	0.00734023	0.128661719	2.904823276	0.044292	0.964671295	NA	brown	0.001702542	0.983116162	GO:0070403	AT5G09230.7	AtSRT2,SRT2	sirtuin 2	NAD-dependent protein deacetylase
Sapur.008G038200.2	0.006454126	0.128661719	2.904823276	0.044292	0.964671295	NA	brown	0.078548531	0.32814497	GO:0016021,GO:0015116,GO:0008272,GO:0055085,GO:0016020,GO:0008271	AT5G13550.1	SULTR4;1	sulfate transporter 4.1	sulfate/bicarbonate/oxalate exchanger and transporter sat-1, putative
Sapur.010G043200.2	0.006454126	0.128661719	2.904823276	0.044292	0.964671295	NA	brown	0.078548531	0.32814497		AT4G28600.1	NPGR2	no pollen germination related 2	O-linked N-acetylglucosamine transferase, ogt protein%2C putative
Sapur.002G159200.1	0.006449611	0.128661719	2.904823276	0.044292	0.964671295	NA	brown	0.078548531	0.32814497		AT2G47540.1		Pollen Ole e 1 allergen and extensin family protein	pollen protein Ole E I-like protein
Sapur.007G018800.2	0.006449611	0.128661719	2.904823276	0.044292	0.964671295	NA	brown	0.078548531	0.32814497	GO:0008168	AT4G36470.1		S-adenosyl-L-methionine-dependent methyltransferases superfamily protein	S-adenosyl-L-methionine:jasmonic acid carboxyl methyltransferase, putative
Sapur.002G084200.1	0.00611461	0.128661719	2.904823276	0.044292	0.964671295	NA	brown	0.078548531	0.32814497		AT4G24560.1	UBP16	ubiquitin-specific protease 16	ubiquitin carboxyl-terminal hydrolase
Sapur.004G158000.1	0.00611461	0.128661719	2.904823276	0.044292	0.964671295	NA	brown	0.078548531	0.32814497		AT2G17390.1	AKR2B	ankyrin repeat-containing 2B	TGB12K interacting protein
Sapur.003G051100.1	0.006088772	0.128661719	2.904823276	0.044292	0.964671295	NA	brown	0.078548531	0.32814497	GO:0006468,GO:0005524,GO:0004672	AT4G16970.1		Protein kinase superfamily protein	cyclin-dependent kinase-like Serine/Threonine kinase family protein, putative
Sapur.008G070900.3	0.006088772	0.128661719	2.904823276	0.044292	0.964671295	NA	brown	0.078548531	0.32814497	GO:0015934,GO:0006412,GO:0003735	AT1G26740.1		Ribosomal L32p protein family	ribosomal protein L32
Sapur.010G090100.2	0.006088772	0.128661719	2.904823276	0.044292	0.964671295	NA	brown	0.078548531	0.32814497	GO:0030247,GO:0006468,GO:0004672	AT1G66920.2		Protein kinase superfamily protein	NA
Sapur.010G108100.1	0.006088772	0.128661719	2.904823276	0.044292	0.964671295	NA	brown	0.078548531	0.32814497		AT2G03810.1		18S pre-ribosomal assembly protein gar2-related	hypothetical protein
Sapur.014G017500.1	0.006088772	0.128661719	2.904823276	0.044292	0.964671295	NA	brown	0.078548531	0.32814497					NA
Sapur.016G181100.1	0.006088772	0.128661719	2.904823276	0.044292	0.964671295	NA	brown	0.078548531	0.32814497	GO:0019441,GO:0004061	AT4G35220.1		Cyclase family protein	cyclase family protein
Sapur.003G071700.2	0.005967487	0.128661719	2.904823276	0.044292	0.964671295	NA	brown	0.078548531	0.32814497		AT1G31500.1		DNAse I-like superfamily protein	carbon catabolite repressor-like protein
Sapur.014G003000.2	0.005967487	0.128661719	2.904823276	0.044292	0.964671295	NA	brown	0.078548531	0.32814497	GO:0016757,GO:0016021	AT2G22900.1		Galactosyl transferase GMA12/MNN10 family protein	alpha galactosyltransferase, putative
Sapur.009G058300.1	0.005789239	0.128661719	2.904823276	0.044292	0.964671295	NA	brown	0.078548531	0.32814497		AT5G58560.1		Phosphatidate cytidylyltransferase family protein	phytol kinase, putative
Sapur.010G139100.3	0.005789239	0.128661719	2.904823276	0.044292	0.964671295	NA	brown	0.078548531	0.32814497	GO:0003676	AT5G16260.1	ELF9	RNA binding (RRM/RBD/RNP motifs) family protein	splicing factor U2AF 35 kDa subunit B RNA recognition motif protein, putative
Sapur.017G128200.2	0.005659149	0.128661719	2.904823276	0.044292	0.964671295	NA	brown	-0.00072186	0.992840993		AT1G73390.1		Endosomal targeting BRO1-like domain-containing protein	BRO1-like domain protein
Sapur.001G135500.1	0.005637254	0.128661719	2.904823276	0.044292	0.964671295	NA	brown	0.078548531	0.32814497					NA
Sapur.008G131400.1	0.005637254	0.128661719	2.904823276	0.044292	0.964671295	NA	brown	0.078548531	0.32814497	GO:0003677	AT3G42170.1		BED zinc finger ;hAT family dimerisation domain	NA
Sapur.012G028200.1	0.005637254	0.128661719	2.904823276	0.044292	0.964671295	NA	brown	0.078548531	0.32814497	GO:0055114,GO:0020037,GO:0006979,GO:0004601	AT1G05260.1	RCI3,RCI3A	Peroxidase superfamily protein	cationic peroxidase kDa protein
Sapur.014G094400.1	0.005637254	0.128661719	2.904823276	0.044292	0.964671295	NA	brown	0.078548531	0.32814497		AT1G02380.1			transmembrane protein, putative
Sapur.016G118500.1	0.005637254	0.128661719	2.904823276	0.044292	0.964671295	NA	brown	0.078548531	0.32814497					NA
Sapur.016G302900.1	0.005637254	0.128661719	2.904823276	0.044292	0.964671295	NA	brown	-0.078477539	0.328583686		AT4G03500.1		Ankyrin repeat family protein	ankyrin repeat protein
Sapur.017G053400.1	0.005637254	0.128661719	2.904823276	0.044292	0.964671295	NA	brown	0.078548531	0.32814497					NA
Sapur.001G010500.1	0.005474546	0.128661719	2.904823276	0.044292	0.964671295	NA	brown	0.078548531	0.32814497	GO:0016020,GO:0006810,GO:0005215	AT5G19640.1		Major facilitator superfamily protein	peptide transporter PTR1
Sapur.005G118800.2	0.005474546	0.128661719	2.904823276	0.044292	0.964671295	NA	brown	0.078548531	0.32814497		AT4G38040.1		Exostosin family protein	exostosin family protein
Sapur.007G044300.2	0.005474546	0.128661719	2.904823276	0.044292	0.964671295	NA	brown	0.078548531	0.32814497	GO:0055114,GO:0016491	AT3G50210.1		2-oxoglutarate (2OG) and Fe(II)-dependent oxygenase superfamily protein	2-oxoglutarate-Fe(II)-dependent oxygenase domain protein
Sapur.008G019400.2	0.005474546	0.128661719	2.904823276	0.044292	0.964671295	NA	brown	0.078548531	0.32814497					hypothetical protein
Sapur.008G048800.1	0.005474546	0.128661719	2.904823276	0.044292	0.964671295	NA	brown	0.078548531	0.32814497					hypothetical protein
Sapur.009G044400.1	0.005474546	0.128661719	2.904823276	0.044292	0.964671295	NA	brown	0.078548531	0.32814497		AT5G13690.1	CYL1,NAGLU	alpha-N-acetylglucosaminidase family / NAGLU family	alpha-N-acetylglucosaminidase
Sapur.011G112300.3	0.005474546	0.128661719	2.904823276	0.044292	0.964671295	NA	brown	-0.081608864	0.309590942		AT3G15850.1	ADS3,FAD5,FADB,JB67	fatty acid desaturase 5	palmitoyl-monogalactosyldiacylglycerol delta-7 desaturase, putative
Sapur.009G097200.2	0.005161387	0.128661719	2.904823276	0.044292	0.964671295	NA	brown	-0.108970111	0.174295651	GO:0003723	AT2G21350.1		RNA-binding CRS1 / YhbY (CRM) domain protein	NA
Sapur.003G009500.2	0.004974636	0.128661719	2.904823276	0.044292	0.964671295	NA	brown	-0.144103906	0.071766275	GO:0005622,GO:0003676,GO:0005524,GO:0043140,GO:0006281,GO:0006260	AT1G10930.1	ATRECQ4A.ATSGS1.RECQ4A	DNA helicase (RECQl4A)	ATP-dependent DNA helicase
Sapur.004G147500.1	0.004974636	0.128661719	2.904823276	0.044292	0.964671295	NA	brown	0.078548531	0.32814497	GO:0005515	AT3G62020.2	GLP10	germin-like protein 10	NA
Sapur.005G140800.1	0.004974636	0.128661719	2.904823276	0.044292	0.964671295	NA	brown	0.078548531	0.32814497		AT3G49600.1	ATUBP26,SUP32,UBP26	ubiquitin-specific protease 26	ubiquitin carboxyl-terminal hydrolase
Sapur.008G017800.2	0.004974636	0.128661719	2.904823276	0.044292	0.964671295	NA	brown	0.078548531	0.32814497		AT5G03760.1	ATCSLA09.ATCSLA9.CSLA09	Nucleotide-diphospho-sugar transferases superfamily protein	cellulose synthase-like protein
Sapur.012G067100.4	0.004974636	0.128661719	2.904823276	0.044292	0.964671295	NA	brown	0.078548531	0.32814497	GO:0006464,GO:0004719	AT5G50240.1	PIMT2	protein-l-isoaspartate methyltransferase 2	L-isoaspartate O-methyltransferase
Sapur.014G008100.1	0.004974636	0.128661719	2.904823276	0.044292	0.964671295	NA	brown	0.078548531	0.32814497	GO:0005515	AT1G27752.1		Ubiquitin system component Cue protein	ubiquitin system component CUE protein
Sapur.15WG124300.3	0.004974636	0.128661719	2.904823276	0.044292	0.964671295	NA	brown	0.078548531	0.32814497		AT4G21340.1	B70	basic helix-loop-helix (bHLH) DNA-binding superfamily protein	NA
Sapur.T174200.2	0.004974636	0.128661719	2.904823276	0.044292	0.964671295	NA	brown	0.078548531	0.32814497		AT3G24880.1		Helicase/SANT-associated, DNA binding protein	hypothetical protein
Sapur.016G100400.7	0.004856032	0.128661719	2.904823276	0.044292	0.964671295	NA	brown	-0.081608864	0.309590942		AT5G01520.1		RING/U-box superfamily protein	RING finger protein
Sapur.016G112100.3	0.004438202	0.128661719	2.904823276	0.044292	0.964671295	NA	brown	-0.109902748	0.170615866	GO:0034755,GO:0016021,GO:0005381	AT5G03570.1	ATIREG2,FPN2,IREG2	iron regulated 2	solute carrier family 40 protein
Sapur.005G179800.1	0.004340041	0.128661719	2.904823276	0.044292	0.964671295	NA	brown	-0.16128342	0.043595696	GO:0016567,GO:0004842,GO:0005515	AT3G54790.1		ARM repeat superfamily protein	ubiquitin-protein ligase, PUB2
Sapur.006G176300.5	0.004340041	0.128661719	2.904823276	0.044292	0.964671295	NA	brown	-0.15489623	0.052740196	GO:0008270,GO:0003676	AT3G53500.2	RSZ32	RNA-binding (RRM/RBD/RNP motifs) family protein with retrovirus zinc finger-like domain	RNA recognition motif, a.k.a. RRM%2C RBD protein

Sapur.010G128600.4	0.003883051	0.128661719	2.904823276	0.044292	0.964671296	NA	brown	-0.081608864	0.309590942		AT5G64130.1		cAMP-regulated phosphoprotein 19-related protein	NA
Sapur.001G167300.2	0.00377543	0.128661719	2.904823276	0.044292	0.964671296	NA	brown	0.05320448	0.508104592		AT1G51710.1	ATUBP6,UBP6	ubiquitin-specific protease 6	ubiquitin carboxyl-terminal hydrolase
Sapur.005G153100.2	0.003577442	0.128661719	2.904823276	0.044292	0.964671296	NA	brown	-0.157663184	0.048600536		AT1G76730.1		NagB/RpiA/CoA transferase-like superfamily protein	NA
Sapur.006G055800.2	0.002869527	0.128661719	2.904823276	0.044292	0.964671296	NA	brown	-0.016056727	0.841797072		AT3G15760.1			transmembrane protein, putative
Sapur.016G245700.3	0.002731927	0.128661719	2.904823276	0.044292	0.964671296	NA	brown	-0.010073523	0.900353013	GO:0006468,GO:0004672	AT1G53440.1		Leucine-rich repeat transmembrane protein kinase	LRR receptor-like Serine/Threonine-kinase, putative
Sapur.15WG110600.5	0.044067065	0.12680913	2.904822183	0.043655	0.965179652	NA	brown	-0.040502715	0.614508158	GO:0005515,GO:0006468,GO:0004672	AT5G14210.1		Leucine-rich repeat protein kinase family protein	LRR receptor-like kinase
Sapur.012G036800.6	0.027415712	0.12680913	2.904822183	0.043655	0.965179652	NA	brown	-0.061017889	0.447762781		AT1G15060.1		Uncharacterised conserved protein UCP031088, alpha/beta hydrolase	alpha/beta hydrolase family protein
Sapur.009G130100.2	0.017462463	0.126809129	2.904822183	0.043655	0.965179652	NA	brown	0.056491496	0.482216866	GO:0005515	AT2G17200.1	DSK2	ubiquitin family protein	ubiquitin domain protein
Sapur.002G006600.1	0.015664048	0.126809129	2.904822183	0.043655	0.965179652	NA	brown	0.003446978	0.965824693	GO:0005515	AT2G30600.1		BTB/POZ domain-containing protein	BTB/POZ domain protein
Sapur.005G167400.3	0.016851209	0.126809129	2.904822183	0.043655	0.965179652	NA	brown	0.03785685	0.637838393	GO:0005515	AT3G63220.2		Galactose oxidase/kelch repeat superfamily protein	F-box/kelch-repeat protein SKIP30
Sapur.005G180600.1	0.015996415	0.126809129	2.904822183	0.043655	0.965179652	NA	brown	0.04742922	0.555278126	GO:0072588,GO:0042254,GO:0030515,GO:0001522	AT2G20490.1	EDA27,NOP10	nucleolar RNA-binding Nop10p family protein	H/ACA ribonucleoprotein complex subunit-like protein
Sapur.014G137400.1	0.015657108	0.126809129	2.904822183	0.043655	0.965179652	NA	brown	0.047572789	0.554080231	GO:0016747,GO:0008152	AT5G48230.2	ACAT2,EMB1276	acetoacetyl-CoA thiolase 2	NA
Sapur.017G103600.1	0.014698426	0.126809129	2.904822183	0.043655	0.965179652	NA	brown	0.04260577	0.596234376		AT1G70780.1			NA
Sapur.003G089700.2	0.014612884	0.126809129	2.904822183	0.043655	0.965179652	NA	brown	0.040535123	0.61422472	GO:0006468,GO:0004672	AT1G63430.2		Leucine-rich repeat protein kinase family protein	LRR receptor-like kinase
Sapur.008G022700.2	0.015112397	0.126809129	2.904822183	0.043655	0.965179652	NA	brown	0.043440318	0.589051433		AT5G04410.1	anac078,NAC2	NAC domain containing protein 2	NAC transcription factor
Sapur.009G085300.2	0.019932624	0.126809129	2.904822183	0.043655	0.965179652	NA	brown	0.048818951	0.543735617	GO:0003677	AT3G18990.1	REM39,VRN1	AP2/B3-like transcriptional factor family protein	B3 domain transcription factor VRN1
Sapur.013G098800.1	0.047679246	-0.098108135	2.257640023	-0.04346	0.965337992	NA	brown	-0.089277238	0.266171101	GO:0055114,GO:0016616,GO:0006694,GO:0003854	AT1G75280.1		NmrA-like negative transcriptional regulator family protein	phenylcoumaran benzylic ether reductase-like protein
Sapur.004G096700.2	0.044220524	0.087347062	2.031720158	0.042992	0.965708166	NA	brown	-0.077994376	0.331579518		AT5G16110.1			hypothetical protein
Sapur.006G087000.1	0.044707994	-0.09625272	2.264150489	-0.04251	0.966090843	NA	brown	-0.117209794	0.143756961		AT2G38420.1		Pentatricopeptide repeat (PPR) superfamily protein	PPR containing plant protein
Sapur.008G019200.1	0.021518901	0.103698156	2.450252727	0.042321	0.966242476	NA	brown	-0.018631208	0.816846408					hypothetical protein
Sapur.009G086500.1	0.03652975	0.087357304	2.070419341	0.042193	0.966344804	NA	brown	0.017346685	0.829274084		AT3G50830.1	ATCOR413-PM2.COR413-PI	cold-regulated 413-plasma membrane 2	cold acclimation protein COR413-PM1
Sapur.018G046300.1	0.042123546	0.085496265	2.046696739	0.041773	0.966679814	NA	brown	-0.03346279	0.677370824	GO:0003677	AT3G43240.1		ARID/BRIGHT DNA-binding domain-containing protein	ARID/bright DNA-binding domain protein
Sapur.010G167500.2	0.037694866	-0.09624499	2.30648289	-0.04173	0.966715508	NA	brown	-0.13006725	0.104457081	GO:0005975,GO:0004553	AT3G55260.1	ATHEX2,HEXO1	beta-hexosaminidase 1	beta-hexosaminidase
Sapur.014G000300.1	0.037105418	-0.096244285	2.310470774	-0.04166	0.966773167	NA	brown	-0.120413839	0.133046593	GO:0005515.GO:0008270.GO:0006355.GO:0005634.GO:0004402.GO:0003712.GO:001657	AT1G16710.1	HAC12	histone acetyltransferase of the CBP family 12	histone acetyltransferase HAC12
Sapur.007G026000.2	0.030494107	0.087366765	2.108313435	0.041439	0.966945784	NA	brown	-0.390499906	4.28E-07	GO:0045735	AT4G36700.1		RmlC-like cupins superfamily protein	cupin protein
Sapur.T186600.1	0.034363118	-0.094387953	2.323526839	-0.04062	0.96759668	NA	brown	-0.17793585	0.025778253					NA
Sapur.009G097900.1	0.043239251	-0.10446583	2.575406358	-0.04056	0.967644399	NA	brown	-0.080233326	0.317843859	GO:0043565,GO:0008270,GO:0006355,GO:0003700	AT5G66320.1	GATA5	GATA transcription factor 5	GATA transcription factor
Sapur.004G062000.3	0.048441709	-0.08146922	2.019016724	-0.04035	0.967813344	NA	brown	-0.09005458	0.262013698		AT1G23890.1		NHL domain-containing protein	NHL domain protein, putative
Sapur.016G137200.1	0.032245877	-0.09438518	2.340027568	-0.04034	0.967825993	NA	brown	-0.18291329	0.021850183	GO:0017025,GO:0008270,GO:0006355,GO:0006352,GO:0006384,GO:0000126	AT3G09360.1		Cyclin/Brf1-like TBP-binding protein	transcription factor, putative
Sapur.008G005700.3	0.031167864	-0.116705819	2.904823918	-0.04018	0.967952369	NA	brown	-0.156367673	0.050503831		AT1G16880.1		uridylyltransferase-related	DS12 from 2D-PAGE of leaf protein, putative
Sapur.013G044800.1	0.028530913	-0.094379897	2.372521763	-0.03978	0.968268191	NA	brown	-0.183803844	0.021204707	GO:0034968,GO:0018024,GO:0008270,GO:0005634,GO:0005515	AT3G04380.2	SDG31,SUVR4	SET-domain containing protein lysine methyltransferase family protein	histone-lysine N-methyltransferase SUVR4, putative
Sapur.004G160100.2	0.028075487	-0.094379212	2.376841188	-0.03971	0.968326057	NA	brown	-0.183659699	0.021308054		AT2G28305.1	ATLOG1,LOG1	Putative lysine decarboxylase family protein	cytokinin riboside 5'-monophosphate phosphoribohydrolase LOG1
Sapur.013G137800.2	0.04754522	0.087396877	2.245296795	0.038924	0.968950646	NA	brown	0.025615566	0.75014396		AT1G16870.1		mitochondrial 28S ribosomal protein S29-related	28S ribosomal protein S29
Sapur.016G080100.1	0.048565699	-0.098063102	2.537326538	-0.03865	0.969170873	NA	brown	-0.065882186	0.41233246		AT5G02790.1	GSTL3	Glutathione S-transferase family protein	NA
Sapur.005G070500.1	0.04670293	-0.098061533	2.549174474	-0.03847	0.96931458	NA	brown	-0.070357031	0.381240962	GO:0016021,GO:0006481,GO:0004671	AT5G08335.1	ATICMTB,ATSTE14B,ICMTB	Isoprenylcysteine carboxyl methyltransferase (ICMT) family	S-isoprenylcysteine O-methyltransferase
Sapur.T114100.1	0.022494034	0.103704107	2.774616817	0.037376	0.9701852	NA	brown	0.011471894	0.886607323		AT4G34990.1	AtMYB32,MYB32	myb domain protein 32	NA
Sapur.005G176500.6	0.001005719	0.108126894	2.904823918	0.037223	0.970307026	NA	brown	-0.163772258	0.040411148	GO:0006629	AT2G42450.1		alpha/beta-Hydrolases superfamily protein	NA
Sapur.008G105000.2	0.001005719	0.108126894	2.904823918	0.037223	0.970307026	NA	brown	0.008772239	0.913171092		AT1G14990.2			transmembrane protein, putative
Sapur.016G075100.2	0.001005719	0.108126894	2.904823918	0.037223	0.970307026	NA	brown	-0.057035529	0.478001764		AT3G55140.1		Pectin lyase-like superfamily protein	pectate lyase
Sapur.007G130400.1	0.035479215	-0.096198741	2.626187088	-0.03663	0.970779565	NA	brown	-0.101944723	0.203917658		AT2G44100.1	AT-GDI1,ATGDI1,GDI1	guanosine nucleotide diphosphate dissociation inhibitor 1	Rab GDP dissociation inhibitor
Sapur.010G077600.2	0.035170928	-0.096198409	2.629030159	-0.03659	0.970811251	NA	brown	-0.103552652	0.196837198	GO:0005634	AT1G70210.1	ATCYCD1;1,CYCD1;1	CYCLIN D1;1	cyclin-D1-1 protein
Sapur.011G054900.6	0.019145878	0.103704869	2.834364291	0.036588	0.970813189	NA	brown	-0.114798493	0.152239988	GO:0016020	AT5G46150.1		LEM3 (ligand-effect modulator 3) family protein / CDC50 family protein	LEM3/CDC50 family protein
Sapur.001G028000.2	0.034680147	-0.096197874	2.633620018	-0.03653	0.97086226	NA	brown	-0.103521761	0.196971521	GO:0006355,GO:0005739,GO:0003690	AT5G07900.1		Mitochondrial transcription termination factor family protein	mTERF protein
Sapur.002G164500.3	0.009590219	0.105911962	2.904822821	0.036461	0.97091499	NA	brown	-0.008419291	0.91665175	GO:0006396,GO:0004526	AT2G47300.2		ribonuclease Ps	ribonuclease P/MRP protein subunit POP1, putative
Sapur.006G095500.1	0.010584001	0.105911962	2.904822821	0.036461	0.97091499	NA	brown	0.005081244	0.949639547	GO:0046872,GO:0005634,GO:0003723	AT5G42820.1	ATU2AF35B,U2AF35B	Zinc finger C-x8-C-x5-C-x3-H type family protein	splicing factor U2af subunit
Sapur.004G135800.1	0.012569815	0.105911962	2.904822821	0.036461	0.97091499	NA	brown	0.022416021	0.780503861		AT5G39130.1		RmlC-like cupins superfamily protein	rhicadhesin receptor, putative
Sapur.003G023600.2	0.01343399	0.105911962	2.904822821	0.036461	0.97091499	NA	brown	0.027541209	0.732051634	GO:0006334,GO:0005634	AT2G19480.3	NAP1;2,NFA02,NFA2	nucleosome assembly protein 1;2	NA
Sapur.002G173400.1	0.030776856	-0.094341169	2.665102049	-0.0354	0.971761815	NA	brown	-0.162061789	0.04257793		AT1G05000.1		Phosphotyrosine protein phosphatases superfamily protein	tyrosine phosphatase family protein, putative
Sapur.004G138800.3	0.028199644	-0.094337976	2.694786846	-0.03501	0.972073694	NA	brown	-0.159062696	0.046611854	GO:0009378,GO:0006310,GO:0006281	AT2G27600.1	ATSKD1,SKD1,VPS4	AAA-type ATPase family protein	vacuolar sorting protein 4b
Sapur.008G005700.4	0.025615205	-0.094334519	2.728164172	-0.03458	0.972416227	NA	brown	-0.091991429	0.251849008		AT1G16880.1		uridylyltransferase-related	DS12 from 2D-PAGE of leaf protein, putative
Sapur.15WG106900.2	0.032067966	0.080369697	2.346707021	0.034248	0.972679501	NA	brown	-0.141657552	0.076774537	GO:0016020,GO:0006075,GO:0003843,GO:0000148	AT2G36850.1	ATGSL08.ATGSL8.CHOR.GSL	glucan synthase-like 8	1%2C3-beta-glucan synthase
Sapur.016G056100.2	0.049559367	-0.062604756	1.837168315	-0.03408	0.972815935	NA	brown	-0.076509582	0.340894959	GO:0045454,GO:0015035,GO:0006662	AT5G06690.2	WCRKC1	WCRKC thioredoxin 1	thioredoxin-like protein
Sapur.016G228600.1	0.050155235	-0.060752514	1.826800674	-0.03326	0.973470253	NA	brown	-0.101608867	0.205419477	GO:0005515	AT1G30440.1		Phototropic-responsive NPH3 family protein	phototropic-responsive NPH3 family protein
Sapur.006G153400.1	0.033418836	-0.096170985	2.904823276	-0.03311	0.973588986	NA	brown	-0.074061582	0.356611991	GO:0000160	AT2G25180.1	ARR12,RR12	response regulator 12	type-B response regulator
Sapur.15WG027300.1	0.034156924	-0.096170985	2.904823276	-0.03311	0.973588986	NA	brown	-0.072035438	0.369956897	GO:0008270,GO:0006355,GO:0005634,GO:0003700	AT1G10170.1	ATNFXL1,NFXL1	NF-X-like 1	NA
Sapur.013G088100.2	0.046440166	-0.060747864	1.839346773	-0.03303	0.973653161	NA	brown	-0.113329359	0.157590298		AT1G65230.1		Uncharacterized conserved protein (DUF2358)	thioredoxin F, putative
Sapur.018G091900.1	0.02521435	0.089320755	2.731109885	0.032705	0.973909893	NA	brown	0.025822957	0.748188673	GO:0031515,GO:0030488,GO:0016429	AT5G14600.1		S-adenosyl-L-methionine-dependent methyltransferases superfamily protein	NA
Sapur.002G101600.4	0.025572424	-0.094318339	2.904824395	-0.03247	0.974097599	NA	brown	-0.081608864	0.309590942		AT1G45688.1			late embryogenesis abundant protein
Sapur.002G193200.3	0.02501269	-0.094318339	2.904824395	-0.03247	0.974097599	NA	brown	-0.123045121	0.124717357		AT3G07370.1	ATCHIP,CHIP	carboxyl terminus of HSC70-interacting protein	NA
Sapur.002G201500.2	0.026365264	-0.094318339	2.904824395	-0.03247	0.974097599	NA	brown	-0.101396337	0.206373933		AT1G04030.1			hypothetical protein
Sapur.003G039600.5	0.026643677	-0.094318339	2.904824395	-0.03247	0.974097599	NA	brown	-0.102193443	0.20281059	GO:0016491	AT3G14130.1		Aldolase-type TIM barrel family protein	peroxisomal (S)-2-hydroxy-acid oxidase
Sapur.004G121500.1	0.025411094	-0.094318339	2.904824395	-0.03247	0.974097599	NA	brown	-0.099240611	0.216235759	GO:0043565,GO:0008270,GO:0006355,GO:0003700	AT5G66320.2	GATA5	GATA transcription factor 5	GATA transcription factor
Sapur.004G135500.3	0.025572424	-0.094318339	2.904824395	-0.03247	0.974097599	NA	brown	-0.081608864	0.309590942	GO:0005089	AT4G38430.1	ATROPGEF1,ROPGEF1	rho guanyl-nucleotide exchange factor 1	Rop guanine nucleotide exchange factor, putative
Sapur.005G093300.1	0.026053335	-0.094318339	2.904824395	-0.03247	0.974097599	NA	brown	-0.125744812	0.116596087	GO:0008017,GO:0007018,GO:0005524,GO:0003777,GO:0005871	AT3G51150.2		ATP binding microtubule motor family protein	kinesin motor domain protein
Sapur.005G163400.1	0.02769774	-0.094318339	2.904824395	-0.03247	0.974097599	NA	brown	-0.129073551	0.107155794	GO:0046983	AT3G59060.4	PIF5,PIL6	phytochrome interacting factor 3-like 6	helix loop helix DNA-binding domain protein
Sapur.006G139400.2	0.026602861	-0.094318339	2.904824395	-0.03247	0.974097599	NA	brown	-0.102149732	0.203004834	GO:0006412,GO:0005840,GO:0005622,GO:0003735	AT1G07210.1		Ribosomal protein S18	structural constituent of ribosome protein, putative
Sapur.008G083200.4	0.028123362	-0.094318339	2.904824395	-0.03247	0.974097599	NA	brown	-0.129822626	0.105116425	GO:0006355,GO:0006468,GO:0004672	AT3G06620.1		PAS domain-containing protein tyrosine kinase family protein	tyrosine kinase domain protein
Sapur.009G042300.2	0.025223114	-0.094318339	2.904824395	-0.03247	0.974097599	NA	brown	-0.098435559	0.220003449	GO:0008168,GO:0008152	AT3G60910.1		S-adenosyl-L-methionine-dependent methyltransferases superfamily protein	methyltransferase domain protein
Sapur.010G059900.1	0.025572424	-0.094318339	2.904824395	-0.03247	0.974097599	NA	brown	-0.081608864	0.309590942		AT2G32150.1		Haloacid dehalogenase-like hydrolase (HAD) superfamily protein	phosphatase, putative
Sapur.012G107800.2	0.025411094	-0.094318339	2.904824395	-0.03247	0.974097599	NA	brown	-0.099240611	0.216235759	GO:0006378,GO:0005849,GO:0003729	AT4G25550.1		Cleavage/polyadenylation specificity factor, 25kDa subunit	cleavage and polyadenylation specificity factor 5
Sapur.013G011400.1	0.025572424	-0.094318339	2.904824395	-0.03247	0.974097599	NA	brown	-0.081608864	0.309590942		AT3G05625.1		Tetratricopeptide repeat (TPR)-like superfamily protein	TPR repeat protein
Sapur.016G048200.1	0.02555025	-0.094318339	2.904824395	-0.03247	0.974097599	NA	brown	-0.099501084	0.215026636		AT3G20898.1			hypothetical protein
Sapur.016G073900.1	0.026348676	-0.094318339	2.904824395	-0.03247	0.974097599	NA	brown	-0.101608081	0.205423001		AT2G37400.1		Tetratricopeptide repeat (TPR)-like superfamily protein	TPR protein
Sapur.15WG140200.2	0.022812093	0.089324389	2.766924068	0.032283	0.97424643	NA	brown	-0.004716117	0.953254076	GO:0008017,GO:0000910,GO:0000226	AT5G51600.1	ATMAP65-3,MAP65-3,PLE	Microtubule associated protein (MAP65/ASE1) family protein	protein regulator of cytokinesis protein
Sapur.005G086900.2	0.022175285	0.089325403	2.777206387	0.032164	0.974341456	NA	brown	0.034593505	0.667108081		AT1G63855.3		Putative methyltransferase family protein	methyltransferase, putative
Sapur.011G115100.1	0.021216378	0.089326976	2.79340942	0.031978	0.974489787	NA	brown	-0.027051488	0.736639286	GO:0006508,GO:0004252	AT4G20430.1		Subtilase family protein	subtilisin-like protease
Sapur.017G057600.2	0.020795419	0.089327686	2.800815747	0.031893	0.97455702	NA	brown	-0.009608869	0.904927233		AT3G27100.1			enhancer of yellow 2 transcription factor-like protein
Sapur.T128300.1	0.020792694	0.08932769	2.800864304	0.031893	0.974557459	NA	brown	0.024619562	0.759556282					NA
Sapur.019G005400.1	0.020630512	0.089327967	2.803768635	0.03186	0.974583727	NA	brown	-0.027941972	0.728304377					NA
Sapur.014G082600.2	0.019009049	0.089330849	2.834681889	0.031514	0.974859997	NA	brown	-0.007597811	0.924758925	GO:0019205,GO:0006139,GO:0005524	AT5G26667.2	PYR6	P-loop containing nucleoside triphosphate hydrolases superfamily protein	adenylate kinase
Sapur.014G031100.5	0.022409295	0.091189602	2.904823276	0.031392	0.97495654	NA	brown	-0.114846535	0.152067369		AT1G75060.1			histone deacetylase complex subunit SAP30 Sin3-binding protein
Sapur.011G052400.2	0.019548011	0.091189602	2.904823276	0.031392	0.97495654	NA	brown	-0.225162003	0.004579305	GO:0046983,GO:0003677,GO:0006355,GO:0005634,GO:0003700	AT4G18960.1	AG	K-box region and MADS-box transcription factor family protein	MADS-box transcription factor
Sapur.15WG006600.3	0.017729487	0.091189602	2.904823276	0.031392	0.97495654	NA	brown	-0.156176775	0.050789448	GO:0016021,GO:0015689,GO:0015098	AT4G27720.1		Major facilitator superfamily protein	MFS transporter
Sapur.017G118700.1	0.013642903	0.091189602	2.904823276	0.031392	0.97495654	NA	brown	0.026340003	0.74332094	GO:0003677,GO:0046983	AT3G42170.1		BED zinc finger ;hAT family dimerisation domain	BED zinc finger protein
Sapur.002G165100.1	0.012266991	0.091189602	2.904823276	0.031392	0.97495654	NA	brown	0.028095007	0.726875137					NA
Sapur.003G057800.1	0.013095454	0.091189602	2.904823276	0.031392	0.97495654	NA	brown	0.022181399	0.782743872		AT2G35330.1		RING/U-box superfamily protein	synaptonemal complex protein, putative
Sapur.005G117800.2	0.013095454	0.091189602	2.904823276	0.031392	0.97495654	NA	brown	0.022181399	0.782743872		AT2G34320.1		Polynucleotidyl transferase, ribonuclease H-like superfamily protein	ribonuclease HI
Sapur.016G001100.1	0.012903815	0.091189602	2.904823276	0.031392	0.97495654	NA	brown	0.021942346	0.785028019		AT3G13300.2	VCS	Transducin/WD40 repeat-like superfamily protein	peptidase S26A, signal peptidase I; WD40-like protein, putative
Sapur.001G175100.2	0.012714087	0.091189602	2.904823276	0.031392	0.97495654	NA	brown	-0.081608864	0.309590942		AT1G60995.1			S3 self-incompatibility locus-linked pollen 3.15 protein
Sapur.017G041000.3	0.012523554	0.091189602	2.904823276	0.031392	0.97495654	NA	brown	0.020171654	0.802002862	GO:0006508,GO:0004197	AT1G02170.1	AMC1.ATMC1.ATMCPB1.LO	metacaspase 1	ICE-like protease (caspase) p20 domain protein
Sapur.016G218200.7	0.012428271	0.091189602	2.904823276	0.031392	0.97495654	NA	brown	0.018369268	0.819377074					hypothetical protein
Sapur.007G130100.1	0.011774057	0.091189602	2.904823276	0.031392	0.97495654	NA	brown	0.014727107	0.854746663		AT2G44060.1		Late embryogenesis abundant protein, group 2	late-embryogenesis abundant protein
Sapur.018G049400.1	0.011568611	0.091189602	2.904823276	0.031392	0.97495654	NA	brown	0.011149897	0.889769642		AT5G20120.1			transmembrane protein, putative
Sapur.005G200000.2	0.011564096	0.091189602	2.904823276	0.031392	0.97495654	NA	brown	0.012546957	0.876062381		AT1G42430.1			hypothetical protein
Sapur.007G021800.2	0.011229095	0.091189602	2.904823276	0.031392	0.97495654	NA	brown	0.010180568	0.899299686		AT2G18360.1		alpha/beta-Hydrolases superfamily protein	epoxide hydrolase
Sapur.010G180800.3	0.011218833	0.091189602	2.904823276	0.031392	0.97495654	NA	brown	0.006954198	0.931116185	GO:0005525	AT3G54840.1	ARA-6,ARA6,ATRABF5C,ATR	Ras-related small GTP-binding family protein	small GTPase family Rab protein
Sapur.004G127200.3	0.011203257	0.091189602	2.904823276	0.031392	0.97495654	NA	brown	0.008772239	0.913171092		AT3G19860.2	bHLH121	basic helix-loop-helix (bHLH) DNA-binding superfamily protein	DNA-binding protein, putative
Sapur.007G007600.4	0.011710772	0.091189602	2.904823276	0.031392	0.97495654	NA	brown	-0.081608864	0.309590942	GO:0005975,GO:0004553,GO:0051015,GO:0007015				NA
Sapur.005G071000.2	0.011081971	0.091189602	2.904823276	0.031392	0.97495654	NA	brown	0.007969154	0.921093135		AT1G05460.1	SDE3	P-loop containing nucleoside triphosphate hydrolases superfamily protein	NA
Sapur.002G013900.3	0.010903724	0.091189602	2.904823276	0.031392	0.97495654	NA	brown	0.006556545	0.935046189	GO:0055085,GO:0042626,GO:0016021,GO:0006810,GO:0005524,GO:0016887	AT1G27940.1	PGP13	P-glycoprotein 13	ABC transporter family B protein, group MDR/PGP protein PpABCB18 protein
Sapur.013G138200.1	0.010773633	0.091189602	2.904823276	0.031392	0.97495654	NA	brown	0.00331438	0.967138596	GO:0009982,GO:0009451,GO:0003723,GO:0001522	AT2G30320.1		Pseudouridine synthase family protein	tRNA pseudouridine synthase, putative
Sapur.016G179000.6	0.010751738	0.091189602	2.904823276	0.031392	0.97495654	NA	brown	0.003318844	0.967094359		AT4G34260.1	FUC95A	1,2-alpha-L-fucosidases	NA
Sapur.T145500.1	0.011608632	0.091189602	2.904823276	0.031392	0.97495654	NA	brown	-0.081608864	0.309590942		AT1G55000.3		peptidoglycan-binding LysM domain-containing protein	NA
Sapur.001G090700.2	0.010089121	0.091189602	2.904823276	0.031392	0.97495654	NA	brown	-0.000231945	0.99769967	GO:0055085	AT1G12600.1		UDP-N-acetylglucosamine (UAA) transporter family	adenosine 3'-phospho 5'-phosphosulfate transporter 2
Sapur.010G053200.3	0.010089121	0.091189602	2.904823276	0.031392	0.97495654	NA	brown	-0.000231945	0.99769967		AT1G67600.1		Acid phosphatase/vanadium-dependent haloperoxidase-related protein	acid phosphatase/vanadium-dependent haloperoxidase-like protein
Sapur.009G106700.4	0.009934049	0.091189602	2.904823276	0.031392	0.97495654	NA	brown	-0.002730798	0.972922257	GO:0055114,GO:0020037,GO:0006979,GO:0004601	AT4G35000.1	APX3	ascorbate peroxidase 3	L-ascorbate peroxidase 3, peroxisomal protein
Sapur.006G157100.2	0.009843971	0.091189602	2.904823276	0.031392	0.97495654	NA	brown	-0.004345567	0.956923261	GO:0016787	AT2G42070.1	ATNUDT23,ATNUDX23,NU	nudix hydrolase homolog 23	nudix hydrolase
Sapur.016G140400.1	0.009843971	0.091189602	2.904823276	0.031392	0.97495654	NA	brown	0.055370559	0.49096448		AT2G29670.1		Tetratricopeptide repeat (TPR)-like superfamily protein	plant/F22G5-32 protein, putative
Sapur.001G092800.7	0.009840551	0.091189602	2.904823276	0.031392	0.97495654	NA	brown	-0.004238737	0.957981268		AT4G11970.2		YTH family protein	splicing factor YT521-B, putative
Sapur.T128800.1	0.009578282	0.091189602	2.904823276	0.031392	0.97495654	NA	brown	-0.00697611	0.930899682					NA
Sapur.016G274400.2	0.012307605	0.091189602	2.904823276	0.031392	0.97495654	NA	brown	-0.081608864	0.309590942	GO:0050662,GO:0003824	AT4G27250.1		NAD(P)-binding Rossmann-fold superfamily protein	oxidoreductase, short chain dehydrogenase/reductase family protein
Sapur.008G047900.3	0.009552687	0.091189602	2.904823276	0.031392	0.97495654	NA	brown	-0.0065031	0.93557451		AT5G05987.1	PRA1.A2	prenylated RAB acceptor 1.A2	prenylated rab acceptor family protein
Sapur.016G119700.2	0.009331617	0.091189602	2.904823276	0.031392	0.97495654	NA	brown	-0.22916433	0.003889383	GO:0005975,GO:0004553	AT2G44480.1	BGLU17	beta glucosidase 17	glycoside hydrolase family 1 protein
Sapur.014G137800.2	0.009152712	0.091189602	2.904823276	0.031392	0.97495654	NA	brown	-0.014073346	0.861128318	GO:0005515	AT3G07670.1		Rubisco methyltransferase family protein	ribulose-1%2C5-bisphosphate carboxylase/oxygenase small subunit N-methyltransferase I, putative
Sapur.014G117200.2	0.00892009	0.091189602	2.904823276	0.031392	0.97495654	NA	brown	-0.015859544	0.84371491		AT3G19340.1		Protein of unknown function (DUF3754)	NA
Sapur.018G039800.3	0.00892009	0.091189602	2.904823276	0.031392	0.97495654	NA	brown	-0.015859544	0.84371491	GO:0055085,GO:0046873,GO:0030001,GO:0016020	AT1G10970.1	ATZIP4,ZIP4	zinc transporter 4 precursor	ZIP-like zinc transporter
Sapur.017G100300.1	0.009976959	0.091189602	2.904823276	0.031392	0.97495654	NA	brown	-0.027403511	0.733340627		AT1G60640.2			C2H2 type zf-met: zinc-finger protein
Sapur.T140300.2	0.008911822	0.091189602	2.904823276	0.031392	0.97495654	NA	brown	-0.016085734	0.841515014	GO:0009086,GO:0008270,GO:0003871,GO:0008652	AT5G17920.2	ATCIMS,ATMETS,ATMS1	Cobalamin-independent synthase family protein	5-methyltetrahydropteroyltriglutamate-homocysteine S-methyltransferase
Sapur.005G021600.2	0.008895934	0.091189602	2.904823276	0.031392	0.97495654	NA	brown	-0.016977253	0.832856309		AT1G09020.1	ATSNF4,SNF4	homolog of yeast sucrose nonfermenting 4	5'-AMP-activated kinase subunit gamma
Sapur.002G069000.1	0.008889915	0.091189602	2.904823276	0.031392	0.97495654	NA	brown	-0.016867407	0.833922108		AT1G21930.1			transmembrane protein, putative
Sapur.005G034100.2	0.008691927	0.091189602	2.904823276	0.031392	0.97495654	NA	brown	-0.019082061	0.812495058	GO:0055085,GO:0016021,GO:0015299,GO:0006812	AT3G05030.1	ATNHX2,NHX2	sodium hydrogen exchanger 2	sodium/hydrogen exchanger
Sapur.001G027600.1	0.00863665	0.091189602	2.904823276	0.031392	0.97495654	NA	brown	-0.021570425	0.788585369	GO:0042546,GO:0016020,GO:0008107	AT2G03220.1	ATFT1,ATFUT1,FT1,MUR2	fucosyltransferase 1	galactoside 2-alpha-L-fucosyltransferase, putative
Sapur.002G023700.3	0.00863665	0.091189602	2.904823276	0.031392	0.97495654	NA	brown	-0.114024224	0.155042406	GO:0016021	AT1G35180.1		TRAM, LAG1 and CLN8 (TLC) lipid-sensing domain containing protein	TRAM, LAG1 and CLN8 (TLC) lipid-sensing domain protein, putative
Sapur.007G037500.3	0.0085845	0.091189602	2.904823276	0.031392	0.97495654	NA	brown	-0.022011075	0.784371127	GO:0005515,GO:0006457,GO:0003755,GO:0000413	AT2G15790.1	CYP40,SQN	peptidyl-prolyl cis-trans isomerase / cyclophilin-40 (CYP40) / rotamase	cyclophilin-type peptidylprolyl cis-trans isomerase family protein
Sapur.019G029600.1	0.0085845	0.091189602	2.904823276	0.031392	0.97495654	NA	brown	-0.022011075	0.784371127	GO:0016787,GO:0005524,GO:0003677	AT1G08600.4	ATRX,CHR20	P-loop containing nucleoside triphosphate hydrolases superfamily protein	chromatin remodeling complex subunit
Sapur.011G044600.2	0.008452824	0.091189602	2.904823276	0.031392	0.97495654	NA	brown	-0.023056553	0.774397724		AT1G29050.1	TBL38	TRICHOME BIREFRINGENCE-LIKE 38	Pmr5/Cas1p GDSL/SGNH-like acyl-esterase family protein
Sapur.013G012800.2	0.008760264	0.091189601	2.904823276	0.031392	0.97495654	NA	brown	-0.03766135	0.639576796	GO:0003723	AT1G09150.1		pseudouridine synthase and archaeosine transglycosylase (PUA) domain-containing protein	pseudouridine synthase and archaeosine transglycosylase domain protein
Sapur.T050100.7	0.009300923	0.091189601	2.904823276	0.031392	0.97495654	NA	brown	-0.040918931	0.610872238		AT3G24870.1		Helicase/SANT-associated, DNA binding protein	helicase/SANT-associated, DNA-binding protein, putative
Sapur.012G096800.2	0.007846412	0.091189601	2.904823276	0.031392	0.97495654	NA	brown	-0.033275808	0.679073724	GO:0005992,GO:0003824	AT5G51460.2	ATTPPA	Haloacid dehalogenase-like hydrolase (HAD) superfamily protein	trehalose-phosphate phosphatase
Sapur.008G165600.1	0.018380274	0.089332011	2.84746766	0.031372	0.974972519	NA	brown	-0.067382325	0.401747272	GO:0005515	AT5G18590.1		Galactose oxidase/kelch repeat superfamily protein	acyl-CoA-binding domain protein
Sapur.016G142000.1	0.01793132	0.089332861	2.856940498	0.031269	0.975055239	NA	brown	-0.019669504	0.806834033		AT5G01450.1		RING/U-box superfamily protein	ubiquitin-protein ligase, putative
Sapur.004G104100.3	0.041473548	0.064011049	2.051161124	0.031207	0.975104277	NA	brown	-0.003754575	0.962777104	GO:0055114,GO:0016702	AT5G39890.1		Protein of unknown function (DUF1637)	2-aminoethanethiol dioxygenase, putative
Sapur.008G148600.1	0.027669758	0.06621559	2.12926103	0.031098	0.975191448	NA	brown	-0.059275462	0.460859069		AT5G14420.4	RGLG2	RING domain ligase2	E3 ubiquitin-protein ligase RGLG2
Sapur.017G019500.4	0.049591092	-0.062544835	2.022844657	-0.03092	0.97533394	NA	brown	-0.08130332	0.311411914	GO:0005524,GO:0005515	AT1G05910.1		cell division cycle protein 48-related / CDC48-related	bromodomain protein with A AAA ATPase domain protein
Sapur.019G060200.4	0.023073428	0.089337012	2.904822183	0.030755	0.975465146	NA	brown	0.011851962	0.882876988	GO:0071805,GO:0016020,GO:0015079	AT2G40540.2	ATKT2.ATKUP2.KT2.KUP2.SI	potassium transporter 2	potassium transporter 2
Sapur.016G000400.1	0.021527804	0.089337012	2.904822183	0.030755	0.975465146	NA	brown	0.006275982	0.937819946					transmembrane protein, putative
Sapur.016G167400.2	0.019086657	0.089337012	2.904822183	0.030755	0.975465146	NA	brown	-0.007703152	0.923718866		AT2G02560.2	ATCAND1.CAND1.ETA2.HVE	cullin-associated and neddylation dissociated	NA
Sapur.007G079800.1	0.019065983	0.089337012	2.904822183	0.030755	0.975465146	NA	brown	-0.00933215	0.90765285					hypothetical protein
Sapur.15WG042900.1	0.018122954	0.089337012	2.904822183	0.030755	0.975465146	NA	brown	-0.016549074	0.83701248		AT3G18210.2		2-oxoglutarate (2OG) and Fe(II)-dependent oxygenase superfamily protein	NA
Sapur.003G095200.2	0.038312349	0.056936921	1.875454255	0.030359	0.975780741	NA	brown	-0.061494039	0.44422077		AT5G41470.1		Nuclear transport factor 2 (NTF2) family protein	NA
Sapur.005G000200.1	0.047416505	-0.060689683	2.023206289	-0.03	0.976069618	NA	brown	-0.10119057	0.207301049		AT3G05940.1		Protein of unknown function (DUF300)	organic solute transporter ostalpha protein
Sapur.006G218500.3	0.042424674	-0.046242581	1.561804135	-0.02961	0.976379336	NA	brown	-0.059348307	0.460307346		AT4G31850.1	PGR3	proton gradient regulation 3	PPR containing plant protein
Sapur.011G091100.1	0.007902174	0.085433515	2.904823455	0.029411	0.976536868	NA	brown	-0.050985013	0.525984256	GO:0005515,GO:0016459,GO:0005524,GO:0003774	AT5G54280.2	ATM2,ATM4,ATMYOS1	myosin 2	NA
Sapur.018G068000.1	0.008534419	0.085433515	2.904823455	0.029411	0.976536868	NA	brown	-0.053462954	0.506043093		AT4G25845.1			DUF688 family protein
Sapur.017G039600.3	0.047469489	0.047888604	1.68179538	0.028475	0.977283553	NA	brown	-0.122087571	0.12770056		AT3G21110.2	ATPURC,PUR7,PURC	purin 7	phosphoribosylaminoimidazole-succinocarboxamide synthase
Sapur.018G042100.1	0.036226584	-0.058821913	2.072142458	-0.02839	0.977353491	NA	brown	-0.124830669	0.119298652	GO:0009626	AT1G23070.1		Protein of unknown function (DUF300)	organic solute transporter ostalpha protein
Sapur.006G101700.2	0.031597734	0.06622319	2.347961819	0.028205	0.977499014	NA	brown	-0.024652941	0.759240267	GO:0016567,GO:0004842	AT3G53690.1		RING/U-box superfamily protein	zinc finger protein, putative
Sapur.016G172300.3	0.025315327	0.066224832	2.40787767	0.027503	0.978058225	NA	brown	-0.073781691	0.35843736	GO:0008144,GO:0016773,GO:0005515	AT1G50030.1	TOR	target of rapamycin	Serine/Threonine-kinase TOR
Sapur.016G062400.2	0.049586285	-0.060638918	2.241799301	-0.02705	0.978420478	NA	brown	-0.067807227	0.398778705	GO:0006760,GO:0004150	AT3G11750.1	FOLB1	Dihydroneopterin aldolase	dihydroneopterin aldolase
Sapur.010G014900.1	0.049532247	-0.060638499	2.243926432	-0.02702	0.978441078	NA	brown	-0.07952322	0.322159701		AT5G18800.2		Cox19-like CHCH family protein	NADH dehydrogenase (ubiquinone) 1 alpha subcomplex protein
Sapur.016G035000.1	0.047813394	-0.060637469	2.24918703	-0.02696	0.978491855	NA	brown	-0.120515451	0.132717222					hypothetical protein
Sapur.010G114000.3	0.043096889	0.049816877	1.84909365	0.026941	0.9785066	NA	brown	-0.081905797	0.307827974					hypothetical protein
Sapur.010G114000.4	0.037654961	0.049825016	1.871734003	0.02662	0.978763053	NA	brown	-0.081608864	0.309590942					hypothetical protein
Sapur.005G053800.1	0.035942777	-0.058771261	2.314622803	-0.02539	0.979742858	NA	brown	-0.106425877	0.184632049		AT4G08330.1			hypothetical protein
Sapur.018G048700.4	0.026871348	0.068437766	2.708832111	0.025265	0.979843849	NA	brown	-0.115782905	0.148732318		AT5G56750.1	NDL1	N-MYC downregulated-like 1	pollen specific protein SF21, putative
Sapur.002G147500.3	0.044417266	-0.046216464	1.850675353	-0.02497	0.980076694	NA	brown	-0.077142294	0.336905284	GO:0016853,GO:0005975	AT3G61610.1		Galactose mutarotase-like superfamily protein	aldose 1-epimerase
Sapur.009G064900.2	0.029968981	-0.05876322	2.36203947	-0.02488	0.980152139	NA	brown	-0.081608864	0.309590942	GO:0008152,GO:0003824	AT1G50110.1		D-aminoacid aminotransferase-like PLP-dependent enzymes superfamily protein	branched-chain-amino-acid aminotransferase
Sapur.009G108300.2	0.04087632	0.049879648	2.050117001	0.02433	0.980589266	NA	brown	-0.022014613	0.784337319		AT2G16920.1	PFU2,UBC23	ubiquitin-conjugating enzyme 23	ubiquitin-conjugating enzyme E2, putative
Sapur.002G179800.2	0.005958874	0.070654775	2.904823918	0.024323	0.980594763	NA	brown	-0.064087446	0.425209107	GO:0006886,GO:0008536	AT2G31660.1	SAD2,URM9	ARM repeat superfamily protein	importin-7 protein, putative
Sapur.003G041300.1	0.006120203	0.070654775	2.904823918	0.024323	0.980594763	NA	brown	-0.063922497	0.426404114	GO:0044237	AT1G53880.1		Eukaryotic translation initiation factor 2B (eIF-2B) family protein	translation initiation factor eIF-2B subunit alpha
Sapur.006G023200.1	0.007079334	0.070654775	2.904823918	0.024323	0.980594763	NA	brown	-0.067295305	0.402356848	GO:0005515	AT1G79590.2	ATSYP52,SYP52	syntaxin of plants 52	syntaxin-52 protein
Sapur.008G130800.2	0.006120203	0.070654775	2.904823918	0.024323	0.980594763	NA	brown	-0.110170366	0.16957068	GO:0006355,GO:0005634	AT5G43700.1	ATAUX2-11,IAA4	AUX/IAA transcriptional regulator family protein	indole-3-acetic acid inducible protein
Sapur.013G139600.1	0.007205671	0.070654775	2.904823918	0.024323	0.980594763	NA	brown	-0.067256072	0.402631859		AT2G30370.1	CHAL,EPFL6	allergen-related	epidermal patterning factor-like protein, putative
Sapur.016G127500.2	0.006942776	0.070654775	2.904823918	0.024323	0.980594763	NA	brown	-0.067037927	0.404162998	GO:0006468,GO:0005524,GO:0004672	AT1G07870.2		Protein kinase superfamily protein	Serine/Threonine kinase
Sapur.016G283000.1	0.007079334	0.070654775	2.904823918	0.024323	0.980594763	NA	brown	-0.067295305	0.402356848	GO:0005515	AT2G33060.1	AtRLP27,RLP27	receptor like protein 27	NA
Sapur.018G037600.2	0.006913044	0.070654775	2.904823918	0.024323	0.980594763	NA	brown	-0.066603996	0.407218976	GO:0005515	AT4G33210.1	SLOMO	F-box family protein	F-box/LRR protein
Sapur.15WG149900.1	0.005929524	0.070654775	2.904823918	0.024323	0.980594763	NA	brown	-0.063790949	0.427358531		AT5G23760.1		Copper transport protein family	heavy-metal-associated domain protein, putative
Sapur.004G064700.3	0.048256067	-0.060591237	2.534580357	-0.02391	0.980927727	NA	brown	-0.043999629	0.584259702		AT1G23170.2		Protein of unknown function DUF2359, transmembrane	DUF2359 family protein
Sapur.014G113300.2	0.034079916	0.049889276	2.087464023	0.023899	0.9809328	NA	brown	-0.037506934	0.64095126	GO:0016021,GO:0015693,GO:0015095	AT3G26670.1		Protein of unknown function (DUF803)	magnesium transporter NIPA protein
Sapur.018G063000.1	0.033223875	0.049890606	2.092792715	0.023839	0.980980833	NA	brown	-0.048149202	0.54928348	GO:0005515	AT5G19680.1		Leucine-rich repeat (LRR) family protein	protein phosphatase 1 regulatory subunit
Sapur.009G055200.2	0.016021615	0.06843981	2.900878619	0.023593	0.981177426	NA	brown	-0.064989651	0.418707252		AT5G12330.3	LRP1	Lateral root primordium (LRP) protein-related	short internode-related sequence protein, putative
Sapur.004G024500.4	0.015117739	0.068439844	2.904822821	0.023561	0.98120297	NA	brown	-0.03850726	0.632069124	GO:0006508,GO:0004185	AT1G11080.2	scpl31	serine carboxypeptidase-like 31	serine carboxypeptidase-like protein
Sapur.005G003400.2	0.015347223	0.068439844	2.904822821	0.023561	0.98120297	NA	brown	-0.037686308	0.639354756		AT4G10710.1	SPT16	global transcription factor C	global transcription factor group protein
Sapur.013G011500.3	0.040465863	-0.060584265	2.588559296	-0.0234	0.981327514	NA	brown	-0.063403266	0.430178433	GO:0003824	AT3G12070.1	AtRGTB2,RGTB2	RAB geranylgeranyl transferase beta subunit 2	RAB geranylgeranyl transferase type II beta subunit, putative
Sapur.009G116800.3	0.039392263	-0.060583206	2.597081805	-0.02333	0.981389103	NA	brown	-0.075419299	0.347839913		AT5G40270.1		HD domain-containing metal-dependent phosphohydrolase family protein	metal-dependent phosphohydrolase HD domain protein
Sapur.15WG027600.2	0.047454423	-0.046206049	2.028832382	-0.02277	0.981829989	NA	brown	-0.064127011	0.424922758	GO:0006807,GO:0004356	AT5G37600.1	ATGLN1:1.ATGSR1.GLN1:1.(	glutamine synthase clone R1	glutamine synthetase
Sapur.016G216100.1	0.042924904	-0.046205075	2.048663719	-0.02255	0.982006227	NA	brown	-0.061228834	0.446191638		AT3G27460.1		SGF29 tudor-like domain	SGF29 tudor-like domain protein
Sapur.013G143500.3	0.036023015	-0.058727595	2.617173632	-0.02244	0.982097516	NA	brown	-0.101938181	0.203946835		AT5G10860.1		Cystathionine beta-synthase (CBS) family protein	named after animal CBS
Sapur.005G206700.2	0.03206466	-0.058723339	2.65400961	-0.02213	0.98234723	NA	brown	-0.096176977	0.230822061	GO:0006096,GO:0003872	AT1G20950.1		Phosphofructokinase family protein	pyrophosphate-fructose 6-phosphate 1-phosphotransferase alpha subunit
Sapur.016G040700.1	0.03157691	-0.058722616	2.660429745	-0.02207	0.982390039	NA	brown	-0.091635103	0.253698284	GO:0055114,GO:0050660,GO:0016491,GO:0019139,GO:0009690	AT2G41510.1	ATCKX1,CKX1	cytokinin oxidase/dehydrogenase 1	cytokinin oxidase/dehydrogenase
Sapur.002G090600.2	0.030381326	-0.058721177	2.673369657	-0.02197	0.982475692	NA	brown	-0.106007023	0.186375913		AT5G49630.1	AAP6	amino acid permease 6	amino acid transporter
Sapur.003G041600.2	0.029788165	0.051799612	2.361019229	0.02194	0.982496206	NA	brown	-0.05016977	0.532630777		AT3G14280.1			hypothetical protein
Sapur.016G216700.2	0.02908762	0.051800663	2.367352084	0.021881	0.982542668	NA	brown	-0.041708601	0.604000061	GO:0016020,GO:0006487,GO:0003830	AT3G27540.1		beta-1,4-N-acetylglucosaminyltransferase family protein	beta-1%2C4-N-acetylglucosaminyltransferase family protein
Sapur.15WG076800.1	0.038126759	0.049935716	2.303605887	0.021677	0.982705455	NA	brown	-0.010882319	0.892398849		AT5G08050.1		Protein of unknown function (DUF1118)	plant/F13G24-250 protein, putative
Sapur.016G250000.2	0.024691834	0.051807841	2.412097152	0.021478	0.982864082	NA	brown	-0.033841108	0.673930363	GO:0043565	AT3G58680.1	ATMBF1B,MBF1B	multiprotein bridging factor 1B	multiprotein bridging factor 1
Sapur.010G122000.7	0.023605051	0.051809767	2.424567327	0.021369	0.98295157	NA	brown	-0.035600283	0.658021779		AT2G03510.1		SPFH/Band 7/PHB domain-containing membrane-associated protein family	SPFH domain/band 7 family protein
Sapur.014G011500.3	0.028900093	-0.056866958	2.683785726	-0.02119	0.983094823	NA	brown	-0.159205512	0.046412785		AT5G01450.1		RING/U-box superfamily protein	zinc finger, RING-type protein
Sapur.005G053900.4	0.035068845	-0.043994226	2.081505582	-0.02114	0.98313735	NA	brown	-0.130505748	0.103283325		AT5G08720.1			cyclase/dehydrase
Sapur.010G195100.1	0.02652052	-0.056863852	2.713404874	-0.02096	0.983280244	NA	brown	-0.163092255	0.041261209	GO:0006412,GO:0005840,GO:0005622,GO:0003735	AT5G04800.1		Ribosomal S17 family protein	40S ribosomal protein S17
Sapur.010G104200.1	0.039016576	-0.060551457	2.904822183	-0.02085	0.98336918	NA	brown	-0.058389069	0.467601816	GO:0016780,GO:0016020,GO:0008654	AT1G13560.1	AAPT1,ATAAPT1	aminoalcoholphosphotransferase 1	aminoalcoholphosphotransferase
Sapur.006G108800.2	0.023627395	-0.056859745	2.754275287	-0.02064	0.9835295	NA	brown	-0.163989577	0.040142602	GO:0006468,GO:0004672	AT2G37050.3		Leucine-rich repeat protein kinase family protein	LRR receptor-like kinase
Sapur.016G152100.4	0.020580461	-0.056854948	2.804673031	-0.02027	0.983826785	NA	brown	-0.161000866	0.043970152	GO:0006265,GO:0005524,GO:0003918,GO:0003677	AT3G23890.2	ATTOPII,TOPII	topoisomerase II	DNA topoisomerase
Sapur.007G117200.2	0.020212705	-0.056854331	2.811387081	-0.02022	0.98386558	NA	brown	-0.161507534	0.043300589		AT3G20320.1	TGD2	trigalactosyldiacylglycerol2	ABC-type transport system protein
Sapur.001G069000.5	0.03036492	-0.058698868	2.904823276	-0.02021	0.983877941	NA	brown	-0.085213816	0.288633027		AT4G27745.1		Yippee family putative zinc-binding protein	Fad NAD-binding oxidoreductase, putative
Sapur.013G137400.3	0.030692568	-0.058698867	2.904823276	-0.02021	0.983877941	NA	brown	-0.063930187	0.426348364	GO:0043565,GO:0006355,GO:0003700	AT2G38470.1	ATWRKY33,WRKY33	WRKY DNA-binding protein 33	WRKY transcription factor
Sapur.001G054000.1	0.031046969	-0.058698867	2.904823276	-0.02021	0.983877941	NA	brown	-0.062870203	0.434073206		AT5G13390.1	NEF1	no exine formation 1	No exine formation protein
Sapur.011G007300.1	0.037707821	-0.058698867	2.904823276	-0.02021	0.983877941	NA	brown	-0.052463236	0.514040611		AT1G11925.1		Stigma-specific Stig1 family protein	NA
Sapur.004G004200.1	0.019855485	-0.056853722	2.8180575	-0.02017	0.983903937	NA	brown	-0.159692525	0.045739265		AT1G11925.1		Stigma-specific Stig1 family protein	Stig1 stigma-specific protein
Sapur.016G001800.2	0.019086281	-0.056852362	2.833150625	-0.02007	0.983990058	NA	brown	-0.161871712	0.042824614		AT2G01060.1		myb-like HTH transcriptional regulator family protein	Myb family transcription factor, putative
Sapur.009G027800.2	0.033304309	0.051838427	2.638851047	0.019644	0.984327111	NA	brown	-0.005558305	0.944918495	GO:0016787	AT2G39840.1	TOPP4	type one serine/threonine protein phosphatase 4	serine/threonine-protein phosphatase
Sapur.001G025800.5	0.020457939	-0.056846222	2.904824395	-0.01957	0.984386723	NA	brown	-0.081608864	0.309590942	GO:0051726,GO:0005634,GO:0006357,GO:0007049,GO:0006351	AT3G12280.1	ATRBR1,RB,RB1,RBR,RBR1	retinoblastoma-related 1	retinoblastoma-related protein
Sapur.001G122400.4	0.020457939	-0.056846222	2.904824395	-0.01957	0.984386723	NA	brown	-0.081608864	0.309590942	GO:0005515,GO:0006468,GO:0004672	AT1G14390.1		Leucine-rich repeat protein kinase family protein	LRR receptor-like Serine/Threonine-kinase, putative
Sapur.001G185900.1	0.021866437	-0.056846222	2.904824395	-0.01957	0.984386723	NA	brown	-0.10740469	0.180603527		AT1G10310.1		NAD(P)-binding Rossmann-fold superfamily protein	short-chain dehydrogenase/reductase
Sapur.003G016000.1	0.022228333	-0.056846222	2.904824395	-0.01957	0.984386723	NA	brown	-0.108271905	0.177088647		AT1G15290.1		Tetratricopeptide repeat (TPR)-like superfamily protein	eukaryotic translation initiation factor 3 subunit, putative
Sapur.003G078100.3	0.018358213	-0.056846222	2.904824395	-0.01957	0.984386723	NA	brown	-0.12931693	0.106489813		AT1G12250.2		Pentapeptide repeat-containing protein	thylakoid lumenal protein
Sapur.004G081000.1	0.025925866	-0.056846222	2.904824395	-0.01957	0.984386723	NA	brown	-0.13913716	0.082225669					NA
Sapur.004G088100.5	0.020457939	-0.056846222	2.904824395	-0.01957	0.984386723	NA	brown	-0.081608864	0.309590942					hypothetical protein
Sapur.005G048000.1	0.021197687	-0.056846222	2.904824395	-0.01957	0.984386723	NA	brown	-0.135859954	0.089773714		AT4G33440.1		Pectin lyase-like superfamily protein	glycoside hydrolase family 28 protein
Sapur.005G129300.3	0.020354716	-0.056846222	2.904824395	-0.01957	0.984386723	NA	brown	-0.103718669	0.196116427	GO:0003676	AT5G64200.1	ATSC35,SC35	ortholog of human splicing factor SC35	arginine/serine-rich splicing factor SC30 transcript protein
Sapur.006G066000.3	0.023340259	-0.056846222	2.904824395	-0.01957	0.984386723	NA	brown	-0.110278816	0.169148484	GO:0046872	AT1G75340.1		Zinc finger C-x8-C-x5-C-x3-H type family protein	zinc finger CCCH domain protein, putative
Sapur.006G079000.2	0.020457939	-0.056846222	2.904824395	-0.01957	0.984386723	NA	brown	-0.081608864	0.309590942		AT3G09090.1	DEX1	defective in exine formation protein (DEX1)	defective in exine formation protein
Sapur.007G038100.8	0.021128523	-0.056846222	2.904824395	-0.01957	0.984386723	NA	brown	-0.105598514	0.188088286		AT4G37080.2		Protein of unknown function, DUF547	ternary complex factor MIP1 leucine-zipper protein
Sapur.007G084400.3	0.020457939	-0.056846222	2.904824395	-0.01957	0.984386723	NA	brown	-0.081608864	0.309590942		AT4G39870.1		TLD-domain containing nucleolar protein	TLD family protein
Sapur.008G067500.6	0.021759911	-0.056846222	2.904824395	-0.01957	0.984386723	NA	brown	-0.136678295	0.087839046		AT3G19895.1		RING/U-box superfamily protein	hypothetical protein
Sapur.008G069100.1	0.020457939	-0.056846222	2.904824395	-0.01957	0.984386723	NA	brown	-0.081608864	0.309590942					transmembrane protein, putative
Sapur.008G112700.1	0.020457939	-0.056846222	2.904824395	-0.01957	0.984386723	NA	brown	-0.081608864	0.309590942		AT1G68080.1		2-oxoglutarate (2OG) and Fe(II)-dependent oxygenase superfamily protein	2-oxoglutarate (2OG) and Fe(II)-dependent oxygenase domain protein
Sapur.010G019100.2	0.026365436	-0.056846222	2.904824395	-0.01957	0.984386723	NA	brown	-0.112836444	0.159416651		AT1G13195.1		RING/U-box superfamily protein	ubiquitin-protein ligase, putative
Sapur.010G112900.3	0.021174758	-0.056846222	2.904824395	-0.01957	0.984386723	NA	brown	-0.105977688	0.186498496	GO:0006457,GO:0003755,GO:0000413	AT3G66654.1		Cyclophilin-like peptidyl-prolyl cis-trans isomerase family protein	NA
Sapur.010G162800.2	0.020457939	-0.056846222	2.904824395	-0.01957	0.984386723	NA	brown	-0.081608864	0.309590942		AT3G55390.1		Uncharacterised protein family (UPF0497)	CASP-like protein POPTRDRAFT-822486 protein
Sapur.011G042700.6	0.026222579	-0.056846222	2.904824395	-0.01957	0.984386723	NA	brown	-0.113128144	0.158333937	GO:0019478,GO:0016788,GO:0005737	AT4G18460.1		D-Tyr-tRNA(Tyr) deacylase family protein	D-tyrosyl-tRNA(Tyr) deacylase
Sapur.012G089200.2	0.022108255	-0.056846222	2.904824395	-0.01957	0.984386723	NA	brown	-0.107737513	0.179248553		AT5G45100.1		SBP (S-ribonuclease binding protein) family protein	baculoviral IAP repeat protein, putative
Sapur.013G035100.1	0.020457939	-0.056846222	2.904824395	-0.01957	0.984386723	NA	brown	-0.081608864	0.309590942	GO:0008270,GO:0003676,GO:0004527	AT1G54490.1	AIN1,ATXRN4,EIN5,XRN4	exoribonuclease 4	5'-3' exoribonuclease
Sapur.013G079700.1	0.025400055	-0.056846222	2.904824395	-0.01957	0.984386723	NA	brown	-0.105012054	0.190566643					NA
Sapur.013G099900.2	0.021941903	-0.056846222	2.904824395	-0.01957	0.984386723	NA	brown	-0.107478127	0.180303909	GO:0043039,GO:0005737,GO:0005524,GO:0004812,GO:0000049	AT3G58140.1		phenylalanyl-tRNA synthetase class IIc family protein	phenylalanyl-tRNA synthetase
Sapur.014G070900.3	0.020457939	-0.056846222	2.904824395	-0.01957	0.984386723	NA	brown	-0.081608864	0.309590942		AT2G46170.1		Reticulon family protein	24 kDa seed maturation protein, putative
Sapur.016G174200.1	0.024204528	-0.056846222	2.904824395	-0.01957	0.984386723	NA	brown	-0.139061109	0.082394863	GO:0005634	AT1G70210.1	ATCYCD1;1,CYCD1;1	CYCLIN D1;1	D-type cyclin
Sapur.016G307800.1	0.021508659	-0.056846222	2.904824395	-0.01957	0.984386723	NA	brown	-0.10678713	0.183137633		AT1G10010.1	AAP8,ATAAP8	amino acid permease 8	amino acid permease
Sapur.017G082300.2	0.023197451	-0.056846222	2.904824395	-0.01957	0.984386723	NA	brown	-0.110221558	0.169371291		AT3G62870.1		Ribosomal protein L7Ae/L30e/S12e/Gadd45 family protein	NA
Sapur.018G052500.2	0.020457939	-0.056846222	2.904824395	-0.01957	0.984386723	NA	brown	-0.081608864	0.309590942	GO:0008270	AT1G11020.1		RING/FYVE/PHD zinc finger superfamily protein	C3HC4-type zinc finger protein, putative
Sapur.018G053600.1	0.024831371	-0.056846222	2.904824395	-0.01957	0.984386723	NA	brown	-0.140277395	0.079722247	GO:0016021	AT5G19930.1		Protein of unknown function DUF92, transmembrane	integral membrane family protein
Sapur.018G086900.2	0.023340259	-0.056846222	2.904824395	-0.01957	0.984386723	NA	brown	-0.110278816	0.169148484	GO:0006468,GO:0004672,GO:0005975,GO:0004553	AT4G19810.1		Glycosyl hydrolase family protein with chitinase insertion domain	tyrosine kinase family protein
Sapur.018G118200.1	0.021941903	-0.056846222	2.904824395	-0.01957	0.984386723	NA	brown	-0.107478127	0.180303909		AT4G18240.1	ATSS4,SS4,SSIV	starch synthase 4	starch synthase
Sapur.019G012400.1	0.020457939	-0.056846222	2.904824395	-0.01957	0.984386723	NA	brown	-0.081608864	0.309590942	GO:0055114,GO:0016491,GO:0008270	AT5G63620.2		GroES-like zinc-binding alcohol dehydrogenase family protein	NA
Sapur.019G021900.3	0.021924083	-0.056846222	2.904824395	-0.01957	0.984386723	NA	brown	-0.137373899	0.086220856		AT5G28640.1	AN3,ATGIF1,GIF,GIF1	SSXT family protein	SSXT protein (amino-terminal region) protein
Sapur.15WG009000.2	0.019933177	-0.056846222	2.904824395	-0.01957	0.984386723	NA	brown	-0.102301584	0.202330601	GO:0005515	AT4G03220.1		Protein with RNI-like/FBD-like domains	F-box protein
Sapur.15WG111600.1	0.021655571	-0.056846222	2.904824395	-0.01957	0.984386723	NA	brown	-0.137184375	0.086659366	GO:0006414,GO:0005853,GO:0003746	AT2G18110.1		Translation elongation factor EF1B/ribosomal protein S6 family protein	elongation factor 1-beta, putative
Sapur.011G020600.3	0.029634676	0.051842861	2.677857956	0.01936	0.984554059	NA	brown	-0.006010505	0.94044524	GO:0048544,GO:0006468,GO:0004672	AT1G11340.1		S-locus lectin protein kinase family protein	Serine/Threonine kinase domain protein

Sapur.008G166200.4	0.03933537	0.049979566	2.591246917	0.019288	0.984611481	NA	brown	-0.007111965	0.929557429	GO:0046872,GO:0008270,GO:0016567	AT5G18650.1		CHY-type/CTCHY-type/RING-type Zinc finger protein	CHY zinc finger protein
Sapur.001G081800.1	0.038827273	-0.04398701	2.297701893	-0.01914	0.984726299	NA	brown	-0.089599614	0.264441534		AT1G69230.1	SP1L2	SPIRAL1-like2	hypothetical protein
Sapur.001G001000.1	0.005114485	0.05557013	2.904824396	0.01913	0.984737169	NA	brown	-0.081608864	0.309590942		AT1G55535.1			transmembrane protein, putative
Sapur.001G072500.2	0.004765175	0.05557013	2.904824396	0.01913	0.984737169	NA	brown	-0.081608864	0.309590942	GO:0003677,GO:0009725,GO:0006355,GO:0005634	AT4G23980.1	ARF9	auxin response factor 9	auxin response factor
Sapur.001G093600.2	0.005114485	0.05557013	2.904824396	0.01913	0.984737169	NA	brown	-0.081608864	0.309590942	GO:0060236,GO:0032147,GO:0005874,GO:0005819	AT4G22860.1		Cell cycle regulated microtubule associated protein	cell cycle regulated microtubule-associated protein
Sapur.001G107000.1	0.005114485	0.05557013	2.904824396	0.01913	0.984737169	NA	brown	-0.081608864	0.309590942					hypothetical protein
Sapur.001G107800.2	0.005114485	0.05557013	2.904824396	0.01913	0.984737169	NA	brown	-0.081608864	0.309590942		AT5G45410.2			transmembrane protein, putative
Sapur.001G115300.1	0.005114485	0.05557013	2.904824396	0.01913	0.984737169	NA	brown	-0.081608864	0.309590942	GO:0005515,GO:0016567,GO:0004842	AT1G32340.1	NHL8	NDR1/HIN1-like 8	E3 ubiquitin-protein ligase RNF14
Sapur.001G119600.2	0.005733648	0.05557013	2.904824396	0.01913	0.984737169	NA	brown	-0.081608864	0.309590942	GO:0006355,GO:0003677	AT4G17980.1	anac071,NAC071	NAC domain containing protein 71	NAC transcription factor 045
Sapur.001G141600.1	0.005733648	0.05557013	2.904824396	0.01913	0.984737169	NA	brown	-0.1168153	0.145119686		AT4G19160.2			transglutaminase family protein
Sapur.001G145000.2	0.005114485	0.05557013	2.904824396	0.01913	0.984737169	NA	brown	-0.081608864	0.309590942	GO:0005515	AT1G15420.1			ubiquitin-like Rad60 SUMO-like protein
Sapur.001G150000.2	0.006431396	0.05557013	2.904824396	0.01913	0.984737169	NA	brown	-0.081608864	0.309590942		AT3G13890.2	ATMYB26,MS35,MYB26	myb domain protein 26	R2R3-Myb transcription factor, putative
Sapur.001G152300.1	0.005114485	0.05557013	2.904824396	0.01913	0.984737169	NA	brown	-0.081608864	0.309590942					hypothetical protein
Sapur.001G157800.1	0.005114485	0.05557013	2.904824396	0.01913	0.984737169	NA	brown	-0.081608864	0.309590942					NA
Sapur.001G158800.1	0.005114485	0.05557013	2.904824396	0.01913	0.984737169	NA	brown	-0.081608864	0.309590942					NA
Sapur.001G161500.4	0.005114485	0.05557013	2.904824396	0.01913	0.984737169	NA	brown	-0.081608864	0.309590942	GO:0006355,GO:0005634	AT1G51950.1	IAA18	indole-3-acetic acid inducible 18	auxin-responsive protein IAA26
Sapur.001G165800.2	0.005114485	0.05557013	2.904824396	0.01913	0.984737169	NA	brown	-0.081608864	0.309590942		AT1G79830.2	GC5	golgin candidate 5	coiled-coil vesicle tethering protein, putative
Sapur.001G174000.4	0.005114485	0.05557013	2.904824396	0.01913	0.984737169	NA	brown	-0.081608864	0.309590942		AT1G71240.1		Plant protein of unknown function (DUF639)	transmembrane protein, putative
Sapur.002G002300.1	0.005890737	0.05557013	2.904824396	0.01913	0.984737169	NA	brown	-0.081608864	0.309590942		AT1G76500.1	AHL29,SOB3	Predicted AT-hook DNA-binding family protein	DNA-binding protein, putative
Sapur.002G006500.6	0.005114485	0.05557013	2.904824396	0.01913	0.984737169	NA	brown	-0.081608864	0.309590942	GO:0005634	AT1G76310.1	CYCB2;4	CYCLIN B2;4	NA
Sapur.002G023100.2	0.005114485	0.05557013	2.904824396	0.01913	0.984737169	NA	brown	-0.081608864	0.309590942	GO:0006355,GO:0003677	AT1G75430.1	BLH11	BEL1-like homeodomain 11	BEL1 homeotic protein, putative
Sapur.002G039500.2	0.006428371	0.05557013	2.904824396	0.01913	0.984737169	NA	brown	-0.017065582	0.831999502		AT3G22550.1		Protein of unknown function (DUF581)	senescence-associated protein SAG102
Sapur.002G055000.5	0.007234977	0.05557013	2.904824396	0.01913	0.984737169	NA	brown	-0.081608864	0.309590942	GO:0017069,GO:0000398	AT1G21320.2		nucleotide binding;nucleic acid binding	RNA recognition motif, a.k.a. RRM%2C RBD protein
Sapur.002G061900.3	0.005114485	0.05557013	2.904824396	0.01913	0.984737169	NA	brown	-0.081608864	0.309590942	GO:0016020,GO:0015171,GO:0003333	AT2G01170.1	BAT1	bidirectional amino acid transporter 1	amino acid permease
Sapur.002G061900.2	0.005114485	0.05557013	2.904824396	0.01913	0.984737169	NA	brown	-0.081608864	0.309590942	GO:0055085,GO:0016020,GO:0006810,GO:0015171,GO:0003333	AT2G01170.1	BAT1	bidirectional amino acid transporter 1	amino acid permease
Sapur.002G073400.4	0.005937058	0.05557013	2.904824396	0.01913	0.984737169	NA	brown	-0.081608864	0.309590942		AT5G02130.1	NDP1	Tetratricopeptide repeat (TPR)-like superfamily protein	transmembrane protein, putative
Sapur.002G087100.3	0.005114485	0.05557013	2.904824396	0.01913	0.984737169	NA	brown	-0.081608864	0.309590942	GO:0046983	AT5G65640.1	bHLH093	beta HLH protein 93	transcription factor BHLH protein
Sapur.002G087500.2	0.005114485	0.05557013	2.904824396	0.01913	0.984737169	NA	brown	-0.081608864	0.309590942		AT3G28670.1		oxidoreductase, zinc-binding dehydrogenase family protein	zinc-binding dehydrogenase, putative
Sapur.002G100600.2	0.005114485	0.05557013	2.904824396	0.01913	0.984737169	NA	brown	-0.055945801	0.486464866		AT3G57870.1	AHUS5,ATSCE1,EMB1637,S	sumo conjugation enzyme 1	SUMO E2 conjugating enzyme SCE1
Sapur.002G102000.3	0.005114485	0.05557013	2.904824396	0.01913	0.984737169	NA	brown	-0.081608864	0.309590942		AT1G45207.2		Remorin family protein	remorin
Sapur.002G106600.1	0.006431396	0.05557013	2.904824396	0.01913	0.984737169	NA	brown	-0.114846535	0.152067369	GO:0006468,GO:0004672	AT3G13690.1		Protein kinase protein with adenine nucleotide alpha hydrolases-like domain	receptor tyrosine kinase
Sapur.002G106900.2	0.005890737	0.05557013	2.904824396	0.01913	0.984737169	NA	brown	-0.081608864	0.309590942	GO:0006468,GO:0005524,GO:0004672	AT2G23450.1		Protein kinase superfamily protein	tyrosine kinase domain protein
Sapur.002G118400.2	0.005114485	0.05557013	2.904824396	0.01913	0.984737169	NA	brown	-0.081608864	0.309590942	GO:0016021,GO:0006888,GO:0005801,GO:0000139	AT2G45200.1	ATGOS12,GOS12	golgi snare 12	SNAP receptor complex protein
Sapur.002G133400.4	0.004880298	0.05557013	2.904824396	0.01913	0.984737169	NA	brown	-0.081608864	0.309590942	GO:0055085,GO:0016021	AT2G48020.2		Major facilitator superfamily protein	sugar transporter ERD6-like protein
Sapur.002G156900.1	0.005114485	0.05557013	2.904824396	0.01913	0.984737169	NA	brown	-0.081608864	0.309590942		AT4G02425.1			hypothetical protein
Sapur.002G181500.2	0.005114485	0.05557013	2.904824396	0.01913	0.984737169	NA	brown	-0.081608864	0.309590942	GO:0005737,GO:0005094	AT3G07880.1	SCN1	Immunoglobulin E-set superfamily protein	Rho GDP-dissociation inhibitor
Sapur.002G182600.2	0.005114485	0.05557013	2.904824396	0.01913	0.984737169	NA	brown	-0.081608864	0.309590942		AT3G23740.1			hypothetical protein
Sapur.002G188200.2	0.00651968	0.05557013	2.904824396	0.01913	0.984737169	NA	brown	-0.072876299	0.364381813		AT4G13630.2		Protein of unknown function, DUF593	zein-binding protein
Sapur.002G192000.6	0.005114485	0.05557013	2.904824396	0.01913	0.984737169	NA	brown	-0.081608864	0.309590942	GO:0006468,GO:0005524,GO:0004672	AT4G13020.4	MHK	Protein kinase superfamily protein	cyclin-dependent kinase-like Serine/Threonine kinase family protein
Sapur.002G199900.2	0.00593829	0.05557013	2.904824396	0.01913	0.984737169	NA	brown	-0.081608864	0.309590942	GO:0006355,GO:0005634	AT4G28640.2	IAA11	indole-3-acetic acid inducible 11	indoleacetic acid-induced protein
Sapur.002G202600.2	0.005114485	0.05557013	2.904824396	0.01913	0.984737169	NA	brown	-0.081608864	0.309590942	GO:0055085,GO:0016021	AT1G75220.1		Major facilitator superfamily protein	sugar transporter, putative
Sapur.003G026100.2	0.005114485	0.05557013	2.904824396	0.01913	0.984737169	NA	brown	-0.081608864	0.309590942	GO:0008152,GO:0003824	AT3G16190.1		Isochorismatase family protein	isochorismatase hydrolase family protein
Sapur.003G028300.2	0.005114485	0.05557013	2.904824396	0.01913	0.984737169	NA	brown	-0.081608864	0.309590942	GO:0008601,GO:0000159	AT1G17720.2	ATB BETA	Protein phosphatase 2A, regulatory subunit PR55	serine/threonine protein phosphatase 2A 55 kDa regulatory subunit B beta
Sapur.003G051300.1	0.005114485	0.05557013	2.904824396	0.01913	0.984737169	NA	brown	-0.081608864	0.309590942	GO:0043565,GO:0008270,GO:0006355,GO:0003700	AT4G17570.3	GATA26	GATA transcription factor 26	GATA transcription factor, putative
Sapur.003G053500.2	0.005114485	0.05557013	2.904824396	0.01913	0.984737169	NA	brown	-0.081608864	0.309590942	GO:0016788	AT4G17100.2			endoribonuclease XendoU protein
Sapur.003G057000.3	0.005114485	0.05557013	2.904824396	0.01913	0.984737169	NA	brown	-0.081608864	0.309590942		AT1G32470.1		Single hybrid motif superfamily protein	glycine cleavage system H protein
Sapur.003G057900.2	0.006073616	0.05557013	2.904824396	0.01913	0.984737169	NA	brown	-0.081608864	0.309590942	GO:0005515	AT4G35470.1	PIRL4	plant intracellular ras group-related LRR 4	plant intracellular Ras-group-related LRR protein
Sapur.003G077000.5	0.005011262	0.05557013	2.904824396	0.01913	0.984737169	NA	brown	-0.081608864	0.309590942		AT4G22540.1	ORP2A	OSBP(oxysterol binding protein)-related protein 2A	oxysterol-binding protein-related protein
Sapur.003G152100.7	0.005114485	0.05557013	2.904824396	0.01913	0.984737169	NA	brown	-0.081608864	0.309590942					hypothetical protein
Sapur.003G159600.3	0.005114485	0.05557013	2.904824396	0.01913	0.984737169	NA	brown	-0.081608864	0.309590942		AT1G73730.1	AtEIL3,ATSLIM,EIL3,SLIM1	ETHYLENE-INSENSITIVE3-like 3	ethylene insensitive (EIN3/EIL)-like transcription regulator
Sapur.004G001600.1	0.005114485	0.05557013	2.904824396	0.01913	0.984737169	NA	brown	-0.081608864	0.309590942	GO:0055114,GO:0016491	AT1G05010.1	ACO4,EAT1,EFE	ethylene-forming enzyme	1-aminocyclopropane-1-carboxylate oxidase
Sapur.004G010500.2	0.005114485	0.05557013	2.904824396	0.01913	0.984737169	NA	brown	-0.081608864	0.309590942	GO:0005515	AT4G21820.1		binding;calmodulin binding	abnormal spindle-like microcephaly-associated-like protein, putative
Sapur.004G011000.1	0.005114485	0.05557013	2.904824396	0.01913	0.984737169	NA	brown	-0.081608864	0.309590942	GO:0016780,GO:0016020,GO:0008654	AT4G04870.1	CLS	cardiolipin synthase	phosphatidylglycerotransferase, putative
Sapur.004G020400.2	0.005114485	0.05557013	2.904824396	0.01913	0.984737169	NA	brown	-0.081608864	0.309590942	GO:0046854	AT4G05090.1		Inositol monophosphatase family protein	inositol monophosphatase family protein
Sapur.004G021700.4	0.006179623	0.05557013	2.904824396	0.01913	0.984737169	NA	brown	-0.081608864	0.309590942	GO:0016887,GO:0005524,GO:0055085,GO:0042626,GO:0016021,GO:0006810	AT1G30410.1	ATMRP13,MRP13	multidrug resistance-associated protein 13	NA
Sapur.004G029300.2	0.005114485	0.05557013	2.904824396	0.01913	0.984737169	NA	brown	-0.081608864	0.309590942		AT4G32480.1		Protein of unknown function (DUF506)	plant/F8B4-180 protein, putative
Sapur.004G041900.2	0.005114485	0.05557013	2.904824396	0.01913	0.984737169	NA	brown	-0.081608864	0.309590942		AT1G29270.1			hypothetical protein
Sapur.004G087800.2	0.005114485	0.05557013	2.904824396	0.01913	0.984737169	NA	brown	-0.081608864	0.309590942		AT1G72160.1		Sec14p-like phosphatidylinositol transfer family protein	NA
Sapur.004G088800.2	0.005114485	0.05557013	2.904824396	0.01913	0.984737169	NA	brown	-0.081608864	0.309590942	GO:0055114,GO:0020037,GO:0016705,GO:0005506	AT3G19270.1	CYP707A4	cytochrome P450, family 707, subfamily A, polypeptide 4	cytochrome P450 family 707 ABA 8'-hydroxylase
Sapur.004G091400.2	0.005114485	0.05557013	2.904824396	0.01913	0.984737169	NA	brown	-0.081608864	0.309590942		AT4G33945.1		ARM repeat superfamily protein	armadillo/beta-catenin-like repeat protein
Sapur.004G105100.2	0.005937058	0.05557013	2.904824396	0.01913	0.984737169	NA	brown	-0.081608864	0.309590942		AT3G28880.1		Ankyrin repeat family protein	NA
Sapur.004G105800.4	0.005114485	0.05557013	2.904824396	0.01913	0.984737169	NA	brown	-0.081608864	0.309590942		AT5G23610.2			DYAD protein, putative
Sapur.004G119900.2	0.005114485	0.05557013	2.904824396	0.01913	0.984737169	NA	brown	-0.081608864	0.309590942	GO:0005975,GO:0004553	AT4G36360.1	BGAL3	beta-galactosidase 3	beta-galactosidase
Sapur.004G133100.1	0.005114485	0.05557013	2.904824396	0.01913	0.984737169	NA	brown	-0.081608864	0.309590942	GO:0071949	AT5G05320.1		FAD/NAD(P)-binding oxidoreductase family protein	FAD-binding domain protein
Sapur.004G155800.2	0.005114485	0.05557013	2.904824396	0.01913	0.984737169	NA	brown	-0.081608864	0.309590942		AT4G35360.1		Uncharacterised conserved protein (UCP030210)	pantothenate kinase
Sapur.004G162700.1	0.006085689	0.05557013	2.904824396	0.01913	0.984737169	NA	brown	-0.081608864	0.309590942					NA
Sapur.005G020500.9	0.005114485	0.05557013	2.904824396	0.01913	0.984737169	NA	brown	-0.081608864	0.309590942		AT1G09060.3		Zinc finger, RING-type;Transcription factor jumonji/aspartyl beta-hydroxylase	transcription factor jumonji domain protein
Sapur.005G034400.2	0.00602393	0.05557013	2.904824396	0.01913	0.984737169	NA	brown	-0.038330437	0.633635406	GO:0006468,GO:0005524,GO:0004672	AT1G54610.1		Protein kinase superfamily protein	cyclin-dependent kinase-like Serine/Threonine kinase family protein
Sapur.005G045100.2	0.005114485	0.05557013	2.904824396	0.01913	0.984737169	NA	brown	-0.081608864	0.309590942	GO:0016773,GO:0048015,GO:0046854,GO:0006661,GO:0004430	AT5G64070.1	PI-4KBETA1,PI4KBETA1	phosphatidylinositol 4-OH kinase beta1	phosphatidylinositol 4-kinase, putative
Sapur.005G045200.1	0.006444464	0.05557013	2.904824396	0.01913	0.984737169	NA	brown	-0.081608864	0.309590942					chitinase
Sapur.005G048600.5	0.005114485	0.05557013	2.904824396	0.01913	0.984737169	NA	brown	-0.081608864	0.309590942	GO:0016624,GO:0008152	AT5G09300.1		Thiamin diphosphate-binding fold (THDP-binding) superfamily protein	2-oxoisovalerate dehydrogenase alpha subunit, putative
Sapur.005G052000.2	0.005114485	0.05557013	2.904824396	0.01913	0.984737169	NA	brown	-0.081608864	0.309590942	GO:0006629	AT3G52430.1	ATPAD4,PAD4	alpha/beta-Hydrolases superfamily protein	NA
Sapur.005G070600.2	0.005114485	0.05557013	2.904824396	0.01913	0.984737169	NA	brown	-0.081608864	0.309590942	GO:0055114,GO:0046872,GO:0006801,GO:0004784	AT5G23310.1	FSD3	Fe superoxide dismutase 3	iron superoxide dismutase
Sapur.005G088200.2	0.009759838	0.05557013	2.904824396	0.01913	0.984737169	NA	brown	-0.081608864	0.309590942	GO:0008270,GO:0005515	AT2G26000.2	BRIZ2	zinc finger (C3HC4-type RING finger) family protein	ubiquitin carboxyl-terminal hydrolase
Sapur.005G114900.1	0.006083092	0.05557013	2.904824396	0.01913	0.984737169	NA	brown	-0.081608864	0.309590942	GO:0043565,GO:0006355,GO:0003700,GO:0003677	AT4G40060.1	ATHB-16,ATHB16,HB16	homeobox protein 16	homeobox leucine zipper protein
Sapur.005G124900.1	0.005114485	0.05557013	2.904824396	0.01913	0.984737169	NA	brown	-0.081608864	0.309590942	GO:0016021	AT4G39220.1	ATRER1A	Rer1 family protein	RER1A protein
Sapur.005G126700.1	0.005114485	0.05557013	2.904824396	0.01913	0.984737169	NA	brown	0.107160336	0.181603109		AT3G28370.1			NA
Sapur.005G128900.1	0.005114485	0.05557013	2.904824396	0.01913	0.984737169	NA	brown	-0.081608864	0.309590942		AT1G34670.1	AtMYB93,MYB93	myb domain protein 93	transcription factor MYB39, putative
Sapur.005G137900.5	0.005114485	0.05557013	2.904824396	0.01913	0.984737169	NA	brown	-0.081608864	0.309590942		AT1G44780.2			histone chaperone domain CHZ protein
Sapur.005G140900.2	0.005114485	0.05557013	2.904824396	0.01913	0.984737169	NA	brown	-0.081608864	0.309590942		AT3G63420.2	AGG1,ATAGG1,GG1	Ggamma-subunit 1	hypothetical protein
Sapur.005G144500.3	0.005114485	0.05557013	2.904824396	0.01913	0.984737169	NA	brown	-0.081608864	0.309590942	GO:0005515	AT1G77250.1		RING/FYVE/PHD-type zinc finger family protein	PHD-finger protein
Sapur.005G161400.1	0.005114485	0.05557013	2.904824396	0.01913	0.984737169	NA	brown	-0.081608864	0.309590942	GO:0003723,GO:0008168	AT1G06560.1		NOL1/NOP2/sun family protein	ribosomal RNA small subunit methyltransferase B
Sapur.005G169700.4	0.005114485	0.05557013	2.904824396	0.01913	0.984737169	NA	brown	-0.081608864	0.309590942		AT4G14720.1	PPD2,TIFY4B	TIFY domain/Divergent CCT motif family protein	jasmonate ZIM domain protein K.2
Sapur.005G181300.1	0.005114485	0.05557013	2.904824396	0.01913	0.984737169	NA	brown	-0.081608864	0.309590942	GO:0007034	AT1G03950.1	VPS2.3	vacuolar protein sorting-associated protein 2.3	charged multivesicular body protein 2a, putative
Sapur.005G195200.1	0.005114485	0.05557013	2.904824396	0.01913	0.984737169	NA	brown	-0.105883216	0.186893673	GO:0043565,GO:0006355,GO:0003700	AT4G35900.1	atbzip14,FD,FD-1	Basic-leucine zipper (bZIP) transcription factor family protein	basic leucine zipper transcription factor
Sapur.005G202000.1	0.005114485	0.05557013	2.904824396	0.01913	0.984737169	NA	brown	-0.081608864	0.309590942	GO:0006468,GO:0005524,GO:0004672	AT1G20650.1		Protein kinase superfamily protein	Serine/Threonine kinase domain protein
Sapur.006G008500.2	0.005114485	0.05557013	2.904824396	0.01913	0.984737169	NA	brown	-0.081608864	0.309590942		AT5G20130.1			transmembrane protein, putative
Sapur.006G008600.1	0.005114485	0.05557013	2.904824396	0.01913	0.984737169	NA	brown	-0.081608864	0.309590942	GO:0005515	AT4G22360.1		SWIB complex BAF60b domain-containing protein	Brg-1 associated factor, putative
Sapur.006G028600.1	0.005114485	0.05557013	2.904824396	0.01913	0.984737169	NA	brown	-0.081608864	0.309590942	GO:0008017,GO:0007010	AT1G24764.1	ATMAP70-2,MAP70-2	microtubule-associated proteins 70-2	microtubule-associated protein 70-2
Sapur.006G035300.3	0.005114485	0.05557013	2.904824396	0.01913	0.984737169	NA	brown	-0.081608864	0.309590942	GO:0005509	AT3G10300.4		Calcium-binding EF-hand family protein	EF hand protein
Sapur.006G042500.1	0.005114485	0.05557013	2.904824396	0.01913	0.984737169	NA	brown	-0.081608864	0.309590942	GO:0016746,GO:0008152	AT3G57650.1	LPAT2	lysophosphatidyl acyltransferase 2	1-acyl-sn-glycerol-3-phosphate acyltransferase
Sapur.006G043800.2	0.005114485	0.05557013	2.904824396	0.01913	0.984737169	NA	brown	-0.081608864	0.309590942		AT3G12170.1		Chaperone DnaJ-domain superfamily protein	chaperone protein DnaJ
Sapur.006G070600.5	0.005114485	0.05557013	2.904824396	0.01913	0.984737169	NA	brown	-0.081608864	0.309590942		AT5G02250.1	ATMTRNASEII,EMB2730,RN	Ribonuclease II/R family protein	ribonuclease II
Sapur.006G073700.1	0.005114485	0.05557013	2.904824396	0.01913	0.984737169	NA	brown	-0.081608864	0.309590942		AT2G31280.1	CPUORF7	conserved peptide upstream open reading frame 7	bHLH-MYC/R2R3-MYB transcription factor amino-terminal protein
Sapur.006G080400.1	0.005114485	0.05557013	2.904824396	0.01913	0.984737169	NA	brown	-0.081608864	0.309590942	GO:0016020,GO:0006810,GO:0005215	AT4G00430.1	PIP1;4,PIP1E,TMP-C	plasma membrane intrinsic protein 1;4	plasma membrane intrinsic protein PIP1-1
Sapur.006G085800.2	0.005114485	0.05557013	2.904824396	0.01913	0.984737169	NA	brown	-0.081608864	0.309590942		AT3G51670.1		SEC14 cytosolic factor family protein / phosphoglyceride transfer family protein	patellin-6 protein
Sapur.006G092300.1	0.005114485	0.05557013	2.904824396	0.01913	0.984737169	NA	brown	-0.081608864	0.309590942		AT3G54190.1		Transducin/WD40 repeat-like superfamily protein	plant/F24B22-150 protein
Sapur.006G092300.4	0.005114485	0.05557013	2.904824396	0.01913	0.984737169	NA	brown	-0.081608864	0.309590942		AT2G38630.1		Transducin/WD40 repeat-like superfamily protein	plant/F24B22-150 protein
Sapur.006G118400.7	0.005114485	0.05557013	2.904824396	0.01913	0.984737169	NA	brown	-0.081608864	0.309590942	GO:0032502,GO:0006351,GO:0005634	AT3G13960.1	AtGRF5,GRF5	growth-regulating factor 5	QLQ protein
Sapur.006G150100.2	0.005114485	0.05557013	2.904824396	0.01913	0.984737169	NA	brown	-0.081608864	0.309590942	GO:0006508,GO:0004185	AT4G30610.1	BRS1,SCPL24	alpha/beta-Hydrolases superfamily protein	serine carboxypeptidase
Sapur.006G181100.1	0.006083092	0.05557013	2.904824396	0.01913	0.984737169	NA	brown	-0.081608864	0.309590942	GO:0005515,GO:0006468,GO:0005524,GO:0004672	AT2G26330.1	ER,QRP1	Leucine-rich receptor-like protein kinase family protein	LRR receptor-like Serine/Threonine-kinase
Sapur.006G200100.2	0.005114485	0.05557013	2.904824396	0.01913	0.984737169	NA	brown	-0.081608864	0.309590942	GO:0003723	AT4G32720.2	AtLa1,La1	La protein 1	lupus LA ribonucleoprotein, putative
Sapur.006G207700.1	0.005114485	0.05557013	2.904824396	0.01913	0.984737169	NA	brown	-0.081608864	0.309590942		AT5G25480.1	AtDNMT2,DNMT2	DNA methyltransferase-2	DNA (cytosine-5)-methyltransferase
Sapur.006G228300.8	0.004953155	0.05557013	2.904824396	0.01913	0.984737169	NA	brown	-0.081608864	0.309590942		AT2G30650.1		ATP-dependent caseinolytic (Clp) protease/crotonase family protein	3-hydroxyisobutyryl-CoA hydrolase
Sapur.007G001900.4	0.006598449	0.05557013	2.904824396	0.01913	0.984737169	NA	brown	-0.081608864	0.309590942	GO:0003824	AT1G78950.1		Terpenoid cyclases family protein	beta-amyrin synthase
Sapur.007G015700.1	0.005114485	0.05557013	2.904824396	0.01913	0.984737169	NA	brown	-0.081608864	0.309590942	GO:0055114,GO:0050660,GO:0016491,GO:0003824	AT4G36400.1		FAD-linked oxidases family protein	NA
Sapur.007G031200.2	0.00602393	0.05557013	2.904824396	0.01913	0.984737169	NA	brown	-0.081608864	0.309590942		AT4G37235.1		Uncharacterised protein family (UPF0497)	transmembrane protein, putative
Sapur.007G032500.2	0.006085689	0.05557013	2.904824396	0.01913	0.984737169	NA	brown	-0.081608864	0.309590942		AT3G50380.1		Protein of unknown function (DUF1162)	Pol-like polyprotein/retrotransposon, putative
Sapur.007G071000.3	0.005114485	0.05557013	2.904824396	0.01913	0.984737169	NA	brown	-0.081608864	0.309590942	GO:0030244,GO:0016760,GO:0016020	AT5G64740.1	CESA6,E112,IXR2,PRC1	cellulose synthase 6	cellulose synthase A catalytic subunit 5 [UDP-forming] protein
Sapur.007G103700.1	0.005114485	0.05557013	2.904824396	0.01913	0.984737169	NA	brown	-0.081608864	0.309590942		AT1G61000.1			NA
Sapur.007G106400.1	0.006444464	0.05557013	2.904824396	0.01913	0.984737169	NA	brown	-0.081608864	0.309590942					B3 DNA-binding domain protein
Sapur.007G108500.6	0.005114485	0.05557013	2.904824396	0.01913	0.984737169	NA	brown	-0.081608864	0.309590942		AT1G05270.1		TraB family protein	TraB family protein
Sapur.007G128600.1	0.004953155	0.05557013	2.904824396	0.01913	0.984737169	NA	brown	-0.081608864	0.309590942					NA
Sapur.008G006700.6	0.005114485	0.05557013	2.904824396	0.01913	0.984737169	NA	brown	-0.081608864	0.309590942	GO:0008270,GO:0006355	AT2G27110.2	FRS3	FAR1-related sequence 3	FAR1-related sequence protein
Sapur.008G011800.2	0.006165205	0.05557013	2.904824396	0.01913	0.984737169	NA	brown	-0.081608864	0.309590942		AT5G04840.1		bZIP protein	BZIP protein, putative
Sapur.008G013100.2	0.006431396	0.05557013	2.904824396	0.01913	0.984737169	NA	brown	-0.081608864	0.309590942	GO:0004523,GO:0003723	AT2G25100.1		Polynucleotidyl transferase, ribonuclease H-like superfamily protein	ribonuclease H
Sapur.008G017700.1	0.004953155	0.05557013	2.904824396	0.01913	0.984737169	NA	brown	-0.081608864	0.309590942		AT3G57810.3		Cysteine proteinases superfamily protein	OTU-like cysteine protease
Sapur.008G022500.1	0.005114485	0.05557013	2.904824396	0.01913	0.984737169	NA	brown	-0.081608864	0.309590942	GO:0005515	AT5G04420.2		Galactose oxidase/kelch repeat superfamily protein	acyl-CoA-binding domain protein
Sapur.008G024500.2	0.005907325	0.05557013	2.904824396	0.01913	0.984737169	NA	brown	-0.081608864	0.309590942	GO:0007034	AT5G22950.1	VPS24.1	SNF7 family protein	charged multivesicular body protein
Sapur.008G033800.9	0.006073616	0.05557013	2.904824396	0.01913	0.984737169	NA	brown	-0.081608864	0.309590942		AT2G37060.1	NF-YB8	nuclear factor Y, subunit B8	nuclear transcription factor Y subunit B-1
Sapur.008G042700.2	0.005114485	0.05557013	2.904824396	0.01913	0.984737169	NA	brown	-0.081608864	0.309590942	GO:0006355,GO:0003677	AT3G55370.2	OBP3	OBF-binding protein 3	Dof zinc finger protein
Sapur.008G044300.5	0.007810412	0.05557013	2.904824396	0.01913	0.984737169	NA	brown	-0.081608864	0.309590942	GO:0003676	AT3G13570.1	SCL30A	SC35-like splicing factor 30A	RNA recognition motif, a.k.a. RRM%2C RBD protein
Sapur.008G053100.3	0.005114485	0.05557013	2.904824396	0.01913	0.984737169	NA	brown	-0.081608864	0.309590942	GO:0006355,GO:0005634,GO:0003682,GO:0003677	AT3G51880.2	HMGB1,NFD1	high mobility group B1	NA
Sapur.008G053100.1	0.005114485	0.05557013	2.904824396	0.01913	0.984737169	NA	brown	-0.081608864	0.309590942	GO:0006355,GO:0005634,GO:0003682,GO:0003677	AT3G51880.2	HMGB1,NFD1	high mobility group B1	NA
Sapur.008G053800.3	0.005890737	0.05557013	2.904824396	0.01913	0.984737169	NA	brown	-0.081608864	0.309590942		AT3G55490.1		GINS complex protein	DNA replication complex GINS protein Psf3
Sapur.008G055000.2	0.005114485	0.05557013	2.904824396	0.01913	0.984737169	NA	brown	-0.081608864	0.309590942	GO:0006629,GO:0055114,GO:0016717	AT5G05580.1	FAD8	fatty acid desaturase 8	omega-3 fatty acid desaturase
Sapur.008G067700.1	0.005114485	0.05557013	2.904824396	0.01913	0.984737169	NA	brown	-0.081608864	0.309590942		AT1G69600.1	ATHB29,ZFHD1	zinc finger homeodomain 1	ZF-HD homeobox domain protein
Sapur.008G077700.8	0.006083092	0.05557013	2.904824396	0.01913	0.984737169	NA	brown	-0.081608864	0.309590942	GO:0006355,GO:0005634,GO:0003700	AT1G69120.1	AGL7,AP1	K-box region and MADS-box transcription factor family protein	MADS-box transcription factor APETALA 1
Sapur.008G084900.2	0.005114485	0.05557013	2.904824396	0.01913	0.984737169	NA	brown	-0.081608864	0.309590942	GO:0008270,GO:0006351,GO:0003676	AT3G16980.1	NRPB9A,NRPD9A,NRPE9A	RNA polymerases M/15 Kd subunit	DNA-directed RNA polymerase subunit
Sapur.008G092600.2	0.005114485	0.05557013	2.904824396	0.01913	0.984737169	NA	brown	-0.081608864	0.309590942	GO:0016021	AT1G26100.1		Cytochrome b561/ferric reductase transmembrane protein family	cytochrome protein b561, putative
Sapur.008G107100.2	0.005114485	0.05557013	2.904824396	0.01913	0.984737169	NA	brown	-0.081608864	0.309590942		AT1G23170.2		Protein of unknown function DUF2359, transmembrane	hypothetical protein
Sapur.008G119700.1	0.005114485	0.05557013	2.904824396	0.01913	0.984737169	NA	brown	-0.081608864	0.309590942	GO:0043565,GO:0006355,GO:0003700,GO:0003677	AT1G69780.1	ATHB13	Homeobox-leucine zipper protein family	homeobox leucine zipper protein
Sapur.008G136200.4	0.00347127	0.05557013	2.904824396	0.01913	0.984737169	NA	brown	0.078548531	0.32814497	GO:0006468,GO:0005524,GO:0004674,GO:0004672	AT3G23310.1		AGC (cAMP-dependent, cGMP-dependent and protein kinase C) kinase family protein	AGC (cAMP-dependent, cGMP-dependent and kinase C) kinase family protein
Sapur.008G143900.2	0.005114485	0.05557013	2.904824396	0.01913	0.984737169	NA	brown	-0.081608864	0.309590942	GO:0055114,GO:0046872,GO:0016702	AT1G67560.1		PLAT/LH2 domain-containing lipoxygenase family protein	lipoxygenase
Sapur.008G152200.2	0.006910738	0.05557013	2.904824396	0.01913	0.984737169	NA	brown	-0.098639342	0.219045351	GO:0005975,GO:0004650	AT1G60590.1		Pectin lyase-like superfamily protein	polygalacturonase
Sapur.009G006900.3	0.005114485	0.05557013	2.904824396	0.01913	0.984737169	NA	brown	-0.081608864	0.309590942	GO:0016021,GO:0008146	AT1G08030.1	TPST	tyrosylprotein sulfotransferase	tyrosine sulfotransferase
Sapur.009G028400.2	0.005114485	0.05557013	2.904824396	0.01913	0.984737169	NA	brown	-0.081608864	0.309590942		AT5G02020.2			hypothetical protein
Sapur.009G029200.2	0.005114485	0.05557013	2.904824396	0.01913	0.984737169	NA	brown	-0.081608864	0.309590942		AT5G59020.1		Protein of unknown function (DUF3527)	DUF3527 domain protein
Sapur.009G050000.2	0.005937058	0.05557013	2.904824396	0.01913	0.984737169	NA	brown	-0.081608864	0.309590942	GO:0046983	AT2G42280.1		basic helix-loop-helix (bHLH) DNA-binding superfamily protein	transcription factor bHLH122, putative
Sapur.009G050600.3	0.005114485	0.05557013	2.904824396	0.01913	0.984737169	NA	brown	-0.081608864	0.309590942	GO:0051082,GO:0031072	AT5G48030.1	GFA2	gametophytic factor 2	chaperone protein DnaJ
Sapur.009G058300.3	0.005114485	0.05557013	2.904824396	0.01913	0.984737169	NA	brown	-0.081608864	0.309590942		AT5G58560.1		Phosphatidate cytidylyltransferase family protein	phytol kinase, putative
Sapur.009G063000.5	0.005114485	0.05557013	2.904824396	0.01913	0.984737169	NA	brown	0.051190765	0.524313464		AT5G64460.4		Phosphoglycerate mutase family protein	phosphoglycerate mutase-like protein
Sapur.009G064900.1	0.005114485	0.05557013	2.904824396	0.01913	0.984737169	NA	brown	-0.126802523	0.113528777	GO:0008152,GO:0003824	AT1G50110.1		D-aminoacid aminotransferase-like PLP-dependent enzymes superfamily protein	branched-chain-amino-acid aminotransferase
Sapur.009G071100.4	0.005114485	0.05557013	2.904824396	0.01913	0.984737169	NA	brown	-0.081608864	0.309590942	GO:0005515	AT3G43740.1		Leucine-rich repeat (LRR) family protein	somatic embryogenesis receptor kinase
Sapur.009G075200.3	0.005114485	0.05557013	2.904824396	0.01913	0.984737169	NA	brown	-0.081608864	0.309590942		AT2G15240.1		UNC-50 family protein	UNC-50
Sapur.009G076500.3	0.005114485	0.05557013	2.904824396	0.01913	0.984737169	NA	brown	-0.081608864	0.309590942	GO:0005515	AT4G34150.1		Calcium-dependent lipid-binding (CaLB domain) family protein	C2 domain protein
Sapur.009G089300.2	0.005832192	0.05557013	2.904824396	0.01913	0.984737169	NA	brown	-0.081608864	0.309590942		AT4G36800.2	RCE1	RUB1 conjugating enzyme 1	ubiquitin-conjugating enzyme
Sapur.009G119000.3	0.007245688	0.05557013	2.904824396	0.01913	0.984737169	NA	brown	-0.081608864	0.309590942	GO:0030131,GO:0016192,GO:0006886,GO:0030117	AT5G22770.1	alpha-ADR	alpha-adaptin	AP-2 complex subunit alpha, putative
Sapur.009G126600.4	0.009759838	0.05557013	2.904824396	0.01913	0.984737169	NA	brown	-0.081608864	0.309590942	GO:0006355,GO:0005667,GO:0003700	AT5G22220.2	ATE2FB,E2F1,E2FB	E2F transcription factor 1	transcription factor E2F
Sapur.010G002500.2	0.005114485	0.05557013	2.904824396	0.01913	0.984737169	NA	brown	-0.081608864	0.309590942		AT1G48320.1		Thioesterase superfamily protein	acyl-CoA thioesterase
Sapur.010G010000.1	0.005114485	0.05557013	2.904824396	0.01913	0.984737169	NA	brown	-0.149286102	0.062031195	GO:0008152,GO:0003824,GO:0016114,GO:0008661	AT4G15560.1	CLA,CLA1,DEF,DXPS2,DXS	Deoxyxylulose-5-phosphate synthase	1-deoxy-D-xylulose-5-phosphate synthase
Sapur.010G014700.1	0.005114485	0.05557013	2.904824396	0.01913	0.984737169	NA	brown	-0.081608864	0.309590942	GO:0005634,GO:0003677	AT5G18830.3	ATSPL7,SPL7	squamosa promoter binding protein-like 7	squamosa promoter-binding-like protein
Sapur.010G020300.1	0.005114485	0.05557013	2.904824396	0.01913	0.984737169	NA	brown	-0.081608864	0.309590942	GO:0034314,GO:0007015,GO:0005885,GO:0005524	AT1G13180.1	ARP3,ATARP3,DIS1	Actin-like ATPase superfamily protein	actin-related protein ARP3 subunit
Sapur.010G023100.2	0.005011262	0.05557013	2.904824396	0.01913	0.984737169	NA	brown	-0.081608864	0.309590942		AT1G67840.2	CSK	chloroplast sensor kinase	ATP-binding region, ATPase-like domain protein, putative
Sapur.010G024500.3	0.005114485	0.05557013	2.904824396	0.01913	0.984737169	NA	brown	-0.081608864	0.309590942		AT1G70630.1		Nucleotide-diphospho-sugar transferase family protein	NA
Sapur.010G031000.2	0.005114485	0.05557013	2.904824396	0.01913	0.984737169	NA	brown	-0.081608864	0.309590942		AT1G70070.1	EMB25,ISE2,PDE317	DEAD/DEAH box helicase, putative	DEAD-box ATP-dependent RNA helicase ISE2
Sapur.010G057000.7	0.005114485	0.05557013	2.904824396	0.01913	0.984737169	NA	brown	-0.081608864	0.309590942	GO:0008601,GO:0007165,GO:0000159	AT3G26020.2		Protein phosphatase 2A regulatory B subunit family protein	serine/threonine protein phosphatase 2A 59 kDa regulatory subunit B' protein
Sapur.010G076900.1	0.005114485	0.05557013	2.904824396	0.01913	0.984737169	NA	brown	-0.081608864	0.309590942	GO:0006468,GO:0005524,GO:0004672	AT1G24030.2		Protein kinase superfamily protein	Serine/Threonine kinase domain protein
Sapur.010G079600.1	0.005114485	0.05557013	2.904824396	0.01913	0.984737169	NA	brown	-0.081608864	0.309590942		AT2G02820.2	AtMYB88,MYB88	myb domain protein 88	Myb-like DNA-binding domain protein
Sapur.010G080900.2	0.005114485	0.05557013	2.904824396	0.01913	0.984737169	NA	brown	-0.081608864	0.309590942	GO:0016787	AT2G01670.1	atnudt17,NUDT17	nudix hydrolase homolog 17	nudix hydrolase
Sapur.010G091800.3	0.005114485	0.05557013	2.904824396	0.01913	0.984737169	NA	brown	-0.083293387	0.299676877		AT2G01260.2		Protein of unknown function (DUF789)	DUF789 family protein
Sapur.010G109500.3	0.005114485	0.05557013	2.904824396	0.01913	0.984737169	NA	brown	-0.081608864	0.309590942	GO:0032040	AT1G69070.1			Nop14 family protein
Sapur.010G114000.1	0.005114485	0.05557013	2.904824396	0.01913	0.984737169	NA	brown	-0.081569362	0.309825971					hypothetical protein
Sapur.010G128200.1	0.005114485	0.05557013	2.904824396	0.01913	0.984737169	NA	brown	-0.081608864	0.309590942	GO:0006355	AT2G03150.1	emb1579	ATP/GTP-binding protein family	P30 dbc protein, putative
Sapur.010G136600.2	0.005011262	0.05557013	2.904824396	0.01913	0.984737169	NA	brown	-0.081608864	0.309590942	GO:0055114,GO:0016616,GO:0006102,GO:0004450	AT1G54340.1	ICDH	isocitrate dehydrogenase	isocitrate dehydrogenase [NADP] protein
Sapur.010G148700.1	0.004765175	0.05557013	2.904824396	0.01913	0.984737169	NA	brown	-0.081608864	0.309590942	GO:0033588	AT3G11220.1	ELO1	Paxneb protein-related	elongator component
Sapur.010G172300.6	0.005114485	0.05557013	2.904824396	0.01913	0.984737169	NA	brown	-0.081608864	0.309590942		AT2G37060.2	NF-YB8	nuclear factor Y, subunit B8	nuclear transcription factor Y subunit B-1
Sapur.010G195500.1	0.006444464	0.05557013	2.904824396	0.01913	0.984737169	NA	brown	-0.081608864	0.309590942	GO:0019538	AT3G52490.1		Double Clp-N motif-containing P-loop nucleoside triphosphate hydrolases superfamily protein	heat shock protein, putative
Sapur.011G002800.3	0.005114485	0.05557013	2.904824396	0.01913	0.984737169	NA	brown	-0.081608864	0.309590942	GO:0016020,GO:0008378,GO:0006486	AT1G05170.1		Galactosyltransferase family protein	beta-1%2C3-galactosyltransferase, putative
Sapur.011G008700.2	0.005114485	0.05557013	2.904824396	0.01913	0.984737169	NA	brown	-0.103717051	0.196123443		AT4G04330.1		Chaperonin-like RbcX protein	RbcX protein
Sapur.011G030700.5	0.005114485	0.05557013	2.904824396	0.01913	0.984737169	NA	brown	-0.081608864	0.309590942	GO:0005515,GO:0006468,GO:0004672	AT4G03390.1	SRF3	STRUBBELIG-receptor family 3	LRR receptor-like kinase, putative
Sapur.011G056100.1	0.005114485	0.05557013	2.904824396	0.01913	0.984737169	NA	brown	-0.081608864	0.309590942	GO:0006260,GO:0005664,GO:0003677	AT1G26840.1	ATORC6,ORC6	origin recognition complex protein 6	origin recognition complex subunit 6
Sapur.011G112300.2	0.005114485	0.05557013	2.904824396	0.01913	0.984737169	NA	brown	0.078548531	0.32814497		AT3G15850.1	ADS3,FAD5,FADB,JB67	fatty acid desaturase 5	palmitoyl-monogalactosyldiacylglycerol delta-7 desaturase, putative
Sapur.011G115200.2	0.005114485	0.05557013	2.904824396	0.01913	0.984737169	NA	brown	-0.081608864	0.309590942	GO:0009052,GO:0004751	AT5G44520.1		NagB/RpiA/CoA transferase-like superfamily protein	ribose-5-phosphate isomerase A
Sapur.011G116200.2	0.005114485	0.05557013	2.904824396	0.01913	0.984737169	NA	brown	-0.081608864	0.309590942	GO:0042254,GO:0005622	AT1G25260.1		Ribosomal protein L10 family protein	ribosomal protein L10
Sapur.012G042700.4	0.005907325	0.05557013	2.904824396	0.01913	0.984737169	NA	brown	-0.081608864	0.309590942		AT3G18520.2	ATHDA15,HDA15	histone deacetylase 15	histone deacetylase family protein
Sapur.012G076300.2	0.005114485	0.05557013	2.904824396	0.01913	0.984737169	NA	brown	-0.081608864	0.309590942	GO:0008641	AT4G24940.1	AT-SAE1-1,ATSAE1A,SAE1A	SUMO-activating enzyme 1A	SUMO-activating enzyme subunit 1A
Sapur.012G077100.1	0.006598449	0.05557013	2.904824396	0.01913	0.984737169	NA	brown	-0.081608864	0.309590942		AT5G50720.1	ATHVA22E,HVA22E	HVA22 homologue E	HVA22-like protein E
Sapur.012G077400.2	0.005114485	0.05557013	2.904824396	0.01913	0.984737169	NA	brown	-0.081608864	0.309590942		AT3G47350.2	ATHSD2,HSD2	hydroxysteroid dehydrogenase 2	short-chain dehydrogenase reductase
Sapur.013G008600.4	0.005114485	0.05557013	2.904824396	0.01913	0.984737169	NA	brown	-0.081608864	0.309590942	GO:0006415,GO:0003747,GO:0016149,GO:0005737	AT1G56350.1		Peptide chain release factor 2	peptide chain release factor 2
Sapur.013G012400.1	0.006044912	0.05557013	2.904824396	0.01913	0.984737169	NA	brown	-0.081608864	0.309590942		AT5G64620.1	ATC/VIF2,C/VIF2	cell wall / vacuolar inhibitor of fructosidase 2	pectinesterase inhibitor domain protein
Sapur.013G041100.1	0.005114485	0.05557013	2.904824396	0.01913	0.984737169	NA	brown	-0.081608864	0.309590942		AT1G54650.2		Methyltransferase family protein	methyltransferase domain protein
Sapur.013G069500.1	0.005114485	0.05557013	2.904824396	0.01913	0.984737169	NA	brown	-0.081608864	0.309590942		AT5G35405.1		Protein of unknown function (DUF784)	NA
Sapur.013G069600.1	0.005114485	0.05557013	2.904824396	0.01913	0.984737169	NA	brown	-0.081608864	0.309590942		AT5G35405.1		Protein of unknown function (DUF784)	NA
Sapur.013G074200.2	0.005114485	0.05557013	2.904824396	0.01913	0.984737169	NA	brown	-0.081608864	0.309590942	GO:0051082,GO:0015631,GO:0007021	AT2G30410.1	KIS,TFCA	tubulin folding cofactor A (KIESEL)	tubulin folding cofactor A
Sapur.013G082400.2	0.005114485	0.05557013	2.904824396	0.01913	0.984737169	NA	brown	-0.081608864	0.309590942	GO:0016987,GO:0006355,GO:0006352,GO:0003700,GO:0003677	AT1G64860.1	RPOD1,SIG1,SIG2,SIGA,SIGB	sigma factor A	RNA polymerase sigma factor
Sapur.013G091600.1	0.005907325	0.05557013	2.904824396	0.01913	0.984737169	NA	brown	-0.081608864	0.309590942	GO:0008270,GO:0005515	AT4G10150.1		RING/U-box superfamily protein	RING-H2 zinc finger protein
Sapur.013G131200.2	0.005890737	0.05557013	2.904824396	0.01913	0.984737169	NA	brown	-0.081608864	0.309590942	GO:0055114,GO:0016491	AT4G28220.1	NDB1	NAD(P)H dehydrogenase B1	external alternative NAD(P)H-ubiquinone oxidoreductase B1
Sapur.013G143300.1	0.005114485	0.05557013	2.904824396	0.01913	0.984737169	NA	brown	-0.081608864	0.309590942	GO:0005622,GO:0003676,GO:0005524,GO:0043140,GO:0006281,GO:0006260	AT1G60930.1	ATRECQ4B,RECQ4B,RECQL	RECQ helicase L4B	ATP-dependent DNA helicase RecQ
Sapur.014G014600.3	0.005114485	0.05557013	2.904824396	0.01913	0.984737169	NA	brown	-0.081608864	0.309590942		AT3G57870.1	AHUS5,ATSCE1,EMB1637,S	sumo conjugation enzyme 1	ubiquitin-conjugating enzyme E2
Sapur.014G069800.1	0.004923806	0.05557013	2.904824396	0.01913	0.984737169	NA	brown	-0.081608864	0.309590942	GO:0008270,GO:0005515	AT4G01270.1		RING/U-box superfamily protein	RING zinc finger protein, putative
Sapur.014G090000.1	0.00527888	0.05557013	2.904824396	0.01913	0.984737169	NA	brown	-0.081608864	0.309590942	GO:0016887,GO:0005524,GO:0055085,GO:0042626,GO:0016021,GO:0006810	AT1G02520.1	PGP11	P-glycoprotein 11	NA
Sapur.014G099100.1	0.005114485	0.05557013	2.904824396	0.01913	0.984737169	NA	brown	-0.081608864	0.309590942		AT5G47900.1		Protein of unknown function (DUF1624)	heparan-alpha-glucosaminide N-acetyltransferase
Sapur.014G102700.1	0.005114485	0.05557013	2.904824396	0.01913	0.984737169	NA	brown	-0.081608864	0.309590942		AT2G47590.1	PHR2	photolyase/blue-light receptor 2	photolyase/blue-light receptor 2, putative
Sapur.014G121300.4	0.005114485	0.05557013	2.904824396	0.01913	0.984737169	NA	brown	-0.081608864	0.309590942	GO:0006457	AT5G48570.1	ATFKBP65,FKBP65,ROF2	FKBP-type peptidyl-prolyl cis-trans isomerase family protein	peptidylprolyl isomerase, putative
Sapur.014G129600.3	0.005114485	0.05557013	2.904824396	0.01913	0.984737169	NA	brown	-0.081608864	0.309590942		AT1G05000.1		Phosphotyrosine protein phosphatases superfamily protein	tyrosine phosphatase family protein, putative
Sapur.014G129800.2	0.005114485	0.05557013	2.904824396	0.01913	0.984737169	NA	brown	-0.081608864	0.309590942	GO:0005515,GO:0003676	AT2G32940.1	AGO6	Argonaute family protein	NA
Sapur.014G135700.5	0.005114485	0.05557013	2.904824396	0.01913	0.984737169	NA	brown	-0.081608864	0.309590942		AT3G07740.2	ADA2A,ATADA2A,HAC10,HX	homolog of yeast ADA2 2A	transcriptional adapter ADA2
Sapur.014G144200.3	0.005114485	0.05557013	2.904824396	0.01913	0.984737169	NA	brown	-0.081608864	0.309590942		AT3G07525.2	ATATG10,ATG10	autophagocytosis-associated family protein	autophagocytosis associated protein, active-site protein
Sapur.016G044800.1	0.005937058	0.05557013	2.904824396	0.01913	0.984737169	NA	brown	-0.081608864	0.309590942	GO:0009055	AT5G26330.1		Cupredoxin superfamily protein	NA
Sapur.016G048000.2	0.006179623	0.05557013	2.904824396	0.01913	0.984737169	NA	brown	-0.081608864	0.309590942	GO:0046872	AT2G41900.1		CCCH-type zinc finger protein with ARM repeat domain	zinc finger CCCH domain protein
Sapur.016G081800.2	0.006444464	0.05557013	2.904824396	0.01913	0.984737169	NA	brown	-0.081608864	0.309590942		AT2G37080.1	RIP3	ROP interactive partner 3	interactor of constitutive active ROPs protein, putative
Sapur.016G100000.3	0.006044912	0.05557013	2.904824396	0.01913	0.984737169	NA	brown	-0.081608864	0.309590942		AT2G40070.1			proteophosphoglycan protein, putative
Sapur.016G104900.3	0.005114485	0.05557013	2.904824396	0.01913	0.984737169	NA	brown	-0.081608864	0.309590942	GO:0033699,GO:0006281,GO:0003677	AT5G01310.1	APTX	APRATAXIN-like	transcription factor bHLH140
Sapur.016G106400.1	0.006044912	0.05557013	2.904824396	0.01913	0.984737169	NA	brown	-0.081608864	0.309590942	GO:0043565,GO:0006355,GO:0003700	AT2G42380.2	ATBZIP34,BZIP34	Basic-leucine zipper (bZIP) transcription factor family protein	BZIP transcription factor
Sapur.016G114700.4	0.005114485	0.05557013	2.904824396	0.01913	0.984737169	NA	brown	-0.081608864	0.309590942	GO:0006355,GO:0003677	AT2G35940.1	BLH1,EDA29	BEL1-like homeodomain 1	BEL1-related homeotic protein, putative
Sapur.016G126500.4	0.005890737	0.05557013	2.904824396	0.01913	0.984737169	NA	brown	-0.081608864	0.309590942	GO:0055114,GO:0016491,GO:0008270	AT3G45770.1		Polyketide synthase, enoylreductase family protein	trans-2-enoyl-CoA reductase, putative
Sapur.016G143600.1	0.005114485	0.05557013	2.904824396	0.01913	0.984737169	NA	brown	-0.081608864	0.309590942		AT4G31530.1		NAD(P)-binding Rossmann-fold superfamily protein	NAD(P)H-binding family protein
Sapur.016G148900.1	0.005927541	0.05557013	2.904824396	0.01913	0.984737169	NA	brown	-0.081608864	0.309590942					NA
Sapur.016G157400.1	0.005114485	0.05557013	2.904824396	0.01913	0.984737169	NA	brown	-0.081608864	0.309590942	GO:0016779,GO:0009058	AT5G19485.1		transferases;nucleotidyltransferases	translation initiation factor eIF-2B subunit gamma
Sapur.016G165100.2	0.005114485	0.05557013	2.904824396	0.01913	0.984737169	NA	brown	-0.081608864	0.309590942	GO:0005515	AT2G30105.1			ubiquitin-like protein
Sapur.016G176900.6	0.005114485	0.05557013	2.904824396	0.01913	0.984737169	NA	brown	-0.081608864	0.309590942		AT3G43740.1		Leucine-rich repeat (LRR) family protein	somatic embryogenesis receptor kinase
Sapur.016G189200.2	0.005114485	0.05557013	2.904824396	0.01913	0.984737169	NA	brown	-0.081608864	0.309590942		AT1G22310.1	ATMBD8,MBD8	methyl-CPG-binding domain 8	methyl-binding domain protein, putative
Sapur.016G220000.2	0.005114485	0.05557013	2.904824396	0.01913	0.984737169	NA	brown	-0.081608864	0.309590942		AT3G27770.1			plant/MGF10-16 protein, putative
Sapur.016G240800.3	0.005114485	0.05557013	2.904824396	0.01913	0.984737169	NA	brown	-0.081608864	0.309590942		AT1G73140.1	TBL31	Plant protein of unknown function (DUF828)	Pmr5/Cas1p GDSL/SGNH-like acyl-esterase family protein
Sapur.016G253800.5	0.005114485	0.05557013	2.904824396	0.01913	0.984737169	NA	brown	-0.081608864	0.309590942	GO:0016757	AT1G16570.2		UDP-Glycosyltransferase superfamily protein	glycosyltransferase group 1 protein
Sapur.016G260500.5	0.006199952	0.05557013	2.904824396	0.01913	0.984737169	NA	brown	-0.081608864	0.309590942		AT3G15430.2		Regulator of chromosome condensation (RCC1) family protein	chromosome condensation regulator repeat protein
Sapur.016G260700.4	0.006083092	0.05557013	2.904824396	0.01913	0.984737169	NA	brown	-0.081608864	0.309590942		AT4G27450.1		Aluminium induced protein with YGL and LRDR motifs	stem-specific protein tsjt1 protein, putative
Sapur.016G271100.5	0.005114485	0.05557013	2.904824396	0.01913	0.984737169	NA	brown	-0.081608864	0.309590942	GO:0048544,GO:0006468,GO:0005524,GO:0004672	AT4G21380.1	ARK3,RK3	receptor kinase 3	Serine/Threonine-kinase
Sapur.016G275400.1	0.005114485	0.05557013	2.904824396	0.01913	0.984737169	NA	brown	-0.081608864	0.309590942	GO:0016757,GO:0047262	AT5G54690.1	GAUT12,IRX8,LGT6	galacturonosyltransferase 12	CAZy family GT8 glycosyltransferase
Sapur.016G290200.4	0.005114485	0.05557013	2.904824396	0.01913	0.984737169	NA	brown	-0.081608864	0.309590942	GO:0016020,GO:0008378,GO:0006486	AT1G32930.1		Galactosyltransferase family protein	beta-1%2C3-galactosyltransferase, putative
Sapur.016G291300.2	0.005114485	0.05557013	2.904824396	0.01913	0.984737169	NA	brown	-0.081608864	0.309590942	GO:0055114,GO:0016491	AT4G10490.1		2-oxoglutarate (2OG) and Fe(II)-dependent oxygenase superfamily protein	1-aminocyclopropane-1-carboxylate oxidase, putative
Sapur.016G292400.2	0.005114485	0.05557013	2.904824396	0.01913	0.984737169	NA	brown	-0.081608864	0.309590942	GO:0016021,GO:0016020,GO:0022857	AT3G30340.1		nodulin MtN21 /EamA-like transporter family protein	auxin-induced protein 5NG4, putative
Sapur.016G292800.1	0.005114485	0.05557013	2.904824396	0.01913	0.984737169	NA	brown	-0.081608864	0.309590942	GO:0016491,GO:0008152	AT5G15940.1		NAD(P)-binding Rossmann-fold superfamily protein	short chain dehydrogenase
Sapur.016G307900.3	0.006598449	0.05557013	2.904824396	0.01913	0.984737169	NA	brown	-0.081608864	0.309590942	GO:0003333	AT5G23810.2	AAP7	amino acid permease 7	transmembrane amino acid transporter protein
Sapur.017G022100.1	0.005011262	0.05557013	2.904824396	0.01913	0.984737169	NA	brown	-0.081608864	0.309590942		AT1G21280.1			NA
Sapur.017G037800.2	0.006083092	0.05557013	2.904824396	0.01913	0.984737169	NA	brown	-0.081608864	0.309590942	GO:0055114,GO:0016491	AT2G36690.1		2-oxoglutarate (2OG) and Fe(II)-dependent oxygenase superfamily protein	flavonol synthase/flavanone 3-hydroxylase, putative
Sapur.017G070400.1	0.005114485	0.05557013	2.904824396	0.01913	0.984737169	NA	brown	-0.081608864	0.309590942		AT3G29010.1		Biotin/lipoate A/B protein ligase family	lipoate-protein ligase A
Sapur.017G078900.1	0.005114485	0.05557013	2.904824396	0.01913	0.984737169	NA	brown	-0.081608864	0.309590942		AT3G02160.1		Bromodomain transcription factor	Tbp-associated factor taf protein, putative
Sapur.017G084200.5	0.005114485	0.05557013	2.904824396	0.01913	0.984737169	NA	brown	-0.081608864	0.309590942	GO:0007165,GO:0005515,GO:0043531	AT5G17680.1		disease resistance protein (TIR-NBS-LRR class), putative	TIR-NBS-LRR TIR type disease resistance protein
Sapur.017G106900.2	0.005114485	0.05557013	2.904824396	0.01913	0.984737169	NA	brown	-0.081608864	0.309590942		AT1G23890.2		NHL domain-containing protein	NA
Sapur.017G107700.1	0.005927541	0.05557013	2.904824396	0.01913	0.984737169	NA	brown	-0.081608864	0.309590942		AT5G16610.2			hypothetical protein
Sapur.017G107700.2	0.005114485	0.05557013	2.904824396	0.01913	0.984737169	NA	brown	-0.081608864	0.309590942		AT5G16610.2			hypothetical protein
Sapur.017G108000.1	0.005114485	0.05557013	2.904824396	0.01913	0.984737169	NA	brown	-0.081608864	0.309590942		AT3G54650.1	FBL17	RNI-like superfamily protein	hypothetical protein
Sapur.017G114300.1	0.005114485	0.05557013	2.904824396	0.01913	0.984737169	NA	brown	-0.081608864	0.309590942	GO:0016740,GO:0009058	AT5G17230.1	PSY	PHYTOENE SYNTHASE	phytoene synthase
Sapur.018G000900.7	0.005907325	0.05557013	2.904824396	0.01913	0.984737169	NA	brown	-0.081608864	0.309590942	GO:0006468,GO:0004672	AT2G24360.1		Protein kinase superfamily protein	Serine/Threonine/tyrosine kinase, putative
Sapur.018G016800.1	0.005114485	0.05557013	2.904824396	0.01913	0.984737169	NA	brown	-0.081608864	0.309590942	GO:0043531	AT3G14460.1		LRR and NB-ARC domains-containing disease resistance protein	CC-NBS-LRR resistance protein

Sapur.018G048500.2	0.005907325	0.05557013	2.904824396	0.01913	0.984737169	NA	brown	-0.081608864	0.309590942	GO:0006334,GO:0005634	AT2G19480.3	NAP1;2,NFA02,NFA2	nucleosome assembly protein 1;2	nucleosome assembly protein
Sapur.018G050900.3	0.005114485	0.05557013	2.904824396	0.01913	0.984737169	NA	brown	-0.081608864	0.309590942	GO:0055114,GO:0050113,GO:0019310,GO:0005737,GO:0005506	AT2G19800.1	MIOX2	myo-inositol oxygenase 2	Myo-inositol oxygenase
Sapur.018G055200.5	0.005114485	0.05557013	2.904824396	0.01913	0.984737169	NA	brown	-0.081608864	0.309590942	GO:0016020	AT3G24460.1		Serinc-domain containing serine and sphingolipid biosynthesis protein	maternal effect embryo arrest protein 55-1
Sapur.018G060600.2	0.005114485	0.05557013	2.904824396	0.01913	0.984737169	NA	brown	-0.081608864	0.309590942	GO:0016567,GO:0004842	AT3G06880.2		Transducin/WD40 repeat-like superfamily protein	E3 ubiquitin-protein ligase LIN, putative
Sapur.018G066100.3	0.005114485	0.05557013	2.904824396	0.01913	0.984737169	NA	brown	-0.081608864	0.309590942	GO:0003779	AT4G30160.1	ATVLN4,VLN4	villin 4	villin, putative
Sapur.018G071800.4	0.005114485	0.05557013	2.904824396	0.01913	0.984737169	NA	brown	-0.081608864	0.309590942		AT5G57630.1	CIPK21,SnRK3.4	CBL-interacting protein kinase 21	CBL-interacting kinase
Sapur.018G071800.7	0.006044912	0.05557013	2.904824396	0.01913	0.984737169	NA	brown	-0.081608864	0.309590942	GO:0007165	AT5G57630.1	CIPK21,SnRK3.4	CBL-interacting protein kinase 21	CBL-interacting kinase
Sapur.018G079600.3	0.005937058	0.05557013	2.904824396	0.01913	0.984737169	NA	brown	-0.081608864	0.309590942		AT5G17420.1	ATCESA7,CESA7,IRX3,MUR1	Cellulose synthase family protein	cellulose synthase A catalytic subunit 9 [UDP-forming] protein
Sapur.018G104900.2	0.005114485	0.05557013	2.904824396	0.01913	0.984737169	NA	brown	-0.081608864	0.309590942	GO:0006468,GO:0004672	AT2G17220.1		Protein kinase superfamily protein	Serine/Threonine kinase domain protein
Sapur.019G015800.2	0.009759838	0.05557013	2.904824396	0.01913	0.984737169	NA	brown	-0.081608864	0.309590942	GO:0016021,GO:0015693,GO:0015095	AT2G21120.1		Protein of unknown function (DUF803)	Non-imprinted in prader-willi/angelman syndrome region protein
Sapur.019G023900.2	0.006598449	0.05557013	2.904824396	0.01913	0.984737169	NA	brown	-0.081608864	0.309590942	GO:0055114,GO:0016491,GO:0050660,GO:0016021,GO:0004506	AT1G58440.1	SQE1,XF1	FAD/NAD(P)-binding oxidoreductase family protein	squalene monooxygenase
Sapur.019G024600.3	0.005114485	0.05557013	2.904824396	0.01913	0.984737169	NA	brown	-0.081608864	0.309590942	GO:0006629	AT2G26560.1	PLA IIA,PLA2A,PLP2	phospholipase A 2A	NA
Sapur.019G030600.3	0.005114485	0.05557013	2.904824396	0.01913	0.984737169	NA	brown	-0.081608864	0.309590942		AT5G37360.1			ammonium transporter, putative
Sapur.019G046800.1	0.005011262	0.05557013	2.904824396	0.01913	0.984737169	NA	brown	-0.081608864	0.309590942					hypothetical protein
Sapur.019G053900.4	0.005114485	0.05557013	2.904824396	0.01913	0.984737169	NA	brown	-0.081608864	0.309590942	GO:0051082,GO:0006457,GO:0005783,GO:0005509	AT1G08450.1	AtCRT3,CRT3,EBS2,PSL1	calreticulin 3	calreticulin, putative
Sapur.019G090200.1	0.005114485	0.05557013	2.904824396	0.01913	0.984737169	NA	brown	-0.081608864	0.309590942		AT4G28300.1		Protein of unknown function (DUF1421)	structural constituent of cell wall protein, putative
Sapur.019G111000.1	0.006522982	0.05557013	2.904824396	0.01913	0.984737169	NA	brown	-0.081608864	0.309590942		AT2G30370.2	CHAL,EPFL6	allergen-related	transmembrane protein, putative
Sapur.15WG009100.3	0.005114485	0.05557013	2.904824396	0.01913	0.984737169	NA	brown	-0.081608864	0.309590942		AT5G53050.2		alpha/beta-Hydrolases superfamily protein	alpha/beta fold hydrolase
Sapur.15WG010900.12	0.005907325	0.05557013	2.904824396	0.01913	0.984737169	NA	brown	-0.081608864	0.309590942		AT5G24270.2	ATSOS3,CBL4,SOS3	Calcium-binding EF-hand family protein	calcineurin B-like protein 4-1, putative
Sapur.15WG031300.2	0.005114485	0.05557013	2.904824396	0.01913	0.984737169	NA	brown	-0.081608864	0.309590942	GO:0006468,GO:0005524,GO:0004672	AT3G18040.1	MPK9	MAP kinase 9	NA
Sapur.15WG033100.2	0.005114485	0.05557013	2.904824396	0.01913	0.984737169	NA	brown	-0.081608864	0.309590942		AT1G04420.1		NAD(P)-linked oxidoreductase superfamily protein	aldo-keto reductase family protein
Sapur.15WG098000.3	0.005114485	0.05557013	2.904824396	0.01913	0.984737169	NA	brown	-0.081608864	0.309590942	GO:0016757,GO:0008152	AT5G17990.1	pat1,TRP1	tryptophan biosynthesis 1	anthranilate phosphoribosyltransferase
Sapur.15WG099800.2	0.005114485	0.05557013	2.904824396	0.01913	0.984737169	NA	brown	-0.081608864	0.309590942	GO:0016491,GO:0008152	AT5G50130.2		NAD(P)-binding Rossmann-fold superfamily protein	short-chain dehydrogenase/reductase family protein
Sapur.15WG105700.1	0.00527888	0.05557013	2.904824396	0.01913	0.984737169	NA	brown	-0.081608864	0.309590942					hypothetical protein
Sapur.15WG108900.1	0.005114485	0.05557013	2.904824396	0.01913	0.984737169	NA	brown	-0.081608864	0.309590942	GO:0055114,GO:0016620	AT1G13440.1	GAPC-2,GAPC2	glyceraldehyde-3-phosphate dehydrogenase C2	glyceraldehyde-3-phosphate dehydrogenase
Sapur.15WG117600.2	0.005733648	0.05557013	2.904824396	0.01913	0.984737169	NA	brown	-0.081608864	0.309590942	GO:0009982,GO:0009451,GO:0003723,GO:0001522	AT5G51140.1		Pseudouridine synthase family protein	RNA pseudouridine synthase
Sapur.15WG117600.3	0.005114485	0.05557013	2.904824396	0.01913	0.984737169	NA	brown	-0.081608864	0.309590942	GO:0009982,GO:0009451,GO:0003723,GO:0001522	AT5G51140.1		Pseudouridine synthase family protein	RNA pseudouridine synthase
Sapur.15WG130200.1	0.00593829	0.05557013	2.904824396	0.01913	0.984737169	NA	brown	-0.081608864	0.309590942	GO:0016021	AT1G31300.2		TRAM, LAG1 and CLN8 (TLC) lipid-sensing domain containing protein	plant/T19E23-12 protein
Sapur.T026700.1	0.005937058	0.05557013	2.904824396	0.01913	0.984737169	NA	brown	-0.081608864	0.309590942					transmembrane protein, putative
Sapur.T046700.3	0.005114485	0.05557013	2.904824396	0.01913	0.984737169	NA	brown	-0.081608864	0.309590942	GO:0046872,GO:0030001	AT1G29000.1		Heavy metal transport/detoxification superfamily protein	heavy metal-associated domain protein
Sapur.T048400.2	0.005114485	0.05557013	2.904824396	0.01913	0.984737169	NA	brown	-0.081608864	0.309590942	GO:0055114,GO:0020037,GO:0016705,GO:0005506	AT1G75130.1	CYP721A1	cytochrome P450, family 721, subfamily A, polypeptide 1	cytochrome P450 family protein
Sapur.T051600.1	0.005114485	0.05557013	2.904824396	0.01913	0.984737169	NA	brown	-0.081608864	0.309590942		AT5G44010.1			fanconi anemia group F protein (FANCF)
Sapur.T063500.2	0.00527888	0.05557013	2.904824396	0.01913	0.984737169	NA	brown	-0.081608864	0.309590942		AT2G43465.1		RNA-binding ASCH domain protein	NA
Sapur.T090200.2	0.005114485	0.05557013	2.904824396	0.01913	0.984737169	NA	brown	-0.081608864	0.309590942	GO:0008270,GO:0005515	AT5G40250.1		RING/U-box superfamily protein	RING finger protein
Sapur.T128200.1	0.005114485	0.05557013	2.904824396	0.01913	0.984737169	NA	brown	-0.081608864	0.309590942					NA
Sapur.T137000.1	0.00602393	0.05557013	2.904824396	0.01913	0.984737169	NA	brown	-0.081608864	0.309590942		AT5G24280.1	GMI1	gamma-irradiation and mitomycin c induced 1	NA
Sapur.T174100.1	0.00602393	0.05557013	2.904824396	0.01913	0.984737169	NA	brown	-0.081608864	0.309590942		AT3G24880.1		Helicase/SANT-associated, DNA binding protein	NA
Sapur.016G305500.1	0.025801812	0.051848008	2.725613373	0.019023	0.984823148	NA	brown	-0.048764172	0.544188343	GO:0005515,GO:0008017,GO:0007018,GO:0005524,GO:0003777,GO:0005871	AT3G44730.1	ATKP1,KP1	kinesin-like protein 1	kinesin heavy chain, putative
Sapur.014G101600.2	0.025427026	0.051848545	2.730755351	0.018987	0.984851566	NA	brown	-0.043986897	0.584368577	GO:0005975,GO:0004553	AT4G02290.1	AtGH9B13,GH9B13	glycosyl hydrolase 9B13	endo-1%2C4-beta-glucanase
Sapur.002G026400.2	0.034729625	0.049984705	2.633151294	0.018983	0.98485479	NA	brown	-0.02334408	0.771661217		AT1G19530.1			hypothetical protein
Sapur.005G049300.1	0.024353205	0.051850119	2.746029058	0.018882	0.984935356	NA	brown	-0.05399988	0.501774717		AT5G20680.3	TBL16	TRICHOME BIREFRINGENCE-LIKE 16	Pmr5/Cas1p GDSL/SGNH-like acyl-esterase family protein
Sapur.15WG088300.1	0.02191989	0.051853914	2.784037423	0.018625	0.985139911	NA	brown	-0.036842797	0.646876645		AT1G74850.1	PTAC2	plastid transcriptionally active 2	NA
Sapur.005G084400.2	0.028624143	0.053717485	2.904823276	0.018493	0.98524595	NA	brown	0.025814703	0.748266456	GO:0006468,GO:0004672	AT4G35780.1		ACT-like protein tyrosine kinase family protein	tyrosine kinase family protein
Sapur.018G043800.6	0.025147923	0.053717485	2.904823276	0.018493	0.98524595	NA	brown	0.018637658	0.816784116		AT4G32820.1		Tetratricopeptide repeat (TPR)-like superfamily protein	TPR domain protein
Sapur.008G044200.2	0.021964599	0.053717485	2.904823276	0.018493	0.98524595	NA	brown	0.002666115	0.973563397	GO:0051536,GO:0003824,GO:0008173,GO:0006364,GO:0005737	AT2G39670.1		Radical SAM superfamily protein	ribosomal RNA large subunit methyltransferase N
Sapur.009G067500.4	0.022074124	0.053717485	2.904823276	0.018493	0.98524595	NA	brown	-0.141729488	0.076623342	GO:0006629	AT5G14930.2	SAG101	senescence-associated gene 101	lipase
Sapur.003G169000.4	0.018209939	0.053717485	2.904823276	0.018493	0.98524595	NA	brown	-0.016056727	0.841797072	GO:0006468,GO:0004672	AT5G56460.1		Protein kinase superfamily protein	tyrosine kinase domain protein
Sapur.017G029000.1	0.018187765	0.053717485	2.904823276	0.018493	0.98524595	NA	brown	-0.020342024	0.800365436	GO:0016021	AT5G35730.1		EXS (ERD1/XPR1/SYG1) family protein	small molecule transporter, putative
Sapur.004G057100.2	0.017828572	0.053717485	2.904823276	0.018493	0.98524595	NA	brown	-0.018053186	0.822433287	GO:0016157,GO:0005985	AT1G73370.1	ATSUS6,SUS6	sucrose synthase 6	sucrose synthase
Sapur.006G087600.3	0.017640885	0.053717485	2.904823276	0.018493	0.98524595	NA	brown	-0.0181084	0.82189923	GO:0030170,GO:0009058,GO:0008483,GO:0006520	AT2G30970.2	ASP1	aspartate aminotransferase 1	aspartate aminotransferase
Sapur.006G147300.2	0.019992137	0.053717485	2.904823276	0.018493	0.98524595	NA	brown	-0.035924095	0.655109856		AT3G58700.1		Ribosomal L5P family protein	NA
Sapur.018G119000.1	0.017640885	0.053717485	2.904823276	0.018493	0.98524595	NA	brown	-0.047045965	0.558481995		AT4G27690.1	VPS26B	vacuolar protein sorting 26B	vacuolar protein sorting-associated protein
Sapur.001G135300.1	0.020155691	0.053717485	2.904823276	0.018493	0.98524595	NA	brown	-0.034607615	0.6669804					NA
Sapur.016G250400.1	0.017187763	0.053717485	2.904823276	0.018493	0.98524595	NA	brown	-0.022104473	0.783478692	GO:0043087	AT3G14910.1			Rab3 GTPase-activating protein non-catalytic subunit, putative
Sapur.008G119000.1	0.019329767	0.053717484	2.904823276	0.018493	0.98524595	NA	brown	-0.040561716	0.613992169	GO:0071805,GO:0016020,GO:0015079	AT1G70300.1	KUP6	K+ uptake permease 6	high-affinity potassium transporter protein, putative
Sapur.004G132000.1	0.015676336	0.053717484	2.904823276	0.018493	0.98524595	NA	brown	-0.030860057	0.701217972	GO:0016780,GO:0016020,GO:0008654	AT1G68000.1	ATPIS,ATPIS1,PIS1	phosphatidylinositol synthase 1	CDP-diacylglycerol-inositol 3-phosphatidyltransferase
Sapur.010G123400.1	0.016537851	0.053717484	2.904823276	0.018493	0.98524595	NA	brown	-0.027932366	0.728394124	GO:0043565,GO:0006355,GO:0003700	AT1G69310.1	ATWRKY57,WRKY57	WRKY DNA-binding protein 57	WRKY transcription factor, putative
Sapur.003G114600.1	0.016649834	0.053717484	2.904823276	0.018493	0.98524595	NA	brown	-0.041631409	0.60467031		AT5G44710.1			ribosomal subunit S27
Sapur.013G054600.1	0.016161459	0.053717484	2.904823276	0.018493	0.98524595	NA	brown	-0.036776592	0.647468543		AT1G64770.1	NDF2,NDH45	NDH-dependent cyclic electron flow 1	NA
Sapur.013G028200.2	0.016206094	0.053717484	2.904823276	0.018493	0.98524595	NA	brown	-0.081608864	0.309590942	GO:0055085,GO:0016021	AT5G27360.1	SFP2	Major facilitator superfamily protein	sugar porter (SP) family MFS transporter
Sapur.012G073300.1	0.016840782	0.053717484	2.904823276	0.018493	0.98524595	NA	brown	-0.045413426	0.572228213	GO:0005524,GO:0003676,GO:0031204,GO:0008565	AT1G20960.2	emb1507	U5 small nuclear ribonucleoprotein helicase, putative	RNA helicase
Sapur.014G127700.2	0.017762838	0.053717484	2.904823276	0.018493	0.98524595	NA	brown	0.005592399	0.944581172		AT1G05060.1			hypothetical protein
Sapur.012G000300.7	0.015866223	0.053717484	2.904823276	0.018493	0.98524595	NA	brown	-0.03367695	0.675422411		AT3G49590.3		Autophagy-related protein 13	autophagy-related protein
Sapur.001G165100.1	0.016452512	0.053717484	2.904823276	0.018493	0.98524595	NA	brown	-0.050615199	0.528994069	GO:0008152,GO:0003824	AT3G16170.1		AMP-dependent synthetase and ligase family protein	long-chain-fatty-acid-CoA ligase
Sapur.007G076100.1	0.015708968	0.053717484	2.904823276	0.018493	0.98524595	NA	brown	-0.03499378	0.663489665		AT2G21940.4	ATSK1,SK1	shikimate kinase 1	shikimate kinase
Sapur.013G141500.6	0.015349114	0.053717484	2.904823276	0.018493	0.98524595	NA	brown	-0.036132092	0.653242148		AT5G58320.2		Kinase interacting (KIP1-like) family protein	kinase interacting (KIP1-like) family protein
Sapur.016G099700.2	0.016752097	0.053717484	2.904823276	0.018493	0.98524595	NA	brown	-0.055043867	0.493529722	GO:0030132,GO:0030130,GO:0016192,GO:0006886,GO:0005198	AT3G51890.1		Clathrin light chain protein	clathrin light chain
Sapur.010G165900.3	0.016505156	0.053717484	2.904823276	0.018493	0.98524595	NA	brown	-0.05881906	0.464324153	GO:0019898,GO:0015979,GO:0009654,GO:0009523,GO:0005509	AT3G55330.1	PPL1	PsbP-like protein 1	thylakoid lumenal 25.6 kDa protein, putative
Sapur.016G299400.2	0.01723007	0.053717484	2.904823276	0.018493	0.98524595	NA	brown	-0.421685503	3.79E-08	GO:0043139,GO:0003697	AT1G30680.1		toprim domain-containing protein	DNA primase
Sapur.011G008500.4	0.015676162	0.053717484	2.904823276	0.018493	0.98524595	NA	brown	-0.056232252	0.48423248		AT1G62310.1		transcription factor jumonji (jmjC) domain-containing protein	lysine-specific demethylase 3A-B, putative
Sapur.15WG149700.3	0.014682578	0.053717484	2.904823276	0.018493	0.98524595	NA	brown	-0.042566126	0.59657657		AT5G52300.1	LTI65,RD29B	CAP160 protein	Low-temperature-induced protein, putative
Sapur.010G195700.3	0.014476493	0.053717484	2.904823276	0.018493	0.98524595	NA	brown	-0.053633855	0.504682437	GO:0006887,GO:0000145	AT5G03540.1	ATEXO70A1,EXO70A1	exocyst subunit exo70 family protein A1	exocyst subunit EXO70
Sapur.005G048600.4	0.017380362	0.053717484	2.904823276	0.018493	0.98524595	NA	brown	-0.075055914	0.350174282	GO:0016624,GO:0008152	AT5G09300.1		Thiamin diphosphate-binding fold (THDP-binding) superfamily protein	2-oxoisovalerate dehydrogenase alpha subunit, putative
Sapur.018G097400.3	0.014745053	0.053717484	2.904823276	0.018493	0.98524595	NA	brown	-0.300250459	0.000133128	GO:0055085,GO:0016020,GO:0006821,GO:0005247	AT5G33280.1		Voltage-gated chloride channel family protein	chloride channel ClC protein, putative
Sapur.006G005300.2	0.014010418	0.053717484	2.904823276	0.018493	0.98524595	NA	brown	-0.048158713	0.549204503	GO:0009058,GO:0003824	AT2G32260.1	ATCCT1,CCT1	phosphorylcholine cytidylyltransferase	choline-phosphate cytidylyltransferase
Sapur.003G057200.1	0.0140044	0.053717484	2.904823276	0.018493	0.98524595	NA	brown	-0.048089215	0.549781733	GO:0003824,GO:0006470,GO:0004722	AT2G35350.1	PLL1	poltergeist like 1	protein phosphatase 2C
Sapur.013G002900.1	0.014727222	0.053717484	2.904823276	0.018493	0.98524595	NA	brown	-0.085588434	0.286510781		AT1G28200.1	FIP1	FH interacting protein 1	GLABRA 2 expression modulator, putative
Sapur.005G114500.1	0.012922672	0.053717484	2.904823276	0.018493	0.98524595	NA	brown	-0.06570763	0.413574696					hypothetical protein
Sapur.013G052200.1	0.013594852	0.053717484	2.904823276	0.018493	0.98524595	NA	brown	-0.068833196	0.391664935		AT3G03810.1	EDA30	O-fucosyltransferase family protein	auxin-independent growth promoter protein
Sapur.001G144100.1	0.016052245	0.053717484	2.904823276	0.018493	0.98524595	NA	brown	-0.083916554	0.29606304	GO:0007031,GO:0005777,GO:0005524,GO:0042623,GO:0006625,GO:0005778	AT5G08470.1	PEX1	peroxisome 1	peroxisome biogenesis factor 1
Sapur.005G173800.2	0.02867901	0.051864895	2.904822183	0.017855	0.985754722	NA	brown	-0.011476295	0.886564112	GO:0016301,GO:0008152,GO:0005524	AT1G03030.1		P-loop containing nucleoside triphosphate hydrolases superfamily protein	NA
Sapur.016G123000.1	0.025323293	0.051864895	2.904822183	0.017855	0.985754722	NA	brown	-0.027245219	0.734823314					NA
Sapur.013G039700.2	0.023542955	0.051864895	2.904822183	0.017855	0.985754722	NA	brown	-0.035569932	0.658294983		AT1G54460.1		TPX2 (targeting protein for Xklp2) protein family	targeting protein for Xklp2 protein
Sapur.011G107700.2	0.022347928	0.051864895	2.904822183	0.017855	0.985754722	NA	brown	-0.041863195	0.602658729	GO:0055085,GO:0016021	AT1G76520.2		Auxin efflux carrier family protein	auxin efflux carrier family protein
Sapur.002G154500.8	0.024423984	-0.041767708	2.415108448	-0.01729	0.986201801	NA	brown	-0.106919253	0.182593296		AT1G02960.2			hypothetical protein
Sapur.002G092100.6	0.012049728	0.047961395	2.904823455	0.016511	0.986826766	NA	brown	-0.088835877	0.268551501	GO:0016020	AT1G58520.1	RXW8	lipases;hydrolases, acting on ester bonds	lipase/hydrolase
Sapur.018G076400.2	0.022797777	0.045407439	2.77235351	0.016379	0.986932303	NA	brown	-0.059549503	0.458785398	GO:0050662,GO:0003824	AT2G23910.1		NAD(P)-binding Rossmann-fold superfamily protein	cinnamoyl-CoA reductase
Sapur.002G128500.2	0.050281114	0.024297126	1.548228629	0.015694	0.987478912	NA	brown	-0.060993963	0.447941183		AT1G01770.1			propionyl-CoA carboxylase, putative
Sapur.008G012000.1	0.03555561	-0.029426488	1.884174535	-0.01562	0.987539378	NA	brown	-0.168029452	0.035416971	GO:0051082,GO:0031072	AT5G22060.1	ATJ2,J2	DNAJ homologue 2	chaperone protein DnaJ
Sapur.014G049300.3	0.023900332	-0.041762901	2.752765769	-0.01517	0.987895557	NA	brown	-0.132661124	0.097664142		AT2G45270.1	GCP1	glycoprotease 1	O-sialoglycoprotein endopeptidase, putative
Sapur.017G045900.2	0.043581466	0.026525963	1.852558939	0.014319	0.988575838	NA	brown	-0.050296216	0.531597139	GO:0034314,GO:0030041,GO:0015629,GO:0005885	AT4G14147.1	ARPC4	protein binding	actin-related protein 2/3 complex subunit 4
Sapur.15WG111700.5	0.032867694	0.028741779	2.095059171	0.013719	0.989054294	NA	brown	-0.065396218	0.415796316		AT5G63440.3		Protein of unknown function (DUF167)	plant/MLE2-7 protein
Sapur.004G143300.5	0.031398469	0.028742203	2.104723892	0.013656	0.989104391	NA	brown	-0.0815184	0.310129359		AT1G08200.1	AXS2	UDP-D-apiose/UDP-D-xylose synthase 2	UDP-D-apiose/UDP-D-xylose synthase
Sapur.001G060400.2	0.028457732	0.028743105	2.125797973	0.013521	0.98921206	NA	brown	-0.115365221	0.150213079					hypothetical protein
Sapur.001G063900.1	0.029734661	0.028751479	2.368062791	0.012141	0.990312842	NA	brown	-0.063155237	0.43198813		AT5G51180.2		alpha/beta-Hydrolases superfamily protein	serine esterase family protein
Sapur.006G209400.1	0.045320254	-0.023215617	2.029486509	-0.01144	0.990873071	NA	brown	-0.058050134	0.470194311	GO:0016787,GO:0005524,GO:0003677	AT5G11170.1		DEAD/DEAH box RNA helicase family protein	DEAD-box ATP-dependent RNA helicase
Sapur.003G020500.1	0.011131465	0.033182656	2.904823918	0.011423	0.990885729	NA	brown	-0.103290009	0.197981389	GO:0003824,GO:0006470,GO:0004722	AT1G72770.3	HAB1	homology to ABI1	protein phosphatase 2C
Sapur.006G181300.2	0.012996108	0.033182656	2.904823918	0.011423	0.990885729	NA	brown	-0.191031956	0.016547523	GO:0016020	AT5G21920.1	ATYLMG2,YLMG2	YGGT family protein	YGGT family protein
Sapur.008G007400.2	0.013030941	0.033182656	2.904823918	0.011423	0.990885729	NA	brown	-0.104022976	0.194800246	GO:0005515	AT5G06600.1	UBP12	ubiquitin-specific protease 12	ubiquitin carboxyl-terminal hydrolase
Sapur.006G133100.2	0.037782662	-0.023206018	2.066124028	-0.01123	0.991038614	NA	brown	-0.11646894	0.146324199		AT4G13270.1		Late embryogenesis abundant (LEA) hydroxyproline-rich glycoprotein family	late embryogenesis abundant protein
Sapur.002G024000.1	0.037312442	-0.023205365	2.068693024	-0.01122	0.991049995	NA	brown	-0.104524038	0.192647063		AT1G75400.1		RING/U-box superfamily protein	ubiquitin-protein ligase, putative
Sapur.010G125900.5	0.022337797	0.030966428	2.777151243	0.01115	0.991103429	NA	brown	-0.048817514	0.543747491		AT1G26640.1		Amino acid kinase family protein	amino acid kinase family protein
Sapur.010G107600.1	0.021413285	0.030966604	2.792610943	0.011089	0.991152628	NA	brown	-0.071383339	0.374316374	GO:0016773	AT2G03890.1	ATPI4K GAMMA 7,PI4K GAM	phosphoinositide 4-kinase gamma 7	phosphatidylinositol 3- and 4-kinase
Sapur.018G048400.6	0.033231383	-0.023148731	2.338748785	-0.0099	0.992102737	NA	brown	-0.061840741	0.441651696	GO:0009432,GO:0006281,GO:0005524,GO:0003697	AT2G19490.1		recA DNA recombination family protein	DNA repair protein RecA
Sapur.004G081000.2	0.050073331	-0.024973145	2.528309973	-0.00988	0.992119098	NA	brown	-0.029394079	0.714781077					NA
Sapur.001G057600.2	0.048658824	-0.024972281	2.534654382	-0.00985	0.992139096	NA	brown	-0.043914983	0.584983726	GO:0006221,GO:0003883	AT1G30820.1		CTP synthase family protein	CTP synthase
Sapur.003G122300.5	0.030743401	-0.021292485	2.35267254	-0.00905	0.992778972	NA	brown	-0.106512245	0.184273955		AT5G13220.1	JAS1,JAZ10,TIFY9	jasmonate-zim-domain protein 10	ZIM motif protein
Sapur.016G145900.1	0.029717509	-0.021291004	2.361651025	-0.00902	0.992806925	NA	brown	-0.088741742	0.269061071	GO:0003676	AT3G51950.2		Zinc finger (CCCH-type) family protein / RNA recognition motif (RRM)-containing protein	nucleic acid-binding protein, putative
Sapur.010G191900.8	0.028753553	-0.021289571	2.37043934	-0.00898	0.992834075	NA	brown	-0.114117458	0.15470291	GO:0055085,GO:0016021	AT2G16980.2		Major facilitator superfamily protein	hippocampus abundant transcript protein, putative
Sapur.013G080100.2	0.041164379	-0.023113255	2.578924658	-0.00896	0.992849166	NA	brown	-0.035351464	0.660262822	GO:0055085,GO:0016020	AT5G12080.2	ATMSL10,MSL10	mechanosensitive channel of small conductance-like 10	mechanosensitive ion channel domain protein, putative
Sapur.001G164800.4	0.025165507	-0.021283806	2.406875004	-0.00884	0.992944462	NA	brown	-0.399180318	2.23E-07		AT4G29100.1		basic helix-loop-helix (bHLH) DNA-binding superfamily protein	transcription factor, putative
Sapur.002G164000.1	0.036654375	-0.023108734	2.615620224	-0.00883	0.992950864	NA	brown	-0.066897456	0.405150779	GO:0006468,GO:0004672	AT4G02010.1		Protein kinase superfamily protein	tyrosine kinase domain protein
Sapur.010G163600.2	0.024608205	-0.021282859	2.413033644	-0.00882	0.992962782	NA	brown	-0.12725527	0.112235226	GO:0016597,GO:0008152	AT2G39570.1		ACT domain-containing protein	ACT domain protein
Sapur.002G181000.1	0.032432266	-0.021251864	2.647620259	-0.00803	0.993595625	NA	brown	-0.104831229	0.191335585	GO:0016818,GO:0008270,GO:0003676,GO:0016788	AT1G48360.2		zinc ion binding;nucleic acid binding;hydrolases, acting on acid anhydrides, in phosphorus-containing anhydrides	fanconi-associated nuclease-like protein
Sapur.T190000.1	0.040554958	-0.02307934	2.904822183	-0.00795	0.993660729	NA	brown	-0.035132181	0.662240341	GO:0008270	AT1G48570.1		zinc finger (Ran-binding) family protein	Zn-finger in Ran-binding protein, putative
Sapur.013G070200.2	0.034998114	-0.02307934	2.904822183	-0.00795	0.993660729	NA	brown	-0.066651165	0.406886121		AT3G28910.1	ATMYB30,MYB30	myb domain protein 30	R2R3-MYB transcription factor, putative
Sapur.019G039000.3	0.028312057	-0.021246807	2.69342129	-0.00789	0.993706026	NA	brown	-0.096996409	0.226854512	GO:0016020,GO:0015171,GO:0003333	AT3G03720.1	CAT4	cationic amino acid transporter 4	NA
Sapur.013G126900.1	0.027960028	-0.021246347	2.697716621	-0.00788	0.993716183	NA	brown	-0.076927309	0.338257563		AT2G14830.1		Regulator of Vps4 activity in the MVB pathway protein	regulator of Vps4 activity in the MVB pathway protein
Sapur.016G123700.4	0.027658384	-0.021245949	2.70145119	-0.00786	0.993724988	NA	brown	-0.100308983	0.211307067	GO:0005515	AT2G44430.1		DNA-binding bromodomain-containing protein	bromodomain protein
Sapur.005G110000.2	0.027271622	-0.021245433	2.70631465	-0.00785	0.993736416	NA	brown	-0.096940737	0.227122531		AT4G36970.1		Remorin family protein	remorin
Sapur.014G004900.4	0.027105966	-0.021245211	2.70842414	-0.00784	0.99374136	NA	brown	-0.089676622	0.264029522	GO:0005515	AT1G44910.1	ATPRP40A,PRP40A	pre-mRNA-processing protein 40A	FF domain protein
Sapur.009G124500.2	0.026560042	-0.021244469	2.715491651	-0.00782	0.993757867	NA	brown	-0.108410838	0.176530267	GO:0005525	AT4G35860.1	ATGB2,ATRAB2C,ATRABB1C	GTP-binding 2	Ras small GTPase family Ras protein
Sapur.002G157500.1	0.023189866	-0.02122675	2.904823276	-0.00731	0.994169578	NA	brown	-0.070086478	0.383079297	GO:0032259,GO:0008168	AT4G02405.1		S-adenosyl-L-methionine-dependent methyltransferases superfamily protein	leucine carboxyl methyltransferase
Sapur.018G053800.3	0.023327466	-0.02122675	2.904823276	-0.00731	0.994169578	NA	brown	-0.069189664	0.389211252	GO:0016790,GO:0006633	AT1G08510.1	FATB	fatty acyl-ACP thioesterases B	acyl-ACP thioesterase B
Sapur.009G038700.2	0.024993092	-0.02122675	2.904823276	-0.00731	0.994169578	NA	brown	-0.088062602	0.272756841	GO:0016887,GO:0005524,GO:0016020	AT3G21090.1		ABC-2 type transporter family protein	white-brown-complex ABC transporter family protein
Sapur.008G035200.1	0.023782877	-0.02122675	2.904823276	-0.00731	0.994169578	NA	brown	-0.081147683	0.312342159	GO:0005515,GO:0000160	AT5G02810.1	APRR7,PRR7	pseudo-response regulator 7	two-component response regulator-like PRR73 protein
Sapur.006G207800.3	0.024972983	-0.02122675	2.904823276	-0.00731	0.994169578	NA	brown	-0.081608864	0.309590942	GO:0006457,GO:0003755,GO:0000413	AT4G32420.1		Cyclophilin-like peptidyl-prolyl cis-trans isomerase family protein	peptidyl-prolyl cis-trans isomerase, cyclophilin-type protein
Sapur.001G187500.2	0.024496166	-0.02122675	2.904823276	-0.00731	0.994169578	NA	brown	-0.113693096	0.156252676		AT5G49820.1	EMB1879,RUS6	Protein of unknown function, DUF647	DUF647 domain protein
Sapur.001G183100.1	0.024896141	-0.02122675	2.904823276	-0.00731	0.994169578	NA	brown	-0.061867584	0.441453141					NA
Sapur.008G018100.1	0.024083084	-0.02122675	2.904823276	-0.00731	0.994169578	NA	brown	-0.076190894	0.342915801		AT5G04550.1		Protein of unknown function (DUF668)	plant/T32M21-140 protein
Sapur.004G088700.2	0.026021949	-0.02122675	2.904823276	-0.00731	0.994169578	NA	brown	-0.067216155	0.402911776	GO:0055114,GO:0016491,GO:0051536,GO:0020037	AT2G15620.1	ATHNIR,NIR,NIR1	nitrite reductase 1	ferredoxin-nitrite reductase
Sapur.002G082300.3	0.028299979	-0.02122675	2.904823276	-0.00731	0.994169578	NA	brown	-0.066873694	0.405318015	GO:0010181,GO:0055114,GO:0016638	AT5G49970.1	ATPPOX,PDX3,PPOX	pyridoxin (pyrodoxamine) 5\'-phosphate oxidase	pyridoxamine 5'-phosphate oxidase
Sapur.002G161200.3	0.038218251	-0.02122675	2.904823276	-0.00731	0.994169578	NA	brown	-0.01034166	0.897714866	GO:0006420,GO:0005737,GO:0005524,GO:0004814,GO:0000166	AT4G26300.1	emb1027	Arginyl-tRNA synthetase, class Ic	arginyl-tRNA synthetase
Sapur.016G133700.1	0.041024673	0.012281821	1.699458714	0.007227	0.994233817	NA	brown	-0.06291224	0.433765333		AT5G59410.1		Rab5-interacting family protein	Rab5-interacting protein
Sapur.006G216600.1	0.038016774	0.014265342	2.062223267	0.006917	0.994480711	NA	brown	-0.087011438	0.27854464		AT1G13940.1		Plant protein of unknown function (DUF863)	hypothetical protein
Sapur.003G028500.1	0.033831686	0.01427141	2.086360985	0.00684	0.994542244	NA	brown	-0.195365654	0.014204935					hypothetical protein
Sapur.016G279600.2	0.031835542	0.01427454	2.099162176	0.0068	0.994574337	NA	brown	-0.091256519	0.255673311					NA
Sapur.001G009300.3	0.015343454	-0.019374104	2.904824395	-0.00667	0.994678445	NA	brown	-0.081608864	0.309590942		AT1G51110.1		Plastid-lipid associated protein PAP / fibrillin family protein	plastid-lipid-associated protein, putative
Sapur.001G020500.2	0.015343454	-0.019374104	2.904824395	-0.00667	0.994678445	NA	brown	-0.081608864	0.309590942	GO:0005516	AT2G38800.1		Plant calmodulin-binding protein-related	plant calmodulin-binding domain protein
Sapur.001G051600.1	0.01689596	-0.019374104	2.904824395	-0.00667	0.994678445	NA	brown	-0.10715922	0.181607687		AT5G13270.1	RARE1	Pentatricopeptide repeat (PPR) superfamily protein	NA
Sapur.001G099600.5	0.01370024	-0.019374104	2.904824395	-0.00667	0.994678445	NA	brown	-0.104765698	0.191614804		AT1G63370.1		Flavin-binding monooxygenase family protein	flavin containing monooxygenase FMO GS-OX protein
Sapur.001G147300.2	0.016200829	-0.019374104	2.904824395	-0.00667	0.994678445	NA	brown	-0.140923119	0.078331976	GO:0007034	AT2G06530.1	VPS2.1	SNF7 family protein	charged multivesicular body protein 2a
Sapur.001G187500.4	0.017139707	-0.019374104	2.904824395	-0.00667	0.994678445	NA	brown	-0.064493937	0.42227248		AT5G49820.1	EMB1879,RUS6	Protein of unknown function, DUF647	DUF647 domain protein
Sapur.002G041700.2	0.01636298	-0.019374104	2.904824395	-0.00667	0.994678445	NA	brown	-0.140373495	0.079514089		AT4G02800.1			hypothetical protein
Sapur.002G056400.2	0.01565997	-0.019374104	2.904824395	-0.00667	0.994678445	NA	brown	-0.110986343	0.166413222	GO:0006508,GO:0004185	AT5G42240.1	scpl42	serine carboxypeptidase-like 42	serine carboxypeptidase
Sapur.002G062000.1	0.015343454	-0.019374104	2.904824395	-0.00667	0.994678445	NA	brown	-0.081608864	0.309590942	GO:0055085,GO:0016020,GO:0006810,GO:0015171,GO:0003333	AT2G01170.1	BAT1	bidirectional amino acid transporter 1	amino acid permease
Sapur.002G125700.2	0.0162402	-0.019374104	2.904824395	-0.00667	0.994678445	NA	brown	-0.085084984	0.289365288	GO:0016559,GO:0005779	AT2G45740.2	PEX11D	peroxin 11D	peroxisomal biogenesis factor 11
Sapur.003G002900.1	0.015343454	-0.019374104	2.904824395	-0.00667	0.994678445	NA	brown	-0.080726832	0.314866659		AT1G33980.1	ATUPF3,UPF3	Smg-4/UPF3 family protein	regulator of nonsense transcripts protein 3A, putative
Sapur.003G161700.2	0.014886943	-0.019374104	2.904824395	-0.00667	0.994678445	NA	brown	-0.108950416	0.174373986	GO:0005515	AT3G57980.1		DNA-binding bromodomain-containing protein	DNA-binding bromodomain protein
Sapur.004G007900.3	0.018711201	-0.019374104	2.904824395	-0.00667	0.994678445	NA	brown	-0.140855828	0.078475937	GO:0016020,GO:0008378,GO:0006486	AT1G05170.2		Galactosyltransferase family protein	beta-1%2C3-galactosyltransferase, putative
Sapur.004G038900.1	0.018828819	-0.019374104	2.904824395	-0.00667	0.994678445	NA	brown	-0.13971929	0.08093981		AT4G18593.1		dual specificity protein phosphatase-related	dual specificity protein phosphatase
Sapur.004G043500.2	0.015152775	-0.019374104	2.904824395	-0.00667	0.994678445	NA	brown	-0.109667403	0.171538952	GO:0006412,GO:0005840,GO:0005622,GO:0003735	AT5G23535.1		KOW domain-containing protein	ribosomal protein L24
Sapur.004G061600.3	0.015343454	-0.019374104	2.904824395	-0.00667	0.994678445	NA	brown	-0.081608864	0.309590942		AT5G37690.1		SGNH hydrolase-type esterase superfamily protein	GDSL esterase/lipase
Sapur.004G082900.1	0.015743222	-0.019374104	2.904824395	-0.00667	0.994678445	NA	brown	-0.111059283	0.166133122		AT1G43760.1		DNAse I-like superfamily protein	NA
Sapur.004G126100.1	0.016905521	-0.019374104	2.904824395	-0.00667	0.994678445	NA	brown	-0.140559085	0.079113334		AT4G34830.1	MRL1	Pentatricopeptide repeat (PPR) superfamily protein	PPR containing plant protein
Sapur.005G015700.3	0.01699377	-0.019374104	2.904824395	-0.00667	0.994678445	NA	brown	-0.11334257	0.157541566	GO:0005515	AT5G27030.1	TPR3	TOPLESS-related 3	topless-related protein
Sapur.005G069900.2	0.015743222	-0.019374104	2.904824395	-0.00667	0.994678445	NA	brown	-0.111059283	0.166133122		AT5G08350.1		GRAM domain-containing protein / ABA-responsive protein-related	PH-GRAM-GEM domain protein
Sapur.005G079500.4	0.016746437	-0.019374104	2.904824395	-0.00667	0.994678445	NA	brown	-0.113335126	0.157569023	GO:0005634	AT1G20610.1	CYCB2;3	Cyclin B2;3	cyclin B2
Sapur.005G135000.1	0.016066679	-0.019374104	2.904824395	-0.00667	0.994678445	NA	brown	-0.141288439	0.077554124	GO:0005515	AT2G17900.1	SDG37	SET domain group 37	histone-lysine N-methyltransferase ASHR1
Sapur.006G155200.1	0.017977277	-0.019374104	2.904824395	-0.00667	0.994678445	NA	brown	-0.105921399	0.186733877	GO:0005515,GO:0006468,GO:0004672	AT3G46920.1		Protein kinase superfamily protein with octicosapeptide/Phox/Bem1p domain	tyrosine kinase domain protein
Sapur.006G189200.2	0.017910229	-0.019374104	2.904824395	-0.00667	0.994678445	NA	brown	-0.141909329	0.076246407	GO:0043565,GO:0008270,GO:0006355,GO:0003700	AT4G26150.1	CGA1,GATA22	cytokinin-responsive gata factor 1	GATA zinc finger protein
Sapur.006G196200.1	0.015343454	-0.019374104	2.904824395	-0.00667	0.994678445	NA	brown	-0.081608864	0.309590942	GO:0005515,GO:0006468,GO:0005524,GO:0004672	AT2G25790.1		Leucine-rich receptor-like protein kinase family protein	LRR receptor-like kinase
Sapur.006G200000.4	0.01560465	-0.019374104	2.904824395	-0.00667	0.994678445	NA	brown	-0.141668786	0.076750909		AT4G32730.2	ATMYB3R-1,ATMYB3R1,MY	Homeodomain-like protein	Myb transcription factor
Sapur.006G217600.2	0.016302585	-0.019374104	2.904824395	-0.00667	0.994678445	NA	brown	-0.112511804	0.160628137	GO:0043565,GO:0006355,GO:0003700	AT1G80840.1	ATWRKY40,WRKY40	WRKY DNA-binding protein 40	WRKY transcription factor
Sapur.007G102300.1	0.015343454	-0.019374104	2.904824395	-0.00667	0.994678445	NA	brown	-0.081608864	0.309590942		AT5G09520.1		hydroxyproline-rich glycoprotein family protein	transmembrane protein, putative
Sapur.007G103400.3	0.015946499	-0.019374104	2.904824395	-0.00667	0.994678445	NA	brown	-0.111674679	0.163783888		AT4G33625.1			COPI associated protein
Sapur.008G030400.4	0.021198105	-0.019374104	2.904824395	-0.00667	0.994678445	NA	brown	-0.135101632	0.091596671	GO:0009073,GO:0004107	AT1G48850.1	EMB1144	chorismate synthase, putative / 5-enolpyruvylshikimate-3-phosphate phospholyase, putative	chorismate synthase
Sapur.008G130800.3	0.015321281	-0.019374104	2.904824395	-0.00667	0.994678445	NA	brown	-0.063922497	0.426404114	GO:0006355,GO:0005634	AT5G43700.1	ATAUX2-11,IAA4	AUX/IAA transcriptional regulator family protein	indole-3-acetic acid inducible protein
Sapur.009G046800.5	0.01472906	-0.019374104	2.904824395	-0.00667	0.994678445	NA	brown	-0.108881201	0.174649489	GO:0006355,GO:0003700	AT5G12840.4	ATHAP2A,EMB2220,HAP2A,	nuclear factor Y, subunit A1	CCAAT-binding transcription factor
Sapur.009G070700.3	0.015343454	-0.019374104	2.904824395	-0.00667	0.994678445	NA	brown	-0.081608864	0.309590942	GO:0055114,GO:0016972	AT1G49880.1		Erv1/Alr family protein	FAD-linked sulfhydryl oxidase Alr protein, putative
Sapur.010G115300.1	0.019002027	-0.019374104	2.904824395	-0.00667	0.994678445	NA	brown	-0.139862023	0.080627008		AT3G06550.3		O-acetyltransferase family protein	Cas1p 10-TM acyl transferase domain protein
Sapur.011G008700.3	0.013532953	-0.019374104	2.904824395	-0.00667	0.994678445	NA	brown	-0.081608864	0.309590942		AT4G04330.1		Chaperonin-like RbcX protein	RbcX protein
Sapur.012G009200.3	0.015343454	-0.019374104	2.904824395	-0.00667	0.994678445	NA	brown	-0.081608864	0.309590942		AT4G27700.1		Rhodanese/Cell cycle control phosphatase superfamily protein	rhodanese-like domain protein
Sapur.012G020900.2	0.015343454	-0.019374104	2.904824395	-0.00667	0.994678445	NA	brown	-0.081608864	0.309590942	GO:0033314,GO:0010212,GO:0006260,GO:0005634	AT5G52950.1			LRR receptor-like kinase PEPR2, putative
Sapur.012G065300.1	0.017139707	-0.019374104	2.904824395	-0.00667	0.994678445	NA	brown	-0.113693096	0.156252676		AT4G24680.1	MOS1	modifier of snc1	modifier OF SNC1 1, putative
Sapur.012G066400.14	0.015343454	-0.019374104	2.904824395	-0.00667	0.994678445	NA	brown	-0.081608864	0.309590942	GO:0006468,GO:0004672	AT4G24740.1	AFC2,AME1,FC2	FUS3-complementing gene 2	Serine/Threonine kinase AFC2
Sapur.013G043100.6	0.016836611	-0.019374104	2.904824395	-0.00667	0.994678445	NA	brown	-0.141845144	0.076380763	GO:0016829,GO:0006520	AT1G08630.4	THA1	threonine aldolase 1	L-allo-threonine aldolase
Sapur.013G115400.2	0.018930546	-0.019374104	2.904824395	-0.00667	0.994678445	NA	brown	-0.108038452	0.178029846	GO:0005515	AT3G59660.1		C2 domain-containing protein / GRAM domain-containing protein	GRAM domain protein
Sapur.014G093000.1	0.015343454	-0.019374104	2.904824395	-0.00667	0.994678445	NA	brown	-0.081608864	0.309590942		AT5G14670.1	ARFA1B,ATARFA1B	ADP-ribosylation factor A1B	ADP-ribosylation factor(Arf)/Arf-like (Arl) small GTPase family protein
Sapur.014G093800.1	0.015343454	-0.019374104	2.904824395	-0.00667	0.994678445	NA	brown	-0.081608864	0.309590942	GO:0045892,GO:0005634,GO:0043968,GO:0043967,GO:0035267,GO:0006338,GO:00062	AT2G47210.1		myb-like transcription factor family protein	DNA methyltransferase 1-associated protein
Sapur.014G095500.3	0.016136294	-0.019374104	2.904824395	-0.00667	0.994678445	NA	brown	-0.111947836	0.162749112		AT2G47350.2		HIT zinc finger ;PAPA-1-like conserved region	INO80 complex subunit B, putative
Sapur.016G063400.3	0.012282353	-0.019374104	2.904824395	-0.00667	0.994678445	NA	brown	-0.114085747	0.154818319	GO:0016888	AT3G52390.2		TatD related DNase	deoxyribonuclease TATDN1-like protein
Sapur.016G091800.1	0.022147566	-0.019374104	2.904824395	-0.00667	0.994678445	NA	brown	-0.115431234	0.149978313					NA
Sapur.016G146000.1	0.01472906	-0.019374104	2.904824395	-0.00667	0.994678445	NA	brown	-0.108881201	0.174649489	GO:0003676,GO:0046872	AT5G12440.1		CCCH-type zinc fingerfamily protein with RNA-binding domain	zinc finger C-x8-C-x5-C-x3-H type protein, putative
Sapur.016G150600.2	0.016292485	-0.019374104	2.904824395	-0.00667	0.994678445	NA	brown	-0.141280927	0.077570056	GO:0006412,GO:0005840,GO:0005622,GO:0003735	AT5G59240.1		Ribosomal protein S8e family protein	40S ribosomal protein S8
Sapur.017G002700.1	0.015187171	-0.019374104	2.904824395	-0.00667	0.994678445	NA	brown	-0.109832681	0.170890301		AT2G31820.1		Ankyrin repeat family protein	ankyrin-like protein
Sapur.017G058900.1	0.015343454	-0.019374104	2.904824395	-0.00667	0.994678445	NA	brown	-0.081608864	0.309590942	GO:0005524	AT5G20420.1	CHR42	chromatin remodeling 42	NA
Sapur.017G072000.2	0.015321281	-0.019374104	2.904824395	-0.00667	0.994678445	NA	brown	-0.110170366	0.16957068		AT5G39530.1		Protein of unknown function (DUF1997)	DUF1997 family protein
Sapur.019G062300.1	0.015343454	-0.019374104	2.904824395	-0.00667	0.994678445	NA	brown	-0.081608864	0.309590942		AT1G43760.1		DNAse I-like superfamily protein	NA
Sapur.019G110100.1	0.016045318	-0.019374104	2.904824395	-0.00667	0.994678445	NA	brown	-0.141106831	0.077940028	GO:0016021,GO:0012511	AT3G18570.1		Oleosin family protein	oleosin protein
Sapur.15WG005000.1	0.018039382	-0.019374104	2.904824395	-0.00667	0.994678445	NA	brown	-0.114746599	0.152426615		AT5G53620.2			hypothetical protein
Sapur.15WG106900.4	0.016325699	-0.019374104	2.904824395	-0.00667	0.994678445	NA	brown	-0.005752765	0.942994639	GO:0016020,GO:0006075,GO:0003843,GO:0000148	AT2G36850.1	ATGSL08,ATGSL8,CHOR,GSL	glucan synthase-like 8	1%2C3-beta-glucan synthase
Sapur.T064500.2	0.015343454	-0.019374104	2.904824395	-0.00667	0.994678445	NA	brown	-0.081608864	0.309590942		AT5G08420.1		RNA-binding KH domain-containing protein	KRR1 small subunit processome component
Sapur.018G108700.1	0.042660772	0.012346061	1.853441671	0.006661	0.994685207	NA	brown	-0.055373128	0.490944336	GO:0006310,GO:0006281,GO:0003910,GO:0003677,GO:0005524	AT5G57160.1	ATLIG4,LIG4	DNA ligase IV	ATP-dependent DNA ligase
Sapur.013G001700.1	0.03772616	-0.015283561	2.312803794	-0.00661	0.994727426	NA	brown	-0.071439856	0.373937298		AT3G05858.1			hypothetical protein
Sapur.016G027500.1	0.011042025	0.018098013	2.904824395	0.00623	0.995028949	NA	brown	-0.115376283	0.150173717		AT5G45790.2		Ubiquitin carboxyl-terminal hydrolase family protein	plant organelle RNA recognition domain protein
Sapur.001G000900.2	0.010393365	0.018098013	2.904824395	0.00623	0.995028949	NA	brown	-0.115695728	0.149040462		AT4G26410.1		Uncharacterised conserved protein UCP022280	hypothetical protein
Sapur.001G009700.3	0.011558949	0.018098013	2.904824395	0.00623	0.995028949	NA	brown	-0.114951456	0.151690895		AT3G06880.2		Transducin/WD40 repeat-like superfamily protein	ubox-like protein, putative
Sapur.001G016200.2	0.011781475	0.018098013	2.904824395	0.00623	0.995028949	NA	brown	-0.081608864	0.309590942	GO:0046872,GO:0030001,GO:0000166	AT1G63440.1	HMA5	heavy metal atpase 5	heavy metal P-type ATPase
Sapur.001G041200.1	0.010022524	0.018098013	2.904824395	0.00623	0.995028949	NA	brown	-0.081608864	0.309590942	GO:0071949	AT3G24200.1		FAD/NAD(P)-binding oxidoreductase family protein	ubiquinone biosynthesis monooxygenase COQ6
Sapur.001G074300.5	0.009772458	0.018098013	2.904824395	0.00623	0.995028949	NA	brown	-0.115740333	0.148882736		AT1G64430.1		Pentatricopeptide repeat (PPR) superfamily protein	PPR domain protein
Sapur.001G093300.2	0.010228969	0.018098013	2.904824395	0.00623	0.995028949	NA	brown	-0.081608864	0.309590942	GO:0006355,GO:0005634,GO:0005524,GO:0032502,GO:0006351	AT4G37740.1	AtGRF2,GRF2	growth-regulating factor 2	NA
Sapur.001G162900.2	0.01003829	0.018098013	2.904824395	0.00623	0.995028949	NA	brown	-0.115783338	0.148730788	GO:0043565,GO:0008270,GO:0006355,GO:0003700	AT4G32890.1	GATA9	GATA transcription factor 9	GATA transcription factor
Sapur.002G023700.4	0.012360172	0.018098013	2.904824395	0.00623	0.995028949	NA	brown	-0.021570425	0.788585369	GO:0016021	AT1G35180.1		TRAM, LAG1 and CLN8 (TLC) lipid-sensing domain containing protein	TRAM, LAG1 and CLN8 (TLC) lipid-sensing domain protein, putative
Sapur.002G044100.3	0.010852461	0.018098013	2.904824395	0.00623	0.995028949	NA	brown	-0.115531724	0.149621469		AT3G10260.3		Reticulon family protein	reticulon protein
Sapur.002G106600.2	0.011712934	0.018098013	2.904824395	0.00623	0.995028949	NA	brown	-0.081608864	0.309590942	GO:0006468,GO:0004672	AT1G55200.1		Protein kinase protein with adenine nucleotide alpha hydrolases-like domain	receptor tyrosine kinase
Sapur.002G136500.2	0.011712934	0.018098013	2.904824395	0.00623	0.995028949	NA	brown	-0.114846535	0.152067369		AT1G02270.1		Calcium-binding endonuclease/exonuclease/phosphatase family	calcium-binding endonuclease/exonuclease/phosphatase family protein
Sapur.002G167500.4	0.01210369	0.018098013	2.904824395	0.00623	0.995028949	NA	brown	-0.114218666	0.154335013		AT1G02475.1		Polyketide cyclase/dehydrase and lipid transport superfamily protein	polyketide cyclase/dehydrase and lipid transporter
Sapur.002G189800.3	0.010228969	0.018098013	2.904824395	0.00623	0.995028949	NA	brown	-0.081608864	0.309590942	GO:0042765,GO:0016255	AT3G07180.1		GPI transamidase component PIG-S-related	phosphatidylinositol-glycan biosynthesis class S protein
Sapur.002G202900.1	0.010228969	0.018098013	2.904824395	0.00623	0.995028949	NA	brown	-0.081608864	0.309590942	GO:0032259,GO:0008168	AT5G42760.1		Leucine carboxyl methyltransferase	leucine carboxyl methyltransferase
Sapur.003G026700.1	0.01186289	0.018098013	2.904824395	0.00623	0.995028949	NA	brown	-0.114519661	0.15324477	GO:0005515	AT1G49450.1		Transducin/WD40 repeat-like superfamily protein	F-box and WD40 domain protein, putative
Sapur.003G072100.4	0.010228969	0.018098013	2.904824395	0.00623	0.995028949	NA	brown	-0.081608864	0.309590942	GO:0005089	AT1G31650.1	ATROPGEF14,ROPGEF14	RHO guanyl-nucleotide exchange factor 14	Rop guanine nucleotide exchange factor, putative
Sapur.003G095400.2	0.010228969	0.018098013	2.904824395	0.00623	0.995028949	NA	brown	-0.081608864	0.309590942	GO:0055114,GO:0016491,GO:0006633,GO:0005506	AT4G25700.1	B1,BCH1,BETA-OHASE 1,ch	beta-hydroxylase 1	beta-carotene hydroxylase
Sapur.003G096400.1	0.010228969	0.018098013	2.904824395	0.00623	0.995028949	NA	brown	-0.081608864	0.309590942	GO:0003676	AT1G63980.2		D111/G-patch domain-containing protein	Pin2-interacting protein x1, putative
Sapur.003G111900.5	0.009879659	0.018098013	2.904824395	0.00623	0.995028949	NA	brown	-0.115749337	0.148850914		AT5G51180.2		alpha/beta-Hydrolases superfamily protein	serine esterase family protein
Sapur.003G117500.1	0.011159397	0.018098013	2.904824395	0.00623	0.995028949	NA	brown	-0.115228114	0.150701564		AT5G47900.1		Protein of unknown function (DUF1624)	transmembrane protein, putative
Sapur.003G152200.1	0.010228969	0.018098013	2.904824395	0.00623	0.995028949	NA	brown	-0.081608864	0.309590942	GO:0016787	AT3G55850.1	LAF3,LAF3 ISF1,LAF3 ISF2	Amidohydrolase family	amidohydrolase
Sapur.004G001200.3	0.010228969	0.018098013	2.904824395	0.00623	0.995028949	NA	brown	-0.081608864	0.309590942	GO:0055114,GO:0009916	AT4G22260.1	IM,IM1	Alternative oxidase family protein	ubiquinol oxidase
Sapur.004G011700.1	0.011649124	0.018098013	2.904824395	0.00623	0.995028949	NA	brown	-0.114798493	0.152239988	GO:0009982,GO:0009451,GO:0003723,GO:0001522	AT4G21770.1		Pseudouridine synthase family protein	RNA pseudouridine synthase
Sapur.004G012100.5	0.010228969	0.018098013	2.904824395	0.00623	0.995028949	NA	brown	-0.081608864	0.309590942	GO:0005975,GO:0004553	AT4G21760.1	BGLU47	beta-glucosidase 47	NA
Sapur.004G016000.3	0.010228969	0.018098013	2.904824395	0.00623	0.995028949	NA	brown	-0.07302262	0.363417017	GO:0006468,GO:0004672	AT4G05200.1	CRK25	cysteine-rich RLK (RECEPTOR-like protein kinase) 25	cysteine-rich receptor-like kinase
Sapur.004G034200.3	0.009970499	0.018098013	2.904824395	0.00623	0.995028949	NA	brown	-0.041134674	0.608991308	GO:0048193,GO:0016020,GO:0005515	AT1G28490.2	ATSYP61,OSM1,SYP61	syntaxin of plants 61	syntaxin-61 protein
Sapur.004G051700.2	0.009530349	0.018098013	2.904824395	0.00623	0.995028949	NA	brown	-0.081608864	0.309590942		AT4G25700.1	B1,BCH1,BETA-OHASE 1,ch	beta-hydroxylase 1	beta-carotene hydroxylase
Sapur.004G062300.1	0.010228969	0.018098013	2.904824395	0.00623	0.995028949	NA	brown	-0.081608864	0.309590942		AT1G70750.1		Protein of unknown function, DUF593	zein-binding protein
Sapur.004G115600.2	0.010228969	0.018098013	2.904824395	0.00623	0.995028949	NA	brown	-0.081608864	0.309590942	GO:0005975,GO:0004553	AT2G16230.1		O-Glycosyl hydrolases family 17 protein	glucan endo-1%2C3-beta-glucosidase
Sapur.004G158700.1	0.010228969	0.018098013	2.904824395	0.00623	0.995028949	NA	brown	-0.081608864	0.309590942		AT5G60550.1	ATSNAK1,GRIK2	geminivirus rep interacting kinase 2	calcium/calmodulin-dependent kinase kinase
Sapur.005G000800.2	0.011634165	0.018098013	2.904824395	0.00623	0.995028949	NA	brown	-0.114778505	0.152311849		AT1G09540.1	ATMYB61,MYB61	myb domain protein 61	R2R3-MYB transcription factor, putative
Sapur.005G053700.2	0.010228969	0.018098013	2.904824395	0.00623	0.995028949	NA	brown	-0.081608864	0.309590942	GO:0005515	AT3G52950.1		CBS / octicosapeptide/Phox/Bemp1 (PB1) domains-containing protein	meiotically up-regulated protein
Sapur.005G063400.2	0.010228969	0.018098013	2.904824395	0.00623	0.995028949	NA	brown	-0.081608864	0.309590942	GO:0008270,GO:0005515	AT1G15100.1	RHA2A	RING-H2 finger A2A	zinc finger, C3HC4 type (RING finger) protein
Sapur.005G141300.1	0.011052775	0.018098013	2.904824395	0.00623	0.995028949	NA	brown	-0.115320782	0.150371276	GO:0006355,GO:0003677	AT1G01720.1	ANAC002,ATAF1	NAC (No Apical Meristem) domain transcriptional regulator superfamily protein	NAC transcription factor, putative
Sapur.005G142400.3	0.011649124	0.018098013	2.904824395	0.00623	0.995028949	NA	brown	-0.114798493	0.152239988	GO:0005524	AT1G21690.1	EMB1968,RFC4	ATPase family associated with various cellular activities (AAA)	replication factor C subunit 2/4
Sapur.005G162500.2	0.010228969	0.018098013	2.904824395	0.00623	0.995028949	NA	brown	-0.081608864	0.309590942	GO:0006412,GO:0005840,GO:0005622,GO:0003735	AT2G32060.2		Ribosomal protein L7Ae/L30e/S12e/Gadd45 family protein	40S ribosomal protein S12
Sapur.005G162800.8	0.012580222	0.018098013	2.904824395	0.00623	0.995028949	NA	brown	-0.113764496	0.155991112	GO:0005515	AT5G08560.1		transducin family protein / WD-40 repeat family protein	transducin/WD40 domain protein
Sapur.005G173300.2	0.011545881	0.018098013	2.904824395	0.00623	0.995028949	NA	brown	-0.114902006	0.151868242	GO:0006355,GO:0005634	AT5G43700.1	ATAUX2-11,IAA4	AUX/IAA transcriptional regulator family protein	indole-3-acetic acid inducible protein
Sapur.005G174700.1	0.010228969	0.018098013	2.904824395	0.00623	0.995028949	NA	brown	-0.081608864	0.309590942	GO:0043565,GO:0006355,GO:0003700	AT2G30590.1	WRKY21	WRKY DNA-binding protein 21	WRKY transcription factor, putative
Sapur.005G178300.2	0.011043891	0.018098013	2.904824395	0.00623	0.995028949	NA	brown	-0.1075461	0.180026907		AT4G14385.1			histone acetyltransferase subunit NuA4
Sapur.006G025200.2	0.011005222	0.018098013	2.904824395	0.00623	0.995028949	NA	brown	-0.115365221	0.150213079	GO:0005634	AT5G06150.1	CYC1BAT,CYCB1;2	Cyclin family protein	cyclin B

Sapur.006G069500.2	0.011200174	0.018098013	2.904824395	0.00623	0.995028949	NA	brown	-0.114869942	0.151983321	GO:0008017,GO:0007018,GO:0005524,GO:0003777,GO:0005871	AT5G02370.1		ATP binding microtubule motor family protein	kinesin motor domain protein
Sapur.006G081500.2	0.01055776	0.018098013	2.904824395	0.00623	0.995028949	NA	brown	-0.081608864	0.309590942		AT5G01510.1	RUS5	Protein of unknown function, DUF647	DUF647 domain protein
Sapur.006G090700.4	0.011637467	0.018098013	2.904824395	0.00623	0.995028949	NA	brown	-0.114879715	0.151948239	GO:0005515,GO:0006468,GO:0005524,GO:0004672	AT5G01950.1		Leucine-rich repeat protein kinase family protein	LRR receptor-like kinase
Sapur.006G101900.1	0.010228969	0.018098013	2.904824395	0.00623	0.995028949	NA	brown	-0.081608864	0.309590942	GO:0017183	AT3G59630.1		diphthamide synthesis DPH2 family protein	diphthamide biosynthesis enzyme DPH2
Sapur.006G103100.5	0.010228969	0.018098013	2.904824395	0.00623	0.995028949	NA	brown	-0.081608864	0.309590942		AT2G36960.3	TKI1	TSL-kinase interacting protein 1	MYB DNA-binding domain protein, putative
Sapur.006G122300.4	0.011188101	0.018098013	2.904824395	0.00623	0.995028949	NA	brown	-0.115202025	0.150794649	GO:0006468,GO:0005524,GO:0004672	AT4G26100.1	CK1,CKL1	casein kinase 1	casein kinase I-like protein
Sapur.006G129400.2	0.010228969	0.018098013	2.904824395	0.00623	0.995028949	NA	brown	-0.081608864	0.309590942	GO:0030244,GO:0016760,GO:0016020	AT2G21770.1	CESA09,CESA9	cellulose synthase A9	cellulose synthase A catalytic subunit 6 [UDP-forming] protein, putative
Sapur.006G147700.2	0.011005222	0.018098013	2.904824395	0.00623	0.995028949	NA	brown	-0.115365221	0.150213079	GO:0030244,GO:0016760,GO:0016020	AT5G17420.1	ATCESA7,CESA7,IRX3,MUR1	Cellulose synthase family protein	cellulose synthase A catalytic subunit 9 [UDP-forming] protein
Sapur.006G175800.2	0.010228969	0.018098013	2.904824395	0.00623	0.995028949	NA	brown	-0.081608864	0.309590942		AT1G07710.1		Ankyrin repeat family protein	viral ankyrin protein
Sapur.006G179300.3	0.010228969	0.018098013	2.904824395	0.00623	0.995028949	NA	brown	-0.081608864	0.309590942		AT2G22620.1		Rhamnogalacturonate lyase family protein	rhamnogalacturonate lyase
Sapur.006G180900.2	0.010921843	0.018098013	2.904824395	0.00623	0.995028949	NA	brown	-0.143610377	0.072754712	GO:0044237	AT2G05830.4		NagB/RpiA/CoA transferase-like superfamily protein	methylthioribose-1-phosphate isomerase
Sapur.006G207800.5	0.010228969	0.018098013	2.904824395	0.00623	0.995028949	NA	brown	-0.131590434	0.100424477	GO:0006457,GO:0003755,GO:0000413	AT4G32420.1		Cyclophilin-like peptidyl-prolyl cis-trans isomerase family protein	peptidyl-prolyl cis-trans isomerase, cyclophilin-type protein
Sapur.007G025500.5	0.010228969	0.018098013	2.904824395	0.00623	0.995028949	NA	brown	-0.081608864	0.309590942	GO:0003676	AT4G36690.4	ATU2AF65A	U2 snRNP auxilliary factor, large subunit, splicing factor	U2 snRNP auxilliary factor, large subunit%2C splicing factor
Sapur.007G104000.3	0.010848133	0.018098013	2.904824395	0.00623	0.995028949	NA	brown	-0.115490588	0.149767465	GO:0005515	AT3G21175.3	GATA24,TIFY2B,ZML1	ZIM-like 1	CCT motif protein
Sapur.008G037700.2	0.011200174	0.018098013	2.904824395	0.00623	0.995028949	NA	brown	-0.114869942	0.151983321		AT2G39420.1		alpha/beta-Hydrolases superfamily protein	alpha/beta fold hydrolase
Sapur.008G069000.2	0.011279689	0.018098013	2.904824395	0.00623	0.995028949	NA	brown	-0.115135415	0.151032512		AT1G69580.1		Homeodomain-like superfamily protein	transcription factor, putative
Sapur.008G077300.1	0.011294107	0.018098013	2.904824395	0.00623	0.995028949	NA	brown	-0.115107846	0.151131044	GO:0007275	AT1G69180.1	CRC	Plant-specific transcription factor YABBY family protein	YABBY-like transcription factor CRABS CLAW-like protein
Sapur.008G082700.2	0.010228969	0.018098013	2.904824395	0.00623	0.995028949	NA	brown	-0.081608864	0.309590942	GO:0003824,GO:0006470,GO:0004722	AT3G17090.1		Protein phosphatase 2C family protein	protein phosphatase 2C
Sapur.008G105500.3	0.010228969	0.018098013	2.904824395	0.00623	0.995028949	NA	brown	-0.081608864	0.309590942	GO:0016021,GO:0015116,GO:0008272,GO:0055085,GO:0016020,GO:0008271	AT1G23090.1	AST91,SULTR3;3	sulfate transporter 91	sulfate/bicarbonate/oxalate exchanger and transporter sat-1
Sapur.008G146200.7	0.010228969	0.018098013	2.904824395	0.00623	0.995028949	NA	brown	-0.081608864	0.309590942		AT2G02710.1	PLP,PLPA,PLPB,PLPC	PAS/LOV protein B	TWIN LOV protein, putative
Sapur.009G001500.2	0.010228969	0.018098013	2.904824395	0.00623	0.995028949	NA	brown	-0.081608864	0.309590942	GO:0005515	AT2G27980.1		Acyl-CoA N-acyltransferase with RING/FYVE/PHD-type zinc finger domain	chromodomain helicase-DNA-binding protein, putative
Sapur.009G030800.1	0.012360172	0.018098013	2.904824395	0.00623	0.995028949	NA	brown	-0.114024224	0.155042406	GO:0006468,GO:0005524,GO:0004672	AT5G58940.1	CRCK1	calmodulin-binding receptor-like cytoplasmic kinase 1	calmodulin-binding receptor-like cytoplasmic kinase
Sapur.009G050000.5	0.010228969	0.018098013	2.904824395	0.00623	0.995028949	NA	brown	-0.081608864	0.309590942		AT1G51140.1		basic helix-loop-helix (bHLH) DNA-binding superfamily protein	transcription factor bHLH122, putative
Sapur.009G090300.2	0.010228969	0.018098013	2.904824395	0.00623	0.995028949	NA	brown	-0.081608864	0.309590942		AT1G11750.2	CLPP6,NCLPP1,NCLPP6	CLP protease proteolytic subunit 6	ATP-dependent Clp protease proteolytic subunit
Sapur.009G112600.4	0.010228969	0.018098013	2.904824395	0.00623	0.995028949	NA	brown	-0.081608864	0.309590942		AT3G45970.1	ATEXLA1,ATEXPL1,ATHEXP I	expansin-like A1	expansin-like protein A1
Sapur.010G000500.1	0.010228969	0.018098013	2.904824395	0.00623	0.995028949	NA	brown	-0.081608864	0.309590942	GO:0043565,GO:0008270,GO:0006355,GO:0003700	AT3G06740.1	GATA15	GATA transcription factor 15	GATA transcription factor, putative
Sapur.010G028000.9	0.010228969	0.018098013	2.904824395	0.00623	0.995028949	NA	brown	-0.081608864	0.309590942	GO:0005975,GO:0004650	AT1G60590.1		Pectin lyase-like superfamily protein	polygalacturonase
Sapur.010G043600.3	0.009400506	0.018098013	2.904824395	0.00623	0.995028949	NA	brown	-0.115571053	0.149481983		AT1G32580.1		plastid developmental protein DAG, putative	plastid developmental protein DAG, putative
Sapur.010G055700.6	0.010228969	0.018098013	2.904824395	0.00623	0.995028949	NA	brown	-0.081608864	0.309590942	GO:0017069,GO:0000398				NA
Sapur.010G068700.2	0.011197577	0.018098013	2.904824395	0.00623	0.995028949	NA	brown	-0.115231604	0.150689116	GO:0006355,GO:0005634	AT5G43700.1	ATAUX2-11,IAA4	AUX/IAA transcriptional regulator family protein	auxin inducible protein IAA3
Sapur.010G078400.2	0.010228969	0.018098013	2.904824395	0.00623	0.995028949	NA	brown	-0.081608864	0.309590942	GO:0071805,GO:0016020,GO:0015079	AT1G60160.1		Potassium transporter family protein	potassium transporter
Sapur.010G113600.4	0.008585755	0.018098013	2.904824395	0.00623	0.995028949	NA	brown	-0.114330019	0.153931003					iron-sulfur-binding 4Fe-4S ferredoxin
Sapur.010G140000.3	0.011294107	0.018098013	2.904824395	0.00623	0.995028949	NA	brown	-0.115107846	0.151131044		AT3G48480.1		Cysteine proteinases superfamily protein	sentrin/sumo-specific protease, putative
Sapur.010G157400.2	0.009879659	0.018098013	2.904824395	0.00623	0.995028949	NA	brown	-0.115749337	0.148850914	GO:0005524	AT2G39730.2	RCA	rubisco activase	ribulose-1%2C5-bisphosphate carboxylase/oxygenase activase
Sapur.011G054900.7	0.011649124	0.018098013	2.904824395	0.00623	0.995028949	NA	brown	-0.014680064	0.855205564	GO:0016020	AT5G46150.1		LEM3 (ligand-effect modulator 3) family protein / CDC50 family protein	LEM3/CDC50 family protein
Sapur.011G055700.3	0.012862793	0.018098013	2.904824395	0.00623	0.995028949	NA	brown	-0.081608864	0.309590942	GO:0048544,GO:0006468,GO:0004672	AT1G11340.1		S-locus lectin protein kinase family protein	Serine/Threonine-kinase
Sapur.011G057900.2	0.011138415	0.018098013	2.904824395	0.00623	0.995028949	NA	brown	-0.115259176	0.15059079		AT2G34460.1		NAD(P)-binding Rossmann-fold superfamily protein	NAD-dependent epimerase/dehydratase family protein
Sapur.011G073800.2	0.010228969	0.018098013	2.904824395	0.00623	0.995028949	NA	brown	-0.081608864	0.309590942	GO:0016757	AT1G16900.1		Alg9-like mannosyltransferase family	alpha-1%2C2-mannosyltransferase ALG9
Sapur.011G105600.2	0.012214214	0.018098013	2.904824395	0.00623	0.995028949	NA	brown	-0.114085747	0.154818319		AT3G12920.1		SBP (S-ribonuclease binding protein) family protein	zinc ion-binding protein
Sapur.011G119300.1	0.010228969	0.018098013	2.904824395	0.00623	0.995028949	NA	brown	-0.081608864	0.309590942		AT2G34780.1	EMB1611,MEE22	maternal effect embryo arrest 22	EMB1611/MEE22 protein, putative
Sapur.012G084000.2	0.010228969	0.018098013	2.904824395	0.00623	0.995028949	NA	brown	-0.081608864	0.309590942	GO:0005515,GO:0016818,GO:0008270,GO:0003676,GO:0008081,GO:0006281,GO:000563	AT5G07400.1		forkhead-associated domain-containing protein / FHA domain-containing protein	tyrosyl-DNA phosphodiesterase
Sapur.012G097000.1	0.012476308	0.018098013	2.904824395	0.00623	0.995028949	NA	brown	-0.115744615	0.148867602	GO:0006355,GO:0003677	AT1G12260.1	ANAC007,EMB2749,NAC007	NAC 007	NAC transcription factor
Sapur.013G003300.3	0.010228969	0.018098013	2.904824395	0.00623	0.995028949	NA	brown	-0.081608864	0.309590942		AT2G05790.1		O-Glycosyl hydrolases family 17 protein	hydrolyzing O-glycosyl compounds hydrolase, putative
Sapur.013G019500.1	0.010848939	0.018098013	2.904824395	0.00623	0.995028949	NA	brown	-0.115385142	0.150142205		AT1G09030.1	NF-YB4	nuclear factor Y, subunit B4	nuclear transcription factor protein
Sapur.013G033700.1	0.011545881	0.018098013	2.904824395	0.00623	0.995028949	NA	brown	-0.114902006	0.151868242	GO:0008017,GO:0007018,GO:0005524,GO:0003777,GO:0005871	AT5G27950.1		P-loop containing nucleoside triphosphate hydrolases superfamily protein	kinesin motor domain protein
Sapur.013G069800.1	0.009400506	0.018098013	2.904824395	0.00623	0.995028949	NA	brown	-0.115571053	0.149481983		AT5G35405.1		Protein of unknown function (DUF784)	NA
Sapur.013G090700.14	0.010228969	0.018098013	2.904824395	0.00623	0.995028949	NA	brown	-0.081608864	0.309590942		AT3G10840.1		alpha/beta-Hydrolases superfamily protein	alpha/beta hydrolase, putative
Sapur.014G084400.2	0.012399904	0.018098013	2.904824395	0.00623	0.995028949	NA	brown	-0.081608864	0.309590942		AT1G01060.3	LHY,LHY1	Homeodomain-like superfamily protein	late elongated hypocotyl and circadian clock associated-like protein
Sapur.014G091400.1	0.010228969	0.018098013	2.904824395	0.00623	0.995028949	NA	brown	-0.081608864	0.309590942		AT5G67460.1		O-Glycosyl hydrolases family 17 protein	NA
Sapur.014G129000.4	0.010228969	0.018098013	2.904824395	0.00623	0.995028949	NA	brown	-0.081608864	0.309590942		AT2G32970.2			rice genome annotation project protein, putative
Sapur.014G132800.3	0.010999636	0.018098013	2.904824395	0.00623	0.995028949	NA	brown	-0.113158656	0.158221001	GO:0043039,GO:0016876,GO:0005524,GO:0006418,GO:0004812,GO:0000166	AT2G04842.1	EMB2761	threonyl-tRNA synthetase, putative / threonine--tRNA ligase, putative	threonyl-tRNA synthetase/threonine-tRNA ligase, putative
Sapur.016G030700.3	0.010228969	0.018098013	2.904824395	0.00623	0.995028949	NA	brown	-0.081608864	0.309590942	GO:0006508,GO:0004185	AT2G33530.1	scpl46	serine carboxypeptidase-like 46	serine carboxypeptidase, putative
Sapur.016G050000.2	0.010228969	0.018098013	2.904824395	0.00623	0.995028949	NA	brown	-0.081608864	0.309590942		AT2G41810.1		Protein of unknown function, DUF642	transmembrane protein, putative
Sapur.016G087200.2	0.019519677	0.018098013	2.904824395	0.00623	0.995028949	NA	brown	-0.081608864	0.309590942		AT5G52970.1		thylakoid lumen 15.0 kDa protein	thylakoid lumen 15.0 kDa protein, putative
Sapur.016G090600.5	0.010228969	0.018098013	2.904824395	0.00623	0.995028949	NA	brown	-0.081608864	0.309590942		AT2G37680.1			vacuolar import and degradation protein
Sapur.016G091200.2	0.010228969	0.018098013	2.904824395	0.00623	0.995028949	NA	brown	-0.081608864	0.309590942		AT3G53850.1		Uncharacterised protein family (UPF0497)	CASP-like protein Os09g0249400
Sapur.016G129000.2	0.010228969	0.018098013	2.904824395	0.00623	0.995028949	NA	brown	-0.081608864	0.309590942		AT5G59770.1		Protein-tyrosine phosphatase-like, PTPLA	tyrosine phosphatase-like protein PTPLB protein, putative
Sapur.016G142700.2	0.010228969	0.018098013	2.904824395	0.00623	0.995028949	NA	brown	-0.081608864	0.309590942	GO:0016021,GO:0015693,GO:0015095	AT1G34470.1		Protein of unknown function (DUF803)	Non-imprinted in prader-willi/angelman syndrome region protein, putative
Sapur.016G169400.2	0.010455254	0.018098013	2.904824395	0.00623	0.995028949	NA	brown	-0.115729365	0.148921509	GO:0055085,GO:0016021	AT5G64500.1		Major facilitator superfamily protein	NA
Sapur.016G173700.1	0.010228969	0.018098013	2.904824395	0.00623	0.995028949	NA	brown	-0.150928516	0.059181848	GO:0006508,GO:0004185	AT1G33540.1	scpl18	serine carboxypeptidase-like 18	serine carboxypeptidase, putative
Sapur.016G203800.2	0.010228969	0.018098013	2.904824395	0.00623	0.995028949	NA	brown	-0.081608864	0.309590942	GO:0006412,GO:0005840,GO:0005622,GO:0003735	AT5G40950.1	RPL27	ribosomal protein large subunit 27	50S ribosomal protein L27
Sapur.016G261000.5	0.01169444	0.018098013	2.904824395	0.00623	0.995028949	NA	brown	-0.114728867	0.152490423	GO:0016491,GO:0008152	AT5G54190.1	PORA	protochlorophyllide oxidoreductase A	NADPH-protochlorophyllide oxidoreductase
Sapur.016G282800.1	0.010228969	0.018098013	2.904824395	0.00623	0.995028949	NA	brown	-0.081608864	0.309590942		AT4G13530.2			hypothetical protein
Sapur.016G287100.1	0.01107595	0.018098013	2.904824395	0.00623	0.995028949	NA	brown	-0.115274554	0.150535973	GO:0007034	AT5G44560.1	VPS2.2	SNF7 family protein	charged multivesicular body protein
Sapur.016G290200.3	0.010228969	0.018098013	2.904824395	0.00623	0.995028949	NA	brown	-0.081608864	0.309590942	GO:0016020,GO:0008378,GO:0006486	AT1G32930.1		Galactosyltransferase family protein	beta-1%2C3-galactosyltransferase, putative
Sapur.016G290400.2	0.010228969	0.018098013	2.904824395	0.00623	0.995028949	NA	brown	-0.081608864	0.309590942	GO:0006508,GO:0004252	AT1G32960.1	ATSBT3.3,SBT3.3	Subtilase family protein	subtilisin-like protease, putative
Sapur.017G016500.2	0.01186289	0.018098013	2.904824395	0.00623	0.995028949	NA	brown	-0.114519661	0.15324477	GO:0055114,GO:0004174	AT2G43400.1	ETFQO	electron-transfer flavoprotein:ubiquinone oxidoreductase	electron transfer flavoprotein ubiquinone oxidoreductase
Sapur.017G071600.2	0.009994783	0.018098013	2.904824395	0.00623	0.995028949	NA	brown	-0.115780113	0.148742181	GO:0016021	AT3G29060.1		EXS (ERD1/XPR1/SYG1) family protein	phosphate transporter PHO1-like protein
Sapur.017G092500.5	0.010228969	0.018098013	2.904824395	0.00623	0.995028949	NA	brown	-0.081608864	0.309590942	GO:0044237	AT5G38640.1		NagB/RpiA/CoA transferase-like superfamily protein	translation initiation factor 2B, delta subunit%2C putative
Sapur.017G108600.2	0.010946677	0.018098013	2.904824395	0.00623	0.995028949	NA	brown	-0.115431541	0.149977224	GO:0016787	AT2G01670.1	atnudt17,NUDT17	nudix hydrolase homolog 17	nudix hydrolase
Sapur.018G023400.3	0.010228969	0.018098013	2.904824395	0.00623	0.995028949	NA	brown	-0.081608864	0.309590942		AT4G32330.1		TPX2 (targeting protein for Xklp2) protein family	targeting protein for Xklp2 protein
Sapur.018G048700.3	0.01006764	0.018098013	2.904824395	0.00623	0.995028949	NA	brown	-0.015631176	0.845937207		AT5G56750.1	NDL1	N-MYC downregulated-like 1	pollen specific protein SF21, putative
Sapur.018G060900.2	0.011051543	0.018098013	2.904824395	0.00623	0.995028949	NA	brown	-0.115294919	0.150463403	GO:0003676,GO:0006414,GO:0005622,GO:0003746	AT4G29060.1	emb2726	elongation factor Ts family protein	elongation factor Ts protein
Sapur.018G108100.2	0.010228969	0.018098013	2.904824395	0.00623	0.995028949	NA	brown	-0.081608864	0.309590942		AT4G26020.1			4/1 protein short form protein, putative
Sapur.019G018500.2	0.010228969	0.018098013	2.904824395	0.00623	0.995028949	NA	brown	-0.015718223	0.845089993	GO:0005515,GO:0006468,GO:0004672	AT3G14840.2		Leucine-rich repeat transmembrane protein kinase	NA
Sapur.019G070600.2	0.010228969	0.018098013	2.904824395	0.00623	0.995028949	NA	brown	-0.081608864	0.309590942	GO:0016021,GO:0006665,GO:0004348	AT5G49900.1		Beta-glucosidase, GBA2 type family protein	NA
Sapur.019G086400.1	0.010228969	0.018098013	2.904824395	0.00623	0.995028949	NA	brown	-0.081608864	0.309590942					transmembrane protein, putative
Sapur.019G107600.2	0.010228969	0.018098013	2.904824395	0.00623	0.995028949	NA	brown	-0.081608864	0.309590942	GO:0005515	AT4G14096.1		F-box/RNI-like superfamily protein	F-box protein
Sapur.15WG019700.1	0.010228969	0.018098013	2.904824395	0.00623	0.995028949	NA	brown	-0.081608864	0.309590942	GO:0006810,GO:0005643	AT2G41620.1		Nucleoporin interacting component (Nup93/Nic96-like) family protein	nucleoporin interacting component, putative
Sapur.15WG072900.1	0.012456334	0.018098013	2.904824395	0.00623	0.995028949	NA	brown	-0.113839493	0.155716725	GO:0016592,GO:0006357,GO:0001104	AT5G20170.1		RNA polymerase II transcription mediators	mediator of RNA polymerase II transcription subunit 17
Sapur.15WG128900.2	0.010228969	0.018098013	2.904824395	0.00623	0.995028949	NA	brown	-0.081608864	0.309590942	GO:0034477,GO:0004518	AT5G51170.1			NA
Sapur.T001300.3	0.010228969	0.018098013	2.904824395	0.00623	0.995028949	NA	brown	-0.081608864	0.309590942		AT4G26980.1		RNI-like superfamily protein	hypothetical protein
Sapur.T032600.1	0.012025222	0.018098013	2.904824395	0.00623	0.995028949	NA	brown	-0.114460008	0.153460381		AT4G13440.1		Calcium-binding EF-hand family protein	NA
Sapur.T053000.3	0.010228969	0.018098013	2.904824395	0.00623	0.995028949	NA	brown	-0.081608864	0.309590942	GO:0046938,GO:0046872,GO:0016756,GO:0010038	AT5G44070.1	ARA8,ATPCS1,CAD1,PCS1	phytochelatin synthase 1 (PCS1)	phytochelatin synthase
Sapur.T074100.5	0.010228969	0.018098013	2.904824395	0.00623	0.995028949	NA	brown	-0.081608864	0.309590942		AT4G16440.1		ferredoxin hydrogenases	cytosolic Fe-S cluster assembly factor narfl protein
Sapur.T080300.1	0.011314437	0.018098013	2.904824395	0.00623	0.995028949	NA	brown	-0.115216798	0.150741936	GO:0016787	AT4G19180.1		GDA1/CD39 nucleoside phosphatase family protein	nucleoside phosphatase family protein
Sapur.T090300.1	0.010228969	0.018098013	2.904824395	0.00623	0.995028949	NA	brown	-0.081608864	0.309590942	GO:0005634,GO:0003677,GO:0005515	AT3G01460.1	ATMBD9,MBD9	methyl-CPG-binding domain 9	methyl-CpG-binding domain protein
Sapur.T117400.1	0.012596601	0.018098013	2.904824395	0.00623	0.995028949	NA	brown	-0.115765136	0.148795088					NA
Sapur.016G222500.1	0.034491131	0.0143212	2.325189287	0.006159	0.995085737	NA	brown	-0.045054967	0.575267665		AT1G74860.1			NA
Sapur.004G016000.2	0.034393697	0.014321328	2.325917872	0.006157	0.995087232	NA	brown	-0.081608864	0.309590942	GO:0006468,GO:0004672	AT4G05200.1	CRK25	cysteine-rich RLK (RECEPTOR-like protein kinase) 25	cysteine-rich receptor-like kinase
Sapur.016G287000.3	0.033579912	0.014322406	2.332102795	0.006141	0.995099892	NA	brown	-0.073139303	0.362648776		AT4G20380.2	LSD1	LSD1 zinc finger family protein	zinc finger protein LSD1
Sapur.009G020400.2	0.033084873	0.014323074	2.335954986	0.006132	0.995107745	NA	brown	-0.103163625	0.198533692	GO:0005525	AT5G59840.1		Ras-related small GTP-binding family protein	Ras small GTPase family Ras protein
Sapur.018G052000.1	0.03199956	0.014324567	2.344651233	0.006109	0.995125382	NA	brown	-0.047850525	0.551766453	GO:0006468,GO:0005524,GO:0004672	AT5G20050.1		Protein kinase superfamily protein	receptor-like kinase, putative
Sapur.008G019300.1	0.030742152	0.014326373	2.355306664	0.006083	0.995146822	NA	brown	-0.076184745	0.34295487					NA
Sapur.001G118400.2	0.040918227	0.012408284	2.053878366	0.006041	0.995179696	NA	brown	-0.067615298	0.400117993		AT5G46800.1	BOU	Mitochondrial substrate carrier family protein	carnitine/acylcarnitine carrier-like protein
Sapur.018G023000.2	0.025360131	0.016229114	2.729078489	0.005947	0.995255218	NA	brown	-0.090002999	0.262288178		AT4G32295.2			hypothetical protein
Sapur.010G084000.1	0.023007687	0.016232604	2.763839061	0.005873	0.995313884	NA	brown	-0.066694381	0.406581313	GO:0009607,GO:0006952	AT1G70890.1	MLP43	MLP-like protein 43	Bet V I allergen family protein
Sapur.014G077300.1	0.022997886	0.016232619	2.763992924	0.005873	0.995314141	NA	brown	-0.051232755	0.523972815		AT3G61750.1		Cytochrome b561/ferric reductase transmembrane with DOMON related domain	dopamine beta-monooxygenase
Sapur.001G183400.1	0.022344488	0.016233639	2.774439674	0.005851	0.995331491	NA	brown	-0.082428444	0.304740904					transmembrane protein, putative
Sapur.003G102900.2	0.021528549	0.016234949	2.788036405	0.005823	0.995353884	NA	brown	-0.101980824	0.203756702	GO:0008152,GO:0003824	AT4G23850.1	LACS4	AMP-dependent synthetase and ligase family protein	long-chain fatty acyl CoA ligase, putative
Sapur.009G132900.3	0.021493086	0.016235006	2.788641984	0.005822	0.995354876	NA	brown	-0.068883388	0.391318889	GO:0032012,GO:0005086	AT4G38200.1		SEC7-like guanine nucleotide exchange family protein	brefeldin A-inhibited guanine nucleotide-exchange protein
Sapur.018G031100.1	0.019958643	0.016237586	2.816114656	0.005766	0.99539946	NA	brown	-0.101113393	0.207649557		AT4G32730.1	ATMYB3R-1,ATMYB3R1,MY	Homeodomain-like protein	Myb-like DNA-binding domain protein
Sapur.001G151200.16	0.019416904	0.016238535	2.826455518	0.005745	0.995416024	NA	brown	-0.086308382	0.282461582	GO:0035091	AT1G15240.2		Phox-associated domain;Phox-like;Sorting nexin, C-terminal	phox (PX) domain protein
Sapur.012G014400.1	0.018233371	0.016240702	2.85053586	0.005697	0.995454141	NA	brown	-0.112164538	0.161931676	GO:0005515	AT5G53430.1	ATX5,SDG29,SET29	SET domain group 29	histone-lysine N-methyltransferase ATX5
Sapur.002G029300.1	0.017193839	0.016242683	2.873173236	0.005653	0.995489406	NA	brown	-0.107347264	0.180838074		AT2G36070.1	ATTIM44-2,TIM44-2	translocase inner membrane subunit 44-2	import inner membrane translocase subunit Tim44
Sapur.014G055200.1	0.033007636	0.016245368	2.904823276	0.005593	0.995537814	NA	brown	-0.003342419	0.969860757	GO:0055114,GO:0004616,GO:0051287,GO:0016616,GO:0008152	AT2G45630.2		D-isomer specific 2-hydroxyacid dehydrogenase family protein	D-isomer specific 2-hydroxyacid dehydrogenase-like protein
Sapur.016G062200.8	0.027135728	0.016245367	2.904823276	0.005593	0.995537815	NA	brown	-0.031819303	0.692393557		AT5G06440.4			START-2 domain protein, putative
Sapur.019G016400.1	0.023086596	0.016245367	2.904823276	0.005593	0.995537815	NA	brown	-0.036434311	0.650532198	GO:0007165,GO:0005515,GO:0043531	AT5G36930.2		Disease resistance protein (TIR-NBS-LRR class) family	TIR-NBS-LRR disease resistance-like protein
Sapur.016G222600.3	0.022003026	0.016245367	2.904823276	0.005593	0.995537815	NA	brown	-0.04218652	0.599857698		AT3G28370.1			hypothetical protein
Sapur.006G211600.1	0.022852381	0.016245367	2.904823276	0.005593	0.995537815	NA	brown	-0.065378542	0.415922628	GO:0006468,GO:0004672	AT4G32250.1		Protein kinase superfamily protein	kinase, putative
Sapur.014G028100.1	0.022254485	0.016245367	2.904823276	0.005593	0.995537815	NA	brown	-0.071841173	0.371252336	GO:0055114,GO:0020037,GO:0016705,GO:0005506	AT4G31950.1	CYP82C3	cytochrome P450, family 82, subfamily C, polypeptide 3	NA
Sapur.007G024200.2	0.022920232	0.016245367	2.904823276	0.005593	0.995537815	NA	brown	-0.076477481	0.341098177	GO:0016021	AT2G25737.1		Sulfite exporter TauE/SafE family protein	sulfite exporter TauE/SafE
Sapur.006G108700.3	0.020504841	0.016245367	2.904823276	0.005593	0.995537815	NA	brown	-0.051947637	0.518190593	GO:0004842	AT5G02880.1	UPL4	ubiquitin-protein ligase 4	E3 ubiquitin-protein ligase
Sapur.006G033900.5	0.023000462	0.016245367	2.904823276	0.005593	0.995537815	NA	brown	-0.074437417	0.354170051		AT5G03960.1	IQD12	IQ-domain 12	IQ calmodulin-binding motif protein, putative
Sapur.012G028300.1	0.021868125	0.016245367	2.904823276	0.005593	0.995537815	NA	brown	-0.075128651	0.349706238	GO:0055114,GO:0020037,GO:0006979,GO:0004601	AT1G05260.1	RCI3,RCI3A	Peroxidase superfamily protein	cationic peroxidase kDa protein
Sapur.006G068200.2	0.01882198	0.016245367	2.904823276	0.005593	0.995537815	NA	brown	-0.070359747	0.381222537		AT3G09470.2		Major facilitator superfamily protein	UNC93-like protein
Sapur.010G036400.11	0.018855032	0.016245367	2.904823276	0.005593	0.995537815	NA	brown	-0.070476793	0.380428929		AT3G23090.1		TPX2 (targeting protein for Xklp2) protein family	targeting protein for Xklp2 protein
Sapur.002G039200.1	0.019560586	0.016245367	2.904823276	0.005593	0.995537815	NA	brown	-0.056743526	0.480261685		AT3G22600.1		Bifunctional inhibitor/lipid-transfer protein/seed storage 2S albumin superfamily protein	alpha-amylase inhibitors, lipid transfer (LT) and seed storage (SS) family protein
Sapur.003G002900.2	0.022072223	0.016245367	2.904823276	0.005593	0.995537815	NA	brown	-0.081608864	0.309590942		AT1G33980.1	ATUPF3,UPF3	Smg-4/UPF3 family protein	regulator of nonsense transcripts protein 3A, putative
Sapur.013G000600.2	0.019560586	0.016245367	2.904823276	0.005593	0.995537815	NA	brown	-0.056743526	0.480261685		AT1G57600.1		MBOAT (membrane bound O-acyl transferase) family protein	membrane bound O-acyl transferase family protein
Sapur.001G089800.2	0.021814812	0.016245367	2.904823276	0.005593	0.995537815	NA	brown	-0.086912476	0.27909376		AT1G12640.1		MBOAT (membrane bound O-acyl transferase) family protein	membrane bound O-acyl transferase (MBOAT) family protein
Sapur.016G248200.1	0.047926317	0.010546818	2.029503691	0.005197	0.995853614	NA	brown	-0.041495969	0.605847123	GO:0006260,GO:0005634	AT1G78650.1	POLD3	DNA-directed DNA polymerases	DNA polymerase subunit Cdc27
Sapur.016G201500.2	0.047365369	0.010548015	2.033810043	0.005186	0.995861924	NA	brown	-0.046044937	0.56689195	GO:0034314,GO:0007015,GO:0005885,GO:0005856,GO:0005524	AT3G27000.1	ARP2,ATARP2,WRM	actin related protein 2	actin-related protein
Sapur.007G059700.1	0.029870623	0.014392777	2.904822183	0.004955	0.996046668	NA	brown	-0.04266963	0.595683342		AT1G21280.1			NA
Sapur.011G014200.2	0.038002874	0.012509509	2.608540868	0.004796	0.996173682	NA	brown	-0.039973629	0.61914364		AT5G28237.1		Pyridoxal-5\'-phosphate-dependent enzyme family protein	tryptophan synthase beta chain
Sapur.018G106300.2	0.04696749	-0.008744966	1.837495363	-0.00476	0.99620274	NA	brown	-0.03332328	0.678641226	GO:0043565,GO:0006355,GO:0003700	AT4G31550.1	ATWRKY11,WRKY11	WRKY DNA-binding protein 11	WRKY transcription factor 11
Sapur.008G100900.2	0.018646603	0.010493359	2.8445909	0.003689	0.997056705	NA	brown	-0.121584748	0.129288935		AT3G18210.2		2-oxoglutarate (2OG) and Fe(II)-dependent oxygenase superfamily protein	oxidoreductase, putative
Sapur.15WG117700.1	0.017326071	0.010491418	2.872800249	0.003652	0.997086145	NA	brown	-0.120423261	0.133016025	GO:0016021	AT5G50790.1		Nodulin MtN3 family protein	bidirectional sugar transporter SWEET14, putative
Sapur.016G221200.1	0.032056311	-0.006521242	2.097700845	-0.00311	0.997519575	NA	brown	-0.087302003	0.276936552	GO:0055114,GO:0016491	AT5G14760.1	AO	L-aspartate oxidase	L-aspartate oxidase
Sapur.003G053700.2	0.03696623	-0.006514446	2.307478177	-0.00282	0.997747425	NA	brown	-0.080992451	0.313271786		AT1G32740.1		SBP (S-ribonuclease binding protein) family protein	SBP (S-ribonuclease-binding protein) family protein
Sapur.004G115700.3	0.028150259	-0.006512632	2.378746172	-0.00274	0.997815521	NA	brown	-0.087707043	0.274705394	GO:0007010,GO:0003779	AT4G34490.1	ATCAP1,CAP 1,CAP1	cyclase associated protein 1	adenylyl cyclase-associated protein
Sapur.013G053100.1	0.033867551	-0.006507626	2.637260826	-0.00247	0.998031166	NA	brown	-0.039595468	0.62246606	GO:0045454	AT3G03860.1	APRL5,ATAPRL5	APR-like 5	5'-adenylylsulfate reductase-like protein, putative
Sapur.008G066200.1	0.021444496	-0.004290386	2.789474412	-0.00154	0.998772804	NA	brown	-0.126706886	0.113803503	GO:0055085,GO:0016021,GO:0008324,GO:0006812	AT1G16310.1		Cation efflux family protein	cation diffusion facilitator
Sapur.002G086500.3	0.017258618	-0.004289463	2.904823918	-0.00148	0.998821789	NA	brown	-0.105015026	0.190554024	GO:0006094,GO:0005524,GO:0004612	AT4G37870.1	PCK1,PEPCK	phosphoenolpyruvate carboxykinase 1	phosphoenolpyruvate carboxykinase [ATP] protein
Sapur.005G195200.2	0.017320377	-0.004289463	2.904823918	-0.00148	0.998821789	NA	brown	-0.081608864	0.309590942	GO:0043565,GO:0006355,GO:0003700	AT4G35900.1	atbzip14,FD,FD-1	Basic-leucine zipper (bZIP) transcription factor family protein	basic leucine zipper transcription factor
Sapur.016G119500.8	0.016349173	-0.004289463	2.904823918	-0.00148	0.998821789	NA	brown	-0.075994714	0.344163569	GO:0007165	AT2G26980.4	CIPK3,SnRK3.17	CBL-interacting protein kinase 3	CBL-interacting Serine/Threonine-kinase
Sapur.016G110300.1	0	0	0	0	1	NA	brown	-0.081608864	0.309590942					transmembrane protein, putative
Sapur.T038900.1	0	0	0	0	1	NA	brown	-0.081608864	0.309590942					hypothetical protein
Sapur.002G023800.3	0	NA	NA	NA	NA	NA	brown	-0.206232582	0.009559862	GO:0016021	AT1G35180.1		TRAM, LAG1 and CLN8 (TLC) lipid-sensing domain containing protein	TRAM, LAG1 and CLN8 (TLC) lipid-sensing domain protein, putative
Sapur.005G078800.4	0	NA	NA	NA	NA	NA	brown	-0.028646119	0.721735977	GO:0055085,GO:0016021	AT2G17500.2		Auxin efflux carrier family protein	auxin efflux carrier family protein
Sapur.007G023900.2	0	NA	NA	NA	NA	NA	brown	-0.081608864	0.309590942	GO:0055114,GO:0020037,GO:0016705,GO:0005506	AT3G50660.1	CLM,CYP90B1,DWF4,PSC1,S	Cytochrome P450 superfamily protein	cytochrome P450 family protein
Sapur.008G131900.4	0	NA	NA	NA	NA	NA	brown	-0.044356672	0.581210261	GO:0016301,GO:0005524	AT1G06750.2		P-loop containing nucleoside triphosphate hydrolases superfamily protein	nucleoside/nucleotide kinase domain protein
Sapur.013G018000.2	0	NA	NA	NA	NA	NA	brown	-0.102850076	0.19990872	GO:0006412,GO:0005840,GO:0005622,GO:0003735	AT5G27700.1		Ribosomal protein S21e	40S ribosomal protein S21
Sapur.014G062200.2	0	NA	NA	NA	NA	NA	brown	-0.081608864	0.309590942	GO:0006813,GO:0055085,GO:0016021,GO:0015299,GO:0006812	AT4G00630.1	ATKEA2,KEA2	K+ efflux antiporter 2	glutathione-regulated potassium-efflux system protein KefB, putative
Sapur.014G084900.7	0	NA	NA	NA	NA	NA	brown	-0.081608864	0.309590942		AT1G01020.1	ARV1	Arv1-like protein	Arv1
Sapur.016G282200.1	0	NA	NA	NA	NA	NA	brown	-0.010354245	0.89759107	GO:0043531	AT4G27190.1		NB-ARC domain-containing disease resistance protein	NA
Sapur.017G040000.1	1724.676115	6.714739414	0.184945819	36.30652	1.28E-288	6.78E-287	cyan	0.650722899	2.85E-20	GO:0016758,GO:0008152	AT1G22360.1	AtUGT85A2,UGT85A2	UDP-glucosyl transferase 85A2	UDP-glucosyltransferase family protein
Sapur.009G102600.1	651.9949749	6.763251857	0.197683298	34.21256	1.57E-256	6.76E-255	cyan	0.60068239	9.09E-17	GO:0033926	AT4G34860.2		Plant neutral invertase family protein	neutral/alkaline invertase
Sapur.008G080000.1	543.0010543	4.618285596	0.140366283	32.90167	2.08E-237	7.93E-236	cyan	0.723415284	1.01E-26		AT3G06490.1	AtMYB108,BOS1,MYB108	myb domain protein 108	Myb-like transcription factor
Sapur.004G077300.1	78.53194967	6.24922685	0.211027112	29.61338	1.00E-192	2.84E-191	cyan	0.597293468	1.49E-16		AT2G24762.1	AtGDU4,GDU4	glutamine dumper 4	transmembrane protein, putative
Sapur.010G116200.1	529.6857433	5.744027808	0.195745043	29.34444	2.81E-189	7.77E-188	cyan	0.601283103	8.32E-17		AT3G06490.1	AtMYB108,BOS1,MYB108	myb domain protein 108	Myb transcription factor, putative
Sapur.006G019300.1	495.379775	8.53501147	0.294028144	29.02787	2.93E-185	7.75E-184	cyan	0.522683429	2.19E-12	GO:0046872,GO:0030001	AT3G56891.1		Heavy metal transport/detoxification superfamily protein	copper transporter atox1-like protein
Sapur.001G097000.1	344.0857183	4.460669294	0.156282601	28.54233	3.50E-179	8.73E-178	cyan	0.742433211	9.26E-29	GO:0055114,GO:0020037,GO:0016705,GO:0005506	AT4G12320.1	CYP706A6	cytochrome P450, family 706, subfamily A, polypeptide 6	cytochrome P450 family protein
Sapur.002G038200.1	1000.782142	3.814935494	0.153727253	24.81626	5.99E-136	1.03E-134	cyan	0.622806391	3.07E-18	GO:0016021,GO:0015116,GO:0008272,GO:0055085,GO:0016020,GO:0008271,GO:00095	AT3G51895.1	AST12,SULTR3;1	sulfate transporter 3;1	sulfate/bicarbonate/oxalate exchanger and transporter sat-1
Sapur.013G090200.1	238.4124963	6.456076949	0.260206752	24.81134	6.76E-136	1.16E-134	cyan	0.489557123	7.69E-11		AT2G30300.1		Major facilitator superfamily protein	nodulin-like protein
Sapur.003G116900.1	1031.015831	1.821144269	0.079935098	22.78279	6.79E-115	9.29E-114	cyan	0.751389671	8.77E-30		AT5G47860.1		Protein of unknown function (DUF1350)	plant/MCA23-20 protein
Sapur.007G129300.1	238.6258931	4.42419318	0.196043816	22.56737	9.07E-113	1.21E-111	cyan	0.545139689	1.56E-13		AT2G43890.1		Pectin lyase-like superfamily protein	polygalacturonase, putative
Sapur.007G025200.1	292.9218368	8.308022986	0.378565773	21.94605	9.45E-107	1.17E-105	cyan	0.474879325	3.30E-10		AT4G13260.1	YUC2	Flavin-binding monooxygenase family protein	flavine containing monooxygenase
Sapur.009G057000.1	245.2148673	2.562617198	0.119535613	21.43811	5.90E-102	6.89E-101	cyan	0.633373786	5.52E-19	GO:0006468,GO:0005524,GO:0004672	AT1G07150.1	MAPKKK13	mitogen-activated protein kinase kinase kinase 13	Serine/Threonine kinase family protein
Sapur.007G121100.1	124.6179163	4.198604984	0.199036786	21.09462	8.91E-99	1.00E-97	cyan	0.501571693	2.21E-11		AT1G17950.1	ATMYB52,BW52,MYB52	myb domain protein 52	R2R3-Myb transcription factor, putative
Sapur.012G029400.1	182.3217723	2.806285612	0.134308617	20.89431	6.03E-97	6.61E-96	cyan	0.588740909	5.09E-16		AT1G67920.1			hypothetical protein
Sapur.016G020000.1	382.6700395	4.864778415	0.238913349	20.3621	3.63E-92	3.75E-91	cyan	0.376221547	1.20E-06	GO:0016758,GO:0008152	AT1G22340.1	AtUGT85A7,UGT85A7	UDP-glucosyl transferase 85A7	UDP-glucosyltransferase family protein
Sapur.007G098000.1	53.24914484	2.45847067	0.121081115	20.30433	1.18E-91	1.21E-90	cyan	0.699961459	1.99E-24		AT4G33467.2			hypothetical protein
Sapur.012G084900.1	965.3292789	3.855716731	0.196057016	19.6663	4.19E-86	3.99E-85	cyan	0.592733979	2.88E-16	GO:0042545,GO:0030599,GO:0005618	AT1G69940.1	ATPPME1,PPME1	Pectin lyase-like superfamily protein	pectinesterase
Sapur.018G035700.1	1074.298938	1.613011588	0.083688689	19.27395	8.89E-83	8.05E-82	cyan	0.704600181	7.29E-25		AT5G11650.1		alpha/beta-Hydrolases superfamily protein	alpha/beta hydrolase family protein
Sapur.15WG137400.1	27.33511735	5.774491706	0.303333827	19.03675	8.46E-81	7.45E-80	cyan	0.517885461	3.76E-12	GO:0004857	AT4G25250.1		Plant invertase/pectin methylesterase inhibitor superfamily protein	pectinesterase/pectinesterase inhibitor, putative
Sapur.001G119300.1	36.46733466	3.750674717	0.198499863	18.8951	1.25E-79	1.09E-78	cyan	0.594739376	2.16E-16	GO:0005515	AT4G35470.1	PIRL4	plant intracellular ras group-related LRR 4	plant intracellular Ras-group-related LRR protein, putative
Sapur.004G045500.1	35.07505401	6.389927269	0.341750328	18.69765	5.17E-78	4.37E-77	cyan	0.486872231	1.01E-10	GO:0055114,GO:0016491	AT1G30040.1	ATGA2OX2,GA2OX2	gibberellin 2-oxidase	gibberellin 2-oxidase
Sapur.006G174200.1	13.44732586	4.503403953	0.245735855	18.3262	5.11E-75	4.13E-74	cyan	0.624496436	2.34E-18		AT3G10120.1			DUF4228 domain protein
Sapur.005G152200.1	33.0240132	8.200408039	0.448676918	18.27687	1.26E-74	1.02E-73	cyan	0.469967682	5.30E-10		AT1G20180.1		Protein of unknown function (DUF677)	transmembrane protein, putative
Sapur.15WG109400.1	773.6793519	2.506047584	0.137467216	18.23015	2.98E-74	2.38E-73	cyan	0.623854032	2.60E-18		AT5G50360.1			hypothetical protein
Sapur.011G045200.1	118.7266704	3.330817155	0.18500521	18.00391	1.82E-72	1.42E-71	cyan	0.537685281	3.84E-13		AT2G27430.1		ARM repeat superfamily protein	ubiquitin-protein ligase, putative
Sapur.15WG101400.1	532.5194942	1.532694448	0.091002092	16.84241	1.19E-63	8.14E-63	cyan	0.682891033	6.79E-23	GO:0005515	AT3G48240.1		Octicosapeptide/Phox/Bem1p family protein	PB1 domain protein
Sapur.006G013400.1	322.7451444	4.677382171	0.280564246	16.67134	2.12E-62	1.42E-61	cyan	0.500004216	2.61E-11		AT3G09950.1			hypothetical protein
Sapur.15WG109500.1	29.54479042	4.942148281	0.299350309	16.50958	3.13E-61	2.06E-60	cyan	0.402010865	1.80E-07	GO:0006097,GO:0004474	AT5G03860.1	MLS	malate synthase	malate synthase
Sapur.006G019200.1	96.72625994	2.313541963	0.142489844	16.23654	2.78E-59	1.78E-58	cyan	0.557514085	3.35E-14		AT2G41000.1		Chaperone DnaJ-domain superfamily protein	chaperone protein DnaJ
Sapur.014G102200.1	1258.172759	1.913773222	0.118160831	16.19634	5.35E-59	3.40E-58	cyan	0.693406001	7.94E-24	GO:0006950	AT4G02380.1	AtLEA5,SAG21	senescence-associated gene 21	NA
Sapur.006G128900.1	24.73410001	7.707956342	0.481228028	16.01726	9.68E-58	6.03E-57	cyan	0.328103844	2.73E-05		AT3G49070.1		Protein of unknown function (DUF677)	NA
Sapur.013G111300.1	51.44536909	2.190936662	0.138524453	15.81624	2.40E-56	1.46E-55	cyan	0.611004459	1.93E-17		AT2G20520.1	FLA6	FASCICLIN-like arabinogalactan 6	fasciclin-like arabinogalactan protein
Sapur.014G097400.1	280.2595013	2.155384528	0.137887975	15.63142	4.45E-55	2.65E-54	cyan	0.547088112	1.23E-13	GO:0008270	AT1G02610.1		RING/FYVE/PHD zinc finger superfamily protein	E3 ubiquitin-protein ligase MARCH6, putative
Sapur.002G182500.1	3173.232963	1.530401099	0.100813188	15.18056	4.76E-52	2.70E-51	cyan	0.624304846	2.42E-18	GO:0048046,GO:0016762,GO:0006073,GO:0005618,GO:0005975,GO:0004553	AT3G23730.1	XTH16	xyloglucan endotransglucosylase/hydrolase 16	xyloglucan endotransglycosylase/hydrolase
Sapur.005G090500.1	14.48797784	2.414457319	0.16160428	14.94055	1.79E-50	9.91E-50	cyan	0.568592875	7.97E-15		AT2G17880.1		Chaperone DnaJ-domain superfamily protein	chaperone protein DnaJ
Sapur.006G175300.1	123.1461731	1.421222066	0.099645864	14.26273	3.74E-46	1.92E-45	cyan	0.569751579	6.84E-15	GO:0006887,GO:0000145	AT3G09520.1	ATEXO70H4,EXO70H4	exocyst subunit exo70 family protein H4	exo70 exocyst complex subunit
Sapur.013G121600.1	327.2594224	3.709389976	0.267352178	13.87455	9.04E-44	4.45E-43	cyan	0.429114118	2.05E-08		AT5G13900.1		Bifunctional inhibitor/lipid-transfer protein/seed storage 2S albumin superfamily protein	NA
Sapur.006G019200.2	18.66503998	2.437276206	0.17624112	13.82921	1.70E-43	8.32E-43	cyan	0.546392152	1.34E-13		AT2G41000.2		Chaperone DnaJ-domain superfamily protein	chaperone protein DnaJ
Sapur.016G187300.1	483.097342	1.590921274	0.119418464	13.32224	1.72E-40	8.01E-40	cyan	0.569488759	7.08E-15	GO:0004866	AT1G17860.1		Kunitz family trypsin and protease inhibitor protein	kunitz-type trypsin inhibitor C4
Sapur.010G105500.1	10.16349618	4.001191097	0.306495141	13.05466	5.98E-39	2.70E-38	cyan	0.478200056	2.39E-10	GO:0043565,GO:0006355,GO:0003700	AT3G30530.1	ATBZIP42,bZIP42	basic leucine-zipper 42	BZIP transcription factor
Sapur.004G060400.1	14962.57187	1.299317743	0.100940591	12.8721	6.46E-38	2.86E-37	cyan	0.618818682	5.76E-18	GO:0006807,GO:0006542,GO:0004356	AT5G37600.1	ATGLN1;1,ATGSR1,GLN1;1,(	glutamine synthase clone R1	glutamine synthetase
Sapur.003G113300.1	6.135142928	4.864159445	0.383896158	12.67051	8.62E-37	3.74E-36	cyan	0.484882312	1.23E-10					hypothetical protein
Sapur.011G091000.1	564.4713269	2.618170851	0.208679244	12.54639	4.16E-36	1.78E-35	cyan	0.46647946	7.39E-10		AT1G61260.1		Protein of unknown function (DUF761)	cotton fiber
Sapur.016G123100.1	65.73249079	3.8419523	0.307085001	12.51104	6.50E-36	2.77E-35	cyan	0.391026524	4.12E-07	GO:0048544,GO:0006468,GO:0005524,GO:0004672	AT5G60900.1	RLK1	receptor-like protein kinase 1	NA
Sapur.011G041300.1	75.47463284	1.786311813	0.145280486	12.29561	9.56E-35	3.97E-34	cyan	0.572396295	4.81E-15	GO:0046983,GO:0008171	AT4G35160.1		O-methyltransferase family protein	orcinol O-methyltransferase-like protein
Sapur.008G102900.1	398.2588026	2.52630391	0.207890852	12.15207	5.59E-34	2.29E-33	cyan	0.500020736	2.60E-11		AT2G01300.1			by genscan and genefinder, putative
Sapur.011G102200.1	382.9002019	1.567247506	0.1300147	12.05439	1.84E-33	7.46E-33	cyan	0.597054298	1.55E-16	GO:0008270	AT3G28210.1	PMZ,SAP12	zinc finger (AN1-like) family protein	stress-associated protein
Sapur.013G081400.1	7.103326046	3.280195275	0.290468016	11.29279	1.42E-29	5.31E-29	cyan	0.421988019	3.70E-08					hypothetical protein
Sapur.15WG089600.1	42.47050231	1.516849763	0.135689193	11.17885	5.18E-29	1.91E-28	cyan	0.513704983	5.97E-12		AT3G48180.1			hypothetical protein
Sapur.003G113600.1	6.132931216	2.810576604	0.252554962	11.12857	9.11E-29	3.34E-28	cyan	0.53453127	5.57E-13					hypothetical protein
Sapur.016G039100.1	79.0754465	1.854097715	0.168899639	10.97751	4.90E-28	1.77E-27	cyan	0.509559827	9.39E-12					NA
Sapur.007G049200.1	3.25169922	3.053211504	0.279279298	10.93247	8.06E-28	2.90E-27	cyan	0.563958432	1.46E-14		AT5G43120.1		ARM-repeat/Tetratricopeptide repeat (TPR)-like protein	heat shock protein 70 (HSP70)-interacting protein, putative
Sapur.009G034400.1	3.901308899	5.078096043	0.468892899	10.82997	2.48E-27	8.82E-27	cyan	0.397621214	2.51E-07	GO:0050662,GO:0003824	AT5G58750.1		NAD(P)-binding Rossmann-fold superfamily protein	progesterone 5-beta-reductase, putative
Sapur.005G030900.1	12.35368912	1.85825802	0.17347338	10.71206	8.93E-27	3.13E-26	cyan	0.568232491	8.36E-15		AT1G54740.1		Protein of unknown function (DUF3049)	DUF3049 family protein
Sapur.010G062200.1	265.9085324	0.631839982	0.059217948	10.66974	1.41E-26	4.92E-26	cyan	0.553019828	5.90E-14		AT3G22530.1			hypothetical protein
Sapur.007G000900.1	1160.697944	0.75392675	0.071049696	10.61126	2.64E-26	9.14E-26	cyan	0.574521582	3.62E-15		AT3G19990.1			TPR1
Sapur.011G015300.1	5.300207196	2.706446564	0.266945259	10.13858	3.72E-24	1.23E-23	cyan	0.511920861	7.26E-12		AT2G46410.1	CPC	Homeodomain-like superfamily protein	MYB transcription factor
Sapur.003G054900.1	213.699152	2.081491029	0.206875313	10.06157	8.17E-24	2.66E-23	cyan	0.326294672	3.04E-05		AT4G17030.1	AT-EXPR,ATEXLB1,ATEXPR1	expansin-like B1	major pollen allergen Ory S protein, putative
Sapur.012G091400.1	92.24894955	1.814397072	0.18118672	10.01396	1.32E-23	4.29E-23	cyan	0.426105103	2.64E-08	GO:0043531	AT3G14470.1		NB-ARC domain-containing disease resistance protein	CC-NBS-LRR resistance protein
Sapur.012G001400.1	62.58739607	1.140788293	0.115699545	9.85992	6.21E-23	1.98E-22	cyan	0.506107839	1.36E-11	GO:0005515	AT1G45616.1	AtRLP6,RLP6	receptor like protein 6	receptor-like kinase, putative
Sapur.009G009000.1	170.1758929	1.413823864	0.144357819	9.793885	1.20E-22	3.79E-22	cyan	0.482830402	1.51E-10		AT5G60520.1		Late embryogenesis abundant (LEA) protein-related	structural constituent of cell wall protein, putative
Sapur.016G118600.1	74.47696174	2.295792062	0.234768388	9.778966	1.39E-22	4.39E-22	cyan	0.446689255	4.52E-09		AT1G20030.2		Pathogenesis-related thaumatin superfamily protein	allergen Pru protein, putative
Sapur.014G080500.1	155.192993	1.805709519	0.185061703	9.757338	1.72E-22	5.42E-22	cyan	0.459974903	1.36E-09	GO:0005524	AT2G46620.1		P-loop containing nucleoside triphosphate hydrolases superfamily protein	P-loop nucleoside triphosphate hydrolase-like protein
Sapur.008G172700.1	171.0595597	1.520201902	0.15688895	9.689668	3.34E-22	1.05E-21	cyan	0.506825743	1.26E-11		AT5G18860.1		inosine-uridine preferring nucleoside hydrolase family protein	inosine-uridine preferring nucleoside hydrolase, putative
Sapur.019G089200.1	418.1431646	1.212318414	0.127331637	9.520952	1.72E-21	5.29E-21	cyan	0.467648233	6.61E-10		AT2G20520.1	FLA6	FASCICLIN-like arabinogalactan 6	fasciclin-like arabinogalactan protein
Sapur.003G086400.1	196.5859738	0.597441596	0.063191768	9.454421	3.25E-21	9.94E-21	cyan	0.565716163	1.16E-14		AT1G12710.1	AtPP2-A12,PP2-A12	phloem protein 2-A12	F-box protein PP2-A13
Sapur.010G051700.1	7.844320269	2.122077394	0.2256074	9.406063	5.15E-21	1.57E-20	cyan	0.48100323	1.81E-10					NA
Sapur.004G025300.1	1232.698955	0.552395806	0.059094397	9.347685	8.96E-21	2.71E-20	cyan	0.556913494	3.61E-14	GO:0055114,GO:0004097				polyphenol oxidase
Sapur.005G146900.2	225.9108466	1.119361354	0.119944803	9.332304	1.04E-20	3.13E-20	cyan	0.470845508	4.88E-10		AT1G21460.1		Nodulin MtN3 family protein	bidirectional sugar transporter SWEET1
Sapur.005G145600.1	64.53270697	1.819603132	0.19514844	9.3242	1.12E-20	3.38E-20	cyan	0.493823299	4.96E-11	GO:0016624,GO:0008152	AT1G21400.1		Thiamin diphosphate-binding fold (THDP-binding) superfamily protein	2-oxoisovalerate dehydrogenase subunit alpha 1
Sapur.008G104000.1	1977.021579	0.496698493	0.053584692	9.26941	1.87E-20	5.61E-20	cyan	0.539263572	3.18E-13		AT1G70990.1		proline-rich family protein	transmembrane protein, putative
Sapur.014G140800.1	2.121302291	3.206454533	0.348493764	9.200895	3.55E-20	1.06E-19	cyan	0.447848594	4.07E-09					NA
Sapur.T091700.1	81.69589235	0.689748329	0.076342891	9.034873	1.64E-19	4.80E-19	cyan	0.539070303	3.25E-13		AT5G40470.1		RNI-like superfamily protein	F-box/LRR protein, putative
Sapur.006G195900.1	1389.775853	0.603512188	0.067994155	8.875942	6.93E-19	1.99E-18	cyan	0.530932966	8.49E-13		AT5G11650.1		alpha/beta-Hydrolases superfamily protein	alpha/beta hydrolase family protein
Sapur.009G109700.2	1.317093267	2.560214981	0.290842305	8.80276	1.33E-18	3.81E-18	cyan	0.494747078	4.51E-11	GO:0003824,GO:0006470,GO:0004722	AT4G38520.1		Protein phosphatase 2C family protein	protein phosphatase 2C
Sapur.003G084300.1	2730.989518	0.736229382	0.084715176	8.690643	3.60E-18	1.02E-17	cyan	0.525527072	1.58E-12		AT4G22920.1	ATNYE1,NYE1	non-yellowing 1	senescence-inducible stay-green protein
Sapur.004G138200.1	35.95744564	1.101229815	0.126715751	8.690552	3.61E-18	1.02E-17	cyan	0.418359052	4.97E-08		AT1G08290.1	WIP3	WIP domain protein 3	transparent TESTA 1 protein, putative
Sapur.018G078600.1	3.067128702	2.45239503	0.28460867	8.616726	6.89E-18	1.93E-17	cyan	0.445621433	4.96E-09		AT3G26390.1			NA
Sapur.016G101800.1	117.2284298	1.475860288	0.171330625	8.614107	7.05E-18	1.98E-17	cyan	0.386978841	5.54E-07					NA
Sapur.018G102200.1	396.574819	1.490027256	0.174117388	8.557602	1.15E-17	3.21E-17	cyan	0.419563977	4.51E-08	GO:0005515	AT2G25490.1	EBF1,FBL6	EIN3-binding F box protein 1	Ein3-binding F-box protein
Sapur.15WG149100.1	209.5955834	0.660273125	0.077425839	8.527814	1.49E-17	4.14E-17	cyan	0.463465629	9.80E-10	GO:0005515	AT5G23730.1		Transducin/WD40 repeat-like superfamily protein	ubiquitin ligase COP1, putative
Sapur.014G121000.1	701.3493256	0.508590264	0.06002641	8.472775	2.40E-17	6.61E-17	cyan	0.568103872	8.50E-15	GO:0046983	AT3G07340.1		basic helix-loop-helix (bHLH) DNA-binding superfamily protein	transcription factor, putative
Sapur.016G101600.1	120.065711	1.455952278	0.172384387	8.445964	3.02E-17	8.29E-17	cyan	0.379375311	9.58E-07					NA
Sapur.005G146200.1	56.51738527	1.132488946	0.135358053	8.366617	5.93E-17	1.62E-16	cyan	0.460713476	1.27E-09		AT5G44860.1			NA
Sapur.003G086500.1	231.2888959	0.907684632	0.110955968	8.180584	2.82E-16	7.54E-16	cyan	0.443055276	6.22E-09	GO:0004857	AT1G47960.1	ATC/VIF1,C/VIF1	cell wall / vacuolar inhibitor of fructosidase 1	plant invertase/pectin methylesterase inhibitor protein
Sapur.005G054300.1	70.40394058	0.72421195	0.088558375	8.177792	2.89E-16	7.72E-16	cyan	0.537107321	4.11E-13	GO:0008270,GO:0005515	AT5G10380.1	ATRING1,RING1	RING/U-box superfamily protein	RING-H2 zinc finger protein
Sapur.012G074700.1	19.49207886	0.922654503	0.113391179	8.136916	4.05E-16	1.08E-15	cyan	0.425544852	2.76E-08					NA
Sapur.018G037000.1	182.8835515	0.792357545	0.097689938	8.110943	5.02E-16	1.33E-15	cyan	0.433722461	1.39E-08		AT5G21940.1			hypothetical protein
Sapur.013G023400.1	21.05935509	2.701032072	0.333078448	8.109297	5.09E-16	1.35E-15	cyan	0.342252001	1.15E-05	GO:0016757	AT1G08990.1	PGSIP5	plant glycogenin-like starch initiation protein 5	NA
Sapur.004G126900.1	634.1105453	0.621081057	0.076714327	8.096024	5.68E-16	1.50E-15	cyan	0.497010426	3.57E-11		AT2G21180.1			transmembrane protein, putative

Sapur.018G046700.1	1.96253913	2.808154876	0.348629079	8.05485	7.96E-16	2.10E-15	cyan	0.374540376	1.35E-06					NA
Sapur.008G085800.1	2.886022336	2.934373274	0.365595763	8.026278	1.00E-15	2.64E-15	cyan	0.360889086	3.43E-06					NA
Sapur.003G031400.1	381.1778689	0.789999309	0.099165018	7.966512	1.63E-15	4.26E-15	cyan	0.478104266	2.41E-10	GO:0055114,GO:0016491	AT1G15550.1	ATGA3OX1,GA3OX1,GA4	gibberellin 3-oxidase 1	gibberellin 3-beta-hydroxylase
Sapur.012G094600.1	23.71390233	0.966453087	0.122469283	7.891392	2.99E-15	7.75E-15	cyan	0.471509218	4.58E-10	GO:0051603,GO:0005839,GO:0004298	AT1G79210.2		N-terminal nucleophile aminohydrolases (Ntn hydrolases) superfamily protein	NA
Sapur.014G029900.1	3.377261389	2.648052143	0.33954666	7.798787	6.25E-15	1.60E-14	cyan	0.306404467	9.51E-05	GO:0005515	AT1G13570.1		F-box/RNI-like superfamily protein	F-box/FBD/LRR protein
Sapur.001G168200.1	32.10104387	1.388049961	0.181434314	7.650427	2.00E-14	5.07E-14	cyan	0.372780966	1.52E-06		AT1G17620.1		Late embryogenesis abundant (LEA) hydroxyproline-rich glycoprotein family	late embryogenesis abundant protein
Sapur.016G163400.1	231.6807463	1.122298847	0.147477258	7.609979	2.74E-14	6.90E-14	cyan	0.465301591	8.25E-10	GO:0006468,GO:0005524,GO:0004672	AT1G07150.1	MAPKKK13	mitogen-activated protein kinase kinase kinase 13	Serine/Threonine kinase domain protein
Sapur.013G068000.1	454.8913847	1.127482182	0.151599327	7.437251	1.03E-13	2.54E-13	cyan	0.367734354	2.16E-06	GO:0055085,GO:0016021	AT5G17860.1	CAX7	calcium exchanger 7	cation/calcium exchanger
Sapur.017G019300.1	312.895579	0.743395565	0.101405996	7.330884	2.29E-13	5.59E-13	cyan	0.399170984	2.24E-07		AT1G05910.1		cell division cycle protein 48-related / CDC48-related	NA
Sapur.008G125500.1	24.57679956	1.08636745	0.149556761	7.263914	3.76E-13	9.12E-13	cyan	0.353992515	5.41E-06		AT3G22600.1		Bifunctional inhibitor/lipid-transfer protein/seed storage 2S albumin superfamily protein	lipid transfer/seed storage/trypsin-alpha amylase inhibitor, putative
Sapur.016G257300.1	205.5943175	0.978652971	0.134905698	7.254349	4.04E-13	9.77E-13	cyan	0.341581018	1.20E-05		AT1G28280.2		VQ motif-containing protein	DNA-binding WRKY; VQ protein, putative
Sapur.016G008000.1	5.849569609	2.191971924	0.303660076	7.218505	5.26E-13	1.27E-12	cyan	0.396210875	2.80E-07		AT4G17240.1			NA
Sapur.003G082800.1	160.6654901	0.62689865	0.086977648	7.207583	5.70E-13	1.37E-12	cyan	0.440763832	7.60E-09	GO:0055114,GO:0020037,GO:0016705,GO:0005506	AT2G45550.1	CYP76C4	cytochrome P450, family 76, subfamily C, polypeptide 4	cytochrome P450 family protein
Sapur.003G060200.1	1.968380469	2.137418043	0.302323048	7.06998	1.55E-12	3.68E-12	cyan	0.428656234	2.13E-08					transmembrane protein, putative
Sapur.005G157600.1	9.119436245	1.820213847	0.258287447	7.047241	1.83E-12	4.33E-12	cyan	0.371465837	1.67E-06	GO:0055114,GO:0016491,GO:0005507	AT5G09360.1	LAC14	laccase 14	NA
Sapur.T131100.1	16.38750164	2.179641475	0.312918919	6.965515	3.27E-12	7.69E-12	cyan	0.379466812	9.52E-07		AT4G17240.1			hypothetical protein
Sapur.007G104900.1	5711.132644	0.916653215	0.131729487	6.958603	3.44E-12	8.07E-12	cyan	0.474755901	3.34E-10	GO:0016829,GO:0010333,GO:0008152,GO:0000287	AT4G16740.1	ATTPS03,TPS03	terpene synthase 03	(E)-beta-ocimene synthase
Sapur.007G013600.1	1.593243619	1.945615603	0.283777691	6.856126	7.08E-12	1.64E-11	cyan	0.467551435	6.67E-10		AT2G16460.1		Protein of unknown function (DUF1640)	coiled-coil protein 90B
Sapur.014G006000.1	133.9250692	0.558591297	0.082851789	6.742055	1.56E-11	3.57E-11	cyan	0.393846137	3.34E-07		AT1G58420.1		Uncharacterised conserved protein UCP031279	hypothetical protein
Sapur.001G191100.1	2079.039697	0.52900511	0.079375283	6.664608	2.65E-11	6.03E-11	cyan	0.40937139	1.02E-07	GO:0003993	AT4G25150.1		HAD superfamily, subfamily IIIB acid phosphatase	acid phosphatase
Sapur.013G053300.1	852.0182413	0.654057332	0.099778062	6.555122	5.56E-11	1.25E-10	cyan	0.382307404	7.77E-07		AT3G13520.1	AGP12,ATAGP12	arabinogalactan protein 12	transmembrane protein, putative
Sapur.007G013700.1	713.0921928	0.871109002	0.138728493	6.279236	3.40E-10	7.40E-10	cyan	0.437661383	9.94E-09	GO:0055114,GO:0020037,GO:0016705,GO:0005506	AT4G36220.1	CYP84A1,FAH1	ferulic acid 5-hydroxylase 1	coniferyl aldehyde 5-hydroxylase
Sapur.018G007000.1	83.53945249	0.375725383	0.063562174	5.911147	3.40E-09	7.10E-09	cyan	0.434816026	1.27E-08	GO:0006887,GO:0000145	AT4G31540.1	ATEXO70G1,EXO70G1	exocyst subunit exo70 family protein G1	exocyst complex component
Sapur.016G056300.1	4.9693625	1.038248228	0.184425458	5.629636	1.81E-08	3.66E-08	cyan	0.3810109	8.53E-07	GO:0007275,GO:0006511,GO:0005634	AT3G58040.1	SINAT2	seven in absentia of Arabidopsis 2	E3 ubiquitin-protein ligase SINAT2
Sapur.007G028100.1	2703.572335	-0.17273224	0.031065258	-5.5603	2.69E-08	5.42E-08	cyan	-0.464025578	9.30E-10	GO:0018279,GO:0005789	AT5G66680.1	DGL1	dolichyl-diphosphooligosaccharide-protein glycosyltransferase 48kDa subunit family protein	dolichyl-diphosphooligosaccharide-protein glycosyltransferase, putative
Sapur.006G053900.1	77.93437069	0.752750466	0.142120761	5.296555	1.18E-07	2.31E-07	cyan	0.289808696	0.000231771	GO:0006355,GO:0005634	AT1G04550.2	BDL,IAA12	AUX/IAA transcriptional regulator family protein	IAA5, putative
Sapur.15WG137200.1	3640.854754	0.80522314	0.153495501	5.245907	1.56E-07	3.03E-07	cyan	0.329730112	2.48E-05	GO:0004857	AT5G51520.1		Plant invertase/pectin methylesterase inhibitor superfamily protein	NA
Sapur.018G006900.1	645.0428586	0.342595926	0.066722218	5.13466	2.83E-07	5.45E-07	cyan	0.345859364	9.14E-06		AT5G24890.1			hypothetical protein
Sapur.008G125500.2	3.295409298	1.011767896	0.199897691	5.061429	4.16E-07	7.94E-07	cyan	0.35036155	6.85E-06		AT3G22600.1		Bifunctional inhibitor/lipid-transfer protein/seed storage 2S albumin superfamily protein	lipid transfer/seed storage/trypsin-alpha amylase inhibitor, putative
Sapur.15WG136800.1	1.104829271	2.123699498	0.422088627	5.031407	4.87E-07	9.26E-07	cyan	0.245806217	0.001915159	GO:0005975,GO:0004553	AT1G70710.1	ATGH9B1,CEL1,GH9B1	glycosyl hydrolase 9B1	endo-1%2C4-beta-glucanase
Sapur.006G083000.1	810.1428444	0.23424624	0.046937598	4.990589	6.02E-07	1.14E-06	cyan	0.389474559	4.62E-07	GO:0006468,GO:0004672	AT3G51850.1	CPK13	calcium-dependent protein kinase 13	calcium-dependent kinase
Sapur.013G064700.1	227.6438073	0.498460031	0.104060788	4.790085	1.67E-06	3.09E-06	cyan	0.328232873	2.71E-05		AT4G17900.1		PLATZ transcription factor family protein	PLATZ transcription factor
Sapur.008G135400.1	25.50356349	0.623422967	0.142222372	4.383438	1.17E-05	2.07E-05	cyan	0.287949106	0.00025526		AT4G14380.1			DUF761 domain protein
Sapur.002G103400.1	24.77376533	0.490789742	0.112210156	4.373844	1.22E-05	2.16E-05	cyan	0.310600506	7.52E-05		AT5G42750.1	BKI1	BRI1 kinase inhibitor 1	hypothetical protein
Sapur.017G010000.1	190.6288951	0.577859334	0.13489571	4.283749	1.84E-05	3.22E-05	cyan	0.275081834	0.000488953	GO:0045454,GO:0015035,GO:0009055	AT1G28480.1	GRX480,roxy19	Thioredoxin superfamily protein	glutaredoxin family protein
Sapur.016G201200.1	435.2881369	0.596445432	0.144082675	4.139605	3.48E-05	6.01E-05	cyan	0.277924438	0.000424687	GO:0016747	AT5G41040.1		HXXXD-type acyl-transferase family protein	hydroxycinnamoyl-CoA shikimate/quinate hydroxycinnamoyl transferase
Sapur.010G141700.1	383.2178198	0.763235144	0.184498543	4.136809	3.52E-05	6.08E-05	cyan	0.206388836	0.009504255		AT3G10985.1	ATWI-12,SAG20,WI12	senescence associated gene 20	wound-induced protein
Sapur.008G082100.1	653.2605622	0.225388816	0.059022377	3.818701	0.000134156	0.000223846	cyan	0.262735689	0.000886208		AT3G17100.1		sequence-specific DNA binding transcription factors	transcription factor bHLH148, putative
Sapur.005G146300.1	40.03569099	0.667528133	0.177516152	3.76038	0.000169656	0.000281206	cyan	0.266891874	0.000727694		AT1G17950.1	ATMYB52,BW52,MYB52	myb domain protein 52	R2R3-MYB transcription factor, putative
Sapur.010G147800.1	326.3019749	0.469018519	0.127489959	3.678866	0.000234273	0.000384438	cyan	0.251445249	0.001490024	GO:0006468,GO:0005524,GO:0004672	AT3G55950.1	ATCRR3,CCR3	CRINKLY4 related 3	Serine/Threonine kinase-like protein
Sapur.008G138500.1	59.65257085	0.668446169	0.185527637	3.602947	0.00031463	0.000511758	cyan	0.261295221	0.000948157		AT3G23530.1		Cyclopropane-fatty-acyl-phospholipid synthase	cyclopropane-fatty-acyl-phospholipid synthase
Sapur.001G094400.1	52.54561324	0.429558111	0.136805923	3.139909	0.001690005	0.00261645	cyan	0.227400845	0.00418094		AT4G22810.1		Predicted AT-hook DNA-binding family protein	AT-hook motif nuclear localized protein
Sapur.016G101500.1	16.78800539	0.487767879	0.155642791	3.133893	0.001725036	0.002668812	cyan	0.169884016	0.033410331					NA
Sapur.002G193100.1	802.6789954	0.177442182	0.064448968	2.75322	0.005901224	0.008744494	cyan	0.204688781	0.010124892	GO:0016567,GO:0004842	AT3G07360.1	ATPUB9,PUB9	plant U-box 9	spotted leaf protein, putative
Sapur.003G115700.1	336.4053881	0.154478896	0.078419281	1.96991	0.04884873	0.066100275	cyan	0.153954429	0.05421415		AT5G44670.1		Domain of unknown function (DUF23)	glycosyltransferase family 92 protein
Sapur.014G075300.1	574.6387238	0.12265956	0.078184239	1.568853	0.11668225	0.150565672	cyan	0.118547482	0.139208386		AT4G01037.1	AtWTF1,WTF1	Ubiquitin carboxyl-terminal hydrolase family protein	plant organelle RNA recognition domain protein
Sapur.016G125700.1	65.0121775	0.190752808	0.154300155	1.236245	0.216367478	0.268371947	cyan	0.078459349	0.32869616		AT2G31930.1			hypothetical protein
Sapur.002G009700.6	0.004272546	0.128661719	2.904823276	0.044292	0.964671295	NA	cyan	0.424001365	3.14E-08	GO:0043169,GO:0005975,GO:0003824	AT1G76130.1	AMY2,ATAMY2	alpha-amylase-like 2	alpha-amylase
Sapur.001G000300.1	10773.68226	9.059815264	0.234581051	38.62126	0	0	cyan	0.769931034	4.77E-32	GO:0055114,GO:0016491,GO:0005507	AT1G55570.1	sks12	SKU5 similar 12	L-ascorbate oxidase-like protein
Sapur.001G030200.1	5855.287879	9.691498673	0.25714514	37.68883	0	0	cyan	0.734315369	7.22E-28		AT3G12660.1	FLA14	FASCICLIN-like arabinogalactan protein 14 precursor	fasciclin-like arabinogalactan protein
Sapur.001G042100.1	474.9743915	8.087384333	0.15312367	52.81603	0	0	cyan	0.815793015	1.08E-38	GO:0005515,GO:0006468,GO:0004672	AT3G24715.1		Protein kinase superfamily protein with octicosapeptide/Phox/Bem1p domain	tyrosine kinase family protein
Sapur.001G059400.1	874.0380629	5.699357394	0.117713686	48.41712	0	0	cyan	0.780697057	1.83E-33		AT2G34920.1	EDA18	RING/U-box superfamily protein	ubiquitin-protein ligase, putative
Sapur.001G073800.1	3206.717814	5.18816364	0.129164005	40.16726	0	0	cyan	0.723814582	9.23E-27	GO:0005515,GO:0016773	AT2G46500.1	ATPI4K GAMMA 4.PI4K GAN	phosphoinositide 4-kinase gamma 4	phosphatidylinositol 3- and 4-kinase family protein
Sapur.001G116600.1	277.70899	7.308791289	0.182736089	39.99643	0	0	cyan	0.75631197	2.30E-30	GO:0008270,GO:0005515	AT4G17920.1		RING/U-box superfamily protein	RING-H2 finger protein ATL28
Sapur.002G003900.1	422.2778444	8.5172619	0.173115146	49.19998	0	0	cyan	0.78273697	9.69E-34		AT2G33690.1		Late embryogenesis abundant protein, group 6	late embryogenesis abundant protein
Sapur.002G004400.1	410.1227825	7.514465009	0.168829533	44.50919	0	0	cyan	0.779724007	2.48E-33	GO:0016021,GO:0006952	AT2G33670.1	ATMLO5,MLO5	Seven transmembrane MLO family protein	MLO-like protein
Sapur.002G083900.1	2413.073924	7.865907545	0.195808928	40.17134	0	0	cyan	0.756274638	2.32E-30		AT1G58120.1			NA
Sapur.002G088800.1	4717.817401	5.513026618	0.123974076	44.46919	0	0	cyan	0.730001446	2.09E-27		AT1G09890.1		Rhamnogalacturonate lyase family protein	rhamnogalacturonate lyase
Sapur.002G165500.1	276.3111632	9.614018367	0.230667954	41.67904	0	0	cyan	0.828979741	5.87E-41		AT1G02380.1			transmembrane protein, putative
Sapur.003G001800.1	356.0745781	7.119010221	0.162917996	43.69689	0	0	cyan	0.773361798	1.72E-32	GO:0006468,GO:0005524,GO:0004672	AT1G09600.1		Protein kinase superfamily protein	cyclin-dependent kinase-like Serine/Threonine kinase family protein
Sapur.003G008400.1	373.7470254	8.703218943	0.213556674	40.75367	0	0	cyan	0.77636046	6.96E-33	GO:0003824,GO:0005515	AT4G35790.2	ATPLDDELTA,PLDDELTA	phospholipase D delta	NA
Sapur.003G023100.1	202.0772315	9.810071525	0.251186048	39.055	0	0	cyan	0.846088389	3.35E-44		AT3G13850.1	LBD22	LOB domain-containing protein 22	NA
Sapur.003G025600.1	815.562709	5.679860989	0.109625933	51.81129	0	0	cyan	0.812222384	4.11E-38	GO:0005089	AT1G79860.1	ATROPGEF12,MEF64,ROPG	RHO guanyl-nucleotide exchange factor 12	Rop guanine nucleotide exchange factor
Sapur.003G045800.1	280.292572	7.486249801	0.153748568	48.69151	0	0	cyan	0.814704793	1.62E-38	GO:0016021,GO:0016020,GO:0022857	AT5G47470.1		Nodulin MtN21 /EamA-like transporter family protein	auxin-induced protein 5NG4
Sapur.003G067400.1	175.1231007	6.886801877	0.172752315	39.86518	0	0	cyan	0.735065759	5.99E-28		AT5G14890.1		NHL domain-containing protein	potassium transporter, putative
Sapur.003G089300.1	1647.433612	9.68392378	0.23422729	41.34413	0	0	cyan	0.742408473	9.32E-29	GO:0008270	AT3G61230.1		GATA type zinc finger transcription factor family protein	LIM2-SF3 domain protein
Sapur.003G090200.1	728.5019273	7.405755388	0.142887688	51.82921	0	0	cyan	0.793714198	2.77E-35	GO:0005622,GO:0003779,GO:0030042,GO:0015629	AT4G00680.1	ADF8	actin depolymerizing factor 8	actin depolymerizing factor
Sapur.003G100400.1	534.7874859	7.152804172	0.182595675	39.17291	0	0	cyan	0.686840215	3.06E-23	GO:0006468,GO:0004672	AT1G64300.1		Protein kinase family protein	NA
Sapur.003G115500.1	567.9485651	5.309402106	0.141488207	37.5254	0	0	cyan	0.710951753	1.79E-25		AT2G34920.1	EDA18	RING/U-box superfamily protein	ubiquitin-protein ligase, putative
Sapur.003G119000.1	2299.661594	9.759332897	0.185008406	52.75075	0	0	cyan	0.814594911	1.69E-38		AT1G63060.1			hypothetical protein
Sapur.003G141700.1	431.7860863	6.236194687	0.128338993	48.59158	0	0	cyan	0.792901675	3.63E-35		AT1G79640.1		Protein kinase superfamily protein	NA
Sapur.003G170000.1	17324.49723	8.965643112	0.23660347	37.89312	0	0	cyan	0.759539848	9.39E-31	GO:0055114,GO:0016491,GO:0005507	AT1G55570.1	sks12	SKU5 similar 12	L-ascorbate oxidase-like protein
Sapur.004G029800.1	2134.145807	8.949010578	0.226112973	39.57761	0	0	cyan	0.809701974	1.04E-37		AT1G28270.1	RALFL4	ralf-like 4	rapid alkalinization factor
Sapur.004G029900.1	1981.491862	7.965787944	0.182346709	43.68485	0	0	cyan	0.788417858	1.58E-34		AT1G28270.1	RALFL4	ralf-like 4	rapid alkalinization factor
Sapur.004G040200.1	1186.986086	5.964894706	0.133080686	44.82164	0	0	cyan	0.760244437	7.71E-31	GO:0006468,GO:0004672	AT4G18640.1	MRH1	Leucine-rich repeat protein kinase family protein	LRR receptor-like Serine/Threonine-kinase, putative
Sapur.004G119400.1	952.3278474	7.229951781	0.19006993	38.03838	0	0	cyan	0.662448456	3.43E-21	GO:0006468,GO:0004672	AT4G39110.1		Malectin/receptor-like protein kinase family protein	receptor-like kinase, putative
Sapur.004G131000.1	11387.48882	6.760677858	0.138533123	48.80189	0	0	cyan	0.740453394	1.54E-28	GO:0030246,GO:0005975,GO:0004553	AT2G16730.1	BGAL13	glycosyl hydrolase family 35 protein	beta-galactosidase
Sapur.004G143700.1	1388.727107	7.457569669	0.159577691	46.73316	0	0	cyan	0.774152976	1.36E-32		AT2G27180.1			hypothetical protein
Sapur.004G175900.1	290.111545	4.155945418	0.105989688	39.21085	0	0	cyan	0.808553695	1.58E-37	GO:0016787	AT2G39840.1	TOPP4	type one serine/threonine protein phosphatase 4	serine/threonine-protein phosphatase
Sapur.005G014000.1	526.0449069	6.328468051	0.115487082	54.79806	0	0	cyan	0.806650032	3.14E-37		AT3G05725.1		Protein of unknown function (DUF3511)	DUF3511 domain protein
Sapur.005G038000.1	376.6302939	7.038077386	0.182270899	38.61328	0	0	cyan	0.715919249	5.81E-26	GO:0005515,GO:0006468,GO:0004672	AT3G23750.1		Leucine-rich repeat protein kinase family protein	receptor-like kinase Rhg4, putative
Sapur.005G044700.1	4695.45022	8.92314804	0.199195639	44.7959	0	0	cyan	0.726583746	4.77E-27	GO:0016020,GO:0006075,GO:0003843,GO:0000148	AT2G13680.1	ATGSL02,CALS5,GLS2	callose synthase 5	1%2C3-beta-glucan synthase
Sapur.005G066100.1	324.0335345	4.436645893	0.105804216	41.9326	0	0	cyan	0.793756081	2.73E-35		AT2G21990.1		Protein of unknown function, DUF617	plant-specific domain TIGR01570 family protein
Sapur.005G096200.1	1929.275805	8.614320053	0.172755889	49.86412	0	0	cyan	0.827232905	1.20E-40		AT4G36490.1	ATSFH12,SFH12	SEC14-like 12	Sec14 cytosolic factor, putative
Sapur.005G099400.1	1960.235457	6.33881086	0.127123058	49.86358	0	0	cyan	0.78042667	2.00E-33	GO:0006468,GO:0004672	AT2G18470.1	PERK4	roline-rich extensin-like receptor kinase 4	proline-rich receptor-like kinase
Sapur.005G132200.1	216.6911264	7.987595425	0.17788485	44.90318	0	0	cyan	0.807298555	2.49E-37		AT1G22110.1		structural constituent of ribosome	DUF3049 family protein
Sapur.005G181000.1	594.7014653	5.370625321	0.115589514	46.46291	0	0	cyan	0.773829926	1.50E-32	GO:0006468,GO:0005524,GO:0004674,GO:0004672	AT2G20470.1		AGC (cAMP-dependent, cGMP-dependent and protein kinase C) kinase family protein	Serine/Threonine kinase family protein
Sapur.005G181700.1	294.6142808	5.420836806	0.108673979	49.88164	0	0	cyan	0.854389092	6.40E-46		AT4G28556.1	RIC7	PAK-box/P21-Rho-binding family protein	P21-Rho-binding domain protein
Sapur.005G194400.1	318.8239921	8.252205929	0.209691191	39.35409	0	0	cyan	0.758357181	1.31E-30		AT4G35070.1		SBP (S-ribonuclease binding protein) family protein	zinc finger, C3HC4 type (RING finger) protein
Sapur.006G021300.1	574.9371504	8.194252227	0.206604679	39.6615	0	0	cyan	0.794624174	2.04E-35	GO:0008270	AT5G05070.1		DHHC-type zinc finger family protein	zinc finger protein, putative
Sapur.006G063900.1	2315.436721	7.019788572	0.121537523	57.7582	0	0	cyan	0.791895803	5.06E-35	GO:0005515,GO:0006468,GO:0005524,GO:0004672	AT5G35390.1		Leucine-rich repeat protein kinase family protein	LRR receptor-like kinase
Sapur.006G068900.1	290.6576794	8.500124694	0.214160812	39.69038	0	0	cyan	0.750644079	1.07E-29	GO:0005515	AT2G37670.1		Transducin/WD40 repeat-like superfamily protein	WD domain, G-beta repeat protein
Sapur.006G078000.1	1454.671485	3.99212835	0.088287558	45.21734	0	0	cyan	0.812331398	3.95E-38	GO:0003924,GO:0007017,GO:0005874	AT5G23860.1	TUB8	tubulin beta 8	beta-tubulin
Sapur.006G085900.1	586.9083843	6.238271994	0.123730829	50.41809	0	0	cyan	0.805016827	5.63E-37	GO:0003824,GO:0006470,GO:0004722	AT5G01700.2		Protein phosphatase 2C family protein	protein phosphatase 2C
Sapur.006G091500.1	517.7738868	7.04904456	0.13875395	50.80248	0	0	cyan	0.840869803	3.59E-43		AT5G01970.1			hypothetical protein
Sapur.006G097100.1	850.2585677	8.660907964	0.223048508	38.82971	0	0	cyan	0.739353507	2.04E-28	GO:0005543	AT1G03050.1		ENTH/ANTH/VHS superfamily protein	NA
Sapur.006G103700.1	580.4600683	7.953665089	0.179680987	44.26548	0	0	cyan	0.716974096	4.56E-26	GO:0016887,GO:0005524	AT2G37010.1	ATNAP12,NAP12	non-intrinsic ABC protein 12	white-brown-complex ABC transporter family protein, putative
Sapur.006G105400.1	1663.392568	8.852472955	0.212794661	41.60101	0	0	cyan	0.781671412	1.35E-33					hypothetical protein
Sapur.006G133300.1	568.8511842	8.954784026	0.207413768	43.17353	0	0	cyan	0.768887042	6.48E-32	GO:0016021,GO:0015116,GO:0008272,GO:0055085,GO:0016020,GO:0008271,GO:190235	AT5G19600.1	SULTR3;5	sulfate transporter 3;5	sulfate/bicarbonate/oxalate exchanger and transporter sat-1
Sapur.006G158200.1	369.1951416	8.172471119	0.205642191	39.74122	0	0	cyan	0.756393638	2.25E-30	GO:0006468,GO:0004672	AT2G41970.1		Protein kinase superfamily protein	tyrosine kinase domain protein
Sapur.006G167000.1	272.180104	5.850272393	0.11867589	49.29622	0	0	cyan	0.81204856	4.38E-38	GO:0006887,GO:0000145	AT5G52340.1	ATEXO70A2,EXO70A2	exocyst subunit exo70 family protein A2	EXO70 exocyst complex subunit
Sapur.006G175700.1	176.6271334	6.934299483	0.159217658	43.55233	0	0	cyan	0.810339394	8.23E-38		AT3G09550.1		Ankyrin repeat family protein	ankyrin repeat plant protein
Sapur.006G185600.1	358.4191949	6.692168531	0.166496686	40.194	0	0	cyan	0.738628935	2.45E-28	GO:0006950,GO:0006468,GO:0005524,GO:0004672	AT5G12000.1		Protein kinase protein with adenine nucleotide alpha hydrolases-like domain	kinase domain protein
Sapur.006G227200.1	6767.133906	6.715974404	0.12401459	54.15471	0	0	cyan	0.778522237	3.60E-33		AT2G24450.1	FLA3	FASCICLIN-like arabinogalactan protein 3 precursor	NA
Sapur.007G009000.1	1160.306313	5.942948882	0.111574118	53.26458	0	0	cyan	0.767589204	9.46E-32		AT2G22560.1		Kinase interacting (KIP1-like) family protein	kinase interacting protein
Sapur.007G017400.1	2262.896386	8.442606412	0.158650502	53.21513	0	0	cyan	0.849888773	5.64E-45		AT4G36490.1	ATSFH12,SFH12	SEC14-like 12	phosphatidylinositol/phophatidylcholine transfer protein, putative
Sapur.007G074300.1	2050.459557	9.112744893	0.218452394	41.71501	0	0	cyan	0.750014854	1.27E-29	GO:0005975,GO:0004553	AT5G64790.1		O-Glycosyl hydrolases family 17 protein	glucan endo-1%2C3-beta-glucosidase
Sapur.007G080600.1	115.95604	6.494822877	0.167305093	38.82023	0	0	cyan	0.782871751	9.29E-34	GO:0055114,GO:0020037,GO:0016705,GO:0005506	AT1G64940.1	CYP89A6	cytochrome P450, family 87, subfamily A, polypeptide 6	cytochrome P450 family protein
Sapur.007G083000.1	417.439135	7.363304543	0.17060692	43.15947	0	0	cyan	0.755390402	2.96E-30	GO:0005515	AT5G65160.1		tetratricopeptide repeat (TPR)-containing protein	TPR protein
Sapur.007G092000.1	174.482592	9.23236182	0.24342553	37.92684	0	0	cyan	0.831837601	1.79E-41		AT4G30770.1		Putative membrane lipoprotein	transmembrane protein, putative
Sapur.007G094100.1	475.7495024	9.380171092	0.206296347	45.4694	0	0	cyan	0.784009025	6.49E-34	GO:0055085,GO:0016021,GO:0015299,GO:0006812	AT2G13620.1	ATCHX15,CHX15	cation/hydrogen exchanger 15	cation/H(+) antiporter
Sapur.007G112700.1	1009.070255	7.849443075	0.199435906	39.35822	0	0	cyan	0.765440781	1.76E-31	GO:0006468,GO:0004672	AT2G31500.1	CPK24	calcium-dependent protein kinase 24	calcium-dependent kinase
Sapur.008G026100.1	373.4726213	5.122986367	0.125679232	40.76239	0	0	cyan	0.770937884	3.54E-32	GO:0003723	AT3G10360.1	APUM4,PUM4	pumilio 4	NA
Sapur.008G054000.1	194.6199006	6.820967038	0.150097324	45.44363	0	0	cyan	0.811265305	5.86E-38	GO:0005515,GO:0035556,GO:0007165,GO:0006629,GO:0004435	AT2G40116.1		Phosphoinositide-specific phospholipase C family protein	phosphoinositide-specific phospholipase C
Sapur.008G063900.1	212.0250733	6.449066196	0.144541224	44.61749	0	0	cyan	0.809701552	1.04E-37	GO:0006468,GO:0004672,GO:0005524	AT1G79250.2	AGC1.7	AGC kinase 1.7	Serine/Threonine kinase domain protein
Sapur.008G109300.1	666.286606	4.809297054	0.093708131	51.32209	0	0	cyan	0.821226732	1.32E-39		AT1G24620.1		EF hand calcium-binding protein family	calcium-binding pollen allergen protein
Sapur.008G125800.1	4482.996586	8.669523361	0.216549823	40.03477	0	0	cyan	0.82500136	2.96E-40					NA
Sapur.008G152700.1	308.1033588	7.987844919	0.201051604	39.73032	0	0	cyan	0.727888916	3.48E-27	GO:0006468,GO:0004672	AT1G70460.1	RHS10	root hair specific 10	tyrosine kinase family protein
Sapur.009G026200.1	2136.471254	6.655431408	0.120413432	55.2715	0	0	cyan	0.78714492	2.38E-34		AT3G46750.1			eukaryotic RNA polymerase II heptapeptide repeat protein, putative
Sapur.009G026800.1	235.6792064	5.028986602	0.133264022	37.73702	0	0	cyan	0.763034569	3.51E-31		AT1G07473.1			hypothetical protein
Sapur.009G079900.1	478.5497993	8.769396839	0.208740294	42.01104	0	0	cyan	0.797583019	7.50E-36		AT3G19230.1		Leucine-rich repeat (LRR) family protein	receptor-like kinase
Sapur.009G107000.1	9791.968935	9.011847705	0.236762246	38.06286	0	0	cyan	0.770992573	3.49E-32	GO:0030246,GO:0005975,GO:0004553	AT2G16730.1	BGAL13	glycosyl hydrolase family 35 protein	beta-galactosidase
Sapur.010G028600.1	313.2427022	7.61925233	0.185468004	41.08122	0	0	cyan	0.759031759	1.08E-30	GO:0006468,GO:0004672	AT1G70460.1	RHS10	root hair specific 10	tyrosine kinase domain protein
Sapur.010G045500.1	660.5635029	6.741402583	0.156449825	43.08987	0	0	cyan	0.756178736	2.39E-30	GO:0005515	AT1G04500.1		CCT motif family protein	CCT motif protein
Sapur.010G046900.1	369.5808514	7.600867425	0.159077342	47.78096	0	0	cyan	0.820569065	1.71E-39	GO:0006950	AT4G13450.1		Adenine nucleotide alpha hydrolases-like superfamily protein	universal stress family protein
Sapur.010G055800.1	2245.341623	7.32636873	0.185604439	39.47303	0	0	cyan	0.73056297	1.82E-27		AT3G48140.1		B12D protein	NA
Sapur.010G056200.1	2266.371625	8.133042297	0.195097722	41.68702	0	0	cyan	0.743871388	6.39E-29		AT3G48140.1		B12D protein	NA
Sapur.010G085500.1	4959.077129	9.319814041	0.204221298	45.63586	0	0	cyan	0.735190859	5.81E-28	GO:0016020,GO:0006810,GO:0005215	AT5G37810.1	NIP4;1,NLM4	NOD26-like intrinsic protein 4;1	aquaporin NIP-type protein
Sapur.010G121500.1	496.9346271	7.659308456	0.1611586	47.52653	0	0	cyan	0.77786571	4.40E-33		AT2G03630.1			hypothetical protein
Sapur.010G128900.1	377.4711835	5.719035513	0.118257459	48.36089	0	0	cyan	0.789456157	1.13E-34	GO:0046983,GO:0003677	AT2G03060.2	AGL30	AGAMOUS-like 30	MADS-box transcription factor
Sapur.010G133000.1	489.8960343	5.418038281	0.119398694	45.3777	0	0	cyan	0.763684708	2.91E-31		AT1G16290.1			transglycosylase SLT domain protein
Sapur.010G141800.1	458.2150845	8.949986453	0.198435878	45.10266	0	0	cyan	0.775396525	9.33E-33		AT3G56180.1		Protein of unknown function (DUF567)	tubby C 2 protein
Sapur.010G205300.1	1149.652407	8.902252683	0.2274206	39.14444	0	0	cyan	0.749528884	1.44E-29	GO:0009733	AT3G20210.2	DELTA-VPE,DELTAVPE	delta vacuolar processing enzyme	auxin-induced-like protein
Sapur.011G000900.1	1843.125174	9.403954101	0.23155575	40.61205	0	0	cyan	0.741767062	1.10E-28	GO:0004857	AT4G02250.1		Plant invertase/pectin methylesterase inhibitor superfamily protein	plant invertase/pectin methylesterase inhibitor protein
Sapur.012G004100.1	261.520044	5.417054818	0.134699461	40.21586	0	0	cyan	0.801348383	2.05E-36		AT5G54130.2		Calcium-binding endonuclease/exonuclease/phosphatase family	carbon catabolite repressor protein, putative
Sapur.012G039300.1	671.7458248	7.833713804	0.190731875	41.07186	0	0	cyan	0.720118319	2.20E-26	GO:0005515,GO:0008017,GO:0007018,GO:0005524,GO:0003777,GO:0005871	AT1G63640.1		P-loop nucleoside triphosphate hydrolases superfamily protein with CH (Calponin Homology) domain	NA
Sapur.012G108700.1	887.8707462	5.856074767	0.129629179	45.17559	0	0	cyan	0.769682373	5.13E-32	GO:0005622,GO:0003779,GO:0030042,GO:0015629	AT4G25590.1	ADF7	actin depolymerizing factor 7	actin depolymerizing factor
Sapur.013G035400.1	116.8294589	4.892551646	0.125943543	38.84718	0	0	cyan	0.794125573	2.41E-35	GO:0005515,GO:0006468,GO:0004672	AT3G23750.1		Leucine-rich repeat protein kinase family protein	receptor-like kinase Rhg4
Sapur.013G065900.1	1504.910205	8.053937351	0.170654246	47.19447	0	0	cyan	0.799099502	4.46E-36		AT3G02970.1	EXL6	EXORDIUM like 6	phosphate-induced protein 1 motif protein
Sapur.013G122100.1	914.0464201	7.949327603	0.161227239	49.30512	0	0	cyan	0.805032189	5.60E-37		AT2G20700.1	LLG2	LORELEI-LIKE-GPI ANCHORED PROTEIN 2	GPI-anchored protein lorelei protein, putative
Sapur.014G072700.1	584.6763397	8.854966234	0.222259678	39.84063	0	0	cyan	0.788819735	1.39E-34		AT4G01140.1		Protein of unknown function (DUF1191)	Atpob1, putative
Sapur.014G095300.1	1718.463777	6.920389581	0.182300931	37.96135	0	0	cyan	0.708159449	3.33E-25	GO:0004857	AT1G02550.1		Plant invertase/pectin methylesterase inhibitor superfamily protein	plant invertase/pectin methylesterase inhibitor domain protein
Sapur.014G124200.1	813.6948984	8.964901877	0.238263513	37.626	0	0	cyan	0.796668862	1.02E-35	GO:0007264,GO:0005525	AT3G21700.3	ATSGP2,SGP2	Ras-related small GTP-binding family protein	septum-promoting GTP-binding protein
Sapur.014G133500.1	1499.544981	8.52748579	0.158408835	53.83214	0	0	cyan	0.794447119	2.17E-35	GO:0005515	AT5G35700.1	FIM2	fimbrin-like protein 2	fimbrin, putative
Sapur.016G004600.1	255.3820186	5.445937697	0.122210096	44.56209	0	0	cyan	0.79937095	4.07E-36		AT1G12150.1		Plant protein of unknown function (DUF827)	weak movement under blue light protein
Sapur.016G046600.1	1165.416298	6.027193285	0.147132355	40.96443	0	0	cyan	0.738299616	2.66E-28		AT2G41905.1			NA
Sapur.016G071000.1	4103.530788	6.940757386	0.123095481	56.38515	0	0	cyan	0.78068554	1.84E-33		AT3G52600.1	AtcwINV2,CWINV2	cell wall invertase 2	cell-wall invertase
Sapur.016G077000.1	368.0824598	8.140629683	0.20234852	40.23073	0	0	cyan	0.739727829	1.85E-28		AT5G02730.1		CAP (Cysteine-rich secretory proteins, Antigen 5, and Pathogenesis-related 1 protein) superfamily protein	cysteine-rich secretory family protein
Sapur.016G097700.1	344.9053494	9.349148319	0.209974741	44.52511	0	0	cyan	0.812243095	4.08E-38	GO:0055114,GO:0016491,GO:0050664,GO:0004601	AT5G60010.1		ferric reductase-like transmembrane component family protein	respiratory burst oxidase-like protein
Sapur.016G111600.1	303.1798208	8.585222195	0.227753179	37.69529	0	0	cyan	0.761495704	5.42E-31	GO:0003824,GO:0006470,GO:0004722	AT5G01700.2		Protein phosphatase 2C family protein	protein phosphatase 2C
Sapur.016G137400.1	81.88425083	5.054318755	0.132916468	38.02628	0	0	cyan	0.811399564	5.57E-38		AT3G54020.1	AtIPCS1	Arabidopsis Inositol phosphorylceramide synthase 1	phosphatidylinositol:ceramide inositolphosphotransferase
Sapur.016G139300.1	951.0704383	8.62321718	0.168006991	51.32654	0	0	cyan	0.781179428	1.58E-33		AT2G29620.1			transmembrane protein, putative
Sapur.016G200400.1	878.3661828	9.579609936	0.247982886	38.63013	0	0	cyan	0.754631378	3.64E-30	GO:0006887,GO:0000145	AT5G13990.1	ATEXO70C2,EXO70C2	exocyst subunit exo70 family protein C2	Exo70 exocyst complex subunit family protein
Sapur.016G236600.1	477.9228713	8.817907121	0.181145176	48.67867	0	0	cyan	0.815656051	1.13E-38		AT5G55980.1		serine-rich protein-related	hypothetical protein
Sapur.016G276700.1	3834.437498	8.887032493	0.21854608	40.66634	0	0	cyan	0.771523077	2.98E-32	GO:0031225,GO:0016049,GO:0010215	AT3G20580.1	COBL10	COBRA-like protein 10 precursor	COBRA-like protein
Sapur.016G276800.1	672.6442511	4.710918335	0.125154191	37.64092	0	0	cyan	0.773366188	1.72E-32		AT4G27100.2		Ypt/Rab-GAP domain of gyp1p superfamily protein	Rab GTPase activator
Sapur.016G296100.1	1213.082545	7.89332894	0.188587585	41.85498	0	0	cyan	0.733768015	8.27E-28	GO:0055114,GO:0050662,GO:0015936,GO:0004420	AT1G76490.1	HMG1,HMGR1	hydroxy methylglutaryl CoA reductase 1	3-hydroxy-3-methylglutaryl coenzyme A reductase, hydroxymethylglutaryl-CoA reductase
Sapur.017G060800.1	268.5143203	8.236735449	0.205711405	40.04025	0	0	cyan	0.753426149	5.06E-30					hypothetical protein
Sapur.017G104700.1	1517.330896	8.884491142	0.211881743	41.93137	0	0	cyan	0.786292876	3.13E-34		AT3G03430.1		Calcium-binding EF-hand family protein	polcalcin Nic protein
Sapur.018G001200.1	1237.109313	8.469639945	0.200977105	42.14231	0	0	cyan	0.752450884	6.59E-30	GO:0006468,GO:0005524,GO:0004672	AT2G24370.1		Protein kinase protein with adenine nucleotide alpha hydrolases-like domain	NA
Sapur.018G004900.1	1102.690016	9.51739161	0.21165306	44.96695	0	0	cyan	0.816978951	6.85E-39	GO:0005516	AT5G24880.1			plant calmodulin-binding domain protein
Sapur.018G006800.1	2173.467245	9.200697541	0.243635371	37.76421	0	0	cyan	0.731810292	1.34E-27	GO:0016788	AT4G18970.1		GDSL-like Lipase/Acylhydrolase superfamily protein	GDSL-like lipase/acylhydrolase
Sapur.018G028900.1	1003.980288	4.848537471	0.128980602	37.59121	0	0	cyan	0.736774804	3.91E-28	GO:0005096	AT4G32630.1		ArfGap/RecO-like zinc finger domain-containing protein	Arf GTPase activating protein, putative
Sapur.018G056200.1	396.0281711	7.453527763	0.189344898	39.36482	0	0	cyan	0.718102697	3.51E-26	GO:0032012,GO:0005086	AT5G19610.1	GNL2	GNOM-like 2	ARF guanine nucleotide exchange factor
Sapur.018G059200.1	448.8685264	8.341196048	0.163802876	50.92216	0	0	cyan	0.814719323	1.62E-38	GO:0005089	AT3G24620.1	ATROPGEF8,ROPGEF8	RHO guanyl-nucleotide exchange factor 8	NA
Sapur.018G084400.1	1852.037377	6.716982155	0.125050936	53.71397	0	0	cyan	0.7956637	1.44E-35	GO:0008081,GO:0006629,GO:0008889	AT5G58050.1	SVL4	SHV3-like 4	glycerophosphoryl diester phosphodiesterase, putative
Sapur.018G087300.1	19146.8912	7.782646915	0.151736919	51.2904	0	0	cyan	0.768404075	7.46E-32	GO:0046872,GO:0000166	AT3G42640.1	AHA8,HA8	H(+)-ATPase 8	plasma membrane H+ ATPase
Sapur.018G109300.1	418.072347	6.689566333	0.139690989	47.88832	0	0	cyan	0.822210079	8.99E-40	GO:0005543	AT5G57200.1		ENTH/ANTH/VHS superfamily protein	clathrin assembly protein
Sapur.019G057800.1	1689.192097	8.785118188	0.221431478	39.67421	0	0	cyan	0.798610077	5.28E-36	GO:0006468,GO:0005524,GO:0004672	AT3G07070.1		Protein kinase superfamily protein	receptor kinase-like protein
Sapur.15WG001000.1	3400.505802	7.105068972	0.150204368	47.30268	0	0	cyan	0.7820756	1.19E-33					hypothetical protein
Sapur.15WG084400.1	2085.664919	9.415059117	0.231360543	40.69432	0	0	cyan	0.791422665	5.92E-35					hypothetical protein
Sapur.15WG118900.1	1855.368813	6.836518437	0.165654903	41.26964	0	0	cyan	0.714200401	8.59E-26		AT4G25040.1		Uncharacterised protein family (UPF0497)	plant integral membrane protein
Sapur.T001800.1	3009.810838	8.648923959	0.1697926	50.93817	0	0	cyan	0.808383189	1.68E-37		AT4G03620.1		myosin heavy chain-related	NA
Sapur.010G110000.1	611.6401496	8.460858404	0.225889376	37.45576	4.84E-307	2.82E-305	cyan	0.818966617	3.19E-39		AT2G47480.1		Protein of unknown function (DUF3511)	NA
Sapur.003G095900.1	571.6046431	3.894540995	0.104164968	37.3882	6.08E-306	3.51E-304	cyan	0.777820936	4.46E-33		AT1G63930.1	ROH1	from the Czech \'roh\' meaning \'corner\'	plant/F6A14-15 protein, putative
Sapur.013G094500.1	186.7465357	6.750012826	0.180566819	37.38235	7.56E-306	4.36E-304	cyan	0.725310268	6.47E-27	GO:0006468,GO:0005524,GO:0004672	AT1G71530.1		Protein kinase superfamily protein	cyclin-dependent kinase-like Serine/Threonine kinase family protein
Sapur.003G164600.1	705.9334526	3.978448211	0.106689635	37.28992	2.39E-304	1.37E-302	cyan	0.75604491	2.47E-30		AT1G55260.1		Bifunctional inhibitor/lipid-transfer protein/seed storage 2S albumin superfamily protein	protease inhibitor/seed storage/lipid transfer family protein
Sapur.008G059500.1	295.490981	8.632488713	0.232140773	37.19333	8.75E-303	4.95E-301	cyan	0.762448145	4.14E-31		AT3G56180.1		Protein of unknown function (DUF567)	tubby C 2 protein
Sapur.006G022100.1	1158.541844	8.430941117	0.227673792	37.03079	3.66E-300	2.06E-298	cyan	0.747162584	2.70E-29	GO:0016773	AT2G40850.1	ATPI4K GAMMA 1.PI4K GAN	phosphoinositide 4-kinase gamma 1	phosphatidylinositol 3- and 4-kinase
Sapur.006G115200.1	479.0298387	5.712408786	0.154317847	37.01716	6.06E-300	3.40E-298	cyan	0.698728373	2.59E-24	GO:0005515,GO:0006468,GO:0005524,GO:0004672	AT3G42880.1		Leucine-rich repeat protein kinase family protein	receptor-like kinase
Sapur.001G000200.1	13531.02545	8.693081031	0.234956173	36.99873	1.20E-299	6.72E-298	cyan	0.78600281	3.44E-34	GO:0055114,GO:0016491,GO:0005507	AT1G55570.1	sks12	SKU5 similar 12	L-ascorbate oxidase-like protein
Sapur.001G087200.1	358.3869757	5.304266878	0.143758243	36.89713	5.14E-298	2.85E-296	cyan	0.763614356	2.97E-31	GO:0008270	AT3G61230.1		GATA type zinc finger transcription factor family protein	pollen-specific protein SF3, putative
Sapur.005G050400.1	136.1280339	8.432547706	0.229729427	36.70643	5.77E-295	3.14E-293	cyan	0.784379609	5.77E-34		AT4G30770.1		Putative membrane lipoprotein	transmembrane protein, putative
Sapur.011G073600.1	5505.380051	8.53597818	0.23376046	36.51592	6.20E-292	3.36E-290	cyan	0.796345022	1.14E-35		AT1G29140.1		Pollen Ole e 1 allergen and extensin family protein	pollen protein Ole E I-like protein
Sapur.002G029900.1	2620.06621	8.721809962	0.238876766	36.51176	7.22E-292	3.90E-290	cyan	0.764543528	2.28E-31	GO:0055085,GO:0016021	AT2G38940.1	ATPT2,PHT1;4	phosphate transporter 1;4	inorganic phosphate transporter 1-7, putative
Sapur.010G126100.1	363.1494401	4.322932864	0.119009541	36.32425	6.70E-289	3.57E-287	cyan	0.736487551	4.20E-28		AT1G13970.1		Protein of unknown function (DUF1336)	DUF1336 family protein
Sapur.016G252000.1	2724.394147	8.727045733	0.240738785	36.2511	9.55E-288	5.03E-286	cyan	0.798790872	4.96E-36		AT1G29140.1		Pollen Ole e 1 allergen and extensin family protein	anther-specific protein LAT52
Sapur.014G086000.1	370.5641904	3.186416023	0.087957274	36.22686	2.30E-287	1.21E-285	cyan	0.804215435	7.49E-37		AT4G01575.1		serine protease inhibitor, Kazal-type family protein	serine-type endopeptidase inhibitor, putative
Sapur.010G124200.1	394.2706654	2.998817785	0.082996411	36.1319	7.16E-286	3.71E-284	cyan	0.82473709	3.29E-40		AT1G69330.1		RING/U-box superfamily protein	ubiquitin-protein ligase, putative
Sapur.018G114500.1	3129.264262	8.469737179	0.234488901	36.11999	1.10E-285	5.69E-284	cyan	0.751008222	9.72E-30	GO:0005515,GO:0006468,GO:0005524,GO:0004672	AT5G35390.1		Leucine-rich repeat protein kinase family protein	LRR receptor-like kinase
Sapur.008G137100.1	212.5792412	6.490804948	0.179727614	36.11468	1.33E-285	6.89E-284	cyan	0.717061829	4.47E-26					NA
Sapur.010G046400.1	569.4360474	8.736615445	0.243001798	35.95288	4.56E-283	2.32E-281	cyan	0.77386126	1.48E-32	GO:0005515	AT1G04540.1		Calcium-dependent lipid-binding (CaLB domain) family protein	calcium-dependent lipid-binding domain protein, putative
Sapur.012G053200.1	2238.851746	8.91940102	0.248687013	35.86597	1.04E-281	5.24E-280	cyan	0.718006009	3.59E-26		AT1G74830.1		Protein of unknown function, DUF593	zein-binding protein
Sapur.001G118000.1	260.98377	8.241976407	0.229893811	35.85123	1.76E-281	8.87E-280	cyan	0.746213674	3.47E-29		AT2G35215.1			pollen-specific small CDPK-interacting protein, putative
Sapur.016G213800.1	2838.885268	8.832974859	0.246867615	35.78021	2.24E-280	1.13E-278	cyan	0.766289714	1.38E-31					transmembrane protein, putative
Sapur.007G032600.1	411.6233233	9.63457127	0.269537755	35.74479	7.97E-280	3.99E-278	cyan	0.708786281	2.90E-25	GO:0005515	AT3G50390.1		Transducin/WD40 repeat-like superfamily protein	NA
Sapur.005G072000.1	13570.21789	8.563124386	0.239808633	35.70816	2.95E-279	1.47E-277	cyan	0.773360227	1.72E-32	GO:0055085,GO:0016021	AT3G19940.1		Major facilitator superfamily protein	monosaccharide transporter
Sapur.006G186400.1	84.0939725	9.289043665	0.260683676	35.63339	4.26E-278	2.10E-276	cyan	0.825064142	2.89E-40					hypothetical protein
Sapur.15WG121200.1	411.2844678	4.199203716	0.117997854	35.58712	2.22E-277	1.08E-275	cyan	0.736205451	4.51E-28	GO:0005737,GO:0005515,GO:0005096	AT5G61980.1	AGD1	ARF-GAP domain 1	ADP-ribosylation factor GTPase-activating protein AGD3
Sapur.002G106800.1	215.2796493	5.908876253	0.166355032	35.51967	2.44E-276	1.19E-274	cyan	0.723879356	9.09E-27	GO:0005515	AT2G26490.1		Transducin/WD40 repeat-like superfamily protein	F-box and WD40 domain protein, putative
Sapur.014G098800.1	878.7195709	6.877132991	0.194065202	35.43723	4.56E-275	2.22E-273	cyan	0.729929579	2.12E-27		AT5G61710.1			DUF761 domain protein
Sapur.012G078800.1	244.0292398	9.173044329	0.259064036	35.40841	1.27E-274	6.13E-273	cyan	0.737938637	2.92E-28		AT5G50830.2			hypothetical protein
Sapur.017G104400.1	312.0607715	9.237491406	0.261053247	35.38547	2.86E-274	1.38E-272	cyan	0.741825715	1.08E-28	GO:0080048	AT4G26850.1	VTC2	mannose-1-phosphate guanylyltransferase (GDP)s:GDP-galactose:mannose-1-phosphate guanylyltransferases:GD	GDP-L-galactose phosphorylase
Sapur.006G206900.1	322.88266	7.63704451	0.215851111	35.38108	3.34E-274	1.61E-272	cyan	0.717566983	3.98E-26	GO:0055085,GO:0016020,GO:0006811,GO:0005216	AT2G25600.1	AKT6,SPIK	Shaker pollen inward K+ channel	potassium channel AKT1 protein
Sapur.008G069500.1	323.3106762	6.231029576	0.176160312	35.37136	4.71E-274	2.26E-272	cyan	0.70889634	2.83E-25	GO:0046983,GO:0003677	AT2G03060.2	AGL30	AGAMOUS-like 30	MADS-box transcription factor
Sapur.013G046000.1	251.9307885	7.941898391	0.22468409	35.34695	1.12E-273	5.35E-272	cyan	0.701631304	1.39E-24	GO:0016021	AT5G18520.1		Lung seven transmembrane receptor family protein	lung seven transmembrane receptor, putative
Sapur.018G004500.1	2430.496205	7.777258059	0.220216722	35.31638	3.29E-273	1.57E-271	cyan	0.73409128	7.64E-28		AT2G24450.1	FLA3	FASCICLIN-like arabinogalactan protein 3 precursor	NA
Sapur.016G155200.1	3825.644962	8.654752331	0.2453191	35.27957	1.21E-272	5.77E-271	cyan	0.809412682	1.16E-37		AT2G41905.1			NA
Sapur.010G043900.1	3997.959981	8.787968643	0.249465021	35.22726	7.65E-272	3.64E-270	cyan	0.763052825	3.49E-31		AT2G33420.1		Protein of unknown function (DUF810)	DUF810 family protein
Sapur.009G095400.1	2303.318225	7.580824424	0.215241011	35.22017	9.82E-272	4.67E-270	cyan	0.673848616	4.00E-22	GO:0006468,GO:0004672	AT4G39110.1		Malectin/receptor-like protein kinase family protein	receptor-like kinase, putative
Sapur.010G063600.1	3483.538022	8.432016854	0.239973024	35.13735	1.81E-270	8.56E-269	cyan	0.778913274	3.19E-33					pollen allergen ole E 6 protein
Sapur.016G252100.1	4938.481461	8.77802369	0.250217249	35.08161	1.29E-269	6.02E-268	cyan	0.778072975	4.13E-33		AT1G29140.1		Pollen Ole e 1 allergen and extensin family protein	anther-specific protein LAT52
Sapur.010G160100.1	418.4200319	6.804927931	0.194078845	35.0627	2.50E-269	1.17E-267	cyan	0.701279389	1.50E-24	GO:0005975,GO:0004553	AT3G55430.1		O-Glycosyl hydrolases family 17 protein	NA
Sapur.005G055300.1	64.16260539	6.151360412	0.175484349	35.05361	3.44E-269	1.60E-267	cyan	0.80763384	2.21E-37		AT4G40020.1		Myosin heavy chain-related protein	hypothetical protein
Sapur.016G056200.1	190.183722	8.694150103	0.248635775	34.96741	7.04E-268	3.27E-266	cyan	0.733471014	8.90E-28	GO:0006468,GO:0004672	AT2G41970.1		Protein kinase superfamily protein	tyrosine kinase domain protein
Sapur.011G056000.1	448.0920054	8.82293445	0.252951118	34.88	1.49E-266	6.87E-265	cyan	0.775699106	8.51E-33		AT2G34610.1			NA
Sapur.005G042800.1	1873.97907	8.066977181	0.231435476	34.85627	3.42E-266	1.56E-264	cyan	0.712578343	1.24E-25	GO:0006468,GO:0004672	AT5G28680.1	ANX2	Malectin/receptor-like protein kinase family protein	receptor-like kinase ANXUR1
Sapur.010G080700.1	507.6344018	2.844633191	0.081623275	34.85076	4.15E-266	1.89E-264	cyan	0.827964163	8.91E-41		AT1G24620.1		EF hand calcium-binding protein family	NA
Sapur.012G023600.1	444.8421722	7.193146864	0.207257492	34.70633	6.32E-264	2.85E-262	cyan	0.676028928	2.62E-22	GO:0005096	AT3G17660.1	AGD15	ARF-GAP domain 15	NA
Sapur.012G085000.1	2881.296832	7.943286709	0.229094432	34.67254	2.04E-263	9.20E-262	cyan	0.652062417	2.25E-20	GO:0042545,GO:0030599,GO:0005618	AT1G69940.1	ATPPME1,PPME1	Pectin lyase-like superfamily protein	pectinesterase
Sapur.004G118500.1	1128.189411	6.606659408	0.190774342	34.63075	8.71E-263	3.90E-261	cyan	0.65892533	6.55E-21		AT2G21540.1	ATSFH3,SFH3	SEC14-like 3	SEC14 cytosolic factor, putative
Sapur.004G003700.1	557.3963333	4.266219492	0.123353328	34.58536	4.19E-262	1.87E-260	cyan	0.738194291	2.73E-28	GO:0016020	AT4G22120.2		ERD (early-responsive to dehydration stress) family protein	early-responsive to dehydration stress-like protein
Sapur.016G186300.1	2665.104001	6.054006341	0.175162057	34.56232	9.31E-262	4.13E-260	cyan	0.688294824	2.28E-23					hypothetical protein
Sapur.002G074100.1	400.008381	8.79428214	0.254571303	34.54546	1.67E-261	7.38E-260	cyan	0.709950508	2.24E-25	GO:0006355,GO:0005634,GO:0003700,GO:0046872,GO:0030001	AT4G08570.1		Heavy metal transport/detoxification superfamily protein	NA
Sapur.013G127000.1	1008.432353	9.614698327	0.279202946	34.43624	7.24E-260	3.16E-258	cyan	0.70156745	1.41E-24		AT1G60815.1	RALFL7	RALF-like 7	rapid aLkalinization factor
Sapur.008G028600.1	405.0614093	4.964910084	0.144185461	34.43419	7.76E-260	3.38E-258	cyan	0.708504622	3.09E-25	GO:0008289	AT3G54800.1		Pleckstrin homology (PH) and lipid-binding START domains-containing protein	pleckstrin-like (PH) and lipid-binding START domain protein
Sapur.002G163900.1	931.97976	8.497847566	0.24679434	34.43291	8.12E-260	3.53E-258	cyan	0.724942685	7.06E-27	GO:0004857	AT1G02550.1		Plant invertase/pectin methylesterase inhibitor superfamily protein	plant invertase/pectin methylesterase inhibitor protein
Sapur.T075400.1	1702.411019	7.65672919	0.222730542	34.37665	5.63E-259	2.44E-257	cyan	0.632175945	6.73E-19	GO:0055085,GO:0016021,GO:0022857	AT4G16480.1	ATINT4,INT4	inositol transporter 4	inositol transporter, putative
Sapur.019G035200.1	4627.444465	2.798227804	0.08152116	34.32517	3.31E-258	1.43E-256	cyan	0.801867143	1.71E-36	GO:0003924,GO:0007017,GO:0005874	AT5G19780.1	TUA5	tubulin alpha-5	alpha-tubulin
Sapur.T049100.1	198.4702882	8.194792127	0.239268031	34.24942	4.45E-257	1.91E-255	cyan	0.713359595	1.04E-25		AT1G75160.1		Protein of unknown function (DUF620)	DUF620 family protein
Sapur.014G077400.1	160.4793529	7.097999752	0.20747404	34.21151	1.63E-256	6.99E-255	cyan	0.747909906	2.22E-29		AT2G45360.1		Protein of unknown function (DUF1442)	NA
Sapur.019G067300.1	3104.994381	10.67300769	0.312186822	34.18789	3.66E-256	1.56E-254	cyan	0.657374013	8.68E-21	GO:0005975,GO:0004650	AT3G07820.1		Pectin lyase-like superfamily protein	NA
Sapur.013G127100.1	448.7141428	9.323980663	0.272822977	34.17594	5.51E-256	2.35E-254	cyan	0.714362147	8.28E-26		AT1G65690.1		Late embryogenesis abundant (LEA) hydroxyproline-rich glycoprotein family	hypothetical protein
Sapur.019G063600.1	113.308408	8.70198991	0.254976063	34.12865	2.77E-255	1.17E-253	cyan	0.807350034	2.44E-37	GO:0045454,GO:0015035,GO:0006662	AT3G08710.2	ATH9,TH9,TRX H9	thioredoxin H-type 9	thioredoxin H-type protein, putative
Sapur.016G254500.1	99.78684857	6.373641264	0.186864426	34.10837	5.54E-255	2.34E-253	cyan	0.736979972	3.71E-28		AT2G20562.1			transmembrane protein, putative
Sapur.15WG003100.1	255.9208199	5.101273599	0.149989138	34.01095	1.53E-253	6.42E-252	cyan	0.721066734	1.76E-26		AT5G54130.2		Calcium-binding endonuclease/exonuclease/phosphatase family	carbon catabolite repressor protein, putative
Sapur.008G112800.1	945.0126515	4.316107945	0.127587972	33.82849	7.52E-251	3.09E-249	cyan	0.771943978	2.63E-32	GO:0016787	AT1G14700.1	ATPAP3,PAP3	purple acid phosphatase 3	tartrate-resistant acid phosphatase type 5, putative

Sapur.006G113000.1	184.6815677	7.758746027	0.230170002	33.70876	4.30E-249	1.75E-247	cyan	0.737371335	3.36E-28	GO:0042545,GO:0030599,GO:0005618	AT1G69940.1	ATPPME1,PPME1	Pectin lyase-like superfamily protein	pectinesterase
Sapur.006G211300.1	243.6390726	4.877671443	0.145371506	33.55315	8.10E-247	3.25E-245	cyan	0.712913539	1.15E-25		AT4G32270.1		Ubiquitin-like superfamily protein	hypothetical protein
Sapur.014G062300.1	2154.760095	2.849958857	0.085163433	33.46458	1.58E-245	6.32E-244	cyan	0.800361938	2.89E-36	GO:0008270	AT1G01780.1		GATA type zinc finger transcription factor family protein	LIM domain protein PLIM-2
Sapur.011G029000.1	131.5994386	8.521461611	0.254691749	33.45794	1.97E-245	7.88E-244	cyan	0.766958796	1.14E-31	GO:0005515,GO:0016020	AT1G61290.1	ATSYP124,SYP124	syntaxin of plants 124	syntaxin-124 protein
Sapur.007G001500.1	504.0296889	8.28231949	0.248157854	33.37521	3.14E-244	1.24E-242	cyan	0.706523892	4.78E-25	GO:0005515,GO:0006468,GO:0004672	AT3G20190.1		Leucine-rich repeat protein kinase family protein	receptor-like kinase
Sapur.009G095300.1	515.1930294	3.462151734	0.103800358	33.35395	6.39E-244	2.52E-242	cyan	0.774432941	1.25E-32		AT3G03170.1			hypothetical protein
Sapur.009G052600.1	274.4852587	5.457978245	0.163849188	33.31099	2.68E-243	1.06E-241	cyan	0.697561589	3.31E-24		AT5G19473.1		RPM1-interacting protein 4 (RIN4) family protein	pathogenic type III effector avirulence factor Avr AvrRpt-cleavage: cleavage site protein
Sapur.006G003000.1	180.9529334	6.17975535	0.185942162	33.23483	3.38E-242	1.33E-240	cyan	0.6802088	1.16E-22		AT1G12150.1		Plant protein of unknown function (DUF827)	weak movement under blue light protein
Sapur.007G001400.1	566.1772712	7.718936657	0.232714394	33.16914	3.00E-241	1.17E-239	cyan	0.64023561	1.75E-19	GO:0006950,GO:0006468,GO:0005524,GO:0004672	AT1G16760.1		Protein kinase protein with adenine nucleotide alpha hydrolases-like domain	adenine nucleotide alpha hydrolase-domain kinase
Sapur.017G050500.1	956.0985059	8.368732476	0.252673374	33.12075	1.49E-240	5.78E-239	cyan	0.684221086	5.20E-23					NA
Sapur.002G160800.1	601.6133075	7.562869477	0.228689342	33.07049	7.90E-240	3.05E-238	cyan	0.680259856	1.14E-22		AT5G61710.1			DUF761 domain protein
Sapur.019G067500.1	3011.193171	10.20221252	0.308983677	33.01861	4.39E-239	1.69E-237	cyan	0.680233439	1.15E-22	GO:0005975,GO:0004650	AT3G07820.1		Pectin lyase-like superfamily protein	NA
Sapur.004G022200.1	217.8243935	8.856965464	0.269351254	32.88258	3.90E-237	1.48E-235	cyan	0.71910594	2.78E-26	GO:0005515,GO:0016020	AT1G61290.1	ATSYP124,SYP124	syntaxin of plants 124	syntaxin-124 protein
Sapur.013G143100.1	118.8189234	5.299270121	0.1622657	32.65798	6.17E-234	2.30E-232	cyan	0.781819982	1.29E-33		AT2G46170.1		Reticulon family protein	NA
Sapur.010G105800.1	4232.271596	9.194551364	0.282224906	32.57881	8.19E-233	3.03E-231	cyan	0.692577039	9.43E-24	GO:0005975,GO:0004650	AT3G07820.1		Pectin lyase-like superfamily protein	NA
Sapur.008G089000.1	48.325345	5.717730677	0.17593287	32.4995	1.08E-231	3.98E-230	cyan	0.766913142	1.15E-31		AT3G25600.1		Calcium-binding EF-hand family protein	NA
Sapur.010G003800.1	1265.178099	8.480584581	0.261665809	32.40998	1.99E-230	7.21E-229	cyan	0.750274464	1.18E-29	GO:0055085,GO:0016021,GO:0015299,GO:0006812	AT3G17630.1	ATCHX19,CHX19	cation/H+ exchanger 19	cation proton exchanger
Sapur.006G043600.1	544.9175788	7.635315015	0.235753611	32.38684	4.21E-230	1.52E-228	cyan	0.670301886	7.89E-22		AT2G41905.1			transmembrane protein, putative
Sapur.002G020400.1	978.5595028	7.123762838	0.220128165	32.36189	9.44E-230	3.41E-228	cyan	0.633381166	5.51E-19	GO:0008017,GO:0007018,GO:0005524,GO:0003777,GO:0005871	AT3G51150.2		ATP binding microtubule motor family protein	kinesin motor domain protein
Sapur.009G046600.1	597.5176507	11.66772542	0.360552324	32.3607	9.81E-230	3.54E-228	cyan	0.549864256	8.74E-14	GO:0005975,GO:0004650	AT3G07970.1	QRT2	Pectin lyase-like superfamily protein	polygalacturonase
Sapur.008G122000.1	7067.613639	8.519931976	0.263493384	32.33452	2.29E-229	8.26E-228	cyan	0.719634474	2.46E-26	GO:0055085,GO:0016021	AT5G26250.1		Major facilitator superfamily protein	sugar transporter, putative
Sapur.019G067100.1	2343.264848	9.637325019	0.298081096	32.33122	2.55E-229	9.18E-228	cyan	0.584932095	8.69E-16	GO:0005975,GO:0004650	AT3G07820.1		Pectin lyase-like superfamily protein	NA
Sapur.005G052700.1	174.7256781	6.123388618	0.1899109	32.24348	4.34E-228	1.55E-226	cyan	0.681984376	8.13E-23		AT1G07710.1		Ankyrin repeat family protein	ankyrin-like protein
Sapur.012G000900.1	2297.963216	8.115089995	0.252150326	32.18354	3.00E-227	1.07E-225	cyan	0.772688629	2.11E-32					hypothetical protein
Sapur.012G044800.1	583.3884434	4.119667491	0.128007083	32.18312	3.04E-227	1.08E-225	cyan	0.712490107	1.27E-25		AT1G74220.1			hypothetical protein
Sapur.001G171800.1	1214.249263	6.309818989	0.19658751	32.09674	4.90E-226	1.72E-224	cyan	0.687774936	2.53E-23					hypothetical protein
Sapur.018G073100.1	87.09112622	7.715616796	0.240924543	32.02503	4.89E-225	1.71E-223	cyan	0.748669503	1.81E-29		AT1G70420.1		Protein of unknown function (DUF1645)	hypothetical protein
Sapur.007G050900.1	1293.608594	2.836431373	0.08858276	32.02013	5.72E-225	1.99E-223	cyan	0.784785005	5.07E-34	GO:0008270,GO:0006355,GO:0005634,GO:0004402,GO:0003712,GO:0005515	AT5G67480.2	ATBT4,BT4	BTB and TAZ domain protein 4	speckle-type POZ protein-like protein B
Sapur.002G157800.1	2072.512937	3.760317392	0.117522605	31.99655	1.22E-224	4.22E-223	cyan	0.726135845	5.31E-27	GO:0006950	AT4G02380.1	AtLEA5,SAG21	senescence-associated gene 21	late embryogenesis abundant protein
Sapur.001G106100.1	116.2754705	8.563202407	0.267852222	31.96988	2.86E-224	9.89E-223	cyan	0.731970596	1.29E-27	GO:0016021	AT2G23810.1	TET8	tetraspanin8	NA
Sapur.15WG092700.1	921.3570305	8.41442025	0.265106289	31.7398	4.39E-221	1.48E-219	cyan	0.696388008	4.25E-24		AT5G62850.1	AtVEX1	Nodulin MtN3 family protein	bidirectional sugar transporter SWEET6b
Sapur.013G070100.1	274.7593007	2.898909612	0.091421884	31.70914	1.16E-220	3.93E-219	cyan	0.789981266	9.50E-35		AT3G45400.1		exostosin family protein	secondary cell wall-related glycosyltransferase family 47 protein
Sapur.006G196800.1	107.0274544	4.536392415	0.143428541	31.62824	1.51E-219	5.04E-218	cyan	0.761468127	5.46E-31	GO:0008270,GO:0005515	AT5G11620.1		SWIM zinc finger family protein / mitogen-activated protein kinase kinase kinase (MAPKKK)-related	SWIM zinc finger protein
Sapur.008G113400.1	363.3004779	8.191034983	0.258997995	31.62586	1.63E-219	5.43E-218	cyan	0.733001002	1.00E-27	GO:0005543	AT1G25240.1		ENTH/VHS/GAT family protein	clathrin assembly protein
Sapur.002G147300.1	238.7402308	4.186573216	0.132463972	31.60537	3.11E-219	1.04E-217	cyan	0.747694531	2.35E-29		AT2G46300.1		Late embryogenesis abundant (LEA) hydroxyproline-rich glycoprotein family	late embryogenesis abundant protein
Sapur.010G179700.1	250.0179803	9.538023054	0.302067496	31.5758	7.94E-219	2.62E-217	cyan	0.713275489	1.06E-25		AT5G36805.1		Plant thionin family protein	NA
Sapur.001G049400.1	317.7088645	7.399239311	0.234963695	31.49099	1.15E-217	3.79E-216	cyan	0.725843153	5.69E-27	GO:0006887,GO:0000145	AT5G13150.1	ATEXO70C1,EXO70C1	exocyst subunit exo70 family protein C1	Exo70 exocyst complex subunit
Sapur.004G070200.1	2488.372711	9.40326911	0.300787968	31.26212	1.53E-214	4.93E-213	cyan	0.669584088	9.04E-22	GO:0042545,GO:0030599,GO:0005618	AT1G69940.1	ATPPME1,PPME1	Pectin lyase-like superfamily protein	pectinesterase
Sapur.006G075100.1	447.8342182	5.9425096	0.190363451	31.21665	6.33E-214	2.04E-212	cyan	0.734425061	7.03E-28	GO:0016020,GO:0006810,GO:0005215	AT5G28470.1		Major facilitator superfamily protein	peptide/nitrate transporter, putative
Sapur.006G174000.1	2821.195883	8.200454943	0.262835451	31.19996	1.07E-213	3.43E-212	cyan	0.722542926	1.25E-26		AT3G52720.1	ACA1,ATACA1,CAH1	alpha carbonic anhydrase 1	carbonate dehydratase
Sapur.019G072800.1	3085.416525	8.701761401	0.279699251	31.11114	1.70E-212	5.41E-211	cyan	0.658952059	6.52E-21	GO:0005975,GO:0004650	AT3G07840.1		Pectin lyase-like superfamily protein	NA
Sapur.019G054500.1	2707.52804	9.397809959	0.304715504	30.84126	7.34E-209	2.30E-207	cyan	0.668715337	1.07E-21	GO:0005975,GO:0004650	AT3G07820.1		Pectin lyase-like superfamily protein	polygalacturonase
Sapur.011G034900.1	1658.542582	2.363548954	0.076637645	30.84057	7.49E-209	2.34E-207	cyan	0.79833804	5.80E-36	GO:0006629	AT4G04930.1	DES-1-LIKE	fatty acid desaturase family protein	sphingolipid Delta4-desaturase
Sapur.006G153000.1	756.5258166	7.806088554	0.254333604	30.69232	7.21E-207	2.23E-205	cyan	0.634109016	4.89E-19	GO:0008081,GO:0006629,GO:0008889	AT5G58050.1	SVL4	SHV3-like 4	glycerophosphoryl diester phosphodiesterase family protein
Sapur.003G100100.1	1283.579434	4.007353367	0.13057382	30.69033	7.66E-207	2.37E-205	cyan	0.713006473	1.13E-25		AT3G61640.1	AGP20,AtAGP20	arabinogalactan protein 20	transmembrane protein, putative
Sapur.019G085900.1	115.0095728	8.585047205	0.279863735	30.67581	1.20E-206	3.69E-205	cyan	0.694630387	6.15E-24		AT2G42560.1		late embryogenesis abundant domain-containing protein / LEA domain-containing protein	late embryogenesis abundant protein
Sapur.005G167900.1	1566.145237	8.715237929	0.28424034	30.66151	1.86E-206	5.72E-205	cyan	0.723044324	1.11E-26					NA
Sapur.012G099900.1	183.82936	4.26218494	0.139013456	30.66023	1.93E-206	5.94E-205	cyan	0.753813484	4.55E-30	GO:0060236,GO:0032147,GO:0005874,GO:0005819	AT4G22860.1		Cell cycle regulated microtubule associated protein	cell cycle regulated microtubule-associated protein
Sapur.002G006400.1	79.25397718	8.252943108	0.26919142	30.65827	2.05E-206	6.30E-205	cyan	0.746860585	2.92E-29	GO:0016567,GO:0004842,GO:0005515	AT5G42340.1	PUB15	Plant U-Box 15	spotted leaf protein, putative
Sapur.019G066900.1	475.0086605	9.564468964	0.312243597	30.63143	4.67E-206	1.43E-204	cyan	0.63396189	5.01E-19		AT3G07830.1		Pectin lyase-like superfamily protein	NA
Sapur.016G310700.1	1148.949501	8.409724583	0.274559725	30.62986	4.90E-206	1.50E-204	cyan	0.659005472	6.45E-21		AT1G58110.2		Basic-leucine zipper (bZIP) transcription factor family protein	BZIP transcription factor, putative
Sapur.005G060000.1	93.95243174	7.330121245	0.239373011	30.62217	6.20E-206	1.90E-204	cyan	0.721032907	1.78E-26		AT2G22180.1		hydroxyproline-rich glycoprotein family protein	transmembrane protein, putative
Sapur.018G026200.1	280.126673	5.243033076	0.17123925	30.61817	7.01E-206	2.15E-204	cyan	0.656341819	1.05E-20		AT4G30380.1		Barwin-related endoglucanase	rare lipoprotein A-like double-psi beta-barrel protein
Sapur.15ZG048000.1	207.4650839	8.473845665	0.277459272	30.54086	7.48E-205	2.27E-203	cyan	0.695090755	5.58E-24		AT1G68490.1			hypothetical protein
Sapur.017G063000.1	131.1495988	5.068183982	0.166265822	30.48242	4.46E-204	1.35E-202	cyan	0.713364797	1.04E-25		AT1G27100.1		Actin cross-linking protein	actin cross-linking protein, putative
Sapur.014G028700.1	779.4725255	6.81356173	0.223621458	30.46918	6.68E-204	2.01E-202	cyan	0.641604887	1.38E-19	GO:0005515	AT2G26490.1		Transducin/WD40 repeat-like superfamily protein	F-box and WD40 domain protein, putative
Sapur.010G165400.1	436.6265589	8.300838939	0.272471359	30.465	7.58E-204	2.29E-202	cyan	0.63145601	7.57E-19	GO:0004857	AT5G64620.1	ATC/VIF2,C/VIF2	cell wall / vacuolar inhibitor of fructosidase 2	pectinesterase inhibitor
Sapur.002G069400.1	956.374816	9.34442024	0.30785105	30.35371	2.24E-202	6.73E-201	cyan	0.643212076	1.05E-19	GO:0055114,GO:0016491	AT5G08640.1	ATFLS1,FLS,FLS1	flavonol synthase 1	flavonol synthase/flavanone 3-hydroxylase
Sapur.016G065400.1	67.91748231	8.449893215	0.279065161	30.27928	2.15E-201	6.41E-200	cyan	0.750246228	1.19E-29		AT3G52460.1		hydroxyproline-rich glycoprotein family protein	transmembrane protein, putative
Sapur.011G041200.1	69.62998232	5.664421181	0.187109682	30.27327	2.58E-201	7.67E-200	cyan	0.669952113	8.43E-22		AT5G46060.1		Protein of unknown function, DUF599	NBS-LRR type disease resistance protein
Sapur.016G159800.1	452.612517	7.992158765	0.264008347	30.27237	2.65E-201	7.87E-200	cyan	0.693745485	7.40E-24	GO:0006468,GO:0004672	AT5G12180.1	CPK17	calcium-dependent protein kinase 17	calcium-dependent kinase
Sapur.011G071800.1	89.10127851	9.559215991	0.315978405	30.25275	4.80E-201	1.43E-199	cyan	0.699193792	2.34E-24		AT5G55980.1		serine-rich protein-related	hypothetical protein
Sapur.001G146400.1	540.7314243	3.676035202	0.121643314	30.21979	1.30E-200	3.85E-199	cyan	0.70209076	1.26E-24		AT1G80160.1		Lactoylglutathione lyase / glyoxalase I family protein	lactoylglutathione lyase, putative
Sapur.006G139200.1	54.18785578	8.202351137	0.271653648	30.19415	2.83E-200	8.35E-199	cyan	0.776055857	7.64E-33		AT5G12060.1		Plant self-incompatibility protein S1 family	self-incompatibility protein, putative
Sapur.001G008800.1	296.4778286	4.64647445	0.154565994	30.06143	1.55E-198	4.53E-197	cyan	0.68907645	1.94E-23		AT5G06700.1	TBR	Plant protein of unknown function (DUF828)	NA
Sapur.018G018100.1	515.0211101	2.919247318	0.097139591	30.05209	2.05E-198	5.99E-197	cyan	0.771421113	3.07E-32		AT5G25170.1		PPPDE putative thiol peptidase family protein	PPPDE peptidase domain protein, putative
Sapur.012G041400.1	1466.814928	1.973076971	0.065688499	30.03687	3.24E-198	9.44E-197	cyan	0.82510068	2.85E-40	GO:0006468,GO:0005524,GO:0004672	AT5G08160.1	ATPK3,PK3	serine/threonine protein kinase 3	Serine/Threonine kinase
Sapur.001G175200.1	46.71608081	8.06555575	0.26866374	30.02101	5.22E-198	1.52E-196	cyan	0.788618151	1.48E-34		AT1G11040.1		HSP40/DnaJ peptide-binding protein	NA
Sapur.002G127100.1	880.3358539	2.157768583	0.072095785	29.92919	8.21E-197	2.38E-195	cyan	0.823913561	4.58E-40	GO:0005515	AT1G63220.1		Calcium-dependent lipid-binding (CaLB domain) family protein	elicitor-responsive protein
Sapur.006G176600.1	810.6673124	7.797933838	0.261039068	29.87267	4.46E-196	1.29E-194	cyan	0.694989618	5.70E-24		AT1G01310.1		CAP (Cysteine-rich secretory proteins, Antigen 5, and Pathogenesis-related 1 protein) superfamily protein	cysteine-rich secretory family protein
Sapur.002G012700.1	74.68344541	5.035929642	0.169044791	29.7905	5.19E-195	1.48E-193	cyan	0.626856157	1.60E-18		AT1G20180.1		Protein of unknown function (DUF677)	NA
Sapur.017G050400.1	813.2407777	8.654329691	0.290859688	29.75431	1.53E-194	4.36E-193	cyan	0.60123032	8.38E-17					NA
Sapur.018G000200.1	3067.086882	2.449338497	0.082782317	29.5877	2.15E-192	6.05E-191	cyan	0.786033503	3.41E-34		AT4G31130.1		Protein of unknown function (DUF1218)	transmembrane protein, putative
Sapur.016G006700.1	115.703306	8.804371998	0.298977909	29.44824	1.33E-190	3.70E-189	cyan	0.671759053	5.98E-22		AT5G55630.2	ATKCO1,ATTPK1,KCO1,TPK1	Outward rectifying potassium channel protein	outward rectifying potassium channel protein
Sapur.012G056100.1	1409.485484	8.01672929	0.272259661	29.44516	1.45E-190	4.04E-189	cyan	0.692421651	9.74E-24	GO:0055114,GO:0020037,GO:0006979,GO:0004601	AT4G17690.1		Peroxidase superfamily protein	peroxidase
Sapur.010G006400.1	4819.242069	8.363475493	0.28450862	29.39621	6.14E-190	1.70E-188	cyan	0.683395133	6.14E-23	GO:0004857,GO:0042545,GO:0030599,GO:0005618	AT5G27870.1		Plant invertase/pectin methylesterase inhibitor superfamily	pectinesterase/pectinesterase inhibitor, putative
Sapur.003G036500.1	149.2291472	2.713010039	0.092426101	29.35329	2.17E-189	6.00E-188	cyan	0.787372644	2.21E-34		AT1G72600.2		hydroxyproline-rich glycoprotein family protein	transmembrane protein, putative
Sapur.018G062100.1	2995.865015	5.115107067	0.1743686	29.33502	3.71E-189	1.02E-187	cyan	0.713499809	1.01E-25	GO:0055085,GO:0016021	AT1G77210.2	AtSTP14,STP14	sugar transporter 14	sugar transporter, putative
Sapur.008G120100.1	3690.127178	8.17133114	0.279131349	29.27414	2.21E-188	6.07E-187	cyan	0.698831322	2.53E-24	GO:0030570	AT5G15110.1		Pectate lyase family protein	pectate lyase
Sapur.006G049100.1	835.2460373	9.931930861	0.339473275	29.25689	3.67E-188	1.00E-186	cyan	0.561559548	1.99E-14		AT3G48675.1		Protein of unknown function (DUF1278)	NA
Sapur.006G111400.1	2855.703811	8.39549154	0.287008178	29.25175	4.27E-188	1.17E-186	cyan	0.698776098	2.56E-24	GO:0004857,GO:0042545,GO:0030599,GO:0005618	AT2G26450.1		Plant invertase/pectin methylesterase inhibitor superfamily	pectinesterase/pectinesterase inhibitor, putative
Sapur.019G067400.1	2783.907274	9.33370633	0.319656343	29.19919	1.99E-187	5.38E-186	cyan	0.641917933	1.31E-19	GO:0005975,GO:0004650	AT3G07820.1		Pectin lyase-like superfamily protein	NA
Sapur.017G002800.1	738.562902	4.612147494	0.15838848	29.11921	2.05E-186	5.51E-185	cyan	0.708496853	3.09E-25		AT2G43900.1		Endonuclease/exonuclease/phosphatase family protein	type I inositol 1%2C4%2C5-trisphosphate 5-phosphatase
Sapur.010G163400.1	32.26356448	7.749470418	0.266443258	29.08488	5.58E-186	1.50E-184	cyan	0.77154775	2.96E-32	GO:0005515	AT4G12560.1	CPR30	F-box and associated interaction domains-containing protein	F-box protein interaction domain protein
Sapur.006G126200.1	713.6604267	7.332278608	0.252253551	29.0671	9.36E-186	2.50E-184	cyan	0.610522932	2.08E-17	GO:0055114,GO:0050660,GO:0016491,GO:0019139,GO:0009690	AT5G56970.1	ATCKX3,CKX3	cytokinin oxidase 3	cytokinin oxidase/dehydrogenase
Sapur.007G107000.1	203.9673709	3.681709776	0.126674679	29.06429	1.02E-185	2.71E-184	cyan	0.716187746	5.46E-26		AT5G54650.2	ATFH5,Fh5	formin homology5	NA
Sapur.019G072900.1	628.3346356	9.827974031	0.338340997	29.04754	1.65E-185	4.40E-184	cyan	0.589645826	4.48E-16	GO:0005975,GO:0004650	AT3G07820.1		Pectin lyase-like superfamily protein	NA
Sapur.016G048300.1	259.8419555	3.833633576	0.132259973	28.98559	1.00E-184	2.63E-183	cyan	0.676997309	2.17E-22		AT3G21550.1	AtDMP2,DMP2	DUF679 domain membrane protein 2	plant/MIL23-11 protein
Sapur.009G094500.1	230.6643233	7.387025324	0.254946455	28.97481	1.37E-184	3.59E-183	cyan	0.626713479	1.64E-18		AT2G21540.2	ATSFH3,SFH3	SEC14-like 3	SEC14 cytosolic factor, putative
Sapur.019G051700.1	243.2394941	3.341608774	0.115470771	28.939	3.86E-184	1.01E-182	cyan	0.726809188	4.52E-27		AT5G16490.1	RIC4	ROP-interactive CRIB motif-containing protein 4	wiscott-aldrich syndrome, carboxy-terminal protein%2C putative
Sapur.009G084900.1	3622.386664	8.123328625	0.281248825	28.88307	1.95E-183	5.05E-182	cyan	0.637632503	2.71E-19	GO:0005515	AT3G19020.1		Leucine-rich repeat (LRR) family protein	NA
Sapur.018G005000.1	47.91395837	4.044170022	0.140145522	28.85693	4.15E-183	1.07E-181	cyan	0.701201152	1.52E-24	GO:0008270,GO:0005515	AT5G24870.2		RING/U-box superfamily protein	zinc finger protein, putative
Sapur.008G147500.1	1532.442979	8.162582985	0.283221109	28.82053	1.19E-182	3.06E-181	cyan	0.714151626	8.69E-26		AT1G24520.1	BCP1	homolog of Brassica campestris pollen protein 1	transmembrane protein, putative
Sapur.010G056700.1	115.2079775	7.444123206	0.258366863	28.81222	1.51E-182	3.89E-181	cyan	0.661650842	3.98E-21		AT1G67670.1			hypothetical protein
Sapur.018G038700.1	2074.883045	8.285495203	0.287718038	28.79727	2.32E-182	5.97E-181	cyan	0.706669536	4.63E-25	GO:0004857,GO:0042545,GO:0030599,GO:0005618	AT2G26450.1		Plant invertase/pectin methylesterase inhibitor superfamily	pectinesterase/pectinesterase inhibitor, putative
Sapur.019G029400.1	579.5832435	8.171790846	0.28391579	28.78245	3.56E-182	9.13E-181	cyan	0.677001303	2.17E-22	GO:0005515	AT2G33320.1		Calcium-dependent lipid-binding (CaLB domain) family protein	calcium-dependent lipid-binding (CaLB domain) family protein, putative
Sapur.008G140200.1	97.15763064	6.840074697	0.23800333	28.73941	1.23E-181	3.13E-180	cyan	0.675587286	2.86E-22	GO:0005515	AT2G33320.1		Calcium-dependent lipid-binding (CaLB domain) family protein	calcium-dependent lipid-binding domain protein, putative
Sapur.005G015200.1	527.1164838	4.336110612	0.150922216	28.73076	1.58E-181	4.01E-180	cyan	0.727515932	3.81E-27	GO:0005515,GO:0008017,GO:0007018,GO:0005524,GO:0003777,GO:0005871	AT5G27000.1	ATK4,KATD	kinesin 4	kinesin heavy chain, putative
Sapur.019G067200.1	1505.453195	9.138544225	0.318326997	28.70804	3.03E-181	7.70E-180	cyan	0.633685146	5.24E-19	GO:0005975,GO:0004650	AT3G07820.1		Pectin lyase-like superfamily protein	NA
Sapur.009G065200.1	934.1340363	8.553726141	0.298348519	28.67025	8.97E-181	2.26E-179	cyan	0.64274029	1.14E-19		AT2G14610.1	ATPR1,PR 1,PR1	pathogenesis-related gene 1	pathogenesis-related protein
Sapur.010G014500.1	277.025777	5.479282727	0.191204674	28.65664	1.33E-180	3.34E-179	cyan	0.66832059	1.15E-21	GO:0006468,GO:0004672	AT5G18910.1		Protein kinase superfamily protein	receptor tyrosine kinase
Sapur.002G187000.1	77.4566565	8.301252744	0.289984057	28.62658	3.14E-180	7.90E-179	cyan	0.728557119	2.96E-27	GO:0006468,GO:0005524,GO:0004672	AT3G07070.1		Protein kinase superfamily protein	Serine/Threonine kinase domain protein
Sapur.012G069000.1	535.8429042	5.266099542	0.184480387	28.54558	3.19E-179	7.97E-178	cyan	0.717885318	3.69E-26		AT2G04410.1		RPM1-interacting protein 4 (RIN4) family protein	pathogenic type III effector avirulence factor Avr AvrRpt-cleavage: cleavage site protein
Sapur.016G104700.1	545.9679116	2.402951207	0.084198771	28.53903	3.84E-179	9.59E-178	cyan	0.777424642	5.03E-33		AT3G08970.1	ATERDJ3A,TMS1	DNAJ heat shock N-terminal domain-containing protein	heat shock protein-binding protein, putative
Sapur.008G121700.1	4321.5577	8.208932907	0.288262559	28.47728	2.24E-178	5.57E-177	cyan	0.665654645	1.89E-21	GO:0055085,GO:0016021	AT5G26250.1		Major facilitator superfamily protein	NA
Sapur.003G159100.1	257.040191	8.450373452	0.297227252	28.43068	8.45E-178	2.09E-176	cyan	0.649051445	3.82E-20		AT5G12930.1			hypothetical protein
Sapur.001G115500.1	1184.905333	3.446470638	0.121376993	28.39476	2.35E-177	5.76E-176	cyan	0.692848203	8.92E-24	GO:0016887,GO:0005524,GO:0055085,GO:0042626,GO:0016021,GO:0006810	AT4G25960.1	PGP2	P-glycoprotein 2	multidrug/pheromone exporter, MDR family, ABC transporter family protein
Sapur.011G055000.1	116.6222291	8.503522241	0.300914429	28.25894	1.11E-175	2.66E-174	cyan	0.658910149	6.57E-21					transmembrane protein, putative
Sapur.006G167100.1	597.4887112	6.278945167	0.222308805	28.24425	1.67E-175	4.03E-174	cyan	0.723446935	1.01E-26		AT3G52460.1		hydroxyproline-rich glycoprotein family protein	transmembrane protein, putative
Sapur.008G112900.1	117.7148319	7.633663904	0.270867904	28.18224	9.65E-175	2.31E-173	cyan	0.680074025	1.19E-22	GO:0005544,GO:0005509	AT1G68090.1	ANN5,ANNAT5	annexin 5	annexin
Sapur.006G204200.1	181.4557019	3.528291372	0.125507448	28.11221	6.95E-174	1.66E-172	cyan	0.766278422	1.38E-31		AT5G25530.1		DNAJ heat shock family protein	DnaJ family B protein
Sapur.010G053900.1	1876.861654	7.420495471	0.264252676	28.08106	1.67E-173	3.96E-172	cyan	0.60220891	7.25E-17		AT5G40780.1	LHT1	lysine histidine transporter 1	NA
Sapur.017G054100.1	1505.064411	10.23109591	0.365143025	28.01942	9.42E-173	2.23E-171	cyan	0.494420669	4.67E-11	GO:0005975,GO:0004650	AT3G07820.1		Pectin lyase-like superfamily protein	NA
Sapur.001G173000.1	6857.834356	8.260178872	0.295553053	27.94821	6.93E-172	1.63E-170	cyan	0.701560149	1.41E-24	GO:0004857,GO:0042545,GO:0030599,GO:0005618	AT5G49180.1		Plant invertase/pectin methylesterase inhibitor superfamily	pectinesterase/pectinesterase inhibitor, putative
Sapur.006G040300.1	180.5976937	9.509928062	0.340424594	27.93549	9.89E-172	2.32E-170	cyan	0.603897171	5.65E-17	GO:0005975,GO:0004650	AT3G07970.1	QRT2	Pectin lyase-like superfamily protein	polygalacturonase
Sapur.014G095400.1	434.3703594	8.035269602	0.287915743	27.90841	2.11E-171	4.93E-170	cyan	0.659769382	5.61E-21	GO:0004857	AT1G02550.1		Plant invertase/pectin methylesterase inhibitor superfamily protein	NA
Sapur.016G235600.1	4634.277526	8.415625644	0.302337201	27.83523	1.63E-170	3.77E-169	cyan	0.668508121	1.11E-21	GO:0055085	AT4G26590.1	ATOPT5,OPT5	oligopeptide transporter 5	oligopeptide transporter OPT family protein
Sapur.019G067000.1	1784.414155	8.64143519	0.310648876	27.81737	2.67E-170	6.19E-169	cyan	0.582697334	1.18E-15	GO:0005975,GO:0004650	AT3G07820.1		Pectin lyase-like superfamily protein	NA
Sapur.019G087500.1	1803.52979	7.043375319	0.253222048	27.81502	2.86E-170	6.60E-169	cyan	0.663931385	2.61E-21		AT1G23145.1	RALFL2	RALF-like 2	rapid aLkalinization factor
Sapur.010G083700.1	643.5916477	4.078840148	0.146668903	27.80985	3.30E-170	7.62E-169	cyan	0.681226047	9.45E-23	GO:0005515	AT1G70790.1		Calcium-dependent lipid-binding (CaLB domain) family protein	Arf GTPase activator, putative
Sapur.008G096200.1	233.4938814	3.706743984	0.133332876	27.80068	4.26E-170	9.83E-169	cyan	0.720774354	1.89E-26	GO:0051861,GO:0046836,GO:0017089,GO:0005737	AT4G39670.1		Glycolipid transfer protein (GLTP) family protein	glycolipid transfer protein
Sapur.15WG087100.1	97.16194501	5.790100627	0.208342278	27.79129	5.53E-170	1.27E-168	cyan	0.707928257	3.51E-25		AT1G74830.1		Protein of unknown function, DUF593	zein-binding protein
Sapur.005G198800.1	515.563319	1.801647787	0.06507878	27.68411	1.08E-168	2.47E-167	cyan	0.822183664	9.09E-40		AT1G20460.1			NA
Sapur.016G189800.1	106.1127928	9.101251646	0.328813004	27.67911	1.25E-168	2.83E-167	cyan	0.660321301	5.08E-21		AT5G09430.1		alpha/beta-Hydrolases superfamily protein	alpha/beta-hydrolase domain protein
Sapur.005G017000.1	4125.528164	8.141843089	0.294867972	27.61183	8.02E-168	1.81E-166	cyan	0.663981332	2.59E-21	GO:0004857	AT5G46930.1		Plant invertase/pectin methylesterase inhibitor superfamily protein	plant invertase/pectin methylesterase inhibitor protein
Sapur.001G040300.1	544.6020214	3.894964312	0.141309159	27.56342	3.06E-167	6.83E-166	cyan	0.800865859	2.42E-36		AT2G33100.1	ATCSLD1,CSLD1	cellulose synthase-like D1	cellulose synthase-like protein D
Sapur.008G121800.1	5120.691213	8.164925116	0.29682654	27.5074	1.43E-166	3.18E-165	cyan	0.658943342	6.53E-21	GO:0055085,GO:0016021	AT5G26250.1		Major facilitator superfamily protein	NA
Sapur.009G091100.1	228.6258668	7.482853318	0.272251154	27.48511	2.65E-166	5.87E-165	cyan	0.622436868	3.25E-18	GO:0006468,GO:0004672	AT1G49270.1		Protein kinase superfamily protein	proline-rich receptor-like kinase
Sapur.016G305400.1	70.73450479	3.769452238	0.137392108	27.43573	1.03E-165	2.28E-164	cyan	0.77523276	9.80E-33		AT2G04220.1		Plant protein of unknown function (DUF868)	kinesin-4 protein, putative
Sapur.016G094300.1	146.7465623	3.452939466	0.125915511	27.42267	1.47E-165	3.25E-164	cyan	0.694020606	6.98E-24		AT3G54040.1		PAR1 protein	PAR1 protein
Sapur.007G008900.1	2253.781013	2.942790124	0.107354166	27.41198	1.97E-165	4.35E-164	cyan	0.726264637	5.15E-27	GO:0006094,GO:0005524,GO:0004612	AT4G37870.1	PCK1,PEPCK	phosphoenolpyruvate carboxykinase 1	phosphoenolpyruvate carboxykinase (ATP)
Sapur.003G093900.1	1025.997503	7.322361892	0.267245447	27.39939	2.79E-165	6.15E-164	cyan	0.579225305	1.91E-15	GO:0006468,GO:0005524,GO:0004672	AT5G55090.1	MAPKKK15	mitogen-activated protein kinase kinase kinase 15	Serine/Threonine kinase family protein
Sapur.002G204100.1	43.80283826	8.382204306	0.306076714	27.38596	4.03E-165	8.86E-164	cyan	0.736678876	4.00E-28		AT1G75160.1		Protein of unknown function (DUF620)	NA
Sapur.011G071200.1	57.42527414	6.298149173	0.230082916	27.37339	5.69E-165	1.25E-163	cyan	0.713561509	9.94E-26	GO:0055114,GO:0016491	AT1G78440.1	ATGA2OX1,GA2OX1	Arabidopsis thaliana gibberellin 2-oxidase 1	gibberellin 2-oxidase
Sapur.002G065200.1	89.50207971	6.425392506	0.234853296	27.35918	8.40E-165	1.84E-163	cyan	0.621239299	3.94E-18	GO:0055114,GO:0016491,GO:0008152	AT1G44170.2	ALDH3H1,ALDH4	aldehyde dehydrogenase 3H1	aldehyde dehydrogenase (NAD) family protein
Sapur.002G032900.1	91.71176603	8.348461593	0.305448514	27.33181	1.78E-164	3.89E-163	cyan	0.724010991	8.81E-27	GO:0016301,GO:0005524	AT2G30630.1		P-loop containing nucleoside triphosphate hydrolases superfamily protein	NA
Sapur.009G090200.1	29.13309338	7.598318434	0.278322287	27.30043	4.19E-164	9.15E-163	cyan	0.726776927	4.55E-27		AT1G49330.1		hydroxyproline-rich glycoprotein family protein	hypothetical protein
Sapur.019G045600.1	46.03896724	7.864176435	0.288096953	27.29698	4.61E-164	1.01E-162	cyan	0.749977802	1.28E-29		AT5G17590.1		Putative membrane lipoprotein	transmembrane protein, putative
Sapur.004G016800.1	923.9984975	4.555683424	0.167270955	27.23535	2.48E-163	5.39E-162	cyan	0.675066774	3.16E-22		AT5G54490.1	PBP1	pinoid-binding protein 1	centrin protein
Sapur.016G054300.1	634.6119453	5.007249211	0.184939145	27.07512	1.93E-161	4.13E-160	cyan	0.555723684	4.20E-14	GO:0016758,GO:0008152	AT2G15490.1	UGT73B4	UDP-glycosyltransferase 73B4	trans-zeatin O-beta-D-glucosyltransferase
Sapur.017G054000.1	1525.862432	8.7984813	0.325152625	27.05954	2.95E-161	6.27E-160	cyan	0.497036115	3.56E-11	GO:0005975,GO:0004650	AT3G07840.1		Pectin lyase-like superfamily protein	NA
Sapur.15ZG040000.1	51.29677132	8.08918631	0.299926221	26.97059	3.27E-160	6.92E-159	cyan	0.660185479	5.20E-21	GO:0016021	AT1G18520.1	TET11	tetraspanin11	tetraspanin family protein
Sapur.004G087600.1	3407.41115	8.096696503	0.300215073	26.96965	3.36E-160	7.09E-159	cyan	0.641034057	1.53E-19	GO:0030570	AT5G15110.1		Pectate lyase family protein	pectate lyase, putative
Sapur.002G168500.1	258.5142015	4.41408422	0.16369524	26.96526	3.78E-160	7.98E-159	cyan	0.627466481	1.45E-18	GO:0016887,GO:0005524,GO:0016020	AT3G53480.1	ABCG37,ATPDR9,PDR9,PIS1	pleiotropic drug resistance 9	ABC transporter G family protein
Sapur.017G029500.1	125.1428486	3.866224217	0.143833543	26.87985	3.78E-159	7.90E-158	cyan	0.675911134	2.68E-22	GO:0016757,GO:0016020	AT1G05280.1		Protein of unknown function (DUF604)	fringe-like protein
Sapur.003G012700.1	8086.634811	8.0711859	0.301116877	26.80416	2.89E-158	6.00E-157	cyan	0.676520542	2.38E-22	GO:0004857,GO:0042545,GO:0030599,GO:0005618	AT5G27870.1		Plant invertase/pectin methylesterase inhibitor superfamily	pectinesterase/pectinesterase inhibitor, putative
Sapur.019G098100.1	2141.226607	7.994691739	0.298773117	26.7584	9.86E-158	2.04E-156	cyan	0.68989051	1.64E-23		AT1G60815.1	RALFL7	RALF-like 7	rapid aLkalinization factor
Sapur.006G180400.1	102.2352334	2.768772536	0.103530328	26.74359	1.47E-157	3.03E-156	cyan	0.7878759	1.88E-34		AT4G33180.1		alpha/beta-Hydrolases superfamily protein	NA
Sapur.008G083000.1	1283.98063	8.394132038	0.318084355	26.38964	1.80E-153	3.64E-152	cyan	0.615286525	1.00E-17	GO:0042545,GO:0030599,GO:0005618	AT3G17060.1		Pectin lyase-like superfamily protein	pectinesterase
Sapur.018G061100.1	3339.287371	7.873111876	0.299590105	26.27961	3.28E-152	6.54E-151	cyan	0.635513752	3.87E-19		AT5G19580.1		glyoxal oxidase-related protein	glyoxal oxidase
Sapur.012G072000.1	563.1251815	7.936246625	0.302002914	26.27871	3.36E-152	6.69E-151	cyan	0.651187642	2.62E-20		AT1G24520.1	BCP1	homolog of Brassica campestris pollen protein 1	transmembrane protein, putative
Sapur.018G038600.1	791.5357048	1.563967291	0.059596792	26.24247	8.71E-152	1.73E-150	cyan	0.839405648	6.88E-43		AT5G35460.1			plant/MOK9-4 protein
Sapur.010G183800.1	407.773024	5.372701405	0.204756185	26.23951	9.42E-152	1.87E-150	cyan	0.58781375	5.80E-16		AT5G04390.1		C2H2-type zinc finger family protein	C2H2-type zinc-finger protein, putative
Sapur.15WG010800.1	570.3853669	1.876220477	0.072038734	26.04461	1.55E-149	3.01E-148	cyan	0.814456399	1.78E-38	GO:0007275,GO:0006511,GO:0005634	AT4G27880.1		Protein with RING/U-box and TRAF-like domains	E3 ubiquitin-protein ligase SINAT4
Sapur.005G016800.1	4655.409526	7.98792425	0.307188446	26.00334	4.54E-149	8.79E-148	cyan	0.626691419	1.65E-18	GO:0004857,GO:0042545,GO:0030599,GO:0005618	AT3G05610.1		Plant invertase/pectin methylesterase inhibitor superfamily	pectinesterase/pectinesterase inhibitor, putative
Sapur.15WG153000.1	902.3738498	8.095388457	0.312344356	25.91815	4.16E-148	7.99E-147	cyan	0.616694306	8.04E-18		AT1G58110.2		Basic-leucine zipper (bZIP) transcription factor family protein	NA
Sapur.001G117700.1	1542.26778	1.141244205	0.044099984	25.87856	1.16E-147	2.22E-146	cyan	0.843284948	1.21E-43	GO:0005096	AT4G17890.1	AGD8	ARF-GAP domain 8	ADP-ribosylation factor GTPase-activating protein AGD10
Sapur.019G098200.1	1263.999342	7.831737917	0.302708523	25.87221	1.37E-147	2.62E-146	cyan	0.699874677	2.03E-24		AT1G60815.1	RALFL7	RALF-like 7	rapid aLkalinization factor
Sapur.006G114600.1	2368.38303	6.723268261	0.260133396	25.84546	2.74E-147	5.22E-146	cyan	0.528532726	1.12E-12	GO:0030246,GO:0005975,GO:0004553	AT5G20710.1	BGAL7	beta-galactosidase 7	beta-galactosidase
Sapur.011G090900.1	1858.213479	1.607703125	0.06221874	25.83953	3.19E-147	6.09E-146	cyan	0.807382245	2.41E-37	GO:0005096	AT5G54310.1	AGD5,NEV	ARF-GAP domain 5	ADP-ribosylation factor GTPase-activating protein AGD5, putative
Sapur.018G028400.1	302.4165041	2.706969802	0.105113408	25.75285	3.00E-146	5.67E-145	cyan	0.744475444	5.46E-29		AT5G25530.1		DNAJ heat shock family protein	DnaJ family B protein
Sapur.003G137500.1	1173.929261	1.138899784	0.044354578	25.67716	2.10E-145	3.96E-144	cyan	0.846246825	3.12E-44	GO:0005634	AT1G80780.1		Polynucleotidyl transferase, ribonuclease H-like superfamily protein	CCR4-associated factor, putative
Sapur.006G039700.1	1262.476772	2.526137638	0.098550169	25.63301	6.54E-145	1.23E-143	cyan	0.750171223	1.22E-29		AT2G41830.1		Uncharacterized protein	cyclin-like protein
Sapur.005G049400.1	1452.197725	2.863383956	0.11183386	25.60391	1.38E-144	2.58E-143	cyan	0.750001102	1.27E-29	GO:0008168	AT5G64030.1		S-adenosyl-L-methionine-dependent methyltransferases superfamily protein	methyltransferase PMT26, putative
Sapur.011G030500.1	918.0710207	1.229192513	0.048022204	25.59634	1.68E-144	3.13E-143	cyan	0.847389195	1.83E-44		AT1G61240.4		Protein of unknown function (DUF707)	lysine ketoglutarate reductase trans-splicing-like protein
Sapur.017G128500.1	94.42272675	5.49958499	0.215040985	25.57459	2.93E-144	5.46E-143	cyan	0.635485492	3.89E-19	GO:0006468,GO:0005524,GO:0004672	AT1G48480.1	RKL1	receptor-like kinase 1	receptor kinase
Sapur.005G042700.1	714.7186346	8.039251095	0.314509849	25.56121	4.12E-144	7.67E-143	cyan	0.647786318	4.77E-20		AT1G08790.1		Protein of unknown function (DUF1685)	DUF1685 family protein
Sapur.008G170100.1	863.5104136	3.059561305	0.120104122	25.47424	3.80E-143	7.02E-142	cyan	0.74605508	3.61E-29		AT5G18980.1		ARM repeat superfamily protein	armadillo/beta-catenin-like repeat protein
Sapur.011G053800.1	801.450362	7.866655719	0.3092864	25.43486	1.04E-142	1.91E-141	cyan	0.62973711	1.00E-18	GO:0048046,GO:0016762,GO:0006073,GO:0005618,GO:0005975,GO:0004553	AT4G30270.1	MERI-5,MERI5B,SEN4,XTH24	xyloglucan endotransglucosylase/hydrolase 24	xyloglucan endotransglycosylase/hydrolase
Sapur.001G106200.1	34.51851141	5.252906384	0.207116052	25.3628	6.49E-142	1.19E-140	cyan	0.716615715	4.95E-26		AT4G12560.2	CPR30	F-box and associated interaction domains-containing protein	cytochrome P450 family protein
Sapur.004G109900.1	1825.613692	7.806365482	0.308129945	25.33465	1.33E-141	2.42E-140	cyan	0.628662665	1.20E-18	GO:0005515	AT2G15880.1		Leucine-rich repeat (LRR) family protein	NA
Sapur.018G064500.1	1025.851089	1.690365438	0.066894392	25.26917	6.97E-141	1.26E-139	cyan	0.804774897	6.14E-37	GO:0016757	AT2G19880.2		Nucleotide-diphospho-sugar transferases superfamily protein	ceramide glucosyltransferase
Sapur.017G114200.1	261.5530789	4.486277064	0.177540552	25.26903	7.00E-141	1.27E-139	cyan	0.574191181	3.78E-15	GO:0005515	AT5G17220.1	ATGSTF12,GST26,GSTF12,TT19	glutathione S-transferase phi 12	glutathione S-transferase
Sapur.003G141400.1	929.8320751	1.640201996	0.065063081	25.20941	3.16E-140	5.68E-139	cyan	0.834252233	6.44E-42		AT1G79660.1			plant/F20B17-9 protein
Sapur.003G151800.1	431.0698967	3.2676668	0.129723625	25.18945	5.23E-140	9.40E-139	cyan	0.73694282	3.75E-28	GO:0046488,GO:0016307	AT3G07960.1		Phosphatidylinositol-4-phosphate 5-kinase family protein	phosphatidylinositol-4-phosphate 5-kinase
Sapur.007G082700.1	255.9876955	7.700201817	0.305852066	25.17623	7.30E-140	1.31E-138	cyan	0.65618806	1.07E-20		AT2G22180.1		hydroxyproline-rich glycoprotein family protein	late embryogenesis abundant protein
Sapur.017G117800.1	52.04063176	4.001842853	0.159702417	25.05812	1.42E-138	2.51E-137	cyan	0.693924356	7.13E-24	GO:0005515,GO:0006468,GO:0005524,GO:0004672	AT3G47570.1		Leucine-rich repeat protein kinase family protein	NA
Sapur.15WG017700.1	709.2612899	7.77204859	0.311314019	24.9653	1.46E-137	2.54E-136	cyan	0.636389714	3.34E-19		AT3G61640.1	AGP20,AtAGP20	arabinogalactan protein 20	arabinogalactan peptide protein
Sapur.005G016100.1	1166.784014	7.549079774	0.302745612	24.93539	3.08E-137	5.36E-136	cyan	0.636821732	3.11E-19	GO:0004857				plant invertase/pectin methylesterase inhibitor protein
Sapur.019G017800.1	49.99934901	5.82906449	0.234744996	24.83147	4.10E-136	7.08E-135	cyan	0.601380412	8.20E-17	GO:0006468,GO:0004672	AT1G07650.2		Leucine-rich repeat transmembrane protein kinase	NA
Sapur.014G049800.1	910.629664	2.570874254	0.10354453	24.82868	4.39E-136	7.58E-135	cyan	0.72207318	1.39E-26	GO:0055114,GO:0016616,GO:0006694,GO:0003854	AT4G00110.1	GAE3	UDP-D-glucuronate 4-epimerase 3	UDP-D-glucuronate 4-epimerase
Sapur.016G266700.1	332.5575877	7.087156331	0.28780982	24.62444	6.91E-134	1.17E-132	cyan	0.535005336	5.27E-13		AT5G08240.1			transmembrane protein, putative
Sapur.004G040400.1	555.8515295	2.155617837	0.087780094	24.55702	3.64E-133	6.11E-132	cyan	0.780268685	2.10E-33	GO:0007165	AT4G18700.1	ATWL4,CIPK12,SnRK3.9,WL4	CBL-interacting protein kinase 12	CBL-interacting Serine/Threonine-kinase
Sapur.007G101400.1	2092.307967	3.176281544	0.129754277	24.47921	2.46E-132	4.10E-131	cyan	0.661308899	4.24E-21		AT1G58120.1			transmembrane protein, putative
Sapur.007G100000.1	142.9404941	5.97786206	0.244296366	24.46971	3.10E-132	5.17E-131	cyan	0.560829791	2.19E-14	GO:0055085,GO:0016021,GO:0015299,GO:0006812	AT1G05580.1	ATCHX23,CHX23	cation/H+ exchanger 23	cation proton exchanger
Sapur.010G042600.1	67.90582342	3.812605759	0.155881535	24.45835	4.10E-132	6.82E-131	cyan	0.694958656	5.74E-24		AT2G33460.1	RIC1	ROP-interactive CRIB motif-containing protein 1	P21-Rho-binding domain protein
Sapur.004G160700.1	86.25195978	7.992545751	0.328366329	24.34033	7.34E-131	1.21E-129	cyan	0.671685194	6.06E-22					NA
Sapur.013G011800.1	1290.883714	7.671639923	0.315846819	24.28912	2.55E-130	4.18E-129	cyan	0.638556019	2.32E-19	GO:0004857	AT5G50030.1		Plant invertase/pectin methylesterase inhibitor superfamily protein	plant invertase/pectin methylesterase inhibitor protein
Sapur.016G133500.1	186.6760495	3.90951569	0.161129106	24.26325	4.79E-130	7.83E-129	cyan	0.636158408	3.47E-19	GO:0016021,GO:0004252,GO:0006508	AT2G29050.1	ATRBL1,RBL1	RHOMBOID-like 1	rhomboid family protein
Sapur.013G012500.1	3038.831047	[illegible]	[illegible]	[illegible]	[illegible]	[illegible]	cyan	[illegible]	[illegible]	[illegible]	[illegible]		Plant invertase/pectin methylesterase inhibitor superfamily	pectinesterase/pectinesterase inhibitor, putative
Sapur.016G129700.1	[illegible]	[illegible]	[illegible]	[illegible]	[illegible]	[illegible]	cyan	[illegible]	[illegible]		[illegible]		Protein of unknown function (DUF679)	hypothetical protein
Sapur.010G108000.1	[illegible]	[illegible]	[illegible]	[illegible]	[illegible]	[illegible]	cyan	[illegible]	[illegible]					hypothetical protein
Sapur.005G179200.1	[illegible]	[illegible]	[illegible]	[illegible]	[illegible]	[illegible]	cyan	[illegible]	[illegible]		[illegible]		Sec14p-like phosphatidylinositol transfer family protein	NA
Sapur.010G041600.1	[illegible]	[illegible]	[illegible]	[illegible]	[illegible]	[illegible]	cyan	[illegible]	[illegible]		[illegible]		VQ motif-containing protein	VQ motif protein
Sapur.15WG111200.1	[illegible]	[illegible]	[illegible]	[illegible]	[illegible]	[illegible]	cyan	[illegible]	[illegible]		[illegible]	BCP1	homolog of Brassica campestris pollen protein 1	transmembrane protein, putative
Sapur.010G004800.1	[illegible]	[illegible]	[illegible]	[illegible]	[illegible]	[illegible]	cyan	[illegible]	[illegible]		[illegible]	ATSYP112,SYP112	syntaxin of plants 112	syntaxin, putative
Sapur.010G071900.1	[illegible]	[illegible]	[illegible]	[illegible]	[illegible]	[illegible]	cyan	[illegible]	[illegible]		[illegible]	ATACP5,ATPAP17,PAP17	purple acid phosphatase 17	[illegible]
[illegible]	[illegible]	[illegible]	[illegible]	[illegible]	[illegible]	[illegible]	cyan	[illegible]	[illegible]		[illegible]		Plant invertase/pectin methylesterase inhibitor superfamily protein	plant invertase/pectin methylesterase inhibitor protein
[illegible]	[illegible]	[illegible]	[illegible]	[illegible]	[illegible]	[illegible]	cyan	[illegible]	[illegible]		[illegible]	ALMT12,ATALMT12	aluminum-activated, malate transporter 12	aluminum activated malate transporter
[illegible]	[illegible]	[illegible]	[illegible]	[illegible]	[illegible]	[illegible]	cyan	[illegible]	[illegible]	[illegible]	[illegible]		Protein kinase protein with adenine nucleotide alpha hydrolases-like domain	Serine/Threonine kinase
[illegible]	[illegible]	[illegible]	[illegible]	[illegible]	[illegible]	[illegible]	cyan	[illegible]	[illegible]		[illegible]	TET11	tetraspanin11	tetraspanin family protein
[illegible]	[illegible]	[illegible]	[illegible]	[illegible]	[illegible]	[illegible]	cyan	[illegible]	[illegible]		[illegible]	RALFL2	RALF-like 2	[illegible]
[illegible]	[illegible]	[illegible]	[illegible]	[illegible]	[illegible]	[illegible]	cyan	[illegible]	[illegible]		[illegible]		AMP-dependent synthetase and ligase family protein	4-coumarate-CoA ligase
Sapur.T055800.1	[illegible]	[illegible]	[illegible]	[illegible]	[illegible]	[illegible]	cyan	[illegible]	[illegible]		[illegible]		[illegible]	NA
[illegible]	[illegible]	[illegible]	[illegible]	[illegible]	[illegible]	[illegible]	cyan	[illegible]	[illegible]		[illegible]	[illegible]	RAS 5	small GTPase family Rab protein
Sapur.T043000.1	[illegible]	[illegible]	[illegible]	[illegible]	[illegible]	[illegible]	cyan	[illegible]	[illegible]		[illegible]		Plant invertase/pectin methylesterase inhibitor superfamily	NA
[illegible]	[illegible]	[illegible]	[illegible]	[illegible]	[illegible]	[illegible]	cyan	[illegible]	[illegible]		[illegible]		Pectin lyase-like superfamily protein	glycoside hydrolase family 28 protein/polygalacturonase (pectinase) family protein
Sapur.008G078100.1	[illegible]	[illegible]	[illegible]	[illegible]	[illegible]	[illegible]	cyan	[illegible]	[illegible]	[illegible]	[illegible]	CYP89A6	[illegible]	cytochrome P450 family protein
Sapur.006G177400.1	[illegible]	[illegible]	[illegible]	[illegible]	[illegible]	[illegible]	cyan	[illegible]	[illegible]	[illegible]	[illegible]		Haloacid dehalogenase-like hydrolase (HAD) superfamily protein	5' nucleotidase, deoxy (pyrimidine)%2C cytosolic type C protein (NT5C)
[illegible]	[illegible]	[illegible]	[illegible]	[illegible]	[illegible]	[illegible]	cyan	[illegible]	[illegible]	[illegible]	[illegible]		Protein with RING/U-box and TRAF-like domains	[illegible]
[illegible]	[illegible]	[illegible]	[illegible]	[illegible]	[illegible]	[illegible]	cyan	[illegible]	[illegible]		[illegible]		[illegible]	nodulin-like protein
[illegible]	[illegible]	[illegible]	[illegible]	[illegible]	[illegible]	[illegible]	cyan	[illegible]	[illegible]		[illegible]		[illegible]	acid phosphatase/vanadium-dependent haloperoxidase-related protein
[illegible]	[illegible]	[illegible]	[illegible]	[illegible]	[illegible]	[illegible]	cyan	[illegible]	[illegible]		[illegible]		Nucleotide-diphospho-sugar transferase family protein	[illegible]
[illegible]	[illegible]	[illegible]	[illegible]	[illegible]	[illegible]	[illegible]	cyan	[illegible]	[illegible]	[illegible]	[illegible]		Leucine-rich repeat protein kinase family protein	[illegible]
[illegible]	[illegible]	[illegible]	[illegible]	[illegible]	[illegible]	[illegible]	cyan	[illegible]	[illegible]	[illegible]	[illegible]	CML42	calmodulin like 42	calcium-binding protein CML27, putative
[illegible]	[illegible]	[illegible]	[illegible]	[illegible]	[illegible]	[illegible]	cyan	[illegible]	[illegible]		[illegible]		Stigma-specific Stig1 family protein	[illegible]
[illegible]	[illegible]	[illegible]	[illegible]	[illegible]	[illegible]	[illegible]	cyan	[illegible]	[illegible]		[illegible]		Homeodomain-like superfamily protein	MYB transcription factor-like protein, putative
[illegible]	[illegible]	[illegible]	[illegible]	[illegible]	[illegible]	[illegible]	cyan	[illegible]	[illegible]	[illegible]	[illegible]		Galactose mutarotase-like superfamily protein	aldose 1-epimerase
[illegible]	[illegible]	[illegible]	[illegible]	[illegible]	[illegible]	[illegible]	cyan	[illegible]	[illegible]	[illegible]	[illegible]	[illegible]	SOS3-interacting protein 4	CBL-interacting Serine/Threonine-kinase
[illegible]	[illegible]	[illegible]	[illegible]	[illegible]	[illegible]	[illegible]	cyan	[illegible]	[illegible]	[illegible]	[illegible]	[illegible]	calcium-dependent protein kinase 32	calcium-dependent kinase
[illegible]	[illegible]	[illegible]	[illegible]	[illegible]	[illegible]	[illegible]	cyan	[illegible]	[illegible]	[illegible]	[illegible]	ATCHX4,CHX4	cation/H+ exchanger 4	cation proton exchanger
[illegible]	[illegible]	[illegible]	[illegible]	[illegible]	[illegible]	[illegible]	cyan	[illegible]	[illegible]	[illegible]	[illegible]	ATPTEN1,PTEN1	Calcium/lipid-binding (CaLB) phosphatase	phosphatidylinositol-3%2C4%2C5-trisphosphate 3-phosphatase
[illegible]	[illegible]	[illegible]	[illegible]	[illegible]	[illegible]	[illegible]	cyan	[illegible]	[illegible]	[illegible]	[illegible]	BGLU41	beta glucosidase 41	glycoside hydrolase family 1 protein
[illegible]	[illegible]	[illegible]	[illegible]	[illegible]	[illegible]	[illegible]	cyan	[illegible]	[illegible]	[illegible]	[illegible]	AtTudor1,TSN1,Tudor1	TUDOR-SN protein 1	tudor protein
[illegible]	[illegible]	[illegible]	[illegible]	[illegible]	[illegible]	[illegible]	cyan	[illegible]	[illegible]	[illegible]	[illegible]		Calcium-binding EF-hand family protein	calcium-binding protein CML16, putative
[illegible]	[illegible]	[illegible]	[illegible]	[illegible]	[illegible]	[illegible]	cyan	[illegible]	[illegible]	[illegible]	[illegible]		Calcium-dependent lipid-binding (CaLB domain) family protein	NA
[illegible]	[illegible]	[illegible]	[illegible]	[illegible]	[illegible]	[illegible]	cyan	[illegible]	[illegible]	[illegible]	[illegible]		Major facilitator superfamily protein	peptide transporter, putative
[illegible]	[illegible]	[illegible]	[illegible]	[illegible]	[illegible]	[illegible]	cyan	[illegible]	[illegible]	[illegible]	[illegible]		Calcium-binding EF-hand family protein	EF-hand calcium-binding protein, putative
[illegible]	[illegible]	[illegible]	[illegible]	[illegible]	[illegible]	[illegible]	cyan	[illegible]	[illegible]	[illegible]	[illegible]	ATTPPB,TPPB	trehalose-6-phosphate phosphatase	trehalose-phosphate phosphatase
Sapur.T018100.1	[illegible]	[illegible]	[illegible]	[illegible]	[illegible]	[illegible]	cyan	[illegible]	[illegible]	[illegible]	[illegible]		Leucine-rich repeat transmembrane protein kinase	NA
[illegible]	[illegible]	[illegible]	[illegible]	[illegible]	[illegible]	[illegible]	cyan	[illegible]	[illegible]	[illegible]	[illegible]		Homeodomain-like superfamily protein	MYB transcription factor
[illegible]	[illegible]	[illegible]	[illegible]	[illegible]	[illegible]	[illegible]	cyan	[illegible]	[illegible]	[illegible]	[illegible]		Calmodulin-binding protein	calmodulin-binding family protein
[illegible]	[illegible]	[illegible]	[illegible]	[illegible]	[illegible]	[illegible]	cyan	[illegible]	[illegible]	[illegible]	[illegible]		Calcium-binding EF hand family protein	calcium-binding EF hand family protein
[illegible]	[illegible]	[illegible]	[illegible]	[illegible]	[illegible]	[illegible]	cyan	[illegible]	[illegible]	[illegible]	[illegible]	VGDH1	Plant invertase/pectin methylesterase inhibitor superfamily	pectinesterase/pectinesterase inhibitor, putative
[illegible]	[illegible]	[illegible]	[illegible]	[illegible]	[illegible]	[illegible]	cyan	[illegible]	[illegible]	[illegible]	[illegible]	CPK17	calcium-dependent protein kinase 17	calcium-dependent kinase
[illegible]	[illegible]	[illegible]	[illegible]	[illegible]	[illegible]	[illegible]	cyan	[illegible]	[illegible]	[illegible]	[illegible]	ATSOS3,CBL4,SOS3	Calcium-binding EF-hand family protein	calcineurin B-like protein
Sapur.010G196100.1	[illegible]	[illegible]	[illegible]	[illegible]	[illegible]	[illegible]	cyan	[illegible]	[illegible]	[illegible]	[illegible]	GATL4	galacturonosyltransferase-like 4	CAZy family GT8 glycosyltransferase

Sapur.016G227900.1	989.2866305	1.268197588	0.055919207	22.67911	7.20E-114	9.73E-113	cyan	0.802791697	1.24E-36		AT3G20290.1	ATEHD1,EHD1	EPS15 homology domain 1	calcium-binding EF hand protein
Sapur.009G079600.1	1152.055893	1.156478207	0.051295518	22.5454	1.49E-112	1.98E-111	cyan	0.805751786	4.34E-37	GO:0016021,GO:0005783	AT3G19260.1	LAG1 HOMOLOG 2,LOH2	LAG1 homologue 2	longevity assurance-like protein
Sapur.018G074500.1	137.0325842	7.127710091	0.318698861	22.36503	8.62E-111	1.12E-109	cyan	0.601381343	8.20E-17	GO:0055085,GO:0016021,GO:0015299,GO:0006812	AT5G22900.1	ATCHX3,CHX3	cation/H+ exchanger 3	cation/H(+) antiporter
Sapur.002G050700.1	19.02382929	7.440798143	0.333375545	22.31957	2.39E-110	3.08E-109	cyan	0.650742924	2.84E-20	GO:0009733	AT1G20470.1		SAUR-like auxin-responsive protein family	NA
Sapur.018G115100.1	553.4461817	2.899686359	0.129987352	22.30745	3.13E-110	4.04E-109	cyan	0.680089448	1.18E-22	GO:0055085,GO:0008324,GO:0006812	AT4G10310.1	ATHKT1,HKT1	high-affinity K+ transporter 1	sodium transporter HKT1
Sapur.016G181900.1	102.8906582	3.869401599	0.173925596	22.24745	1.19E-109	1.53E-108	cyan	0.653629208	1.70E-20		AT4G06744.1		Leucine-rich repeat (LRR) family protein	Serine/Threonine kinase, plant-type protein%2C putative
Sapur.001G152500.1	194.015342	3.542040199	0.159667672	22.18383	4.92E-109	6.26E-108	cyan	0.650465266	2.98E-20		AT1G79910.1		Regulator of Vps4 activity in the MVB pathway protein	regulator of Vps4 activity in the MVB pathway protein
Sapur.008G050000.1	511.8106314	2.600904997	0.117435321	22.14755	1.10E-108	1.39E-107	cyan	0.649689953	3.42E-20		AT2G39890.1	ATPROT1,PROT1	proline transporter 1	betaine/proline transporter
Sapur.019G029900.1	275.8980185	7.653871511	0.346465982	22.09126	3.84E-108	4.83E-107	cyan	0.586389097	7.09E-16	GO:0008408,GO:0006139,GO:0003676	AT5G06450.1		Polynucleotidyl transferase, ribonuclease H-like superfamily protein	3'-5' exonuclease
Sapur.003G064800.1	264.9654501	1.3417373	0.060861664	22.04569	1.05E-107	1.32E-106	cyan	0.808078023	1.88E-37		AT5G45610.1	SUV2	protein dimerizations	ATR interacting protein, putative
Sapur.008G008200.1	2028.667702	7.215641573	0.329197922	21.91886	1.72E-106	2.12E-105	cyan	0.552670633	6.16E-14	GO:0005975,GO:0004553	AT5G20950.2		Glycosyl hydrolase family protein	B-1%2C4-glucanase
Sapur.001G180400.1	183.3211908	1.333971006	0.060937343	21.89086	3.17E-106	3.91E-105	cyan	0.801747228	1.78E-36	GO:0051536,GO:0016226,GO:0005506	AT5G49940.1	ATCNFU2,NFU2	NIFU-like protein 2	NA
Sapur.014G001000.1	215.4308314	7.419616781	0.339308917	21.86685	5.37E-106	6.61E-105	cyan	0.555359746	4.40E-14	GO:0005515,GO:0006468,GO:0005524,GO:0004672	AT3G20190.1		Leucine-rich repeat protein kinase family protein	LRR receptor-like kinase
Sapur.004G161700.1	634.4731391	3.39579789	0.155514084	21.83595	1.06E-105	1.30E-104	cyan	0.630840137	8.38E-19	GO:0016758,GO:0008152	AT2G28080.1		UDP-Glycosyltransferase superfamily protein	UDP-glucosyltransferase, putative
Sapur.010G028800.1	166.0677164	2.527656404	0.115762064	21.83493	1.08E-105	1.32E-104	cyan	0.72342382	1.01E-26		AT1G23710.1		Protein of unknown function (DUF1645)	DUF1645 family protein
Sapur.009G114600.1	779.9568733	2.113496109	0.096838301	21.825	1.34E-105	1.64E-104	cyan	0.720826902	1.86E-26	GO:0008270,GO:0003677	AT2G36320.1		A20/AN1-like zinc finger family protein	zinc finger A20 and AN1 domain stress-associated protein
Sapur.010G176900.1	526.6233654	1.322193886	0.060846725	21.72991	1.07E-104	1.30E-103	cyan	0.817295287	6.07E-39	GO:0016833,GO:0009058	AT2G28880.1	ADCS,emb1997	para-aminobenzoate (PABA) synthase family protein	aminodeoxychorismate synthase
Sapur.006G113100.2	43.64779394	2.280378711	0.105023707	21.71299	1.55E-104	1.87E-103	cyan	0.569913313	6.69E-15	GO:0009733	AT5G20810.1		SAUR-like auxin-responsive protein family	SAUR-like auxin-responsive protein
Sapur.016G249900.1	847.1070705	1.144063628	0.052711878	21.7041	1.88E-104	2.27E-103	cyan	0.800264994	2.99E-36	GO:0005515	AT1G53320.1	AtTLP7,TLP7	tubby like protein 7	Tub transcription factor
Sapur.T053700.1	44.43976922	2.336219748	0.107747024	21.68245	3.00E-104	3.62E-103	cyan	0.77046361	4.08E-32	GO:0006468,GO:0004672	AT1G07650.1		Leucine-rich repeat transmembrane protein kinase	NA
Sapur.002G163800.1	574.6730735	1.317848464	0.060783965	21.68086	3.11E-104	3.74E-103	cyan	0.811113633	6.19E-38	GO:0031011	AT2G47350.1		HIT zinc finger ;PAPA-1-like conserved region	INO80 complex subunit B
Sapur.007G051500.1	67.17275786	8.148256607	0.375877386	21.67796	3.31E-104	3.98E-103	cyan	0.598047521	1.34E-16	GO:0006468,GO:0005524,GO:0004672	AT4G28670.1		Protein kinase family protein with domain of unknown function (DUF26)	NA
Sapur.017G064700.1	2094.166271	7.289612263	0.336643052	21.65383	5.59E-104	6.70E-103	cyan	0.548741193	1.00E-13	GO:0030570	AT5G15110.1		Pectate lyase family protein	pectate lyase, putative
Sapur.008G028300.1	672.502491	1.883266839	0.087009335	21.64442	6.86E-104	8.19E-103	cyan	0.731914871	1.31E-27		AT2G38970.1		Zinc finger (C3HC4-type RING finger) family protein	NA
Sapur.005G202100.1	78.31972575	1.810086678	0.08363291	21.64323	7.04E-104	8.40E-103	cyan	0.783050917	8.78E-34		AT4G37420.1		Domain of unknown function (DUF23)	glycosyltransferase family 92 protein
Sapur.016G189400.1	41.59343491	7.934753964	0.367754176	21.57624	3.00E-103	3.56E-102	cyan	0.599124338	1.14E-16		AT5G09430.1		alpha/beta-Hydrolases superfamily protein	alpha/beta-hydrolase domain protein
Sapur.003G126600.1	17.11050954	5.244583304	0.243218808	21.56323	3.98E-103	4.72E-102	cyan	0.681823429	8.40E-23	GO:0006355,GO:0005634,GO:0003700,GO:0046983,GO:0003677	AT1G26310.1	AGL10,CAL,CAL1	K-box region and MADS-box transcription factor family protein	NA
Sapur.005G182100.1	340.048709	7.594230455	0.35259024	21.5384	6.80E-103	8.03E-102	cyan	0.535935308	4.72E-13					NA
Sapur.019G059000.1	63.45111673	2.405378543	0.111883883	21.49888	1.59E-102	1.88E-101	cyan	0.747006133	2.81E-29	GO:0005515,GO:0006468,GO:0004672	AT3G14840.2		Leucine-rich repeat transmembrane protein kinase	NA
Sapur.003G137200.1	241.6428895	1.009956478	0.04706227	21.46	3.68E-102	4.31E-101	cyan	0.810951372	6.57E-38	GO:0016021,GO:0006888,GO:0005801,GO:0000139	AT1G15880.1	atgos11,GOS11	golgi snare 11	SNAP receptor complex protein
Sapur.003G082100.1	744.4745643	1.258296681	0.058763245	21.41299	1.01E-101	1.18E-100	cyan	0.777238402	5.33E-33		AT1G12450.1		SNARE associated Golgi protein family	SNARE associated family protein
Sapur.007G101300.1	373.4728795	3.441488787	0.160917509	21.38666	1.78E-101	2.06E-100	cyan	0.599331543	1.11E-16		AT1G58122.1	CPuORF45	conserved peptide upstream open reading frame 45	peptide upstream ORF protein
Sapur.014G000800.1	64.39913855	6.184522877	0.290063207	21.32129	7.20E-101	8.30E-100	cyan	0.610015405	2.25E-17	GO:0005515,GO:0006468,GO:0004672	AT1G78980.1	SRF5	STRUBBELIG-receptor family 5	NA
Sapur.002G148800.1	4214.338852	2.276736733	0.106795361	21.31869	7.62E-101	8.77E-100	cyan	0.70079251	1.66E-24	GO:0006629	AT2G46210.1		Fatty acid/sphingolipid desaturase	fatty acid/sphingolipid desaturase
Sapur.019G098300.1	146.9618897	7.723865551	0.362758452	21.29203	1.35E-100	1.54E-99	cyan	0.533021675	6.65E-13		AT1G65690.1		Late embryogenesis abundant (LEA) hydroxyproline-rich glycoprotein family	late embryogenesis abundant protein
Sapur.006G120500.1	95.09255408	2.921107056	0.137339116	21.2693	2.19E-100	2.50E-99	cyan	0.684226277	5.19E-23		AT5G35460.1			plant/MOK9-4 protein
Sapur.006G013600.1	266.784578	5.20672977	0.246727979	21.10312	7.45E-99	8.36E-98	cyan	0.564595911	1.35E-14		AT5G41761.1			NA
Sapur.016G273400.1	215.922701	2.744479495	0.130216089	21.07635	1.31E-98	1.47E-97	cyan	0.645814032	6.72E-20		AT5G54585.1			hypothetical protein
Sapur.003G003300.1	374.0775061	3.780384444	0.180222968	20.97615	1.08E-97	1.20E-96	cyan	0.652893753	1.94E-20	GO:0005515	AT5G49920.1		Octicosapeptide/Phox/Bem1p family protein	PB1 domain protein
Sapur.013G039400.1	148.2209441	6.581604872	0.31423899	20.94458	2.10E-97	2.32E-96	cyan	0.538515013	3.47E-13		AT1G08790.1		Protein of unknown function (DUF1685)	NA
Sapur.004G115400.1	224.2668748	5.964440731	0.28649347	20.81877	2.93E-96	3.18E-95	cyan	0.508508556	1.05E-11	GO:0006468,GO:0004672	AT4G34440.1		Protein kinase superfamily protein	NA
Sapur.001G114300.1	963.7195304	0.903019957	0.043569621	20.72591	2.02E-95	2.17E-94	cyan	0.806591149	3.21E-37	GO:0005515	AT5G46860.1	ATSYP22.ATVAM3.SGR3.SYF	Syntaxin/t-SNARE family protein	syntaxin
Sapur.014G051800.1	103.2715764	2.590073851	0.125413811	20.65222	9.32E-95	9.95E-94	cyan	0.65173852	2.38E-20		AT4G00220.1	JLO,LBD30	Lateral organ boundaries (LOB) domain family protein	LOB domain protein
Sapur.006G099900.1	67.21016804	3.34850287	0.162174687	20.64751	1.03E-94	1.10E-93	cyan	0.665674221	1.89E-21		AT2G28200.1		C2H2-type zinc finger family protein	C2H2-type zinc-finger protein, putative
Sapur.15WG003200.1	38.97343227	4.361060375	0.211365522	20.63279	1.39E-94	1.49E-93	cyan	0.507120815	1.22E-11		AT5G19650.1	ATOFP8,OFP8	ovate family protein 8	ovate transcriptional repressor
Sapur.011G099800.1	651.8480521	2.212047561	0.10748231	20.58057	4.10E-94	4.34E-93	cyan	0.666335528	1.67E-21		AT4G27100.2		Ypt/Rab-GAP domain of gyp1p superfamily protein	GTPase-activating protein
Sapur.007G110700.1	1014.46333	0.953329728	0.046438109	20.52904	1.18E-93	1.25E-92	cyan	0.811991049	4.48E-38	GO:0006468,GO:0004672	AT2G43230.2		Protein kinase superfamily protein	PTI1-like tyrosine-kinase
Sapur.016G243700.1	1107.222471	2.374216685	0.115918784	20.48173	3.13E-93	3.28E-92	cyan	0.679461925	1.34E-22		AT3G62730.1			plant/F26K9-160 protein
Sapur.010G146500.1	28.52575236	5.60334316	0.274122092	20.44105	7.22E-93	7.53E-92	cyan	0.621799596	3.60E-18	GO:0003824,GO:0006470,GO:0004722	AT2G40180.1	ATHPP2C5,PP2C5	phosphatase 2C5	protein phosphatase 2C
Sapur.013G011300.1	415.778334	2.435456366	0.11926615	20.42035	1.10E-92	1.15E-91	cyan	0.678758571	1.54E-22	GO:0003824	AT3G05630.1	PDLZ2,PLDP2,PLDZETA2	phospholipase D P2	phospholipase D
Sapur.008G083500.1	31.90842178	3.790000432	0.186044861	20.37143	3.00E-92	3.10E-91	cyan	0.661668825	3.96E-21	GO:0006468,GO:0004672	AT3G04690.1	ANX1	Malectin/receptor-like protein kinase family protein	feronia receptor-like kinase
Sapur.005G071900.1	18.40676006	6.788733554	0.333454696	20.35879	3.88E-92	4.01E-91	cyan	0.634925374	4.27E-19					NA
Sapur.006G180700.1	1169.558239	7.134595163	0.350525379	20.354	4.28E-92	4.41E-91	cyan	0.505188567	1.50E-11	GO:0005975,GO:0004553	AT3G43860.1	AtGH9A4,GH9A4	glycosyl hydrolase 9A4	endoglucanase
Sapur.017G078100.1	15.32669743	5.309081016	0.261414747	20.30903	1.07E-91	1.10E-90	cyan	0.689460938	1.79E-23		AT5G39200.1			NA
Sapur.017G103000.1	2321.373952	1.81871106	0.089644525	20.28803	1.64E-91	1.68E-90	cyan	0.717692187	3.86E-26		AT1G23170.2		Protein of unknown function DUF2359, transmembrane	DUF2359 family protein
Sapur.T001000.1	28.24927387	5.278734979	0.260805849	20.24009	4.34E-91	4.43E-90	cyan	0.589745277	4.41E-16	GO:0006468,GO:0004672	AT1G07650.2		Leucine-rich repeat transmembrane protein kinase	NA
Sapur.007G017200.1	1238.633332	2.149842555	0.106615671	20.16441	2.01E-90	2.03E-89	cyan	0.715015955	7.14E-26	GO:0006468,GO:0005524,GO:0004672	AT2G18170.1	ATMPK7,MPK7	MAP kinase 7	MAP kinase
Sapur.012G079500.1	1807.936497	0.947479929	0.047043784	20.14039	3.27E-90	3.28E-89	cyan	0.783290297	8.14E-34	GO:0016829,GO:0006520	AT5G23670.2	LCB2	long chain base2	long chain base biosynthesis protein 2a
Sapur.010G005600.1	627.7069461	6.952550611	0.345378908	20.13021	4.01E-90	4.02E-89	cyan	0.566811176	1.01E-14		AT1G02790.1	PGA4	polygalacturonase 4	polygalacturonase
Sapur.010G051900.1	424.9368817	4.721932549	0.234665512	20.12197	4.74E-90	4.74E-89	cyan	0.514382819	5.54E-12					hypothetical protein
Sapur.011G060700.1	171.1958292	1.582884115	0.078671192	20.12025	4.91E-90	4.91E-89	cyan	0.785145891	4.52E-34	GO:0030983,GO:0006298,GO:0005524	AT3G20475.1	ATMSH5,MSH5	MUTS-homologue 5	NA
Sapur.016G278500.1	197.6739698	7.391623806	0.367753005	20.09942	7.47E-90	7.45E-89	cyan	0.540865928	2.62E-13	GO:0006468,GO:0005524,GO:0004672	AT3G20530.1		Protein kinase superfamily protein	receptor Serine/Threonine kinase, putative
Sapur.010G068000.1	629.3482304	1.547675177	0.077037074	20.09	9.03E-90	8.99E-89	cyan	0.740543515	1.50E-28	GO:0007165	AT4G14580.1	CIPK4,SnRK3.3	CBL-interacting protein kinase 4	CBL-interacting kinase
Sapur.019G019700.1	194.3571715	3.00275965	0.149555144	20.07794	1.15E-89	1.14E-88	cyan	0.646348034	6.12E-20					hypothetical protein
Sapur.001G081500.1	378.6935257	2.678907021	0.133572176	20.05588	1.79E-89	1.78E-88	cyan	0.667380364	1.37E-21		AT1G63930.1	ROH1	from the Czech \'roh\' meaning \'corner\'	plant/F6A14-15 protein, putative
Sapur.003G084600.1	4604.830099	1.358898984	0.06784763	20.02869	3.10E-89	3.07E-88	cyan	0.747095171	2.75E-29		AT1G63000.1	NRS/ER,UER1	nucleotide-rhamnose synthase/epimerase-reductase	3%2C5-epimerase/4-reductase
Sapur.009G106400.1	80.60135856	3.432045736	0.171830433	19.97345	9.38E-89	9.20E-88	cyan	0.604356379	5.27E-17		AT5G09220.1	AAP2	amino acid permease 2	amino acid transporter
Sapur.001G075900.1	2159.154032	2.42345905	0.121741659	19.90657	3.57E-88	3.48E-87	cyan	0.728594249	2.94E-27		AT5G41860.1			hypothetical protein
Sapur.005G017700.1	608.1427502	0.983553701	0.049431755	19.8972	4.30E-88	4.19E-87	cyan	0.801273546	2.10E-36	GO:0016787	AT5G27840.1	TOPP8	Calcineurin-like metallo-phosphoesterase superfamily protein	serine/threonine-protein phosphatase PP1
Sapur.003G128000.1	1091.744937	1.144193496	0.057526628	19.88981	4.99E-88	4.85E-87	cyan	0.784243405	6.02E-34	GO:0005783	AT1G27350.1		Ribosome associated membrane protein RAMP4	ribosome associated membrane protein RAMP4
Sapur.005G000600.1	874.9591146	2.606979078	0.131594361	19.81072	2.41E-87	2.32E-86	cyan	0.664012001	2.57E-21		AT3G62020.1	GLP10	germin-like protein 10	germin-like protein
Sapur.012G064400.1	76.32360717	2.305651424	0.116724179	19.75299	7.56E-87	7.25E-86	cyan	0.777421624	5.04E-33	GO:0006468,GO:0005524,GO:0004672	AT5G40380.1	CRK42	cysteine-rich RLK (RECEPTOR-like protein kinase) 42	cysteine-rich receptor-like kinase
Sapur.016G061500.1	152.0404799	4.16801268	0.211446861	19.71187	1.71E-86	1.63E-85	cyan	0.627096198	1.54E-18		AT3G52360.1			transmembrane protein, putative
Sapur.018G006500.1	48.5116205	5.599442334	0.286677031	19.53223	5.84E-85	5.46E-84	cyan	0.627064707	1.55E-18		AT1G61566.1	RALFL9	ralf-like 9	rapid aLkalinization factor
Sapur.002G008300.2	10.07672767	4.401501904	0.225366305	19.53043	6.05E-85	5.65E-84	cyan	0.507873522	1.13E-11		AT4G35190.1		Putative lysine decarboxylase family protein	cytokinin riboside 5'-monophosphate phosphoribohydrolase LOG5, putative
Sapur.008G011500.1	219.3939466	6.32990917	0.324879406	19.48387	1.50E-84	1.40E-83	cyan	0.46404659	9.29E-10	GO:0016757	AT3G06260.1	GATL4	galacturonosyltransferase-like 4	CAZy family GT8 glycosyltransferase
Sapur.002G158900.1	1047.592427	7.199621449	0.369683731	19.47508	1.79E-84	1.66E-83	cyan	0.535922536	4.73E-13	GO:0005975,GO:0004650	AT1G02790.1	PGA4	polygalacturonase 4	polygalacturonase
Sapur.002G058700.1	25.72297618	3.318135627	0.170846169	19.42177	5.05E-84	4.66E-83	cyan	0.605533377	4.42E-17					NA
Sapur.016G089600.1	454.3676471	7.74996074	0.399284732	19.40961	6.40E-84	5.90E-83	cyan	0.49130261	6.43E-11		AT2G39700.1	ATEXP4.ATEXPA4.ATHEXP A	expansin A4	alpha expansin
Sapur.018G114700.1	142.8818122	2.634665928	0.135768021	19.40564	6.91E-84	6.37E-83	cyan	0.660837242	4.62E-21	GO:0055085,GO:0008324,GO:0006812	AT4G10310.1	ATHKT1,HKT1	high-affinity K+ transporter 1	NA
Sapur.013G006200.1	419.7227123	0.963266845	0.049675449	19.39121	9.16E-84	8.42E-83	cyan	0.803987847	8.12E-37	GO:0033926	AT1G56560.1		Plant neutral invertase family protein	neutral/alkaline invertase
Sapur.013G066300.1	410.9238977	2.052020674	0.10594786	19.36821	1.43E-83	1.31E-82	cyan	0.70667065	4.63E-25	GO:0043565,GO:0006355,GO:0003700	AT2G40620.1		Basic-leucine zipper (bZIP) transcription factor family protein	BZIP transcription factor
Sapur.010G172600.1	25.15910534	3.832351428	0.198620135	19.29488	5.93E-83	5.39E-82	cyan	0.591763457	3.31E-16					NA
Sapur.016G147300.1	44.89362404	1.798582715	0.09335154	19.26677	1.02E-82	9.24E-82	cyan	0.727174526	4.14E-27	GO:0046872	AT5G12850.1		CCCH-type zinc finger protein with ARM repeat domain	zinc finger CCCH domain protein
Sapur.002G148400.1	40.90752802	2.278720814	0.11848072	19.23284	1.97E-82	1.77E-81	cyan	0.69628408	4.34E-24	GO:0051087	AT2G46240.1	ATBAG6,BAG6	BCL-2-associated athanogene 6	BAG domain protein
Sapur.016G307600.1	559.3219466	1.877360154	0.097748548	19.20602	3.30E-82	2.96E-81	cyan	0.71775132	3.81E-26		AT5G23810.1	AAP7	amino acid permease 7	amino acid permease
Sapur.008G158400.1	8.616994546	6.184219461	0.322077083	19.20105	3.63E-82	3.26E-81	cyan	0.654062102	1.57E-20	GO:0046872,GO:0030001	AT3G21490.1		Heavy metal transport/detoxification superfamily protein	NA
Sapur.010G007000.1	371.0867595	6.862644009	0.358376437	19.14926	9.82E-82	8.76E-81	cyan	0.534455313	5.62E-13		AT1G02790.1	PGA4	polygalacturonase 4	polygalacturonase
Sapur.001G094100.1	664.3245897	1.607830538	0.083981919	19.14496	1.07E-81	9.52E-81	cyan	0.722597634	1.23E-26	GO:0008270,GO:0003677	AT4G12040.2		A20/AN1-like zinc finger family protein	zinc finger A20 and AN1 domain stress-associated protein
Sapur.008G061000.1	20.66237491	4.3578685	0.227637372	19.14391	1.09E-81	9.70E-81	cyan	0.591432204	3.47E-16	GO:0006511	AT2G29070.2		Ubiquitin fusion degradation UFD1 family protein	ubiquitin fusion degradation protein
Sapur.019G072500.1	635.2574375	2.62709257	0.138110588	19.02166	1.13E-80	9.92E-80	cyan	0.719660736	2.45E-26	GO:0080019	AT4G33790.1	CER4,FAR3,G7	Jojoba acyl CoA reductase-related male sterility protein	fatty acyl-CoA reductase
Sapur.016G107300.1	1153.50698	1.671700474	0.087985763	18.99967	1.72E-80	1.50E-79	cyan	0.706818602	4.48E-25	GO:0005515	AT3G51770.2	ATEOL1,ETO1	tetratricopeptide repeat (TPR)-containing protein	ethylene-overproduction protein
Sapur.018G102100.1	62.08634879	2.770847838	0.146118538	18.96301	3.45E-80	3.01E-79	cyan	0.607591988	3.24E-17		AT1G80133.1			epidermal patterning factor-like protein, putative
Sapur.011G031800.1	45.71266415	5.305510172	0.280492124	18.91501	8.58E-80	7.46E-79	cyan	0.47463549	3.38E-10		AT4G04980.1			CHUP1, putative
Sapur.008G033500.1	15.50918005	2.368350224	0.125231267	18.91181	9.12E-80	7.93E-79	cyan	0.750132462	1.23E-29		AT3G53320.1			hypothetical protein
Sapur.018G085100.1	205.5292802	1.826381017	0.096596038	18.90741	9.91E-80	8.61E-79	cyan	0.745273509	4.43E-29		AT4G31020.2		alpha/beta-Hydrolases superfamily protein	alpha/beta hydrolase family protein
Sapur.004G058700.1	17.96951739	3.157911065	0.167303329	18.87536	1.82E-79	1.57E-78	cyan	0.677362818	2.02E-22	GO:0055085,GO:0016020,GO:0006811,GO:0005216	AT3G02850.1	SKOR	STELAR K+ outward rectifier	outward rectifying potassium channel protein
Sapur.009G003300.1	645.6301569	3.872549801	0.205283883	18.86436	2.24E-79	1.93E-78	cyan	0.524792785	1.72E-12	GO:0055085,GO:0016021	AT2G28120.1		Major facilitator superfamily protein	nitrate and chloride transporter
Sapur.003G061100.1	113.0130815	3.319047134	0.176037397	18.85422	2.71E-79	2.34E-78	cyan	0.615000048	1.05E-17		AT5G46840.1		RNA-binding (RRM/RBD/RNP motifs) family protein	RNA recognition motif protein
Sapur.011G112400.1	1555.632823	1.282001007	0.068059938	18.83635	3.80E-79	3.27E-78	cyan	0.76804729	8.28E-32		AT5G42110.1			hypothetical protein
Sapur.017G053900.1	89.26248864	5.674599552	0.302983798	18.72905	2.87E-78	2.43E-77	cyan	0.526618959	1.40E-12	GO:0016788	AT1G71691.2		GDSL-like Lipase/Acylhydrolase superfamily protein	NA
Sapur.001G112100.1	922.9782519	1.867861433	0.09976531	18.72255	3.24E-78	2.75E-77	cyan	0.681479529	8.99E-23	GO:0048046,GO:0016762,GO:0006073,GO:0005618,GO:0005975,GO:0004553	AT1G32170.1	XTH30,XTR4	xyloglucan endotransglucosylase/hydrolase 30	xyloglucan endotransglycosylase/hydrolase
Sapur.012G027100.1	101.310173	2.390285633	0.128101597	18.6593	1.06E-77	8.94E-77	cyan	0.670982772	6.93E-22	GO:0005544,GO:0005509	AT1G68090.1	ANN5,ANNAT5	annexin 5	annexin
Sapur.014G060000.1	185.3849154	2.354655608	0.126725494	18.58076	4.60E-77	3.84E-76	cyan	0.659269143	6.15E-21	GO:0006468,GO:0004672	AT4G00720.1	ASKTHETA,ATSK32,SK32	shaggy-like protein kinase 32	shaggy-related kinase
Sapur.003G089400.3	11.95156055	4.24976624	0.228747843	18.57839	4.81E-77	4.01E-76	cyan	0.671422844	6.37E-22		AT5G23750.2		Remorin family protein	remorin, carboxy-terminal region protein
Sapur.016G238800.1	1486.865002	1.008179617	0.054280358	18.57356	5.26E-77	4.39E-76	cyan	0.782435062	1.07E-33	GO:0008270,GO:0005515	AT1G53190.2		RING/U-box superfamily protein	RING finger protein
Sapur.15WG009700.1	373.7513223	1.569367062	0.084745228	18.51865	1.46E-76	1.21E-75	cyan	0.702091251	1.26E-24		AT5G52990.1		SNARE-like superfamily protein	VAMP-like protein, putative
Sapur.004G053500.1	4215.979654	1.251564933	0.067611363	18.51116	1.68E-76	1.39E-75	cyan	0.775208088	9.88E-33		AT1G65840.1	ATPAO4,PAO4	polyamine oxidase 4	polyamine oxidase, putative
Sapur.013G094700.1	255.8714179	2.717905482	0.147003021	18.48877	2.54E-76	2.10E-75	cyan	0.644628135	8.24E-20		AT4G32480.1		Protein of unknown function (DUF506)	plant/F8B4-180 protein, putative
Sapur.008G147100.1	2865.976211	1.785514043	0.096671915	18.46983	3.61E-76	2.98E-75	cyan	0.722251665	1.33E-26		AT1G67730.1	ATKCR1,KCR1,YBR159	beta-ketoacyl reductase 1	3-ketoacyl-CoA reductase
Sapur.007G042900.1	4.962723236	5.016335645	0.272120275	18.43426	6.98E-76	5.73E-75	cyan	0.711169182	1.70E-25	GO:0008270,GO:0005515	AT4G34040.1		RING/U-box superfamily protein	NA
Sapur.018G118000.1	1270.276616	4.18876838	0.227369476	18.42274	8.63E-76	7.08E-75	cyan	0.504632586	1.60E-11	GO:0055114,GO:0020037,GO:0016705,GO:0005506	AT5G36110.1	CYP716A1	cytochrome P450, family 716, subfamily A, polypeptide 1	cytochrome P450 family 716 monooxygenase
Sapur.012G075500.1	769.6677752	1.080358953	0.058767763	18.38353	1.78E-75	1.45E-74	cyan	0.745438704	4.25E-29	GO:0005515	AT5G50530.1		CBS / octicosapeptide/Phox/Bemp1 (PB1) domains-containing protein	meiotically up-regulated protein
Sapur.002G130200.1	1311.263707	1.864198816	0.101434021	18.37844	1.96E-75	1.59E-74	cyan	0.659861233	5.52E-21		AT2G45900.1		Phosphatidylinositol N-acetyglucosaminlytransferase subunit P-related	DUF4378 domain protein
Sapur.016G001000.1	874.4376184	0.715136323	0.03898807	18.34244	3.79E-75	3.07E-74	cyan	0.797324059	8.20E-36	GO:0005525	AT4G17170.1	AT-RAB2.ATRAB-B1B.ATRAE	RAB GTPase homolog B1C	small GTPase family Rab protein
Sapur.011G118700.1	40.41143569	6.150478153	0.335333945	18.34135	3.87E-75	3.13E-74	cyan	0.428247749	2.21E-08	GO:0055114,GO:0050660,GO:0016491	AT2G34790.1	EDA28,MEE23	FAD-binding Berberine family protein	reticuline oxidase, putative
Sapur.011G119400.1	1633.334163	1.057918415	0.057683502	18.34005	3.96E-75	3.21E-74	cyan	0.789740668	1.03E-34	GO:0055114,GO:0016491,GO:0006633,GO:0005506	AT2G34770.1	ATFAH1,FAH1	fatty acid hydroxylase 1	fatty acid hydroxylase
Sapur.002G158800.1	811.4676937	6.624622777	0.362290784	18.28537	1.08E-74	8.70E-74	cyan	0.503676242	1.77E-11	GO:0005975,GO:0004650	AT1G02790.1	PGA4	polygalacturonase 4	polygalacturonase
Sapur.T001100.1	50.33192774	2.391703922	0.131230387	18.22523	3.26E-74	2.60E-73	cyan	0.709765708	2.33E-25	GO:0006468,GO:0004672	AT1G07650.2		Leucine-rich repeat transmembrane protein kinase	NA
Sapur.019G035900.1	1434.240938	1.052929803	0.058042113	18.14079	1.52E-73	1.20E-72	cyan	0.759215481	1.03E-30	GO:0005515,GO:0016020	AT3G03800.1	ATSYP131,SYP131	syntaxin of plants 131	syntaxin-132 protein
Sapur.008G098000.1	43.44281724	1.574716291	0.086991919	18.10187	3.08E-73	2.43E-72	cyan	0.736104169	4.62E-28					NA
Sapur.016G176000.1	1102.991863	1.740206668	0.096260371	18.07812	4.74E-73	3.73E-72	cyan	0.718609082	3.12E-26		AT1G27730.1	STZ,ZAT10	salt tolerance zinc finger	C2H2-type zinc-finger protein
Sapur.006G153500.1	84.38229818	2.552338993	0.141599712	18.02503	1.24E-72	9.70E-72	cyan	0.658769786	6.74E-21		AT4G31020.1		alpha/beta-Hydrolases superfamily protein	alpha/beta hydrolase family protein
Sapur.008G138900.1	76.75810816	1.633374172	0.090625167	18.02341	1.28E-72	9.98E-72	cyan	0.723632639	9.63E-27		AT2G33390.1			hypothetical protein
Sapur.016G181700.1	1892.062423	1.323985138	0.073736139	17.95572	4.33E-72	3.36E-71	cyan	0.67261174	5.08E-22	GO:0008234,GO:0006508	AT1G47128.1	RD21,RD21A	Granulin repeat cysteine protease family protein	cysteine protease
Sapur.016G114400.1	19.20561756	5.872201781	0.327277503	17.94258	5.49E-72	4.25E-71	cyan	0.536953499	4.18E-13	GO:0016567,GO:0004842	AT3G11840.1	PUB24	plant U-box 24	spotted leaf protein, putative
Sapur.018G096400.1	176.307103	2.289914932	0.127943346	17.89788	1.22E-71	9.45E-71	cyan	0.631021938	8.13E-19	GO:0016758,GO:0009247	AT5G20410.1	ATMGD2,MGD2	monogalactosyldiacylglycerol synthase 2	monogalactosyldiacylglycerol synthase
Sapur.T017100.1	79.49422117	2.29502321	0.128359841	17.8796	1.70E-71	1.31E-70	cyan	0.706235639	5.10E-25	GO:0006468,GO:0004672	AT3G14840.2		Leucine-rich repeat transmembrane protein kinase	NA
Sapur.014G056500.1	465.8349801	2.507589576	0.140347982	17.86694	2.13E-71	1.64E-70	cyan	0.683326569	6.22E-23	GO:0003824,GO:0005515	AT2G42010.1	PLDBETA,PLDBETA1	phospholipase D beta 1	phospholipase D
Sapur.014G113200.1	101.9229527	1.44170577	0.081057545	17.7862	9.04E-71	6.89E-70	cyan	0.724097502	8.63E-27		AT3G26680.3	ATSNM1,SNM1	DNA repair metallo-beta-lactamase family protein	DNA cross-link repair protein SNM1
Sapur.002G160200.1	855.7649374	1.030763224	0.058017225	17.7665	1.28E-70	9.77E-70	cyan	0.751560027	8.38E-30	GO:0016757	AT3G62660.1	GATL7	galacturonosyltransferase-like 7	glycosyltransferase family 8 protein
Sapur.018G058100.1	1336.365055	1.08217905	0.060937422	17.75886	1.47E-70	1.12E-69	cyan	0.743415879	7.19E-29	GO:0005524	AT3G24530.1		AAA-type ATPase family protein / ankyrin repeat family protein	AAA-type ATPase family protein/ankyrin repeat protein
Sapur.011G069800.1	1231.442052	1.06539751	0.060135224	17.7167	3.12E-70	2.35E-69	cyan	0.750681274	1.06E-29	GO:0008270,GO:0005515	AT1G53190.2		RING/U-box superfamily protein	zinc finger protein, putative
Sapur.001G139700.1	276.8521138	2.605776049	0.147269983	17.69387	4.68E-70	3.52E-69	cyan	0.641039216	1.52E-19	GO:0016021,GO:0016020,GO:0022857	AT5G47470.1		Nodulin MtN21 /EamA-like transporter family protein	auxin-induced protein 5NG4
Sapur.014G004800.1	27.11026424	2.271745459	0.12912325	17.59362	2.76E-69	2.05E-68	cyan	0.64377601	9.55E-20					NA
Sapur.002G174100.1	154.9275161	1.710366657	0.097619945	17.52067	9.96E-69	7.34E-68	cyan	0.66440253	2.39E-21		AT2G32970.2			NA
Sapur.019G020200.1	42.55491965	3.017466202	0.172358032	17.50697	1.27E-68	9.33E-68	cyan	0.630627991	8.67E-19					NA
Sapur.001G077700.1	1011.897871	1.400336362	0.08012185	17.47758	2.12E-68	1.56E-67	cyan	0.732910429	1.02E-27		AT3G61640.1	AGP20,AtAGP20	arabinogalactan protein 20	transmembrane protein, putative
Sapur.007G105100.1	610.1812593	2.612368237	0.149600257	17.46232	2.77E-68	2.03E-67	cyan	0.615934157	9.04E-18	GO:0016829,GO:0010333,GO:0008152,GO:0000287	AT4G16740.1	ATTPS03,TPS03	terpene synthase 03	(E)-beta-ocimene synthase
Sapur.008G056400.1	38.1569631	2.906167517	0.16655908	17.44827	3.55E-68	2.59E-67	cyan	0.654839129	1.37E-20	GO:0016747	AT2G40230.1		HXXXD-type acyl-transferase family protein	taxadien-5-alpha-ol O-acetyltransferase, putative
Sapur.016G102900.1	66.0872699	3.16394439	0.181728917	17.41024	6.90E-68	5.02E-67	cyan	0.554350292	4.99E-14	GO:0016192,GO:0016021	AT5G05570.1		transducin family protein / WD-40 repeat family protein	synaptobrevin protein
Sapur.002G013600.1	25.80658841	2.310538974	0.132752763	17.40483	7.58E-68	5.51E-67	cyan	0.673961705	3.92E-22	GO:0006468,GO:0005524,GO:0004672	AT1G77280.1		Protein kinase protein with adenine nucleotide alpha hydrolases-like domain	tyrosine kinase domain protein
Sapur.010G185400.1	497.2961825	1.33622495	0.076789688	17.4011	8.09E-68	5.88E-67	cyan	0.720439766	2.04E-26		AT3G20898.1			cyclin-dependent kinase inhibitor siamese protein, putative
Sapur.019G056700.1	736.114178	0.732572818	0.042139209	17.38459	1.08E-67	7.83E-67	cyan	0.776482606	6.71E-33	GO:0016021,GO:0006810,GO:0005524,GO:0005471	AT5G05310.2		TLC ATP/ADP transporter	TLC ATP/ADP transporter
Sapur.018G020000.1	88.67674179	2.439148814	0.140532153	17.35652	1.76E-67	1.27E-66	cyan	0.621101291	4.02E-18	GO:0005515	AT2G25290.1		Octicosapeptide/Phox/Bem1p (PB1) domain-containing protein / tetratricopeptide repeat (TPR)-containing protei	heat shock protein 70 (HSP70)-interacting protein, putative
Sapur.009G076700.1	2476.336951	1.684876204	0.097415849	17.29571	5.07E-67	3.64E-66	cyan	0.683882022	5.56E-23	GO:0008234,GO:0006508	AT1G47128.1	RD21,RD21A	Granulin repeat cysteine protease family protein	cysteine protease
Sapur.006G063800.1	208.5807876	2.025059608	0.117189358	17.28023	6.63E-67	4.75E-66	cyan	0.608886942	2.67E-17	GO:0006950,GO:0006468,GO:0005524,GO:0004672	AT2G24370.1		Protein kinase protein with adenine nucleotide alpha hydrolases-like domain	tyrosine kinase domain protein
Sapur.006G096700.1	373.1562933	0.897452161	0.052080633	17.23197	1.53E-66	1.09E-65	cyan	0.734003792	7.80E-28		AT3G53000.1	AtPP2-A15,PP2-A15	phloem protein 2-A15	F-box protein PP2-A15
Sapur.001G148600.1	1392.601517	1.036809454	0.060174276	17.23011	1.58E-66	1.12E-65	cyan	0.698101426	2.96E-24	GO:0015969	AT1G54130.1	AT-RSH3,ATRSH3,RSH3	RELA/SPOT homolog 3	RelA/SpoT-like protein, putative
Sapur.T009900.1	6.439279168	4.288066408	0.249351996	17.19684	2.80E-66	1.99E-65	cyan	0.69014793	1.56E-23					NA
Sapur.003G086600.1	20.90172855	2.85087534	0.16610108	17.1635	4.98E-66	3.53E-65	cyan	0.6956166	5.00E-24	GO:0016020,GO:0009055,GO:0008137	AT4G26965.1		NADH:ubiquinone oxidoreductase, 17.2kDa subunit	NA
Sapur.009G019600.1	10.71561712	6.644434281	0.387859153	17.13105	8.71E-66	6.14E-65	cyan	0.538620521	3.43E-13	GO:0006412,GO:0005840,GO:0003735	AT3G55280.1	RPL23AB	ribosomal protein L23AB	60S ribosomal protein L23a
Sapur.016G154400.1	697.9300657	1.156070285	0.067567737	17.1098	1.25E-65	8.82E-65	cyan	0.739118358	2.16E-28	GO:0008270,GO:0005515,GO:0046872	AT3G08505.2		zinc finger (CCCH-type/C3HC4-type RING finger) family protein	NA
Sapur.001G079700.3	21.06996042	2.699247138	0.158074434	17.0758	2.25E-65	1.57E-64	cyan	0.700429894	1.80E-24	GO:0009378,GO:0006310,GO:0006281	AT1G64110.2		P-loop containing nucleoside triphosphate hydrolases superfamily protein	P-loop nucleoside triphosphate hydrolase-like protein
Sapur.15WG147700.1	994.4814567	1.146555788	0.067362559	17.02067	5.77E-65	4.03E-64	cyan	0.730483441	1.86E-27		AT5G07270.1	XBAT33	XB3 ortholog 3 in Arabidopsis thaliana	E3 ubiquitin-protein ligase
Sapur.005G149800.1	14.29999087	3.104223739	0.182682542	16.99245	9.34E-65	6.49E-64	cyan	0.57904529	1.96E-15	GO:0030170,GO:0019752,GO:0016831	AT1G43710.1	emb1075	Pyridoxal phosphate (PLP)-dependent transferases superfamily protein	serine decarboxylase
Sapur.001G082300.1	415.9950099	1.556999569	0.091718628	16.97583	1.24E-64	8.60E-64	cyan	0.663059182	3.07E-21	GO:0006355,GO:0003677	AT5G41410.1	BEL1	POX (plant homeobox) family protein	BEL1-related homeotic protein, putative
Sapur.011G081100.1	760.7983898	1.403216818	0.082732174	16.96096	1.60E-64	1.11E-63	cyan	0.680525529	1.09E-22		AT1G64980.1		Nucleotide-diphospho-sugar transferases superfamily protein	hypothetical protein
Sapur.013G059700.1	175.6675257	1.20542641	0.071113723	16.95069	1.90E-64	1.32E-63	cyan	0.758698625	1.19E-30	GO:0016020,GO:0015171,GO:0003333	AT1G58030.1	CAT2	cationic amino acid transporter 2	NA
Sapur.009G067000.1	259.9741728	3.782385445	0.223474297	16.92537	2.92E-64	2.02E-63	cyan	0.568852116	7.70E-15	GO:0008283,GO:0008083,GO:0005576	AT3G49780.1	ATPSK3 (FORMER SYMBOL)	phytosulfokine 4 precursor	phytosulfokine peptide protein, putative
Sapur.006G039800.1	287.6603457	0.782349728	0.046264678	16.9103	3.78E-64	2.60E-63	cyan	0.75564129	2.76E-30	GO:0008270	AT3G57480.1		zinc finger (C2H2 type, AN1-like) family protein	NA
Sapur.002G108000.1	297.4679732	1.030119162	0.061109383	16.85697	9.32E-64	6.38E-63	cyan	0.746622031	3.11E-29	GO:0008270	AT1G19270.1	DA1	DA1	zinc ion-binding protein, putative
Sapur.003G060400.1	415.2835803	6.232033746	0.370533767	16.81907	1.77E-63	1.20E-62	cyan	0.46319194	1.01E-09	GO:0055114,GO:0050113,GO:0019310,GO:0005737,GO:0005506	AT2G19800.1	MIOX2	myo-inositol oxygenase 2	Myo-inositol oxygenase
Sapur.005G018400.1	1372.543357	0.845779497	0.050338652	16.80179	2.37E-63	1.61E-62	cyan	0.76995142	4.74E-32		AT3G16640.1	TCTP	translationally controlled tumor protein	translationally-controlled tumor-like protein
Sapur.008G111700.1	24.23232058	3.506320815	0.20920177	16.76047	4.75E-63	3.21E-62	cyan	0.607610268	3.24E-17	GO:0016567,GO:0004842	AT5G37490.1		ARM repeat superfamily protein	Avr9/Cf-9 rapidly elicited protein
Sapur.018G078300.1	739.0942466	0.711019272	0.04248022	16.73765	6.97E-63	4.70E-62	cyan	0.760419253	7.34E-31		AT2G24100.1			hypothetical protein
Sapur.009G053000.1	245.7638847	2.903395112	0.174126315	16.67407	2.02E-62	1.36E-61	cyan	0.567560685	9.13E-15	GO:0005515	AT2G32560.1		F-box family protein	F-box plant protein
Sapur.016G253700.1	1717.163097	1.907111968	0.114607527	16.64037	3.55E-62	2.37E-61	cyan	0.559852102	2.48E-14		AT3G14440.1	ATNCED3.NCED3.SIS7.STO1	nine-cis-epoxycarotenoid dioxygenase 3	9-cis-epoxycarotenoid dioxygenase, putative
Sapur.005G018600.1	123.5952514	4.059111361	0.244203906	16.62181	4.84E-62	3.23E-61	cyan	0.499737456	2.68E-11	GO:0016788	AT1G28650.1		GDSL-like Lipase/Acylhydrolase superfamily protein	GDSL-like lipase/acylhydrolase
Sapur.003G040200.1	14.26983746	7.136167183	0.4293913	16.61926	5.05E-62	3.37E-61	cyan	0.498404441	3.08E-11		AT1G54070.1		Dormancy/auxin associated family protein	NA
Sapur.002G138800.1	175.9654041	1.777280242	0.106959035	16.61646	5.30E-62	3.53E-61	cyan	0.696048143	4.56E-24	GO:0003676	AT2G46780.1		RNA-binding (RRM/RBD/RNP motifs) family protein	RNA recognition motif 2 in RNA-binding protein musashi-like musashi protein
Sapur.018G043700.1	396.6019586	0.760535843	0.045782641	16.61188	5.72E-62	3.80E-61	cyan	0.74208528	1.01E-28	GO:0016021,GO:0015693,GO:0015095	AT5G11960.1		Protein of unknown function (DUF803)	magnesium transporter NIPA protein
Sapur.014G005700.1	471.7359478	0.917638701	0.055248501	16.6093	5.97E-62	3.97E-61	cyan	0.737449957	3.30E-28		AT1G10720.1		BSD domain-containing protein	NA
Sapur.014G077500.1	1193.409455	1.339659241	0.08073331	16.59364	7.75E-62	5.15E-61	cyan	0.684751739	4.67E-23	GO:0005515,GO:0016773	AT2G46500.2	ATPI4K GAMMA 4.PI4K GAN	phosphoinositide 4-kinase gamma 4	phosphatidylinositol 3- and 4-kinase family protein
Sapur.019G041000.1	314.3444192	0.94944137	0.05722219	16.59219	7.94E-62	5.27E-61	cyan	0.732449183	1.15E-27		AT3G02910.1		AIG2-like (avirulence induced gene) family protein	AIG2 family protein
Sapur.014G076700.1	14.86455849	2.946199304	0.179194714	16.44133	9.68E-61	6.32E-60	cyan	0.623099318	2.93E-18	GO:0008270,GO:0005515	AT5G53110.1		RING/U-box superfamily protein	RING-H2 finger protein ATL22
Sapur.003G167300.1	5144.067819	1.345045217	0.081887759	16.42547	1.26E-60	8.20E-60	cyan	0.699854216	2.03E-24		AT5G56540.1	AGP14,ATAGP14	arabinogalactan protein 14	NA
Sapur.005G061500.1	87.96908584	1.730311604	0.105528397	16.39664	2.02E-60	1.31E-59	cyan	0.64652752	5.93E-20					NA
Sapur.15WG026400.1	50.82357004	1.644537776	0.100339843	16.38968	2.27E-60	1.47E-59	cyan	0.691713606	1.13E-23		AT1G17950.1	ATMYB52,BW52,MYB52	myb domain protein 52	R2R3-Myb transcription factor MYB3
Sapur.005G019100.1	2266.494538	1.447120601	0.088402652	16.36965	3.15E-60	2.04E-59	cyan	0.668021891	1.21E-21		AT3G05500.1		Rubber elongation factor protein (REF)	rubber elongation factor
Sapur.016G014500.1	1404.210606	0.613924042	0.037561922	16.34432	4.77E-60	3.09E-59	cyan	0.77124496	3.23E-32	GO:0009058,GO:0003824	AT2G32260.1	ATCCT1,CCT1	phosphorylcholine cytidylyltransferase	choline-phosphate cytidylyltransferase
Sapur.006G030000.1	20.69201219	2.161433365	0.132248901	16.34368	4.83E-60	3.12E-59	cyan	0.698093626	2.96E-24		AT2G41290.1	SSL2	strictosidine synthase-like 2	NA
Sapur.011G053300.1	305.86572	1.947425371	0.119468267	16.30078	9.74E-60	6.27E-59	cyan	0.63025301	9.22E-19		AT1G29640.1		Protein of unknown function, DUF584	senescence regulator
Sapur.004G172300.1	114.9246784	1.258517734	0.077441271	16.25125	2.19E-59	1.40E-58	cyan	0.727176302	4.13E-27	GO:0045454,GO:0015035,GO:0006662	AT3G08710.2	ATH9,TH9,TRX H9	thioredoxin H-type 9	thioredoxin H-type 9 protein
Sapur.012G072100.1	4.208277509	5.412834649	0.334263551	16.19331	5.62E-59	3.57E-58	cyan	0.657422656	8.60E-21					NA
Sapur.006G085300.1	579.3108127	1.353915867	0.083673158	16.18101	6.87E-59	4.35E-58	cyan	0.673046311	4.67E-22		AT2G38360.1	PRA1.B4	prenylated RAB acceptor 1.B4	prenylated rab acceptor family protein
Sapur.011G091500.1	873.5353305	1.906547767	0.117887523	16.1726	7.87E-59	4.98E-58	cyan	0.622258702	3.35E-18		AT5G54240.1		Protein of unknown function (DUF1223)	membrane lipoprotein lipid attachment site-like protein, putative
Sapur.016G199400.1	474.1700317	0.999664831	0.061975271	16.13006	1.57E-58	9.89E-58	cyan	0.70961872	2.41E-25		AT2G33490.1		hydroxyproline-rich glycoprotein family protein	hydroxyproline-rich glycoprotein family protein, putative
Sapur.019G053400.1	784.682364	1.381941899	0.086006777	16.06783	4.29E-58	2.68E-57	cyan	0.653643759	1.70E-20	GO:0006508,GO:0004190	AT3G02740.1		Eukaryotic aspartyl protease family protein	aspartyl protease-like protein
Sapur.005G051500.1	6.135140959	4.069078016	0.253860453	16.0288	8.04E-58	5.01E-57	cyan	0.581301299	1.44E-15		AT1G16260.2		Wall-associated kinase family protein	NA
Sapur.T008100.1	3.787667818	4.841246745	0.302266652	16.01648	9.81E-58	6.10E-57	cyan	0.60747219	3.30E-17					NA
Sapur.006G186500.1	684.9556745	1.000855193	0.062556504	15.99922	1.29E-57	8.04E-57	cyan	0.705028387	6.64E-25		AT1G14560.1		Mitochondrial substrate carrier family protein	graves disease carrier protein
Sapur.006G180400.2	8.088068705	2.588955812	0.162365383	15.94525	3.07E-57	1.90E-56	cyan	0.723646304	9.60E-27		AT4G33180.1		alpha/beta-Hydrolases superfamily protein	NA
Sapur.013G040100.1	230.9651069	3.191983308	0.200246565	15.94027	3.33E-57	2.06E-56	cyan	0.470399015	5.09E-10		AT1G08800.1		Protein of unknown function, DUF593	zein-binding protein
Sapur.002G060800.1	303.2279308	0.906017814	0.056857392	15.93492	3.63E-57	2.24E-56	cyan	0.703634405	9.00E-25	GO:0005515	AT4G08455.1		BTB/POZ domain-containing protein	C2-BTB1+-+bric-A-brac+tramtrack+broad+complex+BTB+domain+with+C2+subfamil protein Y
Sapur.005G151800.1	467.0344843	1.509508257	0.094758806	15.93	3.92E-57	2.42E-56	cyan	0.690278474	1.52E-23		AT1G18740.1		Protein of unknown function (DUF793)	plant/F6A14-15 protein
Sapur.016G088600.1	87.57988041	6.414435087	0.403963414	15.87875	8.89E-57	5.44E-56	cyan	0.434154091	1.34E-08		AT2G39700.1	ATEXP4.ATEXPA4.ATHEXP A	expansin A4	NA
Sapur.019G059300.1	34.4784615	2.702280484	0.170896657	15.81237	2.56E-56	1.56E-55	cyan	0.624615477	2.30E-18					NA
Sapur.001G125200.1	24.11202274	4.177874786	0.265400608	15.74177	7.82E-56	4.72E-55	cyan	0.514634163	5.39E-12	GO:0016020,GO:0015171,GO:0003333	AT5G04770.1	ATCAT6,CAT6	cationic amino acid transporter 6	cationic amino acid transporter, putative
Sapur.008G028100.1	5.592436469	4.377883452	0.27833464	15.72885	9.59E-56	5.78E-55	cyan	0.685259887	4.22E-23	GO:0005515	AT5G04220.2	ATSYTC.NTMC2T1.3.NTMC:	Calcium-dependent lipid-binding (CaLB domain) family protein	calcium lipid-binding protein, putative
Sapur.002G074300.1	6.599676274	5.88226326	0.374850492	15.69229	1.71E-55	1.03E-54	cyan	0.547784021	1.13E-13	GO:0006412,GO:0005840,GO:0003735,GO:0019843	ATCG00790.1		ribosomal protein L16	50S ribosomal protein L16
Sapur.013G011900.1	26.48865382	7.807826563	0.501465697	15.57001	1.16E-54	6.90E-54	cyan	0.438597912	9.16E-09	GO:0004857	AT4G24640.1	APPB1	Plant invertase/pectin methylesterase inhibitor superfamily protein	plant invertase/pectin methylesterase inhibitor protein
Sapur.004G130600.1	1216.891993	1.433265711	0.092065919	15.56782	1.20E-54	7.14E-54	cyan	0.687227222	2.83E-23		AT4G38620.1	ATMYB4,MYB4	myb domain protein 4	MYB transcription factor
Sapur.008G149400.1	145.2816015	2.010310898	0.129174673	15.56273	1.30E-54	7.72E-54	cyan	0.606734055	3.69E-17	GO:0016788	AT3G26430.1		GDSL-like Lipase/Acylhydrolase superfamily protein	GDSL esterase/lipase
Sapur.002G123900.1	138.5570873	0.879955641	0.05683468	15.48272	4.54E-54	2.67E-53	cyan	0.735508533	5.37E-28	GO:0016746,GO:0008152	AT2G45670.1		calcineurin B subunit-related	1-acylglycerophosphocholine O-acyltransferase
Sapur.001G044100.1	7.45536224	6.191175659	0.402827341	15.3693	2.63E-53	1.53E-52	cyan	0.445070882	5.21E-09					NA
Sapur.007G107900.1	264.9742914	2.634989356	0.171910173	15.32771	4.99E-53	2.89E-52	cyan	0.69574773	4.86E-24	GO:0008270,GO:0005622	AT4G15248.1		B-box type zinc finger family protein	B-box type zinc finger protein
Sapur.007G105000.1	23.998931	2.756166513	0.179834746	15.32611	5.12E-53	2.96E-52	cyan	0.592510523	2.98E-16	GO:0016829,GO:0010333,GO:0008152,GO:0000287	AT4G16740.1	ATTPS03,TPS03	terpene synthase 03	(E)-beta-ocimene synthase
Sapur.013G028400.1	466.9932572	1.552835247	0.10153381	15.29378	8.41E-53	4.84E-52	cyan	0.667982933	1.22E-21	GO:0006468,GO:0004672	AT3G05140.1	RBK2	ROP binding protein kinases 2	cysteine-rich receptor-like kinase
Sapur.019G016300.1	162.0066838	1.315198705	0.08613391	15.26923	1.23E-52	7.04E-52	cyan	0.688843842	2.04E-23		AT1G34320.1		Protein of unknown function (DUF668)	DUF668 family protein
Sapur.016G310800.1	59.27249051	2.233422739	0.146282772	15.26785	1.25E-52	7.19E-52	cyan	0.605187953	4.66E-17		AT5G37010.1			NA
Sapur.017G057800.1	11.82929359	2.64964996	0.173820268	15.24362	1.82E-52	1.04E-51	cyan	0.591789964	3.30E-16		AT3G28150.1	TBL22	TRICHOME BIREFRINGENCE-LIKE 22	Pmr5/Cas1p GDSL/SGNH-like acyl-esterase family protein
Sapur.016G151700.1	27.14381748	6.231684855	0.408891599	15.24043	1.91E-52	1.09E-51	cyan	0.46902959	5.80E-10		AT3G57450.1			hypothetical protein
Sapur.012G074800.1	196.5610127	1.225818125	0.08063566	15.20194	3.43E-52	1.95E-51	cyan	0.620559332	4.38E-18		AT2G25560.1		DNAJ heat shock N-terminal domain-containing protein	NA
Sapur.15ZG040500.1	149.2998366	0.93041976	0.061459171	15.13883	8.98E-52	5.06E-51	cyan	0.745586527	4.09E-29	GO:0007067,GO:0007049,GO:0005634,GO:0000775	AT5G35520.1	ATMIS12,MIS12	minichromosome instability 12 (mis12)-like	kinetochore protein
Sapur.T008200.1	2.994894565	3.991323051	0.264625441	15.08292	2.10E-51	1.18E-50	cyan	0.668167101	1.18E-21					NA
Sapur.003G155300.1	34.24022919	2.260098371	0.150951834	14.97231	1.11E-50	6.18E-50	cyan	0.597298959	1.49E-16		AT1G29050.1	TBL38	TRICHOME BIREFRINGENCE-LIKE 38	Pmr5/Cas1p GDSL/SGNH-like acyl-esterase family protein
Sapur.002G102600.1	193.4735437	1.105028123	0.073840282	14.96511	1.24E-50	6.89E-50	cyan	0.719861073	2.34E-26		AT4G17650.1		Polyketide cyclase / dehydrase and lipid transport protein	polyketide cyclase/dehydrase
Sapur.014G105400.2	11.06005431	2.419920403	0.161706818	14.96486	1.25E-50	6.91E-50	cyan	0.683528808	5.97E-23		AT4G15802.1	AtHSBP,HSBP	heat shock factor binding protein	NA
Sapur.008G172400.1	943.8710789	1.422783463	0.095101614	14.96067	1.33E-50	7.35E-50	cyan	0.646665863	5.79E-20		AT4G02040.1			proline-rich nuclear receptor coactivator protein
Sapur.006G128500.1	403.203561	1.439014232	0.096406427	14.92654	2.21E-50	1.22E-49	cyan	0.691872739	1.09E-23		AT1G18740.1		Protein of unknown function (DUF793)	NA
Sapur.010G116100.1	22.85817374	2.559608329	0.171802118	14.89858	3.37E-50	1.85E-49	cyan	0.52841096	1.14E-12	GO:0055114,GO:0016491	AT1G47990.1	ATGA2OX4,GA2OX4	gibberellin 2-oxidase 4	gibberellin 2-oxidase
Sapur.006G055400.1	2305.523826	1.817156565	0.122129607	14.87892	4.52E-50	2.48E-49	cyan	0.638481962	2.35E-19	GO:0005515	AT2G25490.1	EBF1,FBL6	EIN3-binding F box protein 1	Ein3-binding F-box protein
Sapur.016G169200.1	450.8366471	0.844322918	0.056808469	14.86262	5.76E-50	3.16E-49	cyan	0.699314211	2.28E-24	GO:0055114,GO:0050661,GO:0033014,GO:0008883	AT1G58290.1	HEMA1	Glutamyl-tRNA reductase family protein	glutamyl-tRNA reductase
Sapur.010G026200.1	378.9995993	1.068698523	0.071969131	14.8494	7.02E-50	3.84E-49	cyan	0.664699202	2.26E-21		AT1G10740.4		alpha/beta-Hydrolases superfamily protein	NA
Sapur.011G066000.1	600.00147	0.770444108	0.051923935	14.83794	8.33E-50	4.55E-49	cyan	0.731896563	1.31E-27	GO:0016773,GO:0016020,GO:0005524	AT3G14720.1	ATMPK19,MPK19	MAP kinase 19	MAP kinase
Sapur.006G206800.1	18.15786605	3.671225226	0.248524462	14.77209	2.22E-49	1.20E-48	cyan	0.625895575	1.87E-18	GO:0016021,GO:0006820,GO:0016020,GO:0005452	AT1G15460.1	ATBOR4,BOR4	HCO3- transporter family	anion exchanger family protein
Sapur.010G148100.1	8.985798074	3.632431506	0.246917625	14.71111	5.47E-49	2.95E-48	cyan	0.613993315	1.22E-17	GO:0035556	AT2G40116.1		Phosphoinositide-specific phospholipase C family protein	NA
Sapur.001G003000.1	2094.876182	1.214594824	0.082645634	14.69642	6.80E-49	3.66E-48	cyan	0.655616825	1.19E-20					transmembrane protein, putative
Sapur.016G235000.1	29.57453799	2.44647732	0.166498642	14.69368	7.08E-49	3.81E-48	cyan	0.672142518	5.55E-22	GO:0055085	AT4G26590.1	ATOPT5,OPT5	oligopeptide transporter 5	oligopeptide transporter OPT family protein
Sapur.019G087300.1	886.2933676	0.848496543	0.057952905	14.64114	1.53E-48	8.22E-48	cyan	0.738168741	2.75E-28	GO:0043565,GO:0006355,GO:0003700	AT3G58120.1	ATBZIP61,BZIP61	Basic-leucine zipper (bZIP) transcription factor family protein	bZIP transcription factor
Sapur.008G118500.1	13.86352611	4.469583503	0.305297919	14.64007	1.56E-48	8.35E-48	cyan	0.538428226	3.51E-13					NA
Sapur.008G166100.1	41.8850906	5.613778876	0.383720315	14.62987	1.81E-48	9.69E-48	cyan	0.492275536	5.82E-11					NA
Sapur.006G094100.1	8749.289929	0.75458785	0.051610652	14.62078	2.07E-48	1.11E-47	cyan	0.704812573	6.96E-25	GO:0055114,GO:0016491	AT3G52880.1	ATMDAR1,MDAR1	monodehydroascorbate reductase 1	monodehydroascorbate reductase
Sapur.006G041000.1	1055.963905	0.77940417	0.053392638	14.5976	2.91E-48	1.55E-47	cyan	0.725419925	6.30E-27		AT3G57550.2	AGK2,GK-2	guanylate kinase	guanylate kinase
Sapur.014G083000.1	446.1622675	1.528284559	0.104762811	14.58804	3.35E-48	1.78E-47	cyan	0.619008499	5.59E-18	GO:0003676	AT2G46780.1		RNA-binding (RRM/RBD/RNP motifs) family protein	RNA recognition motif 2 in RNA-binding protein musashi-like musashi protein
Sapur.019G020300.1	264.4105954	1.035672667	0.071083149	14.56988	4.37E-48	2.32E-47	cyan	0.660993061	4.49E-21		AT2G18465.1		Chaperone DnaJ-domain superfamily protein	hypothetical protein

Sapur.017G081700.1	449.671535	1.474369783	0.101273161	14.55835	5.17E-48	2.74E-47	cyan	0.643094735	1.07E-19		AT3G02300.1		Regulator of chromosome condensation (RCC1) family protein	chromosome condensation regulator repeat protein
Sapur.002G028500.1	156.7237321	1.472758534	0.101490614	14.51128	1.03E-47	5.42E-47	cyan	0.60984193	2.31E-17	GO:0006355,GO:0003677	AT4G28530.1	anac074,NAC074	NAC domain containing protein 74	NAC transcription factor 009
Sapur.010G095600.1	6.146685761	3.326264073	0.23000349	14.4618	2.11E-47	1.11E-46	cyan	0.617978686	6.58E-18		AT1G68510.1	LBD42	LOB domain-containing protein 42	LOB domain protein
Sapur.T183500.1	216.9204324	0.933376072	0.064596369	14.44936	2.53E-47	1.32E-46	cyan	0.747851461	2.25E-29	GO:0055085,GO:0016020,GO:0006810,GO:0015171,GO:0003333	AT1G58030.1	CAT2	cationic amino acid transporter 2	NA
Sapur.002G045200.1	7.105148233	2.929224531	0.202888871	14.43758	3.00E-47	1.57E-46	cyan	0.636402613	3.33E-19		AT2G40260.1		Homeodomain-like superfamily protein	NA
Sapur.010G015300.1	24.44406363	6.246174995	0.432816763	14.43145	3.28E-47	1.71E-46	cyan	0.448431157	3.87E-09	GO:0016021	AT2G47770.1	ATTSPO,TSPO	TSPO(outer membrane tryptophan-rich sensory protein)-related	peripheral-type benzodiazepine receptor, putative
Sapur.012G057500.1	381.0262009	0.925185189	0.064115223	14.43004	3.35E-47	1.75E-46	cyan	0.704816198	6.96E-25		AT3G48030.1		hypoxia-responsive family protein / zinc finger (C3HC4-type RING finger) family protein	NA
Sapur.010G026000.1	137.7219979	0.982184241	0.068160717	14.40983	4.49E-47	2.34E-46	cyan	0.64853697	4.18E-20		AT1G10740.3		alpha/beta-Hydrolases superfamily protein	NA
Sapur.013G064400.1	225.3684368	1.602282235	0.111720384	14.3419	1.20E-46	6.20E-46	cyan	0.633224503	5.66E-19	GO:0046872	AT3G02830.1	ZFN1	zinc finger protein 1	zinc finger CCCH domain protein
Sapur.014G012700.1	765.6501229	0.951059012	0.066519369	14.29747	2.27E-46	1.17E-45	cyan	0.692274169	1.00E-23		AT4G37260.1	ATMYB73,MYB73	myb domain protein 73	R2R3-MYB transcription factor, putative
Sapur.016G171400.1	24.65862956	5.115431485	0.358748548	14.2591	3.93E-46	2.02E-45	cyan	0.401484868	1.87E-07		AT2G14610.1	ATPR1,PR 1,PR1	pathogenesis-related gene 1	pathogenesis-related protein
Sapur.003G076900.1	184.2262856	1.547252277	0.108535939	14.25567	4.13E-46	2.12E-45	cyan	0.680078218	1.19E-22	GO:0016020,GO:0006810,GO:0005215	AT1G12110.1	ATNRT1.B-1.CHL1.CHL1-1.N	nitrate transporter 1.1	nitrate transporter
Sapur.001G076600.1	194.9701955	1.683141119	0.118924519	14.15302	1.79E-45	9.08E-45	cyan	0.567936115	8.69E-15		AT5G41800.1		Transmembrane amino acid transporter family protein	proline transporter
Sapur.016G277700.1	3.487506965	4.207625379	0.297337796	14.15099	1.84E-45	9.34E-45	cyan	0.62984258	9.86E-19		AT3G62230.1		F-box family protein	F-box plant protein, putative
Sapur.001G191900.3	56.63836398	1.435648399	0.101705804	14.1157	3.04E-45	1.54E-44	cyan	0.630536643	8.80E-19	GO:0016787	AT4G19420.1		Pectinacetylesterase family protein	pectinacetylesterase
Sapur.15WG046000.1	2.96042812	4.040198889	0.286430533	14.10534	3.52E-45	1.78E-44	cyan	0.661546583	4.05E-21		AT1G68490.1			hypothetical protein
Sapur.006G078200.1	1.799488401	4.078402214	0.291699181	13.98153	2.02E-44	1.01E-43	cyan	0.656208605	1.07E-20		AT3G54100.1		O-fucosyltransferase family protein	GDP-fucose protein O-fucosyltransferase
Sapur.012G055600.1	289.8202235	0.759840626	0.054364331	13.97682	2.16E-44	1.07E-43	cyan	0.679348867	1.37E-22		AT5G62910.1		RING/U-box superfamily protein	anti-virus transcriptional factor, putative
Sapur.016G133900.1	28.70074677	1.744785591	0.124962393	13.96249	2.64E-44	1.31E-43	cyan	0.644737753	8.09E-20		AT1G07530.1	ATGRAS2,GRAS2,SCL14	SCARECROW-like 14	GRAS family transcription factor
Sapur.005G188500.1	218.3761038	2.763920437	0.198469273	13.92619	4.39E-44	2.18E-43	cyan	0.46172108	1.15E-09		AT1G19900.1		glyoxal oxidase-related protein	glyoxal oxidase, putative
Sapur.T053900.1	21.1354222	1.708253324	0.122767993	13.91448	5.17E-44	2.56E-43	cyan	0.648924656	3.91E-20	GO:0006468,GO:0004672	AT1G07650.2		Leucine-rich repeat transmembrane protein kinase	NA
Sapur.011G073200.1	19.79460878	1.491892414	0.107554714	13.87101	9.49E-44	4.67E-43	cyan	0.677260847	2.06E-22	GO:0016020,GO:0008378,GO:0006486	AT1G53290.1		Galactosyltransferase family protein	NA
Sapur.019G017900.1	72.69303689	1.659783676	0.119797184	13.85495	1.19E-43	5.83E-43	cyan	0.664707178	2.26E-21	GO:0005515,GO:0006468,GO:0004672	AT3G14840.2		Leucine-rich repeat transmembrane protein kinase	NA
Sapur.017G118400.1	5632.551929	0.834949422	0.060382196	13.82774	1.73E-43	8.49E-43	cyan	0.644475084	8.46E-20	GO:0016758,GO:0008152	AT3G16520.3	UGT88A1	UDP-glucosyl transferase 88A1	UDP-glycosyltransferase
Sapur.008G106700.1	1061.77665	0.922424823	0.067086709	13.74974	5.11E-43	2.48E-42	cyan	0.65457504	1.44E-20	GO:0005515	AT1G48590.2		Calcium-dependent lipid-binding (CaLB domain) family protein	Arf GTPase activator, putative
Sapur.018G112700.1	24.76002766	4.112211793	0.299722966	13.72004	7.70E-43	3.73E-42	cyan	0.342421416	1.14E-05	GO:0005515	AT1G54470.2	RPP27	RNI-like superfamily protein	LRR protein with ABC domain protein
Sapur.006G230200.1	415.2417552	2.940958808	0.21448623	13.71164	8.65E-43	4.18E-42	cyan	0.656524561	1.01E-20	GO:0006950,GO:0006468,GO:0005524,GO:0004672	AT2G24370.1		Protein kinase protein with adenine nucleotide alpha hydrolases-like domain	NA
Sapur.010G052200.1	466.3216993	1.053102095	0.076860303	13.70151	9.94E-43	4.80E-42	cyan	0.68130344	9.31E-23	GO:0016567,GO:0004842	AT1G24330.1		ARM repeat superfamily protein	U-box protein
Sapur.014G072500.1	1252.260924	0.655453454	0.047962491	13.66596	1.62E-42	7.81E-42	cyan	0.695354821	5.28E-24	GO:0005515	AT3G61600.1	ATPOB1,POB1	POZ/BTB containin G-protein 1	BTB/POZ domain protein POB1
Sapur.004G011800.1	40.26963941	2.450244756	0.179976126	13.61428	3.29E-42	1.58E-41	cyan	0.589771292	4.40E-16	GO:0005975,GO:0004553	AT1G61820.1	BGLU46	beta glucosidase 46	glycoside hydrolase family 1 protein
Sapur.016G029200.1	2.585629984	4.497423588	0.330874802	13.59252	4.44E-42	2.12E-41	cyan	0.547304773	1.20E-13	GO:0007165,GO:0005515	AT5G36930.2		Disease resistance protein (TIR-NBS-LRR class) family	NA
Sapur.013G056600.1	1025.252958	0.813183997	0.05992529	13.56996	6.04E-42	2.88E-41	cyan	0.709247535	2.62E-25	GO:0045454,GO:0015035,GO:0006662	AT1G08570.1	ACHT4	atypical CYS HIS rich thioredoxin 4	thioredoxin-like protein
Sapur.005G074000.1	919.8619424	1.082479831	0.079854131	13.55571	7.33E-42	3.49E-41	cyan	0.627397725	1.47E-18	GO:0005524	AT3G19740.1		P-loop containing nucleoside triphosphate hydrolases superfamily protein	P-loop NTPase domain protein
Sapur.019G019300.1	18.36408042	1.652811322	0.123016772	13.43566	3.74E-41	1.76E-40	cyan	0.666601492	1.59E-21	GO:0006468,GO:0005524,GO:0004672	AT1G07650.2		Leucine-rich repeat transmembrane protein kinase	NA
Sapur.008G102800.1	2559.08067	1.121382778	0.083465528	13.43528	3.76E-41	1.77E-40	cyan	0.629036971	1.12E-18	GO:0009052,GO:0004751	AT2G01290.1	RPI2	ribose-5-phosphate isomerase 2	ribose-5-phosphate isomerase
Sapur.T035600.1	25.75585132	1.277737194	0.095232782	13.41699	4.81E-41	2.26E-40	cyan	0.682762683	6.96E-23		AT2G46530.2	ARF11	auxin response factor 11	NA
Sapur.15WG070000.1	2.755722235	4.727399482	0.35340022	13.3769	8.25E-41	3.87E-40	cyan	0.533173728	6.54E-13	GO:0016021	AT1G18520.1	TET11	tetraspanin11	NA
Sapur.013G000500.1	404.7059171	2.39910864	0.18023268	13.31117	1.99E-40	9.28E-40	cyan	0.531379536	8.06E-13		AT3G62020.1	GLP10	germin-like protein 10	germin-like protein
Sapur.005G151900.1	67.02559368	1.552216888	0.116610237	13.31115	1.99E-40	9.28E-40	cyan	0.635651329	3.78E-19		AT1G76770.1		HSP20-like chaperones superfamily protein	NA
Sapur.001G030900.1	5.256554294	3.403182832	0.255951043	13.29623	2.43E-40	1.13E-39	cyan	0.592249479	3.09E-16	GO:0016021,GO:0004674,GO:0030247,GO:0006468,GO:0005524,GO:0004672	AT1G16130.1	WAKL2	wall associated kinase-like 2	wall-associated receptor kinase-like protein, putative
Sapur.017G090800.1	1.595271329	4.111026024	0.309508191	13.28245	2.93E-40	1.36E-39	cyan	0.634216258	4.80E-19					NA
Sapur.T008900.1	1.59594521	3.629790093	0.273378857	13.27751	3.13E-40	1.45E-39	cyan	0.659217302	6.21E-21	GO:0004518,GO:0006281,GO:0006289,GO:0005634,GO:0004519,GO:0003697	AT3G28030.1	UVH3,UVR1	5\'-3\' exonuclease family protein	NA
Sapur.002G119700.1	891.6186093	1.004283065	0.075705489	13.26566	3.66E-40	1.69E-39	cyan	0.608476026	2.84E-17	GO:0055114,GO:0016616,GO:0006694,GO:0003854	AT4G00110.1	GAE3	UDP-D-glucuronate 4-epimerase 3	UDP-D-glucuronate 4-epimerase
Sapur.003G064700.1	125.1850253	1.830862888	0.138285262	13.23975	5.17E-40	2.39E-39	cyan	0.527118256	1.32E-12					NA
Sapur.006G073900.1	119.987952	1.919402101	0.144986885	13.23845	5.26E-40	2.43E-39	cyan	0.555568113	4.28E-14		AT2G37750.1			legume-specific protein
Sapur.001G190700.1	185.8279959	1.876552706	0.142241006	13.19277	9.66E-40	4.44E-39	cyan	0.566910734	9.94E-15	GO:0006468,GO:0004672	AT5G49760.1		Leucine-rich repeat protein kinase family protein	somatic embryogenesis receptor-like kinase
Sapur.017G069900.1	334.4812751	0.81585211	0.061990956	13.16082	1.47E-39	6.75E-39	cyan	0.677711542	1.89E-22	GO:0006355,GO:0003677	AT5G39660.1	CDF2	cycling DOF factor 2	Dof zinc finger protein
Sapur.004G166500.1	873.2946949	0.724146488	0.055060317	13.15188	1.66E-39	7.59E-39	cyan	0.688669753	2.11E-23	GO:0006886,GO:0005622	AT5G01760.1		ENTH/VHS/GAT family protein	NA
Sapur.006G156400.1	16.00738341	1.971492239	0.149960668	13.14673	1.78E-39	8.12E-39	cyan	0.626430496	1.72E-18		AT2G42140.1		VQ motif-containing protein	VQ motif protein
Sapur.008G054200.1	232.1532237	1.408258311	0.107359785	13.11719	2.63E-39	1.20E-38	cyan	0.505715161	1.42E-11	GO:0006468,GO:0005524,GO:0004672	AT3G55950.1	ATCRR3,CCR3	CRINKLY4 related 3	receptor kinase-like protein
Sapur.016G149100.1	2.621052607	4.842283935	0.369821268	13.09358	3.58E-39	1.63E-38	cyan	0.498070138	3.19E-11					NA
Sapur.017G065900.1	74.87831771	4.248072276	0.324938578	13.07346	4.67E-39	2.11E-38	cyan	0.528458742	1.13E-12	GO:0016788	AT1G71691.2		GDSL-like Lipase/Acylhydrolase superfamily protein	NA
Sapur.010G158100.1	17.32595868	4.230601047	0.324182575	13.05006	6.35E-39	2.87E-38	cyan	0.509028942	9.95E-12	GO:0006508,GO:0004190	AT2G39710.1		Eukaryotic aspartyl protease family protein	aspartic proteinase nepenthesin-1 protein, putative
Sapur.004G023800.1	267.3564446	0.843526021	0.064667819	13.04398	6.88E-39	3.10E-38	cyan	0.663717442	2.72E-21	GO:0005515	AT1G61215.1	BRD4	bromodomain 4	bromodomain protein
Sapur.011G022300.1	14.72233021	3.487567247	0.267454913	13.03983	7.26E-39	3.28E-38	cyan	0.457121155	1.76E-09	GO:0048544,GO:0006468,GO:0004672	AT1G11340.1		S-locus lectin protein kinase family protein	Serine/Threonine-kinase
Sapur.006G070300.1	3.229129982	4.911921174	0.376863588	13.03368	7.87E-39	3.55E-38	cyan	0.55270331	6.14E-14					NA
Sapur.010G160500.2	7.326491569	2.184504483	0.167927023	13.00865	1.09E-38	4.91E-38	cyan	0.500178713	2.56E-11		AT3G12110.1	ACT11	actin-11	actin
Sapur.005G084900.1	717.6683747	0.985553283	0.075855203	12.99256	1.35E-38	6.05E-38	cyan	0.634247573	4.77E-19		AT4G35750.1		SEC14 cytosolic factor family protein / phosphoglyceride transfer family protein	divergent CRAL/TRIO domain protein
Sapur.019G039700.1	7.854213721	2.50438768	0.193098278	12.9695	1.82E-38	8.14E-38	cyan	0.547389242	1.19E-13		AT3G04440.1		Plasma-membrane choline transporter family protein	plasma-membrane choline transporter
Sapur.006G064300.1	47.20936769	2.731296169	0.211006535	12.94413	2.54E-38	1.13E-37	cyan	0.50779423	1.14E-11	GO:0003824	AT2G07050.1	CAS1	cycloartenol synthase 1	oxidosqualene cyclase
Sapur.15WG101500.1	138.2319445	1.454787887	0.112433166	12.93913	2.71E-38	1.21E-37	cyan	0.686820174	3.08E-23	GO:0005515	AT5G50170.1		C2 calcium/lipid-binding and GRAM domain containing protein	C2 and GRAM domain plant protein
Sapur.006G126600.1	985.8626827	0.620066074	0.047924165	12.93848	2.73E-38	1.22E-37	cyan	0.671088403	6.79E-22	GO:0003993	AT4G29260.1		HAD superfamily, subfamily IIIB acid phosphatase	acid phosphatase
Sapur.010G015400.1	9.673543544	6.042028598	0.467334963	12.92869	3.10E-38	1.38E-37	cyan	0.42771293	2.31E-08	GO:0016757,GO:0016021	AT3G62720.2	ATXT1,XT1,XXT1	xylosyltransferase 1	NA
Sapur.005G043200.1	617.6800702	0.781718705	0.060643955	12.8903	5.10E-38	2.27E-37	cyan	0.625550557	1.98E-18		AT1G54460.1		TPX2 (targeting protein for Xklp2) protein family	targeting protein for Xklp2 protein
Sapur.018G011200.1	1910.689398	1.591526548	0.123632018	12.87309	6.38E-38	2.83E-37	cyan	0.581799965	1.34E-15	GO:0006508,GO:0004190	AT5G10770.1		Eukaryotic aspartyl protease family protein	aspartic proteinase nepenthesin protein
Sapur.011G025800.1	230.5072566	2.247060539	0.174883339	12.84891	8.72E-38	3.86E-37	cyan	0.527901805	1.21E-12		AT4G27280.1		Calcium-binding EF-hand family protein	Ccd1, putative
Sapur.016G257600.1	18.04270952	1.890285935	0.147176464	12.84367	9.33E-38	4.13E-37	cyan	0.633403599	5.49E-19	GO:0016020,GO:0008375	AT3G15350.1		Core-2/I-branching beta-1,6-N-acetylglucosaminyltransferase family protein	core-2/I-branching beta-1%2C6-N-acetylglucosaminyltransferase family protein, putative
Sapur.010G194800.1	552.1472522	0.760206107	0.059341064	12.81079	1.43E-37	6.28E-37	cyan	0.655571553	1.20E-20		AT5G04760.1		Duplicated homeodomain-like superfamily protein	myb-like DNA-binding domain protein
Sapur.017G065600.1	161.2533587	4.348286017	0.339802859	12.7965	1.72E-37	7.54E-37	cyan	0.523520969	1.99E-12	GO:0016788	AT1G71691.2		GDSL-like Lipase/Acylhydrolase superfamily protein	GDSL-like lipase/acylhydrolase
Sapur.001G146100.1	169.5109006	0.956321723	0.07516517	12.72294	4.41E-37	1.92E-36	cyan	0.654898116	1.36E-20		AT1G15400.3			NA
Sapur.011G040300.1	29.35366218	2.26415537	0.178356415	12.69456	6.34E-37	2.76E-36	cyan	0.493332895	5.22E-11		AT4G18425.1		Protein of unknown function (DUF679)	transmembrane protein, putative
Sapur.003G060100.1	732.2493794	0.877598966	0.069301214	12.66354	9.42E-37	4.08E-36	cyan	0.602740441	6.70E-17		AT4G17900.1		PLATZ transcription factor family protein	PLATZ transcription factor
Sapur.012G083000.1	39.38162299	2.27897601	0.181509013	12.55572	3.70E-36	1.58E-35	cyan	0.643695137	9.68E-20		AT5G61820.1			stress up-regulated Nod 19 protein
Sapur.002G009800.1	59.26889736	1.588217901	0.126937033	12.51186	6.43E-36	2.74E-35	cyan	0.586610448	6.87E-16		AT2G17030.1		F-box family protein with a domain of unknown function (DUF295)	ubiquitin-protein ligase, putative
Sapur.004G065900.1	96.34273114	1.338791458	0.10704158	12.50721	6.82E-36	2.90E-35	cyan	0.583963058	9.94E-16		AT2G01300.1			by genscan and genefinder, putative
Sapur.004G103500.1	14.34449168	3.480168475	0.278484941	12.49679	7.77E-36	3.30E-35	cyan	0.451668623	2.89E-09	GO:0046983,GO:0003677	AT3G04100.1	AGL57	AGAMOUS-like 57	NA
Sapur.010G182300.1	9.328206289	3.023582086	0.242541223	12.46626	1.14E-35	4.83E-35	cyan	0.523981864	1.89E-12	GO:0005515,GO:0006468,GO:0004672	AT3G47570.1		Leucine-rich repeat protein kinase family protein	receptor-kinase, putative
Sapur.005G136400.1	40.23230893	1.510393333	0.121279872	12.45378	1.33E-35	5.64E-35	cyan	0.617123102	7.52E-18	GO:0016021	AT2G41705.1		camphor resistance CrcB family protein	camphor resistance CrcB family protein
Sapur.004G059400.1	4.593170179	3.213508353	0.25861882	12.42566	1.90E-35	8.00E-35	cyan	0.642816984	1.13E-19	GO:0016788	AT5G40990.1	GLIP1	GDSL lipase 1	GDSL esterase/lipase
Sapur.019G110900.1	1980.939147	0.963797827	0.077612183	12.41813	2.08E-35	8.78E-35	cyan	0.626464405	1.71E-18	GO:0007165	AT2G30360.1	CIPK11.PKS5.SIP4.SNRK3.22	SOS3-interacting protein 4	CBL-interacting Serine/Threonine-kinase, putative
Sapur.011G120000.1	5.048609	5.539492633	0.446153813	12.41611	2.14E-35	9.00E-35	cyan	0.444321464	5.57E-09	GO:0055114,GO:0050660,GO:0016491	AT4G20820.1		FAD-binding Berberine family protein	NA
Sapur.15ZG053700.1	34.00792815	2.309608692	0.186403817	12.39035	2.95E-35	1.24E-34	cyan	0.538782593	3.37E-13	GO:0043565,GO:0006355,GO:0003700	AT5G60830.1	AtbZIP70,bZIP70	basic leucine-zipper 70	bZIP transcription factor
Sapur.005G171500.1	3445.85595	1.50493021	0.121635915	12.37242	3.69E-35	1.54E-34	cyan	0.641996303	1.30E-19		AT1G03055.1			dwarf 27
Sapur.016G306000.1	2.463978612	4.73509805	0.383278499	12.3542	4.62E-35	1.93E-34	cyan	0.495065605	4.37E-11	GO:0046872,GO:0030001	AT3G20180.1		Copper transport protein family	heavy metal-associated domain protein
Sapur.007G093300.1	214.5330216	1.012418675	0.081998995	12.34672	5.07E-35	2.12E-34	cyan	0.640745137	1.60E-19	GO:0008168	AT5G64030.1		S-adenosyl-L-methionine-dependent methyltransferases superfamily protein	methyltransferase PMT26, putative
Sapur.013G118700.1	16.4541135	2.8007248	0.227171748	12.32867	6.35E-35	2.65E-34	cyan	0.486028453	1.10E-10					NA
Sapur.003G120300.1	804.4776512	0.8450131	0.068580609	12.32146	6.94E-35	2.89E-34	cyan	0.650073629	3.19E-20	GO:0006355,GO:0003700	AT5G13330.1	Rap2.6L	related to AP2 6l	AP2/ERF domain transcription factor
Sapur.009G049300.1	810.1153235	0.728181799	0.059169077	12.3068	8.33E-35	3.46E-34	cyan	0.634457618	4.61E-19	GO:0008270,GO:0003677	AT3G12630.1		A20/AN1-like zinc finger family protein	NA
Sapur.013G110500.1	221.0061929	2.095912417	0.170479761	12.2942	9.73E-35	4.04E-34	cyan	0.568391998	8.18E-15	GO:0016747,GO:0008610,GO:0016020,GO:0006633	AT5G04530.1	KCS19	3-ketoacyl-CoA synthase 19	3-ketoacyl-CoA synthase
Sapur.002G114000.1	55.32034927	1.772519192	0.144407832	12.2744	1.24E-34	5.15E-34	cyan	0.546783707	1.28E-13		AT1G28710.1		Nucleotide-diphospho-sugar transferase family protein	nucleotide-diphospho-sugar transferase
Sapur.001G108400.1	39.18494213	1.147483348	0.093949369	12.21385	2.62E-34	1.08E-33	cyan	0.640160207	1.77E-19		AT5G14890.1		NHL domain-containing protein	NA
Sapur.019G021600.1	415.9431443	0.866960633	0.071066948	12.19921	3.14E-34	1.29E-33	cyan	0.668912115	1.03E-21		AT1G08800.1		Protein of unknown function, DUF593	zein-binding protein
Sapur.018G089800.1	235.6813186	0.781238462	0.064259422	12.15757	5.23E-34	2.14E-33	cyan	0.649498541	3.53E-20	GO:0055085,GO:0016021	AT2G13100.1		Major facilitator superfamily protein	glycerol-3-phosphate transporter
Sapur.003G153700.1	389.1244306	0.833579783	0.068700576	12.13352	7.02E-34	2.87E-33	cyan	0.657355073	8.71E-21	GO:0045454,GO:0015035,GO:0009055	AT5G13810.1		Glutaredoxin family protein	glutaredoxin family protein
Sapur.014G122000.2	5.295539349	1.705234429	0.140847016	12.107	9.70E-34	3.95E-33	cyan	0.647495191	5.01E-20	GO:0016021,GO:0004190	AT2G43070.1	ATSPPL3,SPPL3	SIGNAL PEPTIDE PEPTIDASE-LIKE 3	signal peptide peptidase-like protein
Sapur.001G099000.1	1612.585299	1.02543724	0.084772517	12.09634	1.10E-33	4.50E-33	cyan	0.668838521	1.04E-21	GO:0005992,GO:0003824	AT4G22590.1		Haloacid dehalogenase-like hydrolase (HAD) superfamily protein	trehalose-phosphate phosphatase
Sapur.016G301500.1	643.1871696	0.481415372	0.039971621	12.04393	2.09E-33	8.46E-33	cyan	0.671536601	6.24E-22	GO:0055114,GO:0016491,GO:0006633,GO:0005506	AT2G34770.1	ATFAH1,FAH1	fatty acid hydroxylase 1	fatty acid hydroxylase
Sapur.001G008600.1	245.6375578	0.581847605	0.048335412	12.03771	2.25E-33	9.12E-33	cyan	0.657476332	8.52E-21	GO:0007275,GO:0006511,GO:0005634	AT3G58040.1	SINAT2	seven in absentia of Arabidopsis 2	E3 ubiquitin-protein ligase SINAT5
Sapur.007G017000.1	175.2640661	1.20192319	0.10089591	11.91251	1.02E-32	4.07E-32	cyan	0.605023701	4.77E-17	GO:0043565,GO:0006355,GO:0003700	AT1G75390.1	AtbZIP44,bZIP44	basic leucine-zipper 44	BZIP transcription factor
Sapur.005G186000.1	407.8069418	0.80635108	0.067711525	11.90862	1.07E-32	4.27E-32	cyan	0.631830058	7.12E-19	GO:0008234,GO:0006508	AT1G09850.1	XBCP3	xylem bark cysteine peptidase 3	cysteine protease, putative
Sapur.004G129000.1	180.1017333	1.118769452	0.094118528	11.88681	1.39E-32	5.53E-32	cyan	0.604055884	5.51E-17		AT4G34940.1	ARO1	armadillo repeat only 1	armadillo repeat only 1 protein
Sapur.006G077500.2	16.12133463	1.610530438	0.135748647	11.86406	1.82E-32	7.24E-32	cyan	0.603917352	5.63E-17		AT3G54040.1		PAR1 protein	PAR1 protein
Sapur.007G088200.1	3.016681012	3.064344303	0.259149293	11.82463	2.91E-32	1.15E-31	cyan	0.612373046	1.57E-17		AT2G46550.2			transmembrane protein, putative
Sapur.15ZG044800.1	41.68384148	2.622418984	0.222165254	11.80391	3.73E-32	1.47E-31	cyan	0.436559715	1.09E-08		AT1G68490.1			NA
Sapur.004G059500.1	2.381883104	3.126970591	0.26492464	11.80325	3.76E-32	1.48E-31	cyan	0.670041311	8.29E-22	GO:0016788	AT1G53990.1	GLIP3	GDSL-motif lipase 3	GDSL esterase/lipase
Sapur.014G025000.1	684.2538543	1.017418572	0.086280162	11.79203	4.29E-32	1.69E-31	cyan	0.643534865	9.95E-20		AT5G42680.2		Protein of unknown function, DUF617	plant-specific domain TIGR01570 family protein
Sapur.016G164900.1	2.063444308	3.628513856	0.307734021	11.79107	4.34E-32	1.71E-31	cyan	0.565556271	1.19E-14					NA
Sapur.003G105200.1	89.91005517	1.159357993	0.098511053	11.76881	5.65E-32	2.22E-31	cyan	0.547757187	1.13E-13	GO:0008270,GO:0005515	AT4G24015.1		RING/U-box superfamily protein	RING-H2 zinc finger protein
Sapur.011G020300.1	20.7647975	1.747039947	0.148551376	11.76051	6.24E-32	2.45E-31	cyan	0.532903441	6.75E-13		AT1G61660.1		basic helix-loop-helix (bHLH) DNA-binding superfamily protein	transcription factor bHLH123
Sapur.004G175800.1	898.751045	0.517449589	0.044031154	11.7519	6.91E-32	2.71E-31	cyan	0.64496065	7.78E-20		AT3G54190.1		Transducin/WD40 repeat-like superfamily protein	plant/F24B22-150 protein
Sapur.012G001800.1	58.88884803	1.033769561	0.088381946	11.69661	1.33E-31	5.18E-31	cyan	0.624454306	2.36E-18	GO:0005515	AT1G47890.1	AtRLP7,RLP7	receptor like protein 7	NA
Sapur.008G138300.1	1502.856459	1.167284301	0.100196687	11.64993	2.30E-31	8.90E-31	cyan	0.52544406	1.60E-12	GO:0016020,GO:0006810,GO:0005215	AT5G46050.1	ATPTR3,PTR3	peptide transporter 3	peptide transporter PTR3-A
Sapur.018G111000.1	2.968502397	4.314863838	0.370715137	11.6393	2.60E-31	1.01E-30	cyan	0.464127261	9.22E-10		AT2G34400.1		Pentatricopeptide repeat (PPR-like) superfamily protein	NA
Sapur.009G001700.1	201.7978433	0.76729964	0.065949216	11.6347	2.75E-31	1.06E-30	cyan	0.620748255	4.25E-18	GO:0004519,GO:0003676	AT3G47490.1		HNH endonuclease	HNH endonuclease domain protein
Sapur.016G220200.1	132.234084	1.140440398	0.098148525	11.61954	3.28E-31	1.27E-30	cyan	0.628233558	1.28E-18	GO:0045454,GO:0015035,GO:0009055	AT5G40370.1		Glutaredoxin family protein	glutaredoxin (GRX) family protein
Sapur.007G010300.2	12.86566878	-1.355870403	0.116754501	-11.613	3.54E-31	1.37E-30	cyan	0.492265163	5.83E-11	GO:0006355	AT5G45600.2	GAS41,TAF14B	YEATS family protein	YEATS domain protein, putative
Sapur.016G158700.2	2.060520007	4.035757026	0.347655949	11.60848	3.73E-31	1.44E-30	cyan	0.536649609	4.34E-13					NA
Sapur.001G027700.1	633.2192906	0.456938822	0.03936407	11.60802	3.75E-31	1.45E-30	cyan	0.651562983	2.45E-20	GO:0015031,GO:0006464	AT3G12400.1	ATELC,ELC	Ubiquitin-conjugating enzyme/RWD-like protein	ATELC
Sapur.016G074500.1	194.756233	1.358801697	0.117115602	11.60223	4.01E-31	1.55E-30	cyan	0.591821518	3.29E-16		AT3G55140.1		Pectin lyase-like superfamily protein	NA
Sapur.005G104200.1	8.07399979	4.472772436	0.386403238	11.5754	5.49E-31	2.11E-30	cyan	0.379597555	9.43E-07	GO:0005515	AT3G50390.1		Transducin/WD40 repeat-like superfamily protein	NA
Sapur.006G149200.1	92.66495913	1.056913725	0.091366865	11.5678	6.00E-31	2.30E-30	cyan	0.645476678	7.12E-20	GO:0055114,GO:0009186	AT3G23580.1	RNR2,RNR2A	ribonucleotide reductase 2A	NA
Sapur.008G049900.1	9.257777919	1.839320337	0.159348604	11.54275	8.03E-31	3.08E-30	cyan	0.602153996	7.31E-17					hypothetical protein
Sapur.009G016500.1	100.6114893	0.667115624	0.057810351	11.53973	8.32E-31	3.19E-30	cyan	0.645045166	7.67E-20	GO:0005515	AT2G44430.1		DNA-binding bromodomain-containing protein	DNA-binding bromodomain protein, putative
Sapur.008G167100.2	75.1771539	1.067396603	0.092539188	11.53454	8.84E-31	3.38E-30	cyan	0.650540768	2.94E-20	GO:0016161,GO:0000272	AT5G18670.1	BAM9,BMY3	beta-amylase 3	beta-amylase
Sapur.008G026800.1	117.6433739	1.154857153	0.100176782	11.52819	9.51E-31	3.63E-30	cyan	0.597567903	1.43E-16	GO:0006468,GO:0005524,GO:0004672	AT3G57120.1		Protein kinase superfamily protein	tyrosine kinase domain protein
Sapur.001G079600.1	354.5674416	0.576057954	0.05019993	11.47527	1.76E-30	6.67E-30	cyan	0.623828334	2.61E-18	GO:0016020	AT5G60750.1		CAAX amino terminal protease family protein	CAAX amino terminal protease family protein
Sapur.15WG064300.1	3.230624129	2.248868856	0.19606309	11.47013	1.86E-30	7.08E-30	cyan	0.636970462	3.03E-19		AT5G61280.1		Remorin family protein	remorin, carboxy-terminal region protein
Sapur.019G068300.1	3.717612984	3.258362058	0.284281844	11.46173	2.05E-30	7.79E-30	cyan	0.575523218	3.16E-15	GO:0007165,GO:0005515,GO:0043531	AT5G17680.1		disease resistance protein (TIR-NBS-LRR class), putative	TIR-NBS-LRR resistance protein
Sapur.014G093300.1	1.728477998	4.186387655	0.36568445	11.44809	2.40E-30	9.10E-30	cyan	0.561896939	1.91E-14		AT1G09330.1			NA
Sapur.T017600.1	9.551816734	2.019757615	0.176846895	11.42094	3.29E-30	1.24E-29	cyan	0.586042068	7.44E-16					NA
Sapur.007G017100.1	80.24113821	1.229613034	0.107850219	11.40112	4.13E-30	1.55E-29	cyan	0.5809873	1.50E-15		AT1G75388.1	CPuORF5	conserved peptide upstream open reading frame 5	peptide upstream ORF protein
Sapur.001G019600.1	1009.424378	0.483774575	0.042515771	11.37871	5.34E-30	2.01E-29	cyan	0.657130906	9.07E-21		AT2G41430.5	CID1,ERD15,LSR1	dehydration-induced protein (ERD15)	dehydration-induced protein, putative
Sapur.T105500.1	28.89156982	1.165445283	0.102523631	11.36758	6.06E-30	2.28E-29	cyan	0.638973823	2.16E-19					NA
Sapur.T125400.1	476.4831407	1.062738141	0.09358502	11.35586	6.94E-30	2.60E-29	cyan	0.633467864	5.43E-19		AT1G50140.1		P-loop containing nucleoside triphosphate hydrolases superfamily protein	NA
Sapur.016G154500.1	10.46674658	1.443780366	0.127706973	11.30542	1.23E-29	4.60E-29	cyan	0.594880357	2.12E-16					NA
Sapur.008G159000.1	316.416266	0.428735337	0.038129949	11.24406	2.48E-29	9.19E-29	cyan	0.653358198	1.78E-20		AT4G16100.1		Protein of unknown function (DUF789)	DUF789 family protein
Sapur.005G038500.1	4.894466998	4.576783953	0.407572661	11.22937	2.93E-29	1.08E-28	cyan	0.441897857	6.88E-09		AT1G54530.1		Calcium-binding EF hand family protein	calcium-binding EF hand protein
Sapur.016G084500.1	21.16833213	4.16260413	0.370749514	11.22754	2.99E-29	1.11E-28	cyan	0.314923848	5.89E-05					hypothetical protein
Sapur.011G124500.1	668.1161855	0.364231319	0.032445476	11.22595	3.04E-29	1.13E-28	cyan	0.662023021	3.71E-21	GO:0005515	AT3G13340.1		Transducin/WD40 repeat-like superfamily protein	transducin/WD40 domain protein
Sapur.003G013200.1	27.84063096	3.867739446	0.344799523	11.21736	3.35E-29	1.24E-28	cyan	0.405065674	1.42E-07					hypothetical protein
Sapur.019G091600.1	53.1812422	1.352052552	0.120994703	11.17448	5.44E-29	2.00E-28	cyan	0.618073911	6.48E-18		AT5G49960.1			ion channel castor, putative
Sapur.001G080000.1	940.6236845	1.223191744	0.109525097	11.16814	5.84E-29	2.15E-28	cyan	0.517227193	4.04E-12	GO:0005975,GO:0004553	AT1G13130.1		Cellulase (glycosyl hydrolase family 5) protein	hydrolyzing O-glycosyl compounds hydrolase, putative
Sapur.016G162400.1	2.257037874	4.510506459	0.406009003	11.10938	1.13E-28	4.13E-28	cyan	0.509312944	9.64E-12		AT1G07175.1			transmembrane protein, putative
Sapur.010G086400.1	37.69776797	1.659420411	0.149418913	11.10583	1.18E-28	4.30E-28	cyan	0.507809029	1.13E-11		AT4G38620.1	ATMYB4,MYB4	myb domain protein 4	transcription factor R2R3-MYB2
Sapur.T091800.1	9.711305699	1.270589658	0.114515936	11.09531	1.32E-28	4.83E-28	cyan	0.642850337	1.12E-19		AT5G40670.1		PQ-loop repeat family protein / transmembrane family protein	cystinosin, putative
Sapur.016G090300.4	5.022575451	1.644977029	0.148471039	11.07945	1.58E-28	5.76E-28	cyan	0.419251964	4.63E-08		AT5G02390.1		Protein of unknown function (DUF3741)	DUF4378 domain protein
Sapur.006G128600.1	41.95336236	1.711354835	0.154523795	11.07502	1.66E-28	6.05E-28	cyan	0.557250088	3.46E-14		AT1G76770.1		HSP20-like chaperones superfamily protein	HSP20/alpha crystallin family protein
Sapur.010G182200.1	6.441792155	2.956300326	0.267413783	11.05515	2.07E-28	7.53E-28	cyan	0.494055607	4.85E-11	GO:0005515,GO:0006468,GO:0005524,GO:0004672	AT3G47570.1		Leucine-rich repeat protein kinase family protein	receptor-kinase, putative
Sapur.017G003800.1	1409.318178	0.787150872	0.071238098	11.04958	2.20E-28	8.00E-28	cyan	0.643682175	9.70E-20	GO:0006468,GO:0004672	AT2G43850.1		Integrin-linked protein kinase family	integrin-linked kinase-like protein, putative
Sapur.006G060400.1	182.3856367	1.075196386	0.097866063	10.98641	4.44E-28	1.61E-27	cyan	0.523979327	1.89E-12	GO:0046983	AT5G57150.1		basic helix-loop-helix (bHLH) DNA-binding superfamily protein	transcription factor bHLH protein
Sapur.008G096900.1	18.28309607	2.631982231	0.240172658	10.95871	6.04E-28	2.17E-27	cyan	0.479358831	2.13E-10	GO:0050662,GO:0003824	AT1G68540.1		NAD(P)-binding Rossmann-fold superfamily protein	dihydroflavonol 4-reductase, putative
Sapur.006G018000.1	34.74646349	2.320598285	0.211990129	10.94673	6.89E-28	2.48E-27	cyan	0.417235801	5.44E-08	GO:0016758,GO:0008152	AT1G22400.1	ATUGT85A1,UGT85A1	UDP-Glycosyltransferase superfamily protein	UDP-glucosyl transferase
Sapur.001G095200.1	661.1140145	0.809785238	0.073991503	10.9443	7.08E-28	2.55E-27	cyan	0.580258476	1.66E-15	GO:0055114,GO:0016491,GO:0006633,GO:0005506	AT4G12110.1	ATSMO1.ATSMO1-1.SMO1-	sterol-4alpha-methyl oxidase 1-1	C-4 methylsterol oxidase
Sapur.012G004500.1	4.520646583	5.26241273	0.48277508	10.90034	1.15E-27	4.11E-27	cyan	0.377316003	1.11E-06					NA
Sapur.002G006300.1	9575.056	0.38016722	0.034969476	10.8714	1.58E-27	5.64E-27	cyan	0.641349188	1.45E-19	GO:0055114,GO:0020037,GO:0004096,GO:0006979	AT4G35090.1	CAT2	catalase 2	catalase
Sapur.011G079000.3	187.3864524	1.214849832	0.11201871	10.84506	2.10E-27	7.50E-27	cyan	0.520453348	2.82E-12	GO:0030170,GO:0009058	AT5G53970.1		Tyrosine transaminase family protein	tyrosine aminotransferase
Sapur.001G067900.1	11.80311074	3.999049305	0.369135175	10.83356	2.39E-27	8.49E-27	cyan	0.500667509	2.43E-11	GO:0055114,GO:0020037,GO:0016705,GO:0005506	AT2G46660.1	CYP78A6	cytochrome P450, family 78, subfamily A, polypeptide 6	cytochrome P450 family 78 protein
Sapur.003G135400.1	57.30412338	0.747420449	0.069015525	10.82974	2.49E-27	8.84E-27	cyan	0.63477068	4.38E-19	GO:0008270	AT3G24800.1	PRT1	proteolysis 1	NA
Sapur.15ZG045300.1	11.98177331	2.941698931	0.271979412	10.81589	2.89E-27	1.03E-26	cyan	0.433449934	1.42E-08		AT1G68490.1			NA
Sapur.014G073500.1	1344.112238	0.748072934	0.069261513	10.8007	3.42E-27	1.21E-26	cyan	0.597161198	1.52E-16		AT2G46330.1	AGP16,ATAGP16	arabinogalactan protein 16	transmembrane protein, putative
Sapur.001G134400.1	15.57063309	1.977858902	0.183551531	10.7755	4.49E-27	1.59E-26	cyan	0.506203935	1.35E-11	GO:0008152,GO:0003824	AT5G16340.1		AMP-dependent synthetase and ligase family protein	NA
Sapur.005G125000.1	2586.89573	0.747205802	0.069353981	10.7738	4.58E-27	1.62E-26	cyan	0.578677155	2.06E-15	GO:0008234,GO:0006508	AT5G43060.1		Granulin repeat cysteine protease family protein	cysteine protease
Sapur.003G156300.1	39.99845849	1.266311294	0.117671065	10.76145	5.23E-27	1.85E-26	cyan	0.514969519	5.19E-12	GO:0055114,GO:0050660,GO:0016491,GO:0019139,GO:0009690	AT3G63440.1	ATCKX6,ATCKX7,CKX6	cytokinin oxidase/dehydrogenase 6	cytokinin oxidase/dehydrogenase
Sapur.001G147600.1	609.1086427	0.696212281	0.064729893	10.75565	5.57E-27	1.97E-26	cyan	0.606797612	3.66E-17		AT1G17455.2	ELF4-L4	ELF4-like 4	ELF4-like protein
Sapur.016G048600.1	500.9049539	0.610797741	0.056832613	10.74731	6.10E-27	2.15E-26	cyan	0.612378501	1.57E-17	GO:0006468,GO:0004672	AT3G57530.1	ATCPK32,CDPK32,CPK32	calcium-dependent protein kinase 32	calcium-dependent kinase
Sapur.006G160700.1	807.1302429	0.723945638	0.067405924	10.74009	6.60E-27	2.32E-26	cyan	0.616285215	8.56E-18		AT3G58490.1		Phosphatidic acid phosphatase (PAP2) family protein	sphingosine-1-phosphate phosphatase
Sapur.014G079400.1	529.3610968	0.389081857	0.036309116	10.71582	8.58E-27	3.01E-26	cyan	0.609497567	2.43E-17	GO:0016021,GO:0005789	AT1G01230.1		ORMDL family protein	ORMDL family protein
Sapur.016G175300.1	101.075631	1.174991638	0.109838845	10.69741	1.05E-26	3.66E-26	cyan	0.545598661	1.48E-13		AT3G19615.1			hypothetical protein
Sapur.005G177800.1	1.484574389	3.857954998	0.361963949	10.6584	1.59E-26	5.55E-26	cyan	0.485559465	1.15E-10	GO:0006355,GO:0003700	AT3G23240.1	ATERF1,ERF1	ethylene response factor 1	ethylene-responsive transcription factor
Sapur.009G059000.1	139.7898302	0.890528527	0.083581595	10.6546	1.66E-26	5.78E-26	cyan	0.566300598	1.08E-14	GO:0045454,GO:0015035,GO:0009055	AT5G58530.1		Glutaredoxin family protein	glutaredoxin family protein
Sapur.003G100200.1	69.16078391	1.269666299	0.119255605	10.6466	1.81E-26	6.29E-26	cyan	0.547244401	1.21E-13		AT5G41761.1			hypothetical protein
Sapur.016G217700.1	216.7956109	0.694874491	0.065284984	10.64371	1.87E-26	6.49E-26	cyan	0.617562553	7.02E-18		AT5G40500.2			hypothetical protein
Sapur.003G015700.1	23.16003003	1.336551401	0.125680312	10.63453	2.06E-26	7.15E-26	cyan	0.578118134	2.22E-15	GO:0016787	AT3G52820.1	ATPAP22,PAP22	purple acid phosphatase 22	purple acid phosphatase
Sapur.003G051200.1	1462.116069	0.504147536	0.047408463	10.63413	2.07E-26	7.18E-26	cyan	0.601210842	8.41E-17		AT5G47120.1	ATBI-1,ATBI1,BI-1,BI1	BAX inhibitor 1	Bax inhibitor
Sapur.010G114600.1	37.96462268	1.230713526	0.115801067	10.62783	2.21E-26	7.67E-26	cyan	0.597665907	1.41E-16	GO:0016272,GO:0006457	AT5G49510.2	PFD3	prefoldin 3	prefoldin subunit 3, putative
Sapur.016G131500.1	3.104572423	3.010675671	0.28341441	10.62287	2.33E-26	8.09E-26	cyan	0.542801692	2.08E-13		AT1G53540.1		HSP20-like chaperones superfamily protein	small heat shock protein class I
Sapur.009G026700.1	28.4753628	1.452227721	0.136954882	10.60369	2.86E-26	9.91E-26	cyan	0.566355151	1.07E-14	GO:0006468,GO:0005524,GO:0004672,GO:0030246	AT3G53810.1		Concanavalin A-like lectin protein kinase family protein	tyrosine kinase
Sapur.15WG103600.1	13.59507477	1.744604286	0.164673712	10.59431	3.17E-26	1.09E-25	cyan	0.567334662	9.41E-15	GO:0055114,GO:0020037,GO:0016705,GO:0005506	AT3G48270.1	CYP71A26	cytochrome P450, family 71, subfamily A, polypeptide 26	cytochrome P450 family protein, putative
Sapur.016G065600.1	593.8230192	1.151337586	0.108926531	10.56985	4.11E-26	1.42E-25	cyan	0.544790762	1.63E-13		AT2G35980.1	ATNHL10,NHL10,YLS9	Late embryogenesis abundant (LEA) hydroxyproline-rich glycoprotein family	NA
Sapur.012G025500.1	10581.17697	0.569605739	0.053901159	10.5676	4.21E-26	1.45E-25	cyan	0.59778019	1.39E-16	GO:0030170,GO:0019752,GO:0016831	AT2G02010.1	GAD4	glutamate decarboxylase 4	glutamate decarboxylase
Sapur.010G042900.1	741.5964953	1.154923606	0.109429891	10.554	4.87E-26	1.68E-25	cyan	0.576887012	2.63E-15	GO:0055114,GO:0016614,GO:0050660	AT3G23410.1	ATFAO3,FAO3	fatty alcohol oxidase 3	long-chain-alcohol oxidase FAO1
Sapur.002G099700.1	412.4487082	0.710867147	0.067468642	10.53626	5.88E-26	2.02E-25	cyan	0.633858572	5.09E-19		AT4G37260.1	ATMYB73,MYB73	myb domain protein 73	MYB transcription factor
Sapur.T000700.1	5.43192376	1.607127228	0.152625735	10.52986	6.29E-26	2.16E-25	cyan	0.586991888	6.51E-16	GO:0006468,GO:0004672	AT3G14840.2		Leucine-rich repeat transmembrane protein kinase	NA
Sapur.016G029500.1	2493.106704	0.361117376	0.034369682	10.50686	8.03E-26	2.75E-25	cyan	0.63607838	3.52E-19	GO:0003676	AT3G56680.1		Single-stranded nucleic acid binding R3H protein	single-stranded nucleic acid-binding R3H protein, putative
Sapur.005G080200.1	78.13423577	1.124391799	0.107048752	10.50355	8.32E-26	2.85E-25	cyan	0.57250173	4.74E-15		AT1G64160.1		Disease resistance-responsive (dirigent-like protein) family protein	disease resistance response protein
Sapur.017G019400.1	266.2339636	1.07642144	0.102483564	10.50336	8.34E-26	2.85E-25	cyan	0.501554072	2.21E-11		AT1G76950.1	PRAF1	Regulator of chromosome condensation (RCC1) family with FYVE zinc finger domain	root/shoot growth-regulatory transcription factor
Sapur.014G087700.1	336.6866969	0.674754419	0.064284103	10.49644	8.97E-26	3.07E-25	cyan	0.580137231	1.69E-15		AT4G01650.1		Polyketide cyclase / dehydrase and lipid transport protein	polyketide cyclase/dehydrase and lipid transporter
Sapur.002G008700.1	214.048987	0.53966639	0.051642728	10.45	1.47E-25	4.99E-25	cyan	0.609953228	2.27E-17		AT1G20460.1			NA
Sapur.004G019800.1	16.59506101	2.23476561	0.214034144	10.44116	1.61E-25	5.47E-25	cyan	0.382826137	7.49E-07	GO:0016021,GO:0006810,GO:0005337	AT4G05120.1	ATENT3,ENT3,FUR1	Major facilitator superfamily protein	NA
Sapur.004G066200.1	2400.962032	0.3313934	0.031785137	10.42605	1.89E-25	6.41E-25	cyan	0.636185158	3.46E-19	GO:0005515,GO:0006413,GO:0003743	AT1G77840.1		Translation initiation factor IF2/IF5	eukaryotic translation initiation factor 5
Sapur.008G102300.1	6.064799304	2.773744512	0.266620256	10.40335	2.39E-25	8.11E-25	cyan	0.48637989	1.06E-10		AT3G03280.1			DUF4228 domain protein
Sapur.017G018800.1	698.2498308	1.84821996	0.177926882	10.38753	2.83E-25	9.56E-25	cyan	0.462124921	1.11E-09		AT2G43290.1	MSS3	Calcium-binding EF-hand family protein	calcium-binding EF-hand protein
Sapur.006G043900.1	8.629280322	1.308335207	0.12627976	10.36061	3.75E-25	1.26E-24	cyan	0.5765928	2.73E-15	GO:0008270,GO:0003677	AT1G51200.1		A20/AN1-like zinc finger family protein	zinc finger A20 and AN1 domain stress-associated protein
Sapur.008G149700.1	682.8651189	0.999236973	0.09672422	10.33078	5.11E-25	1.72E-24	cyan	0.54019136	2.84E-13	GO:0016020,GO:0006487,GO:0003830	AT1G12990.1		beta-1,4-N-acetylglucosaminyltransferase family protein	beta-1%2C4-N-acetylglucosaminyltransferase family protein
Sapur.T125600.1	171.7941127	0.973964947	0.094280907	10.33046	5.13E-25	1.73E-24	cyan	0.604074719	5.50E-17		AT3G19740.1		P-loop containing nucleoside triphosphate hydrolases superfamily protein	NA
Sapur.007G093300.2	14.6117017	1.865356423	0.180596892	10.32884	5.22E-25	1.75E-24	cyan	0.550413851	8.16E-14	GO:0008168	AT5G64030.1		S-adenosyl-L-methionine-dependent methyltransferases superfamily protein	methyltransferase PMT26, putative
Sapur.013G133900.1	1.649627484	4.071685511	0.396417674	10.2712	9.50E-25	3.18E-24	cyan	0.518153844	3.65E-12					NA
Sapur.005G040200.1	11.47246198	2.93878761	0.286297957	10.26479	1.02E-24	3.39E-24	cyan	0.427531465	2.34E-08		AT4G14746.1			hypothetical protein
Sapur.009G065300.1	1.669177461	3.755751751	0.366337652	10.25216	1.16E-24	3.86E-24	cyan	0.53600545	4.68E-13		AT1G47960.1	ATC/VIF1,C/VIF1	cell wall / vacuolar inhibitor of fructosidase 1	NA
Sapur.009G017200.1	69.74311871	1.250005334	0.121927352	10.25205	1.16E-24	3.86E-24	cyan	0.560390732	2.32E-14		AT2G31945.1			transmembrane protein, putative
Sapur.002G036800.1	1788.444572	0.636890543	0.062140362	10.24922	1.19E-24	3.98E-24	cyan	0.617824962	6.74E-18	GO:0005515	AT4G02680.1	EOL1	ETO1-like 1	ethylene overproducer-like protein
Sapur.T040300.1	544.616617	0.922208463	0.090058513	10.2401	1.31E-24	4.36E-24	cyan	0.595745892	1.87E-16		AT3G19740.1		P-loop containing nucleoside triphosphate hydrolases superfamily protein	NA
Sapur.018G098600.1	11.89229108	5.100496877	0.498195619	10.23794	1.34E-24	4.46E-24	cyan	0.337946387	1.50E-05	GO:0016192,GO:0016021	AT5G11150.1	ATVAMP713,VAMP713	vesicle-associated membrane protein 713	NA
Sapur.T025000.1	21.63852905	1.099290857	0.107605772	10.21591	1.68E-24	5.59E-24	cyan	0.579013146	1.97E-15		AT3G19740.1		P-loop containing nucleoside triphosphate hydrolases superfamily protein	NA
Sapur.010G011100.1	540.8752879	0.334590195	0.032804527	10.19951	1.99E-24	6.60E-24	cyan	0.589837453	4.36E-16	GO:0006629	AT5G18640.1		alpha/beta-Hydrolases superfamily protein	triacylglycerol lipase
Sapur.012G084600.1	1.550977251	3.454215621	0.338754143	10.19682	2.05E-24	6.79E-24	cyan	0.551486245	7.15E-14					NA
Sapur.010G104000.1	91.19959012	1.618578965	0.15933941	10.15806	3.05E-24	1.01E-23	cyan	0.500471578	2.48E-11	GO:0008270,GO:0005515	AT2G04240.2	XERICO	RING/U-box superfamily protein	E3 ubiquitin-protein ligase
Sapur.006G193100.1	4.189829147	4.527013028	0.447191275	10.12321	4.36E-24	1.43E-23	cyan	0.434011876	1.36E-08		AT4G29090.1		Ribonuclease H-like superfamily protein	NA
Sapur.012G041300.1	1519.721042	0.277109586	0.027387214	10.11821	4.59E-24	1.51E-23	cyan	0.620786114	4.23E-18		AT5G23040.1	CDF1	Protein of unknown function (DUF3353)	cell growth defect factor-like protein, putative
Sapur.002G043000.1	96.90755991	0.644944076	0.063806563	10.1078	5.10E-24	1.67E-23	cyan	0.586175275	7.30E-16		AT3G52420.1	ATOEP7,OEP7	outer envelope membrane protein 7	transmembrane protein, putative
Sapur.007G014600.1	167.64414	0.589363188	0.058380344	10.09523	5.80E-24	1.90E-23	cyan	0.581165049	1.46E-15					oxidoreductase-like protein, amino-terminal protein
Sapur.014G002900.1	1407.010027	0.698138917	0.069165419	10.09376	5.89E-24	1.93E-23	cyan	0.618803748	5.78E-18	GO:0016021	AT4G37680.1	HHP4	heptahelical protein 4	adiponectin receptor protein
Sapur.006G105200.1	561.1040108	1.264529865	0.125537345	10.07294	7.28E-24	2.38E-23	cyan	0.550649121	7.93E-14	GO:0016020,GO:0006810,GO:0005215	AT2G37170.1	PIP2;2,PIP2B	plasma membrane intrinsic protein 2	plasma membrane intrinsic protein PIP2.2
Sapur.005G200200.1	381.3738779	0.473325927	0.047099194	10.04955	9.23E-24	3.00E-23	cyan	0.618410636	6.15E-18		AT1G76270.1		O-fucosyltransferase family protein	GDP-fucose protein O-fucosyltransferase
Sapur.004G077400.1	7.252106698	1.99341989	0.19906053	10.01414	1.32E-23	4.28E-23	cyan	0.488616868	8.46E-11		AT2G24762.1	AtGDU4,GDU4	glutamine dumper 4	transmembrane protein, putative
Sapur.016G158800.3	26.67389886	0.828688527	0.082849916	10.00229	1.49E-23	4.82E-23	cyan	0.46845152	6.13E-10	GO:0016787	AT5G12200.1	PYD2	pyrimidine 2	dihydropyrimidinase
Sapur.008G160300.1	39.25267015	0.766970973	0.076688306	10.00115	1.51E-23	4.88E-23	cyan	0.569488353	7.08E-15		AT3G06780.1		glycine-rich protein	hypothetical protein
Sapur.013G138800.1	2393.123319	0.777355725	0.077774664	9.994974	1.60E-23	5.19E-23	cyan	0.59284829	2.84E-16		AT2G40610.1	ATEXP8.ATEXPA8.ATHEXP A	expansin A8	expansin
Sapur.012G086300.1	147.0100457	0.964563399	0.096550003	9.990299	1.68E-23	5.43E-23	cyan	0.554951555	4.63E-14	GO:0043531	AT3G14470.1		NB-ARC domain-containing disease resistance protein	NA
Sapur.004G138600.1	530.9368291	0.595372693	0.060257647	9.88045	5.06E-23	1.62E-22	cyan	0.585159352	8.42E-16	GO:0008270,GO:0003677	AT2G27580.2		A20/AN1-like zinc finger family protein	zinc finger A20 and AN1 domain stress-associated protein
Sapur.007G033300.1	1.164946818	3.23917003	0.327928926	9.877659	5.20E-23	1.66E-22	cyan	0.544346837	1.72E-13		AT3G07820.1		Pectin lyase-like superfamily protein	polygalacturonase
Sapur.009G104700.1	24.43166219	1.886759751	0.1911002	9.873144	5.44E-23	1.74E-22	cyan	0.50592546	1.39E-11		AT4G38690.1		PLC-like phosphodiesterases superfamily protein	phosphatidylinositol-specific phospholipase C domain protein
Sapur.010G038000.1	1367.121204	0.580826402	0.059016483	9.841766	7.44E-23	2.37E-22	cyan	0.586485884	6.99E-16	GO:0005515	AT1G04300.1		TRAF-like superfamily protein	MATH domain plant protein
Sapur.15WG058400.1	16.56826507	2.061971261	0.210610166	9.790464	1.24E-22	3.92E-22	cyan	0.551544062	7.09E-14		AT1G13360.1			hypothetical protein
Sapur.002G170100.1	1030.195012	-0.569098704	0.058153386	-9.78617	1.29E-22	4.09E-22	cyan	-0.659592707	5.80E-21	GO:0005525	AT4G02930.1		GTP binding Elongation factor Tu family protein	elongation factor Tu protein
Sapur.011G110200.1	1105.578134	0.517550753	0.053223083	9.724178	2.38E-22	7.48E-22	cyan	0.588284395	5.43E-16	GO:0006810,GO:0005643	AT1G10390.1		Nucleoporin autopeptidase	nucleoporin autopeptidase, putative
Sapur.006G135400.1	3.568818007	2.059165012	0.212226228	9.702689	2.94E-22	9.22E-22	cyan	0.538430298	3.51E-13	GO:0007010,GO:0003779	AT4G30160.1	ATVLN4,VLN4	villin 4	villin, putative
Sapur.003G072500.1	15.26942647	1.653122428	0.170411823	9.70075	2.99E-22	9.40E-22	cyan	0.455275607	2.09E-09		AT5G51330.1	DYAD,SWI1	SWITCH1	DYAD protein
Sapur.013G082900.1	993.3736715	-0.42387473	0.043777234	-9.68254	3.58E-22	1.12E-21	cyan	-0.629493372	1.04E-18	GO:0006412,GO:0005840,GO:0003735,GO:0003723	AT1G64880.1		Ribosomal protein S5 family protein	30S ribosomal protein S5
Sapur.010G009100.1	16.14985432	2.393621656	0.24730698	9.678747	3.71E-22	1.16E-21	cyan	0.543677848	1.04E-18	GO:0008270,GO:0006355,GO:0005634,GO:0006402,GO:0003712	AT5G67480.1	ATBT4,BT4	BTB and TAZ domain protein 4	BTB and TAZ domain protein
Sapur.006G168100.2	35.56146792	1.803005261	0.182996618	9.675173	3.84E-22	1.20E-21	cyan	0.31277323	6.83E-05	GO:0006355,GO:0005667,GO:0003700	AT2G36010.1	ATE2FA,E2F3	E2F transcription factor-3	transcription factor E2F
Sapur.T035500.1	124.2481543	0.499258795	0.051768897	9.643991	5.21E-22	1.63E-21	cyan	0.606478608	3.84E-17	GO:0005515	AT5G67420.1	RPN5B	regulatory particle non-ATPase subunit 5B	NA
Sapur.008G111700.1	2.898941	2.721638916	0.283066022	9.594722	8.41E-22	2.59E-21	cyan	0.549090263	4.46E-14		AT1G68190.1	AtRLP9,RLP9	receptor like protein 9	NA
Sapur.016G020300.1	14.60112929	1.519633164	0.158719581	9.574327	1.03E-21	3.18E-21	cyan	0.472026888	4.78E-10	GO:0016758,GO:0008152	AT1G22380.1	AtUGT85A3,UGT85A3	UDP-glucosyl transferase 85A3	UDP-glycosyltransferase
Sapur.003G121100.1	5.519512462	2.231852882	0.233489922	9.558869	1.19E-21	3.69E-21	cyan	0.44529446	5.12E-09		AT4G26930.1	ATMYB97,MYB97	myb domain protein 97	Myb-like DNA-binding domain protein
Sapur.018G082700.1	178.6284484	1.005018403	0.105588419	9.519221	1.74E-21	5.37E-21	cyan	0.499779862	2.67E-11	GO:0051260	AT2G30050.1		BTB/POZ domain with WD40/YVTN repeat-like protein	potassium channel tetramerization domain protein
Sapur.004G072300.1	31.18185716	1.080527324	0.113825545	9.492837	2.25E-21	6.91E-21	cyan	0.546511395	1.32E-13		AT2G03630.1			hypothetical protein

Sapur.006G028100.1	362.0953958	1.391666997	0.146799577	9.480048	2.54E-21	7.80E-21	cyan	0.567315234	9.43E-15	GO:0003333	AT2G41190.1		Transmembrane amino acid transporter family protein	amino acid transporter, putative
Sapur.001G191900.4	13.19510933	1.088567511	0.114856816	9.477605	2.60E-21	7.98E-21	cyan	0.683417552	6.11E-23	GO:0016787	AT4G19420.1		Pectinacetylesterase family protein	pectinacetylesterase
Sapur.010G180600.1	6.240632201	1.472294374	0.155541432	9.465609	2.92E-21	8.94E-21	cyan	0.511005833	8.02E-12					hypothetical protein
Sapur.014G075800.1	29.46250201	1.007749729	0.106468002	9.465283	2.93E-21	8.97E-21	cyan	0.504829561	1.56E-11	GO:0006629	AT3G61680.1		alpha/beta-Hydrolases superfamily protein	triacylglycerol lipase, putative
Sapur.009G004200.1	5415.974986	0.496023593	0.052618247	9.426836	4.23E-21	1.29E-20	cyan	0.555180636	4.50E-14	GO:0016887,GO:0005524	AT5G60790.1	ATGCN1,GCN1	ABC transporter family protein	ABC transporter family protein
Sapur.005G003600.1	3873.634705	0.367900104	0.039073571	9.415574	4.71E-21	1.43E-20	cyan	0.60237199	7.08E-17	GO:0006468,GO:0004672	AT5G26751.1	ATSK11,SK 11	shaggy-related kinase 11	glycogen synthase kinase-3 protein
Sapur.016G135900.1	5.790660677	2.301180895	0.244641095	9.406355	5.14E-21	1.57E-20	cyan	0.397631806	2.51E-07	GO:0006468,GO:0005524,GO:0004672,GO:0030246	AT3G53810.1		Concanavalin A-like lectin protein kinase family protein	lectin-like receptor kinase
Sapur.T105300.1	35.97843898	0.825409992	0.087769034	9.404342	5.24E-21	1.59E-20	cyan	0.577877949	2.30E-15	GO:0055114,GO:0009186	AT3G23580.1	RNR2,RNR2A	ribonucleotide reductase 2A	NA
Sapur.016G016800.1	1270.874608	0.42173873	0.044880737	9.396876	5.62E-21	1.71E-20	cyan	0.59059506	3.91E-16		AT3G22270.1		Topoisomerase II-associated protein PAT1	topoisomerase II-associated protein PAT1
Sapur.002G145000.1	31.36482257	1.168903209	0.125042588	9.348041	8.93E-21	2.70E-20	cyan	0.588855125	5.01E-16	GO:0055085,GO:0016020,GO:0006811,GO:0005216	AT1G01340.2	ACBK1,ATCNGC10,CNGC10	cyclic nucleotide gated channel 10	cyclic nucleotide-gated ion channel protein, putative
Sapur.017G044300.1	24.54090236	1.139977834	0.122098541	9.336539	9.95E-21	3.01E-20	cyan	0.588405894	5.34E-16	GO:0016787	AT3G07130.1	ATPAP15,PAP15	purple acid phosphatase 15	purple acid phosphatase
Sapur.002G183800.1	688.2663831	0.547013825	0.058670745	9.323451	1.13E-20	3.40E-20	cyan	0.577535639	2.41E-15	GO:0031011	AT5G13950.2			nuclear factor kappa-B-binding-like protein, putative
Sapur.012G056900.1	7.49596087	1.680369113	0.180742212	9.297049	1.44E-20	4.34E-20	cyan	0.509737533	9.21E-12	GO:0003682,GO:0006351,GO:0005634,GO:0003677	AT3G48050.2		BAH domain ;TFIIS helical bundle-like domain	BAH and TFIIS domain protein, putative
Sapur.014G143000.1	725.8806708	0.369923392	0.039838355	9.285609	1.61E-20	4.83E-20	cyan	0.571212719	5.63E-15		AT3G07560.1	APM2,PEX13	peroxin 13	peroxisome biogenesis factor 13, putative
Sapur.016G162300.1	13.22272287	3.278899078	0.355375556	9.226575	2.79E-20	8.34E-20	cyan	0.473522072	3.77E-10	GO:0005544,GO:0005509	AT5G12380.1	ANNAT8	annexin 8	annexin
Sapur.002G051500.1	857.1826589	1.240853766	0.135321795	9.169652	4.75E-20	1.41E-19	cyan	0.489662031	7.60E-11		AT4G09890.1		Protein of unknown function (DUF3511)	DUF3511 domain protein
Sapur.T125700.1	348.1852528	0.935200462	0.102015755	9.167216	4.85E-20	1.44E-19	cyan	0.565122158	1.26E-14		AT1G50140.1		P-loop containing nucleoside triphosphate hydrolases superfamily protein	NA
Sapur.005G102100.1	121.7775061	0.657381437	0.07171352	9.166771	4.87E-20	1.44E-19	cyan	0.550226804	8.36E-14		AT2G23780.1		RING/U-box superfamily protein	RING zinc finger protein, putative
Sapur.010G013200.1	961.3993563	0.468464893	0.051402104	9.11373	7.96E-20	2.35E-19	cyan	0.569235454	7.32E-15		AT1G03350.1		BSD domain-containing protein	BSD domain protein
Sapur.019G000700.1	735.2016709	0.513802346	0.056383897	9.112572	8.05E-20	2.37E-19	cyan	0.565614601	1.18E-14	GO:0016491,GO:0008198,GO:0006725	AT4G15093.1		catalytic LigB subunit of aromatic ring-opening dioxygenase family	NA
Sapur.003G141100.1	14.04791563	0.905368107	0.099642106	9.0862	1.03E-19	3.01E-19	cyan	0.529725788	9.77E-13					NA
Sapur.001G015700.1	885.9789887	0.383202907	0.042364063	9.045471	1.49E-19	4.36E-19	cyan	0.564119597	1.43E-14	GO:0008270,GO:0003677	AT1G51200.1		A20/AN1-like zinc finger family protein	zinc finger A20 and AN1 domain stress-associated protein
Sapur.012G066700.1	465.0159268	0.893429817	0.098850281	9.038212	1.59E-19	4.66E-19	cyan	0.573192184	4.32E-15	GO:0055114,GO:0020037,GO:0016705,GO:0005506	AT4G13310.1	CYP71A20	cytochrome P450, family 71, subfamily A, polypeptide 20	NA
Sapur.018G110900.1	2.682961798	2.842848777	0.315749186	9.003503	2.19E-19	6.37E-19	cyan	0.421854073	3.74E-08	GO:0005515	AT1G58190.2	AtRLP9,RLP9	receptor like protein 9	Serine/Threonine kinase, plant-type protein%2C putative
Sapur.012G001700.1	10.56272441	1.08713934	0.121117223	8.975927	2.81E-19	8.17E-19	cyan	0.514969024	5.19E-12	GO:0005515	AT1G71400.1	AtRLP12,RLP12	receptor like protein 12	NA
Sapur.009G034800.1	608.2813506	0.537369575	0.059874027	8.975003	2.83E-19	8.24E-19	cyan	0.582963747	1.14E-15	GO:0019140,GO:0010264,GO:0005524	AT5G58730.1		pfkB-like carbohydrate kinase family protein	pfkB family carbohydrate kinase
Sapur.011G045600.1	538.6381379	0.428656563	0.047922377	8.94481	3.73E-19	1.08E-18	cyan	0.598618463	1.23E-16		AT1G29120.2		Hydrolase-like protein family	hydrolase-like protein
Sapur.001G107600.1	206.3351074	0.346413104	0.038751774	8.939284	3.92E-19	1.13E-18	cyan	0.594733629	2.16E-16		AT4G19140.1			NA
Sapur.017G097600.1	13.93916905	1.343594888	0.150349983	8.936449	4.02E-19	1.16E-18	cyan	0.538141886	3.63E-13	GO:0006468,GO:0005524,GO:0004672	AT1G66920.2		Protein kinase superfamily protein	NA
Sapur.006G028200.1	229.054651	1.122274876	0.12580045	8.921072	4.62E-19	1.33E-18	cyan	0.566331358	1.07E-14		AT2G41190.1		Transmembrane amino acid transporter family protein	NA
Sapur.006G018100.1	69.03227454	1.660852182	0.186687355	8.896436	5.77E-19	1.66E-18	cyan	0.462976028	1.03E-09	GO:0016758,GO:0008152	AT1G22400.1	ATUGT85A1,UGT85A1	UDP-Glycosyltransferase superfamily protein	NA
Sapur.003G084700.1	5.981175676	1.603268311	0.180585958	8.878145	6.80E-19	1.95E-18	cyan	0.497012198	3.57E-11	GO:0006508,GO:0004252	AT5G59190.1		subtilase family protein	cucumisin, putative
Sapur.009G119800.1	144.7915714	0.711363836	0.080240124	8.865438	7.62E-19	2.19E-18	cyan	0.525818233	1.53E-12		AT2G27180.1			hypothetical protein
Sapur.018G070600.1	0.991540538	3.0975482	0.351091269	8.82263	1.12E-18	3.20E-18	cyan	0.518669408	3.44E-12	GO:0000160	AT4G16110.1	ARR2,RR2	response regulator 2	type-B response regulator
Sapur.006G183800.1	20.55054845	2.02693896	0.2300277	8.811717	1.23E-18	3.52E-18	cyan	0.490101978	7.27E-11		AT5G16100.1			RWP-RK domain protein
Sapur.019G109500.1	127.2988261	0.449736298	0.051046963	8.810246	1.25E-18	3.57E-18	cyan	0.563753799	1.50E-14		AT2G30270.1		Protein of unknown function (DUF567)	tubby C 2 protein
Sapur.016G153500.1	2.044373508	3.590507209	0.408246652	8.794946	1.43E-18	4.08E-18	cyan	0.377492414	1.10E-06		AT1G07985.1		Expressed protein	hypothetical protein
Sapur.009G018100.1	401.0671589	0.723249022	0.082470428	8.769798	1.79E-18	5.09E-18	cyan	0.507796089	1.14E-11	GO:0005515,GO:0016459,GO:0005524,GO:0003774	AT2G31900.1	ATMYO5,ATXIF,XIF	myosin-like protein XIF	NA
Sapur.001G056000.1	4281.276682	0.698880235	0.079776168	8.760514	1.94E-18	5.53E-18	cyan	0.506125997	1.36E-11	GO:0048046,GO:0016762,GO:0006073,GO:0005618,GO:0005975,GO:0004553	AT5G13870.1	EXGT-A4,XTH5	xyloglucan endotransglucosylase/hydrolase 5	xyloglucan endotransglycosylase/hydrolase
Sapur.012G001900.1	114.6521151	1.235067477	0.141136551	8.750869	2.12E-18	6.01E-18	cyan	0.521295212	2.56E-12	GO:0005515	AT1G71400.1	AtRLP12,RLP12	receptor like protein 12	NA
Sapur.002G083800.1	2434.039686	0.54205369	0.062027902	8.738869	2.35E-18	6.68E-18	cyan	0.542041471	2.28E-13		AT4G24570.1	DIC2	dicarboxylate carrier 2	dicarboxylate carrier protein, putative
Sapur.T000600.1	9.023144261	0.933906709	0.107071537	8.722269	2.73E-18	7.72E-18	cyan	0.548833413	9.93E-14	GO:0005515,GO:0006468,GO:0004672	AT3G14840.2		Leucine-rich repeat transmembrane protein kinase	NA
Sapur.016G049200.1	405.1979308	0.626004653	0.071868488	8.710419	3.03E-18	8.57E-18	cyan	0.566201324	1.09E-14		AT2G41830.1		Uncharacterized protein	NA
Sapur.014G083100.1	297.2531385	0.482209892	0.055416246	8.701598	3.27E-18	9.25E-18	cyan	0.542470616	2.16E-13		AT3G14430.1			transmembrane protein, putative
Sapur.016G081500.1	1.477126175	3.064828467	0.352561696	8.693027	3.53E-18	9.97E-18	cyan	0.420881431	4.05E-08		AT3G09670.1		Tudor/PWWP/MBT superfamily protein	NA
Sapur.008G030100.1	1.7332022	2.61887618	0.301558639	8.684467	3.81E-18	1.07E-17	cyan	0.511222397	7.84E-12					NA
Sapur.003G103400.1	2.509812916	2.397938211	0.276513171	8.672058	4.24E-18	1.20E-17	cyan	0.479734506	2.06E-10		AT4G23880.1			hypothetical protein
Sapur.010G123600.1	10.18001748	1.091933354	0.125972158	8.668053	4.40E-18	1.24E-17	cyan	0.547585767	1.16E-13		AT1G13890.1	ATSNAP30,SNAP30	soluble N-ethylmaleimide-sensitive factor adaptor protein 30	synaptosomal-associated protein
Sapur.018G095200.1	129.8438461	-0.465937862	0.053833736	-8.65513	4.92E-18	1.39E-17	cyan	-0.578206749	2.20E-15		AT4G20060.1	EMB1895	ARM repeat superfamily protein	embryo defective 1895 protein, putative
Sapur.016G243900.1	665.2603797	0.337818778	0.039092932	8.641429	5.55E-18	1.56E-17	cyan	0.539315258	3.16E-13		AT1G17080.1		Ribosomal protein L18ae family	60S ribosomal protein L18a, putative
Sapur.012G005900.1	105.9505692	1.256246686	0.145883269	8.611314	7.22E-18	2.02E-17	cyan	0.479710277	2.06E-10	GO:0005515	AT1G47890.1	AtRLP7,RLP7	receptor like protein 7	receptor-like kinase, putative
Sapur.018G112900.1	3.991904322	3.160571503	0.367271866	8.605537	7.60E-18	2.13E-17	cyan	0.325519751	3.18E-05	GO:0005515	AT1G58190.2	AtRLP9,RLP9	receptor like protein 9	NA
Sapur.012G002000.1	40.78248903	1.038382801	0.121230312	8.565373	1.08E-17	3.00E-17	cyan	0.50211658	2.09E-11	GO:0005515	AT3G11080.1	AtRLP35,RLP35	receptor like protein 35	NA
Sapur.003G129100.1	212.6492812	1.348850738	0.157646576	8.556169	1.17E-17	3.25E-17	cyan	0.528672768	1.10E-12		AT5G51920.1		Pyridoxal phosphate (PLP)-dependent transferases superfamily protein	molybdenum cofactor sulfurase, putative
Sapur.006G004900.1	5023.286818	0.811078967	0.094832166	8.552783	1.20E-17	3.34E-17	cyan	0.541547268	2.42E-13	GO:0055114,GO:0020037,GO:0016705,GO:0005506	AT4G15110.1	CYP97B3	cytochrome P450, family 97, subfamily B, polypeptide 3	cytochrome P450 family 97 protein
Sapur.004G089700.1	69.35261927	0.750400444	0.087781974	8.548457	1.25E-17	3.47E-17	cyan	0.517683138	3.84E-12		AT2G15695.1		Protein of unknown function DUF829, transmembrane 53	DUF829 domain protein
Sapur.T152600.1	446.9581138	1.039169622	0.121658496	8.541694	1.32E-17	3.68E-17	cyan	0.526967196	1.34E-12		AT1G50140.1		P-loop containing nucleoside triphosphate hydrolases superfamily protein	NA
Sapur.008G069300.1	6.849836489	1.882909372	0.220450025	8.541207	1.33E-17	3.69E-17	cyan	0.508558439	1.05E-11	GO:0005509	AT5G39670.1		Calcium-binding EF-hand family protein	EF-hand pair protein
Sapur.010G085000.1	2018.877827	0.564101723	0.066071439	8.537754	1.37E-17	3.80E-17	cyan	0.536413027	4.46E-13	GO:0006468,GO:0005524,GO:0004672	AT1G73670.1	ATMPK15,MPK15	MAP kinase 15	MAP kinase
Sapur.T120200.1	1.080359316	2.566473859	0.301534324	8.511382	1.72E-17	4.76E-17	cyan	0.51265264	6.70E-12	GO:0005681,GO:0000398,GO:0000387	AT1G54385.2		ARM repeat superfamily protein	NA
Sapur.15WG003400.1	491.1931592	0.809057556	0.095184431	8.499894	1.90E-17	5.25E-17	cyan	0.520303229	2.87E-12	GO:0006468,GO:0004672,GO:0030246	AT5G55830.1		Concanavalin A-like lectin protein kinase family protein	lectin-domain receptor kinase
Sapur.019G020000.1	649.9478027	0.486273154	0.057437314	8.466154	2.54E-17	6.99E-17	cyan	0.55580493	4.16E-14	GO:0005515,GO:0006468,GO:0004672	AT3G14840.2		Leucine-rich repeat transmembrane protein kinase	NA
Sapur.005G028700.1	1.292419098	2.450929407	0.289879506	8.454994	2.79E-17	7.69E-17	cyan	0.517674724	3.85E-12	GO:0016758,GO:0008152	AT2G15490.3	UGT73B4	UDP-glycosyltransferase 73B4	UDP-glucosyltransferase, putative
Sapur.009G058500.1	0.723716396	2.760326659	0.326877016	8.444542	3.05E-17	8.39E-17	cyan	0.52567349	1.56E-12	GO:0043565,GO:0006355,GO:0003700	AT2G42380.2	ATBZIP34,BZIP34	Basic-leucine zipper (bZIP) transcription factor family protein	NA
Sapur.018G047900.1	148.2109144	0.44320058	0.052629964	8.421069	3.73E-17	1.02E-16	cyan	0.517020351	4.14E-12	GO:0008270,GO:0009972,GO:0004126	AT2G19570.1	AT-CDA1,CDA1,DESZ	cytidine deaminase 1	cytidine deaminase
Sapur.009G081600.1	28.05763533	1.021608745	0.121580137	8.40276	4.36E-17	1.19E-16	cyan	0.408895926	1.06E-07		AT2G03550.1		alpha/beta-Hydrolases superfamily protein	gibberellin receptor GID1
Sapur.012G027400.1	3.979551793	2.530631525	0.30117998	8.40239	4.37E-17	1.20E-16	cyan	0.466756516	7.20E-10	GO:0005544,GO:0005509	AT1G68090.1	ANN5,ANNAT5	annexin 5	NA
Sapur.011G007100.1	4.528897241	2.047389382	0.244215052	8.383551	5.14E-17	1.40E-16	cyan	0.446680461	4.52E-09		AT4G22080.1	RHS14	root hair specific 14	NA
Sapur.004G105400.1	514.2999684	0.354733722	0.042319591	8.382258	5.19E-17	1.42E-16	cyan	0.559687724	2.54E-14	GO:0046872	AT1G47570.1		RING/U-box superfamily protein	RING zinc finger protein, putative
Sapur.017G032600.1	87.9472607	0.754325694	0.09014659	8.367767	5.87E-17	1.60E-16	cyan	0.524911726	1.70E-12	GO:0016791	AT2G14110.1		Haloacid dehalogenase-like hydrolase (HAD) superfamily protein	NA
Sapur.005G193400.1	95.8254658	0.815813177	0.097510036	8.366453	5.94E-17	1.62E-16	cyan	0.471842073	4.43E-10		AT1G75810.1			transmembrane protein, putative
Sapur.T017800.1	3.586403896	2.0793235	0.248692088	8.361036	6.22E-17	1.69E-16	cyan	0.444255136	5.60E-09					NA
Sapur.005G031000.1	92.72480539	2.278477157	0.272720624	8.35462	6.56E-17	1.79E-16	cyan	0.441917428	6.87E-09	GO:0005515,GO:0006468,GO:0005524,GO:0004672	AT3G47570.1		Leucine-rich repeat protein kinase family protein	NA
Sapur.011G080500.1	957.7033508	0.763929714	0.091680529	8.332519	7.91E-17	2.15E-16	cyan	0.488975712	8.15E-11	GO:0005634,GO:0003677	AT3G15270.1	SPL5	squamosa promoter binding protein-like 5	squamosa promoter-binding-like protein
Sapur.011G078800.2	18.31633112	1.139309152	0.138239854	8.241539	1.70E-16	4.57E-16	cyan	0.52735438	1.29E-12	GO:0016829,GO:0006520	AT5G53970.1		Tyrosine transaminase family protein	tyrosine aminotransferase
Sapur.016G031100.1	3519.063943	0.592975032	0.072008461	8.234797	1.80E-16	4.83E-16	cyan	0.519115841	3.27E-12		AT3G55290.1		NAD(P)-binding Rossmann-fold superfamily protein	beta-keto acyl carrier reductase
Sapur.002G009900.1	9.253653388	1.306375027	0.1587424	8.229528	1.88E-16	5.04E-16	cyan	0.528354795	1.15E-12					NA
Sapur.003G075400.1	310.6953971	-0.291956114	0.035633649	-8.19327	2.54E-16	6.80E-16	cyan	-0.556617475	3.75E-14	GO:0005525	AT1G45332.1		Translation elongation factor EFG/EF2 protein	elongation factor G
Sapur.006G162200.1	12.15661737	1.167922087	0.143036452	8.165206	3.21E-16	8.55E-16	cyan	0.488342147	8.70E-11	GO:0008270,GO:0005515	AT2G38920.1		SPX (SYG1/Pho81/XPR1) domain-containing protein / zinc finger (C3HC4-type RING finger) protein-related	E3 ubiquitin-protein ligase BAH1-like protein, putative
Sapur.002G170400.1	2825.979018	0.69031659	0.084614736	8.158349	3.40E-16	9.05E-16	cyan	0.500945961	2.36E-11	GO:0016747,GO:0008152	AT2G33150.1	KAT2,PED1,PKT3	peroxisomal 3-ketoacyl-CoA thiolase 3	peroxisomal 3-ketoacyl-CoA thiolase
Sapur.011G100500.1	394.7885288	-0.325745833	0.039951685	-8.15349	3.54E-16	9.41E-16	cyan	-0.574720712	3.52E-15		AT4G27070.1	TSB2	tryptophan synthase beta-subunit 2	tryptophan synthase beta chain
Sapur.T091200.1	9.408378652	2.980647545	0.367459605	8.111497	5.00E-16	1.33E-15	cyan	0.37487614	1.32E-06	GO:0016788	AT1G71691.2		GDSL-like Lipase/Acylhydrolase superfamily protein	GDSL-like lipase/acylhydrolase
Sapur.T017200.1	1.345498642	3.756900486	0.465214895	8.075624	6.71E-16	1.77E-15	cyan	0.4032955	1.63E-07		AT1G33811.1		GDSL-like Lipase/Acylhydrolase superfamily protein	NA
Sapur.005G067700.1	544.0735567	-0.326450488	0.040510574	-8.0584	7.73E-16	2.04E-15	cyan	-0.56293457	1.67E-14		AT4G39460.1	SAMC1,SAMT1	S-adenosylmethionine carrier 1	S-adenosylmethionine carrier protein
Sapur.T105000.1	16.94586388	0.878898002	0.109352362	8.037302	9.18E-16	2.42E-15	cyan	0.517524718	3.91E-12					NA
Sapur.006G049400.1	2.9640183	1.533931748	0.191450176	8.012172	1.13E-15	2.96E-15	cyan	0.490927568	6.68E-11		AT3G54070.1		Ankyrin repeat family protein	NA
Sapur.017G119800.1	41.34098058	1.288799088	0.160937485	8.008073	1.17E-15	3.06E-15	cyan	0.480487303	1.91E-10		AT5G52790.1		CBS domain-containing protein with a domain of unknown function (DUF21)	NA
Sapur.001G093100.1	313.5398656	-0.294905669	0.036869127	-7.99872	1.26E-15	3.30E-15	cyan	-0.55764014	3.29E-14	GO:0046872,GO:0030001,GO:0055114,GO:0006801,GO:0016532,GO:0005507	AT1G12520.1	ATCCS,CCS	copper chaperone for SOD1	Cu/Zn-superoxide dismutase copper chaperone
Sapur.009G040300.1	220.1274955	0.552567947	0.069288699	7.974864	1.53E-15	3.99E-15	cyan	0.512749411	6.63E-12		AT5G19855.1		Chaperonin-like RbcX protein	chaperonin-like RbcX protein
Sapur.004G000500.2	42.80837289	0.54163516	0.06809067	7.954616	1.80E-15	4.69E-15	cyan	0.545728481	1.45E-13	GO:0016811,GO:0016021,GO:0006672	AT4G22330.1	ATCES1	Alkaline phytoceramidase (aPHC)	alkaline ceramidase
Sapur.001G144400.1	1.037751404	2.939106415	0.369528822	7.953659	1.81E-15	4.73E-15	cyan	0.473404861	3.81E-10	GO:0016021	AT5G47530.1		Auxin-responsive family protein	auxin-induced in root cultures protein, putative
Sapur.013G064800.1	1275.505822	0.377658271	0.047545147	7.943151	1.97E-15	5.14E-15	cyan	0.50663762	1.29E-11		AT4G27600.1	NARA5	pfkB-like carbohydrate kinase family protein	pfkB family carbohydrate kinase
Sapur.019G059100.1	8.332721207	0.87431733	0.110308673	7.926098	2.26E-15	5.88E-15	cyan	0.464234763	9.12E-10					NA
Sapur.010G106100.1	12.29583078	1.03438527	0.130855913	7.904765	2.68E-15	6.97E-15	cyan	0.481733993	1.69E-10		AT2G25185.1		Defensin-like (DEFL) family protein	transmembrane protein, putative
Sapur.016G091900.1	929.764487	-0.341301909	0.043359053	-7.87153	3.50E-15	9.07E-15	cyan	-0.563530148	1.55E-14		AT2G37860.3	LCD1	Protein of unknown function (DUF3411)	NA
Sapur.006G089900.1	12.32097195	1.186610919	0.150892193	7.863965	3.72E-15	9.63E-15	cyan	0.49996086	2.62E-11		AT3G08720.1	ATPK19,ATPK2,ATS6K2,S6K	serine/threonine protein kinase 2	ribosomal protein S6 kinase, putative
Sapur.006G182500.1	14.65975284	1.673960806	0.213550839	7.838699	4.55E-15	1.17E-14	cyan	0.376426929	1.18E-06		AT5G35550.1	ATMYB123,ATTT2,MYB123,	Duplicated homeodomain-like superfamily protein	NA
Sapur.002G198500.1	9.441341481	2.725293662	0.347904653	7.83345	4.75E-15	1.22E-14	cyan	0.265912634	0.000762496	GO:0016020,GO:0015696,GO:0008519,GO:0072488	AT4G13510.1	AMT1:1,ATAMT1,ATAMT1:1	ammonium transporter 1;1	ammonium transporter 1
Sapur.T039700.1	576.2127569	0.869400697	0.111141716	7.822452	5.18E-15	1.33E-14	cyan	0.500365681	2.51E-11		AT3G19740.1		P-loop containing nucleoside triphosphate hydrolases superfamily protein	NA
Sapur.003G123000.1	79.78252945	0.441480396	0.056515491	7.811671	5.64E-15	1.45E-14	cyan	0.518960486	3.33E-12		AT2G33510.1			Humj1, putative
Sapur.010G160000.1	311.9883081	0.368743365	0.047240187	7.805713	5.92E-15	1.52E-14	cyan	0.514913136	5.22E-12		AT2G39650.1		Protein of unknown function (DUF506)	plant-specific domain TIGR01615 family protein, putative
Sapur.002G001200.1	44.27848131	1.118005324	0.143312789	7.801155	6.13E-15	1.58E-14	cyan	0.503033431	1.89E-11		AT1G21010.1			mRNA, 952 bp sequence protein, putative
Sapur.005G056200.1	0.79933558	3.007888865	0.386083736	7.790768	6.66E-15	1.71E-14	cyan	0.501507378	2.23E-11					NA
Sapur.002G145600.1	190.8314115	0.990964923	0.127456458	7.774929	7.55E-15	1.94E-14	cyan	0.504715799	1.58E-11		AT4G01037.1	AtWTF1,WTF1	Ubiquitin carboxyl-terminal hydrolase family protein	plant organelle RNA recognition domain protein
Sapur.005G139800.1	211.8090226	0.394089522	0.050739253	7.766956	8.04E-15	2.06E-14	cyan	0.494271711	4.74E-11		AT1G21770.1		Acyl-CoA N-acyltransferases (NAT) superfamily protein	GNAT domain acetyltransferase
Sapur.013G034900.1	5.39635065	2.015435663	0.2595676	7.764589	8.19E-15	2.10E-14	cyan	0.439406234	8.55E-09					NA
Sapur.010G204300.1	712.2493496	0.292290417	0.037668154	7.759616	8.52E-15	2.18E-14	cyan	0.517753425	3.81E-12		AT1G16840.4			hypothetical protein
Sapur.001G191900.2	43.23599057	0.724768133	0.093405546	7.759369	8.54E-15	2.18E-14	cyan	0.601320309	8.27E-17	GO:0016787	AT4G19420.1		Pectinacetylesterase family protein	pectinacetylesterase
Sapur.012G002800.1	43.71825996	1.505820889	0.19423519	7.752565	9.01E-15	2.30E-14	cyan	0.44485253	5.31E-09	GO:0055114,GO:0016491	AT5G24530.1	DMR6	2-oxoglutarate (2OG) and Fe(II)-dependent oxygenase superfamily protein	aminocyclopropanecarboxylate oxidase
Sapur.003G073700.1	11.50201363	1.653727369	0.214339846	7.715445	1.21E-14	3.07E-14	cyan	0.365380082	2.53E-06	GO:0055085,GO:0016021	AT1G20925.1		Auxin efflux carrier family protein	auxin efflux carrier family protein
Sapur.017G041600.1	1.264927611	3.769181538	0.489263039	7.703794	1.32E-14	3.36E-14	cyan	0.376490431	1.18E-06		AT5G09430.1		alpha/beta-Hydrolases superfamily protein	NA
Sapur.005G116500.1	207.5424677	0.691546294	0.089865912	7.695313	1.41E-14	3.59E-14	cyan	0.509241929	9.72E-12		AT1G50140.1		P-loop containing nucleoside triphosphate hydrolases superfamily protein	NA
Sapur.006G095300.1	285.8000239	-0.327703845	0.042692822	-7.67585	1.64E-14	4.17E-14	cyan	-0.546854906	1.27E-13		AT3G52940.1	ELL1,FK,HYD2	Ergosterol biosynthesis ERG4/ERG24 family	sterol C-14 reductase
Sapur.011G031800.2	1.137037705	3.236876237	0.422131885	7.667926	1.75E-14	4.43E-14	cyan	0.460181174	1.33E-09		AT4G04980.1			CHUP1, putative
Sapur.016G030200.1	405.1235429	0.365917248	0.047802979	7.654696	1.94E-14	4.90E-14	cyan	0.509909539	9.04E-12	GO:0006351,GO:0005634,GO:0003677	AT5G05140.1		Transcription elongation factor (TFIIS) family protein	mediator of RNA polymerase II transcription subunit 26b, putative
Sapur.001G093200.1	4773.327286	1.29974418	0.170191505	7.636951	2.22E-14	5.62E-14	cyan	0.518360333	3.56E-12	GO:0055114,GO:0020037,GO:0016705,GO:0005506	AT2G45550.1	CYP76C4	cytochrome P450, family 76, subfamily C, polypeptide 4	cytochrome P450 family protein
Sapur.010G086200.1	1.685190713	2.903944469	0.381504544	7.611821	2.70E-14	6.80E-14	cyan	0.371458888	1.67E-06		AT2G01340.1	At17.1		DUF4228 domain protein
Sapur.013G140200.2	2.407012468	1.803177134	0.237311826	7.598345	3.00E-14	7.54E-14	cyan	0.446018729	4.79E-09		AT2G30340.1	LBD13	LOB domain-containing protein 13	fission regulator
Sapur.016G212800.1	3778.383058	0.386798905	0.050912707	7.597296	3.02E-14	7.60E-14	cyan	0.490558074	6.94E-11	GO:0051536,GO:0009055	AT5G40650.1	SDH2-2	succinate dehydrogenase 2-2	succinate dehydrogenase iron-sulfur subunit
Sapur.018G018300.1	62.40469811	0.802329775	0.105812978	7.582527	3.39E-14	8.51E-14	cyan	0.457715103	1.67E-09	GO:0006355,GO:0003700	AT5G25190.1		Integrase-type DNA-binding superfamily protein	AP2/ERF domain transcription factor
Sapur.018G077000.1	11.55503503	0.750010588	0.098968649	7.578264	3.50E-14	8.79E-14	cyan	0.501841829	2.15E-11					NA
Sapur.T139200.1	267.1341986	0.945437664	0.125430768	7.537526	4.79E-14	1.20E-13	cyan	0.500638915	2.44E-11		AT1G50140.1		P-loop containing nucleoside triphosphate hydrolases superfamily protein	NA
Sapur.005G057400.1	1.582315127	2.946638326	0.392482219	7.507699	6.02E-14	1.50E-13	cyan	0.383180393	7.30E-07		AT2G22320.1			NA
Sapur.006G126000.1	41.48079182	0.85705867	0.114532681	7.483093	7.26E-14	1.80E-13	cyan	0.438613101	9.15E-09		AT5G56980.1			NA
Sapur.011G011400.1	71.31489364	0.611580632	0.081739277	7.48209	7.31E-14	1.82E-13	cyan	0.49353992	5.11E-11		AT4G03960.1		Phosphotyrosine protein phosphatases superfamily protein	tyrosine phosphatase family protein
Sapur.008G097100.1	24.08073122	1.29031084	0.17259447	7.475969	7.66E-14	1.90E-13	cyan	0.414820473	6.61E-08		AT1G68510.1	LBD42	LOB domain-containing protein 42	LOB domain protein
Sapur.012G060900.1	43.41672177	0.648745633	0.087001614	7.456708	8.87E-14	2.20E-13	cyan	0.479281494	2.15E-10	GO:0005515				CO2+/MG2+ efflux protein ApaG, putative
Sapur.008G140300.1	0.902012531	2.938709898	0.39488205	7.441994	9.92E-14	2.45E-13	cyan	0.439861117	8.22E-09					NA
Sapur.005G163500.1	3722.802321	0.405860541	0.054619945	7.430629	1.08E-13	2.67E-13	cyan	0.521138278	2.61E-12		AT2G43020.1	ATPAO2,PAO2	polyamine oxidase 2	polyamine oxidase, putative
Sapur.001G085200.1	309.4434404	0.564382435	0.076134992	7.412918	1.24E-13	3.05E-13	cyan	0.437372052	1.02E-08		AT4G11570.1		Haloacid dehalogenase-like hydrolase (HAD) superfamily protein	haloacid dehalogenase-like hydrolase
Sapur.005G115600.1	147.133381	0.841943301	0.113579005	7.412843	1.24E-13	3.05E-13	cyan	0.475273436	3.18E-10		AT3G19740.1		P-loop containing nucleoside triphosphate hydrolases superfamily protein	NA
Sapur.007G050400.1	1311.953854	0.340016325	0.045901569	7.40751	1.29E-13	3.17E-13	cyan	0.48484251	1.24E-10		AT3G49940.1	LBD38	LOB domain-containing protein 38	lateral organ boundaries domain protein
Sapur.013G133800.1	263.2332673	0.584711057	0.07921837	7.381003	1.57E-13	3.86E-13	cyan	0.529074155	1.05E-12		AT1G62930.1		Tetratricopeptide repeat (TPR)-like superfamily protein	PPR domain protein
Sapur.013G055100.1	391.1472888	-0.274465382	0.037361802	-7.34615	2.04E-13	5.00E-13	cyan	-0.55364114	5.46E-14		AT3G03980.1		NAD(P)-binding Rossmann-fold superfamily protein	NA
Sapur.T139400.1	264.7795524	0.757542264	0.103360495	7.329128	2.32E-13	5.66E-13	cyan	0.488209505	8.81E-11	GO:0005524	AT3G19740.1		P-loop containing nucleoside triphosphate hydrolases superfamily protein	NA
Sapur.15WG043100.1	846.0827132	0.264477172	0.036123443	7.321483	2.45E-13	5.99E-13	cyan	0.513773518	5.92E-12	GO:0016779,GO:0043631,GO:0003723,GO:0004652,GO:0005634	AT1G17980.1	PAPS1	poly(A) polymerase 1	poly(A) polymerase
Sapur.001G114800.1	26.25579817	1.070596912	0.146855447	7.290141	3.10E-13	7.53E-13	cyan	0.42412211	3.11E-08					NA
Sapur.005G023800.1	4.392619085	1.806495062	0.248710476	7.263446	3.77E-13	9.15E-13	cyan	0.387899531	5.18E-07	GO:0008017,GO:0007018,GO:0005524,GO:0003777,GO:0005871	AT5G27550.1		P-loop containing nucleoside triphosphate hydrolases superfamily protein	NA
Sapur.014G024100.2	4.259380737	1.210708076	0.16672227	7.261826	3.82E-13	9.26E-13	cyan	0.471517771	4.57E-10	GO:0016788,GO:0006886,GO:0006505	AT4G34310.1		alpha/beta-Hydrolases superfamily protein	ribonuclease P/mrp subunit, putative
Sapur.003G169900.1	949.8749668	-0.20517876	0.028264453	-7.25925	3.89E-13	9.43E-13	cyan	-0.526518038	1.41E-12		AT2G32080.2	PUR ALPHA-1	purin-rich alpha 1	transcription factor Pur-alpha 1
Sapur.004G162500.1	29.16755605	0.912135292	0.125704437	7.25619	3.98E-13	9.64E-13	cyan	0.438447515	9.28E-09	GO:0006508,GO:0004185	AT3G45010.1	scpl48	serine carboxypeptidase-like 48	serine carboxypeptidase
Sapur.T105400.1	14.83188847	0.890604197	0.123477771	7.212668	5.49E-13	1.32E-12	cyan	0.484355293	1.30E-10					NA
Sapur.017G056400.1	48.77872633	0.554485575	0.076995029	7.201576	5.95E-13	1.43E-12	cyan	0.531774054	7.70E-13					NA
Sapur.014G080800.1	1385.078097	0.79578345	0.110785461	7.183104	6.81E-13	1.64E-12	cyan	0.483363737	1.43E-10		AT2G46640.2			hypothetical protein
Sapur.018G078100.1	0.840833901	3.191412336	0.44459	7.178327	7.06E-13	1.69E-12	cyan	0.391365064	4.01E-07					NA
Sapur.003G061100.2	1.997387328	1.858525254	0.258968846	7.176636	7.14E-13	1.72E-12	cyan	0.484717585	1.25E-10		AT5G46840.1		RNA-binding (RRM/RBD/RNP motifs) family protein	RNA recognition motif protein
Sapur.001G036600.1	1.848521809	2.846015303	0.396664682	7.174864	7.24E-13	1.74E-12	cyan	0.365032858	2.59E-06		AT3G50160.1		Plant protein of unknown function (DUF247)	NA
Sapur.010G172100.1	0.724684543	2.735335114	0.382144113	7.157863	8.19E-13	1.96E-12	cyan	0.447965838	4.03E-09		AT1G49290.1			hypothetical protein
Sapur.T039900.1	340.1796061	0.780558966	0.109067354	7.156669	8.27E-13	1.98E-12	cyan	0.493001606	5.40E-11		AT1G50140.1		P-loop containing nucleoside triphosphate hydrolases superfamily protein	NA
Sapur.004G164600.1	20.92993629	0.836374823	0.117839657	7.097567	1.27E-12	3.03E-12	cyan	0.461453621	1.18E-09		AT2G27930.1		PLATZ transcription factor family protein	PLATZ transcription factor
Sapur.006G142900.1	359.6750357	0.641663717	0.090483985	7.091462	1.33E-12	3.16E-12	cyan	0.453373573	2.48E-09		AT4G30440.1	GAE1	UDP-D-glucuronate 4-epimerase 1	UDP-glucuronic acid 4-epimerase
Sapur.013G139200.1	834.4993642	0.498441092	0.070411061	7.079017	1.45E-12	3.45E-12	cyan	0.442054895	6.79E-09	GO:0006468,GO:0005524,GO:0004672	AT5G58350.1	WNK4,ZIK2	with no lysine (K) kinase 4	MAP kinase
Sapur.008G073100.2	49.94836303	0.585329685	0.082784128	7.070554	1.54E-12	3.67E-12	cyan	0.486871952	1.01E-10		AT1G69340.1		appr-1-p processing enzyme family protein	appr-1-P processing enzyme family protein
Sapur.012G108800.1	716.8360179	1.381447415	0.195716601	7.058407	1.68E-12	4.00E-12	cyan	0.489597234	7.66E-11		AT5G52390.1		PAR1 protein	PAR-1a protein
Sapur.017G043500.1	1571.759699	0.712741072	0.101138285	7.047194	1.83E-12	4.33E-12	cyan	0.512512747	6.80E-12	GO:0006355,GO:0003700	AT4G13620.1		Integrase-type DNA-binding superfamily protein	transcription factor 2
Sapur.013G018800.1	1719.698663	0.646148095	0.091717264	7.045	1.85E-12	4.39E-12	cyan	0.488206882	8.82E-11	GO:0005515	AT1G09070.1	(AT)SRC2,SRC2	soybean gene regulated by cold-2	C2 domain protein
Sapur.008G052300.1	40.77555509	-0.628434902	0.089271297	-7.03961	1.93E-12	4.56E-12	cyan	-0.500364685	2.51E-11		AT2G38120.1	AUX1,MAP1,PIR1,WAV5	Transmembrane amino acid transporter family protein	auxin influx carrier component
Sapur.012G090200.1	216.407892	0.977409491	0.139557626	7.003627	2.49E-12	5.88E-12	cyan	0.40784362	1.15E-07		AT4G19840.1	ATPP2-A1,ATPP2A-1,PP2-A1	phloem protein 2-A1	dimeric phloem specific lectin PP2, putative
Sapur.006G079900.1	451.5182956	0.317259793	0.045357	6.994726	2.66E-12	6.26E-12	cyan	0.483753531	1.38E-10		AT2G38090.1		Duplicated homeodomain-like superfamily protein	MYB transcription factor
Sapur.019G028700.1	196.7710081	0.476860228	0.06832478	6.979316	2.97E-12	6.98E-12	cyan	0.457850749	1.65E-09	GO:0005524	AT5G40010.1	AATP1	AAA-ATPase 1	AAA family ATPase
Sapur.003G092800.1	20260.15614	0.294153406	0.042185042	6.972931	3.10E-12	7.30E-12	cyan	0.448111112	3.98E-09	GO:0016020,GO:0006810,GO:0005215	AT4G00430.1	PIP1;4,PIP1E,TMP-C	plasma membrane intrinsic protein 1;4	NA
Sapur.001G105200.1	788.739382	0.398712707	0.057280449	6.960712	3.39E-12	7.95E-12	cyan	0.474693674	3.37E-10		AT5G45275.1		Major facilitator superfamily protein	major facilitator protein
Sapur.013G021400.1	639.9344419	-0.196080485	0.028190358	-6.95559	3.51E-12	8.24E-12	cyan	-0.553329227	5.68E-14	GO:0005515	AT1G29150.1	ATS9,RPN6	non-ATPase subunit 9	26S proteasome non-ATPase regulatory subunit 11
Sapur.006G221000.1	2617.847529	-0.278235128	0.040010548	-6.95404	3.55E-12	8.33E-12	cyan	-0.496953536	3.59E-11	GO:0003676,GO:0031369,GO:0006413,GO:0005852,GO:0003743,GO:0003723	AT5G27640.1	ATEIF3B-1,ATTIF3B1,EIF3B,	translation initiation factor 3B1	eukaryotic translation initiation factor 3 subunit B
Sapur.001G086300.2	11.21507224	0.84417766	0.12147829	6.949206	3.67E-12	8.61E-12	cyan	0.463647795	9.64E-10	GO:0008152,GO:0003824	AT3G48990.1		AMP-dependent synthetase and ligase family protein	acyl-CoA synthetase
Sapur.005G113400.1	376.150843	0.280656475	0.040408504	6.94548	3.77E-12	8.83E-12	cyan	0.471812115	4.44E-10		AT1G28200.1	FIP1	FH interacting protein 1	GLABRA 2 expression modulator, putative
Sapur.016G096000.1	47.82242508	-0.560844388	0.081102197	-6.91528	4.67E-12	1.09E-11	cyan	-0.529701897	9.80E-13	GO:0016627,GO:0016021,GO:0006629,GO:0005737	AT2G38050.1	ATDET2,DET2,DWF6	3-oxo-5-alpha-steroid 4-dehydrogenase family protein	NA
Sapur.T032300.1	0.702059856	2.927873114	0.423938339	6.906365	4.97E-12	1.16E-11	cyan	0.437725665	9.88E-09					NA
Sapur.010G150000.1	334.5800875	0.627908925	0.091395674	6.870226	6.41E-12	1.49E-11	cyan	0.432932256	1.49E-08	GO:0006952	AT2G40000.1	ATHSPRO2,HSPRO2	ortholog of sugar beet HS1 PRO-1 2	nematode resistance protein-like HSPRO1 protein
Sapur.T145000.1	175.802779	0.37455108	0.054665258	6.851721	7.30E-12	1.69E-11	cyan	0.487679693	9.30E-11	GO:0005515	AT5G64760.2	RPN5B	regulatory particle non-ATPase subunit 5B	NA
Sapur.001G036100.1	1.547339901	2.858904125	0.418466116	6.831865	8.38E-12	1.94E-11	cyan	0.350738277	6.68E-06		AT3G50160.1		Plant protein of unknown function (DUF247)	NA
Sapur.008G043400.1	316.4887547	0.982960881	0.144289088	6.812441	9.60E-12	2.21E-11	cyan	0.457118438	1.76E-09		AT3G46540.1		ENTH/VHS family protein	ENTH domain protein
Sapur.014G064500.1	394.9800663	-0.423528166	0.062370358	-6.79054	1.12E-11	2.57E-11	cyan	-0.478893533	2.23E-10	GO:0016161,GO:0000272	AT4G00490.1	BAM2,BMY9	beta-amylase 2	beta-amylase
Sapur.016G155400.1	720.0620566	0.372012298	0.0549322	6.772208	1.27E-11	2.91E-11	cyan	0.448314498	3.91E-09	GO:0008270,GO:0003677	AT3G12630.1		A20/AN1-like zinc finger family protein	zinc finger A20 and AN1 domain stress-associated protein
Sapur.014G023400.1	8.716442671	1.380035716	0.203797508	6.771603	1.27E-11	2.93E-11	cyan	0.433997752	1.36E-08	GO:0006629	AT2G26560.1	PLA IIA,PLA2A,PLP2	phospholipase A 2A	patatin-like phospholipase
Sapur.018G075100.1	1344.506244	0.473736665	0.070477619	6.721803	1.79E-11	4.10E-11	cyan	0.441882144	6.89E-09		AT4G30410.1		sequence-specific DNA binding transcription factors	transcription factor, putative
Sapur.005G077600.2	0.663445795	2.112690077	0.317660068	6.650789	2.92E-11	6.61E-11	cyan	0.439844169	8.23E-09	GO:0006355,GO:0003677	AT1G56010.2	anac021,ANAC022,NAC1	NAC domain containing protein 1	NAC transcription factor
Sapur.005G130200.1	1297.649991	0.235829233	0.035531508	6.637186	3.20E-11	7.24E-11	cyan	0.458087294	1.61E-09		AT1G36050.2		Endoplasmic reticulum vesicle transporter protein	endoplasmic reticulum vesicle transporter protein
Sapur.002G175700.1	232.9705689	1.814735369	0.273435168	6.636803	3.21E-11	7.25E-11	cyan	0.413714512	7.22E-08	GO:0030244,GO:0016760,GO:0016020	AT2G32540.1	ATCSLB04,ATCSLB4,CSLB04	cellulose synthase-like B4	cellulose synthase-like protein
Sapur.009G015700.1	369.9960711	0.309899404	0.046854841	6.614032	3.74E-11	8.44E-11	cyan	0.479945937	2.01E-10		AT2G26920.1		Ubiquitin-associated/translation elongation factor EF1B protein	ubiquitin-associated/TS-N domain protein, putative
Sapur.T039800.1	54.92705012	0.801028605	0.121566889	6.589201	4.42E-11	9.96E-11	cyan	0.456838522	1.81E-09					NA
Sapur.006G087100.1	236.3709561	0.3141769	0.047889886	6.560402	5.37E-11	1.20E-10	cyan	0.480241815	1.96E-10	GO:0006886,GO:0005622	AT5G01760.1		ENTH/VHS/GAT family protein	VHS/GAT domain protein, putative
Sapur.014G007100.1	162.5900629	0.468877449	0.071496817	6.558019	5.45E-11	1.22E-10	cyan	0.434703594	1.28E-08		AT5G63905.1			hypothetical protein
Sapur.016G216300.1	580.7268408	-0.262601435	0.040141681	-6.54186	6.08E-11	1.36E-10	cyan	-0.461383442	1.19E-09	GO:0006396,GO:0009982,GO:0009451,GO:0003723,GO:0001522	AT5G14460.1		Pseudouridine synthase family protein	tRNA pseudouridine synthase B
Sapur.003G098300.1	0.698261115	2.386373622	0.364839901	6.540879	6.12E-11	1.37E-10	cyan	0.401294702	1.90E-07		AT1G64160.1		Disease resistance-responsive (dirigent-like protein) family protein	plant disease resistance response protein, putative
Sapur.014G137000.1	0.845489938	2.200773212	0.336697508	6.536351	6.30E-11	1.41E-10	cyan	0.456974646	1.79E-09	GO:0008168	AT3G11480.1	ATBSMT1,BSMT1	S-adenosyl-L-methionine-dependent methyltransferases superfamily protein	S-adenosyl-L-methionine:salicylic acid carboxyl methyltransferase
Sapur.008G061200.1	274.7133811	1.222090724	0.18753171	6.516715	7.19E-11	1.61E-10	cyan	0.295132962	0.000175157	GO:0016788	AT1G06990.1		GDSL-like Lipase/Acylhydrolase superfamily protein	GDSL esterase/lipase
Sapur.003G149200.1	34.87993987	0.547806054	0.084274467	6.500261	8.02E-11	1.79E-10	cyan	0.536947845	4.19E-13		AT1G74320.1		Protein kinase superfamily protein	choline/ethanolamine kinase
Sapur.011G026700.1	134.0425997	0.976260474	0.150368343	6.49246	8.44E-11	1.88E-10	cyan	0.433098806	1.47E-08	GO:0005515	AT4G05150.1		Octicosapeptide/Phox/Bem1p family protein	octicosapeptide/phox/Bem1p protein, putative
Sapur.016G007900.1	294.0816516	1.331226474	0.207227535	6.423985	1.33E-10	2.94E-10	cyan	0.440477112	7.79E-09	GO:0055114,GO:0020037,GO:0016705,GO:0005506	AT4G31970.1	CYP82C2	cytochrome P450, family 82, subfamily C, polypeptide 2	cytochrome P450 family protein
Sapur.016G136800.1	457.0718966	0.456888053	0.07122651	6.414579	1.41E-10	3.12E-10	cyan	0.456877223	1.80E-09	GO:0055114,GO:0016491,GO:0006633,GO:0005506	AT1G07420.1	ATSMO2,SMO2-1	sterol 4-alpha-methyl-oxidase 2-1	C-4 methyl sterol oxidase
Sapur.010G201500.1	238.0462707	0.278306561	0.043393836	6.413505	1.42E-10	3.14E-10	cyan	0.432059098	1.60E-08		AT1G51660.1	ATMEK4,ATMKK4,MKK4	mitogen-activated protein kinase kinase 4	MAP kinase kinase
Sapur.010G158000.1	479.0574048	0.389603939	0.060769869	6.411137	1.44E-10	3.19E-10	cyan	0.429738133	1.95E-08	GO:0008270,GO:0005515	AT2G39720.1	RHC2A	RING-H2 finger C2A	zinc-finger protein
Sapur.013G007500.1	2.148914581	2.323751786	0.363956442	6.384698	1.72E-10	3.78E-10	cyan	0.339521381	1.36E-05	GO:0042545,GO:0030599,GO:0005618	AT5G18990.1		Pectin lyase-like superfamily protein	pectinesterase
Sapur.011G096000.1	1.842954251	2.475182854	0.388051529	6.37849	1.79E-10	3.94E-10	cyan	0.330199185	2.41E-05	GO:0004674,GO:0048544,GO:0006468,GO:0004672	AT4G21380.1	ARK3,RK3	receptor kinase 3	Serine/Threonine-kinase
Sapur.005G115400.1	418.7568218	0.574483136	0.090170698	6.371062	1.88E-10	4.13E-10	cyan	0.454249005	2.29E-09	GO:0005524	AT3G19740.1		P-loop containing nucleoside triphosphate hydrolases superfamily protein	NA
Sapur.009G070900.1	190.3486538	0.195762807	0.030774444	6.361213	2.00E-10	4.40E-10	cyan	0.476680654	2.77E-10	GO:0070461	AT4G33890.2			transcriptional regulator of RNA polII, SAGA, subunit
Sapur.004G142100.1	2619.19158	0.424179761	0.066760994	6.353706	2.10E-10	4.61E-10	cyan	0.444495423	5.48E-09		AT2G27830.1			hypothetical protein
Sapur.018G018400.1	0.850713077	2.244624622	0.353313188	6.353073	2.11E-10	4.63E-10	cyan	0.390036694	4.43E-07	GO:0016021,GO:0004659	AT2G18950.1	ATHPT,HPT1,TPT1,VTE2	homogentisate phytyltransferase 1	NA
Sapur.008G058300.1	6.212892746	1.413487988	0.222505886	6.352587	2.12E-10	4.64E-10	cyan	0.388282574	5.04E-07		AT2G40330.1	PYL6,RCAR9	PYR1-like 6	polyketide cyclase/dehydrase and lipid transporter
Sapur.T131000.1	241.887005	1.364782489	0.214938566	6.34964	2.16E-10	4.73E-10	cyan	0.433071461	1.47E-08	GO:0055114,GO:0020037,GO:0016705,GO:0005506	AT4G31970.1	CYP82C2	cytochrome P450, family 82, subfamily C, polypeptide 2	NA
Sapur.010G204000.1	81.45711954	0.463252578	0.07298613	6.347132	2.19E-10	4.81E-10	cyan	0.417028139	5.54E-08		AT1G51560.1		Pyridoxamine 5\'-phosphate oxidase family protein	pyridoxamine 5'-phosphate oxidase family protein
Sapur.018G096300.1	432.8570404	0.28954361	0.045625889	6.346038	2.21E-10	4.84E-10	cyan	0.440062569	8.07E-09		AT4G39140.4		RING/U-box superfamily protein	hypothetical protein
Sapur.006G198600.1	26.91150804	0.794188039	0.125211754	6.342759	2.26E-10	4.94E-10	cyan	0.440600374	7.71E-09	GO:0055085,GO:0016021,GO:0016020,GO:0006810,GO:0005215	AT1G52190.1		Major facilitator superfamily protein	peptide transporter, putative
Sapur.013G083200.1	92.69141955	0.565137761	0.089173636	6.337498	2.34E-10	5.11E-10	cyan	0.424377786	3.04E-08	GO:0055114,GO:0020037,GO:0016705,GO:0005506	AT1G64940.1	CYP89A6	cytochrome P450, family 87, subfamily A, polypeptide 6	cytochrome P450 family protein
Sapur.013G029800.1	3726.676428	0.676867176	0.107084392	6.320876	2.60E-10	5.68E-10	cyan	0.451778499	2.87E-09	GO:0055114,GO:0004764,GO:0003855	AT3G06350.1	EMB3004,MEE32	dehydroquinate dehydratase, putative / shikimate dehydrogenase, putative	dehydroquinate dehydratase/shikimate dehydrogenase
Sapur.016G120800.1	1006.709525	0.516086229	0.081709241	6.31613	2.68E-10	5.86E-10	cyan	0.424807816	2.94E-08	GO:0005524,GO:0050515,GO:0016114	AT2G26930.1	ATCDPMEK,CDPMEK,ISPE,P	4-(cytidine 5\'-phospho)-2-C-methyl-D-erithritol kinase	4-diphosphocytidyl-2-C-methyl-D-erythritol kinase
Sapur.004G038400.1	214.7858981	0.22371185	0.035580282	6.287523	3.23E-10	7.02E-10	cyan	0.453242718	2.51E-09	GO:0003676	AT3G15010.2		RNA-binding (RRM/RBD/RNP motifs) family protein	heterogeneous nuclear ribonucleoprotein G RNA recognition motif protein
Sapur.013G113800.1	557.3407038	1.111407171	0.176815012	6.285706	3.26E-10	7.11E-10	cyan	0.408866994	1.06E-07	GO:0055114,GO:0020037,GO:0016705,GO:0005506	AT3G25180.1	CYP82G1	cytochrome P450, family 82, subfamily G, polypeptide 1	cytochrome P450 family protein
Sapur.007G089100.1	847.773255	0.411168497	0.065492159	6.278133	3.43E-10	7.45E-10	cyan	0.455055886	2.13E-09	GO:0005515	AT3G52950.2		CBS / octicosapeptide/Phox/Bemp1 (PB1) domains-containing protein	meiotically up-regulated protein
Sapur.013G019300.1	136.2199205	0.354895425	0.05655343	6.275401	3.49E-10	7.58E-10	cyan	0.443919723	5.77E-09		AT1G09050.1			homeodomain protein, putative
Sapur.016G090300.1	1.810891188	1.329193841	0.212012154	6.269423	3.62E-10	7.87E-10	cyan	0.638209709	2.46E-19		AT5G02390.1		Protein of unknown function (DUF3741)	DUF4378 domain protein
Sapur.T186300.1	15.17391718	0.675894075	0.10784957	6.267008	3.68E-10	8.00E-10	cyan	0.458188608	1.60E-09		AT5G17020.1	ATCRM1,ATXPO1,HIT2,XPO	exportin 1A	NA
Sapur.010G129200.1	63.80610471	0.649196323	0.103848708	6.251366	4.07E-10	8.82E-10	cyan	0.420457072	4.19E-08	GO:0006355,GO:0003677	AT5G39660.2	CDF2	cycling DOF factor 2	Dof zinc finger protein
Sapur.002G193900.5	11.24865892	0.656807122	0.105174685	6.244916	4.24E-10	9.19E-10	cyan	0.424262935	3.07E-08	GO:0006629	AT3G07400.1		lipase class 3 family protein	lipase
Sapur.006G208800.1	7.9534713	0.939975268	0.150910777	6.228682	4.70E-10	1.02E-09	cyan	0.405000049	1.43E-07		AT2G18660.1	PNP-A	plant natriuretic peptide A	rare lipoprotein A-like double-psi beta-barrel protein
Sapur.006G019600.1	68.40499095	0.597125922	0.095898317	6.226657	4.76E-10	1.03E-09	cyan	0.437889713	9.74E-09		AT2G41120.1			DUF309 domain protein
Sapur.012G002200.1	26.55626676	0.813504321	0.130663737	6.225938	4.79E-10	1.04E-09	cyan	0.397903676	2.46E-07	GO:0005515	AT2G33060.1	AtRLP27,RLP27	receptor like protein 27	NA
Sapur.013G127200.1	1.23984024	3.651048766	0.590281123	6.185271	6.20E-10	1.34E-09	cyan	0.362336373	3.11E-06					hypothetical protein
Sapur.017G003400.1	953.335823	0.25155811	0.040735138	6.175457	6.60E-10	1.42E-09	cyan	0.446560432	4.57E-09		AT2G43970.1		RNA-binding protein	LA-related protein 6 LA RNA-binding domain protein
Sapur.010G050000.1	1.677602416	1.908235072	0.309245993	6.170606	6.80E-10	1.46E-09	cyan	0.385268948	6.28E-07	GO:0005515	AT2G29420.1	ATGSTU7,GST25,GSTU7	glutathione S-transferase tau 7	tau class glutathione transferase GSTU37
Sapur.009G095600.1	400.3124311	-0.199211457	0.032287442	-6.16994	6.83E-10	1.47E-09	cyan	-0.534778318	5.41E-13	GO:0046854	AT4G39120.1	HISN7,IMPL2	myo-inositol monophosphatase like 2	inositol monophosphatase family protein
Sapur.003G135700.1	28.61045105	0.695413598	0.113083742	6.149545	7.77E-10	1.67E-09	cyan	0.415274063	6.37E-08	GO:0008270	AT3G24800.1	PRT1	proteolysis 1	zinc finger, ZZ type protein
Sapur.017G003900.1	230.9552885	0.427913848	0.06958878	6.149179	7.79E-10	1.67E-09	cyan	0.439848506	8.23E-09	GO:0006468,GO:0004672	AT2G43850.1		Integrin-linked protein kinase family	ankyrin-kinase, putative
Sapur.001G130800.1	0.542466165	2.23260028	0.363501797	6.141924	8.15E-10	1.75E-09	cyan	0.476020161	2.96E-10		AT1G72510.1		Protein of unknown function (DUF1677)	DUF1677 family protein
Sapur.016G180500.1	984.6417289	-0.372706737	0.060840705	-6.12594	9.01E-10	1.93E-09	cyan	-0.460540926	1.29E-09	GO:0016597,GO:0008152,GO:0055114,GO:0051287,GO:0016616	AT4G34200.1	EDA9	D-3-phosphoglycerate dehydrogenase	D-3-phosphoglycerate dehydrogenase
Sapur.016G197400.1	6.406340724	0.976604342	0.159489323	6.123321	9.16E-10	1.96E-09	cyan	0.447246016	4.30E-09		AT1G56210.1		Heavy metal transport/detoxification superfamily protein	NA
Sapur.15WG024800.1	3482.789974	0.363659461	0.059469766	6.115031	9.65E-10	2.06E-09	cyan	0.42178012	3.76E-08		AT1G48450.1		Protein of unknown function (DUF760)	plant/T1N15-5 protein
Sapur.016G176200.1	94.9969181	-0.365680216	0.060206209	-6.0738	1.25E-09	2.66E-09	cyan	-0.468383282	6.17E-10	GO:0005515,GO:0009958	AT2G14820.1	NPY2	Phototropic-responsive NPH3 family protein	BTB/POZ domain protein
Sapur.002G106700.1	2.837027875	1.335858415	0.220024963	6.071395	1.27E-09	2.70E-09	cyan	0.325751466	3.14E-05	GO:0008375,GO:0006486	AT4G38240.1	CGL,CGL1,GNTI	alpha-1,3-mannosyl-glycoprotein beta-1,2-N-acetylglucosaminyltransferase, putative	NA
Sapur.016G257600.2	2.18534743	1.312608693	0.216533072	6.061932	1.34E-09	2.85E-09	cyan	0.599923422	1.02E-16	GO:0016020,GO:0008375	AT3G15350.2		Core-2/I-branching beta-1,6-N-acetylglucosaminyltransferase family protein	core-2/I-branching beta-1%2C6-N-acetylglucosaminyltransferase family protein, putative
Sapur.T136400.1	0.414729201	2.041052345	0.337133464	6.054138	1.41E-09	2.99E-09	cyan	0.491089272	6.57E-11					NA
Sapur.017G113800.1	3.504518782	1.447933445	0.239383692	6.048588	1.46E-09	3.10E-09	cyan	0.371601548	1.65E-06	GO:0008152,GO:0003824	AT5G16340.1		AMP-dependent synthetase and ligase family protein	acyl-CoA synthetase
Sapur.010G077700.1	1.924405573	1.329108308	0.220778293	6.020104	1.74E-09	3.68E-09	cyan	0.435252438	1.22E-08					NA
Sapur.004G108100.1	149.6888726	0.320249144	0.053287293	6.009859	1.86E-09	3.92E-09	cyan	0.444753061	5.36E-09		AT1G49560.1		Homeodomain-like superfamily protein	myb-like DNA-binding domain protein, putative
Sapur.013G005000.1	62.04084148	-0.433969946	0.07226504	-6.00525	1.91E-09	4.03E-09	cyan	-0.463386983	9.88E-10		AT1G56690.1		Pentatricopeptide repeat (PPR) superfamily protein	PPR containing plant protein
Sapur.010G171600.1	103.3066258	0.727199655	0.121307535	5.994678	2.04E-09	4.30E-09	cyan	0.459463632	1.42E-09		AT3G45740.1		hydrolase family protein / HAD-superfamily protein	NA

Sapur.017G009200.1	0.650984974	2.310869384	0.386099592	5.985164	2.16E-09	4.55E-09	cyan	0.428390504	2.18E-08		AT4G18425.1		Protein of unknown function (DUF679)	transmembrane protein, putative
Sapur.018G109200.1	66.70126514	0.873716476	0.146142651	5.978518	2.25E-09	4.74E-09	cyan	0.316542163	5.37E-05					NA
Sapur.012G001000.1	0.558955458	1.915818621	0.320865014	5.970793	2.36E-09	4.96E-09	cyan	0.461594171	1.17E-09					NA
Sapur.013G044200.1	14.86851167	0.785976007	0.131934477	5.957321	2.56E-09	5.38E-09	cyan	0.381791403	8.06E-07		AT3G04470.1		Ankyrin repeat family protein	ankyrin repeat protein 13C-B
Sapur.004G019900.1	98.74145817	-0.438281845	0.073868452	-5.93328	2.97E-09	6.22E-09	cyan	-0.450778716	3.14E-09		AT1G62310.1		transcription factor jumonji (jmjC) domain-containing protein	transcription factor, putative
Sapur.006G209500.1	387.6143011	0.186746235	0.031549262	5.919195	3.24E-09	6.76E-09	cyan	0.389814496	4.50E-07		AT2G26660.1	ATSPX2,SPX2	SPX domain gene 2	xenotropic and polytropic murine leukemia virus receptor ids-4, putative
Sapur.018G111400.1	0.843305251	2.253638359	0.381919829	5.900815	3.62E-09	7.55E-09	cyan	0.354054391	5.39E-06	GO:0005515	AT1G58190.2	AtRLP9,RLP9	receptor like protein 9	NA
Sapur.016G221500.2	3.676351231	1.324951675	0.22520463	5.883323	4.02E-09	8.38E-09	cyan	0.375763901	1.24E-06	GO:0008270,GO:0004089	AT3G01500.2	ATBCA1,ATSABP3,CA1,SABP	carbonic anhydrase 1	carbonic anhydrase
Sapur.002G104900.1	15.63371475	0.812187801	0.138136703	5.879595	4.11E-09	8.57E-09	cyan	0.349989097	7.01E-06		AT5G42690.1		Protein of unknown function, DUF547	NA
Sapur.017G078600.1	0.880080412	2.947902425	0.503988885	5.849142	4.94E-09	1.03E-08	cyan	0.392308938	3.74E-07	GO:0016747	AT3G29590.1	AT5MAT	HXXXD-type acyl-transferase family protein	anthocyanin 5-aromatic acyltransferase, putative
Sapur.010G130300.1	2994.671053	0.760384824	0.130611276	5.82174	5.82E-09	1.21E-08	cyan	0.424132694	3.10E-08		AT3G28960.1		Transmembrane amino acid transporter family protein	amino acid transporter, putative
Sapur.T111300.1	0.426452214	1.891569685	0.325216358	5.816342	6.01E-09	1.24E-08	cyan	0.467113598	6.96E-10					NA
Sapur.016G103600.1	0.741119886	2.057054638	0.354667711	5.799949	6.63E-09	1.37E-08	cyan	0.401797388	1.83E-07		AT5G01350.1			NA
Sapur.016G049100.1	42.10610656	0.639348865	0.110422987	5.789998	7.04E-09	1.45E-08	cyan	0.448398336	3.88E-09		AT2G41830.1		Uncharacterized protein	NA
Sapur.006G050200.1	16.65437168	1.526021362	0.265195861	5.754318	8.70E-09	1.79E-08	cyan	0.432953017	1.49E-08		AT3G54070.1		Ankyrin repeat family protein	ankyrin repeat protein
Sapur.002G149300.1	8.111075752	1.050250076	0.183755868	5.715464	1.09E-08	2.24E-08	cyan	0.369474573	1.92E-06		AT2G46150.1		Late embryogenesis abundant (LEA) hydroxyproline-rich glycoprotein family	late embryogenesis abundant protein
Sapur.T079300.1	2.656728253	1.369740307	0.240287671	5.700419	1.20E-08	2.44E-08	cyan	0.360026571	3.63E-06					NA
Sapur.008G009900.1	375.3057431	0.324467649	0.057264172	5.666155	1.46E-08	2.97E-08	cyan	0.399153029	2.24E-07		AT3G11760.1			EEIG1/EHBP1 protein amino-terminal domain protein
Sapur.017G068600.1	3183.775024	0.650697665	0.115284756	5.644265	1.66E-08	3.36E-08	cyan	0.397416711	2.55E-07		AT3G28917.1	MIF2	mini zinc finger 2	ZF-HD protein dimerization region protein
Sapur.15WG022400.1	396.1523865	-0.2414262	0.042861921	-5.63265	1.77E-08	3.60E-08	cyan	-0.433511419	1.42E-08	GO:0016491,GO:0008152	AT4G27760.1	FEY,FEY3	NAD(P)-binding Rossmann-fold superfamily protein	forever young oxidoreductase
Sapur.004G133000.1	868.896138	-0.148993122	0.026477943	-5.62707	1.83E-08	3.71E-08	cyan	-0.42316759	3.36E-08	GO:0005515,GO:0042176,GO:0030234,GO:0000502	AT1G20200.1	EMB2719,HAP15	PAM domain (PCI/PINT associated module) protein	26S proteasome non-ATPase regulatory subunit 3
Sapur.014G117400.1	254.6485144	-0.262408821	0.046858537	-5.60002	2.14E-08	4.33E-08	cyan	-0.428044436	2.24E-08	GO:0005515,GO:0006468,GO:0005524,GO:0004672	AT3G02130.1	RPK2,TOAD2	receptor-like protein kinase 2	LRR receptor-like kinase
Sapur.014G067400.1	444.024543	-0.253696638	0.045588092	-5.56498	2.62E-08	5.28E-08	cyan	-0.430042389	1.90E-08	GO:0006468,GO:0005524,GO:0004672	AT4G00330.1	CRCK2	calmodulin-binding receptor-like cytoplasmic kinase 2	calmodulin-binding receptor-like cytoplasmic kinase
Sapur.016G178400.1	696.15467	0.242312775	0.043576354	5.560648	2.69E-08	5.41E-08	cyan	0.403608143	1.59E-07		AT2G14910.1			seed maturation protein PM23, putative
Sapur.016G123300.1	0.965469209	2.028760374	0.365850904	5.54532	2.93E-08	5.90E-08	cyan	0.376781211	1.15E-06		AT5G06800.1		myb-like HTH transcriptional regulator family protein	NA
Sapur.011G035100.1	68.6605267	-0.317317501	0.057272971	-5.54044	3.02E-08	6.06E-08	cyan	-0.400806826	1.97E-07		AT4G21720.1			hypothetical protein
Sapur.016G019800.1	22.21659532	1.041631384	0.188404383	5.5287	3.23E-08	6.47E-08	cyan	0.434228908	1.33E-08	GO:0016758,GO:0008152	AT1G22380.1	AtUGT85A3,UGT85A3	UDP-glucosyl transferase 85A3	NA
Sapur.012G038900.1	379.1190635	-0.153328939	0.027803293	-5.51478	3.49E-08	6.99E-08	cyan	-0.403126789	1.65E-07	GO:0031124	AT1G73840.1	ESP1	hydroxyproline-rich glycoprotein family protein	cleavage stimulation factor subunit 2 hinge domain protein
Sapur.014G101800.1	485.3541633	0.392581276	0.071240309	5.510662	3.57E-08	7.16E-08	cyan	0.384086227	6.84E-07		AT4G02340.1		alpha/beta-Hydrolases superfamily protein	NA
Sapur.014G119700.1	17.64040226	0.599808454	0.109102626	5.497654	3.85E-08	7.69E-08	cyan	0.39738876	2.56E-07	GO:0005515	AT5G56190.2		Transducin/WD40 repeat-like superfamily protein	WD40 domain protein
Sapur.016G259600.2	9.558099395	0.616291098	0.112481484	5.479045	4.28E-08	8.54E-08	cyan	0.400620449	2.00E-07		AT4G27470.1	ATRMA3,RMA3	RING membrane-anchor 3	zinc-RING finger protein
Sapur.001G061200.1	14.64165106	1.557252456	0.285147572	5.461216	4.73E-08	9.42E-08	cyan	0.411059032	8.91E-08					NA
Sapur.009G053000.2	33.80924698	0.770788255	0.141501579	5.447206	5.12E-08	1.02E-07	cyan	0.383683066	7.04E-07	GO:0005515	AT2G32560.1		F-box family protein	F-box plant protein
Sapur.001G003900.1	97.6780724	-0.526857736	0.096751781	-5.44546	5.17E-08	1.03E-07	cyan	-0.424294364	3.06E-08	GO:0004045	AT5G19830.1		Peptidyl-tRNA hydrolase family protein	peptidyl-tRNA hydrolase
Sapur.012G031300.1	93.56150101	-0.436193381	0.080217235	-5.43765	5.40E-08	1.07E-07	cyan	-0.41005449	9.64E-08	GO:0006355,GO:0005739,GO:0003690	AT5G07900.1		Mitochondrial transcription termination factor family protein	mTERF protein
Sapur.003G106400.1	0.445105941	1.974940393	0.364757135	5.414398	6.15E-08	1.22E-07	cyan	0.448266779	3.93E-09		AT1G64585.1	DVL12,RTFL22	ROTUNDIFOLIA like 22	NA
Sapur.017G115700.1	2327.64553	-0.282184307	0.052176902	-5.40822	6.37E-08	1.26E-07	cyan	-0.411875955	8.35E-08	GO:0006355,GO:0005634,GO:0003677,GO:0009725	AT5G37020.1	ARF8,ATARF8	auxin response factor 8	auxin response factor
Sapur.001G093000.1	292.2008382	0.210592946	0.038981076	5.402441	6.57E-08	1.30E-07	cyan	0.376873441	1.14E-06		AT3G14180.1		sequence-specific DNA binding transcription factors	Myb/SANT-like DNA-binding domain protein
Sapur.006G130700.1	75.63746961	0.651601487	0.120624314	5.401908	6.59E-08	1.30E-07	cyan	0.348783318	7.58E-06					transmembrane protein, putative
Sapur.018G043000.1	638.3219432	-0.244910986	0.045344271	-5.40115	6.62E-08	1.31E-07	cyan	-0.402089015	1.79E-07	GO:0003824,GO:0006470,GO:0004722	AT1G72770.3	HAB1	homology to ABI1	abscisic insensitive protein 1B
Sapur.006G204900.2	60.6526717	-0.376261491	0.069940363	-5.37975	7.46E-08	1.47E-07	cyan	-0.410047505	9.65E-08		AT4G32570.1	TIFY8	TIFY domain protein 8	TIFY domain protein, putative
Sapur.017G108800.1	9315.322625	1.028378858	0.191741191	5.36337	8.17E-08	1.61E-07	cyan	0.380981362	8.55E-07	GO:0006807,GO:0006542,GO:0004356	AT5G37600.1	ATGLN1:1,ATGSR1,GLN1:1,(	glutamine synthase clone R1	glutamine synthetase
Sapur.012G004300.1	59.32391033	0.677376118	0.126381066	5.359791	8.33E-08	1.64E-07	cyan	0.423457527	3.28E-08	GO:0005515	AT3G28890.1	AtRLP43,RLP43	receptor like protein 43	NA
Sapur.011G119500.1	107.1142658	0.491196904	0.09194096	5.342525	9.17E-08	1.80E-07	cyan	0.306545245	9.43E-05		AT4G20880.1		ethylene-responsive nuclear protein / ethylene-regulated nuclear protein (ERT2)	transmembrane protein, putative
Sapur.011G002100.1	388.4968429	0.276514039	0.051873353	5.33056	9.79E-08	1.92E-07	cyan	0.389667932	4.55E-07		AT1G11710.1		Pentatricopeptide repeat (PPR) superfamily protein	PPR containing plant protein
Sapur.006G229700.1	103.5535836	0.234633974	0.044036949	5.328116	9.92E-08	1.95E-07	cyan	0.390045894	4.43E-07		AT4G31290.1		ChaC-like family protein	NA
Sapur.006G088300.1	68.05428176	0.324477459	0.061467247	5.278868	1.30E-07	2.54E-07	cyan	0.390612292	4.24E-07	GO:0016567,GO:0004842	AT5G01830.1		ARM repeat superfamily protein	U-box protein
Sapur.011G034000.1	175.2768718	0.296040157	0.056172658	5.270182	1.36E-07	2.67E-07	cyan	0.409323214	1.02E-07		AT4G21670.1	ATCPL1,CPL1,FRY2	C-terminal domain phosphatase-like 1	double-stranded RNA-binding motif protein
Sapur.005G115700.1	30.44215698	0.480935515	0.091543163	5.253648	1.49E-07	2.91E-07	cyan	0.369652972	1.89E-06		AT1G78310.1		VQ motif-containing protein	VQ motif protein
Sapur.006G147100.1	125.532225	0.363226605	0.069501538	5.226166	1.73E-07	3.37E-07	cyan	0.345421377	9.39E-06		AT5G57830.1		Protein of unknown function, DUF593	NA
Sapur.017G098800.1	236.8868558	0.252343906	0.048377471	5.216145	1.83E-07	3.55E-07	cyan	0.386346515	5.80E-07	GO:0005515	AT5G16220.1		Octicosapeptide/Phox/Bem1p family protein	PB1 domain protein
Sapur.002G193800.1	1906.770042	0.299125852	0.05739599	5.211616	1.87E-07	3.64E-07	cyan	0.372961814	1.50E-06	GO:0016021	AT3G25290.1		Auxin-responsive family protein	auxin-induced in root cultures protein
Sapur.010G123000.1	262.6227634	0.169828001	0.032882239	5.164733	2.41E-07	4.66E-07	cyan	0.424355139	3.05E-08		AT1G26580.1			ATP-binding protein, putative
Sapur.001G115000.1	649.5343126	0.773006962	0.149972153	5.154337	2.55E-07	4.92E-07	cyan	0.404866802	1.45E-07	GO:0055085,GO:0016021,GO:0015299,GO:0006812	AT3G53720.1	ATCHX20,CHX20	cation/H+ exchanger 20	monovalent cation:proton antiporter, putative
Sapur.011G019500.1	10293.51566	0.858049351	0.16671161	5.146908	2.65E-07	5.11E-07	cyan	0.370904563	1.74E-06	GO:0016829,GO:0010333,GO:0008152,GO:0000287	AT1G61680.1	ATTPS14,TPS14	terpene synthase 14	(3S)-linalool/(E)-nerolidol/(E,E)-geranyl linalool synthase
Sapur.005G190700.1	1923.502178	0.266808888	0.051933282	5.137532	2.78E-07	5.37E-07	cyan	0.365828383	2.46E-06	GO:0005975,GO:0004553	AT1G19940.1	AtGH9B5,GH9B5	glycosyl hydrolase 9B5	endo-1%2C4-beta-glucanase
Sapur.001G033900.1	176.2885845	-0.283218405	0.055146346	-5.13576	2.81E-07	5.42E-07	cyan	-0.426485465	2.56E-08	GO:0008170,GO:0006644	AT1G80860.2	ATPLMT,PLMT	phospholipid N-methyltransferase	phospholipid N-methyltransferase
Sapur.003G017300.1	37.57241459	0.633090179	0.123308587	5.134194	2.83E-07	5.46E-07	cyan	0.385348822	6.24E-07	GO:0005975,GO:0004553	AT3G15800.1		Glycosyl hydrolase superfamily protein	glucan endo-1%2C3-beta-glucosidase
Sapur.016G138600.1	465.3459324	-0.23957744	0.046723714	-5.12753	2.94E-07	5.65E-07	cyan	-0.429928712	1.92E-08		AT5G58970.1	ATUCP2,UCP2	uncoupling protein 2	uncoupling protein
Sapur.010G188200.1	128.125019	-0.304678995	0.059433698	-5.12637	2.95E-07	5.68E-07	cyan	-0.401398574	1.89E-07	GO:0008270,GO:0005515	AT4G40070.1		RING/U-box superfamily protein	zinc finger, C3HC4 type (RING finger) protein
Sapur.006G012300.1	738.8619719	0.282090647	0.055312387	5.099954	3.40E-07	6.51E-07	cyan	0.355276522	4.97E-06	GO:0005783	AT1G27350.1		Ribosome associated membrane protein RAMP4	ribosome associated membrane protein RAMP4
Sapur.018G050300.1	690.8309441	0.634813965	0.124952981	5.080423	3.77E-07	7.20E-07	cyan	0.314722992	5.96E-05	GO:0042545,GO:0030599,GO:0005618	AT5G19730.1		Pectin lyase-like superfamily protein	pectinesterase
Sapur.002G150300.1	87.66229714	0.333639642	0.065695812	5.078553	3.80E-07	7.27E-07	cyan	0.360968583	3.41E-06		AT1G01500.1		Erythronate-4-phosphate dehydrogenase family protein	NA
Sapur.003G032500.1	42.9143941	-0.463101621	0.091492712	-5.06162	4.16E-07	7.94E-07	cyan	-0.423979756	3.14E-08		AT1G52720.1			plant/MSJ11-3 protein, putative
Sapur.016G019700.1	21.58121423	0.786870116	0.156031626	5.043017	4.58E-07	8.73E-07	cyan	0.3825012	7.66E-07	GO:0016758,GO:0008152	AT1G22380.1	AtUGT85A3,UGT85A3	UDP-glucosyl transferase 85A3	UDP-glucosyltransferase family protein
Sapur.15WG021000.1	59.90499257	-0.640336589	0.127703421	-5.01425	5.32E-07	1.01E-06	cyan	-0.452136038	2.78E-09		AT5G52870.1			hypothetical protein
Sapur.011G119000.1	61.71422049	0.662968154	0.13321435	4.976702	6.47E-07	1.22E-06	cyan	0.316968747	5.24E-05	GO:0055114,GO:0050660,GO:0016491	AT1G30760.1		FAD-binding Berberine family protein	reticuline oxidase, putative
Sapur.002G050300.1	122.1750173	0.416817528	0.083981314	4.963218	6.93E-07	1.31E-06	cyan	0.341141342	1.23E-05	GO:0005515,GO:0006468,GO:0005524,GO:0004672	AT1G34110.1		Leucine-rich receptor-like protein kinase family protein	LRR receptor-like kinase
Sapur.010G000300.1	571.5592381	0.335566137	0.067698803	4.956751	7.17E-07	1.35E-06	cyan	0.346423091	8.81E-06	GO:0031225,GO:0016049,GO:0010215	AT4G16120.1	ATSEB1,COBL7,SEB1	COBRA-like protein-7 precursor	COBRA-like protein
Sapur.013G042700.1	830.225167	0.175113878	0.035593039	4.919891	8.66E-07	1.63E-06	cyan	0.361325502	3.33E-06	GO:0016787	AT2G39840.1	TOPP4	type one serine/threonine protein phosphatase 4	serine/threonine-protein phosphatase
Sapur.016G188500.1	342.8089623	-0.262171965	0.053323412	-4.91664	8.80E-07	1.66E-06	cyan	-0.364457991	2.70E-06	GO:0003676	AT5G65260.1		RNA-binding (RRM/RBD/RNP motifs) family protein	polyadenylate-binding family protein
Sapur.006G172800.1	354.9794215	0.43854097	0.089245265	4.913885	8.93E-07	1.68E-06	cyan	0.605587881	4.39E-17	GO:0007165	AT5G22400.1		Rho GTPase activating protein with PAK-box/P21-Rho-binding domain	Rho GTPase-activating protein
Sapur.005G109300.1	0.428809127	2.125508021	0.432746919	4.911665	9.03E-07	1.70E-06	cyan	0.442585222	6.48E-09		AT4G36945.1		PLC-like phosphodiesterases superfamily protein	MAP3K-like kinase, putative
Sapur.005G092700.1	2.820107325	1.201448474	0.245440664	4.895067	9.83E-07	1.84E-06	cyan	0.294495915	0.000181178		AT2G36430.1		Plant protein of unknown function (DUF247)	plant/F13I12-260 protein, putative
Sapur.018G010800.1	9.497917504	0.996815699	0.204726872	4.869003	1.12E-06	2.10E-06	cyan	0.349049567	7.45E-06	GO:0006508,GO:0004190	AT5G10770.1		Eukaryotic aspartyl protease family protein	NA
Sapur.014G064400.1	223.8049429	-0.25298129	0.051998023	-4.86521	1.14E-06	2.14E-06	cyan	-0.360465909	3.53E-06	GO:0016161,GO:0000272	AT2G45880.1	BAM7,BMY4	beta-amylase 7	beta-amylase
Sapur.016G030300.1	0.534380109	2.367528841	0.487168654	4.859773	1.18E-06	2.19E-06	cyan	0.391120188	4.09E-07	GO:0016818,GO:0008270,GO:0003676,GO:0005524	AT5G05130.1		DNA/RNA helicase protein	chromatin remodeling complex subunit, putative
Sapur.15WG046200.1	0.593770118	1.581669509	0.325506544	4.859102	1.18E-06	2.20E-06	cyan	0.395992334	2.84E-07		AT1G73860.1		P-loop containing nucleoside triphosphate hydrolases superfamily protein	NA
Sapur.002G000500.1	1246.227157	-0.258154018	0.053140837	-4.85792	1.19E-06	2.21E-06	cyan	-0.373369274	1.46E-06		AT1G76660.1			hypothetical protein
Sapur.002G165000.1	925.7032198	0.544705211	0.113560111	4.796624	1.61E-06	2.99E-06	cyan	0.36347896	2.88E-06		AT2G47270.1		sequence-specific DNA binding transcription factors;transcription regulators	transcription factor, putative
Sapur.016G215100.1	1097.326522	-0.197802735	0.0414203	-4.7755	1.79E-06	3.32E-06	cyan	-0.382988581	7.40E-07		AT4G35850.1		Pentatricopeptide repeat (PPR) superfamily protein	PPR containing plant protein
Sapur.013G127600.1	4.374806352	1.809743507	0.37917583	4.772835	1.82E-06	3.36E-06	cyan	0.307969481	8.72E-05	GO:0046983,GO:0008171	AT4G35160.1		O-methyltransferase family protein	caffeic acid 3-O-methyltransferase
Sapur.017G116200.1	2984.49112	0.210621801	0.044278628	4.756737	1.97E-06	3.63E-06	cyan	0.305269066	0.000101216		AT1G65720.1			transmembrane protein, putative
Sapur.014G017200.1	37.86983304	0.387667762	0.081685379	4.745865	2.08E-06	3.83E-06	cyan	0.383396009	7.19E-07	GO:0043565,GO:0006355,GO:0003700	AT1G45249.1	ABF2,AREB1,ATAREB1	abscisic acid responsive elements-binding factor 2	ABA responsive element-binding factor
Sapur.007G120700.1	853.9699347	-0.173103576	0.036592778	-4.73054	2.24E-06	4.13E-06	cyan	-0.387705838	5.26E-07		AT5G62270.2			eukaryotic regulator protein
Sapur.001G132200.1	23.13928198	2.599724452	0.550951876	4.718605	2.37E-06	4.37E-06	cyan	0.294315289	0.00018292	GO:0005515,GO:0006468,GO:0004672	AT5G20690.1		Leucine-rich repeat protein kinase family protein	NA
Sapur.T018300.1	0.335150971	1.695322305	0.359515457	4.715576	2.41E-06	4.43E-06	cyan	0.472595025	4.12E-10					NA
Sapur.016G178900.1	177.4979863	-0.472102637	0.100456949	-4.69955	2.61E-06	4.79E-06	cyan	-0.374180757	1.38E-06		AT4G34260.1	FUC95A	1,2-alpha-L-fucosidases	alpha-L-fucosidase
Sapur.002G028200.1	2279.373833	-0.234237437	0.050092334	-4.67611	2.92E-06	5.36E-06	cyan	-0.379261108	9.66E-07	GO:0016021,GO:0008250,GO:0006487	AT4G21150.3	HAP6	ribophorin II (RPN2) family protein	dolichyl-diphosphooligosaccharide-protein glycosyltransferase subunit 2
Sapur.009G123300.1	686.5658122	0.211352451	0.045647158	4.630134	3.65E-06	6.66E-06	cyan	0.350767458	6.67E-06	GO:0005515	AT5G21040.1	FBX2	F-box protein 2	NA
Sapur.006G146700.1	352.6752433	0.32874324	0.071247033	4.614132	3.95E-06	7.19E-06	cyan	0.307628279	8.88E-05	GO:0006914	AT4G30790.1			autophagy-related protein
Sapur.010G046200.1	700.0936844	-0.159413716	0.034675243	-4.59734	4.28E-06	7.78E-06	cyan	-0.356600931	4.56E-06	GO:0003676,GO:0006810,GO:0005622	AT5G43960.1		Nuclear transport factor 2 (NTF2) family protein with RNA binding (RRM-RBD-RNP motifs) domain	Ras GTPase-activating protein-binding protein
Sapur.019G050200.1	2.238684177	0.744956916	0.162242336	4.591631	4.40E-06	7.99E-06	cyan	0.423011497	3.40E-08		AT5G17020.1	ATCRM1,ATXPO1,HIT2,XPO	exportin 1A	NA
Sapur.016G266500.2	1.516907549	1.360919588	0.297127269	4.580258	4.64E-06	8.42E-06	cyan	0.339013081	1.40E-05	GO:0016301,GO:0007205,GO:0004143	AT2G20900.3	ATDGK5,DGK5	diacylglycerol kinase 5	diacylglycerol kinase
Sapur.016G160800.1	353.6305009	0.293788459	0.064257442	4.572053	4.83E-06	8.75E-06	cyan	0.34703293	8.48E-06	GO:0008270,GO:0005515	AT5G19430.1		RING/U-box superfamily protein	zinc finger, C3HC4 type (RING finger) protein
Sapur.005G073100.1	14.98398829	0.568646827	0.124995331	4.549345	5.38E-06	9.72E-06	cyan	0.34778149	8.08E-06		AT5G08240.1			transmembrane protein, putative
Sapur.007G086700.1	924.5339152	-0.164294421	0.036448551	-4.50757	6.56E-06	1.18E-05	cyan	-0.363034467	2.97E-06	GO:0005525	AT5G65270.1	AtRABA4a,RABA4a	RAB GTPase homolog A4A	Ras-related protein Rab11D
Sapur.012G031200.1	228.8209308	-0.17949216	0.039828225	-4.50666	6.59E-06	1.18E-05	cyan	-0.346282752	8.89E-06	GO:0005515	AT5G42150.1		Glutathione S-transferase family protein	glutathione S-transferase, carboxy-terminal domain protein
Sapur.003G051500.1	0.647077876	2.260866433	0.504203067	4.484039	7.32E-06	1.31E-05	cyan	0.282918755	0.000330335					transmembrane protein, putative
Sapur.006G025500.1	874.6741802	0.180889558	0.040363182	4.481548	7.41E-06	1.33E-05	cyan	0.334432614	1.86E-05	GO:0006351,GO:0005634,GO:0003677	AT5G05140.1		Transcription elongation factor (TFIIS) family protein	mediator of RNA polymerase II transcription subunit 26b, putative
Sapur.019G079700.1	1267.173473	0.224866059	0.050289278	4.471451	7.77E-06	1.39E-05	cyan	0.310802589	7.44E-05	GO:0033926	AT4G09510.1	CINV2	cytosolic invertase 2	neutral/alkaline invertase
Sapur.004G148900.1	3312.933111	0.878953944	0.196635824	4.469958	7.82E-06	1.40E-05	cyan	0.328651826	2.64E-05	GO:0016705,GO:0016117	AT3G10230.1	LYC	lycopene cyclase	capsanthin/capsorubin synthase, chromoplast protein
Sapur.019G068200.1	523.7298033	-0.457110278	0.102579881	-4.45614	8.34E-06	1.49E-05	cyan	-0.362725837	3.03E-06	GO:0008152,GO:0003824	AT5G16340.1		AMP-dependent synthetase and ligase family protein	acyl:CoA ligase acetate-CoA synthetase-like protein
Sapur.006G118600.1	328.2110834	-0.145327437	0.032769837	-4.43479	9.22E-06	1.64E-05	cyan	-0.386974333	5.54E-07	GO:0016592,GO:0006357,GO:0001104	AT2G28230.1		TATA-binding related factor (TRF) of subunit 20 of Mediator complex	mediator of RNA polymerase II transcription subunit 20, putative
Sapur.001G027900.1	154.5663192	-0.255420453	0.05771057	-4.42589	9.60E-06	1.71E-05	cyan	-0.403925007	1.55E-07	GO:0006355,GO:0005739,GO:0003690	AT5G07900.1		Mitochondrial transcription termination factor family protein	mTERF protein
Sapur.005G193800.1	590.2357346	-0.169132478	0.03843882	-4.40004	1.08E-05	1.92E-05	cyan	-0.377759322	1.07E-06	GO:0005515	AT1G20080.1	ATSYTB,NTMC2T1.2,NTMC	Calcium-dependent lipid-binding (CaLB domain) family protein	synaptotagmin protein
Sapur.016G171200.1	0.695772357	1.777304237	0.404447349	4.394402	1.11E-05	1.97E-05	cyan	0.317890051	4.97E-05		AT2G14610.1	ATPR1,PR 1,PR1	pathogenesis-related gene 1	pathogenesis-related protein 1a, putative
Sapur.006G066400.1	200.9855569	0.637722844	0.145453523	4.384375	1.16E-05	2.06E-05	cyan	0.317773962	5.00E-05	GO:0005515	AT4G07400.1	VFB3	VIER F-box proteine 3	skip-2, putative
Sapur.019G106000.1	4.784609079	0.663921997	0.152682321	4.348388	1.37E-05	2.42E-05	cyan	0.326666797	2.97E-05	GO:0005515	AT4G03260.2		Outer arm dynein light chain 1 protein	protein phosphatase 1 regulatory subunit, putative
Sapur.014G130500.1	2439.305606	0.331286576	0.076238218	4.345413	1.39E-05	2.45E-05	cyan	0.307817566	8.79E-05	GO:0016788	AT2G42990.1		GDSL-like Lipase/Acylhydrolase superfamily protein	GDSL esterase/lipase
Sapur.014G113900.1	447.9836035	-0.158708209	0.036587618	-4.33776	1.44E-05	2.54E-05	cyan	-0.371459219	1.67E-06	GO:0006418,GO:0005524,GO:0004812,GO:0000166,GO:0004831	AT3G02660.1	emb2768	Tyrosyl-tRNA synthetase, class Ib, bacterial/mitochondrial	tyrosyl-tRNA synthetase
Sapur.002G175000.1	274.5875421	0.225844904	0.052367937	4.312656	1.61E-05	2.84E-05	cyan	0.333816639	1.93E-05	GO:0006468,GO:0005524,GO:0004672	AT3G21630.1	CERK1,LYSM RLK1	chitin elicitor receptor kinase 1	LysM receptor-like kinase
Sapur.011G117900.1	3485.700053	0.722296636	0.168231835	4.29346	1.76E-05	3.09E-05	cyan	0.346797992	8.61E-06	GO:0055114,GO:0050660,GO:0016491	AT1G30700.1		FAD-binding Berberine family protein	reticuline oxidase, putative
Sapur.008G065400.1	661.935145	0.178060049	0.041508469	4.289728	1.79E-05	3.14E-05	cyan	0.303983737	0.000108627	GO:0055114,GO:0003824,GO:0006164,GO:0003938	AT1G16350.1		Aldolase-type TIM barrel family protein	NA
Sapur.011G056400.1	8146.264212	0.640381662	0.149597343	4.280702	1.86E-05	3.27E-05	cyan	0.381347028	8.33E-07		AT1G30100.1	ATNCED5,NCED5	nine-cis-epoxycarotenoid dioxygenase 5	9-cis-epoxycarotenoid dioxygenase
Sapur.018G105400.1	564.71425	1.649801686	0.385452684	4.280167	1.87E-05	3.27E-05	cyan	0.391639437	3.93E-07	GO:0006468,GO:0004672	AT2G17220.1		Protein kinase superfamily protein	NA
Sapur.T184400.1	0.286900905	1.574049851	0.369477825	4.260201	2.04E-05	3.57E-05	cyan	0.460704321	1.27E-09					NA
Sapur.006G056400.1	1619.043106	0.245973862	0.057958051	4.243998	2.20E-05	3.84E-05	cyan	0.299078908	0.000141822	GO:0005515	AT2G41475.1		Embryo-specific protein 3, (ATS3)	embryo-specific protein
Sapur.008G168600.1	162.0398452	0.219568071	0.051740622	4.24363	2.20E-05	3.84E-05	cyan	0.321378371	4.06E-05		AT3G06150.1			cytochrome P450 family protein, putative
Sapur.005G162000.1	3277.476531	0.367920571	0.087271169	4.215832	2.49E-05	4.33E-05	cyan	0.335714188	1.72E-05	GO:0016740,GO:0009058	AT5G17230.1	PSY	PHYTOENE SYNTHASE	phytoene synthase
Sapur.006G057400.3	24.19034156	0.385444553	0.091530973	4.211083	2.54E-05	4.42E-05	cyan	0.328785229	2.62E-05	GO:0009058,GO:0003824	AT2G38670.1	PECT1	phosphorylethanolamine cytidylyltransferase 1	ethanolamine-phosphate cytidylyltransferase
Sapur.006G168100.1	60.91194287	0.326596204	0.077619896	4.207635	2.58E-05	4.49E-05	cyan	0.579738845	1.78E-15	GO:0006355,GO:0005667,GO:0003700	AT2G36010.1	ATE2FA,E2F3	E2F transcription factor 3	transcription factor E2F
Sapur.006G179500.1	4.979892833	0.743519533	0.176797253	4.205493	2.61E-05	4.53E-05	cyan	0.312002489	6.95E-05		AT2G26270.1			DNA topoisomerase 2-binding protein
Sapur.012G059100.1	76.57141188	-0.208922601	0.049729869	-4.20115	2.66E-05	4.61E-05	cyan	-0.359202573	3.84E-06		AT2G36330.1		Uncharacterised protein family (UPF0497)	DUF588 domain protein
Sapur.005G193600.1	551.4784251	0.278121334	0.066213612	4.200365	2.66E-05	4.63E-05	cyan	0.294864777	0.000177669	GO:0006351,GO:0005634,GO:0003677	AT4G24200.1		Transcription elongation factor (TFIIS) family protein	TFIIS helical bundle-like domain protein
Sapur.008G045600.1	431.9491874	0.164603628	0.039805208	4.135228	3.55E-05	6.12E-05	cyan	0.290518362	0.000223349	GO:0008270,GO:0005515	AT2G39720.1	RHC2A	RING-H2 finger C2A	zinc-finger protein
Sapur.006G136100.1	12.02575353	0.702471694	0.170072619	4.130422	3.62E-05	6.25E-05	cyan	0.279137688	0.000399714	GO:0046872,GO:0000166	AT4G30190.1	AHA2,HA2,PMA2	H(+)-ATPase 2	plasma membrane H+-ATPase
Sapur.013G118100.1	4.570718825	0.754976783	0.182971147	4.126207	3.69E-05	6.36E-05	cyan	0.247049246	0.001812944		AT1G12300.1		Tetratricopeptide repeat (TPR)-like superfamily protein	PPR containing plant protein, putative
Sapur.010G085300.1	24.12051094	0.712954045	0.173653897	4.105603	4.03E-05	6.93E-05	cyan	0.325419986	3.20E-05	GO:0043565,GO:0006355,GO:0003700,GO:0003677	AT2G01430.1	ATHB-17,ATHB17,HB17	homeobox-leucine zipper protein 17	homeobox leucine zipper protein ATHB-4
Sapur.010G171100.1	923.5047081	-0.244868366	0.059851078	-4.09129	4.29E-05	7.37E-05	cyan	-0.303353595	0.000112442	GO:0005515,GO:0000160	AT5G02810.1	APRR7,PRR7	pseudo-response regulator 7	two-component response regulator-like PRR73 protein
Sapur.016G140900.1	159.1999105	0.832939678	0.20397595	4.083519	4.44E-05	7.61E-05	cyan	0.32216585	3.88E-05		AT5G58760.1	DDB2	damaged DNA binding 2	NA
Sapur.017G016400.1	859.7038926	0.180557522	0.044869261	4.024081	5.72E-05	9.75E-05	cyan	0.294787646	0.000178397	GO:0043565,GO:0006355,GO:0003700	AT1G06070.1		Basic-leucine zipper (bZIP) transcription factor family protein	transcription factor RF2a
Sapur.008G031300.1	41.13771474	0.528313523	0.132493799	3.987458	6.68E-05	0.000113402	cyan	0.274244068	0.00050954	GO:0046983,GO:0003677	AT5G60440.1	AGL62	AGAMOUS-like 62	MADS box protein, putative
Sapur.006G059100.1	46.90089928	-0.498755474	0.126691652	-3.93677	8.26E-05	0.00013939	cyan	-0.313543738	6.37E-05	GO:0051205,GO:0016021	AT5G62050.1	ATOXA1,OXA1,OXA1AT	homolog of yeast oxidase assembly 1 (OXA1)	inner membrane protein Oxa1
Sapur.001G120100.1	1450.920499	0.59616771	0.15158328	3.932938	8.39E-05	0.000141535	cyan	0.314086094	6.18E-05	GO:0055085,GO:0016021,GO:0016020,GO:0006810,GO:0005215	AT1G32450.1	NRT1.5	nitrate transporter 1.5	peptide transporter, putative
Sapur.008G022900.1	64.98464936	0.304829926	0.077507545	3.932906	8.39E-05	0.000141547	cyan	0.29120576	0.000215463	GO:0005525	AT5G03530.1	ATRAB,ATRAB ALPHA,ATRA	RAB GTPase homolog C2A	Ras small GTPase family Ras protein
Sapur.011G070300.1	0.476684366	2.090473762	0.53355998	3.917973	8.93E-05	0.000150369	cyan	0.349466637	7.25E-06	GO:0006281,GO:0003678,GO:0000723	AT5G28780.1		PIF1 helicase	NA
Sapur.011G060300.1	17.38466171	0.9430481	0.24078541	3.91655	8.98E-05	0.000151236	cyan	0.268289911	0.000680537		AT1G78600.2	DBB3,LZF1,STH3	light-regulated zinc finger protein 1	NA
Sapur.005G074700.1	1901.242816	-0.087713913	0.022509459	-3.89676	9.75E-05	0.000163864	cyan	-0.298390414	0.000147176	GO:0006418,GO:0005524,GO:0004812,GO:0000166,GO:0006434,GO:0005737,GO:000482	AT5G27470.1		seryl-tRNA synthetase / serine--tRNA ligase	seryl-tRNA synthetase
Sapur.001G064000.1	37.94550397	0.270702152	0.069879553	3.873839	0.000107134	0.000179738	cyan	0.340278283	1.30E-05					NA
Sapur.018G094800.1	212.6798317	-0.276624196	0.073472877	-3.76498	0.00016656	0.0002762	cyan	-0.321583042	4.01E-05	GO:0016798,GO:0016020	AT5G34940.2	AtGUS3,GUS3	glucuronidase 3	glycoside hydrolase family 79 amino-terminal domain protein
Sapur.003G141300.1	2.609367581	0.877476458	0.237651262	3.692286	0.000222247	0.000365338	cyan	0.286600182	0.000273661	GO:0005634	AT5G22250.1		Polynucleotidyl transferase, ribonuclease H-like superfamily protein	CAF1 family ribonuclease
Sapur.012G031500.1	766.2935612	-0.094894147	0.025755074	-3.68448	0.000229167	0.000376301	cyan	-0.295853522	0.000168571	GO:0016884	AT3G17970.1	atToc64-III,TOC64-III	translocon at the outer membrane of chloroplasts 64-III	amidase
Sapur.010G195600.1	1096.011035	0.302934107	0.082503428	3.671776	0.000240871	0.00039499	cyan	0.264060233	0.000832546		AT5G04830.2		Nuclear transport factor 2 (NTF2) family protein	nuclear transport factor 2 (NTF2) family protein
Sapur.006G033300.1	81.13514204	0.276782105	0.075465568	3.667661	0.00024478	0.000401102	cyan	0.268581606	0.00067106	GO:0008080	AT2G41450.1		N-acetyltransferases;N-acetyltransferases	GCN5-related N-acetyltransferase; BRCT protein
Sapur.014G138300.1	2054.398307	-0.15532267	0.043947944	-3.53424	0.000408946	0.000660328	cyan	-0.353507437	5.58E-06		AT2G04280.1			transmembrane protein, putative
Sapur.016G044300.1	0.470780996	2.295574537	0.657392894	3.491937	0.000479531	0.000771102	cyan	0.314635939	5.99E-05					NA
Sapur.013G120700.1	13.13982769	0.612504516	0.178488177	3.431625	0.000599976	0.000958536	cyan	0.209641383	0.00840983					NA
Sapur.003G046000.1	657.4097098	-0.11952434	0.035220101	-3.39364	0.000689703	0.00109738	cyan	-0.290392085	0.000224826		AT4G16650.1		O-fucosyltransferase family protein	GDP-fucose protein O-fucosyltransferase
Sapur.001G052700.1	150.7786876	0.249419097	0.075680818	3.295671	0.000981868	0.001544042	cyan	0.25789194	0.001110615	GO:0046872,GO:0042393	AT1G57820.1	ORTH2,VIM1	Zinc finger (C3HC4-type RING finger) family protein	E3 ubiquitin-protein ligase orthrus protein
Sapur.005G144400.1	114.806422	-0.128968542	0.039234374	-3.28713	0.001012136	0.001590431	cyan	-0.272874549	0.000544925	GO:0005515	AT4G08455.1		BTB/POZ domain-containing protein	C2-BTB1+-+bric-A-brac+tramtrack+broad+complex+BTB+domain+with+C2+subfamil protein Y
Sapur.004G028700.1	2632.750998	-0.098174606	0.029948786	-3.27808	0.001045146	0.001641288	cyan	-0.261520825	0.000938199	GO:0055114,GO:0009082,GO:0004455,GO:0016491	AT3G58610.3		ketol-acid reductoisomerase	ketol-acid reductoisomerase
Sapur.018G105500.1	274.5248211	1.351236936	0.414013475	3.263751	0.001099477	0.001723662	cyan	0.362958486	2.99E-06		AT2G17220.1		Protein kinase superfamily protein	NA
Sapur.018G105300.1	165.7816591	1.303887503	0.399764525	3.261639	0.001107702	0.001735898	cyan	0.363279178	2.92E-06	GO:0006468,GO:0004672	AT2G17220.1		Protein kinase superfamily protein	NA
Sapur.016G135700.1	76.56006557	0.270740043	0.085822142	3.154664	0.001606829	0.00249106	cyan	0.213926018	0.007139145		AT5G59600.1		Tetratricopeptide repeat (TPR)-like superfamily protein	NA
Sapur.011G075800.1	218.0568102	0.272988792	0.087029502	3.136739	0.001708384	0.002644005	cyan	0.218282546	0.006025042		AT1G53380.1		Plant protein of unknown function (DUF641)	UNE1-like protein
Sapur.010G193600.1	946.9488424	-0.223088827	0.072620793	-3.07197	0.002126518	0.003269908	cyan	-0.235194766	0.003025433	GO:0006526,GO:0005524,GO:0004055	AT4G24830.1		arginosuccinate synthase family	argininosuccinate synthase
Sapur.002G121300.1	1377.193485	-0.084181968	0.028536471	-2.94998	0.003177964	0.004821467	cyan	-0.275368151	0.000482095	GO:0051603,GO:0005839,GO:0004298	AT3G60820.3	PBF1	N-terminal nucleophile aminohydrolases (Ntn hydrolases) superfamily protein	proteasome subunit beta type-1 protein
Sapur.009G061000.1	1205.936212	-0.114458578	0.03918454	-2.92101	0.003488945	0.005274885	cyan	-0.263119829	0.000870327		AT2G30200.1		catalytics;transferases;[acyl-carrier-protein] S-malonyltransferases;binding	malonyl CoA-acyl carrier transacylase
Sapur.016G113800.1	190.4814312	0.104750538	0.036752275	2.850178	0.004369472	0.006557281	cyan	0.252846562	0.0013987	GO:0019867	AT3G44160.1		Outer membrane OMP85 family protein	outer envelope protein of 80 kDa protein
Sapur.009G085700.1	8042.602104	0.478925324	0.168481026	2.842607	0.004474623	0.006709905	cyan	0.280819332	0.000367342	GO:0055114,GO:0020037,GO:0016705,GO:0005506	AT4G31940.1	CYP82C4	cytochrome P450, family 82, subfamily C, polypeptide 4	cytochrome P450 family protein
Sapur.15WG097200.1	2530.670287	-0.097578233	0.035340432	-2.76109	0.005760819	0.008545246	cyan	-0.262189645	0.00090924	GO:0016021,GO:0016020	AT5G33320.1	ARAPPT,CUE1,PPT	Glucose-6-phosphate/phosphate translocator-related	triose phosphate/phosphate translocator, non-green plastid protein
Sapur.001G097300.1	524.3036805	0.371658165	0.135126572	2.750445	0.005951444	0.008816934	cyan	0.219621678	0.005715241	GO:0055114,GO:0020037,GO:0016705,GO:0005506	AT4G12320.1	CYP706A6	cytochrome P450, family 706, subfamily A, polypeptide 6	NA
Sapur.006G229800.1	444.913736	0.124173658	0.04637414	2.677649	0.007414092	0.010893545	cyan	0.213503513	0.007256376		AT4G31290.1		ChaC-like family protein	cation transporter ChaC
Sapur.009G093500.1	13.51289984	-0.25799602	0.101271385	-2.54757	0.010847585	0.015704663	cyan	0.325596395	3.17E-05	GO:0016021	AT2G21600.1	ATRER1B,RER1B	endoplasmatic reticulum retrieval protein 1B	RER1A protein
Sapur.009G082300.1	1352.121869	0.153207733	0.061864972	2.476486	0.013268286	0.019032627	cyan	0.169261148	0.034073254		AT2G03550.1		alpha/beta-Hydrolases superfamily protein	gibberellin receptor GID1
Sapur.016G199500.1	0.226458817	1.462732301	0.597856682	2.446627	0.014419999	0.020609612	cyan	0.366919702	2.28E-06					NA
Sapur.006G023100.1	5.696986555	0.280742664	0.119976122	2.339988	0.019284369	0.027216182	cyan	0.469776378	5.40E-10	GO:0005515	AT1G79590.1	ATSYP52,SYP52	syntaxin of plants 52	syntaxin-52 protein
Sapur.006G086100.1	568.0095244	0.190621168	0.082353963	2.314657	0.020631717	0.02903346	cyan	0.195945772	0.01391446		AT5G01710.1		methyltransferases	methyltransferase FkbM domain protein
Sapur.007G109500.4	0.330352207	0.975791007	0.436113434	2.237471	0.025255596	0.03523447	cyan	0.436534929	1.09E-08	GO:0006413,GO:0003743	AT5G54760.3		Translation initiation factor SUI1 family protein	translation initiation factor SUI1, eukaryotic protein
Sapur.004G012100.2	0.260094556	1.5248588	0.717142855	2.126297	0.033478526	0.046136199	cyan	0.267353037	0.000711817	GO:0005975,GO:0004553	AT4G21760.1	BGLU47	beta-glucosidase 47	NA
Sapur.003G038600.1	185.4733342	0.096894417	0.047980886	2.019438	0.04344173	0.059139402	cyan	0.186783388	0.019163266	GO:0006241,GO:0006228,GO:0006183,GO:0006165,GO:0004550	AT1G17410.1		Nucleoside diphosphate kinase family protein	nucleoside diphosphate kinase
Sapur.006G018200.1	134.7229272	0.191271032	0.096644953	1.97911	0.047803578	0.064754896	cyan	0.162714015	0.041740498	GO:0006281	AT4G14970.1			fanconi anaemia protein FancD2 nuclease
Sapur.014G051200.1	1515.789287	-0.043507229	0.022886842	-1.90097	0.057305776	0.076955265	cyan	-0.218303235	0.006020144	GO:0051603,GO:0005839,GO:0004298	AT3G60820.1	PBF1	N-terminal nucleophile aminohydrolases (Ntn hydrolases) superfamily protein	proteasome subunit beta type-1 protein
Sapur.008G036900.1	1505.019963	0.136576274	0.080232021	1.702266	0.08870544	0.116260897	cyan	0.134297626	0.093561502	GO:0016887,GO:0005524,GO:0016020	AT3G55090.1		ABC-2 type transporter family protein	white-brown-complex ABC transporter family protein
Sapur.011G117600.1	0.245430328	1.23969239	0.860188107	1.441188	0.149531705	0.190193885	cyan	0.232406541	0.003400663	GO:0055114,GO:0050660,GO:0016491	AT4G20820.1		FAD-binding Berberine family protein	NA
Sapur.003G032200.1	856.600899	0.077455155	0.055001978	1.408225	0.159064486	0.201489805	cyan	0.121705652	0.128905629		AT1G80280.1		alpha/beta-Hydrolases superfamily protein	alpha/beta fold hydrolase
Sapur.018G078400.2	0.129743309	0.845840993	1.007067037	0.839905	0.400961457	0.471835168	cyan	0.438023874	9.63E-09		AT1G75400.1		RING/U-box superfamily protein	hypothetical protein
Sapur.017G099100.1	156.0565581	0.051481074	0.066135841	0.778414	0.4363249	0.509102157	cyan	0.068376038	0.394825267		AT3G02600.1	ATLPP3,LPP3	lipid phosphate phosphatase 3	lipid phosphate phosphatase
Sapur.009G039800.1	1544.934546	0.017403195	0.023836751	0.730099	0.4653295	0.539329544	cyan	0.055229162	0.492073883	GO:0006468,GO:0004672	AT5G19450.1	CDPK19,CPK8	calcium-dependent protein kinase 19	calcium-dependent kinase
Sapur.016G034200.1	0.793373754	-0.178391849	0.297269144	-0.6001	0.548438166	0.623849357	cyan	0.670079605	8.23E-22		AT3G10250.1		Plant protein 1589 of unknown function	plant-specific domain TIGR01589 family protein, putative
Sapur.013G052700.1	700.9118456	0.026680225	0.057075611	0.467454	0.640175071	0.712780687	cyan	0.033779125	0.674493579	GO:0003924,GO:0007017,GO:0005874	AT5G19780.1	TUA5	tubulin alpha-5	alpha-tubulin
Sapur.003G049900.1	0.975505857	0.097923413	0.316446604	0.309447	0.756981618	0.819441955	cyan	0.521331039	2.55E-12		AT4G16845.1	VRN2	VEFS-Box of polycomb protein	polycomb protein embryonic flower, putative
Sapur.001G088000.1	6.281562182	0.041442297	0.154160099	0.268826	0.788063314	0.846301905	cyan	0.543527674	1.90E-13	GO:0043565,GO:0006355,GO:0005634,GO:0003700	AT4G11660.1	AT-HSFB2B,HSFB2B	winged-helix DNA-binding transcription factor family protein	heat shock transcription factor
Sapur.006G057400.1	0.286798911	-0.004051243	0.442164079	-0.00916	0.99268964	0.995700162	cyan	0.479075326	2.19E-10	GO:0009058,GO:0003824	AT2G38670.1	PECT1	phosphorylethanolamine cytidylyltransferase 1	ethanolamine-phosphate cytidylyltransferase
Sapur.019G090800.1	0.049306714	0.339441653	1.676739266	0.202442	0.839571569	NA	cyan	0.452985381	2.57E-09	GO:0005515	AT3G59690.1	IQD13	IQ-domain 13	IQ-domain protein
Sapur.012G031700.2	0.011145836	0.164281247	2.904822183	0.056555	0.954899948	NA	cyan	0.621106407	4.02E-18	GO:0005515	AT3G17980.1		Calcium-dependent lipid-binding (CaLB domain) family protein	pollen-specific C2 domain protein
Sapur.003G018000.1	0.005937058	0.05557013	2.904824396	0.01913	0.984737169	NA	cyan	0.386339239	5.81E-07		AT1G52080.1	AR791	actin binding protein family	CHUP1, putative
Sapur.007G030300.1	0.019313279	0.053717485	2.904823276	0.018493	0.98524595	NA	cyan	0.309335212	8.08E-05	GO:0006355,GO:0003677	AT2G23760.3	BLH4,SAW2	BEL1-like homeodomain 4	BEL1-like homeodomain protein
Sapur.15WG133300.1	218.7167815	2.233559677	0.07032993	31.75831	2.44E-221	8.27E-220	cyan	0.883024423	8.67E-53		AT2G35140.2		DCD (Development and Cell Death) domain protein	development and cell death domain protein
Sapur.15WG055300.1	1921.760686	1.471606673	0.051820155	28.39835	2.12E-177	5.21E-176	cyan	0.886433221	1.00E-53	GO:0016832,GO:0008270,GO:0005975	AT1G18270.2		ketose-bisphosphate aldolase class-II family protein	NA
Sapur.010G007600.1	5143.787047	1.429564922	0.051668699	27.66791	1.70E-168	3.86E-167	cyan	0.865748942	1.87E-48	GO:0055114,GO:0016491,GO:0006631,GO:0003857	AT3G06860.1	ATMFP2,MFP2	multifunctional protein 2	glyoxysomal fatty acid beta-oxidation MFP-A protein
Sapur.008G105700.1	475.7944282	1.336356431	0.049169295	27.17868	1.16E-162	2.51E-161	cyan	0.859559719	4.79E-47		AT2G01480.1		O-fucosyltransferase family protein	GDP-fucose protein O-fucosyltransferase, plant specific subfamily protein
Sapur.001G027300.1	703.6916485	1.078880018	0.042973734	25.10557	4.32E-139	7.68E-138	cyan	0.876907299	3.52E-51		AT2G26920.1		Ubiquitin-associated/translation elongation factor EF1B protein	UBA/TS-N domain protein
Sapur.016G251700.1	9591.651706	1.399579736	0.057488949	24.3452	6.52E-131	1.07E-129	cyan	0.843045151	1.35E-43	GO:0015977,GO:0008964,GO:0006099	AT3G14940.1	ATPPC3,PPC3	phosphoenolpyruvate carboxylase 3	phosphoenolpyruvate carboxylase
Sapur.019G062400.1	1457.05045	0.816080827	0.034244797	23.8308	1.60E-125	2.48E-124	cyan	0.879611373	7.02E-52	GO:0006468,GO:0005524,GO:0004672	AT2G40730.1		Protein kinase family protein with ARM repeat domain	amino-terminal kinase-like protein
Sapur.005G003500.1	515.4575748	1.081378942	0.045553885	23.73846	1.45E-124	2.22E-123	cyan	0.87645361	4.60E-51		AT4G10710.1	SPT16	global transcription factor C	global transcription factor group protein
Sapur.006G116000.1	1375.860668	0.836196863	0.036399485	22.97277	8.73E-117	1.22E-115	cyan	0.854548698	5.92E-46		AT5G56930.1	emb1789	CCCH-type zinc finger family protein	NA
Sapur.004G053200.1	2872.867422	1.05048688	0.046212487	22.73167	2.18E-114	2.96E-113	cyan	0.851993282	2.06E-45	GO:0016021	AT5G37310.1		Endomembrane protein 70 protein family	transmembrane 9 family protein
Sapur.019G028200.1	1182.716292	0.930382926	0.042513487	21.88442	3.66E-106	4.51E-105	cyan	0.850364667	4.50E-45		AT5G28850.2		Calcium-binding EF-hand family protein	serine/threonine-protein phosphatase 2A regulatory subunit B protein
Sapur.001G109200.1	969.7809337	0.825086266	0.038258027	21.56636	3.72E-103	4.41E-102	cyan	0.857415046	1.42E-46	GO:0008289	AT4G19040.1	EDR2	ENHANCED DISEASE RESISTANCE 2	enhanced disease resistance 2 protein
Sapur.008G002200.1	4134.096133	0.858833838	0.039955239	21.4949	1.74E-102	2.04E-101	cyan	0.846706719	2.52E-44	GO:0046034,GO:0015992,GO:0033178,GO:0016820,GO:0015991,GO:0005524	AT1G78900.2	VHA-A	vacuolar ATP synthase subunit A	V-type proton ATPase catalytic subunit A
Sapur.005G015300.2	61.33166452	2.003231913	0.093488571	21.42756	7.40E-102	8.63E-101	cyan	0.362387547	3.10E-06	GO:0003824,GO:0006470,GO:0004722	AT1G09160.2		Protein phosphatase 2C family protein	protein phosphatase 2C
Sapur.004G053100.1	2126.072731	0.991972579	0.047018573	21.09746	8.39E-99	9.42E-98	cyan	0.826075703	1.92E-40	GO:0016021	AT2G01970.1		Endomembrane protein 70 protein family	transmembrane 9 family protein
Sapur.019G046100.1	457.7535721	1.201159061	0.056965451	21.08575	1.08E-98	1.21E-97	cyan	0.822160389	9.17E-40		AT5G17910.1			hypothetical protein
Sapur.018G099400.1	1814.262025	0.618224759	0.029541217	20.92753	3.01E-97	3.31E-96	cyan	0.854958978	4.84E-46	GO:0005515	AT5G32440.3		Ubiquitin system component Cue protein	ubiquitin system component CUE protein
Sapur.003G135200.1	145.4173875	-1.017323741	0.049897045	-20.3885	2.12E-92	2.20E-91	cyan	-0.869710496	2.16E-49		AT1G80620.1		S15/NS1, RNA-binding protein	NA
Sapur.002G089400.1	2617.346363	1.467731553	0.072691718	20.19118	1.17E-90	1.18E-89	cyan	0.763102919	3.44E-31		AT1G58250.1	SAB	Golgi-body localisation protein domain ;RNA pol II promoter Fmp27 protein domain	RNA pol II promoter Fmp27 domain protein
Sapur.009G131200.1	256.889046	1.255361167	0.063114659	19.89017	4.95E-88	4.81E-87	cyan	0.78548149	4.06E-34		AT4G38250.1		Transmembrane amino acid transporter family protein	aromatic and neutral amino acid transporter
Sapur.008G081500.1	1116.899944	1.233074005	0.062140064	19.84346	1.26E-87	1.21E-86	cyan	0.80630644	3.56E-37	GO:0005524	AT3G06580.1	GAL1,GALK	Mevalonate/galactokinase family protein	NA
Sapur.010G104300.1	93.00716869	1.944423862	0.098438855	19.75261	7.62E-87	7.30E-86	cyan	0.776325031	7.04E-33		AT5G15948.1	CPuORF10	conserved peptide upstream open reading frame 10	S-adenosyl-L-methionine decarboxylase leader peptide protein
Sapur.016G228800.1	838.044403	0.876822167	0.044508192	19.70024	2.15E-86	2.04E-85	cyan	0.830455848	3.19E-41	GO:0016020,GO:0015171,GO:0003333,GO:0006811,GO:0005215	AT1G30450.1	ATCCC1,CCC1,HAP5	cation-chloride co-transporter 1	cation-chloride cotransporter
Sapur.003G020700.1	4489.390459	0.678113335	0.034543069	19.63095	8.41E-86	7.96E-85	cyan	0.837862722	1.35E-42	GO:0016746,GO:0008152	AT3G13930.1		Dihydrolipoamide acetyltransferase, long form protein	dihydrolipoamide acetyltransferase component of pyruvate dehydrogenase, putative
Sapur.15WG030400.1	1317.278898	1.976494953	0.1008989	19.58887	1.92E-85	1.81E-84	cyan	0.773905962	1.46E-32	GO:0051287,GO:0055114,GO:0004616	AT1G18270.3		ketose-bisphosphate aldolase class-II family protein	NA
Sapur.005G079300.1	817.8952603	0.673251683	0.034414529	19.56301	3.20E-85	3.00E-84	cyan	0.850592072	4.03E-45	GO:0005515,GO:0000042	AT5G66030.1	ATGRIP,GRIP	Golgi-localized GRIP domain-containing protein	GRIP protein
Sapur.15WG081100.1	814.6191317	0.787035493	0.040447029	19.45843	2.47E-84	2.29E-83	cyan	0.827325832	1.16E-40	GO:0048193,GO:0005783	AT3G47700.1	MAG2	RINT-1 / TIP-1 family	RINT-1/TIP-1 family protein
Sapur.016G149500.1	2291.131595	0.994024078	0.051124535	19.44319	3.33E-84	3.08E-83	cyan	0.808882157	1.40E-37		AT3G63460.1		transducin family protein / WD-40 repeat family protein	protein transporter SEC31
Sapur.016G286100.1	362.3555122	0.73461664	0.03795479	19.35504	1.85E-83	1.69E-82	cyan	0.855276051	4.14E-46	GO:0005634	AT4G20400.1	JMJ14,PKDM7B	JUMONJI 14	NA
Sapur.016G310000.1	554.755571	0.627365461	0.032565293	19.26485	1.06E-82	9.59E-82	cyan	0.829455858	4.82E-41	GO:0055085,GO:0016020,GO:0006821,GO:0005247	AT1G55620.2	ATCLC-F,CLC-F,CLCF	chloride channel F	chloride channel, ClC protein
Sapur.006G218200.1	4278.960073	0.78030375	0.040967759	19.04678	6.99E-81	6.16E-80	cyan	0.808551776	1.58E-37	GO:0016021	AT5G10840.1		Endomembrane protein 70 protein family	NA
Sapur.004G133900.1	3688.495148	0.824734639	0.043324085	19.0364	8.52E-81	7.50E-80	cyan	0.818546354	3.76E-39	GO:0046034,GO:0015992,GO:0033178,GO:0016820,GO:0015991,GO:0005524	AT4G38510.5		ATPase, V1 complex, subunit B protein	V-type proton ATPase subunit B
Sapur.002G133100.1	866.3299486	0.89075531	0.046938109	18.97723	2.63E-80	2.30E-79	cyan	0.826102347	1.90E-40	GO:0006468,GO:0004672	AT3G58640.2		Mitogen activated protein kinase kinase kinase-related	kinase, putative
Sapur.016G056700.1	1292.243162	0.893275956	0.047340895	18.86901	2.05E-79	1.77E-78	cyan	0.816984999	6.84E-39		AT3G58050.1			hypothetical protein
Sapur.010G147000.1	647.7275826	0.790578741	0.042505251	18.59955	3.24E-77	2.71E-76	cyan	0.819617211	2.48E-39	GO:0016192,GO:0016021	AT5G05570.2		transducin family protein / WD-40 repeat family protein	syntaxin-binding protein 5 (tomosyn)
Sapur.001G082800.1	1465.644531	0.800915703	0.043323326	18.48694	2.63E-76	2.17E-75	cyan	0.816456906	8.36E-39	GO:0016787,GO:0005524,GO:0003677	AT5G41370.1	ATXPB1,XPB1	homolog of xeroderma pigmentosum complementation group B 1	DNA repair helicase XPB1
Sapur.008G119800.1	1449.054141	0.685099198	0.037085095	18.47371	3.36E-76	2.77E-75	cyan	0.811037319	6.37E-38	GO:0031625,GO:0006511	AT1G26830.1	ATCUL3,ATCUL3A,CUL3,CU	cullin 3	cullin protein
Sapur.018G040000.1	834.4738045	0.90433077	0.049284022	18.34937	3.34E-75	2.71E-74	cyan	0.812053787	4.37E-38	GO:0006468,GO:0004672	AT5G11850.1		Protein kinase superfamily protein	MAP3K delta-1 kinase, putative
Sapur.018G013600.1	3649.868422	0.715193689	0.039018232	18.32973	4.79E-75	3.87E-74	cyan	0.800484511	2.77E-36	GO:0016021	AT5G10840.1		Endomembrane protein 70 protein family	endomembrane family protein
Sapur.002G044300.1	1438.964866	0.574316311	0.031386345	18.29829	8.54E-75	6.88E-74	cyan	0.818524813	3.79E-39	GO:0005515	AT5G08560.1		transducin family protein / WD-40 repeat family protein	transducin/WD40 domain protein
Sapur.003G023700.1	1995.695875	0.837652063	0.045790448	18.29316	9.38E-75	7.55E-74	cyan	0.804402483	7.01E-37		AT3G13870.1	RHD3	Root hair defective 3 GTP-binding protein (RHD3)	ROOT HAIR defective-like protein
Sapur.010G087800.1	998.5824056	0.704580624	0.03854399	18.27991	1.20E-74	9.61E-74	cyan	0.803989864	8.11E-37	GO:0055085,GO:0016021	AT1G71090.1		Auxin efflux carrier family protein	auxin efflux carrier family protein
Sapur.002G122600.1	2780.778229	1.62391511	0.089776091	18.0885	3.93E-73	3.09E-72	cyan	0.7233539	1.03E-26	GO:0005515	AT2G45540.1		WD-40 repeat family protein / beige-related	beige-related and WD-40 repeat protein
Sapur.013G105400.1	505.8320551	0.759355415	0.042291714	17.95518	4.37E-72	3.39E-71	cyan	0.794977529	1.81E-35		AT1G34550.1	EMB2756	Protein of unknown function (DUF616)	transmembrane protein, putative
Sapur.008G029500.1	650.3764269	0.761785634	0.042528661	17.91229	9.46E-72	7.31E-71	cyan	0.787217493	2.33E-34		AT5G20660.1		Zn-dependent exopeptidases superfamily protein	endoplasmic reticulum metallopeptidase
Sapur.15ZG052500.1	24.57199415	1.523201948	0.085223961	17.87293	1.92E-71	1.47E-70	cyan	0.771887023	2.67E-32	GO:0016798,GO:0016020	AT5G07830.1	AtGUS2,GUS2	glucuronidase 2	glycoside hydrolase family 79 amino-terminal domain protein
Sapur.002G053800.1	2100.580405	0.923675231	0.051751328	17.84834	2.98E-71	2.28E-70	cyan	0.797821464	6.92E-36	GO:0006893,GO:0000145	AT1G21170.1	SEC5B	Exocyst complex component SEC5	exocyst complex component, putative
Sapur.018G015700.1	589.8056381	0.850041222	0.047750376	17.80177	6.85E-71	5.23E-70	cyan	0.794582235	2.07E-35	GO:0020037,GO:0017004	AT5G54830.1		DOMON domain-containing protein / dopamine beta-monooxygenase N-terminal domain-containing protein	DOMON and dopamine beta-monooxygenase amino-terminal domain protein, putative

Sapur.002G056100.1	846.0342844	0.528377625	0.029892143	17.67614	6.40E-70	4.81E-69	cyan	0.809397246	1.16E-37		AT1G77220.1		Protein of unknown function (DUF300)	organic solute transporter ostalpha protein
Sapur.001G185700.1	1345.563287	0.787513828	0.04490885	17.53583	7.63E-69	5.63E-68	cyan	0.795403824	1.57E-35	GO:0005525,GO:0003924	AT1G59610.1	ADL3,CF1,DL3,DRP2B	dynamin-like 3	dynamin-2A protein
Sapur.018G079500.1	9.870341664	3.149187802	0.179879779	17.50718	1.26E-68	9.29E-68	cyan	0.728256245	3.19E-27		AT4G30710.1		Family of unknown function (DUF566)	DUF566 family protein
Sapur.016G202100.1	1006.105845	0.928970904	0.053346179	17.41401	6.46E-68	4.70E-67	cyan	0.764097464	2.59E-31	GO:0055085	AT3G27020.1	YSL6	YELLOW STRIPE like 6	metal-nicotianamine transporter YSL6, putative
Sapur.15WG126800.1	9171.92315	0.601483553	0.034844028	17.26217	9.06E-67	6.48E-66	cyan	0.8041737	7.60E-37	GO:0035194,GO:0003712	AT5G07350.2	AtTudor1,TSN1,Tudor1	TUDOR-SN protein 1	tudor protein
Sapur.014G096600.1	934.7885492	0.538810828	0.031657032	17.02026	5.81E-65	4.06E-64	cyan	0.827732358	9.79E-41		AT4G02030.1		Vps51/Vps67 family (components of vesicular transport) protein	Fat-free-like protein
Sapur.003G094300.1	841.3117989	0.842419145	0.050005054	16.84668	1.11E-63	7.58E-63	cyan	0.785835424	3.63E-34	GO:0030131,GO:0016192,GO:0006886,GO:003[illegible]	AT4G23460.1		Adaptin family protein	beta-adaptin-like protein
Sapur.002G036300.1	1022.981978	1.235252722	0.073570709	16.79001	2.89E-63	1.96E-62	cyan	0.738631174	2.45E-28	GO:0005515	AT1G03060.1	SPI	Beige/BEACH domain ;WD domain, G-beta repeat protein	WD repeat and FYVE domain protein
Sapur.004G058400.1	4228.427638	0.674147046	0.04018058	16.77793	3.54E-63	2.40E-62	cyan	0.782747817	9.66E-34	GO:0055114,GO:0051536,GO:0016651,GO:001[illegible]	AT5G37510.1	CI76,EMB1467	NADH-ubiquinone dehydrogenase, mitochondrial, putative	NADH-ubiquinone oxidoreductase, putative
Sapur.012G051900.1	9569.923471	0.919030045	0.055660363	16.51139	3.04E-61	2.00E-60	cyan	0.779413363	2.73E-33	GO:0046872,GO:0000166	AT5G62670.1	AHA11,HA11	H(+)-ATPase 11	plasma membrane H+-ATPase
Sapur.010G016300.1	2045.68554	0.492821293	0.030634466	16.08715	3.14E-58	1.97E-57	cyan	0.795438148	1.55E-35	GO:0031625,GO:0006511	AT4G02570.4	ATCUL1,AXR6,CUL1	cullin 1	CULLIN-like protein
Sapur.010G008800.1	1380.988597	0.739001454	0.045990817	16.06846	4.25E-58	2.66E-57	cyan	0.769078236	6.13E-32	GO:0008017,GO:0007018,GO:0005524,GO:000[illegible]	AT3G16630.1	ATKINESIN-13A.KINESIN-13	P-loop containing nucleoside triphosphate hydrolases superfamily protein	kinesin motor domain protein
Sapur.016G184600.1	2049.377597	0.64761464	0.040623744	15.94178	3.25E-57	2.01E-56	cyan	0.772051174	2.55E-32	GO:0030127,GO:0008270,GO:0006888,GO:000[illegible]	AT1G05520.1		Sec23/Sec24 protein transport family protein	protein transporter Sec23
Sapur.001G082900.1	2272.613075	0.626474814	0.039398813	15.90086	6.25E-57	3.84E-56	cyan	0.77844721	3.68E-33	GO:0030131,GO:0016192,GO:0006886,GO:003[illegible]	AT4G23460.1		Adaptin family protein	beta-adaptin-like protein
Sapur.017G065100.1	646.3354726	0.864633478	0.054607112	15.83372	1.82E-56	1.11E-55	cyan	0.750221224	1.20E-29	GO:0032012,GO:0005086	AT1G13980.2	EMB30,GN,VAN7	sec7 domain-containing protein	pattern formation protein, putative
Sapur.013G013500.1	267.5552274	1.344155523	0.085128954	15.78964	3.67E-56	2.23E-55	cyan	0.722095784	1.38E-26	GO:0016787	AT3G05580.1		Calcineurin-like metallo-phosphoesterase superfamily protein	serine/threonine-protein phosphatase PP1
Sapur.016G041800.1	11.29712279	3.142541356	0.200852088	15.64605	3.54E-55	2.11E-54	cyan	0.685495054	4.02E-23	GO:0009790	AT5G06760.1	LEA4-5	Late Embryogenesis Abundant 4-5	late embryogenesis abundant group 1 protein
Sapur.006G076200.1	1866.851177	0.583858684	0.03747083	15.58169	9.70E-55	5.76E-54	cyan	0.762033362	4.66E-31	GO:0030131,GO:0016192,GO:0006886,GO:0030117	AT1G60070.1		Adaptor protein complex AP-1, gamma subunit	AP-1 complex subunit gamma-2
Sapur.010G123700.1	1208.944465	0.86027687	0.055425276	15.52138	2.49E-54	1.47E-53	cyan	0.735231727	5.75E-28	GO:0016787	AT1G13900.1		Purple acid phosphatases superfamily protein	inactive purple acid phosphatase, putative
Sapur.016G280400.1	551.2644381	0.626082912	0.040402891	15.49599	3.69E-54	2.17E-53	cyan	0.760124261	7.98E-31		AT4G27020.1			plant/F10M23-360 protein
Sapur.014G006100.1	1279.931438	0.653319307	0.042289183	15.44885	7.68E-54	4.50E-53	cyan	0.781148091	1.59E-33	GO:0046872,GO:0000166	AT1G10130.1	ATECA3,ECA3	endoplasmic reticulum-type calcium-transporting ATPase 3	calcium-transporting ATPase sarcoplasmic/endoplasmic reticulum type protein, putative
Sapur.15WG089400.3	9.892775175	-1.632902087	0.106016988	-15.4023	1.58E-53	9.21E-53	cyan	0.311374777	7.20E-05		AT1G74910.2		ADP-glucose pyrophosphorylase family protein	mannose-1-phosphate guanyltransferase
Sapur.001G019100.1	1657.508199	0.464690127	0.030309879	15.33131	4.72E-53	2.73E-52	cyan	0.757770651	1.54E-30		AT3G08550.1	ABI8,ELD1,KOB1	elongation defective 1 protein / ELD1 protein	SDL protein
Sapur.017G114600.1	943.5897923	0.649931344	0.042494426	15.29451	8.32E-53	4.79E-52	cyan	0.752236753	6.98E-30	GO:0016020	AT1G54320.1		LEM3 (ligand-effect modulator 3) family protein / CDC50 family protein	NA
Sapur.010G137800.1	1093.104489	0.717644292	0.046966742	15.27984	1.04E-52	5.99E-52	cyan	0.754664148	3.61E-30		AT5G16300.1		Vps51/Vps67 family (components of vesicular transport) protein	Vps51/Vps67 family (component of vesicular transport) protein
Sapur.019G058000.1	464.450559	1.208838155	0.080490909	15.01832	5.57E-51	3.11E-50	cyan	0.736616301	4.07E-28		AT4G30390.1			NA
Sapur.012G060300.1	1657.518878	0.534297032	0.035644781	14.98949	8.60E-51	4.78E-50	cyan	0.773454438	1.67E-32	GO:0015937,GO:0005524,GO:0004594	AT4G32180.1	ATPANK2,PANK2	pantothenate kinase 2	pantothenate kinase
Sapur.006G126800.1	751.672412	0.738973384	0.049645856	14.8849	4.13E-50	2.27E-49	cyan	0.761050392	6.15E-31	GO:0005515,GO:0006468,GO:0005524,GO:0004672	AT4G29380.1		protein kinase family protein / WD-40 repeat family protein	phosphoinositide-3-kinase, regulatory subunit 4, p150
Sapur.014G070000.1	1154.790435	0.862508173	0.058065698	14.85401	6.55E-50	3.59E-49	cyan	0.748415341	1.94E-29	GO:0048278,GO:0006887,GO:0005737	AT5G12370.2	SEC10	exocyst complex component sec10	exocyst complex component Sec10
Sapur.011G106900.1	678.0868011	0.544117207	0.037226235	14.6165	2.20E-48	1.18E-47	cyan	0.761000037	6.24E-31		AT5G65950.1			NA
Sapur.017G125000.1	1963.144374	0.638605749	0.044512641	14.34662	1.12E-46	5.79E-46	cyan	0.736800403	3.88E-28	GO:0016021	AT2G01970.1		Endomembrane protein 70 protein family	transmembrane 9 family protein
Sapur.003G008800.1	4205.121866	0.481078733	0.03354557	14.34105	1.21E-46	6.27E-46	cyan	0.74320811	7.58E-29	GO:0016021	AT1G10950.1	AtTMN1,TMN1	transmembrane nine 1	NA
Sapur.007G023200.1	647.725222	0.709266017	0.049596248	14.3008	2.16E-46	1.12E-45	cyan	0.733864348	8.08E-28	GO:0016192,GO:0006886	AT4G36630.1	EMB2754	Vacuolar sorting protein 39	tubby-like F-box protein
Sapur.001G161400.1	1196.96787	0.842990089	0.059086508	14.26705	3.51E-46	1.81E-45	cyan	0.732068384	1.26E-27	GO:0016773.GO:0005975.GO:0005524.GO:0046835.GO:0005536.GO:0004396.GO:000167	AT2G19860.1	ATHXK2,HXK2	hexokinase 2	hexokinase
Sapur.005G158800.1	2372.652214	0.767284032	0.055001803	13.95016	3.14E-44	1.56E-43	cyan	0.730877027	1.69E-27	GO:0016773,GO:0016020,GO:0005524	AT2G42880.1	ATMPK20,MPK20	MAP kinase 20	MAP kinase
Sapur.012G068400.1	1278.97727	0.947168879	0.068449132	13.83756	1.51E-43	7.42E-43	cyan	0.718206032	3.43E-26		AT5G63190.2		MA3 domain-containing protein	NA
Sapur.001G185400.1	304.7223961	0.464203609	0.033672707	13.78575	3.11E-43	1.51E-42	cyan	0.732797399	1.05E-27	GO:0016020,GO:0008375	AT1G10280.1		Core-2/I-branching beta-1,6-N-acetylglucosaminyltransferase family protein	core-2/I-branching enzyme
Sapur.009G024900.1	2555.107721	0.686978898	0.050526446	13.59642	4.21E-42	2.01E-41	cyan	0.710181618	2.13E-25	GO:0016021,GO:0008270,GO:0005524,GO:0004222,GO:0006508	AT1G07510.1	ftsh10	FTSH protease 10	ATP-dependent zinc metalloprotease FtsH
Sapur.005G088300.1	2159.461637	0.364224571	0.027136793	13.4218	4.51E-41	2.12E-40	cyan	0.735608914	5.23E-28	GO:0042147,GO:0030904,GO:0015031,GO:0008565	AT2G17790.1	VPS35A,ZIP3	VPS35 homolog A	vacuolar protein sorting-associated protein
Sapur.003G032100.1	650.7147729	0.655932418	0.04893978	13.40285	5.82E-41	2.73E-40	cyan	0.721067104	1.76E-26	GO:0003824	AT5G17250.1		Alkaline-phosphatase-like family protein	phosphatidylinositol glycan protein, putative
Sapur.014G142600.1	1729.242254	0.703234235	0.052626691	13.36269	9.99E-41	4.67E-40	cyan	0.711206734	1.69E-25		AT4G12770.1		Chaperone DnaJ-domain superfamily protein	auxilin-related protein, putative
Sapur.010G098000.1	2424.335992	0.502645316	0.037699892	13.33281	1.49E-40	6.95E-40	cyan	0.724623134	7.62E-27		AT3G25800.1	PDF1,PP2AA2,PR 65	protein phosphatase 2A subunit A2	serine/threonine protein phosphatase 2A regulatory subunit A, putative
Sapur.003G058400.1	1575.692003	0.415598888	0.031376511	13.24554	4.79E-40	2.21E-39	cyan	0.723401351	1.02E-26		AT5G46630.1		Clathrin adaptor complexes medium subunit family protein	AP-2 complex subunit mu
Sapur.012G054700.1	1093.236908	0.57563926	0.043645359	13.18901	1.02E-39	4.66E-39	cyan	0.72935174	2.44E-27	GO:0016773,GO:0048015,GO:0046854	AT1G60490.1	ATVPS34,PI3K,VPS34	vacuolar protein sorting 34	phosphatidylinositol 3-kinase
Sapur.15WG091300.1	1270.675934	0.686653264	0.052078589	13.18494	1.07E-39	4.92E-39	cyan	0.725471813	6.22E-27	GO:0003682,GO:0006351,GO:0005634,GO:0003677	AT3G48050.1		BAH domain ;TFIIS helical bundle-like domain	BAH and TFIIS domain protein
Sapur.007G016600.1	157.7623922	0.791869555	0.060112421	13.17314	1.25E-39	5.74E-39	cyan	0.700780965	1.67E-24		AT4G36440.1			transmembrane protein, putative
Sapur.005G081700.1	1347.76804	0.624322389	0.047724774	13.08173	4.19E-39	1.90E-38	cyan	0.72993963	2.12E-27	GO:0005509	AT1G21630.1		Calcium-binding EF hand family protein	calcium-binding EF-hand protein
Sapur.001G169200.1	870.7873123	0.477899199	0.036720072	13.01466	1.01E-38	4.55E-38	cyan	0.721173748	1.72E-26	GO:0071805,GO:0016020,GO:0015079	AT5G09400.1	KUP7	K+ uptake permease 7	NA
Sapur.005G186900.1	685.1374273	0.810414117	0.062381191	12.99132	1.37E-38	6.14E-38	cyan	0.698707255	2.60E-24	GO:0033179,GO:0015991,GO:0015078	AT2G21410.1	VHA-A2	vacuolar proton ATPase A2	vacuolar proton ATPase a3
Sapur.008G119300.1	3133.68857	0.444642095	0.034292496	12.96616	1.90E-38	8.50E-38	cyan	0.712966984	1.14E-25	GO:0008168	AT1G26850.2		S-adenosyl-L-methionine-dependent methyltransferases superfamily protein	methyltransferase PMT2, putative
Sapur.004G115300.1	4508.827409	0.513985693	0.039890089	12.88505	5.46E-38	2.42E-37	cyan	0.713235845	1.07E-25	GO:0030126,GO:0016192,GO:0006886,GO:0005198,GO:0030117	AT4G34450.1		coatomer gamma-2 subunit, putative / gamma-2 coat protein, putative / gamma-2 COP, putative	coatomer subunit gamma
Sapur.012G029200.1	1797.902839	0.456505371	0.035461338	12.87333	6.36E-38	2.82E-37	cyan	0.706688165	4.61E-25	GO:0030170,GO:0009058	AT4G36480.2	ATLCB1.EMB2779.FBR11.LC	long-chain base1	long chain base biosynthesis protein 1a
Sapur.006G184800.1	3868.460554	0.760176691	0.06049553	12.56583	3.25E-36	1.39E-35	cyan	0.695044637	5.64E-24	GO:0008270,GO:0008237,GO:0006508	AT4G33090.1	APM1,ATAPM1	aminopeptidase M1	puromycin-sensitive aminopeptidase
Sapur.016G081000.1	68.64061111	1.077467986	0.085750929	12.56509	3.29E-36	1.41E-35	cyan	0.706567229	4.74E-25	GO:0006468,GO:0004672	AT2G37050.3		Leucine-rich repeat protein kinase family protein	NA
Sapur.006G194200.1	2453.983465	0.829346282	0.066157071	12.53602	4.74E-36	2.02E-35	cyan	0.691619403	1.15E-23		AT5G11700.2			transmembrane protein, putative
Sapur.002G000300.1	616.0368461	0.493433505	0.039390205	12.52681	5.33E-36	2.27E-35	cyan	0.689222068	1.89E-23		AT5G25070.1			hypothetical protein
Sapur.006G024500.1	650.1561045	0.561863308	0.044885332	12.51775	5.97E-36	2.54E-35	cyan	0.695496366	5.13E-24	GO:0043565,GO:0006355,GO:0003700	AT2G40950.1	BZIP17	Basic-leucine zipper (bZIP) transcription factor family protein	BZIP transcription factor, putative
Sapur.014G009200.1	104.4706273	1.013912308	0.081124651	12.4982	7.64E-36	3.25E-35	cyan	0.640338374	1.72E-19		AT2G26680.1			FkbM family methyltransferase
Sapur.003G000800.1	1064.902882	0.687464148	0.055110802	12.47422	1.03E-35	4.38E-35	cyan	0.703112306	1.01E-24	GO:0005525,GO:0003924	AT1G59610.1	ADL3,CF1,DL3,DRP2B	dynamin-like 3	dynamin-2A protein
Sapur.008G149300.1	844.7596374	0.728399325	0.058487317	12.45397	1.33E-35	5.63E-35	cyan	0.675161136	3.11E-22		AT3G26400.1	EIF4B1	eukaryotic translation initiation factor 4B1	eukaryotic translation initiation factor 4B
Sapur.014G141600.1	4937.029482	0.66718029	0.053575301	12.45313	1.34E-35	5.69E-35	cyan	0.691216925	1.25E-23	GO:0016021	AT4G12650.1		Endomembrane protein 70 protein family	transmembrane 9 family protein
Sapur.002G138300.1	1303.040751	0.589276477	0.04734417	12.44665	1.46E-35	6.16E-35	cyan	0.688159646	2.34E-23	GO:0048193,GO:0005783	AT4G01400.2			oligomeric subunit 4
Sapur.009G057100.1	7727.631474	0.793371925	0.064183582	12.36098	4.25E-35	1.78E-34	cyan	0.679963199	1.21E-22	GO:0030132,GO:0030130,GO:0016192,GO:0006886,GO:0005198	AT3G11130.1		Clathrin, heavy chain	clathrin heavy chain
Sapur.005G059400.1	1937.284002	0.671821058	0.054375635	12.35519	4.57E-35	1.91E-34	cyan	0.689705133	1.71E-23	GO:0016887,GO:0005524,GO:0055085,GO:0042626,GO:0016021,GO:0006810	AT4G39850.1	ACN2.AtABCD1.CTS.PED3.P:	peroxisomal ABC transporter 1	ABC transporter D family protein
Sapur.012G043900.1	1526.839914	0.499717642	0.040710709	12.27484	1.24E-34	5.12E-34	cyan	0.686866973	3.05E-23	GO:0030173,GO:0006891	AT3G18480.1	AtCASP,CASP	CCAAT-displacement protein alternatively spliced product	CASP carboxy-terminal protein
Sapur.008G101800.1	14464.32901	0.44841455	0.036976241	12.1271	7.59E-34	3.10E-33	cyan	0.697418053	3.42E-24	GO:0016887.GO:0006754.GO:0005524.GO:0000275.GO:0046034.GO:0015992.GO:003317	AT5G08680.1		ATP synthase alpha/beta family protein	ATP synthase F1, beta subunit
Sapur.010G083300.1	810.4846035	0.650480118	0.053675557	12.11874	8.40E-34	3.43E-33	cyan	0.685127022	4.33E-23		AT1G23170.2		Protein of unknown function DUF2359, transmembrane	DUF2359 family protein
Sapur.15WG079700.1	288.5419139	0.95095091	0.078587053	12.10061	1.05E-33	4.27E-33	cyan	0.66707397	1.45E-21					NA
Sapur.017G006900.1	1700.977954	0.690216089	0.057618691	11.97903	4.58E-33	1.84E-32	cyan	0.699122093	2.38E-24	GO:0046872.GO:0000166.GO:0016021.GO:0015914.GO:0005524.GO:0004012.GO:000028	AT5G44240.2	ALA2	aminophospholipid ATPase 2	phospholipid-translocating ATPase
Sapur.006G031900.1	1055.77932	0.478977467	0.040047435	11.96025	5.74E-33	2.31E-32	cyan	0.680042178	1.20E-22	GO:0008168	AT3G10200.1		S-adenosyl-L-methionine-dependent methyltransferases superfamily protein	methyltransferase PMT7, putative
Sapur.005G085700.1	7953.827064	0.510887138	0.04271761	11.95964	5.78E-33	2.32E-32	cyan	0.688060948	2.39E-23	GO:0008152	AT4G35830.1	ACO1	aconitase 1	aconitate hydratase
Sapur.001G008000.1	2755.858515	0.399371122	0.034223192	11.6696	1.82E-31	7.08E-31	cyan	0.694274802	6.62E-24	GO:0006468,GO:0005524,GO:0004672	AT3G13670.1		Protein kinase family protein	casein kinase, putative
Sapur.T046600.1	405.6654717	0.484328488	0.041536997	11.66017	2.04E-31	7.90E-31	cyan	0.662482736	3.41E-21	GO:0030127,GO:0008270,GO:0006888,GO:0006886	AT2G21630.1		Sec23/Sec24 protein transport family protein	protein transporter Sec23
Sapur.004G171000.1	1756.920163	0.628101962	0.054127897	11.60403	3.93E-31	1.52E-30	cyan	0.673642697	4.16E-22		AT3G52850.1	ATELP.ATELP1.ATVSR1.BP-8	vacuolar sorting receptor homolog 1	vacuolar sorting receptor protein, putative
Sapur.008G083400.1	72.91766436	1.114897389	0.096480505	11.55568	6.91E-31	2.65E-30	cyan	0.645854587	6.67E-20	GO:0008152,GO:0003824,GO:0046912	AT3G06650.1	ACLB-1	ATP-citrate lyase B-1	ATP:citrate lyase
Sapur.006G215100.1	2914.732315	0.563936883	0.048855575	11.54294	8.01E-31	3.07E-30	cyan	0.656001626	1.11E-20	GO:0030170,GO:0009058,GO:0008483,GO:0006520	AT4G31990.1	AAT3,ASP5,ATAAT1	aspartate aminotransferase 5	aspartate aminotransferase
Sapur.014G089400.1	1067.476446	0.504923187	0.044204842	11.42235	3.23E-30	1.22E-29	cyan	0.694026295	6.98E-24	GO:0030127,GO:0008270,GO:0006888,GO:0006886	AT4G01810.1		Sec23/Sec24 protein transport family protein	protein transporter Sec23
Sapur.001G032100.1	70.38053762	0.776060688	0.068001648	11.41238	3.63E-30	1.37E-29	cyan	0.665319896	2.02E-21		AT5G57820.1		zinc ion binding	hypothetical protein
Sapur.004G153700.1	3.383356674	2.448602218	0.216379942	11.31622	1.09E-29	4.07E-29	cyan	0.627069493	1.55E-18	GO:0009626	AT4G38360.2	LAZ1	Protein of unknown function (DUF300)	organic solute transporter ostalpha protein
Sapur.006G223800.1	3852.93188	0.551781244	0.04891158	11.2812	1.63E-29	6.05E-29	cyan	0.65242415	2.11E-20	GO:0030126,GO:0016192,GO:0006886,GO:0005198,GO:0030117,GO:0005737	AT4G31490.1		Coatomer, beta subunit	coatomer subunit beta
Sapur.018G028200.1	680.8502204	0.513362707	0.045663983	11.24218	2.53E-29	9.39E-29	cyan	0.673435425	4.33E-22	GO:0008601,GO:0007165,GO:0000159	AT5G25510.1		Protein phosphatase 2A regulatory B subunit family protein	serine/threonine protein phosphatase 2A 57 kDa regulatory subunit B' protein iota
Sapur.005G170500.1	1652.181856	0.801532385	0.071325852	11.23761	2.66E-29	9.88E-29	cyan	0.641669009	1.37E-19	GO:0005515	AT1G03060.1	SPI	Beige/BEACH domain ;WD domain, G-beta repeat protein	WD repeat and FYVE domain protein
Sapur.013G093400.1	1243.735883	0.477010949	0.043061054	11.07755	1.61E-28	5.88E-28	cyan	0.679603822	1.30E-22	GO:0006468,GO:0005524,GO:0004672	AT1G22870.1		Protein kinase family protein with ARM repeat domain	kinase domain protein
Sapur.007G038000.1	2717.812399	0.604372913	0.054884947	11.01163	3.36E-28	1.22E-27	cyan	0.671320391	6.50E-22		AT3G50590.1		Transducin/WD40 repeat-like superfamily protein	vascular protein
Sapur.010G167600.1	935.7947333	0.463394625	0.042182703	10.98542	4.49E-28	1.62E-27	cyan	0.668353589	1.14E-21	GO:0030127,GO:0008270,GO:0006888,GO:0006886	AT3G07100.1	ERMO2,SEC24A	Sec23/Sec24 protein transport family protein	protein transporter Sec24-like protein
Sapur.014G052000.1	3155.638312	0.658154234	0.060565538	10.86681	1.66E-27	5.92E-27	cyan	0.641662193	1.37E-19	GO:0032012,GO:0005086	AT3G60860.1		SEC7-like guanine nucleotide exchange family protein	brefeldin A-inhibited guanine nucleotide-exchange protein
Sapur.016G289100.1	1155.660794	0.311817268	0.028758155	10.84274	2.16E-27	7.69E-27	cyan	0.676320027	2.48E-22	GO:0003676	AT1G32790.1	CID11	CTC-interacting domain 11	RNA recognition motif 1 in plant thaliana CTC-interacting domain protein CID8
Sapur.005G138700.1	533.7373483	0.597143122	0.055608498	10.73834	6.72E-27	2.37E-26	cyan	0.660653476	4.78E-21	GO:0055085	AT1G77610.1		EamA-like transporter family protein	UAA transporter family protein
Sapur.006G103200.1	6621.202468	0.450580536	0.04224923	10.66482	1.49E-26	5.18E-26	cyan	0.66149159	4.10E-21	GO:0005524,GO:0009378,GO:0006310,GO:0006281	AT5G03340.1		ATPase, AAA-type, CDC48 protein	cell division control protein
Sapur.016G248500.1	7714.887273	0.362528675	0.034005979	10.66073	1.55E-26	5.41E-26	cyan	0.657262656	8.85E-21	GO:0016020,GO:0015031	AT2G34250.2		SecY protein transport family protein	preprotein translocase, SecY subunit
Sapur.004G167900.1	974.1192946	0.706618925	0.066441213	10.63525	2.04E-26	7.10E-26	cyan	0.626861728	1.60E-18		AT3G51650.1			transmembrane protein, putative
Sapur.009G088400.1	1830.5127	0.334356676	0.032055097	10.43069	1.80E-25	6.11E-25	cyan	0.651230283	2.60E-20	GO:0005515	AT3G18860.1		transducin family protein / WD-40 repeat family protein	phospholipase A-2-activating protein
Sapur.002G142900.1	1499.989479	0.539258839	0.052388334	10.29349	7.54E-25	2.53E-24	cyan	0.651997664	2.27E-20	GO:0005515	AT2G46560.1		transducin family protein / WD-40 repeat family protein	transducin family protein
Sapur.T049200.2	18.67391574	-0.891795951	0.086695486	-10.2865	8.10E-25	2.71E-24	cyan	-0.46069469	1.27E-09		AT4G36660.1		Protein of unknown function (DUF1195)	sugar transporter, putative
Sapur.009G038000.1	530.3844448	0.382454413	0.037277916	10.25954	1.07E-24	3.58E-24	cyan	0.668179441	1.18E-21	GO:0005515	AT5G12430.1		Heat shock protein DnaJ with tetratricopeptide repeat	DnaJ heat shock amino-terminal domain protein, putative
Sapur.005G135400.1	25.80731097	0.938744201	0.091523291	10.25689	1.10E-24	3.68E-24	cyan	0.607373247	3.35E-17		AT1G38131.1		O-fucosyltransferase family protein	growth regulator protein, putative
Sapur.014G116000.1	367.835235	0.490831087	0.048390462	10.14314	3.55E-24	1.17E-23	cyan	0.640244567	1.75E-19	GO:0016192,GO:0006904	AT2G42700.1			Sec1 family containing protein
Sapur.006G064400.1	1787.497011	0.583195019	0.057701252	10.10715	5.14E-24	1.68E-23	cyan	0.590285113	4.09E-16	GO:0003824	AT2G07050.1	CAS1	cycloartenol synthase 1	cycloartenol synthase
Sapur.017G065200.1	1217.926585	0.577948894	0.057289519	10.08821	6.23E-24	2.04E-23	cyan	0.625675216	1.94E-18	GO:0032012,GO:0005086	AT1G13980.1	EMB30,GN,VAN7	sec7 domain-containing protein	ARF guanine nucleotide exchange factor
Sapur.014G068000.1	713.5311707	0.495630047	0.049313917	10.05051	9.14E-24	2.97E-23	cyan	0.648083861	4.53E-20		AT5G28350.1		Quinoprotein amine dehydrogenase, beta chain-like; RIC1-like guanyl-nucleotide exchange factor	quinoprotein amine dehydrogenase, beta chain-like/RIC1-like guanyl-nucleotide exchange factor
Sapur.001G156700.1	8794.296406	0.652690041	0.066551968	9.807224	1.05E-22	3.33E-22	cyan	0.601635394	7.90E-17	GO:0005515,GO:0003824	AT3G15730.1	PLD,PLDALPHA1	phospholipase D alpha 1	phospholipase D alpha
Sapur.014G056900.1	37.3268907	-0.733302104	0.074898599	-9.7906	1.24E-22	3.92E-22	cyan	0.410553861	9.27E-08		AT2G45690.1	ATPEX16,PEX16,SSE,SSE1	shrunken seed protein (SSE1)	shrunken seed protein, putative
Sapur.013G068500.1	33.97646711	1.267774207	0.130078524	9.746222	1.91E-22	6.04E-22	cyan	0.597844624	1.38E-16					NA
Sapur.008G079400.1	2898.260512	0.761500333	0.078233715	9.733659	2.17E-22	6.82E-22	cyan	0.597159172	1.52E-16	GO:0005515	AT1G48090.1		calcium-dependent lipid-binding family protein	vacuolar protein sorting-associated protein, putative
Sapur.014G118000.1	885.6501092	0.386062382	0.039779098	9.705157	2.87E-22	9.00E-22	cyan	0.639394935	2.02E-19		AT3G26730.1		RING/U-box superfamily protein	zinc finger, C3HC4 type (RING finger) protein, putative
Sapur.007G059300.1	142.3712366	0.578214993	0.059678776	9.688788	3.36E-22	1.05E-21	cyan	0.602841268	6.61E-17	GO:0055114,GO:0016616,GO:0051287,GO:0000287	AT4G35260.1	IDH-I,IDH1	isocitrate dehydrogenase 1	NA
Sapur.012G080800.1	8713.902074	0.367128386	0.038047557	9.649197	4.95E-22	1.55E-21	cyan	0.631084517	8.05E-19	GO:0005515,GO:0016887,GO:0005524,GO:0019538	AT5G50920.1	ATHSP93-V.CLPC.CLPC1.DC.	CLPC homologue 1	ATP-dependent Clp protease ATP-binding subunit clpA-like protein
Sapur.001G039000.1	2587.61411	0.66140317	0.068565012	9.646366	5.09E-22	1.59E-21	cyan	0.574319247	3.72E-15	GO:0016887,GO:0005524,GO:0016020	AT1G15520.1	ABCG40.ATABCG40.ATPDR1	pleiotropic drug resistance 12	pleiotropic drug resistance, ABC transporter family protein, putative
Sapur.014G054100.1	677.1275536	0.364608101	0.038110405	9.567154	1.10E-21	3.41E-21	cyan	0.646689872	5.77E-20	GO:0005515	AT2G45540.2		WD-40 repeat family protein / beige-related	NA
Sapur.006G201300.1	1195.170742	0.364482052	0.038219724	9.536491	1.48E-21	4.56E-21	cyan	0.624774713	2.24E-18	GO:0030131,GO:0016192,GO:0006886	AT5G11490.1		adaptin family protein	beta adaptin-like protein
Sapur.003G025000.1	1.172423452	3.342758562	0.352198855	9.491111	2.29E-21	7.02E-21	cyan	0.520802007	2.71E-12	GO:0055085,GO:0042626,GO:0016021,GO:0006810,GO:0005524,GO:0016887	AT3G21250.2	ATMRP6,MRP6	multidrug resistance-associated protein 6	multidrug resistance protein ABC transporter family protein
Sapur.T001500.1	40.47310979	0.966043192	0.10354771	9.32945	1.06E-20	3.21E-20	cyan	0.586528152	6.95E-16	GO:0016020,GO:0015171,GO:0003333,GO:0006811,GO:0005215	AT1G30450.2	ATCCC1,CCC1,HAP5	cation-chloride co-transporter 1	cation-chloride cotransporter
Sapur.006G154600.1	1934.671544	0.583095469	0.062527916	9.325362	1.11E-20	3.34E-20	cyan	0.560268828	2.35E-14	GO:0016021	AT5G35160.2		Endomembrane protein 70 protein family	transmembrane 9 family protein
Sapur.005G204400.1	46.46081632	0.936379714	0.101215993	9.251302	2.22E-20	6.64E-20	cyan	0.59006875	4.22E-16	GO:0055085,GO:0016021,GO:0022857	AT4G35300.4	TMT2	tonoplast monosaccharide transporter2	hexose transporter
Sapur.001G122100.2	14.47826341	1.158249732	0.125636971	9.21902	3.00E-20	8.94E-20	cyan	0.608583158	2.79E-17		AT2G38740.1		Haloacid dehalogenase-like hydrolase (HAD) superfamily protein	haloacid dehalogenase-like hydrolase
Sapur.009G018400.1	4432.673457	0.53838234	0.058784561	9.158567	5.26E-20	1.56E-19	cyan	0.593879699	2.45E-16	GO:0046872,GO:0000166	AT1G07670.1	ATECA4,ECA4	endomembrane-type CA-ATPase 4	endoplasmic reticulum-type calcium-transporting ATPase
Sapur.016G199300.1	25.52231314	0.954417036	0.104738309	9.112397	8.06E-20	2.37E-19	cyan	0.60272045	6.72E-17					NA
Sapur.010G030000.1	1021.256188	0.47652055	0.053064597	8.980009	2.71E-19	7.88E-19	cyan	0.592946464	2.80E-16	GO:0046872.GO:0000166.GO:0016021.GO:0015914.GO:0005524.GO:0004012.GO:000028	AT1G59820.1	ALA3	aminophospholipid ATPase 3	phospholipid-translocating ATPase
Sapur.010G147200.1	97.18133035	-0.774301903	0.087787129	-8.82022	1.14E-18	3.27E-18	cyan	-0.541024687	2.57E-13	GO:0046872	AT2G40140.2	ATSZF2,CZF1,SZF2,ZFAR1	zinc finger (CCCH-type) family protein	zinc finger CCCH domain protein
Sapur.001G187800.1	2443.415398	0.492739805	0.056695466	8.690991	3.59E-18	1.02E-17	cyan	0.562307935	1.81E-14	GO:0030170,GO:0009058	AT5G49810.1	MMT	methionine S-methyltransferase	methionine S-methyltransferase
Sapur.005G050300.1	28.8587242	-0.687715333	0.079277602	-8.67477	4.14E-18	1.17E-17	cyan	-0.531850723	7.63E-13					NA
Sapur.017G038800.1	1123.758995	0.413492101	0.048226797	8.573908	1.00E-17	2.79E-17	cyan	0.563285685	1.60E-14	GO:0055085,GO:0016020,GO:0006814,GO:0005215	AT5G64290.1	DCT,DIT2.1	dicarboxylate transport 2.1	glutamate/malate translocator
Sapur.018G088300.1	1686.776686	0.385064623	0.044937775	8.56884	1.05E-17	2.92E-17	cyan	0.538238578	3.59E-13	GO:0016021	AT5G35160.2		Endomembrane protein 70 protein family	transmembrane 9 family protein
Sapur.002G133200.1	68.64159657	0.926622651	0.108404332	8.547838	1.25E-17	3.49E-17	cyan	0.539254469	3.18E-13					NA
Sapur.006G089300.1	1726.117209	0.330583934	0.038870249	8.504806	1.82E-17	5.04E-17	cyan	0.556021578	4.04E-14		AT3G52850.1	ATELP.ATELP1.ATVSR1.BP-8	vacuolar sorting receptor homolog 1	vacuolar sorting receptor protein, putative
Sapur.017G016700.2	53.15261745	0.734413988	0.086416305	8.498558	1.92E-17	5.31E-17	cyan	0.545453146	1.50E-13		AT1G06050.1		Protein of unknown function (DUF1336)	lipid-binding protein
Sapur.013G117800.1	1175.718255	0.420554343	0.049581076	8.482154	2.21E-17	6.11E-17	cyan	0.570131212	6.50E-15	GO:0005975,GO:0004553	AT2G20680.1		Glycosyl hydrolase superfamily protein	glycoside hydrolase family 5 protein
Sapur.006G177900.1	1902.903402	0.937795656	0.110796672	8.464114	2.58E-17	7.11E-17	cyan	0.543191718	1.98E-13	GO:0032012,GO:0005086	AT3G43300.1	ATMIN7,BEN1	HOPM interactor 7	NA
Sapur.002G109700.1	502.8388723	0.649389499	0.076862311	8.448738	2.94E-17	8.10E-17	cyan	0.541382721	2.46E-13	GO:0055114,GO:0004471,GO:0051287	AT2G13560.1	NAD-ME1	NAD-dependent malic enzyme 1	NAD-dependent malic enzyme
Sapur.001G192800.1	1435.512611	0.356947633	0.042342663	8.429976	3.46E-17	9.49E-17	cyan	0.581566932	1.39E-15	GO:0030117,GO:0016192,GO:0006886	AT1G31730.1		Adaptin family protein	AP-4 complex subunit epsilon-1
Sapur.016G236200.1	728.0685535	0.377258675	0.044894316	8.403262	4.34E-17	1.19E-16	cyan	0.538306006	3.56E-13		AT5G55960.1			transmembrane protein, putative
Sapur.007G075200.1	41.51255306	0.722195792	0.086011922	8.396462	4.60E-17	1.26E-16	cyan	0.548959729	9.78E-14	GO:0007264,GO:0005525	AT5G64813.1	LIP1	Ras-related small GTP-binding family protein	Ras-related small GTP-binding family protein
Sapur.008G116000.1	2670.366832	0.267212813	0.031975371	8.356832	6.44E-17	1.75E-16	cyan	0.533709669	6.14E-13	GO:0005524	AT5G19990.1	ATSUG1,RPT6A	regulatory particle triple-A ATPase 6A	26S proteasome ATPase regulatory subunit 6
Sapur.008G164300.1	3318.659643	0.296556233	0.035677834	8.312058	9.41E-17	2.55E-16	cyan	0.572126617	4.99E-15	GO:0031625,GO:0006511	AT4G02570.3	ATCUL1,AXR6,CUL1	cullin 1	CULLIN-like protein
Sapur.009G129500.1	707.7002825	0.392107489	0.047443389	8.264745	1.40E-16	3.77E-16	cyan	0.553974613	5.23E-14	GO:0016192,GO:0006904	AT2G17980.1	ATSLY1	Sec1/munc18-like (SM) proteins superfamily	Sec1 family transporter SLY1
Sapur.15ZG037700.2	43.43472407	0.712631983	0.086248462	8.262547	1.43E-16	3.84E-16	cyan	0.543913584	1.81E-13		AT5G62370.1		Tetratricopeptide repeat (TPR)-like superfamily protein	NA
Sapur.012G037800.1	223.2228112	0.258858176	0.031339714	8.259749	1.46E-16	3.93E-16	cyan	0.582388072	1.24E-15	GO:0030117,GO:0016192,GO:0006886	AT1G48760.2	delta-ADR	delta-adaptin	delta-adaptin
Sapur.T142000.1	637.4803125	0.366162134	0.044421267	8.242947	1.68E-16	4.52E-16	cyan	0.537320756	4.01E-13		AT1G16560.2		Per1-like family protein	NA
Sapur.001G163500.1	16.72264511	0.928009112	0.112735431	8.231743	1.85E-16	4.95E-16	cyan	0.505098528	1.52E-11	GO:0016021	AT1G52155.1			transmembrane protein, putative
Sapur.017G077700.1	837.7553506	0.311338701	0.037946032	8.204776	2.31E-16	6.18E-16	cyan	0.534992087	5.28E-13	GO:0006887,GO:0000145	AT3G29400.1	ATEXO70E1,EXO70E1	exocyst subunit exo70 family protein E1	Exo70 exocyst complex subunit
Sapur.011G073900.1	2677.403219	0.443031335	0.054020414	8.201184	2.38E-16	6.37E-16	cyan	0.549790286	8.82E-14	GO:0015977,GO:0008964,GO:0006099	AT3G14940.1	ATPPC3,PPC3	phosphoenolpyruvate carboxylase 3	phosphoenolpyruvate carboxylase
Sapur.016G103000.1	404.2448465	0.31931491	0.039193403	8.14716	3.73E-16	9.91E-16	cyan	0.61359055	1.30E-17		AT5G05570.2		transducin family protein / WD-40 repeat family protein	syntaxin-binding protein 5 (tomosyn), putative
Sapur.016G086500.1	6846.132447	0.339609476	0.041764949	8.131447	4.24E-16	1.13E-15	cyan	0.553943998	5.25E-14	GO:0005524,GO:0009378,GO:0006310,GO:0006281	AT5G03340.1		ATPase, AAA-type, CDC48 protein	cell division control protein
Sapur.016G163500.1	5486.173308	0.507594074	0.062650568	8.101987	5.41E-16	1.43E-15	cyan	0.54579567	1.44E-13	GO:0030132,GO:0030130,GO:0016192,GO:0006886,GO:0005198	AT3G11130.1		Clathrin, heavy chain	clathrin heavy chain
Sapur.001G005300.2	10.80917218	-0.845699575	0.104762388	-8.07255	6.88E-16	1.82E-15	cyan	-0.438207033	9.48E-09	GO:0003676	AT1G55310.3	ATSCL33,SCL33,SR33	SC35-like splicing factor 33	serine/arginine-rich splicing factor 10 (SRSF10)-like RNA recognition motif protein
Sapur.005G074600.1	2919.448195	0.308222405	0.038313468	8.044753	8.64E-16	2.28E-15	cyan	0.541656552	2.38E-13	GO:0003676.GO:0043039.GO:0016876.GO:0005524.GO:0006419.GO:0004813.GO:000016	AT1G50200.1	ACD,ALATS	Alanyl-tRNA synthetase	alanyl-tRNA synthetase
Sapur.016G142500.1	675.0530785	0.474793966	0.05908805	8.035364	9.33E-16	2.46E-15	cyan	0.537007339	4.16E-13		AT5G58620.1		zinc finger (CCCH-type) family protein	transcription factor, putative
Sapur.005G062600.1	865.396977	0.257855793	0.032198911	8.008215	1.16E-15	3.05E-15	cyan	0.544336349	1.72E-13		AT4G39690.1			inner membrane protein
Sapur.002G149900.1	1134.515126	0.358885657	0.044884113	7.995828	1.29E-15	3.37E-15	cyan	0.550942188	7.65E-14	GO:0048278,GO:0006887,GO:0005737	AT5G12370.1	SEC10	exocyst complex component sec10	exocyst complex component Sec10
Sapur.004G101900.1	1097.614973	0.270313332	0.033885376	7.977286	1.50E-15	3.91E-15	cyan	0.55294793	5.95E-14	GO:0031625,GO:0006511	AT5G46210.1	ATCUL4,CUL4	cullin4	ubiquitin-protein ligase, cullin 4
Sapur.009G097000.1	1230.433957	0.377867457	0.047553142	7.946214	1.92E-15	5.02E-15	cyan	0.536242446	4.55E-13	GO:0008017,GO:0007018,GO:0005524,GO:0003777,GO:0005871	AT4G39050.1		Kinesin motor family protein	kinesin motor domain protein
Sapur.003G023900.1	2942.14244	0.377001112	0.047801713	7.88677	3.10E-15	8.04E-15	cyan	0.520156539	2.91E-12	GO:0016021	AT3G13772.1	AtTMN7,TMN7	transmembrane nine 7	multispanning membrane protein, putative
Sapur.002G163000.1	1834.609478	-0.512786874	0.065137621	-7.87236	3.48E-15	9.01E-15	cyan	-0.560123578	2.40E-14		AT3G62410.1	CP12,CP12-2	CP12 domain-containing protein 2	calvin cycle protein CP12
Sapur.016G210800.1	687.0492079	0.268421273	0.03410664	7.870059	3.54E-15	9.18E-15	cyan	0.555510684	4.31E-14		AT3G01720.1			peptidyl serine alpha-galactosyltransferase
Sapur.012G011200.1	1734.791329	0.334294484	0.042687374	7.831226	4.83E-15	1.25E-14	cyan	0.541147394	2.53E-13	GO:0008536,GO:0006886	AT5G53480.1		ARM repeat superfamily protein	importin subunit beta-1
Sapur.011G002900.1	14.44768232	-0.831972742	0.106398186	-7.81943	5.31E-15	1.37E-14	cyan	-0.487642559	9.33E-11					NA
Sapur.006G209900.1	76.90091383	0.842552061	0.107849368	7.812304	5.62E-15	1.44E-14	cyan	0.540233584	2.83E-13	GO:0005515,GO:0003824	AT3G15730.1	PLD,PLDALPHA1	phospholipase D alpha 1	phospholipase D alpha
Sapur.004G106300.2	3.574790603	1.208591373	0.154772985	7.808801	5.77E-15	1.48E-14	cyan	0.652306508	2.15E-20	GO:0016757,GO:0047262	AT5G15470.1	GAUT14	galacturonosyltransferase 14	CAZy family GT8 glycosyltransferase
Sapur.15WG112500.1	157.9337675	0.568678149	0.073049754	7.784806	6.98E-15	1.79E-14	cyan	0.522784412	2.17E-12		AT5G50390.1		Pentatricopeptide repeat (PPR-like) superfamily protein	NA
Sapur.15ZG037700.1	85.29170906	0.529527603	0.068394836	7.742216	9.77E-15	2.50E-14	cyan	0.564936389	1.29E-14		AT5G62370.1		Tetratricopeptide repeat (TPR)-like superfamily protein	NA
Sapur.014G069100.1	2032.522371	0.190086434	0.024618568	7.721263	1.15E-14	2.94E-14	cyan	0.535991643	4.69E-13	GO:0006508,GO:0004222,GO:0071586,GO:0008233	AT4G01320.1	ATSTE24,STE24	Peptidase family M48 family protein	CAAX prenyl protease
Sapur.013G030700.1	234.3053445	-0.60546139	0.078942232	-7.66968	1.72E-14	4.37E-14	cyan	-0.520247662	2.88E-12					hypothetical protein
Sapur.010G031600.1	88.78546026	-0.502795291	0.065764265	-7.64542	2.08E-14	5.26E-14	cyan	-0.503016553	1.90E-11					hypothetical protein
Sapur.009G106800.1	90.24674259	0.676807022	0.088808614	7.620961	2.52E-14	6.35E-14	cyan	0.5153054	5.00E-12	GO:0005515	AT1G20080.1	ATSYTB.NTMC2T1.2.NTMC:	Calcium-dependent lipid-binding (CaLB domain) family protein	synaptotagmin
Sapur.016G208900.1	965.9923688	0.345232603	0.045301463	7.620783	2.52E-14	6.36E-14	cyan	0.540626675	2.70E-13		AT3G01780.1	TPLATE	ARM repeat superfamily protein	tplate protein
Sapur.003G005100.1	1091.313683	0.296759822	0.039000669	7.609096	2.76E-14	6.95E-14	cyan	0.523455867	2.01E-12	GO:0016021,GO:0015693,GO:0015095	AT1G71900.1		Protein of unknown function (DUF803)	magnesium transporter NIPA protein
Sapur.007G098300.1	3041.756011	-0.327912636	0.043532506	-7.53259	4.97E-14	1.24E-13	cyan	-0.504244542	1.66E-11	GO:0045454,GO:0015035,GO:0006662	AT4G03520.1	ATHM2	Thioredoxin superfamily protein	thioredoxin M-type protein
Sapur.013G000400.1	1657.529088	0.406631025	0.054101143	7.516126	5.64E-14	1.41E-13	cyan	0.500482948	2.48E-11	GO:0005515,GO:0006355,GO:0018298,GO:0009584	AT1G09570.1	FHY2,FRE1,HY8,PHYA	phytochrome A	phytochrome protein A
Sapur.002G072400.1	137.4906324	0.299418356	0.039971559	7.490785	6.85E-14	1.70E-13	cyan	0.509317437	9.64E-12	GO:0016740	AT1G36370.1	SHM7	serine hydroxymethyltransferase 7	serine hydroxymethyltransferase
Sapur.006G041800.1	63.21149012	0.558835488	0.074820692	7.468996	8.08E-14	2.01E-13	cyan	0.522001867	2.37E-12		AT1G68030.1		RING/FYVE/PHD zinc finger superfamily protein	NA
Sapur.011G125600.1	680.0644567	0.52334279	0.070250043	7.449715	9.35E-14	2.32E-13	cyan	0.522695444	2.19E-12		AT5G56290.1	ATPEX5,PEX5	peroxin 5	peroxisomal targeting signal 1 receptor
Sapur.15WG097600.1	316.3828618	0.322886048	0.043569313	7.410859	1.25E-13	3.09E-13	cyan	0.543937961	1.81E-13	GO:0035091	AT3G48195.1		Phox (PX) domain-containing protein	differentially in FDCP-like protein
Sapur.009G047800.1	33.53749979	-0.672241408	0.09076169	-7.40666	1.30E-13	3.19E-13	cyan	-0.503964219	1.71E-11		AT3G52110.1			CCP-like protein, putative
Sapur.014G062500.1	3476.120473	0.186417843	0.025193929	7.399316	1.37E-13	3.37E-13	cyan	0.512834207	6.57E-12	GO:0005524,GO:0003676	AT4G00660.1	ATRH8,RH8	RNAhelicase-like 8	DEAD-box ATP-dependent RNA helicase
Sapur.003G017500.1	1967.259864	0.447200054	0.061461102	7.276148	3.43E-13	8.34E-13	cyan	0.495345456	4.24E-11	GO:0005515,GO:0003824	AT3G15730.1	PLD,PLDALPHA1	phospholipase D alpha 1	phospholipase D alpha
Sapur.016G212100.1	400.3666389	0.315036587	0.043407178	7.257707	3.94E-13	9.54E-13	cyan	0.52365602	1.96E-12	GO:0070652,GO:0051297,GO:0051225	AT5G40740.1			HAUS augmin-like complex subunit 6 amino-terminus protein
Sapur.017G086000.1	1124.453565	0.427909012	0.059440257	7.198976	6.07E-13	1.46E-12	cyan	0.516102066	4.58E-12		AT5G15680.1		ARM repeat superfamily protein	cell morphogenesis protein, central domain protein
Sapur.001G083400.1	9995.876154	0.335154921	0.04685577	7.152906	8.50E-13	2.04E-12	cyan	0.516687799	4.29E-12	GO:0008270,GO:0008237,GO:0006508	AT1G63770.3		Peptidase M1 family protein	peptidase M1 family aminopeptidase N
Sapur.016G221100.1	675.1304977	0.253062353	0.035386508	7.15138	8.59E-13	2.06E-12	cyan	0.515757809	4.76E-12		AT5G14540.1		Protein of unknown function (DUF1421)	DNA-binding protein, putative
Sapur.005G158200.1	11.29323232	-0.734597253	0.103338078	-7.10868	1.17E-12	2.79E-12	cyan	-0.468270457	6.23E-10		AT3G58830.1		haloacid dehalogenase (HAD) superfamily protein	haloacid dehalogenase superfamily protein
Sapur.019G105100.1	2474.209216	-0.259837204	0.036802177	-7.06038	1.66E-12	3.94E-12	cyan	-0.473114885	3.92E-10	GO:0055114,GO:0051537,GO:0016491,GO:0042651,GO:0009496,GO:0016679	AT4G03280.1	PETC,PGR1	photosynthetic electron transfer C	cytochrome b6-F complex iron-sulfur subunit
Sapur.010G115600.1	1392.976062	0.19180429	0.027331532	7.017693	2.26E-12	5.33E-12	cyan	0.493388597	5.19E-11		AT5G49580.1		Chaperone DnaJ-domain superfamily protein	chaperone protein DnaJ
Sapur.018G100600.1	666.6366554	0.402348279	0.057687899	6.97457	3.07E-12	7.22E-12	cyan	0.494674164	4.55E-11		AT3G33530.1		Transducin family protein / WD-40 repeat family protein	transducin/WD40 domain protein
Sapur.T077100.1	251.7891765	-0.437737871	0.062769156	-6.97377	3.09E-12	7.26E-12	cyan	-0.452218848	2.75E-09	GO:0006457,GO:0005737	AT5G20720.1	ATCPN21.CHCPN10.CPN10.	chaperonin 20	NA
Sapur.001G063500.1	60.81259959	-0.603837487	0.086600262	-6.9727	3.11E-12	7.31E-12	cyan	-0.4589887	1.49E-09	GO:0005975,GO:0004553	AT5G49720.1	ATGH9A1.DEC.GH9A1.IRX2.	glycosyl hydrolase 9A1	NA
Sapur.003G028400.1	901.7181678	0.253397003	0.036498916	6.94259	3.85E-12	9.01E-12	cyan	0.481906753	1.66E-10	GO:0008017,GO:0007018,GO:0005524,GO:0003777,GO:0005871	AT3G16060.1		ATP binding microtubule motor family protein	kinesin motor domain protein
Sapur.014G115700.1	1490.104745	0.268914494	0.038785847	6.933315	4.11E-12	9.62E-12	cyan	0.508064092	1.10E-11	GO:0006468,GO:0004672	AT3G58640.2		Mitogen activated protein kinase kinase kinase-related	tyrosine kinase family protein
Sapur.005G086200.1	283.1185662	0.413564495	0.05970115	6.927245	4.29E-12	1.00E-11	cyan	0.46373411	9.56E-10	GO:0016020	AT4G35870.1		early-responsive to dehydration stress protein (ERD4)	transmembrane protein 63A
Sapur.003G153400.1	41.14714245	-0.453837871	0.065909886	-6.88573	5.75E-12	1.34E-11	cyan	-0.488734569	8.36E-11					NA
Sapur.001G006500.1	1520.725076	0.383863254	0.055867039	6.871015	6.37E-12	1.48E-11	cyan	0.49918805	2.84E-11	GO:0005524,GO:0003676	AT2G42520.1		P-loop containing nucleoside triphosphate hydrolases superfamily protein	DEAD-box ATP-dependent RNA helicase
Sapur.018G044400.1	417.5263084	-0.431659082	0.062907414	-6.86182	6.80E-12	1.58E-11	cyan	-0.463756422	9.54E-10	GO:0006457,GO:0005737	AT5G20720.3	ATCPN21.CHCPN10.CPN10.	chaperonin 20	NA
Sapur.010G146400.1	730.3412622	0.396517529	0.058002826	6.836176	8.13E-12	1.88E-11	cyan	0.48172252	1.69E-10	GO:0030132,GO:0030130,GO:0016192,GO:0006886,GO:0005198	AT3G11130.1		Clathrin, heavy chain	clathrin heavy chain
Sapur.013G066000.1	415.8185051	-0.27841267	0.04075379	-6.83158	8.40E-12	1.94E-11	cyan	-0.466683909	7.24E-10	GO:0006397,GO:0000445	AT5G16790.1	AtTHO7,THO7	Tho complex subunit 7/Mft1p	Ngg1 interacting factor-like 1 binding protein
Sapur.018G054000.1	1743.700556	0.517292906	0.07644852	6.766552	1.32E-11	3.03E-11	cyan	0.491345235	6.40E-11		AT2G26890.1	GRV2,KAM2	DNAJ heat shock N-terminal domain-containing protein	DnaJ family C protein
Sapur.003G117100.1	78.62009191	-0.350083878	0.051773315	-6.76186	1.36E-11	3.12E-11	cyan	-0.470186352	5.19E-10		AT3G51980.1		ARM repeat superfamily protein	NA
Sapur.006G044500.1	5.173780195	-1.013031227	0.150623368	-6.72559	1.75E-11	4.00E-11	cyan	-0.492665444	5.59E-11	GO:0035556,GO:0016020	AT3G12180.1		Cornichon family protein	cornichon protein
Sapur.004G163400.1	86.11542611	-0.378166198	0.056275366	-6.71992	1.82E-11	4.15E-11	cyan	-0.455433969	2.06E-09	GO:0000184				DUF2146 family protein
Sapur.016G223400.1	727.9693233	-0.234678854	0.035085	-6.68887	2.25E-11	5.12E-11	cyan	-0.472236954	4.27E-10	GO:0055114,GO:0016730	AT2G04700.1		ferredoxin thioredoxin reductase catalytic beta chain family protein	ferredoxin-thioredoxin reductase catalytic chain
Sapur.T069800.1	168.9602116	0.502029691	0.075626098	6.638313	3.17E-11	7.18E-11	cyan	0.533588494	6.23E-13		AT1G67140.3	SWEETIE	HEAT repeat-containing protein	NA
Sapur.005G205600.1	34.59758829	-0.50451291	0.076049703	-6.63399	3.27E-11	7.39E-11	cyan	-0.472248445	4.26E-10	GO:0006468,GO:0005524,GO:0004672	AT3G57750.2		Protein kinase superfamily protein	receptor-like kinase
Sapur.002G203600.1	50.80817108	-0.448827621	0.067854446	-6.61456	3.73E-11	8.41E-11	cyan	-0.446387898	4.64E-09		AT5G63690.1		Nucleic acid-binding, OB-fold-like protein	NA
Sapur.002G026600.1	640.1220335	0.271216998	0.041019833	6.61185	3.80E-11	8.57E-11	cyan	0.474355103	3.48E-10	GO:0005515	AT1G47270.2	AtTLP6,TLP6	tubby like protein 6	tubby-like F-box protein
Sapur.016G302200.1	157.4781362	-0.279508517	0.042373573	-6.59629	4.22E-11	9.50E-11	cyan	-0.463454555	9.81E-10	GO:0007017,GO:0005874	AT5G62700.1	TUB3	tubulin beta chain 3	NA
Sapur.004G071400.1	1129.834713	0.26088725	0.039675424	6.575538	4.85E-11	1.09E-10	cyan	0.47600479	2.96E-10		AT5G16390.2	BCCP.BCCP-1.BCCP1.CAC1.(	chloroplastic acetylcoenzyme A carboxylase 1	NA
Sapur.003G093500.1	250.3316293	0.300124811	0.045863434	6.54388	5.99E-11	1.34E-10	cyan	0.461267166	1.20E-09	GO:0007165,GO:0005515	AT4G23440.1		Disease resistance protein (TIR-NBS class)	TIR-NBS resistance protein
Sapur.016G163000.1	259.4599093	-0.437641116	0.066939007	-6.53791	6.24E-11	1.40E-10	cyan	-0.445177037	5.16E-09		AT5G58600.1	PMR5,TBL44	Plant protein of unknown function (DUF828)	Pmr5/Cas1p GDSL/SGNH-like acyl-esterase family protein
Sapur.004G010100.1	1434.905112	0.295715901	0.045304044	6.527362	6.69E-11	1.50E-10	cyan	0.450889037	3.11E-09	GO:0016226	AT4G04770.1	ABC1.ATABC1.ATNAP1.LAF6	ATP binding cassette protein 1	ABC(membrane) family transporter (Fe-S assembly/SufBCD system)
Sapur.017G088500.1	1953.362441	0.567480174	0.086974711	6.524657	6.82E-11	1.52E-10	cyan	0.442830209	6.34E-09		AT5G15948.1	CPuORF10	conserved peptide upstream open reading frame 10	S-adenosyl-L-methionine decarboxylase leader peptide protein
Sapur.001G193200.1	978.7216382	0.265938426	0.04087918	6.505474	7.74E-11	1.73E-10	cyan	0.475704068	3.05E-10		AT2G35610.1	XEG113	xyloglucanase 113	Reticulon protein 3-A3, putative
Sapur.005G130400.2	66.33688428	0.439787072	0.067710173	6.49514	8.30E-11	1.85E-10	cyan	0.455743447	2.00E-09	GO:0005515	AT1G78070.1		Transducin/WD40 repeat-like superfamily protein	plant/F28K19-28 protein
Sapur.008G161000.1	411.0679875	-0.2862917	0.044383159	-6.45046	1.12E-10	2.48E-10	cyan	-0.468328394	6.20E-10	GO:0007017,GO:0005875	AT4G15930.1		Dynein light chain type 1 family protein	dynein light chain 1, putative
Sapur.004G090800.2	57.42234652	-0.446734658	0.069482755	-6.42943	1.28E-10	2.84E-10	cyan	-0.442785648	6.37E-09	GO:0003676	AT3G19130.1	ATRBP47B,RBP47B	RNA-binding protein 47B	RNA-binding protein
Sapur.008G031800.1	38.36961896	0.521672599	0.081481207	6.402367	1.53E-10	3.38E-10	cyan	0.446119725	4.75E-09		AT4G25030.2			hypothetical protein
Sapur.006G136500.1	425.365019	0.307517903	0.048352421	6.359928	2.02E-10	4.43E-10	cyan	0.447731179	4.12E-09	GO:0005515	AT5G57360.1	ADO1,FKL2,LKP1,ZTL	Galactose oxidase/kelch repeat superfamily protein	PAS protein zeitlupe protein
Sapur.009G019200.1	862.3607233	0.375213135	0.05902967	6.356348	2.07E-10	4.54E-10	cyan	0.440732746	7.62E-09	GO:0006468,GO:0004672	AT3G46290.1	HERK1	hercules receptor kinase 1	NA
Sapur.15WG140800.1	28.80833551	-0.511041355	0.080727555	-6.33045	2.44E-10	5.35E-10	cyan	-0.418612917	4.87E-08		AT5G51680.1		hydroxyproline-rich glycoprotein family protein	hydroxyproline-rich glycoprotein family protein, putative
Sapur.002G000700.1	540.1934788	-0.222407296	0.035142291	-6.32876	2.47E-10	5.41E-10	cyan	-0.438844612	8.97E-09		AT1G21065.1			NA
Sapur.008G154900.1	1392.702728	0.224572391	0.035852071	6.263861	3.76E-10	8.16E-10	cyan	0.437012396	1.05E-08	GO:0016624,GO:0008152	AT1G59900.1	AT-E1 ALPHA,E1 ALPHA	pyruvate dehydrogenase complex E1 alpha subunit	pyruvate dehydrogenase E1 component alpha subunit
Sapur.019G052700.2	7.842754363	0.768735525	0.124039865	6.197488	5.74E-10	1.24E-09	cyan	0.715085628	7.02E-26	GO:0003824,GO:0006470,GO:0004722	AT3G02750.2		Protein phosphatase 2C family protein	protein phosphatase 2C
Sapur.002G121800.1	890.1621055	0.360353761	0.05837738	6.172832	6.71E-10	1.44E-09	cyan	0.442531148	6.51E-09	GO:0032012,GO:0005086	AT3G60860.1		SEC7-like guanine nucleotide exchange family protein	brefeldin A-inhibited guanine nucleotide-exchange protein
Sapur.T065200.1	101.8755906	-0.396611878	0.064389226	-6.1596	7.29E-10	1.57E-09	cyan	-0.425244176	2.83E-08	GO:0005515,GO:0003723	AT2G39260.1		binding;RNA binding	NA
Sapur.004G159400.1	1925.511292	0.141261226	0.023082362	6.119878	9.36E-10	2.00E-09	cyan	0.449997332	3.36E-09		AT1G07990.1		SIT4 phosphatase-associated family protein	SIT4 phosphatase-associated protein
Sapur.006G207500.1	35.12339788	-0.450693329	0.073875575	-6.10071	1.06E-09	2.25E-09	cyan	-0.400974364	1.95E-07					NA
Sapur.002G132400.1	518.7641565	0.266615567	0.04373073	6.096755	1.08E-09	2.31E-09	cyan	0.433249649	1.45E-08	GO:0030127,GO:0008270,GO:0006888,GO:0006886	AT4G01810.1		Sec23/Sec24 protein transport family protein	protein transporter Sec23
Sapur.007G070100.1	277.9524704	0.674045421	0.111040405	6.070272	1.28E-09	2.71E-09	cyan	0.448704758	3.78E-09	GO:0005515,GO:0016459,GO:0005524,GO:0003774	AT3G19960.1	ATM1	myosin 1	myosin VIII, putative
Sapur.T006600.1	31.21096401	-0.480149071	0.079897933	-6.00953	1.86E-09	3.93E-09	cyan	-0.415038679	6.49E-08	GO:0006355,GO:0005634	AT5G18230.2		transcription regulator NOT2/NOT3/NOT5 family protein	NA
Sapur.001G043400.1	43.53160756	-0.469392776	0.078118674	-6.00871	1.87E-09	3.95E-09	cyan	-0.409572101	1.00E-07		AT2G33180.1			hypothetical protein
Sapur.15WG028300.1	383.1177034	-0.220907231	0.03701191	-5.96854	2.39E-09	5.03E-09	cyan	-0.422367635	3.59E-08	GO:0006468,GO:0004672	AT3G17850.1		Protein kinase superfamily protein	Serine/Threonine kinase domain protein
Sapur.019G105700.1	79.33675807	0.587003674	0.098551681	5.956303	2.58E-09	5.42E-09	cyan	0.426758103	2.50E-08					NA
Sapur.018G094600.1	400.8059026	0.169239267	0.02854752	5.928335	3.06E-09	6.40E-09	cyan	0.440646526	7.67E-09	GO:0008270	AT5G20350.1	TIP1	Ankyrin repeat family protein with DHHC zinc finger domain	S-acyltransferase TIP1
Sapur.018G054900.1	65.03222407	0.63295762	0.106999698	5.915508	3.31E-09	6.91E-09	cyan	0.429458653	1.99E-08	GO:0005515	AT3G24440.1	VIL1,VRN5	Fibronectin type III domain-containing protein	PHD finger protein
Sapur.010G054300.1	674.5610324	0.30366895	0.051478072	5.898996	3.66E-09	7.63E-09	cyan	0.464902662	8.57E-10	GO:0018342,GO:0008318	AT4G24490.1	ATRGTA1,RGTA1	RAB geranylgeranyl transferase alpha subunit 1	NA
Sapur.011G099100.1	148.6938967	-0.251535422	0.042957336	-5.85547	4.76E-09	9.88E-09	cyan	-0.461298584	1.20E-09		AT3G20600.1	NDR1	Late embryogenesis abundant (LEA) hydroxyproline-rich glycoprotein family	Non-race specific disease resistance-like protein
Sapur.016G240500.1	5.624811537	-0.66056456	0.113178868	-5.83647	5.33E-09	1.11E-08	cyan	-0.472795821	4.04E-10	GO:0005515	AT1G06230.1	GTE4	global transcription factor group E4	global transcription factor group protein, putative
Sapur.016G045600.1	79.92736118	-0.515208305	0.088825957	-5.8002	6.62E-09	1.37E-08	cyan	-0.4076457	1.16E-07					NA
Sapur.016G223700.1	694.4742905	-0.197918158	0.034179784	-5.7905	7.02E-09	1.45E-08	cyan	-0.435651575	1.18E-08		AT3G55250.1			calcium homeostasis regulator CHoR1
Sapur.018G035100.1	31.79729606	0.51915599	0.089810086	5.780598	7.44E-09	1.53E-08	cyan	0.427961782	2.26E-08		AT5G11580.1		Regulator of chromosome condensation (RCC1) family protein	NA

Sapur.016G217600.2	10.57985485	0.653101554	0.113542023	5.752069	8.82E-09	1.81E-08	cyan	0.532099417	7.41E-13	GO:0006468,GO:0004672,GO:0005524	AT5G47750.1	D6PKL2,PK5	D6 protein kinase like 2	Serine/Threonine kinase domain protein
Sapur.003G096800.1	212.1389097	0.253157321	0.044056656	5.746177	9.13E-09	1.87E-08	cyan	0.447814227	4.09E-09	GO:0008270,GO:0019789	AT5G41580.1		RING/U-box superfamily protein	zinc finger MIZ domain protein
Sapur.013G109900.2	11.25089185	-0.51646092	0.090546529	-5.70382	1.17E-08	2.39E-08	cyan	-0.463713516	9.58E-10	GO:0005515	AT1G06230.1	GTE4	global transcription factor group E4	global transcription factor group protein, putative
Sapur.018G062000.1	1604.548272	0.291849123	0.052147767	5.59658	2.19E-08	4.42E-08	cyan	0.379614079	9.42E-07	GO:0007165	AT4G28910.2	NINJA	novel interactor of JAZ	ninja-family protein mc410, putative
Sapur.001G150700.1	76.37479129	-0.491554836	0.08797694	-5.58731	2.31E-08	4.65E-08	cyan	-0.43770812	9.90E-09	GO:0007017,GO:0005875	AT1G52240.2	ATROPGEF11.PIRF1.ROPGEI	RHO guanyl-nucleotide exchange factor 11	dynein light chain LC6, flagellar outer arm protein
Sapur.002G064300.1	200.3827319	-0.358493897	0.064208908	-5.58324	2.36E-08	4.76E-08	cyan	-0.389216729	4.70E-07	GO:0051603,GO:0005839,GO:0004298	AT1G21720.1	PBC1	proteasome beta subunit C1	NA
Sapur.016G256800.1	2418.739818	-0.402617354	0.072295968	-5.56902	2.56E-08	5.16E-08	cyan	-0.441505677	7.12E-09		AT5G53880.1			NA
Sapur.001G161800.1	5109.306887	0.243339591	0.043708967	5.567269	2.59E-08	5.21E-08	cyan	0.435482675	1.20E-08	GO:0055114,GO:0004471,GO:0051287	AT1G79750.1	ATNADP-ME4,NADP-ME4	NADP-malic enzyme 4	malic enzyme NAD(P) binding domain protein
Sapur.005G073700.1	718.427831	0.373532927	0.06740794	5.541379	3.00E-08	6.03E-08	cyan	0.416058973	5.98E-08		AT5G23110.1		Zinc finger, C3HC4 type (RING finger) family protein	C3HC4-type RING finger protein
Sapur.014G131400.3	53.005515	-0.382923214	0.069194749	-5.53399	3.13E-08	6.28E-08	cyan	-0.407019858	1.22E-07	GO:0006352,GO:0005634,GO:0051090	AT1G04950.3	ATTAF6,TAF6,TAFII59	TATA BOX ASSOCIATED FACTOR II 59	transcription initiation factor TFIID protein, putative
Sapur.004G111100.1	113.7878075	-0.31434014	0.057163253	-5.49899	3.82E-08	7.64E-08	cyan	-0.39831321	2.39E-07					NA
Sapur.004G144500.1	73.58259438	0.622741735	0.113409169	5.491106	3.99E-08	7.98E-08	cyan	0.400354596	2.04E-07	GO:0051536,GO:0009055,GO:0055114,GO:0046872,GO:0016491	AT3G43600.1	AAO2.AO3.AOgamma.atAO	aldehyde oxidase 2	NA
Sapur.003G119400.1	1181.870658	0.20796718	0.037934282	5.482302	4.20E-08	8.38E-08	cyan	0.406889918	1.24E-07		AT5G13390.1	NEF1	no exine formation 1	No exine formation protein
Sapur.017G000100.1	633.3700777	0.567929456	0.104289664	5.445693	5.16E-08	1.03E-07	cyan	0.401633407	1.85E-07	GO:0055114,GO:0006555,GO:0004489				NA
Sapur.013G129100.1	28.78808302	0.521813505	0.095860111	5.443489	5.22E-08	1.04E-07	cyan	0.380652936	8.75E-07	GO:0016020,GO:0008375	AT1G03520.1		Core-2/I-branching beta-1,6-N-acetylglucosaminyltransferase family protein	core-2/I-branching beta-1%2C6-N-acetylglucosaminyltransferase-like protein
Sapur.004G136400.1	57.55141818	-0.390769867	0.072168974	-5.41465	6.14E-08	1.22E-07	cyan	-0.41284624	7.73E-08	GO:0030170,GO:0009058	AT5G53970.1		Tyrosine transaminase family protein	NA
Sapur.004G100200.1	3303.513569	0.237425029	0.044329986	5.355856	8.52E-08	1.68E-07	cyan	0.380449841	8.88E-07	GO:0016021	AT5G08500.1		Transmembrane CLPTM1 family protein	cleft lip and palate transmembrane protein
Sapur.019G109400.1	905.4984032	0.26981786	0.050402435	5.35327	8.64E-08	1.70E-07	cyan	0.406918399	1.23E-07	GO:0008270,GO:0005515	AT5G58410.1		HEAT/U-box domain-containing protein	E3 ubiquitin-protein ligase listerin, putative
Sapur.014G090400.1	30.76152409	0.645342359	0.120757106	5.344136	9.08E-08	1.79E-07	cyan	0.394988344	3.07E-07	GO:0055085,GO:0042626,GO:0016021,GO:0006810,GO:0005524	AT1G02520.1	PGP11	P-glycoprotein 11	NA
Sapur.014G113300.1	215.2354553	0.440356245	0.082611481	5.330449	9.80E-08	1.93E-07	cyan	0.398508731	2.35E-07	GO:0016021,GO:0015693,GO:0015095	AT3G26670.1		Protein of unknown function (DUF803)	magnesium transporter NIPA protein
Sapur.016G233800.1	24.2451049	-0.506659833	0.095617055	-5.29884	1.17E-07	2.29E-07	cyan	-0.354105853	5.37E-06					hypothetical protein
Sapur.006G182300.1	229.3906904	-0.44286785	0.083641634	-5.29483	1.19E-07	2.34E-07	cyan	-0.410497659	9.31E-08		AT3G13540.1	ATMYB5,MYB5	myb domain protein 5	Myb-like transcription factor 2, putative
Sapur.004G143000.1	1105.969739	0.301158841	0.056915935	5.291292	1.21E-07	2.38E-07	cyan	0.408821197	1.06E-07	GO:0005515	AT5G22760.1		PHD finger family protein	DNA-binding and zinc-finger protein, putative
Sapur.003G015200.1	103.5289634	-0.321559935	0.060859545	-5.28364	1.27E-07	2.48E-07	cyan	-0.391386495	4.01E-07		AT3G18430.1		Calcium-binding EF-hand family protein	calcineurin B subunit (protein phosphatase 2b regulatory subunit)-like protein
Sapur.008G022700.1	591.1835185	0.379590364	0.0725205	5.234249	1.66E-07	3.23E-07	cyan	0.402076419	1.79E-07	GO:0006355,GO:0003677	AT5G04410.1	anac078,NAC2	NAC domain containing protein 2	NAC transcription factor
Sapur.018G013000.1	43.8270808	-0.396624236	0.076265026	-5.2006	1.99E-07	3.85E-07	cyan	-0.391905195	3.86E-07	GO:0045747,GO:0007049,GO:0006221	AT5G10810.1	ATER,ER	enhancer of rudimentary protein, putative	enhancer of rudimentary-like protein
Sapur.004G086600.1	18.25324114	0.399548136	0.077115353	5.181175	2.20E-07	4.27E-07	cyan	0.449080773	3.65E-09		AT1G67140.3	SWEETIE	HEAT repeat-containing protein	NA
Sapur.006G231100.2	24.55673239	-0.445061569	0.085937871	-5.17888	2.23E-07	4.32E-07	cyan	-0.379200091	9.70E-07		AT4G20325.1			Ydr279p family (RNase H2 complex component) protein
Sapur.008G039200.1	67.11805876	-0.343817921	0.067180665	-5.11781	3.09E-07	5.94E-07	cyan	-0.391275901	4.04E-07		AT2G39500.1			transmembrane protein, putative
Sapur.019G022900.1	67.88687973	0.428769432	0.084233428	5.090253	3.58E-07	6.85E-07	cyan	0.375119799	1.29E-06	GO:0005515	AT3G04580.1	EIN4	Signal transduction histidine kinase, hybrid-type, ethylene sensor	NA
Sapur.T047700.1	46.14239298	-0.311487216	0.061230468	-5.08713	3.64E-07	6.96E-07	cyan	-0.382915049	7.44E-07	GO:0009165,GO:0004749,GO:0000287	AT2G44530.1		Phosphoribosyltransferase family protein	ribose-phosphate pyrophosphokinase
Sapur.010G136300.2	3.658948245	-0.768960141	0.152073574	-5.0565	4.27E-07	8.15E-07	cyan	-0.409932227	9.74E-08		AT1G79260.1			UPF0678 fatty acid-binding protein-like protein
Sapur.T069200.1	113.5374294	0.419491541	0.084056683	4.990579	6.02E-07	1.14E-06	cyan	0.414903653	6.56E-08		AT1G67140.3	SWEETIE	HEAT repeat-containing protein	NA
Sapur.001G102200.3	8.241816017	0.561629387	0.112952576	4.972258	6.62E-07	1.25E-06	cyan	0.611211181	1.87E-17	GO:0005515,GO:0008270	AT4G25230.1	RIN2	RPM1 interacting protein 2	E3 ubiquitin protein ligase RIN3
Sapur.006G184600.1	412.8012415	0.175463784	0.035473021	4.946401	7.56E-07	1.43E-06	cyan	0.366262772	2.39E-06		AT5G20610.1			EEIG1/EHBP1 protein amino-terminal domain protein
Sapur.007G037300.1	108.5718159	-0.493642437	0.100202381	-4.92645	8.37E-07	1.58E-06	cyan	-0.379788488	9.31E-07					NA
Sapur.014G043500.1	71.30318989	-0.249362802	0.050802144	-4.90851	9.18E-07	1.72E-06	cyan	-0.354420923	5.26E-06		AT3G15351.1			NA
Sapur.017G042700.1	4083.597518	0.207007659	0.042352945	4.88768	1.02E-06	1.91E-06	cyan	0.352773286	5.86E-06		AT2G32730.1		26S proteasome regulatory complex, non-ATPase subcomplex, Rpn2/Psmd1 subunit	26S proteasome non-ATPase regulatory subunit
Sapur.019G033300.2	4.565919613	-0.651214473	0.13350157	-4.87795	1.07E-06	2.01E-06	cyan	-0.444225484	5.61E-09		AT2G39050.1		hydroxyproline-rich glycoprotein family protein	hypothetical protein
Sapur.014G033000.1	1872.220133	0.142224497	0.029411587	4.835662	1.33E-06	2.47E-06	cyan	0.363313384	2.91E-06	GO:0055114,GO:0004471,GO:0051287	AT2G13560.1	NAD-ME1	NAD-dependent malic enzyme 1	NAD-dependent malic enzyme
Sapur.017G008000.1	3861.167836	0.217580307	0.045017404	4.833249	1.34E-06	2.50E-06	cyan	0.371113979	1.71E-06		AT2G20580.1	ATRPN1A,RPN1A	26S proteasome regulatory subunit S2 1A	26S proteasome non-ATPase regulatory subunit
Sapur.018G044200.1	1264.845239	0.254446218	0.052792864	4.819709	1.44E-06	2.67E-06	cyan	0.386488851	5.74E-07		AT5G11700.2			transmembrane protein, putative
Sapur.003G017600.1	232.3343677	-0.355756877	0.073857743	-4.81679	1.46E-06	2.71E-06	cyan	-0.344629267	9.88E-06	GO:0006355,GO:0003700	AT1G15360.1	SHN1,WIN1	Integrase-type DNA-binding superfamily protein	ethylene-responsive-element-binding factor 1
Sapur.016G141700.1	120.5985088	-0.280909459	0.058401935	-4.80993	1.51E-06	2.80E-06	cyan	-0.360635767	3.49E-06		AT2G29950.1	ELF4-L1	ELF4-like 1	NA
Sapur.006G114000.1	115.0853876	-0.322320982	0.067239722	-4.79361	1.64E-06	3.04E-06	cyan	-0.366919524	2.28E-06					NA
Sapur.019G002000.1	1661.97582	0.209340517	0.04384931	4.774089	1.81E-06	3.34E-06	cyan	0.35205284	6.14E-06	GO:0016887,GO:0016021,GO:0006812	AT5G23630.1	MIA,PDR2	phosphate deficiency response 2	cation-transporting ATPase, putative
Sapur.006G076300.1	191.6297284	0.163656902	0.034524116	4.740365	2.13E-06	3.93E-06	cyan	0.375932577	1.22E-06	GO:0006468,GO:0005524,GO:0004672	AT2G37840.1		Protein kinase superfamily protein	Serine/Threonine kinase domain protein
Sapur.010G030800.1	13.73845481	0.582819463	0.122975077	4.73933	2.14E-06	3.95E-06	cyan	0.36834417	2.07E-06	GO:0006355	AT1G70060.1	SNL4	SIN3-like 4	paired amphipathic helix Sin3-like protein
Sapur.016G248300.1	162.5204323	-0.18336876	0.039019177	-4.69945	2.61E-06	4.79E-06	cyan	-0.365452792	2.52E-06		AT1G53400.1		Ubiquitin domain-containing protein	ubiquitin domain protein
Sapur.012G059100.2	24.27542518	-0.330518597	0.070332458	-4.69938	2.61E-06	4.79E-06	cyan	-0.357298057	4.35E-06		AT2G36330.1		Uncharacterised protein family (UPF0497)	DUF588 domain protein
Sapur.003G167200.2	5.220244603	0.568426486	0.122970356	4.622468	3.79E-06	6.91E-06	cyan	0.469286472	5.66E-10	GO:0016592,GO:0006357,GO:0001104	AT1G55325.2	GCT	RNA polymerase II transcription mediators	mediator of RNA polymerase II transcription subunit 13
Sapur.018G040400.1	75.6673396	0.360309147	0.078902397	4.566517	4.96E-06	8.98E-06	cyan	0.302781639	0.000116014	GO:0016791,GO:0008152	AT1G17710.1		Pyridoxal phosphate phosphatase-related protein	2%2C3-diketo-5-methylthio-1-phosphopentane phosphatase
Sapur.006G047200.2	11.39427176	0.528765343	0.115899649	4.562269	5.06E-06	9.16E-06	cyan	0.742818526	8.39E-29		AT5G25270.1		Ubiquitin-like superfamily protein	large proline-rich protein bat3
Sapur.002G154100.1	1021.364055	0.228197633	0.050021234	4.562015	5.07E-06	9.17E-06	cyan	0.39267027	3.64E-07	GO:0008270	AT3G62900.1		CW-type Zinc Finger	CW-type zinc finger protein, putative
Sapur.001G022500.1	239.8596319	0.241193139	0.052989022	4.551757	5.32E-06	9.62E-06	cyan	0.370516872	1.78E-06	GO:0046872	AT5G19420.1		Regulator of chromosome condensation (RCC1) family with FYVE zinc finger domain	regulator of chromosome condensation-like protein with FYVE zinc finger protein
Sapur.014G054200.1	784.2245203	0.51989456	0.114608783	4.536254	5.73E-06	1.03E-05	cyan	0.361744117	3.24E-06		AT2G45540.1		WD-40 repeat family protein / beige-related	beige-related and WD-40 repeat protein
Sapur.006G158000.1	136.913472	-0.292801945	0.064628977	-4.53051	5.88E-06	1.06E-05	cyan	-0.341740076	1.18E-05		AT5G06700.1	TBR	Plant protein of unknown function (DUF828)	Pmr5/Cas1p GDSL/SGNH-like acyl-esterase family protein
Sapur.010G012200.1	251.8312407	0.262413681	0.057963953	4.527187	5.98E-06	1.08E-05	cyan	0.367557765	2.18E-06		AT3G23590.1	MED33A,RFR1	REF4-related 1	mediator of RNA polymerase II transcription subunit 33A
Sapur.014G052800.1	343.7684892	-0.196869716	0.044263733	-4.44765	8.68E-06	1.55E-05	cyan	-0.376349165	1.19E-06	GO:0006457,GO:0003755,GO:0000413	AT1G01940.1		Cyclophilin-like peptidyl-prolyl cis-trans isomerase family protein	peptidyl-prolyl cis-trans isomerase, cyclophilin-type protein
Sapur.T034200.1	233.2473806	-0.199705877	0.045760399	-4.36416	1.28E-05	2.26E-05	cyan	-0.322002845	3.91E-05		AT5G23890.1			NA
Sapur.014G079700.1	5.690456981	0.631536662	0.145737944	4.333372	1.47E-05	2.59E-05	cyan	0.351192823	6.49E-06	GO:0005524,GO:0016772	AT1G01220.1	AtFKGP,FKGP	L-fucokinase/GDP-L-fucose pyrophosphorylase	fucokinase/fucose pyrophosphorylase
Sapur.15WG016500.1	83.14595978	0.279442587	0.064993355	4.299556	1.71E-05	3.01E-05	cyan	0.315038322	5.85E-05		AT4G35905.1			Trm112p-like protein
Sapur.014G066400.1	78.30776168	0.292127277	0.068092546	4.290151	1.79E-05	3.13E-05	cyan	0.457061313	1.77E-09		AT4G00355.2			transmembrane protein, putative
Sapur.016G079500.1	248.3580611	0.298040924	0.07002959	4.255928	2.08E-05	3.64E-05	cyan	0.348829083	7.56E-06		AT1G07910.1	ATRNL,RNL	RNAligase	NA
Sapur.003G129500.2	93.51250249	-0.254425983	0.060931625	-4.1756	2.97E-05	5.15E-05	cyan	-0.306798341	9.30E-05	GO:0008270	AT1G67325.1		Ran BP2/NZF zinc finger-like superfamily protein	RanBP2-type zinc finger protein
Sapur.010G000900.1	1481.192403	0.200889178	0.048271107	4.161686	3.16E-05	5.47E-05	cyan	0.290068443	0.000228654		AT3G17430.1		Nucleotide-sugar transporter family protein	sugar phosphate/phosphate translocator, putative
Sapur.006G222200.1	1070.523539	0.280336503	0.067511755	4.15241	3.29E-05	5.69E-05	cyan	0.30589198	9.78E-05		AT4G31590.1	ATCSLC05.ATCSLC5.CSLC05	Cellulose-synthase-like C5	cellulose synthase-like C1-2, glycosyltransferase family 2 protein
Sapur.003G112400.1	5127.286702	0.151629367	0.036527765	4.151072	3.31E-05	5.72E-05	cyan	0.306004763	9.72E-05	GO:0005975,GO:0004553	AT5G49720.1	ATGH9A1.DEC.GH9A1.IRX2.	glycosyl hydrolase 9A1	membrane-anchored endo-1%2C4-beta-glucanase
Sapur.016G081800.4	7.16141635	-0.486069787	0.11768778	-4.13016	3.63E-05	6.25E-05	cyan	0.37109578	1.71E-06		AT2G37080.1	RIP3	ROP interactive partner 3	interactor of constitutive active ROPs protein, putative
Sapur.002G070200.1	36.12089012	0.426583059	0.103725248	4.112625	3.91E-05	6.73E-05	cyan	0.31361942	6.34E-05		AT1G77720.1	PPK1	putative protein kinase 1	NA
Sapur.003G110500.1	609.0003093	0.18167597	0.044216062	4.108823	3.98E-05	6.84E-05	cyan	0.312474218	6.77E-05	GO:0016192,GO:0006886	AT1G22860.1		Vacuolar sorting protein 39	vacuolar sorting protein 39 domain protein
Sapur.005G134200.1	573.8720318	0.148859846	0.036250066	4.106471	4.02E-05	6.91E-05	cyan	0.313018929	6.56E-05		AT1G36990.1			hypothetical protein
Sapur.008G138700.1	133.0308221	-0.310358947	0.076062162	-4.08033	4.50E-05	7.71E-05	cyan	-0.33611284	1.68E-05	GO:0003676	AT2G33410.1		RNA-binding (RRM/RBD/RNP motifs) family protein	RNA recognition motif 2 in heterogeneous nuclear ribonucleoprotein A1 (hnRNP A1)-like protein
Sapur.002G049100.1	67.5368387	0.288758831	0.070810633	4.077902	4.54E-05	7.79E-05	cyan	0.315161703	5.81E-05	GO:0055114,GO:0016491,GO:0006633,GO:0005506	AT1G14290.1	SBH2	sphingoid base hydroxylase 2	SUR2 hydroxylase/desaturase, putative
Sapur.016G138800.2	103.9208968	-0.269797353	0.06618618	-4.07634	4.58E-05	7.84E-05	cyan	-0.29296035	0.000196493	GO:0055085,GO:0016021	AT3G46980.3	PHT4;3	phosphate transporter 4;3	anion transporter, putative
Sapur.008G162400.1	632.6391661	-0.146089599	0.036120108	-4.04455	5.24E-05	8.96E-05	cyan	-0.323735421	3.54E-05		AT4G15802.1	AtHSBP,HSBP	heat shock factor binding protein	heat shock factor-binding protein
Sapur.010G175000.1	450.2814083	0.212206075	0.052495691	4.042352	5.29E-05	9.04E-05	cyan	0.347579132	8.19E-06	GO:0008270,GO:0031668,GO:0019789,GO:0016925,GO:0016049,GO:0005634	AT5G60410.2	ATSIZ1,SIZ1	DNA-binding protein with MIZ/SP-RING zinc finger, PHD-finger and SAP domain	E3 SUMO-protein ligase SIZ1
Sapur.011G006200.1	1690.113203	-0.166396563	0.041229658	-4.03585	5.44E-05	9.29E-05	cyan	-0.390034175	4.43E-07	GO:0016021	AT4G04470.1	PMP22	Peroxisomal membrane 22 kDa (Mpv17/PMP22) family protein	peroxisomal membrane protein PMP22
Sapur.008G042000.1	94.77553417	0.309815708	0.077775742	3.983449	6.79E-05	0.000115298	cyan	0.315348533	5.75E-05		AT3G12570.1	FYD	FYD	sodium- and lithium-tolerant protein
Sapur.018G081300.1	2062.476538	0.164530143	0.041666871	3.948704	7.86E-05	0.000132816	cyan	0.301502009	0.000124391	GO:0055085,GO:0016020,GO:0006811,GO:0005216	AT5G57940.1	ATCNGC5,CNGC5	cyclic nucleotide gated channel 5	cyclic nucleotide-gated ion channel protein, putative
Sapur.013G077800.1	1214.689473	0.185507854	0.047047645	3.942979	8.05E-05	0.000135965	cyan	0.32513388	3.26E-05		AT5G16680.1		RING/FYVE/PHD zinc finger superfamily protein	NA
Sapur.014G125800.1	766.4694913	0.138188294	0.035050018	3.942603	8.06E-05	0.000136165	cyan	0.299241925	0.000140582	GO:0008270,GO:0005515,GO:0005524	AT1G05120.1		Helicase protein with RING/U-box domain	DNA repair protein rad5
Sapur.010G059700.1	1256.92685	0.239547478	0.061331589	3.905776	9.39E-05	0.000157967	cyan	0.32659314	2.99E-05		AT3G22380.1	TIC	time for coffee	time for coffee protein, putative
Sapur.016G183800.1	325.6225141	-0.218761171	0.056180019	-3.89393	9.86E-05	0.00016576	cyan	-0.280577956	0.000371835		AT5G13340.1			arginine and glutamate-rich 1 protein
Sapur.006G203700.1	1090.608881	0.188764224	0.048526078	3.889954	0.000100263	0.00016845	cyan	0.328827308	2.61E-05		AT4G32620.1		Enhancer of polycomb-like transcription factor protein	enhancer of polycomb-like transcription factor protein, putative
Sapur.016G023900.1	160.464276	-0.273670921	0.07052198	-3.88065	0.000104179	0.000174869	cyan	-0.274716208	0.000497841		AT4G27640.1		ARM repeat superfamily protein	NA
Sapur.15WG082200.1	10.6798958	0.445603519	0.114985862	3.87529	0.000106498	0.000178698	cyan	0.315605226	5.67E-05	GO:0016021,GO:0006810,GO:0005215	AT3G47740.1	ATATH2,ATH2	ABC2 homolog 2	NA
Sapur.006G230900.1	1462.303578	0.188024538	0.048521076	3.875111	0.000106576	0.00017882	cyan	0.334176548	1.89E-05		AT4G31160.1	DCAF1	DDB1-CUL4 associated factor 1	DDB1- and CUL4-associated factor-like protein
Sapur.011G112600.2	6.158378397	-0.470900975	0.12269649	-3.83793	0.000124074	0.000207442	cyan	-0.567062875	9.75E-15	GO:0005975,GO:0004553	AT1G32860.1		Glycosyl hydrolase superfamily protein	glucan endo-1%2C3-beta-D-glucosidase
Sapur.016G096200.1	1698.029129	0.152415299	0.040250522	3.786666	0.000152682	0.000253823	cyan	0.276156557	0.000463667		AT3G09090.1	DEX1	defective in exine formation protein (DEX1)	defective in exine formation protein
Sapur.003G029900.1	55.71615581	-0.264534461	0.070007026	-3.77868	0.000157659	0.000261873	cyan	-0.294366413	0.000182426					transmembrane protein, putative
Sapur.016G221000.2	5.073312189	-0.476854581	0.12672026	-3.76305	0.000167854	0.000278262	cyan	-0.332258155	2.13E-05	GO:0016020,GO:0008375	AT5G14550.1		Core-2/I-branching beta-1,6-N-acetylglucosaminyltransferase family protein	core-2/I-branching enzyme
Sapur.006G038400.1	1794.103206	0.192607781	0.051764385	3.720855	0.000198549	0.000327604	cyan	0.282270406	0.000341378		AT2G41790.1		Insulinase (Peptidase family M16) family protein	insulin-degrading enzyme, putative
Sapur.014G089600.1	331.8199629	-0.175810811	0.047699343	-3.68581	0.000227975	0.000374474	cyan	-0.268646881	0.000668956		AT3G62140.1			NEFA-interacting nuclear protein NIP30 Nefa-Nip30-N: amino-terminal domain protein
Sapur.016G143900.1	139.8599428	0.402818001	0.11057863	3.64282	0.000269668	0.000440379	cyan	0.304610158	0.000104954		AT5G12400.1		DNA binding;zinc ion binding;DNA binding	NA
Sapur.017G106200.1	215.5007272	-0.250498043	0.069297282	-3.61483	0.000300542	0.000489397	cyan	-0.264733232	0.000806439		AT4G09460.1	AtMYB6,MYB6	myb domain protein 6	MYB-like transcription factor 6, putative
Sapur.008G065500.1	162.5193294	0.318558388	0.088187671	3.612278	0.000303519	0.000494098	cyan	0.279060751	0.000401257	GO:0055114,GO:0003824,GO:0006164,GO:0003938	AT1G79470.1		Aldolase-type TIM barrel family protein	NA
Sapur.017G105400.1	1342.502261	0.142770155	0.039988305	3.570298	0.000356576	0.000578192	cyan	0.30555208	9.96E-05	GO:0005515	AT3G03380.1	DegP7	DegP protease 7	DegP protease-like protein
Sapur.010G164800.1	6.571799823	0.707608833	0.199709593	3.543189	0.000395319	0.00063901	cyan	0.279105807	0.000400353	GO:0016624,GO:0008152,GO:0055114,GO:0030976,GO:0006099,GO:0004591	AT5G65750.1		2-oxoglutarate dehydrogenase, E1 component	2-oxoglutarate dehydrogenase, E1 component
Sapur.002G046700.1	129.8219049	0.331255767	0.09426894	3.513944	0.000441506	0.000711443	cyan	0.26022492	0.000996732	GO:0016020,GO:0006075,GO:0003843,GO:0000148	AT1G06490.1	ATGSL07,atgsl7,gsl07	glucan synthase-like 7	callose synthase
Sapur.009G021800.2	26.7606478	0.24690217	0.071530664	3.451697	0.000557073	0.000892018	cyan	0.484085676	1.33E-10	GO:0016567,GO:0004842,GO:0005515	AT3G46510.1	ATPUB13,PUB13	plant U-box 13	spotted leaf protein, putative
Sapur.004G130100.1	480.365739	-0.212154105	0.06161886	-3.44301	0.000575287	0.000920203	cyan	-0.279360361	0.000395281	GO:0005622,GO:0003779,GO:0030042,GO:0015629	AT2G16700.1	ADF5,ATADF5	actin depolymerizing factor 5	actin-depolymerizing factor 5
Sapur.014G115800.1	78.81226718	0.354139604	0.10352845	3.420698	0.000624606	0.000996681	cyan	0.284165004	0.000310033					NA
Sapur.019G084300.1	0.948617574	-0.900581911	0.264154105	-3.40931	0.000651286	0.001038003	cyan	-0.474235905	3.52E-10	GO:0046933,GO:0015986	AT4G09650.1	ATPD	ATP synthase delta-subunit gene	ATP synthase F1, delta subunit
Sapur.15WG089200.1	31.06204709	0.333089352	0.0987335	3.37362	0.000741866	0.001177713	cyan	0.276363839	0.000458932	GO:0016773,GO:0048015,GO:0046854	AT1G60490.1	ATVPS34,PI3K,VPS34	vacuolar protein sorting 34	phosphatidylinositol 3-kinase
Sapur.007G077400.1	23.92256334	0.384536612	0.114236098	3.366157	0.000762234	0.001209003	cyan	0.272310007	0.00056016	GO:0005515,GO:0006468,GO:0005524,GO:0004672	AT5G10020.1		Leucine-rich receptor-like protein kinase family protein	inactive receptor kinase, putative
Sapur.006G088000.1	553.9094901	0.196078956	0.059446473	3.298412	0.000972334	0.001529777	cyan	0.265036603	0.000794919		AT3G51650.1			NA
Sapur.016G120900.1	432.7790178	0.157709576	0.047819009	3.298052	0.000973581	0.001531667	cyan	0.246649697	0.001845245		AT2G26920.1		Ubiquitin-associated/translation elongation factor EF1B protein	ubiquitin-associated/TS-N domain protein, putative
Sapur.011G047100.1	710.8548632	0.156144967	0.048130177	3.244222	0.00117772	0.001842744	cyan	0.229567382	0.00382537	GO:0007165	AT4G18700.1	ATWL4.CIPK12.SnRK3.9.WL	CBL-interacting protein kinase 12	CBL-interacting Serine/Threonine-kinase
Sapur.014G102400.1	291.6881834	-0.196461503	0.06176494	-3.18079	0.001468724	0.002282949	cyan	-0.241788372	0.002282419		AT3G52860.1			mediator of RNA polymerase II transcription subunit 28, putative
Sapur.007G008800.2	0.368241547	1.217542972	0.384126792	3.169638	0.001526288	0.002369641	cyan	0.564859	1.30E-14	GO:0055085,GO:0016020	AT5G10490.1	MSL2	MSCS-like 2	mechanosensitive channel of small conductance-like protein
Sapur.006G219100.1	532.138871	0.224401233	0.071393385	3.143166	0.001671313	0.002587753	cyan	0.264555293	0.000813268	GO:0005515,GO:0003723	AT5G57870.2	eIFiso4G1	MIF4G domain-containing protein / MA3 domain-containing protein	NA
Sapur.010G039000.2	3.839979534	-0.404032048	0.131808509	-3.0653	0.002174549	0.003340504	cyan	-0.340133363	1.31E-05	GO:0031011,GO:0006338	AT5G43500.2	ARP9,ATARP9	actin-related protein 9	actin-related protein
Sapur.017G082800.2	6.171090823	-0.521944397	0.17409931	-2.99797	0.00271785	0.004146608	cyan	0.297599708	0.000153556		AT5G15740.1		O-fucosyltransferase family protein	GDP-fucose O-fucosyltransferase-like protein
Sapur.017G044400.2	1.816207813	-0.569833609	0.191171399	-2.98075	0.002875461	0.004377381	cyan	0.359157666	3.85E-06	GO:0046923,GO:0016021,GO:0006621	AT3G25040.1	ERD2B	endoplasmic reticulum retention defective 2B	ER lumen protein retaining receptor
Sapur.006G071100.2	0.528690392	-1.124483215	0.384997425	-2.92076	0.003491841	0.005279022	cyan	-0.388901465	4.81E-07		AT2G37660.1		NAD(P)-binding Rossmann-fold superfamily protein	NAD-dependent epimerase/dehydratase
Sapur.012G109100.1	3140.297998	0.109376656	0.037581125	2.910415	0.003609496	0.00544967	cyan	0.245748945	0.001919993	GO:0006508,GO:0004252	AT4G20850.1	TPP2	tripeptidyl peptidase ii	tripeptidyl peptidase II, putative
Sapur.010G086000.1	71.02909053	0.346800774	0.120387962	2.880693	0.003968018	0.005974063	cyan	0.232987647	0.003319165					NA
Sapur.005G160500.1	429.3259404	0.161937936	0.056457736	2.868304	0.004126792	0.006204068	cyan	0.216262553	0.006520731	GO:0017119,GO:0006891	AT1G67930.1		Golgi transport complex protein-related	oligomeric subunit 5
Sapur.011G101100.1	523.9171826	0.121652121	0.042650615	2.852295	0.004340485	0.006515258	cyan	0.24831542	0.001713953		AT4G27060.1	CN,SPR2,TOR1	ARM repeat superfamily protein	microtubule-associated protein TORTIFOLIA protein
Sapur.008G158600.1	1217.597998	0.068292999	0.023959293	2.850376	0.004366754	0.006553797	cyan	0.229882096	0.003776047	GO:0008565,GO:0006606,GO:0005737,GO:0005634,GO:0005515	AT3G06720.2	AIMP ALPHA.AT-IMP.ATKAP	importin alpha isoform 1	importin subunit alpha
Sapur.007G052700.2	32.74294541	0.261980471	0.09221794	2.840884	0.004498867	0.006744426	cyan	0.22769913	0.004130285	GO:0008270,GO:0006281,GO:0004518	AT4G36050.2		endonuclease/exonuclease/phosphatase family protein	NA
Sapur.008G122400.2	1.757306547	0.575285182	0.20534306	2.801581	0.00508529	0.007587444	cyan	0.670117207	8.17E-22		AT4G14950.1		SNARE associated Golgi protein family	SNARE associated protein
Sapur.005G091100.1	2975.197079	0.16497933	0.060614946	2.72176	0.00649353	0.009589097	cyan	0.227275816	0.004202337	GO:0005515,GO:0016773	AT2G17930.1		Phosphatidylinositol 3- and 4-kinase family protein with FAT domain	NA
Sapur.006G072500.1	1080.08599	-0.132138227	0.048652508	-2.71596	0.006608406	0.009751335	cyan	-0.228669146	0.003969347		AT5G02120.1	OHP	one helix protein	one helix protein
Sapur.018G040900.1	69.34756951	0.280265409	0.104538291	2.680983	0.007340619	0.010788945	cyan	0.237446205	0.002750192					NA
Sapur.002G174800.1	229.6079623	0.107959534	0.041210709	2.619696	0.008800815	0.012841495	cyan	0.181657527	0.022789093	GO:0008601,GO:0007165,GO:0000159	AT3G21650.1		Protein phosphatase 2A regulatory B subunit family protein	serine/threonine protein phosphatase 2A 59 kDa regulatory subunit B' protein
Sapur.016G159900.1	73.2574685	0.291140117	0.111579039	2.609272	0.009073496	0.013223619	cyan	0.217359503	0.006247219	GO:0008270,GO:0005515,GO:0006468,GO:0005524,GO:0004672	AT5G13530.1	KEG	protein kinases;ubiquitin-protein ligases	E3 ubiquitin-protein ligase KEG
Sapur.018G028800.1	775.6942882	0.133323808	0.051824137	2.57262	0.010093198	0.014656763	cyan	0.21997565	0.005635766		AT4G32620.1		Enhancer of polycomb-like transcription factor protein	enhancer of polycomb-like transcription factor protein, putative
Sapur.008G003100.2	1.305702074	-0.574286903	0.223232701	-2.57259	0.010093981	0.014657256	cyan	0.553737957	5.39E-14		AT1G51540.1		Galactose oxidase/kelch repeat superfamily protein	kelch motif protein
Sapur.003G054400.1	1680.718259	0.11752488	0.046487869	2.528076	0.011468942	0.016563634	cyan	0.223054754	0.004985012	GO:0008270,GO:0005737,GO:0005524,GO:0004386,GO:0003677,GO:0000184	AT5G47010.1	ATUPF1,LBA1,UPF1	RNA helicase, putative	regulator of nonsense transcripts-like protein
Sapur.004G090000.1	2760.981866	0.109622572	0.043945307	2.494523	0.012612662	0.018129913	cyan	0.207831681	0.009004129	GO:0006508	AT1G49630.3	ATPREP2,PREP2	presequence protease 2	presequence protease
Sapur.005G107900.1	2671.070655	0.152336763	0.06161784	2.472283	0.013425304	0.019247009	cyan	0.214226637	0.007056762		AT3G50370.1			NA
Sapur.002G083500.1	1001.701981	0.142149574	0.057782479	2.460081	0.013890569	0.019881282	cyan	0.197871841	0.012986958		AT4G24610.1			plant/MNA5-17 protein
Sapur.002G067500.1	564.8901961	0.12178589	0.050254762	2.42337	0.01537725	0.021913359	cyan	0.203612481	0.010536044	GO:0007049,GO:0006281,GO:0005634	AT1G77620.1		P-loop containing nucleoside triphosphate hydrolases superfamily protein	P-loop nucleoside triphosphate hydrolase-like protein, putative
Sapur.002G167900.2	13.33212895	0.203068689	0.084047625	2.416115	0.015687126	0.022339548	cyan	0.401783706	1.83E-07	GO:0055114,GO:0016491,GO:0008152	AT1G23800.1	ALDH2B,ALDH2B7	aldehyde dehydrogenase 2B7	aldehyde dehydrogenase (NAD) family protein
Sapur.002G174000.1	986.0408949	-0.113418034	0.04711134	-2.40745	0.016064513	0.02284255	cyan	-0.200808644	0.011676941		AT5G55160.1	ATSUMO2.SUM2.SUMO 2.S	small ubiquitin-like modifier 2	small ubiquitin-related modifier
Sapur.006G051400.1	137.6548727	0.163647152	0.069461774	2.355931	0.018476347	0.026115932	cyan	0.19194482	0.016027895	GO:0006468,GO:0004672	AT2G11520.1	CRCK3	calmodulin-binding receptor-like cytoplasmic kinase 3	calmodulin-binding receptor-like cytoplasmic kinase
Sapur.005G133800.2	3.582146054	-0.327613861	0.140542444	-2.33107	0.019749824	0.027846941	cyan	-0.270134513	0.000622618	GO:0016747,GO:0006355	AT5G09740.1	HAM2	histone acetyltransferase of the MYST family 2	MYST histone acetyltransferase, putative
Sapur.016G002600.1	21.97454644	0.248705583	0.107259046	2.318738	0.020409268	0.028732649	cyan	0.195310286	0.014232933	GO:0005815,GO:0000922,GO:0000226	AT1G80260.1	emb1427	Spc97 / Spc98 family of spindle pole body (SBP) component	gamma-tubulin complex protein
Sapur.006G058900.2	25.95051476	-0.277675604	0.120965052	-2.2955	0.021704332	0.030485797	cyan	0.6813658	9.19E-23	GO:0005516,GO:0046872,GO:0000166	AT5G57110.2	ACA8,AT-ACA8	autoinhibited Ca2+ -ATPase, isoform 8	NA
Sapur.017G016300.1	25.50774932	0.25305975	0.110705211	2.285888	0.022260792	0.03121702	cyan	0.201988324	0.011184326	GO:0003676	AT2G43410.2	FPA	RNA binding	flowering time control protein FPA, putative
Sapur.006G022400.1	2379.140332	0.109356156	0.049541812	2.207351	0.027289566	0.037964798	cyan	0.219510481	0.005740413	GO:0003677,GO:0032784,GO:0006357	AT1G65440.2	GTB1	global transcription factor group B1	global transcription factor group protein, putative
Sapur.010G205100.1	342.1767791	0.132659275	0.060247996	2.201887	0.027673297	0.038477598	cyan	0.205154517	0.009951409		AT1G16800.1		P-loop containing nucleoside triphosphate hydrolases superfamily protein	splicing endonuclease positive effector sen1, putative
Sapur.019G002100.1	302.586138	0.268463747	0.121931101	2.201766	0.027681845	0.038486247	cyan	0.181875678	0.022623537	GO:0046872,GO:0000166,GO:0016887,GO:0016021,GO:0006812	AT5G23630.1	MIA,PDR2	phosphate deficiency response 2	NA
Sapur.011G098100.2	6.318691746	0.305825545	0.139691859	2.189287	0.028576001	0.039679359	cyan	0.176593379	0.026935968	GO:0046983	AT5G54680.1	bHLH105,ILR3	basic helix-loop-helix (bHLH) DNA-binding superfamily protein	BHLH transcription factor
Sapur.010G017600.1	53.24254607	0.203514272	0.093913995	2.167028	0.030232706	0.041862033	cyan	0.207470815	0.009126981					NA
Sapur.002G028600.2	51.18030539	0.204978356	0.094812966	2.161923	0.030624093	0.042370267	cyan	0.190767757	0.01670062		AT1G08620.2	PKDM7D	Transcription factor jumonji (jmj) family protein / zinc finger (C5HC2 type) family protein	transcription factor jumonji family protein
Sapur.014G032300.1	203.199916	0.124001596	0.060021236	2.065962	0.038832061	0.053137426	cyan	0.150511285	0.059895288		AT1G19360.1		Nucleotide-diphospho-sugar transferase family protein	NA
Sapur.019G016600.2	5.654875107	-0.232427682	0.117841647	-1.97237	0.048567041	0.06573793	cyan	-0.300875537	0.000128695		AT5G13340.1			arginine and glutamate-rich 1 protein
Sapur.001G115600.2	1.103428579	-0.502749363	0.258165306	-1.94739	0.051487612	0.069528878	cyan	0.399137153	2.24E-07	GO:0016887,GO:0005524,GO:0055085,GO:0042626,GO:0016021,GO:0006810	AT4G25960.1	PGP2	P-glycoprotein 2	multidrug/pheromone exporter, MDR family, ABC transporter family protein
Sapur.018G083400.2	6.776163123	-0.23235349	0.119668268	-1.94165	0.052179892	0.070411936	cyan	-0.24315565	0.002150826	GO:0005739,GO:0004129	AT5G58005.1		Cytochrome c oxidase, subunit Vib family protein	NA
Sapur.016G292900.4	11.45095158	0.232524974	0.124021904	1.87487	0.06081058	0.081413905	cyan	0.296496333	0.000162892		AT5G09810.1	ACT7	actin 7	actin
Sapur.006G161200.1	868.388099	0.084103138	0.047568825	1.768031	0.077055787	0.101811406	cyan	0.140176756	0.079940704	GO:0005634,GO:0003676	AT3G11960.1		Cleavage and polyadenylation specificity factor (CPSF) A subunit protein	cleavage and polyadenylation specificity factor (CPSF) A subunit protein
Sapur.005G180300.1	6.188441508	0.249839857	0.145627087	1.715614	0.08623273	0.113221966	cyan	0.437740558	9.87E-09	GO:0008168	AT1G04430.2		S-adenosyl-L-methionine-dependent methyltransferases superfamily protein	S-adenosyl-L-methionine-dependent methyltransferase, putative
Sapur.006G042400.2	0.624580772	0.486055412	0.293516073	1.655975	0.09772679	0.127357888	cyan	0.616276154	8.58E-18		AT3G57630.1		exostosin family protein	exostosin family protein
Sapur.T090100.1	134.5707301	0.156235345	0.099422753	1.571424	0.116084092	0.149858129	cyan	0.135362616	0.090965988		AT3G27700.2		zinc finger (CCCH-type) family protein / RNA recognition motif (RRM)-containing protein	NA
Sapur.010G022400.2	1.081593055	-0.382523827	0.246689882	-1.55063	0.120991258	0.155815936	cyan	0.608135873	2.99E-17		AT3G26370.1		O-fucosyltransferase family protein	auxin-independent growth promoter-like protein
Sapur.014G129900.3	9.516556312	0.159596947	0.103763447	1.538084	0.124027962	0.159534119	cyan	0.215048164	0.006835921	GO:0046872	AT1G04990.1		Zinc finger C-x8-C-x5-C-x3-H type family protein	NA
Sapur.013G102000.2	3.546418501	0.376298697	0.247685898	1.519258	0.128697653	0.165219353	cyan	0.389466078	4.62E-07	GO:0042578	AT1G22620.1	ATSAC1	Phosphoinositide phosphatase family protein	phosphoinositide phosphatase SAC1
Sapur.004G046200.2	9.768039442	-0.147145285	0.098935894	-1.48728	0.136941119	0.175210467	cyan	0.334152435	1.89E-05	GO:0051603,GO:0005839,GO:0004298	AT5G40580.1	PBB2	20S proteasome beta subunit PBB2	proteasome subunit beta type protein
Sapur.006G219500.2	7.18768074	0.178484305	0.120658816	1.479248	0.139074061	0.177774425	cyan	0.237220213	0.002776759	GO:0003676,GO:0006396,GO:0003723	AT5G25060.1		RNA recognition motif (RRM)-containing protein	RNA recognition motif (RRM) in U2-associated SR140-like protein
Sapur.012G023500.1	226.909818	-0.075389059	0.05152146	-1.46326	0.143397481	0.182919172	cyan	-0.182655312	0.022040299		AT1G55805.1		BolA-like family protein	UV-induced protein uvi31, putative
Sapur.003G140800.2	0.915597663	-0.393072516	0.276442097	-1.4219	0.155055811	0.19675859	cyan	0.690133954	1.56E-23		AT1G79690.1	atnudt3,NUDT3	nudix hydrolase homolog 3	nudix hydrolase
Sapur.002G130400.4	1.297865169	-0.314015599	0.222803951	-1.40938	0.158722613	0.201095257	cyan	0.463796348	9.51E-10	GO:0016567,GO:0004842,GO:0006468,GO:0005524,GO:0004672	AT2G45910.1		U-box domain-containing protein kinase family protein	receptor kinase, putative
Sapur.002G199200.1	4238.052828	0.072455963	0.052473953	1.380799	0.167340883	0.211221324	cyan	0.122278399	0.1271017	GO:0016887,GO:0005524,GO:0055085,GO:0042626,GO:0016021,GO:0006810	AT1G04120.1	ABCC5.ATABCC5.ATMRP5.N	multidrug resistance-associated protein 5	multidrug resistance protein ABC transporter family protein
Sapur.003G091200.2	48.40554134	0.100491758	0.077119024	1.303074	0.192549663	0.240588876	cyan	-0.332909265	2.04E-05	GO:0042398,GO:0004357	AT4G23100.3	ATECS1.CAD2.GSH1.GSHA.P	glutamate-cysteine ligase	glutamate-cysteine ligase
Sapur.017G030600.1	1602.331955	0.072134658	0.058748436	1.227857	0.219500785	0.271962574	cyan	0.12930471	0.106523175	GO:0005515	AT4G15180.1	ATXR3,SDG2	SET domain protein 2	SET domain protein
Sapur.001G132800.1	634.8407389	0.060247666	0.04914574	1.225898	0.220237045	0.272782834	cyan	0.13071521	0.102726323	GO:0003712,GO:0000124	AT1G72390.1			Spt20 family protein
Sapur.012G014500.2	8.433715622	-0.122519952	0.106702595	-1.14824	0.250870414	0.307705844	cyan	-0.338502977	1.45E-05		AT5G53440.1			hypothetical protein
Sapur.017G093000.2	0.309188101	-0.429296372	0.400937414	-1.07073	0.284290115	0.345341436	cyan	-0.274129177	0.000512425					NA
Sapur.016G261400.2	0.839670585	0.356725023	0.337743504	1.056201	0.2908764	0.352681503	cyan	0.505129741	1.51E-11		AT4G27430.1	CIP7	COP1-interacting protein 7	Cop1-interacting protein 7 (CIP7), putative
Sapur.007G076800.2	0.627339139	0.320254302	0.316983246	1.010319	0.312342321	0.376527552	cyan	0.300729414	0.000129719		AT5G64930.1	CPR5,HYS1	CPR5 protein, putative	CPR-5, putative
Sapur.008G020000.2	4.102764044	-0.127942599	0.131744541	-0.97114	0.331477791	0.397739677	cyan	0.333510745	1.97E-05	GO:0016021,GO:0015012,GO:0006024	AT5G04500.1		glycosyltransferase family protein 47	glycosyltransferase family protein
Sapur.006G062000.2	0.279598408	0.438414782	0.453248387	0.967273	0.333407738	0.399852329	cyan	0.602262511	7.20E-17	GO:0016020,GO:0008375	AT2G19160.1		Core-2/I-branching beta-1,6-N-acetylglucosaminyltransferase family protein	core-2/I-branching beta-1%2C6-N-acetylglucosaminyltransferase family protein
Sapur.001G123800.4	1.488421218	-0.243465539	0.253461529	-0.96056	0.336772392	0.40347787	cyan	0.286317362	0.000277673		AT4G17140.3		pleckstrin homology (PH) domain-containing protein	vacuolar protein sorting-associated protein, putative
Sapur.006G112200.2	0.283215013	0.515282705	0.542456882	0.949905	0.342160339	0.409265887	cyan	0.692027948	1.06E-23	GO:0042578	AT3G43220.1		Phosphoinositide phosphatase family protein	phosphoinositide phosphatase
Sapur.008G039600.2	0.315807503	0.418076792	0.442800928	0.944164	0.345085737	0.412287891	cyan	0.429272399	2.03E-08	GO:0016624,GO:0008152,GO:0055114,GO:0030976,GO:0006099,GO:0004591	AT3G55410.1		2-oxoglutarate dehydrogenase, E1 component	2-oxoglutarate dehydrogenase, E1 component
Sapur.008G108200.1	1362.329907	0.046395646	0.05188257	0.894243	0.371191676	0.440312513	cyan	0.086152386	0.283335671		AT1G23230.2			mediator of RNA polymerase II transcription subunit 23
Sapur.006G187800.2	2.195039708	-0.14326989	0.182494561	-0.78506	0.432416111	0.504915462	cyan	0.75442958	3.85E-30		AT5G11910.1		alpha/beta-Hydrolases superfamily protein	valacyclovir hydrolase, putative
Sapur.005G058200.2	29.44826009	-0.071158492	0.091924145	-0.7741	0.438871583	0.511838878	cyan	0.243836259	0.002087923		AT2G22310.1	ATUBP4,UBP4	ubiquitin-specific protease 4	ubiquitin carboxyl-terminal hydrolase
Sapur.15WG078000.2	0.852030698	0.228433198	0.299556526	0.762571	0.445719128	0.518910009	cyan	0.48048274	1.91E-10	GO:0005515	AT1G18610.1		Galactose oxidase/kelch repeat superfamily protein	NA
Sapur.017G075000.2	2.619503581	-0.14892478	0.198934923	-0.74861	0.454091978	0.527728947	cyan	-0.346168684	8.96E-06		AT3G49645.1			FAD+binding+protein
Sapur.017G073200.2	0.235543278	0.320362142	0.455161718	0.703842	0.48153089	0.556080749	cyan	0.361919071	3.20E-06		AT1G69360.1		Plant protein of unknown function (DUF863)	DUF863 family protein
Sapur.003G094500.2	0.211317412	0.319529447	0.522725162	0.611276	0.541016748	0.616446341	cyan	0.21988511	0.005656		AT4G23470.1		PLAC8 family protein	NA
Sapur.006G211900.2	0.4179467	-0.227943882	0.397709912	-0.57314	0.566549186	0.641977792	cyan	0.595451806	1.95E-16		AT4G32160.1		Phox (PX) domain-containing protein	phox (PX) domain protein
Sapur.014G022500.2	0.182108688	0.355966261	0.645571962	0.551397	0.58136175	0.65613235	cyan	0.738277274	2.68E-28		AT4G33380.1			plant/F17M5-140 protein
Sapur.012G043300.2	0.302607758	0.196774457	0.428401865	0.459322	0.646002859	0.718398795	cyan	0.300213785	0.000133393	GO:0005515	AT1G49040.1	SCD1	stomatal cytokinesis defective / SCD1 protein (SCD1)	stomatal cytokinesis defective protein, putative
Sapur.014G043000.2	0.650443573	0.129619322	0.329325284	0.393591	0.693883354	0.763253228	cyan	0.305585312	9.95E-05		AT2G44980.2		SNF2 domain-containing protein / helicase domain-containing protein	SNF2 and helicase domain protein
Sapur.012G016400.2	0.112299148	0.319365377	0.859592109	0.371531	0.710241844	0.777986976	cyan	0.49502719	4.38E-11	GO:0055114.GO:0016491.GO:0008152.GO:0015930.GO:0006807.GO:0016638.GO:000653	AT5G53460.2	GLT1	NADH-dependent glutamate synthase 1	glutamate synthase (NADH)
Sapur.016G027800.2	0.349597994	0.137481642	0.373868443	0.367727	0.713076564	0.780471715	cyan	0.545365183	1.52E-13	GO:0006468,GO:0005524,GO:0004672,GO:0003824	AT2G40860.1		protein kinase family protein / protein phosphatase 2C (PP2C) family protein	kinase and PP2C-like domain protein
Sapur.001G071700.2	0.122871077	-0.318960956	0.873325309	-0.36523	0.714942843	0.78228139	cyan	0.322309449	3.84E-05	GO:0005515	AT4G10840.1		Tetratricopeptide repeat (TPR)-like superfamily protein	NA
Sapur.008G160200.2	0.656688478	-0.112694001	0.309862868	-0.36369	0.716089572	0.783151835	cyan	0.403920825	1.55E-07	GO:0006629	AT4G16070.1		Mono-/di-acylglycerol lipase, N-terminal;Lipase, class 3	lipase class 3 family protein
Sapur.006G074000.2	1.683871123	-0.065009172	0.199869258	-0.32526	0.744985449	0.80894477	cyan	0.489906282	7.42E-11	GO:0016779,GO:0009058	AT2G39770.1	CYT1.EMB101.GMP1.SOZ1.	Glucose-1-phosphate adenylyltransferase family protein	mannose-1-phosphate guanyltransferase
Sapur.017G116800.2	0.095922574	0.264185222	1.277958653	0.206724	0.836225109	0.885113666	cyan	0.251875844	0.001461399		AT5G52530.3		dentin sialophosphoprotein-related	hypothetical protein
Sapur.001G169100.2	0.242721143	-0.102174693	0.505707988	-0.20204	0.83988321	0.887914407	cyan	-0.40613422	1.31E-07		AT5G09390.1		CD2-binding protein-related	CD2 antigen cytoplasmic tail-binding protein, putative
Sapur.003G142600.2	0.727646555	-0.064098104	0.331121182	-0.19358	0.846505551	0.893769341	cyan	0.492133027	5.91E-11	GO:0031047	AT5G23570.1	ATSGS3,SGS3	XS domain-containing protein / XS zinc finger domain-containing protein-related	NA
Sapur.001G037400.2	0.580277985	-0.064933384	0.434415401	-0.14947	0.881180394	0.919577205	cyan	-0.471085216	4.77E-10	GO:0006412,GO:0005840,GO:0005622,GO:0003735	AT2G32060.3		Ribosomal protein L7Ae/L30e/S12e/Gadd45 family protein	40S ribosomal protein S12
Sapur.006G200400.2	0.104697209	-0.130034807	0.910980889	-0.14274	0.886494315	0.923668864	cyan	0.528290814	1.15E-12	GO:0016021	AT2G25737.1		Sulfite exporter TauE/SafE family protein	sulfite exporter TauE/SafE
Sapur.005G074500.2	0.059486888	0.134392999	1.334749658	0.100688	0.919798301	0.947589366	cyan	0.460371753	1.31E-09	GO:0006468,GO:0005524,GO:0004672	AT3G13530.1	MAP3KE1,MAPKKK7	mitogen-activated protein kinase kinase kinase 7	MAP3K epsilon kinase
Sapur.002G188300.2	0.086324095	-0.029444435	1.133899199	-0.02597	0.979283326	0.986621687	cyan	0.680703424	1.05E-22		AT3G24840.1		Sec14p-like phosphatidylinositol transfer family protein	phosphatidylinositol/phosphatidylcholine transfer-like protein
Sapur.019G080100.2	0.0867884	-0.017917392	1.075421195	-0.01666	0.986707209	0.99156185	cyan	0.672652464	5.04E-22		AT1G35620.1	ATPDI8.ATPDIL5-2.PDI8.PDI	PDI-like 5-2	protein disulfide isomerase-like protein, putative
Sapur.008G079900.2	0.013020124	0.199900828	2.904821116	0.068817	0.945135354	NA	cyan	0.217839059	0.006130894	GO:0004842	AT3G17205.3	UPL6	ubiquitin protein ligase 6	E3 ubiquitin-protein ligase UPL6
Sapur.009G030700.2	0.003634244	0.128661719	2.904823276	0.044292	0.964671296	NA	cyan	0.357379143	4.33E-06	GO:0008184,GO:0005975	AT3G46970.1	ATPHS2,PHS2	alpha-glucan phosphorylase 2	alpha-glucan phosphorylase, H
Sapur.005G139500.1	0.00333834	0.128661719	2.904823276	0.044292	0.964671296	NA	cyan	0.68877245	2.07E-23		AT1G77580.2		Plant protein of unknown function (DUF869)	DNA double-strand break repair rad50 ATPase, putative
Sapur.014G123700.2	0.019342238	0.053717484	2.904823276	0.018493	0.98524595	NA	cyan	0.580764469	1.55E-15		AT2G43160.2		ENTH/VHS family protein	clathrin interactor epsin 2, putative
Sapur.013G056900.1	0.025378014	-0.021284163	2.404571151	-0.00885	0.992937583	NA	cyan	0.790053189	9.28E-35	GO:0008652,GO:0008270,GO:0003871,GO:0009086	AT5G17920.1	ATCIMS,ATMETS,ATMS1	Cobalamin-independent synthase family protein	5-methyltetrahydropteroyltriglutamate-homocysteine methyltransferase
Sapur.001G016400.1	4385.731019	3.801111328	0.071898926	52.86743	0	0	cyan	0.877739819	2.15E-51		AT5G13490.1	AAC2	ADP/ATP carrier 2	ADP%2CATP carrier protein, putative
Sapur.001G042000.1	6099.013592	1.797618009	0.045159431	39.80604	0	0	cyan	0.922237267	7.42E-66	GO:0016747,GO:0008152	AT2G33150.1	KAT2,PED1,PKT3	peroxisomal 3-ketoacyl-CoA thiolase 3	peroxisomal 3-ketoacyl-CoA thiolase
Sapur.001G042500.1	812.8337893	12.33856514	0.249727917	49.40803	0	0	cyan	0.891110099	4.65E-55		AT1G04670.1			hypothetical protein
Sapur.001G062500.1	77.65224213	4.124196683	0.103728204	39.75965	0	0	cyan	0.897088382	7.42E-57					hypothetical protein
Sapur.001G064700.1	146.3839176	7.579661876	0.158173628	47.91988	0	0	cyan	0.894405823	4.90E-56		AT5G61620.1		myb-like transcription factor family protein	MYB-like DNA-binding domain protein
Sapur.001G086600.1	592.8785346	9.76423202	0.167992453	58.12304	0	0	cyan	0.890106343	9.10E-55	GO:0005622,GO:0003779,GO:0030042,GO:0015629	AT5G59890.1	ADF4,ATADF4	actin depolymerizing factor 4	actin-depolymerizing factor
Sapur.001G126500.1	1755.025051	6.73329466	0.101429984	66.38367	0	0	cyan	0.839955128	5.39E-43	GO:0055085,GO:0016021	AT4G17550.1		Major facilitator superfamily protein	glycerol-3-phosphate transporter, putative
Sapur.001G129800.1	561.3559278	2.871151646	0.061215151	46.90263	0	0	cyan	0.919551875	9.28E-65	GO:0055114,GO:0003995,GO:0006635,GO:0005777,GO:0003997	AT4G16760.1	ACX1,ATACX1	acyl-CoA oxidase 1	acyl-coenzyme A oxidase
Sapur.001G145100.1	7308.696374	6.29017904	0.105145406	59.82362	0	0	cyan	0.883638966	5.91E-53		AT1G52690.2		Late embryogenesis abundant protein (LEA) family protein	late embryogenesis abundant protein
Sapur.002G028400.1	2423.087255	1.91685388	0.050080371	38.27555	0	0	cyan	0.902627696	1.27E-58	GO:0008168	AT1G04430.1		S-adenosyl-L-methionine-dependent methyltransferases superfamily protein	methyltransferase PMT8, putative
Sapur.002G050000.1	157.2787711	4.949457868	0.123982609	39.92058	0	0	cyan	0.830538857	3.08E-41	GO:0003723,GO:0003676	AT1G22760.1	PAB3	poly(A) binding protein 3	polyadenylate-binding protein, animal type 1, 2, 3, 4 family protein
Sapur.002G098800.1	247.0877186	7.145558395	0.13747291	51.97794	0	0	cyan	0.855484226	3.73E-46	GO:0055114,GO:0020037,GO:0016705,GO:0005506	AT4G37370.1	CYP81D8	cytochrome P450, family 81, subfamily D, polypeptide 8	NA
Sapur.002G113700.1	97.56371761	3.92616734	0.099331629	39.52585	0	0	cyan	0.861000399	2.29E-47	GO:0006457,GO:0003755,GO:0000413	AT4G17070.1		peptidyl-prolyl cis-trans isomerases	peptidyl-prolyl cis-trans isomerase, cyclophilin-type protein
Sapur.002G115100.1	91.66008193	9.337252079	0.242409225	38.51855	0	0	cyan	0.938461248	1.86E-73		AT3G60460.1	DUO1	myb-like HTH transcriptional regulator family protein	R2r3-Myb transcription factor, putative
Sapur.002G117300.1	173.8076013	5.083790087	0.105164351	48.34138	0	0	cyan	0.868277697	4.75E-49		AT1G02040.1		C2H2-type zinc finger family protein	zinc finger protein
Sapur.002G139400.1	1420.988428	3.453719497	0.076305431	45.26178	0	0	cyan	0.865762705	1.86E-48	GO:0016747,GO:0008610,GO:0016020,GO:0006633	AT1G01120.1	KCS1	3-ketoacyl-CoA synthase 1	3-ketoacyl-CoA synthase
Sapur.002G144200.1	381.4778751	4.416575068	0.091080387	48.49096	0	0	cyan	0.894273799	5.37E-56	GO:0005525,GO:0003924	AT3G61760.1	ADL1B,DL1B	DYNAMIN-like 1B	NA
Sapur.002G155500.1	219.55688	8.535708073	0.169388302	50.39137	0	0	cyan	0.91260317	4.34E-62	GO:0016021	AT2G47770.1	ATTSPO,TSPO	TSPO(outer membrane tryptophan-rich sensory protein)-related	peripheral-type benzodiazepine receptor, putative
Sapur.002G171900.1	811.506089	9.310812606	0.127995056	72.74353	0	0	cyan	0.926323563	1.33E-67		AT5G47540.1		Mo25 family protein	calcium-binding protein, putative
Sapur.003G033300.1	3598.16539	9.634820531	0.10908031	88.32777	0	0	cyan	0.889672266	1.21E-54		AT1G52680.1		late embryogenesis abundant protein-related / LEA protein-related	hypothetical protein
Sapur.003G034100.1	298.8932741	7.306891946	0.124579931	58.65224	0	0	cyan	0.899988418	9.08E-58		AT5G11280.1			transmembrane protein, putative
Sapur.003G062700.1	690.3810917	2.749541222	0.058118601	47.30914	0	0	cyan	0.922174393	7.88E-66		AT1G32250.1		Calcium-binding EF-hand family protein	calcium-binding protein CML11, putative
Sapur.003G072800.1	997.8149008	6.54876391	0.079892278	81.96992	0	0	cyan	0.912220106	5.99E-62		AT4G39730.1		Lipase/lipooxygenase, PLAT/LH2 family protein	PLAT/LH2 domain protein
Sapur.003G074200.1	124.0519239	7.547507668	0.160013651	47.1679	0	0	cyan	0.920363547	4.37E-65	GO:0016020,GO:0006810,GO:0005215	AT3G47440.1	TIP5;1	tonoplast intrinsic protein 5;1	aquaporin, MIP family%2C TIP subfamily protein
Sapur.003G100700.1	578.0170475	4.572061295	0.094083666	48.5957	0	0	cyan	0.869180688	2.89E-49		AT5G41780.1		myosin heavy chain-related	coiled-coil protein, putative
Sapur.003G103500.1	360.0865188	2.857630052	0.068567297	41.67628	0	0	cyan	0.906708829	5.40E-60		AT4G23885.1			NA
Sapur.003G132100.1	953.1116876	8.042066841	0.106918157	75.21704	0	0	cyan	0.886643475	8.77E-54					plant self-incompatibility protein S1
Sapur.003G132500.1	312.7876334	11.59793439	0.254698607	45.53592	0	0	cyan	0.864303067	4.05E-48		AT1G04670.1			NA
Sapur.003G149900.1	108.9446132	9.740331651	0.253206157	38.46799	0	0	cyan	0.867908943	5.81E-49		AT5G06990.1		Protein of unknown function, DUF617	plant-specific domain TIGR01570 family protein, putative
Sapur.003G150300.1	411.4198144	9.900554163	0.209265517	47.31097	0	0	cyan	0.835485381	3.80E-42	GO:0055085,GO:0016021,GO:0015299,GO:0006812	AT1G79400.1	ATCHX2,CHX2	cation/H+ exchanger 2	NA

Sapur.004G003900.1	127.5583497	8.168016353	0.19024739	42.93366	0	0	cyan	0.911881911	7.97E-62		AT2G32580.1		Protein of unknown function (DUF1068)	plant/T7A14-6 protein, putative
Sapur.004G036000.1	656.7652369	9.560016266	0.170061229	56.21514	0	0	cyan	0.86389027	5.04E-48		AT1G53210.1		sodium/calcium exchanger family protein / calcium-binding EF hand family protein	sodium/calcium exchanger family protein, putative
Sapur.004G062900.1	419.1354289	5.96829391	0.113470125	52.59793	0	0	cyan	0.806691131	3.10E-37		AT3G03380.1	DegP7	DegP protease 7	NA
Sapur.004G077900.1	12534.35327	12.05499031	0.217765187	55.35775	0	0	cyan	0.851562112	2.53E-45		AT5G38760.1		Late embryogenesis abundant protein (LEA) family protein	pollen coat-like protein
Sapur.004G078000.1	7282.246813	12.1264568	0.146299859	82.88769	0	0	cyan	0.902414437	1.49E-58		AT5G38760.1		Late embryogenesis abundant protein (LEA) family protein	pollen coat-like protein
Sapur.004G079200.1	1484.984006	12.61856308	0.250732169	50.32686	0	0	cyan	0.83882591	8.88E-43		AT5G38760.1		Late embryogenesis abundant protein (LEA) family protein	NA
Sapur.004G108000.1	169.8786514	8.544866611	0.196141366	43.56484	0	0	cyan	0.889692707	1.20E-54		AT3G19090.1		RNA-binding protein	NA
Sapur.004G117400.1	75.14517446	5.787465869	0.144535266	40.04189	0	0	cyan	0.849965665	5.44E-45	GO:0006950	AT2G21620.1	RD2	Adenine nucleotide alpha hydrolases-like superfamily protein	universal stress protein, putative
Sapur.004G168600.1	88.10441808	3.056529087	0.078318181	39.02707	0	0	cyan	0.909096834	7.96E-61		AT2G38500.1		2-oxoglutarate (2OG) and Fe(II)-dependent oxygenase superfamily protein	transmembrane protein, putative
Sapur.004G173400.1	1155.358865	5.264368506	0.093056736	56.5716	0	0	cyan	0.873772034	2.18E-50	GO:0046961,GO:0033178,GO:0015991,GO:0016747	AT4G11150.1	emb2448,TUF,TUFF,VHA-E1	vacuolar ATP synthase subunit E1	archaeal/vacuolar-type H+-ATPase subunit E
Sapur.005G028300.1	2549.746144	11.78661565	0.172992646	68.13362	0	0	cyan	0.9049231	2.19E-59		AT1G54860.1		Glycoprotein membrane precursor GPI-anchored	GPI-anchored protein, putative
Sapur.005G033700.1	455.0431929	8.278662211	0.134833968	61.39894	0	0	cyan	0.881101901	2.84E-52		AT4G08630.1			NA
Sapur.005G049500.1	404.6263154	11.44399871	0.253899056	45.07303	0	0	cyan	0.876420732	4.69E-51		AT2G13570.1	NF-YB7	nuclear factor Y, subunit B7	nuclear transcription factor Y subunit B-3
Sapur.005G058600.1	416.5015784	3.97269152	0.081980206	48.45915	0	0	cyan	0.900013434	8.92E-58	GO:0005525	AT5G10260.1	AtRABH1e,RABH1e	RAB GTPase homolog H1E	rab6/RabH-family small GTPase
Sapur.005G082100.1	171.6768056	7.444991668	0.158326201	47.02312	0	0	cyan	0.87233402	4.96E-50	GO:0003824,GO:0006470,GO:0004722	AT3G51470.1		Protein phosphatase 2C family protein	protein phosphatase 2C, putative
Sapur.005G086300.1	159.9287207	3.313956001	0.081277544	40.77333	0	0	cyan	0.873672707	2.31E-50		AT5G16110.1			hypothetical protein
Sapur.005G094600.1	2701.836992	7.182718879	0.093209331	77.06008	0	0	cyan	0.850723909	3.79E-45	GO:0008168	AT3G51070.1		S-adenosyl-L-methionine-dependent methyltransferases superfamily protein	S-adenosyl-L-methionine-dependent methyltransferase, putative
Sapur.005G099100.1	107.177532	4.391764276	0.104365188	42.08074	0	0	cyan	0.872138494	5.54E-50		AT2G18460.1	LCV3	like COV 3	NA
Sapur.005G122800.1	1869.068975	9.469031009	0.130672489	72.46385	0	0	cyan	0.916002664	2.29E-63		AT2G33690.1		Late embryogenesis abundant protein, group 6	late embryogenesis abundant protein
Sapur.005G138400.1	120.1857835	3.071607499	0.071057902	43.22682	0	0	cyan	0.905951574	9.81E-60	GO:0046872	AT1G66810.1		Zinc finger C-x8-C-x5-C-x3-H type family protein	zinc finger C-x8-C-x5-C-x3-H type protein
Sapur.005G155900.1	365.75567	3.472358307	0.090899523	38.19996	0	0	cyan	0.896298093	1.30E-56	GO:0003723,GO:0003676	AT1G22760.1	PAB3	poly(A) binding protein 3	polyadenylate-binding protein, animal type 1, 2, 3, 4 family protein
Sapur.005G156600.1	1475.116142	11.8359275	0.212493285	55.70024	0	0	cyan	0.8493335	7.34E-45		AT1G22730.1		MA3 domain-containing protein	NA
Sapur.005G175100.1	304.6871324	2.742665829	0.072910937	37.61666	0	0	cyan	0.878775889	1.16E-51	GO:0016301,GO:0005524	AT2G30630.1		P-loop containing nucleoside triphosphate hydrolases superfamily protein	nucleoside/nucleotide kinase domain protein
Sapur.006G029500.1	119.6282099	3.388012992	0.088891042	38.11422	0	0	cyan	0.911021686	1.64E-61	GO:0008138,GO:0006470	AT4G18593.1		dual specificity protein phosphatase-related	dual specificity phosphatase domain protein
Sapur.006G044900.1	203.1815462	5.771866306	0.111361081	51.83019	0	0	cyan	0.907015467	4.24E-60	GO:0005525	AT3G12160.1	ATRABA4D,RABA4D	RAB GTPase homolog A4D	Ras small GTPase family Ras protein
Sapur.006G093800.1	4784.960046	11.86415242	0.152410143	77.84359	0	0	cyan	0.834151888	6.72E-42	GO:0006508,GO:0004252	AT5G03620.1		Subtilisin-like serine endopeptidase family protein	xylem serine proteinase
Sapur.006G150500.1	569.7089134	5.405439607	0.093842473	57.60121	0	0	cyan	0.892481938	1.84E-55		AT4G31830.1			transmembrane protein, putative
Sapur.006G162900.1	185.2185041	3.724283534	0.091362917	40.76362	0	0	cyan	0.89768878	4.83E-57	GO:0006468,GO:0004672	AT2G38910.1	CPK20	calcium-dependent protein kinase 20	calcium/calmodulin-dependent kinase
Sapur.006G223300.1	2185.471007	8.472338457	0.149799174	56.55798	0	0	cyan	0.871235697	9.21E-50	GO:0055114,GO:0016616,GO:0006694,GO:0003854	AT1G78570.1	ATRHM1,RHM1,ROL1	rhamnose biosynthesis 1	rhamnose synthase
Sapur.007G014800.1	1309.545511	6.197594047	0.112900055	54.89452	0	0	cyan	0.801103798	2.23E-36	GO:0008168	AT3G51070.1		S-adenosyl-L-methionine-dependent methyltransferases superfamily protein	S-adenosyl-L-methionine-dependent methyltransferase, putative
Sapur.007G015200.1	959.6594618	2.457754186	0.06144681	39.99808	0	0	cyan	0.887737518	4.32E-54	GO:0045454,GO:0015035,GO:0006662	AT3G51030.1	ATTRX H1,ATTRX1,TRX1	thioredoxin H-type 1	thioredoxin H-type 1 protein
Sapur.007G036800.1	157.9763102	6.095390527	0.130197034	46.81666	0	0	cyan	0.861060229	2.22E-47		AT5G66870.1	ASL1,LBD36	ASYMMETRIC LEAVES 2-like 1	asymmetric LEAVES protein, putative
Sapur.007G045700.1	624.1994798	9.241820339	0.161717436	57.14795	0	0	cyan	0.849745936	6.04E-45		AT5G67265.1			hypothetical protein
Sapur.007G051200.1	3500.466452	4.8889192	0.083934036	58.24716	0	0	cyan	0.843161351	1.28E-43	GO:0005516,GO:0046872,GO:0000166	AT4G37640.1	ACA2	calcium ATPase 2	calcium-transporting ATPase 2, plasma membrane-type protein
Sapur.007G051300.1	2808.423433	1.619405527	0.039056123	41.46355	0	0	cyan	0.928819469	1.01E-68	GO:0055085,GO:0005741	AT5G67500.1	ATVDAC2,VDAC2	voltage dependent anion channel 2	voltage-dependent anion-selective channel protein, putative
Sapur.007G088600.1	115.899835	5.721107446	0.112652964	50.78524	0	0	cyan	0.90643543	6.71E-60		AT1G16010.1	MGT2,MRS2-1	magnesium transporter 2	NA
Sapur.007G103000.1	4583.723788	11.71308288	0.170571444	68.66966	0	0	cyan	0.863883552	5.06E-48		AT5G09550.1		GDP dissociation inhibitor family protein / Rab GTPase activator family protein	Rab-GDP dissociation inhibitor
Sapur.007G111200.1	132.4737107	5.307608968	0.129833934	40.87998	0	0	cyan	0.893092008	1.21E-55		AT5G49150.1	ATGEX2,GEX2	gamete expressed 2	gamete protein
Sapur.008G004500.1	416.1479424	2.755038048	0.070491475	39.08328	0	0	cyan	0.908130489	1.74E-60	GO:0046961,GO:0046933,GO:0015986,GO:0000275	AT1G51650.1		ATP synthase epsilon chain, mitochondrial	NA
Sapur.008G072000.1	82.50232012	5.225546512	0.118877041	43.95758	0	0	cyan	0.866398612	1.32E-48		AT1G26650.1			ATP synthase subunit beta
Sapur.008G073200.1	943.9198463	6.514071771	0.100599759	64.75236	0	0	cyan	0.877913993	1.94E-51		AT2G03440.1	ATNRP1,NRP1	nodulin-related protein 1	hypothetical protein
Sapur.008G085100.1	291.9252088	7.928293642	0.149650311	52.9788	0	0	cyan	0.860008123	3.81E-47		AT4G16160.2	ATOEP16-2,ATOEP16-S	Mitochondrial import inner membrane translocase subunit Tim17/Tim22/Tim23 family protein	translocase of inner membrane protein
Sapur.008G095500.1	391.1225727	3.036403809	0.070233398	43.23305	0	0	cyan	0.89999104	9.07E-58	GO:0015743	AT1G25480.1		Aluminium activated malate transporter family protein	aluminum activated malate transporter
Sapur.008G127100.1	189.2477986	7.794480482	0.148491058	52.49125	0	0	cyan	0.901633312	2.68E-58		AT1G03070.1		Bax inhibitor-1 family protein	transmembrane BAX inhibitor motif protein
Sapur.008G135800.1	352.973765	5.733374338	0.098519862	58.19511	0	0	cyan	0.867521322	7.18E-49	GO:0046872	AT4G14368.1		Regulator of chromosome condensation (RCC1) family protein	chromosome condensation regulator repeat protein
Sapur.008G142400.1	336.7123867	4.494073875	0.076481057	58.76061	0	0	cyan	0.910189694	3.26E-61		AT1G67420.2		Zn-dependent exopeptidases superfamily protein	M28 Zn-peptidase family protein
Sapur.008G144800.1	271.5897291	9.660100358	0.224409147	43.04682	0	0	cyan	0.85823758	9.39E-47	GO:0003333	AT5G40780.1	LHT1	lysine histidine transporter 1	lysine/histidine transporter
Sapur.008G160000.1	349.9789198	8.340991326	0.136055624	61.30574	0	0	cyan	0.906795657	5.04E-60		AT3G06778.1		Chaperone DnaJ-domain superfamily protein	heat shock protein-binding protein, putative
Sapur.009G019000.1	617.6025913	7.86147502	0.135792612	57.89325	0	0	cyan	0.853051086	1.23E-45	GO:0016757,GO:0016020	AT1G07850.1		Protein of unknown function (DUF604)	transferring glycosyl group transferase, putative
Sapur.009G022200.1	152.6384966	3.846572287	0.079959765	48.10635	0	0	cyan	0.910132723	3.41E-61		AT5G02420.1			NA
Sapur.009G041500.1	10152.76411	9.026171123	0.167229212	53.97485	0	0	cyan	0.864009546	4.73E-48	GO:0045735	AT2G28490.1		RmlC-like cupins superfamily protein	vicilin-like antimicrobial peptides protein 2-3
Sapur.009G068700.1	121.6499081	7.937667097	0.192010768	41.3397	0	0	cyan	0.865406621	2.25E-48	GO:0005975,GO:0004553	AT4G33820.1		Glycosyl hydrolase superfamily protein	hydrolyzing O-glycosyl compounds hydrolase, putative
Sapur.009G083500.1	106.5128048	3.24456299	0.082360604	39.3946	0	0	cyan	0.891175441	4.45E-55	GO:0055114,GO:0020037,GO:0006979,GO:0004601	AT1G49570.1		Peroxidase superfamily protein	peroxidase
Sapur.009G083600.1	906.8807865	8.937229596	0.160641465	55.63464	0	0	cyan	0.753634793	4.78E-30		AT3G19090.1		RNA-binding protein	LA-related protein 6 LA RNA-binding domain protein
Sapur.009G093700.1	310.5532332	11.47438516	0.26178855	43.83074	0	0	cyan	0.825898487	2.06E-40		AT1G69800.2		Cystathionine beta-synthase (CBS) protein	NA
Sapur.009G107800.1	387.9771551	5.018405448	0.083373167	60.19209	0	0	cyan	0.907287204	3.41E-60		AT4G35070.1		SBP (S-ribonuclease binding protein) family protein	zinc finger, C3HC4 type (RING finger) protein
Sapur.009G128600.1	226.4145425	1.541151616	0.039390723	39.12474	0	0	cyan	0.930909104	1.09E-69	GO:0005515	AT1G67190.1		F-box/RNI-like superfamily protein	F-box/LRR protein
Sapur.010G007800.1	626.9174733	3.03855529	0.080903946	37.55757	0	0	cyan	0.882852496	9.65E-53	GO:0006950	AT4G15910.1	ATDI21,DI21	drought-induced 21	late embryogenesis abundant protein Lea5
Sapur.010G011800.1	74.30994488	7.184550645	0.186800293	38.46113	0	0	cyan	0.883426264	6.75E-53	GO:0008033	AT5G19040.1	ATIPT5,IPT5	isopentenyltransferase 5	cytokinin biosynthetic isopentenyltransferase
Sapur.010G032700.1	446.7400433	3.496739594	0.091982336	38.01534	0	0	cyan	0.859457197	5.05E-47	GO:0016787,GO:0008152	AT1G51760.1	IAR3,JR3	peptidase M20/M25/M40 family protein	IAA-amino acid hydrolase
Sapur.010G048700.1	5231.761259	13.60224994	0.199503977	68.18034	0	0	cyan	0.890679986	6.21E-55		AT4G13560.1	UNE15	Late embryogenesis abundant protein (LEA) family protein	late embryogenesis abundant protein, putative
Sapur.010G052900.1	79.99076954	3.048848887	0.075811785	40.21603	0	0	cyan	0.90349309	6.58E-59	GO:0008152,GO:0003824	AT5G63380.1		AMP-dependent synthetase and ligase family protein	acyl-CoA synthetase
Sapur.010G087000.1	6449.869967	13.01524252	0.170893068	76.16015	0	0	cyan	0.884375838	3.72E-53		AT4G13560.1	UNE15	Late embryogenesis abundant protein (LEA) family protein	late embryogenesis abundant protein, putative
Sapur.010G091700.1	269.7059517	3.527871497	0.081358112	43.36226	0	0	cyan	0.908113819	1.76E-60		AT5G38200.1		Class I glutamine amidotransferase-like superfamily protein	class I glutamine amidotransferase
Sapur.010G111400.1	2291.144485	5.114702547	0.101741347	50.27162	0	0	cyan	0.88862065	2.43E-54		AT4G16160.2	ATOEP16-2,ATOEP16-S	Mitochondrial import inner membrane translocase subunit Tim17/Tim22/Tim23 family protein	import inner membrane translocase, subunit Tim17/22
Sapur.010G115000.1	2143.545547	2.026445143	0.052470326	38.62078	0	0	cyan	0.919866867	6.93E-65	GO:0005524	AT3G06580.1	GAL1,GALK	Mevalonate/galactokinase family protein	galactokinase
Sapur.010G136900.1	95.61829155	5.102248545	0.099500796	51.27847	0	0	cyan	0.917803973	4.58E-64					NA
Sapur.010G140600.1	707.5116277	12.17561169	0.256424741	47.4822	0	0	cyan	0.851619502	2.46E-45	GO:0016788	AT1G06990.1		GDSL-like Lipase/Acylhydrolase superfamily protein	GDSL esterase/lipase
Sapur.010G150900.1	1496.534491	1.373068533	0.032443288	42.32211	0	0	cyan	0.935527423	6.14E-72		AT2G39970.1		Mitochondrial substrate carrier family protein	peroxisomal membrane protein
Sapur.010G172800.1	1202.571817	7.025486466	0.091427464	76.84219	0	0	cyan	0.90370692	5.59E-59	GO:0016021	AT2G38905.1		Low temperature and salt responsive protein family	stress-induced hydrophobic peptide protein
Sapur.010G177500.1	92.64952044	6.614325504	0.170378461	38.82137	0	0	cyan	0.79972593	3.60E-36		AT5G14990.1			hypothetical protein
Sapur.010G181100.1	2820.425421	3.212581124	0.068747611	46.73008	0	0	cyan	0.866513542	1.24E-48	GO:0055114,GO:0016491	AT3G10370.1	SDP6	FAD-dependent oxidoreductase family protein	glycerol-3-phosphate dehydrogenase
Sapur.011G027400.1	5604.167904	11.20453831	0.138823846	80.71047	0	0	cyan	0.853513117	9.84E-46	GO:0055085,GO:0016021	AT1G11260.1	ATSTP1,STP1	sugar transporter 1	hexose transporter
Sapur.011G058700.1	193.4532469	6.354897978	0.115453707	55.04282	0	0	cyan	0.892537802	1.77E-55		AT4G18160.1	ATKCO6,ATTPK3,KCO6,TPK3	Ca2+ activated outward rectifying K+ channel 6	outward rectifying potassium channel protein
Sapur.011G109500.1	2869.933126	4.763835874	0.094559983	50.37898	0	0	cyan	0.871645277	7.31E-50	GO:0008168	AT1G33170.1		S-adenosyl-L-methionine-dependent methyltransferases superfamily protein	dehydration-responsive family protein
Sapur.012G038700.1	118.1501962	8.610697652	0.22427091	38.39418	0	0	cyan	0.905023684	2.02E-59		AT3G18360.1		VQ motif-containing protein	VQ motif protein
Sapur.012G073000.1	1254.894929	3.217291482	0.077438414	41.54645	0	0	cyan	0.877054865	3.23E-51		AT3G48530.1	KING1	SNF1-related protein kinase regulatory subunit gamma 1	SNF1-related kinase regulatory subunit gamma
Sapur.013G027000.1	764.709929	3.858130727	0.097010054	39.77042	0	0	cyan	0.8217837	1.06E-39		AT1G54740.1		Protein of unknown function (DUF3049)	DUF3049 family protein
Sapur.013G057700.1	453.5472491	9.981859937	0.199241002	50.09943	0	0	cyan	0.871097167	9.95E-50		AT2G21720.1		Plant protein of unknown function (DUF639)	transmembrane protein, putative
Sapur.013G093900.1	293.9954921	4.15359476	0.091351715	45.46816	0	0	cyan	0.837833742	1.37E-42	GO:0006470,GO:0004722	AT1G07160.1		Protein phosphatase 2C family protein	protein phosphatase 2C
Sapur.013G102200.1	295.5201037	5.4543767	0.120672224	45.19994	0	0	cyan	0.869600045	2.29E-49	GO:0055085,GO:0016020,GO:0006811,GO:0005216	AT5G14870.1	ATCNGC18,CNGC18	cyclic nucleotide-gated channel 18	cyclic nucleotide-gated ion channel protein, putative
Sapur.013G102300.1	917.8197768	8.247856963	0.118292611	69.72419	0	0	cyan	0.903496015	6.56E-59	GO:0016820,GO:0016471,GO:0015992	AT4G23710.1	VAG2,VATG2,VHA-G2	vacuolar ATP synthase subunit G2	vacuolar ATP synthase subunit G
Sapur.013G131300.1	990.723185	12.69873295	0.257254773	49.36248	0	0	cyan	0.857451706	1.40E-46	GO:0055114,GO:0016491	AT2G20800.1	NDB4	NAD(P)H dehydrogenase B4	external alternative NAD(P)H-ubiquinone oxidoreductase B4
Sapur.014G041400.1	86.02272246	6.874164774	0.152076735	45.20195	0	0	cyan	0.931916507	3.64E-70		AT3G60460.1	DUO1	myb-like HTH transcriptional regulator family protein	R2r3-Myb transcription factor, putative
Sapur.014G048700.1	137.1177409	3.250233324	0.084258175	38.57469	0	0	cyan	0.867834707	6.05E-49	GO:0009733	AT3G60690.1		SAUR-like auxin-responsive protein family	auxin-induced SAUR-like protein
Sapur.014G070500.1	1262.916342	7.484431422	0.142752099	52.42957	0	0	cyan	0.913500904	2.02E-62		AT2G46140.1		Late embryogenesis abundant protein	late embryogenesis abundant protein Lea14-A
Sapur.014G100700.1	531.234737	11.3514584	0.245032107	46.32641	0	0	cyan	0.900222323	7.65E-58		AT1G56300.1		Chaperone DnaJ-domain superfamily protein	heat shock protein-binding protein, putative
Sapur.014G141000.1	831.7046569	10.95344287	0.208413011	52.55643	0	0	cyan	0.871845683	6.53E-50		AT1G10385.1		Vps51/Vps67 family (components of vesicular transport) protein	Vps51/Vps67 family (component of vesicular transport) protein
Sapur.016G071900.1	630.2162283	6.960888031	0.11770315	59.13935	0	0	cyan	0.815581154	1.17E-38		AT3G52620.1			NA
Sapur.016G078600.1	533.3877901	6.408328987	0.123340606	51.95636	0	0	cyan	0.852243951	1.82E-45		AT3G52620.1			transmembrane protein, putative
Sapur.016G091300.1	495.8120525	11.25070193	0.256345753	43.88878	0	0	cyan	0.838038708	1.25E-42		AT2G37770.2		NAD(P)-linked oxidoreductase superfamily protein	aldo/keto reductase family protein
Sapur.016G138400.1	414.8133448	3.999974367	0.094226194	42.45077	0	0	cyan	0.893444895	9.51E-56	GO:0055085	AT3G11320.1		Nucleotide-sugar transporter family protein	sugar phosphate/phosphate translocator, putative
Sapur.016G141300.1	1564.747907	6.180530859	0.107670303	57.40237	0	0	cyan	0.808191052	1.80E-37	GO:0016887,GO:0005524,GO:0016020	AT2G29940.1	ATPDR3,PDR3	pleiotropic drug resistance 3	pleiotropic drug resistance ABC transporter family protein
Sapur.016G151200.1	2801.686479	2.373139951	0.059965135	39.57533	0	0	cyan	0.897966419	3.95E-57		AT4G05320.2	UBQ10	polyubiquitin 10	NA
Sapur.016G183900.1	611.9396071	5.554814834	0.125549481	44.24403	0	0	cyan	0.772541906	2.20E-32	GO:0006508,GO:0004190	AT2G03200.1		Eukaryotic aspartyl protease family protein	aspartic proteinase nepenthesin-1 protein
Sapur.016G210400.1	2146.843991	3.771725587	0.069376606	54.36596	0	0	cyan	0.879320185	8.37E-52	GO:0055114,GO:0016620	AT3G04120.1	GAPC,GAPC-1,GAPC1	glyceraldehyde-3-phosphate dehydrogenase C subunit 1	glyceraldehyde-3-phosphate dehydrogenase
Sapur.016G277300.1	136.7012731	10.40308192	0.253934438	40.96759	0	0	cyan	0.859107775	6.04E-47	GO:0008289	AT4G04890.1	PDF2	protodermal factor 2	NA
Sapur.017G029200.1	580.4588054	6.252963573	0.100281387	62.35418	0	0	cyan	0.865318074	2.36E-48	GO:0005515	AT3G21880.1		B-box type zinc finger protein with CCT domain	constans-like zinc finger protein
Sapur.017G041900.1	744.1089866	5.241176178	0.089679495	58.44342	0	0	cyan	0.894793039	3.74E-56		AT1G69800.2		Cystathionine beta-synthase (CBS) protein	SNF1-related kinase regulatory subunit gamma
Sapur.018G082900.1	179.09334	5.256815408	0.104012849	50.54006	0	0	cyan	0.888433507	2.74E-54		AT5G46220.1		Protein of unknown function (DUF616)	NA
Sapur.018G094300.1	165.8962179	9.933740923	0.254129431	39.0893	0	0	cyan	0.858064882	1.03E-46	GO:0005509	AT4G32060.1		calcium-binding EF hand family protein	NA
Sapur.018G106700.1	81.43855649	6.784964968	0.170189869	39.86703	0	0	cyan	0.839293487	7.23E-43		AT2G19330.1	PIRL6	plant intracellular ras group-related LRR 6	plant intracellular Ras-group-related LRR protein
Sapur.018G113400.1	255.2548147	8.30500664	0.170081331	48.82962	0	0	cyan	0.835944821	3.12E-42	GO:0005524	AT4G16130.1	ARA1,ATISA1,ISA1	arabinose kinase	galactokinase
Sapur.019G022200.1	1269.60769	10.38328553	0.165698776	62.66362	0	0	cyan	0.82445123	3.69E-40	GO:0016459,GO:0005524,GO:0003774,GO:0005515	AT1G54560.1	ATXIE,XIE	Myosin family protein with Dil domain	myosin XI, putative
Sapur.019G038300.1	3432.192924	10.4843852	0.214184521	48.95025	0	0	cyan	0.857444066	1.40E-46					NA
Sapur.019G064800.1	3167.751955	12.20130133	0.177935768	68.57138	0	0	cyan	0.864682188	3.31E-48		AT4G10260.1		pfkB-like carbohydrate kinase family protein	fructokinase
Sapur.15WG025300.1	586.6673224	2.979046662	0.064670679	46.06487	0	0	cyan	0.925174611	4.21E-67		AT1G73480.1		alpha/beta-Hydrolases superfamily protein	NA
Sapur.15WG085800.1	413.0152014	6.681915873	0.106666458	62.64308	0	0	cyan	0.894223617	5.56E-56		AT3G47870.1	LBD27,SCP	LOB domain-containing protein 27	LOB domain protein, putative
Sapur.15WG137700.1	2126.703545	10.13561989	0.211139178	48.00445	0	0	cyan	0.862065254	1.31E-47	GO:0004857	AT3G47380.1		Plant invertase/pectin methylesterase inhibitor superfamily protein	plant invertase/pectin methylesterase inhibitor protein
Sapur.15WG142100.1	199.8955176	3.059612672	0.07189957	42.55398	0	0	cyan	0.903557936	6.26E-59	GO:0003824,GO:0006470,GO:0004722	AT5G51760.1	AHG1	Protein phosphatase 2C family protein	protein phosphatase 2C
Sapur.15ZG035300.2	80.00227734	4.49863177	0.094919235	47.39431	0	0	cyan	0.909628875	5.16E-61	GO:0006468,GO:0005524,GO:0004672	AT1G73670.1	ATMPK15,MPK15	MAP kinase 15	MAP kinase
Sapur.15ZG040200.1	268.3950513	10.88840564	0.248102491	43.88672	0	0	cyan	0.912317144	5.52E-62	GO:0016021	AT1G18520.1	TET11	tetraspanin11	NA
Sapur.15ZG054200.1	387.371774	1.732715484	0.041553713	41.69821	0	0	cyan	0.924090737	1.23E-66	GO:0006468,GO:0004672	AT5G08160.1	ATPK3,PK3	serine/threonine protein kinase 3	Serine/Threonine kinase domain protein
Sapur.T046800.1	2892.565986	13.02149283	0.215344676	60.46814	0	0	cyan	0.892301529	2.08E-55	GO:0009790				late embryogenesis abundant group 1 protein
Sapur.T090900.1	311.2105464	8.364112131	0.154259552	54.22103	0	0	cyan	0.877015206	3.31E-51	GO:0016301,GO:0005524	AT3G27440.1	UKL5	uridine kinase-like 5	uridine kinase
Sapur.016G237200.1	98.40980133	3.632413672	0.096951494	37.4663	3.26E-307	1.91E-305	cyan	0.86709107	9.08E-49	GO:0005515,GO:0005096	AT4G05330.1	AGD13	ARF-GAP domain 13	ADP-ribosylation factor GTPase-activating protein AGD12
Sapur.008G038400.1	3873.672083	4.498754232	0.120275864	37.40363	3.41E-306	1.97E-304	cyan	0.886292899	1.10E-53	GO:0016020,GO:0006810,GO:0005215	AT4G01470.1	ATTIP1.3,GAMMA-TIP3,TIP	tonoplast intrinsic protein 1;3	tonoplast intrinsic protein
Sapur.016G235900.1	234.7717147	2.891773487	0.07768757	37.22312	2.89E-303	1.64E-301	cyan	0.913085171	2.88E-62		AT5G55950.1		Nucleotide/sugar transporter family protein	solute carrier family 35 protein
Sapur.012G066300.1	63.78236694	4.424273118	0.119125763	37.13952	6.47E-302	3.66E-300	cyan	0.847524898	1.72E-44		AT5G50210.1	OLD5,QS,SUFE3	quinolinate synthase	Fe-S metabolism associated domain protein
Sapur.002G196000.1	97.51948798	8.794164592	0.237958274	36.95675	5.67E-299	3.16E-297	cyan	0.937122332	9.38E-73	GO:0003677	AT3G24650.1	ABI3,SIS10	AP2/B3-like transcriptional factor family protein	abscisic acid insensitive-like factor
Sapur.010G200400.1	72.77567516	3.797229886	0.103120115	36.82337	7.81E-297	4.30E-295	cyan	0.869971992	1.87E-49		AT5G04980.2		DNAse I-like superfamily protein	NA
Sapur.003G056600.1	118.2735783	3.093328497	0.084415284	36.64418	5.67E-294	3.08E-292	cyan	0.886063056	1.27E-53	GO:0030955,GO:0006096,GO:0004743,GO:0000287	AT1G32440.1	PKp3	plastidial pyruvate kinase 3	pyruvate kinase
Sapur.003G131600.1	56.78459581	8.982314506	0.24685821	36.38653	6.95E-290	3.72E-288	cyan	0.902162964	1.80E-58					NA
Sapur.011G027500.1	595.7819535	2.100762493	0.0578705	36.30109	1.55E-288	8.24E-287	cyan	0.899895845	9.72E-58	GO:0005515	AT1G61350.1		ARM repeat superfamily protein	ubiquitin-protein ligase, putative
Sapur.018G021400.1	131.9357216	3.327626264	0.091692188	36.29127	2.22E-288	1.18E-286	cyan	0.854642791	5.65E-46	GO:0005515	AT5G11100.1	ATSYTD,NTMC2T2.2,NTMC	Calcium-dependent lipid-binding (CaLB domain) family protein	NA
Sapur.010G070100.1	146.5194858	2.431488566	0.067105143	36.23401	1.77E-287	9.32E-286	cyan	0.918601297	2.22E-64		AT2G01770.1	ATVIT1,VIT1	vacuolar iron transporter 1	vacuolar iron transporter 1
Sapur.007G031400.1	2830.69771	2.17761143	0.060136951	36.21087	4.11E-287	2.15E-285	cyan	0.863479225	6.27E-48		AT3G50830.1	ATCOR413-PM2,COR413-P	cold-regulated 413-plasma membrane 2	cold acclimation protein COR413-PM1
Sapur.016G085200.1	739.9794283	3.823301337	0.105606555	36.20326	5.41E-287	2.83E-285	cyan	0.829817683	4.15E-41	GO:0005515	AT5G03250.1		Phototropic-responsive NPH3 family protein	BTB/POZ domain protein
Sapur.012G018500.1	118.6215278	9.337788381	0.258118354	36.17638	1.43E-286	7.47E-285	cyan	0.835408483	3.93E-42		AT5G24130.1			polypyrimidine tract-binding-like protein, putative
Sapur.006G199900.1	2650.726972	1.254616259	0.034682267	36.17457	1.53E-286	7.96E-285	cyan	0.917662854	5.20E-64	GO:0030170,GO:0009058,GO:0008483,GO:0006520	AT5G11520.1	ASP3,YLS4	aspartate aminotransferase 3	aspartate aminotransferase
Sapur.012G022100.1	786.0327101	2.397667989	0.06637039	36.12557	9.00E-286	4.66E-284	cyan	0.916052881	2.19E-63		AT2G46170.1		Reticulon family protein	24 kDa seed maturation protein
Sapur.T171700.1	50.75767798	4.551192205	0.126745267	35.90818	2.28E-282	1.15E-280	cyan	0.832585394	1.31E-41		AT2G18460.1	LCV3	like COV 3	NA
Sapur.010G105600.1	101.2882632	7.947516626	0.221778236	35.83542	3.10E-281	1.56E-279	cyan	0.754341554	3.94E-30	GO:0055085,GO:0046873,GO:0030001,GO:0016020	AT2G04032.1	ZIP7	zinc transporter 7 precursor	iron regulated transporter
Sapur.005G171200.1	92.50518008	2.08114987	0.058159073	35.78375	1.98E-280	9.93E-279	cyan	0.91991859	6.61E-65		AT5G20030.1		Plant Tudor-like RNA-binding protein	NA
Sapur.010G150100.1	366.2415865	3.006365395	0.08410907	35.74365	8.30E-280	4.15E-278	cyan	0.876953936	3.43E-51		AT1G07280.1		Tetratricopeptide repeat (TPR)-like superfamily protein	plant/F22G5-32 protein, putative
Sapur.009G056600.1	140.010795	4.30109277	0.12044049	35.71135	2.63E-279	1.31E-277	cyan	0.762575243	4.00E-31	GO:0005515	AT5G58610.1		PHD finger transcription factor, putative	acyl-CoA N-acyltransferase with RING/FYVE/PHD-type zinc finger protein, putative
Sapur.004G052000.1	8037.451612	1.872940085	0.052473924	35.69278	5.12E-279	2.54E-277	cyan	0.886962325	7.14E-54	GO:0070569,GO:0008152	AT5G17310.2	AtUGP2,UGP2	UDP-glucose pyrophosphorylase 2	NA
Sapur.016G013300.1	452.3139462	2.806622694	0.078696877	35.66371	1.44E-278	7.17E-277	cyan	0.840945089	3.47E-43	GO:0016758,GO:0008152	AT3G21780.1	UGT71B6	UDP-glucosyl transferase 71B6	UDP-glucose flavonoid 3-O-glucosyltransferase
Sapur.016G141100.1	48.36001091	8.904785486	0.249688586	35.66357	1.45E-278	7.20E-277	cyan	0.89502766	3.18E-56		AT2G29940.1	ATPDR3,PDR3	pleiotropic drug resistance 3	NA
Sapur.016G103100.1	1336.313042	1.405889862	0.039429901	35.65542	1.94E-278	9.58E-277	cyan	0.927911133	2.61E-68	GO:0055114,GO:0016627,GO:0003995,GO:0050660,GO:0008152	AT3G51840.1	ACX4,ATG6,ATSCX	acyl-CoA oxidase 4	acyl-CoA dehydrogenase
Sapur.013G036000.1	194.5222937	3.262125204	0.091602157	35.61188	9.17E-278	4.50E-276	cyan	0.867433132	7.54E-49	GO:0007165,GO:0005515,GO:0043531	AT5G36930.1		Disease resistance protein (TIR-NBS-LRR class) family	NA
Sapur.011G063400.1	125.7481216	9.085476996	0.255483614	35.56188	5.44E-277	2.66E-275	cyan	0.828278271	7.83E-41	GO:0005515	AT3G11010.1	AtRLP34,RLP34	receptor like protein 34	NA
Sapur.004G081300.1	172.406002	2.790883691	0.07935566	35.16931	5.89E-271	2.79E-269	cyan	0.914095353	1.21E-62		AT5G16020.1	GEX3	gamete-expressed 3	NA
Sapur.004G007200.1	106.4389353	8.922935657	0.255327501	34.94702	1.44E-267	6.66E-266	cyan	0.820289958	1.91E-39	GO:0005524	AT5G40010.1	AATP1	AAA-ATPase 1	AAA family ATPase
Sapur.016G177200.1	756.9707734	1.858507861	0.053231159	34.91391	4.57E-267	2.12E-265	cyan	0.897094696	7.39E-57	GO:0055114,GO:0016491	AT4G33910.1		2-oxoglutarate (2OG) and Fe(II)-dependent oxygenase superfamily protein	prolyl 4-hydroxylase alpha subunit, putative
Sapur.016G279200.1	142.3920184	1.75989712	0.050641887	34.75181	1.30E-264	5.89E-263	cyan	0.901805571	2.36E-58					NA
Sapur.003G050300.1	2512.640512	1.029417738	0.029649367	34.71972	3.97E-264	1.79E-262	cyan	0.923596063	2.00E-66	GO:0005525	AT1G02130.1	ARA-5,ARA5,ATRAB1B,ATRA	RAS 5	small GTPase family Rab protein
Sapur.014G072300.1	37.97459016	5.621090299	0.161921777	34.71485	4.70E-264	2.12E-262	cyan	0.871950447	6.16E-50	GO:0046488,GO:0016307	AT1G01460.1	ATPIPK11,PIPK11	Phosphatidylinositol-4-phosphate 5-kinase, core	NA
Sapur.018G081400.1	180.1094331	4.23483688	0.122219087	34.64955	4.54E-263	2.03E-261	cyan	0.791858831	5.12E-35		AT4G31830.1			transmembrane protein, putative
Sapur.004G076800.1	222.8191015	2.045538637	0.059183629	34.56258	9.23E-262	4.10E-260	cyan	0.899062897	1.79E-57	GO:0016757,GO:0047262	AT3G02350.1	GAUT9	galacturonosyltransferase 9	CAZy family GT8 glycosyltransferase
Sapur.019G057900.1	1343.416349	1.574246529	0.04559593	34.52603	3.26E-261	1.44E-259	cyan	0.904416803	3.24E-59	GO:0030244,GO:0016866	AT5G16510.2		Alpha-1,4-glucan-protein synthase family protein	alpha-1%2C4-glucan-protein synthase [UDP-forming] protein, putative
Sapur.008G089800.1	67.44655361	9.423417694	0.27305378	34.51121	5.45E-261	2.40E-259	cyan	0.761609886	5.25E-31					NA
Sapur.008G021100.1	79.09232332	9.460882516	0.274230819	34.49971	8.10E-261	3.55E-259	cyan	0.735221747	5.76E-28					NA
Sapur.004G031800.1	160.4709729	1.463753682	0.04243447	34.49445	9.72E-261	4.25E-259	cyan	0.930938676	1.06E-69		AT1G28380.1	NSL1	MAC/Perforin domain-containing protein	MACPF domain protein
Sapur.010G038900.1	173.9986225	2.928737666	0.085720321	34.1662	7.69E-256	3.27E-254	cyan	0.829415837	4.90E-41		AT4G14450.1			hypothetical protein
Sapur.018G092000.1	38.24175544	8.640480948	0.253082164	34.14101	1.82E-255	7.70E-254	cyan	0.871614619	7.44E-50		AT4G32060.1		calcium-binding EF hand family protein	NA
Sapur.006G146000.1	48.5900378	8.378192153	0.245488546	34.12865	2.77E-255	1.17E-253	cyan	0.909484082	5.81E-61	GO:0006355,GO:0003677	AT3G44350.2	anac061,NAC061	NAC domain containing protein 61	NA
Sapur.008G074200.1	3202.018472	2.471511652	0.072625721	34.0308	7.81E-254	3.27E-252	cyan	0.866438096	1.29E-48	GO:0055114,GO:0051287,GO:0016616	AT3G29360.2		UDP-glucose 6-dehydrogenase family protein	UDP-glucose 6-dehydrogenase
Sapur.001G048300.1	1986.574487	6.786882645	0.200226457	33.89603	7.62E-252	3.15E-250	cyan	0.902803887	1.11E-58		AT1G27990.1			transmembrane protein, putative
Sapur.006G055100.1	120.0671963	4.187048517	0.124260905	33.69562	6.70E-249	2.73E-247	cyan	0.793874678	2.62E-35		AT5G20190.1		Tetratricopeptide repeat (TPR)-like superfamily protein	TPR domain protein
Sapur.005G081100.1	396.3089675	2.167697569	0.064453159	33.63214	5.69E-248	2.30E-246	cyan	0.90936122	6.42E-61		AT4G35560.1		Transducin/WD40 repeat-like superfamily protein	NA
Sapur.010G104400.1	387.9675273	1.896006003	0.056425207	33.60211	1.56E-247	6.30E-246	cyan	0.912586641	4.40E-62	GO:0008295,GO:0006597,GO:0004014	AT3G25570.1		Adenosylmethionine decarboxylase family protein	S-adenosylmethionine decarboxylase proenzyme, putative
Sapur.15WG020900.1	2326.458599	1.511944577	0.045058518	33.55513	7.58E-247	3.05E-245	cyan	0.906493811	6.40E-60		AT4G23630.1	BTI1,RTNLB1	VIRB2-interacting protein 1	24 kDa seed maturation protein
Sapur.019G008900.1	384.0101587	1.874423997	0.055876697	33.54572	1.04E-246	4.17E-245	cyan	0.919779531	7.52E-65	GO:0006468,GO:0005524,GO:0004672	AT5G63650.1	SNRK2-5,SNRK2.5,SRK2H	SNF1-related protein kinase 2.5	Serine/Threonine kinase domain protein
Sapur.003G019900.1	799.3561738	1.861942318	0.05574349	33.40197	1.28E-244	5.11E-243	cyan	0.896412237	1.20E-56		AT2G25930.1	ELF3,PYK20	hydroxyproline-rich glycoprotein family protein	early flowering protein, putative
Sapur.005G080000.1	2534.209488	2.010258025	0.06021999	33.38191	2.51E-244	9.94E-243	cyan	0.860264938	3.34E-47	GO:0042578,GO:0052866	AT5G66020.1	ATSAC1B,ATSAC6,IBS2,SAC	Phosphoinositide phosphatase family protein	phosphatidylinositide phosphatase SAC1
Sapur.006G190800.1	418.252184	1.661556235	0.05009535	33.16787	3.13E-241	1.22E-239	cyan	0.90331044	7.56E-59	GO:0003950	AT1G70440.1	SRO3	similar to RCD one 3	inactive poly [ADP-ribose] polymerase SRO3, putative
Sapur.15ZG057100.1	52.17969495	3.026052889	0.09131447	33.13881	8.21E-241	3.18E-239	cyan	0.876669075	4.06E-51		AT1G61415.1			hypothetical protein
Sapur.017G124600.1	4803.67583	1.480614582	0.04479586	33.05249	1.43E-239	5.53E-238	cyan	0.927189253	5.50E-68	GO:0055114,GO:0016616,GO:0006102,GO:0004450	AT1G65930.1	cICDH	cytosolic NADP+-dependent isocitrate dehydrogenase	isocitrate dehydrogenase [NADP] protein
Sapur.008G080400.1	194.0004092	1.861906831	0.056367504	33.03156	2.86E-239	1.10E-237	cyan	0.898164348	3.43E-57	GO:0005515	AT5G49610.1		F-box family protein	F-box plant protein
Sapur.016G175400.1	267.7990548	2.808638471	0.08588954	32.70059	1.53E-234	5.74E-233	cyan	0.889056976	1.82E-54		AT3G19610.1		Plant protein of unknown function (DUF936)	DUF936 family protein
Sapur.002G095500.1	29.94128044	4.307484837	0.131792843	32.68375	2.66E-234	9.93E-233	cyan	0.871643049	7.32E-50	GO:0005515	AT5G58610.1		PHD finger transcription factor, putative	acyl-CoA N-acyltransferase with RING/FYVE/PHD-type zinc finger protein, putative
Sapur.009G130300.1	35.82439778	7.906325421	0.243283426	32.49841	1.12E-231	4.12E-230	cyan	0.92289926	3.93E-66		AT4G35280.1		C2H2-like zinc finger protein	NA
Sapur.003G145500.1	28.73939187	5.359688818	0.165110577	32.46121	3.76E-231	1.38E-229	cyan	0.885195987	2.21E-53	GO:0006468,GO:0005524,GO:0004672	AT4G19110.1		Protein kinase superfamily protein	Serine/Threonine kinase family protein
Sapur.15ZG049100.1	338.0221034	1.160833315	0.03577921	32.44435	6.51E-231	2.37E-229	cyan	0.912803039	3.66E-62	GO:0030117,GO:0016192,GO:0006886	AT1G48760.3	delta-ADR	delta-adaptin	AP-3 complex subunit delta
Sapur.012G067800.1	974.5239893	1.280701891	0.039539169	32.39071	3.71E-230	1.34E-228	cyan	0.916778833	1.15E-63	GO:0055114,GO:0008113,GO:0030091,GO:0016671,GO:0006979	AT5G61640.1	ATMSRA1,PMSR1	peptidemethionine sulfoxide reductase 1	peptide methionine sulfoxide reductase
Sapur.002G027400.1	62.4950577	8.298762701	0.256739234	32.3237	3.25E-229	1.17E-227	cyan	0.839866707	5.61E-43	GO:0051861,GO:0046836,GO:0017089,GO:0005737	AT2G33470.2	ATGLTP1,GLTP1	glycolipid transfer protein 1	glycolipid transfer protein, putative
Sapur.014G116400.1	3087.857069	1.399163768	0.043474295	32.1837	2.98E-227	1.06E-225	cyan	0.911098011	1.53E-61	GO:0030170,GO:0009058,GO:0008483,GO:0006520	AT2G30970.1	ASP1	aspartate aminotransferase 1	aspartate aminotransferase
Sapur.001G148000.1	73.43330593	1.79520007	0.055845069	32.14608	1.00E-226	3.56E-225	cyan	0.902701442	1.20E-58		AT1G54215.1		proline-rich family protein	NA
Sapur.005G134600.1	41.24682208	8.376902739	0.260594725	32.14533	1.03E-226	3.64E-225	cyan	0.807581953	2.25E-37		AT1G77765.2			NA
Sapur.018G092500.1	30.91862291	8.071697539	0.251124948	32.14216	1.14E-226	4.03E-225	cyan	0.886494112	9.66E-54	GO:0005509	AT4G32060.1		calcium-binding EF hand family protein	NA
Sapur.15ZG053600.1	230.2984185	1.094005651	0.034059871	32.12008	2.31E-226	8.16E-225	cyan	0.912802445	3.66E-62	GO:0008270,GO:0005515	AT5G08139.1		RING/U-box superfamily protein	zinc finger protein
Sapur.016G257400.1	62.13195979	8.889995264	0.277190091	32.07184	1.09E-225	3.82E-224	cyan	0.733944662	7.92E-28		AT1G53130.1	GRI	Stigma-specific Stig1 family protein	Stig1 stigma-specific protein
Sapur.001G064800.1	735.4755759	1.358105963	0.04241135	32.02223	5.35E-225	1.86E-223	cyan	0.90177375	2.41E-58	GO:0005525	AT1G02130.1	ARA-5,ARA5,ATRAB1B,ATRA	RAS 5	small GTPase family Rab protein
Sapur.005G154400.1	43.92466308	5.268445772	0.16452873	32.02143	5.49E-225	1.91E-223	cyan	0.828458087	7.27E-41					NA
Sapur.011G106500.1	32.54491005	8.065779844	0.251900461	32.01971	5.80E-225	2.01E-223	cyan	0.881244118	2.60E-52		AT3G28150.1	TBL22	TRICHOME BIREFRINGENCE-LIKE 22	Pmr5/Cas1p GDSL/SGNH-like acyl-esterase family protein
Sapur.013G036300.1	78.79880447	2.530071032	0.079156492	31.9629	3.58E-224	1.23E-222	cyan	0.857275189	1.53E-46		AT1G79590.2	ATSYP52,SYP52	syntaxin of plants 52	transmembrane protein, putative
Sapur.002G137300.1	1895.945326	1.911292805	0.059940363	31.88657	4.10E-223	1.40E-221	cyan	0.878340119	1.51E-51	GO:0006796,GO:0005737,GO:0004427,GO:0000287	AT1G01050.1	AtPPa1,PPa1	pyrophosphorylase 1	soluble inorganic pyrophosphatase
Sapur.T190800.1	67.88369478	5.045186121	0.15867124	31.79648	7.24E-222	2.47E-220	cyan	0.723250248	1.05E-26	GO:0006412,GO:0005840,GO:0005622,GO:0003735	AT3G16780.1		Ribosomal protein L19e family protein	ribosomal protein L19
Sapur.002G115200.1	884.0047647	1.299476481	0.040924876	31.75273	2.91E-221	9.87E-220	cyan	0.919863449	6.95E-65		AT3G60450.1		Phosphoglycerate mutase family protein	phosphoglycerate mutase family protein
Sapur.007G109400.1	33.20632379	8.475040252	0.267505091	31.68179	2.77E-220	9.31E-219	cyan	0.790677969	7.56E-35	GO:0005515	AT2G32360.1		Ubiquitin-like superfamily protein	ubiquitin-like protein
Sapur.010G043100.1	152.8602007	2.362421515	0.074673972	31.63648	1.16E-219	3.89E-218	cyan	0.856336885	2.44E-46		AT3G23440.1	EDA6,MEE37	embryo sac development arrest 6	embryo sac development arrest 6 protein, putative
Sapur.006G104100.1	1279.97055	0.89018414	0.028229928	31.53335	3.03E-218	1.00E-216	cyan	0.913089068	2.87E-62	GO:0055114,GO:0016616,GO:0051287,GO:0000287	AT5G03290.1	IDH-V	isocitrate dehydrogenase V	isocitrate dehydrogenase (NAD+)
Sapur.001G030100.1	475.9635789	1.900688266	0.060332843	31.50338	7.81E-218	2.56E-216	cyan	0.871267325	9.05E-50	GO:0016853,GO:0005975	AT5G57330.1		Galactose mutarotase-like superfamily protein	aldose 1-epimerase, putative
Sapur.15WG081700.1	127.3375622	3.960409412	0.125817897	31.47731	1.78E-217	5.82E-216	cyan	0.789524191	1.10E-34	GO:0016887,GO:0005524,GO:0016021,GO:0006810,GO:0005215	AT3G47730.1	ABCA2,ATATH1,ATH1	ATP-binding cassette A2	ABC transporter A family protein
Sapur.15WG086500.1	265.7275106	1.706960185	0.054253214	31.46284	2.80E-217	9.17E-216	cyan	0.886643092	8.78E-54	GO:0016020,GO:0008378,GO:0006486,GO:0030246	AT5G62620.1		Galactosyltransferase family protein	galactosyltransferase
Sapur.001G066700.1	97.22055867	2.102654949	0.066859502	31.44886	4.35E-217	1.42E-215	cyan	0.883883187	5.07E-53	GO:0008270,GO:0006355,GO:0006352	AT4G19550.1		zinc ion binding;transcription regulators	brf1-like TBP-binding domain protein
Sapur.004G052900.1	912.6698789	6.047505445	0.193237285	31.29575	5.33E-215	1.73E-213	cyan	0.846581505	2.67E-44	GO:0030170,GO:0019752,GO:0016831	AT5G17330.1	GAD,GAD1	glutamate decarboxylase	glutamate decarboxylase
Sapur.007G093400.1	988.0203411	1.647876644	0.052863487	31.1723	2.53E-213	8.08E-212	cyan	0.870734652	1.22E-49		AT5G20680.3	TBL16	TRICHOME BIREFRINGENCE-LIKE 16	Pmr5/Cas1p GDSL/SGNH-like acyl-esterase family protein
Sapur.001G018600.1	21.65892018	7.784075038	0.25080866	31.03591	1.77E-211	5.60E-210	cyan	0.888031086	3.57E-54	GO:0005515				F-box protein
Sapur.007G080800.1	543.1098633	1.368752672	0.044141786	31.00809	4.19E-211	1.33E-209	cyan	0.895369372	2.50E-56	GO:0055114,GO:0020037,GO:0016705,GO:0005506	AT1G64940.1	CYP89A6	cytochrome P450, family 87, subfamily A, polypeptide 6	cytochrome P450 family protein
Sapur.016G279000.1	421.0348614	1.611585516	0.052120368	30.92046	6.34E-210	1.99E-208	cyan	0.892998503	1.29E-55					NA
Sapur.009G092900.1	589.0931644	2.162364127	0.070245014	30.78317	4.40E-208	1.37E-206	cyan	0.855624143	3.48E-46		AT2G32380.1		Transmembrane protein 97, predicted	transmembrane protein
Sapur.011G065700.1	62.69078394	4.367169074	0.141975848	30.75994	9.00E-208	2.80E-206	cyan	0.758463988	1.27E-30	GO:0005515,GO:0006468,GO:0005524,GO:0004672	AT3G47570.1		Leucine-rich repeat protein kinase family protein	receptor-kinase, putative
Sapur.014G009100.1	41.69326476	3.579586707	0.116584823	30.70371	5.08E-207	1.58E-205	cyan	0.849998587	5.36E-45	GO:0016021	AT5G43140.1		Peroxisomal membrane 22 kDa (Mpv17/PMP22) family protein	22 kDa peroxisomal membrane protein, putative
Sapur.008G095800.1	2784.391332	1.025343087	0.033544726	30.56645	3.42E-205	1.04E-203	cyan	0.909907894	4.10E-61	GO:0006096,GO:0006094,GO:0004347	AT5G42740.1		Sugar isomerase (SIS) family protein	cytosolic glucose-6-phosphate isomerase
Sapur.007G017900.1	808.6379066	1.065446592	0.034880935	30.54524	6.54E-205	1.99E-203	cyan	0.911394631	1.20E-61		AT5G66420.2			Tm-1^GCR26 protein
Sapur.004G138100.1	107.300957	2.658088573	0.087460451	30.39189	7.03E-203	2.11E-201	cyan	0.88450661	3.42E-53	GO:0055085,GO:0016020,GO:0015297,GO:0015238,GO:0006855	AT4G38380.1		MATE efflux family protein	MATE efflux family protein
Sapur.004G177100.1	1280.333951	1.606452124	0.052893083	30.37169	1.30E-202	3.90E-201	cyan	0.870023996	1.81E-49	GO:0016757,GO:0047262	AT2G38650.1	GAUT7,LGT7	galacturonosyltransferase 7	glycosyltransferase family 8 protein
Sapur.010G022100.1	44.74457616	3.212029891	0.105938731	30.31969	6.31E-202	1.88E-200	cyan	0.83367324	8.24E-42		AT3G26350.1			late embryogenesis abundant protein
Sapur.004G131900.1	99.29690839	2.579953721	0.08520704	30.27865	2.19E-201	6.52E-200	cyan	0.828182484	8.15E-41		AT4G35070.1		SBP (S-ribonuclease binding protein) family protein	zinc finger, C3HC4 type (RING finger) protein
Sapur.012G045600.1	386.527015	1.418811034	0.047055294	30.152	1.01E-199	2.98E-198	cyan	0.881264883	2.57E-52	GO:0016757,GO:0047262	AT1G18580.1	GAUT11	galacturonosyltransferase 11	glycosyltransferase family 8 protein
Sapur.014G048600.1	69.89399446	3.251524981	0.108058596	30.09039	6.47E-199	1.90E-197	cyan	0.82779581	9.54E-41					NA
Sapur.008G053700.1	1127.224799	1.562519473	0.051929395	30.08931	6.69E-199	1.96E-197	cyan	0.873909424	2.02E-50	GO:0016788	AT3G11210.1		SGNH hydrolase-type esterase superfamily protein	NA
Sapur.014G083800.1	130.0383285	2.402443587	0.080517699	29.83746	1.28E-195	3.67E-194	cyan	0.885345762	2.01E-53	GO:0055085,GO:0016021,GO:0008324,GO:0006812	AT2G46800.1	ATCDF1,ATMTP1,MTP1,ZAT	zinc transporter of Arabidopsis thaliana	metal transporter, putative
Sapur.016G151600.1	83.27948805	2.336845868	0.078478996	29.7767	7.83E-195	2.24E-193	cyan	0.85895716	6.52E-47	GO:0055114,GO:0016491	AT5G13700.1	APAO,ATPAO1,PAO1	polyamine oxidase 1	polyamine oxidase
Sapur.010G157600.1	88.96097443	3.14192709	0.105820051	29.69123	9.97E-194	2.83E-192	cyan	0.846855069	2.35E-44		AT5G02140.1		Pathogenesis-related thaumatin superfamily protein	thaumatin-like protein
Sapur.013G064300.1	1564.724454	1.117687833	0.037773407	29.58928	2.05E-192	5.78E-191	cyan	0.899201525	1.62E-57	GO:0016020	AT5G36290.1		Uncharacterized protein family (UPF0016)	GDT1-like protein
Sapur.001G174500.1	412.2648983	1.378434224	0.046655599	29.54488	7.64E-192	2.14E-190	cyan	0.889197741	1.66E-54	GO:0016021,GO:0006810	AT1G09580.1		emp24/gp25L/p24 family/GOLD family protein	transmembrane emp24 domain protein, putative
Sapur.011G093400.1	64.88663798	2.116372965	0.071776454	29.48562	4.40E-191	1.23E-189	cyan	0.881199131	2.68E-52	GO:0048544,GO:0006468,GO:0004672	AT4G27290.1		S-locus lectin protein kinase family protein	Serine/Threonine kinase domain protein
Sapur.006G079600.1	546.6278959	2.205564733	0.074920391	29.43878	1.75E-190	4.88E-189	cyan	0.861111966	2.16E-47	GO:0055085,GO:0016021,GO:0016020,GO:0006810,GO:0005215	AT3G54140.1	ATPTR1,PTR1	peptide transporter 1	peptide transporter, putative
Sapur.002G134200.1	431.9760196	1.485372036	0.050631704	29.3368	3.52E-189	9.71E-188	cyan	0.886824235	7.81E-54		AT4G01660.1	ABC1,ATABC1,ATATH10	ABC transporter 1	ubiquinone biosynthesis protein, putative
Sapur.003G130400.1	23.845856	5.414911761	0.184768347	29.30649	8.57E-189	2.36E-187	cyan	0.858699295	7.43E-47		AT1G67370.1	ASY1,ATASY1	DNA-binding HORMA family protein	NA
Sapur.010G123100.1	2477.730076	1.136226003	0.038880632	29.22344	9.77E-188	2.66E-186	cyan	0.916671116	1.27E-63	GO:0055114,GO:0051287,GO:0016616	AT5G15490.1		UDP-glucose 6-dehydrogenase family protein	UDP-glucose 6-dehydrogenase
Sapur.010G136000.1	36.99051746	3.577626412	0.122590176	29.18363	3.13E-187	8.47E-186	cyan	0.809844239	9.87E-38		AT1G53540.1		HSP20-like chaperones superfamily protein	class I small heat shock protein
Sapur.004G071300.1	5115.50967	0.970954534	0.033284367	29.17149	4.46E-187	1.21E-185	cyan	0.904145973	3.99E-59		AT3G02090.1	MPPBETA	Insulinase (Peptidase family M16) protein	processing peptidase beta subunit
Sapur.001G077900.1	2694.986155	0.964441514	0.033138407	29.10344	3.25E-186	8.73E-185	cyan	0.915189557	4.68E-63		AT1G64230.2	UBC28	ubiquitin-conjugating enzyme 28	ubiquitin-conjugating enzyme E2
Sapur.008G111500.1	25.66294187	6.142537787	0.211345448	29.06397	1.02E-185	2.74E-184	cyan	0.878912879	1.07E-51	GO:0005515	AT2G01810.1		RING/FYVE/PHD zinc finger superfamily protein	PHD-finger protein
Sapur.004G126200.1	23.53266019	7.232268962	0.248888539	29.05826	1.21E-185	3.23E-184	cyan	0.876354062	4.88E-51		AT1G75700.1	HVA22G	HVA22-like protein G	TB2/DP1, HVA22 family protein
Sapur.001G148300.1	63.1219027	2.946701212	0.101444237	29.0475	1.65E-185	4.40E-184	cyan	0.809064546	1.31E-37		AT1G17430.1		alpha/beta-Hydrolases superfamily protein	alpha/beta hydrolase family protein
Sapur.002G117400.1	336.3536288	1.879425558	0.064750542	29.02563	3.12E-185	8.27E-184	cyan	0.85290578	1.32E-45		AT3G60580.1		C2H2-like zinc finger protein	zinc finger protein, putative
Sapur.017G051500.1	1326.536495	0.787127244	0.027135328	29.00747	5.30E-185	1.40E-183	cyan	0.90957629	5.38E-61	GO:0033180,GO:0015991,GO:0015078	AT1G12840.1	ATVHA-C,DET3	vacuolar ATP synthase subunit C (VATC) / V-ATPase C subunit / vacuolar proton pump C subunit (DET3)	vacuolar H+-ATPase subunit C
Sapur.016G246100.1	1393.998309	1.805842542	0.062426473	28.92751	5.38E-184	1.40E-182	cyan	0.836261804	2.72E-42	GO:0055114,GO:0016616,GO:0006694,GO:0003854	AT1G78570.1	ATRHM1,RHM1,ROL1	rhamnose biosynthesis 1	rhamnose biosynthetic enzyme, putative
Sapur.009G097600.1	1445.644693	1.141793359	0.039471694	28.92689	5.48E-184	1.43E-182	cyan	0.908364304	1.44E-60	GO:0016740,GO:0009058	AT4G34640.1	ERG9,SQS1	squalene synthase 1	squalene synthase
Sapur.008G168000.1	20.44621066	4.958997632	0.171444924	28.92473	5.84E-184	1.52E-182	cyan	0.868387223	4.47E-49	GO:0006508,GO:0004190	AT1G03220.1		Eukaryotic aspartyl protease family protein	basic 7S globulin 2 small subunit, putative
Sapur.005G007500.1	749.9120158	1.369429673	0.047514232	28.82146	1.15E-182	2.98E-181	cyan	0.870299594	1.55E-49	GO:0016021	AT1G09330.1			FAM18-like protein, putative
Sapur.006G153100.1	728.1791975	0.911272339	0.03162439	28.81549	1.37E-182	3.54E-181	cyan	0.898793218	2.18E-57	GO:0016192,GO:0016021	AT5G58060.1	ATGP1,ATYKT61,YKT61	SNARE-like superfamily protein	VAMP-like protein YKT62

Sapur.15WG032200.1	975.1921065	1.486746118	0.051651291	28.7843	3.37E-182	8.66E-181	cyan	0.871646011	7.31E-50	GO:0005515	AT3G18060.1		transducin family protein / WD-40 repeat family protein	66 kDa stress protein
Sapur.012G025900.1	37.83667314	3.053710009	0.106127993	28.77384	4.56E-182	1.17E-180	cyan	0.86116287	2.10E-47		AT1G17950.1	ATMYB52,BW52,MYB52	myb domain protein 52	R2R3-Myb transcription factor MYB3, putative
Sapur.016G221300.1	1152.125054	1.367114422	0.047518721	28.77002	5.09E-182	1.30E-180	cyan	0.871264992	9.06E-50		AT3G27930.1			plant/K24A2-2 protein
Sapur.016G243800.1	905.4667889	1.173482116	0.040889993	28.69852	3.98E-181	1.01E-179	cyan	0.901225759	3.64E-58		AT1G53580.1	ETHE1,GLX2-3,GLY3	glyoxalase II 3	hydroxyacylglutathione hydrolase
Sapur.011G006300.1	348.3773088	1.866401994	0.065057802	28.68837	5.33E-181	1.35E-179	cyan	0.890721164	6.04E-55	GO:0016021,GO:0006886,GO:0005783	AT5G42570.1		B-cell receptor-associated 31-like	B-cell receptor-associated-like protein
Sapur.008G063000.1	245.5062205	3.610284805	0.12596625	28.66073	1.18E-180	2.97E-179	cyan	0.835463151	3.83E-42	GO:0005975,GO:0004553	AT1G65610.1	ATGH9A2,KOR2	Six-hairpin glycosidases superfamily protein	endo-1%2C4-beta-glucanase
Sapur.016G074400.1	872.0084066	0.904094293	0.03160453	28.60648	5.58E-180	1.40E-178	cyan	0.900200716	7.77E-58	GO:0055114,GO:0016616,GO:0006694,GO:0003854	AT3G53520.1	ATUXS1,UXS1	UDP-glucuronic acid decarboxylase 1	UDP-glucuronate decarboxylase
Sapur.010G069300.1	3692.20214	0.869117012	0.030387027	28.60158	6.42E-180	1.61E-178	cyan	0.899950002	9.34E-58	GO:0005525,GO:0003924	AT1G14830.1	ADL1C,ADL5,DL1C,DRP1C	DYNAMIN-like 1C	dynamin-related protein 1C
Sapur.012G076400.1	31.19863438	6.96874764	0.244153961	28.54243	3.49E-179	8.71E-178	cyan	0.859423912	5.14E-47					transmembrane protein, putative
Sapur.007G026700.1	2026.530301	1.04537412	0.036738861	28.45418	4.33E-178	1.07E-176	cyan	0.888795465	2.16E-54	GO:0016787	AT4G36760.1	APP1,ATAPP1	aminopeptidase P1	Xaa-pro aminopeptidase
Sapur.006G111500.1	28.80493715	5.530353722	0.194384704	28.45056	4.80E-178	1.19E-176	cyan	0.811286038	5.81E-38	GO:0004857,GO:0042545,GO:0030599,GO:0005618	AT5G20860.1		Plant invertase/pectin methylesterase inhibitor superfamily	pectinesterase/pectinesterase inhibitor, putative
Sapur.008G107600.1	8261.02188	1.753086541	0.06190839	28.31743	2.11E-176	5.13E-175	cyan	0.83798511	1.28E-42	GO:0071704,GO:0016868,GO:0005975	AT1G70730.1		Phosphoglucomutase/phosphomannomutase family protein	cytoplasmic phosphoglucomutase
Sapur.006G196500.1	161.011016	2.27221326	0.080319261	28.28977	4.62E-176	1.12E-174	cyan	0.820717149	1.62E-39	GO:0043565,GO:0008270,GO:0006355,GO:0003700	AT5G25830.1	GATA12	GATA transcription factor 12	GATA transcription factor
Sapur.15WG135400.1	19.10210892	4.715137851	0.166725672	28.28081	5.95E-176	1.44E-174	cyan	0.869572093	2.33E-49					transmembrane protein, putative
Sapur.019G051100.1	551.0054427	1.25780646	0.044596796	28.20396	5.23E-175	1.26E-173	cyan	0.887920615	3.84E-54		AT1G65320.1		Cystathionine beta-synthase (CBS) family protein	CBS domain protein CBSX6
Sapur.012G063600.1	1350.315075	0.940864327	0.03341522	28.15676	1.98E-174	4.74E-173	cyan	0.887440776	5.24E-54	GO:0005515	AT3G11330.1	PIRL9	plant intracellular ras group-related LRR 9	plant intracellular Ras-group-related LRR protein
Sapur.017G125600.1	278.2350081	2.29625752	0.081648652	28.12364	5.04E-174	1.20E-172	cyan	0.893469957	9.35E-56		AT1G65840.1	ATPAO4,PAO4	polyamine oxidase 4	polyamine oxidase, putative
Sapur.15WG079600.1	45.54522724	7.747957312	0.275633352	28.10965	7.47E-174	1.78E-172	cyan	0.720863799	1.85E-26	GO:0006468,GO:0004672	AT5G61350.1		Protein kinase superfamily protein	receptor-like kinase, putative
Sapur.004G122900.1	2965.451612	1.242887541	0.044217399	28.10856	7.70E-174	1.83E-172	cyan	0.876141061	5.53E-51	GO:0033177,GO:0015991,GO:0015078	AT4G34720.1	ATVHA-C1,AVA-P1,VHA-C1	ATPase, F0/V0 complex, subunit C protein	NA
Sapur.007G032300.1	98.5446508	4.122439817	0.146682467	28.10452	8.63E-174	2.05E-172	cyan	0.737112022	3.59E-28					NA
Sapur.001G017600.1	56.75109748	8.495831434	0.302493389	28.08601	1.45E-173	3.45E-172	cyan	0.701695915	1.37E-24	GO:0005516,GO:0046872,GO:0000166	AT2G41560.1	ACA4	autoinhibited Ca(2+)-ATPase, isoform 4	calcium-transporting ATPase 4, plasma membrane-type protein
Sapur.016G278700.1	250.5342833	1.529734359	0.054801184	27.91426	1.79E-171	4.19E-170	cyan	0.881166754	2.73E-52					hypothetical protein
Sapur.018G094000.1	22.66665917	7.240297912	0.259390734	27.91271	1.87E-171	4.38E-170	cyan	0.821841341	1.04E-39		AT4G32060.1		calcium-binding EF hand family protein	NA
Sapur.002G064500.1	61.20113349	3.682717604	0.132001945	27.89896	2.75E-171	6.41E-170	cyan	0.786957501	2.53E-34	GO:0008270,GO:0005515	AT3G19910.1		RING/U-box superfamily protein	zinc-RING finger protein
Sapur.009G110000.1	4931.92356	0.965157732	0.0346156	27.88216	4.39E-171	1.02E-169	cyan	0.891196798	4.39E-55	GO:0046034,GO:0015992,GO:0033178,GO:0016820,GO:0015991,GO:0005524	AT4G38510.5		ATPase, V1 complex, subunit B protein	V-type proton ATPase subunit B
Sapur.019G061300.1	767.3096564	2.377221172	0.085510017	27.8005	4.28E-170	9.87E-169	cyan	0.798937439	4.72E-36		AT3G56360.1			hypothetical protein
Sapur.008G157000.1	3717.764399	0.881005124	0.031707298	27.78556	6.48E-170	1.49E-168	cyan	0.901462287	3.05E-58	GO:0055085,GO:0005741	AT3G01280.1	ATVDAC1,VDAC1	voltage dependent anion channel 1	voltage-dependent anion-selective channel protein, putative
Sapur.010G164700.1	1549.199605	0.717319891	0.025832057	27.76859	1.04E-169	2.39E-168	cyan	0.899725823	1.10E-57	GO:0005525	AT3G46060.1	ARA-3.ARA3.ATRAB8A.ATRA	RAB GTPase homolog 8A	Ras small GTPase family Ras protein
Sapur.009G065800.1	40.11626971	3.429703741	0.123755028	27.71365	4.78E-169	1.09E-167	cyan	0.84897457	8.70E-45	GO:0046983,GO:0003677	AT5G60440.1	AGL62	AGAMOUS-like 62	MADS box protein, putative
Sapur.004G132400.1	37.86330357	3.290793357	0.119064442	27.63876	3.81E-168	8.61E-167	cyan	0.832743132	1.22E-41		AT2G16920.1	PFU2,UBC23	ubiquitin-conjugating enzyme 23	NA
Sapur.014G033500.1	197.9085146	2.645047516	0.095929343	27.57287	2.35E-167	5.28E-166	cyan	0.814358116	1.85E-38	GO:0004857	AT5G46940.1		Plant invertase/pectin methylesterase inhibitor superfamily protein	pectinesterase inhibitor, putative
Sapur.013G104000.1	900.0648979	0.935282504	0.033956445	27.54359	5.28E-167	1.18E-165	cyan	0.89396098	6.67E-56	GO:0045454	AT1G35620.1	ATPDI8.ATPDIL5-2.PDI8.PDI	PDI-like 5-2	protein disulfide isomerase-like protein, putative
Sapur.006G179900.1	26.8664682	4.14818952	0.150770044	27.51335	1.22E-166	2.70E-165	cyan	0.75599018	2.51E-30	GO:0008270,GO:0005515	AT5G08139.1		RING/U-box superfamily protein	NA
Sapur.001G174700.1	366.0137058	1.64212077	0.059869792	27.4282	1.26E-165	2.79E-164	cyan	0.860369485	3.16E-47	GO:0046488,GO:0016307	AT1G60890.1		Phosphatidylinositol-4-phosphate 5-kinase family protein	phosphatidylinositol-4-phosphate 5-kinase
Sapur.006G179800.1	25.8198339	4.112337749	0.150113316	27.39489	3.16E-165	6.95E-164	cyan	0.759862887	8.58E-31	GO:0008270,GO:0005515	AT5G08139.1		RING/U-box superfamily protein	RING finger protein
Sapur.T120100.1	24.71856554	3.584852938	0.130985133	27.3684	6.52E-165	1.43E-163	cyan	0.846780541	2.43E-44	GO:0005681,GO:0000398,GO:0000387	AT1G54385.2		ARM repeat superfamily protein	NA
Sapur.006G199500.1	72.14975234	1.412315334	0.051895667	27.21451	4.37E-163	9.49E-162	cyan	0.880244518	4.79E-52		AT4G32750.1			transmembrane protein, putative
Sapur.002G070500.1	482.686297	1.515008862	0.055699381	27.19974	6.54E-163	1.41E-161	cyan	0.871532475	7.79E-50		AT4G08180.2	ORP1C	OSBP(oxysterol binding protein)-related protein 1C	oxysterol-binding protein-related protein 1C
Sapur.018G029400.1	534.3401499	0.845547746	0.031229777	27.07505	1.94E-161	4.13E-160	cyan	0.893454245	9.45E-56		AT4G32680.1			transmembrane protein, putative
Sapur.016G295100.1	272.7920319	1.893385332	0.069975085	27.05799	3.08E-161	6.54E-160	cyan	0.818061491	4.53E-39	GO:0055085,GO:0016021	AT1G20925.1		Auxin efflux carrier family protein	auxin efflux carrier family protein
Sapur.011G065100.1	5049.889314	1.081956267	0.040103373	26.97918	2.59E-160	5.49E-159	cyan	0.885442742	1.89E-53	GO:0055114,GO:0016616,GO:0006694,GO:0003854	AT1G78570.1	ATRHM1,RHM1,ROL1	rhamnose biosynthesis 1	rhamnose biosynthetic enzyme, putative
Sapur.005G051300.1	436.999336	1.128773345	0.041910421	26.933	9.02E-160	1.90E-158	cyan	0.868225677	4.89E-49	GO:0007034	AT5G63880.1	VPS20.1	SNF7 family protein	vacuolar protein sorting-associated-like protein
Sapur.002G003300.1	141.1843075	3.993001928	0.148338676	26.91814	1.35E-159	2.83E-158	cyan	0.656865812	9.51E-21	GO:0046872,GO:0030001	AT1G06330.1		Heavy metal transport/detoxification superfamily protein	heavy metal-associated domain protein
Sapur.019G079300.1	232.3953455	2.104137639	0.078179453	26.9142	1.50E-159	3.15E-158	cyan	0.850082838	5.14E-45		AT1G22640.1	ATMYB3,MYB3	myb domain protein 3	transcription factor MYB3
Sapur.001G181900.1	24.26077875	7.865330039	0.292769568	26.86526	5.60E-159	1.17E-157	cyan	0.674368513	3.62E-22	GO:0055114,GO:0020037,GO:0016705,GO:0005506	AT4G37370.1	CYP81D8	cytochrome P450, family 81, subfamily D, polypeptide 8	cytochrome P450 family protein
Sapur.004G153200.1	13.08412136	6.675705749	0.248987527	26.81141	2.38E-158	4.94E-157	cyan	0.872812801	3.78E-50	GO:0043565,GO:0006355,GO:0003700	AT3G44460.1	AtbZIP67,DPBF2	Basic-leucine zipper (bZIP) transcription factor family protein	NA
Sapur.003G001100.1	131.1386228	2.091594142	0.078797999	26.54375	3.03E-155	6.19E-154	cyan	0.786801431	2.66E-34	GO:0016020,GO:0008375	AT1G10280.1		Core-2/I-branching beta-1,6-N-acetylglucosaminyltransferase family protein	core-2/I-branching enzyme
Sapur.010G149500.1	1158.776012	0.851612576	0.032088431	26.53955	3.39E-155	6.91E-154	cyan	0.893944035	6.75E-56	GO:0030132,GO:0030130,GO:0016192,GO:0006886,GO:0005198	AT2G40060.1		Clathrin light chain protein	clathrin light chain
Sapur.15WG112200.1	2316.205304	1.805696655	0.068228245	26.46553	2.42E-154	4.92E-153	cyan	0.812492712	3.72E-38		AT3G48530.1	KING1	SNF1-related protein kinase regulatory subunit gamma 1	SNF1-related kinase regulatory subunit gamma
Sapur.006G188200.1	16.44457153	6.568717064	0.248818273	26.39966	1.38E-153	2.80E-152	cyan	0.889307419	1.55E-54		AT2G30130.1	ASL5,LBD12,PCK1	Lateral organ boundaries (LOB) domain family protein	NA
Sapur.002G056000.1	387.2843201	0.788384728	0.029895531	26.37132	2.92E-153	5.90E-152	cyan	0.900395326	6.73E-58		AT1G21370.1			transmembrane protein, putative
Sapur.005G157200.1	1776.240496	1.162508837	0.044113246	26.35283	4.76E-153	9.58E-152	cyan	0.868792148	3.58E-49	GO:0016491	AT4G09670.1		Oxidoreductase family protein	oxidoreductase family protein
Sapur.004G176800.1	176.1918307	1.407847927	0.053482083	26.32373	1.03E-152	2.05E-151	cyan	0.871572941	7.62E-50	GO:0009058,GO:0003824	AT2G38670.1	PECT1	phosphorylethanolamine cytidylyltransferase 1	ethanolamine-phosphate cytidylyltransferase
Sapur.006G220600.1	141.5216319	1.423253638	0.054177208	26.27034	4.19E-152	8.34E-151	cyan	0.878252672	1.59E-51		AT2G02240.1	MEE66	F-box family protein	F-box protein PP2-B1
Sapur.006G111700.1	550.3978077	0.921027113	0.035109612	26.2329	1.12E-151	2.22E-150	cyan	0.894998303	3.24E-56	GO:0008299	AT2G34630.2	GPPS,GPS1	geranyl diphosphate synthase 1	NA
Sapur.004G120300.1	2210.092998	0.985160888	0.03767541	26.14864	1.02E-150	2.00E-149	cyan	0.875024451	1.06E-50	GO:0008234,GO:0006508	AT4G39090.1	RD19,RD19A	Papain family cysteine protease	cysteine protease
Sapur.005G109100.1	1510.006153	0.76027581	0.02910129	26.12516	1.89E-150	3.69E-149	cyan	0.908099686	1.78E-60		AT2G23420.1	NAPRT2	nicotinate phosphoribosyltransferase 2	nicotinate phosphoribosyltransferase family protein
Sapur.003G136100.1	291.3586088	1.061335977	0.040735918	26.05406	1.21E-149	2.35E-148	cyan	0.885503615	1.82E-53		AT1G80630.1		RNI-like superfamily protein	F-box/LRR protein
Sapur.007G007100.1	240.8034243	2.209521469	0.084999417	25.99455	5.71E-149	1.10E-147	cyan	0.815706234	1.11E-38	GO:0016773.GO:0005975.GO:0005524.GO:0046835.GO:0005536.GO:0004396.GO:000167	AT4G37840.1	HKL3	hexokinase-like 3	hexokinase
Sapur.013G098500.1	212.5445448	1.852310988	0.071502955	25.90538	5.79E-148	1.11E-146	cyan	0.794081776	2.45E-35		AT1G10010.1	AAP8,ATAAP8	amino acid permease 8	NA
Sapur.008G100000.1	497.2196704	0.842411734	0.032534973	25.8925	8.09E-148	1.55E-146	cyan	0.908038371	1.87E-60		AT1G15060.1		Uncharacterised conserved protein UCP031088, alpha/beta hydrolase	alpha/beta fold hydrolase
Sapur.007G111700.1	22.90267699	7.145528493	0.276591866	25.8342	3.66E-147	6.97E-146	cyan	0.738786738	2.35E-28	GO:0030247,GO:0006468,GO:0005524,GO:0004672	AT1G67000.1		Protein kinase superfamily protein	tyrosine kinase domain protein
Sapur.008G105400.1	1493.059474	0.678170194	0.026253096	25.83201	3.88E-147	7.38E-146	cyan	0.899000746	1.87E-57		AT2G01470.1	ATSEC12,STL2P	SEC12P-like 2 protein	prolactin regulatory element-binding protein, putative
Sapur.008G073700.1	36.8035313	8.586218068	0.333378339	25.75518	2.82E-146	5.35E-145	cyan	0.680685016	1.05E-22					NA
Sapur.006G203300.1	912.0617972	2.89591855	0.112832161	25.66572	2.82E-145	5.31E-144	cyan	0.741903978	1.06E-28		AT3G24480.1		Leucine-rich repeat (LRR) family protein	NA
Sapur.002G092600.1	1166.928675	2.515185277	0.09811793	25.63431	6.33E-145	1.19E-143	cyan	0.836782708	2.17E-42	GO:0071949	AT1G58440.1	SQE1,XF1	FAD/NAD(P)-binding oxidoreductase family protein	squalene monooxygenase
Sapur.T109700.1	321.2817444	3.717667435	0.145419104	25.56519	3.72E-144	6.93E-143	cyan	0.754925634	3.36E-30		AT3G59500.1		Integral membrane HRF1 family protein	NA
Sapur.15WG151600.1	1688.082717	2.577646423	0.100876105	25.5526	5.14E-144	9.55E-143	cyan	0.859151534	5.90E-47		AT5G52420.1			plant/K24M7-17 protein
Sapur.008G024600.1	1558.136684	1.345237965	0.052673796	25.53904	7.27E-144	1.35E-142	cyan	0.84210809	2.06E-43	GO:0006508,GO:0004185	AT3G10410.1	CPY,SCPL49	SERINE CARBOXYPEPTIDASE-LIKE 49	serine carboxypeptidase
Sapur.002G142700.1	18.05748653	3.699491625	0.145016359	25.51086	1.49E-143	2.76E-142	cyan	0.841883197	2.28E-43	GO:0033926	AT4G09510.1	CINV2	cytosolic invertase 2	neutral/alkaline invertase
Sapur.017G081200.1	10659.40256	1.774352446	0.069800466	25.42035	1.50E-142	2.75E-141	cyan	0.851029892	3.27E-45	GO:0030244,GO:0016866	AT3G02230.1	ATRGP1,RGP1	reversibly glycosylated polypeptide 1	alpha-1%2C4-glucan-protein synthase [UDP-forming] protein, putative
Sapur.003G133900.1	18.25626771	7.040290573	0.277663913	25.35544	7.83E-142	1.43E-140	cyan	0.66919339	9.74E-22	GO:0000160	AT3G04280.1	ARR22,RR22	response regulator 22	extra response regulator
Sapur.010G032200.1	3499.376973	0.72414951	0.028595865	25.32357	1.76E-141	3.20E-140	cyan	0.890797943	5.73E-55		AT1G51980.1		Insulinase (Peptidase family M16) protein	processing peptidase subunit alpha
Sapur.018G084500.1	973.2998772	0.884066833	0.0349134	25.32171	1.84E-141	3.35E-140	cyan	0.867275312	8.21E-49	GO:0016192,GO:0016021	AT5G58060.1	ATGP1,ATYKT61,YKT61	SNARE-like superfamily protein	VAMP-like protein YKT62
Sapur.010G192400.1	1832.594604	0.909424782	0.035969285	25.28337	4.87E-141	8.83E-140	cyan	0.880122065	5.16E-52	GO:0005524,GO:0016831,GO:0008299	AT3G54250.1		GHMP kinase family protein	mevalonate diphosphate decarboxylase
Sapur.007G034800.1	37.73018176	8.698094636	0.344648647	25.23757	1.55E-140	2.80E-139	cyan	0.591921615	3.24E-16					NA
Sapur.013G047700.1	3141.572386	0.731645183	0.02909507	25.14671	1.54E-139	2.74E-138	cyan	0.891876284	2.77E-55	GO:0046961,GO:0033178,GO:0015991,GO:0016747	AT4G11150.1	emb2448.TUF.TUFF.VHA-E1	vacuolar ATP synthase subunit E1	NA
Sapur.T004700.1	386.4382555	-0.757840893	0.030209347	-25.0863	7.02E-139	1.24E-137	cyan	-0.902369941	1.54E-58	GO:0043565,GO:0006355,GO:0003700	AT4G26640.2	AtWRKY20,WRKY20	WRKY family transcription factor family protein	WRKY transcription factor
Sapur.010G015200.1	572.6559889	0.972670974	0.038816391	25.05825	1.42E-138	2.51E-137	cyan	0.871056406	1.02E-49	GO:0016757,GO:0016021	AT4G02500.1	ATXT2,XT2,XXT2	UDP-xylosyltransferase 2	xyloglucan 6-xylosyltransferase
Sapur.006G111900.1	725.0537206	1.069708925	0.042692806	25.05595	1.50E-138	2.65E-137	cyan	0.886001223	1.32E-53	GO:0046983	AT5G58010.1	LRL3	LJRHL1-like 3	DNA-directed RNA polymerase beta chain, putative
Sapur.014G023000.1	142.9424451	2.055207486	0.082071665	25.04162	2.15E-138	3.79E-137	cyan	0.771366857	3.12E-32		AT3G48700.1	ATCXE13,CXE13	carboxyesterase 13	gibberellin receptor GID1, putative
Sapur.013G101700.1	1622.621819	1.45623845	0.05832817	24.9663	1.42E-137	2.48E-136	cyan	0.864036402	4.67E-48		AT1G72100.1		late embryogenesis abundant domain-containing protein / LEA domain-containing protein	late embryogenesis abundant domain protein, putative
Sapur.014G026800.1	647.3035628	0.782483403	0.031390574	24.92734	3.76E-137	6.54E-136	cyan	0.880250433	4.77E-52		AT1G47640.1			seven transmembrane domain protein
Sapur.006G152600.1	66.41942868	1.943796277	0.078037895	24.90836	6.04E-137	1.05E-135	cyan	0.806344547	3.51E-37	GO:0005634	AT2G32070.1		Polynucleotidyl transferase, ribonuclease H-like superfamily protein	CCR4-associated factor, putative
Sapur.016G205700.1	2895.789106	0.713454902	0.028680254	24.87617	1.35E-136	2.33E-135	cyan	0.87903757	9.92E-52	GO:0042626	AT3G58730.1		vacuolar ATP synthase subunit D (VATD) / V-ATPase D subunit / vacuolar proton pump D subunit (VATPD)	vacuolar ATP synthase subunit D, putative
Sapur.15WG136400.1	11.41138677	6.907380961	0.278158184	24.83256	3.99E-136	6.89E-135	cyan	0.762915516	3.63E-31	GO:0004857,GO:0042545,GO:0030599,GO:0005618	AT2G45220.1		Plant invertase/pectin methylesterase inhibitor superfamily	pectinesterase/pectinesterase inhibitor, putative
Sapur.007G036900.1	18.81582199	4.193716788	0.168932587	24.82479	4.84E-136	8.34E-135	cyan	0.821682771	1.11E-39		AT5G66870.1	ASL1,LBD36	ASYMMETRIC LEAVES 2-like 1	asymmetric LEAVES protein, putative
Sapur.004G142200.1	1547.920527	1.087153115	0.043913844	24.7565	2.64E-135	4.51E-134	cyan	0.868920798	3.34E-49	GO:0006122,GO:0005750,GO:0005743	AT3G52730.1		ubiquinol-cytochrome C reductase UQCRX/QCR9-like family protein	ubiquinol-cytochrome C reductase complex protein
Sapur.002G191800.1	818.271672	1.163861933	0.047112219	24.70404	9.68E-135	1.65E-133	cyan	0.866777878	1.08E-48	GO:0046654,GO:0005737,GO:0003934	AT3G07270.1		GTP cyclohydrolase I	GTP cyclohydrolase I
Sapur.012G033400.1	1073.683596	1.211968085	0.04914848	24.65932	2.92E-134	4.96E-133	cyan	0.860606084	2.80E-47	GO:0005515	AT3G18060.1		transducin family protein / WD-40 repeat family protein	66 kDa stress protein
Sapur.008G037400.1	255.4704914	1.515059245	0.061884747	24.48195	2.30E-132	3.84E-131	cyan	0.800349108	2.90E-36		AT3G55140.1		Pectin lyase-like superfamily protein	pectate lyase
Sapur.009G051900.1	218.675224	1.375529091	0.056283093	24.43947	6.51E-132	1.08E-130	cyan	0.809776529	1.01E-37		AT5G12260.1			transferring glycosyl group transferase, putative
Sapur.002G031900.1	15.57615498	6.885090631	0.282010599	24.4143	1.21E-131	1.99E-130	cyan	0.6853833	4.11E-23		AT3G09290.1	TAC1	telomerase activator1	transcriptional regulator superman protein, putative
Sapur.016G257100.1	500.7154877	-0.706761472	0.029018757	-24.3553	5.09E-131	8.39E-130	cyan	-0.883765725	5.46E-53		AT5G53860.2	emb2737,EMB64	embryo defective 2737	NA
Sapur.004G065700.1	35.96151972	3.432688056	0.141348354	24.28531	2.80E-130	4.58E-129	cyan	0.726924057	4.39E-27		AT2G01300.1			NA
Sapur.006G089100.1	536.9092005	0.853324644	0.035179228	24.25649	5.65E-130	9.22E-129	cyan	0.886618778	8.91E-54		AT3G51580.1			transmembrane protein, putative
Sapur.019G017000.2	18.12663324	4.124771479	0.170318052	24.21805	1.44E-129	2.33E-128	cyan	0.791471578	5.82E-35	GO:0007165,GO:0005515,GO:0043531	AT5G17680.1		disease resistance protein (TIR-NBS-LRR class), putative	TIR-NBS-LRR resistance protein, putative
Sapur.016G211800.1	4610.424618	0.856563954	0.035369592	24.21752	1.45E-129	2.36E-128	cyan	0.876001382	6.00E-51	GO:0055114,GO:0050661,GO:0006006,GO:0004345	AT5G40760.1	G6PD6	glucose-6-phosphate dehydrogenase 6	glucose-6-phosphate 1-dehydrogenase
Sapur.018G035300.1	403.339226	1.931300168	0.079816384	24.19679	2.40E-129	3.89E-128	cyan	0.906709515	5.40E-60	GO:0043565,GO:0008270,GO:0006355,GO:0003700	AT5G25830.1	GATA12	GATA transcription factor 12	GATA transcription factor
Sapur.006G115300.1	821.4287165	0.770706473	0.03185353	24.19532	2.49E-129	4.03E-128	cyan	0.880044776	5.40E-52		AT1G19140.1			ubiquinone biosynthesis protein COQ9
Sapur.010G044800.1	98.80451386	2.084474402	0.086206277	24.18008	3.61E-129	5.83E-128	cyan	0.78594952	3.50E-34		AT5G13890.2		Family of unknown function (DUF716)	transmembrane protein, putative
Sapur.004G045400.1	46.75138306	3.139737641	0.130122523	24.12909	1.24E-128	1.99E-127	cyan	0.753491587	4.97E-30		AT2G34540.2			transmembrane protein, putative
Sapur.15WG102600.1	392.1171948	2.666010425	0.110718791	24.07911	4.14E-128	6.60E-127	cyan	0.731910765	1.31E-27		AT4G24730.4		Calcineurin-like metallo-phosphoesterase superfamily protein	component of high affinity nitrate transporter
Sapur.001G165900.1	45.24275118	1.995819328	0.083126978	24.00929	2.22E-127	3.52E-126	cyan	0.853109857	1.20E-45		AT2G20110.2		Tesmin/TSO1-like CXC domain-containing protein	tesmin/TSO1-like CXC protein, putative
Sapur.006G178100.1	730.4120271	0.649791987	0.027080845	23.99452	3.17E-127	5.01E-126	cyan	0.884763133	2.91E-53	GO:0046872	AT2G26210.1		Ankyrin repeat family protein	zinc finger protein, putative
Sapur.010G081400.1	1526.781235	1.220144846	0.050851428	23.99431	3.19E-127	5.03E-126	cyan	0.844446973	7.14E-44	GO:0016021,GO:0006886,GO:0005783	AT3G03160.1			erwinia induced protein
Sapur.017G044500.1	531.1649154	0.968267134	0.040387216	23.97459	5.12E-127	8.05E-126	cyan	0.891761992	3.00E-55	GO:0046923,GO:0016021,GO:0006621	AT3G25040.1	ERD2B	endoplasmic reticulum retention defective 2B	ER lumen protein retaining receptor
Sapur.010G152500.1	24.11040075	2.910785029	0.121488384	23.95937	7.38E-127	1.16E-125	cyan	0.810173068	8.75E-38					NA
Sapur.001G050400.1	203.2873556	1.428677981	0.059673356	23.94164	1.13E-126	1.77E-125	cyan	0.830393017	3.27E-41		AT2G33510.1			Humj1, putative
Sapur.T064700.1	679.6309599	0.806823468	0.033704804	23.93794	1.23E-126	1.93E-125	cyan	0.888162589	3.28E-54	GO:0055114,GO:0016616,GO:0051287,GO:0000287	AT4G35260.1	IDH-I,IDH1	isocitrate dehydrogenase 1	NA
Sapur.007G011400.1	2385.560822	1.17778982	0.049414336	23.83498	1.45E-125	2.25E-124	cyan	0.872901304	3.59E-50		AT3G51160.1	GMD2,MUR1,MUR_1	NAD(P)-binding Rossmann-fold superfamily protein	GDP-mannose 4%2C6-dehydratase
Sapur.014G125900.1	1318.710004	0.84052803	0.035304576	23.80791	2.77E-125	4.28E-124	cyan	0.876227684	5.26E-51	GO:0016746,GO:0008152	AT4G26910.1		Dihydrolipoamide succinyltransferase	2-oxoglutarate dehydrogenase, E2 component, dihydrolipoamide succinyltransferase
Sapur.011G036600.1	848.3531442	1.23161316	0.05174226	23.80285	3.12E-125	4.82E-124	cyan	0.841701837	2.48E-43	GO:0005524	AT4G04910.1	NSF	AAA-type ATPase family protein	vesicle-fusing ATPase
Sapur.011G043500.1	23.19801325	2.352841376	0.09888555	23.79358	3.89E-125	6.00E-124	cyan	0.867311034	8.05E-49		AT4G18490.1			NA
Sapur.003G044800.1	1500.400452	0.620485927	0.026114566	23.76015	8.63E-125	1.33E-123	cyan	0.875492855	8.07E-51	GO:0005789	AT2G45140.1	PVA12	plant VAP homolog 12	vesicle-associated membrane protein, putative
Sapur.010G065000.1	29.38074053	6.893267853	0.291074741	23.68212	5.51E-124	8.38E-123	cyan	0.682795199	6.92E-23		AT3G22800.1		Leucine-rich repeat (LRR) family protein	NA
Sapur.003G084900.1	4712.769184	1.566183757	0.066171643	23.6685	7.61E-124	1.16E-122	cyan	0.866147395	1.51E-48	GO:0008299,GO:0004421	AT4G11820.2	EMB2778.FKP1.HMGS.MVA	hydroxymethylglutaryl-CoA synthase / HMG-CoA synthase / 3-hydroxy-3-methylglutaryl coenzyme A synthase	3-hydroxy-3-methylglutaryl coenzyme A synthase
Sapur.012G013500.1	609.4182253	1.012785398	0.042953963	23.57839	6.42E-123	9.64E-122	cyan	0.862667631	9.60E-48	GO:0006508	AT5G24260.1		prolyl oligopeptidase family protein	dipeptidyl peptidase IV, putative
Sapur.009G113800.1	925.1267151	0.537661633	0.022893076	23.48577	5.70E-122	8.50E-121	cyan	0.892302878	2.08E-55	GO:0016746,GO:0008152	AT5G60620.1	GPAT9	glycerol-3-phosphate acyltransferase 9	1-acyl-sn-glycerol-3-phosphate acyltransferase
Sapur.017G032700.1	231.8930157	0.942164319	0.0401333	23.47587	7.20E-122	1.07E-120	cyan	0.864105874	4.50E-48	GO:0016791	AT2G14110.1		Haloacid dehalogenase-like hydrolase (HAD) superfamily protein	magnesium-dependent phosphatase
Sapur.016G145300.1	227.5305009	1.131532554	0.048255868	23.4486	1.37E-121	2.03E-120	cyan	0.854580223	5.83E-46		AT5G12470.1		Protein of unknown function (DUF3411)	DUF3411 domain protein
Sapur.001G043500.1	422.2118996	0.552059077	0.023548061	23.44393	1.52E-121	2.26E-120	cyan	0.887583316	4.78E-54	GO:0016020,GO:0008375	AT3G24040.1		Core-2/I-branching beta-1,6-N-acetylglucosaminyltransferase family protein	core-2/I-branching beta-1%2C6-N-acetylglucosaminyltransferase family protein
Sapur.018G038300.1	457.3538876	-0.643355076	0.027544882	-23.3566	1.18E-120	1.74E-119	cyan	-0.892673212	1.61E-55		AT1G72820.1		Mitochondrial substrate carrier family protein	carrier protein
Sapur.003G101000.1	5.379992086	5.903864102	0.252943264	23.34067	1.71E-120	2.52E-119	cyan	0.843482435	1.11E-43		AT3G61660.1			NA
Sapur.008G074800.1	780.2160974	1.085452338	0.046535328	23.32534	2.45E-120	3.60E-119	cyan	0.840448447	4.33E-43	GO:0008168	AT2G03480.1	QUL2	QUASIMODO2 LIKE 2	S-adenosyl-L-methionine-dependent methyltransferase, putative
Sapur.017G095500.1	732.4727707	1.161585975	0.049827686	23.31206	3.35E-120	4.91E-119	cyan	0.839497383	6.61E-43	GO:0008270,GO:0005975,GO:0004476,GO:0009298	AT3G02570.1	MEE31,PMI1	Mannose-6-phosphate isomerase, type I	mannose-6-phosphate isomerase
Sapur.016G197500.1	1339.722425	0.844455757	0.036260717	23.28845	5.81E-120	8.49E-119	cyan	0.839591149	6.34E-43	GO:0033180,GO:0015991,GO:0015078	AT1G12840.1	ATVHA-C,DET3	vacuolar ATP synthase subunit C (VATC) / V-ATPase C subunit / vacuolar proton pump C subunit (DET3)	vacuolar H+-ATPase subunit C
Sapur.009G023800.1	220.2184831	1.082050186	0.046525946	23.25692	1.21E-119	1.77E-118	cyan	0.881808776	1.84E-52		AT5G59410.1		Rab5-interacting family protein	Rab5-interacting protein
Sapur.017G067200.1	554.7182822	0.712303656	0.030629081	23.2558	1.24E-119	1.81E-118	cyan	0.877131457	3.09E-51		AT5G16890.1		Exostosin family protein	NA
Sapur.006G141700.1	154.5816533	1.22383124	0.052657599	23.2413	1.74E-119	2.53E-118	cyan	0.863644361	5.74E-48	GO:0008270,GO:0005515	AT2G18670.1		RING/U-box superfamily protein	RING zinc finger protein, putative
Sapur.001G136600.1	906.9086805	0.613796962	0.026499483	23.1626	1.09E-118	1.57E-117	cyan	0.872464606	4.60E-50	GO:0016787	AT1G53710.1		Calcineurin-like metallo-phosphoesterase superfamily protein	serine/threonine phosphatase
Sapur.001G184700.1	319.1862089	1.528412586	0.066008648	23.15473	1.30E-118	1.88E-117	cyan	0.819577434	2.52E-39	GO:0006468,GO:0005524,GO:0004672	AT1G09600.1		Protein kinase superfamily protein	cyclin-dependent kinase-like Serine/Threonine kinase family protein
Sapur.15WG116500.1	470.5033324	2.381042958	0.102927749	23.13315	2.15E-118	3.08E-117	cyan	0.788630701	1.47E-34		AT3G47350.1	ATHSD2,HSD2	hydroxysteroid dehydrogenase 2	short-chain dehydrogenase reductase
Sapur.002G148600.1	570.082453	0.96042087	0.041571683	23.10277	4.34E-118	6.19E-117	cyan	0.845860513	3.73E-44		AT4G01220.1		Nucleotide-diphospho-sugar transferase family protein	alpha 1%2C3-xylosyltransferase, putative
Sapur.017G124300.1	4591.05607	1.381481607	0.059797437	23.10269	4.35E-118	6.20E-117	cyan	0.815180755	1.36E-38	GO:0070569,GO:0008152	AT5G17310.2	AtUGP2,UGP2	UDP-glucose pyrophosphorylase 2	UTP-glucose-1-phosphate uridylyltransferase
Sapur.004G027600.1	242.2458589	1.605566241	0.069567304	23.07932	7.47E-118	1.06E-116	cyan	0.812408833	3.83E-38		AT4G21720.1			hypothetical protein
Sapur.017G000700.1	6138.237006	0.480255805	0.020815005	23.07258	8.73E-118	1.24E-116	cyan	0.879565945	7.22E-52		AT2G44100.1	AT-GDI1,ATGDI1,GDI1	guanosine nucleotide diphosphate dissociation inhibitor 1	Rab GDP dissociation inhibitor
Sapur.15ZG034900.1	364.8047765	0.927424693	0.040202213	23.069	9.48E-118	1.34E-116	cyan	0.846975901	2.22E-44		AT1G18190.1	GC2	golgin candidate 2	coiled-coil vesicle tethering protein, putative
Sapur.010G010800.1	13.61133422	3.982619252	0.172671454	23.06472	1.05E-117	1.48E-116	cyan	0.772534947	2.20E-32	GO:0008138,GO:0006470	AT3G23610.2	DSPTP1	dual specificity protein phosphatase 1	NA
Sapur.016G252400.1	356.6571889	0.997736472	0.043264244	23.06146	1.13E-117	1.60E-116	cyan	0.874451169	1.48E-50	GO:0016020,GO:0008378,GO:0006486	AT1G53290.1		Galactosyltransferase family protein	beta-1%2C3-galactosyltransferase, putative
Sapur.013G035000.1	93.29146694	1.949773996	0.084573129	23.0543	1.33E-117	1.88E-116	cyan	0.799514536	3.87E-36		AT4G14880.3	ATCYS-3A.CYTACS1.OASA1.(	O-acetylserine (thiol) lyase (OAS-TL) isoform A1	cysteine synthase
Sapur.010G028200.1	10.80621143	3.119304589	0.135380256	23.04106	1.81E-117	2.55E-116	cyan	0.875430592	8.37E-51		AT1G60460.1			NA
Sapur.012G060800.1	185.2970428	0.926652774	0.04037396	22.95174	1.42E-116	1.97E-115	cyan	0.859500408	4.94E-47		AT1G10650.1		SBP (S-ribonuclease binding protein) family protein	S-ribonuclease-binding protein SBP1, putative
Sapur.011G008300.1	395.0318181	0.799911641	0.034864672	22.94333	1.72E-116	2.39E-115	cyan	0.871161324	9.60E-50	GO:0016020,GO:0008375	AT1G11940.1		Core-2/I-branching beta-1,6-N-acetylglucosaminyltransferase family protein	core-2/I-branching beta-1%2C6-N-acetylglucosaminyltransferase family protein
Sapur.002G171600.1	692.8635883	0.872767472	0.038100485	22.90699	3.96E-116	5.48E-115	cyan	0.868623872	3.93E-49	GO:0003676	AT3G07810.1		RNA-binding (RRM/RBD/RNP motifs) family protein	RNA recognition motif 1 in heterogeneous nuclear ribonucleoprotein A subfamily protein
Sapur.006G021000.1	43.45665749	2.298165513	0.1004946	22.86855	9.56E-116	1.32E-114	cyan	0.780024132	2.26E-33	GO:0008270	AT3G56930.2		DHHC-type zinc finger family protein	zinc finger protein, putative
Sapur.15WG140300.1	638.7926822	1.428500897	0.062529168	22.84535	1.63E-115	2.24E-114	cyan	0.779169246	2.95E-33		AT5G51640.1	TBL17,YLS7	Plant protein of unknown function (DUF828)	Pmr5/Cas1p GDSL/SGNH-like acyl-esterase family protein
Sapur.008G142900.1	68.72277905	1.694316568	0.074187145	22.83841	1.91E-115	2.62E-114	cyan	0.833506295	8.84E-42	GO:0015450,GO:0006886,GO:0005744	AT1G20350.1	ATTIM17-1,TIM17-1	translocase inner membrane subunit 17-1	import inner membrane translocase subunit Tim17
Sapur.001G113700.1	964.6649906	-0.66626432	0.029249629	-22.7786	7.48E-115	1.02E-113	cyan	-0.878050465	1.79E-51		AT2G35490.1		Plastid-lipid associated protein PAP / fibrillin family protein	NA
Sapur.005G136800.1	103.5658102	1.306941441	0.057631449	22.67757	7.46E-114	1.01E-112	cyan	0.840523882	4.19E-43					hypothetical protein
Sapur.008G007600.1	203.7331078	3.419227702	0.150815398	22.67161	8.54E-114	1.15E-112	cyan	0.753956698	4.38E-30	GO:0016627,GO:0016021,GO:0006629,GO:0005737	AT5G16010.1		3-oxo-5-alpha-steroid 4-dehydrogenase family protein	3-oxo-5-alpha-steroid 4-dehydrogenase
Sapur.009G119300.1	3537.654225	0.904867698	0.039915739	22.66945	8.97E-114	1.21E-112	cyan	0.855970733	2.93E-46	GO:0055114,GO:0016616,GO:0006694,GO:0003854	AT1G08200.1	AXS2	UDP-D-apiose/UDP-D-xylose synthase 2	UDP-D-apiose/UDP-D-xylose synthase
Sapur.018G085200.1	39.90814806	3.423703342	0.151109076	22.65717	1.19E-113	1.60E-112	cyan	0.668194607	1.18E-21	GO:0006468,GO:0004672,GO:0005975,GO:0004553	AT4G23180.1	CRK10,RLK4	cysteine-rich RLK (RECEPTOR-like protein kinase) 10	glycoside hydrolase family 18 protein
Sapur.014G036000.1	692.9777918	-0.976046359	0.043097313	-22.6475	1.48E-113	1.99E-112	cyan	-0.86703351	9.36E-49		AT3G60320.1		Protein of unknown function (DUF630 and DUF632)	BZIP transcription factor bZIP80, putative
Sapur.005G174000.1	1114.685725	1.06284653	0.046986022	22.62048	2.72E-113	3.66E-112	cyan	0.879211167	8.94E-52	GO:0016757,GO:0047262	AT1G06780.1	GAUT6	galacturonosyltransferase 6	glycosyltransferase family 8 protein
Sapur.011G058500.1	660.3443786	-0.824434832	0.036457964	-22.6133	3.21E-113	4.30E-112	cyan	-0.885016218	2.48E-53		AT1G29950.2		basic helix-loop-helix (bHLH) DNA-binding superfamily protein	transcription factor bHLH144
Sapur.014G123600.1	212.395472	1.091153451	0.04826013	22.60983	3.47E-113	4.65E-112	cyan	0.847417502	1.81E-44	GO:0030150,GO:0005744	AT3G59280.1	TXR1	Protein Transporter, Pam16	import inner membrane translocase subunit TIM16
Sapur.001G018800.1	7.312522034	6.337020295	0.280380818	22.60148	4.19E-113	5.61E-112	cyan	0.744578373	5.31E-29	GO:0005515				F-box protein
Sapur.014G088400.1	47.33906159	1.855278676	0.082148566	22.58443	6.16E-113	8.23E-112	cyan	0.848505775	1.09E-44		AT2G46980.2			hypothetical protein
Sapur.010G184300.1	1004.959406	0.946021554	0.041990435	22.52945	2.14E-112	2.83E-111	cyan	0.853276886	1.10E-45	GO:0005515	AT5G04420.3		Galactose oxidase/kelch repeat superfamily protein	acyl-CoA-binding domain protein
Sapur.009G120200.1	476.4959657	1.079731889	0.047936449	22.52424	2.40E-112	3.18E-111	cyan	0.82395119	4.51E-40	GO:0016020	AT2G36300.1		Integral membrane Yip1 family protein	membrane protein sb140
Sapur.016G145200.1	1904.856714	0.976077408	0.043351752	22.51529	2.94E-112	3.89E-111	cyan	0.867966819	5.63E-49	GO:0050662,GO:0003824	AT5G19440.1		NAD(P)-binding Rossmann-fold superfamily protein	cinnamyl alcohol dehydrogenase
Sapur.009G099300.1	3583.976482	0.836099	0.037139923	22.51214	3.16E-112	4.18E-111	cyan	0.83859138	9.85E-43	GO:0033177,GO:0015991,GO:0015078	AT4G34720.1	ATVHA-C1,AVA-P1,VHA-C1	ATPase, F0/V0 complex, subunit C protein	V-type proton ATPase 16 kDa proteolipid subunit c2
Sapur.014G022600.1	188.7682746	0.829717953	0.036904256	22.48299	6.09E-112	8.02E-111	cyan	0.878235142	1.60E-51		AT4G10130.1		DNAJ heat shock N-terminal domain-containing protein	DnaJ heat shock amino-terminal domain protein
Sapur.006G163900.1	10.20007539	3.412734228	0.151954468	22.45893	1.05E-111	1.37E-110	cyan	0.841321062	2.94E-43	GO:0006468,GO:0004672	AT5G04870.1	ATCPK1,CPK1	calcium dependent protein kinase 1	calcium-dependent kinase
Sapur.011G093600.1	33.16582349	4.331579837	0.192889439	22.45628	1.11E-111	1.46E-110	cyan	0.683140979	6.46E-23	GO:0048544,GO:0006468,GO:0005524,GO:0004672	AT4G27290.1		S-locus lectin protein kinase family protein	NA
Sapur.010G089100.1	6545.797424	0.99031477	0.044110593	22.45072	1.26E-111	1.65E-110	cyan	0.843501538	1.10E-43	GO:0016887.GO:0006754.GO:0005524.GO:0000275.GO:0046034.GO:0015992.GO:003317	AT5G08680.1		ATP synthase alpha/beta family protein	ATP synthase F1, beta subunit
Sapur.004G030100.1	142.4052031	0.834871661	0.037190372	22.4486	1.32E-111	1.73E-110	cyan	0.889146362	1.72E-54		AT1G28250.1			NA
Sapur.005G067300.1	3127.649436	0.762339908	0.033966647	22.44378	1.47E-111	1.92E-110	cyan	0.853886182	8.20E-46		AT2G21870.1	MGP1	copper ion binding;cobalt ion binding;zinc ion binding	ATP synthase
Sapur.15WG072300.1	7695.70115	0.670402989	0.029878442	22.43768	1.69E-111	2.21E-110	cyan	0.871827765	6.60E-50	GO:0046961,GO:0046933,GO:0045261,GO:0015986	AT2G33040.1	ATP3	gamma subunit of Mt ATP synthase	ATP synthase F1, gamma subunit
Sapur.013G019800.1	53.88122586	2.495733051	0.111250822	22.43339	1.86E-111	2.43E-110	cyan	0.759351914	9.90E-31	GO:0031625,GO:0006511	AT4G02570.2	ATCUL1,AXR6,CUL1	cullin 1	NA
Sapur.017G088100.1	17522.49613	2.165842283	0.096799974	22.37441	6.99E-111	9.08E-110	cyan	0.877238286	2.90E-51		AT5G38760.1		Late embryogenesis abundant protein (LEA) family protein	ABA-inducible-like protein
Sapur.010G066300.1	6.446986145	5.78396037	0.258557957	22.37007	7.70E-111	1.00E-109	cyan	0.810877002	6.76E-38		AT3G22880.1	ARLIM15,ATDMC1,DMC1	DNA repair (Rad51) family protein	NA
Sapur.008G063300.1	471.1485038	1.203115072	0.053814655	22.35664	1.04E-110	1.35E-109	cyan	0.81814087	4.39E-39		AT5G16300.1		Vps51/Vps67 family (components of vesicular transport) protein	NA
Sapur.016G104800.1	1369.535538	1.405068395	0.062894529	22.34007	1.51E-110	1.95E-109	cyan	0.820776448	1.58E-39	GO:0030976,GO:0003824,GO:0000287	AT4G33070.1		Thiamine pyrophosphate dependent pyruvate decarboxylase family protein	pyruvate decarboxylase
Sapur.014G015500.1	15.73318168	3.744118667	0.16771649	22.32409	2.16E-110	2.79E-109	cyan	0.7797481	2.46E-33	GO:0046983	AT1G49770.1	RGE1,ZOU	basic helix-loop-helix (bHLH) DNA-binding superfamily protein	helix loop helix protein
Sapur.014G098300.1	1713.458238	2.029892809	0.090934708	22.32253	2.23E-110	2.89E-109	cyan	0.835577638	3.65E-42	GO:0005783,GO:0045454	AT2G47470.1	ATPDI11.ATPDIL2-1.MEE30.	thioredoxin family protein	protein disulfide isomerase-like protein
Sapur.005G130700.1	914.267653	3.716246066	0.166615425	22.30433	3.35E-110	4.32E-109	cyan	0.626712458	1.64E-18	GO:0005975,GO:0004553	AT1G78060.1		Glycosyl hydrolase family protein	xylan 1 4-beta-xylosidase
Sapur.011G041800.1	1383.481606	2.498476127	0.112207036	22.26666	7.78E-110	9.98E-109	cyan	0.749606568	1.41E-29	GO:0016758,GO:0008152	AT5G54010.1		UDP-Glycosyltransferase superfamily protein	anthocyanidin 3-O-glucosyltransferase
Sapur.019G099600.1	501.5081909	1.306218398	0.058782448	22.22123	2.14E-109	2.74E-108	cyan	0.845819218	3.80E-44	GO:0016021,GO:0015031	AT2G20840.1		Secretory carrier membrane protein (SCAMP) family protein	secretory carrier-associated membrane protein
Sapur.016G156900.1	405.5477675	0.819602338	0.036949111	22.18192	5.13E-109	6.53E-108	cyan	0.85649211	2.26E-46		AT5G48020.1		2-oxoglutarate (2OG) and Fe(II)-dependent oxygenase superfamily protein	NA
Sapur.007G111900.1	12.31449959	3.295421369	0.148701389	22.16134	8.11E-109	1.03E-107	cyan	0.802122997	1.56E-36		AT1G66980.1	SNC4	suppressor of npr1-1 constitutive 4	NA
Sapur.013G054100.1	128.8623734	1.098868273	0.049591046	22.1586	8.62E-109	1.09E-107	cyan	0.846902026	2.30E-44		AT5G18150.1		Methyltransferase-related protein	transmembrane protein, putative
Sapur.012G030800.1	363.5170084	3.299430523	0.148977114	22.14723	1.11E-108	1.40E-107	cyan	0.787657415	2.02E-34	GO:0016853,GO:0005975	AT3G17940.1		Galactose mutarotase-like superfamily protein	aldose 1-epimerase
Sapur.005G072400.1	2330.905766	0.602094776	0.027201433	22.13467	1.47E-108	1.85E-107	cyan	0.877480561	2.51E-51	GO:0008152,GO:0003824,GO:0048037	AT5G23250.1		Succinyl-CoA ligase, alpha subunit	succinyl-CoA ligase [ADP-forming] subunit alpha, putative
Sapur.002G092400.1	1680.838112	0.977731131	0.044236793	22.10222	3.01E-108	3.79E-107	cyan	0.866908175	1.00E-48		AT4G07960.1	ATCSLC12,CSLC12	Cellulose-synthase-like C12	cellulose synthase catalytic subunit
Sapur.001G091500.1	712.2036718	1.093832535	0.049634475	22.03776	1.25E-107	1.57E-106	cyan	0.847186361	2.01E-44		AT1G63000.1	NRS/ER,UER1	nucleotide-rhamnose synthase/epimerase-reductase	3%2C5-epimerase/4-reductase
Sapur.15WG091700.1	921.0814941	1.325395298	0.060150005	22.03483	1.34E-107	1.67E-106	cyan	0.828450281	7.30E-41		AT3G48030.1		hypoxia-responsive family protein / zinc finger (C3HC4-type RING finger) family protein	RING-H2 finger protein ATL3I
Sapur.007G073700.1	1160.391185	0.785970254	0.035744003	21.98887	3.68E-107	4.59E-106	cyan	0.875074705	1.03E-50		AT4G09810.1		Nucleotide-sugar transporter family protein	nucleotide sugar transporter protein
Sapur.013G064900.1	3820.89109	0.828009362	0.03766171	21.98544	3.97E-107	4.95E-106	cyan	0.849326542	7.37E-45	GO:0016820,GO:0015991,GO:0000221,GO:0046961	AT3G42050.1		vacuolar ATP synthase subunit H family protein	vacuolar proton ATPase subunit H
Sapur.013G102400.1	14.56881915	2.536335895	0.115382183	21.98204	4.28E-107	5.33E-106	cyan	0.819345271	2.76E-39	GO:0005515	AT1G35470.2		SPla/RYanodine receptor (SPRY) domain-containing protein	Ran-binding protein in the microtubule-organising centre protein
Sapur.016G042000.1	713.6463419	1.236691762	0.056290894	21.96966	5.62E-107	6.99E-106	cyan	0.852920856	1.31E-45		AT2G05990.1	ENR1,MOD1	NAD(P)-binding Rossmann-fold superfamily protein	NA
Sapur.15ZG047800.1	5.615338855	5.785697805	0.264388251	21.88334	3.74E-106	4.61E-105	cyan	0.801843777	1.72E-36		AT1G73860.1		P-loop containing nucleoside triphosphate hydrolases superfamily protein	NA
Sapur.003G012100.1	11.12844178	4.655781385	0.213074634	21.85047	7.69E-106	9.45E-105	cyan	0.753343523	5.17E-30	GO:0031047	AT5G23570.1	ATSGS3,SGS3	XS domain-containing protein / XS zinc finger domain-containing protein-related	suppressor OF protein silencing protein, putative
Sapur.15ZG050700.1	203.7600549	1.379158273	0.063483962	21.72451	1.20E-104	1.46E-103	cyan	0.840601458	4.05E-43		AT3G18215.1		Protein of unknown function, DUF599	transmembrane protein, putative
Sapur.003G108000.1	1586.515127	1.295829093	0.059656028	21.72168	1.28E-104	1.55E-103	cyan	0.799859816	3.43E-36	GO:0016021,GO:0015689,GO:0015098	AT1G64650.1		Major facilitator superfamily protein	MFS transporter
Sapur.001G184000.1	31.90970463	1.928380211	0.088801317	21.71567	1.46E-104	1.76E-103	cyan	0.824671981	3.38E-40		AT1G09645.1			transmembrane protein, putative
Sapur.002G199300.1	3915.443009	0.634598246	0.029284198	21.67033	3.91E-104	4.69E-103	cyan	0.854986145	4.77E-46		AT2G20360.1		NAD(P)-binding Rossmann-fold superfamily protein	NADH dehydrogenase [ubiquinone] 1 alpha subcomplex subunit 9
Sapur.T053100.1	3308.714173	0.500847589	0.023112582	21.66991	3.95E-104	4.73E-103	cyan	0.870489405	1.40E-49	GO:0008152,GO:0003824	AT2G20420.1		ATP citrate lyase (ACL) family protein	succinyl-CoA ligase [GDP-forming] subunit beta
Sapur.005G150700.1	2233.076513	0.998352202	0.046103182	21.65474	5.48E-104	6.57E-103	cyan	0.85347113	1.00E-45	GO:0042578,GO:0042132,GO:0005975	AT1G43670.1		Inositol monophosphatase family protein	fructose-1%2C6-bisphosphatase, cytosolic protein
Sapur.003G124100.1	548.7201431	0.870681682	0.040257448	21.62784	9.83E-104	1.17E-102	cyan	0.846757825	2.46E-44	GO:0006468,GO:0005524,GO:0004672	AT5G13160.1	PBS1	Protein kinase superfamily protein	NA
[illegible]	[illegible]	[illegible]	[illegible]	[illegible]	[illegible]	[illegible]	cyan	[illegible]	[illegible]	[illegible]	[illegible]	[illegible]	[illegible]	[illegible]

Sapur.018G008000.1	738.1219258	0.59232267	0.028862799	20.52201	1.37E-93	1.44E-92	cyan	0.860209829	3.44E-47		AT2G24640.1	UBP19	ubiquitin-specific protease 19	ubiquitin carboxyl-terminal hydrolase
Sapur.005G012000.1	14.91428316	3.627401291	0.176807196	20.51614	1.55E-93	1.63E-92	cyan	0.725376014	6.36E-27	GO:0048544,GO:0006468,GO:0004672	AT4G21390.1	B120	S-locus lectin protein kinase family protein	NA
Sapur.016G296300.1	241.3563938	1.207178745	0.058881072	20.50198	2.07E-93	2.17E-92	cyan	0.813052415	3.02E-38		AT5G65950.1			gryzun, trafficking through protein%2C putative
Sapur.003G068000.1	101.7400095	1.502975327	0.073322425	20.49817	2.24E-93	2.35E-92	cyan	0.82178464	1.06E-39					hypothetical protein
Sapur.008G044400.1	44.58376619	1.813645882	0.088604042	20.46911	4.06E-93	4.25E-92	cyan	0.822454293	8.17E-40	GO:0005515	AT3G55470.2		Calcium-dependent lipid-binding (CaLB domain) family protein	elicitor-responsive protein
Sapur.007G049900.1	176.0982797	-1.351570854	0.066035483	-20.4673	4.21E-93	4.40E-92	cyan	-0.880836377	3.34E-52	GO:0005515	AT5G67385.1		Phototropic-responsive NPH3 family protein	root phototropism protein, putative
Sapur.010G034600.1	5107.1396	0.714384383	0.03490894	20.46422	4.49E-93	4.69E-92	cyan	0.848369233	1.16E-44	GO:0055085,GO:0005741	AT3G01280.1	ATVDAC1,VDAC1	voltage dependent anion channel 1	NA
Sapur.007G003500.1	2305.214506	0.753852011	0.036930073	20.41296	1.28E-92	1.33E-91	cyan	0.845050007	5.41E-44	GO:0022900,GO:0016651	AT5G67590.1	FRO1	NADH-ubiquinone oxidoreductase-related	NADH-ubiquinone oxidoreductase 18 kDa subunit
Sapur.004G063100.1	264.1249584	1.046094388	0.051252894	20.41045	1.35E-92	1.40E-91	cyan	0.788741274	1.42E-34	GO:0005515	AT3G03440.1		ARM repeat superfamily protein	ubiquitin-protein ligase
Sapur.016G022700.1	72.61797639	1.200452579	0.058898207	20.38182	2.42E-92	2.51E-91	cyan	0.849964439	5.44E-45	GO:0055085,GO:0016021	AT5G13750.1	ZIFL1	zinc induced facilitator-like 1	carbohydrate transporter, putative
Sapur.002G076900.1	2609.632467	1.439255143	0.070836741	20.31792	8.93E-92	9.18E-91	cyan	0.851346251	2.81E-45	GO:0005544,GO:0005509	AT1G35720.1	ANNAT1,ATOXY5,OXY5	annexin 1	annexin AnxGb3
Sapur.012G089000.1	55.75383181	1.578361762	0.077926908	20.25439	3.25E-91	3.32E-90	cyan	0.801285903	2.09E-36		AT3G05320.1		O-fucosyltransferase family protein	GDP-fucose protein O-fucosyltransferase
Sapur.001G154600.1	55.0344391	2.027604974	0.100136431	20.24842	3.67E-91	3.74E-90	cyan	0.799813868	3.49E-36	GO:0016021,GO:0015116,GO:0008272,GO:0055085,GO:0016020,GO:0008271	AT3G15990.1	SULTR3;4	sulfate transporter 3;4	sulfate/bicarbonate/oxalate exchanger and transporter sat-1
Sapur.011G073000.1	117.7603913	-0.849672998	0.042026833	-20.2174	6.88E-91	6.99E-90	cyan	-0.84616035	3.24E-44	GO:0006468,GO:0005524,GO:0004672	AT1G16670.1		Protein kinase superfamily protein	tyrosine kinase domain protein
Sapur.011G117000.1	416.3878407	0.822831885	0.040707608	20.21322	7.49E-91	7.59E-90	cyan	0.842370207	1.83E-43	GO:0016491,GO:0008152	AT4G20760.1		NAD(P)-binding Rossmann-fold superfamily protein	short-chain dehydrogenase/reductase
Sapur.010G193400.1	194.9475072	0.923296345	0.045755177	20.17906	1.50E-90	1.51E-89	cyan	0.823196953	6.09E-40		AT2G16710.1		Iron-sulphur cluster biosynthesis family protein	iron-sulfur cluster assembly protein IscA
Sapur.001G115900.1	83.27576843	1.431085502	0.070929455	20.17618	1.59E-90	1.60E-89	cyan	0.809731781	1.03E-37	GO:0052726,GO:0052725,GO:0047325,GO:0032957,GO:0005622,GO:0005524,GO:000028	AT5G16760.1		Inositol 1,3,4-trisphosphate 5/6-kinase family protein	NA
Sapur.006G066200.1	535.3000787	0.590539499	0.029302362	20.15331	2.52E-90	2.53E-89	cyan	0.846040702	3.43E-44		AT4G27690.1	VPS26B	vacuolar protein sorting 26B	vacuolar protein sorting-associated protein
Sapur.004G048200.1	6.72176363	6.058932588	0.300665154	20.15176	2.60E-90	2.61E-89	cyan	0.661543951	4.06E-21		AT5G08350.1		GRAM domain-containing protein / ABA-responsive protein-related	NA
Sapur.016G225800.1	380.913466	-0.908295369	0.045199502	-20.0953	8.12E-90	8.09E-89	cyan	-0.856735993	2.00E-46	GO:0070569,GO:0008152,GO:0006364	AT5G46420.1		16S rRNA processing protein RimM family	16S rRNA processing protein RimM, putative
Sapur.002G143700.1	108.9801587	-0.808436591	0.040394083	-20.0137	4.18E-89	4.13E-88	cyan	-0.858626957	7.71E-47	GO:0008270,GO:0003676,GO:0006355,GO:0003713,GO:0003677	AT4G00980.1		zinc knuckle (CCHC-type) family protein	Copia-like polyprotein/retrotransposon
Sapur.010G137000.1	12.77206634	5.797151115	0.289669714	20.01297	4.25E-89	4.19E-88	cyan	0.654536148	1.45E-20		AT2G21820.1			seed maturation protein PM41
Sapur.016G200300.1	216.8404053	0.818498619	0.040935141	19.99501	6.09E-89	5.99E-88	cyan	0.830025006	3.81E-41		AT3G26950.1			transmembrane protein, putative
Sapur.T046900.1	1159.176971	0.457222714	0.022924709	19.94454	1.67E-88	1.64E-87	cyan	0.845440847	4.52E-44	GO:0008152,GO:0003824	AT5G65940.1	CHY1	beta-hydroxyisobutyryl-CoA hydrolase 1	3-hydroxyisobutyryl-CoA hydrolase
Sapur.001G180800.1	353.1473494	0.659976101	0.033140063	19.91475	3.03E-88	2.96E-87	cyan	0.844262057	7.77E-44		AT5G49950.1		alpha/beta-Hydrolases superfamily protein	alpha/beta-hydrolase domain protein
Sapur.005G200100.1	790.2876701	0.545202261	0.027391927	19.90376	3.78E-88	3.68E-87	cyan	0.845992569	3.51E-44		AT1G20540.1		Transducin/WD40 repeat-like superfamily protein	transducin family protein
Sapur.008G114300.1	2612.901919	0.595476093	0.029973464	19.86678	7.89E-88	7.65E-87	cyan	0.844105889	8.34E-44	GO:0005515	AT2G30050.1		transducin family protein / WD-40 repeat family protein	transporter SEC13, putative
Sapur.006G093600.1	1232.660211	0.741037889	0.037308866	19.86225	8.64E-88	8.36E-87	cyan	0.840638872	3.98E-43	GO:0016757,GO:0047262	AT2G38650.1	GAUT7,LGT7	galacturonosyltransferase 7	glycosyltransferase family 8 protein
Sapur.15WG098500.1	290.639144	0.671947157	0.034013025	19.75558	7.18E-87	6.89E-86	cyan	0.833697579	8.15E-42		AT2G04940.1		scramblase-related	scramblase
Sapur.011G026300.1	63.12828292	2.897944556	0.146828291	19.73696	1.04E-86	9.93E-86	cyan	0.628012068	1.33E-18	GO:0005634	AT4G03270.1	CYCD6;1	Cyclin D6;1	D6-type cyclin
Sapur.010G135900.1	7.271329154	5.401282957	0.273817635	19.72584	1.29E-86	1.24E-85	cyan	0.721037409	1.77E-26	GO:0055114,GO:0020037,GO:0006979,GO:0004601	AT1G30870.1		Peroxidase superfamily protein	peroxidase
Sapur.005G198200.1	1734.024554	1.03045221	0.052314627	19.69721	2.28E-86	2.17E-85	cyan	0.813553536	2.50E-38	GO:0008236,GO:0006508,GO:0070008,GO:0004252	AT1G76140.1		Prolyl oligopeptidase family protein	NA
Sapur.006G002400.1	2252.003909	0.722747182	0.036829255	19.62427	9.59E-86	9.07E-85	cyan	0.83199257	1.68E-41	GO:0008299	AT5G47770.1	FPS1	farnesyl diphosphate synthase 1	farnesyl diphosphate synthase
Sapur.15WG001700.1	301.2350534	0.874688325	0.044638281	19.59503	1.70E-85	1.61E-84	cyan	0.820946383	1.48E-39		AT5G24460.1			hypothetical protein
Sapur.016G070500.1	204.9024641	1.142741021	0.058513493	19.52953	6.16E-85	5.75E-84	cyan	0.794076526	2.45E-35		AT3G15040.1		Protein of unknown function, DUF584	senescence regulator
Sapur.005G012600.1	25.75850518	3.133724167	0.160724934	19.49744	1.15E-84	1.07E-83	cyan	0.637201564	2.92E-19	GO:0004674,GO:0048544,GO:0006468,GO:0004672	AT4G21390.1	B120	S-locus lectin protein kinase family protein	NA
Sapur.010G049400.1	23.52092887	5.218042706	0.268338691	19.44573	3.17E-84	2.93E-83	cyan	0.466430643	7.42E-10	GO:0005515	AT2G29420.1	ATGSTU7,GST25,GSTU7	glutathione S-transferase tau 7	tau class glutathione transferase GSTU37
Sapur.002G103100.1	4.841002957	5.221858923	0.269215615	19.39657	8.25E-84	7.59E-83	cyan	0.74682113	2.96E-29		AT2G26570.1		Plant protein of unknown function (DUF827)	WEAK movement UNDER BLUE LIGHT protein
Sapur.008G069700.1	124.6841302	-1.419247277	0.073294115	-19.3637	1.56E-83	1.43E-82	cyan	-0.853418087	1.03E-45	GO:0008168,GO:0008152	AT1G69523.1		S-adenosyl-L-methionine-dependent methyltransferases superfamily protein	S-adenosylmethionine-dependent methyltransferase family, protein
Sapur.006G153200.1	836.5638276	0.777578842	0.040170882	19.35678	1.79E-83	1.63E-82	cyan	0.84056521	4.11E-43		AT2G24290.1		Protein of unknown function (DUF1068)	NA
Sapur.012G075300.1	4.755831752	5.192772265	0.268360697	19.34997	2.04E-83	1.86E-82	cyan	0.778393602	3.74E-33	GO:0005515	AT3G28410.1		F-box/RNI-like superfamily protein	F-box/LRR protein
Sapur.012G034700.1	663.715863	0.552571299	0.028676716	19.26899	9.78E-83	8.86E-82	cyan	0.856817091	1.92E-46	GO:0015031,GO:0008565	AT4G35410.2		Clathrin adaptor complex small chain family protein	AP-1 complex subunit sigma-1 protein
Sapur.005G047000.1	224.8183305	2.309137088	0.120091224	19.22819	2.15E-82	1.94E-81	cyan	0.802403643	1.42E-36	GO:0004857,GO:0042545,GO:0030599,GO:0005618	AT5G09760.1		Plant invertase/pectin methylesterase inhibitor superfamily	NA
Sapur.010G050600.1	17.835859	3.372535433	0.17548033	19.21888	2.57E-82	2.31E-81	cyan	0.702112094	1.25E-24		AT1G60420.1		DC1 domain-containing protein	protein disulfide isomerase (PDI)-like protein
Sapur.002G154300.1	315.410184	0.748396141	0.038998103	19.19058	4.44E-82	3.98E-81	cyan	0.829058578	5.68E-41	GO:0055085,GO:0016021,GO:0008324,GO:0006812	AT2G47830.1		Cation efflux family protein	cation diffusion facilitator family transporter
Sapur.15WG029600.1	268.3897325	0.813206359	0.042403538	19.17779	5.67E-82	5.08E-81	cyan	0.834973405	4.73E-42	GO:0006397,GO:0005634,GO:0004721	AT1G73820.1		Ssu72-like family protein	RNA polymerase II subunit A carboxy-terminal domain phosphatase Ssu72
Sapur.003G139400.1	652.8858855	0.539533242	0.028168048	19.15409	8.95E-82	8.00E-81	cyan	0.828422264	7.38E-41	GO:0003824,GO:0006470,GO:0004722	AT4G31860.1		Protein phosphatase 2C family protein	protein phosphatase 2C
Sapur.008G162300.1	910.2870208	0.823955632	0.043063423	19.13354	1.33E-81	1.18E-80	cyan	0.810746946	7.09E-38	GO:0016757,GO:0016021	AT4G02500.1	ATXT2,XT2,XXT2	UDP-xylosyltransferase 2	xyloglucan 6-xylosyltransferase
Sapur.005G185700.2	6.710157866	5.382655907	0.281840479	19.09824	2.61E-81	2.32E-80	cyan	0.671132171	6.74E-22	GO:0030246,GO:0005975,GO:0004553	AT4G36360.1	BGAL3	beta-galactosidase 3	beta-galactosidase
Sapur.006G212000.1	331.0567944	0.772720924	0.040577191	19.04323	7.48E-81	6.59E-80	cyan	0.819772116	2.34E-39	GO:0016192,GO:0016021	AT5G11150.1	ATVAMP713,VAMP713	vesicle-associated membrane protein 713	vesicle-associated membrane protein
Sapur.014G135800.1	7.584360245	4.165349695	0.220060822	18.92817	6.68E-80	5.83E-79	cyan	0.733162529	9.61E-28		AT5G35770.1	SAP	Transducin/WD40 repeat-like superfamily protein	transcriptional regulator sterile apetala protein
Sapur.014G144900.1	8.531870024	3.399027386	0.179595896	18.92597	6.97E-80	6.08E-79	cyan	0.807799141	2.08E-37		AT3G26380.1		Melibiase family protein	NA
Sapur.004G045200.1	484.5615744	0.899093857	0.047573436	18.89907	1.16E-79	1.01E-78	cyan	0.829495104	4.75E-41		AT5G46230.1		Protein of unknown function, DUF538	DUF538 family protein
Sapur.010G141400.1	1265.617012	0.602851935	0.031928528	18.88129	1.63E-79	1.41E-78	cyan	0.817329976	5.99E-39	GO:0006886	AT3G56190.1	ALPHA-SNAP2,ASNAP	alpha-soluble NSF attachment protein 2	alpha-soluble NSF attachment protein
Sapur.002G182800.1	725.8517933	0.777870164	0.041218411	18.87191	1.94E-79	1.68E-78	cyan	0.837757576	1.42E-42		AT3G26780.1		Phosphoglycerate mutase family protein	fructose-2%2C6-bisphosphatase, putative
Sapur.006G207000.1	153.976639	1.101687059	0.058432915	18.85388	2.73E-79	2.35E-78	cyan	0.803920245	8.31E-37	GO:0006122,GO:0005750	AT5G25450.1		Cytochrome bd ubiquinol oxidase, 14kDa subunit	cytochrome B-c1 complex subunit 7
Sapur.007G104600.1	3.593259617	5.051045652	0.268829601	18.78902	9.29E-79	7.94E-78	cyan	0.754015563	4.31E-30		AT4G38040.1		Exostosin family protein	NA
Sapur.011G122000.1	535.7393537	0.788026146	0.041942668	18.78817	9.44E-79	8.06E-78	cyan	0.79314975	3.34E-35	GO:0016301,GO:0005524	AT1G55810.1	UKL3	uridine kinase-like 3	uridine kinase
Sapur.009G013100.1	4.910975483	4.915068349	0.261768897	18.77636	1.18E-78	1.01E-77	cyan	0.755885931	2.59E-30	GO:0016747	AT5G39050.1		HXXXD-type acyl-transferase family protein	NA
Sapur.010G189200.1	12.87881051	3.604609519	0.192849994	18.69126	5.83E-78	4.93E-77	cyan	0.682454872	7.40E-23		AT3G44590.2		60S acidic ribosomal protein family	60S acidic ribosomal protein P2
Sapur.004G010200.1	424.5186993	1.117384773	0.059822663	18.67829	7.44E-78	6.27E-77	cyan	0.796407074	1.12E-35		AT5G52890.2		AT hook motif-containing protein	AT hook motif protein
Sapur.014G047800.1	253.3902153	1.362237105	0.073375534	18.56528	6.14E-77	5.12E-76	cyan	0.755282899	3.05E-30		AT3G60580.1		C2H2-like zinc finger protein	C2H2 zinc-finger protein
Sapur.010G090300.1	19.04128881	2.127812887	0.115032301	18.49753	2.16E-76	1.79E-75	cyan	0.720864561	1.85E-26	GO:0046872	AT1G68200.2		Zinc finger C-x8-C-x5-C-x3-H type family protein	zinc finger CCCH domain protein
Sapur.005G036100.1	127.1122038	0.767184356	0.041560816	18.45932	4.39E-76	3.61E-75	cyan	0.803341577	1.02E-36		AT3G22470.1		Pentatricopeptide repeat (PPR) superfamily protein	PPR domain protein
Sapur.006G021200.1	914.5594306	0.755770873	0.040971257	18.44637	5.58E-76	4.59E-75	cyan	0.800624683	2.64E-36		AT1G71270.1	ATVPS52,POK,TTD8	Vps52 / Sac2 family	vacuolar protein sorting-associated protein, putative
Sapur.016G047300.1	1766.98577	0.648656056	0.035267723	18.39234	1.51E-75	1.24E-74	cyan	0.815405921	1.25E-38	GO:0016746,GO:0008152	AT3G57650.1	LPAT2	lysophosphatidyl acyltransferase 2	1-acyl-sn-glycerol-3-phosphate acyltransferase
Sapur.007G085100.1	557.3758677	0.457680038	0.024942028	18.34975	3.32E-75	2.69E-74	cyan	0.829333656	5.07E-41	GO:0036459,GO:0016579	AT4G39910.1	ATUBP3,UBP3	ubiquitin-specific protease 3	ubiquitin carboxyl-terminal hydrolase
Sapur.003G142000.1	577.7164488	0.699098078	0.038098567	18.34972	3.32E-75	2.69E-74	cyan	0.79077726	7.32E-35	GO:0016853,GO:0005975	AT5G57330.1		Galactose mutarotase-like superfamily protein	aldose 1-epimerase, putative
Sapur.019G017100.1	91.406903	1.188488927	0.064780299	18.34646	3.52E-75	2.85E-74	cyan	0.786940764	2.55E-34		AT1G15190.1		Fasciclin-like arabinogalactan family protein	hypothetical protein
Sapur.005G135900.1	2614.848506	1.33914651	0.073040546	18.33429	4.41E-75	3.56E-74	cyan	0.785965102	3.48E-34		AT1G77690.1	LAX3	like AUX1 3	auxin influx transporter
Sapur.010G011900.1	1302.881457	0.550082851	0.030026796	18.31973	5.76E-75	4.65E-74	cyan	0.821952672	9.95E-40		AT4G14240.1		CBS domain-containing protein with a domain of unknown function (DUF21)	transmembrane protein, putative
Sapur.003G032900.1	1021.285218	0.683353721	0.03738823	18.27724	1.26E-74	1.01E-73	cyan	0.79342491	3.05E-35	GO:0005740,GO:0004129	AT1G80230.1		Rubredoxin-like superfamily protein	cytochrome C oxidase, putative
Sapur.15ZG055800.1	191.4681914	0.65752112	0.036379517	18.07394	5.11E-73	4.02E-72	cyan	0.797106237	8.83E-36	GO:0003677	AT3G19184.1		AP2/B3-like transcriptional factor family protein	B3 DNA-binding domain protein
Sapur.007G021700.1	5.930254906	3.253464649	0.180185768	18.05617	7.06E-73	5.54E-72	cyan	0.812296351	4.00E-38	GO:0055085,GO:0016021	AT1G75220.1		Major facilitator superfamily protein	sugar transporter ERD6-like protein
Sapur.016G160700.1	179.550704	1.060117049	0.058727749	18.05138	7.69E-73	6.03E-72	cyan	0.813002501	3.07E-38	GO:0005515	AT5G12300.1		Calcium-dependent lipid-binding (CaLB domain) family protein	C2 domain protein
Sapur.013G040300.1	9.141175421	5.214656634	0.289443517	18.01615	1.46E-72	1.14E-71	cyan	0.610141118	2.20E-17					hypothetical protein
Sapur.008G004000.1	1763.156964	0.64795349	0.035967817	18.01481	1.49E-72	1.17E-71	cyan	0.813587527	2.47E-38	GO:0016020,GO:0005509,GO:0004571	AT1G51590.1	MANIB,MNS1	alpha-mannosidase 1	mannosyl-oligosaccharide 1%2C2-alpha-mannosidase, putative
Sapur.014G138800.1	310.4908019	0.787832604	0.043790605	17.9909	2.30E-72	1.79E-71	cyan	0.808020385	1.92E-37	GO:0016192,GO:0016021,GO:0006810	AT4G26550.1		Got1/Sft2-like vescicle transport protein family	protein transporter SFT2
Sapur.008G018500.1	72.40689095	0.853203142	0.047516694	17.95586	4.32E-72	3.36E-71	cyan	0.828046155	8.61E-41	GO:0016746,GO:0008152	AT3G52200.1	LTA3	Dihydrolipoamide acetyltransferase, long form protein	pyruvate dehydrogenase complex dihydrolipoamide acetyltransferase
Sapur.018G101800.1	295.3336311	1.218706057	0.06801197	17.91899	8.38E-72	6.49E-71	cyan	0.827043835	1.30E-40		AT5G20190.1		Tetratricopeptide repeat (TPR)-like superfamily protein	TPR domain protein, putative
Sapur.005G018900.1	261.6412698	0.772726051	0.043137701	17.91301	9.33E-72	7.22E-71	cyan	0.779820129	2.41E-33	GO:0016746,GO:0008152,GO:0006644	AT3G05510.1		Phospholipid/glycerol acyltransferase family protein	phospholipid/glycerol acyltransferase family protein
Sapur.15WG126700.1	5538.650467	0.669935505	0.037452683	17.88752	1.48E-71	1.14E-70	cyan	0.800104734	3.16E-36	GO:0051082,GO:0006457,GO:0005783,GO:0005509	AT5G61790.1	ATCNX1,CNX1	calnexin 1	calnexin
Sapur.010G149800.1	278.1624471	0.551109394	0.030904875	17.83244	3.96E-71	3.03E-70	cyan	0.83244549	1.39E-41	GO:0005515	AT5G05760.1	ATSED5.ATSYP31.SED5.SYP3	syntaxin of plants 31	syntaxin-31 protein
Sapur.016G049300.1	1061.905264	0.896847414	0.050408914	17.79144	8.23E-71	6.28E-70	cyan	0.795806728	1.37E-35		AT1G01170.1		Protein of unknown function (DUF1138)	ozone-responsive stress-like protein
Sapur.010G126300.1	162.2381475	0.764476621	0.043129064	17.72532	2.67E-70	2.02E-69	cyan	0.820037928	2.11E-39		AT1G26650.1			ATP synthase subunit beta
Sapur.008G158700.1	3.865652657	4.929143187	0.278328142	17.70983	3.52E-70	2.65E-69	cyan	0.708602279	3.02E-25					NA
Sapur.008G103500.1	791.5200254	0.685774339	0.038770678	17.68796	5.19E-70	3.91E-69	cyan	0.813342116	2.71E-38	GO:0016567,GO:0004842,GO:0005515	AT1G23030.1		ARM repeat superfamily protein	E3 ubiquitin ligase PUB14, putative
Sapur.016G307200.1	10.39753033	2.291365338	0.129622846	17.67717	6.29E-70	4.72E-69	cyan	0.735523174	5.35E-28					NA
Sapur.018G022000.1	808.8294802	0.915785643	0.052079479	17.58439	3.24E-69	2.41E-68	cyan	0.724539884	7.77E-27	GO:0016192,GO:0016021	AT5G11150.1	ATVAMP713,VAMP713	vesicle-associated membrane protein 713	vesicle-associated membrane protein
Sapur.008G134300.1	69.54784167	1.011059155	0.057622188	17.54635	6.34E-69	4.69E-68	cyan	0.782581043	1.02E-33	GO:0046983	AT4G14410.1	bHLH104	basic helix-loop-helix (bHLH) DNA-binding superfamily protein	basic helix loop helix transcription factor
Sapur.010G078700.1	1838.373934	0.782274864	0.044605469	17.53764	7.39E-69	5.46E-68	cyan	0.78390876	6.70E-34	GO:0008168	AT1G26850.1		S-adenosyl-L-methionine-dependent methyltransferases superfamily protein	methyltransferase PMT2, putative
Sapur.009G048100.1	12552.50132	0.583529742	0.033275672	17.53623	7.58E-69	5.59E-68	cyan	0.810255489	8.49E-38		AT5G13490.1	AAC2	ADP/ATP carrier 2	ADP%2CATP carrier protein
Sapur.017G062700.1	873.4248984	0.452057256	0.025867054	17.47618	2.18E-68	1.59E-67	cyan	0.824396759	3.77E-40	GO:0016020,GO:0008375	AT5G15050.1		Core-2/I-branching beta-1,6-N-acetylglucosaminyltransferase family protein	BGGP beta-1-3-galactosyl-O-glycosyl-glycoprotein
Sapur.018G066200.1	545.3240599	0.993708645	0.056973734	17.44152	3.99E-68	2.91E-67	cyan	0.751415854	8.71E-30	GO:0016853,GO:0005975	AT5G57330.1		Galactose mutarotase-like superfamily protein	aldose 1-epimerase, putative
Sapur.009G008300.1	148.4912511	1.247833256	0.071872109	17.36186	1.60E-67	1.16E-66	cyan	0.741425846	1.20E-28		AT3G45230.1		hydroxyproline-rich glycoprotein family protein	transmembrane protein, putative
Sapur.T005500.1	120.432633	1.728123511	0.099572805	17.35538	1.80E-67	1.30E-66	cyan	0.809767523	1.02E-37	GO:0055085,GO:0042626,GO:0016021,GO:0006810,GO:0005524,GO:0016887	AT3G13080.1	ATMRP3,MRP3	multidrug resistance-associated protein 3	multidrug resistance protein ABC transporter family protein
Sapur.014G074100.1	5.927011664	3.510671352	0.202499932	17.33665	2.49E-67	1.79E-66	cyan	0.790838045	7.17E-35	GO:0080123,GO:0009864,GO:0009694,GO:0009611	AT2G46370.4	FIN219,JAR1	Auxin-responsive GH3 family protein	indole-3-acetic acid-amido synthetase GH3.5 protein, putative
Sapur.009G042700.1	166.3232788	1.779992849	0.102795805	17.31581	3.57E-67	2.57E-66	cyan	0.744334177	5.66E-29	GO:0006508,GO:0004185	AT3G63470.1	scpl40	serine carboxypeptidase-like 40	serine carboxypeptidase
Sapur.018G089000.1	71.75442656	2.632854143	0.152074432	17.31293	3.76E-67	2.70E-66	cyan	0.821261774	1.31E-39		AT5G25240.1			stress induced protein, putative
Sapur.010G031100.1	2917.453352	0.515372785	0.029789857	17.30028	4.68E-67	3.37E-66	cyan	0.815440272	1.23E-38	GO:0016624,GO:0008152	AT1G59900.1	AT-E1 ALPHA,E1 ALPHA	pyruvate dehydrogenase complex E1 alpha subunit	pyruvate dehydrogenase E1 component alpha subunit
Sapur.010G165600.1	3695.665691	1.505545716	0.087102743	17.28471	6.13E-67	4.40E-66	cyan	0.838922538	8.51E-43	GO:0016020,GO:0006810,GO:0005215	AT4G01470.1	ATTIP1.3.GAMMA-TIP3.TIP	tonoplast intrinsic protein 1;3	tonoplast intrinsic protein
Sapur.006G084300.1	4.093746966	4.915270688	0.284665432	17.26683	8.36E-67	5.98E-66	cyan	0.673936363	3.93E-22	GO:0006468,GO:0005524,GO:0004672,GO:0030246	AT3G08870.1		Concanavalin A-like lectin protein kinase family protein	NA
Sapur.005G073900.1	357.0665493	1.072851908	0.062211599	17.24521	1.22E-66	8.68E-66	cyan	0.77329693	1.76E-32	GO:0006807,GO:0006542,GO:0004356	AT5G37600.1	ATGLN1:1.ATGSR1.GLN1:1.	glutamine synthase clone R1	glutamine synthetase
Sapur.016G021900.1	6.538755441	4.548946643	0.26464254	17.18902	3.21E-66	2.28E-65	cyan	0.700666303	1.71E-24		AT2G41050.1		PQ-loop repeat family protein / transmembrane family protein	PQ loop protein
Sapur.016G214000.1	328.6906201	-0.574837948	0.033488926	-17.165	4.85E-66	3.44E-65	cyan	-0.820574151	1.71E-39		AT5G14390.1		alpha/beta-Hydrolases superfamily protein	alpha/beta hydrolase family protein
Sapur.003G008200.1	275.9686677	0.495087576	0.028862583	17.15327	5.94E-66	4.20E-65	cyan	0.82773065	9.80E-41		AT1G58110.2		Basic-leucine zipper (bZIP) transcription factor family protein	BZIP transcription factor
Sapur.15WG139400.1	575.4626743	0.667163511	0.038904957	17.14855	6.44E-66	4.56E-65	cyan	0.794228915	2.33E-35	GO:0016020	AT5G51570.1		SPFH/Band 7/PHB domain-containing membrane-associated protein family	hypersensitive-induced reaction protein
Sapur.003G093200.1	774.4312463	1.582999546	0.092677382	17.08075	2.06E-65	1.45E-64	cyan	0.683382152	6.15E-23	GO:0055114,GO:0020037,GO:0016705,GO:0005506	AT2G45970.1	CYP86A8,LCR	cytochrome P450, family 86, subfamily A, polypeptide 8	cytochrome P450 family 86 protein
Sapur.014G124800.1	1505.154874	1.240362171	0.072751719	17.04925	3.54E-65	2.48E-64	cyan	0.758371609	1.30E-30	GO:0008152	AT2G05710.1	ACO3	aconitase 3	aconitate hydratase
Sapur.005G042100.1	19.11559993	4.527585386	0.267046853	16.95427	1.79E-64	1.24E-63	cyan	0.578496662	2.11E-15		AT2G30580.1	DRIP2	DREB2A-interacting protein 2	zinc finger, C3HC4 type (ring finger) protein
Sapur.019G046500.1	12.59830751	4.089258223	0.24204381	16.8947	4.92E-64	3.38E-63	cyan	0.643895447	9.35E-20					NA
Sapur.016G146600.1	74.19821462	-1.257774175	0.074488779	-16.8854	5.76E-64	3.95E-63	cyan	-0.815227628	1.33E-38		AT2G01870.1			transmembrane protein, putative
Sapur.002G079600.1	730.6336213	1.060146313	0.062858154	16.86569	8.05E-64	5.51E-63	cyan	0.78168658	1.35E-33		AT1G78280.1		transferases, transferring glycosyl groups	F-box plant protein
Sapur.008G143500.1	2.294365458	4.665200277	0.276648582	16.86327	8.38E-64	5.74E-63	cyan	0.694469182	6.36E-24		AT1G67540.2			NA
Sapur.012G093200.1	659.3362674	1.016423454	0.060320067	16.8505	1.04E-63	7.11E-63	cyan	0.779542349	2.62E-33		AT2G35120.1		Single hybrid motif superfamily protein	glycine cleavage system H protein
Sapur.018G037100.1	55.42323669	1.552959265	0.092182744	16.84653	1.11E-63	7.59E-63	cyan	0.748935826	1.69E-29		AT2G26310.1		Chalcone-flavanone isomerase family protein	chalcone-flavanone isomerase
Sapur.009G091400.1	1221.003011	0.814282475	0.04840298	16.82298	1.66E-63	1.13E-62	cyan	0.767961753	8.49E-32	GO:0007010,GO:0003779	AT4G34490.1	ATCAP1,CAP 1,CAP1	cyclase associated protein 1	adenylyl cyclase-associated protein
Sapur.016G234000.1	2133.509964	0.523891256	0.031237792	16.77107	3.97E-63	2.69E-62	cyan	0.805912126	4.09E-37	GO:0016021,GO:0006810	AT3G07680.1		emp24/gp25L/p24 family/GOLD family protein	transmembrane emp24 domain protein
Sapur.016G109300.1	76.65834972	1.555906225	0.092838634	16.75925	4.85E-63	3.28E-62	cyan	0.75527539	3.06E-30		AT5G01650.1		Tautomerase/MIF superfamily protein	macrophage migration inhibitory factor family protein
Sapur.013G005900.1	860.278214	0.533468657	0.032011016	16.66516	2.35E-62	1.57E-61	cyan	0.781190407	1.57E-33	GO:0016021	AT1G09330.1			FAM18-like protein, putative
Sapur.016G095900.1	3181.687636	0.587107645	0.035243571	16.65857	2.62E-62	1.75E-61	cyan	0.798769366	5.00E-36		AT2G38040.2	CAC3	acetyl Co-enzyme a carboxylase carboxyltransferase alpha subunit	acetyl-CoA carboxylase, carboxyl transferase%2C alpha subunit
Sapur.002G142000.1	87.78269237	2.127508198	0.127763582	16.65191	2.93E-62	1.96E-61	cyan	0.770600865	3.91E-32		AT2G46600.1		Calcium-binding EF-hand family protein	calcium-binding protein with EF-hand protein, putative
Sapur.006G168600.1	30.86928382	1.303274681	0.078419645	16.61924	5.06E-62	3.37E-61	cyan	0.774025478	1.41E-32	GO:0005634	AT5G22250.1		Polynucleotidyl transferase, ribonuclease H-like superfamily protein	CCR4-associated factor-like protein, putative
Sapur.001G027100.1	18.94780966	1.958971742	0.118463102	16.53656	2.00E-61	1.32E-60	cyan	0.67574647	2.77E-22		AT1G15780.1			NA
Sapur.014G107100.1	162.1984825	0.872194292	0.053057369	16.4387	1.01E-60	6.60E-60	cyan	0.7775336	4.87E-33		AT3G62920.1			hypothetical protein
Sapur.006G122000.1	1259.480864	0.422947196	0.025751336	16.42428	1.28E-60	8.36E-60	cyan	0.806730431	3.05E-37	GO:0004379,GO:0006499	AT5G57020.1	ATNMT1,NMT1	myristoyl-CoA:protein N-myristoyltransferase	glycylpeptide N-tetradecanoyltransferase
Sapur.010G115700.1	186.8677456	0.722335034	0.044002379	16.41582	1.47E-60	9.60E-60	cyan	0.767592716	9.45E-32	GO:0005515	AT5G49610.1		F-box family protein	F-box plant protein
Sapur.004G075000.1	840.2533271	-1.121261399	0.068606795	-16.3433	4.86E-60	3.14E-59	cyan	-0.864179245	4.33E-48		AT3G30300.1		O-fucosyltransferase family protein	auxin-independent growth promoter protein
Sapur.007G115500.1	1119.225239	0.770428059	0.047199975	16.32264	6.81E-60	4.39E-59	cyan	0.795965188	1.30E-35		AT3G59480.1		pfkB-like carbohydrate kinase family protein	pfkB-type carbohydrate kinase family protein
Sapur.010G156100.1	7.891471793	4.000500281	0.245926436	16.26706	1.69E-59	1.08E-58	cyan	0.709489565	2.48E-25					NA
Sapur.013G112200.1	590.5739641	0.633867255	0.039008581	16.24943	2.25E-59	1.44E-58	cyan	0.80318938	1.08E-36	GO:0005525	AT5G60860.1	AtRABA1f,RABA1f	RAB GTPase homolog A1F	Rab-related GTPase family protein
Sapur.018G117700.1	1180.480804	0.867899026	0.053506488	16.22044	3.62E-59	2.30E-58	cyan	0.763009823	3.53E-31	GO:0003824	AT2G07050.1	CAS1	cycloartenol synthase 1	cycloartenol synthase
Sapur.014G140900.1	3.077694443	4.235301813	0.261228033	16.21304	4.08E-59	2.60E-58	cyan	0.758140499	1.39E-30		AT3G62140.1			NA
Sapur.013G100400.1	525.9124713	0.611806969	0.037742957	16.20983	4.30E-59	2.73E-58	cyan	0.800238102	3.01E-36		AT1G22520.2		Domain of unknown function (DUF543)	transmembrane protein, putative
Sapur.010G139300.1	1459.128592	0.680136404	0.042140164	16.13986	1.34E-58	8.45E-58	cyan	0.748079296	2.12E-29	GO:0055114,GO:0045300,GO:0006631	AT3G02630.1		Plant stearoyl-acyl-carrier-protein desaturase family protein	acyl-[acyl-carrier] desaturase
Sapur.005G164300.1	47.55156208	1.521932062	0.094632549	16.08254	3.38E-58	2.12E-57	cyan	0.70262182	1.12E-24		AT4G23140.2	CRK6	cysteine-rich RLK (RECEPTOR-like protein kinase) 6	salt stress response/antifungal domain protein
Sapur.005G116100.1	593.1512117	0.503206893	0.031322362	16.06542	4.46E-58	2.79E-57	cyan	0.778929806	3.17E-33	GO:0019211	AT4G08960.1		phosphotyrosyl phosphatase activator (PTPA) family protein	phosphotyrosyl phosphatase activator protein, putative
Sapur.018G096200.1	10088.34203	1.057786691	0.066136856	15.99391	1.41E-57	8.75E-57	cyan	0.745969946	3.70E-29	GO:0016020,GO:0015992,GO:0009678,GO:0004427	AT1G15690.1	ATAVP3.AtVHP1:1.AVP-3.AV	Inorganic H pyrophosphatase family protein	pyrophosphate-energized vacuolar membrane proton pump protein
Sapur.006G069000.1	41.19236241	1.51570062	0.094791024	15.98992	1.50E-57	9.32E-57	cyan	0.787067076	2.44E-34		AT5G02420.1			hypothetical protein
Sapur.018G047000.1	231.8451284	0.528879199	0.033151512	15.9534	2.70E-57	1.67E-56	cyan	0.79530272	1.62E-35	GO:0003676,GO:0006260,GO:0005634,GO:0003677	AT2G06510.1	ATRPA1A,ATRPA70A,RPA1A	replication protein A 1A	replication protein A 70 kDa DNA-binding subunit A
Sapur.010G041100.1	6913.851421	0.498495618	0.031304755	15.92396	4.32E-57	2.66E-56	cyan	0.780817757	1.77E-33	GO:0055114,GO:0016491,GO:0016616,GO:0016615,GO:0006108	AT5G43330.1		Lactate/malate dehydrogenase family protein	malate dehydrogenase
Sapur.008G128200.1	1528.401424	0.673244706	0.042283832	15.92204	4.46E-57	2.75E-56	cyan	0.755791347	2.65E-30		AT5G43830.1		Aluminium induced protein with YGL and LRDR motifs	aluminum-induced-like protein
Sapur.009G114200.1	31.97843777	1.619763454	0.101786143	15.9134	5.12E-57	3.15E-56	cyan	0.731982647	1.29E-27		AT1G08290.1	WIP3	WIP domain protein 3	NA
Sapur.007G082100.1	55.02823306	2.377637979	0.149554031	15.89819	6.52E-57	4.01E-56	cyan	0.644525581	8.39E-20	GO:0008168	AT1G15125.1		S-adenosyl-L-methionine-dependent methyltransferases superfamily protein	NA
Sapur.15ZG040400.1	6.109793736	2.982301396	0.187924969	15.86964	1.03E-56	6.29E-56	cyan	0.74381769	6.48E-29		AT5G24060.2		Pentatricopeptide repeat (PPR) superfamily protein	NA
Sapur.016G117900.1	9.03200682	1.940337889	0.12295802	15.78049	4.24E-56	2.57E-55	cyan	0.75508691	3.22E-30	GO:0043565,GO:0006355,GO:0003700	AT3G56850.1	AREB3,DPBF3	ABA-responsive element binding protein 3	BZIP transcription factor
Sapur.008G115200.1	497.4001923	0.502314366	0.031837665	15.77736	4.45E-56	2.70E-55	cyan	0.80178859	1.76E-36	GO:0005515	AT1G14570.1		UBX domain-containing protein	UBX domain protein
Sapur.019G016900.1	2.126657192	4.589854553	0.291921675	15.7229	1.05E-55	6.34E-55	cyan	0.655207325	1.28E-20		AT1G15190.1		Fasciclin-like arabinogalactan family protein	hypothetical protein
Sapur.016G059800.1	3.69192268	4.842573209	0.309439089	15.64949	3.35E-55	2.00E-54	cyan	0.596097879	1.78E-16	GO:0006950	AT3G11930.2		Adenine nucleotide alpha hydrolases-like superfamily protein	universal stress A-like protein
Sapur.017G111700.1	16.25294306	2.134151795	0.136670474	15.61531	5.73E-55	3.41E-54	cyan	0.709526207	2.46E-25		AT1G66170.1	MMD1	RING/FYVE/PHD zinc finger superfamily protein	PHD finger protein
Sapur.002G127300.1	330.6346029	0.820461629	0.052730576	15.5595	1.37E-54	8.12E-54	cyan	0.749958871	1.29E-29	GO:0016787	AT3G09970.1		Calcineurin-like metallo-phosphoesterase superfamily protein	calcineurin-like phosphoesterase
Sapur.006G050600.1	59.40492321	1.065120247	0.068464345	15.5573	1.42E-54	8.40E-54	cyan	0.750010548	1.27E-29	GO:0034314,GO:0030833,GO:0005885,GO:0005856	AT1G60430.1	ARPC3	actin-related protein C3	actin-related protein 2/3 complex subunit 3
Sapur.016G294400.1	353.2323907	-1.422584982	0.091466189	-15.5531	1.52E-54	8.96E-54	cyan	-0.852329172	1.75E-45	GO:0055114,GO:0016491	AT1G14130.1		2-oxoglutarate (2OG) and Fe(II)-dependent oxygenase superfamily protein	2-oxoglutarate-dependent dioxygenase
Sapur.002G116800.1	23.52559033	1.536272876	0.098876666	15.53726	1.94E-54	1.15E-53	cyan	0.708908979	2.82E-25	GO:0006886,GO:0005784	AT3G60540.1		Preprotein translocase Sec, Sec61-beta subunit protein	protein transporter Sec61 beta subunit, putative
Sapur.001G136000.1	12.51638456	2.041106392	0.131644749	15.50465	3.23E-54	1.90E-53	cyan	0.738862779	2.31E-28		AT1G53760.1			K+-H+ exchange-like protein
Sapur.001G114000.1	118.4866106	0.945574716	0.061063018	15.48523	4.37E-54	2.57E-53	cyan	0.749152208	1.60E-29		AT1G32260.1			hypothetical protein
Sapur.010G144700.1	651.4796455	0.50839739	0.032872647	15.46567	5.92E-54	3.47E-53	cyan	0.773550551	1.63E-32	GO:0008168	AT2G40280.1		S-adenosyl-L-methionine-dependent methyltransferases superfamily protein	S-adenosyl-L-methionine-dependent methyltransferase, putative
Sapur.002G161300.1	8524.211607	0.41889993	0.027208256	15.39606	1.74E-53	1.01E-52	cyan	0.764345604	2.41E-31	GO:0005783,GO:0045454	AT2G47470.1	ATPDI11.ATPDIL2-1.MEE30.	thioredoxin family protein	protein disulfide isomerase-like protein
Sapur.003G106100.1	7914.865513	0.643344826	0.041804907	15.38922	1.93E-53	1.12E-52	cyan	0.769016094	6.24E-32		AT5G42020.1	BIP,BIP2	Heat shock protein 70 (Hsp 70) family protein	mediator of RNA polymerase II transcription protein
Sapur.008G141100.1	144.2572485	1.191945992	0.077752703	15.32996	4.82E-53	2.79E-52	cyan	0.73342334	9.01E-28	GO:0055085,GO:0016021	AT1G04570.1		Major facilitator superfamily protein	folate/biopterin transporter
Sapur.008G035100.1	1037.089482	0.7347283	0.048062637	15.28689	9.35E-53	5.38E-52	cyan	0.745463693	4.22E-29	GO:0019205,GO:0006139,GO:0005524	AT2G37250.1	ADK,ATPADK1	adenosine kinase	adenylate kinase
Sapur.008G058900.1	2194.217674	0.485839236	0.031862532	15.24798	1.70E-52	9.72E-52	cyan	0.76524887	1.86E-31		AT3G56130.1		biotin/lipoyl attachment domain-containing protein	biotin carboxyl carrier of acetyl-CoA carboxylase
Sapur.016G155700.1	86.33031916	1.167836747	0.076604976	15.24492	1.78E-52	1.02E-51	cyan	0.720198364	2.16E-26		AT3G52115.1	ATCOM1.ATGR1.COM1.GR1	gamma response gene 1	DNA repair protein endonuclease SAE2/CtIP carboxy-terminal protein
Sapur.011G100000.1	892.4108919	0.689402773	0.045298187	15.21921	2.64E-52	1.50E-51	cyan	0.746118664	3.55E-29	GO:0045454	AT3G20560.1	ATPDI12.ATPDIL5-3.PDI12.F	PDI-like 5-3	protein disulfide isomerase
Sapur.019G014200.1	62.66411724	1.211955579	0.079639955	15.21793	2.69E-52	1.53E-51	cyan	0.779233605	2.89E-33	GO:0055114,GO:0016491	AT5G08640.1	ATFLS1,FLS,FLS1	flavonol synthase 1	NA
Sapur.003G169400.1	2214.394931	0.593677534	0.039027465	15.21179	2.95E-52	1.68E-51	cyan	0.751482834	8.55E-30	GO:0006491	AT5G56360.1	PSL4	calmodulin-binding protein	glucosidase II beta subunit, putative
Sapur.002G070700.1	11.2444035	4.671639007	0.307891908	15.17298	5.34E-52	3.03E-51	cyan	0.549876001	8.73E-14		AT1G37140.1	MCT1	MEI2 C-terminal RRM only like 1	RNA recognition motif 3 in plant Mei2-like protein
Sapur.004G043300.1	419.1509159	0.567264054	0.037434567	15.15348	7.19E-52	4.06E-51	cyan	0.775523303	8.98E-33		AT1G78700.1	BEH4	BES1/BZR1 homolog 4	brassinosteroid signaling positive regulator-like protein, putative
Sapur.002G011400.1	167.111794	0.896482354	0.059201759	15.14283	8.45E-52	4.77E-51	cyan	0.731075459	1.61E-27		AT1G76060.1	EMB1793	LYR family of Fe/S cluster biogenesis protein	complex 1 protein, LYR family protein
Sapur.006G063300.1	1135.323292	0.491827036	0.032551898	15.10901	1.41E-51	7.94E-51	cyan	0.741335184	1.23E-28	GO:0051603,GO:0005839,GO:0004298	AT4G31300.3	PBA1	N-terminal nucleophile aminohydrolases (Ntn hydrolases) superfamily protein	proteasome subunit beta type protein
Sapur.007G090100.1	2773.942219	0.879427344	0.058224457	15.10409	1.52E-51	8.55E-51	cyan	0.732652006	1.09E-27	GO:0005975,GO:0004553	AT5G63840.1	PSL5,RSW3	Glycosyl hydrolases family 31 protein	neutral alpha-glucosidase AB, putative
Sapur.15WG146900.1	341.2255246	0.617356565	0.04090475	15.09254	1.81E-51	1.02E-50	cyan	0.76131007	5.71E-31	GO:0007264,GO:0005525	AT5G52210.1	ATARLB1,ATGB1,GB1	GTP-binding protein 1	ADP-ribosylation factor(Arf)/Arf-like (Arl) small GTPase family protein
Sapur.006G179200.1	717.6676773	0.608143443	0.040332821	15.07813	2.26E-51	1.26E-50	cyan	0.743050341	7.90E-29		AT2G26260.1	3BETAHSD/D2,AT3BETAHSD	3beta-hydroxysteroid-dehydrogenase/decarboxylase isoform 2	3beta-hydroxysteroid-dehydrogenase/decarboxylase
Sapur.008G071500.1	168.3206774	0.727467727	0.048280818	15.06743	2.65E-51	1.48E-50	cyan	0.777306445	5.22E-33		AT1G14020.1		O-fucosyltransferase family protein	GDP-fucose O-fucosyltransferase-like protein
Sapur.010G025600.1	696.7597677	0.554614769	0.03692259	15.02101	5.35E-51	2.98E-50	cyan	0.774480299	1.23E-32		AT1G60780.1	HAP13	Clathrin adaptor complexes medium subunit family protein	clathrin adaptor medium chain protein, putative
Sapur.004G056500.1	197.9079588	0.628817931	0.041878079	15.01544	5.82E-51	3.24E-50	cyan	0.75495068	3.34E-30		AT3G03210.1			Pmr5/Cas1p GDSL/SGNH-like acyl-esterase family protein
Sapur.15WG110500.1	282.6578592	0.480019727	0.031998144	15.00149	7.18E-51	4.00E-50	cyan	0.748860547	1.72E-29		AT2G40190.1	LEW3	UDP-Glycosyltransferase superfamily protein	glycosyl transferase family 1 protein
Sapur.012G045900.1	1747.010804	0.483853133	0.032283986	14.9874	8.88E-51	4.93E-50	cyan	0.765890292	1.55E-31		AT3G25800.1	PDF1,PP2AA2,PR 65	protein phosphatase 2A subunit A2	serine/threonine protein phosphatase 2A regulatory subunit A, putative
Sapur.016G295700.1	1046.245663	0.553151051	0.037078406	14.91842	2.50E-50	1.38E-49	cyan	0.735677887	5.14E-28	GO:0055114,GO:0016491	AT2G17420.1	ATNTRA,NTR2,NTRA	NADPH-dependent thioredoxin reductase A	thioredoxin-disulfide reductase
Sapur.15WG097300.1	1408.078929	0.379415497	0.025445267	14.91104	2.79E-50	1.54E-49	cyan	0.749991321	1.28E-29	GO:0016627	AT5G09600.3	SDH3-1	succinate dehydrogenase 3-1	succinate dehydrogenase/fumarate reductase transmembrane subunit
Sapur.002G060900.1	123.4382826	1.233483157	0.083045816	14.85304	6.65E-50	3.64E-49	cyan	0.673558215	4.23E-22	GO:0008168	AT1G77260.1		S-adenosyl-L-methionine-dependent methyltransferases superfamily protein	S-adenosyl-L-methionine-dependent methyltransferase, putative
Sapur.15WG015500.1	2.693950318	4.30913267	0.291731936	14.77086	2.26E-49	1.22E-48	cyan	0.620365084	4.52E-18	GO:0005515	AT4G22430.1		F-box family protein	F-box protein interaction domain protein
Sapur.005G097400.1	20.04754029	1.349299921	0.091612904	14.72827	4.24E-49	2.29E-48	cyan	0.756076096	2.45E-30	GO:0005515	AT2G18280.1	AtTLP2,TLP2	tubby like protein 2	NA
Sapur.15WG137800.1	16.19612276	5.297837643	0.361117388	14.67068	9.93E-49	5.34E-48	cyan	0.596444407	1.69E-16	GO:0004857	AT3G47380.1		Plant invertase/pectin methylesterase inhibitor superfamily protein	plant invertase/pectin methylesterase inhibitor protein
Sapur.005G079400.1	3099.210316	0.91048465	0.062510698	14.56526	4.67E-48	2.48E-47	cyan	0.763823148	2.80E-31		AT2G17630.1		Pyridoxal phosphate (PLP)-dependent transferases superfamily protein	phosphoserine aminotransferase
Sapur.009G129600.1	504.6844304	0.528060985	0.036649436	14.40843	4.58E-47	2.39E-46	cyan	0.759960552	8.35E-31		AT4G38370.1		Phosphoglycerate mutase family protein	phosphoglycerate mutase family protein
Sapur.006G209100.1	2984.301737	0.588831307	0.040923217	14.38869	6.09E-47	3.17E-46	cyan	0.735662553	5.16E-28		AT4G32390.1		Nucleotide-sugar transporter family protein	sugar phosphate/phosphate translocator, putative
Sapur.006G095600.1	5481.830484	0.464374597	0.03227509	14.38802	6.15E-47	3.20E-46	cyan	0.760318422	7.55E-31	GO:0006096,GO:0004634,GO:0000287,GO:0000015	AT2G36530.1	ENO2,LOS2	Enolase	phosphopyruvate hydratase
Sapur.003G052300.1	138.9986844	0.552677414	0.038431887	14.3807	6.84E-47	3.56E-46	cyan	0.732192832	1.22E-27		AT2G44730.1		Alcohol dehydrogenase transcription factor Myb/SANT-like family protein	Myb/SANT-like DNA-binding domain protein
Sapur.005G097000.1	882.1491056	0.466349071	0.032559036	14.32318	1.57E-46	8.10E-46	cyan	0.767210039	1.06E-31		AT1G19580.1	GAMMA CA1	gamma carbonic anhydrase 1	gamma carbonic anhydrase gamma carbonic anhydrase-like protein
Sapur.004G162200.1	4.585202663	3.503147175	0.245259424	14.28344	2.78E-46	1.43E-45	cyan	0.659352099	6.06E-21					NA
Sapur.010G056100.1	5312.388298	0.726407881	0.051194643	14.18914	1.07E-45	5.44E-45	cyan	0.716227161	5.41E-26	GO:0055114,GO:0016620	AT3G04120.1	GAPC,GAPC-1,GAPC1	glyceraldehyde-3-phosphate dehydrogenase C subunit 1	cytosolic glyceraldehyde-3-phosphate dehydrogenase
Sapur.001G101100.1	63.06230669	1.192569324	0.084136764	14.17418	1.32E-45	6.73E-45	cyan	0.674298415	3.67E-22		AT2G35736.2			DUF4535 domain protein
Sapur.018G018200.1	244.0008113	-0.90531319	0.0638989	-14.1679	1.45E-45	7.36E-45	cyan	-0.78477957	5.08E-34		AT5G11000.1		Plant protein of unknown function (DUF868)	DUF868 family protein
Sapur.008G116200.1	172.1244904	-0.582196457	0.041117149	-14.1595	1.63E-45	8.29E-45	cyan	-0.752144106	7.16E-30		AT2G02170.2		Remorin family protein	remorin protein
Sapur.005G036200.1	36.25295541	3.528485241	0.250533436	14.08389	4.77E-45	2.40E-44	cyan	0.560830212	2.19E-14		AT1G05460.1	SDE3	P-loop containing nucleoside triphosphate hydrolases superfamily protein	RNA helicase
Sapur.002G028700.1	502.6792965	0.578106722	0.04106539	14.07771	5.21E-45	2.62E-44	cyan	0.747735555	2.32E-29	GO:0030246,GO:0005975	AT3G54690.1		Sugar isomerase (SIS) family protein	KpsF/GutQ family sugar isomerase
Sapur.001G045000.1	9.9705986	1.843165294	0.131152134	14.05364	7.32E-45	3.67E-44	cyan	0.70594636	5.43E-25		AT1G67370.1	ASY1,ATASY1	DNA-binding HORMA family protein	NA
Sapur.006G046900.1	626.5882493	0.327302084	0.02340279	13.9856	1.91E-44	9.51E-44	cyan	0.750845234	1.02E-29		AT5G06830.1			CDK5 regulatory subunit associated protein
Sapur.005G017600.1	39.5379973	1.055668914	0.075508147	13.98086	2.04E-44	1.02E-43	cyan	0.695203151	5.45E-24		AT1G02816.1		Protein of unknown function, DUF538	DUF538 family protein
Sapur.006G034200.1	142.7172747	1.338021292	0.096154097	13.91539	5.11E-44	2.53E-43	cyan	0.681997755	8.11E-23	GO:0005759	AT2G41600.5		Mitochondrial glycoprotein family protein	glycoprotein
Sapur.002G183700.1	668.0541929	0.577515154	0.041525742	13.9074	5.71E-44	2.82E-43	cyan	0.72041264	2.05E-26		AT3G19340.1		Protein of unknown function (DUF3754)	transmembrane protein, putative
Sapur.019G013700.1	21.03838071	4.23987875	0.306557503	13.83061	1.67E-43	8.16E-43	cyan	0.579228118	1.91E-15	GO:0005515,GO:0043531	AT4G27220.1		NB-ARC domain-containing disease resistance protein	NA
Sapur.006G128800.1	92.48079239	1.095869039	0.07957275	13.77191	3.76E-43	1.83E-42	cyan	0.718759961	3.02E-26	GO:0043565,GO:0006355,GO:0003700	AT5G44080.1		Basic-leucine zipper (bZIP) transcription factor family protein	NA
Sapur.006G096500.1	5496.975547	0.466755614	0.033920013	13.76048	4.41E-43	2.14E-42	cyan	0.729606168	2.30E-27	GO:0030955,GO:0006096,GO:0004743,GO:0000287	AT3G52990.1		Pyruvate kinase family protein	pyruvate kinase
Sapur.016G207700.1	561.2674084	0.735095989	0.053866005	13.64675	2.11E-42	1.01E-41	cyan	0.697284768	3.52E-24	GO:0019205,GO:0006139,GO:0005524	AT3G01820.1		P-loop containing nucleoside triphosphate hydrolases superfamily protein	adenylate kinase
Sapur.003G116400.1	591.9335268	0.411300564	0.030148568	13.64246	2.24E-42	1.08E-41	cyan	0.730813902	1.71E-27	GO:0034314,GO:0030833,GO:0015629,GO:0005885	AT1G30825.1	ARPC2A,DIS2	Arp2/3 complex, 34 kD subunit p34-Arc	actin-related protein 2/3 complex subunit
Sapur.005G013200.1	8.619220798	1.973528543	0.145106101	13.60059	3.97E-42	1.90E-41	cyan	0.71673935	4.81E-26	GO:0006950	AT3G62550.1		Adenine nucleotide alpha hydrolases-like superfamily protein	universal stress protein
Sapur.15WG142800.1	794.2352153	0.634688357	0.046780522	13.56736	6.25E-42	2.98E-41	cyan	0.727963782	3.42E-27		AT5G51830.1		pfkB-like carbohydrate kinase family protein	fructokinase
Sapur.014G054400.1	5.478320314	2.113696516	0.156565887	13.50036	1.56E-41	7.38E-41	cyan	0.707904574	3.53E-25	GO:0005515,GO:0006950,GO:0006355	AT3G61050.2	NTMC2T4,NTMC2TYPE4	Calcium-dependent lipid-binding (CaLB domain) family protein	integral membrane single C2 domain protein
Sapur.014G003300.1	535.0233703	0.412097365	0.030531098	13.49763	1.61E-41	7.65E-41	cyan	0.736323627	4.38E-28		AT3G49800.1		BSD domain-containing protein	BSD domain protein
Sapur.006G196400.1	391.3604544	0.49397238	0.036844204	13.40706	5.50E-41	2.58E-40	cyan	0.733224267	9.46E-28		AT5G11640.1		Thioredoxin superfamily protein	thioredoxin-related transmembrane protein
Sapur.15WG049600.1	4.590411179	3.860550474	0.288278504	13.39174	6.76E-41	3.17E-40	cyan	0.565143217	1.25E-14	GO:0006412,GO:0005840,GO:0005622,GO:0003735	AT3G16780.1		Ribosomal protein L19e family protein	ribosomal protein L19
Sapur.002G073500.1	4.429984245	3.866355386	0.288820382	13.38671	7.23E-41	3.39E-40	cyan	0.61086024	1.98E-17	GO:0030170,GO:0009058	AT2G13810.1	ALD1	AGD2-like defense response protein 1	aspartate aminotransferase
Sapur.006G186000.1	4530.592679	0.364836775	0.027370138	13.32974	1.55E-40	7.24E-40	cyan	0.722480472	1.26E-26	GO:0016787,GO:0005524,GO:0003677	AT3G13920.1	EIF4A1,RH4,TIF4A1	eukaryotic translation initiation factor 4A1	eukaryotic initiation factor 4A
Sapur.007G122000.1	643.9403493	0.434559164	0.032662485	13.30453	2.18E-40	1.01E-39	cyan	0.719566354	2.50E-26		AT2G20560.1		DNAJ heat shock family protein	heat-shock protein DnaJ
Sapur.013G008900.1	13181.70772	0.580512404	0.04402574	13.18575	1.06E-39	4.87E-39	cyan	0.709060556	2.73E-25	GO:0051082,GO:0006457,GO:0005783,GO:0005509	AT1G56340.2	AtCRT1a,CRT1,CRT1a	calreticulin 1a	NA
Sapur.016G102800.1	735.8280229	0.683936087	0.052115509	13.12347	2.42E-39	1.10E-38	cyan	0.716485832	5.10E-26		AT3G47300.1	SELT	SELT-like protein precursor	selenoprotein
Sapur.004G080500.1	1056.957525	0.635379809	0.048643695	13.06191	5.43E-39	2.46E-38	cyan	0.693672442	7.51E-24	GO:0052862,GO:0052861,GO:0016998	AT5G15870.1		glycosyl hydrolase family 81 protein	NA
Sapur.019G070200.1	615.4858393	-0.383521545	0.029409455	-13.0408	7.17E-39	3.24E-38	cyan	-0.731544183	1.43E-27	GO:0016021	AT1G22850.1		SNARE associated Golgi protein family	membrane protein SNARE domain protein
Sapur.003G044600.1	7.263028012	2.640116565	0.203860771	12.95059	2.33E-38	1.04E-37	cyan	0.592230917	3.10E-16		AT4G39720.1		VQ motif-containing protein	VQ motif protein
Sapur.012G061800.1	1341.551754	0.486122036	0.037560215	12.94247	2.59E-38	1.16E-37	cyan	0.704039432	8.24E-25		AT3G48680.1	GAMMA CAL2	gamma carbonic anhydrase-like 2	gamma carbonic anhydrase-like protein
Sapur.003G059500.1	245.3457149	-0.557694589	0.043104727	-12.9381	2.74E-38	1.22E-37	cyan	-0.735726817	5.08E-28		AT2G35230.1	IKU1	VQ motif-containing protein	VQ motif protein
Sapur.002G139900.1	581.4150926	0.335556497	0.025966384	12.92273	3.35E-38	1.49E-37	cyan	0.707577352	3.79E-25	GO:0007264,GO:0005525	AT5G65960.1		GTP binding	alpha/gamma adaptin-binding protein p34
Sapur.004G132800.1	61.97474389	0.993262082	0.076929504	12.91133	3.89E-38	1.73E-37	cyan	0.725754671	5.81E-27		AT5G47900.1		Protein of unknown function (DUF1624)	aquaporin PIP2-8, putative
Sapur.T052000.1	1361.847428	0.383209649	0.029730253	12.88955	5.15E-38	2.29E-37	cyan	0.711305883	1.65E-25	GO:0005524	AT4G29040.1	RPT2a	regulatory particle AAA-ATPase 2A	26S proteasome regulatory subunit-like protein
Sapur.T025700.1	135.1379023	0.523274646	0.040896857	12.79498	1.75E-37	7.69E-37	cyan	0.705258148	6.32E-25	GO:0055114,GO:0016616,GO:0051287,GO:0000287	AT4G35260.1	IDH-I,IDH1	isocitrate dehydrogenase 1	NA
Sapur.018G066500.1	10784.25401	0.486489251	0.038099927	12.76877	2.45E-37	1.07E-36	cyan	0.70345641	9.36E-25	GO:0006096,GO:0004332	AT2G36460.1		Aldolase superfamily protein	fructose-bisphosphate aldolase
Sapur.010G047200.1	15.28030038	2.041385064	0.15994959	12.76268	2.65E-37	1.16E-36	cyan	0.589202932	4.77E-16	GO:0055114,GO:0020037,GO:0006979,GO:0004601	AT1G14550.1		Peroxidase superfamily protein	lignin-forming anionic peroxidase, putative
Sapur.017G080300.1	2.584593545	3.529916863	0.278216503	12.68766	6.92E-37	3.01E-36	cyan	0.64384846	9.43E-20					hypothetical protein
Sapur.013G001200.1	3.000012449	4.195196238	0.332004407	12.63597	1.34E-36	5.78E-36	cyan	0.551938105	6.75E-14					NA
Sapur.002G107200.1	120.6626854	0.618146467	0.048958735	12.62587	1.52E-36	6.56E-36	cyan	0.702762986	1.09E-24	GO:0006464	AT1G47578.1		Biotin/lipoate A/B protein ligase family	lipoate-protein ligase B
Sapur.014G088200.1	99.446167	-1.158831783	0.092621638	-12.5115	6.46E-36	2.75E-35	cyan	-0.743627978	6.80E-29		AT4G01680.1	AtMYB55,MYB55	myb domain protein 55	R2R3-MYB transcription factor, putative
Sapur.15WG144600.1	539.1347829	0.39951671	0.032013588	12.4796	9.65E-36	4.09E-35	cyan	0.692166365	1.03E-23	GO:0046872,GO:0005515	AT4G25440.1	ZFWD1	zinc finger WD40 repeat protein 1	zinc finger CCCH domain protein
Sapur.007G023000.1	127.6116752	0.829626096	0.066508589	12.47397	1.04E-35	4.39E-35	cyan	0.712636402	1.22E-25	GO:0015031,GO:0008565,GO:0030123	AT3G50860.1		Clathrin adaptor complex small chain family protein	AP-3 complex subunit sigma protein
Sapur.011G076900.1	27.71624448	-1.370205214	0.110713048	-12.3762	3.52E-35	1.47E-34	cyan	-0.663270216	2.95E-21	GO:0006468,GO:0004672	AT1G53440.1		Leucine-rich repeat transmembrane protein kinase	NA
Sapur.003G131500.1	8.663215712	2.247703033	0.181747749	12.36716	3.94E-35	1.65E-34	cyan	0.642184753	1.25E-19		AT2G17030.1		F-box family protein with a domain of unknown function (DUF295)	NA

Sapur.009G105800.1	2567.543365	0.252494922	0.020471612	12.33391	5.95E-35	2.48E-34	cyan	0.675395158	2.97E-22	GO:0008540,GO:0006511	AT4G38630.1	ATMCB1.MBP1.MCB1.RPN1	regulatory particle non-ATPase 10	26S proteasome non-ATPase regulatory subunit, putative
Sapur.019G031400.1	8574.634435	0.68734918	0.056035304	12.26636	1.37E-34	5.68E-34	cyan	0.684215457	5.20E-23	GO:0016788	AT5G33370.1		GDSL-like Lipase/Acylhydrolase superfamily protein	GDSL esterase/lipase
Sapur.005G192400.1	557.2888392	0.619394622	0.050865741	12.17705	4.12E-34	1.69E-33	cyan	0.700761939	1.67E-24	GO:0003924,GO:0007017,GO:0005874	AT1G20010.1	TUB5	tubulin beta-5 chain	beta-tubulin
Sapur.014G048500.1	186.9561816	0.524431464	0.043327055	12.10402	1.01E-33	4.10E-33	cyan	0.679145406	1.43E-22	GO:0016021,GO:0006888,GO:0005801,GO:0000139	AT2G45200.1	ATGOS12,GOS12	golgi snare 12	SNAP receptor complex protein
Sapur.017G118800.1	646.6926164	0.563319164	0.046700541	12.06237	1.67E-33	6.78E-33	cyan	0.677587515	1.94E-22	GO:0022904,GO:0016651,GO:0005743	AT5G52840.1		NADH-ubiquinone oxidoreductase-related	NADH dehydrogenase [ubiquinone] 1 alpha subcomplex subunit 5, putative
Sapur.002G109200.1	3.442110172	2.200558935	0.184744172	11.91138	1.03E-32	4.13E-32	cyan	0.656869791	9.50E-21	GO:0019205,GO:0006139,GO:0005524	AT5G26667.3	PYR6	P-loop containing nucleoside triphosphate hydrolases superfamily protein	adenylate kinase
Sapur.006G179400.1	1833.363358	0.330781088	0.02777223	11.9105	1.04E-32	4.17E-32	cyan	0.685293884	4.19E-23	GO:0055114,GO:0016491	AT5G20080.1		FAD/NAD(P)-binding oxidoreductase	NADH-cytochrome b5 reductase, putative
Sapur.016G024500.1	30.68064079	1.430405541	0.120627365	11.85805	1.95E-32	7.77E-32	cyan	0.653983924	1.60E-20		AT2G01570.1	RGA,RGA1	GRAS family transcription factor family protein	NA
Sapur.016G133800.1	702.5801757	0.686461261	0.057936498	11.84851	2.19E-32	8.69E-32	cyan	0.673050183	4.67E-22		AT1G07540.1	TRFL2	TRF-like 2	double-strand telomere-binding protein, putative
Sapur.016G092200.1	144.236303	1.271253486	0.10812398	11.75737	6.47E-32	2.54E-31	cyan	0.653557309	1.72E-20	GO:0055085,GO:0016021,GO:0016020,GO:0006810,GO:0005215	AT2G37900.1		Major facilitator superfamily protein	peptide transporter, putative
Sapur.010G057600.1	199.6144483	0.477560673	0.040839656	11.69355	1.38E-31	5.36E-31	cyan	0.675270728	3.04E-22	GO:0008270,GO:0005515	AT4G14220.1	RHF1A	RING-H2 group F1A	RING-H2 zinc finger protein
Sapur.002G124600.1	862.8584789	0.363718444	0.031162043	11.67184	1.78E-31	6.90E-31	cyan	0.663298121	2.94E-21	GO:0016887,GO:0005524	AT1G01910.4		P-loop containing nucleoside triphosphate hydrolases superfamily protein	ATPase ASNA1-like protein
Sapur.002G143200.1	240.8009142	-0.66959144	0.057511175	-11.6428	2.50E-31	9.67E-31	cyan	-0.713316502	1.05E-25		AT4G00950.1	MEE47	Protein of unknown function (DUF688)	DUF688 family protein
Sapur.010G100700.1	2.209370223	3.233772497	0.278624079	11.60622	3.83E-31	1.48E-30	cyan	0.653008611	1.90E-20		AT1G68800.1	BRC2,TCP12	TCP domain protein 12	TCP transcription factor, putative
Sapur.017G063500.1	2084.646333	0.442118853	0.038275773	11.55088	7.31E-31	2.80E-30	cyan	0.657106296	9.11E-21	GO:0008152,GO:0003824	AT2G34590.1		Transketolase family protein	pyruvate dehydrogenase E1 component subunit beta
Sapur.018G031700.1	99.99284869	0.473110217	0.040974891	11.54634	7.70E-31	2.95E-30	cyan	0.676581889	2.36E-22		AT4G32750.1			transmembrane protein, putative
Sapur.15WG004900.1	259.1224481	0.699304388	0.060692373	11.52211	1.02E-30	3.90E-30	cyan	0.634580029	4.52E-19		AT5G53650.1			transmembrane protein, putative
Sapur.016G056800.1	835.4532663	0.395111754	0.034397065	11.48679	1.54E-30	5.85E-30	cyan	0.651427812	2.51E-20	GO:0016020	AT5G06660.1		Protein of unknown function DUF106, transmembrane	transmembrane and coiled-coil protein
Sapur.004G176100.1	758.4002891	0.66911142	0.058399587	11.45747	2.16E-30	8.18E-30	cyan	0.646211649	6.27E-20	GO:0030145,GO:0006007,GO:0005737,GO:0004619,GO:0046872,GO:0003824	AT1G09780.1		Phosphoglycerate mutase, 2,3-bisphosphoglycerate-independent	2%2C3-bisphosphoglycerate-independent phosphoglycerate mutase
Sapur.008G144500.1	1649.103058	0.547836816	0.048844941	11.21584	3.41E-29	1.26E-28	cyan	0.665048367	2.12E-21		AT1G24360.1		NAD(P)-binding Rossmann-fold superfamily protein	3-ketoacyl-(acyl-carrier) reductase
Sapur.011G058100.1	2540.941109	0.38990934	0.034832259	11.19391	4.37E-29	1.61E-28	cyan	0.669583724	9.04E-22		AT5G46290.1	KAS I,KAS1	3-ketoacyl-acyl carrier protein synthase I	3-oxoacyl-[acyl-carrier] synthase I
Sapur.002G059000.1	60.76219283	0.936953304	0.084117749	11.13859	8.14E-29	2.99E-28	cyan	0.619096303	5.52E-18		AT1G77010.1		Pentatricopeptide repeat (PPR) superfamily protein	PPR containing plant protein, putative
Sapur.008G149100.1	1271.901214	0.283536518	0.02559642	11.07719	1.62E-28	5.90E-28	cyan	0.662098569	3.66E-21		AT3G26370.1		O-fucosyltransferase family protein	auxin-independent growth promoter-like protein
Sapur.012G110000.1	340.0525497	1.011238163	0.091928985	11.00021	3.81E-28	1.38E-27	cyan	0.609236508	2.53E-17		AT5G52420.1			plant/K24M7-17 protein, putative
Sapur.006G124300.1	1.284218752	3.759348863	0.342647931	10.97146	5.24E-28	1.89E-27	cyan	0.574027434	3.87E-15					NA
Sapur.15WG022900.1	317.4946252	0.341880856	0.031203821	10.95638	6.19E-28	2.23E-27	cyan	0.673506385	4.27E-22		AT4G27750.1	ISI1	binding	impaired sucrose induction-like protein
Sapur.004G151700.1	820.9714497	0.512590419	0.04725691	10.84689	2.06E-27	7.35E-27	cyan	0.615171919	1.02E-17		AT2G27730.1		copper ion binding	F1F0-ATPase inhibitor protein, putative
Sapur.016G266900.1	425.5995406	0.524553926	0.048551889	10.80399	3.30E-27	1.17E-26	cyan	0.624799825	2.23E-18	GO:0006950	AT5G54430.1	ATPHOS32,PHOS32	Adenine nucleotide alpha hydrolases-like superfamily protein	universal stress family protein
Sapur.016G170200.1	1322.191638	0.371184928	0.034454811	10.77309	4.61E-27	1.63E-26	cyan	0.641117778	1.50E-19		AT2G14260.1	PIP	proline iminopeptidase	proline iminopeptidase
Sapur.009G122500.1	419.5854479	0.427292276	0.039888879	10.71207	8.93E-27	3.13E-26	cyan	0.607183715	3.45E-17	GO:0005515	AT3G03740.1	ATBPM4,BPM4	BTB-POZ and MATH domain 4	BTB-POZ and MATH domain protein
Sapur.15WG052400.1	11.06085212	2.031110713	0.189734947	10.70499	9.64E-27	3.38E-26	cyan	0.491430314	6.35E-11	GO:0006412,GO:0005840,GO:0005622,GO:0003735	AT3G16780.1		Ribosomal protein L19e family protein	NA
Sapur.014G065500.1	17.86485295	0.959164936	0.089792436	10.68202	1.24E-26	4.32E-26	cyan	0.64933322	3.64E-20		AT1G01690.1	ATPRD3,PRD3	putative recombination initiation defects 3	recombination initiation defects protein, putative
Sapur.007G047900.1	538.6190636	0.463667979	0.043442761	10.67308	1.36E-26	4.75E-26	cyan	0.595232658	2.01E-16	GO:0016020,GO:0006810,GO:0005215	AT2G23150.1	ATNRAMP3,NRAMP3	natural resistance-associated macrophage protein 3	metal transporter NRAMP2
Sapur.016G125500.1	130.2295467	0.970517643	0.091211586	10.64029	1.94E-26	6.73E-26	cyan	0.565185259	1.25E-14		AT2G44300.1		Bifunctional inhibitor/lipid-transfer protein/seed storage 2S albumin superfamily protein	protease inhibitor/seed storage/lipid transfer family protein
Sapur.016G026000.1	1.067233249	2.888925916	0.272758264	10.59152	3.26E-26	1.13E-25	cyan	0.604200333	5.40E-17	GO:0006511	AT1G75950.1	ASK1.ATSKP1.SKP1.SKP1A.U	S phase kinase-associated protein 1	Skp1 family, tetramerisation domain protein
Sapur.014G084600.1	85.95567884	0.693901549	0.065710777	10.55994	4.57E-26	1.57E-25	cyan	0.766187646	1.42E-31	GO:0006796,GO:0005737,GO:0004427,GO:0000287	AT1G01050.1	AtPPa1,PPa1	pyrophosphorylase 1	soluble inorganic pyrophosphatase
Sapur.T111700.1	2.085830123	3.391519283	0.321222942	10.55815	4.66E-26	1.60E-25	cyan	0.531100459	8.33E-13					NA
Sapur.018G084200.1	174.6756612	0.350130336	0.033228581	10.53702	5.83E-26	2.00E-25	cyan	0.637749118	2.66E-19	GO:0016887,GO:0005524	AT1G63270.1	ATNAP10,NAP10	non-intrinsic ABC protein 10	ATP-binding ABC transporter
Sapur.016G269800.1	11.67699039	1.812027144	0.172234361	10.52071	6.94E-26	2.38E-25	cyan	0.588685903	5.13E-16	GO:0004674,GO:0006468,GO:0004672	AT4G27300.1		S-locus lectin protein kinase family protein	Serine/Threonine kinase domain protein
Sapur.002G139800.1	41.70182287	-0.822783912	0.07834118	-10.5026	8.41E-26	2.88E-25	cyan	-0.646923745	5.54E-20		AT1G01180.1		S-adenosyl-L-methionine-dependent methyltransferases superfamily protein	methyltransferase, putative
Sapur.004G127400.1	4736.1514	0.35101061	0.033538891	10.46578	1.24E-25	4.23E-25	cyan	0.639713837	1.91E-19	GO:0005789	AT2G16595.1		Translocon-associated protein (TRAP), alpha subunit	translocon-associated protein subunit alpha
Sapur.T064400.1	994.5769549	0.393558643	0.037677233	10.44553	1.54E-25	5.23E-25	cyan	0.624099113	2.50E-18		AT1G16560.2		Per1-like family protein	post-GPI attachment to protein factor
Sapur.15WG139800.1	15452.10517	0.42170828	0.040374226	10.44499	1.54E-25	5.26E-25	cyan	0.634369998	4.68E-19	GO:0006096,GO:0004634,GO:0000287,GO:0000015	AT2G36530.1	ENO2,LOS2	Enolase	phosphopyruvate hydratase
Sapur.002G033600.1	300.9366628	0.430036253	0.041333188	10.40414	2.37E-25	8.04E-25	cyan	0.625314818	2.05E-18	GO:0046872	AT2G30580.1	DRIP2	DREB2A-interacting protein 2	E3 ubiquitin protein ligase DRIP2
Sapur.016G193700.1	541.9771928	0.31295269	0.030159017	10.37675	3.16E-25	1.07E-24	cyan	0.645042206	7.68E-20		AT3G23910.1			reverse transcriptase-like protein
Sapur.013G042500.1	5.034018154	1.966153123	0.190119773	10.34166	4.57E-25	1.54E-24	cyan	0.553070917	5.86E-14					NA
Sapur.004G104200.1	162.734366	1.24938471	0.121140635	10.31351	6.12E-25	2.06E-24	cyan	0.686795639	3.09E-23		AT5G15240.1		Transmembrane amino acid transporter family protein	amino acid transporter, putative
Sapur.013G098100.1	4.64037626	2.257200701	0.220313065	10.24542	1.24E-24	4.13E-24	cyan	0.529701523	9.80E-13	GO:0055114,GO:0016491,GO:0010181	AT1G76690.1	ATOPR2,OPR2	12-oxophytodienoate reductase 2	NA
Sapur.017G033600.1	7.942681076	1.404357617	0.137859048	10.18691	2.27E-24	7.51E-24	cyan	0.573392568	4.21E-15					NA
Sapur.006G026900.1	30.86088542	-0.784089016	0.076994357	-10.1837	2.34E-24	7.76E-24	cyan	-0.617706188	6.86E-18					NA
Sapur.016G136600.1	3.261787017	1.769053838	0.173846845	10.17593	2.54E-24	8.39E-24	cyan	0.629261216	1.08E-18		AT2G29150.1		NAD(P)-binding Rossmann-fold superfamily protein	3-ketoacyl-(acyl-carrier) reductase
Sapur.017G083300.1	1738.71352	0.477035147	0.046910566	10.16903	2.73E-24	9.00E-24	cyan	0.620569894	4.37E-18	GO:0055114,GO:0016616,GO:0006694,GO:0003854,GO:0019752	AT3G47520.1	MDH	malate dehydrogenase	NAD-malate dehydrogenase
Sapur.006G171400.1	140.2015046	0.526124401	0.051766355	10.16344	2.89E-24	9.53E-24	cyan	0.609710215	2.35E-17		AT3G15040.1		Protein of unknown function, DUF584	senescence regulator
Sapur.017G007100.1	447.3853801	-0.783499818	0.077574161	-10.1	5.52E-24	1.81E-23	cyan	-0.620577938	4.37E-18	GO:0006355,GO:0003700	AT5G44210.1	ATERF-9,ATERF9,ERF9	erf domain protein 9	AP2/ERF domain transcription factor
Sapur.016G141600.1	512.10474	1.007327924	0.100242362	10.04892	9.29E-24	3.02E-23	cyan	0.613732944	1.27E-17	GO:0019140,GO:0010264,GO:0005524	AT5G58730.1		pfkB-like carbohydrate kinase family protein	pfkB family carbohydrate kinase
Sapur.T189400.1	171.5103895	0.489086618	0.048732005	10.03625	1.06E-23	3.43E-23	cyan	0.610659172	2.04E-17	GO:0006355,GO:0005739,GO:0003690	AT5G07900.1		Mitochondrial transcription termination factor family protein	NA
Sapur.152G053800.1	113.7899716	0.565591447	0.056630359	9.987425	1.73E-23	5.59E-23	cyan	0.583501458	1.06E-15		AT1G18485.1		Pentatricopeptide repeat (PPR) superfamily protein	pentatricopeptide (PPR) repeat protein, putative
Sapur.017G038600.1	655.5293729	-0.699458729	0.070178243	-9.96689	2.13E-23	6.86E-23	cyan	-0.641282928	1.46E-19		AT5G64310.1	AGP1,ATAGP1	arabinogalactan protein 1	transmembrane protein, putative
Sapur.15WG018500.1	416.7995114	0.358462674	0.035998979	9.957579	2.34E-23	7.53E-23	cyan	0.600025167	1.00E-16	GO:0016020,GO:0008378,GO:0006486	AT5G53340.1		Galactosyltransferase family protein	beta-1%2C3-galactosyltransferase sqv-2, putative
Sapur.007G100700.1	238.0975904	-0.657007302	0.066376149	-9.89824	4.24E-23	1.36E-22	cyan	-0.613355645	1.35E-17	GO:0005515,GO:0016567	AT5G64330.1	JK218,NPH3,RPT3	Phototropic-responsive NPH3 family protein	root phototropism protein
Sapur.019G060800.1	627.017638	0.444824673	0.044992302	9.886684	4.76E-23	1.52E-22	cyan	0.629835737	9.87E-19	GO:0055114,GO:0046872,GO:0006801,GO:0004784	AT3G10920.1	ATMSD1,MEE33,MSD1	manganese superoxide dismutase 1	superoxide dismutase [Mn] protein
Sapur.T148900.1	118.0409527	1.189921588	0.120895193	9.842588	7.38E-23	2.35E-22	cyan	0.572374416	4.82E-15	GO:0016020,GO:0006810,GO:0005215	AT1G15960.1	ATNRAMP6,NRAMP6	NRAMP metal ion transporter 6	NA
Sapur.001G091100.1	2698.894817	0.526953125	0.05358265	9.834398	8.00E-23	2.55E-22	cyan	0.583031411	1.13E-15	GO:0055114,GO:0016614,GO:0050660	AT1G12570.1		Glucose-methanol-choline (GMC) oxidoreductase family protein	glucose-methanol-choline (GMC) oxidoreductase, putative
Sapur.019G036100.1	31.73562238	0.806332927	0.082229965	9.805828	1.06E-22	3.37E-22	cyan	0.553938208	5.26E-14	GO:0043531	AT3G50950.2	ZAR1	HOPZ-ACTIVATED RESISTANCE 1	NBS-LRR disease resistance protein, putative
Sapur.004G170700.1	112.784391	0.473609089	0.048449541	9.775306	1.44E-22	4.55E-22	cyan	0.611020339	1.93E-17		AT3G51580.1			transmembrane protein, putative
Sapur.018G073000.1	19.80626772	1.004376728	0.102887546	9.761888	1.64E-22	5.19E-22	cyan	0.5503371	8.24E-14	GO:0016020,GO:0004970	AT2G29100.1	ATGLR2.9,GLR2.9	glutamate receptor 2.9	glutamate receptor
Sapur.008G127300.1	95.24431386	-1.142143291	0.117747564	-9.69993	3.02E-22	9.47E-22	cyan	-0.562054772	1.87E-14	GO:0055114,GO:0010309	AT5G43850.1	ARD4,ATARD4	RmlC-like cupins superfamily protein	1%2C2-dihydroxy-3-keto-5-methylthiopentene dioxygenase
Sapur.016G240600.1	3538.027108	0.317391105	0.032877992	9.653604	4.75E-22	1.48E-21	cyan	0.601047288	8.61E-17	GO:0055114,GO:0016616,GO:0006694,GO:0003854,GO:0019752	AT1G53240.1	mMDH1	Lactate/malate dehydrogenase family protein	malate dehydrogenase
Sapur.011G075900.1	2072.431252	0.318878031	0.033209425	9.602034	7.84E-22	2.44E-21	cyan	0.615347922	9.91E-18	GO:0016020,GO:0015031	AT2G34250.1		SecY protein transport family protein	preprotein translocase, SecY subunit
Sapur.003G061300.1	543.6483703	0.322381595	0.033609831	9.591884	8.65E-22	2.68E-21	cyan	0.615548246	9.60E-18	GO:0052726,GO:0052725,GO:0047325,GO:0032957,GO:0005622,GO:0005524,GO:000028	AT5G16760.1		Inositol 1,3,4-trisphosphate 5/6-kinase family protein	inositol-tetrakisphosphate 1-kinase
Sapur.009G010800.1	7.878245935	1.376368367	0.144234925	9.542546	1.39E-21	4.30E-21	cyan	0.56630866	1.08E-14		AT5G06100.2	ATMYB33,MYB33	myb domain protein 33	transcription factor Myb3, putative
Sapur.018G015000.1	7.963822211	1.620259686	0.170258368	9.516476	1.79E-21	5.52E-21	cyan	0.574872608	3.45E-15	GO:0007165,GO:0006468,GO:0005524,GO:0004672	AT5G10930.1	CIPK5,SnRK3.24	CBL-interacting protein kinase 5	calcineurin B-like protein interacting kinase
Sapur.010G070600.1	329.8587611	0.277518093	0.029520308	9.400921	5.41E-21	1.65E-20	cyan	0.596015298	1.80E-16		AT1G67960.1			transmembrane anterior posterior transformation-like protein, putative
Sapur.018G039600.1	254.2128412	0.300084735	0.032012723	9.373921	6.99E-21	2.12E-20	cyan	0.583830236	1.01E-15	GO:0006413,GO:0003743	AT5G11900.1		Translation initiation factor SUI1 family protein	translation initiation factor SUI1
Sapur.002G013100.1	1.715525625	2.473726663	0.265369413	9.321823	1.14E-20	3.45E-20	cyan	0.586228779	7.25E-16	GO:0006511	AT5G42190.1	ASK2,SKP1B	E3 ubiquitin ligase SCF complex subunit SKP1/ASK1 family protein	SKP1-like protein
Sapur.002G022100.1	252.9893423	-0.444334175	0.047848797	-9.28621	1.60E-20	4.80E-20	cyan	-0.612925088	1.44E-17	GO:0005525,GO:0003924,GO:0000287	AT5G18570.1	ATOBGC,CPSAR1,EMB269	GTP1/OBG family protein	NA
Sapur.004G106500.1	1549.018707	0.60599857	0.065661234	9.229168	2.73E-20	8.14E-20	cyan	0.556238346	3.93E-14	GO:0008184,GO:0005975	AT3G29320.1		Glycosyl transferase, family 35	alpha-1%2C4 glucan phosphorylase L isozyme/amyloplastic protein
Sapur.017G110700.1	167.390224	-0.447280755	0.048495084	-9.22322	2.88E-20	8.60E-20	cyan	-0.579587139	1.82E-15	GO:0043531	AT3G14470.1		NB-ARC domain-containing disease resistance protein	CC-NBS-LRR resistance protein
Sapur.T100100.1	0.685508613	2.686453821	0.294751998	9.114285	7.92E-20	2.33E-19	cyan	0.574171329	3.79E-15					NA
Sapur.008G059400.1	121.9384043	0.44465036	0.049078667	9.059952	1.31E-19	3.82E-19	cyan	0.558910734	2.80E-14		AT2G38450.1			Sel1 repeat protein
Sapur.009G098400.1	27.19651043	-0.579400385	0.064048152	-9.04632	1.48E-19	4.33E-19	cyan	-0.569136702	7.42E-15		AT4G38980.1			hypothetical protein
Sapur.009G131500.1	390.7752253	0.358111782	0.03984342	8.987978	2.52E-19	7.33E-19	cyan	0.578667698	2.06E-15	GO:0055085,GO:0016021	AT5G44370.1	PHT4;6	phosphate transporter 4;6	anion transporter, putative
Sapur.009G124000.1	576.1160644	0.294639264	0.032803095	8.982057	2.66E-19	7.73E-19	cyan	0.556939244	3.60E-14		AT5G21070.1			plant/T10F18-100 protein
Sapur.002G079000.1	878.3502162	-0.842836899	0.094007253	-8.96566	3.08E-19	8.96E-19	cyan	-0.570587854	6.12E-15		AT4G08950.1	EXO	Phosphate-responsive 1 family protein	phosphate-induced protein 1 motif protein
Sapur.002G146200.1	615.3922564	0.347294434	0.038771499	8.957467	3.32E-19	9.64E-19	cyan	0.564035171	1.45E-14		AT3G58800.1			transmembrane protein, putative
Sapur.002G045500.1	162.2692452	0.439112637	0.049104783	8.94236	3.81E-19	1.10E-18	cyan	0.559746802	2.52E-14		AT4G39235.1			hypothetical protein
Sapur.002G049400.1	1.436606492	2.429898269	0.273080463	8.898104	5.68E-19	1.64E-18	cyan	0.574832037	3.47E-15		AT5G63190.2		MA3 domain-containing protein	NA
Sapur.008G066700.1	5345.321732	0.345402653	0.039002959	8.855806	8.31E-19	2.38E-18	cyan	0.541564284	2.41E-13	GO:0006096,GO:0004618	AT1G79550.1	PGK	phosphoglycerate kinase	phosphoglycerate kinase
Sapur.005G046500.1	11.27936613	0.859760284	0.097161106	8.848811	8.85E-19	2.54E-18	cyan	0.549705476	8.91E-14		AT1G48880.1	TBL7	TRICHOME BIREFRINGENCE-LIKE 7	Pmr5/Cas1p GDSL/SGNH-like acyl-esterase family protein
Sapur.016G248100.1	1119.310869	-0.210825242	0.024025918	-8.77491	1.71E-18	4.87E-18	cyan	-0.582055506	1.29E-15	GO:0016787,GO:0006541,GO:0008242,GO:0003824	AT1G78680.1	ATGGH2,GGH2	gamma-glutamyl hydrolase 2	gamma-glutamyl hydrolase
Sapur.010G141300.1	109.5351882	-0.82806045	0.094483009	-8.76412	1.88E-18	5.35E-18	cyan	-0.554048192	5.19E-14	GO:0003333	AT3G56200.1		Transmembrane amino acid transporter family protein	sodium-coupled neutral amino acid transporter
Sapur.006G193000.1	229.3147113	0.3052911	0.034857724	8.758205	1.98E-18	5.64E-18	cyan	0.548915793	9.83E-14		AT4G32930.1			DUF866 domain protein
Sapur.016G092100.1	552.5854991	0.442909936	0.050764942	8.724721	2.67E-18	7.56E-18	cyan	0.537143742	4.09E-13		AT3G53950.1		glyoxal oxidase-related protein	glyoxal oxidase
Sapur.012G028100.1	2047.435527	0.334063872	0.03833652	8.713985	2.93E-18	8.30E-18	cyan	0.569734303	6.85E-15	GO:0016021,GO:0006886,GO:0005783	AT1G48440.1		B-cell receptor-associated 31-like	erwinia induced protein
Sapur.001G121800.1	663.3413651	0.287024593	0.033196922	8.646121	5.33E-18	1.50E-17	cyan	0.580319814	1.64E-15	GO:0016868,GO:0005975,GO:0071704	AT5G18070.1	DRT101	phosphoglucosamine mutase-related	phosphoacetylglucosamine mutase
Sapur.016G162100.1	47.04269756	0.701133457	0.081333219	8.620505	6.67E-18	1.87E-17	cyan	0.574889657	3.44E-15	GO:0055114,GO:0020037,GO:0016705,GO:0005506	AT3G56630.1	CYP94D2	cytochrome P450, family 94, subfamily D, polypeptide 2	NA
Sapur.004G169000.1	347.5421052	-0.278324689	0.032370169	-8.59818	8.10E-18	2.27E-17	cyan	-0.553429432	5.60E-14		AT5G01850.1		Protein kinase superfamily protein	kinase domain protein
Sapur.016G157900.1	157.8026969	0.400394295	0.046753525	8.563938	1.09E-17	3.04E-17	cyan	0.564239763	1.41E-14		AT5G12260.1			transferring glycosyl group transferase, putative
Sapur.006G011600.1	7.855372681	1.105207546	0.129531115	8.532371	1.43E-17	3.98E-17	cyan	0.552523137	6.28E-14		AT4G16460.1			hypothetical protein
Sapur.T189000.1	0.583499945	2.576100324	0.3025145	8.515626	1.66E-17	4.59E-17	cyan	0.575631681	3.11E-15					NA
Sapur.009G125900.1	597.3500039	-0.487840686	0.057938555	-8.41997	3.77E-17	1.03E-16	cyan	-0.578388421	2.14E-15		AT3G11590.1			plant/F24K9-26 protein, putative
Sapur.007G093900.1	96.58041844	0.499495314	0.059901433	8.33862	7.52E-17	2.04E-16	cyan	0.53306581	6.62E-13		AT4G33470.1	ATHDA14,hda14	histone deacetylase 14	NA
Sapur.009G084500.1	298.1281789	-0.689682618	0.082751979	-8.33433	7.79E-17	2.11E-16	cyan	-0.572946841	4.47E-15	GO:0055114,GO:0016491	AT3G19000.1		2-oxoglutarate (2OG) and Fe(II)-dependent oxygenase superfamily protein	2-oxoglutarate (2OG) and Fe(II)-dependent oxygenase-like protein
Sapur.003G020800.1	4456.199938	0.278022671	0.033725514	8.24369	1.67E-16	4.49E-16	cyan	0.527349434	1.29E-12	GO:0016787,GO:0005524,GO:0003677	AT1G54270.1	EIF4A-2	eif4a-2	eukaryotic initiation factor 4A-2
Sapur.016G125600.2	39.98847468	-0.537329982	0.065258986	-8.23381	1.81E-16	4.87E-16	cyan	-0.543925165	1.81E-13		AT3G60070.1		Major facilitator superfamily protein	plant/T2O9-50 protein
Sapur.003G104400.1	173.485178	0.293626623	0.035671895	8.231316	1.85E-16	4.97E-16	cyan	0.521750918	2.43E-12		AT4G10925.3		Nuclear transport factor 2 (NTF2) family protein	F-box protein SKIP8
Sapur.016G021900.2	1.984039294	2.278346739	0.277949744	8.196974	2.47E-16	6.60E-16	cyan	0.530911678	8.51E-13		AT2G41050.1		PQ-loop repeat family protein / transmembrane family protein	PQ loop protein
Sapur.008G117200.1	9.725549031	-1.178729126	0.144151128	-8.17704	2.91E-16	7.76E-16	cyan	-0.558644116	2.90E-14	GO:0055114,GO:0016491	AT4G21200.1	ATGA2OX8,GA2OX8	gibberellin 2-oxidase 8	gibberellin 2-beta-dioxygenase
Sapur.009G105400.1	37.67717677	0.929348113	0.118043264	7.872945	3.46E-15	8.97E-15	cyan	0.503018969	1.90E-11		AT4G34950.1		Major facilitator superfamily protein	nodulin family protein
Sapur.016G277000.1	695.3923274	0.363750776	0.046205954	7.872379	3.48E-15	9.01E-15	cyan	0.5187623	3.41E-12	GO:0045454	AT3G20560.1	ATPDI12.ATPDIL5-3.PDI12.F	PDI-like 5-3	protein disulfide isomerase
Sapur.008G058500.1	140.9263339	-0.455093139	0.057879228	-7.86281	3.76E-15	9.72E-15	cyan	-0.511574131	7.54E-12	GO:0006355,GO:0003700	AT2G40340.1	AtERF48,DREB2C	Integrase-type DNA-binding superfamily protein	dehydration responsive element-binding protein
Sapur.002G004300.1	751.2664627	0.331424044	0.042327753	7.829947	4.88E-15	1.26E-14	cyan	0.499940919	2.62E-11	GO:0016021,GO:0006486,GO:0005783,GO:0004579	AT1G76400.1		Ribophorin I	dolichyl-diphosphooligosaccharide-protein glycosyltransferase subunit 1
Sapur.001G121900.1	858.1024823	0.329510374	0.042172264	7.813438	5.56E-15	1.43E-14	cyan	0.505970333	1.38E-11		AT5G45420.1		Duplicated homeodomain-like superfamily protein	Myb DNA-binding domain protein, putative
Sapur.005G200600.1	435.947108	0.286333337	0.036670668	7.808239	5.80E-15	1.49E-14	cyan	0.523067228	2.10E-12	GO:0008270,GO:0006508,GO:0004181	AT5G42320.1		Zn-dependent exopeptidases superfamily protein	zinc carboxypeptidase
Sapur.15WG117900.1	672.2227211	0.99092995	0.127828358	7.752035	9.04E-15	2.31E-14	cyan	0.5193449	3.19E-12	GO:0016021	AT5G50790.1		Nodulin MtN3 family protein	sugar efflux transporter for intercellular exchange protein
Sapur.014G113500.1	252.9087535	0.50518117	0.065708636	7.688201	1.49E-14	3.79E-14	cyan	0.473097388	3.93E-10		AT5G47920.1			plant/MAC12-16 protein, putative
Sapur.010G124500.1	601.4914031	-0.462564656	0.060946455	-7.58969	3.21E-14	8.06E-14	cyan	-0.510296493	8.67E-12		AT2G03420.1			NA
Sapur.001G023300.1	35.40270082	0.601262806	0.079231297	7.588703	3.23E-14	8.11E-14	cyan	0.596058016	1.79E-16		AT5G13500.1			root determined nodulation protein
Sapur.T068300.1	1.151700532	1.928706469	0.25709207	7.502007	6.28E-14	1.57E-13	cyan	0.604135492	5.45E-17		AT4G15110.1	CYP97B3	cytochrome P450, family 97, subfamily B, polypeptide 3	NA
Sapur.014G092800.1	192.0320496	-0.691288417	0.092325955	-7.48748	7.02E-14	1.75E-13	cyan	-0.509365124	9.59E-12	GO:0016021,GO:0006820,GO:0016020,GO:0005452	AT3G62270.1		HCO3- transporter family	boron transporter
Sapur.003G050700.1	589.8474337	-0.780923167	0.104964485	-7.43988	1.01E-13	2.49E-13	cyan	-0.524862893	1.71E-12					hypothetical protein
Sapur.010G132700.1	4153.205394	0.24587151	0.033102351	7.427615	1.11E-13	2.73E-13	cyan	0.489413456	7.80E-11	GO:0006096,GO:0004618	AT1G79550.2	PGK	phosphoglycerate kinase	phosphoglycerate kinase
Sapur.T102000.1	0.479666545	2.334116856	0.314854642	7.413316	1.23E-13	3.04E-13	cyan	0.564306021	1.40E-14					NA
Sapur.010G097000.1	1513.40392	0.280327441	0.038095545	7.358536	1.86E-13	4.56E-13	cyan	0.513349958	6.21E-12	GO:0016746,GO:0008152	AT3G25860.1	LTA2,PLE2	2-oxoacid dehydrogenases acyltransferase family protein	pyruvate dehydrogenase complex dihydrolipoyllysine-residue acetyltransferase component
Sapur.016G193400.1	98.73808962	0.366612446	0.050047379	7.325308	2.38E-13	5.82E-13	cyan	0.484943943	1.22E-10	GO:0016811,GO:0016021,GO:0006672	AT4G22330.1	ATCES1	Alkaline phytoceramidase (aPHC)	alkaline ceramidase
Sapur.014G096200.1	539.8708235	0.438723786	0.060796646	7.21625	5.34E-13	1.29E-12	cyan	0.483137763	1.47E-10		AT2G16760.1		Calcium-dependent phosphotriesterase superfamily protein	calcium-dependent phosphotriesterase-like protein
Sapur.008G070800.1	376.425993	0.463396504	0.066031829	7.017775	2.25E-12	5.33E-12	cyan	0.496914676	3.60E-11	GO:0042546,GO:0016020,GO:0008107	AT2G03220.1	ATFT1,ATFUT1,FT1,MUR2	fucosyltransferase 1	galactoside 2-alpha-L-fucosyltransferase
Sapur.005G060200.1	3101.769525	-0.365678115	0.053138803	-6.88156	5.92E-12	1.38E-11	cyan	-0.532025398	7.48E-13	GO:0006355,GO:0003700	AT1G78080.1	RAP2.4	related to AP2 4	dehydration responsive element-binding protein
Sapur.003G032300.1	618.0542001	0.221626441	0.032546183	6.809599	9.79E-12	2.26E-11	cyan	0.44533423	5.09E-09	GO:0005515	AT3G15610.1		Transducin/WD40 repeat-like superfamily protein	Serine/Threonine kinase receptor-associated protein, putative
Sapur.001G124800.1	701.2835963	0.217049232	0.032186591	6.743468	1.55E-11	3.54E-11	cyan	0.476676018	2.77E-10	GO:0006457,GO:0003755,GO:0000413	AT4G17070.1		peptidyl-prolyl cis-trans isomerases	peptidyl-prolyl cis-trans isomerase, cyclophilin-type protein
Sapur.004G131500.1	0.822066005	2.235717045	0.34400673	6.49905	8.08E-11	1.80E-10	cyan	0.699893354	2.02E-24	GO:0006950,GO:0006468,GO:0005524,GO:0004672	AT2G16750.1		Protein kinase protein with adenine nucleotide alpha hydrolases-like domain	tyrosine kinase domain protein
Sapur.017G095100.1	225.1228377	0.212107325	0.040052152	5.295778	1.19E-07	2.32E-07	cyan	0.378870898	9.93E-07	GO:0055085,GO:0016021,GO:0008324,GO:0006812	AT5G38380.1			EamA-like transporter family protein
Sapur.011G164500.1	283.7044223	0.172383423	0.035628594	4.838345	1.31E-06	2.44E-06	cyan	0.357206995	4.38E-06		AT3G16200.1			transmembrane protein, putative
Sapur.009G036200.5	0.971102122	1.292587225	0.274125105	4.715319	2.41E-06	4.44E-06	cyan	0.939808384	3.53E-74	GO:0016021,GO:0015693,GO:0015095	AT2G21120.1		Protein of unknown function (DUF803)	Non-imprinted in prader-willi/angelman syndrome region protein, putative
Sapur.005G015300.1	50.75823113	0.358527462	0.077501592	4.626066	3.73E-06	6.79E-06	cyan	0.78020962	2.13E-33	GO:0003824,GO:0006470,GO:0004722	AT1G09160.2		Protein phosphatase 2C family protein	protein phosphatase 2C
Sapur.014G078700.1	0.510705438	-0.559781837	0.387498403	-1.4446	0.14856908	0.189085841	cyan	0.662951118	3.13E-21		AT2G46550.1			transmembrane protein, putative
Sapur.007G036900.2	0.143130509	0.877129625	0.692689376	1.266267	0.205417545	0.255633815	cyan	0.73612729	4.60E-28		AT5G66870.1	ASL1,LBD36	ASYMMETRIC LEAVES 2-like 1	asymmetric LEAVES protein, putative
Sapur.014G074100.3	42.84290648	-0.061897826	0.090943114	-0.68062	0.496111109	0.570825494	cyan	0.764464233	2.33E-31	GO:0080123,GO:0009864,GO:0009694,GO:0009611	AT2G46370.4	FIN219,JAR1	Auxin-responsive GH3 family protein	indole-3-acetic acid-amido synthetase GH3.5 protein, putative
Sapur.15WG078400.4	0.765860151	-0.166059456	0.268590575	-0.61826	0.536402376	0.611736567	cyan	0.455705923	2.01E-09	GO:0031225,GO:0016049,GO:0010215	AT5G60920.1	COB	COBRA-like extracellular glycosyl-phosphatidyl inositol-anchored protein family	phytochelatin synthetase-like protein
Sapur.004G119100.1	2.940290208	0.088308421	0.169782605	0.520126	0.602975453	0.677084674	cyan	0.762326366	4.29E-31		AT4G39140.5		RING/U-box superfamily protein	mandelonitrile lyase
Sapur.016G113200.2	0.055574416	-0.208929148	1.532437967	-0.13634	0.89155428	0.927185054	cyan	-0.7420042	1.03E-28	GO:0007275	AT1G08465.1	YAB2	Plant-specific transcription factor YABBY family protein	transcription factor YABBY2
Sapur.012G017100.3	0.042153324	0.171999641	1.562571406	0.110075	0.912350101	NA	cyan	0.698825321	2.53E-24					protein phosphatase 2C
Sapur.005G105900.1	0.019898044	0.235512502	2.817250426	0.083597	0.933377186	NA	cyan	0.849892452	5.63E-45		AT5G66870.1	ASL1,LBD36	ASYMMETRIC LEAVES 2-like 1	LOB domain protein
Sapur.014G074100.2	0.003577442	0.128661719	2.904823276	0.044292	0.964671296	NA	cyan	0.739882761	1.78E-28	GO:0080123,GO:0009864,GO:0009694,GO:0009611	AT2G46370.4	FIN219,JAR1	Auxin-responsive GH3 family protein	indole-3-acetic acid-amido synthetase GH3.5 protein, putative
Sapur.005G185700.3	0.004765175	0.05557013	2.904824396	0.01913	0.984737169	NA	cyan	0.720964733	1.81E-26	GO:0005975,GO:0004553	AT1G45130.1	BGAL5	beta-galactosidase 5	beta-galactosidase
Sapur.001G023100.1	675.6384333	4.459948935	0.117202895	38.05323	0	0	cyan	0.77092843	3.55E-32	GO:0046488,GO:0016307	AT3G07960.1		Phosphatidylinositol-4-phosphate 5-kinase family protein	phosphatidylinositol-4-phosphate 5-kinase
Sapur.001G033600.1	259.8826516	5.073315655	0.104597767	48.5031	0	0	cyan	0.850099836	5.10E-45	GO:0055085,GO:0016020,GO:0006811,GO:0005216	AT1G19780.1	ATCNGC8,CNGC8	cyclic nucleotide gated channel 8	cyclic nucleotide-gated ion channel protein, putative
Sapur.001G088700.1	135.7543761	5.619284812	0.108291859	51.89019	0	0	cyan	0.898475391	2.74E-57		AT4G11720.1	GCS1,HAP2	hapless 2	hapless protein
Sapur.001G092000.1	589.4661738	3.009691565	0.077405772	38.882	0	0	cyan	0.84580036	3.83E-44		AT4G22920.1	ATNYE1,NYE1	non-yellowing 1	senescence-inducible stay-green protein
Sapur.001G145200.1	9677.364431	11.35266166	0.14552773	78.0103	0	0	cyan	0.880841514	3.33E-52		AT1G52680.1		late embryogenesis abundant protein-related / LEA protein-related	hypothetical protein
Sapur.001G161000.1	2543.40471	4.011994237	0.090346541	44.40673	0	0	cyan	0.862330807	1.14E-47		AT2G19770.1	PRF5	profilin 5	profilin
Sapur.001G166300.1	222.6463281	7.843560149	0.164054455	47.81071	0	0	cyan	0.849911215	5.58E-45	GO:0005089	AT1G79860.1	ATROPGEF12.MEE64.ROPGI	RHO guanyl-nucleotide exchange factor 12	NA
Sapur.002G000900.1	711.7925024	11.3619094	0.256819189	44.24089	0	0	cyan	0.808491183	1.62E-37	GO:0015031,GO:0008565	AT2G17380.1	AP19	associated protein 19	NA
Sapur.002G065100.1	399.7419938	6.929411329	0.108112675	64.09435	0	0	cyan	0.881580863	2.12E-52		AT1G11040.1		HSP40/DnaJ peptide-binding protein	NA
Sapur.002G088900.1	814.5986363	6.676044015	0.125107636	53.3624	0	0	cyan	0.835341312	4.04E-42		AT1G09890.1		Rhamnogalacturonate lyase family protein	rhamnogalacturonate lyase-like protein
Sapur.002G098700.1	314.8987328	7.708524027	0.144107274	53.49157	0	0	cyan	0.838206803	1.17E-42	GO:0055114,GO:0020037,GO:0016705,GO:0005506	AT4G37340.1	CYP81D3	cytochrome P450, family 81, subfamily D, polypeptide 3	NA
Sapur.002G115800.1	296.4700535	7.943334516	0.151902981	52.29216	0	0	cyan	0.8565039	2.25E-46		AT2G45010.1		PLAC8 family protein	cell number regulator 12
Sapur.002G128300.1	1694.567301	5.590035967	0.097252812	57.47943	0	0	cyan	0.845605951	4.19E-44	GO:0008270	AT1G01780.1		GATA type zinc finger transcription factor family protein	LIM domain protein PLIM-2
Sapur.002G131400.1	3744.569754	9.242293324	0.140374075	65.84046	0	0	cyan	0.836266909	2.71E-42	GO:0055085,GO:0016020,GO:0015297,GO:0015238,GO:0006855	AT4G00350.1		MATE efflux family protein	MATE efflux family protein
Sapur.002G184800.1	519.8877323	6.556647227	0.125887846	52.08324	0	0	cyan	0.840084936	5.09E-43		AT3G07490.1	AGD11	ARF-GAP domain 11	calcium-binding EF-hand protein
Sapur.002G192300.1	743.8323137	5.326533789	0.097452433	54.65778	0	0	cyan	0.840050086	5.17E-43	GO:0005975,GO:0004553	AT3G07320.1		O-Glycosyl hydrolases family 17 protein	glucan endo-1%2C3-beta-glucosidase, putative
Sapur.002G196100.1	8743.209057	5.21391912	0.090246965	57.7739	0	0	cyan	0.873051317	3.30E-50		AT4G13230.1		Late embryogenesis abundant protein (LEA) family protein	hypothetical protein
Sapur.003G027700.1	2311.283411	2.600046658	0.064964043	40.02286	0	0	cyan	0.86890087	3.37E-49		AT2G19770.1	PRF5	profilin 5	profilin
Sapur.003G054800.1	846.5483466	1.593398883	0.041534729	38.36305	0	0	cyan	0.908482625	1.31E-60		AT2G38740.1		Haloacid dehalogenase-like hydrolase (HAD) superfamily protein	haloacid dehalogenase-like hydrolase family protein
Sapur.003G092100.1	650.7035577	4.227874653	0.104121463	40.60522	0	0	cyan	0.795422267	1.56E-35	GO:0005515,GO:0008017,GO:0007018,GO:0005524,GO:0003777,GO:0005871	AT1G63640.1		P-loop nucleoside triphosphate hydrolases superfamily protein with CH (Calponin Homology) domain	NA
Sapur.003G106600.1	614.9448989	5.084662523	0.118344829	42.96481	0	0	cyan	0.783962185	6.58E-34	GO:0005991,GO:0004555	AT4G24040.1	ATTRE1,TRE1	trehalase 1	trehalase
Sapur.003G120000.1	3501.701858	1.929888621	0.048476678	39.81066	0	0	cyan	0.898352742	2.99E-57	GO:0055114,GO:0051537,GO:0016491,GO:0008121,GO:0016679	AT5G13430.1		Ubiquinol-cytochrome C reductase iron-sulfur subunit	cytochrome B-c1 complex subunit Rieske protein
Sapur.003G125800.1	1104.275469	6.997202988	0.153889777	45.46893	0	0	cyan	0.84069635	3.88E-43	GO:0016887,GO:0005524	AT5G60740.1		ABC transporter family protein	white-brown-complex ABC transporter family protein, putative
Sapur.003G133000.1	475.2508529	4.683975197	0.093855529	49.90623	0	0	cyan	0.842979204	1.39E-43	GO:0005515,GO:0006468,GO:0004672	AT1G04700.1		PB1 domain-containing protein tyrosine kinase	tyrosine kinase family protein
Sapur.003G134300.1	1452.017328	7.616074064	0.132941527	57.2889	0	0	cyan	0.827420876	1.11E-40	GO:0030244,GO:0016760,GO:0016020	AT2G33100.1	ATCSLD1,CSLD1	cellulose synthase-like D1	cellulose synthase-like protein D
Sapur.003G138600.1	439.288058	7.119343347	0.126963569	56.07391	0	0	cyan	0.826409277	1.68E-40	GO:0055085,GO:0016020,GO:0006811,GO:0005216	AT1G19780.1	ATCNGC8,CNGC8	cyclic nucleotide gated channel 8	cyclic nucleotide-gated ion channel protein, putative
Sapur.003G154700.1	51.19955266	5.968788565	0.155893861	38.28752	0	0	cyan	0.876204676	5.33E-51	GO:0016020	AT3G54510.2		Early-responsive to dehydration stress protein (ERD4)	early-responsive to dehydration stress protein (ERD4)
Sapur.004G003100.1	201.0531941	7.510144958	0.14889376	50.43962	0	0	cyan	0.867041693	9.32E-49		AT1G62330.1		O-fucosyltransferase family protein	GDP-fucose protein O-fucosyltransferase, plant specific subfamily protein
Sapur.004G024800.1	3254.033113	3.073449928	0.072511517	42.38568	0	0	cyan	0.903964608	4.58E-59	GO:0006355,GO:0003677	AT1G61110.1	anac025,NAC025	NAC domain containing protein 25	NAC transcription factor
Sapur.004G063400.1	1600.252229	9.760437438	0.196000116	49.79812	0	0	cyan	0.845892771	3.67E-44		AT3G03430.1		Calcium-binding EF-hand family protein	calcium-binding protein, putative
Sapur.004G089800.1	284.2341382	7.422819194	0.161208864	46.04473	0	0	cyan	0.786373449	3.05E-34	GO:0007050,GO:0005634,GO:0004861	AT1G49620.1	ICK5,ICN6,KRP7	Cyclin-dependent kinase inhibitor family protein	NA
Sapur.004G110100.1	367.7340421	5.911734528	0.112970051	52.3301	0	0	cyan	0.855126576	4.45E-46		AT3G44150.1			CSL zinc finger protein
Sapur.004G134800.1	565.3916877	9.924813491	0.229830211	43.18324	0	0	cyan	0.741306459	1.24E-28	GO:0055085,GO:0016020	AT1G78610.1	MSL6	mechanosensitive channel of small conductance-like 6	mechanosensitive ion channel domain protein
Sapur.004G156700.1	1452.860864	9.463063564	0.211192949	44.80767	0	0	cyan	0.789540261	1.10E-34	GO:0030244,GO:0016760,GO:0016020	AT4G38190.1	ATCSLD4,CSLD4	cellulose synthase like D4	cellulose synthase D-like protein
Sapur.004G174800.1	354.8878102	3.149007305	0.075945229	41.46419	0	0	cyan	0.865229097	2.47E-48		AT5G01970.1			hypothetical protein
Sapur.005G018500.1	207.8218406	7.063083502	0.177258387	39.84626	0	0	cyan	0.81492908	1.49E-38	GO:0016788	AT1G28650.1		GDSL-like Lipase/Acylhydrolase superfamily protein	NA
Sapur.005G030800.1	343.0360929	5.75266041	0.128435644	44.79022	0	0	cyan	0.771544711	2.96E-32	GO:0016021,GO:0008381	AT2G48060.1			FAM38B protein
Sapur.005G051600.1	146.6137205	6.060977396	0.144299339	42.00281	0	0	cyan	0.797176882	8.62E-36		AT2G04220.1		Plant protein of unknown function (DUF868)	kinesin-4 protein, putative
Sapur.005G059900.1	261.0908945	9.221983699	0.214971565	42.89862	0	0	cyan	0.836150626	2.85E-42		AT5G65160.1		tetratricopeptide repeat (TPR)-containing protein	TPR protein
Sapur.005G097800.1	8579.002689	8.695088907	0.131303284	66.22141	0	0	cyan	0.871283087	8.97E-50		AT4G36600.1		Late embryogenesis abundant (LEA) protein	late embryogenesis abundant protein, putative
Sapur.005G099900.1	708.6460555	7.93900613	0.128130103	61.96051	0	0	cyan	0.859396655	5.21E-47		AT5G66740.1		Protein of unknown function (DUF620)	DUF620 family protein
Sapur.005G113000.1	1254.218345	3.261076235	0.068065544	47.91082	0	0	cyan	0.892195873	2.23E-55		AT1G02070.1			hypothetical protein
Sapur.005G180100.1	314.8241848	4.522686946	0.117050884	38.63864	0	0	cyan	0.778372475	3.76E-33	GO:0006355,GO:0003677	AT4G28530.1	anac074,NAC074	NAC domain containing protein 74	NAC transcription factor 009
Sapur.005G195500.1	195.2724802	3.804982187	0.085110307	44.70648	0	0	cyan	0.851769988	2.29E-45		AT1G20180.1		Protein of unknown function (DUF677)	transmembrane protein, putative
Sapur.006G008400.1	560.6364833	5.071357232	0.089241277	56.82748	0	0	cyan	0.864537974	3.58E-48	GO:0005737,GO:0005094	AT3G07880.1	SCN1	Immunoglobulin E-set superfamily protein	Rho protein GDP dissociation inhibitor
Sapur.006G037000.1	506.2863573	4.991747303	0.08948877	55.78071	0	0	cyan	0.86434625	3.96E-48	GO:0006355,GO:0005634,GO:0003700,GO:0046983,GO:0003677	AT3G57390.1	AGL18	AGAMOUS-like 18	agamous-like MADS-box protein AGL15
Sapur.006G054000.1	456.461394	2.640888966	0.069969614	37.74337	0	0	cyan	0.887335941	5.61E-54	GO:0016020,GO:0006487,GO:0003830	AT2G13290.1		beta-1,4-N-acetylglucosaminyltransferase family protein	beta-1%2C4-N-acetylglucosaminyltransferase family protein
Sapur.006G058700.1	575.454177	5.661451772	0.102789252	55.07825	0	0	cyan	0.863274606	6.98E-48	GO:0005515	AT2G19330.1	PIRL6	plant intracellular ras group-related LRR 6	plant intracellular Ras-group-related LRR protein
Sapur.006G061100.1	337.2215691	5.785793706	0.099939182	57.89315	0	0	cyan	0.877369629	2.68E-51	GO:0005543	AT4G25940.1		ENTH/ANTH/VHS superfamily protein	clathrin assembly plant protein, putative
Sapur.006G126300.1	975.228681	9.52210657	0.170287524	55.91782	0	0	cyan	0.80323838	1.06E-36	GO:0006096,GO:0003872	AT4G26270.1	PFK3	phosphofructokinase 3	6-phosphofructokinase
Sapur.006G157000.1	4842.678576	4.840650383	0.071353111	67.84078	0	0	cyan	0.869860638	1.98E-49		AT3G12110.1	ACT11	actin-11	actin
Sapur.007G002000.1	758.4455479	9.78389825	0.178368042	54.85231	0	0	cyan	0.844931792	5.72E-44	GO:0005515,GO:0006468,GO:0004672	AT1G78980.1	SRF5	STRUBBELIG-receptor family 5	strubbelig-receptor family protein
Sapur.007G068400.1	1051.433559	8.97936529	0.15009988	59.8226	0	0	cyan	0.805036323	5.60E-37	GO:0055085,GO:0016021	AT3G19940.1		Major facilitator superfamily protein	sugar transporter
Sapur.007G080500.1	249.7583517	9.005580645	0.188165255	47.85995	0	0	cyan	0.898330357	3.04E-57	GO:0046983,GO:0003677	AT1G77980.1	AGL66	AGAMOUS-like 66	MADS-box protein
Sapur.007G087300.1	368.2417705	9.834987214	0.224485734	43.81119	0	0	cyan	0.811893372	4.64E-38		AT4G40020.1		Myosin heavy chain-related protein	hypothetical protein
Sapur.007G129100.1	808.9088486	7.649289814	0.142583307	53.64786	0	0	cyan	0.81090789	6.68E-38		AT2G43900.1		Endonuclease/exonuclease/phosphatase family protein	type I inositol 1%2C4%2C5-trisphosphate 5-phosphatase
Sapur.008G011200.1	440.1411662	5.468037451	0.117777282	46.42693	0	0	cyan	0.822364498	8.46E-40		AT5G04830.1		Nuclear transport factor 2 (NTF2) family protein	nuclear transport factor 2 (NTF2) family protein
Sapur.008G023100.1	178.389705	4.478587066	0.105838535	42.31528	0	0	cyan	0.829593921	4.56E-41		AT5G04390.1		C2H2-type zinc finger family protein	C2H2-type zinc-finger protein, putative
Sapur.008G047500.1	455.7445249	3.742513746	0.072165025	51.86049	0	0	cyan	0.881803415	1.85E-52		AT2G39770.2	CYT1.EMB101.GMP1.SOZ1.\	Glucose-1-phosphate adenylyltransferase family protein	NA
Sapur.008G048600.1	383.6879442	6.682130154	0.110878039	60.26559	0	0	cyan	0.865600044	2.03E-48	GO:0005525	AT1G07410.1	ATRAB-A2B.ATRABA2B.RAB	RAB GTPase homolog A2B	Rab-related GTPase family protein
Sapur.008G074900.1	1762.82551	7.379341638	0.094761667	77.87265	0	0	cyan	0.867750492	6.34E-49	GO:0019904	AT1G26480.1	GF14 IOTA,GRF12	general regulatory factor 12	14-3-3 family protein Lil 1433-3 protein
Sapur.008G079600.1	295.9210575	6.86039199	0.176183939	38.9388	0	0	cyan	0.637397871	2.82E-19	GO:0016757	AT2G47180.1	AtGolS1,GolS1	galactinol synthase 1	galactinol synthase
Sapur.008G153800.1	606.9816089	3.352427617	0.085481344	39.21824	0	0	cyan	0.86586881	1.76E-48	GO:0046872,GO:0000166,GO:0016021,GO:0015914,GO:0005524,GO:0004012,GO:000028	AT1G59820.1	ALA3	aminophospholipid ATPase 3	phospholipid-translocating ATPase
Sapur.008G155400.1	1065.620421	10.39878777	0.17905615	58.07557	0	0	cyan	0.863419011	6.47E-48		AT1G59960.1		NAD(P)-linked oxidoreductase superfamily protein	aldo/keto reductase family protein
Sapur.009G024000.1	418.2266912	8.659139434	0.200303019	43.2302	0	0	cyan	0.759389926	9.79E-31		AT2G29040.1		Exostosin family protein	NA
Sapur.009G045700.1	583.1215027	8.006897045	0.144352552	55.46765	0	0	cyan	0.818749444	3.47E-39	GO:0055085,GO:0016021,GO:0015299,GO:0006812	AT3G52080.1	chx28	cation/hydrogen exchanger 28	cation/H(+) antiporter
Sapur.009G090000.1	1753.348599	4.569159102	0.115870121	39.43345	0	0	cyan	0.722484798	1.26E-26	GO:0055085,GO:0022857,GO:0016021	AT3G18830.1	ATPLT5,ATPMT5,PMT5	polyol/monosaccharide transporter 5	polyol transporter
Sapur.009G123400.1	140.5718424	8.434987668	0.223227935	37.78643	0	0	cyan	0.797181032	8.60E-36		AT5G21050.1			hyccin protein
Sapur.010G014100.1	280.0775079	4.800237299	0.127695777	37.5912	0	0	cyan	0.730949237	1.66E-27		AT3G21550.1	AtDMP2,DMP2	DUF679 domain membrane protein 2	plant/MIL23-11 protein
Sapur.010G014200.1	2343.077224	5.149797492	0.10236125	50.31003	0	0	cyan	0.803261945	1.05E-36		AT3G21550.1	AtDMP2,DMP2	DUF679 domain membrane protein 2	plant/MIL23-11 protein
Sapur.010G032600.1	1089.60477	7.604204769	0.135643288	56.06031	0	0	cyan	0.819109676	3.02E-39	GO:0055114,GO:0020037,GO:0006979,GO:0004601	AT1G24110.1		Peroxidase superfamily protein	peroxidase
Sapur.010G052300.1	594.3290044	5.746930493	0.090334323	63.61846	0	0	cyan	0.867843037	6.03E-49		AT1G67540.2			transmembrane protein, putative
Sapur.010G122600.1	939.6192638	6.881404574	0.117309399	58.6603	0	0	cyan	0.787719204	1.98E-34	GO:0019904	AT1G26480.1	GF14 IOTA,GRF12	general regulatory factor 12	14-3-3 family protein Lil 1433-3 protein
Sapur.010G134300.1	1203.039427	11.77892616	0.300596161	39.18522	0	0	cyan	0.55139195	7.23E-14	GO:0055085,GO:0046873,GO:0030001,GO:0016020	AT3G12750.1	ZIP1	zinc transporter 1 precursor	ZIP zinc/iron transport family protein
Sapur.010G136500.1	494.825046	6.070490461	0.139161274	43.62198	0	0	cyan	0.873044497	3.31E-50	GO:0006468,GO:0004672,GO:0005524	AT3G12690.3	AGC1.5	AGC kinase 1.5	NA
Sapur.010G142800.1	511.2597124	9.783511336	0.22130903	44.20747	0	0	cyan	0.767980505	8.44E-32	GO:0055085,GO:0016021,GO:0015299,GO:0006812	AT2G13620.1	ATCHX15,CHX15	cation/hydrogen exchanger 15	cation proton exchanger
Sapur.010G154200.1	326.6513245	7.792888463	0.139341146	55.92669	0	0	cyan	0.865880498	1.75E-48	GO:0005525	AT1G07410.1	ATRAB-A2B.ATRABA2B.RAB	RAB GTPase homolog A2B	Rab-related GTPase family protein
Sapur.011G029800.1	2380.675502	4.725944289	0.083228312	56.78289	0	0	cyan	0.865340299	2.33E-48	GO:0006308,GO:0004519,GO:0003676	AT1G11190.1	BFN1,ENDO1	bifunctional nuclease i	nuclease S1
Sapur.011G042000.1	462.0221718	4.588592366	0.083874534	54.70781	0	0	cyan	0.881560065	2.14E-52	GO:0005525	AT5G60860.1	AtRABA1f,RABA1f	RAB GTPase homolog A1F	Rab-related GTPase family protein
Sapur.011G047000.1	437.6806806	9.702799708	0.192360746	50.44064	0	0	cyan	0.864334737	3.98E-48	GO:0007165	AT5G45820.1	CIPK20,PKS18,SnRK3.6	CBL-interacting protein kinase 20	CBL-interacting Serine/Threonine-kinase
Sapur.011G101300.1	506.2239234	7.131510207	0.116160501	61.39359	0	0	cyan	0.86344995	6.36E-48	GO:0006468,GO:0005524,GO:0004672	AT3G20530.1		Protein kinase superfamily protein	NA
Sapur.012G067700.1	9005.573196	4.886843905	0.115977448	42.13616	0	0	cyan	0.789096175	1.27E-34	GO:0008234,GO:0006508	AT5G50260.1		Cysteine proteinases superfamily protein	KDEL-tailed cysteine endopeptidase CEP3
Sapur.012G080100.1	2206.373907	11.23122989	0.191917139	58.52124	0	0	cyan	0.761825829	4.94E-31		AT5G14040.1	PHT3;1	phosphate transporter 3;1	phosphate carrier protein, putative
Sapur.012G097700.1	346.395742	7.576091941	0.144816805	52.31501	0	0	cyan	0.808914089	1.39E-37	GO:0006468,GO:0004672	AT5G62310.1	IRE	AGC (cAMP-dependent, cGMP-dependent and protein kinase C) kinase family protein	Serine/Threonine kinase domain protein
Sapur.013G017600.1	980.8522087	5.401739556	0.111967894	48.24365	0	0	cyan	0.78985525	9.90E-35		AT3G05500.1		Rubber elongation factor protein (REF)	rubber elongation factor
Sapur.013G039800.1	1565.693443	7.316401002	0.092443456	79.14461	0	0	cyan	0.881621726	2.06E-52	GO:0003676	AT3G04620.1		Alba DNA/RNA-binding protein	alba DNA/RNA-binding protein
Sapur.013G040800.1	1574.512359	11.11248903	0.194490261	57.13648	0	0	cyan	0.798662744	5.19E-36	GO:0016459,GO:0005524,GO:0003774,GO:0005515	AT1G54560.1	ATXIE,XIE	Myosin family protein with Dil domain	myosin XI, putative
Sapur.013G137200.1	413.4096767	10.99014427	0.264506003	41.5497	0	0	cyan	0.801439681	1.99E-36	GO:0055085,GO:0016021,GO:0015299,GO:0006812	AT2G13620.1	ATCHX15,CHX15	cation/hydrogen exchanger 15	cation proton exchanger
Sapur.014G047900.1	358.3871898	5.268967508	0.108657868	48.49136	0	0	cyan	0.828692238	6.61E-41		AT1G02040.1		C2H2-type zinc finger family protein	zinc finger protein
Sapur.014G067600.1	197.521915	7.413918482	0.172649617	42.94199	0	0	cyan	0.779578848	2.60E-33	GO:0005515	AT3G09740.1	ATSYP71,SYP71	syntaxin of plants 71	syntaxin-71 protein
Sapur.014G073800.1	231.2920473	7.209529307	0.154157346	46.76734	0	0	cyan	0.812373285	3.89E-38	GO:0006468,GO:0004672	AT1G64300.1		Protein kinase family protein	NA
Sapur.014G117300.1	385.9131433	9.173976131	0.177579787	51.66115	0	0	cyan	0.871089117	1.00E-49		AT3G19340.1		Protein of unknown function (DUF3754)	aminopeptidase

Sapur.014G132700.1	217.3230613	6.79073443	0.118296226	57.40449	0	0	cyan	0.893448707	9.49E-56		AT5G47540.1		Mo25 family protein	NA
Sapur.016G032700.1	1095.402063	9.712163543	0.232753687	41.72722	0	0	cyan	0.789952146	9.59E-35	GO:0046488,GO:0016307	AT3G56960.1	PIP5K4	phosphatidyl inositol monophosphate 5 kinase 4	phosphatidylinositol-4-phosphate 5-kinase
Sapur.016G041100.1	4244.428553	4.119273974	0.078446962	52.51031	0	0	cyan	0.855466164	3.76E-46		AT3G12110.1	ACT11	actin-11	actin
Sapur.016G061100.1	399.773243	4.051929866	0.08475564	47.8072	0	0	cyan	0.858000757	1.06E-46	GO:0006468,GO:0004672	AT2G38910.1	CPK20	calcium-dependent protein kinase 20	calcium/calmodulin-dependent kinase
Sapur.016G065300.1	208.3093267	5.711657043	0.109533264	52.14541	0	0	cyan	0.86366559	5.68E-48	GO:0006887,GO:0000145	AT5G52340.1	ATEXO70A2,EXO70A2	exocyst subunit exo70 family protein A2	exocyst subunit EXO70
Sapur.016G094800.1	860.6501828	6.825746453	0.109392942	62.39659	0	0	cyan	0.829154476	5.46E-41	GO:0003924,GO:0007017,GO:0005874	AT5G23860.1	TUB8	tubulin beta 8	beta-tubulin
Sapur.016G151800.1	2254.853443	3.888788566	0.077902194	49.91886	0	0	cyan	0.868087022	5.27E-49	GO:0008168,GO:0008152,GO:0006694	AT5G13710.1	CPH,SMT1	sterol methyltransferase 1	S-adenosyl-L-methionine:delta24-sterol-C-methyltransferase, putative
Sapur.016G166900.1	386.4238155	10.71167543	0.276096512	38.79685	0	0	cyan	0.76105615	6.14E-31	GO:0055085,GO:0016021,GO:0015299,GO:0006812	AT5G37060.1	ATCHX24,CHX24	cation/H+ exchanger 24	cation/H(+) antiporter
Sapur.016G194200.1	221.3840957	6.348619466	0.108550222	58.48555	0	0	cyan	0.892756285	1.53E-55	GO:0016021,GO:0015693,GO:0015095	AT4G13800.1		Protein of unknown function (DUF803)	NA
Sapur.016G245000.1	126.0705611	5.06444663	0.107742265	47.0052	0	0	cyan	0.879362832	8.16E-52	GO:0016020	AT1G79450.1	ALIS5	ALA-interacting subunit 5	cell division control protein
Sapur.016G251900.1	1299.227115	9.451802888	0.24506645	38.56833	0	0	cyan	0.73254651	1.12E-27		AT1G29140.1		Pollen Ole e 1 allergen and extensin family protein	anther-specific protein LAT52, putative
Sapur.016G262000.1	1174.901135	5.904885882	0.099849967	59.13758	0	0	cyan	0.846002342	3.49E-44	GO:0006355,GO:0003677	AT3G15510.1	ANAC056.ATNAC2.NAC2.NA	NAC domain containing protein 2	NAC transcription factor
Sapur.016G306800.1	1923.019634	9.598236454	0.177761226	53.99511	0	0	cyan	0.832653382	1.27E-41	GO:0006508,GO:0004252	AT5G59810.1	ATSBT5.4,SBT5.4	Subtilase family protein	subtilisin-like serine protease
Sapur.017G046200.1	1386.369378	5.809770979	0.119468361	48.63021	0	0	cyan	0.77925249	2.87E-33	GO:0016157,GO:0005985	AT1G04920.1	ATSPS3F,SPS3F	sucrose phosphate synthase 3F	sucrose-phosphate synthase
Sapur.017G052100.1	99.47601331	4.540289175	0.112554197	40.33869	0	0	cyan	0.832989022	1.10E-41		AT5G55490.1	ATGEX1,GEX1	gamete expressed protein 1	NA
Sapur.017G056200.1	285.1521761	4.261857557	0.088248176	48.294	0	0	cyan	0.85475544	5.35E-46	GO:0055085,GO:0016020,GO:0006811,GO:0005216	AT3G48010.1	ATCNGC16,CNGC16	cyclic nucleotide-gated channel 16	cyclic nucleotide-gated ion channel protein, putative
Sapur.018G021800.1	696.4966725	6.984320151	0.139611975	50.02666	0	0	cyan	0.84130576	2.96E-43	GO:0016597,GO:0008152	AT5G25320.1		ACT-like superfamily protein	ACT domain protein
Sapur.018G071400.1	527.7759454	9.814666763	0.202032371	48.57967	0	0	cyan	0.817329321	5.99E-39	GO:0016758,GO:0008152	AT5G17050.1	UGT78D2	UDP-glucosyl transferase 78D2	UDP-glucose:flavonoid 3-O-glucosyltransferase
Sapur.018G075600.1	139.2403508	6.290203357	0.155048545	40.56925	0	0	cyan	0.846034121	3.44E-44	GO:0003333	AT3G28960.1		Transmembrane amino acid transporter family protein	amino acid transporter
Sapur.018G089200.1	180.3765476	6.012473954	0.131002286	45.89595	0	0	cyan	0.812625364	3.54E-38		AT5G35090.1			hypothetical protein
Sapur.018G090800.1	151.1419635	7.441685063	0.182735178	40.72388	0	0	cyan	0.792177333	4.61E-35	GO:0005509	AT4G32060.1		calcium-binding EF hand family protein	NA
Sapur.019G017600.1	161.7227131	4.900881921	0.127856649	38.33107	0	0	cyan	0.834838914	5.01E-42	GO:0006468,GO:0004672	AT1G07650.2		Leucine-rich repeat transmembrane protein kinase	NA
Sapur.019G080900.1	527.9999101	4.783162589	0.120861848	39.57545	0	0	cyan	0.820481089	1.77E-39	GO:0006468,GO:0004672	AT4G09570.1	ATCPK4,CPK4	calcium-dependent protein kinase 4	calcium-dependent kinase
Sapur.019G097900.1	657.018266	9.596537637	0.172859353	55.51645	0	0	cyan	0.861024933	2.26E-47	GO:0030132,GO:0030130,GO:0016192,GO:0006886,GO:0005198	AT2G20760.1		Clathrin light chain protein	clathrin light chain
Sapur.019G108200.1	386.9133093	11.11734007	0.262973131	42.27557	0	0	cyan	0.823553307	5.28E-40	GO:0055085,GO:0016021,GO:0015299,GO:0006812,GO:0006814,GO:0006813,GO:000545	AT2G13620.1	ATCHX15,CHX15	cation/hydrogen exchanger 15	cation proton exchanger
Sapur.15WG105300.1	4494.645327	4.763490038	0.095752328	49.74803	0	0	cyan	0.848167342	1.27E-44	GO:0008234,GO:0006508	AT5G50260.1		Cysteine proteinases superfamily protein	KDEL-tailed cysteine endopeptidase CEP2
Sapur.15WG126200.1	682.3149363	6.839238835	0.113345588	60.3397	0	0	cyan	0.850256905	4.74E-45		AT5G51030.1		NAD(P)-binding Rossmann-fold superfamily protein	short-chain dehydrogenase/reductase family protein
Sapur.15WG150200.1	1169.123181	9.097099238	0.167111013	54.43746	0	0	cyan	0.841260942	3.02E-43	GO:0005622,GO:0003779,GO:0030042,GO:0015629	AT4G25590.1	ADF7	actin depolymerizing factor 7	actin depolymerizing factor
Sapur.15WG150700.1	64.26981606	5.018351636	0.126183017	39.77042	0	0	cyan	0.840501473	4.23E-43	GO:0055114,GO:0020037,GO:0016705,GO:0005506	AT5G52400.1	CYP715A1	cytochrome P450, family 715, subfamily A, polypeptide 1	cytochrome P450 family protein
Sapur.15ZG034700.1	789.4567782	2.586413086	0.060473449	42.7694	0	0	cyan	0.89305627	1.24E-55		AT1G18210.2		Calcium-binding EF-hand family protein	calcium-binding protein CML27, putative
Sapur.15ZG047600.1	838.5699077	7.078053872	0.12184398	58.09113	0	0	cyan	0.826565181	1.58E-40	GO:0008017,GO:0007018,GO:0005524,GO:0003777,GO:0005871	AT1G63640.2		P-loop nucleoside triphosphate hydrolases superfamily protein with CH (Calponin Homology) domain	NA
Sapur.T030600.1	110.411367	5.68763764	0.136887137	41.54983	0	0	cyan	0.826930976	1.36E-40					NA
Sapur.T088600.1	82.32296784	6.322029726	0.159919666	39.53253	0	0	cyan	0.828413719	7.41E-41					NA
Sapur.T106600.1	113.3107151	5.76489533	0.136618988	42.19688	0	0	cyan	0.831644546	1.94E-41					NA
Sapur.T110800.1	118.5744709	6.884293691	0.170032556	40.48809	0	0	cyan	0.777720191	4.60E-33					NA
Sapur.017G112600.1	68.97703964	4.564457521	0.122025553	37.40575	3.15E-306	1.82E-304	cyan	0.822957069	6.69E-40		AT5G55490.1	ATGEX1,GEX1	gamete expressed protein 1	NA
Sapur.002G027300.1	166.6692771	9.861915672	0.263893374	37.37083	1.16E-305	6.69E-304	cyan	0.828673123	6.66E-41		AT4G28556.1	RIC7	PAK-box/P21-Rho-binding family protein	P21-Rho-binding domain protein
Sapur.008G108800.1	522.9678014	3.960866229	0.106219266	37.28953	2.43E-304	1.39E-302	cyan	0.781681605	1.35E-33		AT1G70670.1		Caleosin-related family protein	calcium ion-binding protein, putative
Sapur.004G016700.1	110.5895459	4.889885063	0.13138268	37.21864	3.41E-303	1.94E-301	cyan	0.816822211	7.28E-39					NA
Sapur.15WG076000.1	65.04384205	4.637391063	0.125405985	36.97903	2.49E-299	1.39E-297	cyan	0.829995431	3.86E-41					NA
Sapur.010G019700.1	48.80028971	5.89438472	0.159822759	36.88076	9.40E-298	5.20E-296	cyan	0.87266566	4.10E-50	GO:0008168,GO:0008152	AT1G24480.1		S-adenosyl-L-methionine-dependent methyltransferases superfamily protein	NA
Sapur.010G169700.1	2538.072545	3.664472356	0.099610147	36.78814	2.86E-296	1.57E-294	cyan	0.786352711	3.07E-34	GO:0016887,GO:0005524,GO:0016020	AT3G55090.1		ABC-2 type transporter family protein	white-brown-complex ABC transporter family protein
Sapur.001G120600.1	122.7164692	7.387755699	0.200824139	36.78719	2.96E-296	1.62E-294	cyan	0.771375498	3.11E-32	GO:0055114,GO:0020037,GO:0006979,GO:0004601	AT2G35380.1		Peroxidase superfamily protein	peroxidase
Sapur.006G212200.1	295.4856639	8.54342384	0.232269471	36.78238	3.53E-296	1.93E-294	cyan	0.649666755	3.43E-20	GO:0016597,GO:0008152	AT5G25320.1		ACT-like superfamily protein	ACT domain protein
Sapur.016G307100.1	149.5794686	3.555950639	0.097413834	36.50355	9.74E-292	5.26E-290	cyan	0.744320835	5.68E-29		AT3G13275.1			transmembrane protein, putative
Sapur.014G119800.1	309.2229852	4.027918832	0.110404682	36.48322	2.05E-291	1.10E-289	cyan	0.749802516	1.34E-29		AT1G10800.1			transmembrane protein, putative
Sapur.011G068700.1	164.5746413	5.540050194	0.152700634	36.28047	3.29E-288	1.74E-286	cyan	0.731262612	1.53E-27	GO:0016758,GO:0008152	AT5G54010.1		UDP-Glycosyltransferase superfamily protein	NA
Sapur.15WG105400.1	191.2524871	4.943434702	0.136364433	36.25164	9.36E-288	4.94E-286	cyan	0.759576898	9.30E-31	GO:0008234,GO:0006508	AT5G50260.1		Cysteine proteinases superfamily protein	KDEL-tailed cysteine endopeptidase CEP2
Sapur.005G163300.1	930.0719407	-2.489023301	0.068969947	-36.0885	3.43E-285	1.77E-283	cyan	-0.837402834	1.66E-42		AT1G06690.1		NAD(P)-linked oxidoreductase superfamily protein	aldo/keto reductase family protein
Sapur.008G074100.1	358.6646291	4.076987503	0.113014603	36.07487	5.62E-285	2.89E-283	cyan	0.818153311	4.37E-39	GO:0005975,GO:0004553	AT1G26560.1	BGLU40	beta glucosidase 40	NA
Sapur.15ZG044100.1	354.1911294	4.883942679	0.135689447	35.99353	1.06E-283	5.40E-282	cyan	0.838185296	1.18E-42	GO:0008017,GO:0007018,GO:0005524,GO:0003777,GO:0005871	AT1G63640.1		P-loop nucleoside triphosphate hydrolases superfamily protein with CH (Calponin Homology) domain	NA
Sapur.009G036000.1	365.9320368	4.14903431	0.116729259	35.54408	1.03E-276	5.00E-275	cyan	0.789912952	9.71E-35					transmembrane protein, putative
Sapur.15ZG043900.1	362.1908767	2.821827043	0.079459983	35.51256	3.15E-276	1.53E-274	cyan	0.849698374	6.18E-45	GO:0008270,GO:0005515	AT5G05810.1	ATL43	RING/U-box superfamily protein	RING-H2 zinc finger protein, putative
Sapur.018G026000.1	471.1432379	3.137105162	0.088573521	35.41809	8.99E-275	4.36E-273	cyan	0.831609779	1.97E-41	GO:0055085,GO:0016020,GO:0006811,GO:0005216	AT2G25600.1	AKT6,SPIK	Shaker pollen inward K+ channel	NA
Sapur.001G181200.1	88.32962415	3.189868644	0.090095889	35.40526	1.42E-274	6.84E-273	cyan	0.818721028	3.51E-39		AT3G02430.1		Protein of unknown function (DUF679)	transmembrane protein, putative
Sapur.T000800.1	121.3404162	5.189747885	0.147278467	35.23766	5.30E-272	2.53E-270	cyan	0.834591944	5.57E-42	GO:0006468,GO:0004672	AT1G07650.2		Leucine-rich repeat transmembrane protein kinase	NA
Sapur.002G109900.1	978.0862247	1.593347031	0.04538803	35.10501	5.65E-270	2.66E-268	cyan	0.897637386	5.01E-57	GO:0005525	AT2G44610.1	ATRAB6A.ATRABH1B.RAB6.	Ras-related small GTP-binding family protein	Ras-related protein Rab-6
Sapur.008G054100.1	451.8979902	2.556071077	0.07325307	34.89371	9.26E-267	4.27E-265	cyan	0.850017357	5.31E-45	GO:0005515,GO:0035556,GO:0007165,GO:0006629,GO:0004435	AT3G08510.2	ATPLC2,PLC2	phospholipase C 2	phosphoinositide-specific phospholipase C
Sapur.15WG130700.1	90.18065863	3.432327277	0.098429152	34.87104	2.04E-266	9.37E-265	cyan	0.831763142	1.85E-41		AT3G05320.1		O-fucosyltransferase family protein	GDP-fucose protein O-fucosyltransferase
Sapur.014G050800.1	514.2658006	2.527232324	0.072914833	34.66006	3.15E-263	1.41E-261	cyan	0.855014755	4.71E-46	GO:0016192,GO:0006904	AT1G02010.1	SEC1A	secretory 1A	NA
Sapur.003G028700.1	574.4211678	4.575024827	0.132494734	34.52986	2.86E-261	1.26E-259	cyan	0.794723838	1.97E-35		AT4G32350.1		Regulator of Vps4 activity in the MVB pathway protein	regulator of Vps4 activity in the MVB pathway protein
Sapur.006G155300.1	49.07623264	5.156987389	0.15021728	34.33019	2.78E-258	1.20E-256	cyan	0.788854184	1.37E-34	GO:0005515,GO:0006468,GO:0005524,GO:0004672	AT5G06820.1	SRF2	STRUBBELIG-receptor family 2	strubbelig-receptor family protein
Sapur.T031200.1	42.59505097	5.318515015	0.155566949	34.18795	3.65E-256	1.56E-254	cyan	0.822221585	8.95E-40	GO:0006468,GO:0005524,GO:0004672	AT5G18610.1		Protein kinase superfamily protein	NA
Sapur.15WG106100.1	115.8496158	2.824328969	0.082642582	34.17523	5.64E-256	2.40E-254	cyan	0.860751326	2.60E-47	GO:0008168,GO:0008152	AT1G24480.1		S-adenosyl-L-methionine-dependent methyltransferases superfamily protein	methyltransferase domain protein
Sapur.013G053900.1	1700.450567	2.736509612	0.080143565	34.14509	1.58E-255	6.70E-254	cyan	0.813999437	2.12E-38		AT3G03900.1	APK3	adenosine-5\'-phosphosulfate (APS) kinase 3	adenylylsulfate kinase
Sapur.011G117300.1	37.80152737	8.471376277	0.248517122	34.0877	1.12E-254	4.72E-253	cyan	0.880860228	3.29E-52	GO:0006468,GO:0004672	AT4G20790.1		Leucine-rich repeat protein kinase family protein	tyrosine kinase domain protein
Sapur.005G074200.1	1587.712818	3.523651988	0.103645834	33.99704	2.46E-253	1.03E-251	cyan	0.777686495	4.65E-33	GO:0055085	AT1G65730.1	YSL7	YELLOW STRIPE like 7	OPT superfamily oligopeptide transporter
Sapur.010G102400.1	4661.193327	4.243978215	0.125444779	33.83145	6.80E-251	2.80E-249	cyan	0.790639468	7.65E-35	GO:0015977,GO:0008964,GO:0006099	AT1G68750.1	ATPPC4,PPC4	phosphoenolpyruvate carboxylase 4	phosphoenolpyruvate carboxylase
Sapur.15ZG044000.1	107.6750368	4.90189478	0.145396121	33.71407	3.60E-249	1.47E-247	cyan	0.85150597	2.60E-45					NA
Sapur.003G069600.1	1913.879267	4.09747104	0.121666923	33.67777	1.22E-248	4.97E-247	cyan	0.771902852	2.66E-32	GO:0055114,GO:0009916	AT1G32350.1	AOX1D	alternative oxidase 1D	ubiquinol oxidase
Sapur.004G016600.1	163.5210083	8.976978205	0.266672838	33.66289	2.02E-248	8.19E-247	cyan	0.683386663	6.15E-23	GO:0008270,GO:0005622	AT4G27310.1		B-box type zinc finger family protein	B-box zinc finger protein
Sapur.001G081700.1	5078.82426	4.604013363	0.136855097	33.64152	4.15E-248	1.68E-246	cyan	0.704290915	7.80E-25		AT4G23500.1		Pectin lyase-like superfamily protein	glycoside hydrolase family 28 protein
Sapur.001G032600.1	1247.581824	3.418908628	0.102199663	33.45323	2.31E-245	9.22E-244	cyan	0.802574861	1.34E-36					transmembrane protein, putative
Sapur.001G017900.1	424.0230122	3.334695151	0.099733983	33.4359	4.13E-245	1.64E-243	cyan	0.758804874	1.15E-30	GO:0055114,GO:0050660,GO:0016491,GO:0019139,GO:0009690	AT3G63440.1	ATCKX6,ATCKX7,CKX6	cytokinin oxidase/dehydrogenase 6	cytokinin oxidase/dehydrogenase
Sapur.005G199800.1	400.1995759	4.175064012	0.125056766	33.38535	2.24E-244	8.88E-243	cyan	0.727101601	4.21E-27		AT1G76240.1		Arabidopsis protein of unknown function (DUF241)	DUF241 domain protein
Sapur.016G001200.1	224.0492171	3.874633023	0.116475362	33.26569	1.21E-242	4.76E-241	cyan	0.853282801	1.10E-45					hypothetical protein
Sapur.016G070400.1	100.0563217	5.551410277	0.16696411	33.24912	2.10E-242	8.25E-241	cyan	0.816612947	7.88E-39	GO:0006355,GO:0003677	AT5G22380.1	anac090,NAC090	NAC domain containing protein 90	NAC transcription factor
Sapur.010G168600.1	915.1310401	5.393211679	0.162583069	33.17204	2.73E-241	1.06E-239	cyan	0.879786791	6.32E-52		AT2G39420.1		alpha/beta-Hydrolases superfamily protein	alpha/beta hydrolase family protein
Sapur.016G111800.1	509.2536535	2.237790858	0.067521146	33.14207	7.37E-241	2.86E-239	cyan	0.851495303	2.62E-45	GO:0016020	AT2G38480.1		Uncharacterised protein family (UPF0497)	CASP-like protein
Sapur.15WG116200.1	545.7740211	6.347144429	0.192635335	32.94901	4.37E-238	1.67E-236	cyan	0.786470359	2.96E-34	GO:0016790,GO:0006633	AT1G08510.1	FATB	fatty acyl-ACP thioesterases B	acyl acyl-carrier thioesterase, putative
Sapur.016G305200.1	395.4671373	2.220025981	0.06739071	32.94261	5.40E-238	2.06E-236	cyan	0.88251065	1.19E-52	GO:0005515,GO:0006468,GO:0005524,GO:0004672	AT5G56040.2		Leucine-rich receptor-like protein kinase family protein	leucine-rich receptor-like kinase
Sapur.019G108300.1	72.39143033	9.370520127	0.284458504	32.94161	5.58E-238	2.13E-236	cyan	0.744010089	6.16E-29	GO:0055085,GO:0016021,GO:0015299,GO:0006812	AT1G06970.1	ATCHX14,CHX14	cation/hydrogen exchanger 14	cation proton exchanger
Sapur.001G192700.1	138.9988646	2.829427085	0.085987185	32.90522	1.85E-237	7.06E-236	cyan	0.843952708	8.95E-44		AT5G45330.1	DCP5-L	decapping 5-like	Scd6-like-Sm domain protein
Sapur.006G101400.1	14888.29871	2.334629125	0.071087248	32.84174	1.49E-236	5.67E-235	cyan	0.832273799	1.49E-41	GO:0006556,GO:0004478,GO:0005524	AT2G36880.2	MAT3	methionine adenosyltransferase 3	S-adenosylmethionine synthase
Sapur.15WG127700.1	2166.118825	2.296344162	0.069940899	32.83264	2.02E-236	7.64E-235	cyan	0.838215194	1.16E-42	GO:0019538,GO:0016887,GO:0005524	AT5G51070.1	CLPD,ERD1,SAG15	Clp ATPase	ATP-dependent Clp protease ATP-binding subunit
Sapur.017G050000.1	4744.206423	3.114349531	0.09501206	32.77847	1.19E-235	4.50E-234	cyan	0.813954239	2.15E-38	GO:0016740	AT4G13930.1	SHM4	serine hydroxymethyltransferase 4	serine hydroxymethyltransferase
Sapur.008G145500.1	1108.817365	2.082542683	0.063572746	32.75842	2.30E-235	8.67E-234	cyan	0.85462135	5.71E-46		AT3G26000.1		Ribonuclease inhibitor	F-box protein SKIP14
Sapur.006G047900.1	40.25345726	5.973050863	0.18250783	32.72764	6.32E-235	2.37E-233	cyan	0.838762109	9.13E-43		AT5G11090.1		serine-rich protein-related	hypothetical protein
Sapur.006G092400.1	546.3254275	2.88603044	0.08824526	32.70465	1.34E-234	5.03E-233	cyan	0.864057085	4.62E-48	GO:0016787	AT2G29400.1	PP1-AT,TOPP1	type one protein phosphatase 1	serine/threonine-protein phosphatase
Sapur.013G080900.1	301.5398709	3.673425934	0.112389563	32.68476	2.57E-234	9.62E-233	cyan	0.84416012	8.14E-44	GO:0016788	AT3G03530.1	NPC4	non-specific phospholipase C4	Non-specific phospholipase C3
Sapur.001G008400.1	103.1328468	3.790273906	0.116202917	32.61772	2.30E-233	8.54E-232	cyan	0.775962628	7.86E-33	GO:0055085,GO:0016021,GO:0008324,GO:0006812	AT3G58060.1		Cation efflux family protein	metal tolerance protein
Sapur.001G152100.1	385.5064473	2.183144627	0.067303841	32.43715	8.22E-231	2.99E-229	cyan	0.837876401	1.35E-42		AT1G79900.1	ATMBAC2,BAC2	Mitochondrial substrate carrier family protein	carnitine/acylcarnitine carrier protein, putative
Sapur.004G163600.1	324.7234887	3.348853786	0.103658542	32.30659	5.65E-229	2.03E-227	cyan	0.768227935	7.85E-32		AT2G28200.1		C2H2-type zinc finger family protein	C2H2-type zinc-finger protein
Sapur.001G143900.1	334.1842535	3.163415128	0.097929475	32.30299	6.35E-229	2.28E-227	cyan	0.775629317	8.70E-33		AT5G25460.1		Protein of unknown function, DUF642	plant/F18G18-200 protein
Sapur.011G110300.1	151.3450368	3.378184666	0.104995199	32.17466	3.99E-227	1.42E-225	cyan	0.790811677	7.23E-35	GO:0016021,GO:0016020,GO:0022857	AT3G30340.1		nodulin MtN21 /EamA-like transporter family protein	auxin-induced protein 5NG4, putative
Sapur.009G088600.1	180.2477121	3.262936629	0.101634627	32.10458	3.81E-226	1.34E-224	cyan	0.78386759	6.78E-34		AT4G34320.1		Protein of unknown function (DUF677)	transmembrane protein, putative
Sapur.007G004000.1	726.3049826	1.998584161	0.062355329	32.05154	2.09E-225	7.32E-224	cyan	0.880849743	3.31E-52	GO:0016021	AT4G37680.2	HHP4	heptahelical protein 4	adiponectin receptor protein
Sapur.001G008700.1	282.226888	3.430844817	0.107097911	32.03466	3.59E-225	1.26E-223	cyan	0.736091174	4.64E-28		AT2G31945.1			transmembrane protein, putative
Sapur.016G069500.1	38.43640964	6.282220805	0.196721653	31.93457	8.85E-224	3.05E-222	cyan	0.847622245	1.64E-44		AT3G11570.1	TBL8	TRICHOME BIREFRINGENCE-LIKE 8	Pmr5/Cas1p GDSL/SGNH-like acyl-esterase family protein
Sapur.010G156600.1	234.9202074	3.417039235	0.107054724	31.91862	1.47E-223	5.07E-222	cyan	0.764376806	2.39E-31	GO:0003824,GO:0006470,GO:0004722	AT1G07430.1	HAI2	highly ABA-induced PP2C gene 2	protein phosphatase 2C
Sapur.003G045900.1	58.61915721	5.851127887	0.183508522	31.88477	4.34E-223	1.49E-221	cyan	0.765870797	1.56E-31	GO:0031012,GO:0008270,GO:0006508,GO:0004222	AT4G16640.1		Matrixin family protein	matrix metalloproteinase
Sapur.017G050300.1	565.9467229	1.101272339	0.03482198	31.62578	1.63E-219	5.44E-218	cyan	0.914305123	1.01E-62	GO:0043565,GO:0006355,GO:0005634,GO:0003700	AT4G13980.1	AT-HSFA5,HSFA5	winged-helix DNA-binding transcription factor family protein	heat stress transcription factor A-5
Sapur.15WG075600.1	15.22590015	-7.267965485	0.229899504	-31.6137	2.40E-219	7.97E-218	cyan	-0.820651396	1.66E-39		AT4G13070.1		RNA-binding CRS1 / YhbY (CRM) domain protein	NA
Sapur.018G009900.1	2119.610933	1.542963876	0.048941216	31.52688	3.72E-218	1.23E-216	cyan	0.882718078	1.05E-52	GO:0006470,GO:0004722	AT4G31750.1	WIN2	HOPW1-1-interacting 2	protein phosphatase 2C
Sapur.012G099400.1	884.0807373	1.703100052	0.054032103	31.52015	4.60E-218	1.52E-216	cyan	0.864109407	4.49E-48		AT4G25360.1	TBL18	TRICHOME BIREFRINGENCE-LIKE 18	leaf-senescence-like protein
Sapur.009G131800.1	2197.869421	2.20867319	0.070091143	31.51144	6.05E-218	1.99E-216	cyan	0.837811294	1.39E-42	GO:0055085,GO:0016021	AT4G35300.1	TMT2	tonoplast monosaccharide transporter2	hexose transporter, putative
Sapur.004G154200.1	44.14482738	8.170562106	0.259842442	31.44429	5.02E-217	1.64E-215	cyan	0.823278547	5.89E-40		AT4G35280.1		C2H2-like zinc finger protein	zinc finger protein
Sapur.010G089300.1	83.25710331	6.058807301	0.193476113	31.31553	2.87E-215	9.31E-214	cyan	0.720864404	1.85E-26	GO:0055085,GO:0016020,GO:0015297,GO:0015238,GO:0006855	AT1G15160.1		MATE efflux family protein	MATE efflux family protein
Sapur.006G205500.1	435.7936905	3.814236777	0.121898148	31.29036	6.31E-215	2.04E-213	cyan	0.730227713	1.97E-27		AT2G25625.2			hypothetical protein
Sapur.010G186300.1	124.2535325	3.277482127	0.105061189	31.19594	1.21E-213	3.87E-212	cyan	0.828847343	6.20E-41	GO:0016021,GO:0015012,GO:0006024	AT5G04500.1		glycosyltransferase family protein 47	glycosyltransferase family protein
Sapur.006G086700.1	90.70516854	3.830446807	0.122970533	31.14931	5.18E-213	1.65E-211	cyan	0.707668942	3.72E-25		AT3G51680.1		NAD(P)-binding Rossmann-fold superfamily protein	short-chain dehydrogenase reductase
Sapur.002G087200.1	2131.650458	1.900820429	0.061078635	31.12087	1.26E-212	4.00E-211	cyan	0.841124234	3.21E-43		AT1G58280.1		Phosphoglycerate mutase family protein	phosphoglycerate mutase-like protein
Sapur.010G081100.1	2916.375079	1.371123207	0.044535209	30.7874	3.86E-208	1.21E-206	cyan	0.870642526	1.28E-49	GO:0055114,GO:0016491	AT1G03400.1		2-oxoglutarate (2OG) and Fe(II)-dependent oxygenase superfamily protein	NA
Sapur.002G181800.1	52.97545571	8.660480558	0.281646202	30.7495	1.24E-207	3.86E-206	cyan	0.719054587	2.82E-26		AT1G53708.1	RTFL9	ROTUNDIFOLIA like 9	NA
Sapur.009G115200.1	2037.146519	2.662257916	0.086819473	30.66429	1.70E-206	5.25E-205	cyan	0.800146485	3.11E-36	GO:0050662,GO:0003824,GO:0080019	AT4G33790.1	CER4,FAR3,G7	Jojoba acyl CoA reductase-related male sterility protein	fatty acyl-CoA reductase
Sapur.001G127100.1	2902.142441	1.148640392	0.037690451	30.47563	5.48E-204	1.66E-202	cyan	0.898916279	1.99E-57	GO:0005525	AT1G02130.1	ARA-5.ARA5.ATRAB1B.ATRA	RAS 5	small GTPase family Rab protein
Sapur.15WG153200.1	214.4068837	4.476219382	0.147834084	30.27867	2.19E-201	6.52E-200	cyan	0.631344829	7.71E-19		AT5G36970.1	NHL25	NDR1/HIN1-like 25	NA
Sapur.013G000200.1	305.7138561	1.545415586	0.051097653	30.24436	6.19E-201	1.84E-199	cyan	0.87132137	8.78E-50		AT5G26720.1			hypothetical protein
Sapur.007G001200.1	38.07816998	3.448624513	0.114079905	30.2299	9.59E-201	2.84E-199	cyan	0.846567742	2.69E-44		AT1G78922.1			transmembrane protein, putative
Sapur.008G000500.1	37.77837175	6.587670679	0.218649385	30.12892	2.03E-199	5.96E-198	cyan	0.786960646	2.53E-34	GO:0005515	AT5G08560.2		transducin family protein / WD-40 repeat family protein	transducin/WD40 domain protein
Sapur.005G139600.1	577.2195742	1.489615313	0.049542113	30.06766	1.28E-198	3.76E-197	cyan	0.878277901	1.56E-51	GO:0016021	AT1G21790.1		TRAM, LAG1 and CLN8 (TLC) lipid-sensing domain containing protein	TLC domain protein
Sapur.008G043200.1	3225.856082	1.777699823	0.059327979	29.96394	2.90E-197	8.40E-196	cyan	0.850658314	3.91E-45		AT3G12110.1	ACT11	actin-11	actin
Sapur.011G044300.1	910.5198605	3.244027472	0.108296759	29.95498	3.79E-197	1.10E-195	cyan	0.825717724	2.22E-40	GO:0006629	AT4G18550.1		alpha/beta-Hydrolases superfamily protein	phospholipase A1
Sapur.012G032600.1	2427.004529	2.905784215	0.097016755	29.95136	4.22E-197	1.22E-195	cyan	0.816491431	8.25E-39	GO:0006468,GO:0005524,GO:0004672	AT3G18040.1	MPK9	MAP kinase 9	MAP kinase
Sapur.010G097600.1	148.7513516	2.906976733	0.09714231	29.92493	9.33E-197	2.69E-195	cyan	0.800109586	3.15E-36		AT1G13340.1		Regulator of Vps4 activity in the MVB pathway protein	regulator of Vps4 activity in the MVB pathway protein
Sapur.002G098300.1	209.9752199	4.211588897	0.141014104	29.86644	5.37E-196	1.55E-194	cyan	0.798651676	5.21E-36	GO:0055114,GO:0020037,GO:0016705,GO:0005506	AT4G37370.1	CYP81D8	cytochrome P450, family 81, subfamily D, polypeptide 8	cytochrome P450 family protein
Sapur.001G144700.1	28.19328792	7.278301803	0.245000332	29.70731	6.18E-194	1.75E-192	cyan	0.904168611	3.92E-59		AT3G22760.1	SOL1	Tesmin/TSO1-like CXC domain-containing protein	TSO1, putative
Sapur.017G067800.1	106.4616113	2.403336201	0.080988452	29.67505	1.61E-193	4.55E-192	cyan	0.805474151	4.79E-37	GO:0045454,GO:0015035,GO:0009055	AT5G39865.1		Glutaredoxin family protein	NA
Sapur.15ZG047500.1	41.57941954	7.855993019	0.265289742	29.61288	1.02E-192	2.88E-191	cyan	0.798296491	5.88E-36					NA
Sapur.013G084200.1	1055.488763	1.286846855	0.043610958	29.50742	2.31E-191	6.46E-190	cyan	0.879457939	7.71E-52	GO:0007264,GO:0005525	AT5G17060.1	ARFB1B,ATARFB1B	ADP-ribosylation factor B1B	ADP ribosylation factor 1
Sapur.013G045500.1	268.9629202	4.329426681	0.147445555	29.36288	1.64E-189	4.53E-188	cyan	0.809286059	1.21E-37	GO:0005515,GO:0006468,GO:0005524,GO:0004672	AT1G08590.1		Leucine-rich receptor-like protein kinase family protein	LRR receptor-like kinase
Sapur.002G151800.1	1781.160991	4.041330086	0.137719383	29.34467	2.79E-189	7.72E-188	cyan	0.755297768	3.04E-30		AT3G63010.1	ATGID1B,GID1B	alpha/beta-Hydrolases superfamily protein	gibberellin receptor GID1, putative
Sapur.018G092800.1	107.7781841	9.495802409	0.324355109	29.27595	2.10E-188	5.76E-187	cyan	0.658526383	7.04E-21	GO:0005509	AT4G32060.1		calcium-binding EF hand family protein	NA
Sapur.018G009500.1	2057.127839	2.009253127	0.068681008	29.25486	3.89E-188	1.07E-186	cyan	0.844383208	7.35E-44	GO:0050662,GO:0003824	AT5G10730.1		NAD(P)-binding Rossmann-fold superfamily protein	NAD-dependent epimerase/dehydratase family protein
Sapur.019G035800.1	229.9052557	2.068567008	0.070769849	29.2295	8.18E-188	2.23E-186	cyan	0.845069285	5.37E-44		AT3G03810.1	EDA30	O-fucosyltransferase family protein	auxin-independent growth promoter protein
Sapur.002G179700.1	297.5527302	2.434171004	0.08330604	29.21962	1.09E-187	2.97E-186	cyan	0.821474855	1.20E-39		AT2G43060.1	IBH1	ILI1 binding bHLH 1	NA
Sapur.010G179300.1	251.4601228	1.824344768	0.062468052	29.20444	1.70E-187	4.62E-186	cyan	0.866685373	1.13E-48	GO:0005515	AT5G04220.2	ATSYTC.NTMC2T1.3.NTMC:	Calcium-dependent lipid-binding (CaLB domain) family protein	calcium lipid-binding protein, putative
Sapur.014G028500.1	646.8230142	2.783694914	0.095326982	29.20154	1.85E-187	5.03E-186	cyan	0.782316749	1.11E-33	GO:0055114,GO:0020037,GO:0016705,GO:0005506	AT4G31940.1	CYP82C4	cytochrome P450, family 82, subfamily C, polypeptide 4	cytochrome P450 family protein
Sapur.010G091000.1	869.6816661	1.847319467	0.06338759	29.14324	1.02E-186	2.75E-185	cyan	0.848630519	1.02E-44	GO:0055085,GO:0016021	AT4G22990.1		Major Facilitator Superfamily with SPX (SYG1/Pho81/XPR1) domain-containing protein	SPX domain membrane plant protein
Sapur.004G032800.1	359.7158969	5.99443584	0.206055606	29.09135	4.62E-186	1.24E-184	cyan	0.609591141	2.40E-17		AT4G18425.1		Protein of unknown function (DUF679)	transmembrane protein, putative
Sapur.002G104800.1	71.83750854	9.512236324	0.327764903	29.02152	3.52E-185	9.31E-184	cyan	0.595240186	2.01E-16	GO:0006629	AT2G26560.1	PLA IIA,PLA2A,PLP2	phospholipase A 2A	patatin-like phospholipase
Sapur.018G076700.1	964.6239478	1.342117518	0.046286888	28.99563	7.47E-185	1.97E-183	cyan	0.90691608	4.59E-60	GO:0005524	AT4G28070.2		AFG1-like ATPase family protein	AFG1 family ATPase
Sapur.014G118800.1	845.616873	1.533447152	0.052958231	28.95579	2.37E-184	6.22E-183	cyan	0.874329503	1.58E-50	GO:0006508,GO:0004190	AT1G05840.1		Eukaryotic aspartyl protease family protein	aspartic proteinase-like protein
Sapur.002G094600.1	153.1366753	3.135760359	0.108325614	28.94754	3.01E-184	7.88E-183	cyan	0.78432912	5.86E-34					hypothetical protein
Sapur.002G053600.1	990.2181514	1.549465006	0.053532104	28.94459	3.28E-184	8.58E-183	cyan	0.859116175	6.01E-47		AT1G21200.1		sequence-specific DNA binding transcription factors	transcription factor, putative
Sapur.016G108200.1	750.3680037	1.515587963	0.052603913	28.81132	1.55E-182	3.99E-181	cyan	0.873637083	2.36E-50		AT3G08890.1		Protein of unknown function, DUF538	DUF538 family protein
Sapur.004G015900.1	58.62247473	4.727091002	0.164132921	28.80038	2.12E-182	5.46E-181	cyan	0.71897838	2.87E-26	GO:0006468,GO:0004672	AT4G05200.1	CRK25	cysteine-rich RLK (RECEPTOR-like protein kinase) 25	NA
Sapur.004G016100.1	60.44127106	8.698168535	0.302862243	28.71988	2.15E-181	5.48E-180	cyan	0.679280821	1.39E-22	GO:0006468,GO:0005524,GO:0004672	AT4G21410.1	CRK29	cysteine-rich RLK (RECEPTOR-like protein kinase) 29	NA
Sapur.008G137600.1	40.14330072	7.037114593	0.245142774	28.70619	3.19E-181	8.12E-180	cyan	0.754603111	3.67E-30	GO:0016021	AT3G23430.1	ATPHO1,PHO1	phosphate 1	phosphate transporter Pho1
Sapur.15ZG047700.1	38.90225111	7.60440689	0.265000107	28.69586	4.30E-181	1.09E-179	cyan	0.789727621	1.03E-34					NA
Sapur.006G054200.1	92.26261722	5.130932134	0.178849107	28.68861	5.29E-181	1.34E-179	cyan	0.724955547	7.04E-27	GO:0005543	AT4G32285.2		ENTH/ANTH/VHS superfamily protein	NA
Sapur.011G121100.1	58.52187846	-2.036092159	0.071220311	-28.5886	9.30E-180	2.33E-178	cyan	-0.667329747	1.38E-21	GO:0008152,GO:0003824	AT1G30520.1	AAE14	acyl-activating enzyme 14	O-succinylbenzoate-CoA ligase
Sapur.017G074700.1	1336.184761	1.577415679	0.05523679	28.55734	2.28E-179	5.71E-178	cyan	0.847159424	2.04E-44	GO:0016757,GO:0047262	AT3G01040.2	GAUT13	galacturonosyltransferase 13	CAZy family GT8 glycosyltransferase
Sapur.017G116500.1	1149.529322	1.369727632	0.048026748	28.5201	6.60E-179	1.64E-177	cyan	0.870297142	1.56E-49		AT3G02700.1		NC domain-containing protein-related	lecithin retinol acyltransferase
Sapur.006G061900.1	42.2806487	3.947929259	0.138500784	28.50474	1.02E-178	2.54E-177	cyan	0.768562921	7.12E-32		AT1G06980.1			DUF4228 domain protein
Sapur.15WG096100.1	140.2691778	2.442260605	0.085896914	28.43246	8.03E-178	1.98E-176	cyan	0.841404839	2.83E-43	GO:0003950	AT5G62520.1	SRO5	similar to RCD one 5	inactive poly [ADP-ribose] polymerase SRO5, putative
Sapur.005G121300.1	468.0990245	1.53434884	0.05397588	28.42656	9.50E-178	2.34E-176	cyan	0.863972718	4.83E-48	GO:0055085,GO:0005741	AT3G49920.1	ATVDAC5,VDAC5	voltage dependent anion channel 5	porin/voltage-dependent anion-selective channel protein
Sapur.016G074000.1	626.4293626	1.875538403	0.066077812	28.38378	3.21E-177	7.87E-176	cyan	0.860259568	3.35E-47		AT3G53540.1			DUF4378 domain protein
Sapur.017G074100.1	744.5397151	1.565033125	0.055163652	28.37073	4.65E-177	1.14E-175	cyan	0.872632963	4.18E-50	GO:0005516	AT5G39380.1		Plant calmodulin-binding protein-related	plant calmodulin-binding domain protein
Sapur.013G006700.1	7955.896896	1.628569563	0.057447569	28.3488	8.66E-177	2.12E-175	cyan	0.873224409	2.99E-50					NA
Sapur.009G029900.1	46.01700832	4.310425558	0.152204637	28.31994	1.96E-176	4.78E-175	cyan	0.777238375	5.33E-33		AT5G05820.1		Nucleotide-sugar transporter family protein	NA
Sapur.012G017700.1	758.3533335	1.854474329	0.065489754	28.31701	2.13E-176	5.19E-175	cyan	0.871944975	6.18E-50	GO:0006355,GO:0003677	AT5G61430.1	ANAC100,ATNAC5,NAC100	NAC domain containing protein 100	NAC transcription factor
Sapur.010G205200.1	49.34242792	3.348187633	0.118253781	28.31358	2.35E-176	5.72E-175	cyan	0.83435868	6.15E-42	GO:0009733	AT2G24400.1		SAUR-like auxin-responsive protein family	auxin-responsive family protein
Sapur.012G006900.1	31.64712512	5.159223857	0.182245252	28.30924	2.66E-176	6.46E-175	cyan	0.803565692	9.42E-37	GO:0016021,GO:0016020,GO:0022857	AT2G37460.1		nodulin MtN21 /EamA-like transporter family protein	auxin-induced protein 5NG4
Sapur.008G131100.1	1122.12218	0.825511992	0.029177157	28.29309	4.20E-176	1.02E-174	cyan	0.893570885	8.72E-56	GO:0055085,GO:0046873,GO:0030001,GO:0016020	AT5G64560.1	ATMGT9,MGT9,MRS2-2	magnesium transporter 9	magnesium transporter MRS2-I
Sapur.010G090800.1	138.772754	1.682843426	0.059847705	28.11876	5.78E-174	1.38E-172	cyan	0.882480871	1.22E-52		AT3G02600.2	ATLPP3,LPP3	lipid phosphate phosphatase 3	lipid phosphate phosphatase
Sapur.008G126200.1	1678.814006	2.380208152	0.084725859	28.09305	1.19E-173	2.83E-172	cyan	0.784359922	5.80E-34	GO:0006468,GO:0004672	AT3G22750.1		Protein kinase superfamily protein	tyrosine kinase domain protein
Sapur.008G142000.1	92.81541715	4.391772917	0.156472067	28.06746	2.45E-173	5.79E-172	cyan	0.644664486	8.19E-20	GO:0005515	AT3G09270.1	ATGSTU8,GSTU8	glutathione S-transferase TAU 8	tau class glutathione transferase GSTU19
Sapur.001G088500.1	754.417664	1.664710411	0.059390296	28.03001	7.00E-173	1.66E-171	cyan	0.827109265	1.26E-40	GO:0016021,GO:0004252,GO:0006508	AT1G63120.1	ATRBL2,RBL2	RHOMBOID-like 2	membrane protein,; 61952-60281, putative
Sapur.004G038000.1	346.3881982	2.43985627	0.087215256	27.97511	3.26E-172	7.69E-171	cyan	0.774071174	1.39E-32		AT1G29050.1	TBL38	TRICHOME BIREFRINGENCE-LIKE 38	Pmr5/Cas1p GDSL/SGNH-like acyl-esterase family protein
Sapur.003G124400.1	24.99510677	7.196359332	0.258907306	27.79512	4.97E-170	1.15E-168	cyan	0.813768617	2.31E-38		AT4G24970.1		Histidine kinase-, DNA gyrase B-, and HSP90-like ATPase family protein	NA
Sapur.002G177300.1	54.00919084	7.972965478	0.28713179	27.76762	1.07E-169	2.45E-168	cyan	0.667010184	1.47E-21		AT3G21680.1			DUF4228 domain protein
Sapur.016G077500.1	19.08736156	-3.822292662	0.137774335	-27.7431	2.11E-169	4.84E-168	cyan	-0.862046003	1.33E-47	GO:0008270,GO:0005515	AT5G02750.1		RING/U-box superfamily protein	zinc finger protein, putative
Sapur.019G062100.1	213.2166485	3.516123328	0.126783359	27.73332	2.77E-169	6.34E-168	cyan	0.862793184	8.99E-48					hypothetical protein
Sapur.005G081600.1	1618.384864	1.5527887	0.055993765	27.73146	2.92E-169	6.68E-168	cyan	0.835568808	3.66E-42	GO:0006468,GO:0005524,GO:0004672	AT2G17530.1		Protein kinase superfamily protein	Serine/Threonine kinase domain protein
Sapur.019G007400.1	87.17309336	7.595183233	0.275367888	27.58195	1.83E-167	4.12E-166	cyan	0.607865192	3.11E-17	GO:0005524,GO:0007165,GO:0005515	AT5G36930.2		Disease resistance protein (TIR-NBS-LRR class) family	NA
Sapur.001G152700.1	309.2958896	1.038231578	0.037646288	27.57859	2.01E-167	4.51E-166	cyan	0.89074722	5.93E-55		AT1G52320.2			DUF632 family protein
Sapur.011G083800.1	19.74129877	4.217701238	0.153149423	27.53978	5.87E-167	1.31E-165	cyan	0.827585383	1.04E-40		AT4G03510.1	ATRMA1,RMA1	RING membrane-anchor 1	zinc finger, C3HC4 type (RING finger) protein
Sapur.018G081800.1	675.0812576	1.121503294	0.040784937	27.49798	1.86E-166	4.12E-165	cyan	0.877454658	2.55E-51	GO:0016021	AT2G24170.1		Endomembrane protein 70 protein family	NA
Sapur.014G132100.1	1585.380631	1.304693504	0.047505146	27.46426	4.69E-166	1.04E-164	cyan	0.862525417	1.03E-47	GO:0003676	AT3G07810.1		RNA-binding (RRM/RBD/RNP motifs) family protein	RNA recognition motif 1 in heterogeneous nuclear ribonucleoprotein A subfamily protein
Sapur.016G032900.1	362.3112508	2.225122244	0.081119742	27.4301	1.20E-165	2.65E-164	cyan	0.770970734	3.51E-32					transmembrane protein, putative
Sapur.001G081100.1	27.2582648	5.195515095	0.189835422	27.36852	6.50E-165	1.43E-163	cyan	0.732785381	1.05E-27	GO:0016114,GO:0008685	AT1G63970.1	ISPF,MECPS	isoprenoid F	2-C-methyl-D-erythritol 2%2C4-cyclodiphosphate synthase
Sapur.010G082500.1	1588.003857	1.738122166	0.063623244	27.31898	2.52E-164	5.52E-163	cyan	0.873560012	2.46E-50	GO:0071704,GO:0016868,GO:0005975	AT1G70730.1		Phosphoglucomutase/phosphomannomutase family protein	cytoplasmic phosphoglucomutase
Sapur.003G134200.1	681.6004672	1.41625672	0.051948759	27.26257	1.18E-163	2.57E-162	cyan	0.850112956	5.07E-45	GO:0016192,GO:0016021	AT1G04760.1	ATVAMP726,VAMP726	vesicle-associated membrane protein 726	vesicle-associated membrane protein, synaptobrevin 7B, putative
Sapur.018G032300.1	226.3134511	2.274213161	0.083541895	27.22243	3.53E-163	7.66E-162	cyan	0.826124733	1.88E-40	GO:0055114,GO:0050660,GO:0016491,GO:0016020,GO:0003885	AT5G11540.1		D-arabinono-1,4-lactone oxidase family protein	FAD-dependent oxidoreductase
Sapur.007G005700.1	4452.536966	1.514534445	0.055677728	27.2018	6.18E-163	1.34E-161	cyan	0.833987341	7.21E-42	GO:0071949	AT1G58440.1	SQE1,XF1	FAD/NAD(P)-binding oxidoreductase family protein	squalene monooxygenase
Sapur.011G031700.1	361.3664587	2.012616406	0.074118452	27.15405	2.27E-162	4.88E-161	cyan	0.844533637	6.86E-44		AT1G61100.1		disease resistance protein (TIR class), putative	TIR class disease resistance protein, putative
Sapur.012G079600.1	316.5613364	3.090000419	0.113872645	27.13558	3.75E-162	8.05E-161	cyan	0.684453016	4.96E-23	GO:0031012,GO:0008270,GO:0006508,GO:0004222	AT1G24140.1		Matrixin family protein	matrix metalloproteinase
Sapur.001G160400.1	30.06111899	3.326804115	0.122690461	27.11543	6.48E-162	1.39E-160	cyan	0.825158777	2.78E-40	GO:0003676	AT3G16380.1	PAB6	poly(A) binding protein 6	RNA recognition motif 4 in type I polyadenylate-binding family protein, putative
Sapur.T089400.1	27.09265838	5.51231314	0.203309719	27.11289	6.94E-162	1.49E-160	cyan	0.720303135	2.11E-26	GO:0006468,GO:0005524,GO:0004672	AT5G18610.2		Protein kinase superfamily protein	receptor Serine/Threonine kinase, putative
Sapur.006G216400.1	43.50813869	2.867716425	0.105919484	27.07449	1.97E-161	4.19E-160	cyan	0.781296588	1.52E-33	GO:0020037,GO:0017004	AT5G54830.1		DOMON domain-containing protein / dopamine beta-monooxygenase N-terminal domain-containing protein	DOMON and dopamine beta-monooxygenase amino-terminal domain protein, putative
Sapur.007G066400.1	370.869252	0.941674222	0.034870156	27.00516	1.29E-160	2.72E-159	cyan	0.873947637	1.97E-50		AT5G23130.1		Peptidoglycan-binding LysM domain-containing protein	carbohydrate-binding module family 50 protein
Sapur.006G087400.1	27.46623814	2.939801328	0.109106653	26.94429	6.65E-160	1.40E-158	cyan	0.841710661	2.47E-43	GO:0030170,GO:0008483	AT3G08860.1	PYD4	PYRIMIDINE 4	alanine-glyoxylate aminotransferase, putative
Sapur.004G121200.1	1597.500658	0.95853214	0.03561483	26.91385	1.51E-159	3.18E-158	cyan	0.895128639	2.96E-56	GO:0016740,GO:0009058	AT4G34640.1	ERG9,SQS1	squalene synthase 1	squalene synthase
Sapur.017G065500.1	3931.880914	0.795189389	0.029610331	26.85513	7.35E-159	1.53E-157	cyan	0.885706986	1.60E-53	GO:0033179,GO:0015991,GO:0015078	AT3G28715.1		ATPase, V0/A0 complex, subunit C/D	vacuolar H+ ATPase V0 sector, subunit D
Sapur.010G134100.1	205.4035607	7.374327568	0.2746531	26.84961	8.52E-159	1.78E-157	cyan	0.548711531	1.01E-13	GO:0055085,GO:0046873,GO:0030001,GO:0016020	AT3G12750.1	ZIP1	zinc transporter 1 precursor	NA
Sapur.005G041900.1	1651.426321	-1.489664379	0.055675678	-26.7561	1.05E-157	2.17E-156	cyan	-0.89439129	4.95E-56	GO:0006355,GO:0005634	AT5G43700.1	ATAUX2-11,IAA4	AUX/IAA transcriptional regulator family protein	indole-3-acetic acid inducible protein
Sapur.009G061300.1	458.1165493	1.887624891	0.070577748	26.74533	1.40E-157	2.89E-156	cyan	0.802433294	1.40E-36		AT2G23970.1		Class I glutamine amidotransferase-like superfamily protein	GMP synthase [glutamine-hydrolyzing] subunit A, putative
Sapur.002G028900.1	96.95833672	2.001253031	0.074978353	26.69108	5.97E-157	1.23E-155	cyan	0.870785174	1.19E-49	GO:0016567,GO:0004842,GO:0005515	AT3G54790.1		ARM repeat superfamily protein	NA
Sapur.008G081900.1	74.4661208	-1.742032809	0.065274732	-26.6877	6.54E-157	1.34E-155	cyan	-0.867435331	7.53E-49	GO:0043565,GO:0006355,GO:0003700	AT5G49520.1	ATWRKY48,WRKY48	WRKY DNA-binding protein 48	WRKY transcription factor, putative
Sapur.018G088500.1	1226.185065	3.033452205	0.113702639	26.67882	8.29E-157	1.70E-155	cyan	0.778373618	3.76E-33		AT5G35110.1			hypothetical protein
Sapur.011G122900.1	426.3416993	1.981604493	0.074549907	26.58091	1.13E-155	2.31E-154	cyan	0.813165789	2.89E-38		AT3G13275.1			NA
Sapur.016G153100.1	39.37500794	5.568422439	0.210566419	26.44497	4.17E-154	8.47E-153	cyan	0.717945717	3.64E-26		AT3G63520.1	ATCCD1.ATNCED1.CCD1.NC	carotenoid cleavage dioxygenase 1	NA
Sapur.004G127700.1	235.4315286	1.144196896	0.043274438	26.44048	4.70E-154	9.53E-153	cyan	0.885344145	2.01E-53	GO:0016021,GO:0015693,GO:0015095	AT2G21120.1		Protein of unknown function (DUF803)	Non-imprinted in prader-willi/angelman syndrome region protein, putative
Sapur.003G134800.1	2585.652497	0.7245267	0.027433984	26.40982	1.06E-153	2.14E-152	cyan	0.89509	3.04E-56		AT3G24160.1	PMP	putative type 1 membrane protein	PMP protein, putative
Sapur.001G182200.1	39.33667182	8.379932103	0.317868429	26.3629	3.65E-153	7.36E-152	cyan	0.640672086	1.62E-19	GO:0055114,GO:0020037,GO:0016705,GO:0005506	AT4G37370.1	CYP81D8	cytochrome P450, family 81, subfamily D, polypeptide 8	cytochrome P450 family protein
Sapur.001G160900.1	1051.918748	-1.607963761	0.061003256	-26.3587	4.08E-153	8.22E-152	cyan	-0.903306019	7.59E-59	GO:0003993	AT4G25150.1		HAD superfamily, subfamily IIIB acid phosphatase	stem 28 kDa glycoprotein
Sapur.010G160700.1	43.66689681	7.283203266	0.276369362	26.35315	4.72E-153	9.50E-152	cyan	0.749339071	1.52E-29		AT2G16050.1		Cysteine/Histidine-rich C1 domain family protein	DC1; C1-like protein, putative
Sapur.012G038000.1	155.1306168	1.605136691	0.060939729	26.33974	6.73E-153	1.35E-151	cyan	0.840287239	4.66E-43		AT1G25370.1		Protein of unknown function (DUF1639)	DUF1639 family protein
Sapur.006G036700.1	723.5056819	1.770829042	0.067242639	26.33491	7.64E-153	1.53E-151	cyan	0.814439156	1.79E-38	GO:0016757	AT2G41640.1		Glycosyltransferase family 61 protein	glycosyltransferase
Sapur.001G074900.1	83.62834967	-2.591267973	0.098413102	-26.3305	8.58E-153	1.72E-151	cyan	-0.837576426	1.54E-42		AT4G23880.1			NA
Sapur.15WG089800.1	160.1723123	3.176553119	0.120652665	26.32808	9.15E-153	1.83E-151	cyan	0.802615175	1.32E-36	GO:0005515	AT5G50120.1		Transducin/WD40 repeat-like superfamily protein	F-box and WD40 domain protein, putative
Sapur.010G005000.1	148.5423701	1.591098453	0.06045259	26.31977	1.14E-152	2.28E-151	cyan	0.840562603	4.12E-43		AT3G06780.1		glycine-rich protein	hypothetical protein
Sapur.006G051600.1	806.4146824	1.745308147	0.066477756	26.25402	6.43E-152	1.28E-150	cyan	0.819965083	2.17E-39	GO:0016021	AT5G20270.1	HHP1	heptahelical transmembrane protein1	hemolysin-III-like protein
Sapur.002G112600.1	907.0485547	1.000635685	0.038123874	26.24696	7.74E-152	1.54E-150	cyan	0.874254776	1.65E-50	GO:0005525	AT1G02130.1	ARA-5.ARA5.ATRAB1B.ATRA	RAS 5	Ras small GTPase family Ras protein
Sapur.016G126000.1	393.3506597	3.99154346	0.152093634	26.24399	8.37E-152	1.66E-150	cyan	0.630387278	9.02E-19		AT2G44260.1		Plant protein of unknown function (DUF946)	vacuolar protein sorting-associated protein, putative
Sapur.014G133000.1	306.9831754	1.611818082	0.061586158	26.17176	5.57E-151	1.10E-149	cyan	0.815805606	1.07E-38	GO:0005515	AT3G07870.1		F-box and associated interaction domains-containing protein	F-box protein
Sapur.018G094500.1	360.5978507	1.684647761	0.064427425	26.14799	1.04E-150	2.04E-149	cyan	0.838538273	1.01E-42	GO:0005515	AT2G25290.1		Octicosapeptide/Phox/Bem1p (PB1) domain-containing protein / tetratricopeptide repeat (TPR)-containing protei	NA
Sapur.002G074400.1	265.4897544	3.835809903	0.146740483	26.14009	1.28E-150	2.50E-149	cyan	0.88118071	2.71E-52	GO:0016021,GO:0015116,GO:0008272,GO:0055085,GO:0016020,GO:0008271,GO:000588	AT1G22150.1	SULTR1;3	sulfate transporter 1;3	high affinity sulfate transporter
Sapur.006G224600.1	19.72611827	5.312650314	0.204011098	26.04099	1.70E-149	3.30E-148	cyan	0.785976134	3.47E-34	GO:0016020,GO:0008233,GO:0006465	AT1G52600.1		Peptidase S24/S26A/S26B/S26C family protein	microsomal signal peptidase subunit
Sapur.008G156000.1	29.73464189	7.823549575	0.300709841	26.01694	3.19E-149	6.17E-148	cyan	0.634277502	4.75E-19					transmembrane protein, putative
Sapur.T009200.1	17.94499699	4.43993231	0.171578581	25.87696	1.21E-147	2.32E-146	cyan	0.79295208	3.57E-35					NA
Sapur.010G059800.1	2189.01969	1.337740946	0.051701747	25.87419	1.30E-147	2.49E-146	cyan	0.847550861	1.70E-44		AT4G14950.1		SNARE associated Golgi protein family	SNARE associated protein
Sapur.014G064100.1	1501.966192	1.223579837	0.047372805	25.82874	4.22E-147	8.02E-146	cyan	0.862227085	1.21E-47	GO:0047617,GO:0006637	AT1G01710.1		Acyl-CoA thioesterase family protein	acyl-CoA thioesterase
Sapur.012G019400.1	238.8859771	1.760161114	0.068291478	25.77424	1.72E-146	3.27E-145	cyan	0.841462124	2.76E-43		AT3G61640.1	AGP20,AtAGP20	arabinogalactan protein 20	arabinogalactan peptide protein
Sapur.018G093200.1	56.27811763	8.971775733	0.348750545	25.72548	6.06E-146	1.15E-144	cyan	0.617942451	6.61E-18	GO:0005509	AT4G32060.1		calcium-binding EF hand family protein	NA
Sapur.014G133200.1	12116.78518	2.18889303	0.085113154	25.71745	7.46E-146	1.41E-144	cyan	0.837187401	1.82E-42					hypothetical protein
Sapur.011G029700.1	1763.28535	1.507449496	0.058678822	25.68984	1.52E-145	2.86E-144	cyan	0.849649683	6.32E-45	GO:0006308,GO:0004519,GO:0003676	AT4G21585.1	ENDO4	endonuclease 4	nuclease
Sapur.013G073700.1	393.7519573	-1.127300878	0.044080116	-25.5739	2.98E-144	5.55E-143	cyan	-0.867316208	8.03E-49	GO:0016788,GO:0006886,GO:0006505	AT5G17670.1		alpha/beta-Hydrolases superfamily protein	hydrolase-like protein
Sapur.002G088700.1	478.9606556	2.271534311	0.088890998	25.55415	4.94E-144	9.18E-143	cyan	0.755569172	2.82E-30		AT1G09890.1		Rhamnogalacturonate lyase family protein	rhamnogalacturonate lyase family protein
Sapur.002G052800.1	22.10207238	-2.716443632	0.106403298	-25.5297	9.23E-144	1.71E-142	cyan	-0.779705583	2.50E-33		AT1G21090.1		Cupredoxin superfamily protein	hypothetical protein
Sapur.004G113300.1	28.44448573	4.324483281	0.169760422	25.47404	3.82E-143	7.05E-142	cyan	0.675222171	3.07E-22		AT4G34320.1		Protein of unknown function (DUF677)	transmembrane protein, putative
Sapur.010G049800.1	58.00725301	5.393998309	0.212024963	25.44039	9.02E-143	1.66E-141	cyan	0.66780921	1.26E-21	GO:0005515	AT2G29420.1	ATGSTU7,GST25,GSTU7	glutathione S-transferase tau 7	tau class glutathione transferase GSTU36
Sapur.017G099500.1	46.17713964	-2.738466488	0.107663485	-25.4354	1.02E-142	1.88E-141	cyan	-0.842306697	1.89E-43		AT2G47485.1			nucleolar protein, putative
Sapur.007G112600.1	652.0319183	0.898641859	0.035342177	25.4269	1.27E-142	2.33E-141	cyan	0.872926236	3.54E-50	GO:0016567,GO:0004842	AT2G31510.1	ARI7,ATARI7	IBR domain-containing protein	NA
Sapur.007G079000.1	2521.952877	-1.222407613	0.048095061	-25.4165	1.66E-142	3.04E-141	cyan	-0.889179785	1.68E-54					transmembrane protein, putative
Sapur.005G156300.1	154.8205109	1.607564822	0.063312712	25.39087	3.18E-142	5.82E-141	cyan	0.842521239	1.71E-43		AT1G71780.1			transmembrane protein, putative

Sapur.006G006800.1	2015.076445	-1.139275038	0.044970114	-25.334	1.35E-141	2.45E-140	cyan	-0.908818391	9.98E-61	GO:0009055	AT2G32300.1	UCC1	uclacyanin 1	NA
Sapur.009G054700.1	80.25343984	-3.962944025	0.156759514	-25.2804	5.25E-141	9.51E-140	cyan	-0.822626947	7.63E-40					hypothetical protein
Sapur.002G142600.1	877.3355795	1.462604642	0.057973926	25.22866	1.94E-140	3.50E-139	cyan	0.845154793	5.16E-44		AT4G10810.1			hypothetical protein
Sapur.006G059400.1	70.9538533	-3.042041309	0.120751351	-25.1926	4.83E-140	8.68E-139	cyan	-0.822802044	7.12E-40		AT5G57123.1			NA
Sapur.016G127900.1	1009.346921	1.519364011	0.060362647	25.1706	8.41E-140	1.51E-138	cyan	0.849987037	5.38E-45	GO:0016747,GO:0008610,GO:0016020,GO:0006633	AT2G28630.1	KCS12	3-ketoacyl-CoA synthase 12	3-ketoacyl-CoA synthase
Sapur.006G000900.1	1233.701157	0.741985637	0.029500096	25.15197	1.34E-139	2.40E-138	cyan	0.869168449	2.91E-49	GO:0005525	AT4G17170.1	AT-RAB2.ATRAB-B1B.ATRAI	RAB GTPase homolog B1C	small GTPase family Rab protein
Sapur.013G128100.1	226.904524	1.461838162	0.058198479	25.11815	3.15E-139	5.61E-138	cyan	0.849086498	8.25E-45					NA
Sapur.009G077700.1	20.32644395	4.731093489	0.188422237	25.109	3.97E-139	7.05E-138	cyan	0.772016855	2.57E-32		AT4G34120.1	CDCP1,LEJ1	Cystathionine beta-synthase (CBS) family protein	CBS-pair-ACT-assoc protein, putative
Sapur.010G012700.1	171.6670718	2.912588364	0.116016868	25.10487	4.40E-139	7.81E-138	cyan	0.681115959	9.66E-23		AT5G18980.1		ARM repeat superfamily protein	armadillo/beta-catenin-like repeat protein
Sapur.008G076000.1	304.1852537	2.686312268	0.107189739	25.06128	1.32E-138	2.33E-137	cyan	0.793263966	3.22E-35		AT2G03630.1			hypothetical protein
Sapur.003G146900.1	415.9481147	1.471009905	0.058732962	25.04573	1.94E-138	3.42E-137	cyan	0.839558443	6.43E-43		AT2G26920.1		Ubiquitin-associated/translation elongation factor EF1B protein	UBA/TS-N domain protein
Sapur.009G005300.1	81.56632215	2.827600223	0.112995654	25.02397	3.35E-138	5.90E-137	cyan	0.821302031	1.29E-39	GO:0006355,GO:0003677	AT2G27990.1	BLH8,PNF	BEL1-like homeodomain 8	BEL1 homeotic protein, putative
Sapur.006G059800.1	557.8833625	1.485180213	0.059383246	25.01009	4.75E-138	8.34E-137	cyan	0.822795877	7.13E-40	GO:0005515,GO:0006468,GO:0004672	AT3G23750.1		Leucine-rich repeat protein kinase family protein	NA
Sapur.007G104500.1	101.8193054	1.391490235	0.05563864	25.00942	4.83E-138	8.47E-137	cyan	0.845443151	4.52E-44		AT5G61840.1	GUT1	Exostosin family protein	beta-1%2C4-xylosyltransferase IRX10, putative
Sapur.018G001700.1	160.1288232	3.327054346	0.133124753	24.992	7.47E-138	1.31E-136	cyan	0.762133646	4.53E-31		AT4G31290.1		ChaC-like family protein	cation transporter ChaC
Sapur.011G066200.1	33.54258342	2.481639563	0.099497152	24.94182	2.62E-137	4.57E-136	cyan	0.837832977	1.37E-42					NA
Sapur.001G080300.1	40.39222266	3.426073728	0.137406551	24.93385	3.20E-137	5.57E-136	cyan	0.71064162	1.92E-25		AT1G64065.1		Late embryogenesis abundant (LEA) hydroxyproline-rich glycoprotein family	late embryogenesis abundant hydroxyproline-rich glycoprotein, putative
Sapur.006G166300.1	744.9375797	1.404275792	0.056654155	24.78681	1.24E-135	2.14E-134	cyan	0.848911117	8.97E-45		AT5G06370.1		NC domain-containing protein-related	lecithin retinol acyltransferase
Sapur.010G057800.1	20.08324131	2.781035622	0.112217351	24.78258	1.38E-135	2.37E-134	cyan	0.794936061	1.84E-35	GO:0046983,GO:0003677	AT5G60440.1	AGL62	AGAMOUS-like 62	MADS-box protein AGL75, putative
Sapur.005G184100.1	620.2725018	1.017867222	0.04109299	24.76985	1.89E-135	3.25E-134	cyan	0.887109834	6.49E-54		AT2G21520.1		Sec14p-like phosphatidylinositol transfer family protein	Sec.4-like phosphatidylinositol transfer protein
Sapur.002G033000.1	1896.907161	-1.044200689	0.042164688	-24.7648	2.15E-135	3.67E-134	cyan	-0.851395193	2.75E-45	GO:0006334,GO:0005634,GO:0003677,GO:0000786	AT2G30620.1		winged-helix DNA-binding transcription factor family protein	histone H1 subtype 5
Sapur.013G112100.1	4533.823197	1.105759151	0.044711762	24.73083	4.99E-135	8.51E-134	cyan	0.880648446	3.75E-52	GO:0055114,GO:0016627,GO:0003995,GO:0006635,GO:0005777,GO:0003997	AT1G06290.1	ACX3,ATACX3	acyl-CoA oxidase 3	acyl-coenzyme A oxidase
Sapur.006G053300.1	1110.657932	0.750988914	0.030367669	24.72988	5.10E-135	8.71E-134	cyan	0.867944552	5.70E-49	GO:0005515	AT5G32440.1		Ubiquitin system component Cue protein	ubiquitin system component CUE protein, putative
Sapur.T017700.1	429.1345105	2.103806669	0.085152476	24.70635	9.14E-135	1.56E-133	cyan	0.836827801	2.13E-42	GO:0005515,GO:0006468,GO:0004672	AT3G14840.2		Leucine-rich repeat transmembrane protein kinase	NA
Sapur.014G060200.1	28.39719436	3.026892892	0.122521276	24.70504	9.44E-135	1.61E-133	cyan	0.824052616	4.33E-40	GO:0005515	AT2G45760.1	BAL,BAP2	BON association protein 2	C2 domain protein
Sapur.002G175100.1	26.33882835	4.287200167	0.173668285	24.68614	1.51E-134	2.56E-133	cyan	0.740001158	1.73E-28	GO:0006396,GO:0004525,GO:0003723	AT4G15417.1	ATRTL1,RTL1	RNAse II-like 1	endoribonuclease dicer-like protein
Sapur.008G008000.1	20.37864985	3.432102182	0.139088517	24.67567	1.95E-134	3.31E-133	cyan	0.746244276	3.44E-29	GO:0004857	AT5G64620.1	ATC/VIF2,C/VIF2	cell wall / vacuolar inhibitor of fructosidase 2	plant invertase/pectin methylesterase inhibitor protein
Sapur.011G118300.1	1829.291676	1.655939636	0.067221366	24.63413	5.45E-134	9.22E-133	cyan	0.831856027	1.77E-41	GO:0055114,GO:0050660,GO:0016491	AT2G34790.1	EDA28,MEE23	FAD-binding Berberine family protein	reticuline oxidase, putative
Sapur.016G023400.1	447.9840082	1.710193668	0.069427753	24.63271	5.64E-134	9.54E-133	cyan	0.847022671	2.17E-44	GO:0008270	AT3G56930.1		DHHC-type zinc finger family protein	zinc finger protein, putative
Sapur.010G176300.1	340.3038166	2.32809895	0.0945583	24.62078	7.57E-134	1.28E-132	cyan	0.771820373	2.73E-32					NA
Sapur.15WG085400.1	266.5016253	1.125642426	0.045796266	24.57935	2.10E-133	3.53E-132	cyan	0.852778272	1.41E-45		AT3G47940.1		DNAJ heat shock family protein	heat-shock protein, putative
Sapur.013G051300.1	8.398184043	6.275287461	0.255775065	24.5344	6.35E-133	1.06E-131	cyan	0.820513379	1.75E-39	GO:0009733	AT4G31320.1		SAUR-like auxin-responsive protein family	NA
Sapur.15WG077700.1	21.93966863	-2.146170216	0.08756565	-24.5093	1.18E-132	1.97E-131	cyan	-0.858935441	6.59E-47		AT4G26190.1		Haloacid dehalogenase-like hydrolase (HAD) superfamily protein	NA
Sapur.014G031000.1	448.9170209	2.939480161	0.120001957	24.49527	1.66E-132	2.78E-131	cyan	0.711808665	1.48E-25		AT5G42610.1		Protein of unknown function (DUF607)	transcription factor, putative
Sapur.018G092300.1	70.51598184	9.598343535	0.392135467	24.47711	2.59E-132	4.32E-131	cyan	0.621880808	3.55E-18	GO:0005509	AT4G32060.1		calcium-binding EF hand family protein	NA
Sapur.018G069400.1	1153.068678	1.138874178	0.046532395	24.47487	2.74E-132	4.56E-131	cyan	0.828818418	6.27E-41	GO:0048193,GO:0016020	AT4G30240.1		Syntaxin/t-SNARE family protein	syntaxin family protein
Sapur.15WG043900.1	9.962617453	6.253831764	0.255614957	24.46583	3.41E-132	5.68E-131	cyan	0.826422885	1.67E-40		AT4G35280.1		C2H2-like zinc finger protein	zinc finger protein ZAT3, putative
Sapur.010G039600.1	626.7904233	0.807603532	0.033099686	24.39913	1.75E-131	2.89E-130	cyan	0.86639369	1.32E-48	GO:0055114,GO:0016491	AT1G06650.2		2-oxoglutarate (2OG) and Fe(II)-dependent oxygenase superfamily protein	1-aminocyclopropane-1-carboxylate oxidase
Sapur.010G046300.1	509.7901019	1.962756038	0.080453559	24.39614	1.88E-131	3.10E-130	cyan	0.827643638	1.02E-40		AT1G04530.1		Tetratricopeptide repeat (TPR)-like superfamily protein	O-linked N-acetylglucosamine transferase, ogt protein%2C putative
Sapur.010G132600.1	1695.367231	-1.12238733	0.046055338	-24.3704	3.52E-131	5.81E-130	cyan	-0.891439217	3.73E-55	GO:0006096,GO:0004618	AT3G12780.1	PGK1	phosphoglycerate kinase 1	phosphoglycerate kinase
Sapur.014G069600.1	422.6174156	1.318219666	0.054116199	24.35906	4.65E-131	7.66E-130	cyan	0.832013791	1.66E-41		AT1G01500.1		Erythronate-4-phosphate dehydrogenase family protein	2-hydroxyacid dehydrogenase, putative
Sapur.018G008800.1	275.7595165	1.689901141	0.069434825	24.33795	7.78E-131	1.28E-129	cyan	0.835856442	3.24E-42	GO:0016020,GO:0004970	AT2G29110.1	ATGLR2.8,GLR2.8	glutamate receptor 2.8	glutamate receptor
Sapur.016G078700.1	203.9948334	-1.263603453	0.051962473	-24.3176	1.28E-130	2.09E-129	cyan	-0.872583323	4.30E-50	GO:0006614,GO:0005525,GO:0048500,GO:0008312	AT5G03940.1	54CP.CPSRP54.FFC.SRP54CF	chloroplast signal recognition particle 54 kDa subunit	NA
Sapur.014G131100.1	574.21958	0.977597501	0.040252418	24.28668	2.71E-130	4.44E-129	cyan	0.861864123	1.46E-47		AT1G04960.1		Protein of unknown function (DUF1664)	BZIP transcription factor 63-a04, putative
Sapur.001G104700.1	202.8218748	-1.442287116	0.059582663	-24.2065	1.90E-129	3.08E-128	cyan	-0.886322745	1.08E-53	GO:0005089	AT1G31650.1	ATROPGEF14,ROPGEF14	RHO guanyl-nucleotide exchange factor 14	Rop guanine nucleotide exchange factor, putative
Sapur.001G076100.1	206.7768088	-0.99669825	0.041179387	-24.2038	2.03E-129	3.29E-128	cyan	-0.900635187	5.64E-58		AT5G41850.1		alpha/beta-Hydrolases superfamily protein	alpha/beta hydrolase family protein
Sapur.006G033200.1	45.74909029	2.637496457	0.109014373	24.19402	2.57E-129	4.16E-128	cyan	0.839063475	8.00E-43	GO:0005515	AT5G04010.1		F-box family protein	F-box protein
Sapur.003G113000.1	78.05038153	2.455005663	0.101590048	24.16581	5.09E-129	8.22E-128	cyan	0.781657124	1.36E-33					hypothetical protein
Sapur.005G150200.1	1008.933195	1.208148689	0.050049859	24.1389	9.77E-129	1.57E-127	cyan	0.879273753	8.61E-52	GO:0008121,GO:0006122	AT1G15120.1		Ubiquinol-cytochrome C reductase hinge protein	cytochrome B-c1 complex subunit 6
Sapur.15WG003800.1	115.242719	-2.085454683	0.086402088	-24.1366	1.03E-128	1.66E-127	cyan	-0.887877799	3.95E-54					transmembrane protein, putative
Sapur.014G034800.1	1283.677693	0.977751561	0.040552058	24.11152	1.89E-128	3.03E-127	cyan	0.845581964	4.24E-44	GO:0008138,GO:0006470	AT2G35680.1		Phosphotyrosine protein phosphatases superfamily protein	tyrosine phosphatase, putative
Sapur.010G090000.1	717.5009185	1.535337316	0.063720834	24.09475	2.84E-128	4.53E-127	cyan	0.81200705	4.45E-38	GO:0008270,GO:0005515	AT1G15100.1	RHA2A	RING-H2 finger A2A	RING finger protein
Sapur.005G196100.1	18.80599004	2.907217244	0.120680832	24.09013	3.17E-128	5.06E-127	cyan	0.802950898	1.17E-36	GO:0008270,GO:0005515	AT2G17730.1	NIP2	NEP-interacting protein 2	NA
Sapur.004G055100.1	581.1086473	1.362858628	0.05660556	24.07641	4.42E-128	7.03E-127	cyan	0.844853544	5.93E-44		AT3G02700.1		NC domain-containing protein-related	lecithin retinol acyltransferase
Sapur.010G056800.1	1056.467979	1.307195643	0.054300038	24.07357	4.73E-128	7.53E-127	cyan	0.831721026	1.88E-41		AT3G26000.1		Ribonuclease inhibitor	F-box protein
Sapur.006G138600.1	920.4473021	0.777400733	0.032336109	24.04126	1.03E-127	1.64E-126	cyan	0.871626622	7.39E-50	GO:0055085,GO:0005741	AT5G57490.1	ATVDAC4,VDAC4	voltage dependent anion channel 4	voltage-dependent anion-selective channel protein, putative
Sapur.004G037700.1	1895.967907	1.473026412	0.061274104	24.03995	1.06E-127	1.69E-126	cyan	0.826450913	1.65E-40	GO:0016788	AT5G45910.1		GDSL-like Lipase/Acylhydrolase superfamily protein	GDSL esterase/lipase
Sapur.016G043800.1	1316.747544	0.968826115	0.040357761	24.00594	2.41E-127	3.81E-126	cyan	0.84094846	3.47E-43		AT3G57890.1		Tubulin binding cofactor C domain-containing protein	tubulin-binding cofactor C
Sapur.019G016100.1	2545.246935	-1.698561946	0.070758514	-24.0051	2.46E-127	3.89E-126	cyan	-0.875650433	7.36E-51	GO:0016747	AT5G48930.1	HCT	hydroxycinnamoyl-CoA shikimate/quinate hydroxycinnamoyl transferase	NA
Sapur.019G018800.1	28.22403924	1.830492154	0.076295666	23.99209	3.36E-127	5.30E-126	cyan	0.863512216	6.16E-48	GO:0005515	AT3G14840.2		Leucine-rich repeat transmembrane protein kinase	NA
Sapur.007G119600.1	33.95084115	4.153026991	0.173107488	23.99103	3.45E-127	5.43E-126	cyan	0.610500319	2.09E-17	GO:0055114,GO:0020037,GO:0006979,GO:0004601	AT5G24070.1		Peroxidase superfamily protein	peroxidase, putative
Sapur.002G049800.1	348.8807554	0.94307578	0.039339251	23.9729	5.33E-127	8.38E-126	cyan	0.857619926	1.28E-46	GO:0005525	AT4G09720.1	ATRABG3A,RABG3A	RAB GTPase homolog G3A	Rab-related GTPase family protein
Sapur.001G126300.1	468.3290993	1.116549922	0.046643868	23.93776	1.24E-126	1.94E-125	cyan	0.877440671	2.57E-51	GO:0005789	AT5G47180.2		Plant VAMP (vesicle-associated membrane protein) family protein	vesicle-associated membrane protein, putative
Sapur.009G121200.1	39.8428812	5.11907821	0.214906069	23.82007	2.07E-125	3.21E-124	cyan	0.687919657	2.46E-23	GO:0016021,GO:0004674,GO:0030247,GO:0006468,GO:0005524,GO:0004672	AT1G21270.1	WAK2	wall-associated kinase 2	tyrosine kinase domain protein
Sapur.001G108800.1	15.77870256	6.173937059	0.259382899	23.80241	3.15E-125	4.87E-124	cyan	0.799419565	4.00E-36	GO:0046983,GO:0003677	AT2G24840.1	AGL61,DIA	AGAMOUS-like 61	NA
Sapur.019G065400.1	835.9216857	1.678489977	0.070524162	23.80021	3.32E-125	5.13E-124	cyan	0.823782791	4.82E-40	GO:0055114,GO:0020037,GO:0016705,GO:0005506	AT5G24910.1	CYP714A1	cytochrome P450, family 714, subfamily A, polypeptide 1	cytochrome P450 family protein
Sapur.014G130700.1	275.0274493	1.104999943	0.046557988	23.73384	1.61E-124	2.47E-123	cyan	0.846010834	3.48E-44		AT5G35690.1			ubiquitin and WLM domain protein
Sapur.016G115100.1	45.66292838	3.867563017	0.163034472	23.72236	2.12E-124	3.24E-123	cyan	0.640171541	1.77E-19	GO:0015743	AT1G08440.1		Aluminium activated malate transporter family protein	aluminum activated malate transporter
Sapur.009G000600.1	379.7714154	0.771686816	0.032602066	23.66987	7.37E-124	1.12E-122	cyan	0.873945162	1.98E-50		AT1G08480.1			NA
Sapur.016G257900.1	348.5580337	1.369336468	0.057866909	23.66355	8.56E-124	1.30E-122	cyan	0.814719141	1.62E-38		AT1G53040.2		Protein of unknown function (DUF616)	DUF616 family protein
Sapur.016G103400.1	33.03730119	6.017064586	0.25427571	23.66354	8.56E-124	1.30E-122	cyan	0.595087764	2.06E-16	GO:0005515	AT2G29420.1	ATGSTU7,GST25,GSTU7	glutathione S-transferase tau 7	tau class glutathione transferase GSTU37
Sapur.15WG049100.1	10.6522641	6.781982529	0.286769632	23.64958	1.19E-123	1.81E-122	cyan	0.67413956	3.78E-22	GO:0016844,GO:0009058	AT1G74000.1	SS3	strictosidine synthase 3	NA
Sapur.003G091500.1	180.413933	2.471592715	0.10454174	23.64216	1.42E-123	2.15E-122	cyan	0.782135942	1.17E-33		AT4G05030.1		Copper transport protein family	hypothetical protein
Sapur.009G023600.1	988.7643584	0.996361898	0.042157418	23.63432	1.71E-123	2.59E-122	cyan	0.833877738	7.55E-42	GO:0016021,GO:0004252,GO:0006508	AT2G29050.1	ATRBL1,RBL1	RHOMBOID-like 1	rhomboid family protein
Sapur.002G192900.1	1797.490119	1.06220961	0.044989121	23.61037	3.02E-123	4.54E-122	cyan	0.86061869	2.78E-47		AT3G07330.1	ATCSLC06.ATCSLC6.CSLC06	Cellulose-synthase-like C6	cellulose synthase-like C2, glycosyltransferase family 2 protein
Sapur.018G098300.1	287.0887125	0.634370969	0.026872576	23.60663	3.29E-123	4.96E-122	cyan	0.85204789	2.00E-45		AT2G10950.1		BSD domain-containing protein	BSD domain protein, putative
Sapur.018G066700.1	4275.321878	1.283611439	0.054376222	23.60612	3.34E-123	5.02E-122	cyan	0.850222536	4.81E-45	GO:0046872,GO:0000166	AT2G18960.1	AHA1,HA1,OST2,PMA	H(+)-ATPase 1	plasma membrane H+-ATPase
Sapur.005G037200.1	30.26949515	4.153310635	0.176188259	23.57314	7.27E-123	1.09E-121	cyan	0.682224476	7.75E-23		AT3G22490.1		Seed maturation protein	NA
Sapur.010G142100.1	56.39345276	2.042076348	0.086699536	23.55349	1.16E-122	1.73E-121	cyan	0.832416847	1.40E-41		AT2G40410.2		Staphylococcal nuclease homologue	Ca(2+)-dependent nuclease
Sapur.018G091500.1	70.77988714	9.603635457	0.408611529	23.50309	3.79E-122	5.66E-121	cyan	0.60681533	3.65E-17	GO:0005509	AT4G32060.1		calcium-binding EF hand family protein	NA
Sapur.014G072400.1	27.90941617	-2.405290851	0.102634135	-23.4356	1.85E-121	2.75E-120	cyan	-0.809176077	1.26E-37		AT5G41620.1			hypothetical protein
Sapur.004G028800.1	1586.496717	1.212790753	0.051793553	23.41586	2.95E-121	4.36E-120	cyan	0.833743414	8.00E-42	GO:0005524	AT4G04910.1	NSF	AAA-type ATPase family protein	vesicle-fusing ATPase
Sapur.005G135700.1	38.50716063	2.3225566	0.099217365	23.40877	3.48E-121	5.15E-120	cyan	0.814395997	1.82E-38		AT1G77710.1			NA
Sapur.006G003700.1	347.0279594	-1.055996703	0.045118912	-23.4047	3.82E-121	5.65E-120	cyan	-0.862922048	8.40E-48	GO:0005515	AT5G47710.1		Calcium-dependent lipid-binding (CaLB domain) family protein	Arf GTPase activator, putative
Sapur.014G010500.1	7779.449779	1.091828682	0.046678187	23.39055	5.33E-121	7.87E-120	cyan	0.852284063	1.79E-45	GO:0050662,GO:0003824	AT4G24220.1	AWI31,VEP1	NAD(P)-binding Rossmann-fold superfamily protein	progesterone 5 beta-reductase, putative
Sapur.15WG017800.1	531.6113142	1.755427936	0.075158879	23.35623	1.19E-120	1.76E-119	cyan	0.826219933	1.81E-40		AT4G27940.1	ATMTM1,MTM1	manganese tracking factor for mitochondrial SOD2	carrier CGI protein
Sapur.012G032300.1	355.3328991	-1.256501347	0.053858727	-23.3296	2.22E-120	3.27E-119	cyan	-0.872455431	4.63E-50	GO:0006457	AT1G18170.1		FKBP-like peptidyl-prolyl cis-trans isomerase family protein	peptidyl-prolyl cis-trans isomerase
Sapur.010G094500.1	118.281965	2.359264987	0.101212933	23.30992	3.52E-120	5.16E-119	cyan	0.765628898	1.67E-31		AT1G68440.1			transmembrane protein, putative
Sapur.009G011400.1	33.73213541	5.235484518	0.224828994	23.28652	6.07E-120	8.88E-119	cyan	0.675278085	3.04E-22	GO:0015743	AT1G08440.1		Aluminium activated malate transporter family protein	aluminum activated malate transporter
Sapur.T146400.1	29.47068895	-3.139141238	0.135220853	-23.2149	3.22E-119	4.68E-118	cyan	-0.839572033	6.39E-43	GO:0016853,GO:0005975	AT5G57330.1		Galactose mutarotase-like superfamily protein	NA
Sapur.016G170600.1	1397.816191	0.781799144	0.033709627	23.19216	5.46E-119	7.92E-118	cyan	0.84387758	9.26E-44		AT2G14520.1		CBS domain-containing protein with a domain of unknown function (DUF21)	metal transporter CNNM4
Sapur.011G018600.1	1337.723162	-1.08589663	0.046832971	-23.1866	6.22E-119	9.01E-118	cyan	-0.865665481	1.96E-48	GO:0019898,GO:0015979,GO:0009654,GO:0009523,GO:0005509	AT4G05180.1	PSBQ,PSBQ-2,PSII-Q	photosystem II subunit Q-2	oxygen-evolving enhancer protein
Sapur.009G102500.1	731.0582282	0.944914547	0.040754752	23.18538	6.39E-119	9.26E-118	cyan	0.848550477	1.06E-44	GO:0046923,GO:0016021,GO:0006621	AT4G38790.1		ER lumen protein retaining receptor family protein	ER lumen protein retaining receptor, putative
Sapur.003G129800.1	23.89325016	7.539131054	0.325318928	23.17458	8.22E-119	1.19E-117	cyan	0.637348959	2.84E-19	GO:0000160	AT3G04280.3	ARR22,RR22	response regulator 22	response regulators protein
Sapur.016G301300.1	288.8890739	-1.334867378	0.057649818	-23.1548	1.30E-118	1.88E-117	cyan	-0.890337938	7.80E-55		AT3G26710.1	CCB1	cofactor assembly of complex C	transmembrane protein, putative
Sapur.001G182000.1	22.88379147	7.782662266	0.336252929	23.14526	1.62E-118	2.33E-117	cyan	0.66947635	9.23E-22	GO:0055114,GO:0020037,GO:0016705,GO:0005506	AT4G37370.1	CYP81D8	cytochrome P450, family 81, subfamily D, polypeptide 8	NA
Sapur.018G058200.1	2932.409444	1.430843032	0.061906955	23.1128	3.44E-118	4.92E-117	cyan	0.766978138	1.13E-31		AT4G13250.1	NYC1	NAD(P)-binding Rossmann-fold superfamily protein	chlorophyll(ide) B reductase
Sapur.002G025500.1	439.5525639	1.506330631	0.065185554	23.10835	3.82E-118	5.45E-117	cyan	0.864710428	3.26E-48	GO:0016779,GO:0009058	AT1G27680.1	APL2	ADPGLC-PPase large subunit	glucose-1-phosphate adenylyltransferase large protein
Sapur.005G205500.1	456.6648344	-1.27475842	0.055181522	-23.1012	4.50E-118	6.42E-117	cyan	-0.878609159	1.28E-51	GO:0019898,GO:0015979,GO:0009654,GO:0009523,GO:0005509	AT1G76450.1		Photosystem II reaction center PsbP family protein	PsbP domain protein
Sapur.T023100.1	37.22040157	4.30454124	0.186522734	23.07784	7.73E-118	1.10E-116	cyan	0.529273284	1.03E-12	GO:0055114,GO:0020037,GO:0006979,GO:0004601	AT5G24070.1		Peroxidase superfamily protein	NA
Sapur.013G055800.1	87.92480177	1.755992673	0.076121942	23.06815	9.67E-118	1.37E-116	cyan	0.807601773	2.23E-37	GO:0048544,GO:0006468,GO:0005524,GO:0004672	AT5G60900.1	RLK1	receptor-like protein kinase 1	Serine/Threonine kinase domain protein
Sapur.005G134400.1	371.0879984	1.031771509	0.04474089	23.06104	1.14E-117	1.61E-116	cyan	0.843804634	9.57E-44		AT4G08460.2		Protein of unknown function (DUF1644)	DUF1644 family protein
Sapur.006G194100.1	581.7942353	-1.070099021	0.046438638	-23.0433	1.72E-117	2.42E-116	cyan	-0.853182515	1.16E-45	GO:0016987,GO:0006355,GO:0006352,GO:0003700,GO:0003677	AT3G53920.1	SIG3,SIGC	RNApolymerase sigma-subunit C	RNA polymerase sigma factor
Sapur.008G131200.1	50.14302163	1.664510503	0.072278898	23.029	2.39E-117	3.36E-116	cyan	0.862309866	1.16E-47	GO:0046872	AT2G30580.1	DRIP2	DREB2A-interacting protein 2	E3 ubiquitin protein ligase DRIP2, putative
Sapur.005G044800.1	657.6463217	-1.11629872	0.048473652	-23.029	2.39E-117	3.36E-116	cyan	-0.890732928	5.99E-55	GO:0045454,GO:0015035,GO:0006662	AT4G03520.1	ATHM2	Thioredoxin superfamily protein	thioredoxin M-type protein
Sapur.004G162600.1	406.264078	1.046558656	0.04548218	23.0103	3.68E-117	5.16E-116	cyan	0.838156143	1.19E-42	GO:0007034	AT5G22950.1	VPS24.1	SNF7 family protein	charged multivesicular body protein
Sapur.006G178800.1	11268.95135	1.173020572	0.051008954	22.99637	5.07E-117	7.09E-116	cyan	0.876567937	4.30E-51	GO:0016747,GO:0008610,GO:0016020,GO:0006633	AT2G26250.1	FDH,KCS10	3-ketoacyl-CoA synthase 10	3-ketoacyl-CoA synthase
Sapur.004G149200.1	41.21350068	-2.075361635	0.090289143	-22.9857	6.48E-117	9.06E-116	cyan	-0.718512028	3.20E-26	GO:0005515,GO:0006468,GO:0004672	AT2G27060.1		Leucine-rich repeat protein kinase family protein	receptor kinase, putative
Sapur.014G106900.1	451.5517324	-1.023985505	0.044574588	-22.9724	8.80E-117	1.23E-115	cyan	-0.859998218	3.83E-47	GO:0006415,GO:0003747,GO:0016149,GO:0005737	AT3G62910.1	APG3	Peptide chain release factor 1	peptide chain release factor 1
Sapur.003G150600.1	59.71906579	-2.25238888	0.098098969	-22.9604	1.16E-116	1.62E-115	cyan	-0.850446034	4.33E-45	GO:0006457,GO:0003755	AT2G38730.1		Cyclophilin-like peptidyl-prolyl cis-trans isomerase family protein	peptidyl-prolyl cis-trans isomerase, putative
Sapur.006G097300.1	2748.351188	0.911230951	0.039702357	22.95156	1.42E-116	1.98E-115	cyan	0.84345197	1.12E-43		AT3G10050.1	OMR1	L-O-methylthreonine resistant 1	threonine dehydratase biosynthetic protein
Sapur.006G102500.1	848.0339787	-1.287763969	0.056152575	-22.9333	2.16E-116	3.01E-115	cyan	-0.842547364	1.69E-43	GO:0046872,GO:0030001	AT2G36950.1		Heavy metal transport/detoxification superfamily protein	heavy metal-associated domain protein
Sapur.007G090600.1	125.4248115	-1.496491759	0.065305377	-22.9153	3.27E-116	4.54E-115	cyan	-0.889599316	1.27E-54	GO:0019898,GO:0015979,GO:0009654,GO:0009523,GO:0005509	AT2G28605.1		Photosystem II reaction center PsbP family protein	PsbP domain protein
Sapur.010G005300.1	226.7331619	1.676753679	0.073363354	22.85547	1.29E-115	1.78E-114	cyan	0.770038499	4.62E-32		AT3G06780.1		glycine-rich protein	hypothetical protein
Sapur.006G192700.1	525.6232147	1.149977147	0.050327278	22.84998	1.46E-115	2.01E-114	cyan	0.831500161	2.06E-41		AT1G55340.2		Protein of unknown function (DUF1639)	DUF1639 family protein
Sapur.003G036400.1	83.50518382	-2.319521425	0.101518895	-22.8482	1.52E-115	2.10E-114	cyan	-0.864699199	3.28E-48		AT5G40330.1	ATMYB23.ATMYBRTF.MYB2	myb domain protein 23	Myb-like transcription factor 2, putative
Sapur.016G297200.1	9.231227073	3.67063629	0.160908473	22.81195	3.49E-115	4.79E-114	cyan	0.854858062	5.08E-46	GO:0005515	AT3G06240.1		F-box family protein	F-box protein interaction domain protein
Sapur.002G049700.1	313.7912677	1.375575696	0.060309506	22.80861	3.77E-115	5.16E-114	cyan	0.839653377	6.17E-43		AT1G71780.1			transmembrane protein, putative
Sapur.003G008500.1	10.25496455	6.621539182	0.290468542	22.79606	5.02E-115	6.87E-114	cyan	0.726253562	5.16E-27					NA
Sapur.006G119700.1	87.98593012	-1.690533249	0.074232707	-22.7734	8.41E-115	1.15E-113	cyan	-0.877745281	2.15E-51		AT5G20540.1	ATBRXL4,BRX-LIKE4,BRXL4	BREVIS RADIX-like 4	brevis radix-like protein
Sapur.001G071100.1	186.1909805	1.520738631	0.066797667	22.76634	9.89E-115	1.35E-113	cyan	0.776966188	5.79E-33		AT4G10810.1			hypothetical protein
Sapur.013G026600.1	47.52803842	1.738715704	0.076436817	22.7471	1.53E-114	2.09E-113	cyan	0.844710406	6.33E-44					NA
Sapur.002G026100.1	110.1684196	1.429800983	0.062885448	22.7366	1.95E-114	2.65E-113	cyan	0.844845492	5.95E-44	GO:0016021	AT2G21600.1	ATRER1B,RER1B	endoplasmatic reticulum retrieval protein 1B	RER1A protein
Sapur.016G095400.1	348.2032395	1.665096065	0.073261026	22.72827	2.35E-114	3.20E-113	cyan	0.825497361	2.43E-40		AT3G54110.1	ATPUMP1.ATUCP1.PUMP1.	plant uncoupling mitochondrial protein 1	uncoupling protein
Sapur.011G050300.1	206.6517338	0.853382389	0.037557958	22.72175	2.73E-114	3.71E-113	cyan	0.869090326	3.04E-49	GO:0043565,GO:0006355,GO:0005634,GO:0003700	AT4G18880.1	AT-HSFA4A,HSF A4A	heat shock transcription factor A4A	heat stress transcription factor A-4a
Sapur.011G006400.1	83.52627446	1.840869359	0.081026821	22.71926	2.89E-114	3.92E-113	cyan	0.76571624	1.63E-31	GO:0006468,GO:0005524,GO:0004672	AT5G55090.1	MAPKKK15	mitogen-activated protein kinase kinase kinase 15	MAP kinase kinase kinase, putative
Sapur.009G121500.1	29.86555389	4.468149317	0.196802063	22.70377	4.11E-114	5.57E-113	cyan	0.695316366	5.32E-24	GO:0016021,GO:0004674,GO:0006468,GO:0005524,GO:0004672	AT1G21270.1	WAK2	wall-associated kinase 2	NA
Sapur.016G125400.1	871.7601764	2.118324802	0.093599378	22.63183	2.11E-113	2.83E-112	cyan	0.819182179	2.94E-39		AT2G31940.1			transmembrane protein, putative
Sapur.009G012500.1	323.0996511	1.364484381	0.060304214	22.62668	2.37E-113	3.18E-112	cyan	0.785950557	3.50E-34	GO:0006468,GO:0004672	AT1G66150.1	TMK1	transmembrane kinase 1	receptor-like kinase, putative
Sapur.019G010700.1	766.8332137	-1.505024991	0.066537677	-22.6191	2.81E-113	3.77E-112	cyan	-0.833701143	8.14E-42	GO:0007165,GO:0005515,GO:0043531	AT5G36930.1		Disease resistance protein (TIR-NBS-LRR class) family	NA
Sapur.013G143000.1	139.5218743	2.93804642	0.130112449	22.58082	6.69E-113	8.93E-112	cyan	0.784452424	5.64E-34	GO:0055114,GO:0020037,GO:0016705,GO:0005506	AT5G24910.1	CYP714A1	cytochrome P450, family 714, subfamily A, polypeptide 1	cytochrome P450 family protein
Sapur.016G277400.1	2364.017906	0.827740387	0.036664589	22.57602	7.46E-113	9.95E-112	cyan	0.872598689	4.26E-50		AT5G54800.1	ATGPT1,GPT1	glucose 6-phosphate/phosphate translocator 1	glucose-6-phosphate/phosphate-translocator
Sapur.003G063000.1	1249.542816	1.554314876	0.068876518	22.56669	9.21E-113	1.23E-111	cyan	0.782187384	1.15E-33		AT5G46910.1		Transcription factor jumonji (jmj) family protein / zinc finger (C5HC2 type) family protein	transcription factor jumonji family protein
Sapur.009G096300.1	11193.64909	1.530691003	0.067858488	22.5571	1.14E-112	1.52E-111	cyan	0.777103825	5.55E-33	GO:0008234,GO:0006508	AT4G39090.1	RD19,RD19A	Papain family cysteine protease	cysteine protease
Sapur.008G133800.1	589.2454767	1.084864296	0.048120086	22.54494	1.51E-112	2.00E-111	cyan	0.840458819	4.31E-43	GO:0008152,GO:0003824	AT4G14440.1	ATECI3,ECI3,HCD1	3-hydroxyacyl-CoA dehydratase 1	enoyl-CoA hydratase/isomerase family protein
Sapur.018G005000.5	12.7946615	2.887385029	0.128187351	22.52473	2.38E-112	3.15E-111	cyan	0.769433303	5.52E-32	GO:0008270,GO:0005515	AT5G24870.2		RING/U-box superfamily protein	zinc finger protein, putative
Sapur.007G040100.1	147.1861176	2.115247084	0.093964925	22.51103	3.24E-112	4.28E-111	cyan	0.810820266	6.90E-38	GO:0055085,GO:0016020,GO:0015297,GO:0015238,GO:0006855	AT1G58340.1	ZF14	MATE efflux family protein	multidrug efflux protein
Sapur.004G128700.1	882.6926775	1.394772584	0.062013808	22.49132	5.05E-112	6.66E-111	cyan	0.798430371	5.62E-36		AT4G38690.1		PLC-like phosphodiesterases superfamily protein	phosphatidylinositol-specific phospholipase C domain protein
Sapur.013G142400.1	302.2004327	1.631076975	0.072552213	22.48142	6.31E-112	8.31E-111	cyan	0.808269001	1.75E-37	GO:0008121	AT5G05370.1		Cytochrome b-c1 complex, subunit 8 protein	ubiquinol-cytochrome C reductase complex ubiquinone-binding protein QP-C protein
Sapur.018G025200.1	67.17596788	2.011139541	0.089457858	22.48142	6.31E-112	8.31E-111	cyan	0.825643698	2.29E-40		AT3G22780.1	ATTSO1,TSO1	Tesmin/TSO1-like CXC domain-containing protein	NA
Sapur.004G073700.1	12084.22199	1.78519268	0.079410913	22.48045	6.45E-112	8.48E-111	cyan	0.765813427	1.58E-31	GO:0030244,GO:0016866	AT5G15650.1	ATRGP2,RGP2	reversibly glycosylated polypeptide 2	alpha-1%2C4-glucan-protein synthase [UDP-forming] protein
Sapur.014G122000.1	151.5400598	1.784547078	0.079485847	22.45113	1.25E-111	1.63E-110	cyan	0.78150263	1.43E-33	GO:0016021,GO:0004190	AT2G43070.1	ATSPPL3,SPPL3	SIGNAL PEPTIDE PEPTIDASE-LIKE 3	signal peptide peptidase-like protein
Sapur.016G242700.1	2616.923098	-1.107159528	0.049390012	-22.4167	2.71E-111	3.53E-110	cyan	-0.867158166	8.75E-49		AT1G17100.1		SOUL heme-binding family protein	SOUL heme-binding family protein
Sapur.008G098200.1	600.5290495	2.324495802	0.103862984	22.38041	6.11E-111	7.94E-110	cyan	0.795759082	1.39E-35		AT1G68440.1			transmembrane protein, putative
Sapur.016G253200.1	279.8744815	-0.874530973	0.039081604	-22.377	6.59E-111	8.56E-110	cyan	-0.876324081	4.97E-51		AT1G53250.1			hypothetical protein
Sapur.T008600.1	11.5246134	4.964073666	0.222512343	22.30921	3.01E-110	3.88E-109	cyan	0.787907796	1.86E-34					NA
Sapur.002G085600.1	441.0835622	1.937488135	0.086860915	22.30564	3.26E-110	4.20E-109	cyan	0.823001123	6.58E-40		AT1G58210.1	EMB1674	kinase interacting family protein	kinase interacting family protein
Sapur.009G047600.1	5994.268467	0.680690937	0.030529365	22.29627	4.02E-110	5.17E-109	cyan	0.886579787	9.14E-54	GO:0046933,GO:0015986	AT5G13450.1	ATP5	delta subunit of Mt ATP synthase	ATP synthase F1, delta subunit
Sapur.010G152600.1	47.79548691	1.120788798	0.050292744	22.2853	5.13E-110	6.60E-109	cyan	0.860119412	3.60E-47		AT3G55730.1	AtMYB109,MYB109	myb domain protein 109	MYB transcription factor
Sapur.005G141500.1	7.721781981	6.123474713	0.274972315	22.26942	7.31E-110	9.39E-109	cyan	0.766301817	1.37E-31		AT1G21722.1			hypothetical protein
Sapur.009G018700.1	728.1925944	1.582992427	0.071087901	22.2681	7.53E-110	9.67E-109	cyan	0.783882735	6.75E-34	GO:0008017,GO:0007018,GO:0005524,GO:0003777,GO:0005871	AT3G45850.1		P-loop containing nucleoside triphosphate hydrolases superfamily protein	kinesin motor domain protein
Sapur.007G124500.1	572.4160577	2.588589828	0.116371583	22.24417	1.28E-109	1.65E-108	cyan	0.755838784	2.62E-30	GO:0045017,GO:0004144	AT3G49190.1		O-acyltransferase (WSD1-like) family protein	O-acyltransferase WSD1
Sapur.010G081800.1	75.94756913	-1.098245476	0.049400274	-22.2316	1.70E-109	2.18E-108	cyan	-0.861983901	1.37E-47		AT2G01620.1	MEE11	RNI-like superfamily protein	F-box protein SKIP28
Sapur.003G156100.1	363.0681414	-1.390209525	0.062631617	-22.1966	3.70E-109	4.72E-108	cyan	-0.872557789	4.36E-50		AT4G20740.1		Pentatricopeptide repeat (PPR-like) superfamily protein	PPR containing plant protein
Sapur.005G176600.1	414.6701984	-0.861061793	0.038797015	-22.194	3.92E-109	5.00E-108	cyan	-0.888223622	3.15E-54		AT4G14420.1		HR-like lesion-inducing protein-related	HR-like lesion-inducing protein
Sapur.018G006100.1	12.79673061	6.94598014	0.313103143	22.18432	4.87E-109	6.20E-108	cyan	0.65493071	1.35E-20		AT4G31470.1		CAP (Cysteine-rich secretory proteins, Antigen 5, and Pathogenesis-related 1 protein) superfamily protein	cysteine-rich secretory family protein
Sapur.003G014800.1	475.8754227	1.31028676	0.059074	22.18043	5.31E-109	6.75E-108	cyan	0.790874358	7.09E-35	GO:0003333	AT1G80510.1		Transmembrane amino acid transporter family protein	amino acid transporter
Sapur.002G185700.1	610.4683859	1.064489688	0.048037776	22.15943	8.46E-109	1.07E-107	cyan	0.850415265	4.39E-45		AT5G48370.2		Thioesterase/thiol ester dehydrase-isomerase superfamily protein	acyl-CoA thioesterase
Sapur.004G119100.4	24.30563206	3.523833827	0.159036508	22.15739	8.85E-109	1.12E-107	cyan	0.814751163	1.60E-38		AT4G39140.4		RING/U-box superfamily protein	mandelonitrile lyase
Sapur.014G064000.1	261.8278488	0.838212268	0.037841482	22.15062	1.03E-108	1.30E-107	cyan	0.845348114	4.72E-44	GO:0005515	AT3G61350.1	SKIP4	SKP1 interacting partner 4	F-box/kelch-repeat protein SKIP4
Sapur.004G025600.1	329.9709981	1.242804515	0.056123683	22.14403	1.19E-108	1.51E-107	cyan	0.826144729	1.87E-40	GO:0006355,GO:0003677	AT2G37590.1	ATDOF2.4,DOF2.4	DNA binding with one finger 2.4	Dof zinc finger protein
Sapur.012G000600.1	33.62754357	1.940750878	0.087783368	22.10841	2.62E-108	3.31E-107	cyan	0.840531596	4.18E-43		AT5G24620.1		Pathogenesis-related thaumatin superfamily protein	thaumatin-like protein
Sapur.016G017200.1	626.3568384	2.022732118	0.09151283	22.10326	2.94E-108	3.71E-107	cyan	0.812088944	4.32E-38	GO:0016758,GO:0008152	AT1G22380.1	AtUGT85A3,UGT85A3	UDP-glucosyl transferase 85A3	NA
Sapur.002G202100.2	19.62492738	-2.243751912	0.101542899	-22.0966	3.41E-108	4.30E-107	cyan	-0.861545297	1.72E-47	GO:0005737,GO:0005094	AT3G07880.1	SCN1	Immunoglobulin E-set superfamily protein	Rho GDP-dissociation inhibitor, putative
Sapur.012G096900.1	105.0751498	1.508865495	0.068304401	22.09031	3.92E-108	4.93E-107	cyan	0.829722581	4.32E-41	GO:0055114,GO:0016491,GO:0005507	AT4G25240.1	SKS1	SKU5 similar 1	multi-copper oxidase-like protein
Sapur.012G095100.1	9.493352661	6.40369782	0.289968142	22.08414	4.49E-108	5.65E-107	cyan	0.716137326	5.52E-26					NA
Sapur.014G042400.1	104.2288476	-1.398649557	0.063339435	-22.0818	4.73E-108	5.95E-107	cyan	-0.856314317	2.47E-46	GO:0006355,GO:0003700	AT2G44940.1		Integrase-type DNA-binding superfamily protein	AP2/ERF domain transcription factor
Sapur.017G053300.1	145.548095	1.851766495	0.08386558	22.08017	4.90E-108	6.16E-107	cyan	0.771849389	2.70E-32	GO:0006355,GO:0003677	AT5G14000.1	anac084,NAC084	NAC domain containing protein 84	NAC transcription factor protein
Sapur.001G074000.1	61.09763478	1.624088075	0.073598237	22.06694	6.57E-108	8.25E-107	cyan	0.838532401	1.01E-42	GO:0006629	AT4G10955.2		alpha/beta-Hydrolases superfamily protein	triacylglycerol lipase
Sapur.007G122300.1	10.48168991	5.644083984	0.255951508	22.05138	9.27E-108	1.16E-106	cyan	0.834080457	6.93E-42		AT2G20562.1			transmembrane protein, putative
Sapur.T012800.1	1491.224629	-0.787183535	0.035702317	-22.0485	9.87E-108	1.24E-106	cyan	-0.872985404	3.42E-50		AT1G61730.1		DNA-binding storekeeper protein-related transcriptional regulator	transcription regulator, putative
Sapur.012G051600.1	98.35749805	-1.920742635	0.087239827	-22.0168	1.99E-107	2.49E-106	cyan	-0.850089616	5.13E-45	GO:0016020,GO:0006810,GO:0005215	AT5G62680.1		Major facilitator superfamily protein	nitrate transporter, putative
Sapur.011G022000.1	27.82993918	2.720198324	0.123558906	22.0154	2.05E-107	2.56E-106	cyan	0.718893425	2.93E-26	GO:0048544,GO:0006468,GO:0004672	AT1G11340.1		S-locus lectin protein kinase family protein	Serine/Threonine-kinase
Sapur.008G116300.1	707.0337372	0.784846117	0.035671738	22.0019	2.76E-107	3.45E-106	cyan	0.83913754	7.74E-43		AT2G02180.1	TOM3	tobamovirus multiplication protein 3	tobamovirus multiplication-like protein
Sapur.016G002700.1	370.568548	-0.839562078	0.038165435	-21.998	3.01E-107	3.76E-106	cyan	-0.869273805	2.75E-49	GO:0006351,GO:0003899	AT4G15950.1	NRPD4,NRPE4,RDM2	RNA polymerase II, Rpb4, core protein	hypothetical protein
Sapur.016G012200.1	20.91040043	-2.397730248	0.109130513	-21.9712	5.43E-107	6.76E-106	cyan	-0.84496982	5.62E-44	GO:0009055	AT2G32300.1	UCC1	uclacyanin 1	cucumber peeling cupredoxin, putative
Sapur.011G063900.1	30.88383285	3.298282869	0.150221234	21.95617	7.56E-107	9.38E-106	cyan	0.759033299	1.08E-30	GO:0055114,GO:0016491,GO:0008270	AT5G63620.2		GroES-like zinc-binding alcohol dehydrogenase family protein	NA
Sapur.001G120500.1	457.5090062	1.16855664	0.053280359	21.93222	1.28E-106	1.58E-105	cyan	0.83711687	1.88E-42	GO:0009165,GO:0004749,GO:0000287	AT2G35390.2		Phosphoribosyltransferase family protein	ribose-phosphate pyrophosphokinase
Sapur.001G125100.1	336.3185157	1.76652363	0.080556018	21.92913	1.37E-106	1.69E-105	cyan	0.704660867	7.20E-25		AT2G44730.1		Alcohol dehydrogenase transcription factor Myb/SANT-like family protein	alcohol dehydrogenase transcription factor Myb/SANT-like protein
Sapur.008G061600.1	1273.756572	0.887457258	0.040524461	21.8993	2.64E-106	3.25E-105	cyan	0.832597304	1.30E-41	GO:0016567,GO:0004842	AT2G31510.1	ARI7,ATARI7	IBR domain-containing protein	E3 ubiquitin-protein ligase ARI8, putative
Sapur.001G019900.1	1903.583681	2.77866967	0.126973254	21.8839	3.70E-106	4.56E-105	cyan	0.781444538	1.45E-33	GO:0016787	AT5G50400.1	ATPAP27,PAP27	purple acid phosphatase 27	nucleotide pyrophosphatase/phosphodiesterase
Sapur.002G051100.1	809.2464181	0.891022452	0.040751846	21.86459	5.65E-106	6.94E-105	cyan	0.834202451	6.58E-42		AT4G09830.1		Uncharacterised conserved protein UCP009193	holocarboxylase synthetase
Sapur.003G144600.1	539.513338	2.232149245	0.10218886	21.84337	8.99E-106	1.10E-104	cyan	0.780416838	2.00E-33		AT5G07010.1	ATST2A,ST2A	sulfotransferase 2A	flavonol 4'-sulfotransferase, putative
Sapur.15WG064600.1	7.409524627	-4.71797431	0.216019643	-21.8405	9.57E-106	1.17E-104	cyan	-0.821174572	1.35E-39		AT5G07890.3		myosin heavy chain-related	hypothetical protein
Sapur.007G033600.1	14.09162606	-2.693150427	0.123524026	-21.8026	2.19E-105	2.68E-104	cyan	-0.843999792	8.76E-44					NA
Sapur.002G148300.1	168.0817685	2.960791416	0.135925498	21.78246	3.40E-105	4.15E-104	cyan	0.801974422	1.65E-36	GO:0005515	AT4G01200.1		Calcium-dependent lipid-binding (CaLB domain) family protein	C2 domain protein
Sapur.005G121400.1	426.7827112	1.182434492	0.054319157	21.76828	4.64E-105	5.64E-104	cyan	0.820150689	2.02E-39	GO:0071949	AT1G58440.1	SQE1,XF1	FAD/NAD(P)-binding oxidoreductase family protein	squalene monooxygenase
Sapur.019G071800.1	768.1816777	-1.056042292	0.04852079	-21.7647	5.01E-105	6.09E-104	cyan	-0.867884016	5.89E-49		AT1G71500.1		Rieske (2Fe-2S) domain-containing protein	Rieske (2Fe-2S) domain protein
Sapur.005G012900.1	77.90129776	4.52820406	0.208277557	21.7412	8.37E-105	1.01E-103	cyan	0.677300495	2.05E-22	GO:0048544,GO:0006468,GO:0005524,GO:0004672	AT4G21390.1	B120	S-locus lectin protein kinase family protein	Serine/Threonine-kinase
Sapur.014G099200.1	156.3440671	-1.435666566	0.06606758	-21.7303	1.06E-104	1.29E-103	cyan	-0.884188987	4.18E-53		AT1G77670.1		Pyridoxal phosphate (PLP)-dependent transferases superfamily protein	aminotransferase class I and protein II
Sapur.001G102900.1	185.1502541	2.889090779	0.133043408	21.7154	1.47E-104	1.77E-103	cyan	0.831250498	2.29E-41	GO:0016020,GO:0006810,GO:0005215	AT3G47440.1	TIP5;1	tonoplast intrinsic protein 5;1	NA
Sapur.009G122100.1	81.22231769	1.832680423	0.084479503	21.69379	2.35E-104	2.83E-103	cyan	0.765942869	1.52E-31		AT5G06570.2		alpha/beta-Hydrolases superfamily protein	gibberellin receptor GID1, putative
Sapur.016G082900.1	838.8172944	1.141597611	0.052634505	21.68915	2.60E-104	3.13E-103	cyan	0.802350184	1.44E-36		AT2G37110.1		PLAC8 family protein	cell number regulator
Sapur.004G051600.2	12.89022371	5.505890736	0.25403028	21.67415	3.60E-104	4.32E-103	cyan	0.54641762	1.34E-13		AT3G48950.1		Pectin lyase-like superfamily protein	polygalacturonase-like protein
Sapur.010G099500.1	257.9372003	-1.215224289	0.056118067	-21.6548	5.48E-104	6.57E-103	cyan	-0.865321665	2.35E-48	GO:0016853,GO:0009507	AT3G25780.1	AOC3	allene oxide cyclase 3	allene oxide cyclase
Sapur.014G143300.1	67.14424896	1.476546351	0.068240153	21.6375	7.97E-104	9.51E-103	cyan	0.830235362	3.49E-41		AT4G08330.1			NA
Sapur.014G121800.1	210.8033812	2.989620333	0.138223857	21.62883	9.62E-104	1.15E-102	cyan	0.744928764	4.85E-29		AT2G43060.1	IBH1	ILI1 binding bHLH 1	transcription factor, putative
Sapur.006G224400.1	12.6712377	2.860965214	0.132372401	21.613	1.36E-103	1.61E-102	cyan	0.811749937	4.90E-38	GO:0008270,GO:0005515	AT5G24870.2		RING/U-box superfamily protein	zinc finger protein
Sapur.004G037100.1	85.57958399	2.62715282	0.121657835	21.5946	2.02E-103	2.40E-102	cyan	0.774592127	1.19E-32	GO:0016788	AT5G45920.1		SGNH hydrolase-type esterase superfamily protein	GDSL esterase/lipase
Sapur.006G156900.1	282.4915744	1.032932788	0.047848651	21.5875	2.35E-103	2.80E-102	cyan	0.848295411	1.20E-44	GO:0003824,GO:0006470,GO:0004722	AT5G06750.1		Protein phosphatase 2C family protein	protein phosphatase 2C
Sapur.019G059400.1	105.4780495	1.783764245	0.082750895	21.55583	4.67E-103	5.53E-102	cyan	0.810906105	6.69E-38	GO:0005515,GO:0006468,GO:0004672	AT3G14840.2		Leucine-rich repeat transmembrane protein kinase	receptor-like kinase, putative
Sapur.002G107400.1	34.31809959	1.969508904	0.091376906	21.55368	4.89E-103	5.79E-102	cyan	0.776829204	6.04E-33	GO:0016567,GO:0004842	AT1G65430.1	ARI8,ATARI8	IBR domain-containing protein	E3 ubiquitin-protein ligase ARI5, putative
Sapur.012G063300.1	67.16421536	2.708869465	0.125685681	21.55273	4.99E-103	5.91E-102	cyan	0.630988808	8.17E-19	GO:0005515	AT3G48240.1		Octicosapeptide/Phox/Bem1p family protein	NA
Sapur.018G029300.1	252.730618	1.455467068	0.067547316	21.54737	5.60E-103	6.62E-102	cyan	0.781962399	1.24E-33		AT1G18950.1		DDT domain superfamily	hypothetical protein
Sapur.016G222400.1	205.2297538	1.292547767	0.060026254	21.53304	7.64E-103	9.01E-102	cyan	0.853764721	8.70E-46	GO:0005515,GO:0006468,GO:0004672	AT3G28450.1		Leucine-rich repeat protein kinase family protein	LRR receptor-like kinase, putative
Sapur.001G029800.1	139.8405013	-1.437448543	0.066758997	-21.5319	7.82E-103	9.23E-102	cyan	-0.849980154	5.40E-45	GO:0005524	AT1G73110.1		P-loop containing nucleoside triphosphate hydrolases superfamily protein	ribulose bisphosphate carboxylase/oxygenase activase
Sapur.002G007500.1	154.8729146	2.231660462	0.103667534	21.52709	8.68E-103	1.02E-101	cyan	0.721814691	1.48E-26		AT1G76250.1			hypothetical protein
Sapur.005G150300.1	1719.715414	0.858831105	0.039895846	21.52683	8.73E-103	1.03E-101	cyan	0.866706817	1.12E-48	GO:0008121,GO:0006122	AT1G15120.1		Ubiquinol-cytochrome C reductase hinge protein	cytochrome B-c1 complex subunit 6
Sapur.016G124000.1	117.9744475	-1.442964697	0.067035819	-21.5253	9.03E-103	1.06E-101	cyan	-0.894804098	3.71E-56	GO:0043565,GO:0006355,GO:0003700,GO:0003677	AT5G06710.1	HAT14	homeobox from Arabidopsis thaliana	homeobox leucine zipper protein
Sapur.011G054000.1	782.2305144	-1.082022238	0.050365571	-21.4834	2.23E-102	2.62E-101	cyan	-0.847730343	1.56E-44					DhnA-type fructose-1%2C6-bisphosphate aldolase-like enzyme
Sapur.009G037900.1	396.1789237	-0.839404948	0.039075371	-21.4817	2.31E-102	2.71E-101	cyan	-0.890287075	8.07E-55	GO:0055114,GO:0016491	AT1G50450.1		Saccharopine dehydrogenase	saccharopine dehydrogenase
Sapur.016G063500.1	10.70610228	4.162742354	0.193825451	21.47676	2.57E-102	3.01E-101	cyan	0.777195095	5.40E-33	GO:0046983	AT2G31210.1		basic helix-loop-helix (bHLH) DNA-binding superfamily protein	transcription factor bHLH91
Sapur.005G091800.1	141.5081004	-1.201613621	0.055976494	-21.4664	3.21E-102	3.76E-101	cyan	-0.873022267	3.35E-50		AT2G17972.1			transmembrane protein, putative
Sapur.15WG082300.1	169.0654445	1.601134289	0.074593148	21.4649	3.31E-102	3.88E-101	cyan	0.827350716	1.14E-40	GO:0016887,GO:0005524,GO:0016021,GO:0006810,GO:0005215	AT3G47730.1	ABCA2,ATATH1,ATH1	ATP-binding cassette A2	ABC transporter A family protein
Sapur.007G029100.1	2668.53286	1.153112195	0.05374177	21.45654	3.97E-102	4.64E-101	cyan	0.827374982	1.13E-40		AT2G23790.1		Protein of unknown function (DUF607)	coiled-coil protein 109A
Sapur.002G114300.1	448.0594367	1.286348431	0.060027343	21.42937	7.11E-102	8.30E-101	cyan	0.786496542	2.94E-34		AT4G17080.1		Histone H3 K4-specific methyltransferase SET7/9 family protein	1-phosphatidylinositol-4-phosphate 5-kinase, putative
Sapur.010G186500.1	82.41334685	-1.107935099	0.0517095	-21.4261	7.62E-102	8.89E-101	cyan	-0.868312354	4.66E-49		AT5G04520.1		Protein of unknown function DUF455	oxidoreductase/transition metal ion-binding protein
Sapur.15WG006700.1	137.9452697	1.036016277	0.048376686	21.41561	9.56E-102	1.11E-100	cyan	0.822208538	9.00E-40	GO:0005515	AT5G45360.1		F-box family protein	F-box-like protein
Sapur.013G040900.1	233.2311603	-1.108676481	0.051799289	-21.4033	1.24E-101	1.45E-100	cyan	-0.857439525	1.41E-46		AT3G04590.2		AT hook motif DNA-binding family protein	SAP1 protein, putative
Sapur.009G052300.1	349.2894763	1.890501021	0.088335028	21.40149	1.29E-101	1.50E-100	cyan	0.810455509	7.89E-38	GO:0009055	AT1G08500.1	AtENODL18,ENODL18	early nodulin-like protein 18	early nodulin-like protein, putative
Sapur.006G079500.1	81.40877665	-1.390753186	0.064989671	-21.3996	1.35E-101	1.57E-100	cyan	-0.868391327	4.46E-49		AT3G09050.1			transmembrane protein, putative
Sapur.001G064300.1	125.9483459	-2.606087214	0.121901028	-21.3787	2.11E-101	2.45E-100	cyan	-0.820115568	2.04E-39	GO:0006355,GO:0003700	AT2G44840.1	ATERF13,EREBP,ERF13	ethylene-responsive element binding factor 13	ethylene-responsive transcription factor 2
Sapur.011G045800.1	21.75999756	1.986133396	0.093000484	21.35616	3.42E-101	3.95E-100	cyan	0.825332614	2.59E-40	GO:0006355,GO:0003677	AT1G29160.1		Dof-type zinc finger DNA-binding family protein	Dof zinc finger protein
Sapur.019G033000.1	968.6939769	1.522741755	0.071312397	21.35311	3.65E-101	4.21E-100	cyan	0.789507862	1.11E-34	GO:0016020,GO:0006810,GO:0005215	AT3G04090.1	SIP1;1,SIP1A	small and basic intrinsic protein 1A	aquaporin, MIP family%2C SIP subfamily protein
Sapur.007G090800.1	26.99916563	7.051423759	0.330406761	21.34164	4.66E-101	5.38E-100	cyan	0.607858062	3.12E-17		AT2G04220.1		Plant protein of unknown function (DUF868)	DUF868 family protein
Sapur.005G137100.1	9.382187402	5.682364234	0.266328261	21.33594	5.27E-101	6.07E-100	cyan	0.751927327	7.59E-30	GO:0055085,GO:0016021	AT3G54700.1	PHT1;7	phosphate transporter 1;7	inorganic phosphate transporter, putative
Sapur.008G110300.1	588.9939478	0.720019157	0.033770221	21.32113	7.23E-101	8.33E-100	cyan	0.846255279	3.11E-44		AT1G14820.3		Sec14p-like phosphatidylinositol transfer family protein	aspartate semialdehyde dehydrogenase, putative
Sapur.009G027400.1	506.8221745	-1.025652139	0.048117321	-21.3157	8.13E-101	9.34E-100	cyan	-0.875506673	8.01E-51	GO:0055114,GO:0016491,GO:0006633,GO:0005506	AT1G07420.1	ATSMO2,SMO2-1	sterol 4-alpha-methyl-oxidase 2-1	C-4 methylsterol oxidase
Sapur.007G112500.1	586.4860136	0.78911862	0.037050771	21.2983	1.18E-100	1.35E-99	cyan	0.863806984	5.27E-48	GO:0055085,GO:0022857,GO:0016021	AT2G43330.1	ATINT1,INT1	inositol transporter 1	sugar porter (SP) family MFS transporter
Sapur.007G034700.1	29.24071939	5.175324107	0.243030885	21.29492	1.27E-100	1.45E-99	cyan	0.622534792	3.20E-18		AT2G23620.1	ATMES1,MES1	methyl esterase 1	NA
Sapur.004G033600.1	143.797753	2.240267796	0.105228075	21.28964	1.42E-100	1.62E-99	cyan	0.767881944	8.69E-32	GO:0045454,GO:0015035,GO:0009055	AT1G28480.1	GRX480,roxy19	Thioredoxin superfamily protein	glutaredoxin
Sapur.008G067900.1	23.43894817	-1.47878893	0.069497761	-21.2782	1.81E-100	2.07E-99	cyan	-0.854527246	5.98E-46		AT1G69588.1	CLE45	CLAVATA3/ESR-RELATED 45	CLAVATA 3/ESR (CLE)-related protein, putative
Sapur.006G180500.1	528.9492286	-0.803727129	0.037783779	-21.2718	2.07E-100	2.37E-99	cyan	-0.85418128	7.09E-46	GO:0031683,GO:0019001,GO:0007186,GO:0004871,GO:0003924	AT2G26300.1	ATGPA1,GP ALPHA 1,GPA1	G protein alpha subunit 1	heterotrimeric GTP-binding protein alpha subunit, putative
Sapur.014G110000.1	172.741241	1.281929631	0.060273984	21.26837	2.23E-100	2.55E-99	cyan	0.787245431	2.31E-34		AT2G03430.1		Ankyrin repeat family protein	ankyrin-like protein
Sapur.005G029400.1	64.7250658	1.636477708	0.076962225	21.26339	2.47E-100	2.83E-99	cyan	0.810911148	6.67E-38	GO:0008270,GO:0005515	AT3G05200.1	ATL6	RING/U-box superfamily protein	RING finger protein
Sapur.016G098400.1	6289.572932	1.884227985	0.088621037	21.26163	2.57E-100	2.94E-99	cyan	0.73577196	5.02E-28	GO:0016746,GO:0008152	AT2G38110.1	ATGPAT6,GPAT6	glycerol-3-phosphate acyltransferase 6	glycerol-3-phosphate acyltransferase
Sapur.001G166800.1	229.4279568	1.57640875	0.074158897	21.25718	2.83E-100	3.23E-99	cyan	0.787638841	2.03E-34		AT1G72790.1		hydroxyproline-rich glycoprotein family protein	transmembrane protein, putative

Sapur.016G246500.1	9.712559726	2.775857069	0.1306563	21.24549	3.63E-100	4.14E-99	cyan	0.822616893	7.66E-40	GO:0008270,GO:0005515	AT1G53490.1		RING/U-box superfamily protein	HEI10
Sapur.001G155400.1	11.55476752	2.921858322	0.137550005	21.24215	3.90E-100	4.44E-99	cyan	0.796096804	1.24E-35	GO:0005515	AT1G15670.1		Galactose oxidase/kelch repeat superfamily protein	kelch repeat F-box protein
Sapur.012G073400.1	277.8942215	-1.464583882	0.068970555	-21.2349	4.55E-100	5.18E-99	cyan	-0.888706262	2.30E-54	GO:0016787	AT4G24890.1	ATPAP24,PAP24	purple acid phosphatase 24	NA
Sapur.006G116300.1	393.5141765	1.102638228	0.051938047	21.22987	5.06E-100	5.76E-99	cyan	0.800799032	2.48E-36	GO:0035434,GO:0016021,GO:0005375	AT5G20650.1	COPT5	copper transporter 5	copper transporter
Sapur.014G029200.1	37.54734428	-1.703155634	0.080237872	-21.2263	5.46E-100	6.21E-99	cyan	-0.848106236	1.31E-44		AT1G47580.1		Pentatricopeptide repeat (PPR) superfamily protein	NA
Sapur.001G141900.1	39.22522025	2.759711201	0.130415871	21.16085	2.19E-99	2.48E-98	cyan	0.726615111	4.73E-27	GO:0055114,GO:0016491	AT1G15550.1	ATGA3OX1,GA3OX1,GA4	gibberellin 3-oxidase 1	gibberellin 3-beta-hydroxylase
Sapur.006G165100.1	630.517684	-0.871787598	0.04119931	-21.1602	2.22E-99	2.52E-98	cyan	-0.868675016	3.82E-49	GO:0003676	AT3G52380.1	CP33,PDE322	chloroplast RNA-binding protein 33	RNA recognition motif (RRM) superfamily protein
Sapur.011G012700.1	264.8291032	0.781189748	0.036922609	21.15749	2.35E-99	2.67E-98	cyan	0.844932146	5.72E-44		AT4G22320.2			hypothetical protein
Sapur.004G127000.1	1264.49641	-0.599173072	0.028322282	-21.1555	2.45E-99	2.78E-98	cyan	-0.86230831	1.16E-47	GO:0008152,GO:0004807	AT2G21170.1	PDTPI,TIM	triosephosphate isomerase	triosephosphate isomerase
Sapur.003G141500.1	3120.011906	0.693449765	0.032779737	21.15483	2.49E-99	2.82E-98	cyan	0.837573088	1.54E-42	GO:0016020	AT1G16180.2		Serinc-domain containing serine and sphingolipid biosynthesis protein	TMS membrane protein/tumor differentially protein
Sapur.004G152100.1	1672.424173	1.405595232	0.066529081	21.12753	4.44E-99	5.01E-98	cyan	0.843858547	9.34E-44	GO:0005975,GO:0004553	AT2G27500.2		Glycosyl hydrolase superfamily protein	glucan endo-1%2C3-beta-glucosidase
Sapur.010G057300.1	1490.686743	0.672255434	0.031825065	21.12346	4.84E-99	5.46E-98	cyan	0.841162566	3.15E-43		AT1G27000.1		Protein of unknown function (DUF1664)	BZIP transcription factor
Sapur.007G090900.1	139.8338672	-1.108235583	0.052481121	-21.1168	5.57E-99	6.27E-98	cyan	-0.858170236	9.72E-47	GO:0006355,GO:0003713,GO:0003677	AT5G09250.1	KIWI	ssDNA-binding transcriptional regulator	RNA polymerase II transcriptional coactivator KIWI protein
Sapur.007G029600.1	877.4417023	2.231361092	0.105858042	21.07881	1.24E-98	1.39E-97	cyan	0.743796285	6.51E-29		AT3G50770.1	CML41	calmodulin-like 41	calmodulin, putative
Sapur.007G090000.1	469.9250605	-0.855507415	0.040586835	-21.0784	1.25E-98	1.40E-97	cyan	-0.871227973	9.25E-50		AT4G33480.1			DUF3727 family protein
Sapur.006G127300.1	735.835351	0.472119273	0.022413597	21.06397	1.70E-98	1.91E-97	cyan	0.856699679	2.04E-46		AT3G57340.2		Heat shock protein DnaJ, N-terminal with domain of unknown function (DUF1977)	chaperone protein DnaJ 49
Sapur.006G076100.1	68.92057061	1.680416316	0.079784476	21.06195	1.78E-98	1.99E-97	cyan	0.801276668	2.10E-36	GO:0006950	AT3G53990.1		Adenine nucleotide alpha hydrolases-like superfamily protein	universal stress family protein
Sapur.014G138500.1	262.1538147	-1.340496054	0.063657034	-21.0581	1.93E-98	2.15E-97	cyan	-0.785719477	3.77E-34	GO:0008270,GO:0005515	AT2G04240.1	XERICO	RING/U-box superfamily protein	RING-H2 zinc finger protein, putative
Sapur.003G035000.1	170.7782376	1.628666285	0.077343557	21.05756	1.95E-98	2.18E-97	cyan	0.763099087	3.44E-31	GO:0008374,GO:0006629	AT5G13640.1	ATPDAT,PDAT,PDAT1	phospholipid:diacylglycerol acyltransferase	NA
Sapur.010G098100.1	534.5140123	1.331455877	0.063233139	21.0563	2.00E-98	2.24E-97	cyan	0.796113294	1.24E-35		AT1G68620.1		alpha/beta-Hydrolases superfamily protein	carboxylesterase
Sapur.007G042600.1	504.3375374	1.399866819	0.066537096	21.03889	2.89E-98	3.22E-97	cyan	0.810478854	7.82E-38	GO:0006468,GO:0005524,GO:0004672	AT4G02630.1		Protein kinase superfamily protein	brassinosteroid insensitive 1-associated receptor kinase, putative
Sapur.002G179600.1	3446.064606	0.894824237	0.042559947	21.02503	3.87E-98	4.31E-97	cyan	0.813728075	2.34E-38	GO:0016021,GO:0004190	AT2G43070.1	ATSPPL3,SPPL3	SIGNAL PEPTIDE PEPTIDASE-LIKE 3	signal peptide peptidase-like protein
Sapur.012G110800.1	608.2111396	-0.707924877	0.033676177	-21.0215	4.17E-98	4.64E-97	cyan	-0.863486731	6.24E-48	GO:0015031,GO:0008565	AT5G52440.1	HCF106	Bacterial sec-independent translocation protein mttA/Hcf106	Sec-independent protein translocase tatA/E-like protein, putative
Sapur.009G121100.1	16.27827362	4.285334976	0.20389194	21.01768	4.52E-98	5.03E-97	cyan	0.728737227	2.84E-27	GO:0030247,GO:0006468,GO:0005524,GO:0004672	AT1G21270.1	WAK2	wall-associated kinase 2	NA
Sapur.008G017600.1	42.01322796	1.65871853	0.079015493	20.99232	7.71E-98	8.55E-97	cyan	0.826046522	1.94E-40	GO:0016758,GO:0008152	AT5G49690.1		UDP-Glycosyltransferase superfamily protein	UDP-glucosyltransferase family protein
Sapur.008G071700.1	354.424065	1.172041604	0.055840718	20.98901	8.26E-98	9.16E-97	cyan	0.799999399	3.27E-36	GO:0006468,GO:0004672	AT1G14000.1	VIK	VH1-interacting kinase	tyrosine kinase domain protein
Sapur.15ZG056400.1	123.073185	1.059249536	0.050502487	20.97421	1.13E-97	1.25E-96	cyan	0.86467968	3.32E-48	GO:0016020,GO:0005509,GO:0004571	AT1G27520.1		Glycosyl hydrolase family 47 protein	NA
Sapur.006G180600.1	278.4946114	1.561367319	0.074501399	20.95756	1.60E-97	1.77E-96	cyan	0.765646591	1.66E-31		AT2G26310.1		Chalcone-flavanone isomerase family protein	NA
Sapur.019G039100.1	2541.039907	1.198857735	0.057264726	20.93536	2.55E-97	2.81E-96	cyan	0.806016334	3.94E-37	GO:0016020,GO:0015171,GO:0003333	AT1G58030.1	CAT2	cationic amino acid transporter 2	cationic amino acid transporter
Sapur.006G149900.1	1354.658942	1.210010568	0.057894561	20.90025	5.33E-97	5.85E-96	cyan	0.782175699	1.16E-33		AT4G30630.1			hypothetical protein
Sapur.016G211200.1	327.3203108	1.510526689	0.072273629	20.90011	5.34E-97	5.86E-96	cyan	0.80666066	3.13E-37		AT5G40720.1		Domain of unknown function (DUF23)	NA
Sapur.017G051900.1	371.9849158	1.152040794	0.055155666	20.88708	7.02E-97	7.69E-96	cyan	0.813775638	2.30E-38		AT1G56060.1			hypothetical protein
Sapur.002G039800.1	57.51787621	-1.655276231	0.079251997	-20.8862	7.14E-97	7.82E-96	cyan	-0.848291831	1.20E-44		AT3G22530.1			hypothetical protein
Sapur.001G134600.1	344.1980609	-0.676720858	0.032406475	-20.8823	7.76E-97	8.49E-96	cyan	-0.877916373	1.94E-51	GO:0044237	AT1G53880.1		Eukaryotic translation initiation factor 2B (eIF-2B) family protein	translation initiation factor eIF-2B subunit alpha
Sapur.002G033500.1	180.4555004	1.177829669	0.056407429	20.88075	8.01E-97	8.76E-96	cyan	0.777089476	5.58E-33		AT1G55190.1	PRA1.F2,PRA7	PRA1 (Prenylated rab acceptor) family protein	PRA1 family protein
Sapur.010G103600.1	11.52232247	3.337901074	0.160022531	20.85894	1.26E-96	1.38E-95	cyan	0.783805605	6.92E-34	GO:0008270,GO:0005515	AT2G04240.2	XERICO	RING/U-box superfamily protein	E3 ubiquitin-protein ligase
Sapur.008G127400.1	1776.164491	0.838961252	0.040249959	20.84378	1.74E-96	1.89E-95	cyan	0.810007968	9.30E-38	GO:0055114,GO:0010309	AT4G14710.2	ATARD2	RmlC-like cupins superfamily protein	1%2C2-dihydroxy-3-keto-5-methylthiopentene dioxygenase
Sapur.004G025100.1	1213.195654	1.080257121	0.051835499	20.8401	1.87E-96	2.04E-95	cyan	0.796878074	9.54E-36		AT1G61100.1		disease resistance protein (TIR class), putative	TIR class disease resistance protein, putative
Sapur.008G148400.1	546.8611198	1.3793079	0.066205807	20.83364	2.15E-96	2.33E-95	cyan	0.81900908	3.14E-39	GO:0005975,GO:0004553	AT1G13130.1		Cellulase (glycosyl hydrolase family 5) protein	glycosyl hydrolase family 5 protein
Sapur.004G132200.1	1406.361679	-1.331182271	0.063914632	-20.8275	2.44E-96	2.65E-95	cyan	-0.806971069	2.80E-37	GO:0055114,GO:0051287,GO:0016616,GO:0008152	AT1G68010.1	ATHPR1,HPR	hydroxypyruvate reductase	peroxisomal hydroxypyruvate reductase
Sapur.003G044000.1	24.5245492	4.354696566	0.209279424	20.80805	3.66E-96	3.97E-95	cyan	0.66257215	3.36E-21		AT4G20040.1		Pectin lyase-like superfamily protein	polygalacturonase QRT3
Sapur.002G163600.1	114.0205272	-1.13455069	0.054542245	-20.8013	4.21E-96	4.56E-95	cyan	-0.846812897	2.40E-44	GO:0043565,GO:0006355,GO:0003700	AT2G34830.1	AtWRKY35.MEE24.WRKY35	WRKY DNA-binding protein 35	WRKY transcription factor
Sapur.013G087200.1	11.46478723	3.126464935	0.150395758	20.78825	5.53E-96	5.98E-95	cyan	0.769676562	5.14E-32					NA
Sapur.008G045700.1	235.1590278	4.250828139	0.20453838	20.78255	6.23E-96	6.73E-95	cyan	0.73953257	1.95E-28	GO:0016746,GO:0008152	AT3G11430.1	ATGPAT5,GPAT5	glycerol-3-phosphate acyltransferase 5	glycerol-3-phosphate acyltransferase
Sapur.007G021400.1	198.5742122	-0.923583008	0.044475185	-20.7663	8.74E-96	9.43E-95	cyan	-0.824401164	3.77E-40		AT1G75240.1	AtHB33,HB33	homeobox protein 33	ZF-HD protein dimerization region protein
Sapur.008G053000.1	2255.128118	-1.132783311	0.054549719	-20.7661	8.77E-96	9.47E-95	cyan	-0.860159514	3.53E-47	GO:0016020,GO:0009765	AT2G40100.1	LHCB4.3	light harvesting complex photosystem II	photosystem I light harvesting complex Lhca2/4, chlorophyll A/B-binding protein
Sapur.001G007700.1	130.3875811	3.309638203	0.159399286	20.76319	9.32E-96	1.00E-94	cyan	0.679272408	1.39E-22	GO:0005515	AT5G07610.1		F-box family protein	F-box protein
Sapur.008G093900.1	38.01858648	-2.110450521	0.101653037	-20.7613	9.69E-96	1.04E-94	cyan	-0.819942956	2.19E-39		AT1G13245.1	DVL4,RTFL17	ROTUNDIFOLIA like 17	DVL5
Sapur.010G110900.1	1562.877457	0.647007523	0.031168019	20.7587	1.02E-95	1.10E-94	cyan	0.851493259	2.62E-45	GO:0007264,GO:0005525	AT4G02080.1	ASAR1.ATSAR2.ATSARA1C.S	secretion-associated RAS super family 2	GTP-binding protein SAR1, putative
Sapur.15WG029900.1	20.68400885	-1.528545688	0.07365887	-20.7517	1.18E-95	1.27E-94	cyan	-0.844607776	6.63E-44		AT3G17950.1			hypothetical protein
Sapur.012G084200.1	632.801606	1.00743221	0.048563421	20.74467	1.37E-95	1.47E-94	cyan	0.811100408	6.22E-38	GO:0016887,GO:0005524,GO:0019538	AT5G51070.1	CLPD,ERD1,SAG15	Clp ATPase	Clp protease ATP-binding subunit
Sapur.010G085800.1	224.2287193	1.266093645	0.061050394	20.7385	1.56E-95	1.68E-94	cyan	0.813174628	2.88E-38		AT1G23040.1		hydroxyproline-rich glycoprotein family protein	transmembrane protein, putative
Sapur.005G027700.1	1309.610121	0.927059836	0.044704631	20.73745	1.59E-95	1.71E-94	cyan	0.831721835	1.88E-41		AT3G05290.1	PNC1	peroxisomal adenine nucleotide carrier 1	peroxisomal adenine nucleotide carrier protein
Sapur.017G094600.1	30150.72169	-1.216109774	0.058650511	-20.7349	1.68E-95	1.81E-94	cyan	-0.835460315	3.84E-42		AT1G67090.1	RBCS1A	ribulose bisphosphate carboxylase small chain 1A	ribulose bisphosphate carboxylase small chain
Sapur.016G214800.1	842.7678258	-0.750336447	0.036203554	-20.7255	2.04E-95	2.19E-94	cyan	-0.861626346	1.65E-47	GO:0003676	AT5G50250.1	CP31B	chloroplast RNA-binding protein 31B	eukaryotic RNA-binding protein RBM24 RNA recognition motif protein
Sapur.008G086300.1	9.293580483	2.794030734	0.135132369	20.67625	5.67E-95	6.07E-94	cyan	0.805855135	4.18E-37		AT5G49340.1	TBL4	TRICHOME BIREFRINGENCE-LIKE 4	Pmr5/Cas1p GDSL/SGNH-like acyl-esterase family protein
Sapur.001G119100.4	15.13614136	-2.928413356	0.141771128	-20.6559	8.64E-95	9.23E-94	cyan	-0.816665366	7.73E-39		AT1G32540.2	LOL1	lsd one like 1	zinc finger, LSD1 subclass protein
Sapur.004G141700.1	114.0402745	-1.267184965	0.061367087	-20.6493	9.91E-95	1.06E-93	cyan	-0.574129428	3.81E-15	GO:0055085,GO:0016020,GO:0006810,GO:0005215	AT2G27810.1	ATNAT12,NAT12	nucleobase-ascorbate transporter 12	nucleobase ascorbate transporter
Sapur.008G105300.1	545.4051244	0.879607405	0.042634744	20.63123	1.44E-94	1.53E-93	cyan	0.835420563	3.90E-42	GO:0016301,GO:0008152,GO:0005524	AT2G01460.1		P-loop containing nucleoside triphosphate hydrolases superfamily protein	P-loop nucleoside triphosphate hydrolase-like protein
Sapur.002G059700.1	80.01059174	6.904665297	0.334838595	20.62088	1.78E-94	1.90E-93	cyan	0.621006171	4.08E-18	GO:0005509,GO:0030247,GO:0006468,GO:0005524,GO:0004672	AT1G21270.1	WAK2	wall-associated kinase 2	tyrosine kinase domain protein
Sapur.006G154500.1	672.8816879	1.730391319	0.083929434	20.61722	1.92E-94	2.04E-93	cyan	0.766801995	1.19E-31	GO:0055085,GO:0016021	AT4G02050.1	STP7	sugar transporter protein 7	hexose transporter, putative
Sapur.014G137500.1	848.4876875	0.969060648	0.047007232	20.61514	2.01E-94	2.13E-93	cyan	0.822231044	8.92E-40		AT5G55850.3	NOI	RPM1-interacting protein 4 (RIN4) family protein	RPM1-interacting protein
Sapur.009G064000.1	1165.845879	-0.943924206	0.045788368	-20.6149	2.02E-94	2.14E-93	cyan	-0.859546669	4.83E-47	GO:0055114,GO:0016491,GO:0016616,GO:0019752	AT2G22780.1	PMDH1	peroxisomal NAD-malate dehydrogenase 1	glyoxysomal malate dehydrogenase
Sapur.003G134700.1	2220.894304	1.044372741	0.050677479	20.60822	2.32E-94	2.46E-93	cyan	0.818637792	3.63E-39	GO:0055114,GO:0016491,GO:0045454	AT3G24170.2	ATGR1,GR1	glutathione-disulfide reductase	glutathione reductase
Sapur.007G002600.1	171.0817844	-1.445084532	0.070155753	-20.5982	2.85E-94	3.02E-93	cyan	-0.813255814	2.80E-38		AT1G78995.1			hypothetical protein
Sapur.012G051400.1	274.8195864	-1.240534267	0.060225769	-20.5981	2.86E-94	3.03E-93	cyan	-0.85559	3.54E-46	GO:0043565,GO:0006355,GO:0003700,GO:0003677	AT3G01470.1	ATHB-1.ATHB1.HAT5.HB-1.I	homeobox 1	homeobox leucine zipper protein
Sapur.007G065600.1	79.31952193	-1.066336617	0.051795179	-20.5876	3.55E-94	3.76E-93	cyan	-0.835331534	4.06E-42		AT1G56170.2	ATHAP5B,HAP5B,NF-YC2	nuclear factor Y, subunit C2	nuclear transcription factor Y subunit C4
Sapur.010G101500.1	98.81157495	-1.478600059	0.071832632	-20.584	3.82E-94	4.05E-93	cyan	-0.849802242	5.88E-45	GO:0016021	AT1G26100.1		Cytochrome b561/ferric reductase transmembrane protein family	cytochrome protein b561, putative
Sapur.010G017900.1	323.9512052	0.887138985	0.04313337	20.56735	5.38E-94	5.69E-93	cyan	0.822230627	8.92E-40		AT3G27320.2		alpha/beta-Hydrolases superfamily protein	carboxylesterase, putative
Sapur.007G052400.1	2035.132508	-1.039395059	0.050593871	-20.5439	8.73E-94	9.20E-93	cyan	-0.867063919	9.21E-49	GO:0055114,GO:0016491	AT1G20020.1	ATLFNR2,FNR2	ferredoxin-NADP(+)-oxidoreductase 2	ferredoxin-NADP reductase
Sapur.010G163900.1	154.7601657	-1.344635751	0.065525538	-20.5208	1.40E-93	1.48E-92	cyan	-0.876123179	5.59E-51	GO:0003676	AT5G59950.3		RNA-binding (RRM/RBD/RNP motifs) family protein	RNA recognition motif (RRM) superfamily protein
Sapur.016G134600.1	1946.314926	0.891774368	0.04346431	20.51739	1.51E-93	1.58E-92	cyan	0.817969591	4.69E-39		AT5G59480.1		Haloacid dehalogenase-like hydrolase (HAD) superfamily protein	haloacid dehalogenase-like hydrolase
Sapur.014G073300.1	90.79383417	1.840086336	0.089714139	20.51055	1.73E-93	1.82E-92	cyan	0.763696329	2.90E-31		AT2G16070.2	PDV2	plastid division2	hypothetical protein
Sapur.008G085400.1	2321.293809	0.722708203	0.035236789	20.51005	1.75E-93	1.84E-92	cyan	0.863159363	7.41E-48	GO:0007264,GO:0005525	AT4G02080.1	ASAR1.ATSAR2.ATSARA1C.S	secretion-associated RAS super family 2	GTP-binding protein Sar1, putative
Sapur.008G097300.1	183.2849961	-0.98026113	0.04780207	-20.5067	1.88E-93	1.97E-92	cyan	-0.845190934	5.08E-44		AT1G13380.1		Protein of unknown function (DUF1218)	fiber protein Fb34
Sapur.019G012300.1	30.62544045	-2.395779024	0.116896577	-20.4949	2.39E-93	2.51E-92	cyan	-0.763178896	3.37E-31	GO:0005524,GO:0007165,GO:0005515	AT5G36930.2		Disease resistance protein (TIR-NBS-LRR class) family	NA
Sapur.008G089100.1	37.13606443	2.006960505	0.097941385	20.49145	2.57E-93	2.69E-92	cyan	0.713038855	1.12E-25		AT3G25597.1			hypothetical protein
Sapur.016G225200.1	387.2559861	-0.658715354	0.032150622	-20.4884	2.73E-93	2.86E-92	cyan	-0.853046855	1.23E-45		AT1G60970.1		SNARE-like superfamily protein	nonclathrin coat protein
Sapur.007G100900.1	809.9731746	-0.813552055	0.039774667	-20.454	5.53E-93	5.78E-92	cyan	-0.854964731	4.82E-46		AT5G09460.1		sequence-specific DNA binding transcription factors;transcription regulators	transcription factor, putative
Sapur.002G098400.1	8.582343865	5.938579644	0.290407695	20.44911	6.12E-93	6.39E-92	cyan	0.647841895	4.72E-20	GO:0055114,GO:0020037,GO:0016705,GO:0005506	AT4G37340.1	CYP81D3	cytochrome P450, family 81, subfamily D, polypeptide 3	cytochrome P450 family protein
Sapur.004G021000.1	815.2989894	-0.844292125	0.041300815	-20.4425	7.00E-93	7.31E-92	cyan	-0.855210218	4.27E-46	GO:0007017,GO:0005875	AT4G27360.1		Dynein light chain type 1 family protein	dynein light chain protein
Sapur.012G105100.1	100.8436285	-1.23419839	0.060387266	-20.4381	7.67E-93	8.00E-92	cyan	-0.856366155	2.41E-46	GO:0006351,GO:0003899,GO:0003677	AT5G51940.1	NRPB6A,NRPD6A,NRPE6A	RNA polymerase Rpb6	NA
Sapur.016G286800.1	45.56105036	6.178624711	0.302569236	20.42053	1.10E-92	1.14E-91	cyan	0.545804966	1.44E-13	GO:0006468,GO:0004672	AT4G21390.1	B120	S-locus lectin protein kinase family protein	Serine/Threonine-kinase
Sapur.018G091200.1	24.25355644	8.058930539	0.394718388	20.41691	1.18E-92	1.23E-91	cyan	0.617749856	6.81E-18		AT4G32060.1		calcium-binding EF hand family protein	NA
Sapur.007G067400.1	312.2785439	1.21201214	0.059455564	20.38518	2.26E-92	2.35E-91	cyan	0.815499493	1.20E-38		AT5G23240.1		DNAJ heat shock N-terminal domain-containing protein	DnaJ heat shock amino-terminal domain protein, putative
Sapur.002G027200.1	6.048061044	5.528730262	0.271216491	20.38493	2.28E-92	2.36E-91	cyan	0.696860255	3.85E-24					transmembrane protein, putative
Sapur.016G111500.1	60.95916606	9.161991153	0.449478014	20.38362	2.34E-92	2.42E-91	cyan	0.623168671	2.90E-18	GO:0055085,GO:0016021,GO:0015299,GO:0006812	AT5G01680.1	ATCHX26,CHX26	cation/H+ exchanger 26	NA
Sapur.002G185300.1	1601.133739	-1.283310876	0.062965528	-20.3812	2.46E-92	2.54E-91	cyan	-0.871864385	6.46E-50	GO:0015979,GO:0009538,GO:0009522	AT4G12800.1	PSAL	photosystem I subunit l	photosystem I reaction center subunit XI
Sapur.010G014000.1	489.0188379	1.38093891	0.06777065	20.37665	2.69E-92	2.79E-91	cyan	0.778369758	3.77E-33	GO:0016772,GO:0016310,GO:0016301,GO:0005524,GO:0050242,GO:0006090	AT4G15530.5	PPDK	pyruvate orthophosphate dikinase	pyruvate orthophosphate dikinase
Sapur.010G110600.1	642.3342448	3.376197594	0.165826398	20.35983	3.80E-92	3.92E-91	cyan	0.590818643	3.79E-16	GO:0005975,GO:0004553	AT5G49360.1	ATBXL1,BXL1	beta-xylosidase 1	alpha-L-arabinofuranosidase/beta-D-xylosidase
Sapur.T157700.1	20.8048123	1.863014325	0.091538709	20.3522	4.44E-92	4.58E-91	cyan	0.817523856	5.56E-39		AT5G08110.1		nucleic acid binding;ATP-dependent helicases;ATP binding;helicases;ATP-dependent helicases	NA
Sapur.004G050900.1	482.837976	-1.153734905	0.056706537	-20.3457	5.07E-92	5.22E-91	cyan	-0.870488092	1.40E-49		AT5G52780.1		Protein of unknown function (DUF3464)	transmembrane protein, putative
Sapur.014G045300.1	19.1004891	3.263945694	0.160431133	20.34484	5.16E-92	5.32E-91	cyan	0.698050354	2.99E-24		AT4G25225.1			DUF4535 domain protein
Sapur.005G090300.1	3457.939576	-0.901402227	0.044315326	-20.3406	5.62E-92	5.79E-91	cyan	-0.874591485	1.36E-50	GO:0055114,GO:0016491	AT5G66190.1	ATLFNR1,FNR1	ferredoxin-NADP(+)-oxidoreductase 1	ferredoxin-NADP reductase
Sapur.001G153500.1	13.1435855	4.603699362	0.226349055	20.33894	5.82E-92	5.99E-91	cyan	0.719672463	2.44E-26		AT1G79960.1	ATOFP14,OFP14	ovate family protein 14	ovate transcriptional repressor
Sapur.014G091600.1	30302.84609	-1.026411821	0.050475817	-20.3347	6.34E-92	6.53E-91	cyan	-0.81855858	3.74E-39	GO:0016020,GO:0009765	AT1G29930.1	AB140.CAB1.CAB140.LHCB1	chlorophyll A/B binding protein 1	photosystem I light harvesting chlorophyll A/B-binding protein
Sapur.016G264700.1	199.2188174	1.502205732	0.073903978	20.32645	7.50E-92	7.72E-91	cyan	0.7958483	1.35E-35	GO:0005515,GO:0043531	AT4G27220.1		NB-ARC domain-containing disease resistance protein	NA
Sapur.017G122900.1	12.31089587	3.230956455	0.158966065	20.32482	7.76E-92	7.98E-91	cyan	0.738353097	2.63E-28	GO:0005515	AT3G28410.1		F-box/RNI-like superfamily protein	F-box/LRR protein, putative
Sapur.013G045900.1	159.5385738	0.951054384	0.046822672	20.31183	1.01E-91	1.04E-90	cyan	0.833974436	7.25E-42		AT5G32470.1		Haem oxygenase-like, multi-helical	heme oxygenase-like, multi-helical protein
Sapur.15WG125900.1	677.2095853	-1.678478299	0.082658846	-20.3061	1.14E-91	1.17E-90	cyan	-0.783944792	6.62E-34		AT5G51010.1		Rubredoxin-like superfamily protein	electron transporter
Sapur.15WG058200.1	292.8525761	-1.204611478	0.059323615	-20.3058	1.14E-91	1.17E-90	cyan	-0.855900267	3.03E-46		AT1G18450.1	ARP4,ATARP4	actin-related protein 4	NA
Sapur.016G261100.1	1063.704828	1.312879209	0.064676295	20.29923	1.31E-91	1.34E-90	cyan	0.777896636	4.36E-33	GO:0005515	AT3G15470.1		Transducin/WD40 repeat-like superfamily protein	WD40 domain protein
Sapur.014G029700.1	229.1200055	-1.018859453	0.050193839	-20.2985	1.33E-91	1.36E-90	cyan	-0.864876891	2.99E-48	GO:0006412,GO:0005840,GO:0003735	AT1G74970.1	RPS9,TWN3	ribosomal protein S9	30S ribosomal protein S9
Sapur.012G080300.1	1765.469138	-1.026125716	0.050558591	-20.2958	1.40E-91	1.44E-90	cyan	-0.838493269	1.03E-42		AT4G25050.1	ACP4	acyl carrier protein 4	acyl carrier protein
Sapur.007G085600.1	259.5427891	0.784931234	0.038683328	20.2912	1.54E-91	1.57E-90	cyan	0.807546185	2.28E-37		AT1G34370.1	STOP1	C2H2 and C2HC zinc fingers superfamily protein	transparent TESTA 1 protein, putative
Sapur.007G023600.1	105.1947283	1.941485861	0.095704896	20.28617	1.70E-91	1.74E-90	cyan	0.75830987	1.32E-30					transmembrane protein, putative
Sapur.004G165100.1	7.00816575	5.986605449	0.295461827	20.26186	2.79E-91	2.85E-90	cyan	0.635723205	3.73E-19	GO:0003723,GO:0003676	AT2G36660.1	PAB7	poly(A) binding protein 7	polyadenylate-binding protein, animal type 1, 2, 3, 4 family protein
Sapur.005G041500.1	236.0034482	-1.043167339	0.051497235	-20.2568	3.10E-91	3.16E-90	cyan	-0.85305195	1.23E-45	GO:0015031,GO:0008565	AT5G28750.1		Bacterial sec-independent translocation protein mttA/Hcf106	twin arginine-targeting protein translocase
Sapur.002G000200.1	222.3165623	0.863144555	0.042645381	20.24005	4.35E-91	4.43E-90	cyan	0.826983571	1.33E-40		AT1G21070.1		Nucleotide-sugar transporter family protein	NA
Sapur.001G020300.1	231.5267897	1.505746628	0.074453567	20.22397	6.02E-91	6.13E-90	cyan	0.765941474	1.52E-31		AT3G13650.1		Disease resistance-responsive (dirigent-like protein) family protein	disease resistance response protein, putative
Sapur.016G239200.1	620.7692249	1.026399735	0.050752772	20.22352	6.08E-91	6.18E-90	cyan	0.811760609	4.88E-38	GO:0055114,GO:0006570,GO:0006559,GO:0004411	AT5G54080.2	HGO	homogentisate 1,2-dioxygenase	homogentisate 1%2C2-dioxygenase
Sapur.15WG071900.1	757.6036997	0.918922039	0.045446048	20.22006	6.52E-91	6.63E-90	cyan	0.81319457	2.86E-38	GO:0008081,GO:0006629,GO:0008889	AT1G74210.1		PLC-like phosphodiesterases superfamily protein	NA
Sapur.005G081500.1	448.2419354	0.732068029	0.036209975	20.2173	6.90E-91	7.00E-90	cyan	0.838013685	1.27E-42		AT4G35510.1			hypothetical protein
Sapur.014G088800.1	72.99031716	2.115284236	0.104645131	20.21388	7.39E-91	7.49E-90	cyan	0.722252323	1.33E-26	GO:0043565,GO:0006355,GO:0003700	AT4G01720.1	AtWRKY47,WRKY47	WRKY family transcription factor	WRKY transcription factor
Sapur.009G132500.1	104.5232406	1.241343592	0.061459454	20.19776	1.02E-90	1.04E-89	cyan	0.798768539	5.00E-36		AT2G17350.1			hypothetical protein
Sapur.001G156600.1	1729.248012	0.544425589	0.026966952	20.18862	1.23E-90	1.25E-89	cyan	0.819945815	2.18E-39	GO:0030170	AT1G80360.1		Pyridoxal phosphate (PLP)-dependent transferases superfamily protein	aspartate aminotransferase
Sapur.006G094000.1	234.4994717	-1.253371746	0.062088384	-20.1869	1.28E-90	1.29E-89	cyan	-0.869930893	1.91E-49		AT3G52870.1		IQ calmodulin-binding motif family protein	calmodulin-binding family protein, putative
Sapur.002G010900.1	8709.993754	-1.083282242	0.053700858	-20.1725	1.71E-90	1.72E-89	cyan	-0.832666302	1.26E-41		AT1G76100.1	PETE1	plastocyanin 1	plastocyanin A/A
Sapur.009G071800.1	172.1356413	-1.820554219	0.090314048	-20.158	2.29E-90	2.30E-89	cyan	-0.863616874	5.83E-48	GO:0005515	AT2G14880.1		SWIB/MDM2 domain superfamily protein	SWIB/MDM2 domain protein
Sapur.012G020500.1	236.9326269	0.796535841	0.039524088	20.15317	2.52E-90	2.54E-89	cyan	0.818580399	3.71E-39		AT5G53330.1		Ubiquitin-associated/translation elongation factor EF1B protein	proline-rich cell wall-like protein, putative
Sapur.013G085100.1	2456.003667	1.258490795	0.062449327	20.15219	2.57E-90	2.59E-89	cyan	0.773498476	1.65E-32	GO:0005524,GO:0051082,GO:0006457	AT1G33360.1		ATP-dependent Clp protease	ATP-dependent Clp protease ATP-binding subunit CLPX protein, putative
Sapur.010G040800.1	437.948603	2.026606676	0.100571596	20.15089	2.64E-90	2.66E-89	cyan	0.77869141	3.41E-33	GO:0055085,GO:0016021	AT2G38940.1	ATPT2,PHT1;4	phosphate transporter 1;4	inorganic phosphate transporter, putative
Sapur.009G073300.1	120.7874943	-2.137894885	0.106099295	-20.1499	2.69E-90	2.71E-89	cyan	-0.804080489	7.85E-37		AT1G07870.2		Protein kinase superfamily protein	transmembrane protein, putative
Sapur.002G071300.1	34.69801346	-1.434049122	0.071213431	-20.1373	3.47E-90	3.49E-89	cyan	-0.833579335	8.57E-42					hypothetical protein
Sapur.012G047100.1	109.0193122	1.976305332	0.098156274	20.13427	3.70E-90	3.71E-89	cyan	0.82131298	1.28E-39	GO:0071805,GO:0016020,GO:0015079	AT2G35060.2	KUP11	K+ uptake permease 11	NA
Sapur.002G074500.1	78.37963581	-1.598221901	0.079411159	-20.1259	4.38E-90	4.38E-89	cyan	-0.843595747	1.05E-43	GO:0005525	AT1G78010.1		tRNA modification GTPase, putative	tRNA modification GTPase MnmE
Sapur.016G279700.1	136.199304	1.948059736	0.096853684	20.11343	5.63E-90	5.63E-89	cyan	0.757549184	1.63E-30					NA
Sapur.004G019000.1	4322.124633	-0.945354942	0.047008305	-20.1104	5.99E-90	5.99E-89	cyan	-0.814461825	1.78E-38	GO:0019898,GO:0015979,GO:0009654,GO:0009523,GO:0005509	AT4G05180.1	PSBQ,PSBQ-2,PSII-Q	photosystem II subunit Q-2	oxygen-evolving enhancer protein
Sapur.T009300.1	12.44238974	3.040625019	0.151219019	20.10742	6.35E-90	6.35E-89	cyan	0.767288214	1.03E-31		AT3G28030.1	UVH3,UVR1	5\'-3\' exonuclease family protein	NA
Sapur.005G094700.1	52.01000991	-1.047629551	0.052105611	-20.1059	6.55E-90	6.54E-89	cyan	-0.847302182	1.91E-44		AT3G51060.1	SRS1,STY1	Lateral root primordium (LRP) protein-related	short internodes protein
Sapur.004G152900.1	364.0652772	0.700608149	0.034874218	20.08957	9.10E-90	9.07E-89	cyan	0.829421396	4.89E-41	GO:0005515	AT1G67190.2		F-box/RNI-like superfamily protein	F-box/LRR protein
Sapur.016G007000.1	124.3859032	-1.119622593	0.055735336	-20.0882	9.36E-90	9.32E-89	cyan	-0.856445803	2.31E-46		AT5G55600.1		agenet domain-containing protein / bromo-adjacent homology (BAH) domain-containing protein	agenet domain protein
Sapur.006G060600.2	19.90802899	-2.544159329	0.126653579	-20.0875	9.48E-90	9.44E-89	cyan	-0.798907841	4.77E-36		AT5G57170.1		Tautomerase/MIF superfamily protein	macrophage migration inhibitory factor-like protein
Sapur.006G227300.1	332.5897803	-0.940189802	0.046820786	-20.0806	1.09E-89	1.08E-88	cyan	-0.836120124	2.89E-42					NA
Sapur.016G048100.1	188.5026795	-1.023275187	0.051010064	-20.0603	1.64E-89	1.63E-88	cyan	-0.839278691	7.28E-43	GO:0048544,GO:0006468,GO:0004672	AT2G41890.1		curculin-like (mannose-binding) lectin family protein / PAN domain-containing protein	G-type lectin S-receptor-like Serine/Threonine-kinase, putative
Sapur.15WG090300.1	554.2281606	-2.467691058	0.123015352	-20.06	1.65E-89	1.64E-88	cyan	-0.798071751	6.35E-36		AT5G14920.1		Gibberellin-regulated family protein	gibberellin-regulated protein, putative
Sapur.017G060600.1	686.7776721	0.821376683	0.04094716	20.05943	1.67E-89	1.66E-88	cyan	0.817334801	5.98E-39	GO:0055114,GO:0016491	AT3G28480.1		Oxoglutarate/iron-dependent oxygenase	prolyl 4-hydroxylase
Sapur.005G143000.1	120.4227582	-0.723016377	0.036048797	-20.0566	1.77E-89	1.75E-88	cyan	-0.861568883	1.70E-47		AT1G77360.1		Tetratricopeptide repeat (TPR)-like superfamily protein	PPR containing plant protein
Sapur.002G072700.1	10.57624518	4.215628189	0.210205622	20.05478	1.83E-89	1.82E-88	cyan	0.719594226	2.49E-26	GO:0016021,GO:0004252,GO:0006508	AT1G77860.1	KOM	Rhomboid-related intramembrane serine protease family protein	NA
Sapur.006G018600.1	146.9394583	1.763751148	0.087970128	20.04943	2.04E-89	2.02E-88	cyan	0.78224309	1.13E-33	GO:0008270	AT5G60580.1		RING/U-box superfamily protein	zinc finger (C3HC4 type RING finger) family protein, putative
Sapur.007G114900.1	79.61304397	-1.489949907	0.074359857	-20.037	2.62E-89	2.60E-88	cyan	-0.851471576	2.65E-45	GO:0006355	AT3G07500.1		Far-red impaired responsive (FAR1) family protein	FAR1 DNA-binding domain protein
Sapur.008G001800.1	162.197104	0.760740972	0.037970526	20.03504	2.73E-89	2.70E-88	cyan	0.827127816	1.25E-40	GO:0005515	AT3G20270.3		lipid-binding serum glycoprotein family protein	lipid-binding serum glycoprotein-like protein
Sapur.009G121400.1	12.32091583	4.502014248	0.224727436	20.03322	2.83E-89	2.80E-88	cyan	0.714525608	7.98E-26	GO:0030247,GO:0006468,GO:0005524,GO:0004672	AT1G21270.1	WAK2	wall-associated kinase 2	NA
Sapur.004G062400.1	239.2109232	-0.680463677	0.033966883	-20.0332	2.83E-89	2.80E-88	cyan	-0.84758796	1.67E-44					hypothetical protein
Sapur.15WG063500.1	52.90236207	-1.159444007	0.057898255	-20.0255	3.30E-89	3.26E-88	cyan	-0.839485998	6.64E-43		AT1G48550.1		Vacuolar protein sorting-associated protein 26	NA
Sapur.009G123500.1	1738.344919	1.247362828	0.062336494	20.01015	4.49E-89	4.43E-88	cyan	0.797749105	7.09E-36		AT3G43720.1		Bifunctional inhibitor/lipid-transfer protein/seed storage 2S albumin superfamily protein	protease inhibitor/seed storage/LTP family protein
Sapur.018G049600.1	37.25501893	-1.750120771	0.087464775	-20.0094	4.56E-89	4.49E-88	cyan	-0.819884001	2.24E-39	GO:0005975,GO:0004553	AT5G56590.1		O-Glycosyl hydrolases family 17 protein	glucan endo-1%2C3-beta-glucosidase
Sapur.017G105500.1	50.40640336	-2.660995902	0.133059073	-19.9986	5.66E-89	5.58E-88	cyan	-0.819266464	2.84E-39					NA
Sapur.006G020700.1	3431.361617	1.394996185	0.069770946	19.99394	6.22E-89	6.12E-88	cyan	0.817566165	5.47E-39		AT2G27030.3	ACAM-2,CAM5	calmodulin 5	calmodulin
Sapur.001G038500.1	668.7411075	0.753812718	0.0377088	19.99037	6.68E-89	6.57E-88	cyan	0.815233684	1.33E-38		AT1G15860.2		Domain of unknown function (DUF298)	defective in cullin neddylation protein, putative
Sapur.006G082300.1	80.87980053	-1.744530681	0.087295822	-19.9841	7.57E-89	7.45E-88	cyan	-0.824465769	3.67E-40		AT3G08920.1		Rhodanese/Cell cycle control phosphatase superfamily protein	rhodanese family protein
Sapur.004G112500.1	10.4573341	-2.519278777	0.126064084	-19.9841	7.57E-89	7.45E-88	cyan	-0.81639441	8.57E-39		AT2G23690.1			DUF4228 domain protein
Sapur.005G199900.1	278.0706863	0.871769905	0.043631928	19.98009	8.21E-89	8.06E-88	cyan	0.84040094	4.43E-43		AT1G76250.1			transmembrane protein, putative
Sapur.003G128300.1	763.807682	0.901664514	0.045154333	19.9685	1.04E-88	1.02E-87	cyan	0.81275551	3.37E-38	GO:0005515	AT1G27340.1		Galactose oxidase/kelch repeat superfamily protein	F-box only protein
Sapur.013G141200.1	88.45533285	-1.327980602	0.066557241	-19.9525	1.43E-88	1.40E-87	cyan	-0.838463536	1.04E-42		AT2G30480.3			hypothetical protein
Sapur.003G094600.1	23.19183198	-2.494547111	0.125085526	-19.9427	1.73E-88	1.70E-87	cyan	-0.812739744	3.39E-38	GO:0008270,GO:0005515	AT3G61460.1	BRH1	brassinosteroid-responsive RING-H2	RING zinc finger protein, putative
Sapur.004G097700.1	7.664887822	-3.040343896	0.152457106	-19.9423	1.75E-88	1.71E-87	cyan	-0.803948381	8.23E-37		AT1G67050.1			NA
Sapur.004G051200.1	18.66711824	3.599012165	0.180558188	19.9327	2.12E-88	2.07E-87	cyan	0.660004758	5.38E-21		AT4G01290.2			NA
Sapur.15WG069600.1	406.2980544	-1.385883681	0.069577728	-19.9185	2.81E-88	2.75E-87	cyan	-0.901609793	2.73E-58		AT5G07960.1			asterix protein
Sapur.003G021200.1	1882.993563	0.740010863	0.037153866	19.91747	2.87E-88	2.80E-87	cyan	0.839691182	6.06E-43		AT1G60970.1		SNARE-like superfamily protein	nonclathrin coat protein
Sapur.004G175100.1	1533.013642	1.284728838	0.064531343	19.9086	3.43E-88	3.34E-87	cyan	0.766023195	1.49E-31	GO:0055085,GO:0016021	AT5G01990.1		Auxin efflux carrier family protein	auxin efflux carrier family transporter
Sapur.018G072000.1	244.1279798	-1.577923913	0.079283638	-19.9023	3.89E-88	3.79E-87	cyan	-0.84421604	7.93E-44		AT3G23760.1			transferring glycosyl group transferase, putative
Sapur.009G086800.1	230.2644292	1.714415899	0.086166845	19.89647	4.37E-88	4.25E-87	cyan	0.758224441	1.36E-30		AT3G50810.1		Uncharacterised protein family (UPF0497)	CASP-like protein
Sapur.003G161300.1	8.583308488	6.399369644	0.321794941	19.88648	5.33E-88	5.18E-87	cyan	0.631898671	7.04E-19					transmembrane protein, putative
Sapur.005G062100.1	206.4698624	-0.909595272	0.045767727	-19.8742	6.81E-88	6.61E-87	cyan	-0.855800426	3.19E-46	GO:0006457	AT4G39710.1	FKBP16-2	FK506-binding protein 16-2	peptidyl-prolyl cis-trans isomerase, FKBP-type protein
Sapur.003G086300.1	1133.370035	0.746244921	0.037573999	19.86067	8.91E-88	8.63E-87	cyan	0.83855584	1.00E-42		AT1G12640.1		MBOAT (membrane bound O-acyl transferase) family protein	membrane bound O-acyl transferase (MBOAT) family protein
Sapur.006G067900.1	1971.837191	0.969065301	0.048802355	19.85694	9.60E-88	9.29E-87	cyan	0.817260607	6.15E-39		AT2G36310.1	URH1	uridine-ribohydrolase 1	inosine-uridine preferring nucleoside hydrolase, putative
Sapur.006G210800.1	992.808165	-0.85552938	0.043089356	-19.8548	1.00E-87	9.69E-87	cyan	-0.863208723	7.22E-48		AT3G19720.1	ARC5,DRP5B	P-loop containing nucleoside triphosphate hydrolases superfamily protein	dynamin-related protein 5B, putative
Sapur.013G105100.1	26.07157233	2.166617665	0.109273431	19.82749	1.72E-87	1.66E-86	cyan	0.749188888	1.58E-29		AT1G65280.1		DNAJ heat shock N-terminal domain-containing protein	NA
Sapur.013G083600.1	1821.651884	0.613655966	0.030954755	19.82429	1.84E-87	1.77E-86	cyan	0.822173031	9.13E-40	GO:0008168,GO:0008152	AT1G64970.1	G-TMT,TMT1,VTE4	gamma-tocopherol methyltransferase	gamma-tocopherol methyltransferase
Sapur.003G053800.1	83.288802	-0.895231891	0.045159999	-19.8236	1.86E-87	1.80E-86	cyan	-0.848769841	9.59E-45		AT1G32730.1			electron carrier/iron ion-binding protein, putative
Sapur.004G100500.1	32.86055844	2.550139988	0.128758067	19.80567	2.66E-87	2.56E-86	cyan	0.617624123	6.95E-18		AT5G08480.2		VQ motif-containing protein	NA
Sapur.002G080000.1	5566.205145	0.633484292	0.031992613	19.80096	2.92E-87	2.81E-86	cyan	0.831166036	2.37E-41	GO:0019904	AT1G78300.1	14-3-3OMEGA.GF14 OMEG.	general regulatory factor 2	14-3-3-like protein GF14 phi protein
Sapur.T023200.1	71.14699132	1.292199565	0.065290455	19.79155	3.52E-87	3.39E-86	cyan	0.811465168	5.44E-38		AT3G07565.1		Protein of unknown function (DUF3755)	NA
Sapur.007G014200.1	64.38292643	-1.374948125	0.069477166	-19.7899	3.64E-87	3.50E-86	cyan	-0.851404355	2.73E-45	GO:0005515	AT3G54350.3	emb1967	Forkhead-associated (FHA) domain-containing protein	NA
Sapur.018G114400.1	11.51858244	5.181557531	0.261855497	19.78785	3.79E-87	3.64E-86	cyan	0.737646257	3.14E-28	GO:0006950	AT2G24370.1		Protein kinase protein with adenine nucleotide alpha hydrolases-like domain	universal stress family protein
Sapur.011G012100.1	926.2699207	0.662935959	0.03350444	19.78651	3.89E-87	3.74E-86	cyan	0.856011004	2.87E-46		AT4G05530.1	IBR1,SDRA	indole-3-butyric acid response 1	short-chain dehydrogenase/reductase family protein
Sapur.016G227700.1	833.455765	1.049672183	0.053102239	19.767	5.73E-87	5.50E-86	cyan	0.794429856	2.18E-35	GO:0006355,GO:0003700	AT1G50640.1	ATERF3,ERF3	ethylene responsive element binding factor 3	ethylene-responsive transcription factor, putative
Sapur.002G079900.1	71.74344906	1.897913561	0.09603082	19.76359	6.13E-87	5.88E-86	cyan	0.7764859	6.70E-33	GO:0006468,GO:0005524,GO:0004672	AT4G33950.1	ATOST1.OST1.P44.SNRK2-6	Protein kinase superfamily protein	Serine/Threonine-kinase SAPK1
Sapur.008G097700.1	954.0289365	0.937423379	0.047447298	19.75715	6.96E-87	6.68E-86	cyan	0.795518064	1.51E-35		AT1G25420.1		Regulator of Vps4 activity in the MVB pathway protein	regulator of Vps4 activity in the MVB pathway protein
Sapur.018G058700.1	507.6929489	-1.179461351	0.059749536	-19.7401	9.76E-87	9.34E-86	cyan	-0.812657511	3.50E-38		AT5G21222.1		protein kinase family protein	PPR containing plant protein
Sapur.013G104900.1	256.2478388	0.993543862	0.050336925	19.73787	1.02E-86	9.76E-86	cyan	0.807305069	2.48E-37		AT4G09580.1		SNARE associated Golgi protein family	SNARE associated protein
Sapur.010G081700.1	18.86976043	-1.940072283	0.098384275	-19.7193	1.47E-86	1.41E-85	cyan	-0.831157352	2.38E-41	GO:0005975,GO:0004553	AT2G01630.1		O-Glycosyl hydrolases family 17 protein	glucan endo-1%2C3-beta-glucosidase, putative
Sapur.010G073400.1	1340.2707	0.998613015	0.050672425	19.70723	1.87E-86	1.78E-85	cyan	0.795028701	1.78E-35	GO:0016020,GO:0006810,GO:0005215	AT2G02040.1	ATPTR2.ATPTR2-B.NTR1.PT	peptide transporter 2	peptide transporter
Sapur.003G141600.1	522.6547042	-0.512103506	0.025990315	-19.7036	2.01E-86	1.91E-85	cyan	-0.840806288	3.70E-43	GO:0005515,GO:0043161,GO:0006289,GO:0003684	AT1G79650.2	RAD23,RAD23B	Rad23 UV excision repair protein family	ubiquitin receptor RAD23b
Sapur.008G153700.1	59.39058962	-1.137574472	0.057765356	-19.693	2.47E-86	2.36E-85	cyan	-0.848484343	1.10E-44		AT1G69935.1	SHW1	short hypocotyl in white light1	transmembrane protein, putative
Sapur.003G031100.1	1300.363033	0.575346105	0.02923902	19.67734	3.37E-86	3.21E-85	cyan	0.841546373	2.65E-43	GO:0030170	AT1G80360.1		Pyridoxal phosphate (PLP)-dependent transferases superfamily protein	aspartate aminotransferase
Sapur.010G135100.1	99.19563588	-1.676280129	0.085226873	-19.6684	4.02E-86	3.82E-85	cyan	-0.883342496	7.11E-53		AT1G79420.1		Protein of unknown function (DUF620)	plant/T8K14-16 protein
Sapur.008G005800.1	38.82896246	2.789287	0.141867223	19.66125	4.63E-86	4.40E-85	cyan	0.663097045	3.05E-21	GO:0005515,GO:0003676	AT2G27040.1	AGO4,OCP11	Argonaute family protein	argonaute protein
Sapur.013G079600.1	791.2511498	0.834503216	0.042468168	19.65009	5.77E-86	5.48E-85	cyan	0.773008339	1.91E-32		AT3G54680.1		proteophosphoglycan-related	plant/T5N23-40 protein, putative
Sapur.019G112000.1	510.1201995	0.992739017	0.050538583	19.64319	6.61E-86	6.27E-85	cyan	0.814312784	1.88E-38	GO:0043565,GO:0006355,GO:0003700	AT2G40620.1		Basic-leucine zipper (bZIP) transcription factor family protein	BZIP transcription factor
Sapur.002G193400.1	307.0892321	1.564739528	0.079694894	19.63413	7.90E-86	7.49E-85	cyan	0.79728889	8.29E-36	GO:0006468,GO:0004672,GO:0005524	AT3G25250.1	AGC2,AGC2-1,AtOXI1,OXI1	AGC (cAMP-dependent, cGMP-dependent and protein kinase C) kinase family protein	Serine/Threonine kinase domain protein
Sapur.016G161100.1	85.62239934	-0.789183488	0.040194917	-19.6339	7.94E-86	7.52E-85	cyan	-0.856403112	2.36E-46		AT5G01110.1		Tetratricopeptide repeat (TPR)-like superfamily protein	PPR containing plant protein
Sapur.006G068500.1	2535.943451	0.766377452	0.039038039	19.63156	8.31E-86	7.87E-85	cyan	0.818118907	4.43E-39	GO:0005096	AT3G53710.1	AGD6	ARF-GAP domain 6	ADP-ribosylation factor GTPase-activating protein AGD7, putative
Sapur.004G176300.1	548.647813	-1.159374579	0.059070259	-19.627	9.08E-86	8.59E-85	cyan	-0.862169572	1.25E-47	GO:0006412,GO:0005840,GO:0005622,GO:0003735	AT3G54210.1		Ribosomal protein L17 family protein	50S ribosomal protein L17
Sapur.005G100600.1	1497.391505	0.779488625	0.039716386	19.62637	9.21E-86	8.70E-85	cyan	0.82662299	1.54E-40	GO:0003824	AT4G16580.1		Protein phosphatase 2C family protein	NA
Sapur.016G237800.1	19.64094846	-1.729262678	0.088160904	-19.6148	1.15E-85	1.09E-84	cyan	-0.813955025	2.15E-38		AT2G45880.1	BAM7,BMY4	beta-amylase 7	brassinazole-resistant 1 protein, putative
Sapur.009G130700.1	14.4709013	3.304002264	0.16863747	19.59234	1.80E-85	1.69E-84	cyan	0.722550826	1.24E-26	GO:0016757,GO:0047262	AT4G38270.1	GAUT3	galacturonosyltransferase 3	galacturonosyltransferase, putative
Sapur.001G150200.1	613.969261	1.588228125	0.081088918	19.58625	2.03E-85	1.91E-84	cyan	0.736157683	4.56E-28	GO:0046872,GO:0000166,GO:0016021,GO:0015914,GO:0005524,GO:0004012,GO:000028	AT1G17500.1		ATPase E1-E2 type family protein / haloacid dehalogenase-like hydrolase family protein	phospholipid-transporting ATPase, putative
Sapur.014G057800.1	113.0620651	-0.777024299	0.039683482	-19.5805	2.27E-85	2.13E-84	cyan	-0.852081034	1.97E-45	GO:0006281,GO:0004518	AT1G01880.1		5\'-3\' exonuclease family protein	flap endonuclease GEN-like protein
Sapur.014G056800.1	320.39715	1.768288058	0.090308939	19.58043	2.27E-85	2.13E-84	cyan	0.755979973	2.52E-30		AT2G45680.1		TCP family transcription factor	NA
Sapur.019G014300.1	608.8601393	0.941956588	0.048108948	19.57965	2.31E-85	2.17E-84	cyan	0.81041869	8.00E-38		AT4G08180.1	ORP1C	OSBP(oxysterol binding protein)-related protein 1C	oxysterol-binding protein-related protein 1C
Sapur.002G052900.1	14.14000987	2.847277931	0.145422893	19.5793	2.32E-85	2.18E-84	cyan	0.769764293	5.01E-32					NA
Sapur.15WG066500.1	777.0830892	-0.753607566	0.038504422	-19.572	2.68E-85	2.52E-84	cyan	-0.876271151	5.12E-51	GO:0005524	AT3G18190.1		TCP-1/cpn60 chaperonin family protein	TCP-1 (CTT or eukaryotic type II) chaperonin family, delta subunit
Sapur.016G098200.1	809.7922734	0.76737099	0.039209291	19.57115	2.72E-85	2.56E-84	cyan	0.828440985	7.33E-41	GO:0006614,GO:0005525,GO:0048500,GO:0008312	AT1G48900.1		Signal recognition particle, SRP54 subunit protein	signal recognition particle 54 kDa protein
Sapur.007G043200.1	197.0899304	1.570832213	0.080270553	19.56922	2.83E-85	2.66E-84	cyan	0.769858913	4.87E-32	GO:0005515	AT2G23380.1	CLF,ICU1,SDG1,SET1	SET domain-containing protein	histone-lysine N-methyltransferase CLF
Sapur.005G115800.1	3206.173227	0.807129978	0.041262023	19.56109	3.32E-85	3.11E-84	cyan	0.807705674	2.15E-37	GO:0019904	AT1G78300.1	14-3-3OMEGA.GF14 OMEG.	general regulatory factor 2	14-3-3-like protein GF14 omega
Sapur.006G110200.1	89.45891151	0.955926857	0.04886918	19.56094	3.33E-85	3.12E-84	cyan	0.854610019	5.74E-46	GO:0006468,GO:0005524,GO:0004672	AT5G02800.1		Protein kinase superfamily protein	Serine/Threonine kinase
Sapur.004G100000.1	136.343628	1.547839924	0.079160893	19.55309	3.88E-85	3.63E-84	cyan	0.810860577	6.80E-38	GO:0003676	AT1G33470.2		RNA-binding (RRM/RBD/RNP motifs) family protein	RNA recognition motif 2 in RNA-binding protein musashi-like musashi protein
Sapur.002G054900.1	18.73792435	-2.678210574	0.137022306	-19.5458	4.48E-85	4.19E-84	cyan	-0.814252536	1.93E-38		AT1G21310.1	ATEXT3,EXT3,RSH	extensin 3	NA
Sapur.007G114300.1	87.08108777	-1.669244462	0.085419491	-19.5417	4.85E-85	4.53E-84	cyan	-0.862461517	1.07E-47		AT3G59400.1	GUN4	enzyme binding;tetrapyrrole binding	GUN4 domain protein
Sapur.017G063700.1	981.4062145	1.702622497	0.0871578	19.53494	5.54E-85	5.18E-84	cyan	0.768413678	7.44E-32	GO:0016020,GO:0006810,GO:0005215	AT5G62680.1		Major facilitator superfamily protein	NA
Sapur.016G007400.1	28.33145052	2.182927836	0.111856059	19.51551	8.11E-85	7.56E-84	cyan	0.797610156	7.43E-36	GO:0055114,GO:0020037,GO:0016705,GO:0005506	AT4G31970.1	CYP82C2	cytochrome P450, family 82, subfamily C, polypeptide 2	NA
Sapur.019G081000.1	1809.381279	-0.969994047	0.049726493	-19.5066	9.65E-85	8.99E-84	cyan	-0.860721317	2.64E-47	GO:0005840	AT1G35680.1		Ribosomal protein L21	50S ribosomal protein L21
Sapur.017G057900.1	453.7263659	1.351894444	0.069334008	19.49829	1.14E-84	1.06E-83	cyan	0.785889963	3.57E-34	GO:0016301,GO:0005524,GO:0016887,GO:0055085,GO:0042626,GO:0016021,GO:000681	AT3G28345.1		ABC transporter family protein	multidrug/pheromone exporter, MDR family, ABC transporter family protein, putative
Sapur.012G054100.1	19.75624419	-1.516855456	0.077899489	-19.472	1.90E-84	1.76E-83	cyan	-0.826480169	1.63E-40	GO:0005515	AT5G50120.1		Transducin/WD40 repeat-like superfamily protein	F-box and WD40 domain protein, putative
Sapur.014G116200.1	78.00227747	-1.128446691	0.05796216	-19.4687	2.02E-84	1.88E-83	cyan	-0.842580666	1.67E-43	GO:0006468,GO:0004672	AT3G58690.1		Protein kinase superfamily protein	Serine/Threonine kinase
Sapur.002G132000.2	38.0281926	-1.317239829	0.067661814	-19.468	2.05E-84	1.90E-83	cyan	-0.804437445	6.92E-37	GO:0006468,GO:0005524,GO:0004672	AT4G01370.1	ATMPK4,MPK4	MAP kinase 4	MAP kinase
Sapur.012G019500.1	173.6148943	1.757439219	0.090293388	19.46365	2.23E-84	2.07E-83	cyan	0.787287782	2.28E-34		AT4G27940.1	ATMTM1,MTM1	manganese tracking factor for mitochondrial SOD2	carrier CGI-69 protein
Sapur.010G115900.1	569.7041691	-0.883238334	0.045382886	-19.4619	2.31E-84	2.14E-83	cyan	-0.838107377	1.22E-42	GO:0005975,GO:0004553	AT3G06510.1	ATSFR2,SFR2	Glycosyl hydrolase superfamily protein	beta-glycosidase, putative
Sapur.002G116500.1	49.74949332	1.760770101	0.090485476	19.45915	2.44E-84	2.26E-83	cyan	0.82156295	1.16E-39	GO:0043565,GO:0008270,GO:0006355,GO:0003700	AT3G60530.1	GATA4	GATA transcription factor 4	GATA transcription factor, putative
Sapur.010G100900.1	9.459883958	6.476643117	0.332954249	19.45205	2.80E-84	2.59E-83	cyan	0.609419651	2.46E-17		AT1G73965.1	CLE13	CLAVATA3/ESR-RELATED 13	CLE12, putative
Sapur.013G012600.1	245.8193807	-1.314400341	0.067601016	-19.4435	3.31E-84	3.06E-83	cyan	-0.805742899	4.35E-37		AT3G05600.1		alpha/beta-Hydrolases superfamily protein	NA
Sapur.016G223000.1	1063.226284	-1.086120065	0.05592941	-19.4195	5.28E-84	4.87E-83	cyan	-0.852196247	1.87E-45		AT5G14910.1		Heavy metal transport/detoxification superfamily protein	metal ion-binding protein, putative
Sapur.15WG018200.1	2742.918632	0.693823175	0.035750481	19.40738	6.69E-84	6.16E-83	cyan	0.814708212	1.62E-38		AT5G53300.2	UBC10	ubiquitin-conjugating enzyme 10	ubiquitin-conjugating enzyme E2
Sapur.001G047500.1	1041.423048	0.738926578	0.038085088	19.40199	7.42E-84	6.83E-83	cyan	0.811649763	5.08E-38		AT1G27290.2			transmembrane protein, putative
Sapur.003G143900.1	737.0122652	-0.864514485	0.044585444	-19.3901	9.36E-84	8.61E-83	cyan	-0.868611195	3.96E-49	GO:0008146	AT5G07010.1	ATST2A,ST2A	sulfotransferase 2A	flavonol 4'-sulfotransferase, putative
Sapur.007G045000.1	138.4226398	1.881624533	0.097052138	19.38777	9.79E-84	9.00E-83	cyan	0.689791692	1.68E-23		AT3G50120.1		Plant protein of unknown function (DUF247)	transmembrane protein, putative
Sapur.017G084700.1	41.77189201	-1.445495906	0.074563313	-19.3862	1.01E-83	9.28E-83	cyan	-0.853369973	1.05E-45	GO:0006351,GO:0003899,GO:0003677	AT5G15700.2		DNA/RNA polymerases superfamily protein	DNA-directed RNA polymerase
Sapur.012G105800.1	4008.760163	0.872284666	0.045035652	19.36876	1.42E-83	1.30E-82	cyan	0.818026243	4.59E-39		AT3G48890.1	ATMAPR3.ATMP2.MAPR3.M	membrane-associated progesterone binding protein 3	membrane steroid-binding protein
Sapur.014G066700.1	198.8424249	1.488834984	0.076868364	19.36863	1.42E-83	1.30E-82	cyan	0.7485395	1.88E-29	GO:0050017,GO:0019499,GO:0005739,GO:0004124	AT3G61440.1	ARATH:BSAS3:1.ATCYSC1.C	cysteine synthase C1	L-3-cyanoalanine synthase/cysteine synthase
Sapur.005G103900.1	317.0323989	-0.659380088	0.034050797	-19.3646	1.54E-83	1.41E-82	cyan	-0.854881547	5.03E-46		AT4G37210.1		Tetratricopeptide repeat (TPR)-like superfamily protein	TPR containing protein, putative
Sapur.005G103700.1	1777.427116	-1.004416699	0.051869975	-19.3641	1.55E-83	1.42E-82	cyan	-0.830577054	3.03E-41	GO:0042549,GO:0019898,GO:0015979,GO:0009654,GO:0009523,GO:0005509	AT3G50820.1	OEC33,PSBO-2,PSBO2	photosystem II subunit O-2	oxygen-evolving enhancer protein
Sapur.012G022800.1	1366.928436	0.567062776	0.029290527	19.35994	1.68E-83	1.54E-82	cyan	0.831631574	1.95E-41	GO:0008152,GO:0003824,GO:0048037	AT5G08300.1		Succinyl-CoA ligase, alpha subunit	succinyl-CoA ligase [ADP-forming] subunit alpha, putative
Sapur.018G102500.1	41.20573961	-1.80466459	0.093217149	-19.3598	1.69E-83	1.54E-82	cyan	-0.804402313	7.01E-37		AT3G15760.1			transmembrane protein, putative
Sapur.014G101000.1	49.96154798	2.37730376	0.122810929	19.35743	1.76E-83	1.61E-82	cyan	0.711697381	1.51E-25	GO:0006355,GO:0003700	AT3G16770.1	ATEBP,EBP,ERF72,RAP2.3	ethylene-responsive element binding protein	ethylene response factor 2
Sapur.003G057100.1	207.5173084	0.660794791	0.034146613	19.35169	1.97E-83	1.80E-82	cyan	0.840489499	4.26E-43	GO:0005515	AT2G35360.1		ubiquitin family protein	ubiquitin family protein

Sapur.003G051000.1	9.14406683	5.150509337	0.266332594	19.33864	2.54E-83	2.32E-82	cyan	0.719944941	2.29E-26	GO:0005789	AT5G47180.1		Plant VAMP (vesicle-associated membrane protein) family protein	vesicle-associated membrane protein, putative
Sapur.010G151100.1	1575.312454	0.697947959	0.036108837	19.32901	3.06E-83	2.79E-82	cyan	0.844012028	8.71E-44		AT2G39950.1			hypothetical protein
Sapur.005G203600.1	5028.392598	-0.645272518	0.033393191	-19.3235	3.41E-83	3.11E-82	cyan	-0.808353446	1.70E-37	GO:0055114,GO:0016620	AT1G42970.1	GAPB	glyceraldehyde-3-phosphate dehydrogenase B subunit	glyceraldehyde-3-phosphate dehydrogenase
Sapur.007G111300.1	5.834655946	4.923971472	0.254863133	19.32006	3.64E-83	3.32E-82	cyan	0.790672177	7.57E-35	GO:0006468,GO:0005524,GO:0004672	AT1G67000.1		Protein kinase superfamily protein	tyrosine kinase family protein
Sapur.016G067200.1	4213.363024	-0.585393092	0.030303795	-19.3175	3.83E-83	3.49E-82	cyan	-0.844509427	6.94E-44	GO:0055114,GO:0051920,GO:0016491	AT5G06290.1	2-Cys Prx B,2CPB	2-cysteine peroxiredoxin B	2-cys peroxiredoxin
Sapur.006G219600.1	15.84442548	-2.274817661	0.117768628	-19.316	3.94E-83	3.59E-82	cyan	-0.829694802	4.37E-41					hypothetical protein
Sapur.005G092600.1	279.4020759	0.936461936	0.048488916	19.31291	4.18E-83	3.81E-82	cyan	0.804519713	6.72E-37		AT1G19860.1		Zinc finger C-x8-C-x5-C-x3-H type family protein	NA
Sapur.017G071600.1	62.33916704	-1.333293394	0.069087408	-19.2986	5.51E-83	5.02E-82	cyan	-0.830056012	3.76E-41	GO:0016021	AT3G29060.1		EXS (ERD1/XPR1/SYG1) family protein	phosphate transporter PHO1-like protein
Sapur.010G127100.1	935.9359396	0.558300311	0.028929567	19.29861	5.52E-83	5.02E-82	cyan	0.84537434	4.66E-44	GO:0006468,GO:0004672	AT1G14000.1	VIK	VH1-interacting kinase	tyrosine kinase domain protein
Sapur.T030500.1	85.25144445	3.426038218	0.177554655	19.29568	5.84E-83	5.31E-82	cyan	0.673860545	3.99E-22		AT5G55400.1		Actin binding Calponin homology (CH) domain-containing protein	NA
Sapur.001G149700.1	109.1042963	-1.153973125	0.059824684	-19.2892	6.61E-83	6.01E-82	cyan	-0.856704942	2.03E-46		AT3G13780.1		SMAD/FHA domain-containing protein	SMAD/FHA protein, putative
Sapur.012G028000.1	14.10497861	2.84297545	0.147394762	19.28817	6.75E-83	6.13E-82	cyan	0.742568875	8.94E-29	GO:0016567,GO:0004842	AT5G37490.1		ARM repeat superfamily protein	Avr9/Cf-9 rapidly elicited protein
Sapur.016G046500.1	603.7403904	-0.767370175	0.039791754	-19.2847	7.23E-83	6.56E-82	cyan	-0.851890037	2.16E-45	GO:0006468,GO:0005524,GO:0004672	AT3G44200.1	ATNEK6,IBO1,NEK6	NIMA (never in mitosis, gene A)-related 6	Serine/Threonine kinase Nek2
Sapur.002G045800.1	1675.85502	0.956652587	0.049623901	19.27806	8.21E-83	7.44E-82	cyan	0.760248943	7.70E-31	GO:0055114,GO:0016701,GO:0009072,GO:0003868	AT1G06570.1	HPD,PDS1	phytoene desaturation 1	4-hydroxyphenylpyruvate dioxygenase
Sapur.006G108900.1	330.9764715	1.215474216	0.063059668	19.27499	8.71E-83	7.90E-82	cyan	0.818621769	3.65E-39					NA
Sapur.018G078900.1	119.0970669	-0.877469907	0.045574054	-19.2537	1.31E-82	1.19E-81	cyan	-0.85563852	3.46E-46	GO:0007049,GO:0005634	AT4G30820.1		cyclin-dependent kinase-activating kinase assembly factor-related / CDK-activating kinase assembly factor-relatec	CDK-activating kinase assembly factor MAT1
Sapur.010G116800.1	9312.008849	-1.492046176	0.077495612	-19.2533	1.32E-82	1.20E-81	cyan	-0.846977928	2.22E-44		AT3G17210.1	ATHS1,HS1	heat stable protein 1	stress responsive A/B barrel domain protein
Sapur.009G056800.1	749.5342711	1.790135416	0.092989822	19.25087	1.39E-82	1.25E-81	cyan	0.776033293	7.69E-33	GO:0006529,GO:0004066	AT3G47340.1	ASN1,AT-ASN1,DIN6	glutamine-dependent asparagine synthase 1	asparagine synthetase [glutamine-hydrolyzing] protein
Sapur.008G112000.1	61.26535127	-1.065511296	0.055355404	-19.2486	1.45E-82	1.31E-81	cyan	-0.831351938	2.19E-41		AT1G66140.1	ZFP4	zinc finger protein 4	zinc finger protein, putative
Sapur.010G084900.1	86.50530366	-1.799198417	0.093474962	-19.2479	1.47E-82	1.33E-81	cyan	-0.825817543	2.13E-40	GO:0016787	AT1G30110.2	ATNUDX25,NUDX25	nudix hydrolase homolog 25	nudix hydrolase
Sapur.005G052400.4	38.72702289	-1.51440199	0.078702382	-19.2421	1.64E-82	1.48E-81	cyan	-0.847024635	2.17E-44	GO:0005975,GO:0004553	AT5G63840.1	PSL5,RSW3	Glycosyl hydrolases family 31 protein	neutral alpha-glucosidase AB
Sapur.017G001200.1	82.87408926	1.499909369	0.077965187	19.23819	1.77E-82	1.60E-81	cyan	0.751978186	7.49E-30		AT2G44010.1			hypothetical protein
Sapur.011G050800.1	437.8689048	0.657606767	0.034183074	19.23779	1.79E-82	1.61E-81	cyan	0.813818514	2.27E-38		AT1G29760.1		Putative adipose-regulatory protein (Seipin)	adipose-regulatory protein, putative
Sapur.018G054200.1	421.2169651	0.620727671	0.032269804	19.23556	1.87E-82	1.68E-81	cyan	0.83592831	3.14E-42	GO:0005515	AT3G24350.1	ATSYP32,SYP32	syntaxin of plants 32	Qa-SNARE, SYP3/Sed5p/syntaxin 5-type protein
Sapur.019G113500.1	405.6310125	1.132044589	0.058895766	19.22115	2.46E-82	2.22E-81	cyan	0.79864027	5.23E-36		AT2G38610.2		RNA-binding KH domain-containing protein	nucleic acid-binding protein
Sapur.004G168300.1	134.7936065	1.322492358	0.068807344	19.22022	2.51E-82	2.26E-81	cyan	0.737034954	3.66E-28	GO:0016567,GO:0004842	AT5G01830.1		ARM repeat superfamily protein	U-box protein
Sapur.009G010100.1	548.8692245	0.843481679	0.043888059	19.21893	2.57E-82	2.31E-81	cyan	0.736597695	4.09E-28	GO:0051082,GO:0031072	AT5G22060.1	ATJ2,J2	DNAJ homologue 2	chaperone protein DnaJ
Sapur.010G066200.1	1526.053785	0.658361254	0.034272137	19.20981	3.06E-82	2.75E-81	cyan	0.830304851	3.39E-41		AT5G43830.1		Aluminium induced protein with YGL and LRDR motifs	aluminum-induced-like protein
Sapur.010G198300.1	359.539122	1.93813165	0.100978534	19.1935	4.19E-82	3.76E-81	cyan	0.721167014	1.72E-26		AT5G16010.1		3-oxo-5-alpha-steroid 4-dehydrogenase family protein	3-oxo-5-alpha-steroid 4-dehydrogenase
Sapur.013G002500.1	83.67864787	-1.015716183	0.05292376	-19.1921	4.31E-82	3.87E-81	cyan	-0.828646622	6.73E-41	GO:0010468,GO:0005777	AT5G61190.1		putative endonuclease or glycosyl hydrolase with C2H2-type zinc finger domain	NYN domain protein
Sapur.010G072900.2	5.255048741	-3.997922768	0.208335022	-19.1899	4.50E-82	4.03E-81	cyan	-0.827422629	1.11E-40		AT1G14630.1			hypothetical protein
Sapur.010G156900.1	55.77212187	-1.693060979	0.088288966	-19.1764	5.83E-82	5.22E-81	cyan	-0.81305333	3.02E-38		AT2G44920.2		Tetratricopeptide repeat (TPR)-like superfamily protein	NA
Sapur.005G195800.1	690.4087973	0.582471313	0.030393322	19.16445	7.33E-82	6.56E-81	cyan	0.815117739	1.39E-38		AT1G20225.1		Thioredoxin superfamily protein	thioredoxin f5, putative
Sapur.003G145400.1	51.92349097	-0.951780915	0.049667848	-19.1629	7.55E-82	6.76E-81	cyan	-0.849580717	6.53E-45	GO:0006355,GO:0005739,GO:0003690	AT5G07900.1		Mitochondrial transcription termination factor family protein	mTERF protein
Sapur.008G017300.1	119.6751863	-1.093548186	0.057076503	-19.1593	8.09E-82	7.23E-81	cyan	-0.83449819	5.80E-42		AT5G03680.1	PTL	Duplicated homeodomain-like superfamily protein	transcription factor, putative
Sapur.005G186400.1	537.013053	1.030294632	0.053799408	19.15067	9.56E-82	8.54E-81	cyan	0.827549094	1.06E-40	GO:0016787	AT2G16430.2	ATPAP10,PAP10	purple acid phosphatase 10	purple acid phosphatase
Sapur.017G001400.1	289.2279931	-1.910676082	0.099775913	-19.1497	9.74E-82	8.70E-81	cyan	-0.797887445	6.76E-36	GO:0055114,GO:0016491,GO:0008270	AT1G26320.1		Zinc-binding dehydrogenase family protein	2-alkenal reductase
Sapur.009G097300.1	448.1070795	-0.724033946	0.037809782	-19.1494	9.79E-82	8.75E-81	cyan	-0.838114846	1.21E-42	GO:0055085,GO:0016020,GO:0015297,GO:0015238,GO:0006855	AT2G21340.1		MATE efflux family protein	DNA-damage-inducible protein F, putative
Sapur.012G087800.1	7.789552501	5.574456434	0.291103748	19.14938	9.79E-82	8.75E-81	cyan	0.657894891	7.90E-21		AT5G45160.1		Root hair defective 3 GTP-binding protein (RHD3)	ROOT HAIR defective-like protein
Sapur.019G108700.1	45.44939432	-1.619840784	0.084619322	-19.1427	1.11E-81	9.93E-81	cyan	-0.852780027	1.41E-45	GO:0055114,GO:0016491,GO:0005507	AT2G30210.1	LAC3	laccase 3	laccase
Sapur.012G088300.1	898.5781742	0.695655128	0.036381682	19.12103	1.69E-81	1.50E-80	cyan	0.82189725	1.02E-39	GO:0005525	AT5G45130.1	ATRAB-F2A,ATRAB5A,ATRA	RAB homolog 1	Rab-related small GTP-binding protein
Sapur.006G099700.1	296.6223813	-0.748544952	0.039154172	-19.1179	1.79E-81	1.59E-80	cyan	-0.836702042	2.25E-42	GO:0005525	AT5G03530.1	ATRAB.ATRAB ALPHA.ATRA	RAB GTPase homolog C2A	Ras small GTPase family Ras protein
Sapur.017G075300.1	90.39660885	-0.850155293	0.044469256	-19.1178	1.79E-81	1.60E-80	cyan	-0.836961568	2.01E-42		AT5G61190.1		putative endonuclease or glycosyl hydrolase with C2H2-type zinc finger domain	C2H2 type zf-met: zinc-finger protein
Sapur.004G056800.1	191.0041959	-2.023460653	0.105860906	-19.1143	1.92E-81	1.71E-80	cyan	-0.807196409	2.58E-37	GO:0046872	AT1G65920.1		Regulator of chromosome condensation (RCC1) family with FYVE zinc finger domain	NA
Sapur.014G031800.1	15.55317421	4.80484687	0.251604538	19.09682	2.68E-81	2.38E-80	cyan	0.634095626	4.90E-19	GO:0016559,GO:0005779	AT1G47750.1	PEX11A	peroxin 11A	peroxisomal biogenesis factor 11
Sapur.019G073400.1	3708.594429	1.009131564	0.052851646	19.09366	2.85E-81	2.53E-80	cyan	0.814373416	1.84E-38	GO:0020037,GO:0009055	AT1G22840.1	ATCYTC-A,CYTC-1	CYTOCHROME C-1	cytochrome protein C
Sapur.002G134700.1	363.6514419	1.151259776	0.060299896	19.09223	2.93E-81	2.60E-80	cyan	0.768298758	7.69E-32	GO:0008234,GO:0006508	AT1G02305.1		Cysteine proteinases superfamily protein	cathepsin B-like cysteine protease
Sapur.005G178000.1	1121.374823	1.18031696	0.061872617	19.07656	3.95E-81	3.50E-80	cyan	0.770580941	3.94E-32	GO:0055085,GO:0016021	AT2G38940.1	ATPT2,PHT1;4	phosphate transporter 1;4	inorganic phosphate transporter
Sapur.002G097800.1	1541.329315	1.118335505	0.058628629	19.0749	4.08E-81	3.61E-80	cyan	0.773785604	1.52E-32		AT4G24220.1	AWI31,VEP1	NAD(P)-binding Rossmann-fold superfamily protein	progesterone 5-beta-reductase
Sapur.014G041000.1	225.6905781	-2.060290479	0.108022448	-19.0728	4.25E-81	3.76E-80	cyan	-0.810316039	8.30E-38		AT3G60470.1		Plant protein of unknown function (DUF247)	NA
Sapur.007G031300.1	7625.397296	-0.879495235	0.046119352	-19.07	4.48E-81	3.96E-80	cyan	-0.841422271	2.81E-43	GO:0042549,GO:0019898,GO:0015979,GO:0009654,GO:0009523,GO:0005509	AT5G66570.1	MSP-1.OE33.OEE1.OEE33.P	PS II oxygen-evolving complex 1	oxygen-evolving enhancer protein
Sapur.011G087400.1	779.4469385	1.056458812	0.055458528	19.04953	6.63E-81	5.85E-80	cyan	0.797397038	7.99E-36	GO:0005515	AT3G15470.1		Transducin/WD40 repeat-like superfamily protein	WD domain, G-beta repeat protein
Sapur.014G057700.1	21.9906049	-2.75978844	0.144923582	-19.0431	7.50E-81	6.61E-80	cyan	-0.792747387	3.82E-35		AT4G00770.1			hypothetical protein
Sapur.15WG026800.1	73.74803671	-1.423341189	0.074753071	-19.0406	7.87E-81	6.93E-80	cyan	-0.833733232	8.03E-42	GO:0055085,GO:0016020,GO:0006811,GO:0005216	AT3G17700.1	ATCNGC20.CNBT1.CNGC20	cyclic nucleotide-binding transporter 1	NA
Sapur.018G003000.1	342.6587248	-0.435430931	0.022868773	-19.0404	7.89E-81	6.95E-80	cyan	-0.83985003	5.65E-43	GO:0003723	AT4G26000.1	PEP	RNA-binding KH domain-containing protein	nucleic acid-binding protein
Sapur.017G078300.2	86.77087597	1.988811679	0.104463485	19.03834	8.21E-81	7.23E-80	cyan	0.751277885	9.04E-30	GO:0007165	AT3G29575.4	AFP3	ABI five binding protein 3	ninja-family protein AFP1, putative
Sapur.001G059000.1	307.6729432	-0.882544126	0.046381302	-19.028	1.00E-80	8.79E-80	cyan	-0.837227136	1.79E-42		AT5G44650.1	AtCEST,CEST		transmembrane protein, putative
Sapur.005G190300.1	5643.477913	1.328463606	0.069819537	19.0271	1.02E-80	8.94E-80	cyan	0.784036059	6.43E-34	GO:0004781	AT1G19920.1	APS2,ASA1	Pseudouridine synthase/archaeosine transglycosylase-like family protein	ATP sulfurylase
Sapur.013G106700.1	771.2668072	0.758440871	0.039901629	19.00777	1.47E-80	1.29E-79	cyan	0.813948012	2.16E-38		AT1G34340.1		alpha/beta-Hydrolases superfamily protein	alpha/beta-hydrolase domain protein
Sapur.013G062900.1	23.55218199	2.441774308	0.128477112	19.00552	1.54E-80	1.35E-79	cyan	0.717219488	4.31E-26					NA
Sapur.005G108500.1	768.825354	-0.88803609	0.046755872	-18.993	1.95E-80	1.71E-79	cyan	-0.869594047	2.30E-49	GO:0006355,GO:0003700,GO:0007275	AT4G36920.2	AP2,FL1,FLO2	Integrase-type DNA-binding superfamily protein	AP2 transcription factor
Sapur.009G115100.1	1813.119402	1.487293147	0.078347374	18.98332	2.34E-80	2.05E-79	cyan	0.799886808	3.40E-36	GO:0050662,GO:0003824,GO:0080019	AT4G33790.1	CER4,FAR3,G7	Jojoba acyl CoA reductase-related male sterility protein	NA
Sapur.019G107900.1	326.3015508	-0.797830734	0.042037179	-18.9792	2.54E-80	2.22E-79	cyan	-0.841356613	2.89E-43	GO:0003676,GO:0017069,GO:0000398	AT2G30260.1	U2B\'\'	U2 small nuclear ribonucleoprotein B	NA
Sapur.010G138600.1	2567.566196	-1.19415943	0.062920403	-18.9789	2.55E-80	2.23E-79	cyan	-0.805356797	4.99E-37	GO:0016597,GO:0008152	AT5G16290.2	VAT1	VALINE-TOLERANT 1	acetolactate synthase, small subunit
Sapur.005G140700.1	3197.123505	0.848885558	0.044729307	18.97829	2.58E-80	2.26E-79	cyan	0.809675653	1.05E-37	GO:0055114,GO:0016491,GO:0008152	AT1G44170.2	ALDH3H1,ALDH4	aldehyde dehydrogenase 3H1	aldehyde dehydrogenase family protein
Sapur.016G084900.1	215.6816539	-0.638476368	0.033647614	-18.9754	2.73E-80	2.38E-79	cyan	-0.826850073	1.40E-40		AT2G37195.1			transmembrane protein, putative
Sapur.016G180700.1	520.7103564	-0.999221485	0.052696233	-18.9619	3.52E-80	3.08E-79	cyan	-0.820869182	1.52E-39		AT4G34190.1	43709	stress enhanced protein 1	stress enhanced protein
Sapur.013G020600.1	75.05315944	2.197197203	0.116074824	18.92915	6.56E-80	5.72E-79	cyan	0.695839868	4.77E-24	GO:0006468,GO:0005524,GO:0004672	AT3G57700.1		Protein kinase superfamily protein	NA
Sapur.013G109800.1	217.811005	-0.962020824	0.05084142	-18.922	7.52E-80	6.55E-79	cyan	-0.829255874	5.24E-41		AT2G31090.1			transmembrane protein, putative
Sapur.004G168400.1	14.22816502	2.717160155	0.143598402	18.92194	7.52E-80	6.55E-79	cyan	0.759828256	8.66E-31					hypothetical protein
Sapur.008G163000.1	105.4722571	-1.072696372	0.05670135	-18.9184	8.05E-80	7.01E-79	cyan	-0.844232609	7.87E-44		AT5G30495.1		Fcf2 pre-rRNA processing protein	NA
Sapur.005G131700.1	690.3836201	1.597960347	0.084479699	18.91532	8.53E-80	7.42E-79	cyan	0.787685516	2.00E-34	GO:0016021,GO:0015116,GO:0008272,GO:0055085,GO:0016020,GO:0008271,GO:000588	AT1G22150.1	SULTR1;3	sulfate transporter 1;3	high affinity sulfate transporter
Sapur.016G073800.1	908.0952203	0.709648646	0.037517551	18.91511	8.56E-80	7.45E-79	cyan	0.823944939	4.52E-40	GO:0006468,GO:0004672	AT3G53570.4	AFC1,AME2,FC1	FUS3-complementing gene 1	Serine/Threonine kinase domain protein
Sapur.006G048700.1	159.6100563	1.618495841	0.085617803	18.90373	1.06E-79	9.23E-79	cyan	0.734011226	7.79E-28	GO:0005515	AT2G25290.2		Octicosapeptide/Phox/Bem1p (PB1) domain-containing protein / tetratricopeptide repeat (TPR)-containing protei	heat shock protein 70 (HSP70)-interacting protein, putative
Sapur.005G165600.1	601.4541419	-0.596726461	0.03157852	-18.8966	1.22E-79	1.06E-78	cyan	-0.837152545	1.85E-42	GO:0005525	AT4G02790.1		GTP-binding family protein	ribosome biogenesis GTPase A
Sapur.003G112500.1	76.35124311	2.154040251	0.114051427	18.88657	1.47E-79	1.27E-78	cyan	0.730304107	1.94E-27					NA
Sapur.017G032500.1	335.4396936	0.938952369	0.049721376	18.88428	1.54E-79	1.33E-78	cyan	0.808540751	1.59E-37	GO:0016791	AT2G14110.1		Haloacid dehalogenase-like hydrolase (HAD) superfamily protein	magnesium-dependent phosphatase
Sapur.002G023100.1	23.02120526	-2.335978176	0.123749347	-18.8767	1.77E-79	1.54E-78	cyan	-0.766934587	1.14E-31	GO:0006355,GO:0003677	AT1G75430.1	BLH11	BEL1-like homeodomain 11	BEL1 homeotic protein, putative
Sapur.010G048400.1	140.0350965	-0.788071147	0.041753661	-18.8743	1.86E-79	1.60E-78	cyan	-0.835789466	3.33E-42		AT3G04310.1			hypothetical protein
Sapur.019G051900.1	4700.365501	0.445075857	0.023584627	18.87144	1.96E-79	1.69E-78	cyan	0.826226177	1.81E-40	GO:0005515	AT5G36230.1		ARM repeat superfamily protein	translation initiation factor
Sapur.011G095700.1	180.2751303	0.961944091	0.050990448	18.86518	2.21E-79	1.90E-78	cyan	0.825855443	2.10E-40	GO:0004674,GO:0048544,GO:0006468,GO:0004672	AT4G21380.1	ARK3,RK3	receptor kinase 3	Serine/Threonine-kinase
Sapur.15WG038200.1	895.9283951	1.314772651	0.069711925	18.86008	2.43E-79	2.09E-78	cyan	0.836009148	3.03E-42	GO:0016301,GO:0008152,GO:0005524	AT1G73980.1		Phosphoribulokinase / Uridine kinase family	NA
Sapur.009G121300.1	10.20435712	4.529636739	0.240225241	18.85579	2.63E-79	2.27E-78	cyan	0.708915989	2.82E-25	GO:0030247,GO:0006468,GO:0005524,GO:0004672	AT1G21270.1	WAK2	wall-associated kinase 2	NA
Sapur.006G023500.1	83.58175806	-2.52784949	0.134092715	-18.8515	2.86E-79	2.46E-78	cyan	-0.779361198	2.78E-33	GO:0006457	AT5G48570.1	ATFKBP65,FKBP65,ROF2	FKBP-type peptidyl-prolyl cis-trans isomerase family protein	NA
Sapur.005G073500.1	34.42864244	2.119401848	0.112466046	18.84482	3.24E-79	2.79E-78	cyan	0.740793913	1.41E-28		AT5G23130.1		Peptidoglycan-binding LysM domain-containing protein	peptidoglycan-binding LysM domain protein, putative
Sapur.002G199700.1	1042.834052	-1.040252585	0.055212197	-18.841	3.48E-79	3.00E-78	cyan	-0.81994796	2.18E-39	GO:0016020,GO:0015979,GO:0009654,GO:0009523	AT4G28660.1	PSB28	photosystem II reaction center PSB28 protein	photosystem II reaction center Psb28 protein
Sapur.013G025600.1	176.7701812	1.215721383	0.064530589	18.83946	3.59E-79	3.08E-78	cyan	0.797006405	9.13E-36	GO:0008270,GO:0005515	AT3G05200.1	ATL6	RING/U-box superfamily protein	NA
Sapur.002G040700.1	14.81739745	-2.768104993	0.147119255	-18.8154	5.65E-79	4.85E-78	cyan	-0.84252782	1.71E-43	GO:0016829,GO:0010333,GO:0000287,GO:0008152	AT4G02780.1	ABC33.ATCPS1.CPS.CPS1.GA	Terpenoid cyclases/Protein prenyltransferases superfamily protein	copalyl diphosphate synthase
Sapur.010G075100.1	17.57309791	-2.022524774	0.107495983	-18.8149	5.70E-79	4.89E-78	cyan	-0.809541937	1.10E-37					NA
Sapur.007G011700.1	456.3237036	-1.664863784	0.088510605	-18.8098	6.28E-79	5.38E-78	cyan	-0.784193531	6.12E-34	GO:0006334,GO:0005634,GO:0003677,GO:0000786	AT2G18050.1	HIS1-3	histone H1-3	linker histone H1 and h5 family protein
Sapur.005G162100.1	39.47851828	1.326634916	0.070550228	18.80412	6.99E-79	5.99E-78	cyan	0.796028713	1.27E-35		AT2G42660.1		Homeodomain-like superfamily protein	Myb-like DNA-binding domain, shaqkyf class protein
Sapur.012G060000.1	20.87515202	1.950663487	0.103737684	18.80381	7.03E-79	6.02E-78	cyan	0.760276255	7.64E-31		AT5G62520.1	SRO5	similar to RCD one 5	inactive poly [ADP-ribose] polymerase SRO4, putative
Sapur.014G097800.1	1345.50348	1.335801421	0.071057819	18.7988	7.73E-79	6.61E-78	cyan	0.788170311	1.71E-34	GO:0005515	AT4G02100.1		Heat shock protein DnaJ with tetratricopeptide repeat	DnaJ heat shock amino-terminal domain protein
Sapur.009G122600.1	15.24254557	5.327954177	0.283488163	18.79427	8.41E-79	7.20E-78	cyan	0.595803986	1.85E-16					hypothetical protein
Sapur.T126600.1	17.96746611	2.251240228	0.119814378	18.7894	9.22E-79	7.88E-78	cyan	0.755612673	2.79E-30	GO:0005681,GO:0000398,GO:0000387	AT1G54385.2		ARM repeat superfamily protein	NA
Sapur.004G045900.1	39.23828699	-1.145334629	0.060959451	-18.7885	9.39E-79	8.02E-78	cyan	-0.845023696	5.48E-44					hypothetical protein
Sapur.016G093600.1	84.09398017	-1.053778167	0.056181536	-18.7567	1.71E-78	1.46E-77	cyan	-0.840015775	5.25E-43	GO:0008270	AT5G05830.1		RING/FYVE/PHD zinc finger superfamily protein	RING-variant domain protein
Sapur.003G021800.1	755.8234079	1.310089612	0.069858711	18.75342	1.82E-78	1.55E-77	cyan	0.73796924	2.89E-28	GO:0016787	AT1G72880.2		Survival protein SurE-like phosphatase/nucleotidase	acid phosphatase, putative
Sapur.009G112000.1	503.0954195	-1.629125597	0.086885203	-18.7503	1.92E-78	1.64E-77	cyan	-0.877995359	1.85E-51	GO:0080085,GO:0045038,GO:0009507,GO:0009416	AT2G47450.1	CAO,CPSRP43	chloroplast signal recognition particle component (CAO)	signal recognition particle 43 kDa protein
Sapur.003G169300.2	18.97570334	-1.520507937	0.081101105	-18.7483	2.00E-78	1.70E-77	cyan	-0.834587965	5.58E-42	GO:0006353	AT4G26370.1		antitermination NusB domain-containing protein	antitermination NusB domain protein
Sapur.T050500.1	758.7580228	1.149101285	0.06129474	18.74714	2.04E-78	1.74E-77	cyan	0.780238435	2.12E-33		AT5G43950.1		Plant protein of unknown function (DUF946)	plant/T6K12-3 protein
Sapur.T089800.1	827.6409418	-0.506146031	0.027000781	-18.7456	2.10E-78	1.79E-77	cyan	-0.840333157	4.56E-43	GO:0006457,GO:0003755,GO:0000413	AT3G01480.1	ATCYP38,CYP38	cyclophilin 38	cytochrome P450 family 38 peptidyl-prolyl cis-trans isomerase
Sapur.009G069300.1	145.1967046	-0.85644383	0.04568932	-18.7449	2.13E-78	1.81E-77	cyan	-0.849218917	7.75E-45					hypothetical protein
Sapur.002G059200.1	226.5182536	-1.037165447	0.055340203	-18.7416	2.27E-78	1.93E-77	cyan	-0.822094213	9.41E-40		AT1G76980.1			hypothetical protein
Sapur.009G119900.1	889.7387621	0.634856228	0.033882932	18.73676	2.48E-78	2.11E-77	cyan	0.811129074	6.16E-38		AT5G21990.1		Tetratricopeptide repeat (TPR)-like superfamily protein	NA
Sapur.002G169600.1	2433.883921	0.896696173	0.047879533	18.72817	2.92E-78	2.47E-77	cyan	0.798211537	6.05E-36		AT3G13410.1			NA
Sapur.009G053900.1	386.7945845	0.610919687	0.032629155	18.72312	3.21E-78	2.72E-77	cyan	0.816315453	8.83E-39		AT5G19380.1	CLT1	CRT (chloroquine-resistance transporter)-like transporter 1	CRT (chloroquine-resistance transporter)-like transporter 1
Sapur.014G084000.1	2196.396618	-0.831671693	0.044441894	-18.7137	3.83E-78	3.24E-77	cyan	-0.832430744	1.39E-41		AT2G46820.1	PSAP,PSI-P,PTAC8,TMP14	photosystem I P subunit	cyanobacterial aminoacyl-tRNA synthetase CAAD domain protein
Sapur.003G045700.1	330.2616402	0.902445317	0.048234412	18.70957	4.14E-78	3.50E-77	cyan	0.801267865	2.11E-36		AT1G02030.1		C2H2-like zinc finger protein	C2H2 zinc-finger protein, putative
Sapur.002G090900.1	704.4247812	-0.944978394	0.050512598	-18.7078	4.28E-78	3.62E-77	cyan	-0.827386146	1.13E-40		AT5G17870.1	PSRP6	plastid-specific 50S ribosomal protein 6	50S ribosomal protein
Sapur.007G024600.1	260.2976861	-1.054959715	0.056396876	-18.706	4.42E-78	3.75E-77	cyan	-0.830167374	3.59E-41		AT3G50685.1			NA
Sapur.009G003400.1	1100.659808	0.76708029	0.041009157	18.7051	4.50E-78	3.81E-77	cyan	0.821806795	1.05E-39		AT2G28110.1	FRA8,IRX7	Exostosin family protein	secondary cell wall-related glycosyltransferase family 47 protein
Sapur.016G100300.1	761.1347689	-1.154627829	0.06175932	-18.6956	5.38E-78	4.54E-77	cyan	-0.82187567	1.03E-39	GO:0016020,GO:0009765	AT3G08940.2	LHCB4.2	light harvesting complex photosystem II	photosystem I light harvesting chlorophyll A/B-binding protein
Sapur.017G122700.1	4.166802526	5.378240155	0.287678519	18.69531	5.41E-78	4.57E-77	cyan	0.68038184	1.12E-22	GO:0005515	AT3G28410.1		F-box/RNI-like superfamily protein	NA
Sapur.004G018600.1	1691.496783	-1.452625963	0.07772295	-18.6898	5.99E-78	5.06E-77	cyan	-0.762764258	3.79E-31	GO:0050662,GO:0003824	AT1G61720.1	BAN	NAD(P)-binding Rossmann-fold superfamily protein	anthocyanidin reductase ANR1-1
Sapur.006G140600.1	496.6024166	0.859836379	0.046015104	18.68596	6.44E-78	5.43E-77	cyan	0.788178152	1.71E-34	GO:0008270,GO:0005622,GO:0005515	AT5G57660.1	ATCOL5,COL5	CONSTANS-like 5	constans-like zinc finger protein
Sapur.016G096300.1	29.31942205	-1.456680405	0.077980188	-18.6801	7.18E-78	6.06E-77	cyan	-0.812220166	4.11E-38		AT3G09085.1		Protein of unknown function (DUF962)	transmembrane protein
Sapur.006G209700.1	30.48645382	6.617857585	0.354440935	18.67126	8.48E-78	7.15E-77	cyan	0.675499067	2.91E-22		AT1G15370.1		SNARE-like superfamily protein	NA
Sapur.002G197400.1	2719.461502	-1.308344559	0.070136145	-18.6544	1.16E-77	9.79E-77	cyan	-0.837795765	1.40E-42	GO:0015979,GO:0009538,GO:0009522	AT2G20260.1	PSAE-2	photosystem I subunit E-2	photosystem I reaction center subunit IV
Sapur.018G025900.1	2979.894654	1.101052818	0.059043075	18.6483	1.30E-77	1.10E-76	cyan	0.805615998	4.55E-37	GO:0006122,GO:0005750	AT5G25450.1		Cytochrome bd ubiquinol oxidase, 14kDa subunit	cytochrome B-c1 complex subunit 7
Sapur.008G025100.1	335.4931174	-0.89509258	0.048014428	-18.6422	1.46E-77	1.23E-76	cyan	-0.845318566	4.79E-44	GO:0003723,GO:0009982,GO:0009451,GO:0001522	AT2G39140.1	SVR1	pseudouridine synthase family protein	pseudouridine synthase
Sapur.014G058200.1	14.96960297	-1.825350361	0.097915812	-18.642	1.47E-77	1.23E-76	cyan	-0.846096909	3.34E-44		AT3G61080.1		Protein kinase superfamily protein	NA
Sapur.005G144000.1	186.4805046	-1.223101341	0.065611194	-18.6417	1.48E-77	1.24E-76	cyan	-0.845254708	4.93E-44	GO:0055114,GO:0016655	AT4G37925.1	NDH-M	subunit NDH-M of NAD(P)H:plastoquinone dehydrogenase complex	NAD(P)H-quinone oxidoreductase subunit M
Sapur.008G141700.1	493.2588159	1.159492189	0.062211921	18.63778	1.59E-77	1.33E-76	cyan	0.777850598	4.42E-33	GO:0016459,GO:0005524,GO:0003774,GO:0005515	AT5G43900.3	ATMYA2,MYA2,XI-2,XI-6	myosin 2	myosin XI, putative
Sapur.007G003100.1	20.44608224	4.498843034	0.241417047	18.63515	1.67E-77	1.40E-76	cyan	0.55385435	5.31E-14	GO:0006468,GO:0005524,GO:0004672,GO:0030246	AT5G10530.1		Concanavalin A-like lectin protein kinase family protein	lectin-domain receptor kinase
Sapur.011G001300.1	633.1684694	-0.844712561	0.045329407	-18.635	1.67E-77	1.40E-76	cyan	-0.828451302	7.30E-41		AT1G61900.1			GPI-anchored protein, putative
Sapur.004G098400.1	12.59619055	6.352789677	0.341175475	18.6203	2.20E-77	1.85E-76	cyan	0.514901256	5.23E-12	GO:0016020,GO:0008375	AT5G16170.1		Core-2/I-branching beta-1,6-N-acetylglucosaminyltransferase family protein	core-2/I-branching enzyme
Sapur.013G116600.1	565.2121687	-0.832369216	0.044713425	-18.6156	2.40E-77	2.01E-76	cyan	-0.852205875	1.86E-45		AT3G59780.1		Rhodanese/Cell cycle control phosphatase superfamily protein	rhodanese/cell cycle control phosphatase-like protein, putative
Sapur.003G154600.1	8.943683576	3.1624681	0.170017836	18.6008	3.17E-77	2.65E-76	cyan	0.763216869	3.33E-31	GO:0005544,GO:0005509	AT5G12380.1	ANNAT8	annexin 8	annexin
Sapur.15WG070100.1	15.51542726	7.030119712	0.377974637	18.59945	3.25E-77	2.72E-76	cyan	0.531829336	7.65E-13	GO:0016021	AT1G18520.1	TET11	tetraspanin11	NA
Sapur.010G083800.1	89.40384418	2.044642497	0.109980658	18.59093	3.81E-77	3.18E-76	cyan	0.763886046	2.75E-31	GO:0009607,GO:0006952	AT1G70830.4	MLP28	MLP-like protein 28	pathogenesis-related protein Bet V I family protein
Sapur.010G194400.1	1310.480017	0.66043097	0.035544353	18.58048	4.62E-77	3.86E-76	cyan	0.813255507	2.80E-38		AT2G38710.1		AMMECR1 family	AMME syndrome 1 protein, putative
Sapur.019G061000.1	3659.08543	1.41385915	0.07613724	18.56988	5.63E-77	4.70E-76	cyan	0.789432575	1.14E-34		AT2G40610.1	ATEXP8.ATEXPA8.ATHEXP A	expansin A8	expansin
Sapur.019G065100.1	261.2463551	-1.043109539	0.056191062	-18.5636	6.33E-77	5.27E-76	cyan	-0.778121958	4.07E-33		AT5G38520.1		alpha/beta-Hydrolases superfamily protein	alpha/beta fold hydrolase
Sapur.008G086800.1	7.731663041	-2.689380376	0.144913525	-18.5585	6.96E-77	5.80E-76	cyan	-0.798550181	5.39E-36		AT5G49320.1		Protein of unknown function (DUF1218)	transmembrane protein, putative
Sapur.007G004900.1	77.18613364	1.839415267	0.099174488	18.54726	8.58E-77	7.15E-76	cyan	0.708423629	3.14E-25		AT1G10150.1		Carbohydrate-binding protein	phloem protein-like protein A10
Sapur.018G071100.1	369.734953	0.732173106	0.039490579	18.54045	9.74E-77	8.11E-76	cyan	0.824883067	3.10E-40	GO:0016773,GO:0005975	AT4G30310.2		FGGY family of carbohydrate kinase	FGGY family of carbohydrate kinase
Sapur.017G044100.1	12.38999203	-2.074811326	0.111986923	-18.5273	1.24E-76	1.03E-75	cyan	-0.818848651	3.34E-39		AT4G13600.1		Carbohydrate-binding X8 domain superfamily protein	carbohydrate-binding module family 43 protein
Sapur.008G164000.1	307.9246601	0.870953414	0.047020201	18.52296	1.35E-76	1.12E-75	cyan	0.810984059	6.50E-38		AT3G21400.1			hypothetical protein
Sapur.014G131300.1	24.40785131	-1.443098645	0.07794653	-18.514	1.59E-76	1.32E-75	cyan	-0.817965434	4.70E-39		AT5G35670.1	iqd33	IQ-domain 33	IQ-domain protein
Sapur.003G029200.1	555.5543514	0.859702403	0.046468204	18.50087	2.03E-76	1.68E-75	cyan	0.806429255	3.40E-37		AT1G52343.1			NA
Sapur.009G016300.1	333.4288459	-0.570647109	0.030847846	-18.4988	2.11E-76	1.75E-75	cyan	-0.821643306	1.12E-39		AT5G13020.1		Emsy N Terminus (ENT)/ plant Tudor-like domains-containing protein	emsy amino-terminus domain protein
Sapur.010G083200.1	173.6282648	-1.296045513	0.070062191	-18.4985	2.12E-76	1.76E-75	cyan	-0.812489966	3.72E-38	GO:0055114,GO:0016655	AT1G70760.1	CRR23	inorganic carbon transport protein-related	NADH dehydrogenase transmembrane subunit
Sapur.009G027100.1	611.1310629	-1.199955174	0.064873895	-18.4967	2.19E-76	1.82E-75	cyan	-0.807568402	2.26E-37		AT3G46780.1	PTAC16	plastid transcriptionally active 16	NAD(P)H-binding family protein
Sapur.002G159900.1	999.4706419	0.559881123	0.030271416	18.49537	2.25E-76	1.86E-75	cyan	0.831197517	2.34E-41		AT2G47490.1	ATNDT1,NDT1	NAD+ transporter 1	NA
Sapur.008G015200.1	92.37715885	3.265457321	0.176563087	18.49456	2.28E-76	1.89E-75	cyan	0.578263648	2.18E-15	GO:0055085,GO:0016021	AT2G16980.2		Major facilitator superfamily protein	hippocampus abundant transcript-like protein
Sapur.013G143200.1	1593.853397	-0.766986443	0.041502892	-18.4803	2.97E-76	2.46E-75	cyan	-0.830059567	3.76E-41		AT5G58250.1			hypothetical protein
Sapur.005G012700.1	8.811862156	3.892365998	0.210705297	18.47303	3.40E-76	2.81E-75	cyan	0.756679598	2.08E-30	GO:0048544,GO:0006468,GO:0005524,GO:0004672	AT4G21390.1	B120	S-locus lectin protein kinase family protein	NA
Sapur.003G124700.1	417.2627263	-0.836554096	0.045309699	-18.463	4.10E-76	3.38E-75	cyan	-0.837684832	1.46E-42	GO:0016787	AT2G35450.1		catalytics;hydrolases	aminocarboxymuconate-semialdehyde decarboxylase, putative
Sapur.001G123600.1	761.1259067	1.056976365	0.057253026	18.46149	4.22E-76	3.47E-75	cyan	0.776703042	6.27E-33		AT1G32740.1		SBP (S-ribonuclease binding protein) family protein	SBP (S-ribonuclease-binding protein) family protein, putative
Sapur.T009000.1	7.615662176	3.685686085	0.199708655	18.45531	4.73E-76	3.89E-75	cyan	0.700348075	1.83E-24					NA
Sapur.005G038100.1	1193.10415	1.176694451	0.063809421	18.44076	6.19E-76	5.09E-75	cyan	0.782310247	1.11E-33	GO:0008270	AT5G28050.2		Cytidine/deoxycytidylate deaminase family protein	deoxycytidylate deaminase (cytidine deaminase), putative
Sapur.002G162400.1	13.86049075	2.67882954	0.145280545	18.43901	6.39E-76	5.25E-75	cyan	0.738703134	2.40E-28		AT4G02090.1			multidrug resistance protein ABC transporter family protein
Sapur.004G105000.1	564.7415676	-0.73011707	0.039599619	-18.4375	6.57E-76	5.40E-75	cyan	-0.84497562	5.60E-44	GO:0005515,GO:0006508,GO:0004252	AT5G39830.1	DEG8,DEGP8	Trypsin family protein with PDZ domain	protease Do-like protein
Sapur.007G079100.1	376.3527759	0.971049387	0.052669061	18.43681	6.66E-76	5.47E-75	cyan	0.789837942	9.95E-35	GO:0008270	AT2G22120.2		RING/FYVE/PHD zinc finger superfamily protein	zinc finger C3HC4 type family protein
Sapur.014G070400.2	7.573343093	4.444278698	0.241113405	18.43232	7.23E-76	5.94E-75	cyan	0.747274084	2.62E-29	GO:0043565,GO:0006355,GO:0003700	AT2G46130.1	ATWRKY43,WRKY43	WRKY DNA-binding protein 43	WRKY transcription factor, putative
Sapur.008G029600.1	345.2696779	-0.65602182	0.035597016	-18.4291	7.67E-76	6.29E-75	cyan	-0.813321949	2.73E-38	GO:0016787,GO:0030145,GO:0003723	AT5G13570.1	ATDCP2,DCP2,TDT	decapping 2	decapping-like protein
Sapur.007G084800.2	9.989609944	-1.956062089	0.106162817	-18.4251	8.26E-76	6.77E-75	cyan	-0.815464456	1.22E-38		AT4G39900.1			plant/T5J17-70 protein, putative
Sapur.011G090600.1	106.4280192	-0.901033876	0.048913102	-18.4211	8.90E-76	7.29E-75	cyan	-0.835175045	4.34E-42	GO:0030170				aluminum resistance family protein
Sapur.009G044500.1	8.99527011	3.497975616	0.189911117	18.41901	9.25E-76	7.58E-75	cyan	0.686325095	3.40E-23	GO:0055114,GO:0016491	AT5G13700.1	APAO,ATPAO1,PAO1	polyamine oxidase 1	polyamine oxidase
Sapur.012G037600.1	860.6770745	1.858048058	0.100896466	18.41539	9.89E-76	8.09E-75	cyan	0.823239093	5.98E-40	GO:0009607,GO:0006952	AT1G24020.2	MLP423	MLP-like protein 423	pathogenesis-related protein bet V I family protein
Sapur.010G034500.1	575.6951866	-0.333495112	0.018115801	-18.4091	1.11E-75	9.09E-75	cyan	-0.823963707	4.49E-40	GO:0006468,GO:0004672	AT5G15080.1		Protein kinase superfamily protein	Serine/Threonine kinase domain protein
Sapur.002G193900.1	265.6878447	0.868575758	0.047217489	18.39521	1.43E-75	1.17E-74	cyan	0.808352404	1.70E-37	GO:0006629	AT3G07400.1		lipase class 3 family protein	lipase
Sapur.010G049200.1	319.7322895	0.730729716	0.039739842	18.38784	1.64E-75	1.34E-74	cyan	0.813576776	2.48E-38		AT3G23980.1	BLI,KOS1	BLISTER	hypothetical protein
Sapur.011G042900.1	144.4853238	-1.234539514	0.067171356	-18.379	1.94E-75	1.58E-74	cyan	-0.828671884	6.66E-41		AT2G34040.1		Apoptosis inhibitory protein 5 (API5)	apoptosis inhibitory protein 5 (API5)
Sapur.017G018300.1	749.0644856	-0.668493713	0.036375765	-18.3774	1.99E-75	1.62E-74	cyan	-0.845036816	5.45E-44		AT4G22720.2		Actin-like ATPase superfamily protein	O-sialoglycoprotein endopeptidase
Sapur.007G041500.1	81.98108271	-0.987943555	0.053763344	-18.3758	2.05E-75	1.67E-74	cyan	-0.839090547	7.91E-43		AT4G36970.1		Remorin family protein	remorin
Sapur.001G022300.1	421.6287802	0.848077948	0.046161681	18.3719	2.21E-75	1.79E-74	cyan	0.775460226	9.15E-33	GO:0045454,GO:0015035,GO:0009055	AT5G13810.1		Glutaredoxin family protein	glutaredoxin family protein
Sapur.002G019900.1	39.78852541	3.13844348	0.17092793	18.36121	2.69E-75	2.18E-74	cyan	0.619611012	5.09E-18		AT1G19900.1		glyoxal oxidase-related protein	galactose oxidase
Sapur.017G056100.1	25.74325856	-1.590503629	0.08666581	-18.3521	3.17E-75	2.57E-74	cyan	-0.813050601	3.02E-38	GO:0055085,GO:0005741	AT3G01280.1	ATVDAC1,VDAC1	voltage dependent anion channel 1	NA
Sapur.013G024600.1	25.75764796	-1.34716113	0.073447947	-18.3417	3.84E-75	3.11E-74	cyan	-0.819983785	2.15E-39		AT5G27440.1			NA
Sapur.15WG070800.1	107.6081979	3.391141324	0.18490536	18.33988	3.98E-75	3.22E-74	cyan	0.610968668	1.94E-17		AT5G55400.1		Actin binding Calponin homology (CH) domain-containing protein	NA
Sapur.15WG025100.1	285.8379588	1.158432202	0.063186736	18.33347	4.47E-75	3.62E-74	cyan	0.746291055	3.40E-29		AT5G16560.1	KAN,KAN1	Homeodomain-like superfamily protein	myb-like DNA-binding domain protein
Sapur.15WG025000.1	1962.1243	1.418553574	0.077385734	18.33094	4.69E-75	3.79E-74	cyan	0.737836142	2.99E-28	GO:0016787	AT3G17790.1	ATACP5,ATPAP17,PAP17	purple acid phosphatase 17	tartrate-resistant acid phosphatase type 5, putative
Sapur.001G019000.1	164.551157	-0.904964974	0.049402478	-18.3182	5.92E-75	4.78E-74	cyan	-0.828586521	6.90E-41		AT4G31560.1	HCF153	high chlorophyll fluorescence 153	hypothetical protein
Sapur.006G212600.1	623.2588284	0.983748846	0.053761362	18.29844	8.52E-75	6.86E-74	cyan	0.767913155	8.61E-32	GO:0016021,GO:0016020	AT4G32140.1		EamA-like transporter family	vacuolar membrane protein
Sapur.15WG066300.1	225.1790222	1.39515237	0.076255632	18.29573	8.95E-75	7.21E-74	cyan	0.831418233	2.13E-41	GO:0016301,GO:0008152,GO:0005524	AT1G26190.1		Phosphoribulokinase / Uridine kinase family	NA
Sapur.001G065200.1	15.35657573	6.221229994	0.340041377	18.29551	8.99E-75	7.23E-74	cyan	0.605687923	4.32E-17	GO:0016021,GO:0015914,GO:0005524,GO:0004012,GO:0000287	AT1G72700.1		ATPase E1-E2 type family protein / haloacid dehalogenase-like hydrolase family protein	phospholipid-transporting ATPase, putative
Sapur.002G122000.1	267.2718537	-0.650854897	0.035574727	-18.2954	9.00E-75	7.24E-74	cyan	-0.821142444	1.37E-39	GO:0008017,GO:0007018,GO:0005524,GO:0003777,GO:0005871	AT1G01950.1	ARK2	armadillo repeat kinesin 2	armadillo repeat kinesin-like protein
Sapur.001G009900.1	13.99731853	4.625689779	0.252876769	18.29227	9.54E-75	7.67E-74	cyan	0.662769147	3.24E-21	GO:0006629	AT3G12120.1	FAD2	fatty acid desaturase 2	NA
Sapur.010G140900.1	8.23914039	4.11483689	0.225111306	18.27912	1.21E-74	9.75E-74	cyan	0.659676359	5.71E-21	GO:0046983,GO:0003677	AT5G65330.1	AGL78	AGAMOUS-like 78	NA
Sapur.003G155800.1	5.693956949	-2.666050934	0.14589279	-18.274	1.33E-74	1.07E-73	cyan	-0.782499512	1.04E-33	GO:0005515	AT3G51930.1		Transducin/WD40 repeat-like superfamily protein	F-box/WD repeat protein 1A
Sapur.006G071300.1	5.641038535	-4.011879505	0.219544842	-18.2736	1.34E-74	1.08E-73	cyan	-0.769367868	5.63E-32	GO:0043565,GO:0006355,GO:0003700	AT5G43290.1	ATWRKY49,WRKY49	WRKY DNA-binding protein 49	WRKY transcription factor, putative
Sapur.15WG126300.1	2212.662594	1.852054961	0.101372476	18.2698	1.44E-74	1.15E-73	cyan	0.755361298	2.99E-30		AT5G61820.1			stress up-regulated Nod 19 protein
Sapur.003G071300.1	44.91857918	1.501071912	0.082179015	18.26588	1.55E-74	1.24E-73	cyan	0.732023445	1.27E-27		AT4G19460.1		UDP-Glycosyltransferase superfamily protein	glycosyltransferase family protein
Sapur.005G063400.1	20.34031012	-1.603748986	0.087819462	-18.2619	1.66E-74	1.33E-73	cyan	-0.788499216	1.54E-34	GO:0008270,GO:0005515	AT1G15100.1	RHA2A	RING-H2 finger A2A	zinc finger, C3HC4 type (RING finger) protein
Sapur.014G102300.1	246.0850726	-0.882649808	0.048361852	-18.251	2.03E-74	1.63E-73	cyan	-0.843904953	9.14E-44	GO:0003676,GO:0017069,GO:0000398	AT2G47580.1	U1A	spliceosomal protein U1A	U1 small nuclear ribonucleoprotein A
Sapur.008G094400.1	390.5765117	-0.833567269	0.045686674	-18.2453	2.25E-74	1.80E-73	cyan	-0.839121084	7.80E-43		AT1G13270.1	MAP1B,MAP1C	methionine aminopeptidase 1B	methionine aminopeptidase
Sapur.007G052200.1	300.5532219	0.868863323	0.047624086	18.2442	2.30E-74	1.84E-73	cyan	0.809996191	9.34E-38		AT4G35987.1		S-adenosyl-L-methionine-dependent methyltransferases superfamily protein	S-adenosyl-L-methionine-dependent methyltransferase domain protein
Sapur.011G109900.1	295.2790609	1.507663192	0.082643473	18.24298	2.35E-74	1.88E-73	cyan	0.729533741	2.34E-27	GO:0055114,GO:0016491,GO:0008270	AT1G72680.1	ATCAD1,CAD1	cinnamyl-alcohol dehydrogenase	NA
Sapur.002G184100.1	2193.423787	0.627295594	0.034395231	18.23787	2.58E-74	2.07E-73	cyan	0.803509118	9.61E-37	GO:0008270,GO:0004553,GO:0005975,GO:0004559,GO:0015923,GO:0006013	AT3G26720.1		Glycosyl hydrolase family 38 protein	glycoside hydrolase family 38 protein
Sapur.004G147400.1	29.01827712	2.986948099	0.16379594	18.23579	2.68E-74	2.14E-73	cyan	0.602459636	6.99E-17					hypothetical protein
Sapur.006G011300.1	13.35079216	1.741222613	0.095497636	18.23315	2.82E-74	2.25E-73	cyan	0.808403277	1.67E-37	GO:0003676	AT3G07810.2		RNA-binding (RRM/RBD/RNP motifs) family protein	heterogeneous nuclear ribonucleoprotein A1
Sapur.018G084800.1	1327.052033	0.81120823	0.044501718	18.22869	3.06E-74	2.44E-73	cyan	0.806886793	2.89E-37		AT2G24290.1		Protein of unknown function (DUF1068)	transmembrane protein, putative
Sapur.004G132500.1	21.70183877	2.094335759	0.11494555	18.22024	3.57E-74	2.85E-73	cyan	0.773660795	1.57E-32		AT2G16900.1		Arabidopsis phospholipase-like protein (PEARLI 4) family	phospholipase-like protein (pearli 4)
Sapur.001G047100.1	154.1879766	1.347600332	0.073976774	18.21653	3.82E-74	3.05E-73	cyan	0.739037826	2.21E-28		AT1G27300.1			hypothetical protein
Sapur.010G090200.1	64.89758399	3.43323536	0.188484661	18.21493	3.93E-74	3.14E-73	cyan	0.488185073	8.84E-11					NA
Sapur.003G068700.1	178.8186446	-0.485461068	0.026656451	-18.2118	4.16E-74	3.32E-73	cyan	-0.80846248	1.63E-37		AT4G19140.1			transmembrane protein, putative
Sapur.007G022900.1	242.2329734	1.424724559	0.078254884	18.20621	4.61E-74	3.67E-73	cyan	0.6943883	6.47E-24		AT5G49555.1		FAD/NAD(P)-binding oxidoreductase family protein	pyridine nucleotide-disulfide oxidoreductase domain protein
Sapur.001G057300.1	528.2712922	0.704609228	0.038716408	18.19924	5.23E-74	4.17E-73	cyan	0.798007729	6.49E-36		AT5G59610.1		Chaperone DnaJ-domain superfamily protein	NA
Sapur.007G045100.1	455.198794	0.569405442	0.03129791	18.19308	5.86E-74	4.66E-73	cyan	0.824503896	3.61E-40	GO:0016021	AT4G39220.1	ATRER1A	Rer1 family protein	RER1A protein
Sapur.014G034500.1	31.27785949	-1.469627221	0.080781754	-18.1926	5.91E-74	4.70E-73	cyan	-0.828363731	7.56E-41	GO:0043565,GO:0006355,GO:0003700,GO:0003677,GO:0005634	AT3G60390.1	HAT3	homeobox-leucine zipper protein 3	homeobox leucine zipper protein
Sapur.012G100900.1	125.0578972	-1.797696704	0.098830063	-18.1898	6.22E-74	4.95E-73	cyan	-0.802235104	1.50E-36	GO:0005975,GO:0004560	AT2G28100.1	ATFUC1,FUC1	alpha-L-fucosidase 1	alpha-L-fucosidase
Sapur.007G021600.1	52.08464992	-1.199599939	0.066000809	-18.1755	8.06E-74	6.41E-73	cyan	-0.837230545	1.79E-42	GO:0006284	AT3G50880.1		DNA glycosylase superfamily protein	base excision DNA repair protein, HhH-GPD family protein
Sapur.005G188200.1	1445.633823	0.661937588	0.036433913	18.16817	9.22E-74	7.33E-73	cyan	0.800516615	2.74E-36	GO:0033177,GO:0015991,GO:0015078	AT1G19910.1	ATVHA-C2.AVA-2PE.AVA-P2	ATPase, F0/V0 complex, subunit C protein	V-type proton ATPase 16 kDa proteolipid subunit c2
Sapur.008G027100.1	361.5566145	-1.037080199	0.057082789	-18.168	9.25E-74	7.35E-73	cyan	-0.80736895	2.43E-37	GO:0008080	AT2G39020.1		Acyl-CoA N-acyltransferases (NAT) superfamily protein	tyramine N-feruloyltransferase, putative
Sapur.003G067600.1	9253.676736	0.629752585	0.034666866	18.16584	9.62E-74	7.65E-73	cyan	0.813077452	2.99E-38	GO:0006468,GO:0005524,GO:0004672	AT4G19110.1		Protein kinase superfamily protein	Serine/Threonine-kinase Mak (male germ cell-associated kinase)-like protein
Sapur.15WG076400.1	76.03822158	-0.928716688	0.051138803	-18.1607	1.06E-73	8.39E-73	cyan	-0.836787171	2.16E-42	GO:0005515	AT2G19540.1		Transducin family protein / WD-40 repeat family protein	NA
Sapur.011G016800.1	19.3689444	-3.321003946	0.18291439	-18.1561	1.15E-73	9.12E-73	cyan	-0.73668637	4.00E-28	GO:0006468,GO:0004672	AT4G05200.1	CRK25	cysteine-rich RLK (RECEPTOR-like protein kinase) 25	NA
Sapur.012G048800.1	8.97879644	4.75820212	0.262094566	18.15452	1.18E-73	9.38E-73	cyan	0.706633143	4.67E-25	GO:0005516	AT5G15430.1		Plant calmodulin-binding protein-related	NA
Sapur.016G210600.1	136.6225482	3.224168602	0.177640832	18.14993	1.29E-73	1.02E-72	cyan	0.671254147	6.58E-22		AT5G40690.1			Hop-interacting protein THI031, putative
Sapur.013G062300.1	7.437121126	3.482361487	0.191897829	18.14696	1.36E-73	1.08E-72	cyan	0.70779897	3.61E-25					NA
Sapur.006G166700.1	647.5924163	0.772279912	0.042593426	18.13143	1.80E-73	1.42E-72	cyan	0.804523501	6.71E-37	GO:0016787	AT5G06340.1	ATNUDX27,NUDX27	nudix hydrolase homolog 27	nudix hydrolase
Sapur.T121600.1	7.280999644	3.636169439	0.200601264	18.12635	1.97E-73	1.56E-72	cyan	0.700688306	1.70E-24					NA
Sapur.007G058700.1	131.7775581	1.440543813	0.079493391	18.12155	2.15E-73	1.70E-72	cyan	0.691555192	1.17E-23	GO:0006355,GO:0003677	AT1G56010.2	anac021,ANAC022,NAC1	NAC domain containing protein 1	NAC transcription factor
Sapur.013G141100.1	7.167132603	-2.70742466	0.149431422	-18.1182	2.29E-73	1.81E-72	cyan	-0.831468276	2.09E-41		AT2G40630.1		Uncharacterised conserved protein (UCP030365)	hypothetical protein
Sapur.010G010400.1	239.1992942	-0.510877891	0.028198254	-18.1174	2.32E-73	1.84E-72	cyan	-0.828570658	6.95E-41		AT5G18750.1		DNAJ heat shock N-terminal domain-containing protein	NA
Sapur.012G027600.1	281.2000805	1.332830404	0.073592916	18.11085	2.62E-73	2.07E-72	cyan	0.740541567	1.51E-28		AT5G16560.1	KAN,KAN1	Homeodomain-like superfamily protein	kanadi-like transcription factor, putative
Sapur.006G119100.1	2617.119689	-1.113303525	0.06147247	-18.1106	2.63E-73	2.08E-72	cyan	-0.798153597	6.17E-36	GO:0016020,GO:0015979,GO:0009523	AT2G06520.1	PSBX	photosystem II subunit X	ultraviolet-B-repressible protein
Sapur.001G110600.1	190.1985695	1.965368131	0.108538488	18.10757	2.78E-73	2.19E-72	cyan	0.717725143	3.83E-26	GO:0043531	AT3G07040.1	RPM1,RPS3	NB-ARC domain-containing disease resistance protein	NBS-LRR type disease resistance protein
Sapur.15WG101300.1	395.0674365	1.697478303	0.093795875	18.09758	3.33E-73	2.63E-72	cyan	0.773606208	1.60E-32	GO:0055114,GO:0016491	AT5G50160.1	ATFRO8,FRO8	ferric reduction oxidase 8	ferric-chelate reductase, putative
Sapur.15WG117400.1	1963.17469	-0.609581565	0.033700684	-18.0881	3.95E-73	3.11E-72	cyan	-0.827400617	1.12E-40	GO:0030170,GO:0008483	AT3G48730.1	GSA2	glutamate-1-semialdehyde 2,1-aminomutase 2	glutamate-1-semialdehyde 2%2C1-aminomutase
Sapur.003G163800.1	172.5864616	-1.504773373	0.083208256	-18.0844	4.23E-73	3.33E-72	cyan	-0.876921116	3.50E-51		AT1G55210.2		Disease resistance-responsive (dirigent-like protein) family protein	dirigent protein
Sapur.009G055000.1	705.2040529	0.690295726	0.038175846	18.082	4.42E-73	3.48E-72	cyan	0.81026347	8.47E-38	GO:0008270,GO:0005515	AT5G19430.1		RING/U-box superfamily protein	RING finger protein
Sapur.002G108900.1	166.7863499	-0.772932298	0.042754314	-18.0785	4.71E-73	3.71E-72	cyan	-0.83475834	5.19E-42		AT1G47740.2		PPPDE putative thiol peptidase family protein	PPPDE thiol peptidase family protein, putative
Sapur.016G093200.1	149.9627877	1.636026278	0.090540346	18.06958	5.53E-73	4.35E-72	cyan	0.826872962	1.39E-40	GO:0016758,GO:0008152	AT1G22400.1	ATUGT85A1,UGT85A1	UDP-Glycosyltransferase superfamily protein	UDP-glucosyltransferase family protein
Sapur.017G116600.1	2189.031445	1.255403085	0.069489559	18.06607	5.90E-73	4.63E-72	cyan	0.741500211	1.18E-28		AT5G52510.1	SCL8	SCARECROW-like 8	GRAS family transcription factor
Sapur.018G106200.1	180.511718	1.412306071	0.078179565	18.0649	6.02E-73	4.73E-72	cyan	0.777621102	4.74E-33					hypothetical protein
Sapur.017G105600.1	100.2864597	1.477084781	0.081775561	18.06267	6.27E-73	4.93E-72	cyan	0.761752271	5.04E-31		AT3G03350.2		NAD(P)-binding Rossmann-fold superfamily protein	NA
Sapur.005G162400.1	559.0565867	-1.073038269	0.059411346	-18.0612	6.44E-73	5.06E-72	cyan	-0.823588024	5.21E-40	GO:0010207	AT1G03600.1	PSB27	photosystem II family protein	NA
Sapur.016G260900.1	48.11075029	-1.03617645	0.0573707	-18.0611	6.46E-73	5.07E-72	cyan	-0.816734006	7.53E-39		AT1G52920.1	GCR2,GPCR	G protein coupled receptor	lanthionine synthetase C-like protein
Sapur.012G042600.1	35.87194332	-1.683977977	0.093292611	-18.0505	7.82E-73	6.13E-72	cyan	-0.791605271	5.57E-35		AT1G49010.1		Duplicated homeodomain-like superfamily protein	MYB transcription factor, putative
Sapur.001G067000.1	626.0481132	0.572328671	0.031727174	18.03907	9.62E-73	7.53E-72	cyan	0.785452133	4.10E-34	GO:0019236,GO:0007186,GO:0016021	AT5G42090.1		Lung seven transmembrane receptor family protein	lung seven transmembrane receptor
Sapur.002G144400.1	59.76909441	1.274198266	0.070658708	18.03314	1.07E-72	8.38E-72	cyan	0.789448017	1.13E-34		AT4G18230.1			UDP-N-acetylglucosamine transferase subunit ALG14, putative
Sapur.009G028800.1	8.290901886	2.602446614	0.144343828	18.0295	1.14E-72	8.95E-72	cyan	0.741291984	1.24E-28	GO:0035434,GO:0016021,GO:0005375	AT2G26975.1		Ctr copper transporter family	copper transporter
Sapur.005G110900.1	402.0260653	0.609331435	0.033829633	18.01177	1.58E-72	1.23E-71	cyan	0.812656799	3.50E-38	GO:0052726,GO:0052725,GO:0047325,GO:0032957,GO:0005622,GO:0005524,GO:000028	AT4G08170.2		Inositol 1,3,4-trisphosphate 5/6-kinase family protein	inositol 1, 3, 4-trisphosphate 5/6-kinase, putative
Sapur.016G007100.1	218.5289906	-0.532333064	0.02957221	-18.0011	1.91E-72	1.49E-71	cyan	-0.842887722	1.45E-43	GO:0008270	AT3G07740.1	ADA2A.ATADA2A.HAC10.HX	homolog of yeast ADA2 2A	NA

Sapur.017G085900.1	60.17564051	-1.181065133	0.065662955	-17.9868	2.47E-72	1.93E-71	cyan	-0.823323886	5.79E-40	GO:0006351,GO:0003899,GO:0003677	AT5G15700.1		DNA/RNA polymerases superfamily protein	DNA-directed RNA polymerase
Sapur.016G309200.1	1769.243121	-1.107724735	0.061595519	-17.9839	2.61E-72	2.03E-71	cyan	-0.847636327	1.63E-44	GO:0016020,GO:0015979,GO:0009522	AT1G55670.1	PSAG	photosystem I subunit G	photosystem I reaction center subunit V
Sapur.013G104600.1	385.4402688	0.697476063	0.038801899	17.97531	3.04E-72	2.37E-71	cyan	0.813508093	2.55E-38	GO:0006468,GO:0004672	AT4G09570.1	ATCPK4,CPK4	calcium-dependent protein kinase 4	calcium-dependent kinase
Sapur.004G036800.1	10.49190052	-2.913018596	0.162056823	-17.9753	3.04E-72	2.37E-71	cyan	-0.830960019	2.58E-41					NA
Sapur.006G143400.1	1937.595109	0.608822831	0.033871854	17.9743	3.10E-72	2.41E-71	cyan	0.79886359	4.84E-36		AT1G17890.1	GER2	NAD(P)-binding Rossmann-fold superfamily protein	GDP-L-fucose synthase
Sapur.006G166200.1	778.3652766	0.774644659	0.043097569	17.97421	3.10E-72	2.42E-71	cyan	0.788908986	1.35E-34		AT3G11670.1	DGD1	UDP-Glycosyltransferase superfamily protein	digalactosyldiacylglycerol synthase
Sapur.014G100800.1	3.228618451	5.132265431	0.285572562	17.97184	3.24E-72	2.52E-71	cyan	0.691143339	1.27E-23		AT5G64260.1	EXL2	EXORDIUM like 2	phosphate-induced protein 1 motif protein
Sapur.005G157000.1	693.5998529	0.71493493	0.039789755	17.96781	3.48E-72	2.71E-71	cyan	0.790767606	7.34E-35	GO:0055114,GO:0016491,GO:0006633,GO:0005506	AT1G14290.1	SBH2	sphingoid base hydroxylase 2	SUR2 hydroxylase/desaturase, putative
Sapur.008G059800.1	983.0017901	0.514266004	0.028640792	17.95572	4.33E-72	3.36E-71	cyan	0.820652091	1.66E-39	GO:0006886	AT3G56190.1	ALPHA-SNAP2,ASNAP	alpha-soluble NSF attachment protein 2	alpha-soluble NSF attachment protein
Sapur.010G115200.1	107.6610084	-1.353747753	0.075421834	-17.949	4.88E-72	3.79E-71	cyan	-0.797116162	8.80E-36		AT1G47970.1			hypothetical protein
Sapur.005G118700.1	439.6723074	0.853494737	0.047575005	17.93998	5.75E-72	4.46E-71	cyan	0.796025641	1.27E-35	GO:0008152,GO:0003824	AT2G22570.1	ATNIC1,NIC1	nicotinamidase 1	NA
Sapur.008G060600.1	400.2607407	-1.437070395	0.080146025	-17.9307	6.80E-72	5.27E-71	cyan	-0.801296616	2.09E-36	GO:0016787.GO:0005524.GO:0003677.GO:0005515.GO:0016891.GO:0006396.GO:000452	AT3G03300.3	ATDCL2,DCL2	dicer-like 2	endoribonuclease dicer-like protein
Sapur.005G194700.1	144.9084318	-0.922514284	0.051458284	-17.9274	7.20E-72	5.58E-71	cyan	-0.84250318	1.73E-43		AT1G11320.1			hypothetical protein
Sapur.013G061500.1	5.039829573	4.828760267	0.269359384	17.92683	7.28E-72	5.64E-71	cyan	0.732312183	1.19E-27					NA
Sapur.013G008100.1	228.119359	-0.753306075	0.042025581	-17.9249	7.53E-72	5.83E-71	cyan	-0.821483805	1.20E-39		AT1G56423.1			hypothetical protein
Sapur.004G176900.1	839.0098102	0.574482532	0.032083948	17.90561	1.07E-71	8.24E-71	cyan	0.806452984	3.37E-37		AT5G01010.2			proteinase inhibitor I4, serpin; emp24/gp25L/p24
Sapur.016G224400.1	7.039494589	3.917795079	0.218838429	17.90268	1.12E-71	8.68E-71	cyan	0.75716719	1.82E-30	GO:0016021,GO:0005215	AT4G18210.1	ATPUP10,PUP10	purine permease 10	purine permease, putative
Sapur.005G162900.1	2011.782258	-1.157059432	0.064636945	-17.9009	1.16E-71	8.96E-71	cyan	-0.818266213	4.18E-39	GO:0019898,GO:0015979,GO:0009654,GO:0009523,GO:0005509	AT1G06680.1	OE23,OEE2,PSBP-1,PSII-P	photosystem II subunit P-1	oxygen-evolving enhancer protein
Sapur.004G171900.1	24.98742999	1.926685649	0.107659139	17.89616	1.26E-71	9.75E-71	cyan	0.765072648	1.96E-31					NA
Sapur.018G045700.1	38.6029169	2.548715945	0.142422907	17.89541	1.28E-71	9.88E-71	cyan	0.855026042	4.68E-46	GO:0009733	AT5G20810.1		SAUR-like auxin-responsive protein family	SAUR-like auxin-responsive protein
Sapur.006G069700.1	488.2054136	0.712266673	0.03980285	17.89487	1.29E-71	9.97E-71	cyan	0.800511795	2.74E-36		AT5G02320.1	ATMYB3R5,MYB3R-5	myb domain protein 3r-5	Myb transcription factor
Sapur.016G011100.1	142.6201653	1.819560131	0.101740894	17.88426	1.56E-71	1.21E-70	cyan	0.706003451	5.36E-25	GO:0005737,GO:0005094	AT3G07880.1	SCN1	Immunoglobulin E-set superfamily protein	Rho GDP-dissociation inhibitor, putative
Sapur.005G049700.1	322.5495243	1.290927453	0.072203611	17.87899	1.72E-71	1.32E-70	cyan	0.780406823	2.01E-33		AT5G63970.1		Copine (Calcium-dependent phospholipid-binding protein) family	copine (calcium-dependent phospholipid-binding protein) family protein
Sapur.007G068500.1	318.2419263	-1.110196022	0.062098391	-17.878	1.75E-71	1.35E-70	cyan	-0.783851693	6.82E-34	GO:0045454,GO:0015035,GO:0006662	AT1G50320.1	ATHX,ATX,THX	thioredoxin X	thioredoxin
Sapur.012G022200.1	225.4442197	0.838111	0.046883238	17.87656	1.80E-71	1.38E-70	cyan	0.771093525	3.38E-32	GO:0005515	AT5G52880.1		F-box family protein	F-box protein
Sapur.016G263300.1	41.90982016	7.985338782	0.446844219	17.87052	2.00E-71	1.54E-70	cyan	0.571522009	5.41E-15		AT5G53742.1		Protein of unknown function (DUF1278)	prolamin-like protein
Sapur.016G035500.1	14.06151471	-2.441635213	0.136646798	-17.8682	2.09E-71	1.60E-70	cyan	-0.754095105	4.22E-30		AT3G50120.1		Plant protein of unknown function (DUF247)	transmembrane protein, putative
Sapur.018G002600.1	13.83400472	-2.456850232	0.137515179	-17.866	2.17E-71	1.67E-70	cyan	-0.799384626	4.05E-36					NA
Sapur.004G106100.1	105.6659172	-1.01472628	0.056807056	-17.8627	2.30E-71	1.77E-70	cyan	-0.837909492	1.33E-42	GO:0016887,GO:0005524,GO:0019538	AT5G15450.1	APG6,CLPB-P,CLPB3	casein lytic proteinase B3	chaperone protein ClpB
Sapur.016G039700.1	233.5441752	1.391844106	0.077922889	17.86181	2.34E-71	1.80E-70	cyan	0.77229559	2.37E-32		AT5G03990.1			hypothetical protein
Sapur.004G038600.1	492.7375472	0.81933403	0.045881846	17.85748	2.53E-71	1.94E-70	cyan	0.80522344	5.23E-37	GO:0008641,GO:0006914,GO:0005737	AT5G45900.1	APG7.ATAPG7.ATATG7.ATG	ThiF family protein	ubiquitin-like modifier-activating enzyme ATG7
Sapur.016G183300.1	54.55565091	-0.955274422	0.053496652	-17.8567	2.56E-71	1.97E-70	cyan	-0.825676806	2.26E-40		AT3G21060.1		Transducin/WD40 repeat-like superfamily protein	WD domain, G-beta repeat protein
Sapur.014G019800.1	13.20685075	6.608931995	0.370205427	17.85207	2.79E-71	2.14E-70	cyan	0.539610283	3.05E-13					NA
Sapur.002G034200.1	414.9732877	-1.228192259	0.068843188	-17.8404	3.43E-71	2.63E-70	cyan	-0.851393907	2.75E-45	GO:0003697,GO:0006260	AT1G47720.1	OSB1	Primosome PriB/single-strand DNA-binding	single-stranded DNA-binding protein
Sapur.T142100.1	6.609085727	3.671929181	0.205976041	17.82697	4.36E-71	3.34E-70	cyan	0.72213336	1.37E-26					NA
Sapur.016G010500.1	373.6347433	0.643577676	0.036129373	17.81314	5.59E-71	4.27E-70	cyan	0.807529699	2.29E-37	GO:0005515,GO:0004842	AT4G12570.1	UPL5	ubiquitin protein ligase 5	E3 ubiquitin-protein ligase UPL5
Sapur.003G061600.1	3302.346194	1.101865682	0.061906885	17.79876	7.23E-71	5.52E-70	cyan	0.781350179	1.50E-33	GO:0016887,GO:0005524,GO:0055085,GO:0042626,GO:0016021,GO:0006810	AT4G25960.1	PGP2	P-glycoprotein 2	ABC transporter family B protein, group MDR/PGP protein PpABCB18 protein
Sapur.009G075900.1	105.3233004	-1.002256562	0.056312592	-17.7981	7.31E-71	5.58E-70	cyan	-0.8055363	4.68E-37		AT4G34190.1	43709	stress enhanced protein 1	stress enhanced protein
Sapur.017G057700.1	9.964857699	-1.911488394	0.107412833	-17.7957	7.63E-71	5.82E-70	cyan	-0.818628681	3.64E-39		AT3G27090.1		DCD (Development and Cell Death) domain protein	transmembrane protein, putative
Sapur.15WG114500.1	50.26969813	1.254314885	0.070498199	17.79216	8.13E-71	6.21E-70	cyan	0.812293645	4.00E-38		AT5G53150.1		DNAJ heat shock N-terminal domain-containing protein	curved DNA-binding protein, putative
Sapur.016G113000.1	502.8285011	0.527066098	0.029624261	17.7917	8.19E-71	6.25E-70	cyan	0.818247446	4.21E-39	GO:0006886	AT3G56190.1	ALPHA-SNAP2,ASNAP	alpha-soluble NSF attachment protein 2	alpha-soluble NSF attachment protein
Sapur.006G107200.1	2082.30078	0.8682889	0.048810945	17.78882	8.63E-71	6.58E-70	cyan	0.795194699	1.68E-35		AT2G37110.1		PLAC8 family protein	cell number regulator
Sapur.016G049600.1	3.745636286	5.121717433	0.287956488	17.78643	9.00E-71	6.86E-70	cyan	0.692600901	9.39E-24	GO:0006355,GO:0003677	AT1G69490.1	ANAC029,ATNAP,NAP	NAC-like, activated by AP3/PI	NAC2, putative
Sapur.001G107800.1	197.3547136	1.993960215	0.112165045	17.77702	1.06E-70	8.11E-70	cyan	0.776300398	7.09E-33		AT5G45410.1			transmembrane protein, putative
Sapur.002G066200.1	32.80411061	1.620790584	0.091175861	17.77653	1.07E-70	8.18E-70	cyan	0.735922091	4.84E-28	GO:0030127,GO:0008270,GO:0006888,GO:0006886	AT4G32640.1		Sec23/Sec24 protein transport family protein	NA
Sapur.018G020900.1	335.3924853	1.03210899	0.058093721	17.76627	1.29E-70	9.81E-70	cyan	0.812374061	3.88E-38	GO:0016021,GO:0016020	AT4G32140.1		EamA-like transporter family	vacuolar membrane protein
Sapur.014G141900.1	62.1695619	-1.139282152	0.064129096	-17.7654	1.31E-70	9.95E-70	cyan	-0.833504346	8.85E-42		AT5G48330.2		Regulator of chromosome condensation (RCC1) family protein	chromosome condensation regulator RCC1 repeat protein
Sapur.013G108500.1	179.0156088	-1.159368265	0.065274757	-17.7614	1.41E-70	1.07E-69	cyan	-0.818966329	3.19E-39	GO:0046933,GO:0015986	AT4G09650.1	ATPD	ATP synthase delta-subunit gene	ATP synthase F1, delta subunit
Sapur.006G178000.1	976.4406559	0.907059062	0.051069791	17.76117	1.41E-70	1.07E-69	cyan	0.763583759	3.00E-31	GO:0008270,GO:0005515	AT5G20885.1		RING/U-box superfamily protein	NA
Sapur.016G111200.1	288.3263637	-0.837821153	0.047187567	-17.7551	1.57E-70	1.19E-69	cyan	-0.836533831	2.42E-42	GO:0005515	AT5G01660.1			central domain galactose oxidase
Sapur.001G128300.1	932.7967926	0.535448936	0.03015854	17.75447	1.59E-70	1.21E-69	cyan	0.806620235	3.18E-37	GO:0098599	AT3G60340.1		alpha/beta-Hydrolases superfamily protein	palmitoyl-protein thioesterase
Sapur.016G144800.1	1431.175212	-1.539829059	0.086775924	-17.7449	1.89E-70	1.43E-69	cyan	-0.825677062	2.26E-40		AT1G51400.1		Photosystem II 5 kD protein	photosystem II 5 kDa protein
Sapur.006G065900.1	14.46132037	-2.048577587	0.115446697	-17.7448	1.89E-70	1.43E-69	cyan	-0.779776385	2.44E-33					hypothetical protein
Sapur.004G120600.1	542.9571297	0.817653622	0.046087386	17.74138	2.01E-70	1.52E-69	cyan	0.813305111	2.75E-38		AT4G34630.1			hypothetical protein
Sapur.017G076700.1	211.6283195	0.724009213	0.040811567	17.74029	2.05E-70	1.55E-69	cyan	0.801801827	1.75E-36	GO:0005515	AT5G39250.1		F-box family protein	Non-S-locus F-box protein, putative
Sapur.017G072200.1	804.8340782	0.634957698	0.0358055	17.73352	2.31E-70	1.75E-69	cyan	0.788042022	1.78E-34	GO:0016020,GO:0006886	AT5G39510.1	ATVTI11.ATVTI1A.SGR4.VTI	Vesicle transport v-SNARE family protein	vesicle transport V-SNARE protein
Sapur.008G164900.1	9.892303927	4.59630618	0.259195612	17.73296	2.33E-70	1.76E-69	cyan	0.663972083	2.59E-21					NA
Sapur.012G066600.1	81.1965254	-1.051161094	0.059296134	-17.7273	2.58E-70	1.95E-69	cyan	-0.843260561	1.23E-43		AT5G14420.2	RGLG2	RING domain ligase2	E3 ubiquitin-protein ligase RGLG2
Sapur.009G054500.1	280.315207	0.993253434	0.056047244	17.72172	2.85E-70	2.15E-69	cyan	0.749194216	1.58E-29		AT5G13500.1			NA
Sapur.005G129000.1	44.62812014	-1.479472653	0.083503471	-17.7175	3.07E-70	2.32E-69	cyan	-0.821736075	1.08E-39		AT1G78190.1		Trm112p-like protein	NA
Sapur.15WG137300.1	558.9571106	0.753871253	0.042561064	17.7127	3.35E-70	2.52E-69	cyan	0.782261764	1.13E-33		AT4G37880.1		LisH/CRA/RING-U-box domains-containing protein	sporulation protein RMD5, putative
Sapur.003G165200.1	2419.617594	0.380215731	0.021494439	17.68903	5.09E-70	3.83E-69	cyan	0.811132058	6.15E-38	GO:0016787	AT2G42500.1	PP2A-3	protein phosphatase 2A-3	serine/threonine-protein phosphatase PP2A catalytic subunit
Sapur.006G001000.1	6.318568223	6.119934227	0.346065349	17.68433	5.54E-70	4.16E-69	cyan	0.589118451	4.82E-16	GO:0006508,GO:0004252	AT5G59810.1	ATSBT5.4,SBT5.4	Subtilase family protein	NA
Sapur.T068100.1	11.7639928	2.243507218	0.126879159	17.68224	5.75E-70	4.32E-69	cyan	0.768326352	7.63E-32					NA
Sapur.005G088900.1	744.0250078	-1.197498708	0.067733558	-17.6795	6.03E-70	4.53E-69	cyan	-0.777722755	4.59E-33		AT2G17850.1		Rhodanese/Cell cycle control phosphatase superfamily protein	thiosulfate sulfurtransferase
Sapur.013G126100.1	21.70724564	3.314762888	0.18751149	17.67765	6.23E-70	4.68E-69	cyan	0.574778695	3.49E-15		AT4G28250.1	ATEXPB3.ATHEXP BETA 1.6.	expansin B3	expansin-B3 protein
Sapur.008G034800.1	640.2821133	-0.915610929	0.051817996	-17.6697	7.17E-70	5.38E-69	cyan	-0.805068682	5.53E-37	GO:0042254,GO:0005622	AT5G13510.1		Ribosomal protein L10 family protein	50S ribosomal protein L10
Sapur.016G150300.1	227.2053684	0.857391705	0.048532988	17.66616	7.64E-70	5.73E-69	cyan	0.770294976	4.28E-32		AT5G28040.1		DNA-binding storekeeper protein-related transcriptional regulator	NA
Sapur.014G134800.1	21.68535338	-1.863884762	0.105522669	-17.6634	8.03E-70	6.02E-69	cyan	-0.82901245	5.79E-41		AT4G16447.1			hypothetical protein
Sapur.15WG134000.1	47.45607049	-1.61473458	0.091449224	-17.6572	8.96E-70	6.72E-69	cyan	-0.787794497	1.93E-34	GO:0005524	AT5G18820.1	EMB3007	TCP-1/cpn60 chaperonin family protein	NA
Sapur.003G129000.1	72.28856473	-0.793396125	0.04493561	-17.6563	9.10E-70	6.82E-69	cyan	-0.810782439	7.00E-38		AT1G27385.1			DUF493 family protein
Sapur.004G012200.1	2637.100566	1.457190947	0.082569067	17.64815	1.05E-69	7.87E-69	cyan	0.718908524	2.91E-26		AT1G61800.1	ATGPT2,GPT2	glucose-6-phosphate/phosphate translocator 2	glucose-6-phosphate/phosphate translocator
Sapur.007G078400.1	26.79169929	-1.691489609	0.095859416	-17.6455	1.10E-69	8.25E-69	cyan	-0.78056481	1.91E-33		AT4G39630.1			hypothetical protein
Sapur.010G167800.1	400.9123886	1.107498663	0.062802599	17.6346	1.34E-69	9.98E-69	cyan	0.764337506	2.42E-31	GO:0055085,GO:0016021,GO:0008324,GO:0006812	AT2G39450.1	ATMTP11,MTP11	Cation efflux family protein	metal tolerance protein
Sapur.004G154400.1	136.1901677	-1.03590428	0.058749506	-17.6326	1.39E-69	1.03E-68	cyan	-0.822796107	7.13E-40	GO:0005515	AT4G35270.1		Plant regulator RWP-RK family protein	NA
Sapur.016G288900.1	7.137672201	-2.4490366	0.138949295	-17.6254	1.57E-69	1.17E-68	cyan	-0.818590329	3.69E-39		AT1G64700.1			DUF4228 domain protein
Sapur.008G168200.1	3.025241686	-4.596210904	0.260824245	-17.6219	1.67E-69	1.25E-68	cyan	-0.755523871	2.86E-30	GO:0046983	AT3G06120.1	MUTE	basic helix-loop-helix (bHLH) DNA-binding superfamily protein	HLH DNA-binding domain protein, putative
Sapur.002G187300.1	422.010499	0.615390198	0.034930723	17.61745	1.81E-69	1.35E-68	cyan	0.816777241	7.40E-39	GO:0008270	AT3G24800.1	PRT1	proteolysis 1	E3 ubiquitin-protein ligase PRT1, putative
Sapur.009G031600.1	925.6901752	-0.980727961	0.055672425	-17.616	1.86E-69	1.38E-68	cyan	-0.838376746	1.08E-42		AT2G38090.1		Duplicated homeodomain-like superfamily protein	NA
Sapur.017G052000.1	3.368370365	5.074704691	0.2882316	17.60634	2.20E-69	1.64E-68	cyan	0.66872865	1.06E-21					NA
Sapur.003G042000.1	2516.357304	-1.415825773	0.080421828	-17.605	2.26E-69	1.68E-68	cyan	-0.797643898	7.35E-36	GO:0004857,GO:0042545,GO:0030599,GO:0005618	AT3G14310.1	ATPME3,PME3	pectin methylesterase 3	pectinesterase/pectinesterase inhibitor, putative
Sapur.019G017400.1	18.91903893	1.611164425	0.091535056	17.60161	2.39E-69	1.78E-68	cyan	0.779428212	2.72E-33	GO:0005515,GO:0006468,GO:0004672	AT3G14840.2		Leucine-rich repeat transmembrane protein kinase	NA
Sapur.012G068600.1	10.18891508	-2.447991258	0.139090613	-17.6	2.46E-69	1.83E-68	cyan	-0.808674409	1.51E-37		AT3G48420.1		Haloacid dehalogenase-like hydrolase (HAD) superfamily protein	NA
Sapur.014G005000.1	308.2085116	-0.715605556	0.040681651	-17.5904	2.92E-69	2.17E-68	cyan	-0.805074462	5.52E-37					hypothetical protein
Sapur.008G119100.1	550.7440007	-0.662143288	0.037643737	-17.5897	2.95E-69	2.19E-68	cyan	-0.823374107	5.67E-40	GO:0016020	AT1G14270.1		CAAX amino terminal protease family protein	CAAX amino terminal protease family protein, putative
Sapur.017G041700.1	289.5592076	0.886412453	0.050408786	17.58448	3.24E-69	2.40E-68	cyan	0.765383703	1.79E-31		AT1G80570.2		RNI-like superfamily protein	NA
Sapur.014G003800.2	7.64484704	-2.839763709	0.16151122	-17.5825	3.36E-69	2.49E-68	cyan	-0.774376841	1.27E-32	GO:0006355,GO:0005634,GO:0005524,GO:0032502,GO:0006351	AT2G22840.1	AtGRF1,GRF1	growth-regulating factor 1	growth-regulating factor, putative
Sapur.006G001300.1	62.34247721	2.130005035	0.121208063	17.57313	3.96E-69	2.93E-68	cyan	0.712592969	1.24E-25		AT4G05030.1		Copper transport protein family	heavy metal transport/detoxification protein, putative
Sapur.016G169100.1	79.28166069	1.25083146	0.07122952	17.56058	4.94E-69	3.66E-68	cyan	0.798858917	4.85E-36		AT4G37880.1		LisH/CRA/RING-U-box domains-containing protein	sporulation protein RMD5, putative
Sapur.15WG101900.1	4590.684536	0.612775197	0.034907095	17.55446	5.50E-69	4.07E-68	cyan	0.816509655	8.20E-39	GO:0005515,GO:0008270	AT4G24690.1		ubiquitin-associated (UBA)/TS-N domain-containing protein / octicosapeptide/Phox/Bemp1 (PB1) domain-contain	zinc ion-binding protein, putative
Sapur.008G085700.1	1856.090936	1.008006071	0.057424932	17.55346	5.60E-69	4.14E-68	cyan	0.777254456	5.30E-33	GO:0008152,GO:0003824	AT3G16910.1	AAE7,ACN1	acyl-activating enzyme 7	acyl-CoA synthetase
Sapur.004G167800.1	2469.880168	1.063495887	0.060589644	17.55244	5.70E-69	4.22E-68	cyan	0.747699377	2.34E-29	GO:0007165	AT5G58380.1	CIPK10,PKS2,SIP1,SNRK3.8	SOS3-interacting protein 1	CBL-interacting Serine/Threonine-kinase, putative
Sapur.006G173100.2	16.0830055	-1.706489005	0.097237829	-17.5496	5.99E-69	4.43E-68	cyan	-0.755487627	2.88E-30	GO:0006614,GO:0005525,GO:0048500,GO:0008312	AT5G03940.1	54CP.CPSRP54.FFC.SRP54CF	chloroplast signal recognition particle 54 kDa subunit	signal recognition particle 54 kDa protein
Sapur.019G040700.1	12.48137195	-2.46607013	0.140521849	-17.5494	6.01E-69	4.45E-68	cyan	-0.817749074	5.10E-39		AT1G69560.1	ATMYB105,LOF2,MYB105	myb domain protein 105	MYB-like DNA-binding domain protein
Sapur.016G139100.1	168.1236889	-1.663148233	0.094796008	-17.5445	6.55E-69	4.84E-68	cyan	-0.746806237	2.97E-29		AT2G38090.1		Duplicated homeodomain-like superfamily protein	MYB transcription factor
Sapur.008G125400.1	434.1380515	-0.393493657	0.022429846	-17.5433	6.69E-69	4.94E-68	cyan	-0.818113397	4.44E-39	GO:0016593,GO:0016570,GO:0006368	AT3G22590.1	CDC73,PHP	PLANT HOMOLOGOUS TO PARAFIBROMIN	RNA polymerase II assessory factor cdc73p
Sapur.003G088100.1	220.0498078	1.629369762	0.092902778	17.53844	7.29E-69	5.38E-68	cyan	0.744790722	5.03E-29	GO:0016021,GO:0004252,GO:0006508	AT1G63120.1	ATRBL2,RBL2	RHOMBOID-like 2	NA
Sapur.008G097900.1	124.3864977	1.649263668	0.094045097	17.53694	7.48E-69	5.52E-68	cyan	0.683066842	6.55E-23		AT1G13410.1		Tetratricopeptide repeat (TPR)-like superfamily protein	NA
Sapur.010G177100.2	86.63018371	1.288937181	0.073505639	17.53522	7.72E-69	5.69E-68	cyan	0.669894801	8.53E-22	GO:0006629	AT2G39220.1	PLA IIB,PLP6	PATATIN-like protein 6	patatin-like phospholipase
Sapur.006G151600.1	51.47426777	1.196110308	0.068219456	17.53327	7.98E-69	5.89E-68	cyan	0.786965295	2.53E-34	GO:0051260	AT2G24240.1		BTB/POZ domain with WD40/YVTN repeat-like protein	potassium channel tetramerization domain protein
Sapur.010G017300.1	466.977448	0.522232045	0.029788529	17.53131	8.26E-69	6.09E-68	cyan	0.822122688	9.31E-40	GO:0006468,GO:0005524,GO:0004672	AT5G18610.1		Protein kinase superfamily protein	Serine/Threonine kinase PBS1, putative
Sapur.018G012700.1	513.6881355	0.562307828	0.032104986	17.51466	1.11E-68	8.15E-68	cyan	0.821197366	1.34E-39	GO:0008270	AT5G10790.1	UBP22	ubiquitin-specific protease 22	ubiquitin carboxyl-terminal hydrolase
Sapur.008G082200.1	5.659416617	4.425850064	0.252771633	17.50928	1.22E-68	8.96E-68	cyan	0.771342619	3.14E-32					transmembrane protein, putative
Sapur.011G072600.1	376.6767656	-1.10753603	0.063276084	-17.5032	1.35E-68	9.95E-68	cyan	-0.779055687	3.05E-33		AT4G25210.1		DNA-binding storekeeper protein-related transcriptional regulator	NA
Sapur.T104900.1	25.1237837	1.526529026	0.087252376	17.49556	1.55E-68	1.14E-67	cyan	0.718441394	3.25E-26	GO:0055114,GO:0009186	AT3G23580.1	RNR2,RNR2A	ribonucleotide reductase 2A	NA
Sapur.15WG115900.1	1590.293757	1.242460519	0.071017419	17.49515	1.56E-68	1.15E-67	cyan	0.767014796	1.12E-31	GO:0043565,GO:0006355,GO:0003700	AT5G13080.1	ATWRKY75,WRKY75	WRKY DNA-binding protein 75	WRKY transcription factor, putative
Sapur.014G026700.1	575.0984202	-0.83273667	0.047601338	-17.494	1.59E-68	1.17E-67	cyan	-0.80381732	8.62E-37	GO:0009512	AT2G26500.2		cytochrome b6f complex subunit (petM), putative	PetM family of cytochrome b6f complex subunit 7
Sapur.019G083100.1	44.75235231	1.976102758	0.112964899	17.49307	1.62E-68	1.19E-67	cyan	0.70462499	7.25E-25	GO:0055085,GO:0016020,GO:0015297,GO:0015238,GO:0006855	AT1G71870.1		MATE efflux family protein	MATE efflux family protein
Sapur.013G116900.1	134.5282978	-1.956484133	0.111852497	-17.4916	1.66E-68	1.22E-67	cyan	-0.814251206	1.93E-38		AT1G21500.1			hypothetical protein
Sapur.004G154000.1	1586.625537	0.56251406	0.032170503	17.4854	1.85E-68	1.36E-67	cyan	0.801563753	1.90E-36	GO:0005515	AT2G17200.1	DSK2	ubiquitin family protein	ubiquitin domain protein
Sapur.001G104300.1	395.1299691	0.652602294	0.037325079	17.48428	1.89E-68	1.38E-67	cyan	0.801345511	2.05E-36		AT5G61500.1	ATATG3,ATG3	autophagy 3 (APG3)	autophagy-related protein
Sapur.006G083500.1	1326.660307	-0.591792495	0.033881663	-17.4665	2.58E-68	1.89E-67	cyan	-0.793906887	2.59E-35	GO:0016021,GO:0004659	AT3G51820.1	ATG4,CHLG,G4	UbiA prenyltransferase family protein	bacteriochlorophyll synthase, putative
Sapur.016G143300.1	55.06289532	-1.286808308	0.073797895	-17.4369	4.33E-68	3.16E-67	cyan	-0.801285022	2.10E-36	GO:0055114,GO:0050897,GO:0016636,GO:0010024	AT3G09150.2	ATHY2,GUN3,HY2	phytochromobilin:ferredoxin oxidoreductase, chloroplast / phytochromobilin synthase (HY2)	heme oxygenase 2 protein, putative
Sapur.009G047200.1	88.40144156	-0.687720919	0.039448582	-17.4333	4.61E-68	3.36E-67	cyan	-0.838406922	1.07E-42	GO:0046872	AT2G41900.1		CCCH-type zinc finger protein with ARM repeat domain	zinc finger CCCH domain protein
Sapur.012G080000.1	307.9437633	-0.607928781	0.034872578	-17.4329	4.65E-68	3.39E-67	cyan	-0.835943879	3.12E-42	GO:0016779,GO:0006396,GO:0003723	AT5G23690.1		Polynucleotide adenylyltransferase family protein	polynucleotide adenylyltransferase family protein
Sapur.008G053800.1	92.73672679	-0.812252835	0.046604314	-17.4287	5.00E-68	3.64E-67	cyan	-0.816149005	9.40E-39		AT3G55490.1		GINS complex protein	DNA replication complex GINS protein Psf3
Sapur.016G204200.1	13.27326702	-2.141136623	0.122929554	-17.4176	6.07E-68	4.42E-67	cyan	-0.810012936	9.28E-38		AT1G60550.1	DHNS,ECHID	enoyl-CoA hydratase/isomerase D	1%2C4-dihydroxy-2-naphthoyl-CoA synthase
Sapur.010G095700.1	371.5572127	-1.137852745	0.065331263	-17.4167	6.17E-68	4.49E-67	cyan	-0.796063978	1.26E-35	GO:0008270,GO:0005622,GO:0005515	AT1G25440.1		B-box type zinc finger protein with CCT domain	zinc finger constans-like protein
Sapur.016G274900.1	153.5553232	1.112939053	0.063903455	17.41594	6.24E-68	4.55E-67	cyan	0.749714352	1.37E-29		AT5G54650.1	ATFH5,Fh5	formin homology5	formin-like 2 domain protein
Sapur.006G163300.1	11.72274425	3.604109307	0.206981536	17.41271	6.61E-68	4.81E-67	cyan	0.654774837	1.39E-20		AT3G52360.1			NA
Sapur.013G120100.1	114.0372896	-1.021463301	0.058669517	-17.4105	6.87E-68	5.00E-67	cyan	-0.802853233	1.21E-36	GO:0043531	AT3G14460.1		LRR and NB-ARC domains-containing disease resistance protein	CC-NBS-LRR resistance protein
Sapur.019G018600.1	26.58329205	1.47907705	0.084954431	17.41024	6.90E-68	5.02E-67	cyan	0.789504765	1.11E-34		AT3G14840.2		Leucine-rich repeat transmembrane protein kinase	NA
Sapur.001G041300.1	20.34573789	-1.856171965	0.106646098	-17.405	7.56E-68	5.50E-67	cyan	-0.775993051	7.79E-33		AT1G34010.1			NA
Sapur.016G151900.1	369.5404957	0.568196731	0.032649683	17.40283	7.85E-68	5.71E-67	cyan	0.817938777	4.75E-39		AT5G09680.1	RLF	reduced lateral root formation	reduced lateral root formation protein, putative
Sapur.016G181800.1	4.196338841	5.240688842	0.301144373	17.40258	7.89E-68	5.73E-67	cyan	0.675858825	2.71E-22	GO:0016758,GO:0008152	AT4G34131.1	UGT73B3	UDP-glucosyl transferase 73B3	UDP-glucose:glucosyltransferase
Sapur.008G045900.1	87.75591313	1.717141274	0.098698772	17.3978	8.57E-68	6.22E-67	cyan	0.760820059	6.56E-31	GO:0006468,GO:0005524,GO:0004672,GO:0030246	AT3G55550.1		Concanavalin A-like lectin protein kinase family protein	lectin-domain receptor kinase
Sapur.007G024400.1	41.41622214	2.104318777	0.120980873	17.39381	9.19E-68	6.67E-67	cyan	0.673916377	3.95E-22	GO:0005515	AT5G07610.1		F-box family protein	F-box protein interaction domain protein
Sapur.006G223100.1	933.1244989	-0.51045286	0.029356005	-17.3884	1.01E-67	7.33E-67	cyan	-0.819666192	2.43E-39	GO:0008270,GO:0003676	AT4G31580.2	RSZP22,SRZ-22,SRZ22	serine/arginine-rich 22	RNA recognition motif 1 in vertebrate serine/arginine-rich splicing factor
Sapur.014G063900.1	35.21576965	-0.939579562	0.054057492	-17.3811	1.15E-67	8.32E-67	cyan	-0.835333758	4.05E-42		AT4G00525.1			hypothetical protein
Sapur.15WG082900.1	15.26296776	-1.826822598	0.105122072	-17.3781	1.21E-67	8.76E-67	cyan	-0.739929827	1.76E-28					NA
Sapur.001G185800.1	561.2796205	-0.538722229	0.03100769	-17.3738	1.30E-67	9.44E-67	cyan	-0.827363699	1.14E-40		AT2G23390.1			acetyltransferase (GNAT) domain protein
Sapur.002G082100.1	16.81094946	2.555158062	0.147150433	17.36426	1.54E-67	1.11E-66	cyan	0.697697951	3.22E-24					NA
Sapur.016G164500.1	487.145691	-0.588674278	0.033916927	-17.3564	1.77E-67	1.28E-66	cyan	-0.804249726	7.40E-37		AT5G58560.1		Phosphatidate cytidylyltransferase family protein	phytol kinase, putative
Sapur.013G104500.1	57.31107607	-3.86365671	0.222645339	-17.3534	1.86E-67	1.34E-66	cyan	-0.676694523	2.30E-22	GO:0006950	AT1G68300.1		Adenine nucleotide alpha hydrolases-like superfamily protein	universal stress family protein
Sapur.010G064700.1	1311.283553	-0.745818885	0.042986449	-17.3501	1.97E-67	1.42E-66	cyan	-0.829915802	3.99E-41		AT3G22790.1		Kinase interacting (KIP1-like) family protein	kinase interacting-like protein
Sapur.004G039100.1	16.0628725	2.138285276	0.123295108	17.34282	2.23E-67	1.61E-66	cyan	0.721622783	1.55E-26	GO:0006355,GO:0003677	AT1G29160.1		Dof-type zinc finger DNA-binding family protein	Dof-type zinc finger protein
Sapur.002G015700.1	1399.564827	1.401510531	0.080832391	17.33848	2.41E-67	1.74E-66	cyan	0.738793449	2.35E-28	GO:0006457,GO:0003755,GO:0000413	AT2G16600.1	ROC3	rotamase CYP 3	peptidyl-prolyl cis-trans isomerase, cyclophilin-type protein
Sapur.018G093900.1	15.22226903	7.387042555	0.426269938	17.32949	2.82E-67	2.03E-66	cyan	0.606387993	3.89E-17	GO:0005509	AT4G32060.1		calcium-binding EF hand family protein	calcium ion-binding protein, putative
Sapur.002G167200.1	8.223379642	5.344903424	0.30851549	17.32459	3.07E-67	2.21E-66	cyan	0.669636169	8.95E-22		AT1G02460.1		Pectin lyase-like superfamily protein	polygalacturonase
Sapur.009G063600.1	900.7860513	0.81483443	0.047036728	17.32337	3.13E-67	2.26E-66	cyan	0.789947086	9.60E-35	GO:0016020,GO:0006810,GO:0005215	AT5G64500.1		Major facilitator superfamily protein	sphingolipid transporter spinster-like protein, putative
Sapur.011G075700.1	147.9358259	-1.512686067	0.087368606	-17.3138	3.70E-67	2.66E-66	cyan	-0.787432193	2.17E-34	GO:0055114,GO:0004097,GO:0016491,GO:0008152				polyphenol oxidase
Sapur.15WG145600.1	562.185146	0.650977951	0.037614872	17.3064	4.21E-67	3.03E-66	cyan	0.808867527	1.41E-37		AT5G52120.1	AtPP2-A14,PP2-A14	phloem protein 2-A14	F-box protein PP2-A13
Sapur.007G019700.1	21.67947668	-1.354334234	0.078256323	-17.3064	4.21E-67	3.03E-66	cyan	-0.835325128	4.07E-42		AT5G66500.1		Tetratricopeptide repeat (TPR)-like superfamily protein	NA
Sapur.15WG113600.1	161.0457577	1.4242541	0.082319233	17.3016	4.58E-67	3.29E-66	cyan	0.721007847	1.79E-26		AT3G48550.1			nucleic acid-binding protein, putative
Sapur.016G276400.1	1922.584197	0.563923196	0.032613942	17.29086	5.51E-67	3.96E-66	cyan	0.800933809	2.37E-36	GO:0006413,GO:0003743	AT1G54290.1		Translation initiation factor SUI1 family protein	NA
Sapur.001G084200.1	12.06445	-2.11735901	0.122465358	-17.2895	5.65E-67	4.06E-66	cyan	-0.772720154	2.09E-32	GO:0009058,GO:0005524,GO:0016874	AT1G63680.1	ATMURE,MURE,PDE316	acid-amino acid ligases;ligases;ATP binding;ATP binding;ligases	UDP-N-acetylmuramoyl-L-alanyl-D-glutamate-2, 6-diaminopimelate ligase
Sapur.014G100100.1	264.2774105	0.88217066	0.051032043	17.2866	5.93E-67	4.26E-66	cyan	0.767978026	8.45E-32	GO:0016757	AT3G62660.1	GATL7	galacturonosyltransferase-like 7	glycosyltransferase family 8 protein
Sapur.003G080600.2	3.258457208	-3.970993889	0.22972135	-17.2861	5.98E-67	4.29E-66	cyan	-0.833649623	8.32E-42		AT1G62870.1			hypothetical protein
Sapur.016G076900.1	304.0383147	0.628415741	0.036355582	17.28526	6.07E-67	4.36E-66	cyan	0.800927942	2.37E-36		AT3G53490.1			transmembrane protein, putative
Sapur.009G042400.1	50.30188086	1.361070281	0.0787511	17.28319	6.30E-67	4.52E-66	cyan	0.81264796	3.51E-38	GO:0006355,GO:0005634,GO:0003700,GO:0046983,GO:0003677	AT5G13790.1	AGL15	AGAMOUS-like 15	agamous-like MADS-box protein AGL15
Sapur.016G284700.1	520.492569	-1.418504695	0.082083061	-17.2813	6.50E-67	4.66E-66	cyan	-0.741483698	1.18E-28	GO:0042651,GO:0015979,GO:0009654,GO:0009523	AT1G79040.1	PSBR	photosystem II subunit R	photosystem II 10 kDa polypeptide PsbR
Sapur.T005700.1	36.8796514	2.236556207	0.12942169	17.28115	6.52E-67	4.68E-66	cyan	0.650098963	3.18E-20	GO:0016020,GO:0015171,GO:0003333	AT1G58030.1	CAT2	cationic amino acid transporter 2	NA
Sapur.003G037700.1	363.5656483	1.034422423	0.059862004	17.28012	6.64E-67	4.76E-66	cyan	0.789489713	1.11E-34		AT3G14060.1			hypothetical protein
Sapur.005G062900.1	148.8057173	-0.669724847	0.038763501	-17.2772	6.99E-67	5.00E-66	cyan	-0.813525575	2.53E-38		AT5G65180.1		ENTH/VHS family protein	RNA polymerase II-binding domain protein
Sapur.008G025400.1	767.356383	0.527163478	0.030514063	17.27608	7.12E-67	5.10E-66	cyan	0.803377787	1.01E-36	GO:0005525	AT3G54840.1	ARA-6.ARA6.ATRAB5C.ATRA	Ras-related small GTP-binding family protein	Ras small GTPase family Ras protein
Sapur.011G088400.1	884.2389922	1.343867094	0.077791199	17.27531	7.22E-67	5.17E-66	cyan	0.728438359	3.05E-27	GO:0006355,GO:0003677	AT4G27410.2	ANAC072,RD26	NAC (No Apical Meristem) domain transcriptional regulator superfamily protein	NAC transcription factor
Sapur.T139600.1	21.98571833	-1.504776196	0.087116493	-17.2731	7.49E-67	5.36E-66	cyan	-0.786281447	3.15E-34		AT2G17510.2	EMB2763	ribonuclease II family protein	NA
Sapur.012G012500.1	106.076302	-0.990357187	0.057345009	-17.2702	7.89E-67	5.65E-66	cyan	-0.829587901	4.57E-41		AT5G24280.1	GMI1	gamma-irradiation and mitomycin c induced 1	NA
Sapur.019G007100.1	6.448253001	4.852738497	0.281060862	17.26579	8.51E-67	6.09E-66	cyan	0.684093745	5.33E-23	GO:0007165,GO:0005515,GO:0043531	AT5G36930.2		Disease resistance protein (TIR-NBS-LRR class) family	NA
Sapur.003G003900.1	18.40901775	-1.705662076	0.098803532	-17.2632	8.91E-67	6.37E-66	cyan	-0.77560105	8.77E-33	GO:0009982,GO:0009451,GO:0003723,GO:0001522	AT1G09800.1		Pseudouridine synthase family protein	tRNA pseudouridine synthase
Sapur.008G005300.1	6.781398108	3.487290854	0.202036491	17.2607	9.30E-67	6.64E-66	cyan	0.730927425	1.66E-27	GO:0016757,GO:0047262	AT3G25140.1	GAUT8,QUA1	Nucleotide-diphospho-sugar transferases superfamily protein	glycosyltransferase family 8 protein
Sapur.014G084600.2	6.18994008	3.235993299	0.187528699	17.25599	1.01E-66	7.20E-66	cyan	0.662200452	3.59E-21	GO:0006796,GO:0005737,GO:0004427,GO:0000287	AT1G01050.1	AtPPa1,PPa1	pyrophosphorylase 1	soluble inorganic pyrophosphatase
Sapur.019G113400.1	2285.117028	0.805658339	0.046689605	17.25563	1.02E-66	7.25E-66	cyan	0.779060661	3.05E-33	GO:0008121	AT3G10860.1		Cytochrome b-c1 complex, subunit 8 protein	NA
Sapur.013G008500.1	355.0112451	0.565777488	0.032793087	17.25295	1.06E-66	7.59E-66	cyan	0.793839574	2.65E-35		AT1G56420.1			hypothetical protein
Sapur.006G206300.1	713.9564716	-0.649931355	0.037697963	-17.2405	1.32E-66	9.41E-66	cyan	-0.811013853	6.43E-38	GO:0003677	AT1G52740.1	HTA9	histone H2A protein 9	histone H2A
Sapur.004G001000.1	94.19714293	-0.669609369	0.038858394	-17.232	1.53E-66	1.09E-65	cyan	-0.810878609	6.75E-38		AT4G22290.1		Ubiquitin-specific protease family C19-related protein	ubiquitin thiolesterase, putative
Sapur.012G096400.1	8.982469623	-3.575078291	0.207752263	-17.2084	2.30E-66	1.63E-65	cyan	-0.656784981	9.65E-21	GO:0006355,GO:0005634,GO:0003700,GO:0046983,GO:0003677	AT5G23260.2	ABS,AGL32,TT16	K-box region and MADS-box transcription factor family protein	MADS-box DNA-binding domain transcription factor
Sapur.001G169500.1	12.65201094	3.811713161	0.221731035	17.19071	3.12E-66	2.21E-65	cyan	0.631541401	7.47E-19	GO:0055085,GO:0016021	AT5G57090.1	AGR.AGR1.ATPIN2.EIR1.PIN	Auxin efflux carrier family protein	auxin efflux carrier PIN1 protein
Sapur.014G093200.1	30.75449105	2.271470381	0.132159438	17.18735	3.30E-66	2.34E-65	cyan	0.755442448	2.92E-30	GO:0016757	AT2G47180.1	AtGolS1,GolS1	galactinol synthase 1	galactinol synthase
Sapur.011G123300.1	441.0043495	-0.701062816	0.040801336	-17.1823	3.60E-66	2.55E-65	cyan	-0.822825356	7.05E-40	GO:0055114,GO:0016627,GO:0016021,GO:0006784	AT5G56090.1	COX15	cytochrome c oxidase 15	cytochrome C oxidase assembly protein COX15, putative
Sapur.003G079900.1	19.1099212	1.933345547	0.112624703	17.16627	4.75E-66	3.36E-65	cyan	0.761763126	5.03E-31		AT3G60780.1		Protein of unknown function (DUF1442)	NAD(P)H-quinone oxidoreductase subunit 6, putative
Sapur.010G195400.1	64.2820195	-1.205940849	0.070298025	-17.1547	5.80E-66	4.10E-65	cyan	-0.827635869	1.02E-40		AT5G04820.1	ATOFP13,OFP13	ovate family protein 13	ovate transcriptional repressor
Sapur.007G116700.1	381.3101442	1.058806304	0.061732169	17.15161	6.11E-66	4.32E-65	cyan	0.748329583	1.98E-29	GO:0050662,GO:0003824	AT2G43420.1		3-beta hydroxysteroid dehydrogenase/isomerase family protein	NA
Sapur.003G052000.1	340.1173666	-0.459859038	0.026822368	-17.1446	6.90E-66	4.87E-65	cyan	-0.80591413	4.09E-37		AT4G17050.1	UGLYAH	ureidoglycine aminohydrolase	transcription factor
Sapur.004G138000.1	403.7123736	-1.327826984	0.077468867	-17.1401	7.45E-66	5.26E-65	cyan	-0.799586502	3.77E-36		AT2G17120.1	LYM2	lysm domain GPI-anchored protein 2 precursor	LysM domain GPI-anchored protein
Sapur.010G032100.1	1744.925083	0.479882435	0.028004462	17.13593	8.01E-66	5.65E-65	cyan	0.790001194	9.43E-35		AT1G70160.1			hypothetical protein
Sapur.016G144300.1	48.31619726	1.345593083	0.078526505	17.13553	8.06E-66	5.69E-65	cyan	0.670401813	7.74E-22		AT1G07400.1		HSP20-like chaperones superfamily protein	17.5 kDa class I heat shock protein
Sapur.003G164700.1	916.2518713	0.794835955	0.046401552	17.12951	8.94E-66	6.31E-65	cyan	0.785981097	3.46E-34		AT5G19860.1		Protein of unknown function, DUF538	T4.10 protein, putative
Sapur.005G147100.1	75.28617425	4.108981774	0.239934719	17.12542	9.59E-66	6.76E-65	cyan	0.70808933	3.39E-25	GO:0055114,GO:0045300,GO:0006631	AT1G43800.1		Plant stearoyl-acyl-carrier-protein desaturase family protein	stearoyl acyl carrier desaturase
Sapur.002G138400.1	8435.46043	0.883287201	0.051577929	17.12529	9.61E-66	6.77E-65	cyan	0.796361274	1.14E-35	GO:0016624,GO:0008152	AT1G01090.1	PDH-E1 ALPHA	pyruvate dehydrogenase E1 alpha	pyruvate dehydrogenase E1 component, alpha subunit
Sapur.017G094400.1	77.57951729	-1.774210295	0.103608489	-17.1242	9.80E-66	6.90E-65	cyan	-0.7915127	5.74E-35		AT3G02550.1	LBD41	LOB domain-containing protein 41	LOB domain protein
Sapur.004G000600.1	85.54448498	-0.675988556	0.039494681	-17.1159	1.13E-65	7.95E-65	cyan	-0.58055365	1.59E-15		AT4G22320.2			NA
Sapur.018G091100.1	26.90637738	8.20860297	0.479633414	17.11433	1.16E-65	8.17E-65	cyan	0.582730242	1.18E-15		AT4G32060.1		calcium-binding EF hand family protein	NA
Sapur.005G039900.1	57.07209185	-1.489435792	0.087035174	-17.113	1.19E-65	8.35E-65	cyan	-0.768067647	8.23E-32					NA
Sapur.006G099300.1	633.3655182	-0.999301686	0.058411708	-17.1079	1.30E-65	9.11E-65	cyan	-0.83107969	2.46E-41	GO:0016787,GO:0006807,GO:0004356	AT3G53180.1		glutamate-ammonia ligases;catalytics;glutamate-ammonia ligases	glutamine synthetase, putative
Sapur.001G029700.1	104.7224128	-0.770152084	0.045020459	-17.1067	1.32E-65	9.29E-65	cyan	-0.806525831	3.29E-37	GO:0046872,GO:0016567,GO:0004842	AT3G14250.1		RING/U-box superfamily protein	RING zinc finger protein, putative
Sapur.001G074400.1	494.456757	-0.564866027	0.033027786	-17.1028	1.42E-65	9.94E-65	cyan	-0.803512313	9.60E-37		AT4G10970.1			hypothetical protein
Sapur.010G158600.1	502.1636865	-0.539819886	0.031575877	-17.096	1.59E-65	1.12E-64	cyan	-0.826460123	1.64E-40	GO:0031011,GO:0006355,GO:0006281	AT3G12380.1	ARP5,ATARP5	actin-related protein 5	actin-related protein
Sapur.014G017000.1	20.03070126	-1.306454039	0.076448028	-17.0894	1.78E-65	1.25E-64	cyan	-0.809681578	1.05E-37		AT1G45231.2		S-adenosyl-L-methionine-dependent methyltransferases superfamily protein	trimethylguanosine synthase, putative
Sapur.002G020100.1	685.5972476	0.75672376	0.044294217	17.08403	1.95E-65	1.37E-64	cyan	0.790031349	9.34E-35	GO:0033177,GO:0015991,GO:0015078	AT1G19910.1	ATVHA-C2.AVA-2PE.AVA-P2	ATPase, F0/V0 complex, subunit C protein	V-type proton ATPase 16 kDa proteolipid subunit c2
Sapur.012G048500.1	182.1255409	-0.69339179	0.040589928	-17.0829	1.99E-65	1.40E-64	cyan	-0.821012773	1.44E-39	GO:0006468,GO:0004672	AT1G18670.1	IBS1	Protein kinase superfamily protein	Serine/Threonine kinase family protein
Sapur.004G129200.1	136.5988064	2.72828597	0.159732538	17.08034	2.08E-65	1.46E-64	cyan	0.579696061	1.79E-15	GO:0016758,GO:0008152	AT1G22400.1	ATUGT85A1,UGT85A1	UDP-Glycosyltransferase superfamily protein	UDP-glucosyltransferase family protein
Sapur.T004000.1	384.7583943	-0.759882566	0.044505541	-17.0739	2.32E-65	1.63E-64	cyan	-0.794203948	2.35E-35		AT5G55510.1		Mitochondrial import inner membrane translocase subunit Tim17/Tim22/Tim23 family protein	import inner membrane translocase subunit TIM22
Sapur.013G061600.1	4.593733326	4.796470441	0.2811621	17.05945	2.97E-65	2.08E-64	cyan	0.672255979	5.43E-22					NA
Sapur.002G041100.1	9.444807174	3.468358121	0.203311141	17.05936	2.98E-65	2.08E-64	cyan	0.69847752	2.73E-24	GO:0009055	AT5G26330.1		Cupredoxin superfamily protein	plastocyanin-like domain protein
Sapur.003G088000.1	24.3969867	-1.630457848	0.09560509	-17.0541	3.26E-65	2.28E-64	cyan	-0.825237946	2.69E-40	GO:0005515	AT4G23060.1	IQD22	IQ-domain 22	NA
Sapur.003G011600.1	1084.041038	-1.414161668	0.082922507	-17.054	3.26E-65	2.28E-64	cyan	-0.80827071	1.75E-37	GO:0016020,GO:0009765	AT1G15820.1	CP24,LHCB6	light harvesting complex photosystem II subunit 6	light harvesting chlorophyll A-B-binding protein
Sapur.016G173900.1	47.46451941	-1.573656569	0.092366562	-17.0371	4.36E-65	3.05E-64	cyan	-0.801929697	1.67E-36	GO:0006508,GO:0004185	AT1G33540.1	scpl18	serine carboxypeptidase-like 18	serine carboxypeptidase, putative
Sapur.009G044800.1	595.2647433	1.351004229	0.079320546	17.03221	4.74E-65	3.31E-64	cyan	0.773389091	1.71E-32		AT3G57450.1			hypothetical protein
Sapur.15WG145900.1	2.489613563	4.51248447	0.264981452	17.02944	4.97E-65	3.47E-64	cyan	0.763141192	3.40E-31	GO:0008168	AT4G00750.1		S-adenosyl-L-methionine-dependent methyltransferases superfamily protein	S-adenosyl-L-methionine-dependent methyltransferase, putative
Sapur.018G074300.1	65.48344415	-1.324509243	0.077818401	-17.0205	5.79E-65	4.04E-64	cyan	-0.780678171	1.85E-33	GO:0008270,GO:0005515	AT2G18670.1		RING/U-box superfamily protein	RING-H2 zinc finger protein
Sapur.T121300.1	5.813847995	3.832067277	0.225152127	17.0199	5.85E-65	4.08E-64	cyan	0.737941956	2.91E-28					NA
Sapur.013G143400.1	288.4861809	2.610981487	0.15342299	17.01819	6.02E-65	4.20E-64	cyan	0.716958727	4.57E-26		AT1G09575.1		Protein of unknown function (DUF607)	coiled-coil protein 109A, putative
Sapur.15ZG045500.1	8.053143207	-2.605079485	0.15316842	-17.0079	7.17E-65	5.00E-64	cyan	-0.794862431	1.88E-35					NA
Sapur.006G033800.1	51.12505095	1.25668585	0.073891105	17.00727	7.25E-65	5.05E-64	cyan	0.768779191	6.69E-32		AT5G03990.1			NA
Sapur.018G024000.1	749.5309594	0.535813699	0.031509905	17.00461	7.59E-65	5.29E-64	cyan	0.796049007	1.26E-35		AT2G26660.1	ATSPX2,SPX2	SPX domain gene 2	xenotropic and polytropic murine leukemia virus receptor ids-4, putative
Sapur.002G129100.1	145.569439	1.388865401	0.08171601	16.99625	8.76E-65	6.09E-64	cyan	0.77073155	3.77E-32	GO:0005515	AT4G11610.1		C2 calcium/lipid-binding plant phosphoribosyltransferase family protein	glutathione peroxidase
Sapur.005G159000.1	368.6710235	-0.51272573	0.030171032	-16.994	9.10E-65	6.33E-64	cyan	-0.82272561	7.34E-40	GO:0009116	AT2G42910.1		Phosphoribosyltransferase family protein	ribose-phosphate pyrophosphokinase
Sapur.016G026800.1	1531.145943	-0.708092889	0.041668553	-16.9935	9.18E-65	6.38E-64	cyan	-0.849035459	8.46E-45	GO:0003677	AT1G65440.2	GTB1	global transcription factor group B1	global transcription factor group protein, putative
Sapur.019G051600.2	30.05615037	1.452719775	0.085489411	16.99298	9.26E-65	6.43E-64	cyan	0.68984305	1.66E-23	GO:0046872	AT3G02830.1	ZFN1	zinc finger protein 1	zinc finger CCCH domain protein
Sapur.005G133400.1	759.1827055	0.587370152	0.034573808	16.98888	9.93E-65	6.89E-64	cyan	0.801898185	1.69E-36	GO:0016740	AT1G36370.1	SHM7	serine hydroxymethyltransferase 7	serine hydroxymethyltransferase
Sapur.018G087100.1	146.5944428	-0.674665985	0.039714176	-16.988	1.01E-64	6.99E-64	cyan	-0.820400805	1.83E-39		AT1G15860.2		Domain of unknown function (DUF298)	defective in cullin neddylation protein, putative
Sapur.012G063500.1	88.03927648	-1.036468156	0.061016631	-16.9866	1.03E-64	7.16E-64	cyan	-0.631321614	7.74E-19		AT1G23280.1		MAK16 protein-related	Mak16 protein carboxy-terminal region protein
Sapur.016G243600.1	369.5991821	1.810325286	0.106624669	16.97848	1.19E-64	8.22E-64	cyan	0.697128431	3.63E-24		AT3G62730.1			desiccation-related protein PCC13-62
Sapur.017G096000.1	195.0998866	-0.800054733	0.047133574	-16.9742	1.27E-64	8.84E-64	cyan	-0.802884233	1.20E-36		AT1G67040.1			DUF4378 domain protein
Sapur.016G065500.1	3728.396142	0.860548238	0.050731244	16.96288	1.55E-64	1.07E-63	cyan	0.774865728	1.10E-32		AT3G11660.1	NHL1	NDR1/HIN1-like 1	harpin-induced-like protein
Sapur.005G101500.1	1107.407621	0.527776035	0.03113566	16.95085	1.90E-64	1.31E-63	cyan	0.800275682	2.97E-36		AT4G36800.1	RCE1	RUB1 conjugating enzyme 1	ubiquitin-conjugating enzyme
Sapur.014G075600.1	324.9299198	-0.78307182	0.046235877	-16.9365	2.42E-64	1.67E-63	cyan	-0.826084125	1.91E-40		AT1G01360.1	PYL9,RCAR1	regulatory component of ABA receptor 1	abscisic acid receptor PYL9
Sapur.T121500.1	7.869682712	4.002962658	0.236491863	16.92643	2.87E-64	1.98E-63	cyan	0.663136848	3.02E-21					NA
Sapur.018G076900.1	522.7963582	0.468711177	0.027691965	16.92589	2.90E-64	2.00E-63	cyan	0.798345832	5.78E-36		AT2G23940.1		Protein of unknown function (DUF788)	transmembrane protein
Sapur.016G287700.1	185.922331	-0.964788911	0.057023455	-16.9192	3.25E-64	2.24E-63	cyan	-0.801340034	2.06E-36		AT1G69550.1		disease resistance protein (TIR-NBS-LRR class)	NA
Sapur.004G006600.1	190.4370845	-0.644830785	0.038112463	-16.9192	3.25E-64	2.24E-63	cyan	-0.83418014	6.64E-42		AT1G11800.1		endonuclease/exonuclease/phosphatase family protein	endonuclease/exonuclease/phosphatase family protein
Sapur.T121400.1	9.767961858	2.84662528	0.168250122	16.91901	3.26E-64	2.25E-63	cyan	0.692459086	9.67E-24					NA
Sapur.006G070500.1	8374.877055	0.977655773	0.057799465	16.91462	3.51E-64	2.42E-63	cyan	0.799824936	3.48E-36		AT5G02260.1	ATEXP9.ATEXPA9.ATHEXP A	expansin A9	alpha-expansin
Sapur.15WG120100.1	9.780943938	-2.77711073	0.164185783	-16.9144	3.52E-64	2.43E-63	cyan	-0.776712926	6.26E-33		AT2G47140.1		NAD(P)-binding Rossmann-fold superfamily protein	NA
Sapur.15ZG052500.2	65.93527026	1.583222694	0.093634789	16.90849	3.90E-64	2.68E-63	cyan	0.797104073	8.83E-36	GO:0016798,GO:0016020	AT5G07830.1	AtGUS2,GUS2	glucuronidase 2	glycoside hydrolase family 79 amino-terminal domain protein
Sapur.008G140000.1	559.8851354	-0.904130988	0.053529641	-16.8903	5.30E-64	3.65E-63	cyan	-0.836208524	2.78E-42	GO:0003676	AT1G60000.1		RNA-binding (RRM/RBD/RNP motifs) family protein	ribonucleoprotein, putative
Sapur.012G030000.1	3616.917736	0.85018925	0.050378772	16.87594	6.76E-64	4.64E-63	cyan	0.819919666	2.21E-39		AT3G17860.1	JAI3,JAZ3,TIFY6B	jasmonate-zim-domain protein 3	jasmonate ZIM-domain protein
Sapur.006G009900.1	2625.503294	1.337345492	0.079251455	16.87471	6.91E-64	4.74E-63	cyan	0.722825577	1.17E-26	GO:0055085,GO:0016021	AT5G47560.1	ATSDAT,ATTDT,TDT	tonoplast dicarboxylate transporter	NA
Sapur.006G136700.1	115.450425	-1.409959751	0.083559455	-16.8737	7.02E-64	4.81E-63	cyan	-0.798734577	5.06E-36		AT2G18900.1		Transducin/WD40 repeat-like superfamily protein	transducin family protein
Sapur.001G008100.1	443.355732	-0.770471538	0.045664943	-16.8723	7.20E-64	4.93E-63	cyan	-0.783376982	7.92E-34		AT2G34860.2	EDA3	DnaJ/Hsp40 cysteine-rich domain superfamily protein	DnaJ-like zinc-finger protein, putative
Sapur.002G170200.1	677.3905836	0.518792492	0.030762163	16.86463	8.19E-64	5.61E-63	cyan	0.80368912	9.02E-37	GO:0016021,GO:0004659	AT2G44520.1	COX10	cytochrome c oxidase 10	protoheme IX farnesyltransferase
Sapur.15WG041200.1	8.108366507	-2.034702975	0.120657649	-16.8634	8.36E-64	5.72E-63	cyan	-0.796279435	1.17E-35	GO:0016853,GO:0005975	AT3G04880.1	DRT102	DNA-damage-repair/toleration protein (DRT102)	NA
Sapur.008G056600.1	203.7962424	1.704885413	0.101109956	16.8617	8.61E-64	5.89E-63	cyan	0.728965672	2.68E-27		AT3G11090.1	LBD21	LOB domain-containing protein 21	LOB domain protein
Sapur.004G103200.1	178.6643559	-1.232502261	0.073129288	-16.8537	9.85E-64	6.73E-63	cyan	-0.778384988	3.75E-33		AT2G34450.1		HMG-box (high mobility group) DNA-binding family protein	NA
Sapur.002G071900.1	22.44066155	-1.433045068	0.085056998	-16.8481	1.08E-63	7.40E-63	cyan	-0.785630256	3.87E-34		AT1G36940.2			hypothetical protein
Sapur.016G253900.1	878.6141964	-1.561572591	0.092760011	-16.8345	1.36E-63	9.29E-63	cyan	-0.795270696	1.64E-35	GO:0016491	AT3G14420.2		Aldolase-type TIM barrel family protein	peroxisomal (S)-2-hydroxy-acid oxidase GLO1

Sapur.007G116300.1	681.1251628	0.683114756	0.040597693	16.82644	1.56E-63	1.06E-62	cyan	0.764514851	2.30E-31	GO:0043565,GO:0006355,GO:0003700	AT1G06070.1		Basic-leucine zipper (bZIP) transcription factor family protein	transcription factor RF2a
Sapur.019G111600.1	540.3181631	0.772502544	0.045910864	16.82614	1.57E-63	1.07E-62	cyan	0.785836496	3.63E-34		AT3G51620.2		PAP/OAS1 substrate-binding domain superfamily	nucleic acid-binding protein, putative
Sapur.001G068800.1	1998.818066	1.175217422	0.069845499	16.82596	1.57E-63	1.07E-62	cyan	0.769501688	5.41E-32	GO:0009055	AT1G64640.1	AtENODL8,ENODL8	early nodulin-like protein 8	plastocyanin-like domain protein
Sapur.002G184700.1	1151.58705	0.730128696	0.043403315	16.82196	1.68E-63	1.15E-62	cyan	0.773991351	1.43E-32		AT3G07480.1		2Fe-2S ferredoxin-like superfamily protein	2Fe-2S ferredoxin
Sapur.009G079100.1	17.9433942	-1.482386593	0.088139802	-16.8186	1.78E-63	1.21E-62	cyan	-0.808125354	1.85E-37		AT4G33985.1		Protein of unknown function (DUF1685)	DUF1685 family protein
Sapur.15WG095000.1	9.559123886	-2.827143944	0.16810189	-16.818	1.80E-63	1.23E-62	cyan	-0.738476031	2.54E-28		AT5G14750.1	ATMYB66,MYB66,WER,WEF	myb domain protein 66	Myb transcription factor
Sapur.002G190500.1	147.5630119	-0.697361146	0.041513468	-16.7984	2.51E-63	1.70E-62	cyan	-0.82202439	9.68E-40		AT3G07210.1			hypothetical protein
Sapur.004G087900.1	312.0032771	-0.781552683	0.046534517	-16.7951	2.65E-63	1.80E-62	cyan	-0.820817452	1.55E-39	GO:0006468,GO:0005524,GO:0004672	AT1G10940.1	ASK1,SNRK2-4,SNRK2.4,SRK	Protein kinase superfamily protein	Serine/Threonine kinase domain protein
Sapur.006G070800.1	2903.283373	0.687188412	0.04093629	16.78678	3.05E-63	2.07E-62	cyan	0.79960233	3.75E-36		AT3G09300.1	ORP3B	OSBP(oxysterol binding protein)-related protein 3B	oxysterol-binding protein-related protein 3B
Sapur.016G298700.1	25.68218563	-1.381763777	0.082334434	-16.7823	3.29E-63	2.23E-62	cyan	-0.800983138	2.33E-36		AT1G73720.1	SMU1	transducin family protein / WD-40 repeat family protein	NA
Sapur.007G121400.1	16.65401505	-1.436473733	0.085602243	-16.7808	3.37E-63	2.29E-62	cyan	-0.804430919	6.94E-37		AT5G44150.1			NA
Sapur.006G015900.1	91.58858274	-1.56803944	0.093446997	-16.78	3.42E-63	2.32E-62	cyan	-0.760854394	6.50E-31		AT3G22210.1			transmembrane protein, putative
Sapur.013G131000.1	3.667759526	4.768279059	0.284243803	16.77531	3.70E-63	2.51E-62	cyan	0.703275784	9.73E-25	GO:0005515	AT3G18150.1		RNI-like superfamily protein	F-box protein
Sapur.013G061800.1	4.012319139	4.89211533	0.291654887	16.77364	3.80E-63	2.58E-62	cyan	0.659497469	5.90E-21					NA
Sapur.010G077300.1	48.89677379	-1.806877268	0.107738363	-16.771	3.98E-63	2.69E-62	cyan	-0.792597138	4.01E-35	GO:0055085,GO:0016020,GO:0006810,GO:0005215	AT5G62890.3		Xanthine/uracil permease family protein	nucleobase ascorbate transporter
Sapur.013G115900.1	866.4966975	1.158425194	0.069082814	16.76865	4.14E-63	2.80E-62	cyan	0.686732805	3.13E-23	GO:0016787	AT1G18480.1		Calcineurin-like metallo-phosphoesterase superfamily protein	ser/thr phosphatase family protein
Sapur.006G030800.1	211.1057977	2.004022629	0.119541645	16.76422	4.46E-63	3.02E-62	cyan	0.625759881	1.91E-18		AT2G41380.1		S-adenosyl-L-methionine-dependent methyltransferases superfamily protein	NA
Sapur.014G060100.1	4.499885132	4.438292695	0.264803252	16.76072	4.73E-63	3.20E-62	cyan	0.734941039	6.18E-28	GO:0005515	AT2G45760.1	BAL,BAP2	BON association protein 2	BON1-associated protein, putative
Sapur.002G179500.1	334.7592398	0.629472615	0.037570129	16.7546	5.24E-63	3.54E-62	cyan	0.793861479	2.63E-35	GO:0055114,GO:0016491	AT2G43080.1	AT-P4H-1	P4H isoform 1	prolyl 4-hydroxylase alpha subunit, putative
Sapur.008G080900.1	42.70905024	1.911041522	0.114094819	16.74959	5.70E-63	3.85E-62	cyan	0.685125812	4.33E-23		AT5G49525.1			transmembrane protein, putative
Sapur.016G186600.1	158.6098031	-0.80091586	0.04783604	-16.7429	6.38E-63	4.30E-62	cyan	-0.813978808	2.13E-38		AT1G76890.2	AT-GT2,GT2	Duplicated homeodomain-like superfamily protein	GT-like trihelix DNA-binding protein, putative
Sapur.010G096900.2	188.1148037	1.417436496	0.084688075	16.73714	7.03E-63	4.74E-62	cyan	0.766741688	1.21E-31	GO:0016020,GO:0006810,GO:0005215	AT1G68570.1		Major facilitator superfamily protein	peptide transporter
Sapur.006G154200.1	279.4246205	-1.019780092	0.060937331	-16.7349	7.30E-63	4.92E-62	cyan	-0.825007901	2.95E-40	GO:0005524,GO:0003676,GO:0016891,GO:0006396,GO:0004525,GO:0003723	AT5G20320.1	ATDCL4,DCL4	dicer-like 4	endoribonuclease dicer-like protein
Sapur.004G154500.1	193.219458	1.016654837	0.060784733	16.7255	8.55E-63	5.76E-62	cyan	0.739869131	1.79E-28		AT2G17140.1		Pentatricopeptide repeat (PPR) superfamily protein	PPR containing plant protein
Sapur.005G183500.1	1106.310813	-0.501642782	0.029994359	-16.7246	8.68E-63	5.85E-62	cyan	-0.802210365	1.52E-36	GO:0016743,GO:0006520,GO:0016597	AT1G75330.1	OTC	ornithine carbamoyltransferase	ornithine carbamoyltransferase
Sapur.014G095100.1	116.6015306	-0.610974509	0.036535796	-16.7226	8.97E-63	6.04E-62	cyan	-0.80702856	2.74E-37		AT1G02370.1		Tetratricopeptide repeat (TPR)-like superfamily protein	PPR containing plant protein
Sapur.019G064000.1	606.2034778	-0.574332593	0.03435225	-16.7189	9.54E-63	6.42E-62	cyan	-0.804100963	7.80E-37		AT3G10850.1	GLX2-2,GLY2	Metallo-hydrolase/oxidoreductase superfamily protein	hydroxyacylglutathione hydrolase
Sapur.T177300.1	6.725876706	3.013299399	0.180237638	16.71848	9.61E-63	6.47E-62	cyan	0.718938368	2.89E-26	GO:0005681,GO:0000398,GO:0000387	AT1G54385.2		ARM repeat superfamily protein	NA
Sapur.017G074800.1	33.93367941	-1.146629031	0.068589107	-16.7174	9.80E-63	6.59E-62	cyan	-0.786530685	2.90E-34	GO:0006355	AT4G38180.1	FRS5	FAR1-related sequence 5	NA
Sapur.001G074200.1	4205.356193	1.329257754	0.079575355	16.70439	1.22E-62	8.19E-62	cyan	0.757513525	1.65E-30	GO:0055114,GO:0016616,GO:0006694,GO:0003854	AT4G10960.1	UGE5	UDP-D-glucose/UDP-D-galactose 4-epimerase 5	UDP-glucose 4-epimerase
Sapur.016G197700.1	213.5027002	-0.844981008	0.050587546	-16.7033	1.24E-62	8.33E-62	cyan	-0.794044496	2.48E-35		AT1G12830.1			hypothetical protein
Sapur.012G098000.1	172.3709408	0.89288835	0.053473737	16.6977	1.36E-62	9.15E-62	cyan	0.779724399	2.48E-33		AT1G02000.1	GAE2	UDP-D-glucuronate 4-epimerase 2	UDP-D-glucuronate 4-epimerase
Sapur.016G213600.1	738.0433628	-0.83830845	0.050209048	-16.6964	1.39E-62	9.36E-62	cyan	-0.810678822	7.27E-38	GO:0005515	AT5G14370.1		CCT motif family protein	CCT motif protein
Sapur.012G023100.1	65.84789688	-0.922295826	0.055268712	-16.6875	1.62E-62	1.09E-61	cyan	-0.813541577	2.51E-38	GO:0004518				extracellular ribonuclease
Sapur.005G108700.1	260.2837896	-1.004171613	0.06017995	-16.6861	1.65E-62	1.11E-61	cyan	-0.80789481	2.01E-37		AT2G23360.1		Plant protein of unknown function (DUF869)	myosin-9 protein, putative
Sapur.011G069700.1	45.50080456	1.367319228	0.081959127	16.68294	1.74E-62	1.17E-61	cyan	0.721419141	1.62E-26		AT5G46030.1			transcription elongation factor-like protein
Sapur.001G141900.2	6.273362519	3.082499685	0.184848448	16.67582	1.96E-62	1.32E-61	cyan	0.699281792	2.30E-24	GO:0055114,GO:0016491	AT1G15550.1	ATGA3OX1,GA3OX1,GA4	gibberellin 3-oxidase 1	gibberellin 3-beta-hydroxylase
Sapur.018G098700.1	1794.937603	-1.788968519	0.107305885	-16.6717	2.11E-62	1.41E-61	cyan	-0.784636038	5.32E-34		AT2G10940.1		Bifunctional inhibitor/lipid-transfer protein/seed storage 2S albumin superfamily protein	protease inhibitor/seed storage/LTP family protein
Sapur.010G068200.1	379.0086721	-0.93328131	0.055986823	-16.6697	2.18E-62	1.46E-61	cyan	-0.793258478	3.22E-35		AT5G43750.1	NDH18	NAD(P)H dehydrogenase 18	NAD(P)H dehydrogenase
Sapur.013G023700.1	511.202311	1.376400904	0.082628713	16.65766	2.66E-62	1.78E-61	cyan	0.700699654	1.70E-24		AT3G05290.1	PNC1	peroxisomal adenine nucleotide carrier 1	peroxisomal adenine nucleotide carrier protein
Sapur.001G177700.1	399.901312	-0.617962546	0.03711284	-16.6509	2.98E-62	1.99E-61	cyan	-0.836905265	2.06E-42	GO:0005515	AT4G06599.1		ubiquitin family protein	ubiquitin-like domain CTD phosphatase
Sapur.007G000600.1	11.55985736	-2.749385821	0.165227726	-16.64	3.58E-62	2.39E-61	cyan	-0.810609694	7.46E-38					hypothetical protein
Sapur.004G025500.1	425.8504129	1.137150748	0.068351744	16.63675	3.78E-62	2.52E-61	cyan	0.731211276	1.55E-27		AT1G61065.1		Protein of unknown function (DUF1218)	plant/plant protein
Sapur.002G032600.1	68.91404366	1.730701409	0.104033283	16.63604	3.82E-62	2.55E-61	cyan	0.77667972	6.32E-33	GO:0055114,GO:0020037,GO:0016705,GO:0005506	AT3G48520.1	CYP94B3	cytochrome P450, family 94, subfamily B, polypeptide 3	cytochrome P450 family-dependent fatty acid hydroxylase
Sapur.018G070400.1	133.3532513	-1.264030749	0.076054508	-16.6201	4.99E-62	3.33E-61	cyan	-0.771034899	3.44E-32		AT5G57520.1	ATZFP2,ZFP2	zinc finger protein 2	zinc finger protein
Sapur.011G056500.1	346.5240596	1.104270255	0.066453701	16.61714	5.24E-62	3.49E-61	cyan	0.775064093	1.03E-32		AT2G34585.1			transmembrane protein, putative
Sapur.010G107800.1	133.1927424	-0.926481154	0.055756682	-16.6165	5.29E-62	3.53E-61	cyan	-0.822207796	9.00E-40	GO:0008236,GO:0006508,GO:0070008,GO:0004252	AT1G69020.1		Prolyl oligopeptidase family protein	prolyl oligopeptidase family protein
Sapur.016G223900.1	678.1402734	-0.462409985	0.027828773	-16.6163	5.32E-62	3.54E-61	cyan	-0.793132512	3.36E-35	GO:0006457,GO:0003755,GO:0000413	AT3G01480.1	ATCYP38,CYP38	cyclophilin 38	peptidyl-prolyl cis-trans isomerase
Sapur.008G057300.1	58.06636714	2.290581734	0.137862335	16.61499	5.43E-62	3.61E-61	cyan	0.620723563	4.27E-18	GO:0045454,GO:0015035,GO:0009055	AT5G58530.1		Glutaredoxin family protein	NA
Sapur.005G063600.1	152.0977788	-1.576295807	0.094928573	-16.6051	6.40E-62	4.26E-61	cyan	-0.814131734	2.01E-38		AT5G65207.1			hypothetical protein
Sapur.007G060500.1	37.86570398	-1.20246692	0.072492966	-16.5874	8.60E-62	5.71E-61	cyan	-0.792553313	4.07E-35	GO:0016021,GO:0006952	AT2G17480.1	ATMLO8,MLO8	Seven transmembrane MLO family protein	NA
Sapur.010G130700.1	12.75361299	-1.589536073	0.095883826	-16.5777	1.01E-61	6.70E-61	cyan	-0.80389631	8.38E-37		AT1G69588.1	CLE45	CLAVATA3/ESR-RELATED 45	hypothetical protein
Sapur.15WG084500.1	372.0752291	-0.819619427	0.049442908	-16.5771	1.02E-61	6.77E-61	cyan	-0.81495975	1.48E-38		AT1G74730.1		Protein of unknown function (DUF1118)	NA
Sapur.002G034000.1	526.811277	-1.285115415	0.077574335	-16.5662	1.22E-61	8.10E-61	cyan	-0.815711881	1.11E-38	GO:0006355,GO:0005634	AT3G04730.1	IAA16	indoleacetic acid-induced protein 16	auxin-responsive protein IAA16, putative
Sapur.004G020600.1	77.66930993	-1.518559288	0.091678338	-16.564	1.27E-61	8.40E-61	cyan	-0.783489772	7.64E-34		AT4G21445.1			receptor-interacting protein, putative
Sapur.003G017900.1	585.7731188	-0.829911925	0.050113625	-16.5606	1.34E-61	8.88E-61	cyan	-0.796534231	1.07E-35		AT1G72810.1		Pyridoxal-5\'-phosphate-dependent enzyme family protein	threonine synthase
Sapur.001G065400.1	45.18081179	-1.551677701	0.093713135	-16.5577	1.41E-61	9.31E-61	cyan	-0.803559882	9.44E-37		AT1G31320.1	LBD4	LOB domain-containing protein 4	LOB domain protein
Sapur.004G029200.1	153.5917139	-1.353143511	0.081738796	-16.5545	1.49E-61	9.83E-61	cyan	-0.786144846	3.29E-34	GO:0006468,GO:0005524,GO:0004672,GO:0030246	AT4G04960.1		Concanavalin A-like lectin protein kinase family protein	lectin-receptor-like kinase
Sapur.004G175600.1	49.77093431	1.757771964	0.106190604	16.55299	1.52E-61	1.01E-60	cyan	0.687333898	2.77E-23		AT3G54200.1		Late embryogenesis abundant (LEA) hydroxyproline-rich glycoprotein family	late embryogenesis abundant protein
Sapur.014G130600.1	176.9193308	-0.797961088	0.048218802	-16.5488	1.63E-61	1.08E-60	cyan	-0.809901728	9.67E-38	GO:0009245,GO:0008915	AT2G04560.1		transferases, transferring glycosyl groups	lipid-A-disaccharide synthase
Sapur.004G165600.1	240.2146314	-1.797107613	0.108622423	-16.5445	1.75E-61	1.16E-60	cyan	-0.753733281	4.65E-30		AT5G01750.2		Protein of unknown function (DUF567)	tubby C 2 protein
Sapur.008G042800.1	6.323928739	3.612697058	0.218393573	16.54214	1.82E-61	1.20E-60	cyan	0.697288643	3.51E-24		AT2G16050.1		Cysteine/Histidine-rich C1 domain family protein	DC1; C1-like protein, putative
Sapur.016G212500.1	11.89369405	4.139128497	0.250244607	16.54033	1.88E-61	1.24E-60	cyan	0.535350515	5.06E-13		AT5G40690.1			Hop-interacting protein THI031, putative
Sapur.016G044500.3	29.279246	1.360721925	0.082267665	16.54018	1.88E-61	1.24E-60	cyan	0.772597532	2.16E-32		AT3G12150.1			transmembrane protein, putative
Sapur.001G191900.1	25.13101621	2.291224553	0.13853563	16.53888	1.93E-61	1.27E-60	cyan	0.531795795	7.68E-13	GO:0016787	AT4G19420.1		Pectinacetylesterase family protein	pectinacetylesterase
Sapur.002G069300.1	561.6522494	0.794516171	0.048050557	16.535	2.05E-61	1.35E-60	cyan	0.782124622	1.17E-33		AT4G08230.1		glycine-rich protein	transmembrane protein, putative
Sapur.016G248400.1	702.406342	-0.572669586	0.034640347	-16.5319	2.16E-61	1.43E-60	cyan	-0.808993507	1.35E-37	GO:0005515	AT3G14860.2		NHL domain-containing protein	NHL domain protein
Sapur.002G168200.1	708.0144588	0.714052799	0.043201908	16.52827	2.30E-61	1.51E-60	cyan	0.79010776	9.11E-35		AT3G62240.1		RING/U-box superfamily protein	RING Zn-finger protein
Sapur.004G167500.1	25.0872656	-2.089900148	0.126469528	-16.5249	2.43E-61	1.60E-60	cyan	-0.768332508	7.62E-32		AT2G40475.1			hypothetical protein
Sapur.019G066100.1	66.66855715	-1.090400543	0.066055786	-16.5073	3.25E-61	2.14E-60	cyan	-0.804847936	5.98E-37	GO:0005515	AT2G40815.1		Calcium-dependent lipid-binding (CaLB domain) family protein	C2 domain protein
Sapur.002G018300.1	179.5540417	1.42138855	0.086110464	16.50657	3.29E-61	2.16E-60	cyan	0.726230084	5.19E-27	GO:0009733	AT1G75580.1		SAUR-like auxin-responsive protein family	auxin responsive protein
Sapur.T168700.1	56.57166063	-0.984797936	0.059681601	-16.5009	3.62E-61	2.38E-60	cyan	-0.779354498	2.78E-33	GO:0006284	AT3G07930.3		DNA glycosylase superfamily protein	base excision DNA repair protein, HhH-GPD family protein
Sapur.012G053500.1	324.5527855	0.610985527	0.037041761	16.49451	4.02E-61	2.64E-60	cyan	0.820672481	1.65E-39	GO:0006468,GO:0005524,GO:0004674	AT3G08720.1	ATPK19,ATPK2,ATS6K2,S6K2	serine/threonine protein kinase 2	ribosomal protein S6 kinase, putative
Sapur.018G068200.1	163.4560263	-0.768520054	0.046601143	-16.4914	4.23E-61	2.78E-60	cyan	-0.791258456	6.25E-35		AT5G57410.1		Afadin/alpha-actinin-binding protein	afadin and alpha actinin-binding protein
Sapur.011G030300.1	2237.134951	-0.94412911	0.057281079	-16.4824	4.91E-61	3.22E-60	cyan	-0.829872595	4.06E-41		AT4G21620.1		glycine-rich protein	hypothetical protein
Sapur.009G083400.1	28.12052635	-1.138075489	0.069075393	-16.4758	5.47E-61	3.59E-60	cyan	-0.792939295	3.58E-35		AT2G26520.1			transmembrane protein, putative
Sapur.006G115100.1	511.732626	-1.182233433	0.071762075	-16.4743	5.61E-61	3.68E-60	cyan	-0.822016965	9.71E-40	GO:0016020,GO:0009765	AT1G19150.1	LHCA2*1,LHCA6	photosystem I light harvesting complex gene 6	light-harvesting complex I chlorophyll-A/B-binding protein Lhca5
Sapur.005G077600.1	30.94949601	1.779745824	0.108040337	16.47298	5.74E-61	3.76E-60	cyan	0.661756541	3.90E-21	GO:0006355,GO:0003677	AT1G56010.2	anac021,ANAC022,NAC1	NAC domain containing protein 1	NAC transcription factor
Sapur.017G039200.2	30.84530865	-1.691384747	0.102721771	-16.4657	6.47E-61	4.24E-60	cyan	-0.764649966	2.21E-31	GO:0016021,GO:0004659	AT1G60600.2	ABC4	UbiA prenyltransferase family protein	UbiA family prenyltransferase
Sapur.T066700.1	13.78297024	2.554552107	0.155144191	16.46566	6.47E-61	4.24E-60	cyan	0.655565372	1.20E-20					NA
Sapur.001G010900.1	1029.884214	0.423090777	0.025701385	16.46179	6.90E-61	4.52E-60	cyan	0.791821236	5.19E-35		AT5G13030.1			UPF0061 protein TOL2-C18030 protein
Sapur.011G035900.1	143.6461259	-1.246797647	0.075752989	-16.4587	7.26E-61	4.75E-60	cyan	-0.775604848	8.76E-33		AT5G22620.2		phosphoglycerate/bisphosphoglycerate mutase family protein	phosphoglycerate mutase family protein
Sapur.016G092500.1	514.8724733	0.49493535	0.030076756	16.45574	7.63E-61	4.99E-60	cyan	0.77584594	8.14E-33		AT3G53970.1		proteasome inhibitor-related	proteasome inhibitor, putative
Sapur.017G060700.1	4.479108435	3.518045329	0.213795219	16.45521	7.70E-61	5.03E-60	cyan	0.7825374	1.03E-33		AT3G28470.1	ATMYB35,TDF1	Duplicated homeodomain-like superfamily protein	NA
Sapur.013G129900.1	3.852267642	4.468759052	0.271805958	16.44099	9.73E-61	6.36E-60	cyan	0.733370597	9.13E-28		AT4G03320.1	tic20-IV	translocon at the inner envelope membrane of chloroplasts 20-IV	NA
Sapur.006G199400.1	226.017955	-0.926034239	0.05635316	-16.4327	1.12E-60	7.28E-60	cyan	-0.826768055	1.45E-40	GO:0006413,GO:0003743,GO:0003723	AT4G11175.1		Nucleic acid-binding, OB-fold-like protein	translation initiation factor IF-1
Sapur.15WG035100.1	48.48795359	-0.776043534	0.047246699	-16.4253	1.26E-60	8.22E-60	cyan	-0.789723373	1.03E-34	GO:0003676,GO:0017069,GO:0000398	AT2G30260.1	U2B\'\'	U2 small nuclear ribonucleoprotein B	NA
Sapur.T190900.1	5.461306895	2.414816098	0.147018328	16.42527	1.26E-60	8.22E-60	cyan	0.771794248	2.75E-32		AT1G48550.1		Vacuolar protein sorting-associated protein 26	NA
Sapur.006G080200.1	14.71364994	-1.964038006	0.119657319	-16.4139	1.52E-60	9.92E-60	cyan	-0.812188286	4.16E-38		AT3G09032.1			NA
Sapur.017G077100.1	10.73731188	2.740715566	0.167012836	16.41021	1.62E-60	1.05E-59	cyan	0.707541807	3.82E-25	GO:0017025,GO:0008270,GO:0006355,GO:0006352	AT3G10330.1		Cyclin-like family protein	transcription initiation factor IIB
Sapur.013G109600.1	169.9429232	1.891374984	0.115277334	16.40717	1.70E-60	1.11E-59	cyan	0.728604122	2.93E-27		AT2G42570.1	TBL39	TRICHOME BIREFRINGENCE-LIKE 39	Pmr5/Cas1p GDSL/SGNH-like acyl-esterase family protein
Sapur.005G035800.1	57.82634005	-1.266218999	0.077205674	-16.4006	1.89E-60	1.23E-59	cyan	-0.747138741	2.72E-29		AT4G25170.1		Uncharacterised conserved protein (UCP012943)	hypothetical protein
Sapur.002G017700.1	71.57091136	-1.265583529	0.077177558	-16.3983	1.97E-60	1.28E-59	cyan	-0.799363825	4.08E-36	GO:0008168,GO:0008152	AT4G31790.1		Tetrapyrrole (Corrin/Porphyrin) Methylases	diphthine synthase
Sapur.018G006200.1	265.3248019	1.016054676	0.061973679	16.39494	2.08E-60	1.35E-59	cyan	0.754423644	3.86E-30	GO:0005515	AT2G24540.1	AFR	Galactose oxidase/kelch repeat superfamily protein	F-box protein AFR
Sapur.014G098400.1	73.94109919	-0.643782247	0.039274148	-16.392	2.18E-60	1.42E-59	cyan	-0.801627874	1.86E-36		AT2G25920.1			hypothetical protein
Sapur.008G075400.1	56.65142379	-1.43168533	0.087341741	-16.3918	2.19E-60	1.42E-59	cyan	-0.771891147	2.67E-32	GO:0071705	AT2G03530.2	ATUPS2,UPS2	ureide permease 2	ureide permease
Sapur.014G114000.1	7849.928779	-0.578810713	0.035314118	-16.3903	2.24E-60	1.46E-59	cyan	-0.788474344	1.55E-34	GO:0055114,GO:0016620	AT3G26650.1	GAPA,GAPA-1	glyceraldehyde 3-phosphate dehydrogenase A subunit	glyceraldehyde-3-phosphate dehydrogenase
Sapur.004G155100.1	2368.704369	1.074667377	0.065598389	16.38253	2.55E-60	1.65E-59	cyan	0.775659787	8.62E-33		AT2G17230.1	EXL5	EXORDIUM like 5	phosphate-induced protein 1 motif protein
Sapur.15WG086900.1	51.239275	-1.326360337	0.080982266	-16.3784	2.73E-60	1.77E-59	cyan	-0.782742334	9.68E-34	GO:0016853,GO:0005975	AT3G04880.1	DRT102	DNA-damage-repair/toleration protein (DRT102)	NA
Sapur.012G039400.1	289.7701548	1.361105871	0.083124208	16.37436	2.92E-60	1.89E-59	cyan	0.742112905	1.01E-28		AT3G02550.1	LBD41	LOB domain-containing protein 41	NA
Sapur.006G166800.1	44.50520897	1.413201845	0.086316482	16.37233	3.01E-60	1.95E-59	cyan	0.745756638	3.91E-29					hypothetical protein
Sapur.014G116900.1	2.566260081	4.160266528	0.254109116	16.37197	3.03E-60	1.97E-59	cyan	0.779744008	2.47E-33					hypothetical protein
Sapur.15WG107200.1	122.9544355	2.608716298	0.159363706	16.36958	3.15E-60	2.04E-59	cyan	0.66064833	4.78E-21		AT1G24440.1		RING/U-box superfamily protein	ubiquitin-protein ligase, putative
Sapur.005G089800.1	126.7108085	-1.077874696	0.06584665	-16.3695	3.16E-60	2.05E-59	cyan	-0.804500744	6.77E-37		AT3G51290.2		Protein of unknown function (DUF630) ;Protein of unknown function (DUF632)	DUF632 family protein
Sapur.012G099100.1	23.25354214	-1.454842724	0.088925728	-16.3602	3.68E-60	2.38E-59	cyan	-0.770974555	3.50E-32					transmembrane protein, putative
Sapur.15WG021900.1	371.7539132	0.760833672	0.046507569	16.35935	3.73E-60	2.41E-59	cyan	0.766573477	1.27E-31		AT5G08200.1		peptidoglycan-binding LysM domain-containing protein	peptidoglycan-binding LysM domain protein, putative
Sapur.005G109600.1	1074.88829	-1.155945573	0.070680074	-16.3546	4.03E-60	2.61E-59	cyan	-0.860706446	2.66E-47		AT5G67070.1	RALFL34	ralf-like 34	NA
Sapur.010G011400.1	157.3054137	-0.995692388	0.060965319	-16.3321	5.83E-60	3.77E-59	cyan	-0.79476674	1.95E-35		AT3G06145.1			hypothetical protein
Sapur.011G055900.1	323.0513213	-0.712390997	0.043625363	-16.3297	6.06E-60	3.91E-59	cyan	-0.796722053	1.01E-35	GO:0006508,GO:0004252	AT4G18370.1	DEG5,DEGP5,HHOA	DEGP protease 5	protease Do-like protein
Sapur.001G164000.1	169.3466576	-1.297935311	0.079488886	-16.3285	6.19E-60	3.99E-59	cyan	-0.789825195	9.99E-35	GO:0051536,GO:0009055	AT3G16250.1	NDF4	NDH-dependent cyclic electron flow 1	electron carrier/electron transporter/iron ion-binding protein
Sapur.016G129700.1	88.19276802	-1.198703445	0.073424416	-16.3257	6.48E-60	4.18E-59	cyan	-0.841070201	3.28E-43	GO:0003676	AT3G46020.1		RNA-binding (RRM/RBD/RNP motifs) family protein	cold-inducible RNA-binding protein
Sapur.016G216600.1	1405.709357	0.512648618	0.031413769	16.31923	7.20E-60	4.64E-59	cyan	0.815525068	1.19E-38	GO:0048280,GO:0006886,GO:0005737,GO:0000139,GO:0016020,GO:0008565,GO:00481	AT3G27530.1	GC6,MAG4	golgin candidate 6	coiled-coil vesicle tethering protein, putative
Sapur.016G294500.1	160.3083119	-0.856315572	0.052482224	-16.3163	7.56E-60	4.87E-59	cyan	-0.800432243	2.82E-36	GO:0006468,GO:0005524,GO:0004672,GO:0030246	AT5G06740.1		Concanavalin A-like lectin protein kinase family protein	lectin-like kinase, putative
Sapur.003G022800.1	3.708345618	4.779068523	0.293010629	16.31022	8.35E-60	5.38E-59	cyan	0.665056191	2.12E-21					NA
Sapur.005G160400.1	90.77387682	-1.209562958	0.074159812	-16.3102	8.35E-60	5.38E-59	cyan	-0.789280888	1.19E-34		AT1G06475.1			transmembrane protein, putative
Sapur.014G059500.2	8.803367818	2.252335743	0.13812889	16.30604	8.94E-60	5.75E-59	cyan	0.752457591	6.58E-30	GO:0008168	AT4G00750.1		S-adenosyl-L-methionine-dependent methyltransferases superfamily protein	dehydration-responsive family protein
Sapur.003G062500.1	361.487025	0.608580057	0.037323819	16.30541	9.03E-60	5.81E-59	cyan	0.772712847	2.09E-32		AT1G32260.1			hypothetical protein
Sapur.010G206400.1	700.2311675	-0.599795608	0.036800851	-16.2984	1.01E-59	6.51E-59	cyan	-0.806601127	3.20E-37		AT5G08380.1	AGAL1,AtAGAL1	alpha-galactosidase 1	alpha-galactosidase/alpha-N-acetylgalactosaminidase, putative
Sapur.014G136700.1	236.1374727	-1.087669574	0.06675916	-16.2924	1.12E-59	7.18E-59	cyan	-0.788557445	1.51E-34		AT4G14540.1	NF-YB3	nuclear factor Y, subunit B3	nuclear transcription factor Y subunit B-3
Sapur.010G088100.1	18.06103931	2.545504669	0.156259422	16.29025	1.16E-59	7.44E-59	cyan	0.64815127	4.47E-20	GO:0008270	AT2G01275.1		RING/FYVE/PHD zinc finger superfamily protein	membrane associated RING finger protein 1%2C8, putative
Sapur.011G003200.1	602.1163372	0.794523698	0.04879714	16.28218	1.32E-59	8.48E-59	cyan	0.73338897	9.09E-28	GO:0005737,GO:0000045	AT1G62040.2	ATG8C	Ubiquitin-like superfamily protein	gaba(A) receptor-associated protein, putative
Sapur.002G048500.1	7.145574992	-2.065041389	0.126861025	-16.278	1.41E-59	9.08E-59	cyan	-0.80002654	3.24E-36	GO:0055114,GO:0020037,GO:0016705,GO:0005506	AT2G42850.1	CYP718	cytochrome P450, family 718	cytochrome P450 family protein
Sapur.010G064900.1	136.0604782	-1.50766583	0.092638836	-16.2747	1.49E-59	9.59E-59	cyan	-0.783905174	6.70E-34	GO:0005515	AT3G22800.1		Leucine-rich repeat (LRR) family protein	Serine/Threonine kinase, plant-type protein%2C putative
Sapur.002G192200.1	156.6391923	-1.021828091	0.062793558	-16.2728	1.54E-59	9.88E-59	cyan	-0.73715788	3.55E-28		AT3G07310.1		Protein of unknown function (DUF760)	DUF760 family protein
Sapur.004G096500.1	14444.34992	-0.889573071	0.054667086	-16.2726	1.55E-59	9.92E-59	cyan	-0.77355958	1.62E-32		AT1G67090.1	RBCS1A	ribulose bisphosphate carboxylase small chain 1A	ribulose bisphosphate carboxylase small chain
Sapur.010G145100.1	226.4097717	1.458118762	0.089608036	16.27219	1.56E-59	9.97E-59	cyan	0.65851069	7.06E-21	GO:0006468,GO:0004672	AT5G07150.1		Leucine-rich repeat protein kinase family protein	tyrosine kinase domain protein
Sapur.004G134500.1	21.38115793	-1.254663514	0.0771102	-16.271	1.58E-59	1.02E-58	cyan	-0.811310017	5.76E-38		AT4G35130.1		Tetratricopeptide repeat (TPR)-like superfamily protein	NA
Sapur.009G127900.1	60.21876526	-0.847925772	0.052145433	-16.2608	1.87E-59	1.20E-58	cyan	-0.802607458	1.32E-36		AT2G27470.1	NF-YB11	nuclear factor Y, subunit B11	histone-like transcription factor and archaeal histone protein
Sapur.002G108800.1	261.4586224	0.830712459	0.051090286	16.25969	1.91E-59	1.22E-58	cyan	0.771831287	2.72E-32	GO:0016559,GO:0005779	AT1G47750.1	PEX11A	peroxin 11A	peroxisomal biogenesis factor 11
Sapur.007G107400.1	24.24394762	2.884746659	0.177424739	16.25899	1.93E-59	1.24E-58	cyan	0.531510766	7.94E-13	GO:0009055	AT2G32300.1	UCC1	uclacyanin 1	plastocyanin-like domain protein
Sapur.010G027100.1	350.1326249	-0.765340059	0.047081161	-16.2558	2.03E-59	1.30E-58	cyan	-0.826176444	1.84E-40		AT1G60790.1	TBL2	Plant protein of unknown function (DUF828)	Pmr5/Cas1p GDSL/SGNH-like acyl-esterase family protein
Sapur.016G005400.1	278.6884704	0.650257689	0.040008963	16.2528	2.13E-59	1.37E-58	cyan	0.786483547	2.95E-34	GO:0016787	AT5G47650.1	ATNUDT2,ATNUDX2,NUDT2	nudix hydrolase homolog 2	nudix hydrolase
Sapur.014G016900.1	16.17232895	-1.807284199	0.11120595	-16.2517	2.17E-59	1.39E-58	cyan	-0.759935339	8.41E-31		AT1G45207.2		Remorin family protein	remorin
Sapur.008G047800.1	278.8118222	0.533241894	0.032817212	16.24885	2.28E-59	1.46E-58	cyan	0.783326305	8.05E-34	GO:0016020	AT2G39805.2		Integral membrane Yip1 family protein	NA
Sapur.009G063900.1	744.1459709	0.660790044	0.040669034	16.24799	2.31E-59	1.48E-58	cyan	0.755682667	2.73E-30		AT5G64510.1			nicotiana tabacum ORF protein
Sapur.15WG083300.1	57.70746675	-1.013507858	0.062394172	-16.2436	2.48E-59	1.58E-58	cyan	-0.76539074	1.79E-31	GO:0003676,GO:0017069,GO:0000398	AT2G30260.1	U2B\'\'	U2 small nuclear ribonucleoprotein B	NA
Sapur.010G204100.1	302.2121303	-0.762720587	0.046955338	-16.2435	2.48E-59	1.59E-58	cyan	-0.809385187	1.17E-37		AT1G16860.1		Ubiquitin-specific protease family C19-related protein	cysteine-type endopeptidase/ubiquitin thiolesterase
Sapur.006G043400.1	69.80523359	-1.706749038	0.105086515	-16.2414	2.57E-59	1.64E-58	cyan	-0.752438911	6.61E-30					transmembrane protein, putative
Sapur.005G197100.1	1025.80536	-1.391736075	0.085704573	-16.2388	2.68E-59	1.71E-58	cyan	-0.795769454	1.39E-35		AT1G76100.1	PETE1	plastocyanin 1	NA
Sapur.001G069700.1	169.6056995	0.820305589	0.0505247	16.23573	2.82E-59	1.80E-58	cyan	0.763108862	3.43E-31	GO:0016021,GO:0005789	AT5G42000.1		ORMDL family protein	ORMDL family protein
Sapur.005G190800.1	591.7057307	-0.865425442	0.053305423	-16.2352	2.84E-59	1.81E-58	cyan	-0.778277304	3.88E-33		AT5G42560.1		Abscisic acid-responsive (TB2/DP1, HVA22) family protein	HVA22-like protein I
Sapur.004G163700.1	781.3951839	-0.743073041	0.045781654	-16.2308	3.05E-59	1.95E-58	cyan	-0.815278723	1.31E-38	GO:0006412,GO:0005840,GO:0005622,GO:0003735	AT3G44890.1	RPL9	ribosomal protein L9	50S ribosomal protein L9
Sapur.017G107800.1	126.4468191	-1.22306399	0.075375318	-16.2263	3.29E-59	2.09E-58	cyan	-0.798497559	5.49E-36	GO:0007264,GO:0005525	AT5G37680.1	ARLA1A,ATARLA1A	ADP-ribosylation factor-like A1A	ADP-ribosylation factor-like protein
Sapur.013G125800.1	79.69358383	-1.004349473	0.061913676	-16.2217	3.54E-59	2.26E-58	cyan	-0.79861189	5.28E-36		AT1G03670.1		ankyrin repeat family protein	ankyrin repeat protein
Sapur.004G144400.1	13.6552895	4.929068497	0.303921786	16.21821	3.75E-59	2.39E-58	cyan	0.642375111	1.21E-19					NA
Sapur.006G105700.1	206.695108	1.551653668	0.095708567	16.21228	4.13E-59	2.63E-58	cyan	0.729879293	2.15E-27		AT3G09770.1		RING/U-box superfamily protein	E3 ubiquitin-protein ligase, putative
Sapur.001G099300.1	617.8389944	0.983862156	0.060702167	16.20802	4.43E-59	2.81E-58	cyan	0.726266777	5.14E-27		AT4G22580.1		Exostosin family protein	exostosin family protein
Sapur.013G111400.1	585.3837717	0.651289148	0.040183785	16.20776	4.44E-59	2.83E-58	cyan	0.76556705	1.70E-31		AT3G54680.1		proteophosphoglycan-related	plant/T5N23-40 protein, putative
Sapur.005G182300.1	133.7920319	-1.074613346	0.066311403	-16.2056	4.61E-59	2.93E-58	cyan	-0.813517788	2.54E-38	GO:0016567,GO:0004842	AT4G36550.1		ARM repeat superfamily protein	ubiquitin-protein ligase, putative
Sapur.009G124800.1	1933.334927	0.808929338	0.049934259	16.19989	5.05E-59	3.21E-58	cyan	0.756760547	2.03E-30	GO:0016705,GO:0016117	AT3G10230.1	LYC	lycopene cyclase	capsanthin/capsorubin synthase, chromoplast protein
Sapur.001G187400.1	212.1956338	-0.797686317	0.049252068	-16.196	5.38E-59	3.42E-58	cyan	-0.785126966	4.55E-34	GO:0005524	AT5G49840.1		ATP-dependent Clp protease	ATP-dependent Clp protease ATP-binding subunit CLPX protein, putative
Sapur.019G052100.1	1810.170462	0.724903818	0.044772925	16.19067	5.87E-59	3.72E-58	cyan	0.755159738	3.15E-30	GO:0016787,GO:0008299,GO:0004452	AT3G02780.1	IDI2,IPIAT1,IPP2	isopentenyl pyrophosphate:dimethylallyl pyrophosphate isomerase 2	isopentenyl diphosphate isomerase
Sapur.007G091100.1	224.4089255	0.746942458	0.046156532	16.18281	6.67E-59	4.23E-58	cyan	0.761415806	5.55E-31	GO:0007034	AT5G09260.1	VPS20.2	vacuolar protein sorting-associated protein 20.2	NA
Sapur.008G103100.1	423.9584209	0.543826058	0.033607398	16.18174	6.79E-59	4.30E-58	cyan	0.774060662	1.40E-32	GO:0016020,GO:0008375	AT1G71070.1		Core-2/I-branching beta-1,6-N-acetylglucosaminyltransferase family protein	acetylglucosaminyltransferase, putative
Sapur.007G058000.1	441.1011395	-0.714023424	0.044139459	-16.1765	7.38E-59	4.67E-58	cyan	-0.798118722	6.25E-36		AT1G79190.1		ARM repeat superfamily protein	Tel2-interacting protein
Sapur.016G049700.1	4.144712867	5.365826201	0.331888734	16.16755	8.54E-59	5.40E-58	cyan	0.683451981	6.07E-23	GO:0006355,GO:0003677	AT1G69490.1	ANAC029,ATNAP,NAP	NAC-like, activated by AP3/PI	NAC domain protein
Sapur.005G127900.1	374.530047	0.978004293	0.060498202	16.16584	8.78E-59	5.56E-58	cyan	0.753476989	4.99E-30	GO:0045454	AT1G34780.1	APRL4,ATAPRL4	APR-like 4	5'-adenylylsulfate reductase-like protein
Sapur.T009500.1	5.856553354	3.43694097	0.212692085	16.15923	9.78E-59	6.18E-58	cyan	0.695319126	5.32E-24					NA
Sapur.011G082700.1	3392.326358	0.608842162	0.037685379	16.15592	1.03E-58	6.52E-58	cyan	0.758976678	1.10E-30	GO:0010155,GO:0005886,GO:0005783	AT4G27500.1	PPI1	proton pump interactor 1	DNA double-strand break repair rad50 ATPase, putative
Sapur.009G129000.1	57.08272876	1.122816037	0.069561482	16.14135	1.31E-58	8.25E-58	cyan	0.738264986	2.68E-28		AT3G44510.2		alpha/beta-Hydrolases superfamily protein	alpha/beta hydrolase family protein
Sapur.003G129900.1	2.274221708	4.592648707	0.284574764	16.13864	1.37E-58	8.62E-58	cyan	0.669307627	9.53E-22	GO:0000160	AT3G04280.2	ARR22,RR22	response regulator 22	NA
Sapur.007G059000.1	78.67307528	-0.91914719	0.056966829	-16.1348	1.45E-58	9.17E-58	cyan	-0.801057457	2.27E-36		AT1G79060.1			hypothetical protein
Sapur.008G083600.1	100.8683989	-1.764189712	0.109365059	-16.1312	1.54E-58	9.71E-58	cyan	-0.814954566	1.48E-38		AT4G24972.1	TPD1	tapetum determinant 1	DNA-directed RNA polymerase, putative
Sapur.006G169500.1	135.96671	-0.89868946	0.055717709	-16.1293	1.59E-58	1.00E-57	cyan	-0.831865484	1.77E-41	GO:0016787,GO:0008152	AT3G02875.1	ILR1	Peptidase M20/M25/M40 family protein	IAA-amino acid hydrolase ILR1, putative
Sapur.010G059900.5	10.69535353	-1.874017067	0.11619475	-16.1282	1.62E-58	1.02E-57	cyan	-0.790883409	7.07E-35	GO:0006457	AT5G45680.1	ATFKBP13,FKBP13	FK506-binding protein 13	peptidyl-prolyl cis-trans isomerase
Sapur.012G041600.1	8.568869674	-2.043209002	0.126685948	-16.1281	1.62E-58	1.02E-57	cyan	-0.773197496	1.81E-32					hypothetical protein
Sapur.001G049800.1	556.5377599	1.632493434	0.101256445	16.12237	1.78E-58	1.12E-57	cyan	0.72730861	4.01E-27	GO:0008081,GO:0006629	AT5G41080.1		PLC-like phosphodiesterases superfamily protein	NA
Sapur.007G045500.1	517.6765019	0.814186725	0.050507176	16.12022	1.84E-58	1.16E-57	cyan	0.746587389	3.14E-29	GO:0005515	AT5G67250.1	SKIP2,VFB4	SKP1/ASK1-interacting protein 2	F-box protein SKIP2
Sapur.003G130900.1	1129.380563	0.642392723	0.03986645	16.11362	2.05E-58	1.29E-57	cyan	0.78150621	1.43E-33		AT1G04630.1	MEE4	GRIM-19 protein	NADH dehydrogenase [ubiquinone] 1 alpha subcomplex protein
Sapur.004G052800.1	7652.009698	0.829865244	0.051501388	16.11345	2.05E-58	1.29E-57	cyan	0.772502158	2.23E-32	GO:0030170,GO:0019752,GO:0016831	AT5G17330.1	GAD,GAD1	glutamate decarboxylase	glutamate decarboxylase
Sapur.005G203300.1	656.3202815	0.51250018	0.031809873	16.11136	2.12E-58	1.33E-57	cyan	0.776364465	6.96E-33	GO:0003824,GO:0006470,GO:0004722	AT2G33700.1		Protein phosphatase 2C family protein	protein phosphatase 2C, putative
Sapur.008G035300.1	101.1562179	-0.704043103	0.043700393	-16.1107	2.15E-58	1.35E-57	cyan	-0.78603074	3.41E-34	GO:0033588	AT4G10090.1	ELP6	elongator protein 6	elongator complex protein
Sapur.001G037500.1	512.924973	0.568406238	0.035300958	16.10172	2.48E-58	1.56E-57	cyan	0.756430444	2.23E-30	GO:0005634	AT1G80780.1		Polynucleotidyl transferase, ribonuclease H-like superfamily protein	CCR4-associated factor, putative
Sapur.005G205200.1	10.94998257	-1.73902187	0.108022487	-16.0987	2.61E-58	1.64E-57	cyan	-0.797758151	7.07E-36	GO:0046872,GO:0030001	AT3G56891.1		Heavy metal transport/detoxification superfamily protein	NA
Sapur.007G108200.1	132.3139527	-1.403299897	0.087171879	-16.0981	2.63E-58	1.65E-57	cyan	-0.742870472	8.27E-29		AT3G21870.1	CYCP2;1	cyclin p2;1	cyclin-U1-1 protein
Sapur.016G023000.1	4799.614046	0.918896412	0.05712047	16.08699	3.15E-58	1.97E-57	cyan	0.772041262	2.55E-32		AT2G27030.3	ACAM-2,CAM5	calmodulin 5	calmodulin cam-203
Sapur.008G082500.1	215.3098857	1.301284477	0.080917911	16.08154	3.44E-58	2.15E-57	cyan	0.726744496	4.59E-27	GO:0005509	AT5G49480.1	ATCP1,CP1	Ca2+-binding protein 1	nacl-inducible Ca2+-binding protein
Sapur.15WG004000.1	223.0415786	-0.867012252	0.053924383	-16.0783	3.62E-58	2.27E-57	cyan	-0.810779143	7.01E-38		AT5G24500.1			hypothetical protein
Sapur.014G087100.1	340.1998944	1.054307493	0.065612576	16.06868	4.23E-58	2.65E-57	cyan	0.738683871	2.41E-28		AT1G28200.1	FIP1	FH interacting protein 1	NA
Sapur.004G033000.1	36.18629825	1.917224008	0.119329907	16.06658	4.38E-58	2.74E-57	cyan	0.670474296	7.64E-22	GO:0006468,GO:0004672	AT5G46080.1		Protein kinase superfamily protein	tyrosine kinase domain protein
Sapur.017G039800.1	3341.909815	1.354800141	0.084330953	16.06528	4.47E-58	2.79E-57	cyan	0.662451812	3.43E-21		AT5G64260.1	EXL2	EXORDIUM like 2	phosphate-induced protein 1 motif protein
Sapur.018G040100.1	360.8218069	0.832647723	0.051874478	16.0512	5.61E-58	3.50E-57	cyan	0.759091832	1.06E-30		AT2G26070.1	RTE1	Protein of unknown function (DUF778)	green-ripe protein
Sapur.002G171500.1	70.38906058	-1.239254969	0.077266908	-16.0386	6.87E-58	4.28E-57	cyan	-0.805471822	4.79E-37	GO:0005524,GO:0004797	AT3G07800.1		Thymidine kinase	thymidine kinase
Sapur.017G050100.1	19448.07995	0.890825917	0.055548027	16.03704	7.04E-58	4.39E-57	cyan	0.760798828	6.60E-31	GO:0055114,GO:0051287,GO:0006730,GO:0004013	AT4G13940.1	ATSAHH1,EMB1395,HOG1,N	S-adenosyl-L-homocysteine hydrolase	S-adenosyl-L-homocysteine hydrolase
Sapur.016G260600.1	144.1745746	-1.451955084	0.090542599	-16.0362	7.14E-58	4.45E-57	cyan	-0.757324599	1.74E-30		AT4G27460.1		Cystathionine beta-synthase (CBS) family protein	NA
Sapur.019G068600.1	1478.619903	0.469111387	0.029264825	16.02987	7.90E-58	4.93E-57	cyan	0.800767561	2.51E-36	GO:0005525,GO:0003924	AT1G59610.1	ADL3,CF1,DL3,DRP2B	dynamin-like 3	NA
Sapur.15WG002200.1	9.695440057	-2.237313453	0.139649598	-16.0209	9.13E-58	5.68E-57	cyan	-0.75386694	4.49E-30		AT4G27660.1			hypothetical protein
Sapur.013G087100.1	7.028969968	2.977578477	0.185895733	16.01747	9.65E-58	6.01E-57	cyan	0.685264983	4.21E-23					NA
Sapur.011G075200.1	330.3158717	0.387192219	0.024176356	16.01533	9.99E-58	6.21E-57	cyan	0.792863972	3.67E-35		AT1G78800.1		UDP-Glycosyltransferase superfamily protein	alpha-1%2C3-mannosyltransferase ALG2
Sapur.017G003300.1	312.8894794	-0.671859724	0.04195926	-16.0122	1.05E-57	6.53E-57	cyan	-0.801304988	2.08E-36		AT2G43950.1	ATOEP37,OEP37	chloroplast outer envelope protein 37	outer envelope pore protein
Sapur.012G057900.1	362.478589	-0.750704317	0.046897543	-16.0073	1.14E-57	7.06E-57	cyan	-0.802099558	1.58E-36	GO:0006396,GO:0004525,GO:0003723	AT1G24450.1	NFD2	Ribonuclease III family protein	ribonuclease-III-like protein
Sapur.004G141400.1	62.10358965	1.271206129	0.079445459	16.00099	1.26E-57	7.81E-57	cyan	0.710555908	1.96E-25		AT2G27770.1		Plant protein of unknown function (DUF868)	DUF868 family protein
Sapur.15WG129400.1	129.4525953	-1.062273072	0.066406774	-15.9965	1.35E-57	8.40E-57	cyan	-0.796387481	1.13E-35	GO:0055114,GO:0008113,GO:0030091,GO:0016671,GO:0006979	AT4G25130.1	PMSR4	peptide met sulfoxide reductase 4	peptide methionine sulfoxide reductase
Sapur.011G029600.1	1111.108246	0.621615931	0.038860674	15.99602	1.36E-57	8.46E-57	cyan	0.752038775	7.36E-30	GO:0055114,GO:0016491,GO:0008270	AT4G21580.1		oxidoreductase, zinc-binding dehydrogenase family protein	NADPH quinone oxidoreductase, putative
Sapur.15ZG035800.1	12.71728501	2.144078112	0.134112368	15.98718	1.57E-57	9.74E-57	cyan	0.674056435	3.84E-22	GO:0006468,GO:0005524,GO:0004672	AT5G63610.1	ATCDK8,CDKE;1,HEN3	cyclin-dependent kinase E;1	cyclin-dependent kinase E-1
Sapur.007G055200.1	19.93656143	-1.497591582	0.093692644	-15.9841	1.65E-57	1.02E-56	cyan	-0.75978274	8.78E-31		AT2G17510.1	EMB2763	ribonuclease II family protein	NA
Sapur.016G262900.1	6.895915796	-2.286537395	0.143051631	-15.984	1.65E-57	1.02E-56	cyan	-0.769230294	5.86E-32		AT1G52855.1			hypothetical protein
Sapur.018G078700.1	487.9226711	0.801785301	0.05017428	15.98001	1.76E-57	1.09E-56	cyan	0.755513969	2.86E-30		AT4G30790.1			autophagy-related protein
Sapur.011G105300.1	8085.140657	-0.910279315	0.056972245	-15.9776	1.83E-57	1.13E-56	cyan	-0.800331526	2.92E-36	GO:0042651,GO:0015979,GO:0009654,GO:0009523	AT1G79040.1	PSBR	photosystem II subunit R	photosystem II 10 kDa protein
Sapur.011G054600.1	244.7514176	-1.212308886	0.075878215	-15.977	1.85E-57	1.14E-56	cyan	-0.798680677	5.15E-36	GO:0006355,GO:0005739,GO:0003690	AT2G34620.1		Mitochondrial transcription termination factor family protein	mTERF-like protein
Sapur.012G105700.1	17.44117703	-2.88330427	0.180523714	-15.9719	2.01E-57	1.24E-56	cyan	-0.680624553	1.07E-22	GO:0016788	AT1G29670.1		GDSL-like Lipase/Acylhydrolase superfamily protein	GDSL-lipase, putative
Sapur.013G066700.1	337.6178783	1.36988194	0.085770019	15.97157	2.02E-57	1.25E-56	cyan	0.641420834	1.43E-19					NA
Sapur.002G108100.1	294.2165544	1.13817204	0.071280918	15.96742	2.16E-57	1.33E-56	cyan	0.714876525	7.37E-26	GO:0016757	AT1G19300.1	ATGATL1,GATL1,GLZ1,PARV	Nucleotide-diphospho-sugar transferases superfamily protein	glycosyltransferase family GT8 protein
Sapur.009G007100.1	153.46259	-0.7907931	0.049545038	-15.9611	2.39E-57	1.48E-56	cyan	-0.782052086	1.20E-33	GO:0008289,GO:0003677	AT5G60690.1	IFL,IFL1,REV	Homeobox-leucine zipper family protein / lipid-binding START domain-containing protein	NA
Sapur.001G142000.1	11.07183016	2.823897292	0.177100212	15.94519	3.08E-57	1.90E-56	cyan	0.596312621	1.72E-16					NA
Sapur.002G125900.1	29.80398398	-1.276548873	0.080072721	-15.9424	3.22E-57	1.99E-56	cyan	-0.770458821	4.08E-32	GO:0006355,GO:0003677	AT1G33060.2	ANAC014,NAC014	NAC 014	NAC domain protein
Sapur.008G113600.1	135.8669429	-0.812796866	0.051019251	-15.9299	3.93E-57	2.43E-56	cyan	-0.784308018	5.90E-34		AT1G68130.1	AtIDD14,IDD14	indeterminate(ID)-domain 14	C2H2L domain class transcription factor
Sapur.019G081100.1	397.9625315	0.598005987	0.03754549	15.92751	4.08E-57	2.52E-56	cyan	0.765249632	1.85E-31		AT1G71940.1		SNARE associated Golgi protein family	SNARE associated protein
Sapur.003G100600.1	78.40783397	-0.88235541	0.055407415	-15.9249	4.26E-57	2.63E-56	cyan	-0.760461458	7.26E-31	GO:0006396,GO:0005622	AT5G41770.1		crooked neck protein, putative / cell cycle protein, putative	cell cycle control protein crooked neck-like protein, putative
Sapur.016G291500.1	79.56395587	-1.332463611	0.083673848	-15.9245	4.28E-57	2.64E-56	cyan	-0.775652278	8.63E-33		AT1G61570.1	TIM13	translocase of the inner mitochondrial membrane 13	import inner membrane translocase subunit Tim13, putative
Sapur.005G134700.1	1321.738681	-0.736625934	0.04627793	-15.9174	4.80E-57	2.95E-56	cyan	-0.785018796	4.71E-34	GO:0006412,GO:0005840,GO:0005622,GO:0003735,GO:0003723	AT5G14320.1		Ribosomal protein S13/S18 family	30S ribosomal protein S13
Sapur.006G139300.4	12.86361852	-1.406489373	0.088402325	-15.9101	5.39E-57	3.32E-56	cyan	-0.79967961	3.66E-36	GO:0030170,GO:0008483	AT5G57590.1	BIO1	adenosylmethionine-8-amino-7-oxononanoate transaminases	NA
Sapur.004G076400.1	846.6071023	0.738909898	0.046473048	15.89975	6.36E-57	3.91E-56	cyan	0.744323987	5.68E-29	GO:0003333	AT3G30390.1		Transmembrane amino acid transporter family protein	amino acid transporter
Sapur.002G196900.1	27.25598025	-2.105872426	0.132448818	-15.8995	6.39E-57	3.92E-56	cyan	-0.736901674	3.79E-28	GO:0000160	AT5G26594.1	ARR24,RR24	response regulator 24	extra response regulator
Sapur.010G009000.1	681.1093712	1.011851394	0.063641237	15.8993	6.41E-57	3.94E-56	cyan	0.725054739	6.87E-27	GO:0005515	AT4G15840.1		BTB/POZ domain-containing protein	BTB/POZ domain protein
Sapur.001G046700.1	2830.802748	1.114597574	0.070104635	15.89906	6.43E-57	3.95E-56	cyan	0.7514212	8.70E-30	GO:0005783	AT1G27350.1		Ribosome associated membrane protein RAMP4	ribosome associated membrane protein RAMP4
Sapur.002G136000.1	96.14760401	0.951647499	0.059864634	15.89666	6.68E-57	4.10E-56	cyan	0.72802515	3.37E-27		AT4G01575.1		serine protease inhibitor, Kazal-type family protein	serine-type endopeptidase inhibitor, putative
Sapur.016G232300.1	171.2908107	-0.501044928	0.031523873	-15.8941	6.96E-57	4.27E-56	cyan	-0.780263349	2.10E-33		AT1G55880.1		Pyridoxal-5\'-phosphate-dependent enzyme family protein	cysteine synthase
Sapur.017G030200.1	44.0708443	-1.777361726	0.111827612	-15.8938	7.00E-57	4.30E-56	cyan	-0.767405018	9.98E-32		AT3G22070.1		proline-rich family protein	transmembrane protein, putative
Sapur.007G129900.1	47.94443794	-0.810064324	0.050972751	-15.8921	7.19E-57	4.41E-56	cyan	-0.791540321	5.69E-35	GO:0005975,GO:0004649	AT2G31870.2	TEJ	Poly (ADP-ribose) glycohydrolase (PARG)	NA
Sapur.014G079900.1	13.64725356	2.771668335	0.174479917	15.88531	8.01E-57	4.91E-56	cyan	0.671916673	5.80E-22		AT4G27745.1		Yippee family putative zinc-binding protein	Fad NAD-binding oxidoreductase, putative
Sapur.008G093600.1	455.5961915	-1.654171362	0.104142323	-15.8838	8.21E-57	5.03E-56	cyan	-0.725413841	6.31E-27	GO:0050662,GO:0003824	AT1G75290.1		NAD(P)-binding Rossmann-fold superfamily protein	NA
Sapur.002G096200.1	198.6887775	1.762172918	0.110952688	15.8822	8.42E-57	5.16E-56	cyan	0.760238419	7.72E-31		AT1G27730.1	STZ,ZAT10	salt tolerance zinc finger	Cys2-His2 type zinc finger protein, putative
Sapur.016G148000.1	211.6565328	-0.895449157	0.056380731	-15.8822	8.42E-57	5.16E-56	cyan	-0.791288705	6.18E-35	GO:0016740,GO:0008033	AT1G51310.1		transferases;tRNA (5-methylaminomethyl-2-thiouridylate)-methyltransferases	tRNA (5-methylaminomethyl-2-thiouridylate)-methyltransferase
Sapur.019G066200.1	1551.755897	0.33375906	0.021022178	15.87652	9.21E-57	5.64E-56	cyan	0.753696818	4.70E-30	GO:0055114,GO:0016491,GO:0008270	AT3G56460.1		GroES-like zinc-binding alcohol dehydrogenase family protein	zinc-binding dehydrogenase family oxidoreductase
Sapur.004G163900.1	964.1244771	0.604149207	0.038054546	15.87587	9.31E-57	5.70E-56	cyan	0.74335741	7.30E-29	GO:0055114,GO:0051537,GO:0016491,GO:0010277	AT3G44880.1	ACD1,LLS1,PAO	Pheophorbide a oxygenase family protein with Rieske [2Fe-2S] domain	pheophorbide A oxygenase
Sapur.012G056700.1	141.417072	-1.348687757	0.085050183	-15.8576	1.25E-56	7.62E-56	cyan	-0.778356261	3.78E-33		AT5G50090.2			NA
Sapur.T045800.1	106.1371616	-0.712138679	0.044911127	-15.8566	1.27E-56	7.73E-56	cyan	-0.79376403	2.72E-35	GO:0005634	AT3G25430.1		Polynucleotidyl transferase, ribonuclease H-like superfamily protein	poly(A)-specific ribonuclease PARN protein
Sapur.010G125600.1	197.4746074	-1.108238561	0.069906196	-15.8532	1.34E-56	8.16E-56	cyan	-0.832903516	1.14E-41	GO:0051301,GO:0032955	AT1G69390.1	ARC12,ATMINE1,MINE1	homologue of bacterial MinE 1	cell division topological specificity factor MinE
Sapur.T127700.1	6.018183259	2.199880196	0.138768214	15.85291	1.34E-56	8.20E-56	cyan	0.788888566	1.36E-34					NA
Sapur.T154200.1	6.617725609	3.366815164	0.212551432	15.84	1.65E-56	1.01E-55	cyan	0.717489056	4.05E-26					NA
Sapur.012G043200.1	17.20331863	-1.808296856	0.114193628	-15.8354	1.77E-56	1.08E-55	cyan	-0.751286068	9.02E-30		AT5G11040.1	AtTRS120,TRS120	TRS120	NA
Sapur.003G092300.1	125.1033809	-1.282467194	0.081022718	-15.8285	1.98E-56	1.21E-55	cyan	-0.742866112	8.28E-29	GO:0055114,GO:0016491	AT4G23340.1		2-oxoglutarate (2OG) and Fe(II)-dependent oxygenase superfamily protein	2OG-Fe(II) oxygenase family oxidoreductase
Sapur.016G046000.1	753.4159066	0.792799137	0.050088152	15.82808	1.99E-56	1.22E-55	cyan	0.769731376	5.06E-32		AT2G42310.1			ESSS subunit of NADH:ubiquinone oxidoreductase (complex I) protein
Sapur.15WG000200.1	530.2685389	0.661379659	0.041791812	15.82558	2.07E-56	1.26E-55	cyan	0.789620914	1.07E-34		AT3G49590.2		Autophagy-related protein 13	autophagy-related protein
Sapur.004G067400.1	70.82484599	-1.417635916	0.089586695	-15.8242	2.12E-56	1.29E-55	cyan	-0.774911932	1.08E-32					transmembrane protein, putative
Sapur.009G063300.1	264.4242307	0.90453293	0.057162954	15.82376	2.13E-56	1.30E-55	cyan	0.752279731	6.90E-30	GO:0055114,GO:0050661,GO:0033014,GO:0008883	AT1G58290.1	HEMA1	Glutamyl-tRNA reductase family protein	glutamyl-tRNA reductase

Sapur.014G077800.1	621.8701992	0.85427527	0.054010949	15.81671	2.39E-56	1.45E-55	cyan	0.742404932	9.33E-29		AT3G61800.1			UV-stimulated scaffold A-like protein, putative
Sapur.010G073900.1	263.430946	-0.649760472	0.041088249	-15.8138	2.50E-56	1.52E-55	cyan	-0.796865471	9.58E-36	GO:0003723	AT1G09290.1			THUMP domain protein
Sapur.001G030000.1	82.2133798	-0.591066713	0.037402042	-15.8031	2.96E-56	1.80E-55	cyan	-0.800201287	3.05E-36	GO:0070461	AT2G24530.1			transcriptional regulator of RNA polII, SAGA, subunit
Sapur.010G098200.1	26.35908294	2.353947352	0.148998426	15.79847	3.19E-56	1.94E-55	cyan	0.826036552	1.95E-40		AT1G68610.1	PCR11	PLANT CADMIUM RESISTANCE 11	PLAC8 family protein
Sapur.006G002300.1	255.0154064	0.810403366	0.051310874	15.79399	3.42E-56	2.08E-55	cyan	0.780896237	1.72E-33	GO:0005515	AT1G03370.1		C2 calcium/lipid-binding and GRAM domain containing protein	C2 and GRAM domain plant protein
Sapur.008G040000.1	187.4069462	0.994156795	0.062953922	15.79182	3.54E-56	2.15E-55	cyan	0.724998708	6.96E-27	GO:0005622,GO:0003779,GO:0030042,GO:0015629	AT5G59890.1	ADF4,ATADF4	actin depolymerizing factor 4	actin-depolymerizing factor
Sapur.001G060300.1	1478.413845	0.662046098	0.04192869	15.78981	3.66E-56	2.22E-55	cyan	0.771885197	2.67E-32		AT4G20150.1			NA
Sapur.T045000.1	172.9032291	1.664398648	0.105433092	15.7863	3.87E-56	2.35E-55	cyan	0.701716113	1.36E-24		AT3G59500.1		Integral membrane HRF1 family protein	integral membrane HRF1-like protein
Sapur.T146100.1	7.108425937	3.367332447	0.21341137	15.7786	4.37E-56	2.65E-55	cyan	0.708208246	3.30E-25					NA
Sapur.007G119500.1	82.33466736	1.172147346	0.074297986	15.7763	4.53E-56	2.74E-55	cyan	0.726902339	4.42E-27		AT3G07565.1		Protein of unknown function (DUF3755)	Myb-like DNA-binding domain protein, putative
Sapur.010G093700.1	182.8082435	0.686928019	0.04354304	15.77584	4.56E-56	2.76E-55	cyan	0.738619566	2.45E-28		AT1G25370.1		Protein of unknown function (DUF1639)	plant/T22E19-3 protein, putative
Sapur.003G047100.2	2.24988716	4.476328776	0.284002117	15.7616	5.72E-56	3.46E-55	cyan	0.517151979	4.08E-12		AT4G16745.1		Exostosin family protein	exostosin family protein
Sapur.006G195600.1	444.4855786	1.461671155	0.092789973	15.75247	6.61E-56	3.99E-55	cyan	0.707546891	3.82E-25		AT4G32870.1		Polyketide cyclase/dehydrase and lipid transport superfamily protein	NA
Sapur.009G094000.1	52.13376182	1.36658259	0.086760019	15.75129	6.73E-56	4.06E-55	cyan	0.752544984	6.42E-30		AT4G34560.1			transmembrane protein, putative
Sapur.002G057800.1	132.0932456	-1.578575289	0.100237095	-15.7484	7.04E-56	4.25E-55	cyan	-0.761465329	5.47E-31	GO:0009055	AT2G02850.1	ARPN	plantacyanin	basic blue protein
Sapur.014G126200.1	140.3004712	-1.00221671	0.063645331	-15.7469	7.21E-56	4.35E-55	cyan	-0.784054897	6.39E-34	GO:0006468,GO:0005524,GO:0004672	AT1G05100.1	MAPKKK18	mitogen-activated protein kinase kinase kinase 18	MAP kinase kinase kinase, putative
Sapur.003G079400.1	109.3378088	-0.583621201	0.037071168	-15.7433	7.64E-56	4.61E-55	cyan	-0.781780399	1.31E-33		AT4G12240.1		zinc finger (C2H2 type) family protein	zinc finger protein
Sapur.001G056700.1	1991.083899	0.646809747	0.041086725	15.74255	7.73E-56	4.66E-55	cyan	0.767996683	8.40E-32	GO:0016149,GO:0006415,GO:0005737	AT3G26618.1	ERF1-3	eukaryotic release factor 1-3	eukaryotic peptide chain release factor subunit 1-3
Sapur.005G152100.1	60.51404776	1.258378431	0.079982417	15.73319	8.96E-56	5.40E-55	cyan	0.720130973	2.19E-26	GO:0043565,GO:0006355,GO:0003700	AT5G44080.1		Basic-leucine zipper (bZIP) transcription factor family protein	transcription factor, putative
Sapur.T025500.1	1333.457272	0.458460686	0.029142903	15.73147	9.20E-56	5.55E-55	cyan	0.778208972	3.96E-33		AT1G79230.1	ATMST1.ATRDH1.MST1.ST1	mercaptopyruvate sulfurtransferase 1	thiosulfate/3-mercaptopyruvate sulfurtransferase
Sapur.003G109200.1	132.1364352	2.122675248	0.13494945	15.72941	9.51E-56	5.73E-55	cyan	0.646014124	6.49E-20	GO:0046983	AT1G32640.1	ATMYC2.JAI1.JIN1.MYC2.RD	Basic helix-loop-helix (bHLH) DNA-binding family protein	bHLH-MYC/R2R3-MYB transcription factor amino-terminal protein
Sapur.014G144400.1	2676.469918	-0.909889548	0.057852835	-15.7277	9.78E-56	5.89E-55	cyan	-0.774166797	1.35E-32	GO:0015979,GO:0009538,GO:0009522	AT4G12800.1	PSAL	photosystem I subunit l	photosystem I reaction center subunit XI
Sapur.019G010500.1	695.1775883	-1.004654784	0.063881746	-15.7268	9.91E-56	5.97E-55	cyan	-0.699188805	2.35E-24	GO:0007165,GO:0005515,GO:0043531	AT5G36930.2		Disease resistance protein (TIR-NBS-LRR class) family	NA
Sapur.010G205800.1	724.0766122	0.395988617	0.025179971	15.72633	9.98E-56	6.01E-55	cyan	0.782909547	9.18E-34	GO:0016787	AT1G50370.1		Calcineurin-like metallo-phosphoesterase superfamily protein	phytochrome-associated serine/threonine-protein phosphatase
Sapur.017G071500.1	186.2937029	0.760717435	0.048435502	15.70578	1.38E-55	8.30E-55	cyan	0.725418352	6.30E-27		AT5G15330.1	ATSPX4,SPX4	SPX domain gene 4	xenotropic and polytropic murine leukemia virus receptor ids-4, putative
Sapur.017G071000.1	95.62215784	-0.6191996	0.039437611	-15.7007	1.49E-55	8.99E-55	cyan	-0.791435596	5.89E-35		AT5G15280.1		Pentatricopeptide repeat (PPR) superfamily protein	PPR containing plant protein
Sapur.002G047500.1	27.19894419	-1.213705009	0.077302591	-15.7007	1.50E-55	8.99E-55	cyan	-0.794022719	2.50E-35		AT2G30933.1		Carbohydrate-binding X8 domain superfamily protein	NA
Sapur.016G176800.1	18.16723901	-1.476895392	0.094091877	-15.6963	1.60E-55	9.63E-55	cyan	-0.778828398	3.27E-33	GO:0016021	AT4G33905.1		Peroxisomal membrane 22 kDa (Mpv17/PMP22) family protein	NA
Sapur.017G064800.1	10.19337452	-1.711094034	0.109033117	-15.6933	1.68E-55	1.01E-54	cyan	-0.786499801	2.93E-34	GO:0055085,GO:0005741	AT3G01280.1	ATVDAC1,VDAC1	voltage dependent anion channel 1	eukaryotic porin protein
Sapur.016G225300.1	407.5553814	-0.49059907	0.031272326	-15.688	1.83E-55	1.10E-54	cyan	-0.788463138	1.56E-34	GO:0055085,GO:0046873,GO:0030001,GO:0016020	AT1G29820.2		Magnesium transporter CorA-like family protein	magnesium transporter CorA family protein
Sapur.011G067700.2	22.06010331	1.456764089	0.092927073	15.67642	2.19E-55	1.32E-54	cyan	0.752532701	6.44E-30	GO:0005515,GO:0005096	AT4G21160.3	AGD12,ZAC	Calcium-dependent ARF-type GTPase activating protein family	ADP-ribosylation factor GTPase-activating protein AGD12
Sapur.006G048600.1	954.133865	0.468704308	0.029903313	15.67399	2.28E-55	1.37E-54	cyan	0.798032028	6.44E-36		AT5G35080.1			glucosidase II beta subunit-like protein
Sapur.T120700.1	6.119693054	2.144178405	0.136806589	15.67306	2.31E-55	1.39E-54	cyan	0.804433069	6.93E-37	GO:0005515	AT4G26700.2	ATFIM1,FIM1	fimbrin 1	NA
Sapur.006G178400.1	115.8765074	-0.811972844	0.051840332	-15.663	2.71E-55	1.62E-54	cyan	-0.760374481	7.43E-31	GO:0005515	AT4G33160.1		F-box family protein	F-box only protein
Sapur.15WG114700.1	69.60313316	-0.707535361	0.045180756	-15.6601	2.83E-55	1.70E-54	cyan	-0.792367629	4.33E-35		AT5G63480.1			mediator of RNA polymerase II transcription subunit 30, putative
Sapur.014G091700.1	471.836602	0.642178717	0.0410078	15.65992	2.84E-55	1.70E-54	cyan	0.805125743	5.42E-37		AT3G62240.1		RING/U-box superfamily protein	RING Zn-finger protein
Sapur.016G280900.1	1764.6641	1.335935208	0.08532678	15.65669	2.99E-55	1.79E-54	cyan	0.625601086	1.96E-18	GO:0016787	AT3G20500.1	ATPAP18,PAP18	purple acid phosphatase 18	purple acid phosphatase
Sapur.016G025400.1	1111.582344	-0.878834363	0.056134418	-15.6559	3.03E-55	1.81E-54	cyan	-0.79432657	2.25E-35		AT3G56910.1	PSRP5	plastid-specific 50S ribosomal protein 5	hypothetical protein
Sapur.008G041100.1	421.9609401	0.993769346	0.063484988	15.65361	3.14E-55	1.88E-54	cyan	0.769127103	6.04E-32		AT3G12587.1		Oligosaccaryltransferase	dolichyl-diphosphooligosaccharide-protein glycosyltransferase subunit 4B
Sapur.013G037200.1	34.59198243	2.16769873	0.138508883	15.65025	3.31E-55	1.98E-54	cyan	0.686163746	3.51E-23		AT4G14746.1			transmembrane protein, putative
Sapur.008G093100.1	605.4463539	0.524750086	0.033533057	15.64874	3.39E-55	2.02E-54	cyan	0.752927319	5.79E-30		AT1G25682.1		Family of unknown function (DUF572)	coiled-coil protein
Sapur.008G048500.1	50.49588185	-1.241184859	0.079318719	-15.6481	3.42E-55	2.05E-54	cyan	-0.816478474	8.30E-39	GO:0006355,GO:0003677	AT5G02030.1	BLH9.BLR.HB-6.LSN.PNY.RP	POX (plant homeobox) family protein	BEL1 homeotic protein, putative
Sapur.004G149900.1	1553.768597	-1.194297684	0.076325255	-15.6475	3.46E-55	2.06E-54	cyan	-0.746958543	2.85E-29		AT1G08380.1	PSAO	photosystem I subunit O	NA
Sapur.002G022400.1	515.672017	-0.703986042	0.045009555	-15.6408	3.84E-55	2.29E-54	cyan	-0.787482023	2.14E-34	GO:0006508,GO:0004176	AT1G75460.1		ATP-dependent protease La (LON) domain protein	ATP-dependent protease LA domain protein
Sapur.002G067600.2	9.774510031	-1.998571295	0.127817067	-15.6362	4.13E-55	2.46E-54	cyan	-0.801365327	2.04E-36		AT1G21880.2	LYM1	lysm domain GPI-anchored protein 1 precursor	LysM domain GPI-anchored protein
Sapur.006G146500.1	34.23612838	1.541906079	0.098624836	15.63405	4.27E-55	2.54E-54	cyan	0.729466858	2.38E-27		AT5G18260.1		RING/U-box superfamily protein	hypothetical protein
Sapur.T045200.1	108.0562581	-1.300020267	0.08320512	-15.6243	4.97E-55	2.96E-54	cyan	-0.783572948	7.45E-34		AT3G07510.1			transmembrane protein, putative
Sapur.016G043200.1	62.7598354	-1.357715867	0.086907461	-15.6225	5.11E-55	3.05E-54	cyan	-0.764153825	2.55E-31	GO:0006355,GO:0005739,GO:0003690	AT5G06810.1		Mitochondrial transcription termination factor family protein	transcription termination factor family protein, putative
Sapur.005G130600.1	67.23076824	-1.734392221	0.111033894	-15.6204	5.29E-55	3.15E-54	cyan	-0.75042527	1.14E-29		AT1G70600.1		Ribosomal protein L18e/L15 superfamily protein	NA
Sapur.003G064400.1	667.7757773	0.445246637	0.028506531	15.61911	5.40E-55	3.21E-54	cyan	0.776451614	6.77E-33	GO:0005524	AT1G31910.1		GHMP kinase family protein	NA
Sapur.016G093500.1	20.78216734	-1.291744073	0.082748158	-15.6105	6.17E-55	3.67E-54	cyan	-0.76231221	4.30E-31	GO:0006468,GO:0004672	AT3G54030.1		Protein kinase protein with tetratricopeptide repeat domain	receptor kinase, putative
Sapur.010G137200.1	683.0730842	-0.613644234	0.039310428	-15.6102	6.20E-55	3.69E-54	cyan	-0.794178001	2.37E-35	GO:0016887,GO:0005524,GO:0016021,GO:0006810,GO:0005215	AT1G54350.1		ABC transporter family protein	ABC transporter family D protein, SmABCD protein, putative
Sapur.005G030300.1	49.03413632	1.181691234	0.075711234	15.60789	6.43E-55	3.83E-54	cyan	0.733783433	8.24E-28					hypothetical protein
Sapur.013G094000.1	178.1459242	-1.473320734	0.094412735	-15.6051	6.72E-55	4.00E-54	cyan	-0.722588068	1.23E-26	GO:0016597,GO:0008152,GO:0055114,GO:0050661,GO:0016491,GO:0006520	AT4G19710.2	AK-HSDH,AK-HSDH II	aspartate kinase-homoserine dehydrogenase ii	aspartokinase/homoserine dehydrogenase
Sapur.003G094000.1	70.81271709	-1.549387143	0.099308294	-15.6018	7.08E-55	4.21E-54	cyan	-0.788920443	1.34E-34	GO:0005515	AT1G75060.1			histone deacetylase complex subunit SAP30 Sin3-binding protein
Sapur.014G113400.1	263.7533729	-1.199933814	0.076911327	-15.6015	7.11E-55	4.22E-54	cyan	-0.776329989	7.03E-33		AT1G52140.1			disease resistance protein
Sapur.014G136400.1	244.4659045	-1.079462855	0.069207171	-15.5976	7.56E-55	4.49E-54	cyan	-0.820101296	2.06E-39		AT2G05310.1			transmembrane protein, putative
Sapur.004G128000.1	112.8357034	-0.916984578	0.058866988	-15.5772	1.04E-54	6.17E-54	cyan	-0.800344829	2.90E-36	GO:0016884	AT4G34880.1		Amidase family protein	amidase
Sapur.012G040200.1	788.2988457	0.615422501	0.039523071	15.57122	1.14E-54	6.77E-54	cyan	0.741834581	1.08E-28	GO:0016021,GO:0006810,GO:0005337	AT1G70330.1	ENT1,ENT1AT	equilibrative nucleotide transporter 1	equilibrative nucleoside transporter
Sapur.003G143700.1	9.190998087	-1.854084537	0.119081489	-15.5699	1.17E-54	6.92E-54	cyan	-0.775825757	8.19E-33		AT5G07010.1	ATST2A,ST2A	sulfotransferase 2A	flavonol 4'-sulfotransferase, putative
Sapur.005G046200.1	91.51594325	-0.826556145	0.053110247	-15.563	1.30E-54	7.69E-54	cyan	-0.776831166	6.03E-33	GO:0050660,GO:0008033,GO:0055114,GO:0016491	AT5G67290.1		FAD-dependent oxidoreductase family protein	FAD-dependent oxidoreductase
Sapur.15WG084700.1	57.99160598	-1.247867769	0.080193765	-15.5607	1.35E-54	7.98E-54	cyan	-0.737014283	3.68E-28		AT5G62720.1		Integral membrane HPP family protein	integral membrane HPP family protein
Sapur.007G109800.1	26.00240309	6.701840355	0.430728998	15.5593	1.38E-54	8.14E-54	cyan	0.586569011	6.91E-16	GO:0019752,GO:0004451	AT3G21720.1	ICL	isocitrate lyase	isocitrate lyase
Sapur.T157200.1	2.859983263	4.671122815	0.300345403	15.5525	1.53E-54	9.05E-54	cyan	0.652161517	2.21E-20		AT1G43760.1		DNAse I-like superfamily protein	NA
Sapur.017G039900.1	557.0289448	1.268764945	0.08160683	15.54729	1.66E-54	9.81E-54	cyan	0.736025627	4.72E-28	GO:0016491,GO:0055114,GO:0018580	AT5G64250.2		Aldolase-type TIM barrel family protein	2-nitropropane dioxygenase-like protein
Sapur.004G064500.1	85.60916575	-0.748296692	0.048133454	-15.5464	1.68E-54	9.94E-54	cyan	-0.774062458	1.40E-32		AT5G28040.1		DNA-binding storekeeper protein-related transcriptional regulator	DUF573 family protein
Sapur.005G044200.1	656.8319526	-0.799285837	0.051416899	-15.5452	1.71E-54	1.01E-53	cyan	-0.828881325	6.11E-41	GO:0009041,GO:0006221	AT3G18680.1		Amino acid kinase family protein	uridylate kinase
Sapur.010G128500.1	166.9996693	-1.71315128	0.110211778	-15.5442	1.74E-54	1.03E-53	cyan	-0.762333913	4.28E-31	GO:0019898,GO:0015979,GO:0009654,GO:0009523,GO:0005509	AT1G14150.1	PQL1,PQL2	PsbQ-like 2	oxygen evolving enhancer protein, putative
Sapur.T019000.1	18.25066302	2.037709879	0.131212848	15.5298	2.18E-54	1.29E-53	cyan	0.701226786	1.52E-24		AT5G16890.1		Exostosin family protein	NA
Sapur.004G135400.1	554.4661965	0.70693883	0.045527828	15.52762	2.26E-54	1.33E-53	cyan	0.750115483	1.23E-29	GO:0005515	AT2G17030.1		F-box family protein with a domain of unknown function (DUF295)	F-box protein
Sapur.003G102200.1	78.83817877	-1.996657894	0.12870543	-15.5134	2.82E-54	1.66E-53	cyan	-0.789736542	1.03E-34		AT2G19170.1	SLP3	subtilisin-like serine protease 3	NA
Sapur.018G076500.1	513.8278877	0.796223381	0.051333071	15.51092	2.93E-54	1.72E-53	cyan	0.758347693	1.31E-30		AT2G23890.1		HAD-superfamily hydrolase, subfamily IG, 5\'-nucleotidase	cytosolic purine 5-nucleotidase, putative
Sapur.014G088100.1	656.1309263	-0.800713162	0.051673869	-15.4955	3.72E-54	2.19E-53	cyan	-0.800963118	2.34E-36	GO:0006412,GO:0005840,GO:0005622,GO:0003735,GO:0005761	AT1G07830.1		ribosomal protein L29 family protein	39S ribosomal protein L47
Sapur.019G008400.1	1140.547339	-1.721751571	0.111115721	-15.4951	3.74E-54	2.20E-53	cyan	-0.742868023	8.28E-29		AT2G02130.1	LCR68,PDF2.3	low-molecular-weight cysteine-rich 68	gamma-thionin family protein
Sapur.016G007200.1	14.17736534	-2.310443024	0.149141333	-15.4916	3.95E-54	2.32E-53	cyan	-0.801065404	2.26E-36		AT4G17240.1			NA
Sapur.004G044000.1	352.9077288	-0.976541417	0.06310226	-15.4755	5.07E-54	2.98E-53	cyan	-0.750116548	1.23E-29	GO:0006468,GO:0004672	AT1G29750.2	RKF1	receptor-like kinase in flowers 1	receptor-like kinase, putative
Sapur.018G086200.1	19.91311921	-1.618463882	0.104622683	-15.4695	5.57E-54	3.27E-53	cyan	-0.795423719	1.56E-35	GO:0006468,GO:0004672,GO:0005975,GO:0004553	AT3G16030.1	CES101	lectin protein kinase family protein	NA
Sapur.019G026700.1	294.0056686	0.789689547	0.051079713	15.45994	6.47E-54	3.79E-53	cyan	0.748731253	1.78E-29		AT3G04530.1	ATPPCK2,PEPCK2,PPCK2	phosphoenolpyruvate carboxylase kinase 2	phosphoenolpyruvate carboxylase kinase
Sapur.005G114700.1	549.5479078	-0.791268652	0.051202376	-15.4537	7.12E-54	4.17E-53	cyan	-0.793047875	3.45E-35	GO:0003676	AT4G09040.1		RNA-binding (RRM/RBD/RNP motifs) family protein	RNA recognition motif (RRM) superfamily protein
Sapur.014G072600.1	2883.307458	-0.657808324	0.042585811	-15.4467	7.95E-54	4.65E-53	cyan	-0.753092881	5.54E-30		AT4G01150.1			cyanobacterial aminoacyl-tRNA synthetase CAAD domain protein
Sapur.005G037800.1	325.7157963	-0.7449592	0.048240658	-15.4426	8.47E-54	4.95E-53	cyan	-0.768397023	7.48E-32	GO:0005506	AT1G54500.1		Rubredoxin-like superfamily protein	rubredoxin
Sapur.001G027200.1	65.67295145	-1.109792652	0.071868811	-15.4419	8.55E-54	5.00E-53	cyan	-0.752773358	6.04E-30	GO:0016829,GO:0008152				KDPG and KHG aldolase
Sapur.008G000600.1	2866.88238	-0.945732421	0.061245813	-15.4416	8.60E-54	5.03E-53	cyan	-0.82754603	1.06E-40	GO:0016740	AT4G37930.1	SHM1,SHMT1,STM	serine transhydroxymethyltransferase 1	serine hydroxymethyltransferase
Sapur.003G048500.1	1014.328525	0.62769013	0.040652547	15.44036	8.76E-54	5.12E-53	cyan	0.753880097	4.47E-30	GO:0008138,GO:0006470	AT2G35680.1		Phosphotyrosine protein phosphatases superfamily protein	tyrosine phosphatase, putative
Sapur.006G174500.1	1042.893505	0.511082203	0.033104013	15.43868	8.99E-54	5.26E-53	cyan	0.781323729	1.51E-33	GO:0006468,GO:0004672	AT3G53570.1	AFC1,AME2,FC1	FUS3-complementing gene 1	Serine/Threonine kinase domain protein
Sapur.005G040300.1	479.6980642	-0.855514079	0.05542304	-15.4361	9.36E-54	5.47E-53	cyan	-0.778901456	3.20E-33	GO:0009052,GO:0004751	AT3G04790.1		Ribose 5-phosphate isomerase, type A protein	ribose-5-phosphate isomerase
Sapur.009G113400.2	21.52719648	-1.557158013	0.100904704	-15.432	9.98E-54	5.83E-53	cyan	-0.73055025	1.83E-27		AT4G35250.1		NAD(P)-binding Rossmann-fold superfamily protein	3-beta hydroxysteroid dehydrogenase/isomerase family protein
Sapur.006G055500.1	49.55562192	-1.24512672	0.080747197	-15.4201	1.20E-53	7.00E-53	cyan	-0.761691442	5.13E-31		AT5G25360.1			DUF4050 family protein
Sapur.006G120700.1	273.7521771	-0.709847704	0.046038887	-15.4184	1.23E-53	7.17E-53	cyan	-0.774900742	1.08E-32	GO:0005515	AT4G29830.1	VIP3	Transducin/WD40 repeat-like superfamily protein	vernalization independence protein, putative
Sapur.002G197200.1	921.6198147	0.394807887	0.025627607	15.40557	1.50E-53	8.75E-53	cyan	0.761062154	6.13E-31		AT2G20230.1		Tetraspanin family protein	tetraspanin protein
Sapur.002G177900.1	250.1851861	0.795893803	0.05167303	15.4025	1.57E-53	9.18E-53	cyan	0.743084634	7.83E-29	GO:0016021,GO:0006810	AT3G59310.1		Eukaryotic protein of unknown function (DUF914)	solute carrier family 35 protein
Sapur.15WG102800.1	1341.014934	0.887317395	0.057620358	15.39937	1.65E-53	9.63E-53	cyan	0.762367013	4.24E-31	GO:0051539,GO:0009435,GO:0008987	AT5G50210.1	OLD5,QS,SUFE3	quinolinate synthase	quinolinate synthetase A protein
Sapur.012G101800.1	55.15499379	-1.434392346	0.093180712	-15.3937	1.81E-53	1.05E-52	cyan	-0.779212928	2.91E-33	GO:0055114,GO:0016491	AT5G51810.1	AT2353.ATGA20OX2.GA20C	gibberellin 20 oxidase 2	gibberellin 20-oxidase
Sapur.011G119600.1	1946.522708	0.778271915	0.050575577	15.3883	1.96E-53	1.14E-52	cyan	0.725029372	6.91E-27	GO:0003924,GO:0007017,GO:0005874	AT5G62690.1	TUB2	tubulin beta chain 2	tubulin beta chain
Sapur.013G046900.2	15.65608499	-1.73807738	0.112968761	-15.3855	2.05E-53	1.19E-52	cyan	-0.767938457	8.55E-32	GO:0016788	AT5G33370.2		GDSL-like Lipase/Acylhydrolase superfamily protein	NA
Sapur.004G144700.1	442.6684768	-0.699158276	0.045452536	-15.3822	2.16E-53	1.25E-52	cyan	-0.803896427	8.38E-37		AT5G55710.1			Ycf60-like protein
Sapur.014G035200.1	33.53635194	-0.982515356	0.063903309	-15.375	2.41E-53	1.40E-52	cyan	-0.78649878	2.93E-34		AT2G44880.1		Pentatricopeptide repeat (PPR-like) superfamily protein	PPR containing plant protein
Sapur.009G122000.1	92.50597357	-0.834004781	0.054258339	-15.371	2.56E-53	1.49E-52	cyan	-0.782139695	1.17E-33		AT3G44020.1		thylakoid lumenal P17.1 protein	hypothetical protein
Sapur.15WG143400.1	760.6740044	-0.665516369	0.043311958	-15.3656	2.78E-53	1.62E-52	cyan	-0.806182256	3.72E-37	GO:0005515,GO:0051087	AT5G07220.1	ATBAG3,BAG3	BCL-2-associated athanogene 3	BAG family molecular chaperone regulator
Sapur.006G030900.1	1417.663468	1.947055999	0.126724654	15.36446	2.83E-53	1.64E-52	cyan	0.554205224	5.08E-14	GO:0008168,GO:0008152	AT2G41380.1		S-adenosyl-L-methionine-dependent methyltransferases superfamily protein	S-adenosylmethionine-dependent methyltransferase, putative
Sapur.012G014100.1	251.9924723	-0.873406654	0.056850776	-15.3631	2.89E-53	1.68E-52	cyan	-0.785126085	4.55E-34		AT5G53400.1	BOB1	HSP20-like chaperones superfamily protein	nuclear migration protein nudc protein
Sapur.002G178400.1	175.2361987	0.973052126	0.063359524	15.35763	3.15E-53	1.83E-52	cyan	0.717493551	4.04E-26		AT2G43160.1		ENTH/VHS family protein	clathrin interactor epsin 2, putative
Sapur.016G032400.1	353.9889586	-0.528117392	0.034419838	-15.3434	3.92E-53	2.27E-52	cyan	-0.765337062	1.82E-31	GO:0043565,GO:0006355,GO:0003700,GO:0006351	AT3G12250.2	BZIP45,TGA6	TGACG motif-binding factor 6	TGA-type basic leucine zipper protein TGA2.2
Sapur.011G011600.1	262.9300502	0.923659331	0.06020761	15.34124	4.05E-53	2.35E-52	cyan	0.691454184	1.19E-23	GO:0055114,GO:0009916	AT4G22260.1	IM,IM1	Alternative oxidase family protein	ubiquinol oxidase
Sapur.009G007900.1	665.3579617	0.525660555	0.034281059	15.33385	4.54E-53	2.63E-52	cyan	0.788491562	1.54E-34		AT2G28390.1		SAND family protein	SAND family protein, putative
Sapur.004G027200.1	99.1337695	-0.682455013	0.044509218	-15.3329	4.61E-53	2.67E-52	cyan	-0.80472069	6.26E-37		AT3G47160.1		RING/U-box superfamily protein	RING zinc finger protein, putative
Sapur.010G028000.2	28.02073584	-2.081144604	0.135732514	-15.3327	4.62E-53	2.68E-52	cyan	-0.768058901	8.25E-32	GO:0005975,GO:0004650	AT1G60590.1		Pectin lyase-like superfamily protein	polygalacturonase
Sapur.019G032100.1	1660.977463	0.59668665	0.038919266	15.3314	4.72E-53	2.73E-52	cyan	0.802021802	1.62E-36		AT3G04240.1	SEC	Tetratricopeptide repeat (TPR)-like superfamily protein	UDP-N-acetylglucosamine-peptide N-acetylglucosaminyltransferase, putative
Sapur.007G130300.1	1467.528241	0.525385653	0.034272786	15.32953	4.85E-53	2.81E-52	cyan	0.77012556	4.50E-32		AT3G59910.1		Ankyrin repeat family protein	F-box/ankyrin repeat protein SKIP35
Sapur.018G046800.1	628.8323133	0.797001744	0.05199732	15.32775	4.99E-53	2.89E-52	cyan	0.731927434	1.30E-27	GO:0007034	AT2G06530.1	VPS2.1	SNF7 family protein	charged multivesicular body protein 2a
Sapur.018G054800.4	8.672764192	-1.856702126	0.121140165	-15.3269	5.06E-53	2.92E-52	cyan	-0.74472105	5.12E-29		AT3G24430.1	HCF101	ATP binding	nucleotide-binding (ISS) ATPase, putative
Sapur.018G052800.1	34.58923235	-1.122473462	0.073235854	-15.3268	5.06E-53	2.92E-52	cyan	-0.770490062	4.04E-32		AT1G08070.1	OTP82	Tetratricopeptide repeat (TPR)-like superfamily protein	organelle transcript processing protein, putative
Sapur.001G100700.1	1901.993982	0.724014707	0.047274576	15.3151	6.06E-53	3.50E-52	cyan	0.754411009	3.87E-30		AT5G47570.1			NADH dehydrogenase 1 beta subcomplex subunit 8, putative
Sapur.003G023500.1	13.58576623	-1.677177097	0.109524559	-15.3133	6.24E-53	3.60E-52	cyan	-0.702937743	1.05E-24	GO:0055114,GO:0016614,GO:0050660,GO:0010430	AT1G72970.1	EDA17,HTH	Glucose-methanol-choline (GMC) oxidoreductase family protein	glucose-methanol-choline (GMC) oxidoreductase, putative
Sapur.010G074000.1	72.27528476	0.729278521	0.047640183	15.30805	6.76E-53	3.90E-52	cyan	0.745520868	4.16E-29		AT1G14590.1		Nucleotide-diphospho-sugar transferase family protein	nucleotide-diphospho-sugar transferase
Sapur.006G161600.1	354.3071177	-0.662927739	0.043307279	-15.3075	6.81E-53	3.93E-52	cyan	-0.796923512	9.39E-36	GO:0016021,GO:0004659	AT3G11945.1	ATHST,HST,PDS2	homogentisate prenyltransferase	homogentisate phytyltransferase
Sapur.012G019600.1	234.798148	-0.549470914	0.035911231	-15.3008	7.55E-53	4.35E-52	cyan	-0.809506315	1.12E-37		AT5G53280.1	PDV1	plastid division1	plastid division protein PDV1, putative
Sapur.004G151600.1	77.49461666	-0.766522014	0.050121392	-15.2933	8.47E-53	4.88E-52	cyan	-0.789011414	1.30E-34	GO:0007165	AT5G22400.1		Rho GTPase activating protein with PAK-box/P21-Rho-binding domain	Rho GTPase-activating protein
Sapur.009G072900.1	5.100794616	-2.579254684	0.168687115	-15.2902	8.89E-53	5.12E-52	cyan	-0.750025881	1.26E-29		AT1G24625.1	ZFP7	zinc finger protein 7	zinc finger protein, putative
Sapur.009G050900.1	1063.375791	-0.851969448	0.055729666	-15.2875	9.26E-53	5.33E-52	cyan	-0.793642281	2.83E-35	GO:0003676	AT3G52150.1		RNA-binding (RRM/RBD/RNP motifs) family protein	30S ribosomal protein
Sapur.017G039100.1	98.27995261	-1.410115288	0.092241738	-15.2872	9.31E-53	5.36E-52	cyan	-0.757634343	1.60E-30	GO:0016021,GO:0004659	AT1G60600.2	ABC4	UbiA prenyltransferase family protein	NA
Sapur.15WG036800.1	30.79986776	-1.046241455	0.06845441	-15.2838	9.81E-53	5.64E-52	cyan	-0.735802682	4.99E-28	GO:0003676,GO:0017069,GO:0000398	AT2G30260.1	U2B\'\'	U2 small nuclear ribonucleoprotein B	NA
Sapur.018G075800.1	2331.213499	1.036525121	0.067833468	15.28044	1.03E-52	5.93E-52	cyan	0.742675302	8.70E-29	GO:0016021	AT2G23810.1	TET8	tetraspanin8	tetraspanin protein
Sapur.004G135900.1	1878.069467	1.698958896	0.111268007	15.26907	1.23E-52	7.06E-52	cyan	0.721737423	1.51E-26	GO:0055114,GO:0016491,GO:0005507	AT1G76160.1	sks5	SKU5 similar 5	multicopper oxidase
Sapur.002G117100.1	110.511795	-2.031728352	0.133063554	-15.2689	1.23E-52	7.08E-52	cyan	-0.742901908	8.21E-29		AT3G60550.1	CYCP3;2	cyclin p3;2	cyclin-U2-1 protein
Sapur.004G048900.1	239.658248	-0.777569912	0.050950071	-15.2614	1.38E-52	7.93E-52	cyan	-0.77103387	3.44E-32	GO:0016758,GO:0008152	AT3G16520.3	UGT88A1	UDP-glucosyl transferase 88A1	UDP-glycosyltransferase
Sapur.005G152400.1	282.8082622	-0.803656368	0.052679065	-15.2557	1.51E-52	8.65E-52	cyan	-0.751435511	8.66E-30		AT1G43580.1		Sphingomyelin synthetase family protein	plant/T10P12-6 protein
Sapur.017G038200.1	95.41928318	1.239671499	0.081270682	15.25361	1.56E-52	8.93E-52	cyan	0.739485811	1.97E-28	GO:0008270,GO:0005515	AT1G72220.1		RING/U-box superfamily protein	RING-H2 zinc finger protein
Sapur.008G121600.2	281.3989015	0.94697844	0.062083916	15.2532	1.57E-52	8.98E-52	cyan	0.787582419	2.07E-34	GO:0019901,GO:0006355,GO:0000079	AT4G19600.1	CYCT1;4	Cyclin family protein	cyclin T1
Sapur.003G030600.1	35.24767254	-1.568704197	0.102853075	-15.2519	1.60E-52	9.16E-52	cyan	-0.744840315	4.96E-29	GO:0016747	AT5G67150.1		HXXXD-type acyl-transferase family protein	anthranilate N-hydroxycinnamoyl/benzoyltransferase
Sapur.006G190400.1	160.6865983	-1.605588329	0.105274674	-15.2514	1.61E-52	9.23E-52	cyan	-0.8246912	3.35E-40	GO:0006355,GO:0003677	AT4G32980.1	ATH1	homeobox gene 1	BEL1-like homeodomain protein
Sapur.013G006300.1	74.58528111	-1.319886865	0.086562205	-15.2478	1.70E-52	9.74E-52	cyan	-0.77180629	2.74E-32		AT1G56570.1		Tetratricopeptide repeat (TPR)-like superfamily protein	PPR containing plant protein, putative
Sapur.004G106200.1	509.9805811	0.746664151	0.048974027	15.24613	1.75E-52	1.00E-51	cyan	0.74533375	4.36E-29		AT3G01050.1	MUB1	membrane-anchored ubiquitin-fold protein 1 precursor	membrane-anchored ubiquitin-fold protein
Sapur.006G104700.1	170.4712575	1.046269802	0.068629308	15.24523	1.77E-52	1.01E-51	cyan	0.730098224	2.04E-27	GO:0003677,GO:0009725,GO:0006355,GO:0005634	AT4G30080.1	ARF16	auxin response factor 16	auxin response factor
Sapur.011G033300.1	93.29085547	0.779228491	0.051123994	15.24193	1.86E-52	1.07E-51	cyan	0.758019159	1.43E-30		AT4G24370.1			NA
Sapur.004G071700.1	30.04167128	-1.998998207	0.131152709	-15.2418	1.87E-52	1.07E-51	cyan	-0.818500183	3.82E-39		AT3G29370.1			hypothetical protein
Sapur.018G082300.1	1676.213479	-0.692455338	0.045436501	-15.2401	1.92E-52	1.10E-51	cyan	-0.828711582	6.56E-41	GO:0045905,GO:0045901,GO:0043022,GO:0006452,GO:0003746,GO:0003723	AT1G13950.1	ATELF5A-1.EIF-5A.EIF5A.ELF	eukaryotic elongation factor 5A-1	eukaryotic translation initiation factor 5A
Sapur.001G100900.1	140.7844898	-1.907070159	0.125165425	-15.2364	2.03E-52	1.16E-51	cyan	-0.754555862	3.72E-30		AT4G16515.1	RGF6		hypothetical protein
Sapur.019G000300.1	1293.082052	-0.580508038	0.038103024	-15.2352	2.06E-52	1.18E-51	cyan	-0.806456753	3.37E-37	GO:0031047	AT5G23570.1	ATSGS3,SGS3	XS domain-containing protein / XS zinc finger domain-containing protein-related	suppressor OF protein silencing protein
Sapur.016G218300.1	11.60872965	-2.069788153	0.135876816	-15.2328	2.14E-52	1.22E-51	cyan	-0.777428646	5.03E-33	GO:0005515	AT3G27640.1		Transducin/WD40 repeat-like superfamily protein	cell division cycle protein cdt2, putative
Sapur.008G020200.1	632.5167067	0.598010206	0.039274196	15.22654	2.36E-52	1.35E-51	cyan	0.735458908	5.43E-28	GO:0005515	AT3G52190.1	PHF1	phosphate transporter traffic facilitator1	NA
Sapur.010G027800.1	6.793730255	-2.740445109	0.180042063	-15.2211	2.56E-52	1.46E-51	cyan	-0.80873543	1.48E-37		AT1G10657.2		Plant protein 1589 of unknown function	plant-specific domain TIGR01589 family protein, putative
Sapur.018G073500.1	166.8736206	-0.833868325	0.054804345	-15.2154	2.80E-52	1.59E-51	cyan	-0.804618102	6.49E-37		AT3G46550.1	SOS5	Fasciclin-like arabinogalactan family protein	fasciclin-like arabinogalactan protein
Sapur.005G202200.1	410.5620292	0.728871036	0.0479064	15.21448	2.83E-52	1.62E-51	cyan	0.771935802	2.64E-32	GO:0005515	AT1G20670.1		DNA-binding bromodomain-containing protein	bromodomain domain protein
Sapur.001G099500.1	596.9167119	0.672301548	0.044198878	15.21083	3.00E-52	1.71E-51	cyan	0.777100108	5.56E-33	GO:0055114,GO:0016491	AT1G62620.1		Flavin-binding monooxygenase family protein	flavin containing monooxygenase FMO GS-OX-like protein
Sapur.009G072400.1	1894.779552	0.531282489	0.034929797	15.21001	3.04E-52	1.73E-51	cyan	0.76589112	1.55E-31		AT2G14910.1			seed maturation protein PM23, putative
Sapur.007G050600.1	1626.983449	0.379451604	0.024962081	15.20112	3.48E-52	1.98E-51	cyan	0.762374977	4.23E-31	GO:0006468,GO:0004672	AT2G23070.1		Protein kinase superfamily protein	NA
Sapur.001G045900.1	126.8216943	1.782941567	0.117310251	15.19851	3.62E-52	2.06E-51	cyan	0.729912476	2.13E-27		AT1G67330.1		Protein of unknown function (DUF579)	polysaccharide biosynthesis protein
Sapur.007G068600.1	381.6288103	0.574697343	0.037837427	15.18859	4.21E-52	2.39E-51	cyan	0.745188153	4.53E-29	GO:0008270,GO:0005515	AT3G19950.1		RING/U-box superfamily protein	zinc-RING finger protein, putative
Sapur.003G140400.1	515.7633192	0.632544206	0.041656201	15.18488	4.45E-52	2.53E-51	cyan	0.732426534	1.15E-27		AT1G80940.1			hypothetical protein
Sapur.011G008900.1	303.3583062	0.55014531	0.036233942	15.18315	4.57E-52	2.60E-51	cyan	0.782475854	1.05E-33		AT1G62330.1		O-fucosyltransferase family protein	GDP-fucose protein O-fucosyltransferase
Sapur.007G062900.1	278.2246766	1.411112562	0.092955888	15.18045	4.76E-52	2.70E-51	cyan	0.696596446	4.07E-24		AT3G19740.1		P-loop containing nucleoside triphosphate hydrolases superfamily protein	NA
Sapur.014G104200.2	90.64229127	1.208000445	0.079605159	15.1749	5.19E-52	2.94E-51	cyan	0.714231497	8.53E-26	GO:0046872	AT1G02860.1	BAH1,NLA	SPX (SYG1/Pho81/XPR1) domain-containing protein	E3 ubiquitin-protein ligase BAH1
Sapur.017G107500.1	183.5337971	-0.961994048	0.063413355	-15.1702	5.57E-52	3.16E-51	cyan	-0.812053693	4.37E-38	GO:0005975,GO:0004553	AT1G66250.1		O-Glycosyl hydrolases family 17 protein	glucan endo-1%2C3-beta-glucosidase
Sapur.002G082500.1	1030.371703	0.474404008	0.031274526	15.16902	5.67E-52	3.21E-51	cyan	0.765444156	1.76E-31		AT1G28200.1	FIP1	FH interacting protein 1	GLABRA 2 expression modulator, putative
Sapur.T162300.1	11.71485515	1.486689167	0.098013176	15.16826	5.74E-52	3.25E-51	cyan	0.750789953	1.03E-29		AT5G08110.1		nucleic acid binding;ATP-dependent helicases;ATP binding;helicases;ATP-dependent helicases	NA
Sapur.010G185100.1	289.8110285	1.413451762	0.093199931	15.1658	5.96E-52	3.37E-51	cyan	0.700045535	1.95E-24		AT5G04460.1		RING/U-box superfamily protein	neuralized protein
Sapur.004G020900.1	839.7918415	0.587875242	0.038766029	15.1647	6.06E-52	3.43E-51	cyan	0.761172028	5.94E-31		AT1G61350.1		ARM repeat superfamily protein	ubiquitin-protein ligase, putative
Sapur.016G219200.1	2650.932705	1.133531411	0.074754598	15.16337	6.18E-52	3.50E-51	cyan	0.743662494	6.74E-29		AT3G27810.1	ATMYB21,ATMYB3,MYB21	myb domain protein 21	MYB transcription factor
Sapur.016G295500.1	2.198643606	4.147921447	0.273554555	15.16305	6.21E-52	3.52E-51	cyan	0.693395612	7.96E-24	GO:0016787	AT5G63870.1	ATPP7,PP7	serine/threonine phosphatase 7	NA
Sapur.002G001000.9	5.985095403	-2.005681005	0.132276817	-15.1628	6.24E-52	3.53E-51	cyan	-0.591559393	3.41E-16		AT1G76620.1		Protein of unknown function, DUF547	electron transporter, putative
Sapur.002G091900.1	249.2116278	2.399599334	0.158267809	15.16164	6.35E-52	3.59E-51	cyan	0.69850113	2.72E-24	GO:0008152,GO:0003824	AT1G10070.2	ATBCAT-2,BCAT-2	branched-chain amino acid transaminase 2	branched-chain-amino-acid aminotransferase
Sapur.002G055700.1	107.3813864	1.034695675	0.068246497	15.16115	6.39E-52	3.62E-51	cyan	0.70699703	4.31E-25	GO:0005515,GO:0006468,GO:0005524,GO:0004672	AT3G24240.1		Leucine-rich repeat receptor-like protein kinase family protein	NA
Sapur.014G071600.2	59.23188339	-0.865601601	0.057110365	-15.1566	6.85E-52	3.87E-51	cyan	-0.768609768	7.03E-32		AT2G46225.1	ABIL1	ABI-1-like 1	ABIL1
Sapur.010G086700.1	15.16338239	-1.50325433	0.0992054	-15.1529	7.24E-52	4.09E-51	cyan	-0.734564021	6.79E-28					hypothetical protein
Sapur.018G007300.1	845.6989242	1.049182526	0.069245847	15.15156	7.40E-52	4.18E-51	cyan	0.728900802	2.73E-27		AT2G24580.1		FAD-dependent oxidoreductase family protein	sarcosine oxidase
Sapur.003G013600.1	1394.387739	0.705823769	0.046585592	15.15112	7.45E-52	4.21E-51	cyan	0.746546547	3.18E-29		AT5G64550.1		loricrin-related	loricrin-like protein
Sapur.012G086000.1	382.628472	0.908469082	0.059968839	15.14902	7.69E-52	4.34E-51	cyan	0.732725296	1.07E-27		AT2G44740.1	CYCP4;1	cyclin p4;1	cyclin-U4 protein
Sapur.007G055800.1	15.34063098	-1.215415122	0.080252147	-15.145	8.18E-52	4.62E-51	cyan	-0.774457508	1.24E-32		AT2G17510.1	EMB2763	ribonuclease II family protein	NA
Sapur.006G117800.1	730.4094813	0.814376307	0.053773862	15.14446	8.24E-52	4.65E-51	cyan	0.732817426	1.05E-27		AT5G35570.1		O-fucosyltransferase family protein	GDP-fucose O-fucosyltransferase-like protein
Sapur.019G051600.1	249.2840286	1.509188844	0.099685381	15.13952	8.89E-52	5.01E-51	cyan	0.800636759	2.62E-36	GO:0046872	AT3G02830.1	ZFN1	zinc finger protein 1	zinc finger CCCH domain protein
Sapur.011G003400.1	4.576438107	-2.593388335	0.171319581	-15.1377	9.13E-52	5.15E-51	cyan	-0.750221675	1.20E-29		AT1G62050.1		Ankyrin repeat family protein	GPCR-chaperone protein
Sapur.006G148800.1	27.25545512	1.497593975	0.098931858	15.13763	9.14E-52	5.16E-51	cyan	0.680411962	1.11E-22		AT3G23580.1	RNR2,RNR2A	ribonucleotide reductase 2A	NA
Sapur.003G015800.1	127.1930753	-0.945613395	0.062478308	-15.1351	9.51E-52	5.36E-51	cyan	-0.754415171	3.87E-30		AT3G59310.1		Eukaryotic protein of unknown function (DUF914)	solute carrier family 35 protein
Sapur.008G167500.1	100.2774476	-0.682319125	0.045088503	-15.1329	9.83E-52	5.54E-51	cyan	-0.782852318	9.35E-34		AT5G19151.1			NA
Sapur.005G198900.1	13.69366182	-2.490425577	0.164581971	-15.1318	9.99E-52	5.63E-51	cyan	-0.692767193	9.07E-24	GO:0008152,GO:0003824	AT1G20510.1	OPCL1	OPC-8:0 CoA ligase1	acyl-CoA synthetase
Sapur.007G020000.1	69.76776582	-1.062125053	0.070208017	-15.1283	1.05E-51	5.94E-51	cyan	-0.77511875	1.01E-32		AT3G50900.1			hypothetical protein
Sapur.003G012900.1	25.66794042	1.576833001	0.104264871	15.12334	1.14E-51	6.40E-51	cyan	0.705262568	6.31E-25		AT1G68140.1		Protein of unknown function (DUF1644)	DUF1644 family protein
Sapur.008G171600.1	152.6891412	-0.893002447	0.059052599	-15.1222	1.16E-51	6.51E-51	cyan	-0.78878417	1.40E-34	GO:0003824,GO:0006470,GO:0004722	AT3G06270.1		Protein phosphatase 2C family protein	protein phosphatase 2C and cyclic nucleotide-binding/kinase domain protein
Sapur.001G078900.1	68.96684006	-0.686271218	0.045399124	-15.1164	1.26E-51	7.10E-51	cyan	-0.751256718	9.09E-30		AT5G41620.1			hypothetical protein
Sapur.018G058000.1	487.763172	0.646422381	0.042767592	15.11477	1.29E-51	7.28E-51	cyan	0.744896558	4.89E-29		AT5G19540.1			hypothetical protein
Sapur.016G006100.1	736.8623491	1.153785421	0.076336382	15.11449	1.30E-51	7.31E-51	cyan	0.707579361	3.79E-25	GO:0046872,GO:0030001	AT4G16380.1		Heavy metal transport/detoxification superfamily protein	heavy metal-associated domain protein
Sapur.017G073000.1	660.826253	-0.89272386	0.059068567	-15.1133	1.32E-51	7.43E-51	cyan	-0.768529193	7.19E-32	GO:0009055	AT5G15350.1	AtENODL17,ENODL17	early nodulin-like protein 17	blue copper protein, putative
Sapur.018G064400.1	11.54554649	-1.584502391	0.104853894	-15.1115	1.36E-51	7.64E-51	cyan	-0.717885158	3.69E-26		AT1G60660.1	ATCB5LP,B5		cytochrome b5-like heme/steroid-binding domain protein
Sapur.002G168700.1	575.2923769	0.464028564	0.030709297	15.11036	1.38E-51	7.78E-51	cyan	0.767868751	8.72E-32	GO:0006468,GO:0004672	AT3G62220.1		Protein kinase superfamily protein	Pto kinase interactor 1
Sapur.010G105100.1	3.875786855	-2.628615379	0.174012165	-15.1059	1.48E-51	8.31E-51	cyan	-0.78317453	8.44E-34		AT5G38790.1			hypothetical protein
Sapur.004G131800.1	402.5646558	1.035546692	0.068561886	15.10382	1.53E-51	8.58E-51	cyan	0.707442709	3.91E-25	GO:0046872,GO:0030001	AT4G38580.1	ATFP6,FP6,HIPP26	farnesylated protein 6	heavy metal-associated domain protein
Sapur.010G068800.1	322.3905253	-1.365594809	0.090441464	-15.0992	1.64E-51	9.20E-51	cyan	-0.817842572	4.92E-39	GO:0006355,GO:0005634	AT4G14550.1	IAA14,SLR	indole-3-acetic acid inducible 14	auxin-responsive protein IAA
Sapur.006G202400.1	12.63097537	-1.425097331	0.094395745	-15.0971	1.69E-51	9.50E-51	cyan	-0.785348939	4.24E-34		AT3G15130.1		Tetratricopeptide repeat (TPR)-like superfamily protein	PPR containing plant protein, putative
Sapur.004G022000.1	213.488814	-0.626220818	0.041513661	-15.0847	2.04E-51	1.14E-50	cyan	-0.762020999	4.67E-31		AT1G11240.1			nucleolar protein 12, 25 kDa protein
Sapur.009G131700.1	872.8490681	-0.771225559	0.051166215	-15.0729	2.44E-51	1.37E-50	cyan	-0.777089079	5.58E-33	GO:0016020,GO:0004970	AT4G35290.1	ATGLR3.2.ATGLUR2.GLR3.2	glutamate receptor 2	glutamate receptor
Sapur.010G026400.1	22.07562384	2.514578695	0.166885777	15.06766	2.64E-51	1.48E-50	cyan	0.606100785	4.06E-17		AT1G17100.1		SOUL heme-binding family protein	SOUL heme-binding protein
Sapur.013G046900.1	988.5277558	-1.298562216	0.086211881	-15.0625	2.86E-51	1.60E-50	cyan	-0.782673278	9.89E-34	GO:0016788	AT3G04290.1	ATLTL1,LTL1	Li-tolerant lipase 1	NA
Sapur.018G043100.1	23.33055976	-1.030097787	0.068390817	-15.0619	2.88E-51	1.61E-50	cyan	-0.785960844	3.49E-34		AT2G37310.1		Pentatricopeptide repeat (PPR) superfamily protein	PPR containing plant protein
Sapur.008G007200.2	9.6484224	-1.790991328	0.118958699	-15.0556	3.17E-51	1.78E-50	cyan	-0.60037072	9.52E-17		AT5G06390.1	FLA17	FASCICLIN-like arabinogalactan protein 17 precursor	fasciclin-like protein
Sapur.002G142200.1	19.62009961	1.314132411	0.087321003	15.04944	3.48E-51	1.95E-50	cyan	0.746117086	3.56E-29	GO:0016021,GO:0005789	AT1G01230.1		ORMDL family protein	NA
Sapur.008G135600.1	56.35299392	0.870602999	0.057850682	15.04914	3.50E-51	1.96E-50	cyan	0.747793513	2.29E-29	GO:0005515	AT2G29420.1	ATGSTU7,GST25,GSTU7	glutathione S-transferase tau 7	glutathione S-transferase GST
Sapur.001G048900.2	8.479785675	-1.726495626	0.114729149	-15.0484	3.53E-51	1.98E-50	cyan	-0.747147916	2.71E-29	GO:0006457,GO:0003755,GO:0000413	AT5G13120.1	ATCYP20-2,CYP20-2	cyclophilin 20-2	peptidyl-prolyl cis-trans isomerase, cyclophilin-type protein
Sapur.004G003500.1	1668.488286	1.063584826	0.070691268	15.04549	3.70E-51	2.07E-50	cyan	0.737446859	3.30E-28	GO:0016020	AT4G22120.4		ERD (early-responsive to dehydration stress) family protein	early-responsive to dehydration-like protein
Sapur.009G083700.3	25.23900464	-1.282768402	0.085322805	-15.0343	4.38E-51	2.44E-50	cyan	-0.747699455	2.34E-29		AT4G37180.1		Homeodomain-like superfamily protein	myb-like DNA-binding domain protein, putative
Sapur.004G004800.1	59.58629326	-1.237356219	0.082370131	-15.0219	5.28E-51	2.95E-50	cyan	-0.812092596	4.31E-38	GO:0030247	AT1G11915.1			wall-associated receptor kinase galacturonan-binding protein
Sapur.005G194600.1	1063.369317	-1.42447106	0.094826767	-15.0218	5.28E-51	2.95E-50	cyan	-0.820646392	1.66E-39	GO:0016788	AT1G75900.1		GDSL-like Lipase/Acylhydrolase superfamily protein	GDSL esterase/lipase
Sapur.005G099800.1	431.6663809	0.463977527	0.030887189	15.02168	5.29E-51	2.96E-50	cyan	0.7573605	1.72E-30		AT5G65650.1		Protein of unknown function (DUF1195)	sugar transporter, putative
Sapur.010G095400.1	601.3095363	-0.674582113	0.04493074	-15.0138	5.96E-51	3.32E-50	cyan	-0.800038178	3.23E-36		AT1G13380.1		Protein of unknown function (DUF1218)	fiber protein Fb34, putative
Sapur.T119400.1	3.775563512	3.561011587	0.237183476	15.01374	5.97E-51	3.33E-50	cyan	0.746867499	2.92E-29	GO:0005681,GO:0000398,GO:0000387	AT1G54385.2		ARM repeat superfamily protein	NA
Sapur.007G043000.1	21.20369324	1.518704323	0.101260785	14.99795	7.57E-51	4.22E-50	cyan	0.721829878	1.47E-26	GO:0005515	AT5G67140.1		F-box/RNI-like superfamily protein	F-box plant protein
Sapur.010G067100.1	32.62305862	2.785136981	0.185741076	14.99473	7.95E-51	4.42E-50	cyan	0.722203964	1.35E-26		AT3G22970.1		Protein of unknown function (DUF506)	NA
Sapur.016G159200.1	818.2774899	0.791030376	0.052754983	14.99442	7.99E-51	4.44E-50	cyan	0.765867621	1.56E-31	GO:0005515	AT2G32560.1		F-box family protein	F-box plant protein
Sapur.005G116000.1	1998.28965	-0.786433095	0.052452468	-14.9933	8.13E-51	4.52E-50	cyan	-0.761190144	5.91E-31	GO:0009073,GO:0003849	AT1G22410.1		Class-II DAHP synthetase family protein	NA
Sapur.019G017500.1	26.31232056	1.406326323	0.093816413	14.9902	8.51E-51	4.73E-50	cyan	0.683174733	6.41E-23	GO:0006468,GO:0004672	AT3G14840.2		Leucine-rich repeat transmembrane protein kinase	NA
Sapur.001G129600.1	588.0005374	1.479686285	0.09871788	14.98904	8.66E-51	4.82E-50	cyan	0.656161317	1.08E-20	GO:0055114,GO:0003995,GO:0006635,GO:0005777,GO:0003997	AT4G16760.1	ACX1,ATACX1	acyl-CoA oxidase 1	acyl-coenzyme A oxidase
Sapur.016G094400.1	1944.073031	-0.785696528	0.052426752	-14.9866	8.99E-51	5.00E-50	cyan	-0.772547984	2.20E-32	GO:0042578,GO:0042132,GO:0005975	AT3G54050.2	HCEF1	high cyclic electron flow 1	fructose-1%2C6-bisphosphatase
Sapur.T020800.1	3.878348398	3.038676079	0.20281127	14.98278	9.52E-51	5.29E-50	cyan	0.803322545	1.03E-36		AT5G35700.1	FIM2	fimbrin-like protein 2	NA
Sapur.018G074100.1	84.09765287	1.110123181	0.074096222	14.98218	9.60E-51	5.33E-50	cyan	0.704330571	7.73E-25		AT4G16850.1			transmembrane protein, putative
Sapur.006G074200.1	805.4575431	0.992852138	0.06627323	14.98119	9.74E-51	5.41E-50	cyan	0.802114453	1.57E-36		AT2G37770.2		NAD(P)-linked oxidoreductase superfamily protein	aldo/keto reductase family protein
Sapur.008G159300.1	86.23685307	-0.86974515	0.058061845	-14.9796	9.98E-51	5.54E-50	cyan	-0.792289789	4.44E-35		AT1G09520.1			DNA-binding protein, putative
Sapur.004G097500.1	197.513295	0.811571153	0.054220937	14.96786	1.19E-50	6.61E-50	cyan	0.757822047	1.52E-30		AT1G67060.1			peptidase M50B-like protein
Sapur.013G131400.1	5.171371498	3.212718309	0.214692802	14.96426	1.26E-50	6.97E-50	cyan	0.726286109	5.12E-27	GO:0055114,GO:0016491	AT2G20800.1	NDB4	NAD(P)H dehydrogenase B4	external alternative NAD(P)H-ubiquinone oxidoreductase B4
Sapur.002G134900.1	2745.274967	0.767901537	0.051329141	14.96034	1.33E-50	7.39E-50	cyan	0.744588371	5.30E-29	GO:0008234,GO:0006508	AT4G01610.1		Cysteine proteinases superfamily protein	cathepsin B-like cysteine protease
Sapur.018G003200.1	205.6842042	-0.695838811	0.046513761	-14.9598	1.34E-50	7.44E-50	cyan	-0.801685887	1.82E-36		AT5G24830.1		Tetratricopeptide repeat (TPR)-like superfamily protein	glycosyl phosphatidyl inositol transamidase-like protein, putative
Sapur.005G172300.1	3755.119915	1.04539945	0.069880929	14.95972	1.35E-50	7.45E-50	cyan	0.744068762	6.07E-29	GO:0016747,GO:0008610,GO:0016020,GO:0006633	AT1G04220.1	KCS2	3-ketoacyl-CoA synthase 2	NA
Sapur.016G122100.1	11.66006077	2.482083705	0.16592666	14.95892	1.36E-50	7.54E-50	cyan	0.670604571	7.45E-22		AT4G12735.1			hypothetical protein
Sapur.018G046000.1	1962.00632	0.927792273	0.06208049	14.94499	1.68E-50	9.29E-50	cyan	0.725010994	6.94E-27	GO:0042578	AT3G43220.1		Phosphoinositide phosphatase family protein	phosphoinositide phosphatase
Sapur.013G123200.1	58.79123345	-1.46592535	0.098097608	-14.9435	1.72E-50	9.49E-50	cyan	-0.786089182	3.35E-34		AT2G20710.1		Tetratricopeptide repeat (TPR)-like superfamily protein	NA
Sapur.005G049100.1	4.416834184	3.624247323	0.242545433	14.94255	1.74E-50	9.63E-50	cyan	0.730660616	1.78E-27		AT1G21390.1	emb2170	embryo defective 2170	hypothetical protein
Sapur.016G088100.3	57.37746066	-0.820148404	0.054892544	-14.941	1.78E-50	9.85E-50	cyan	-0.766441693	1.32E-31		AT1G65260.1	PTAC4,VIPP1	plastid transcriptionally active 4	membrane-associated 30 kDa protein
Sapur.019G044500.1	1383.366986	-0.702563963	0.047043974	-14.9342	1.97E-50	1.09E-49	cyan	-0.763417682	3.14E-31		AT5G17170.1	ENH1	rubredoxin family protein	electron transporter, putative
Sapur.003G034400.1	522.2268997	1.057371298	0.070836141	14.927	2.20E-50	1.21E-49	cyan	0.747325584	2.59E-29		AT1G15385.1			DUF761 domain protein
Sapur.005G101300.1	386.0207406	0.648837601	0.04349614	14.91713	2.55E-50	1.41E-49	cyan	0.724430481	7.97E-27	GO:0016491	AT4G36750.1		Quinone reductase family protein	minor allergen Alt protein A, putative
Sapur.005G071300.1	57.11500705	-1.160850866	0.07782206	-14.9167	2.57E-50	1.41E-49	cyan	-0.731034095	1.62E-27		AT5G04190.1	PKS4	phytochrome kinase substrate 4	phytochrome kinase substrate-like protein, putative
Sapur.003G078500.1	1467.245781	-0.720079529	0.048282328	-14.9139	2.68E-50	1.47E-49	cyan	-0.770676516	3.83E-32	GO:0005515	AT4G12400.2		stress-inducible protein, putative	NA
Sapur.008G115900.1	5.090895625	5.155899836	0.345730266	14.91307	2.71E-50	1.49E-49	cyan	0.55182501	6.85E-14	GO:0005515	AT4G38870.1		F-box and associated interaction domains-containing protein	F-box protein interaction domain protein
Sapur.15WG117200.1	457.4115341	0.594028277	0.039845799	14.90818	2.92E-50	1.60E-49	cyan	0.775517346	9.00E-33		AT4G24970.1		Histidine kinase-, DNA gyrase B-, and HSP90-like ATPase family protein	NA
Sapur.010G045900.1	34.12949369	-1.066385508	0.071532104	-14.9078	2.93E-50	1.61E-49	cyan	-0.774678496	1.16E-32					NA
Sapur.017G043100.1	109.3665681	-0.931499559	0.062489571	-14.9065	2.99E-50	1.65E-49	cyan	-0.766197815	1.42E-31		AT4G13640.1	UNE16	Homeodomain-like superfamily protein	Myb transcription factor, putative
Sapur.017G092700.1	71.09936037	-1.373222438	0.092124407	-14.9062	3.00E-50	1.65E-49	cyan	-0.798546335	5.40E-36		AT1G13245.1	DVL4,RTFL17	ROTUNDIFOLIA like 17	DVL protein
Sapur.019G032400.1	178.4359031	-0.970217695	0.065090475	-14.9057	3.03E-50	1.67E-49	cyan	-0.75211028	7.22E-30		AT2G36290.1		alpha/beta-Hydrolases superfamily protein	NA

Sapur.011G064900.1	1267.161846	-0.597334428	0.040086444	-14.9012	3.24E-50	1.78E-49	cyan	-0.769049969	6.18E-32	GO:0005992,GO:0003824	AT1G78580.1	ATTPS1,TPS1	trehalose-6-phosphate synthase	alpha,alpha-trehalose-phosphate synthase [UDP-forming] protein
Sapur.010G088200.1	722.9489399	0.599414991	0.04023754	14.89691	3.45E-50	1.90E-49	cyan	0.748750356	1.77E-29	GO:0045454,GO:0055114,GO:0016972	AT2G01270.1	AtQSOX2,QSOX2	quiescin-sulfhydryl oxidase 2	protein disulfide-isomerase
Sapur.011G118400.1	13.33680604	1.978841984	0.1328776	14.89222	3.70E-50	2.03E-49	cyan	0.66587325	1.82E-21	GO:0055114,GO:0050660,GO:0016491	AT2G34790.1	EDA28,MEE23	FAD-binding Berberine family protein	reticuline oxidase, putative
Sapur.T143600.1	11.13481901	1.773630384	0.119122488	14.88913	3.88E-50	2.13E-49	cyan	0.75309523	5.53E-30		AT5G35700.1	FIM2	fimbrin-like protein 2	NA
Sapur.019G020400.1	96.39699491	-1.009545471	0.067819085	-14.8859	4.07E-50	2.24E-49	cyan	-0.773787414	1.52E-32		AT3G18800.1			transmembrane protein, putative
Sapur.008G045000.1	231.9548045	1.200855971	0.080700666	14.88037	4.42E-50	2.42E-49	cyan	0.714470215	8.08E-26		AT5G02220.1			hypothetical protein
Sapur.016G026500.1	40.55937574	-0.932139318	0.062646981	-14.8792	4.50E-50	2.47E-49	cyan	-0.7595815	9.28E-31					hypothetical protein
Sapur.002G100400.1	69.11783709	-1.041180626	0.069980524	-14.8781	4.57E-50	2.51E-49	cyan	-0.777589988	4.79E-33		AT5G47830.1			hypothetical protein
Sapur.004G037900.1	22.22032139	5.730422922	0.385261899	14.8741	4.85E-50	2.66E-49	cyan	0.592869166	2.83E-16	GO:0006629	AT4G18550.1		alpha/beta-Hydrolases superfamily protein	phospholipase A1
Sapur.007G062600.1	33.84484008	1.262863074	0.08491115	14.87276	4.95E-50	2.71E-49	cyan	0.710367061	2.04E-25		AT3G19740.1		P-loop containing nucleoside triphosphate hydrolases superfamily protein	NA
Sapur.002G014800.1	26.62740327	-1.430674337	0.096249688	-14.8642	5.63E-50	3.08E-49	cyan	-0.781059472	1.64E-33					hypothetical protein
Sapur.013G128500.1	1197.026259	0.52053777	0.035051309	14.85074	6.88E-50	3.76E-49	cyan	0.773818697	1.50E-32	GO:0016021,GO:0015031	AT2G20840.1		Secretory carrier membrane protein (SCAMP) family protein	secretory carrier-associated membrane protein
Sapur.003G099900.1	83.52591423	-1.74468378	0.117549086	-14.8422	7.82E-50	4.27E-49	cyan	-0.799054457	4.53E-36	GO:0006355,GO:0003700	AT4G23750.1	CRF2,TMO3	cytokinin response factor 2	AP2/ERF domain transcription factor
Sapur.016G125100.1	210.5608937	-0.500480343	0.033728229	-14.8386	8.24E-50	4.51E-49	cyan	-0.748048067	2.14E-29	GO:0008270,GO:0005515	AT3G60080.1		RING/U-box superfamily protein	NA
Sapur.004G127600.1	4549.321039	-1.021387044	0.068844577	-14.8361	8.56E-50	4.67E-49	cyan	-0.741655992	1.13E-28	GO:0006457,GO:0003755,GO:0000413	AT2G16600.1	ROC3	rotamase CYP 3	peptidyl-prolyl cis-trans isomerase, cyclophilin-type protein
Sapur.016G119300.1	63.6890562	-0.875768574	0.059042109	-14.8329	8.97E-50	4.90E-49	cyan	-0.738266463	2.68E-28		AT4G15010.1		Mitochondrial substrate carrier family protein	substrate carrier family protein
Sapur.010G111500.1	268.1144098	-0.729356878	0.049188787	-14.8277	9.70E-50	5.30E-49	cyan	-0.752804676	5.99E-30		AT5G49400.1		zinc knuckle (CCHC-type) family protein	zinc knuckle protein
Sapur.006G012200.1	236.6150618	0.591626682	0.039927972	14.81735	1.13E-49	6.18E-49	cyan	0.758631168	1.21E-30		AT4G16440.1		ferredoxin hydrogenases	cytosolic Fe-S cluster assembly factor narfl protein
Sapur.011G085100.1	13.97841595	1.583440386	0.106917339	14.80995	1.26E-49	6.89E-49	cyan	0.700790488	1.66E-24	GO:0005515	AT5G22730.1		F-box/RNI-like/FBD-like domains-containing protein	NA
Sapur.003G019700.1	2470.546162	0.442583302	0.02988843	14.80785	1.30E-49	7.11E-49	cyan	0.748115371	2.10E-29	GO:0007034	AT1G17730.1	CHMP1B,VPS46.1	vacuolar protein sorting 46.1	charged multivesicular body protein 1b
Sapur.006G068600.1	2.966549528	-3.269523333	0.220828464	-14.8057	1.35E-49	7.34E-49	cyan	-0.764855361	2.08E-31	GO:0006355,GO:0003677	AT2G37590.1	ATDOF2.4,DOF2.4	DNA binding with one finger 2.4	Dof zinc finger protein
Sapur.011G019200.1	905.3535912	0.562719729	0.038007977	14.80531	1.35E-49	7.38E-49	cyan	0.775482863	9.09E-33	GO:0046872	AT1G61690.1		phosphoinositide binding	FYVE zinc finger protein, putative
Sapur.009G040100.1	293.8295028	-1.281486492	0.086592966	-14.799	1.49E-49	8.10E-49	cyan	-0.738833424	2.32E-28		AT3G20898.1			hypothetical protein
Sapur.T005600.1	443.9248455	0.940119814	0.063532978	14.79735	1.52E-49	8.30E-49	cyan	0.751253036	9.10E-30	GO:0055085,GO:0016020,GO:0006810,GO:0015171,GO:0003333	AT1G58030.1	CAT2	cationic amino acid transporter 2	NA
Sapur.012G059900.1	32.75618172	-1.329737499	0.089871889	-14.7959	1.56E-49	8.47E-49	cyan	-0.759853343	8.60E-31		AT1G23710.1		Protein of unknown function (DUF1645)	DUF1645 family protein
Sapur.013G125900.1	13.41084975	-1.348643736	0.091153529	-14.7953	1.57E-49	8.55E-49	cyan	-0.743883568	6.37E-29	GO:0008080	AT1G03650.1		Acyl-CoA N-acyltransferases (NAT) superfamily protein	ribosomal-protein alanine acetyltransferase, putative
Sapur.018G071500.1	61.08026384	1.235273842	0.083491388	14.79522	1.57E-49	8.56E-49	cyan	0.697185922	3.59E-24		AT4G30320.1		CAP (Cysteine-rich secretory proteins, Antigen 5, and Pathogenesis-related 1 protein) superfamily protein	allergen V5/Tpx family protein
Sapur.001G071400.1	7853.314054	0.655633649	0.044325441	14.79136	1.67E-49	9.06E-49	cyan	0.736543928	4.14E-28		AT5G42020.1	BIP,BIP2	Heat shock protein 70 (Hsp 70) family protein	mediator of RNA polymerase II transcription protein
Sapur.15WG104000.1	87.17740033	-1.02025343	0.069004797	-14.7853	1.82E-49	9.91E-49	cyan	-0.811640434	5.10E-38		AT5G14410.1			hypothetical protein
Sapur.006G124000.1	147.9702751	-0.543773497	0.03677849	-14.7851	1.83E-49	9.93E-49	cyan	-0.744927305	4.85E-29		AT5G56780.1	ATET2,ET2	effector of transcription2	transcripteion factor
Sapur.018G079700.1	610.6814261	-0.529518473	0.035823974	-14.7811	1.94E-49	1.05E-48	cyan	-0.758348562	1.31E-30	GO:0006413,GO:0003743	AT2G24060.1		Translation initiation factor 3 protein	translation initiation factor IF-3
Sapur.007G094600.1	682.191446	1.265905179	0.085645492	14.78076	1.95E-49	1.06E-48	cyan	0.707136369	4.18E-25		AT5G44860.1			transmembrane protein, putative
Sapur.002G137800.1	68.54895628	-1.464880211	0.099127023	-14.7778	2.04E-49	1.11E-48	cyan	-0.767214091	1.06E-31		AT2G46820.1	PSAP,PSI-P,PTAC8,TMP14	photosystem I P subunit	cyanobacterial aminoacyl-tRNA synthetase CAAD domain protein
Sapur.003G142200.1	238.4420131	-0.973118183	0.065860088	-14.7755	2.11E-49	1.14E-48	cyan	-0.747745976	2.32E-29		AT5G43150.1			hypothetical protein
Sapur.15WG030600.1	31.702645	-1.248442427	0.08451144	-14.7725	2.20E-49	1.20E-48	cyan	-0.743339904	7.33E-29	GO:0006284	AT3G07930.3		DNA glycosylase superfamily protein	NA
Sapur.006G061700.1	39.75439716	1.795963346	0.121614776	14.76764	2.37E-49	1.28E-48	cyan	0.746108104	3.56E-29		AT5G57240.1	ORP4C	OSBP(oxysterol binding protein)-related protein 4C	oxysterol-binding protein OBPa protein
Sapur.002G017900.1	274.6655697	-0.960198009	0.065036211	-14.7641	2.50E-49	1.35E-48	cyan	-0.790726766	7.44E-35	GO:0005975,GO:0004553	AT1G19940.1	AtGH9B5,GH9B5	glycosyl hydrolase 9B5	endo-1%2C4-beta-glucanase
Sapur.017G096700.1	31.31724871	-1.227805761	0.083174343	-14.7618	2.58E-49	1.40E-48	cyan	-0.763607347	2.98E-31					hypothetical protein
Sapur.009G043700.4	21.50726407	-1.118593393	0.075799014	-14.7574	2.76E-49	1.49E-48	cyan	-0.765378223	1.79E-31	GO:0016987,GO:0006355,GO:0006352,GO:0003700,GO:0003677	AT5G13730.1	SIG4,SIGD	sigma factor 4	RNA polymerase sigma factor RpoD, putative
Sapur.15WG057000.1	2.317188291	-4.058135604	0.275030677	-14.7552	2.85E-49	1.54E-48	cyan	-0.689074877	1.94E-23	GO:0016021,GO:0012511	AT3G18570.1		Oleosin family protein	oleosin, putative
Sapur.018G005900.1	18.03748984	2.060285916	0.139637958	14.75448	2.88E-49	1.56E-48	cyan	0.592322019	3.06E-16		AT5G57510.1			DUF761 domain protein
Sapur.013G000300.1	20.82608791	1.212355339	0.082175262	14.75329	2.93E-49	1.59E-48	cyan	0.736315537	4.39E-28					hypothetical protein
Sapur.004G140200.1	32.24943562	-1.236444595	0.083817074	-14.7517	3.00E-49	1.62E-48	cyan	-0.732895081	1.03E-27	GO:0006457,GO:0003755,GO:0000413	AT3G44600.1	CYP71	cyclophilin71	peptidyl-prolyl cis-trans isomerase, cyclophilin-type protein
Sapur.014G013000.1	975.5519397	-0.717289745	0.048624667	-14.7516	3.01E-49	1.63E-48	cyan	-0.761684633	5.14E-31	GO:0016597,GO:0008152,GO:0055114,GO:0051287,GO:0016616	AT4G34200.1	EDA9	D-3-phosphoglycerate dehydrogenase	D-3-phosphoglycerate dehydrogenase
Sapur.011G029400.1	6.213304323	-1.913764407	0.129735788	-14.7512	3.02E-49	1.63E-48	cyan	-0.748065429	2.13E-29		AT4G21570.1		Protein of unknown function (DUF300)	organic solute transporter ostalpha protein
Sapur.019G043900.1	9.008730132	2.925487793	0.198367494	14.74782	3.18E-49	1.72E-48	cyan	0.582496214	1.22E-15		AT1G64980.1		Nucleotide-diphospho-sugar transferases superfamily protein	NA
Sapur.006G131600.1	4.936086788	5.185135444	0.351707583	14.74275	3.43E-49	1.85E-48	cyan	0.547230213	1.21E-13	GO:0016747	AT1G03390.1		HXXXD-type acyl-transferase family protein	taxadien-5-alpha-ol O-acetyltransferase, putative
Sapur.005G108300.1	99.81890923	-0.738339424	0.050098876	-14.7376	3.69E-49	2.00E-48	cyan	-0.788064001	1.77E-34	GO:0006355,GO:0003700	AT5G67190.1	DEAR2	DREB and EAR motif protein 2	ethylene-responsive transcription factor ERF010
Sapur.013G039600.1	647.8286475	-0.850992006	0.057747143	-14.7365	3.76E-49	2.03E-48	cyan	-0.777451486	4.99E-33	GO:0006334,GO:0005634,GO:0003677,GO:0000786	AT2G30620.2		winged-helix DNA-binding transcription factor family protein	histone H1 subtype 5
Sapur.013G068600.1	324.9103504	1.257580005	0.085350049	14.73438	3.88E-49	2.09E-48	cyan	0.746198117	3.48E-29	GO:0005515	AT3G03680.1		C2 calcium/lipid-binding plant phosphoribosyltransferase family protein	anthranilate phosphoribosyltransferase-like protein
Sapur.016G179400.1	4.286509607	3.810487339	0.258674967	14.73079	4.09E-49	2.21E-48	cyan	0.67492528	3.25E-22		AT1G49800.1			transmembrane protein, putative
Sapur.003G133400.1	1355.930713	-1.473656901	0.100057935	-14.728	4.26E-49	2.30E-48	cyan	-0.71108204	1.74E-25	GO:0016747,GO:0008610	AT5G13930.1	ATCHS,CHS,TT4	Chalcone and stilbene synthase family protein	chalcone synthase
Sapur.001G151400.1	836.7243456	0.63570647	0.043167577	14.72648	4.36E-49	2.35E-48	cyan	0.74055387	1.50E-28	GO:0005525	AT1G52280.1	AtRABG3d,RABG3d	RAB GTPase homolog G3D	Rab-related GTPase family protein
Sapur.002G161600.1	1225.736312	0.49265953	0.033458899	14.72432	4.50E-49	2.43E-48	cyan	0.735544421	5.32E-28		AT3G62600.1	ATERDJ3B,ERDJ3B	DNAJ heat shock family protein	chaperone protein DnaJ
Sapur.009G048300.1	17.56544585	-1.371498719	0.093173224	-14.7199	4.81E-49	2.59E-48	cyan	-0.77325484	1.78E-32					hypothetical protein
Sapur.003G065300.1	7.445890671	4.658444887	0.316577217	14.71504	5.16E-49	2.78E-48	cyan	0.609706288	2.36E-17	GO:0043531	AT3G07040.1	RPM1,RPS3	NB-ARC domain-containing disease resistance protein	NBS-LRR type disease resistance protein
Sapur.016G087100.1	21.55497762	-1.143149082	0.077743122	-14.7042	6.06E-49	3.26E-48	cyan	-0.766567999	1.27E-31	GO:0005515	AT1G70280.2		NHL domain-containing protein	NHL domain protein
Sapur.008G129200.1	41.29847887	-1.744870228	0.118748798	-14.6938	7.06E-49	3.80E-48	cyan	-0.672414343	5.27E-22		AT4G14640.1	CAM8	calmodulin 8	calmodulin
Sapur.009G015600.1	4858.364734	0.534153022	0.036367045	14.68783	7.71E-49	4.15E-48	cyan	0.740101151	1.68E-28		AT2G27030.3	ACAM-2,CAM5	calmodulin 5	calmodulin cam-203
Sapur.017G089000.1	173.0710739	-1.023169548	0.069676322	-14.6846	8.09E-49	4.35E-48	cyan	-0.754902511	3.38E-30	GO:0030170,GO:0004834,GO:0000162	AT5G38530.1	TSBtype2	tryptophan synthase beta type 2	tryptophan synthase beta chain
Sapur.007G006300.1	644.03503	-0.971091184	0.066150103	-14.6801	8.64E-49	4.65E-48	cyan	-0.750087037	1.24E-29	GO:0043565,GO:0006355,GO:0003700,GO:0003677	AT4G37790.1	HAT22	Homeobox-leucine zipper protein family	homeobox leucine zipper protein
Sapur.012G035100.1	6.0752527	-2.087618886	0.142310956	-14.6694	1.01E-48	5.44E-48	cyan	-0.767988777	8.42E-32	GO:0005524	AT3G18190.1		TCP-1/cpn60 chaperonin family protein	NA
Sapur.006G056100.1	86.79659742	1.591878343	0.108520357	14.66894	1.02E-48	5.47E-48	cyan	0.687347381	2.76E-23		AT2G26660.1	ATSPX2,SPX2	SPX domain gene 2	xenotropic and polytropic murine leukemia virus receptor ids-4, putative
Sapur.009G081900.1	211.6414888	0.968639035	0.066044673	14.66642	1.06E-48	5.68E-48	cyan	0.738697563	2.41E-28		AT1G19190.1		alpha/beta-Hydrolases superfamily protein	gibberellin receptor GID1, putative
Sapur.15WG105000.1	106.8980713	-0.571819342	0.03900521	-14.6601	1.16E-48	6.23E-48	cyan	-0.783953312	6.60E-34		AT4G24760.1		alpha/beta-Hydrolases superfamily protein	alpha/beta hydrolase family protein
Sapur.001G149400.1	98.8535084	1.059036743	0.072262922	14.65533	1.25E-48	6.68E-48	cyan	0.71153449	1.57E-25	GO:0016020,GO:0008375	AT5G11730.1		Core-2/I-branching beta-1,6-N-acetylglucosaminyltransferase family protein	NA
Sapur.006G133900.1	22.55860883	-1.154952704	0.078809172	-14.6551	1.25E-48	6.71E-48	cyan	-0.795382183	1.58E-35		AT1G77170.1		Tetratricopeptide repeat (TPR)-like superfamily protein	PPR containing plant protein
Sapur.007G091800.1	77.37050522	-0.989849377	0.067560711	-14.6513	1.32E-48	7.09E-48	cyan	-0.772310553	2.36E-32		AT5G09270.2			transmembrane protein, putative
Sapur.011G123900.1	19.95893342	1.662294399	0.113559156	14.63814	1.60E-48	8.59E-48	cyan	0.677004805	2.17E-22		AT5G56110.1	AtMYB103.ATMYB80.MS18	myb domain protein 103	Myb-like DNA-binding domain protein
Sapur.016G233500.1	24.71984071	-1.157819264	0.079100954	-14.6372	1.63E-48	8.70E-48	cyan	-0.757280762	1.76E-30	GO:0006355,GO:0003723	AT3G13180.1		NOL1/NOP2/sun family protein / antitermination NusB domain-containing protein	ribosomal RNA small subunit methyltransferase B
Sapur.017G020000.1	57.99848055	-0.847907705	0.057952969	-14.631	1.78E-48	9.53E-48	cyan	-0.756852607	1.98E-30		AT1G62930.1		Tetratricopeptide repeat (TPR)-like superfamily protein	PPR domain protein
Sapur.006G177000.1	38.82867593	-1.143848324	0.078184453	-14.6301	1.80E-48	9.65E-48	cyan	-0.79812494	6.24E-36		AT2G37310.1		Pentatricopeptide repeat (PPR) superfamily protein	NA
Sapur.003G119700.1	4496.789725	0.497793214	0.034026826	14.62943	1.82E-48	9.75E-48	cyan	0.733582569	8.66E-28	GO:0005975	AT5G13420.1		Aldolase-type TIM barrel family protein	transaldolase
Sapur.009G063200.1	6.289944959	1.867188395	0.12767804	14.62419	1.97E-48	1.05E-47	cyan	0.781269381	1.53E-33		AT4G37880.1		LisH/CRA/RING-U-box domains-containing protein	sporulation protein RMD5, putative
Sapur.15WG084600.1	922.5624976	0.931607913	0.063709303	14.62279	2.01E-48	1.07E-47	cyan	0.686061783	3.59E-23	GO:0003677,GO:0043565,GO:0006355,GO:0003700	AT3G01470.1	ATHB-1.ATHB1.ATHT5.HB-1.I	homeobox 1	homeobox leucine zipper protein, putative
Sapur.003G160500.1	10.86176564	2.296356648	0.157059003	14.62098	2.06E-48	1.10E-47	cyan	0.660818167	4.63E-21	GO:0006468,GO:0005524,GO:0004672	AT5G60900.1	RLK1	receptor-like protein kinase 1	Serine/Threonine kinase domain protein
Sapur.011G106800.1	104.4243831	0.68723865	0.047009578	14.61912	2.12E-48	1.13E-47	cyan	0.783315942	8.08E-34	GO:0008270	AT1G19100.1		Histidine kinase-, DNA gyrase B-, and HSP90-like ATPase family protein	MORC family CW-type zinc finger protein
Sapur.003G104100.1	173.2109214	-0.836084918	0.05719506	-14.6181	2.15E-48	1.15E-47	cyan	-0.778180193	3.99E-33		AT1G64450.1		Glycine-rich protein family	late embryogenesis abundant hydroxyproline-rich glycoprotein
Sapur.005G113700.1	286.0193241	0.548393761	0.037518866	14.61648	2.21E-48	1.18E-47	cyan	0.733789438	8.23E-28		AT4G14600.1		Target SNARE coiled-coil domain protein	NA
Sapur.018G012200.1	108.8892088	-1.292787326	0.08844836	-14.6163	2.21E-48	1.18E-47	cyan	-0.729295495	2.48E-27	GO:0051301,GO:0031110,GO:0008017,GO:0007067,GO:0007059,GO:0005876,GO:000094	AT2G24970.1			hypothetical protein
Sapur.010G151300.1	127.4077267	0.951824608	0.065177664	14.60354	2.67E-48	1.42E-47	cyan	0.729351775	2.44E-27		AT5G05790.1		Duplicated homeodomain-like superfamily protein	MYB transcription factor
Sapur.008G004900.1	7574.25692	0.953588482	0.065298695	14.60348	2.67E-48	1.42E-47	cyan	0.69281683	8.98E-24	GO:0016597,GO:0008152,GO:0055114,GO:0051287,GO:0016616	AT1G17745.1	PGDH	D-3-phosphoglycerate dehydrogenase	D-3-phosphoglycerate dehydrogenase
Sapur.017G027000.1	151.0956597	0.782193626	0.053565141	14.60266	2.70E-48	1.44E-47	cyan	0.751446331	8.64E-30		AT2G43250.1			NA
Sapur.017G071900.1	6609.65108	0.747776278	0.051208359	14.60262	2.70E-48	1.44E-47	cyan	0.718955286	2.88E-26		AT5G39570.1			latex abundant protein, putative
Sapur.019G068800.1	122.4296452	-0.53324771	0.036545599	-14.5913	3.19E-48	1.70E-47	cyan	-0.762712579	3.84E-31	GO:0003676	AT4G10110.1		RNA-binding (RRM/RBD/RNP motifs) family protein	RNA recognition motif protein
Sapur.017G066900.1	529.0088725	2.160197591	0.148171518	14.57903	3.82E-48	2.03E-47	cyan	0.686266606	3.44E-23	GO:0016853,GO:0005975	AT3G47800.1		Galactose mutarotase-like superfamily protein	NA
Sapur.003G041800.1	4823.079427	0.635702169	0.043633117	14.56926	4.41E-48	2.34E-47	cyan	0.735517562	5.35E-28	GO:0030170,GO:0009058	AT1G72330.1	ALAAT2	alanine aminotransferase 2	alanine aminotransferase
Sapur.004G159200.1	383.3496341	-0.614771731	0.042213204	-14.5635	4.79E-48	2.55E-47	cyan	-0.769984962	4.69E-32	GO:0003677,GO:0008289	AT5G60690.1	IFL,IFL1,REV	Homeobox-leucine zipper family protein / lipid-binding START domain-containing protein	homeobox leucine-zipper protein
Sapur.005G055400.1	258.2936465	-0.754554638	0.051859367	-14.55	5.84E-48	3.10E-47	cyan	-0.76841484	7.44E-32		AT5G65280.1	GCL1	GCR2-like 1	G protein coupled receptor
Sapur.15WG090200.1	400.4303067	0.572623302	0.039359808	14.54843	5.98E-48	3.17E-47	cyan	0.738956889	2.25E-28	GO:0016020,GO:0005509,GO:0004571	AT1G27520.1		Glycosyl hydrolase family 47 protein	glycoside hydrolase family 47 protein
Sapur.006G188600.1	336.0934964	-0.714732957	0.049134288	-14.5465	6.14E-48	3.26E-47	cyan	-0.760330861	7.53E-31		AT5G11890.1			harpin-induced protein, putative
Sapur.004G134700.1	83.13053661	-1.471627134	0.101197775	-14.5421	6.56E-48	3.47E-47	cyan	-0.736937423	3.75E-28	GO:0055085,GO:0016021	AT2G16980.2		Major facilitator superfamily protein	NA
Sapur.006G221900.1	63.7382818	1.540507733	0.105939581	14.54138	6.62E-48	3.51E-47	cyan	0.755684788	2.73E-30	GO:0007165,GO:0000155,GO:0000160	AT5G10720.1	AHK5,CKI2,HK5	histidine kinase 5	histidine kinase cytokinin receptor
Sapur.016G290000.1	20.60520734	1.991071323	0.136924721	14.54136	6.63E-48	3.51E-47	cyan	0.704074776	8.18E-25		AT1G32928.1			Avr9/Cf-9 rapidly elicited protein, putative
Sapur.004G074400.1	844.4047589	-0.980639566	0.067452815	-14.5382	6.94E-48	3.67E-47	cyan	-0.74012383	1.67E-28		AT5G15780.1		Pollen Ole e 1 allergen and extensin family protein	pollen protein Ole E I-like protein
Sapur.011G116500.1	77.90663504	-0.937843842	0.064521248	-14.5354	7.23E-48	3.82E-47	cyan	-0.787520026	2.11E-34		AT4G21300.1		Tetratricopeptide repeat (TPR)-like superfamily protein	NA
Sapur.003G073300.1	4.263955241	4.453370514	0.306406643	14.53418	7.36E-48	3.89E-47	cyan	0.666461172	1.63E-21		AT5G61460.1	ATRAD18,MIM,SMC6B	P-loop containing nucleoside triphosphate hydrolases superfamily protein	structural maintenance of chromosomes protein
Sapur.005G116600.1	188.2185603	1.069880472	0.073626297	14.53123	7.68E-48	4.06E-47	cyan	0.718168534	3.46E-26	GO:0005524	AT1G50140.1		P-loop containing nucleoside triphosphate hydrolases superfamily protein	NA
Sapur.013G050200.2	4.205388318	-2.638404657	0.181574484	-14.5307	7.74E-48	4.09E-47	cyan	-0.735122687	5.91E-28	GO:0005515,GO:0016020	AT1G08560.1	ATSYP111,KN,SYP111	syntaxin of plants 111	syntaxin-related protein knolle protein
Sapur.006G027600.1	49.86766398	-1.16958003	0.080501944	-14.5286	7.98E-48	4.22E-47	cyan	-0.717678454	3.87E-26					NA
Sapur.008G165400.1	336.6122112	0.434623034	0.029920821	14.52577	8.32E-48	4.39E-47	cyan	0.762779095	3.77E-31	GO:0008270,GO:0005515	AT2G39100.1		RING/U-box superfamily protein	zinc-RING finger protein
Sapur.016G111400.2	2.337313823	-3.637082793	0.250440962	-14.5227	8.70E-48	4.59E-47	cyan	-0.773563815	1.62E-32		AT3G51670.1		SEC14 cytosolic factor family protein / phosphoglyceride transfer family protein	patellin-6 protein
Sapur.019G022600.1	318.0183026	-0.833561898	0.057418814	-14.5172	9.42E-48	4.97E-47	cyan	-0.7607642	6.66E-31		AT3G04590.2		AT hook motif DNA-binding family protein	SAP1 protein, putative
Sapur.004G043400.1	448.2076134	0.734038588	0.050572616	14.51455	9.80E-48	5.17E-47	cyan	0.724431492	7.97E-27	GO:0046872	AT1G29800.1		RING/FYVE/PHD-type zinc finger family protein	FYVE zinc finger protein
Sapur.010G173800.1	2.411515727	4.716924724	0.325110225	14.50869	1.07E-47	5.62E-47	cyan	0.578975119	1.98E-15					NA
Sapur.016G220600.1	311.1821826	1.144893068	0.078927327	14.50566	1.12E-47	5.88E-47	cyan	0.678817618	1.52E-22	GO:0016758,GO:0008152	AT2G16890.2		UDP-Glycosyltransferase superfamily protein	UDP-glycosyltransferase
Sapur.005G086500.1	9.784355171	-1.558239378	0.107446381	-14.5025	1.17E-47	6.15E-47	cyan	-0.739975151	1.74E-28	GO:0005634	AT3G01160.1			NA
Sapur.018G007400.1	488.3966392	1.580018099	0.108967528	14.4999	1.21E-47	6.39E-47	cyan	0.70421881	7.93E-25	GO:0016758,GO:0008152	AT2G15490.3	UGT73B4	UDP-glycosyltransferase 73B4	UDP-glucosyltransferase family protein
Sapur.007G120600.1	18.63793375	-1.170249264	0.080712734	-14.4989	1.23E-47	6.47E-47	cyan	-0.762074842	4.60E-31		AT2G30700.1			hypothetical protein
Sapur.006G143200.1	134.9835577	0.66809794	0.046087313	14.49635	1.28E-47	6.72E-47	cyan	0.751141607	9.37E-30		AT4G30480.2		Tetratricopeptide repeat (TPR)-like superfamily protein	anaphase-promoting complex, cyclosome%2C subunit 3
Sapur.016G158600.1	350.2326242	-0.712660068	0.049179424	-14.491	1.38E-47	7.26E-47	cyan	-0.765489888	1.74E-31	GO:0003676	AT5G12190.1		RNA-binding (RRM/RBD/RNP motifs) family protein	RNA recognition motif 1 in RNA-binding protein 28 (RBM28)-like protein
Sapur.15WG098600.1	10.04734286	-1.82520943	0.125955051	-14.491	1.38E-47	7.26E-47	cyan	-0.768382212	7.51E-32	GO:0006412,GO:0005840,GO:0005622,GO:0003735	AT5G07090.1		Ribosomal protein S4 (RPS4A) family protein	NA
Sapur.001G123900.1	1110.994276	1.051960642	0.07261008	14.4878	1.45E-47	7.60E-47	cyan	0.719913244	2.31E-26	GO:0016161,GO:0000272	AT4G17090.1	BAM3,BMY8,CT-BMY	chloroplast beta-amylase	beta-amylase
Sapur.001G150400.1	606.5746289	0.841193974	0.058066436	14.48675	1.47E-47	7.72E-47	cyan	0.692211467	1.02E-23		AT5G11970.1		Protein of unknown function (DUF3511)	DUF3511 domain protein
Sapur.006G109200.1	1406.182588	-0.751657152	0.051889396	-14.4858	1.49E-47	7.83E-47	cyan	-0.772376076	2.31E-32	GO:0006412,GO:0005840,GO:0003735	AT3G09630.1		Ribosomal protein L4/L1 family	60S ribosomal protein L4-1
Sapur.008G078800.1	24.91134931	1.820877671	0.125714428	14.48424	1.52E-47	8.00E-47	cyan	0.71623578	5.40E-26	GO:0055114,GO:0051920,GO:0016491,GO:0016209	AT1G48130.1	ATPER1,PER1	1-cysteine peroxiredoxin 1	1-cys peroxiredoxin
Sapur.15WG027000.1	91.94721635	-0.707980328	0.048887467	-14.4818	1.58E-47	8.28E-47	cyan	-0.764238931	2.49E-31		AT3G17690.1	ATCNGC19,CNGC19	cyclic nucleotide gated channel 19	NA
Sapur.007G029200.1	299.3478368	0.812862182	0.056154804	14.47538	1.73E-47	9.10E-47	cyan	0.745630384	4.04E-29	GO:0016021	AT4G36830.1	HOS3-1	GNS1/SUR4 membrane protein family	GNS1/SUR4 membrane family protein
Sapur.T017300.1	33.00567746	1.42399152	0.098487778	14.45856	2.21E-47	1.16E-46	cyan	0.694062298	6.92E-24	GO:0006468,GO:0004672	AT3G14840.2		Leucine-rich repeat transmembrane protein kinase	NA
Sapur.002G169400.1	210.5474026	2.275315873	0.157384913	14.45701	2.26E-47	1.19E-46	cyan	0.591214397	3.58E-16		AT3G63095.1		Tetratricopeptide repeat (TPR)-like superfamily protein	transmembrane protein, putative
Sapur.004G128900.1	269.293811	-1.647680151	0.113986584	-14.455	2.33E-47	1.22E-46	cyan	-0.689737514	1.70E-23		AT4G36040.1		Chaperone DnaJ-domain superfamily protein	NA
Sapur.001G151700.1	41.02507765	-1.3864705	0.09594149	-14.4512	2.46E-47	1.29E-46	cyan	-0.793188279	3.30E-35		AT1G15260.1			ATP-dependent RNA helicase chl1, putative
Sapur.001G181000.1	55.69589249	-0.735763656	0.050926451	-14.4476	2.60E-47	1.36E-46	cyan	-0.769048882	6.18E-32		AT4G24320.1		Ubiquitin carboxyl-terminal hydrolase family protein	plant organelle RNA recognition domain protein
Sapur.016G025600.1	76.99697389	-0.876285191	0.060663096	-14.4451	2.69E-47	1.41E-46	cyan	-0.770818858	3.67E-32					NA
Sapur.013G138700.1	1248.390725	0.467741333	0.032384522	14.44336	2.76E-47	1.44E-46	cyan	0.755726441	2.70E-30		AT1G06900.1		Insulinase (Peptidase family M16) family protein	N-arginine dibasic convertase, putative
Sapur.011G049400.1	328.7876256	-0.706259336	0.048907881	-14.4406	2.87E-47	1.50E-46	cyan	-0.76396245	2.69E-31	GO:0005525	AT5G45750.1	AtRABA1c,RABA1c	RAB GTPase homolog A1C	Rab-related GTPase family protein
Sapur.001G065600.1	5729.186486	-0.647798144	0.04488189	-14.4334	3.19E-47	1.67E-46	cyan	-0.752588745	6.35E-30	GO:0015979,GO:0009538,GO:0009522	AT1G31330.1	PSAF	photosystem I subunit F	photosystem I reaction center subunit III
Sapur.014G023600.1	99.42698446	3.145766302	0.218011822	14.42934	3.38E-47	1.77E-46	cyan	0.586155122	7.32E-16	GO:0006629	AT2G26560.1	PLA IIA,PLA2A,PLP2	phospholipase A 2A	NA
Sapur.018G005000.4	4.724593788	2.754869205	0.190934595	14.42834	3.43E-47	1.79E-46	cyan	0.847360362	1.86E-44	GO:0008270,GO:0005515	AT5G24870.2		RING/U-box superfamily protein	zinc finger protein, putative
Sapur.007G013500.1	636.8232707	0.600042491	0.041592676	14.42664	3.52E-47	1.84E-46	cyan	0.727371606	3.94E-27		AT3G51100.1			hypothetical protein
Sapur.006G148900.1	140.970796	1.22813882	0.085134198	14.42592	3.56E-47	1.86E-46	cyan	0.698152759	2.92E-24		AT5G08110.1		nucleic acid binding;ATP-dependent helicases;ATP binding;helicases;ATP-dependent helicases	NA
Sapur.003G107100.1	273.7093921	0.729370442	0.050568095	14.42353	3.68E-47	1.92E-46	cyan	0.714232963	8.53E-26	GO:0006355,GO:0003677	AT4G24060.1		Dof-type zinc finger DNA-binding family protein	Dof zinc finger protein, putative
Sapur.002G075200.1	4130.691187	1.009750777	0.070008269	14.42331	3.69E-47	1.93E-46	cyan	0.718226491	3.41E-26		AT4G30100.1		P-loop containing nucleoside triphosphate hydrolases superfamily protein	tRNA-splicing endonuclease positive effector-like protein
Sapur.15WG111000.1	214.3879709	-0.738843559	0.051248336	-14.4169	4.05E-47	2.11E-46	cyan	-0.766466287	1.31E-31		AT5G50375.1	CPI1	cyclopropyl isomerase	cycloeucalenol cycloisomerase
Sapur.011G077400.1	2058.320672	-0.742902325	0.051549768	-14.4114	4.39E-47	2.29E-46	cyan	-0.730357886	1.91E-27	GO:0016491	AT3G14420.2		Aldolase-type TIM barrel family protein	peroxisomal (S)-2-hydroxy-acid oxidase, putative
Sapur.010G176600.1	2879.877598	-1.095773777	0.076122188	-14.3949	5.57E-47	2.90E-46	cyan	-0.754919982	3.37E-30	GO:0016020,GO:0009765	AT3G54890.1	LHCA1	photosystem I light harvesting complex gene 1	photosystem I light-harvesting chlorophyll A/B-binding protein
Sapur.010G131900.1	455.5932723	-0.45574926	0.031665695	-14.3925	5.77E-47	3.00E-46	cyan	-0.744899865	4.89E-29		AT3G19840.1	ATPRP40C,PRP40C	pre-mRNA-processing protein 40C	NA
Sapur.007G099100.1	16.44002805	-1.061985051	0.073793216	-14.3914	5.86E-47	3.05E-46	cyan	-0.776490846	6.69E-33		AT3G12490.1	ATCYS6,ATCYSB,CYSB	cystatin B	NA
Sapur.15ZG039800.1	390.931455	0.642654609	0.044661028	14.38961	6.01E-47	3.13E-46	cyan	0.768707383	6.83E-32		AT5G07980.1		dentin sialophosphoprotein-related	hypothetical protein
Sapur.018G093000.1	11.488432	-1.054162249	0.073297298	-14.382	6.71E-47	3.49E-46	cyan	-0.804522154	6.72E-37		AT5G14600.1		S-adenosyl-L-methionine-dependent methyltransferases superfamily protein	NA
Sapur.15WG119300.1	90.58240065	-1.144611023	0.079589534	-14.3814	6.77E-47	3.52E-46	cyan	-0.807254849	2.53E-37	GO:0005515	AT3G48800.1		Sterile alpha motif (SAM) domain-containing protein	BICC1 (bicaudal) family SAM domain protein
Sapur.013G121500.1	1627.598513	1.134657945	0.078900549	14.38086	6.82E-47	3.55E-46	cyan	0.702173001	1.24E-24	GO:0016020,GO:0006075,GO:0003843,GO:0000148	AT4G03550.1	ATGSL05.ATGSL5.GSL05.GSL	glucan synthase-like 5	1%2C3-beta-glucan synthase
Sapur.008G024900.1	706.1749619	0.636868529	0.044293642	14.37833	7.08E-47	3.68E-46	cyan	0.773704554	1.55E-32	GO:0005515	AT3G10390.1	FLD	Flavin containing amine oxidoreductase family protein	lysine-specific histone demethylase-like protein
Sapur.006G140800.1	229.249243	-1.670645159	0.1161964	-14.3778	7.14E-47	3.71E-46	cyan	-0.77068397	3.82E-32		AT5G57685.1	ATGDU3,GDU3,LSB1	glutamine dumper 3	NA
Sapur.016G116700.1	576.4471136	0.493605172	0.034341543	14.37341	7.60E-47	3.95E-46	cyan	0.741471635	1.19E-28	GO:0016192,GO:0016021	AT5G22360.1	ATVAMP714,VAMP714	vesicle-associated membrane protein 714	vesicle-associated membrane family protein, putative
Sapur.001G144900.1	288.9796335	0.521036677	0.03625246	14.37245	7.71E-47	4.00E-46	cyan	0.770114577	4.52E-32		AT3G25910.1		Protein of unknown function (DUF1644)	NA
Sapur.010G033000.1	4814.902268	0.894941884	0.06227444	14.37093	7.88E-47	4.09E-46	cyan	0.737470703	3.28E-28	GO:0005515	AT1G10370.1	ATGSTU17.ERD9.GST30.GST	Glutathione S-transferase family protein	glutathione S-transferase
Sapur.011G118100.1	1959.519526	0.91776349	0.06386595	14.37015	7.97E-47	4.14E-46	cyan	0.696109195	4.51E-24	GO:0055114,GO:0050660,GO:0016491	AT1G11770.1		FAD-binding Berberine family protein	reticuline oxidase, putative
Sapur.15WG002400.1	285.8022029	-2.555462662	0.17785898	-14.3679	8.23E-47	4.27E-46	cyan	-0.642551483	1.18E-19	GO:0055114,GO:0020037,GO:0006979,GO:0004601	AT1G05260.1	RCI3,RCI3A	Peroxidase superfamily protein	peroxidase
Sapur.013G085200.1	29.58660102	-1.406015074	0.097882713	-14.3643	8.67E-47	4.50E-46	cyan	-0.716791144	4.75E-26	GO:0006468,GO:0004672	AT3G01090.2	AKIN10,KIN10,SNRK1.1	SNF1 kinase homolog 10	NA
Sapur.008G033700.1	39.62318911	-1.396937137	0.097258156	-14.3632	8.81E-47	4.57E-46	cyan	-0.787074583	2.44E-34	GO:0003676	AT3G54770.1		RNA-binding (RRM/RBD/RNP motifs) family protein	RNA recognition motif 2 in RNA-binding protein musashi-like musashi protein
Sapur.003G132600.1	8.713133697	-2.084050612	0.145129836	-14.3599	9.24E-47	4.79E-46	cyan	-0.72840301	3.08E-27	GO:0005515,GO:0006468,GO:0004672	AT3G24240.1		Leucine-rich repeat receptor-like protein kinase family protein	LRR receptor-like kinase
Sapur.003G136200.1	8.199672246	-1.716218491	0.119529243	-14.3581	9.47E-47	4.91E-46	cyan	-0.755193406	3.13E-30					NA
Sapur.010G179500.1	13440.09606	0.562914526	0.039219539	14.35291	1.02E-46	5.29E-46	cyan	0.75332312	5.20E-30		AT5G03300.1	ADK2	adenosine kinase 2	adenosine kinase
Sapur.004G113000.1	47.60918466	-0.942886626	0.065694139	-14.3527	1.03E-46	5.31E-46	cyan	-0.764996808	2.00E-31	GO:0006355,GO:0005739,GO:0003690	AT3G18870.1		Mitochondrial transcription termination factor family protein	mTERF protein
Sapur.15WG124000.1	79.6909871	-1.656410402	0.11541057	-14.3523	1.03E-46	5.34E-46	cyan	-0.710953724	1.79E-25		AT3G23760.1			transferring glycosyl group transferase, putative
Sapur.016G062100.1	586.1140323	0.568591085	0.039632076	14.34674	1.12E-46	5.78E-46	cyan	0.737786071	3.03E-28		AT2G35900.1			Mal D 1-associated protein
Sapur.003G062600.1	5.993684515	2.346419496	0.163662457	14.33694	1.29E-46	6.65E-46	cyan	0.722785445	1.18E-26					NA
Sapur.014G138200.1	156.9920655	-0.406680619	0.028394021	-14.3228	1.58E-46	8.15E-46	cyan	-0.769854261	4.88E-32		AT4G12710.1		ARM repeat superfamily protein	vacuolar protein, putative
Sapur.009G117600.2	13.05270958	-1.475096052	0.103031013	-14.317	1.71E-46	8.85E-46	cyan	-0.750099443	1.24E-29	GO:0055085,GO:0016020,GO:0006810,GO:0005215	AT2G27810.1	ATNAT12,NAT12	nucleobase-ascorbate transporter 12	nucleobase ascorbate transporter
Sapur.001G053200.1	3791.244944	0.863509222	0.06032281	14.3148	1.77E-46	9.14E-46	cyan	0.679212518	1.41E-22	GO:0005975	AT5G13420.1		Aldolase-type TIM barrel family protein	transaldolase
Sapur.014G054000.1	425.0970214	-0.87474273	0.061111314	-14.3139	1.79E-46	9.25E-46	cyan	-0.725975742	5.51E-27		AT2G45520.1			hypothetical protein
Sapur.016G248600.1	291.7759058	-1.11541687	0.077958404	-14.3078	1.95E-46	1.01E-45	cyan	-0.785068636	4.63E-34		AT1G53380.3		Plant protein of unknown function (DUF641)	UNE1-like protein
Sapur.014G012300.1	138.4053564	-1.143001462	0.079887441	-14.3076	1.96E-46	1.01E-45	cyan	-0.69191038	1.08E-23	GO:0005515	AT4G24210.1	SLY1	F-box family protein	F-box protein GID2
Sapur.T010400.1	5.405630261	2.353959361	0.164588891	14.30205	2.12E-46	1.10E-45	cyan	0.692327118	9.94E-24					NA
Sapur.004G047300.1	422.0709796	-0.554912768	0.038802466	-14.301	2.16E-46	1.11E-45	cyan	-0.754783193	3.50E-30		AT3G05070.1			coiled-coil protein, putative
Sapur.006G121900.1	152.4575724	-0.617508928	0.043184542	-14.2993	2.21E-46	1.14E-45	cyan	-0.739101329	2.17E-28	GO:0006334,GO:0005634,GO:0003677,GO:0000786	AT1G72740.2		Homeodomain-like/winged-helix DNA-binding family protein	MYB transcription factor
Sapur.005G163800.1	184.3735836	-1.494163561	0.104493944	-14.299	2.22E-46	1.14E-45	cyan	-0.721775385	1.49E-26	GO:0055085,GO:0016020,GO:0015297,GO:0015238,GO:0006855	AT3G59030.1	ATTT12,TT12	MATE efflux family protein	MATE transporter
Sapur.009G001300.1	151.8159249	-0.771402442	0.053973996	-14.2921	2.45E-46	1.26E-45	cyan	-0.785171909	4.48E-34	GO:0016020,GO:0008375	AT5G15050.1		Core-2/I-branching beta-1,6-N-acetylglucosaminyltransferase family protein	core-2/I-branching enzyme
Sapur.019G108400.1	131.9547416	1.413590028	0.098908681	14.29187	2.46E-46	1.27E-45	cyan	0.671211614	6.64E-22		AT2G30230.1			DUF4228 domain protein
Sapur.006G182200.2	13.19121756	-1.524792822	0.106702017	-14.2902	2.52E-46	1.30E-45	cyan	-0.744429653	5.52E-29	GO:0006412,GO:0005840,GO:0003735	AT1G07320.1	RPL4	ribosomal protein L4	50S ribosomal protein L4
Sapur.006G066500.1	480.2626489	0.705186879	0.049347747	14.29015	2.52E-46	1.30E-45	cyan	0.721780651	1.49E-26	GO:0055085,GO:0046873,GO:0030001,GO:0016020	AT3G58970.1	MGT6,MRS2-4	magnesium transporter 6	magnesium transporter MRS2
Sapur.003G087200.1	243.7474321	1.540160944	0.107846738	14.28102	2.87E-46	1.48E-45	cyan	0.688747755	2.08E-23	GO:0016021,GO:0004252,GO:0006508	AT1G63120.1	ATRBL2,RBL2	RHOMBOID-like 2	NA
Sapur.010G065100.1	169.3561277	-0.825553456	0.057808862	-14.2807	2.89E-46	1.48E-45	cyan	-0.783727972	7.09E-34	GO:0005515	AT1G04190.1		Tetratricopeptide repeat (TPR)-like superfamily protein	heat shock protein STI
Sapur.012G020200.1	1582.035696	0.47945291	0.033584664	14.27595	3.09E-46	1.59E-45	cyan	0.712685519	1.21E-25		AT5G53300.2	UBC10	ubiquitin-conjugating enzyme 10	ubiquitin-conjugating enzyme E2
Sapur.003G025700.1	5216.539639	-0.675229139	0.047312626	-14.2716	3.29E-46	1.69E-45	cyan	-0.729683357	2.25E-27	GO:0015979,GO:0009538,GO:0009522	AT1G52230.1	PSAH-2,PSAH2,PSI-H	photosystem I subunit H2	photosystem I reaction center subunit VI
Sapur.002G074200.1	12.51844751	-2.045987051	0.143446121	-14.2631	3.72E-46	1.91E-45	cyan	-0.695489084	5.13E-24	GO:0016021,GO:0015116,GO:0008272,GO:0055085,GO:0016020,GO:0008271	AT1G77990.1	AST56,SULTR2;2	STAS domain / Sulfate transporter family	sulfate/bicarbonate/oxalate exchanger and transporter sat-1
Sapur.008G164800.1	36.87530281	-0.7946169	0.055725409	-14.2595	3.91E-46	2.01E-45	cyan	-0.755361467	2.99E-30	GO:0006355,GO:0005739,GO:0003690	AT5G07900.1		Mitochondrial transcription termination factor family protein	mTERF protein
Sapur.T066600.1	4.585765089	3.670044695	0.25743049	14.25645	4.09E-46	2.10E-45	cyan	0.691798676	1.11E-23					NA
Sapur.005G146700.1	547.5153913	-1.186736892	0.083249506	-14.2552	4.16E-46	2.14E-45	cyan	-0.748842273	1.73E-29	GO:0045454,GO:0015035,GO:0006662	AT3G15360.1	ATHM4,ATM4,TRX-M4	thioredoxin M-type 4	thioredoxin M-type protein
Sapur.013G129200.3	51.51692714	-1.045917682	0.073373524	-14.2547	4.19E-46	2.15E-45	cyan	-0.753109077	5.51E-30	GO:0016740,GO:0008152,GO:0005542	AT2G20830.1		transferases;folic acid binding	formiminotransferase-cyclodeaminase, putative
Sapur.002G013800.2	20.10341007	-1.124832801	0.078916067	-14.2535	4.26E-46	2.18E-45	cyan	-0.731835435	1.33E-27	GO:0007264,GO:0005525	AT1G75840.1	ARAC5.ATGP3.ATROP4.ROP	RAC-like GTP binding protein 5	Ras family small GTPase
Sapur.003G107800.1	691.7899156	0.489084557	0.034325909	14.24826	4.60E-46	2.36E-45	cyan	0.752638392	6.26E-30	GO:0006468,GO:0005524,GO:0004672	AT5G41990.1	ATWNK8,WNK8	with no lysine (K) kinase 8	Serine/Threonine kinase WNK8
Sapur.006G103000.1	1667.728863	0.560725102	0.039364639	14.24439	4.86E-46	2.49E-45	cyan	0.756225436	2.35E-30		AT5G03345.1			membrane magnesium transporter
Sapur.007G126800.1	34.45885652	-1.771779756	0.124386255	-14.2442	4.87E-46	2.50E-45	cyan	-0.731925768	1.30E-27	GO:0016758,GO:0008152	AT2G43820.1	ATSAGT1.GT.SAGT1.SGT1.U	UDP-glucosyltransferase 74F2	NA
Sapur.016G068600.1	44.60179929	1.640772562	0.115230681	14.23903	5.25E-46	2.69E-45	cyan	0.643590825	9.86E-20		AT1G52340.1	ABA2.ATABA2.ATSDR1.GIN1	NAD(P)-binding Rossmann-fold superfamily protein	short chain alcohol dehydrogenase, putative
Sapur.014G120800.6	32.42291606	-1.234932623	0.086739243	-14.2373	5.38E-46	2.75E-45	cyan	-0.729997909	2.09E-27	GO:0003676	AT3G23700.1		Nucleic acid-binding proteins superfamily	S1 RNA-binding domain protein, putative
Sapur.008G044100.1	96.93881256	-0.750676284	0.052730135	-14.2362	5.46E-46	2.80E-45	cyan	-0.746157595	3.52E-29	GO:0006468,GO:0004672	AT1G07570.1	APK1,APK1A	Protein kinase superfamily protein	kinase domain protein
Sapur.003G143500.1	6.621578452	-2.041483258	0.143408313	-14.2355	5.52E-46	2.82E-45	cyan	-0.740286806	1.61E-28	GO:0008146	AT5G07000.1	AST2B,ST2B	sulfotransferase 2B	flavonol 4'-sulfotransferase, putative
Sapur.15WG141400.1	520.9538387	0.605705465	0.042562711	14.23089	5.89E-46	3.01E-45	cyan	0.764235584	2.49E-31	GO:0005515	AT5G62200.1		Embryo-specific protein 3, (ATS3)	embryo-specific protein
Sapur.016G065000.1	300.1046231	0.368897596	0.025927181	14.22822	6.12E-46	3.13E-45	cyan	0.767240057	1.05E-31		AT3G09180.1			mediator of RNA polymerase II transcription subunit 27
Sapur.016G255100.1	1081.241	-0.744302776	0.052320117	-14.2259	6.32E-46	3.23E-45	cyan	-0.750799778	1.03E-29	GO:0006412,GO:0005840,GO:0005622,GO:0003735,GO:0003723	AT3G15190.1		chloroplast 30S ribosomal protein S20, putative	30S ribosomal protein S20
Sapur.019G015500.1	858.1601381	-0.953212558	0.067007276	-14.2255	6.36E-46	3.25E-45	cyan	-0.737649825	3.14E-28	GO:0016829,GO:0010333,GO:0008152,GO:0000287	AT4G16740.1	ATTPS03,TPS03	terpene synthase 03	beta-pinene synthase
Sapur.012G088600.1	65.88707268	-1.179605431	0.082930265	-14.2241	6.50E-46	3.32E-45	cyan	-0.762413104	4.18E-31	GO:0006355,GO:0005739,GO:0003690	AT4G19650.1		Mitochondrial transcription termination factor family protein	transcription termination factor-like protein, putative
Sapur.005G001100.1	50.45534401	-1.076031155	0.075651883	-14.2235	6.55E-46	3.35E-45	cyan	-0.764876363	2.07E-31					NA
Sapur.016G076300.1	10.54046785	-1.817371281	0.127793069	-14.2212	6.77E-46	3.46E-45	cyan	-0.776670459	6.34E-33		AT5G02640.1			hypothetical protein
Sapur.003G115900.1	16.82975112	2.020004735	0.142054837	14.21989	6.90E-46	3.52E-45	cyan	0.646937438	5.53E-20	GO:0016021,GO:0005215	AT1G30840.2	ATPUP4,PUP4	purine permease 4	triose-phosphate transporter family protein
Sapur.T168000.1	7.345597987	-1.675856812	0.117860493	-14.219	6.99E-46	3.57E-45	cyan	-0.712303813	1.32E-25	GO:0006284	AT3G07930.3		DNA glycosylase superfamily protein	NA
Sapur.010G054000.1	126.8183813	-0.569088447	0.040025983	-14.218	7.09E-46	3.62E-45	cyan	-0.749599998	1.42E-29		AT3G04130.1		Tetratricopeptide repeat (TPR)-like superfamily protein	pentatricopeptide (PPR) repeat protein, putative
Sapur.013G057900.1	476.434897	0.641008755	0.045091834	14.21563	7.33E-46	3.74E-45	cyan	0.716889603	4.65E-26		AT2G36900.1	ATMEMB11,MEMB11	membrin 11	membrin, putative
Sapur.016G005600.1	40.71399495	-1.485224166	0.104481034	-14.2153	7.37E-46	3.76E-45	cyan	-0.692208002	1.02E-23		AT5G47635.1		Pollen Ole e 1 allergen and extensin family protein	pollen protein Ole E I-like protein
Sapur.018G069900.1	466.0707088	0.492182143	0.034634818	14.21062	7.87E-46	4.01E-45	cyan	0.725535968	6.13E-27	GO:0016020	AT2G18840.1		Integral membrane Yip1 family protein	NA
Sapur.013G013000.1	4230.984029	1.511480565	0.106367554	14.20998	7.95E-46	4.05E-45	cyan	0.684511986	4.90E-23		AT4G15920.1		Nodulin MtN3 family protein	bidirectional sugar transporter SWEET16
Sapur.010G057200.1	62.20507316	1.071293825	0.07539668	14.20877	8.08E-46	4.12E-45	cyan	0.693097833	8.47E-24	GO:0016747	AT3G26040.1		HXXXD-type acyl-transferase family protein	3'-N-debenzoyl-2'-deoxytaxol N-benzoyltransferase, putative
Sapur.016G088800.1	384.8284963	-0.811298335	0.057117859	-14.2039	8.66E-46	4.41E-45	cyan	-0.732344378	1.18E-27		AT3G10130.1		SOUL heme-binding family protein	SOUL heme-binding protein
Sapur.016G015400.1	3729.494205	0.897907815	0.063223599	14.2021	8.89E-46	4.53E-45	cyan	0.750943006	9.89E-30		AT2G32240.1			Uro-adherence factor A, putative
Sapur.003G040500.1	34.1396751	-1.192295733	0.083980745	-14.1973	9.53E-46	4.85E-45	cyan	-0.754211467	4.09E-30		AT1G54050.1		HSP20-like chaperones superfamily protein	small heat shock protein
Sapur.008G108700.1	499.1981732	0.546654087	0.038514122	14.1936	1.00E-45	5.11E-45	cyan	0.7172468	4.28E-26	GO:0005515	AT2G01650.1	PUX2	plant UBX domain-containing protein 2	CDC48-interacting UBX-domain protein
Sapur.014G068100.1	86.19149119	1.435239135	0.101128608	14.19222	1.02E-45	5.21E-45	cyan	0.636885005	3.08E-19	GO:0055114,GO:0016491	AT1G01580.1	ATFRO2,FRD1,FRO2	ferric reduction oxidase 2	NA
Sapur.014G005900.1	125.3047644	-1.369621302	0.096510843	-14.1914	1.04E-45	5.27E-45	cyan	-0.745029239	4.73E-29		AT1G27480.1		alpha/beta-Hydrolases superfamily protein	lecithin-cholesterol acyltransferase-like protein
Sapur.016G226600.1	3361.450408	1.339210162	0.094426671	14.18254	1.18E-45	5.98E-45	cyan	0.696670348	4.00E-24	GO:0016887,GO:0005524,GO:0055085,GO:0042626,GO:0016021,GO:0006810	AT4G18050.1	PGP9	P-glycoprotein 9	NA
Sapur.007G091300.1	18.93120391	-1.145035747	0.080767721	-14.1769	1.27E-45	6.47E-45	cyan	-0.741624922	1.14E-28	GO:0055114,GO:0016491,GO:0001735	AT5G63910.1	FCLY	farnesylcysteine lyase	NA
Sapur.014G012800.1	427.5379234	1.202675102	0.084895055	14.16661	1.47E-45	7.49E-45	cyan	0.696382261	4.25E-24		AT3G53010.1		Domain of unknown function (DUF303)	carbohydrate esterase family 6 protein
Sapur.002G184500.1	1700.368306	1.251180232	0.08832208	14.16611	1.49E-45	7.54E-45	cyan	0.761937471	4.79E-31		AT4G13710.1		Pectin lyase-like superfamily protein	pectate lyase
Sapur.010G110600.2	41.55663551	2.64232749	0.186543466	14.16467	1.52E-45	7.70E-45	cyan	0.565300619	1.23E-14	GO:0005975,GO:0004553	AT1G02640.1	ATBXL2,BXL2	beta-xylosidase 2	alpha-L-arabinofuranosidase/beta-D-xylosidase
Sapur.005G096700.1	247.3395212	1.142863817	0.080731063	14.15643	1.70E-45	8.65E-45	cyan	0.638526555	2.33E-19	GO:0005975,GO:0004553	AT5G66460.1		Glycosyl hydrolase superfamily protein	mannan endo-1%2C4-beta-mannosidase
Sapur.014G018700.1	18.30385736	2.029638004	0.143405397	14.15315	1.79E-45	9.06E-45	cyan	0.654039935	1.58E-20		AT2G26570.1		Plant protein of unknown function (DUF827)	WEAK movement UNDER BLUE LIGHT protein
Sapur.004G068000.1	451.8910024	1.552425326	0.109713266	14.14984	1.87E-45	9.49E-45	cyan	0.703752325	8.77E-25		AT3G18280.1		Bifunctional inhibitor/lipid-transfer protein/seed storage 2S albumin superfamily protein	Non-specific lipid-transfer protein type 2 subfamily protein, putative
Sapur.002G198900.1	249.1431627	1.220316612	0.086273289	14.14478	2.01E-45	1.02E-44	cyan	0.668214228	1.17E-21		AT2G20320.1		DENN (AEX-3) domain-containing protein	DENN (AEX-3) domain protein
Sapur.006G056800.1	153.4396083	-1.173754779	0.08298474	-14.1442	2.03E-45	1.03E-44	cyan	-0.783260932	8.22E-34	GO:0016042,GO:0005509,GO:0004623	AT2G06925.1	ATSPLA2-ALPHA.PLA2-ALPH	Phospholipase A2 family protein	NA
Sapur.008G084300.1	63.56788407	1.766364027	0.124887932	14.14359	2.05E-45	1.04E-44	cyan	0.615751145	9.31E-18		AT3G01420.1	ALPHA-DOX1.DIOX1.DOX1.F	Peroxidase superfamily protein	NA
Sapur.007G003200.1	1903.505527	0.557729173	0.039445289	14.13931	2.17E-45	1.10E-44	cyan	0.7261714	5.26E-27	GO:0006508,GO:0004222	AT5G65620.1		Zincin-like metalloproteases family protein	peptidase M3A family protein
Sapur.008G141400.1	2078.142041	0.735207727	0.052004788	14.13731	2.24E-45	1.13E-44	cyan	0.732039533	1.27E-27	GO:0016161,GO:0000272	AT3G23920.1	BAM1,BMY7,TR-BAMY	beta-amylase 1	beta-amylase
Sapur.008G053500.1	18.80050885	-1.287380491	0.091078951	-14.1348	2.32E-45	1.17E-44	cyan	-0.759706189	8.97E-31					NA
Sapur.006G173100.1	113.3001172	-1.195520512	0.084580856	-14.1346	2.32E-45	1.18E-44	cyan	-0.829932845	3.96E-41	GO:0006614,GO:0005525,GO:0048500,GO:0008312	AT5G03940.1	54CP.CPSRP54.FFC.SRP54CF	chloroplast signal recognition particle 54 kDa subunit	signal recognition particle 54 kDa protein
Sapur.013G002000.1	27.18654548	-0.97017386	0.0686426	-14.1337	2.35E-45	1.19E-44	cyan	-0.761110801	6.04E-31		AT1G57540.1			hypothetical protein
Sapur.010G052600.1	4492.27603	0.54021779	0.038237262	14.12805	2.55E-45	1.29E-44	cyan	0.725437716	6.27E-27	GO:0051539	AT5G08530.1	CI51	51 kDa subunit of complex I	NADH-ubiquinone oxidoreductase flavoprotein
Sapur.T080200.1	196.097118	0.738785931	0.052323068	14.1197	2.87E-45	1.45E-44	cyan	0.713799334	9.41E-26		AT5G42870.1	ATPAH2,PAH2	phosphatidic acid phosphohydrolase 2	NA
Sapur.006G168800.1	325.6110603	0.570283231	0.040392219	14.11864	2.92E-45	1.47E-44	cyan	0.758430389	1.28E-30	GO:0008270,GO:0003676	AT2G37340.1	ATRSZ33,RSZ33	arginine/serine-rich zinc knuckle-containing protein 33	serine/arginine-rich splicing factor SRSF10 RNA recognition motif protein, putative
Sapur.003G065200.1	7.948497709	2.12981226	0.150871414	14.11674	3.00E-45	1.51E-44	cyan	0.720705489	1.92E-26	GO:0043531	AT3G07040.1	RPM1,RPS3	NB-ARC domain-containing disease resistance protein	NBS-LRR type disease resistance protein
Sapur.005G164200.1	199.7029392	0.663444805	0.046998915	14.11617	3.02E-45	1.53E-44	cyan	0.7581406	1.39E-30		AT2G27310.1		F-box family protein	F-box GID2 protein, putative
Sapur.014G100400.1	2.275542405	4.246244623	0.300955678	14.1092	3.33E-45	1.68E-44	cyan	0.630545201	8.79E-19					NA
Sapur.006G150900.1	184.1914156	-0.488697326	0.034649393	-14.1041	3.59E-45	1.81E-44	cyan	-0.74187686	1.07E-28		AT5G57950.1		26S proteasome regulatory subunit, putative	26S proteasome non-ATPase regulatory subunit, putative
Sapur.016G256900.1	1010.89284	-0.713665392	0.050612889	-14.1005	3.77E-45	1.90E-44	cyan	-0.739217411	2.11E-28	GO:0009055	AT5G53870.1	AtENODL1,ENODL1	early nodulin-like protein 1	plastocyanin-like domain protein
Sapur.008G106800.1	149.4882255	1.086897543	0.0771247	14.09273	4.21E-45	2.12E-44	cyan	0.737345607	3.39E-28		AT1G23149.1	CPuORF29	conserved peptide upstream open reading frame 29	NA
Sapur.008G070200.1	25.66578259	2.042906419	0.14499469	14.08953	4.41E-45	2.22E-44	cyan	0.628840089	1.16E-18	GO:0006355,GO:0003677	AT1G69490.1	ANAC029,ATNAP,NAP	NAC-like, activated by AP3/PI	NAC transcription factor
Sapur.011G057500.1	850.1350677	0.57302066	0.040679449	14.08624	4.61E-45	2.33E-44	cyan	0.72184649	1.47E-26	GO:0003676	AT5G46250.1		RNA-binding protein	NA

Sapur.014G071500.1	275.1482916	-0.559869829	0.039747777	-14.0856	4.66E-45	2.35E-44	cyan	-0.767930143	8.57E-32		AT2G46220.1		Uncharacterized conserved protein (DUF2358)	DUF2358 family protein
Sapur.016G284800.1	388.3570938	-0.649527769	0.046126855	-14.0813	4.95E-45	2.49E-44	cyan	-0.761651587	5.19E-31	GO:0009432,GO:0006281,GO:0005524,GO:0003697	AT1G79050.1		recA DNA recombination family protein	DNA repair protein RecA, putative
Sapur.T134200.1	8.05594294	3.727483816	0.264751846	14.07916	5.10E-45	2.57E-44	cyan	0.602681966	6.76E-17					NA
Sapur.004G072200.1	107.3251219	-0.872668691	0.061999152	-14.0755	5.37E-45	2.70E-44	cyan	-0.736467248	4.22E-28		AT5G39210.1	CRR7	chlororespiratory reduction 7	DUF3571 family protein
Sapur.017G116100.1	138.5690108	1.879577227	0.133622202	14.06635	6.11E-45	3.08E-44	cyan	0.685060614	4.39E-23	GO:0006468,GO:0005524,GO:0004672	AT5G40380.1	CRK42	cysteine-rich RLK (RECEPTOR-like protein kinase) 42	tyrosine kinase domain protein
Sapur.018G052600.1	357.5378514	-0.629961731	0.044785366	-14.0662	6.12E-45	3.08E-44	cyan	-0.755844852	2.61E-30		AT5G20140.1		SOUL heme-binding family protein	SOUL heme-binding protein
Sapur.004G024400.2	181.0702552	0.796159324	0.056617481	14.06208	6.50E-45	3.27E-44	cyan	0.725700217	5.89E-27	GO:0005524	AT1G61140.1	EDA16	SNF2 domain-containing protein / helicase domain-containing protein / zinc finger protein-related	chromatin remodeling complex subunit
Sapur.008G080500.1	82.53955893	1.235103531	0.087843125	14.06033	6.66E-45	3.35E-44	cyan	0.738217511	2.72E-28		AT5G49580.1		Chaperone DnaJ-domain superfamily protein	NA
Sapur.009G089500.1	866.2132839	0.501369755	0.035665552	14.05754	6.93E-45	3.48E-44	cyan	0.755075639	3.23E-30		AT1G19360.1		Nucleotide-diphospho-sugar transferase family protein	nucleotide-diphospho-sugar transferase
Sapur.016G231600.1	614.0161024	-0.517575983	0.036825733	-14.0547	7.20E-45	3.62E-44	cyan	-0.770828083	3.66E-32	GO:0055085,GO:0046873,GO:0030001,GO:0016020	AT1G55910.1	ZIP11	zinc transporter 11 precursor	metal cation transporter, ZIP family protein
Sapur.009G071700.1	43.92897726	-1.029108485	0.073228025	-14.0535	7.33E-45	3.68E-44	cyan	-0.753273651	5.27E-30		AT4G33925.1			zinc finger SWIM domain protein
Sapur.007G028200.1	773.881634	0.668835093	0.047593103	14.05319	7.36E-45	3.70E-44	cyan	0.728011179	3.38E-27	GO:0008299	AT4G36810.1	GGPS1	geranylgeranyl pyrophosphate synthase 1	geranylgeranyl pyrophosphate synthase
Sapur.004G041800.1	71.3708791	1.464031789	0.104179622	14.05296	7.39E-45	3.71E-44	cyan	0.668675983	1.07E-21		AT2G42760.1			DUF1685 family protein
Sapur.016G246900.1	2656.923656	-0.657216533	0.04677149	-14.0516	7.53E-45	3.78E-44	cyan	-0.789893879	9.77E-35	GO:0006412,GO:0005840,GO:0003735	AT1G78630.1	emb1473	Ribosomal protein L13 family protein	50S ribosomal protein L13
Sapur.010G193500.1	5.187704732	4.861972837	0.346060185	14.0495	7.76E-45	3.89E-44	cyan	0.548805173	9.96E-14	GO:0046983,GO:0003677	AT3G18650.1	AGL103	AGAMOUS-like 103	SRF-type transcription factor (DNA-binding and dimerization domain) protein
Sapur.017G119000.1	542.2816264	-0.429386619	0.030565073	-14.0483	7.89E-45	3.96E-44	cyan	-0.737722396	3.08E-28		AT5G62650.1		Tic22-like family protein	plant/MRG21-7 protein, putative
Sapur.007G077500.1	460.5199842	-0.878620988	0.062552901	-14.046	8.15E-45	4.08E-44	cyan	-0.744620947	5.26E-29	GO:0016758,GO:0008152	AT2G43820.1	ATSAGT1.GT.SAGT1.SGT1.U	UDP-glucosyltransferase 74F2	UDP-glucosyltransferase, putative
Sapur.007G074700.1	5138.515119	0.483786108	0.034448126	14.0439	8.40E-45	4.21E-44	cyan	0.731153271	1.58E-27		AT2G21870.1	MGP1	copper ion binding;cobalt ion binding;zinc ion binding	ATP synthase
Sapur.009G008000.1	590.2387402	0.944663844	0.067296722	14.03729	9.22E-45	4.62E-44	cyan	0.682148565	7.87E-23		AT2G28400.1		Protein of unknown function, DUF584	senescence regulator
Sapur.019G082300.1	29.17249777	-0.871295432	0.062111522	-14.0279	1.05E-44	5.27E-44	cyan	-0.738970035	2.25E-28	GO:0005515	AT1G34420.1		leucine-rich repeat transmembrane protein kinase family protein	LRR receptor-like kinase, putative
Sapur.016G265700.1	3285.665833	-0.808540896	0.057654078	-14.024	1.11E-44	5.57E-44	cyan	-0.744125754	5.98E-29	GO:0016020,GO:0009765	AT5G54270.1	LHCB3,LHCB3*1	light-harvesting chlorophyll B-binding protein 3	light-harvesting chlorophyll-A/B-binding protein Lhcb3
Sapur.013G043100.1	53.67758502	-1.378099254	0.098271537	-14.0234	1.12E-44	5.61E-44	cyan	-0.74480701	5.01E-29	GO:0016829,GO:0006520	AT1G08630.3	THA1	threonine aldolase 1	L-allo-threonine aldolase
Sapur.001G075100.1	5.773553271	4.007901397	0.285864139	14.0203	1.17E-44	5.86E-44	cyan	0.66670505	1.56E-21		AT4G23860.3		PHD finger protein-related	NA
Sapur.011G046700.1	219.3240032	-0.953818448	0.068033249	-14.0199	1.18E-44	5.89E-44	cyan	-0.790370891	8.36E-35		AT1G29240.1		Protein of unknown function (DUF688)	DUF688 family protein
Sapur.004G162100.1	356.3474332	-0.876036976	0.062493836	-14.018	1.21E-44	6.05E-44	cyan	-0.791675191	5.44E-35	GO:0046872,GO:0030001	AT5G60800.2		Heavy metal transport/detoxification superfamily protein	heavy metal-associated domain protein
Sapur.013G134800.1	51.53465228	-2.42034958	0.172673541	-14.0169	1.23E-44	6.14E-44	cyan	-0.752039024	7.36E-30		AT5G54510.1	DFL1,GH3.6	Auxin-responsive GH3 family protein	indole-3-acetic acid-amido synthetase GH3.6 protein, putative
Sapur.009G028300.1	142.5773341	-0.879166189	0.062729059	-14.0153	1.26E-44	6.28E-44	cyan	-0.792895722	3.63E-35		AT3G46870.1		Pentatricopeptide repeat (PPR) superfamily protein	PPR containing plant protein
Sapur.003G106900.1	33.82399129	-1.409376128	0.100584381	-14.0119	1.32E-44	6.59E-44	cyan	-0.727637636	3.70E-27		AT5G49330.1	ATMYB111,MYB111,PFG3	myb domain protein 111	R2R3 MYB transcription factor
Sapur.002G025000.1	139.2434576	-1.172680842	0.083716838	-14.0077	1.40E-44	6.98E-44	cyan	-0.674489346	3.54E-22	GO:0046872,GO:0030001	AT1G01490.2		Heavy metal transport/detoxification superfamily protein	NA
Sapur.15WG081000.3	41.92354442	1.109090531	0.079232925	13.99785	1.61E-44	8.02E-44	cyan	0.74012958	1.67E-28	GO:0005515,GO:0008017	AT5G62500.1	ATEB1,ATEB1B,EB1B	end binding protein 1B	microtubule-associated protein RP/EB family protein
Sapur.001G175000.1	263.0266033	0.52003113	0.037170208	13.99054	1.78E-44	8.88E-44	cyan	0.732965842	1.01E-27	GO:0016811,GO:0016021,GO:0006672	AT1G71190.1	SAG18	senescence associated gene 18	senescence associated prorein 18
Sapur.016G060700.1	223.2931487	-0.550563105	0.039361311	-13.9874	1.86E-44	9.28E-44	cyan	-0.757184516	1.81E-30	GO:0005515	AT5G06550.1			transferring glycosyl group transferase, putative
Sapur.007G081800.1	298.2262537	0.943541747	0.06745971	13.98674	1.88E-44	9.36E-44	cyan	0.71430849	8.39E-26		AT1G78020.1		Protein of unknown function (DUF581)	DUF581 family protein
Sapur.017G110500.1	324.4741001	-0.491778292	0.035167309	-13.984	1.95E-44	9.73E-44	cyan	-0.748008	2.16E-29	GO:0043531	AT3G14460.1		LRR and NB-ARC domains-containing disease resistance protein	NA
Sapur.010G007200.1	117.7507543	0.874902583	0.062618167	13.97202	2.31E-44	1.15E-43	cyan	0.70608729	5.27E-25	GO:0016746,GO:0008152	AT3G06850.2	BCE2,DIN3,LTA1	2-oxoacid dehydrogenases acyltransferase family protein	lipoamide acyltransferase component of branched-chain alpha-keto acid dehydrogenase, putative
Sapur.016G003000.1	7.115788404	2.044066816	0.146300737	13.97168	2.32E-44	1.15E-43	cyan	0.690488674	1.45E-23		AT4G26570.1	ATCBL3,CBL3	calcineurin B-like 3	calcineurin B-like protein
Sapur.001G001200.1	1838.425364	0.589992957	0.0422327	13.97005	2.37E-44	1.18E-43	cyan	0.725914875	5.60E-27	GO:0030955,GO:0006096,GO:0004743,GO:0000287	AT5G56350.1		Pyruvate kinase family protein	pyruvate kinase
Sapur.002G126900.1	17.17798711	2.368563973	0.169600314	13.96556	2.53E-44	1.26E-43	cyan	0.631278091	7.80E-19	GO:0005515	AT2G45760.1	BAL,BAP2	BON association protein 2	C2 domain protein
Sapur.018G108900.1	176.6306408	0.724291581	0.051867597	13.96424	2.58E-44	1.28E-43	cyan	0.699444089	2.22E-24	GO:0005515	AT2G19260.1		RING/FYVE/PHD zinc finger superfamily protein	PHD-finger protein
Sapur.15WG149200.1	790.7341504	-0.460099247	0.03295823	-13.9601	2.73E-44	1.36E-43	cyan	-0.747617727	2.40E-29	GO:0006378,GO:0005849,GO:0003729	AT4G25550.1		Cleavage/polyadenylation specificity factor, 25kDa subunit	cleavage and polyadenylation specificity factor 5
Sapur.016G067500.1	97.11960512	0.933359094	0.066871997	13.9574	2.84E-44	1.41E-43	cyan	0.687110297	2.90E-23		AT5G06280.1			hypothetical protein
Sapur.001G116700.1	21.44930342	-1.85606822	0.133044819	-13.9507	3.12E-44	1.55E-43	cyan	-0.768865403	6.52E-32	GO:0046983	AT1G72210.1		basic helix-loop-helix (bHLH) DNA-binding superfamily protein	transcription factor bHLH96
Sapur.008G055200.1	87.69856919	-1.319800503	0.094616048	-13.949	3.19E-44	1.58E-43	cyan	-0.815921092	1.03E-38		AT3G11150.1		2-oxoglutarate (2OG) and Fe(II)-dependent oxygenase superfamily protein	2-oxoglutarate-dependent dioxygenase
Sapur.005G166400.1	422.0538171	-0.615780967	0.04415795	-13.945	3.38E-44	1.68E-43	cyan	-0.76663194	1.25E-31		AT4G02725.1			transmembrane protein, putative
Sapur.011G110700.1	19.70277181	1.346512833	0.096563192	13.94437	3.40E-44	1.69E-43	cyan	0.721638189	1.54E-26		AT1G33055.1			hypothetical protein
Sapur.001G023400.1	3079.699462	-1.062977751	0.076263705	-13.9382	3.71E-44	1.84E-43	cyan	-0.778551772	3.56E-33	GO:0006508,GO:0004190	AT3G54400.1		Eukaryotic aspartyl protease family protein	aspartic proteinase nepenthesin-1 protein
Sapur.004G097100.3	45.21885622	-0.95537148	0.068554729	-13.9359	3.83E-44	1.90E-43	cyan	-0.757584591	1.62E-30		AT5G38360.1		alpha/beta-Hydrolases superfamily protein	alpha/beta hydrolase family protein
Sapur.T134600.1	18.7825242	1.928337015	0.138375594	13.93553	3.85E-44	1.91E-43	cyan	0.649501888	3.53E-20					NA
Sapur.009G128000.1	47.99212573	1.20335291	0.086352818	13.93531	3.87E-44	1.92E-43	cyan	0.711091996	1.73E-25	GO:0005509	AT2G27480.1		Calcium-binding EF-hand family protein	EF-hand calcium-binding protein, putative
Sapur.002G118300.1	189.6936599	-0.848670969	0.060912975	-13.9325	4.02E-44	1.99E-43	cyan	-0.794135434	2.40E-35	GO:0007275	AT2G45190.1	AFO,FIL,YAB1	Plant-specific transcription factor YABBY family protein	YABBY transcription factor
Sapur.006G041900.1	305.1384759	0.455310749	0.032681242	13.93187	4.06E-44	2.01E-43	cyan	0.751571025	8.35E-30		AT5G03780.1	TRFL10	TRF-like 10	NA
Sapur.010G179900.1	75.34190547	-1.006305063	0.072235251	-13.9309	4.11E-44	2.04E-43	cyan	-0.754949658	3.34E-30	GO:0008080	AT2G39020.1		Acyl-CoA N-acyltransferases (NAT) superfamily protein	NA
Sapur.001G185300.2	20.52375107	-1.321222247	0.094876772	-13.9257	4.42E-44	2.19E-43	cyan	-0.718664095	3.08E-26		AT1G10270.1	GRP23	glutamine-rich protein 23	PPR containing plant protein
Sapur.012G107900.1	18.23647619	-0.984303259	0.070684938	-13.9252	4.45E-44	2.20E-43	cyan	-0.751853266	7.74E-30		AT3G29230.1		Tetratricopeptide repeat (TPR)-like superfamily protein	PPR containing plant protein
Sapur.016G250500.1	504.2842446	-0.568860877	0.040860407	-13.9221	4.65E-44	2.30E-43	cyan	-0.758700512	1.19E-30	GO:0006779,GO:0004853	AT3G14930.2	HEME1	Uroporphyrinogen decarboxylase	uroporphyrinogen decarboxylase
Sapur.010G093800.1	31.15057159	-1.890090595	0.135765002	-13.9218	4.67E-44	2.31E-43	cyan	-0.713432284	1.02E-25		AT1G68330.1			hypothetical protein
Sapur.014G080600.1	1038.678951	-0.63710798	0.045780851	-13.9165	5.03E-44	2.49E-43	cyan	-0.775286371	9.65E-33		AT3G61870.1			plant/F21F14-40 protein, putative
Sapur.011G049800.1	55.56096789	-0.990250747	0.071182442	-13.9114	5.40E-44	2.67E-43	cyan	-0.784217089	6.07E-34		AT1G29300.1	UNE1	Plant protein of unknown function (DUF641)	UNE1-like protein
Sapur.009G054000.1	117.7149779	-0.629935135	0.04528922	-13.9092	5.57E-44	2.75E-43	cyan	-0.745748856	3.92E-29		AT1G62350.1		Pentatricopeptide repeat (PPR) superfamily protein	PPR containing plant protein
Sapur.006G112000.1	452.237939	0.619485248	0.044540744	13.90828	5.64E-44	2.79E-43	cyan	0.71702825	4.50E-26	GO:0046872	AT3G43230.1		RING/FYVE/PHD-type zinc finger family protein	zinc finger protein
Sapur.004G161300.1	58.88392369	-1.17877449	0.084763016	-13.9067	5.77E-44	2.85E-43	cyan	-0.703246709	9.79E-25					NA
Sapur.009G126300.1	45.44012109	-1.876211115	0.134983775	-13.8995	6.38E-44	3.15E-43	cyan	-0.813900706	2.20E-38		AT5G04820.1	ATOFP13,OFP13	ovate family protein 13	ovate transcriptional repressor
Sapur.004G112900.1	120.5415181	-1.305651284	0.093935438	-13.8995	6.38E-44	3.15E-43	cyan	-0.74032713	1.59E-28		AT4G34360.1		S-adenosyl-L-methionine-dependent methyltransferases superfamily protein	S-adenosylmethionine-dependent methyltransferase, putative
Sapur.017G101800.1	33.94098025	-1.149671159	0.082753884	-13.8927	7.02E-44	3.46E-43	cyan	-0.744073317	6.06E-29	GO:0007275,GO:0006511,GO:0005634	AT5G37930.1		Protein with RING/U-box and TRAF-like domains	NA
Sapur.014G041500.1	372.5429714	1.678320363	0.120830218	13.88991	7.29E-44	3.60E-43	cyan	0.639377587	2.02E-19		AT3G60450.1		Phosphoglycerate mutase family protein	NA
Sapur.016G104400.1	2210.468817	-0.488709235	0.03518522	-13.8896	7.32E-44	3.61E-43	cyan	-0.742046599	1.02E-28		AT3G51800.3	ATEBP1,ATG2,EBP1	metallopeptidase M24 family protein	proliferation-associated protein 2g4
Sapur.007G122600.1	33.0247444	-1.689189466	0.121701013	-13.8798	8.39E-44	4.14E-43	cyan	-0.666671516	1.57E-21		AT4G28450.1		nucleotide binding;protein binding	NA
Sapur.006G004800.1	1235.111507	-0.997343639	0.071894333	-13.8724	9.32E-44	4.59E-43	cyan	-0.738980672	2.24E-28	GO:0046872,GO:0030001	AT5G50740.3		Heavy metal transport/detoxification superfamily protein	heavy metal-associated domain protein
Sapur.002G190100.1	533.842001	-0.616329741	0.044430123	-13.8719	9.38E-44	4.62E-43	cyan	-0.810586433	7.52E-38	GO:0016021,GO:0006886,GO:0005783	AT3G07190.1		B-cell receptor-associated protein 31-like	B-cell receptor-associated protein-like containing protein
Sapur.004G043000.1	24.83464433	-1.135718059	0.081875473	-13.8713	9.46E-44	4.65E-43	cyan	-0.769296877	5.75E-32					NA
Sapur.008G036400.1	20.7255303	-1.236513863	0.089158233	-13.8688	9.80E-44	4.82E-43	cyan	-0.740236791	1.63E-28		AT3G11591.1			CC-NBS resistance protein, putative
Sapur.006G100000.1	12096.18476	0.930777903	0.067126535	13.86602	1.02E-43	5.00E-43	cyan	0.725213186	6.62E-27	GO:0016020,GO:0006810,GO:0005215	AT2G36830.1	GAMMA-TIP.GAMMA-TIP1.	gamma tonoplast intrinsic protein	aquaporin, MIP family%2C TIP subfamily protein
Sapur.014G021400.1	12.32043836	1.540058797	0.111079211	13.86451	1.04E-43	5.11E-43	cyan	0.69482988	5.90E-24		AT3G24880.1		Helicase/SANT-associated, DNA binding protein	NA
Sapur.013G103200.1	544.3737626	0.719310139	0.051892895	13.86144	1.08E-43	5.33E-43	cyan	0.693445347	7.88E-24		AT4G38620.1	ATMYB4,MYB4	myb domain protein 4	MYB transcription factor 3, putative
Sapur.016G192300.1	836.8565708	0.374727941	0.027038603	13.859	1.12E-43	5.51E-43	cyan	0.78367687	7.21E-34	GO:0046923,GO:0016021,GO:0006621	AT3G25040.1	ERD2B	endoplasmic reticulum retention defective 2B	ER lumen protein retaining receptor
Sapur.012G040400.1	372.4292115	-0.871449624	0.062895613	-13.8555	1.18E-43	5.79E-43	cyan	-0.730509371	1.84E-27	GO:0006457,GO:0003755,GO:0000413	AT1G74070.1		Cyclophilin-like peptidyl-prolyl cis-trans isomerase family protein	peptidyl-prolyl cis-trans isomerase, cyclophilin-type protein
Sapur.003G087400.1	255.6589147	1.513795004	0.109275961	13.85296	1.22E-43	5.99E-43	cyan	0.681469124	9.01E-23	GO:0016021,GO:0004252,GO:0006508	AT1G63120.1	ATRBL2,RBL2	RHOMBOID-like 2	NA
Sapur.002G164800.1	145.1512024	1.929601195	0.139343033	13.84785	1.31E-43	6.43E-43	cyan	0.577300984	2.48E-15		AT4G01970.1	AtSTS,STS	stachyose synthase	galactinol-raffinose galactosyltransferase
Sapur.002G021800.2	8.548340727	-2.192723909	0.15845364	-13.8383	1.50E-43	7.35E-43	cyan	-0.733136816	9.67E-28	GO:0016021,GO:0016020,GO:0022857	AT1G75500.1	WAT1	Walls Are Thin 1	auxin-induced protein 5NG4
Sapur.002G053900.1	825.8042367	0.441344237	0.031896437	13.83679	1.53E-43	7.50E-43	cyan	0.742755739	8.52E-29		AT1G43690.1		ubiquitin interaction motif-containing protein	plant/F2J6-4 protein
Sapur.005G000300.1	66.66964086	1.007075327	0.072791309	13.8351	1.57E-43	7.67E-43	cyan	0.664882951	2.19E-21		AT5G26731.1			hypothetical protein
Sapur.005G201100.3	16.37162317	-1.273069971	0.092054621	-13.8295	1.69E-43	8.29E-43	cyan	-0.494999135	4.40E-11	GO:0043169,GO:0005975,GO:0003824,GO:0004553	AT2G36390.1	BE3,SBE2.1	starch branching enzyme 2.1	starch branching enzyme I
Sapur.002G120500.1	8.969004865	-2.097814233	0.151720573	-13.8268	1.76E-43	8.60E-43	cyan	-0.677419805	2.00E-22		AT2G45360.1		Protein of unknown function (DUF1442)	NAD(P)H-quinone oxidoreductase subunit 6
Sapur.008G139100.1	131.1449918	0.99324436	0.071836909	13.82638	1.77E-43	8.65E-43	cyan	0.663469432	2.84E-21		AT5G13890.1		Family of unknown function (DUF716)	transmembrane protein, putative
Sapur.002G201800.1	44.35453878	-1.091781421	0.07898493	-13.8227	1.86E-43	9.10E-43	cyan	-0.752296519	6.87E-30		AT1G06150.1	EMB1444	basic helix-loop-helix (bHLH) DNA-binding superfamily protein	NA
Sapur.012G091100.1	29.65724234	-1.313428788	0.095051572	-13.8181	1.98E-43	9.70E-43	cyan	-0.726344086	5.50E-27		AT2G35130.2		Tetratricopeptide repeat (TPR)-like superfamily protein	PPR containing plant protein
Sapur.15WG088600.1	592.8156942	-0.474471012	0.034343218	-13.8156	2.05E-43	1.00E-42	cyan	-0.744111796	6.00E-29	GO:0006629	AT3G48090.1	ATEDS1,EDS1	alpha/beta-Hydrolases superfamily protein	enhanced disease susceptibility protein
Sapur.011G058000.1	652.8542925	-0.52206149	0.037806108	-13.8089	2.25E-43	1.10E-42	cyan	-0.723454498	1.00E-26		AT2G34460.1		NAD(P)-binding Rossmann-fold superfamily protein	NAD-dependent epimerase/dehydratase family protein
Sapur.15WG146600.1	655.430662	-0.378821902	0.027454256	-13.7983	2.61E-43	1.27E-42	cyan	-0.749243764	1.56E-29	GO:0005634,GO:0003677	AT5G52230.2	MBD13	methyl-CPG-binding domain protein 13	methyl-binding domain protein, putative
Sapur.001G095300.1	5.072563551	-1.88733833	0.136780771	-13.7983	2.61E-43	1.28E-42	cyan	-0.726807616	4.52E-27		AT1G62870.1			hypothetical protein
Sapur.002G116400.1	4.722036249	-2.99400586	0.217010974	-13.7966	2.67E-43	1.30E-42	cyan	-0.681482083	8.98E-23	GO:0005524	AT3G20050.1	ATTCP-1,TCP-1	T-complex protein 1 alpha subunit	NA
Sapur.013G064100.1	6.288787558	2.373857104	0.172070842	13.79581	2.70E-43	1.32E-42	cyan	0.674310953	3.66E-22	GO:0006281,GO:0004518,GO:0006289,GO:0005634,GO:0004519,GO:0003697	AT3G28030.1	UVH3,UVR1	5\'-3\' exonuclease family protein	NA
Sapur.008G146100.1	284.4375288	0.560177755	0.040607044	13.79509	2.73E-43	1.33E-42	cyan	0.73940973	2.01E-28		AT3G27320.1		alpha/beta-Hydrolases superfamily protein	carboxylesterase, putative
Sapur.008G013800.1	93.47847387	0.646228769	0.046851531	13.79312	2.80E-43	1.37E-42	cyan	0.707444273	3.91E-25		AT2G16710.1		Iron-sulphur cluster biosynthesis family protein	iron-sulfur cluster assembly protein
Sapur.003G063600.1	340.315254	1.349414072	0.097868211	13.78807	3.01E-43	1.47E-42	cyan	0.702002361	1.28E-24	GO:0048046,GO:0016762,GO:0006073,GO:0005618,GO:0005975,GO:0004553	AT1G32170.1	XTH30,XTR4	xyloglucan endotransglucosylase/hydrolase 30	xyloglucan endotransglycosylase/hydrolase
Sapur.010G160500.1	8.193922933	3.082716041	0.223611216	13.78605	3.09E-43	1.51E-42	cyan	0.645724262	6.82E-20		AT3G12110.1	ACT11	actin-11	actin
Sapur.017G000500.1	46.41044204	-0.935296637	0.06784874	-13.785	3.14E-43	1.53E-42	cyan	-0.735087955	5.96E-28		AT4G21065.1		Tetratricopeptide repeat (TPR)-like superfamily protein	PPR containing plant protein
Sapur.15WG136700.1	96.11146837	1.589117769	0.115325648	13.7794	3.39E-43	1.65E-42	cyan	0.632450329	6.43E-19	GO:0005975,GO:0004553	AT1G23210.1	AtGH9B6,GH9B6	glycosyl hydrolase 9B6	endo-1%2C4-beta-glucanase
Sapur.013G068900.1	1.902144396	4.313208806	0.313115519	13.77514	3.60E-43	1.75E-42	cyan	0.594588374	2.21E-16	GO:0055114,GO:0020037,GO:0006979,GO:0004601	AT4G26010.1		Peroxidase superfamily protein	heme peroxidase, plant/fungal/bacterial
Sapur.013G063800.1	3.521866928	2.813215384	0.204275576	13.77167	3.77E-43	1.84E-42	cyan	0.694097396	6.87E-24					NA
Sapur.016G113200.5	39.07025329	-1.37356634	0.099741642	-13.7712	3.80E-43	1.85E-42	cyan	-0.689418967	1.81E-23	GO:0007275	AT1G08465.1	YAB2	Plant-specific transcription factor YABBY family protein	transcription factor YABBY2
Sapur.012G042100.1	6.273199298	-2.011249383	0.14611542	-13.7648	4.15E-43	2.02E-42	cyan	-0.728691659	2.87E-27		AT3G18550.2	ATTCP18,BRC1,TCP18	TCP family transcription factor	transcription factor TCP24
Sapur.005G056900.1	635.8959285	-0.651033989	0.047310449	-13.7609	4.38E-43	2.13E-42	cyan	-0.75084844	1.01E-29	GO:0051082,GO:0031072	AT2G22360.1		DNAJ heat shock family protein	chaperone protein DnaJ
Sapur.010G036600.1	242.4257964	-0.561416444	0.040809491	-13.757	4.62E-43	2.25E-42	cyan	-0.738283065	2.67E-28	GO:0006310,GO:0006302,GO:0005634,GO:0003677	AT3G23100.2	XRCC4	homolog of human DNA ligase iv-binding protein XRCC4	DNA double-strand break repair and V(D)J recombination protein XRCC4
Sapur.016G176600.1	45.80424471	-0.649779385	0.0472372	-13.7557	4.71E-43	2.29E-42	cyan	-0.73428324	7.28E-28	GO:0070461	AT4G33890.1			transcriptional regulator of RNA polII, SAGA, subunit
Sapur.018G080100.1	50.67709892	-1.399974328	0.101783962	-13.7544	4.79E-43	2.33E-42	cyan	-0.76370674	2.90E-31					hypothetical protein
Sapur.006G141400.1	845.2736932	-0.477063652	0.034689887	-13.7522	4.94E-43	2.40E-42	cyan	-0.740353514	1.58E-28	GO:0016020,GO:0015031	AT2G18710.1	SCY1	SECY homolog 1	preprotein translocase subunit SecY
Sapur.004G058200.1	2.803432143	4.29231116	0.312142653	13.75112	5.01E-43	2.44E-42	cyan	0.550385132	8.19E-14					NA
Sapur.005G184700.1	1177.905513	0.61780356	0.044931313	13.74996	5.10E-43	2.48E-42	cyan	0.752572367	6.38E-30		AT1G75388.1	CPuORF5	conserved peptide upstream open reading frame 5	peptide upstream ORF protein
Sapur.013G043300.1	34.03233755	-1.275555653	0.092795912	-13.7458	5.40E-43	2.62E-42	cyan	-0.700145693	1.91E-24		AT1G07090.1	LSH6	Protein of unknown function (DUF640)	light-dependent short hypocotyls protein, putative
Sapur.018G051500.1	1420.871016	0.447158099	0.032535502	13.7437	5.56E-43	2.70E-42	cyan	0.720183238	2.17E-26		AT3G25910.1		Protein of unknown function (DUF1644)	DUF1644 family protein
Sapur.009G070000.1	521.0268127	1.123968629	0.081807474	13.73919	5.91E-43	2.87E-42	cyan	0.674692807	3.40E-22		AT1G27730.1	STZ,ZAT10	salt tolerance zinc finger	C2H2-type zinc-finger protein, putative
Sapur.009G122700.2	24.07481056	-1.472044255	0.107146312	-13.7386	5.96E-43	2.89E-42	cyan	-0.731308325	1.52E-27	GO:0005515	AT2G21530.1		SMAD/FHA domain-containing protein	zeaxanthin epoxidase, putative
Sapur.009G041700.1	14.62478409	1.575255999	0.114660815	13.7384	5.98E-43	2.90E-42	cyan	0.715300866	6.69E-26		AT3G51970.1	ASAT1,ATASAT1,ATSAT1	acyl-CoA sterol acyl transferase 1	NA
Sapur.011G125500.1	742.2048748	-0.4574003	0.033296827	-13.7371	6.09E-43	2.95E-42	cyan	-0.753987551	4.34E-30	GO:0005515	AT5G56280.1	CSN6A	COP9 signalosome subunit 6A	COP9 signalosome complex subunit 6
Sapur.008G024700.1	209.2524485	-0.680849293	0.049574133	-13.734	6.36E-43	3.08E-42	cyan	-0.731609448	1.41E-27		AT3G10405.1			DUF3783 domain protein
Sapur.007G089000.1	13.68515147	1.894664935	0.13797249	13.73219	6.51E-43	3.16E-42	cyan	0.70574525	5.68E-25		AT5G63720.1	KPL	kokopelli	hypothetical protein
Sapur.019G110000.1	939.6372105	-0.957825329	0.069761023	-13.7301	6.70E-43	3.25E-42	cyan	-0.741438835	1.20E-28		AT1G06950.1	ATTIC110,TIC110	translocon at the inner envelope membrane of chloroplasts 110	inner envelope protein, putative
Sapur.002G075500.1	668.0828174	-0.819398994	0.059688823	-13.7278	6.92E-43	3.35E-42	cyan	-0.768981637	6.30E-32	GO:0005975,GO:0004553	AT1G78060.1		Glycosyl hydrolase family protein	xylan 1 4-beta-xylosidase
Sapur.016G157300.1	126.8086578	0.976226356	0.071123206	13.72585	7.11E-43	3.44E-42	cyan	0.719362138	2.62E-26	GO:0006468,GO:0005524,GO:0004672	AT3G45640.1	ATMAPK3,ATMPK3,MPK3	mitogen-activated protein kinase 3	MAP kinase
Sapur.T097800.1	4.348691812	2.782727948	0.202872217	13.71665	8.07E-43	3.91E-42	cyan	0.647122395	5.35E-20	GO:0005681,GO:0000398,GO:0000387	AT1G54385.1		ARM repeat superfamily protein	NA
Sapur.003G145600.1	64.42055455	-0.64654568	0.047137906	-13.716	8.14E-43	3.94E-42	cyan	-0.752658615	6.23E-30	GO:0006355,GO:0005739,GO:0003690	AT5G07900.1		Mitochondrial transcription termination factor family protein	mTERF protein
Sapur.001G150600.1	273.869567	0.455520664	0.033227756	13.70904	8.96E-43	4.33E-42	cyan	0.715381387	6.57E-26		AT1G17530.1	ATTIM23-1,TIM23-1	translocase of inner mitochondrial membrane 23	import inner membrane translocase subunit TIM23
Sapur.006G012700.1	557.6211644	-0.731250505	0.053341299	-13.7089	8.98E-43	4.34E-42	cyan	-0.797294499	8.28E-36		AT4G16410.1			transmembrane protein, putative
Sapur.007G038300.1	671.5270787	0.841878434	0.061413301	13.70841	9.04E-43	4.37E-42	cyan	0.726580537	4.77E-27		AT5G66820.1			transmembrane protein
Sapur.014G040900.1	417.4918341	-1.960461922	0.143015829	-13.708	9.09E-43	4.39E-42	cyan	-0.672386542	5.30E-22		AT3G60470.1		Plant protein of unknown function (DUF247)	transmembrane protein, putative
Sapur.010G121900.1	337.6475635	-1.197213025	0.087377765	-13.7016	9.93E-43	4.80E-42	cyan	-0.752641136	6.26E-30	GO:0030247	AT1G10380.1		Putative membrane lipoprotein	wall-associated receptor kinase galacturonan-binding protein
Sapur.006G148600.1	67.78478318	1.199373946	0.087562489	13.69735	1.05E-42	5.08E-42	cyan	0.6974415	3.40E-24					NA
Sapur.005G096100.1	6.379333164	3.89488132	0.284391826	13.69548	1.08E-42	5.21E-42	cyan	0.633591155	5.32E-19		AT1G27660.1		basic helix-loop-helix (bHLH) DNA-binding superfamily protein	NA
Sapur.016G216400.1	91.88008834	-0.662113325	0.048367707	-13.6892	1.18E-42	5.68E-42	cyan	-0.752434392	6.62E-30		AT3G27520.1			hypothetical protein
Sapur.008G077100.1	132.228158	-0.867191153	0.063358412	-13.6871	1.21E-42	5.85E-42	cyan	-0.786452318	2.98E-34		AT1G26340.1	ATCB5-A,B5		cytochrome protein b5
Sapur.010G065800.1	21.79447863	-1.558253548	0.113887336	-13.6824	1.29E-42	6.24E-42	cyan	-0.698816806	2.54E-24		AT4G14723.1			epidermal patterning factor-like protein, putative
Sapur.002G045700.1	1144.61444	0.3633698	0.026559836	13.68118	1.32E-42	6.34E-42	cyan	0.738205013	2.73E-28		AT2G30880.1		Pleckstrin homology (PH) domain-containing protein	pleckstrin-like domain protein
Sapur.018G096600.1	623.3294985	0.40505794	0.029612192	13.67876	1.36E-42	6.55E-42	cyan	0.738129826	2.78E-28	GO:0008270	AT5G10790.1	UBP22	ubiquitin-specific protease 22	ubiquitin carboxyl-terminal hydrolase
Sapur.001G034500.1	22.55464326	1.708319183	0.124973545	13.66945	1.55E-42	7.44E-42	cyan	0.647235644	5.25E-20		AT4G29300.1	LCR27	low-molecular-weight cysteine-rich 27	NA
Sapur.010G071800.1	771.6979136	0.406423487	0.029754358	13.65929	1.78E-42	8.55E-42	cyan	0.744588405	5.30E-29		AT1G03280.1		Transcription factor TFIIE, alpha subunit	transcription initiation factor IIE, alpha subunit%2C putative
Sapur.004G069200.1	432.6484421	-0.751825387	0.055060235	-13.6546	1.90E-42	9.12E-42	cyan	-0.740117411	1.68E-28		AT3G29240.2		Protein of unknown function (DUF179)	plant/MXO21-9 protein
Sapur.008G111300.1	4.624958824	4.588967834	0.336161294	13.65109	1.99E-42	9.57E-42	cyan	0.585410625	8.13E-16					NA
Sapur.016G206300.1	1141.520901	0.461414886	0.033809725	13.6474	2.09E-42	1.01E-41	cyan	0.704933016	6.78E-25	GO:0016857,GO:0005975	AT3G01850.1		Aldolase-type TIM barrel family protein	ribulose-phosphate 3-epimerase
Sapur.017G028400.1	64.4180766	-1.857906303	0.136140104	-13.647	2.10E-42	1.01E-41	cyan	-0.655417083	1.23E-20	GO:0055114,GO:0020037,GO:0006979,GO:0004601	AT1G05260.1	RCI3,RCI3A	Peroxidase superfamily protein	peroxidase
Sapur.T126800.1	4.904597844	2.058317862	0.150846808	13.64509	2.16E-42	1.04E-41	cyan	0.75421173	4.09E-30					NA
Sapur.006G198400.1	688.6018157	0.403710475	0.029598545	13.63954	2.33E-42	1.12E-41	cyan	0.753653639	4.76E-30	GO:0006468,GO:0005524,GO:0004672	AT4G28880.1	ckl3	casein kinase I-like 3	casein kinase-like protein
Sapur.017G116700.1	851.0061619	-0.394009274	0.028890047	-13.6382	2.37E-42	1.14E-41	cyan	-0.743118549	7.76E-29	GO:0006433,GO:0005737,GO:0005524,GO:0004827,GO:0000166,GO:0006418,GO:000481	AT5G52520.1	OVA6,PRORS1	Class II aaRS and biotin synthetases superfamily protein	prolyl-tRNA synthetase
Sapur.014G126300.1	2394.894329	-0.339200434	0.024872444	-13.6376	2.39E-42	1.15E-41	cyan	-0.729175998	2.55E-27	GO:0006629,GO:0006508,GO:0004190	AT1G11910.1	APA1,ATAPA1	aspartic proteinase A1	fungal proteinase A, aspartic proteinase superfamily protein
Sapur.016G089200.2	19.40611905	-1.117491971	0.081947717	-13.6366	2.42E-42	1.16E-41	cyan	-0.734251285	7.34E-28		AT5G03560.1		Tetratricopeptide repeat (TPR)-like superfamily protein	nucleobase:cation symporter, putative
Sapur.013G049000.1	4.506724423	2.373791741	0.174085693	13.63577	2.45E-42	1.18E-41	cyan	0.699553378	2.17E-24	GO:0005515,GO:0016567,GO:0004842	AT3G07360.1	ATPUB9,PUB9	plant U-box 9	spotted leaf protein, putative
Sapur.012G070900.1	131.4807254	-0.838596297	0.061503376	-13.635	2.48E-42	1.19E-41	cyan	-0.740645133	1.47E-28	GO:0008152,GO:0003824	AT5G63380.1		AMP-dependent synthetase and ligase family protein	acyl:CoA ligase
Sapur.014G004200.1	28.93201474	-1.190993739	0.087356211	-13.6338	2.52E-42	1.21E-41	cyan	-0.708220354	3.29E-25		AT2G14520.1		CBS domain-containing protein with a domain of unknown function (DUF21)	magnesium and cobalt efflux protein CorC, putative
Sapur.013G026200.1	10.13864974	-1.273633683	0.093439549	-13.6306	2.64E-42	1.26E-41	cyan	-0.696950027	3.77E-24		AT1G54780.1	TLP18.3	thylakoid lumen 18.3 kDa protein	NA
Sapur.003G087800.1	11.94853089	-1.993823497	0.146358884	-13.6228	2.93E-42	1.40E-41	cyan	-0.725187274	6.66E-27		AT4G23060.1	IQD22	IQ-domain 22	NA
Sapur.014G078300.1	30.57166315	-1.764989146	0.129573982	-13.6215	2.98E-42	1.43E-41	cyan	-0.764457148	2.34E-31	GO:0006355,GO:0003700	AT1G01250.1		Integrase-type DNA-binding superfamily protein	AP2/ERF domain transcription factor
Sapur.007G062800.1	202.7119053	1.108234086	0.081390443	13.61627	3.21E-42	1.54E-41	cyan	0.691866426	1.09E-23		AT3G19740.1		P-loop containing nucleoside triphosphate hydrolases superfamily protein	NA
Sapur.006G191500.1	2013.769045	-0.601937434	0.044255443	-13.6014	3.93E-42	1.88E-41	cyan	-0.751124301	9.42E-30	GO:0003723	AT2G25970.1		KH domain-containing protein	Far upstream element-binding protein, putative
Sapur.004G060200.1	251.6255252	-0.616615522	0.045340128	-13.5998	4.02E-42	1.92E-41	cyan	-0.767489102	9.74E-32		AT5G37590.1		Tetratricopeptide repeat (TPR)-like superfamily protein	kinesin light chain
Sapur.15WG080900.1	470.5990599	-0.990642127	0.07287407	-13.5939	4.35E-42	2.08E-41	cyan	-0.78679869	2.66E-34		AT1G74520.1	ATHVA22A,HVA22A	HVA22 homologue A	HVA22-like protein
Sapur.018G116900.1	40.20863595	4.695413686	0.345477814	13.59107	4.52E-42	2.16E-41	cyan	0.522833545	2.15E-12	GO:0016021,GO:0004674,GO:0030247,GO:0006468,GO:0005524,GO:0004672	AT1G69730.1		Wall-associated kinase family protein	wall-associated receptor kinase-like protein, putative
Sapur.001G074300.1	47.54714215	-1.187303162	0.087365983	-13.59	4.59E-42	2.19E-41	cyan	-0.724580529	7.69E-27		AT1G64430.1		Pentatricopeptide repeat (PPR) superfamily protein	PPR domain protein
Sapur.018G073300.1	185.4637984	-0.832922844	0.061320676	-13.5831	5.05E-42	2.41E-41	cyan	-0.777444515	5.00E-33		AT4G28020.1			tRNA-thr(GGU) M(6)T(6)A37 methyltransferase TsaA
Sapur.008G046100.1	22.12153764	-1.396041074	0.102810089	-13.5788	5.35E-42	2.55E-41	cyan	-0.690023643	1.60E-23		AT1G74260.1	PUR4	purine biosynthesis 4	NA
Sapur.009G053400.1	13.23290246	-1.419867127	0.104595476	-13.5748	5.65E-42	2.69E-41	cyan	-0.749699782	1.38E-29					transmembrane protein, putative
Sapur.002G083300.1	131.5962858	-1.239495742	0.091327033	-13.5721	5.87E-42	2.80E-41	cyan	-0.736267491	4.44E-28		AT5G16650.1		Chaperone DnaJ-domain superfamily protein	DnaJ heat shock amino-terminal domain protein
Sapur.016G175200.1	391.5045833	-0.376835937	0.027767447	-13.5711	5.94E-42	2.83E-41	cyan	-0.736754776	3.93E-28	GO:0051536,GO:0003824,GO:0008173,GO:0006364,GO:0005737	AT3G19630.1		Radical SAM superfamily protein	ribosomal RNA large subunit methyltransferase N
Sapur.017G008900.1	189.8032778	-0.76261962	0.056229369	-13.5627	6.67E-42	3.18E-41	cyan	-0.76295162	3.59E-31		AT5G40520.1			hypothetical protein
Sapur.006G148700.1	50.18568389	1.188742249	0.087686625	13.55671	7.23E-42	3.44E-41	cyan	0.668498192	1.11E-21		AT5G08110.1		nucleic acid binding;ATP-dependent helicases;ATP binding;helicases;ATP-dependent helicases	NA
Sapur.016G058400.1	33.76119098	-1.031228547	0.076079067	-13.5547	7.43E-42	3.54E-41	cyan	-0.710353109	2.05E-25	GO:0005515	AT1G15670.1		Galactose oxidase/kelch repeat superfamily protein	F-box protein
Sapur.004G147200.1	69.79727897	4.455629633	0.328742085	13.55357	7.55E-42	3.59E-41	cyan	0.612831856	1.46E-17	GO:0006412,GO:0005840,GO:0003735	AT3G52590.1	EMB2167.ERD16.HAP4.UBC	ubiquitin extension protein 1	ubiquitin-60S ribosomal L40 fusion protein
Sapur.010G142400.1	9.036142516	-2.563505778	0.189149252	-13.5528	7.62E-42	3.63E-41	cyan	-0.660606972	4.77E-21	GO:0016758,GO:0008152	AT5G49690.1		UDP-Glycosyltransferase superfamily protein	NA
Sapur.016G044200.1	22.67314373	3.113552696	0.229912897	13.54231	8.80E-42	4.18E-41	cyan	0.697286458	3.51E-24	GO:0005515	AT5G06860.1	ATPGIP1,PGIP1	polygalacturonase inhibiting protein 1	polygalacturonase-inhibiting protein
Sapur.017G054900.1	30.0428979	-1.509812976	0.111620059	-13.5264	1.09E-41	5.19E-41	cyan	-0.731575557	1.42E-27		AT5G14105.1			transmembrane protein, putative
Sapur.016G158500.1	85.98301331	-0.677526003	0.050108273	-13.5212	1.17E-41	5.57E-41	cyan	-0.732302227	1.19E-27		AT1G11900.1		Tetratricopeptide repeat (TPR)-like superfamily protein	PPR containing plant protein
Sapur.001G168700.1	18.52375221	-1.110210854	0.082132282	-13.5174	1.24E-41	5.87E-41	cyan	-0.743088802	7.82E-29	GO:0043565,GO:0006351	AT1G15320.1			seed dormancy control protein
Sapur.T068600.1	3.073622712	3.25474221	0.240963043	13.50723	1.42E-41	6.73E-41	cyan	0.743236902	7.53E-29	GO:0005681,GO:0000398,GO:0000387	AT1G54385.2		ARM repeat superfamily protein	NA
Sapur.010G110600.3	7.501773596	2.675761698	0.198099086	13.50719	1.42E-41	6.73E-41	cyan	0.590564832	3.93E-16	GO:0005975,GO:0004553	AT1G02640.1	ATBXL2,BXL2	beta-xylosidase 2	alpha-L-arabinofuranosidase/beta-D-xylosidase
Sapur.010G160600.1	2265.63437	0.818619829	0.060626573	13.50266	1.51E-41	7.16E-41	cyan	0.698372414	2.79E-24	GO:0016627,GO:0016021,GO:0006629,GO:0005737	AT3G55360.1	ATTSC13,CER10,ECR,TSC13	3-oxo-5-alpha-steroid 4-dehydrogenase family protein	enoyl-CoA reductase
Sapur.012G076700.1	3965.429147	1.105474718	0.081872663	13.50237	1.51E-41	7.18E-41	cyan	0.704383028	7.65E-25	GO:0046872,GO:0030001	AT5G50740.1		Heavy metal transport/detoxification superfamily protein	heavy metal-associated domain protein
Sapur.004G147700.1	603.636562	-0.590084662	0.043705671	-13.5013	1.54E-41	7.28E-41	cyan	-0.742239465	9.74E-29	GO:0005524,GO:0051082,GO:0006457	AT3G11830.1		TCP-1/cpn60 chaperonin family protein	TCP-1 (CTT or eukaryotic type II) chaperonin family, eta subunit
Sapur.008G060400.1	6.179858422	-1.971332034	0.146031761	-13.4993	1.58E-41	7.48E-41	cyan	-0.694740458	6.01E-24		AT3G03300.3	ATDCL2,DCL2	dicer-like 2	endoribonuclease dicer-like protein
Sapur.017G093600.1	18.25787135	1.33950762	0.099241727	13.49742	1.62E-41	7.67E-41	cyan	0.702749824	1.09E-24	GO:0019904	AT3G02520.1	GF14 NU,GRF7	general regulatory factor 7	14-3-like protein GF14 Psi protein
Sapur.004G118100.1	521.6525924	-0.610139593	0.045237507	-13.4875	1.85E-41	8.78E-41	cyan	-0.744836161	4.97E-29		AT2G16365.2		F-box family protein	hypothetical protein
Sapur.010G061200.1	216.0646504	-0.816521695	0.060542681	-13.4867	1.87E-41	8.87E-41	cyan	-0.740993422	1.34E-28	GO:0051536,GO:0009055	AT4G14890.1		2Fe-2S ferredoxin-like superfamily protein	ferredoxin (2Fe-2S)
Sapur.016G130100.1	191.6088831	-0.613375433	0.045483996	-13.4855	1.90E-41	9.01E-41	cyan	-0.734902461	6.24E-28		AT5G59900.1		Pentatricopeptide repeat (PPR) superfamily protein	PPR containing plant protein, putative
Sapur.016G262100.1	13.0469947	2.826088958	0.209590485	13.48386	1.95E-41	9.22E-41	cyan	0.506581548	1.30E-11		AT3G15518.1			NA
Sapur.T080800.1	4167.565846	1.122987592	0.083285084	13.48366	1.95E-41	9.24E-41	cyan	0.743182143	7.63E-29					NA
Sapur.15WG080200.1	3615.301043	-0.856378264	0.063516766	-13.4827	1.98E-41	9.36E-41	cyan	-0.719549552	2.51E-26	GO:0016020,GO:0009765	AT3G47470.1	CAB4,LHCA4	light-harvesting chlorophyll-protein complex I subunit A4	photosystem I light harvesting complex Lhca2/4, chlorophyll A/B-binding protein
Sapur.006G098000.1	1115.234315	0.57264529	0.042480351	13.48024	2.04E-41	9.67E-41	cyan	0.70315049	1.00E-24		AT2G36680.1		Modifier of rudimentary (Mod(r)) protein	modifier of rudimentary protein
Sapur.002G105000.1	82.00540607	1.076502961	0.079880276	13.47646	2.15E-41	1.02E-40	cyan	0.662877047	3.17E-21		AT2G26530.1	AR781	Protein of unknown function (DUF1645)	AR781, yeast pheromone receptor-like protein%2C putative
Sapur.15WG126000.1	240.7290894	-1.940706253	0.144011915	-13.476	2.16E-41	1.02E-40	cyan	-0.749458443	1.47E-29		AT3G23760.1			transferring glycosyl group transferase, putative
Sapur.001G018700.1	2.03983563	4.523393135	0.335726998	13.47343	2.24E-41	1.06E-40	cyan	0.569433724	7.13E-15	GO:0005515				F-box protein
Sapur.004G160000.2	1.878629905	4.181252496	0.310483345	13.46691	2.45E-41	1.16E-40	cyan	0.563547722	1.54E-14	GO:0055114,GO:0016491,GO:0010181	AT2G06050.3	DDE1,OPR3	oxophytodienoate-reductase 3	12-oxophytodienoate reductase
Sapur.T139000.1	13.80645936	1.693997011	0.125796765	13.46614	2.47E-41	1.17E-40	cyan	0.679304687	1.38E-22		AT5G19820.1	emb2734	ARM repeat superfamily protein	NA
Sapur.15WG132700.1	9.087497842	1.58538906	0.117741892	13.46495	2.51E-41	1.19E-40	cyan	0.705069593	6.58E-25		AT1G31175.1			hypothetical protein
Sapur.004G051900.2	6.37470502	2.027786174	0.150625406	13.46244	2.60E-41	1.23E-40	cyan	0.733978505	7.85E-28		AT5G17300.1	RVE1	Homeodomain-like superfamily protein	myb-like DNA-binding domain protein
Sapur.T023400.1	47.38631789	-0.833839552	0.061943006	-13.4614	2.64E-41	1.25E-40	cyan	-0.738284446	2.67E-28		AT3G03320.1		RNA-binding ASCH domain protein	transmembrane protein, putative
Sapur.15ZG027300.1	13.13364373	3.367206606	0.250174255	13.45944	2.71E-41	1.28E-40	cyan	0.617158984	7.47E-18		AT1G67920.1			hypothetical protein
Sapur.017G095600.1	46.96046821	-0.754537743	0.05606415	-13.4585	2.75E-41	1.30E-40	cyan	-0.733082646	9.80E-28		AT2G18410.1			elongator complex protein
Sapur.008G009600.1	105.6077359	-0.550902919	0.040956552	-13.4509	3.04E-41	1.43E-40	cyan	-0.737219925	3.49E-28		AT5G22040.2			disease resistance protein, putative
Sapur.007G011800.1	399.4587178	-0.576865547	0.04288844	-13.4504	3.06E-41	1.44E-40	cyan	-0.734546253	6.82E-28	GO:0003677,GO:0000786	AT4G40030.2		Histone superfamily protein	histone H3
Sapur.016G279300.1	770.6058334	3.422559246	0.254480482	13.4492	3.11E-41	1.47E-40	cyan	0.559938312	2.46E-14					hypothetical protein
Sapur.009G107200.1	225.8411469	-0.884968288	0.065819222	-13.4454	3.27E-41	1.54E-40	cyan	-0.718189227	3.44E-26	GO:0007264,GO:0005525	AT4G35020.3	ARAC3.ATROP6.RAC3.RHO1	RAC-like 3	Rac-like small GTP-binding protein
Sapur.010G119800.1	4.947235884	2.079703342	0.154898512	13.42623	4.24E-41	2.00E-40	cyan	0.704319201	7.75E-25	GO:0016887,GO:0005524,GO:0016020	AT3G53480.1	ABCG37.ATPDR9.PDR9.PIS1	pleiotropic drug resistance 9	ABC transporter G family protein
Sapur.003G097500.1	333.2806809	0.42511284	0.031664392	13.42558	4.28E-41	2.02E-40	cyan	0.712812555	1.18E-25	GO:0016020	AT5G60750.1		CAAX amino terminal protease family protein	CAAX amino terminal protease family protein
Sapur.004G170100.1	23.70930164	-1.430006579	0.106563263	-13.4193	4.66E-41	2.19E-40	cyan	-0.732700426	1.08E-27					transmembrane protein, putative
Sapur.012G070800.1	178.0446816	-0.710608587	0.052956018	-13.4188	4.69E-41	2.21E-40	cyan	-0.759155983	1.05E-30		AT1G67570.1		Protein of unknown function (DUF3537)	transmembrane protein, putative
Sapur.001G176100.1	1.999530269	3.916402289	0.291936633	13.41525	4.92E-41	2.32E-40	cyan	0.629963744	9.67E-19		AT3G50500.2	SNRK2-2.SNRK2.2.SPK-2-2.S	SNF1-related protein kinase 2.2	kinase, putative
Sapur.010G144200.1	815.7956357	-0.662839935	0.049410592	-13.4149	4.94E-41	2.32E-40	cyan	-0.753324499	5.20E-30	GO:0008199,GO:0006879,GO:0006826	AT3G11050.1	ATFER2,FER2	ferritin 2	ferritin
Sapur.003G157300.1	3.117672397	-2.46998146	0.184201637	-13.4091	5.35E-41	2.51E-40	cyan	-0.731379249	1.49E-27		AT1G21200.1		sequence-specific DNA binding transcription factors	NA
Sapur.016G084100.1	1028.309237	0.462645771	0.034523868	13.40075	5.99E-41	2.81E-40	cyan	0.716083345	5.59E-26		AT3G09770.1		RING/U-box superfamily protein	E3 ubiquitin-protein ligase, putative
Sapur.010G178000.1	13029.156	0.969390226	0.072342108	13.40008	6.04E-41	2.84E-40	cyan	0.698916316	2.49E-24	GO:0016020,GO:0006810,GO:0005215	AT2G37170.1	PIP2;2,PIP2B	plasma membrane intrinsic protein 2	plasma membrane intrinsic protein
Sapur.011G087500.1	409.2472254	0.639528931	0.047745979	13.3944	6.52E-41	3.06E-40	cyan	0.691962363	1.07E-23		AT3G15480.1		Protein of unknown function (DUF1218)	fiber protein Fb34
Sapur.001G126400.1	2622.045318	-0.435199759	0.032514035	-13.385	7.40E-41	3.47E-40	cyan	-0.720635315	1.95E-26	GO:0006412,GO:0005840,GO:0005622,GO:0003735	AT4G17560.1		Ribosomal protein L19 family protein	50S ribosomal protein L19, putative
Sapur.016G185900.1	435.8196585	-0.948385726	0.070855869	-13.3847	7.43E-41	3.48E-40	cyan	-0.737008056	3.69E-28	GO:0005515,GO:0006468,GO:0004672	AT3G14840.2		Leucine-rich repeat transmembrane protein kinase	NA
Sapur.004G135500.2	15.02934422	-1.414253586	0.105694109	-13.3806	7.85E-41	3.68E-40	cyan	-0.724303471	8.22E-27	GO:0005089	AT4G38430.1	ATROPGEF1,ROPGEF1	rho guanyl-nucleotide exchange factor 1	Rop guanine nucleotide exchange factor, putative
Sapur.009G087700.1	2681.864388	0.416996918	0.031167811	13.37909	8.01E-41	3.76E-40	cyan	0.723627534	9.64E-27	GO:0051745,GO:0050992,GO:0046872,GO:0019288	AT4G34350.1	CLB6,HDR,ISPH	4-hydroxy-3-methylbut-2-enyl diphosphate reductase	4-hydroxy-3-methylbut-2-enyl diphosphate reductase
Sapur.004G152700.1	588.3670496	-0.841824484	0.06292682	-13.3778	8.15E-41	3.82E-40	cyan	-0.745003183	4.76E-29	GO:0003924	AT2G36250.1	ATFTSZ2-1,FTSZ2-1	Tubulin/FtsZ family protein	cell division FtsZ-like protein
Sapur.006G085600.1	229.075778	-0.387891477	0.028996828	-13.377	8.24E-41	3.86E-40	cyan	-0.734667769	6.62E-28	GO:0046872,GO:0016567,GO:0004842	AT5G60250.1		zinc finger (C3HC4-type RING finger) family protein	C3H4 type zinc finger protein
Sapur.001G119500.1	48.78780157	1.037403942	0.077551867	13.3769	8.25E-41	3.87E-40	cyan	0.70391302	8.47E-25	GO:0015743	AT4G17970.1	ALMT12,ATALMT12	aluminum-activated, malate transporter 12	aluminum-activated malate transporter
Sapur.008G090500.1	29.56331199	1.447961882	0.108261992	13.37461	8.51E-41	3.99E-40	cyan	0.722071092	1.39E-26	GO:0005515,GO:0016567,GO:0004842	AT1G68940.1		Armadillo/beta-catenin-like repeat family protein	ubiquitin-protein ligase, putative
Sapur.15WG096800.1	51.24815989	-1.203379047	0.090024029	-13.3673	9.39E-41	4.39E-40	cyan	-0.730408857	1.89E-27	GO:0016021,GO:0006820,GO:0016020,GO:0005452	AT3G62270.1		HCO3- transporter family	boron transporter
Sapur.008G093200.1	21.6090855	1.541580661	0.11535823	13.36342	9.89E-41	4.63E-40	cyan	0.621945584	3.52E-18		AT1G68800.1	BRC2,TCP12	TCP domain protein 12	teosinte-branched-like protein, putative
Sapur.016G232400.1	59.53139192	-1.385289869	0.103693807	-13.3594	1.04E-40	4.88E-40	cyan	-0.794857782	1.89E-35		AT2G04480.1			transmembrane protein, putative
Sapur.004G042800.1	451.7887473	1.034993833	0.077495691	13.3555	1.10E-40	5.14E-40	cyan	0.688586897	2.15E-23	GO:0016567,GO:0004842,GO:0005515	AT1G29340.1	ATPUB17,PUB17	plant U-box 17	Avr9/Cf-9 rapidly elicited protein
Sapur.006G172300.1	30.72023323	-0.972413726	0.072842218	-13.3496	1.19E-40	5.57E-40	cyan	-0.70451162	7.44E-25	GO:0008017,GO:0007018,GO:0005524,GO:0003777,GO:0005871	AT2G36200.1		P-loop containing nucleoside triphosphate hydrolases superfamily protein	125 kDa kinesin-like protein
Sapur.009G082700.1	1305.622893	-0.407593284	0.03053255	-13.3495	1.19E-40	5.57E-40	cyan	-0.73147382	1.46E-27	GO:0003676	AT3G19130.1	ATRBP47B,RBP47B	RNA-binding protein 47B	RNA-binding protein
Sapur.013G073000.1	4634.892703	-0.478805346	0.035868926	-13.3488	1.20E-40	5.63E-40	cyan	-0.768894526	6.46E-32	GO:0006412,GO:0005840,GO:0005622,GO:0003735	AT2G43030.1		Ribosomal protein L3 family protein	50S ribosomal protein L3
Sapur.005G059100.1	4847.774245	0.680697347	0.051005718	13.34551	1.26E-40	5.87E-40	cyan	0.706169805	5.17E-25	GO:0005544,GO:0005509	AT5G65020.1	ANNAT2	annexin 2	annexin AnxGb1
Sapur.002G112100.1	2255.209991	-0.483130778	0.036227186	-13.3361	1.43E-40	6.65E-40	cyan	-0.740741789	1.43E-28	GO:0006241,GO:0006228,GO:0006183,GO:0006165,GO:0004550	AT4G09320.1	NDPK1	Nucleoside diphosphate kinase family protein	nucleoside diphosphate kinase
Sapur.007G038400.1	19.22503996	-2.369694269	0.177693744	-13.3358	1.43E-40	6.68E-40	cyan	-0.604138574	5.45E-17		AT2G23440.1			hypothetical protein
Sapur.007G115400.1	69.3627389	-0.577937876	0.043339267	-13.3352	1.44E-40	6.74E-40	cyan	-0.730088793	2.04E-27	GO:0003723	AT2G28480.1		RNA-binding CRS1 / YhbY (CRM) domain protein	CRS1/YhbY domain protein
Sapur.001G006200.1	913.8693744	0.980160435	0.073516199	13.33258	1.50E-40	6.97E-40	cyan	0.670822644	7.15E-22	GO:0055085,GO:0016020,GO:0006810,GO:0015171,GO:0003333	AT3G13620.1		Amino acid permease family protein	amino acid transporter, putative
Sapur.007G113500.1	187.8737646	-0.469915607	0.03526678	-13.3246	1.67E-40	7.76E-40	cyan	-0.744224797	5.83E-29	GO:0006284	AT2G31450.2	ATNTH1	DNA glycosylase superfamily protein	endonuclease III-like protein
Sapur.15WG132500.1	30.11513222	-1.389884473	0.104336283	-13.3212	1.74E-40	8.12E-40	cyan	-0.735412508	5.50E-28					NA
Sapur.013G109300.1	7.652561768	2.319289966	0.174120913	13.32	1.77E-40	8.25E-40	cyan	0.652979102	1.91E-20	GO:0016887,GO:0005524,GO:0016020	AT1G53270.1		ABC-2 type transporter family protein	white-brown-complex ABC transporter family protein
Sapur.006G059500.1	99.30581621	-0.887495726	0.066653683	-13.315	1.89E-40	8.81E-40	cyan	-0.763597323	2.99E-31		AT1G07400.1		HSP20-like chaperones superfamily protein	NA
Sapur.009G034100.1	43.14115409	-1.07218793	0.080577433	-13.3063	2.13E-40	9.90E-40	cyan	-0.730280308	1.95E-27	GO:0016020,GO:0015696,GO:0008519,GO:0072488	AT4G13510.1	AMT1:1.ATAMT1.ATAMT1:	ammonium transporter 1;1	ammonium transporter 1 protein
Sapur.004G083000.1	363.6390092	-0.604806158	0.045466389	-13.3023	2.25E-40	1.04E-39	cyan	-0.754224507	4.07E-30		AT5G16040.1		Regulator of chromosome condensation (RCC1) family protein	chromosome condensation regulator repeat protein
Sapur.014G089500.1	714.7614152	-0.541227125	0.04069026	-13.3011	2.28E-40	1.06E-39	cyan	-0.76697836	1.13E-31		AT3G62130.1		Pyridoxal phosphate (PLP)-dependent transferases superfamily protein	cysteine desulfurase
Sapur.016G210500.1	246.7409663	2.478289078	0.186346042	13.29939	2.33E-40	1.08E-39	cyan	0.617839689	6.72E-18		AT1G19250.1	FMO1	flavin-dependent monooxygenase 1	flavin containing monooxygenase, putative

Sapur.003G088300.1	119.7169798	-0.716103488	0.05385043	-13.298	2.38E-40	1.10E-39	cyan	-0.763937667	2.71E-31	GO:0005524	AT1G63160.1	RFC2	replication factor C 2	replication factor C subunit 2
Sapur.002G084400.1	1200.36953	0.287864293	0.021647538	13.29778	2.38E-40	1.11E-39	cyan	0.731620854	1.40E-27		AT4G24550.2		Clathrin adaptor complexes medium subunit family protein	AP-4 complex subunit mu-1
Sapur.15ZG053300.1	34.69484804	0.8998852	0.067684006	13.29539	2.46E-40	1.14E-39	cyan	0.719350623	2.63E-26		AT5G15740.1		O-fucosyltransferase family protein	GDP-fucose protein O-fucosyltransferase
Sapur.009G019500.1	8.511604201	1.960214525	0.147456968	13.29347	2.53E-40	1.17E-39	cyan	0.600451043	9.40E-17	GO:0006887,GO:0000145	AT5G59730.2	ATEXO70H7,EXO70H7	exocyst subunit exo70 family protein H7	exo70 exocyst complex subunit
Sapur.016G007600.1	114.565113	1.908465551	0.143588049	13.29126	2.60E-40	1.21E-39	cyan	0.691860097	1.09E-23	GO:0055114,GO:0020037,GO:0016705,GO:0005506	AT4G31940.1	CYP82C4	cytochrome P450, family 82, subfamily C, polypeptide 4	NA
Sapur.016G046100.5	31.71971162	-1.172498767	0.088233504	-13.2886	2.70E-40	1.25E-39	cyan	-0.692906454	8.81E-24		AT2G42320.2		nucleolar protein gar2-related	LSG protein
Sapur.002G077400.1	110.7047914	0.951048177	0.071588935	13.28485	2.83E-40	1.31E-39	cyan	0.71299051	1.13E-25		AT1G22260.1	ZYP1,ZYP1a	Myosin heavy chain-related protein	synaptonemal complex protein ZYP1
Sapur.004G058600.1	136.2321658	-0.615924215	0.046374823	-13.2814	2.97E-40	1.38E-39	cyan	-0.746636959	3.10E-29	GO:0008641	AT5G37530.1		NAD(P)-binding Rossmann-fold superfamily protein	rossmann-fold NAD(P)-binding domain protein
Sapur.010G076400.1	477.6544296	-0.605844428	0.045616623	-13.2812	2.98E-40	1.38E-39	cyan	-0.722041488	1.40E-26		AT1G68660.1		Ribosomal protein L12/ ATP-dependent Clp protease adaptor protein ClpS family protein	ATP-dependent Clp protease adapter protein ClpS
Sapur.006G056300.2	21.1106428	1.227221438	0.09243323	13.27684	3.15E-40	1.46E-39	cyan	0.673760313	4.07E-22	GO:0055114,GO:0006788,GO:0004392	AT2G26670.1	ATHO1.GUN2.HO1.HY1.HY6	Plant haem oxygenase (decyclizing) family protein	heme oxygenase
Sapur.001G002500.1	672.9517435	-0.772882689	0.05823053	-13.2728	3.33E-40	1.54E-39	cyan	-0.714146466	8.70E-26	GO:0006351,GO:0003899,GO:0003677	AT5G23710.1		DNA binding;DNA-directed RNA polymerases	NA
Sapur.006G149600.1	42.5025147	1.251122753	0.09427651	13.27078	3.42E-40	1.58E-39	cyan	0.641003651	1.53E-19		AT5G08110.1		nucleic acid binding;ATP-dependent helicases;ATP binding;helicases;ATP-dependent helicases	NA
Sapur.15WG081600.1	10.1286243	1.562669703	0.117824367	13.2627	3.81E-40	1.76E-39	cyan	0.708793989	2.90E-25	GO:0016887,GO:0005524,GO:0016021,GO:0006810,GO:0005215	AT3G47780.1	ATATH6,ATH6	ABC2 homolog 6	ABC transporter family, cholesterol/phospholipid flippase%2C putative
Sapur.011G053900.1	33.80643726	-1.384016079	0.104422489	-13.254	4.28E-40	1.98E-39	cyan	-0.727246835	4.07E-27					hypothetical protein
Sapur.001G010600.1	2202.753887	-0.421597292	0.031814659	-13.2517	4.41E-40	2.04E-39	cyan	-0.747786888	2.29E-29	GO:0016491,GO:0008152	AT2G05990.2	ENR1,MOD1	NAD(P)-binding Rossmann-fold superfamily protein	enoyl-(acyl carrier) reductase
Sapur.012G064600.1	68.3395719	1.315002709	0.099264609	13.24745	4.67E-40	2.16E-39	cyan	0.670909081	7.03E-22	GO:0005634,GO:0003677	AT1G23380.1	KNAT6,KNAT6L,KNAT6S	KNOTTED1-like homeobox gene 6	NA
Sapur.016G180000.1	169.7313206	-0.796284112	0.060123339	-13.2442	4.88E-40	2.25E-39	cyan	-0.681265863	9.38E-23		AT2G15270.1			DUF1168 family protein
Sapur.019G086100.1	160.623079	-0.493918586	0.037297154	-13.2428	4.97E-40	2.29E-39	cyan	-0.738122029	2.78E-28		AT1G06240.1		Protein of unknown function DUF455	DUF455 family protein
Sapur.010G198100.1	8.212700958	-1.78925032	0.135155008	-13.2385	5.26E-40	2.43E-39	cyan	-0.695292073	5.35E-24		AT5G06390.1	FLA17	FASCICLIN-like arabinogalactan protein 17 precursor	fasciclin-like arabinogalactan protein
Sapur.011G067600.1	1303.744677	0.345454705	0.026103947	13.23381	5.60E-40	2.58E-39	cyan	0.717014567	4.52E-26	GO:0055114,GO:0009396,GO:0004488,GO:0003824	AT3G12290.1		Amino acid dehydrogenase family protein	5%2C10-methylene-tetrahydrofolate dehydrogenase/methenyl tetrahydrofolate cyclohydrolase
Sapur.15WG009600.1	1115.764613	0.380819401	0.028787374	13.2287	5.99E-40	2.76E-39	cyan	0.721513333	1.59E-26	GO:0009966	AT5G53000.1	TAP46	2A phosphatase associated protein of 46 kD	PP2A regulatory subunit TAP46
Sapur.15WG008700.1	230.1092636	-0.513081813	0.03879501	-13.2255	6.26E-40	2.88E-39	cyan	-0.727984309	3.40E-27		AT5G53080.1		Tetratricopeptide repeat (TPR)-like superfamily protein	kinesin light chain, putative
Sapur.003G049800.1	57.25859529	-1.252808011	0.094754098	-13.2217	6.58E-40	3.03E-39	cyan	-0.758198486	1.36E-30		AT4G16807.1			hypothetical protein
Sapur.009G046200.1	44.30495323	0.791297925	0.059872697	13.21634	7.06E-40	3.25E-39	cyan	0.723486437	9.97E-27		AT5G47940.1			40S ribosomal protein S27, putative
Sapur.003G101500.3	73.80865843	1.010927663	0.076502307	13.21434	7.25E-40	3.34E-39	cyan	0.519898372	3.00E-12		AT5G41800.1		Transmembrane amino acid transporter family protein	proline transporter
Sapur.010G076600.1	808.6763464	-1.287059488	0.097408193	-13.2131	7.38E-40	3.40E-39	cyan	-0.790783942	7.30E-35		AT1G60060.1		Serine/threonine-protein kinase WNK (With No Lysine)-related	bHLH-MYC/R2R3-MYB transcription factor amino-terminal protein
Sapur.005G111200.1	134.9760761	-0.688696552	0.052129622	-13.2112	7.56E-40	3.48E-39	cyan	-0.739043137	2.20E-28		AT1G11290.1	CRR22	Pentatricopeptide repeat (PPR) superfamily protein	PPR containing protein
Sapur.005G035400.1	531.5655642	-0.703101547	0.053221919	-13.2108	7.61E-40	3.50E-39	cyan	-0.705836129	5.56E-25	GO:0016747	AT1G54570.1		Esterase/lipase/thioesterase family protein	NA
Sapur.013G102100.2	10.6558916	-1.686133306	0.127658728	-13.2081	7.88E-40	3.62E-39	cyan	-0.701516412	1.42E-24		AT1G22630.1			hypothetical protein
Sapur.010G080800.1	2.892725811	3.179764121	0.240753152	13.20757	7.93E-40	3.65E-39	cyan	0.697047571	3.70E-24		AT1G67920.1			hypothetical protein
Sapur.010G206100.1	409.9462245	-1.307365871	0.09901175	-13.2041	8.30E-40	3.82E-39	cyan	-0.691898511	1.09E-23	GO:0030955,GO:0006096,GO:0004743,GO:0000287	AT5G08570.1		Pyruvate kinase family protein	pyruvate kinase
Sapur.005G132900.1	35.37889743	-1.224653749	0.092811281	-13.1951	9.36E-40	4.30E-39	cyan	-0.748002523	2.16E-29		AT4G18520.1		Pentatricopeptide repeat (PPR) superfamily protein	PPR containing plant protein
Sapur.016G289700.2	4.481367367	2.583609192	0.195860713	13.19105	9.88E-40	4.54E-39	cyan	0.670353761	7.81E-22		AT5G42110.1			hypothetical protein
Sapur.002G061400.1	122.7875854	-1.337951354	0.101459088	-13.1871	1.04E-39	4.78E-39	cyan	-0.716658133	4.90E-26	GO:0055114,GO:0016655	AT4G37925.1	NDH-M	subunit NDH-M of NAD(P)H:plastoquinone dehydrogenase complex	NAD(P)H-quinone oxidoreductase subunit M
Sapur.014G080300.1	327.1062687	-0.61000282	0.046270812	-13.1833	1.09E-39	5.03E-39	cyan	-0.708253981	3.26E-25	GO:0046933,GO:0015986	AT4G09650.1	ATPD	ATP synthase delta-subunit gene	ATP synthase F1, delta subunit
Sapur.007G078700.1	15.58870742	-1.029473446	0.078102061	-13.1811	1.13E-39	5.17E-39	cyan	-0.758235135	1.35E-30		AT2G22070.1		pentatricopeptide (PPR) repeat-containing protein	PPR containing plant protein
Sapur.005G070600.1	61.92559173	-1.296338646	0.098372972	-13.1778	1.18E-39	5.40E-39	cyan	-0.695588009	5.03E-24	GO:0055114,GO:0046872,GO:0006801,GO:0004784	AT5G23310.1	FSD3	Fe superoxide dismutase 3	iron superoxide dismutase
Sapur.010G020000.1	23.47612422	3.005672826	0.228127681	13.1754	1.22E-39	5.58E-39	cyan	0.519045756	3.30E-12		AT1G67750.1		Pectate lyase family protein	pectate lyase
Sapur.004G035900.1	480.0751161	-0.348300092	0.026440917	-13.1728	1.26E-39	5.77E-39	cyan	-0.725122053	6.76E-27	GO:0004386	AT4G18465.1		RNA helicase family protein	ATP-dependent RNA helicase DHX35, putative
Sapur.011G100900.1	162.7273159	1.14308975	0.086783692	13.17171	1.28E-39	5.85E-39	cyan	0.6334579	5.44E-19		AT4G21780.1			hypothetical protein
Sapur.010G194900.1	494.8654609	0.805794031	0.061183669	13.17008	1.30E-39	5.98E-39	cyan	0.696135689	4.48E-24	GO:0055085,GO:0016020,GO:0006810,GO:0015171,GO:0003333	AT5G04770.1	ATCAT6,CAT6	cationic amino acid transporter 6	cationic amino acid transporter, putative
Sapur.014G024000.1	52.98926269	3.066282569	0.232850108	13.16848	1.33E-39	6.11E-39	cyan	0.5425467	2.14E-13	GO:0006629	AT2G26560.1	PLA IIA,PLA2A,PLP2	phospholipase A 2A	NA
Sapur.008G023200.1	56.03422646	-1.206891828	0.091667115	-13.166	1.38E-39	6.31E-39	cyan	-0.711284645	1.66E-25		AT5G22930.1		Protein of unknown function (DUF1635)	DUF1635 family protein
Sapur.T098800.1	2.269327709	3.709556903	0.281833836	13.16221	1.45E-39	6.63E-39	cyan	0.591979674	3.21E-16	GO:0005681,GO:0000398,GO:0000387	AT1G54385.1		ARM repeat superfamily protein	NA
Sapur.003G117900.1	580.590689	0.965995635	0.073397435	13.16116	1.47E-39	6.72E-39	cyan	0.750287988	1.18E-29	GO:0003824,GO:0006470,GO:0004722	AT3G16560.1		Protein phosphatase 2C family protein	protein phosphatase 2C
Sapur.017G106400.1	1246.406247	-0.726410844	0.055201087	-13.1594	1.50E-39	6.88E-39	cyan	-0.668800944	1.05E-21		AT1G66330.1		senescence-associated family protein	senescence-associated protein, putative
Sapur.006G113300.1	64.65771	-0.908009665	0.069019343	-13.1559	1.57E-39	7.20E-39	cyan	-0.737893817	2.95E-28		AT1G19240.1			transmembrane protein, putative
Sapur.010G202600.1	26.97758607	2.6099469	0.198505407	13.14799	1.75E-39	7.99E-39	cyan	0.594197477	2.34E-16	GO:0005516,GO:0046872,GO:0000166	AT3G21180.1	ACA9,ATACA9	autoinhibited Ca(2+)-ATPase 9	calcium-transporting ATPase 9, plasma membrane-type protein
Sapur.016G055500.1	1061.660161	0.607620819	0.046225698	13.14465	1.83E-39	8.35E-39	cyan	0.705263094	6.31E-25		AT3G58030.2		RING/U-box superfamily protein	zinc-RING finger protein
Sapur.010G075600.1	290.7820172	0.462036401	0.035151237	13.14424	1.84E-39	8.39E-39	cyan	0.686831639	3.07E-23		AT2G37770.2		NAD(P)-linked oxidoreductase superfamily protein	aldo-keto reductase family protein
Sapur.008G001300.1	189.6772546	-0.569692832	0.043341966	-13.1441	1.84E-39	8.40E-39	cyan	-0.75485231	3.43E-30		AT1G78915.1		Tetratricopeptide repeat (TPR)-like superfamily protein	TPR protein
Sapur.018G040700.1	35.47165233	-0.828715241	0.063095004	-13.1344	2.09E-39	9.54E-39	cyan	-0.742866788	8.28E-29		AT1G23550.1	SRO2	similar to RCD one 2	inactive poly [ADP-ribose] polymerase SRO2, putative
Sapur.018G081600.1	1.655304118	3.568493	0.271771787	13.13048	2.20E-39	1.01E-38	cyan	0.68367823	5.80E-23					NA
Sapur.016G310300.1	257.2633256	0.704761268	0.053680193	13.12889	2.25E-39	1.03E-38	cyan	0.721437087	1.62E-26	GO:0006468,GO:0005524,GO:0004672,GO:0005515	AT1G55610.2	BRL1	BRI1 like	receptor-like kinase BRI1-like protein
Sapur.007G120800.1	481.4572732	0.391422975	0.029815414	13.12821	2.27E-39	1.04E-38	cyan	0.73074103	1.74E-27		AT2G30710.1		Ypt/Rab-GAP domain of gyp1p superfamily protein	RabGAP/TBC domain protein
Sapur.002G033700.1	242.4791025	-0.836126226	0.063704061	-13.1252	2.36E-39	1.08E-38	cyan	-0.723112566	1.09E-26	GO:0015979,GO:0009523,GO:0009507	AT2G30570.1	PSBW	photosystem II reaction center W	photosystem II reaction center W
Sapur.009G086200.1	200.7465213	-0.516904792	0.03939315	-13.1217	2.47E-39	1.13E-38	cyan	-0.729345408	2.45E-27	GO:0031683,GO:0019001,GO:0007186,GO:0004871,GO:0003924	AT2G23460.1	ATXLG1,XLG1	extra-large G-protein 1	GTP-binding protein alpha subunit, gna protein%2C putative
Sapur.002G067700.1	242.7863285	0.518759365	0.039555504	13.11472	2.71E-39	1.24E-38	cyan	0.687051521	2.94E-23		AT1G44770.1			transmembrane protein, putative
Sapur.006G021100.1	3272.784878	-0.62729834	0.047832721	-13.1144	2.72E-39	1.24E-38	cyan	-0.771551347	2.95E-32	GO:0055114,GO:0046872,GO:0016491	AT3G56940.1	ACSF,CHL27,CRD1	dicarboxylate diiron protein, putative (Crd1)	magnesium-protoporphyrin IX monomethyl ester [oxidative] cyclase
Sapur.002G188400.1	42.02076661	-0.799207192	0.060944535	-13.1137	2.75E-39	1.25E-38	cyan	-0.738871947	2.30E-28		AT3G24860.1		Homeodomain-like superfamily protein	myb/SANT-like DNA-binding domain protein
Sapur.006G185900.1	244.4153101	-0.576094613	0.043942671	-13.1101	2.88E-39	1.31E-38	cyan	-0.740564029	1.50E-28		AT2G26200.1		S-adenosyl-L-methionine-dependent methyltransferases superfamily protein	methyltransferase domain protein
Sapur.014G105900.1	12.93898552	-1.51987146	0.115938327	-13.1093	2.91E-39	1.33E-38	cyan	-0.703967632	8.37E-25	GO:0005515	AT3G62760.1	ATGSTF13	Glutathione S-transferase family protein	Phi class glutathione transferase GSTF3
Sapur.016G200000.1	1070.094615	-0.283846808	0.021652708	-13.1091	2.92E-39	1.33E-38	cyan	-0.71378058	9.45E-26	GO:0008270	AT3G26935.1		DHHC-type zinc finger family protein	zinc finger protein
Sapur.014G144800.1	2.722042165	3.555497844	0.271425551	13.09935	3.32E-39	1.51E-38	cyan	0.683452381	6.07E-23		AT3G26380.1		Melibiase family protein	NA
Sapur.001G073200.1	26.46481262	-1.113416284	0.085010156	-13.0975	3.41E-39	1.55E-38	cyan	-0.725588664	6.05E-27		AT5G42070.1			hypothetical protein
Sapur.017G027600.1	683.7602332	0.429788839	0.032820735	13.09504	3.51E-39	1.60E-38	cyan	0.730371014	1.91E-27	GO:0005515	AT2G43210.2		Ubiquitin-like superfamily protein	UBX domain protein
Sapur.016G238200.1	813.3251134	-0.550558363	0.042047851	-13.0936	3.58E-39	1.63E-38	cyan	-0.721792411	1.49E-26		AT3G15095.1			hypothetical protein
Sapur.006G224000.1	11.12575127	1.987192121	0.151777661	13.09278	3.62E-39	1.64E-38	cyan	0.66703509	1.46E-21		AT5G57510.1			DUF761 domain protein
Sapur.016G139900.1	512.1356287	-1.44789738	0.110639777	-13.0866	3.93E-39	1.78E-38	cyan	-0.692809224	8.99E-24		AT3G47070.1			thylakoid soluble phosphoprotein TSP9 protein
Sapur.004G095600.1	31.65782083	-0.994453024	0.075996311	-13.0855	3.98E-39	1.81E-38	cyan	-0.759722963	8.92E-31	GO:0006412,GO:0005840,GO:0003735	AT5G15760.1		Ribosomal protein PSRP-3/Ycf65	plastid-specific 30S ribosomal protein, putative
Sapur.006G058000.1	197.0548236	-0.606796356	0.04637747	-13.0839	4.07E-39	1.85E-38	cyan	-0.723636169	9.62E-27					hypothetical protein
Sapur.003G127600.1	716.089922	-0.527939518	0.040350596	-13.0838	4.07E-39	1.85E-38	cyan	-0.721997581	1.42E-26	GO:0005515,GO:0005634,GO:0003677	AT1G67310.1		Calmodulin-binding transcription activator protein with CG-1 and Ankyrin domains	calmodulin-binding transcription activator
Sapur.006G063600.1	3131.895139	0.381721727	0.029175183	13.08378	4.08E-39	1.85E-38	cyan	0.70945838	2.50E-25		AT5G35360.1	CAC2	acetyl Co-enzyme a carboxylase biotin carboxylase subunit	acetyl-CoA carboxylase biotin carboxylase subunit
Sapur.006G065200.1	472.549967	-0.476161511	0.036395373	-13.083	4.12E-39	1.87E-38	cyan	-0.731431591	1.47E-27	GO:0016021	AT4G31040.1		CemA-like proton extrusion protein-related	CemA-like proton extrusion-like protein
Sapur.014G109100.1	94.25357025	-0.767169386	0.058639406	-13.0828	4.13E-39	1.87E-38	cyan	-0.732276946	1.20E-27		AT4G03130.1		BRCT domain-containing DNA repair protein	Pax transcription activation domain interacting protein, putative
Sapur.017G000800.1	657.2699596	-0.670235032	0.051239847	-13.0803	4.26E-39	1.93E-38	cyan	-0.757871646	1.49E-30		AT2G31890.1	ATRAP,RAP	RAP	RAP domain protein
Sapur.007G112000.1	79.17778291	-0.49752911	0.038046375	-13.0769	4.46E-39	2.02E-38	cyan	-0.739800671	1.82E-28	GO:0000123	AT2G31600.1			DNA-binding domain protein, putative
Sapur.T140700.1	15.2356965	1.271931811	0.09730452	13.07166	4.78E-39	2.16E-38	cyan	0.708591268	3.03E-25		AT1G01960.1	EDA10	SEC7-like guanine nucleotide exchange family protein	NA
Sapur.002G026100.2	25.54302209	1.078045244	0.082498224	13.0675	5.05E-39	2.29E-38	cyan	0.745987627	3.68E-29	GO:0016021	AT2G21600.1	ATRER1B,RER1B	endoplasmatic reticulum retrieval protein 1B	RER1A protein
Sapur.007G018300.1	270.3066131	1.940530784	0.148511894	13.0665	5.12E-39	2.31E-38	cyan	0.627298649	1.49E-18	GO:0008168	AT5G66430.1		S-adenosyl-L-methionine-dependent methyltransferases superfamily protein	S-adenosyl-L-methionine:salicylic acid carboxyl methyltransferase
Sapur.018G022500.1	1081.815769	-0.798204441	0.061110619	-13.0616	5.45E-39	2.47E-38	cyan	-0.707917759	3.52E-25	GO:0045263,GO:0015986,GO:0015078	AT4G32260.1		ATPase, F0 complex, subunit B/B\', bacterial/chloroplast	ATP synthase B/B' CF(0) protein
Sapur.019G029800.1	6.696225397	-2.441713731	0.186947382	-13.061	5.50E-39	2.49E-38	cyan	-0.698360879	2.80E-24					NA
Sapur.010G080200.1	84.48136907	-0.438058039	0.033564991	-13.051	6.27E-39	2.83E-38	cyan	-0.724094197	8.63E-27	GO:0009245,GO:0008759	AT1G25054.1		UDP-3-O-acyl N-acetylglycosamine deacetylase family protein	UDP-3-O-[3-hydroxymyristoyl] N-acetylglucosamine deacetylase
Sapur.005G173900.1	4076.312802	-0.703774342	0.053939834	-13.0474	6.58E-39	2.97E-38	cyan	-0.700667959	1.71E-24	GO:0015979,GO:0009523,GO:0009507	AT2G30570.1	PSBW	photosystem II reaction center W	photosystem II reaction center W
Sapur.002G016200.1	17.87745161	-1.11284007	0.085321098	-13.043	6.97E-39	3.15E-38	cyan	-0.745812363	3.85E-29		AT5G11600.1			hypothetical protein
Sapur.005G081900.1	192.6889602	-0.666105362	0.051073579	-13.0421	7.05E-39	3.18E-38	cyan	-0.739378055	2.02E-28		AT2G17525.1		Pentatricopeptide repeat (PPR) superfamily protein	PPR containing plant protein
Sapur.008G135700.1	260.037657	-0.731556595	0.056109606	-13.038	7.44E-39	3.36E-38	cyan	-0.730835766	1.70E-27		AT1G04420.1		NAD(P)-linked oxidoreductase superfamily protein	aldo/keto reductase family protein
Sapur.009G080700.1	149.7884356	-1.230177608	0.094372255	-13.0354	7.70E-39	3.47E-38	cyan	-0.735660686	5.17E-28	GO:0003677	AT3G19184.1		AP2/B3-like transcriptional factor family protein	B3 DNA-binding domain protein
Sapur.003G024700.1	488.3643691	0.81833536	0.062889368	13.0123	1.04E-38	4.69E-38	cyan	0.688963242	1.99E-23		AT1G55190.1	PRA1.F2,PRA7	PRA1 (Prenylated rab acceptor) family protein	PRA1 family protein
Sapur.002G035900.1	4.747975744	2.962240978	0.22766136	13.01161	1.05E-38	4.73E-38	cyan	0.605346421	4.55E-17		AT4G14640.1	CAM8	calmodulin 8	calmodulin, putative
Sapur.019G001200.1	1080.529594	0.672941879	0.051719369	13.01141	1.05E-38	4.74E-38	cyan	0.693813023	7.29E-24	GO:0016491,GO:0008198,GO:0006725	AT4G15093.1		catalytic LigB subunit of aromatic ring-opening dioxygenase family	4%2C5-DOPA dioxygenase extradiol-like protein
Sapur.008G003600.1	6.11955451	5.242721378	0.402933231	13.01139	1.05E-38	4.74E-38	cyan	0.528035587	1.19E-12		AT4G12560.2	CPR30	F-box and associated interaction domains-containing protein	NA
Sapur.15WG152100.1	400.3711853	-0.379485475	0.029167187	-13.0107	1.06E-38	4.79E-38	cyan	-0.719951007	2.29E-26	GO:0009001,GO:0006535,GO:0005737	AT5G56760.1	ATSERAT1:1.SAT-52.SAT5.SE	serine acetyltransferase 1;1	serine O-acetyltransferase
Sapur.019G089400.1	15.13393172	1.079059943	0.082941989	13.00982	1.08E-38	4.84E-38	cyan	0.666547007	1.60E-21	GO:0016567,GO:0004842	AT3G53690.1		RING/U-box superfamily protein	zinc finger protein, putative
Sapur.005G181200.1	44.87078609	0.910601112	0.070009054	13.0069	1.12E-38	5.03E-38	cyan	0.754883672	3.40E-30					hypothetical protein
Sapur.016G144700.1	592.0834933	-0.493814297	0.037969358	-13.0056	1.14E-38	5.11E-38	cyan	-0.739756164	1.84E-28	GO:0055114,GO:0016491,GO:0051536,GO:0020037	AT5G04590.1	SIR	sulfite reductase	sulfite reductase [ferredoxin] protein
Sapur.006G220000.1	934.4238711	0.376442783	0.02894468	13.00559	1.14E-38	5.11E-38	cyan	0.720130779	2.19E-26		AT5G10780.1			plant/T30N20-50 protein
Sapur.012G049800.1	1450.253643	-0.546479711	0.042020835	-13.005	1.15E-38	5.15E-38	cyan	-0.740143936	1.67E-28	GO:0050660,GO:0008033	AT1G74470.1		Pyridine nucleotide-disulphide oxidoreductase family protein	geranylgeranyl diphosphate reductase
Sapur.011G026200.2	62.68858583	1.065772464	0.081953705	13.00457	1.15E-38	5.18E-38	cyan	0.678964049	1.48E-22		AT1G11380.1		PLAC8 family protein	PLAC8 family protein
Sapur.011G069500.1	560.5790473	-0.647842418	0.049825041	-13.0023	1.19E-38	5.33E-38	cyan	-0.744217882	5.84E-29		AT3G15095.1			hypothetical protein
Sapur.005G190200.1	105.9424019	-1.51053693	0.116177933	-13.0019	1.19E-38	5.36E-38	cyan	-0.743336749	7.34E-29	GO:0009733	AT1G75580.1		SAUR-like auxin-responsive protein family	auxin responsive protein
Sapur.018G088400.1	712.7201706	0.492657495	0.037906053	12.9968	1.28E-38	5.72E-38	cyan	0.737033288	3.66E-28	GO:0003676	AT3G09850.1		D111/G-patch domain-containing protein	NA
Sapur.006G039100.1	197.841036	-0.893345611	0.068766853	-12.9909	1.38E-38	6.17E-38	cyan	-0.728657544	2.89E-27		AT4G35910.1		Adenine nucleotide alpha hydrolases-like superfamily protein	cytoplasmic tRNA 2-thiolation protein
Sapur.001G151000.1	9.533583623	2.036251621	0.156816988	12.98489	1.49E-38	6.67E-38	cyan	0.737248351	3.47E-28	GO:0016021	AT2G25737.1		Sulfite exporter TauE/SafE family protein	NA
Sapur.019G072600.1	1348.271271	1.240275662	0.095538696	12.98192	1.55E-38	6.93E-38	cyan	0.70000886	1.97E-24	GO:0050662,GO:0003824,GO:0080019	AT4G33790.1	CER4,FAR3,G7	Jojoba acyl CoA reductase-related male sterility protein	NA
Sapur.002G182100.1	5.403082846	-2.149331706	0.165643922	-12.9756	1.68E-38	7.53E-38	cyan	-0.55274289	6.11E-14	GO:0008017,GO:0007018,GO:0005524,GO:0003777,GO:0005871	AT3G23670.1	KINESIN-12B,PAKRP1L	phragmoplast-associated kinesin-related protein, putative	kinesin motor domain protein
Sapur.014G007600.1	161.4170516	0.809590886	0.06239663	12.97491	1.70E-38	7.59E-38	cyan	0.69295058	8.73E-24	GO:0005515	AT3G01400.1		ARM repeat superfamily protein	E3 ubiquitin ligase PUB14, putative
Sapur.016G172000.1	421.308877	-0.42221557	0.032542147	-12.9744	1.71E-38	7.64E-38	cyan	-0.720247048	2.13E-26	GO:0003676,GO:0006418,GO:0005524,GO:0004812,GO:0000166,GO:0016874	AT4G33760.1		tRNA synthetase class II (D, K and N) family protein	aspartyl-tRNA synthetase
Sapur.001G117200.1	343.5648313	0.825855527	0.063660806	12.97275	1.75E-38	7.81E-38	cyan	0.676357471	2.46E-22		AT1G32690.1			DUF740 family protein
Sapur.016G180400.1	123.3740876	-0.949207533	0.07317958	-12.9709	1.79E-38	8.00E-38	cyan	-0.715446118	6.47E-26		AT4G34215.2		Domain of unknown function (DUF303)	carbohydrate esterase family protein
Sapur.014G143600.1	3427.212958	0.540336441	0.04165985	12.9702	1.81E-38	8.07E-38	cyan	0.709375462	2.54E-25	GO:0033897,GO:0003723	AT2G39780.1	RNS2	ribonuclease 2	ribonuclease T2, putative
Sapur.010G149100.2	23.14462399	1.290255186	0.099488557	12.96888	1.84E-38	8.21E-38	cyan	0.668443645	1.12E-21	GO:0006355,GO:0005634,GO:0003682,GO:0003677	AT3G51880.2	HMGB1,NFD1	high mobility group B1	high mobility group B protein
Sapur.001G075200.1	8.793040178	1.663697459	0.12829028	12.96823	1.85E-38	8.28E-38	cyan	0.656620053	9.94E-21	GO:0030247,GO:0008270,GO:0005515	AT5G53110.1		RING/U-box superfamily protein	RING finger protein
Sapur.018G066800.1	1167.492093	0.543053871	0.041926946	12.95238	2.28E-38	1.02E-37	cyan	0.70749212	3.86E-25	GO:0003676,GO:0006418,GO:0005524,GO:0004812,GO:0000166	AT3G11710.1	ATKRS-1	lysyl-tRNA synthetase 1	lysine-tRNA ligase
Sapur.014G002500.1	161.4297645	-0.726794371	0.056138457	-12.9465	2.46E-38	1.10E-37	cyan	-0.737531406	3.23E-28		AT4G37670.2	NAGS2	N-acetyl-l-glutamate synthase 2	N-acetyl-glutamate synthase
Sapur.15WG090100.1	49.62779986	-0.762768788	0.058932115	-12.9432	2.57E-38	1.15E-37	cyan	-0.689275315	1.86E-23	GO:0006284	AT3G47830.1		DNA glycosylase superfamily protein	NA
Sapur.017G080000.1	2636.156417	0.843143533	0.065167343	12.93813	2.74E-38	1.22E-37	cyan	0.68835356	2.25E-23		AT3G29780.1	RALFL27	ralf-like 27	transmembrane protein, putative
Sapur.011G024600.1	4.675199484	3.797597973	0.293739028	12.92848	3.11E-38	1.39E-37	cyan	0.548721939	1.01E-13	GO:0048544,GO:0006468,GO:0004672	AT1G11330.1		S-locus lectin protein kinase family protein	G-type lectin S-receptor-like Serine/Threonine-kinase
Sapur.T105200.1	49.36037979	1.153888426	0.089262175	12.92696	3.17E-38	1.41E-37	cyan	0.691134519	1.27E-23		AT5G08110.1		nucleic acid binding;ATP-dependent helicases;ATP binding;helicases;ATP-dependent helicases	NA
Sapur.010G044600.1	413.0609203	0.699217238	0.054098262	12.92495	3.26E-38	1.45E-37	cyan	0.671701097	6.04E-22		AT2G33390.1			hypothetical protein
Sapur.010G176200.2	34.10458381	1.223884714	0.094697956	12.92409	3.29E-38	1.47E-37	cyan	0.70820392	3.30E-25	GO:0008270,GO:0005515	AT2G37150.1		RING/U-box superfamily protein	zinc-RING finger protein, putative
Sapur.001G172000.1	159.3626301	-0.676749032	0.052388829	-12.9178	3.57E-38	1.59E-37	cyan	-0.7222788	1.33E-26					NA
Sapur.018G070200.1	8.78042553	2.103596021	0.162856146	12.9169	3.61E-38	1.61E-37	cyan	0.664341865	2.42E-21		AT5G57510.1			DUF761 domain protein
Sapur.006G223700.1	24.425975	2.400694269	0.185882312	12.91513	3.70E-38	1.65E-37	cyan	0.557615584	3.30E-14	GO:0043531	AT4G27190.1		NB-ARC domain-containing disease resistance protein	CC-NBS-LRR resistance protein
Sapur.013G112700.1	354.5313314	-0.512605237	0.0396956	-12.9134	3.78E-38	1.68E-37	cyan	-0.750980308	9.79E-30		AT1G14300.1		ARM repeat superfamily protein	NA
Sapur.007G018100.1	1491.756142	2.063332168	0.159782516	12.91338	3.78E-38	1.68E-37	cyan	0.575464758	3.19E-15	GO:0008168	AT3G11480.1	ATBSMT1,BSMT1	S-adenosyl-L-methionine-dependent methyltransferases superfamily protein	NA
Sapur.002G091100.1	497.146058	0.558638785	0.043267027	12.91142	3.88E-38	1.73E-37	cyan	0.705426949	6.09E-25	GO:0016021	AT1G10030.1	ERG28	homolog of yeast ergosterol28	ergosterol biosynthetic protein
Sapur.018G057000.1	1005.393951	1.105058497	0.085617268	12.90696	4.11E-38	1.83E-37	cyan	0.693284653	8.14E-24		AT1G67910.2			hypothetical protein
Sapur.016G077400.1	35.90566636	1.345272371	0.104231393	12.90659	4.13E-38	1.84E-37	cyan	0.602793512	6.65E-17	GO:0008017,GO:0007018,GO:0005524,GO:0003777,GO:0005871	AT3G45850.1		P-loop containing nucleoside triphosphate hydrolases superfamily protein	kinesin motor domain protein
Sapur.T146200.1	5.797288351	3.659480219	0.283652826	12.90126	4.43E-38	1.97E-37	cyan	0.564054416	1.44E-14					NA
Sapur.001G014400.1	15273.35299	0.8169043	0.063340827	12.89696	4.68E-38	2.08E-37	cyan	0.689550381	1.76E-23	GO:0005515	AT3G20820.1		Leucine-rich repeat (LRR) family protein	DNA-damage-repair/toleration protein DRT100
Sapur.016G108700.1	84.86723903	1.146238789	0.088922333	12.89034	5.10E-38	2.27E-37	cyan	0.67086171	7.09E-22		AT4G00020.2	BRCA2(IV).BRCA2A.EDA20.M	BREAST CANCER 2 like 2A	breast cancer type 2 susceptibility protein brca2 protein, putative
Sapur.T127400.1	2.885395047	2.988044764	0.231882555	12.88603	5.40E-38	2.39E-37	cyan	0.703388166	9.50E-25	GO:0005681,GO:0000398,GO:0000387	AT1G54385.2		ARM repeat superfamily protein	NA
Sapur.15WG008000.1	17.99985435	-1.949420616	0.151283307	-12.8859	5.40E-38	2.40E-37	cyan	-0.595285745	2.00E-16	GO:0005975,GO:0004553	AT5G24318.1		O-Glycosyl hydrolases family 17 protein	glucan endo-1%2C3-beta-glucosidase, putative
Sapur.007G008500.3	6.697609268	-1.698317465	0.131895607	-12.8762	6.13E-38	2.72E-37	cyan	-0.73683572	3.85E-28	GO:0006355,GO:0005634,GO:0003700	AT2G22540.2	AGL22,SVP	K-box region and MADS-box transcription factor family protein	MADS-box protein SVP
Sapur.006G001800.1	409.9682192	-0.576499078	0.044778863	-12.8744	6.28E-38	2.78E-37	cyan	-0.740946337	1.36E-28		AT1G55790.1		Domain of unknown function (DUF2431)	NA
Sapur.008G094000.1	196.1504774	-0.719710253	0.055909214	-12.8728	6.40E-38	2.84E-37	cyan	-0.721527812	1.58E-26	GO:0006468,GO:0005524,GO:0004672	AT1G68830.1	STN7	STT7 homolog STN7	NA
Sapur.010G174200.1	824.9025985	0.801151718	0.062258741	12.8681	6.81E-38	3.01E-37	cyan	0.699358461	2.26E-24	GO:0005525	AT1G07940.1		GTP binding Elongation factor Tu family protein	elongation factor 1-alpha
Sapur.009G046400.1	338.0181775	-0.52234243	0.040636937	-12.8539	8.18E-38	3.62E-37	cyan	-0.741359131	1.22E-28		AT3G60830.1	ARP7,ATARP7	actin-related protein 7	actin-related protein ARP7
Sapur.004G036600.1	3024.238655	-0.452957425	0.035239047	-12.8539	8.18E-38	3.62E-37	cyan	-0.75362818	4.79E-30	GO:0016851,GO:0015995,GO:0015979	AT5G45930.1	CHL I2,CHLI-2,CHLI2	magnesium chelatase i2	magnesium-chelatase subunit ChlI
Sapur.011G079500.1	8.615613908	-1.681944419	0.130919069	-12.8472	8.92E-38	3.94E-37	cyan	-0.703658667	8.95E-25		AT5G53940.1		Yippee family putative zinc-binding protein	yippee zinc-binding/DNA-binding/Mis18, centromere assembly protein
Sapur.010G142300.1	18.92525308	-1.343317292	0.104612006	-12.8409	9.67E-38	4.27E-37	cyan	-0.689084829	1.94E-23	GO:0016758,GO:0008152	AT2G22590.1		UDP-Glycosyltransferase superfamily protein	NA
Sapur.009G114700.1	1970.983438	0.312609503	0.024345822	12.84038	9.74E-38	4.30E-37	cyan	0.71532416	6.65E-26	GO:0009378,GO:0006310,GO:0006281	AT2G27600.1	ATSKD1,SKD1,VPS4	AAA-type ATPase family protein	vacuolar sorting protein 4b
Sapur.013G101500.1	21.07257278	1.252401097	0.097564295	12.83667	1.02E-37	4.51E-37	cyan	0.694323423	6.56E-24	GO:0046983	AT1G35460.1		basic helix-loop-helix (bHLH) DNA-binding superfamily protein	BHLH transcription factor, putative
Sapur.008G141200.2	18.22756834	-1.14783061	0.089443024	-12.8331	1.07E-37	4.72E-37	cyan	-0.700323988	1.84E-24	GO:0005515,GO:0008236,GO:0006508	AT4G17740.1		Peptidase S41 family protein	carboxy-terminal processing peptidase
Sapur.010G005500.1	267.3662495	-0.62240927	0.048500902	-12.8329	1.07E-37	4.73E-37	cyan	-0.718028156	3.57E-26		AT3G06790.2		plastid developmental protein DAG, putative	DAG protein, putative
Sapur.005G080500.1	73.36086583	-1.244640487	0.097004383	-12.8308	1.10E-37	4.87E-37	cyan	-0.708685528	2.97E-25		AT4G35590.1		RWP-RK domain-containing protein	NA
Sapur.010G106500.1	23.6012067	-1.105530047	0.086174407	-12.829	1.13E-37	4.98E-37	cyan	-0.723623515	9.65E-27					NA
Sapur.002G092800.1	280.8134149	-0.541551029	0.042217226	-12.8277	1.15E-37	5.06E-37	cyan	-0.736241643	4.47E-28	GO:0046983	AT1G10120.1		basic helix-loop-helix (bHLH) DNA-binding superfamily protein	helix loop helix DNA-binding domain protein
Sapur.008G053400.1	35.73284257	1.382309068	0.107782884	12.82494	1.19E-37	5.24E-37	cyan	0.63255717	6.32E-19		AT2G40095.1		Alpha/beta hydrolase related protein	alpha/beta hydrolase-like protein
Sapur.018G034400.1	20.86995858	2.215245698	0.172773885	12.82165	1.24E-37	5.47E-37	cyan	0.595277013	2.00E-16		AT3G63520.1	ATCCD1.ATNCED1.CCD1.NC	carotenoid cleavage dioxygenase 1	retinal pigment epithelial membrane protein
Sapur.018G089900.1	380.0134006	0.784984402	0.061252573	12.81553	1.34E-37	5.91E-37	cyan	0.722351635	1.30E-26	GO:0003824,GO:0006470,GO:0004722	AT2G25620.1	AtDBP1,DBP1	DNA-binding protein phosphatase 1	DNA-binding protein phosphatase 2C
Sapur.002G110700.1	245.9637292	0.540907142	0.042212473	12.81392	1.37E-37	6.04E-37	cyan	0.706133821	5.21E-25		AT3G60380.1			transmembrane protein, putative
Sapur.019G069600.1	2615.147504	-1.023632336	0.079894917	-12.8122	1.40E-37	6.17E-37	cyan	-0.763477496	3.09E-31	GO:0045017,GO:0004144	AT5G53390.1		O-acyltransferase (WSD1-like) family protein	Wax ester synthase-like acyl-CoA acyltransferase domain protein
Sapur.007G114400.1	491.677203	0.780870827	0.06096357	12.80881	1.46E-37	6.44E-37	cyan	0.694877561	5.84E-24	GO:0005515,GO:0006468,GO:0004672	AT3G59410.1	GCN2	protein kinase family protein	Serine/Threonine kinase domain protein
Sapur.001G073200.2	13.40172615	-1.334720042	0.104211213	-12.8078	1.48E-37	6.52E-37	cyan	-0.685328293	4.16E-23		AT5G42070.1			hypothetical protein
Sapur.017G016100.1	807.8072969	-0.422700386	0.03302028	-12.8012	1.61E-37	7.10E-37	cyan	-0.720245053	2.14E-26	GO:0051082,GO:0006457,GO:0005783,GO:0005509	AT1G08450.1	AtCRT3,CRT3,EBS2,PSL1	calreticulin 3	calreticulin, putative
Sapur.019G057700.1	2195.106979	0.411403884	0.032149634	12.79653	1.71E-37	7.54E-37	cyan	0.718116127	3.50E-26	GO:0008270,GO:0005515	AT3G16090.1		RING/U-box superfamily protein	E3 ubiquitin-protein ligase synoviolin
Sapur.012G050200.1	133.4548223	-0.736973033	0.057594743	-12.7958	1.73E-37	7.60E-37	cyan	-0.729732375	2.23E-27		AT1G74520.1	ATHVA22A,HVA22A	HVA22 homologue A	HVA22-like protein
Sapur.006G115500.1	1569.172597	0.548042286	0.04283275	12.79494	1.75E-37	7.69E-37	cyan	0.709761317	2.34E-25		AT1G19130.1			cupin family protein
Sapur.016G100800.1	6.752675431	-1.941677	0.151781027	-12.7926	1.80E-37	7.92E-37	cyan	-0.654714914	1.40E-20					NA
Sapur.004G094200.1	2.51710706	3.346542638	0.261679272	12.78872	1.90E-37	8.32E-37	cyan	0.686759542	3.11E-23	GO:0005681,GO:0000398,GO:0000387	AT1G54385.1		ARM repeat superfamily protein	NA
Sapur.011G023500.1	5.987159006	1.66841664	0.130475956	12.78716	1.93E-37	8.49E-37	cyan	0.691576424	1.16E-23	GO:0048544,GO:0006468,GO:0005524,GO:0004672	AT1G11340.1		S-locus lectin protein kinase family protein	NA
Sapur.014G030100.1	147.7883684	0.861967377	0.067435672	12.78207	2.07E-37	9.06E-37	cyan	0.688358558	2.25E-23	GO:0008270	AT1G19270.1	DA1	DA1	zinc ion-binding protein, putative
Sapur.003G119600.1	261.9872982	-0.982649456	0.076879096	-12.7818	2.07E-37	9.09E-37	cyan	-0.729668525	2.26E-27	GO:0055114,GO:0020037,GO:0016705,GO:0005506	AT1G11680.1	CYP51.CYP51A2.CYP51G1.E	CYTOCHROME P450 51G1	cytochrome P450 family obtusifoliol 14-alpha-demethylase
Sapur.009G061900.1	2.685743082	4.630807505	0.362446452	12.77653	2.22E-37	9.72E-37	cyan	0.549281868	9.39E-14		AT1G44080.1		F-box family protein with a domain of unknown function (DUF295)	DUF295 family protein
Sapur.T087000.1	15.81257283	1.635978717	0.128088556	12.77225	2.34E-37	1.03E-36	cyan	0.688781631	2.06E-23		AT5G19820.1	emb2734	ARM repeat superfamily protein	NA
Sapur.014G015100.1	7696.095924	0.474234698	0.037139617	12.76897	2.44E-37	1.07E-36	cyan	0.714238925	8.52E-26	GO:0003723,GO:0003676	AT4G34110.1	ATPAB2,PAB2,PABP2	poly(A) binding protein 2	polyadenylate-binding protein, animal type 1, 2, 3, 4 family protein
Sapur.008G131800.1	106.7835245	-1.491628993	0.116892895	-12.7606	2.72E-37	1.19E-36	cyan	-0.700514452	1.77E-24	GO:0006334,GO:0005634,GO:0003677,GO:0000786	AT2G30620.1		winged-helix DNA-binding transcription factor family protein	histone H1 subtype 5
Sapur.005G181100.2	18.49655117	1.129241378	0.08850722	12.75875	2.79E-37	1.22E-36	cyan	0.710091882	2.17E-25	GO:0006468,GO:0005524,GO:0004672	AT4G28540.1	CKL6,PAPK1	casein kinase I-like 6	casein kinase I
Sapur.017G079000.1	2752.252898	0.757516372	0.059378235	12.75748	2.83E-37	1.24E-36	cyan	0.720076967	2.22E-26		AT1G74160.1			longifolia protein, putative
Sapur.010G159100.1	14.25147502	-1.209059911	0.094777191	-12.7569	2.85E-37	1.25E-36	cyan	-0.709595094	2.42E-25	GO:0031047	AT3G48670.1	IDN2,RDM12	XH/XS domain-containing protein	transcription factor X1-like protein
Sapur.010G178700.1	374.8046922	2.261648625	0.17729972	12.75608	2.88E-37	1.26E-36	cyan	0.521770985	2.43E-12	GO:0008289	AT3G54800.2		Pleckstrin homology (PH) and lipid-binding START domains-containing protein	NA
Sapur.010G100200.1	338.7370444	-0.632269155	0.04957077	-12.7549	2.93E-37	1.28E-36	cyan	-0.716624511	4.94E-26	GO:0006355,GO:0003677	AT1G25580.1	ANAC008,SOG1	NAC (No Apical Meristem) domain transcriptional regulator superfamily protein	NAC domain transcription factor superfamily protein, putative
Sapur.15WG135500.1	1598.032828	-0.491852188	0.038569709	-12.7523	3.03E-37	1.32E-36	cyan	-0.717171779	4.35E-26		AT5G62390.1	ATBAG7,BAG7	BCL-2-associated athanogene 7	BCL-2-associated athanogene protein, putative
Sapur.013G032800.1	105.4441876	-0.748635134	0.058719599	-12.7493	3.14E-37	1.37E-36	cyan	-0.72719109	4.12E-27		AT1G12700.1		ATP binding;nucleic acid binding;helicases	NA
Sapur.15WG131200.1	16.35090922	-0.912475919	0.071575217	-12.7485	3.18E-37	1.39E-36	cyan	-0.730048854	2.06E-27		AT1G31240.1		Bromodomain transcription factor	bromodomain associated protein
Sapur.005G149100.1	333.3382857	-0.587025411	0.046053127	-12.7467	3.25E-37	1.42E-36	cyan	-0.727990219	3.40E-27		AT5G42130.1		Mitochondrial substrate carrier family protein	substrate carrier protein, putative
Sapur.017G069200.1	11.06292644	1.669567189	0.131018916	12.74295	3.41E-37	1.49E-36	cyan	0.59105945	3.66E-16		AT5G15240.1		Transmembrane amino acid transporter family protein	amino acid transporter, putative
Sapur.T078300.1	6.64665873	1.499775134	0.11769772	12.7426	3.43E-37	1.50E-36	cyan	0.727158269	4.15E-27		AT5G06160.1	ATO	splicing factor-related	NA
Sapur.006G191400.1	1939.301765	0.39969799	0.031367882	12.74227	3.44E-37	1.50E-36	cyan	0.684342402	5.07E-23	GO:0055114,GO:0051536	AT5G11770.1		NADH-ubiquinone oxidoreductase 20 kDa subunit, mitochondrial	NADH-ubiquinone oxidoreductase 20 kDa subunit
Sapur.006G220900.1	387.1331471	0.534126663	0.041924239	12.74028	3.53E-37	1.54E-36	cyan	0.720154057	2.18E-26	GO:0055114,GO:0010277,GO:0051537,GO:0016491	AT2G24820.1	TIC55-II	translocon at the inner envelope membrane of chloroplasts 55-II	dioxygenase Rieske iron-sulfur component, putative
Sapur.004G122600.1	33.2405679	-1.007804517	0.079117075	-12.7381	3.63E-37	1.58E-36	cyan	-0.725123136	6.76E-27		AT2G21290.1			hypothetical protein
Sapur.006G149100.1	53.88572491	1.111899302	0.087295125	12.73724	3.67E-37	1.60E-36	cyan	0.667437084	1.36E-21		AT5G08110.1		nucleic acid binding;ATP-dependent helicases;ATP binding;helicases;ATP-dependent helicases	NA
Sapur.005G164800.1	337.3441869	-0.477863971	0.037523554	-12.735	3.78E-37	1.65E-36	cyan	-0.741904809	1.06E-28		AT4G02820.1		Pentatricopeptide repeat (PPR) superfamily protein	PPR containing plant protein
Sapur.15WG077100.1	14.27188194	-1.213575665	0.095299583	-12.7343	3.81E-37	1.66E-36	cyan	-0.704612333	7.27E-25		AT5G60970.1	TCP5	TEOSINTE BRANCHED 1, cycloidea and PCF transcription factor 5	TCP transcription factor
Sapur.003G143000.1	18.07319997	-1.009651422	0.079321584	-12.7286	4.10E-37	1.79E-36	cyan	-0.701056211	1.57E-24	GO:0034968,GO:0018024,GO:0008270,GO:0005634,GO:0005515,GO:0042393	AT1G73100.1	SDG19,SUVH3	SU(VAR)3-9 homolog 3	histone-lysine N-methyltransferase, H3 lysine-9 specific SUVH3 protein
Sapur.003G056500.1	23.47849788	-1.127324303	0.088578107	-12.7269	4.19E-37	1.83E-36	cyan	-0.723112615	1.09E-26		AT1G31430.1		Pentatricopeptide repeat (PPR-like) superfamily protein	NA
Sapur.001G006300.1	6.800029115	3.600465754	0.282908165	12.72662	4.21E-37	1.83E-36	cyan	0.584903692	8.72E-16		AT5G09280.1		Pectin lyase-like superfamily protein	major pollen allergen-like protein
Sapur.016G000600.1	215.7382853	-0.449371033	0.035312766	-12.7255	4.27E-37	1.86E-36	cyan	-0.743840463	6.44E-29	GO:0005975,GO:0004553	AT4G17180.1		O-Glycosyl hydrolases family 17 protein	glucan endo-1%2C3-beta-glucosidase
Sapur.T028200.1	12.81265792	1.533104762	0.120512035	12.72159	4.49E-37	1.95E-36	cyan	0.67154497	6.23E-22		AT5G19820.1	emb2734	ARM repeat superfamily protein	NA
Sapur.15WG001100.1	122.6221188	1.469743872	0.115578685	12.71639	4.79E-37	2.09E-36	cyan	0.638245666	2.45E-19		AT4G01575.1		serine protease inhibitor, Kazal-type family protein	serine-type endopeptidase inhibitor, putative
Sapur.002G104600.1	216.7665203	0.711651279	0.055981094	12.71235	5.05E-37	2.20E-36	cyan	0.704382404	7.65E-25	GO:0033014,GO:0004852	AT2G26540.1	ATDUF3.ATUROS.DUF3.HEM	uroporphyrinogen-III synthase family protein	uroporphyrinogen-III synthase
Sapur.001G181700.1	4.902030874	4.035117952	0.317499616	12.70905	5.27E-37	2.29E-36	cyan	0.595471556	1.94E-16	GO:0055114,GO:0020037,GO:0016705,GO:0005506	AT4G37370.1	CYP81D8	cytochrome P450, family 81, subfamily D, polypeptide 8	cytochrome P450 family protein
Sapur.017G030800.1	13.64033861	1.988762023	0.156543138	12.70424	5.60E-37	2.44E-36	cyan	0.663632013	2.76E-21					hypothetical protein
Sapur.016G163100.1	276.6130876	1.946320526	0.15321661	12.70306	5.69E-37	2.47E-36	cyan	0.615572958	9.57E-18	GO:0006529,GO:0004066	AT3G47340.1	ASN1,AT-ASN1,DIN6	glutamine-dependent asparagine synthase 1	asparagine synthetase [glutamine-hydrolyzing] protein
Sapur.011G104600.1	146.7260951	-0.428946797	0.033786669	-12.6957	6.24E-37	2.72E-36	cyan	-0.716262325	5.37E-26		AT3G15140.1		Polynucleotidyl transferase, ribonuclease H-like superfamily protein	exonuclease
Sapur.018G026500.1	967.7420358	0.924971611	0.072863619	12.69456	6.34E-37	2.76E-36	cyan	0.721896338	1.45E-26	GO:0016747,GO:0008610,GO:0016020,GO:0006633	AT2G26640.1	KCS11	3-ketoacyl-CoA synthase 11	3-ketoacyl-CoA synthase
Sapur.004G038800.1	119.6854862	3.411377942	0.268749619	12.69352	6.42E-37	2.79E-36	cyan	0.537409655	3.96E-13	GO:0008234,GO:0006508	AT5G45890.1	SAG12	senescence-associated gene 12	cysteine protease, putative
Sapur.011G046100.1	4.02482927	-2.297554364	0.181052029	-12.69	6.72E-37	2.92E-36	cyan	-0.687841592	2.50E-23	GO:0048544	AT1G78860.1		D-mannose binding lectin protein with Apple-like carbohydrate-binding domain	epidermis-specific secreted glycoprotein EP1, putative
Sapur.004G132100.1	167.6266376	-0.619855534	0.048863472	-12.6855	7.12E-37	3.09E-36	cyan	-0.723952451	8.93E-27		AT2G16800.1		high-affinity nickel-transport family protein	high-affinity nickel-transport family protein, putative
Sapur.016G210000.1	943.6963063	0.804576717	0.063437961	12.68289	7.36E-37	3.20E-36	cyan	0.651194769	2.62E-20		AT5G40780.1	LHT1	lysine histidine transporter 1	lysine/histidine transporter
Sapur.T066300.1	4.072108268	3.467227123	0.273414834	12.6812	7.52E-37	3.27E-36	cyan	0.641941155	1.31E-19					NA
Sapur.017G123600.1	59.36486784	1.093589391	0.086240245	12.68073	7.56E-37	3.28E-36	cyan	0.690746479	1.38E-23	GO:0005515,GO:0006468,GO:0005524,GO:0004672	AT3G47570.1		Leucine-rich repeat protein kinase family protein	NA
Sapur.006G111300.1	38.21667298	-1.411373058	0.111303049	-12.6805	7.59E-37	3.30E-36	cyan	-0.758312669	1.32E-30	GO:0004857,GO:0042545,GO:0030599,GO:0005618	AT2G26440.1		Plant invertase/pectin methylesterase inhibitor superfamily	pectinesterase/pectinesterase inhibitor, putative
Sapur.016G274200.1	1355.224444	-0.728252149	0.057463553	-12.6733	8.32E-37	3.61E-36	cyan	-0.720772112	1.89E-26	GO:0006412,GO:0005840,GO:0005622,GO:0003735	AT5G54600.1		Translation protein SH3-like family protein	NA
Sapur.15WG023400.1	91.24348649	-0.625063743	0.049323953	-12.6726	8.39E-37	3.64E-36	cyan	-0.725420153	6.30E-27	GO:0005515	AT3G17670.2		tetratricopeptide repeat (TPR)-containing protein	TPR protein
Sapur.016G023200.1	371.2517937	0.68544499	0.054091431	12.67197	8.46E-37	3.67E-36	cyan	0.653438731	1.76E-20	GO:0016020,GO:0006810,GO:0005215	AT3G56950.2	SIP2,SIP2;1	small and basic intrinsic protein 2;1	aquaporin, MIP family%2C SIP subfamily protein
Sapur.15WG071400.1	161.4530606	2.16606598	0.170940988	12.67143	8.52E-37	3.70E-36	cyan	0.568055711	8.56E-15					NA
Sapur.005G206000.1	1.946185845	4.301694057	0.339501769	12.67061	8.60E-37	3.73E-36	cyan	0.505018311	1.53E-11					NA
Sapur.006G223600.1	1530.025392	0.588114253	0.046430997	12.66641	9.08E-37	3.94E-36	cyan	0.686153608	3.52E-23		AT5G24890.1			hypothetical protein
Sapur.004G070500.1	865.7954268	0.499906477	0.039469031	12.66579	9.15E-37	3.97E-36	cyan	0.716701802	4.85E-26	GO:0016020	AT3G01100.1	ATHYP1,HYP1	hypothetical protein 1	early-responsive to dehydration stress protein (ERD4), putative
Sapur.016G209600.1	430.549436	-0.470585126	0.037156551	-12.6649	9.25E-37	4.01E-36	cyan	-0.715518166	6.36E-26		AT5G59440.3	ATTMPK.1,ATTMPK.2,ZEU1	P-loop containing nucleoside triphosphate hydrolases superfamily protein	thymidylate kinase
Sapur.004G069900.1	124.6749416	-0.741758934	0.05858924	-12.6603	9.81E-37	4.25E-36	cyan	-0.74619182	3.49E-29	GO:0051301,GO:0032955	AT1G69390.1	ARC12,ATMINE1,MINE1	homologue of bacterial MinE 1	septum formation topological specificity factor MinE
Sapur.002G181900.1	417.6262168	1.57759211	0.124620164	12.6592	9.95E-37	4.31E-36	cyan	0.645314363	7.32E-20		AT4G14210.1	PDE226,PDS,PDS3	phytoene desaturase 3	chromoplast phytoene desaturase
Sapur.007G111800.1	3.164263179	3.194170539	0.252365429	12.65693	1.02E-36	4.44E-36	cyan	0.694275787	6.62E-24	GO:0030247,GO:0006468,GO:0005524,GO:0004672	AT1G70250.1		receptor serine/threonine kinase, putative	NA
Sapur.016G230800.1	543.5134066	1.476615434	0.116682933	12.65494	1.05E-36	4.55E-36	cyan	0.662905971	3.16E-21	GO:0055085,GO:0042626,GO:0016021,GO:0006810,GO:0005524,GO:0016887	AT3G13080.1	ATMRP3,MRP3	multidrug resistance-associated protein 3	multidrug resistance protein ABC transporter family protein
Sapur.008G014700.1	19.39920407	-1.504026897	0.118849552	-12.6549	1.05E-36	4.55E-36	cyan	-0.680396089	1.11E-22		AT2G36290.1		alpha/beta-Hydrolases superfamily protein	alpha/beta fold hydrolase
Sapur.004G029100.1	75.27708465	1.29574485	0.102406814	12.65292	1.08E-36	4.67E-36	cyan	0.709085047	2.71E-25					NA
Sapur.004G087100.1	9.572302938	-1.387804245	0.109700056	-12.6509	1.11E-36	4.79E-36	cyan	-0.699343113	2.27E-24					NA
Sapur.010G101100.1	157.9601267	-0.427935336	0.033835332	-12.6476	1.15E-36	4.99E-36	cyan	-0.718965198	2.88E-26	GO:0016887,GO:0005524	AT1G26090.1		P-loop containing nucleoside triphosphate hydrolases superfamily protein	anion-transporting ATPase
Sapur.016G054600.1	3.596199204	-3.192102073	0.252389577	-12.6475	1.15E-36	4.99E-36	cyan	-0.644125193	8.99E-20	GO:0005975,GO:0004553	AT3G57270.1	BG1	beta-1,3-glucanase 1	beta-1%2C3-glucanase
Sapur.013G103600.1	68.50802308	-0.590543191	0.046719565	-12.6402	1.27E-36	5.48E-36	cyan	-0.707191226	4.13E-25		AT1G72020.1			NA
Sapur.003G028100.1	912.0374764	0.660205097	0.052245752	12.63653	1.33E-36	5.74E-36	cyan	0.692116848	1.04E-23	GO:0005525	AT1G52280.1	AtRABG3d,RABG3d	RAB GTPase homolog G3D	Rab-related GTPase family protein
Sapur.T086800.1	19.3246002	1.620871096	0.128342245	12.62929	1.46E-36	6.29E-36	cyan	0.678070793	1.76E-22		AT5G19820.1	emb2734	ARM repeat superfamily protein	NA
Sapur.012G066500.1	24647.22265	1.320451241	0.104561066	12.62852	1.47E-36	6.35E-36	cyan	0.680268518	1.14E-22	GO:0055114,GO:0020037,GO:0016705,GO:0005506	AT3G26310.1	CYP71B35	cytochrome P450, family 71, subfamily B, polypeptide 35	NA
Sapur.004G096600.1	5.277269638	3.72803808	0.295208309	12.6285	1.47E-36	6.35E-36	cyan	0.499275348	2.82E-11					hypothetical protein
Sapur.014G136000.1	9.722443504	-1.646829904	0.130426615	-12.6265	1.51E-36	6.51E-36	cyan	-0.702605632	1.13E-24		AT3G13130.1			transmembrane protein, putative
Sapur.016G009100.1	102.9432429	-1.46854996	0.116382518	-12.6183	1.67E-36	7.22E-36	cyan	-0.762829787	3.72E-31	GO:0005515,GO:0006468,GO:0004672	AT1G53440.1		Leucine-rich repeat transmembrane protein kinase	NA
Sapur.002G081800.1	34.39112086	-1.027868211	0.081468911	-12.6167	1.71E-36	7.37E-36	cyan	-0.698276092	2.85E-24	GO:0008270,GO:0005515	AT1G72220.1		RING/U-box superfamily protein	RING finger protein, putative
Sapur.004G040000.1	173.5459026	0.530343572	0.042039686	12.61531	1.75E-36	7.53E-36	cyan	0.699924452	2.00E-24	GO:0008289	AT1G30862.2		Polyketide cyclase/dehydrase and lipid transport superfamily protein	phosphatidylcholine transfer protein, putative
Sapur.007G000800.1	118.5007825	-1.088229677	0.086265588	-12.6149	1.75E-36	7.55E-36	cyan	-0.707774062	3.62E-25	GO:0005515	AT3G50690.1		Leucine-rich repeat (LRR) family protein	Serine/Threonine kinase, plant-type protein%2C putative
Sapur.016G012800.1	4.364200638	-2.127577172	0.168765089	-12.6135	1.78E-36	7.60E-36	cyan	[illegible]	[illegible]					NA
Sapur.013G086800.1	20.93029933	1.543850726	0.122425705	12.61051	1.85E-36	7.96E-36	cyan	0.662034662	3.71E-21	GO:0004518,GO:0006281,GO:0006289,GO:0005634,GO:0004519,GO:0003697	AT3G28030.1	UVH3,UVR1	5'-3' exonuclease family protein	NA
Sapur.007G077700.1	41.71352727	1.819608345	0.143656463	12.60609	1.91E-36	8.12E-36	cyan	0.650572897	9.12E-21	GO:0016758,GO:0008152	AT1G05675.1		UDP-Glycosyltransferase superfamily protein	NA
Sapur.004G056600.1	7.690855197	-1.59139975	0.126240681	-12.6061	1.95E-36	8.24E-36	cyan	-0.699740385	2.08E-24		AT5G17210.1		Protein prenylyltransferase superfamily protein	TPR protein
Sapur.004G143400.1	33.46146544	1.881435963	0.149256542	12.60538	1.97E-36	8.49E-36	cyan	0.564848416	1.30E-14	GO:0008374,GO:0006629	AT3G44830.1		Lecithin:cholesterol acyltransferase family protein	phospholipid:diacylglycerol acyltransferase

Sapur.005G027400.1	4.319369861	3.251355174	0.25807426	12.59853	2.15E-36	9.25E-36	cyan	0.617666154	6.90E-18	GO:0016757	AT1G08990.1	PGSIP5	plant glycogenin-like starch initiation protein 5	NA
Sapur.007G096000.1	264.6241918	-0.738345609	0.058610355	-12.5975	2.18E-36	9.37E-36	cyan	-0.734214326	7.41E-28	GO:0006412,GO:0005840,GO:0005622,GO:0003735	AT5G09770.1		Ribosomal protein L17 family protein	50S ribosomal protein L17
Sapur.005G123800.1	41.22475667	-0.970675558	0.077103116	-12.5893	2.42E-36	1.04E-35	cyan	-0.680684888	1.05E-22	GO:0006468,GO:0004672	AT5G24010.1		Protein kinase superfamily protein	NA
Sapur.005G169200.1	24.45559662	1.332832314	0.105887172	12.58729	2.48E-36	1.07E-35	cyan	0.655643569	1.19E-20		AT1G03070.2		Bax inhibitor-1 family protein	transmembrane BAX inhibitor motif protein, putative
Sapur.014G076600.1	75.61286306	0.765895173	0.060855169	12.58554	2.54E-36	1.09E-35	cyan	0.684494692	4.92E-23		AT4G01000.1		Ubiquitin-like superfamily protein	telomere stability/silencing protein
Sapur.010G140100.1	1349.149023	-0.67992358	0.054036889	-12.5826	2.63E-36	1.13E-35	cyan	-0.720093553	2.21E-26		AT5G36700.1	ATPGLP1,PGLP1	2-phosphoglycolate phosphatase 1	phosphoglycolate phosphatase
Sapur.008G106900.1	471.1242128	0.777609187	0.061808856	12.58087	2.69E-36	1.15E-35	cyan	0.687462599	2.70E-23		AT1G70780.1			senescence-associated protein, putative
Sapur.T089000.1	23.64090695	1.270560991	0.101014519	12.578	2.79E-36	1.20E-35	cyan	0.68974437	1.69E-23		AT4G26190.1		Haloacid dehalogenase-like hydrolase (HAD) superfamily protein	NA
Sapur.010G089900.1	2932.815255	0.517203389	0.041120057	12.57789	2.79E-36	1.20E-35	cyan	0.701022917	1.58E-24	GO:0006096,GO:0004332	AT2G01140.1		Aldolase superfamily protein	fructose-bisphosphate aldolase
Sapur.15WG121000.1	5977.632847	-0.657441536	0.052291029	-12.5727	2.98E-36	1.28E-35	cyan	-0.700084012	1.94E-24	GO:0006355,GO:0005634,GO:0003677,GO:0009725	AT5G62000.3	ARF1-BP,ARF2,HSS,ORE14	auxin response factor 2	auxin response factor, putative
Sapur.003G028800.1	159.3067309	-0.425766334	0.033866161	-12.572	3.01E-36	1.29E-35	cyan	-0.697890063	3.09E-24		AT1G52320.2			BZIP protein,; 48652-45869, putative
Sapur.018G069300.1	15.96717647	-1.076945626	0.085690099	-12.5679	3.17E-36	1.36E-35	cyan	-0.734386764	7.10E-28		AT5G57450.1	ATXRCC3,XRCC3	homolog of X-ray repair cross complementing 3 (XRCC3)	DNA repair protein XRCC3
Sapur.016G272900.1	23.17090077	-1.25753986	0.100061017	-12.5677	3.18E-36	1.36E-35	cyan	-0.680219397	1.15E-22		AT5G54530.1		Protein of unknown function, DUF538	DUF538 family protein
Sapur.001G179900.1	219.3871094	-0.587195385	0.046725739	-12.5669	3.21E-36	1.38E-35	cyan	-0.676369384	2.46E-22		AT1G58100.1		TCP family transcription factor	TCP transcription factor
Sapur.003G165400.1	157.9911551	0.612588666	0.048758623	12.5637	3.34E-36	1.43E-35	cyan	0.680216862	1.15E-22		AT1G55280.1		Lipase/lipooxygenase, PLAT/LH2 family protein	transmembrane protein, putative
Sapur.018G035000.1	271.8553852	0.410525853	0.032679439	12.56221	3.41E-36	1.46E-35	cyan	0.714014016	8.97E-26		AT5G11580.1		Regulator of chromosome condensation (RCC1) family protein	chromosome condensation regulator repeat protein
Sapur.018G041200.1	411.8077229	0.369474032	0.029412428	12.56183	3.42E-36	1.47E-35	cyan	0.710747345	1.87E-25	GO:0070552,GO:0070531,GO:0045739	AT4G32960.1			hypothetical protein
Sapur.016G310500.1	2.446509019	-2.710531774	0.215811876	-12.5597	3.52E-36	1.51E-35	cyan	-0.720401604	2.06E-26	GO:0055085,GO:0016020,GO:0015297,GO:0015238,GO:0006855	AT4G25640.2	ATDTX35,DTX35,FFT	detoxifying efflux carrier 35	NA
Sapur.008G031400.1	27.50235086	-1.045840572	0.083273045	-12.5592	3.54E-36	1.52E-35	cyan	-0.723501993	9.94E-27	GO:0016021	AT3G12090.1	TET6	tetraspanin6	tetraspanin protein
Sapur.012G046500.1	394.6242993	0.430546029	0.03429131	12.55554	3.71E-36	1.59E-35	cyan	0.667684236	1.29E-21	GO:0008270,GO:0005515	AT5G08139.1		RING/U-box superfamily protein	RING finger protein
Sapur.001G068600.1	3.691011696	3.11831118	0.248417721	12.55269	3.84E-36	1.64E-35	cyan	0.623147441	2.91E-18	GO:0008270,GO:0005515	AT5G59550.1		zinc finger (C3HC4-type RING finger) family protein	zinc finger, C3HC4 type (RING finger) protein
Sapur.009G069900.1	2244.09875	0.278505161	0.022195754	12.54768	4.09E-36	1.75E-35	cyan	0.719722009	2.41E-26		AT1G49970.1	CLPR1,NCLPP5,SVR2	CLP protease proteolytic subunit 1	ATP-dependent Clp protease proteolytic subunit
Sapur.016G106600.1	270.4303058	-0.624759305	0.049797683	-12.546	4.18E-36	1.79E-35	cyan	-0.7228531	1.16E-26		AT5G01580.1	OSH1	gamma interferon responsive lysosomal thiol (GILT) reductase family protein	NA
Sapur.008G031200.1	1570.088463	0.873655323	0.069647368	12.54398	4.29E-36	1.83E-35	cyan	0.650782388	2.82E-20	GO:0016021,GO:0006952	AT1G61560.1	ATMLO6,MLO6	Seven transmembrane MLO family protein	MLO-like protein
Sapur.019G064700.1	517.2444117	-1.238256325	0.098717537	-12.5434	4.32E-36	1.84E-35	cyan	-0.694296079	6.59E-24	GO:0055085,GO:0016020,GO:0015297,GO:0015238,GO:0006855	AT3G26590.1		MATE efflux family protein	MATE efflux family protein
Sapur.005G159500.1	35.01448113	-0.709441776	0.056605828	-12.533	4.93E-36	2.10E-35	cyan	-0.712054956	1.40E-25		AT2G42920.1		Pentatricopeptide repeat (PPR-like) superfamily protein	NA
Sapur.019G104700.1	225.4635198	0.59262289	0.047288514	12.53207	4.98E-36	2.13E-35	cyan	0.659442559	5.96E-21	GO:0016747	AT1G03940.1		HXXXD-type acyl-transferase family protein	NA
Sapur.005G145500.1	14.19876221	-1.024788404	0.081781963	-12.5307	5.07E-36	2.16E-35	cyan	-0.686204071	3.49E-23		AT1G43980.1		Tetratricopeptide repeat (TPR)-like superfamily protein	NA
Sapur.003G031300.2	4.453522474	-1.85301491	0.147898181	-12.529	5.18E-36	2.21E-35	cyan	-0.698349386	2.80E-24		AT4G14746.1			hypothetical protein
Sapur.15WG110200.1	1041.903238	0.435078512	0.034727204	12.52846	5.22E-36	2.23E-35	cyan	0.707427487	3.92E-25	GO:0008270,GO:0006508,GO:0004177	AT5G60160.1		Zn-dependent exopeptidases superfamily protein	aspartyl aminopeptidase
Sapur.014G104300.1	13.53056971	-1.397391244	0.11153981	-12.5282	5.23E-36	2.23E-35	cyan	-0.685286511	4.20E-23		AT1G02870.1			ribosome biogenesis protein Nop16
Sapur.019G103000.1	62.12101074	-1.324151177	0.10571012	-12.5262	5.36E-36	2.29E-35	cyan	-0.738593395	2.47E-28	GO:0005789	AT4G21450.1		PapD-like superfamily protein	structural molecule protein, putative
Sapur.003G154600.2	8.54487108	2.261929545	0.180595573	12.52483	5.46E-36	2.33E-35	cyan	0.649971164	3.25E-20	GO:0005544,GO:0005509	AT5G12380.1	ANNAT8	annexin 8	annexin
Sapur.009G070800.1	113.0642207	-0.465394968	0.0371742	-12.5193	5.85E-36	2.50E-35	cyan	-0.719767857	2.39E-26	GO:0070461	AT4G33890.1			transcriptional regulator of RNA polII, SAGA, subunit
Sapur.T157800.1	16.76916705	1.552157615	0.124008877	12.5165	6.06E-36	2.58E-35	cyan	0.620782627	4.23E-18					NA
Sapur.016G142100.1	555.8008136	0.908752247	0.072606727	12.51609	6.10E-36	2.60E-35	cyan	0.677493769	1.97E-22		AT1G07220.1		Arabidopsis thaliana protein of unknown function (DUF821)	glycosyl transferase family 90 protein
Sapur.009G057700.1	684.1255625	1.58064175	0.126337018	12.51131	6.47E-36	2.76E-35	cyan	0.606614448	3.76E-17	GO:0008270,GO:0005515	AT1G35330.1		RING/U-box superfamily protein	RING-H2 zinc finger protein
Sapur.T143100.1	3.847306174	1.962249913	0.156844972	12.51076	6.52E-36	2.78E-35	cyan	0.772409693	2.29E-32					NA
Sapur.012G048200.1	13.69422471	1.437190122	0.114911258	12.50696	6.84E-36	2.91E-35	cyan	0.655619764	1.19E-20					NA
Sapur.017G087000.1	3786.439459	0.402712333	0.032199881	12.50664	6.87E-36	2.92E-35	cyan	0.691948701	1.07E-23	GO:0055114,GO:0004616,GO:0050661,GO:0006098	AT3G02360.1		6-phosphogluconate dehydrogenase family protein	decarboxylating 6-phosphogluconate dehydrogenase
Sapur.016G180900.1	440.063265	-0.693650232	0.055462721	-12.5066	6.87E-36	2.92E-35	cyan	-0.707394184	3.95E-25	GO:0005515	AT2G15320.1		Leucine-rich repeat (LRR) family protein	LRR disease resistance protein, putative
Sapur.001G183600.1	103.9809218	-0.76719992	0.061419634	-12.4911	8.35E-36	3.55E-35	cyan	-0.7071608	4.16E-25	GO:0006310	AT1G12244.1		Polynucleotidyl transferase, ribonuclease H-like superfamily protein	holliday junction resolvase, putative
Sapur.014G109200.1	22.13817111	1.00754271	0.08066524	12.49042	8.42E-36	3.58E-35	cyan	0.698379514	2.79E-24		AT4G03115.1		Mitochondrial substrate carrier family protein	carrier protein
Sapur.017G091200.1	1.384539405	3.947902161	0.316087081	12.48992	8.47E-36	3.60E-35	cyan	0.621759665	3.62E-18					NA
Sapur.016G122300.1	765.6554858	0.963329694	0.077134725	12.48892	8.58E-36	3.64E-35	cyan	0.603513845	5.98E-17		AT2G44500.1		O-fucosyltransferase family protein	GDP-fucose protein O-fucosyltransferase
Sapur.010G156700.1	4.015467033	-2.248025521	0.180012739	-12.4881	8.66E-36	3.68E-35	cyan	-0.672472007	5.21E-22		AT2G37740.1	ATZFP10,ZFP10	zinc-finger protein 10	transcriptional regulator superman protein, putative
Sapur.001G044700.1	380.3951732	-0.529602293	0.04242154	-12.4843	9.10E-36	3.86E-35	cyan	-0.751016251	9.70E-30	GO:0045040,GO:0005742	AT1G27390.1	TOM20-2	translocase outer membrane 20-2	import receptor subunit TOM20
Sapur.016G205200.1	54.02997839	-1.327135898	0.106322992	-12.4821	9.35E-36	3.97E-35	cyan	-0.736352104	4.35E-28	GO:0005524	AT3G27120.1		P-loop containing nucleoside triphosphate hydrolases superfamily protein	P-loop nucleoside triphosphate hydrolase-like protein
Sapur.005G095400.1	486.6602348	-0.279388934	0.02238387	-12.4817	9.39E-36	3.99E-35	cyan	-0.71245923	1.27E-25		AT1G75420.1		UDP-Glycosyltransferase superfamily protein	glycosyltransferase family 4 protein
Sapur.008G135000.1	22.29508509	-2.765517993	0.221612732	-12.4791	9.71E-36	4.12E-35	cyan	-0.646205936	6.28E-20		AT1G08650.1	ATPPCK1,PPCK1	phosphoenolpyruvate carboxylase kinase 1	phosphoenolpyruvate carboxylase kinase
Sapur.T097600.1	6.244540791	1.510222707	0.121031729	12.47791	9.85E-36	4.18E-35	cyan	0.742434566	9.26E-29		AT4G26700.1	ATFIM1,FIM1	fimbrin 1	NA
Sapur.T104800.1	171.5548209	1.20951212	0.096952823	12.47526	1.02E-35	4.32E-35	cyan	0.667866154	1.25E-21		AT5G08110.1		nucleic acid binding;ATP-dependent helicases;ATP binding;helicases;ATP-dependent helicases	NA
Sapur.006G029100.1	20.4937341	0.992611752	0.079572459	12.47431	1.03E-35	4.37E-35	cyan	0.712445892	1.28E-25	GO:0009052,GO:0004751	AT3G04790.1		Ribose 5-phosphate isomerase, type A protein	ribose-5-phosphate isomerase A
Sapur.017G099300.1	31.73623542	-1.012862939	0.081216253	-12.4712	1.07E-35	4.55E-35	cyan	-0.727811939	3.55E-27					NA
Sapur.002G142500.1	176.9418912	-0.98451646	0.07897809	-12.4657	1.15E-35	4.87E-35	cyan	-0.679154707	1.42E-22		AT3G13540.1	ATMYB5,MYB5	myb domain protein 5	MYB transcription factor, putative
Sapur.014G114100.1	13.31210646	-1.208013504	0.096909505	-12.4654	1.15E-35	4.89E-35	cyan	-0.706872069	4.43E-25		AT5G19090.2		Heavy metal transport/detoxification superfamily protein	targeted copper chaperone, putative
Sapur.014G055800.1	25.48554031	1.144139116	0.09178642	12.46523	1.16E-35	4.89E-35	cyan	0.662103056	3.66E-21		AT2G45640.1	ATSAP18,SAP18	SIN3 associated polypeptide P18	Sin3 associated polypeptide p18 (SAP18) protein
Sapur.010G104500.1	39.06441775	-1.91563929	0.153714489	-12.4623	1.20E-35	5.07E-35	cyan	-0.700901766	1.62E-24	GO:0005515,GO:0006468,GO:0005524,GO:0004672	AT3G25560.1	NIK2	NSP-interacting kinase 2	NSP-interacting kinase
Sapur.010G159900.1	6.731459397	-1.541732756	0.123712703	-12.4622	1.20E-35	5.08E-35	cyan	-0.720526062	2.00E-26	GO:0006260,GO:0005634	AT3G12530.1	PSF2	PSF2	DNA replication complex GINS protein psf2
Sapur.003G140500.1	292.0529003	0.557280295	0.0447198	12.4616	1.21E-35	5.12E-35	cyan	0.672427858	5.26E-22		AT2G24100.1			hypothetical protein
Sapur.004G151500.1	420.4247078	-0.453087236	0.036366168	-12.459	1.25E-35	5.28E-35	cyan	-0.712536425	1.25E-25	GO:0016810,GO:0006807	AT2G27450.1	ATNLP1,CPA,NLP1	nitrilase-like protein 1	N-carbamoylputrescine amidohydrolase, putative
Sapur.004G166000.1	30.58266261	-1.387483261	0.111436119	-12.4509	1.38E-35	5.84E-35	cyan	-0.698451893	2.74E-24		AT5G01750.2		Protein of unknown function (DUF567)	tubby C 2 protein
Sapur.012G098700.1	521.6918143	0.644987117	0.051808181	12.44952	1.41E-35	5.95E-35	cyan	0.690444241	1.47E-23	GO:0016020	AT5G51570.1		SPFH/Band 7/PHB domain-containing membrane-associated protein family	hypersensitive-induced response protein
Sapur.013G101200.1	6.288072362	-2.141755852	0.172046859	-12.4487	1.42E-35	6.01E-35	cyan	-0.678371074	1.66E-22	GO:0016020,GO:0006810,GO:0005215	AT1G22540.1		Major facilitator superfamily protein	NA
Sapur.016G278800.1	138.3605777	1.505341093	0.120950696	12.44591	1.47E-35	6.22E-35	cyan	0.649384636	3.60E-20					NA
Sapur.006G127800.1	202.8413635	0.405926365	0.032615342	12.44587	1.47E-35	6.22E-35	cyan	0.684511224	4.90E-23		AT1G21200.1		sequence-specific DNA binding transcription factors	NA
Sapur.004G023400.1	418.5387732	-0.687773426	0.055261806	-12.4457	1.48E-35	6.23E-35	cyan	-0.737680579	3.11E-28		AT1G61255.1			hypothetical protein
Sapur.T034800.1	9.325568194	1.66080822	0.133448009	12.44536	1.48E-35	6.26E-35	cyan	0.6869498	3.00E-23					NA
Sapur.017G085100.1	7.989081212	1.773804555	0.142666335	12.43324	1.73E-35	7.28E-35	cyan	0.684287745	5.13E-23	GO:0007165,GO:0005515,GO:0043531	AT5G17680.1		disease resistance protein (TIR-NBS-LRR class), putative	NA
Sapur.010G096700.1	2076.733062	0.619366615	0.049822666	12.43142	1.76E-35	7.45E-35	cyan	0.664293797	2.44E-21		AT1G13360.1			hypothetical protein
Sapur.010G107300.1	14.8957048	-1.585943495	0.127577605	-12.4312	1.77E-35	7.47E-35	cyan	-0.650947081	2.74E-20		AT1G71691.1		GDSL-like Lipase/Acylhydrolase superfamily protein	GDSL esterase/lipase, putative
Sapur.011G044000.1	179.8895742	-0.973922267	0.078352713	-12.43	1.80E-35	7.58E-35	cyan	-0.699714094	2.10E-24	GO:0016788	AT5G45920.1		SGNH hydrolase-type esterase superfamily protein	GDSL esterase/lipase
Sapur.004G170500.1	25.81916194	-0.758389438	0.061018864	-12.4288	1.82E-35	7.69E-35	cyan	-0.704327084	7.74E-25		AT1G62680.1		Pentatricopeptide repeat (PPR) superfamily protein	NA
Sapur.002G016100.1	37.15777404	-1.149997286	0.092546371	-12.4262	1.88E-35	7.95E-35	cyan	-0.674256224	3.70E-22					hypothetical protein
Sapur.007G018500.1	1191.611199	2.070072391	0.166776544	12.41225	2.24E-35	9.44E-35	cyan	0.584894841	8.62E-16	GO:0008168	AT3G11480.1	ATBSMT1,BSMT1	S-adenosyl-L-methionine-dependent methyltransferases superfamily protein	NA
Sapur.010G067600.1	55.11390088	1.822237806	0.146887319	12.40568	2.43E-35	1.02E-34	cyan	0.594152928	2.35E-16	GO:0016747,GO:0008610,GO:0016020,GO:0006633	AT2G26640.1	KCS11	3-ketoacyl-CoA synthase 11	3-ketoacyl-CoA synthase
Sapur.T134700.1	6.289066021	1.927819714	0.155465159	12.40033	2.60E-35	1.09E-34	cyan	0.604793914	4.94E-17		AT3G10820.2		Transcription elongation factor (TFIIS) family protein	NA
Sapur.017G128300.1	364.0515645	-0.45364134	0.036601492	-12.3941	2.81E-35	1.18E-34	cyan	-0.739151442	2.14E-28	GO:0016592,GO:0006357,GO:0001104	AT3G21350.1	MED6	RNA polymerase transcriptional regulation mediator-related	mediator of RNA polymerase II transcription subunit 6
Sapur.013G032600.1	86.85331645	-0.99266022	0.08009272	-12.3939	2.82E-35	1.19E-34	cyan	-0.691011846	1.30E-23		AT1G12700.1		ATP binding;nucleic acid binding;helicases	NA
Sapur.010G124300.1	1920.816799	-1.207079976	0.097420517	-12.3904	2.95E-35	1.24E-34	cyan	-0.722615999	1.22E-26		AT2G03440.1	ATNRP1,NRP1	nodulin-related protein 1	hypothetical protein
Sapur.008G036700.1	281.361245	-0.740783607	0.059812367	-12.3851	3.15E-35	1.32E-34	cyan	-0.70780035	3.61E-25		AT3G12345.1			FKBP-type peptidyl-prolyl cis-trans isomerase, putative
Sapur.006G077000.1	168.6548017	-0.998467942	0.08062973	-12.3834	3.22E-35	1.35E-34	cyan	-0.720711392	1.92E-26	GO:0006468,GO:0004672	AT3G54030.1		Protein kinase protein with tetratricopeptide repeat domain	receptor kinase, putative
Sapur.010G190400.1	5.715445843	-1.888844019	0.152577969	-12.3795	3.37E-35	1.42E-34	cyan	-0.652384188	2.12E-20		AT4G33950.2	ATOST1.OST1.P44.SNRK2-6	Protein kinase superfamily protein	NA
Sapur.013G112800.1	31.56627737	-0.89419837	0.072244861	-12.3773	3.47E-35	1.45E-34	cyan	-0.714428291	8.16E-26		AT1G06270.1		Pentatricopeptide repeat (PPR) superfamily protein	PPR containing plant protein
Sapur.016G124400.1	839.3398212	0.772279122	0.062402076	12.37586	3.53E-35	1.48E-34	cyan	0.663831076	2.66E-21		AT2G44360.1			hypothetical protein
Sapur.002G007200.1	276.6100299	-0.763217332	0.061678229	-12.3742	3.61E-35	1.51E-34	cyan	-0.681111175	9.67E-23		AT5G42310.1		Pentatricopeptide repeat (PPR-like) superfamily protein	PPR containing plant protein
Sapur.010G001200.1	19.79373573	-1.078975961	0.087202794	-12.3732	3.65E-35	1.53E-34	cyan	-0.695359689	5.28E-24		AT1G09520.1			hypothetical protein
Sapur.019G071000.1	27.87554676	-0.971434516	0.07851859	-12.372	3.70E-35	1.55E-34	cyan	-0.665413982	1.98E-21	GO:0006468,GO:0005524,GO:0004672	AT1G71530.1		Protein kinase superfamily protein	cyclin-dependent kinase-like Serine/Threonine kinase family protein
Sapur.009G084300.1	36.57336191	-1.622092805	0.13113137	-12.37	3.80E-35	1.59E-34	cyan	-0.670291083	7.91E-22					transmembrane protein, putative
Sapur.T151700.1	2.104007443	3.220875367	0.260429389	12.36756	3.92E-35	1.64E-34	cyan	0.676497909	2.39E-22	GO:0005681,GO:0000398,GO:0000387	AT1G54385.1		ARM repeat superfamily protein	NA
Sapur.002G154800.1	136.6478339	-1.189387228	0.096208602	-12.3626	4.17E-35	1.74E-34	cyan	-0.625386592	2.03E-18	GO:0005515	AT2G02930.1	ATGSTF3,GST16,GSTF3	glutathione S-transferase F3	Phi class glutathione transferase GSTF5
Sapur.001G105600.1	181.6541284	-0.703487972	0.056904914	-12.3625	4.17E-35	1.74E-34	cyan	-0.722653625	1.21E-26	GO:0016887,GO:0005524	AT2G03670.1	CDC48B	cell division cycle 48B	AAA family ATPase
Sapur.010G055100.1	90.49663556	-0.671237263	0.054321732	-12.3567	4.48E-35	1.87E-34	cyan	-0.718601518	3.13E-26	GO:0006468,GO:0005524,GO:0004672	AT4G33080.1		AGC (cAMP-dependent, cGMP-dependent and protein kinase C) kinase family protein	NA
Sapur.004G071100.1	10.28421839	-2.024672428	0.16389768	-12.3533	4.67E-35	1.95E-34	cyan	-0.691936824	1.08E-23	GO:0060236,GO:0032147,GO:0005874,GO:0005819	AT5G15510.1		TPX2 (targeting protein for Xklp2) protein family	TPX2 (targeting protein for Xklp2) family protein, putative
Sapur.15WG105100.1	248.0912393	0.372693106	0.030170464	12.35291	4.70E-35	1.96E-34	cyan	0.699888313	2.02E-24	GO:0005515	AT5G50230.1		Transducin/WD40 repeat-like superfamily protein	tipD
Sapur.007G015600.1	438.0438261	-0.60547348	0.049016838	-12.3524	4.73E-35	1.98E-34	cyan	-0.737998242	2.87E-28	GO:0051539,GO:0009451,GO:0003824,GO:0051536	AT4G36390.1		Methylthiotransferase	dimethylallyl, adenosine tRNA methylthiotransferase MiaB
Sapur.012G032900.1	50.86675179	-0.864576614	0.069995623	-12.3519	4.76E-35	1.99E-34	cyan	-0.696927453	3.79E-24	GO:0016758,GO:0008152	AT2G36770.1		UDP-Glycosyltransferase superfamily protein	UDP-glucosyltransferase family protein
Sapur.012G016100.1	27.49733592	0.719461935	0.05827147	12.34673	5.07E-35	2.12E-34	cyan	0.697389789	3.44E-24		AT4G24370.1			NA
Sapur.005G196000.2	418.4961137	0.878424118	0.071163487	12.34375	5.26E-35	2.20E-34	cyan	0.646047218	6.45E-20		AT1G60420.1		DC1 domain-containing protein	nucleoredoxin, putative
Sapur.001G071900.1	45.64318995	1.63406024	0.132539905	12.32882	6.34E-35	2.64E-34	cyan	0.703752436	8.77E-25	GO:0005515	AT4G24020.1	NLP7	NIN like protein 7	transcription factor, putative
Sapur.005G168200.1	156.6948261	-0.751252833	0.060936953	-12.3284	6.37E-35	2.66E-34	cyan	-0.742641125	8.78E-29		AT3G63240.1		DNAse I-like superfamily protein	type I inositol-1%2C4%2C5-trisphosphate 5-phosphatase CVP2
Sapur.T086900.1	19.15802242	1.521694987	0.123462538	12.32516	6.63E-35	2.76E-34	cyan	0.679278924	1.39E-22		AT5G19820.1	emb2734	ARM repeat superfamily protein	NA
Sapur.002G073000.1	540.5663938	1.023517682	0.083074841	12.32043	7.03E-35	2.93E-34	cyan	0.642670515	1.15E-19	GO:0043565,GO:0006355,GO:0003700,GO:0006351	AT1G77920.1		bZIP transcription factor family protein	transcription factor TGA7, putative
Sapur.005G078800.1	4.219981462	2.427707794	0.19707116	12.31894	7.16E-35	2.98E-34	cyan	0.612250835	1.60E-17	GO:0055085,GO:0016021	AT2G17500.2		Auxin efflux carrier family protein	auxin efflux carrier family protein
Sapur.018G042800.1	249.7321526	0.38910071	0.03158622	12.31869	7.19E-35	2.99E-34	cyan	0.724499107	7.84E-27		AT2G06005.1	FIP1	FRIGIDA interacting protein 1	frigida interacting protein
Sapur.008G079500.1	2645.647759	0.354297802	0.028764103	12.31736	7.30E-35	3.04E-34	cyan	0.6797388	1.27E-22	GO:0050660,GO:0008033,GO:0055114,GO:0016491,GO:0045454	AT1G48030.1	mtLPD1	mitochondrial lipoamide dehydrogenase 1	dihydrolipoyl dehydrogenase
Sapur.005G001400.1	68.57264135	1.442547523	0.117121187	12.31671	7.36E-35	3.07E-34	cyan	0.555565021	4.28E-14		AT3G05858.1			hypothetical protein
Sapur.004G114500.1	1.43626465	3.898387724	0.31653916	12.31566	7.46E-35	3.10E-34	cyan	0.589917474	4.31E-16		AT1G49330.1		hydroxyproline-rich glycoprotein family protein	hypothetical protein
Sapur.005G020400.1	148.1453222	0.839636726	0.068187873	12.31358	7.66E-35	3.19E-34	cyan	0.524901519	1.70E-12	GO:0000062	AT3G05420.1	ACBP4	acyl-CoA binding protein 4	acyl-CoA-binding domain protein
Sapur.008G171900.1	292.3805248	-0.651516235	0.05291309	-12.313	7.72E-35	3.21E-34	cyan	-0.7616895	5.13E-31	GO:0008173,GO:0006396,GO:0003723	AT4G15520.1		tRNA/rRNA methyltransferase (SpoU) family protein	TrmH family tRNA/rRNA methyltransferase
Sapur.002G160300.1	704.6692482	0.90968299	0.073881441	12.31274	7.74E-35	3.22E-34	cyan	0.649520282	3.52E-20		AT2G47485.1			hypothetical protein
Sapur.006G208500.4	24.10966425	-1.076864593	0.08746311	-12.3122	7.79E-35	3.24E-34	cyan	-0.711122199	1.72E-25		AT4G32410.1	AtCESA1,CESA1,RSW1	cellulose synthase 1	cellulose synthase A catalytic subunit 1 [UDP-forming] protein
Sapur.008G073500.1	13.11586107	2.259920897	0.183610861	12.30821	8.18E-35	3.40E-34	cyan	0.472660212	4.10E-10		AT1G26600.1	CLE9	CLAVATA3/ESR-RELATED 9	transmembrane protein, putative
Sapur.001G142400.1	2.833428586	3.708437452	0.301307923	12.3078	8.22E-35	3.42E-34	cyan	0.573522776	4.14E-15	GO:0055114,GO:0016491	AT1G52820.1		2-oxoglutarate (2OG) and Fe(II)-dependent oxygenase superfamily protein	NA
Sapur.T028100.1	15.71953468	1.568704026	0.127471298	12.30633	8.37E-35	3.48E-34	cyan	0.681655251	8.68E-23		AT5G19820.1	emb2734	ARM repeat superfamily protein	NA
Sapur.002G044200.1	6163.146901	-0.518615873	0.042144798	-12.3056	8.45E-35	3.51E-34	cyan	-0.668087653	1.20E-21	GO:0019898,GO:0015979,GO:0009654,GO:0009523,GO:0005509	AT1G06680.1	OE23,OEE2,PSBP-1,PSII-P	photosystem II subunit P-1	oxygen-evolving enhancer protein
Sapur.007G116000.1	148.4364946	0.567210534	0.046095946	12.305	8.51E-35	3.54E-34	cyan	0.680101362	1.18E-22	GO:0016021,GO:0004252	AT3G59520.1	ATRBL13,RBL13	RHOMBOID-like protein 13	rhomboid family protein
Sapur.004G108600.1	11.15960508	-1.276821141	0.103786929	-12.3023	8.80E-35	3.66E-34	cyan	-0.677515514	1.96E-22	GO:0055114,GO:0046983,GO:0016620,GO:0008652,GO:0005737,GO:0003942,GO:00512	AT2G19940.1		oxidoreductases, acting on the aldehyde or oxo group of donors, NAD or NADP as acceptor;copper ion binding	N-acetyl-gamma-glutamyl-phosphate reductase
Sapur.003G109500.1	2592.729124	0.482799464	0.039259017	12.2978	9.31E-35	3.86E-34	cyan	0.674184728	3.75E-22	GO:0019236,GO:0007186,GO:0016021	AT5G42090.1		Lung seven transmembrane receptor family protein	lung seven transmembrane receptor
Sapur.016G086200.1	68.51585624	-1.136823661	0.092450667	-12.2965	9.45E-35	3.92E-34	cyan	-0.704460005	7.52E-25		AT1G58100.1		TCP family transcription factor	TCP transcription factor
Sapur.008G131300.1	422.6168084	-0.789928181	0.064260118	-12.2927	9.92E-35	4.12E-34	cyan	-0.695734677	4.88E-24	GO:0055114,GO:0016491,GO:0008270	AT1G23740.1		Oxidoreductase, zinc-binding dehydrogenase family protein	quinone oxidoreductase-like protein
Sapur.T096500.1	38.7905028	-0.623713101	0.050743743	-12.2914	1.01E-34	4.18E-34	cyan	-0.69439415	6.46E-24		AT2G30700.1			NA
Sapur.004G106300.1	209.9480077	1.314749171	0.106972342	12.29055	1.02E-34	4.22E-34	cyan	0.514344244	5.56E-12	GO:0016757,GO:0047262	AT5G15470.1	GAUT14	galacturonosyltransferase 14	CAZy family GT8 glycosyltransferase
Sapur.002G117800.1	1164.120787	0.526481992	0.04284424	12.28828	1.05E-34	4.34E-34	cyan	0.668793933	1.05E-21	GO:0005737,GO:0000045	AT4G16520.1	ATG8F	Ubiquitin-like superfamily protein	gaba(A) receptor-associated protein, putative
Sapur.013G002800.1	49.2756261	-1.227865	0.099943737	-12.2856	1.08E-34	4.49E-34	cyan	-0.701185181	1.53E-24	GO:0010468,GO:0005777	AT5G61190.1		putative endonuclease or glycosyl hydrolase with C2H2-type zinc finger domain	NYN domain protein
Sapur.013G131100.1	606.9206247	-0.569563232	0.046380152	-12.2803	1.16E-34	4.79E-34	cyan	-0.702499264	1.15E-24		AT4G28210.1	emb1923	embryo defective 1923	transmembrane protein, putative
Sapur.008G118700.1	493.4061886	-0.766708681	0.06244063	-12.279	1.17E-34	4.87E-34	cyan	-0.715751722	6.03E-26	GO:0003676	AT1G70200.1		RNA-binding (RRM/RBD/RNP motifs) family protein	RNA recognition motif, a.k.a. RRM%2C RBD protein
Sapur.001G012500.1	1282.063653	1.419932618	0.115650611	12.27778	1.19E-34	4.94E-34	cyan	0.644971128	7.77E-20	GO:0005515,GO:0055114,GO:0046872,GO:0016702	AT3G45140.1	ATLOX2,LOX2	lipoxygenase 2	NA
Sapur.005G085100.1	86.3916558	-0.728520084	0.059340685	-12.2769	1.21E-34	4.99E-34	cyan	-0.727067452	4.24E-27	GO:0005524,GO:0003676	AT4G35740.1	ATRECQ3,RecQl3	DEAD/DEAH box RNA helicase family protein	NA
Sapur.017G048900.1	4.144303932	2.745726511	0.223705971	12.27382	1.25E-34	5.19E-34	cyan	0.606193916	4.00E-17	GO:0006355,GO:0003677	AT5G62380.1	ANAC101,NAC101,VND6	NAC-domain protein 101	NAC domain protein
Sapur.003G087500.1	20.17653868	-1.974329078	0.160877843	-12.2722	1.28E-34	5.29E-34	cyan	-0.694061079	6.93E-24	GO:0005515	AT4G23060.1	IQD22	IQ-domain 22	NA
Sapur.008G026900.1	76.87596034	0.620897317	0.050602597	12.27007	1.31E-34	5.43E-34	cyan	0.66836414	1.14E-21	GO:0006468,GO:0005524,GO:0004672	AT3G57120.1		Protein kinase superfamily protein	tyrosine kinase domain protein
Sapur.006G032600.1	2.489740412	3.79940266	0.309851907	12.262	1.45E-34	6.00E-34	cyan	0.508873128	1.01E-11					hypothetical protein
Sapur.009G067200.1	10.98509071	-1.465503719	0.119523099	-12.2613	1.46E-34	6.05E-34	cyan	-0.730849913	1.70E-27		AT3G19640.1	MGT4,MRS2-3	magnesium transporter 4	magnesium transporter, putative
Sapur.002G062600.1	2.74373189	4.553826515	0.371424306	12.26044	1.48E-34	6.11E-34	cyan	0.546539977	1.32E-13	GO:0030247,GO:0006468,GO:0004672	AT1G21270.1	WAK2	wall-associated kinase 2	tyrosine kinase family protein
Sapur.005G079100.1	839.2582938	0.519011364	0.042334729	12.25971	1.49E-34	6.16E-34	cyan	0.684981981	4.46E-23	GO:0055114,GO:0016616,GO:0051287,GO:0000287	AT4G35260.1	IDH-I,IDH1	isocitrate dehydrogenase 1	NAD-dependent isocitrate dehydrogenase
Sapur.006G110700.1	387.6337251	-0.341810803	0.027881095	-12.2596	1.49E-34	6.17E-34	cyan	-0.694389416	6.47E-24	GO:0016021	AT5G12130.1	ATTERC,PDE149	integral membrane TerC family protein	integral membrane protein, TerC family protein
Sapur.016G123400.1	195.9682285	-0.646786976	0.052771445	-12.2564	1.55E-34	6.42E-34	cyan	-0.71208199	1.39E-25		AT5G13020.1		Emsy N Terminus (ENT)/ plant Tudor-like domains-containing protein	emsy amino-terminus domain protein
Sapur.003G075700.1	4.645634608	-2.079240011	0.169646088	-12.2563	1.55E-34	6.42E-34	cyan	-0.683290443	6.27E-23		AT4G19980.1			NA
Sapur.007G055300.1	7.574945824	-1.606900517	0.131144314	-12.2529	1.62E-34	6.70E-34	cyan	-0.683295277	6.26E-23	GO:0016998,GO:0006032,GO:0004568	AT3G12500.1	ATHCHIB,B-CHI,CHI-B,HCHI	basic chitinase	NA
Sapur.15WG147400.1	177.8336819	0.535526913	0.043711051	12.25152	1.65E-34	6.81E-34	cyan	0.685994728	3.64E-23	GO:0043565,GO:0006355,GO:0005634,GO:0003700	AT5G62020.1	AT-HSFB2A,HSFB2A	heat shock transcription factor B2A	heat shock transcription factor, putative
Sapur.006G017300.1	76.46841378	1.149795051	0.093955329	12.23768	1.96E-34	8.08E-34	cyan	0.668734201	1.06E-21		AT2G32210.1			cysteine-rich TM module stress tolerance protein
Sapur.003G158700.1	1720.420095	-0.705599963	0.057662953	-12.2366	1.98E-34	8.18E-34	cyan	-0.72038801	2.07E-26	GO:0005515	AT3G20820.1		Leucine-rich repeat (LRR) family protein	DNA-damage-repair/toleration protein DRT100
Sapur.002G026400.1	65.64846796	1.43294317	0.117109661	12.23591	2.00E-34	8.25E-34	cyan	0.607509073	3.29E-17		AT1G19530.1			hypothetical protein
Sapur.007G048900.1	10.40942193	1.530923215	0.125137417	12.23394	2.05E-34	8.45E-34	cyan	0.649454004	3.56E-20	GO:0003824,GO:0006470,GO:0004722	AT4G33920.1		Protein phosphatase 2C family protein	protein phosphatase 2C, putative
Sapur.15WG044000.1	57.68200562	0.827802185	0.067682976	12.23058	2.13E-34	8.80E-34	cyan	0.684718097	4.70E-23					hypothetical protein
Sapur.016G078200.1	6.753971529	3.375800489	0.276025522	12.23003	2.15E-34	8.86E-34	cyan	0.55577361	4.17E-14	GO:0009611,GO:0004867	AT2G38870.1		Serine protease inhibitor, potato inhibitor I-type family protein	protease inhibitor
Sapur.008G114600.1	639.328075	0.628446653	0.051395271	12.22771	2.21E-34	9.12E-34	cyan	0.679358129	1.37E-22	GO:0008137,GO:0005739,GO:0003954	AT2G02050.1		NADH-ubiquinone oxidoreductase B18 subunit, putative	NADH-ubiquinone oxidoreductase B18 subunit
Sapur.011G040200.1	26.83992736	-0.815181133	0.066670617	-12.227	2.23E-34	9.20E-34	cyan	-0.717046588	4.48E-26		AT5G46100.1		Pentatricopeptide repeat (PPR) superfamily protein	PPR containing plant protein
Sapur.002G068600.1	379.8186717	0.497769668	0.040715799	12.22547	2.27E-34	9.37E-34	cyan	0.687336556	2.77E-23		AT1G77660.1		Histone H3 K4-specific methyltransferase SET7/9 family protein	phosphatidylinositol-4-phosphate 5-kinase, putative
Sapur.003G108700.2	37.62009485	0.7643832	0.062550222	12.22031	2.42E-34	9.98E-34	cyan	0.716347624	5.26E-26		AT5G41950.1		Tetratricopeptide repeat (TPR)-like superfamily protein	Hop-interacting protein THI002
Sapur.017G096200.1	41.41787392	-0.854621909	0.06994344	-12.2188	2.47E-34	1.02E-33	cyan	-0.695448985	5.18E-24		AT2G27770.1		Plant protein of unknown function (DUF868)	DUF868 family protein
Sapur.15WG004400.1	56.47425809	-0.684336403	0.056017022	-12.2166	2.54E-34	1.04E-33	cyan	-0.697040791	3.70E-24		AT5G64180.1			SKIP interacting protein, putative
Sapur.016G220700.1	41.08981216	2.084986901	0.170689718	12.21507	2.58E-34	1.06E-33	cyan	0.605200556	4.65E-17	GO:0016758,GO:0008152	AT2G16890.2		UDP-Glycosyltransferase superfamily protein	UDP-glycosyltransferase
Sapur.012G106000.1	38.27805487	-1.655069151	0.135497782	-12.2147	2.59E-34	1.07E-33	cyan	-0.624480396	2.35E-18					transmembrane protein, putative
Sapur.T047800.1	1857.573381	0.766463052	0.062758288	12.21294	2.65E-34	1.09E-33	cyan	0.698032342	3.00E-24	GO:0016887,GO:0005524,GO:0055085,GO:0042626,GO:0016021,GO:0006810	AT3G60160.1	ATMRP9,MRP9	multidrug resistance-associated protein 9	multidrug resistance protein ABC transporter family protein
Sapur.004G156900.1	317.7015372	-0.385309794	0.03155122	-12.2122	2.68E-34	1.10E-33	cyan	-0.708480919	3.10E-25	GO:0008270,GO:0006355	AT4G38180.1	FRS5	FAR1-related sequence 5	FAR1-related sequence protein
Sapur.002G066400.1	18.01972357	1.010745469	0.082781794	12.20976	2.76E-34	1.13E-33	cyan	0.640642338	1.63E-19		AT1G44414.1			zinc-ribbon domain protein
Sapur.002G180900.1	512.0715931	0.427211092	0.034991096	12.20914	2.78E-34	1.14E-33	cyan	0.692166375	1.03E-23	GO:0005515	AT3G13340.1		Transducin/WD40 repeat-like superfamily protein	plant/MDC11-13 protein, putative
Sapur.018G110500.1	1.533342317	4.066899256	0.333111332	12.20883	2.79E-34	1.15E-33	cyan	0.577846648	2.31E-15		AT2G34400.1		Pentatricopeptide repeat (PPR-like) superfamily protein	NA
Sapur.016G094200.1	1.811554202	3.609353258	0.295699698	12.20614	2.88E-34	1.19E-33	cyan	0.576173394	2.89E-15					NA
Sapur.001G070900.1	391.106946	0.522111384	0.042776271	12.20563	2.90E-34	1.19E-33	cyan	0.68619192	3.49E-23	GO:0005991,GO:0004555	AT4G24040.1	ATTRE1,TRE1	trehalase 1	trehalase
Sapur.011G114300.2	7.781251263	-1.645407819	0.134809174	-12.2055	2.91E-34	1.20E-33	cyan	-0.685683347	3.87E-23	GO:0016597,GO:0008152	AT2G36840.1		ACT-like superfamily protein	ACT domain protein
Sapur.016G091700.1	308.8172109	-0.508678952	0.041690107	-12.2014	3.05E-34	1.26E-33	cyan	-0.707317649	4.02E-25		AT1G04590.1			PPR containing protein
Sapur.019G008700.2	11.21246026	-1.370937649	0.112388089	-12.1982	3.18E-34	1.31E-33	cyan	-0.668705157	1.07E-21		AT1G72160.1		Sec14p-like phosphatidylinositol transfer family protein	patellin-3 protein, putative
Sapur.009G005400.1	6.26657173	1.959748824	0.160662961	12.19789	3.19E-34	1.31E-33	cyan	0.659567148	5.83E-21	GO:0006355,GO:0003677	AT2G27990.1	BLH8,PNF	BEL1-like homeodomain 8	BEL1 homeotic protein, putative
Sapur.010G159600.2	117.7748395	-0.811454815	0.066577274	-12.1882	3.59E-34	1.48E-33	cyan	-0.672464309	5.22E-22		AT3G55460.1	SCL30	SC35-like splicing factor 30	serine/arginine-rich splicing factor SRSF10 RNA recognition motif protein
Sapur.016G092300.1	127.6819556	-0.586396681	0.048169485	-12.1736	4.30E-34	1.76E-33	cyan	-0.721375074	1.64E-26		AT2G37920.1	emb1513	copper ion transmembrane transporters	ATP-binding protein, putative
Sapur.002G065900.1	1122.445023	0.457932213	0.037637119	12.16704	4.66E-34	1.91E-33	cyan	0.677709779	1.89E-22	GO:0005515	AT1G21760.1	ATFBP7,FBP7	F-box protein 7	F-box only protein
Sapur.005G039200.1	1787.746399	0.867182036	0.071303444	12.16185	4.96E-34	2.03E-33	cyan	0.647671972	4.86E-20		AT3G04870.2	PDE181,SPC1,ZDS	zeta-carotene desaturase	NA
Sapur.014G057100.1	575.5471159	0.320697019	0.026371049	12.16095	5.02E-34	2.06E-33	cyan	0.70506029	6.60E-25	GO:0016887,GO:0005524	AT1G01910.1		P-loop containing nucleoside triphosphate hydrolases superfamily protein	ATPase ASNA1-like protein
Sapur.006G176200.1	41.75532385	1.181212391	0.097131716	12.16093	5.02E-34	2.06E-33	cyan	0.65098174	2.72E-20		AT5G02640.1			hypothetical protein
Sapur.013G086900.1	4.072612459	2.155588243	0.177276634	12.15946	5.11E-34	2.09E-33	cyan	0.662928022	3.14E-21					NA
Sapur.007G064300.1	34.3575234	-1.378095422	0.113411339	-12.1513	5.65E-34	2.31E-33	cyan	-0.65017754	3.13E-20	GO:0006470,GO:0004722	AT4G31860.1		Protein phosphatase 2C family protein	NA
Sapur.017G043200.1	72.18346331	0.891819844	0.073410987	12.14832	5.86E-34	2.40E-33	cyan	0.693142812	8.39E-24	GO:0006468,GO:0004672,GO:0005524	AT2G32800.1	AP4.3A	protein kinase family protein	receptor-like kinase
Sapur.004G020200.1	3.372544999	2.386506822	0.196467318	12.14709	5.94E-34	2.43E-33	cyan	0.65350829	1.74E-20					NA
Sapur.006G032200.1	570.9137352	0.616233328	0.050731581	12.14694	5.96E-34	2.44E-33	cyan	0.686631383	3.20E-23	GO:0006629,GO:0004806	AT5G04040.1	SDP1	Patatin-like phospholipase family protein	patatin family phospholipase
Sapur.014G061300.1	148.3578432	-0.823848989	0.067846835	-12.1428	6.27E-34	2.56E-33	cyan	-0.747643764	2.38E-29					transmembrane protein, putative
Sapur.001G054900.1	32.52172619	1.758150701	0.144830579	12.13936	6.53E-34	2.67E-33	cyan	0.596852526	1.59E-16	GO:0071805,GO:0016020,GO:0015079	AT4G13420.1	ATHAK5,HAK5	high affinity K+ transporter 5	NA
Sapur.016G154700.1	1062.583561	1.263125709	0.104060627	12.13836	6.61E-34	2.71E-33	cyan	0.65747034	8.53E-21					transmembrane protein, putative
Sapur.005G100700.1	12.57579133	2.221883929	0.18306644	12.13704	6.72E-34	2.75E-33	cyan	0.517048062	4.12E-12	GO:0045735	AT2G18540.1		RmlC-like cupins superfamily protein	NA
Sapur.013G104800.1	440.6233874	-0.578765838	0.047689246	-12.1362	6.79E-34	2.78E-33	cyan	-0.671304494	6.52E-22	GO:0005840	AT1G35680.1		Ribosomal protein L21	50S ribosomal protein L21
Sapur.010G140200.1	820.063532	0.381300271	0.031419314	12.13586	6.82E-34	2.79E-33	cyan	0.704471323	7.50E-25	GO:0016567,GO:0004842	AT1G65430.1	ARI8,ATARI8	IBR domain-containing protein	E3 ubiquitin-protein ligase ARI8, putative
Sapur.006G110500.1	803.6368786	0.869035311	0.071663655	12.12658	7.64E-34	3.12E-33	cyan	0.60951276	2.43E-17	GO:0005515	AT5G02790.1	GSTL3	Glutathione S-transferase family protein	glutathione S-transferase
Sapur.016G159100.1	109.7005603	1.380349255	0.113850367	12.12424	7.86E-34	3.21E-33	cyan	0.626512702	1.69E-18					NA
Sapur.018G090300.1	2.779644337	-2.605976582	0.21507311	-12.1167	8.62E-34	3.52E-33	cyan	-0.721164926	1.72E-26		AT2G12646.1		PLATZ transcription factor family protein	zinc ion-binding protein, putative
Sapur.010G026300.1	141.0118404	-0.626009669	0.05166728	-12.1162	8.67E-34	3.54E-33	cyan	-0.753639192	4.78E-30		AT1G70550.1		Protein of Unknown Function (DUF239)	carboxyl-terminal proteinase-like protein
Sapur.006G056300.3	14.0513986	1.418068608	0.117057736	12.11427	8.88E-34	3.62E-33	cyan	0.637569415	2.74E-19		AT2G26670.1	ATHO1.GUN2.HO1.HY1.HY6	Plant haem oxygenase (decyclizing) family protein	heme oxygenase
Sapur.T050400.1	241.7819216	-0.585912932	0.048369467	-12.1133	8.98E-34	3.66E-33	cyan	-0.672179466	5.51E-22		AT4G28740.1			plant/F16A16-150 protein, putative
Sapur.T036100.1	1.196175429	3.669924208	0.30313753	12.10647	9.76E-34	3.98E-33	cyan	0.596476167	1.68E-16					NA
Sapur.006G044100.1	1.579695546	3.717406958	0.307213388	12.10041	1.05E-33	4.28E-33	cyan	0.619734324	4.99E-18	GO:0007275,GO:0006355,GO:0003700	AT1G51190.1	PLT2	Integrase-type DNA-binding superfamily protein	hypothetical protein
Sapur.010G067300.1	65.38977696	1.813806042	0.149921861	12.09834	1.08E-33	4.39E-33	cyan	0.575334128	3.24E-15	GO:0016747,GO:0008610,GO:0016020,GO:0006633	AT2G26640.1	KCS11	3-ketoacyl-CoA synthase 11	NA
Sapur.014G141800.1	5919.610235	-0.521234758	0.043097255	-12.0944	1.13E-33	4.60E-33	cyan	-0.676297441	2.49E-22	GO:0016020,GO:0009765	AT1G61520.3	LHCA3	photosystem I light harvesting complex gene 3	light-harvesting complex I chlorophyll A/B-binding protein
Sapur.005G200500.2	10.86802455	-1.097245802	0.090729601	-12.0936	1.14E-33	4.65E-33	cyan	-0.683243335	6.32E-23		AT5G42310.1		Pentatricopeptide repeat (PPR-like) superfamily protein	PPR containing plant protein
Sapur.013G037000.1	803.770602	-0.729264634	0.060307831	-12.0924	1.16E-33	4.72E-33	cyan	-0.688282502	2.28E-23		AT5G28300.1		Duplicated homeodomain-like superfamily protein	trihelix transcription factor, putative
Sapur.016G259600.1	35.39696355	0.916867444	0.075823377	12.09215	1.16E-33	4.73E-33	cyan	0.707312977	4.02E-25		AT4G27470.1	ATRMA3,RMA3	RING membrane-anchor 3	zinc-RING finger protein
Sapur.009G031100.1	203.2930206	-0.795284713	0.065768863	-12.0921	1.16E-33	4.73E-33	cyan	-0.725753104	5.82E-27		AT5G58920.1			hypothetical protein
Sapur.005G018300.1	1181.491681	0.348154798	0.028799294	12.089	1.21E-33	4.91E-33	cyan	0.69127912	1.23E-23	GO:0008270,GO:0005515	AT3G05545.1		RING/U-box superfamily protein	zinc finger, C3HC4 type (RING finger) protein
Sapur.009G031300.1	42.08587146	-0.916726835	0.075851642	-12.0858	1.26E-33	5.11E-33	cyan	-0.702620865	1.12E-24	GO:0016020,GO:0006810,GO:0005215	AT3G54140.1	ATPTR1,PTR1	peptide transporter 1	NA
Sapur.006G218000.1	52.28773774	-0.600777171	0.049732995	-12.0801	1.35E-33	5.47E-33	cyan	-0.69130765	1.23E-23	GO:0043565,GO:0006355,GO:0003700	AT4G30935.1	ATWRKY32,WRKY32	WRKY DNA-binding protein 32	WRKY transcription factor
Sapur.007G053400.1	902.0403746	0.855371131	0.07082198	12.07776	1.38E-33	5.63E-33	cyan	0.655891909	1.13E-20	GO:0008152,GO:0003824,GO:0016114,GO:0008661	AT4G15560.1	CLA,CLA1,DEF,DXPS2,DXS	Deoxyxylulose-5-phosphate synthase	1-deoxy-D-xylulose-5-phosphate synthase
Sapur.010G072600.1	2.993911225	3.046181484	0.252250202	12.07603	1.41E-33	5.75E-33	cyan	0.645313861	7.32E-20	GO:0055085,GO:0016021,GO:0015299,GO:0006812	AT2G01980.1	ATNHX7,ATSOS1,SOS1	sodium proton exchanger, putative (NHX7) (SOS1)	NA
Sapur.007G064700.1	17.77250193	0.998994268	0.082741796	12.07364	1.46E-33	5.91E-33	cyan	0.699928592	2.00E-24		AT1G50140.1		P-loop containing nucleoside triphosphate hydrolases superfamily protein	NA
Sapur.007G009100.1	322.7429481	-0.551686059	0.045700393	-12.0718	1.49E-33	6.05E-33	cyan	-0.701136733	1.54E-24	GO:0055085,GO:0016020,GO:0006810,GO:0005215	AT4G38050.1		Xanthine/uracil permease family protein	nucleobase ascorbate transporter
Sapur.016G005500.1	85.39525656	-0.766843841	0.063535323	-12.0696	1.53E-33	6.21E-33	cyan	-0.698810247	2.54E-24		AT4G14540.1	NF-YB3	nuclear factor Y, subunit B3	nuclear transcription factor Y subunit B-3
Sapur.001G026100.1	1.595180841	3.431900912	0.284347397	12.06939	1.53E-33	6.22E-33	cyan	0.626044328	1.83E-18	GO:0016021	AT5G47530.1		Auxin-responsive family protein	eukaryotic cytochrome b561 protein
Sapur.014G112700.1	12.28104569	-1.09827846	0.091000939	-12.0689	1.54E-33	6.26E-33	cyan	-0.713323622	1.05E-25		AT3G63095.1		Tetratricopeptide repeat (TPR)-like superfamily protein	hypothetical protein
Sapur.004G073400.1	5.376928807	2.747053135	0.22761624	12.06879	1.54E-33	6.27E-33	cyan	0.59409466	2.37E-16	GO:0031225,GO:0016049,GO:0010215	AT5G15630.1	COBL4,IRX6	COBRA-like extracellular glycosyl-phosphatidyl inositol-anchored protein family	COBRA-like protein
Sapur.007G049000.1	2038.511033	0.627768867	0.052050223	12.06083	1.70E-33	6.90E-33	cyan	0.639945897	1.84E-19		AT4G37470.1		alpha/beta-Hydrolases superfamily protein	sigma factor sigb regulation protein rsbq protein
Sapur.003G087100.1	27.67504003	-1.937260257	0.160674874	-12.057	1.78E-33	7.22E-33	cyan	-0.701569826	1.41E-24	GO:0005515	AT4G23060.1	IQD22	IQ-domain 22	NA
Sapur.T098300.1	5.618173946	1.926857384	0.159824205	12.0561	1.80E-33	7.30E-33	cyan	0.678605742	1.59E-22		AT4G15110.1	CYP97B3	cytochrome P450, family 97, subfamily B, polypeptide 3	NA
Sapur.002G101800.1	13.38975165	-1.474608936	0.122320177	-12.0553	1.82E-33	7.37E-33	cyan	-0.668051929	1.21E-21	GO:0043565,GO:0006355,GO:0005634,GO:0003700	AT1G46264.1	AT-HSFB4,HSFB4,SCZ	heat shock transcription factor B4	heat shock transcription factor
Sapur.007G106600.1	23.19204334	-1.940580271	0.161015804	-12.0521	1.89E-33	7.66E-33	cyan	-0.748403473	1.95E-29		AT2G32280.1		Protein of unknown function (DUF1218)	transmembrane protein, putative
Sapur.003G087600.1	141.3793718	1.353157362	0.112290494	12.05051	1.93E-33	7.81E-33	cyan	0.635042513	4.18E-19	GO:0016021,GO:0004252,GO:0006508	AT1G63120.1	ATRBL2,RBL2	RHOMBOID-like 2	NA
Sapur.006G100800.1	1224.451038	0.526002704	0.043677259	12.04294	2.11E-33	8.56E-33	cyan	0.658740654	6.77E-21		AT5G03460.1			transmembrane protein, putative
Sapur.019G080400.1	337.1481445	0.459635885	0.038173248	12.04079	2.17E-33	8.79E-33	cyan	0.705800563	5.61E-25	GO:0008270,GO:0005515	AT1G71980.1		Protease-associated (PA) RING/U-box zinc finger family protein	protease-associated and C3HC4-type RING finger protein
Sapur.014G052900.1	1753.819484	-0.823644358	0.068419064	-12.0382	2.24E-33	9.06E-33	cyan	-0.740503297	1.52E-28		AT3G60900.1	FLA10	FASCICLIN-like arabinogalactan-protein 10	fasciclin-like arabinogalactan protein
Sapur.004G054700.1	3656.386337	0.636376361	0.052868495	12.03697	2.27E-33	9.20E-33	cyan	0.675112375	3.13E-22		AT1G65820.1		microsomal glutathione s-transferase, putative	microsomal glutathione S-transferase
Sapur.003G087300.1	26.97006236	-1.925094569	0.160015905	-12.0306	2.45E-33	9.93E-33	cyan	-0.699529264	2.18E-24	GO:0005515	AT4G23060.1	IQD22	IQ-domain 22	NA
Sapur.001G072300.1	1291.331997	-0.597920157	0.049700659	-12.0304	2.46E-33	9.95E-33	cyan	-0.681225566	9.45E-23	GO:0019843,GO:0006412,GO:0005840,GO:0003735	AT1G64510.1		Translation elongation factor EF1B/ribosomal protein S6 family protein	30S ribosomal protein S6
Sapur.016G159000.1	6.233066005	-2.319128527	0.192890992	-12.023	2.69E-33	1.09E-32	cyan	-0.678162792	1.73E-22	GO:0043565,GO:0006355,GO:0005634,GO:0003700	AT1G46264.1	AT-HSFB4,HSFB4,SCZ	heat shock transcription factor B4	heat stress transcription factor B-4
Sapur.011G054700.1	920.6734479	0.588459012	0.048952537	12.02101	2.76E-33	1.11E-32	cyan	0.669109512	9.89E-22		AT1G30280.1		Chaperone DnaJ-domain superfamily protein	cyclin-G-associated kinase, putative
Sapur.019G075200.1	11.81936562	1.151945642	0.095840598	12.01939	2.81E-33	1.14E-32	cyan	0.68638831	3.36E-23		AT1G33980.1	ATUPF3,UPF3	Smg-4/UPF3 family protein	regulator of nonsense transcripts UPF3 protein, putative
Sapur.012G007500.1	7.979254191	-1.915253542	0.159350107	-12.0192	2.82E-33	1.14E-32	cyan	-0.647002257	5.46E-20	GO:0006468,GO:0005524,GO:0004672	AT1G10940.2	ASK1.SNRK2-4.SNRK2.4.SRK	Protein kinase superfamily protein	NA
Sapur.009G100100.1	34.42305482	-0.982209654	0.081745267	-12.0155	2.95E-33	1.19E-32	cyan	-0.714173073	8.65E-26	GO:0009733	AT1G75590.1		SAUR-like auxin-responsive protein family	NA
Sapur.013G050500.1	57.32894741	-0.68426468	0.056957943	-12.0135	3.02E-33	1.22E-32	cyan	-0.725763979	5.80E-27		AT5G15010.1		Tetratricopeptide repeat (TPR)-like superfamily protein	PPR containing plant protein
Sapur.012G017200.1	9.288812238	1.48436495	0.12357854	12.01151	3.09E-33	1.25E-32	cyan	0.672152656	5.54E-22	GO:0003824,GO:0006470,GO:0004722	AT5G53140.1		Protein phosphatase 2C family protein	protein phosphatase 2C
Sapur.014G010800.1	27347.30336	0.915423812	0.076217349	12.0107	3.12E-33	1.26E-32	cyan	0.679055998	1.45E-22		AT5G59845.1		Gibberellin-regulated family protein	gibberellin-regulated protein
Sapur.003G153900.4	79.93041605	0.902783021	0.075166308	12.01047	3.13E-33	1.26E-32	cyan	0.724716853	7.45E-27	GO:0005515,GO:0005096	AT3G07940.1		Calcium-dependent ARF-type GTPase activating protein family	ADP-ribosylation factor GTPase-activating protein AGD11, putative
Sapur.T060000.1	8.776750891	1.639803569	0.136531779	12.01042	3.13E-33	1.27E-32	cyan	0.677268951	2.06E-22					NA
Sapur.15WG071200.1	47.36431481	1.94585308	0.162090248	12.00475	3.35E-33	1.36E-32	cyan	0.596233514	1.74E-16		AT3G23580.1	RNR2A,RNR2A	ribonucleotide reductase 2A	NA
Sapur.T111000.1	4.606414888	1.89251443	0.157669954	12.00301	3.43E-33	1.38E-32	cyan	0.684840748	4.39E-23					NA
Sapur.009G124200.1	47.01209583	0.856792198	0.071433235	11.99431	3.81E-33	1.53E-32	cyan	0.682551572	7.30E-23		AT2G07090.1		Protein of unknown function (DUF630 and DUF632)	bZIP transcription factor bZIP114, putative
Sapur.016G051800.1	48.17254104	2.13399821	0.177918228	11.99426	3.81E-33	1.54E-32	cyan	0.512410923	6.88E-12	GO:0007165,GO:0005515,GO:0043531,GO:0000817,GO:0007018,GO:0005524,GO:00037	AT5G27550.1		P-loop containing nucleoside triphosphate hydrolases superfamily protein	nucleoporin-like protein
Sapur.016G101600.1	408.3339287	-0.803109958	0.06711688	-11.9925	3.89E-33	1.56E-32	cyan	-0.662108769	3.40E-25		AT5G08140.1		nucleoporin-related	nucleoporin-like protein
Sapur.T105600.1	16.52635211	1.160008346	0.096730864	11.99212	3.91E-33	1.58E-32	cyan	0.67561828	3.30E-23		AT5G08110.1		nucleic acid binding;ATP-dependent helicases;ATP binding;helicases;ATP-dependent helicases	NA
Sapur.T139100.1	20.49412288	1.573206004	0.131187727	11.99202	3.91E-33	1.58E-32	cyan	0.67651429	2.82E-22		AT5G19820.1	emb2734	ARM repeat superfamily protein	NA

Sapur.010G062900.1	68.52249545	0.890576886	0.074298698	11.98644	4.19E-33	1.69E-32	cyan	0.652533853	2.07E-20	GO:0008080	AT3G22560.1		Acyl-CoA N-acyltransferases (NAT) superfamily protein	acetyltransferase (GNAT) domain protein
Sapur.019G082700.1	57.99547686	-0.780374857	0.065113609	-11.9848	4.27E-33	1.72E-32	cyan	-0.698887881	2.50E-24		AT1G34370.1	STOP1	C2H2 and C2HC zinc fingers superfamily protein	zinc finger, C2H2 type protein
Sapur.006G081300.1	4809.356152	-0.750560369	0.062635004	-11.9831	4.36E-33	1.75E-32	cyan	-0.689797088	1.68E-23	GO:0016020,GO:0009765	AT3G08940.2	LHCB4.2	light harvesting complex photosystem II	light-harvesting complex I chlorophyll A/B-binding protein
Sapur.003G056600.2	4.837833003	2.121978265	0.177095808	11.98209	4.41E-33	1.78E-32	cyan	0.666692248	1.56E-21	GO:0030955,GO:0006096,GO:0004743,GO:0000287	AT1G32440.1	PKp3	plastidial pyruvate kinase 3	pyruvate kinase
Sapur.006G229300.1	24.57901821	-0.940894223	0.07853713	-11.9802	4.51E-33	1.82E-32	cyan	-0.675462483	2.93E-22		AT2G24395.1		chaperone protein dnaJ-related	NA
Sapur.15WG083400.1	41.24898623	0.545601649	0.045544386	11.97956	4.55E-33	1.83E-32	cyan	0.724222477	8.38E-27	GO:0005515	AT5G38390.1		F-box/RNI-like superfamily protein	NA
Sapur.016G226200.1	7.769009419	-1.514714796	0.12650814	-11.9733	4.91E-33	1.97E-32	cyan	-0.676600875	2.35E-22		AT5G46460.1		Pentatricopeptide repeat (PPR) superfamily protein	PPR containing plant protein
Sapur.013G006400.1	23.90991115	-1.410955705	0.117888798	-11.9685	5.19E-33	2.09E-32	cyan	-0.694952573	5.75E-24					NA
Sapur.005G207800.1	53.209665	1.170012711	0.097764543	11.96766	5.25E-33	2.11E-32	cyan	0.656165709	1.08E-20		AT1G21010.1			mRNA, 952 bp sequence protein, putative
Sapur.005G156200.1	650.7516397	0.41441667	0.034655877	11.95805	5.89E-33	2.37E-32	cyan	0.686813349	3.08E-23	GO:0005525	AT4G09720.1	ATRABG3A,RABG3A	RAB GTPase homolog G3A	Rab-related GTPase family protein
Sapur.004G007400.1	600.382274	-0.792332499	0.066262411	-11.9575	5.93E-33	2.38E-32	cyan	-0.670937725	6.99E-22		AT1G11840.6	ATGLX1,GLX1	glyoxalase I homolog	lactoylglutathione lyase
Sapur.010G196400.1	29.3195425	-0.937580159	0.078444161	-11.9522	6.32E-33	2.54E-32	cyan	-0.698741673	2.58E-24		AT5G04840.1		bZIP protein	BZIP protein
Sapur.018G057400.1	6.932829887	-1.596501382	0.133625441	-11.9476	6.68E-33	2.68E-32	cyan	-0.670497702	7.60E-22	GO:0005515	AT1G60770.1		Tetratricopeptide repeat (TPR)-like superfamily protein	PPR containing plant protein
Sapur.004G083100.1	6845.773659	0.336482897	0.028164571	11.94703	6.73E-33	2.70E-32	cyan	0.675046749	3.17E-22	GO:0019904	AT3G02520.1	GF14 NU,GRF7	general regulatory factor 7	14-3-like protein GF14 protein
Sapur.007G027600.1	914.1881341	-0.470957831	0.039426712	-11.9451	6.88E-33	2.76E-32	cyan	-0.705227447	6.36E-25		AT1G75080.1	BZR1	Brassinosteroid signalling positive regulator (BZR1) family protein	brassinazole-resistant 1 protein, putative
Sapur.010G149400.1	302.2015579	-0.739527928	0.061934904	-11.9404	7.29E-33	2.92E-32	cyan	-0.726981643	4.33E-27		AT2G40070.1			proteophosphoglycan protein, putative
Sapur.010G146100.1	1.555028808	3.902112254	0.32708694	11.92989	8.27E-33	3.31E-32	cyan	0.571278332	5.58E-15	GO:0006355,GO:0003700	AT2G40220.1	ABI4.ATABI4.GIN6.ISI3.SAN	Integrase-type DNA-binding superfamily protein	NA
Sapur.013G122700.1	51.70186634	-1.336793999	0.112059699	-11.9293	8.33E-33	3.34E-32	cyan	-0.663423752	2.87E-21	GO:0005515	AT2G20710.1		Tetratricopeptide repeat (TPR)-like superfamily protein	PPR containing plant protein
Sapur.15WG043700.1	1.908740154	3.313455281	0.27780257	11.92737	8.52E-33	3.41E-32	cyan	0.656029031	1.11E-20	GO:0008270,GO:0005515	AT1G73760.1		RING/U-box superfamily protein	zinc finger, C3HC4 type (RING finger) protein, putative
Sapur.014G128800.1	157.3062761	-0.547804642	0.045935726	-11.9255	8.72E-33	3.49E-32	cyan	-0.722463876	1.27E-26	GO:0055085,GO:0016020,GO:0006810,GO:0005215	AT2G05760.1		Xanthine/uracil permease family protein	nucleobase ascorbate transporter
Sapur.004G152400.1	13.02382352	-1.30946939	0.109862611	-11.9192	9.41E-33	3.77E-32	cyan	-0.687266123	2.81E-23		AT1G08360.1		Ribosomal protein L1p/L10e family	60S ribosomal protein L10a-1
Sapur.008G104200.2	33.89907693	-0.956514865	0.080271098	-11.9161	9.76E-33	3.91E-32	cyan	-0.680222332	1.15E-22	GO:0055085,GO:0016021	AT1G70940.1	ATPIN3,PIN3	Auxin efflux carrier family protein	auxin efflux carrier protein
Sapur.005G096000.1	9.571332997	1.692673984	0.14205294	11.9158	9.79E-33	3.92E-32	cyan	0.6621987	3.60E-21	GO:0006468,GO:0005524,GO:0004672	AT2G18170.1	ATMPK7,MPK7	MAP kinase 7	MAP kinase
Sapur.004G092400.1	1.27417736	3.135252173	0.263133189	11.91508	9.88E-33	3.95E-32	cyan	0.713448248	1.02E-25	GO:0005681,GO:0000398,GO:0000387	AT1G54385.1		ARM repeat superfamily protein	NA
Sapur.009G112300.1	51.69136872	1.547336438	0.129882665	11.91334	1.01E-32	4.03E-32	cyan	0.653530048	1.73E-20	GO:0009733	AT1G29450.1		SAUR-like auxin-responsive protein family	auxin responsive protein
Sapur.014G005800.1	208.4926294	-0.881971984	0.074042431	-11.9117	1.03E-32	4.11E-32	cyan	-0.694014487	6.99E-24	GO:0016567,GO:0004842	AT5G65920.1		ARM repeat superfamily protein	E3 ubiquitin ligase PUB14, putative
Sapur.008G108000.1	21.59269834	-1.2927056	0.108531474	-11.9109	1.04E-32	4.15E-32	cyan	-0.684183829	5.24E-23	GO:0007017,GO:0005875	AT1G23220.1		Dynein light chain type 1 family protein	dynein light chain type 1 family protein
Sapur.013G006800.1	127.0494166	-0.738418633	0.061997539	-11.9105	1.04E-32	4.17E-32	cyan	-0.730134339	2.02E-27	GO:0030145,GO:0004177	AT1G09300.1		Metallopeptidase M24 family protein	metallopeptidase family M24 containing protein
Sapur.009G076800.1	161.8485585	1.442224455	0.121107258	11.90865	1.07E-32	4.26E-32	cyan	0.631173304	7.93E-19	GO:0016758,GO:0008152	AT2G15490.1	UGT73B4	UDP-glycosyltransferase 73B4	UDP-glucose:glucosyltransferase
Sapur.005G183000.1	757.9820518	0.564779285	0.047429093	11.90787	1.08E-32	4.30E-32	cyan	0.678929764	1.49E-22	GO:0016021	AT2G21600.1	ATRER1B,RER1B	endoplasmatic reticulum retrieval protein 1B	RER1A protein
Sapur.014G023100.1	184.7895785	-1.313594547	0.110337797	-11.9052	1.11E-32	4.44E-32	cyan	-0.635463566	3.90E-19		AT1G47480.1		alpha/beta-Hydrolases superfamily protein	gibberellin receptor GID1, putative
Sapur.006G149700.1	88.81438597	-0.721578242	0.060671301	-11.8932	1.28E-32	5.12E-32	cyan	-0.655871905	1.14E-20	GO:0016021	AT4G28088.1		Low temperature and salt responsive protein family	proteolipid membrane modulator, putative
Sapur.007G091400.1	211.3374224	0.558466438	0.046958496	11.89277	1.29E-32	5.15E-32	cyan	0.678408903	1.65E-22		AT2G31305.1	INH3	inhibitor-3	protein phosphatase inhibitor protein
Sapur.T040400.1	2.284127642	-2.208823903	0.185778233	-11.8896	1.34E-32	5.35E-32	cyan	-0.812296293	4.00E-38		AT3G28455.1	CLE25	CLAVATA3/ESR-RELATED 25	NA
Sapur.010G162200.1	1635.221435	0.443313915	0.037291115	11.88792	1.37E-32	5.46E-32	cyan	0.664044485	2.56E-21		AT3G12570.4	FYD	FYD	sodium- and lithium-tolerant protein
Sapur.002G057700.1	1.47008776	3.905237533	0.328584914	11.88502	1.42E-32	5.65E-32	cyan	0.581176994	1.46E-15		AT1G17950.1	ATMYB52,BW52,MYB52	myb domain protein 52	R2R3-MYB transcription factor, putative
Sapur.005G081400.1	147.0779422	-0.674299587	0.056737034	-11.8846	1.42E-32	5.67E-32	cyan	-0.722285404	1.32E-26	GO:0030983,GO:0006298,GO:0005524,GO:0032300,GO:0007131	AT4G35520.1	ATMLH3,MLH3	MUTL protein homolog 3	NA
Sapur.T181600.1	1.537783058	3.168392607	0.266611854	11.88391	1.43E-32	5.72E-32	cyan	0.656003859	1.11E-20	GO:0005681,GO:0000398,GO:0000387	AT1G54385.1		ARM repeat superfamily protein	NA
Sapur.002G141800.1	116.8565344	-1.032089823	0.086852122	-11.8833	1.45E-32	5.76E-32	cyan	-0.737382492	3.35E-28	GO:0055114,GO:0020037,GO:0016705,GO:0005506	AT2G46660.1	CYP78A6	cytochrome P450, family 78, subfamily A, polypeptide 6	cytochrome P450 family 78 protein
Sapur.15WG075400.1	11.12862264	-1.869003526	0.157294072	-11.8822	1.46E-32	5.83E-32	cyan	-0.679370264	1.36E-22	GO:0055114,GO:0016655	AT4G37925.1	NDH-M	subunit NDH-M of NAD(P)H:plastoquinone dehydrogenase complex	NA
Sapur.010G031200.1	456.959749	0.778185749	0.065492983	11.88197	1.47E-32	5.85E-32	cyan	0.670121984	8.17E-22	GO:0016757	AT1G70090.1	GATL9,LGT8	glucosyl transferase family 8	CAZy family GT8 glycosyltransferase
Sapur.006G001400.1	167.0429719	-0.574069272	0.048321244	-11.8803	1.50E-32	5.97E-32	cyan	-0.698950136	2.47E-24	GO:0005975,GO:0004553	AT4G17180.1		O-Glycosyl hydrolases family 17 protein	glucan endo-1%2C3-beta-glucosidase
Sapur.T177400.1	3.090741405	2.519677177	0.212102134	11.87955	1.51E-32	6.02E-32	cyan	0.679395922	1.36E-22	GO:0005681,GO:0000398,GO:0000387	AT1G54385.1		ARM repeat superfamily protein	NA
Sapur.T024700.1	42.29554512	1.254104441	0.105626147	11.87305	1.63E-32	6.51E-32	cyan	0.60788984	3.10E-17		AT1G50140.2		P-loop containing nucleoside triphosphate hydrolases superfamily protein	NA
Sapur.005G203500.1	634.7203392	0.304786954	0.025674645	11.87113	1.67E-32	6.66E-32	cyan	0.716798941	4.74E-26	GO:0006468,GO:0004672,GO:0006397,GO:0004540	AT5G24360.1	ATIRE1-1,IRE1-1	inositol requiring 1-1	Serine/Threonine kinase/endoribonuclease Ire1, putative
Sapur.011G021000.1	17.93990803	2.314561916	0.19498171	11.87066	1.68E-32	6.69E-32	cyan	0.605135044	4.69E-17	GO:0048544,GO:0006468,GO:0004672	AT1G11340.1		S-locus lectin protein kinase family protein	Serine/Threonine-kinase
Sapur.T134100.1	1.566106534	3.602489307	0.303560584	11.86745	1.75E-32	6.95E-32	cyan	0.604331532	5.29E-17					NA
Sapur.010G148000.1	1390.673404	-0.601119889	0.050663498	-11.865	1.80E-32	7.16E-32	cyan	-0.683906936	5.54E-23	GO:0005515,GO:0035556,GO:0007165,GO:0006629,GO:0004435	AT3G08510.1	ATPLC2,PLC2	phospholipase C 2	NA
Sapur.002G181600.1	403.4223117	0.441323504	0.03720109	11.86319	1.84E-32	7.31E-32	cyan	0.695709283	4.90E-24		AT1G14780.1		MAC/Perforin domain-containing protein	MAC/perforin domain protein
Sapur.018G090900.1	11.38920199	2.296829021	0.193673018	11.85931	1.93E-32	7.66E-32	cyan	0.499319796	2.80E-11		AT2G12550.1		ubiquitin-associated (UBA)/TS-N domain-containing protein	NA
Sapur.018G009800.1	4735.252587	-0.410909378	0.034650521	-11.8587	1.94E-32	7.71E-32	cyan	-0.684141309	5.28E-23	GO:0008270,GO:0005622,GO:0005515	AT5G24930.1	ATCOL4,COL4	CONSTANS-like 4	constans-like zinc finger protein
Sapur.008G013500.1	3769.437068	0.556708331	0.046949647	11.85756	1.97E-32	7.81E-32	cyan	0.665282411	2.03E-21	GO:0051536,GO:0009055	AT2G27510.1	ATFD3,FD3	ferredoxin 3	ferredoxin-3 protein
Sapur.011G099200.2	155.3903389	1.267362786	0.106905567	11.85497	2.03E-32	8.06E-32	cyan	0.652499592	2.08E-20		AT4G05320.2	UBQ10	polyubiquitin 10	NA
Sapur.T151400.1	5.670665566	1.529621032	0.129059358	11.85207	2.10E-32	8.33E-32	cyan	0.694836802	5.89E-24		AT5G35700.1	FIM2	fimbrin-like protein 2	NA
Sapur.019G054400.1	93.68323366	-0.631610015	0.05330304	-11.8494	2.17E-32	8.60E-32	cyan	-0.662737301	3.26E-21		AT3G56250.1			hypothetical protein
Sapur.009G081100.1	238.8971918	0.422335136	0.03565185	11.84609	2.25E-32	8.94E-32	cyan	0.681137678	9.62E-23		AT2G15695.1		Protein of unknown function DUF829, transmembrane 53	DUF829 domain protein
Sapur.005G180900.1	814.0568847	1.132037711	0.095588593	11.84281	2.34E-32	9.30E-32	cyan	0.675337156	3.00E-22	GO:0016887,GO:0005524	AT1G03905.1		P-loop containing nucleoside triphosphate hydrolases superfamily protein	ABC transporter I family protein
Sapur.001G058600.1	4.870762806	2.269109312	0.19160348	11.84274	2.35E-32	9.31E-32	cyan	0.619874624	4.88E-18	GO:0051716,GO:0046658	AT4G20260.2	ATPCAP1,PCAP1	plasma-membrane associated cation-binding protein 1	DREPP plasma membrane protein
Sapur.001G080800.1	5683.265048	0.865209399	0.073062791	11.842	2.37E-32	9.39E-32	cyan	0.620929213	4.13E-18	GO:0005975,GO:0004553	AT4G23560.1	AtGH9B15,GH9B15	glycosyl hydrolase 9B15	endo-1%2C4-beta-glucanase
Sapur.002G127000.1	432.1071787	0.608692922	0.051406285	11.84083	2.40E-32	9.52E-32	cyan	0.660738656	4.70E-21		AT3G61200.1		Thioesterase superfamily protein	acyl-CoA thioesterase, putative
Sapur.012G079400.1	398.1167947	-0.486119122	0.041061042	-11.8389	2.46E-32	9.73E-32	cyan	-0.718972	2.87E-26	GO:0005515	AT5G50900.1		ARM repeat superfamily protein	armadillo protein
Sapur.002G172500.1	34.89955779	-1.146409821	0.096847009	-11.8373	2.50E-32	9.92E-32	cyan	-0.658659651	6.87E-21					NA
Sapur.009G105900.1	134.3008088	-1.351924555	0.114247057	-11.8333	2.62E-32	1.04E-31	cyan	-0.687101342	2.91E-23	GO:0005622,GO:0003779,GO:0030042,GO:0015629	AT2G16700.1	ADF5,ATADF5	actin depolymerizing factor 5	actin-depolymerizing factor 5
Sapur.010G073700.1	1303.395778	0.409846203	0.034638048	11.83225	2.66E-32	1.05E-31	cyan	0.658531023	7.03E-21	GO:0008137,GO:0005739,GO:0003954	AT2G02050.1		NADH-ubiquinone oxidoreductase B18 subunit, putative	NADH-ubiquinone oxidoreductase B18 subunit
Sapur.005G076200.1	15.78166164	-1.324835061	0.111995595	-11.8293	2.75E-32	1.09E-31	cyan	-0.719443841	2.57E-26	GO:0009733	AT3G12830.1		SAUR-like auxin-responsive protein family	auxin-responsive family protein
Sapur.011G107300.1	16.60183824	1.267386317	0.107173576	11.82555	2.88E-32	1.14E-31	cyan	0.647753017	4.79E-20					hypothetical protein
Sapur.010G189000.1	6.119562559	2.150604921	0.181882261	11.82416	2.93E-32	1.16E-31	cyan	0.585008223	8.60E-16	GO:0006468,GO:0004672,GO:0005524	AT3G44610.1		Protein kinase superfamily protein	Serine/Threonine kinase domain protein
Sapur.014G120500.1	426.8940827	0.558733075	0.047254931	11.8238	2.94E-32	1.16E-31	cyan	0.680875117	1.01E-22		AT3G25400.1			nucleoside triphosphate pyrophosphohydrolase
[truncated: 6,385,495 more chars]
