# Supplementary material for: Integrative genomics reveals paths to sex dimorphism in Salix purpurea L
Source: Hortic Res. 2021 Aug 1;8:170. doi: 10.1038/s41438-021-00606-y (PMC8325687; doi:10.1038/s41438-021-00606-y)
Supplement: Supplementary file 10 — Supplementary Table S12 [file 41438_2021_606_MOESM10_ESM.pdf]

ID	Multimapping	Too Many Loci	Unique	Unique Reads
LIB-10X-317-003-1	2.93%	1.61%	92.67%	16667892
LIB-10X-317-004-1	3.30%	1.36%	92.08%	14912152
LIB-10X-317-005-1	2.55%	13.42%	78.88%	15599256
LIB-10X-317-006-1	2.83%	7.18%	85.96%	16640857
LIB-10X-317-007-1	2.65%	6.32%	87.51%	16937903
LIB-10X-317-008-1	2.98%	11.60%	81.15%	17228442
LIB-10X-317-011-1	2.88%	1.12%	92.50%	17342461
LIB-10X-317-012-1	2.75%	7.04%	86.64%	18624312
LIB-10X-317-013-1	2.77%	11.32%	81.28%	10369926
LIB-10X-317-013-2	2.82%	10.65%	81.57%	34886998
LIB-10X-317-014-1	3.29%	1.07%	92.86%	6162059
LIB-10X-317-014-2	3.29%	1.13%	92.58%	21958768
LIB-10X-317-015-1	2.79%	1.62%	91.85%	16092620
LIB-10X-317-016-1	2.27%	21.37%	69.26%	13170124
LIB-10X-317-017-1	2.79%	0.56%	93.98%	22354210
LIB-10X-317-019-1	2.72%	19.54%	72.35%	3098210
LIB-10X-317-020-1	2.56%	9.08%	84.50%	7192591
LIB-10X-317-020-2	2.64%	8.80%	84.41%	17760302
LIB-10X-317-021-1	2.75%	11.15%	80.39%	15825448
LIB-10X-317-024-1	2.79%	3.61%	88.20%	19277236
LIB-10X-317-028-1	3.29%	3.44%	89.31%	16781121
LIB-10X-317-029-1	2.61%	16.51%	75.58%	13997204
LIB-10X-317-033-1	2.64%	8.39%	83.91%	13715097
LIB-10X-317-034-1	2.63%	18.53%	72.22%	14433204
LIB-10X-317-035-1	2.59%	23.00%	68.72%	29354164
LIB-10X-317-036-1	3.40%	0.83%	92.89%	8584207
LIB-10X-317-036-2	3.22%	0.98%	93.24%	21119986
LIB-10X-317-038-1	3.07%	0.71%	93.71%	9026277
LIB-10X-317-038-2	3.10%	0.71%	93.35%	20583954
LIB-10X-317-040-1	2.48%	16.84%	75.14%	16123911
LIB-10X-317-041-1	2.67%	11.94%	80.70%	16640902
LIB-10X-317-042-1	3.86%	0.63%	92.69%	4377373
LIB-10X-317-044-1	2.99%	1.21%	92.98%	19774499
LIB-10X-317-045-1	3.02%	0.49%	93.51%	8491101
LIB-10X-317-045-2	2.99%	0.52%	93.19%	20913853
LIB-10X-317-046-1	2.91%	0.99%	93.14%	19201702
LIB-10X-317-047-1	3.06%	0.54%	93.60%	9918678
LIB-10X-317-047-2	3.05%	0.54%	93.39%	26325702
LIB-10X-317-049-1	3.44%	0.78%	93.31%	8057677

LIB-10X-317-049-2	3.45%	0.79%	93.04%	18638486
LIB-10X-317-050-1	2.53%	19.50%	73.34%	15951747
LIB-10X-317-052-1	2.47%	27.04%	64.34%	7541144
LIB-10X-317-054-1	3.15%	6.16%	87.25%	14360578
LIB-10X-317-055-1	2.67%	8.73%	83.72%	14901369
LIB-10X-317-056-1	2.68%	8.79%	84.12%	15737966
LIB-10X-317-058-1	2.52%	17.54%	74.28%	14807429
LIB-10X-317-059-1	2.51%	16.40%	74.72%	16276156
LIB-10X-317-060-1	3.21%	1.11%	92.87%	19488827
LIB-10X-317-061-1	2.94%	0.56%	93.81%	20011598
LIB-10X-317-065-1	2.79%	0.97%	93.43%	22096604
LIB-10X-317-068-1	2.73%	16.15%	74.81%	15798298
LIB-10X-317-069-1	2.88%	0.94%	92.21%	17561046
LIB-10X-317-076-1	2.85%	7.84%	84.68%	15362125
LIB-10X-317-078-1	3.29%	3.57%	89.15%	18653163
LIB-10X-317-081-1	2.92%	2.83%	90.50%	16851292
LIB-10X-317-086-1	2.75%	10.52%	82.37%	15778316
LIB-10X-317-089-1	3.10%	2.25%	90.57%	13724617
LIB-10X-317-091-1	3.08%	9.09%	83.83%	9142134
LIB-10X-317-092-1	2.89%	4.55%	88.81%	16264585
LIB-10X-317-093-1	2.81%	12.20%	79.42%	16078319
LIB-10X-317-097-1	1.86%	34.16%	54.41%	10015446
LIB-10X-317-099-1	1.67%	39.24%	48.79%	9101515
LIB-10X-317-100-1	1.68%	51.67%	35.56%	6346474
LIB-10X-317-101-1	1.99%	35.71%	51.83%	10653830
LIB-10X-317-102-1	2.99%	0.91%	93.11%	15300581
LIB-10X-317-104-1	2.69%	16.02%	75.67%	14866482
LIB-10X-317-105-1	2.91%	1.66%	92.27%	15273647
LIB-10X-317-106-1	2.84%	0.60%	93.82%	17095088
LIB-10X-317-107-1	3.38%	0.81%	92.93%	16365308
LIB-10X-317-108-1	3.06%	1.35%	92.36%	21192587
LIB-10X-317-109-1	2.65%	10.98%	80.92%	15715467
LIB-10X-317-112-1	2.69%	13.61%	78.08%	13213751
LIB-10X-317-113-1	3.36%	0.57%	92.13%	22019096
LIB-10X-317-114-1	1.81%	45.14%	41.19%	5482883
LIB-10X-317-115-1	2.69%	19.43%	72.65%	14447187
LIB-10X-317-117-1	3.06%	0.55%	93.43%	19160634
LIB-10X-317-120-1	3.02%	0.82%	93.33%	18902989
LIB-10X-317-121-1	3.28%	0.58%	93.39%	21503386
LIB-10X-317-122-1	2.76%	2.82%	91.54%	16812332
LIB-10X-317-124-1	3.12%	0.49%	93.16%	22176339

LIB-10X-317-127-1	3.70%	1.02%	92.79%	8413713
LIB-10X-317-127-2	3.72%	1.01%	92.45%	18980110
LIB-10X-317-129-1	2.74%	6.46%	87.30%	15244645
LIB-10X-317-134-1	1.47%	42.78%	46.96%	3235766
LIB-10X-317-134-2	1.56%	41.45%	47.80%	9396522
LIB-10X-317-135-1	3.05%	0.74%	92.20%	5029802
LIB-10X-317-135-2	3.11%	0.70%	91.71%	18251258
LIB-10X-317-136-1	2.87%	4.91%	89.45%	5746327
LIB-10X-317-136-2	2.91%	4.99%	89.06%	14220871
LIB-10X-317-138-1	1.77%	36.66%	51.84%	8769333
LIB-10X-317-139-1	2.09%	32.08%	58.56%	5292155
LIB-10X-317-139-2	2.14%	31.37%	58.73%	12404207
LIB-10X-317-140-1	3.01%	5.40%	88.05%	7021534
LIB-10X-317-140-2	3.06%	5.12%	88.04%	17903153
LIB-10X-317-141-1	1.88%	36.45%	52.31%	9982491
LIB-10X-317-145-1	1.86%	37.11%	53.00%	6780562
LIB-10X-317-145-2	1.83%	38.06%	51.64%	12657563
LIB-10X-317-146-1	3.44%	2.08%	91.68%	11668988
LIB-10X-317-146-2	3.38%	2.30%	91.32%	24970050
LIB-10X-317-147-1	2.80%	1.03%	92.58%	16748055
LIB-10X-317-149-1	2.89%	1.55%	91.86%	22276314
LIB-10X-317-152-1	2.99%	1.24%	92.19%	19986617
LIB-10X-317-153-1	2.99%	0.73%	92.74%	20263154
LIB-10X-317-155-1	3.29%	1.25%	92.63%	9558224
LIB-10X-317-155-2	3.31%	1.23%	92.37%	21157118
LIB-10X-317-156-1	3.04%	0.87%	93.62%	15695950
LIB-10X-317-159-1	2.54%	17.20%	74.47%	7624886
LIB-10X-317-159-2	2.54%	17.32%	74.25%	7948678
LIB-10X-317-161-1	2.72%	7.06%	85.11%	5538764
LIB-10X-317-161-2	2.76%	7.06%	84.53%	15423940
LIB-10X-317-163-1	2.86%	0.98%	93.64%	16520816
LIB-10X-317-164-1	3.35%	2.86%	91.16%	7407459
LIB-10X-317-164-2	3.33%	3.09%	90.70%	23003487
LIB-10X-317-165-1	2.92%	6.09%	87.39%	10455268
LIB-10X-317-165-2	2.92%	6.80%	86.36%	25106082
LIB-10X-317-166-1	3.04%	0.70%	93.86%	7447451
LIB-10X-317-168-1	2.75%	9.21%	82.70%	14897405
LIB-10X-317-171-1	2.92%	0.44%	94.07%	17192714
LIB-10X-317-175-1	2.01%	31.61%	56.89%	8977429
LIB-10X-317-176-1	3.05%	3.43%	90.39%	6531132
LIB-10X-317-178-1	3.31%	0.74%	93.24%	11513276

LIB-10X-317-178-2	3.28%	0.80%	92.83%	20802543
LIB-10X-317-181-1	2.94%	1.25%	92.52%	17383416
LIB-10X-317-182-1	3.15%	1.05%	93.31%	6635292
LIB-10X-317-183-1	2.91%	1.34%	93.31%	6691702
LIB-10X-317-184-1	3.05%	1.82%	91.79%	5405208
LIB-10X-317-186-1	3.34%	0.70%	92.74%	3975394
LIB-10X-317-190-1	2.91%	1.75%	92.92%	13144042
LIB-10X-317-191-1	3.41%	0.63%	93.38%	8298361
LIB-10X-317-191-2	3.45%	0.66%	93.00%	18199939
LIB-10X-317-192-1	2.30%	19.98%	71.46%	10272119
LIB-10X-317-195-1	1.85%	33.92%	56.66%	5768655
LIB-10X-317-195-2	1.89%	33.82%	56.23%	12415258
LIB-10X-317-197-1	2.54%	12.70%	79.33%	14107892
LIB-10X-317-198-1	2.44%	19.68%	72.16%	6523278
LIB-10X-317-198-2	2.50%	19.26%	72.19%	17629402
LIB-10X-317-199-1	2.76%	9.96%	82.82%	15742760
LIB-10X-317-201-1	2.80%	5.13%	88.66%	17109967
LIB-10X-317-203-1	3.22%	0.89%	92.55%	12211895
LIB-10X-317-203-2	3.29%	0.90%	92.02%	22032609
LIB-10X-317-204-1	2.68%	13.71%	78.65%	14001970
LIB-11X-317-002-1	2.03%	24.80%	64.91%	11440641
LIB-11X-317-003-1	2.71%	6.96%	86.56%	8285691
LIB-11X-317-003-2	2.69%	7.09%	86.28%	8689441
LIB-11X-317-004-1	3.16%	0.57%	93.71%	8851009
LIB-11X-317-004-2	3.20%	0.58%	93.34%	21150747
LIB-11X-317-008-1	3.16%	3.76%	90.58%	8346009
LIB-11X-317-008-2	3.21%	3.92%	90.08%	20736189
LIB-11X-317-009-1	3.18%	0.64%	93.10%	9851943
LIB-11X-317-009-2	3.24%	0.63%	92.74%	22206506
LIB-11X-317-012-1	2.69%	8.02%	85.20%	5603753
LIB-11X-317-012-2	2.69%	8.79%	83.83%	17508354
LIB-11X-317-018-1	2.31%	17.40%	74.18%	6706434
LIB-11X-317-018-2	2.31%	17.83%	73.56%	7312148
LIB-11X-317-022-1	2.47%	21.40%	68.65%	6488129
LIB-11X-317-022-2	2.47%	21.56%	68.33%	6631724
LIB-11X-317-024-1	2.44%	17.43%	73.76%	4611018
LIB-11X-317-024-2	2.45%	17.64%	73.39%	4877496
LIB-11X-317-029-1	3.17%	9.37%	83.75%	6585239
LIB-11X-317-029-2	3.07%	11.00%	81.62%	15782751
LIB-11X-317-030-1	2.76%	3.87%	88.86%	8171708
LIB-11X-317-030-2	2.74%	3.92%	88.66%	8513187

LIB-11X-317-034-1	2.60%	12.10%	78.56%	6717616
LIB-11X-317-034-2	2.59%	12.15%	77.98%	7696685
LIB-11X-317-039-1	3.28%	1.09%	92.38%	8269411
LIB-11X-317-039-2	3.21%	1.11%	91.87%	18918471
LIB-11X-317-042-1	2.96%	1.22%	92.85%	9065489
LIB-11X-317-042-2	2.95%	1.27%	92.41%	20784842
LIB-11X-317-043-1	2.56%	19.07%	72.58%	8924422
LIB-11X-317-043-2	2.55%	19.69%	71.47%	16667724
LIB-11X-317-046-1	3.03%	2.75%	91.63%	16987284
LIB-11X-317-049-1	2.81%	0.51%	93.50%	18041268
LIB-11X-317-051-1	3.11%	5.73%	86.89%	6262883
LIB-11X-317-051-2	2.95%	6.52%	86.01%	15926093
LIB-11X-317-055-1	1.57%	46.01%	40.48%	6825668
LIB-11X-317-058-1	3.16%	0.59%	92.75%	9743703
LIB-11X-317-058-2	3.18%	0.59%	92.29%	23850310
LIB-11X-317-060-1	2.38%	22.79%	68.14%	5331139
LIB-11X-317-060-2	2.42%	22.27%	68.33%	16279661
LIB-11X-317-063-1	1.84%	40.15%	49.93%	3184831
LIB-11X-317-063-2	1.90%	40.36%	49.19%	11266596
LIB-11X-317-064-1	2.74%	7.83%	84.82%	15546785
LIB-11X-317-068-1	2.06%	37.19%	50.32%	10302792
LIB-11X-317-069-1	3.25%	0.61%	93.54%	7099620
LIB-11X-317-069-2	3.27%	0.63%	93.25%	19148299
LIB-11X-317-075-1	3.17%	0.93%	93.69%	8646239
LIB-11X-317-075-2	3.18%	0.90%	93.55%	22118485
LIB-11X-317-076-1	3.05%	2.78%	90.90%	6910918
LIB-11X-317-076-2	3.11%	2.69%	90.72%	19911466
LIB-11X-317-080-1	3.62%	0.54%	93.79%	15675963
LIB-11X-317-083-1	2.77%	18.75%	73.25%	21332234
LIB-11X-317-084-1	2.85%	0.57%	94.13%	14706404
LIB-11X-317-086-1	3.20%	0.61%	94.24%	13296574
LIB-11X-317-089-1	2.95%	0.74%	94.04%	10741331
LIB-11X-317-093-1	3.41%	0.91%	92.96%	22948150
LIB-11X-317-103-1	1.86%	41.64%	48.34%	5466356
LIB-11X-317-106-1	2.99%	0.66%	93.24%	9428248
LIB-11X-317-108-1	3.44%	0.56%	93.65%	14900429
LIB-11X-317-110-1	2.92%	0.80%	93.87%	10958898
LIB-11X-317-116-1	2.87%	2.73%	91.31%	9695403
LIB-11X-317-117-1	3.24%	0.69%	93.55%	22370040
LIB-11X-317-118-1	2.91%	0.92%	93.22%	28509195
LIB-11X-317-123-1	2.99%	3.46%	90.71%	18810450

LIB-11X-317-128-1	3.16%	0.62%	92.53%	20167179
LIB-11X-317-130-1	3.27%	0.55%	93.77%	24760161
LIB-11X-317-135-1	2.81%	17.51%	74.51%	16292046
LIB-11X-317-190-1	2.95%	11.92%	80.44%	14522438
LIB-11X-317-193-1	2.68%	14.05%	78.49%	15591745
LIB-11X-317-194-1	2.88%	1.86%	91.94%	29174127
LIB-11X-317-197-1	3.00%	1.46%	93.18%	30414610
LIB-11X-317-198-1	2.95%	0.80%	93.10%	29605152
LIB-11X-317-199-1	2.45%	16.44%	74.44%	12501530
LIB-11X-317-200-1	2.91%	0.97%	92.72%	15939606
LIB-11X-317-203-1	2.72%	9.07%	83.52%	12198905
LIB-11X-317-204-1	2.73%	1.05%	92.61%	14478715
LIB-11X-317-205-1	2.51%	15.09%	75.97%	12263539
LIB-11X-317-208-1	2.65%	7.98%	84.21%	15508658
LIB-11X-317-212-1	2.26%	17.74%	72.69%	17540133
LIB-11X-317-213-1	3.08%	4.27%	90.21%	15604239
LIB-11X-317-217-1	1.76%	34.25%	54.12%	12808381
LIB-11X-317-231-1	2.65%	8.98%	83.30%	18670087
LIB-11X-317-232-1	2.74%	2.08%	91.69%	24079526
LIB-11X-317-234-1	2.81%	0.61%	93.83%	22068082
LIB-11X-317-239-1	2.87%	2.58%	90.93%	12630116
LIB-11X-317-240-1	2.81%	4.77%	88.02%	40192100
LIB-94006-1	3.34%	8.37%	85.73%	40586744
